# Bidirectional Modification of a *Galbulimima* Alkaloid Identifies Selective Opioid Ligands

**DOI:** 10.1021/acscentsci.5c00573

**Published:** 2025-07-07

**Authors:** Florian Martin Zielke, Stone Woo, Samuel Kasmali, Allison Volf, Vuong Q. Dang, Jake B. Bailey, Milan Gembicky, Laura M. Bohn, Ryan A. Shenvi

**Affiliations:** † Department of Chemistry, 4356 Scripps Research, La Jolla, California 92037, United States; ‡ Graduate School of Chemical and Biological Sciences, 4356 Scripps Research, La Jolla, California 92037, United States; § Department of Molecular Medicine, 145764 The Herbert Wertheim UF Scripps Institute for Biomedical Innovation & Technology, Jupiter, Florida 33458, United States; ∥ Department of Chemistry and Biochemistry, 8784 University of California, San Diego, La Jolla, California 92093, United States

## Abstract

We report a bidirectional diversification and optimization campaign of the newly identified *mu*- and *kappa*-opioid receptor antagonist GB18, a naturally occurring *Galbulimima* alkaloid. First, we find that replacement of the GB18 piperidine with pyridine alters the pharmacology from antagonism to partial agonism, with reduced potency but markedly higher receptor selectivity for *kappa-* over *mu-*. Second, we optimize this hit via development of a mutually chemoselective cross-coupling of an alkyl iodide/vinyl triflate pair that leads to a series of low- and sub-nanomolar KOR-selective full agonists, some of which demonstrate bias for G protein activation over β-arrestin2 recruitment. Third, we advance three leads to *in vivo* (mouse) analysis and demonstrate brain penetrance and behavioral effects. In an open-field activity assay, we demonstrate that by increasing G protein signaling preference, agonists display an increase in exploratory, anxiolytic-like behaviors with no signs of sedation. The brevity and success of this campaign, combined with *in vitro* and *in vivo* pharmacology, demonstrate GB18 and its analogs as tractable new opioid scaffolds that allow favorable properties to be dialed in and unwanted properties removed.

## Graphical abstract

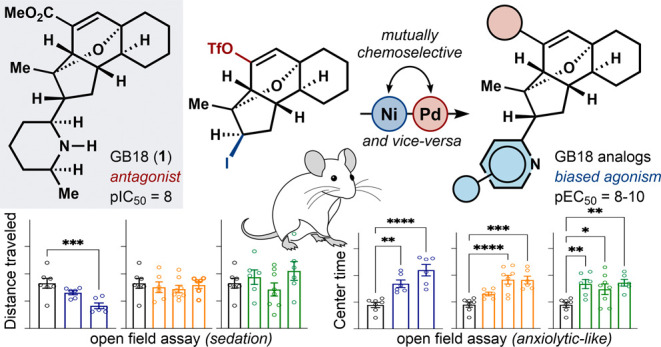

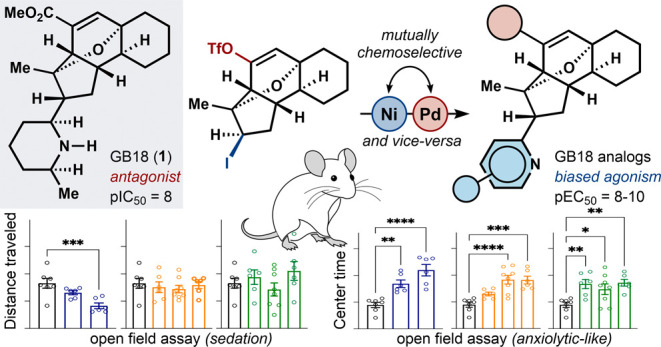

## Introduction

The natural product, GB18 ^1^ (Figure 1), derives from the *Galbulimima* genus of tree, whose bark features in the traditional medicine and ritual of the Gimi Peoples of Papua New Guinea.^2^ Bark ingestion causes psychotropic effects in humans, indicating the presence of one or more orally bioavailable and brain-penetrant neuroactive small molecules.^3^ Seminal studies by Smith, Kline, & French (SKF) associated specific bark alkaloids (*Galbulmima* or “GB” alkaloids^4^) with effects in animals or animal tissue, including the observation that GB18 inhibited mouse grooming with no apparent effect on sensation.^5^ This unusual data led Thomas to hypothesize that GB18 penetrated the brain and affected cognition or mood, perhaps explaining some of the effects of bark in humans.^3^ Recently, we identified GB18 as a mixed *kappa*- (KOR) and *mu*-opioid receptor (MOR) antagonist (pIC_50_ = 8)^6^ via *in vitro* screens against diverse neuronal receptors frequently involved in drug mechanisms of action.^7^ In addition to being unique as a naturally occurring antagonist at *mu-* and *kappa*-opioid receptors, GB18 represents a new scaffold in a relatively limited pharmacological landscape.

**Figure 1:**
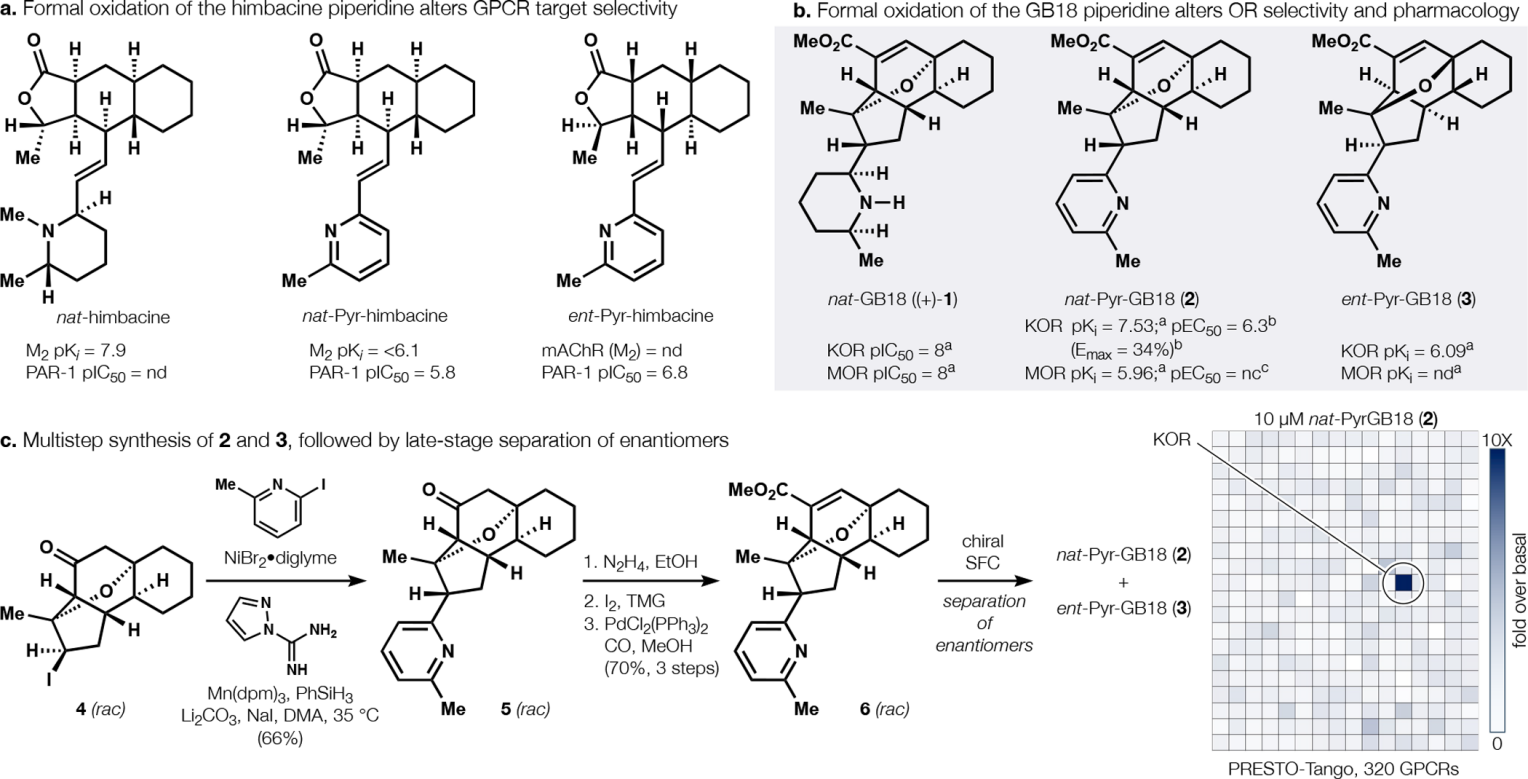
Himbacine vs GB18. **a.** Reversal of absolute configuration and piperidine unsaturation changes GPCR selectivity from mAChRs to PAR-1. **b**. Parallel changes to GB18. **c**. Synthesis of **2** and **3** by analogy to ref 6. Screen across 320 GPCRs (PRESTO-Tango) identifies **2** as a highly selective partial agonist. ^a^TANGO β-arrestin signaling dose–response (response in relative luminescence units, RLU) (*n* = 3);^7^ ^b 35^S-GTPγS binding as % maximal U69,593 response; ^c 35^S-GTPγS binding as % maximal DAMGO response.

Here we report that only minor changes to the GB18 scaffold—removal of 6 hydrogens from its piperidine—convert the KOR/MOR mixed antagonist natural product into a selective KOR agonist. The original total synthesis route^6^ proved tedious for diversification (12 steps, linear), so we revise the route via a bidirectional synthesis strategy to improve efficiency. Now, only 7 steps are necessary to reach a doubly electrophilic scaffold, which we demonstrate to undergo mutually chemoselective sp^2^-sp^3^ (Ni) and sp^2^-sp^2^ (Pd) cross-coupling, in either order. These divergent modifications probe sites that play important roles in receptor activation and functional bias according to extensive *in vitro* pharmacology. Furthermore, the chemistry can be effectively scaled to support *in vivo* assays of top-performing analogs. In mice, we interrogate KOR agonists that show bias for G-protein activation over β-arrestin2 recruitment and determine brain penetrance in addition to efficacy as potential nonsedating anxiolytics. These are the first SAR studies of GB18—*in vitro* or *in vivo—*and demonstrate this series’ potential for lead discovery and development.

## Results and Discussion

Prior to this work, only one other GB alkaloid had been assigned a target: class Ia alkaloid himbacine caused tachycardia in mice via antagonism of muscarinic acetylcholine receptors (mAChRs).^8^ These members of the rhodopsin-like GPCR family attracted attention as potential targets for disorders of cholinergic neurotransmission, especially Alzheimer’s disease.^9^ Schering-Plough explored numerous himbacine analogs, including *ent-*himgaline pyridyls (Figure 1a), which exhibited a different activity profile: selective antagonism of PAR-1,^10^ an alternative GPCR.^11^ To probe the effects of an analogous piperidine to pyridine change to the GB18 scaffold, we synthesized *nat*-PyrGB18 (**2**) and *ent*-PyrGB18 (**3**) (Figure 1b). Each were conveniently accessed by late-stage cross-electrophile coupling^12^ (Figure 1c) involving an unusual Mn­(III), Ni­(II), silane-mediated reaction, by analogy to our existing route to GB18.^6^ A GPCRome PRESTO-Tango screen, provided by the NIH-sponsored Psychoactive Drug Screening Program (PDSP),^13^ demonstrated that **2** agonized KOR with high selectivity among 320 GPCRs (Figure 1c), with no evidence of mAChR (M_1–5_) or MOR activation. More thorough pharmacology identified **2** as a partial KOR agonist with no activation of MOR (Figure 1b) (in contrast, **3** showed low potency; see SI). Thus, removal of 6 hydrogens from (+)-**1** dramatically changed the pharmacology and selectivity of the GB18 scaffold, even though target engagement was maintained. Because this change was also accompanied by a 100× potency loss, we probed the SAR of this lead (**2**) to return the affinity of the original natural product hit while retaining selective KOR agonism. This was a challenging task given the complexity of the scaffold, especially the attached-ring system in which the pyridine resides on the *endo*- face of the carbocycle (see Figures 2 and 3 below). Additionally, the 4-step linear sequence and late-stage resolution of enantiomers did not lend itself to rapid SAR acquisition. Both problems we address below.

**Figure 2:**
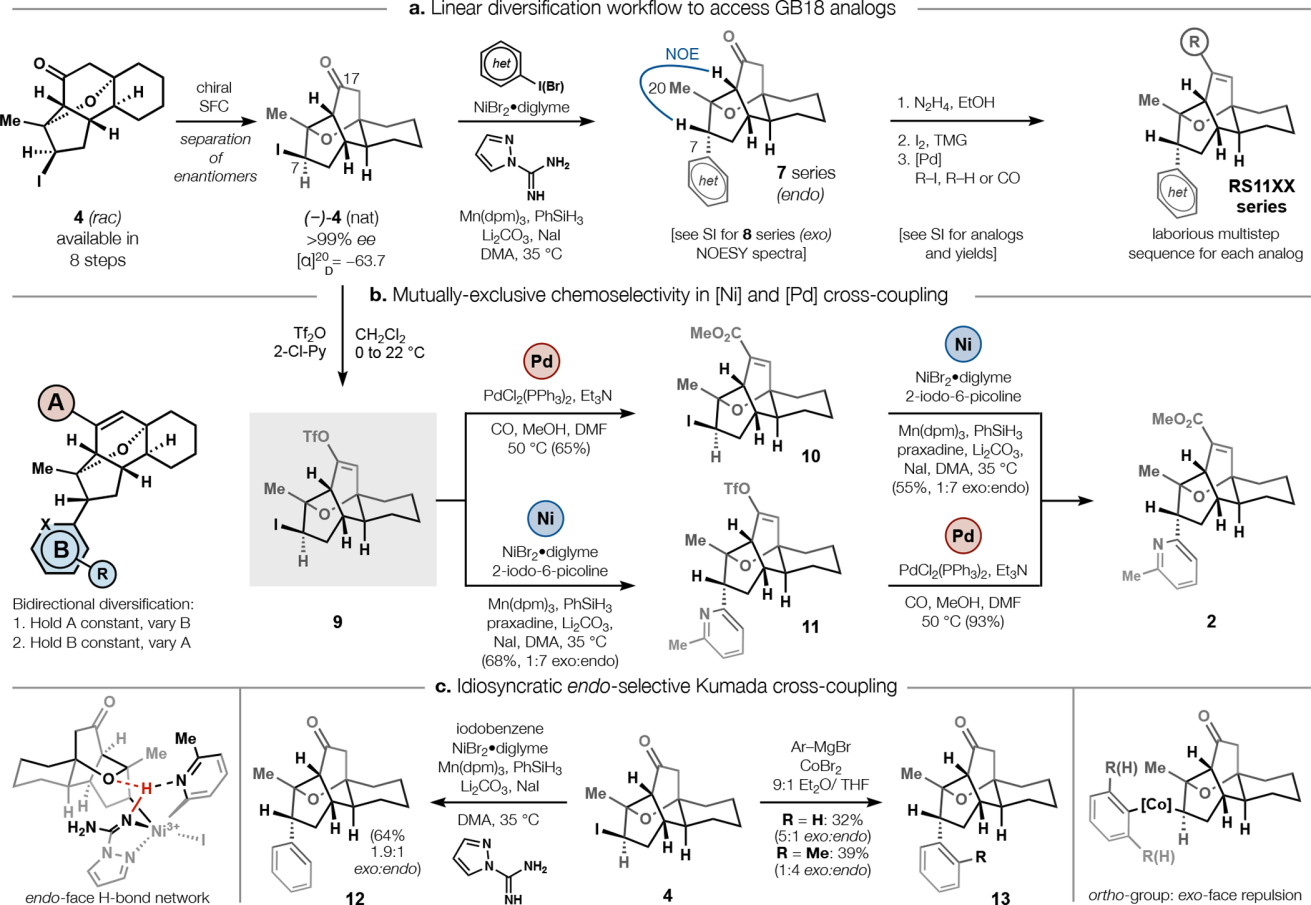
Scalemic iodide **4** (*nat*) can be diversified bidirectionally by either **a**. multistep cross-electrophile coupling, Barton iodination, and carbonylation; or **b**. mutually exclusive Ni/Pd cross-coupling of its enol triflate **5**.

**Figure 3:**
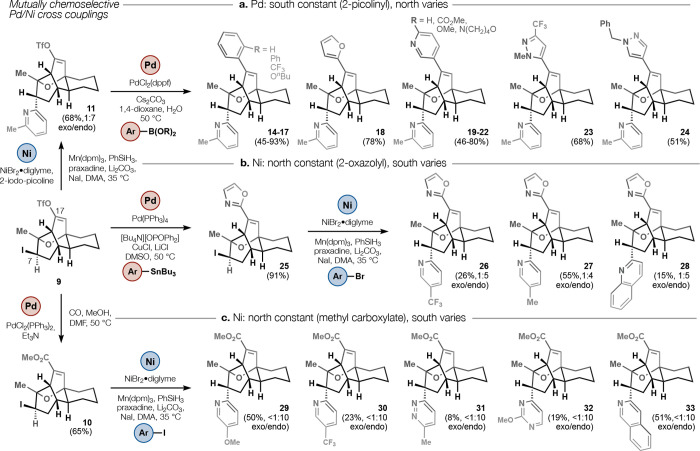
Bidirectional diversification via mutually chemoselective cross-coupling. **a**. Suzuki cross-coupling of the northern vinyl triflate; **b**. Ni/Mn cross-electrophile coupling after Stille reaction; **c**. Ni/Mn cross-electrophile coupling after methoxycarbonylation.

*Rac-*GB18 (±)-**1** had been previously synthesized on a large scale, and its enantiomers were separated for biological assay. However, resolution at the end of each analog synthesis would be impractical, so we explored separation of a diversifiable intermediate. *Rac-*ketoiodide **4** displayed outstanding separation on chiral SFC (Diacel IG, 35% MeOH/CO_2_, α > 2.5), allowing >6 g of racemate to be separated by automated injection over 8 h (Figure 2a). The absolute configuration of *nat*-**4** was assigned by single crystal X-ray analysis (Flack parameter: −0.001); the >99% *ee* crystalline material was stable at −20 °C for at least 12 months in the absence of light (we did not identify high affinity targets of *ent*-pyrGB18 (**3**), so subsequent discussion only involves the *nat-* enantiomer series). The two functional groups of *nat*-**4**—ketone and *sec*-alkyl iodide—allowed bidirectional diversification of the scaffold by analogy to ref 6. Several analogs were accessed in this stepwise fashion before we discovered a more efficient strategy.

The iodide of *nat-* **4** could be substituted with diverse iodoarenes (see SI) using our Ni- and Mn-mediated Weix-type cross-electrophile coupling without modification (Figure 2a). These conditions yielded the more potent *endo-* products preferentially across diverse partners and spared both the ketone and the strained bridging ether. Conditions that employed a strong base or acid tended to cleave the ether by E1Cb or heterolysis. When (hetero)­aryl iodides were not available, greater equivalents of the corresponding (hetero)­aryl bromide sufficed. Despite the diversity of product structures (Figure 2b), a diagnostic upfield shift and doublet-of-doublets (dd) splitting pattern of bridgehead proton H18 consistently identified the *endo*- diastereomer; a diagnostic shielding of C20 (CH_3_) distinguished the alternative *exo*- isomer (upfield shift to ca. 0.8 ppm, see SI for all spectroscopic trends). *Endo*-selectivity decreased when electron-withdrawing groups were placed on the 2-pyridyl partner or when 3- and 4-pyridine partners were coupled. Non-heteroaromatic groups (e.g., phenyl) also coupled, albeit with a preference for *exo*-selectivity using our Ni/Mn conditions (see Figure 2c). These data are consistent with a model for *endo-*selectivity governed by a Ni-amidine-pyridine H-bond network. However, we also found cobalt-catalyzed Kumada cross-coupling (CoBr_2_, TMEDA, 9:1 Et_2_O/THF, 0 °C) to be effective, rapid, and, in the unique case of *ortho-*substituted arenes (e.g., *o-*tolyl), moderately *endo-*selective (1:4 dr, 39%, 2-iodotoluene, see SI). This idiosyncratic *endo-* preference may reflect steric repulsion encountered on the *exo*-face specifically for *ortho-* substituents, which project into the polycyclic core, consistent with stereochemistry-determining reductive elimination proposed in related radical-mediated sp^2^-sp^3^ cross-coupling.^14^ Despite the lack of a hydrogen-bond accepting heteroatom, some all-carbon arenes retained good potency at KOR, depending on the substitution pattern (see **RS1167**, **RS1168**, and **RS1187** in Figure 4 below).

**Figure 4:**
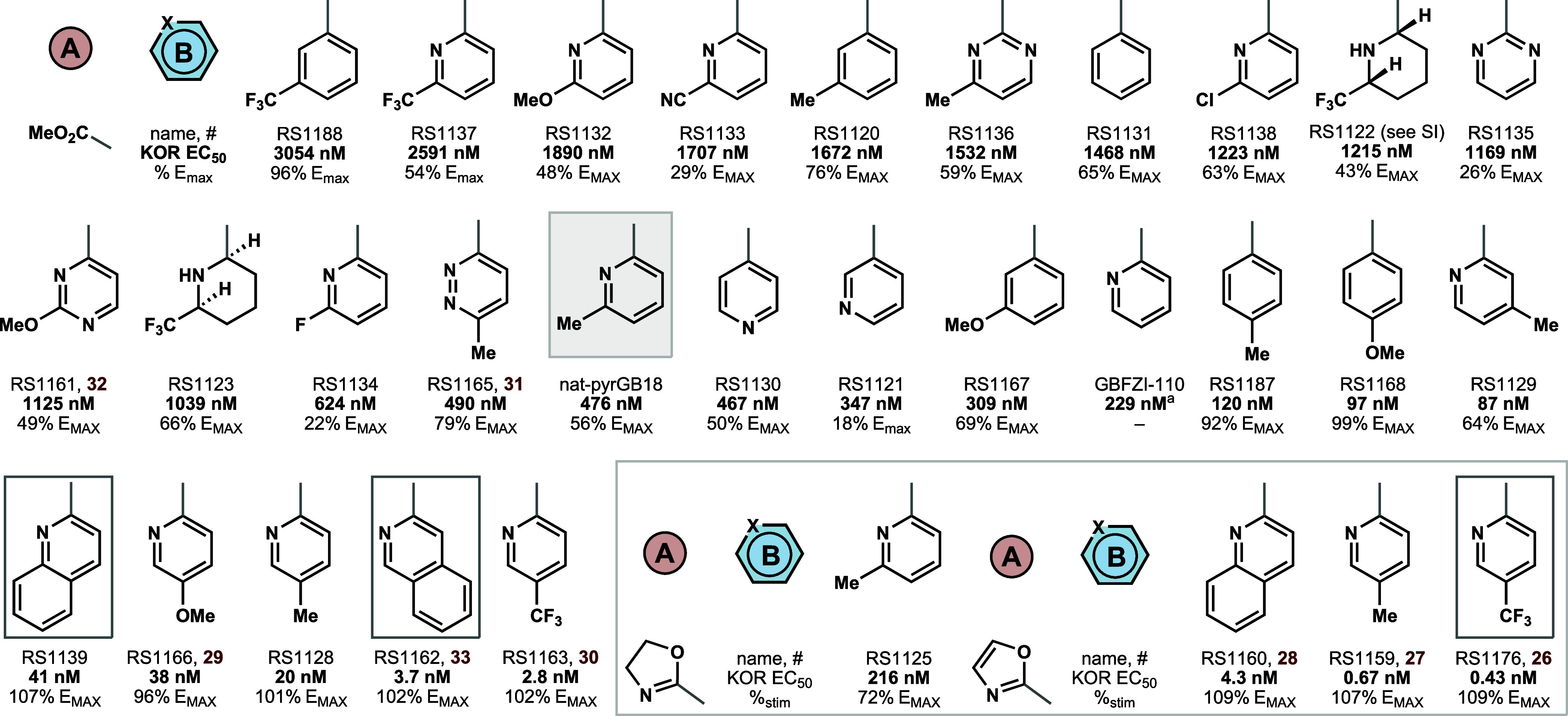
Analogs synthesized in this study. Potencies are EC_50_ for inhibition of forskolin-stimulated cAMP accumulation (KOR agonism), presented as % maximal U69,593 response, unless otherwise noted. No compounds activate MOR at doses tested up to 10 μM (EC_50_ > 10 μM). ^a^EC_50_ of ^35^S-GTPγS binding. See Supplemental Table 1 for *E*_MAX_, pEC_50_ values with SEM.

The northern C17 carbonyl could be functionalized via Barton iodination and Pd-catalyzed cross-coupling (Figure 2a). In this way, each *endo*- arene coupling product could be diversified to peripherally *bis*-functionalized derivatives of the GB18 tetracycle in 3 additional steps. One of these steps, however, required hydrazine, which competitively reacted with halopyridyl motifs via S_N_Ar (e.g., **RS1138**, Figure 4). In addition, the tedious multistep workflow endangered any long-term practicality of the campaign.

Fortunately, we found that this sequence could be supplanted by a shorter choreography of mutually chemoselective cross-couplings, using nickel and palladium. For example, **4** underwent clean triflation of the C17 ketone (Tf_2_O, 2-chloropyridine) to afford **9** in 75% yield. The resulting vinyl triflate could be selectively engaged by Pd catalysts to afford, for example, iodo ester **10** (Pd­(PPh_3_)_2_Cl_2_, CO, Et_3_N, MeOH, and DMF; 65% yield). To our surprise, the unsaturated ester of **10** was spared in the Ni/Mn cross-coupling and yielded *nat-*PyrGB18 (**2**) as a proof-of-principle for this more practical route. Alternatively, the opposite choreography also proved effective, whereby Ni/Mn cross-coupling first reacted the C7 *sec*-alkyl iodide to yield **11** selectively, and then Pd-catalyzed carbonylation reacted the C17 enol triflate: an alternate route to **2**. Although there was no great advantage to reversal of Pd/Ni vs Ni/Pd choreography in the production of **2** itself, the apparent mutually exclusive chemoselectivity allowed the northern motif to be held constant, while the southern motif was diversified, and vice versa, resulting in a highly effective strategy to generate a focused library of analogs. Although some yields of the Ni cross-coupling fell short of ideal, they spared the strained bridging ether and double bond, while delivering extensive SAR at the southern region.

Figure 3 illustrates several embodiments of this Ni/Pd diversification strategy. The first exploration (Figure 3a) probed the capacity of 2-picolinyl scaffold **11** (derived from iodo-triflate **9**) to undergo palladium-catalyzed Suzuki cross-coupling, as a proxy for Lewis-base tolerance generally. Yields proved good to excellent and indicated compatibility between C17 Suzuki reactions and C7 *N*-heterocyclic substituents generally. Conversely, Stille cross-coupling (Figure 3b) proved compatible with iodo-triflate **9**, leading to a C17 2-oxazole (**25**) as a bioisosteric replacement for the native methyl carboxylate (see Figure 4). Longer chain esters, amides, or arenes led to substantial affinity losses, so most analogs retained the methyl ester. A 2-oxazoline derived from amidation of ester **2** also proved effective (see SI and Figure 4). Despite the Lewis basicity of oxazole **25**, it could also be diversified without inhibition of the Ni/Mn sp^2^-sp^3^ cross-coupling, an effective demonstration that both cross-couplings (Pd and Ni) could incorporate Lewis basic heterocycles. Finally, as suggested by the 2-picolinyl coupling in Figure 2, iodo-ester **10** could be diversified to a series of analogs that differed extensively at the southern region (Figure 3c). Neither oxazole **25** nor ester **10** showed any deleterious effect to the *endo-* selectivity of coupling. Some of these analogs were identified to be highly potent full agonists (Figure 4) and could also be formed by reversing the order of Pd or Ni reactions (see SI).

The mutually exclusive chemoselectivity of vinyl triflate and alkyl iodide between palladium and nickel catalysts allowed **9**, **10**, and **11** to be used interchangeably to access *bis*-functionalized analogs. Both **9** (as a solution frozen in benzene) and **10** (crystalline solid) could be stockpiled in multihundred milligram quantities. Typically, **10** and **11** gave even better results for a given aryl iodide (Ar–I) coupling compared to **4**, which occasionally underwent E1cB ether fragmentation. Between the multistep, linear route and the streamlined divergent cross-couplings, 30 analogs were prepared to advance the initial hit, PyrGB18 (**2**) (Figure 4). The weak and partial agonism associated with **PyrGB18** (EC_50_ = 476 nM, 56% *E*_MAX_ stability) led these initial SAR studies to focus on potency and % stimulation. Analogs are rank ordered in Figure 4 by KOR (cAMP, HTRF) potency and northern substituent identity; the initial hit **PyrGB18** is boxed for orientation to losses and gains in potency. Three main drivers of potent agonism became clear. First, the lower basicity of the nitrogen atom, not just its planarity, drove the shift from antagonism (e.g., GB18) to agonism, as illustrated by diastereomeric 6-trifluoromethylpiperidines **RS1122** and **1123**, whose nitrogens are about 5 p*K*_b_ units lower in basicity than piperidine (by analogy to ethylamine vs 2,2,2-trifluoroethylamine: p*K*_b_ 10.7 vs 5.7).^15^ Second, the presence and location of the pyridine nitrogen were important for high potency but not required to surpass the weak partial agonism of **PyrGB18**: even substituted phenyl rings exhibited full agonism of KOR near 100 nM EC_50_ (**RS1168**)—a remarkable example of a KOR agonist devoid of nitrogen atoms. Until the identification of salvinorin A (SalA) as a potent and selective non-nitrogenous KOR agonist,^16^ the presence of a basic group like an amine (conjugate acid p*K*_a(H2O)_ = ca. 9) was considered an essential opioid recognition unit to bind the conserved Asp138 residue in transmembrane domain III (TM III).^17^ The identification of alternative GB chemotype **1168** offers synthetic tractability and analog opportunities that are orthogonal to SalA.^18^ Third, a methyl walk from C6 to C4 identified C5 (*para*- to the attached-ring bridgehead carbon) as an optimal site for substitution. Within the methyl ester series, a C5-trifluoromethyl exhibited the highest potency full agonism at about 3 nM EC_50_. The oxazoline and oxazole isostere effectively replaced the methyl ester without losses in potency and, in fact, 10–20× gains. *Across the series, MOR EC*_50_ *exceeded 10 μM,* likely a consequence of the low basicity of the pyridine vis-à-vis the GB18 piperidine,^19^ analogous to SalA selectivity.^16^ This successful campaign benefitted from the 7-step, gram-scale synthesis of iodide **10** and the ease of bidirectional diversification. Having identified clear SAR trends and several selective KOR full agonists (according to a cAMP accumulation assay), we interrogated in greater detail the *in vitro* and *in vivo* pharmacological properties of **RS1176**, **1162**, and **1139**, which showed a range of potencies (ca. 0.4, 4, and 40 nM EC_50_), as well as different signaling biases, as described below.

Pharmacological activity and radioligand binding were evaluated in stably expressing human KOR cell lines across three signaling platforms and compared to the activity of the reference agonist, U69,593 (Figure 5, Table 1). All three agonists displayed high affinity for KOR as opposed to the MOR (the *mu* opioid receptor) when assessed in membranes from CHO-K1 cells expressing human KOR or MOR cells (Table 1 and SI Table S4). In the same CHO-KOR cells, **RS1176** was potent in promoting ^35^S-GTPyS binding and inhibiting forskolin-stimulated cAMP accumulation relative to U69,593. When tested in the human KOR-expressing PathHunter β-arrestin2 recruitment cell line, **RS1176** was less potent relative to U69,593, while it retained maximum efficacy as defined by U69,593 (Figure 5a). Relative to U69,593, **RS1162** and **RS1139** proved to be partial agonists at the KOR for ^35^S-GTPyS binding, with **RS1139** losing potency relative to the other agonists. In the cAMP assay, the same rank order potency is maintained among the agonists, although **RS1162** and **RS1139** appear to be full agonists in the cAMP assay. However, it is not unusual to lose detection of partial agonism in such highly amplified assays. For β-arrestin2 recruitment (Figure 5a), the agonists retain the same rank order potency and efficacy relative to U69,593; however, the order of magnitude from U69,593 varies (i.e., where RS1162 has a similar potency to U69,593 in the GTPγS binding assay, it is clearly less potent than U69,593 in the βarrestin2 assay). To quantitate such relative shifts in potencies and efficacies, transduction efficiencies were derived by fitting to the operational model with U69,593 as the reference agonist to calculate bias factors, as previously described (Figure 5b).^20–24^ When comparing ^35^S-GTPyS binding over β-arrestin2 recruitment, **RS1162** and **RS1139** show increasing preference for G protein signaling relative to RS1176 (Table 1, bias factors 9 and 12 respectively). While **RS1176** displays some measurable preference for GTPγS binding, it may not prove to be a robust indicator of preference. Of note, there is a modest introduction of bias between ^35^S-GTPγS binding and cAMP accumulation for **RS1162** (Table 1 bias factor of 4 ); however it is not as robust as the separation observed for a series of triazole agonists described by Bohn and Aubé.^23,25^

**Figure 5:**
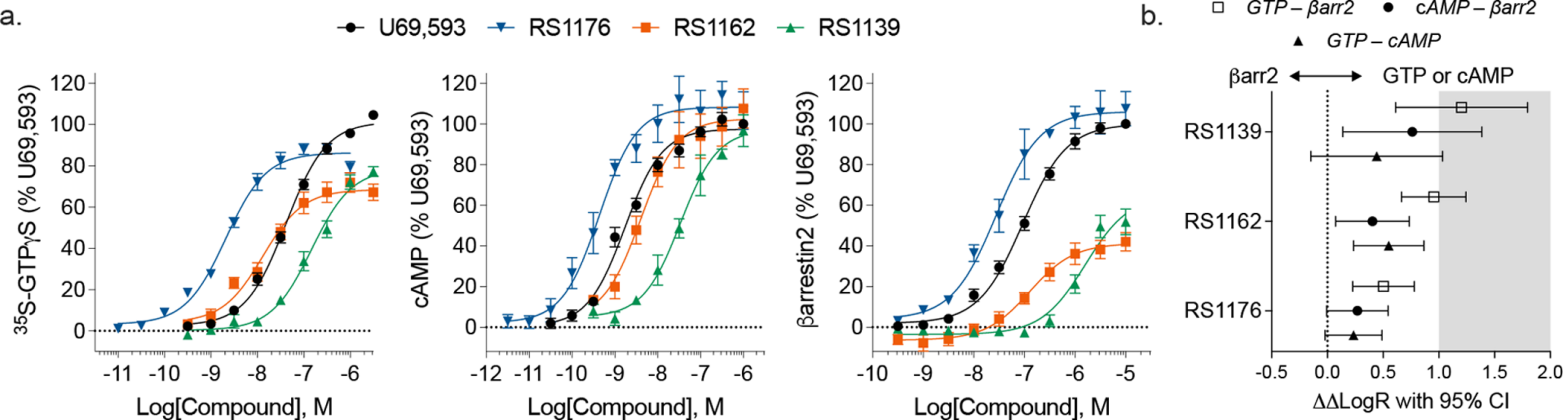
Pharmacological characterization of GB18 derivatives compared to U69,593. **a**. ^35^S-GTPγS binding; inhibition of forskolin-stimulated cAMP accumulation; and β-arrestin2 recruitment are presented as % maximal U69,593 response. Data are presented as mean ± SEM; parameters are detailed in Table 1. **b**. Calculation of biased agonism relative to U69,593 presented with 95% CI following Šídák posthoc comparison of the difference in means of the ΔLogR determined for each replicate; the gray shading indicates bias factors greater than 10 as indicated in Table 1 (*p < 0.5, **p < 0.01, ****p < 0.0001).

**Table 1:**
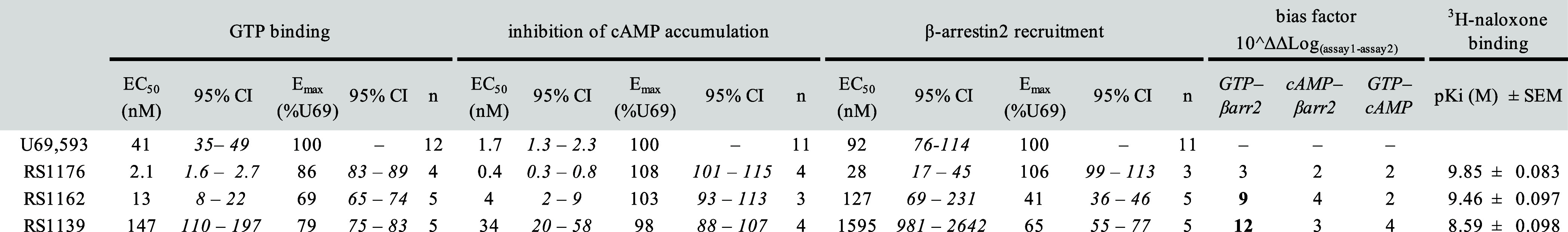
Pharmacological Parameters and Bias Analysis^a^

a Presented as mean of the curve in replicates and presented as EC_50_ with 95% CI. Radioligand binding performed with 2 nM ^3^H-Naloxone, *n* = 3; the p*K*_d_ of naloxone was determined as 8.68 *±* 0.030 M.

When administered to mice, conventional KOR agonists reduce spontaneous locomotor activity in an open field,^26–29^ which is a typical response to sedative drugs and is demonstrated by a maximum efficacious dose of U50,488H, a typical KOR agonist in Figure 6a.^22,24^ Not unexpectedly, **RS1176** dose-dependently decreases locomotor activity in adult, male C57BL6/J mice (Figure 6a, drug dose effect: *p* = 0.0019 vs vehicle, two-way ANOVA, Supplemental Table 2 for detailed statistical comparisons). Importantly, since sedative effects are mediated by the central nervous system, **RS1176** is highly likely to be brain-penetrant. In contrast, **RS1162** and **RS1139** do not produce a significant decrease (*p* > 0.05 vs vehicle) in distance traveled at any doses tested compared to the vehicle (Figure 6a, Supplemental Table 2).

**Figure 6:**
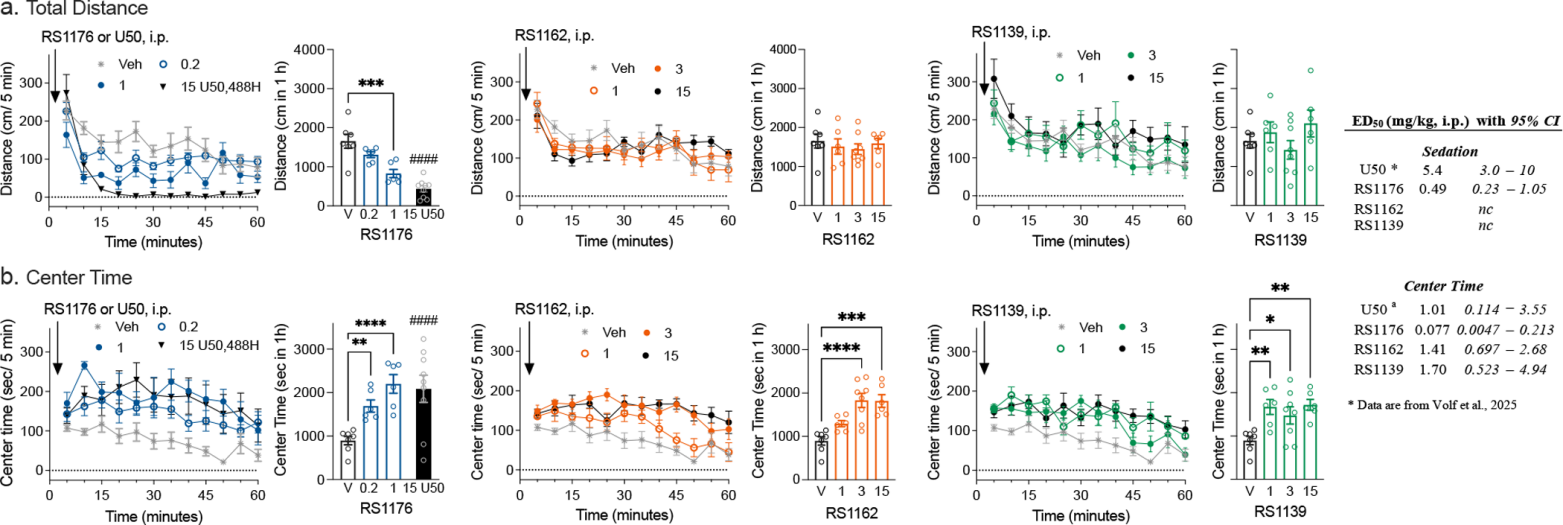
Spontaneous locomotor activity assessment in the open field in male C57BL6/J mice. **a**. The total distance traveled in male mice over 1 h is significantly decreased by **RS1176** (1 mg/kg), while **RS1162** and **RS1139** have no significant effect at any dose tested, compared to vehicle. **b**. All three KOR agonists increase the total time spent in the center of the open field over 1 h compared to vehicle. Data are presented as mean ± S.E.M; *n* = 6 to 8 mice per treatment; injections were administered intraperitoneal (i.p.), dose indicated on the *x*-axis. The sum of activity or time spent over 1 h is presented in the bar charts showing statistical comparisons of dose vs vehicle using ordinary one-way ANOVA with Dunnett’s posthoc test (*p < 0.05, **p < 0.01, ***p < 0.001, ****p < 0.0001), or an unpaired *t* test comparing U50,488H to vehicle (^####^ *p* < 0.0001); statistical comparison of the time effect plots is presented in Supplemental Table 2. The ED_50_ values are presented in the inset table, n.c. refers to not converged as no sedation was observed; *data for U50,488H are from Volf et al., 2025.^24^

In addition to sedation, traditional KOR agonists typically produce anxiogenic behaviors in mice, likely mimicking the effects of elevated dynorphin levels in the nucleus accumbens (NAc—a stress-induced neuroadaptation targeted by KOR antagonists. However, reports have emerged of selective and well-characterized KOR agonists (salvinorin A,^30^ U50,488H,^31^ U69,593 ^31^) that, at nonsedating doses, can induce anxiolytic-like behaviors in mice.^24^ Measuring the increase in the time spent in the center of an open field has been used to model such behaviors.^32^ Importantly, the open field activity monitors provide a way to measure multiple behaviors in the same animal (including sedation, which can confound interpretation of tests that measure location preferences^33^); therefore, in the same mice that we monitored total distance traveled, the time spent in the center was also determined. Administration of **RS1176** increases the time spent in the center in a dose-dependent manner (*p* = 0.0001 vs vehicle, Figure 6b). Notably, **RS1162** and **RS1139** also increase the time spent in the center in a dose dependent manner (**RS1162**: *p* = 0.0001; **RS1139**: *p* = 0.0052, Figure 6b) without decreasing the total distanced traveled (Figure 6a). The increase in the center time indicates that **RS1162** and **RS1139** are brain-penetrant and can have psychoactive effects without inducing sedation. Similar findings were recently reported for a G protein signaling-biased KOR agonist, triazole 187.^24^ These are the first studies to demonstrate behavior modification by any GB opioid analogs and show them to be potent, brain-penetrant leads for KOR-targeted therapeutics.

## Conclusion

The evaluation of anxiolytic-like behavior in mice treated with typical KOR agonists, such as U50,488H, has previously been complicated due to the sedative effects of such compounds. We have recently identified a chemically distinct KOR agonist, triazole 187, which is also a G protein signaling-biased KOR agonist; in mice, triazole 187 does not induce sedation and also promotes anxiolytic-like behaviors.^24^ While **RS1176** is a potent KOR agonist and induces an increase in the time spent in the center of the open field, this is accompanied by sedative behaviors, not unlike that observed for the conventional KOR agonist U50,488H.^24,29^ Compared to U50,488H, **RS1176** shows minimal signaling bias toward G protein pathways over β-arrestin2 recruitment. Like triazole 187, however, **RS1162** and **RS1139** are potent G protein coupling-biased KOR agonists and also increase time spent in the center of the open field without decreasing total activity, demonstrating potential anxiolytic-like effects without inducing sedation in mice. In contrast to triazole 187, **RS1162** and **RS1139** are partial agonists at KOR. The introduction of new G protein coupling-biased KOR agonists, such as **RS1162** and **RS1139**, expand our understanding and contribute to the growing literature that KOR agonists may have therapeutic potential as nonsedating anxiolytics.^24^ These compounds highlight the opportunity to avoid side effects by selectively targeting different receptor active states and the potential for new natural product (NP) scaffolds to promote these outcomes.

A common obstacle to NP use in drug development is synthetic tractability, but that barrier can be lowered by an appropriate investment in chemistry. For example, emerging sp^3^-cross-couplings can lessen the synthetic burden of high Fsp^3^ targets,^34^ as best illustrated by on-demand analogs en route toward a functional goal.^35^ The NP analogs **RS1120**-**1188** were made possible by efficient synthesis of the high Fsp^3^ GB18 core and mutually chemoselective C–C cross-coupling of its two functional groups described here. Bidirectional synthesis has been recognized an as efficient strategy in natural product synthesis, i.e. target access;^36^ its execution here by only two successive and interchangeable steps leads to very efficient library construction as well, i.e. divergent synthesis. Whereas brain penetration can often prove challenging to engineer,^37^ this study began with a brain-penetrant, selective, and potent NP lead (**1**), and its analogs retained these properties—a consequence of staying close to the chemical space of the NP starting point.^38^ These studies introduce the GB18 scaffold as a tractable new opioid ligand class with proof-of-principle for the identification of potent, KOR-selective, G protein-biased agonists.

## Supplementary Material



## Data Availability

All data are made available in the main text or the Supporting Information.

## Notes

Experimental procedures, copies of NMR spectra, X-ray structure reports, full outlines of prior syntheses and all pharmacological protocols (PDF)

## Notes

Structural parameters for X-ray crystal structures are available from the Cambridge Crystallographic Data Centre ((−)-**4**: CCDC 2168687; (+)-**4**: CCDC 2168686; (+)-**2**: CCDC 2171738; RS1122: CCDC 2248063).
