# Supplementary material for: Bidirectional Modification of a Galbulimima Alkaloid Identifies Selective Opioid Ligands
Source: ACS Cent Sci. 2025 Jul 7;11(8):1391–9. doi: 10.1021/acscentsci.5c00573 (PMC12395297; doi:10.1021/acscentsci.5c00573)
Supplement: Supplementary file 1 [file oc5c00573_si_001.pdf]

Supporting Information

Bidirectional modification of a *Galbulimima* alkaloid identifies selective opioid ligands

Florian Martin Zielke,[a,†] Stone Woo,[a,b,†] Samuel Kasmali,[a,b,†] Allison Volf,[b,c] Vuong Dang,[c] Jake B. Bailey,[d] Milan Gembicky,[d] Laura M. Bohn,[b,c,]* Ryan A. Shenvi[a,b,]*

[a]Department of Chemistry, Scripps Research, La Jolla, California 92037, United States; [b]Graduate School of Chemical and Biological Sciences, Scripps Research, La Jolla, California 92037, United States [c]Department of Molecular Medicine, The Herbert Wertheim UF Scripps Institute for Biomedical Innovation & Technology, Jupiter, Florida 33458, United States [d]Department of Chemistry and Biochemistry, University of California, San Diego, La Jolla, California 92093, United States.[†]Authors contributed equally.

*Correspondence to: laura.bohn@ufl.edu and rshenvi@scripps.edu

Table of Contents

Synthesized compound directory *(only endo attached-ring compounds are shown)* S4

General experimental S7

General purification notes for late-stage basic amine analogs S8

^{1}H NMR trends for attached-ring diasteromers S9

Synthetic procedures and compound characterization S13

Notes on enantiopure/racemic intermediates for route scouting and biological evaluation S13

Chiral SFC separation of racemic ketoiodide (4) S14

Synthesis of vinyl triflate-iodide and methyl enoate-iodide (9 and 10) S17

Syntheses of pyr-GB18 (2) and chiral SFC separation............S20

Idiosyncratic Kumada cross-coupling and cross-electrophile coupling (13a)............S26

Idiosyncratic *endo*-selective Kumada cross-coupling (13b) S29

Suzuki cross-coupling phenyl (14) S31

Suzuki cross-coupling 2-biphenyl (15)............S32

Suzuki cross-coupling 2-trifluoromethyl phenyl (16) S33

Suzuki cross-coupling 2-butoxy phenyl (17)............S34

Suzuki cross-coupling 2-furan (18) S35

Suzuki cross-coupling 3-pyrridine (19) S36

Suzuki cross-coupling 2-CO_2Me-pyridine (20)............S37

Suzuki cross-coupling 2-OMe-pyrridine (21) S38

Suzuki cross coupling O$(CH_2)_4$N-pyrridyl (22) S39

Suzuki cross coupling CF_3-pyrazole (23)............S40

Suzuki cross-coupling N-Bn-pyrazole (24)............S41

Syntheses of RS1176 (26) S42

Syntheses of RS1159 (27) S47

Syntheses of RS1160 (28) S51

Synthesis of RS1166 (29)............S55

Synthesis of RS1163 (30) S57
Synthesis of RS1165 (31) S59
Synthesis of RS1161 (32) S61
Synthesis of RS1162 (33) S63
Synthesis of RS1188 ....S65
Synthesis of RS1137 ....S67
Synthesis of RS1132 ....S71
Synthesis of RS1133 ....S74
Synthesis of RS1120 ....S77
Synthesis of RS1136 ....S81
Synthesis of RS1131 ....S85
Synthesis of RS1138 ....S87
Synthesis of RS1122 and RS1123 ....S91
Synthesis of RS1135 ....S94
Synthesis of RS1134 ....S97
Synthesis of GBFZI-110 ....S101
Synthesis of RS1130 ....S105
Synthesis of RS1121 ....S109
Synthesis of RS1187 (7m) S113
Synthesis of RS1187 (7n) S115
Synthesis of RS1129 ....S117
Synthesis of RS1139 ....S121
Synthesis of RS1128 ....S125
Synthesis of RS1125 ....S129
Exo attached-ring product as poor KOR agonist S131
Methods and materials for pharmacological experiments S132
Chemicals and drugs S132
Cell lines and cell culture S132
Radioligand binding assays S132
Signaling assays S132
Animals S133
Locomotor activity S133
Statistical and data analyses (*in vitro*) S133
Statistical and data analyses (*in vivo*) S133
Radio ligand binding S133
ED50 Calculation S133
Pharmacology Data S134

Table S1. cAMP inhibition potencies S134
Table S2. Spontaneous locomotor activity assessment in the open field S140
Figure S8. KOR agonists effects on distance travelled in the center of the open-field and horizontal activity S141
Figure S9. Radioligand competition binding in hKOR and hMOR cells S141
Table S3. MOR agonist and antagonist potencies. S142
Table S4. Radioligand displacement assay S143
PDSP's PRESTO-Tango GPCRome S144
Reference S149
NMR Spectra S150
X-ray crystallographic data S588

Synthesized compound directory *(only endo attached-ring compounds are shown)*

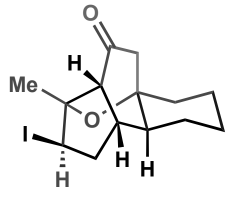

4

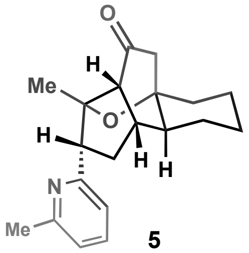

5

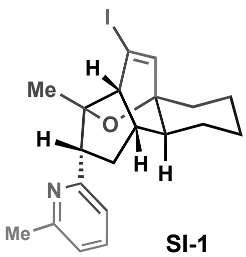

SI-1

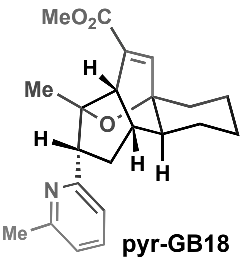

pyr-GB18

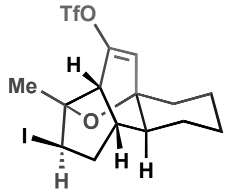

9

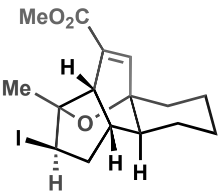

10

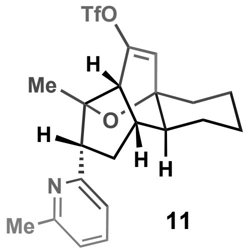

11

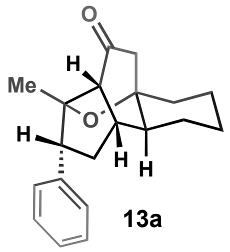

13a

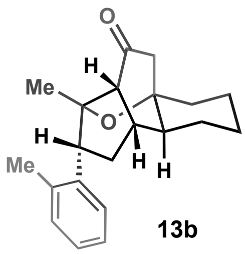

13b

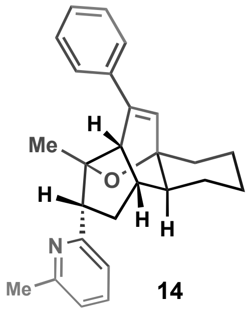

14

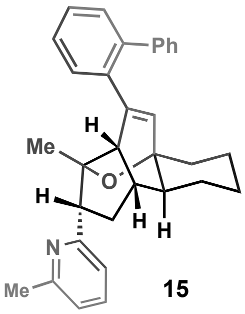

15

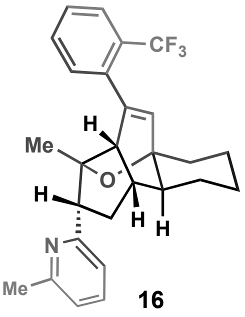

16

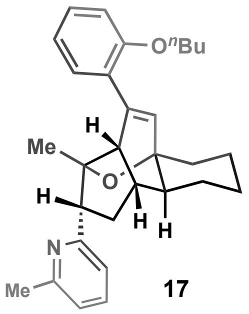

17

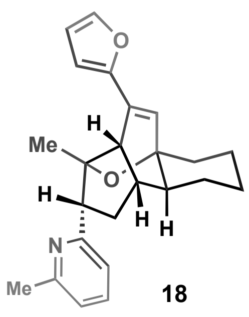

18

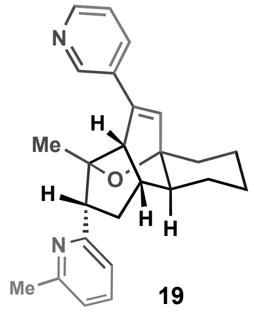

19

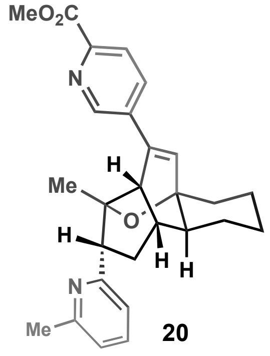

20

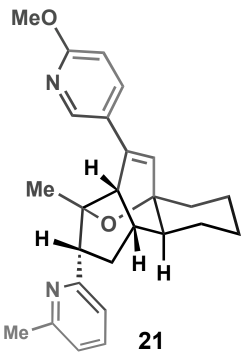

21

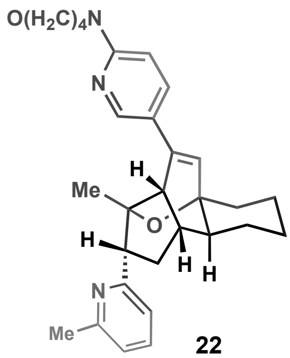

22

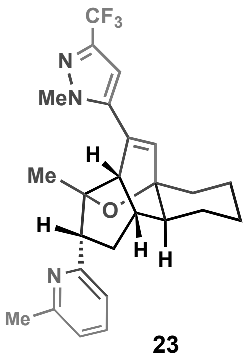

23

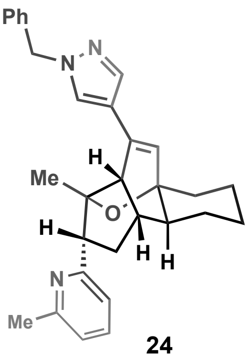

24

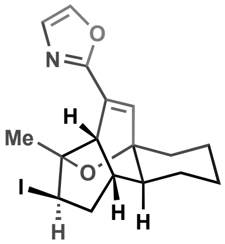

25

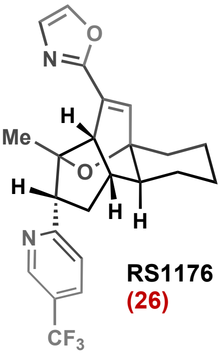

RS1176
(26)

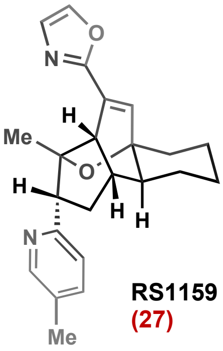

RS1159
(27)

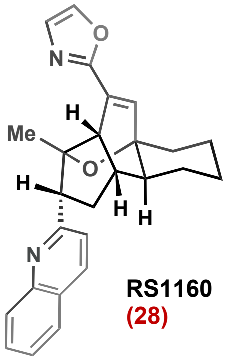

RS1160
(28)

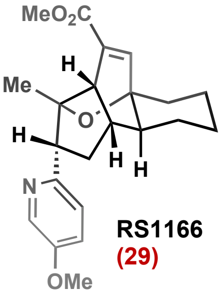

RS1166
(29)

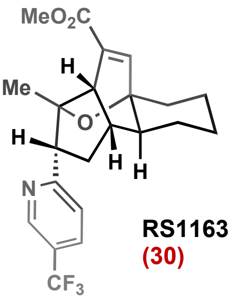

RS1163
(30)

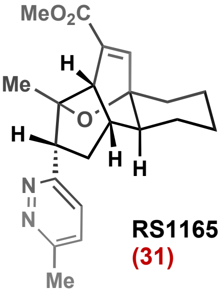

RS1165
(31)

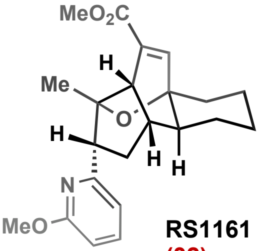

RS1161
(32)

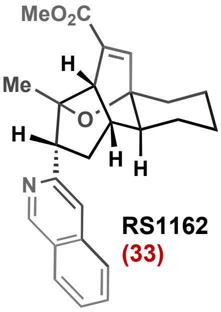

RS1162
(33)

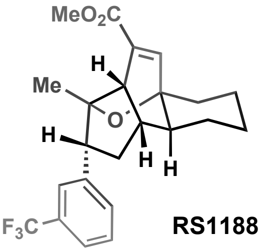

RS1188

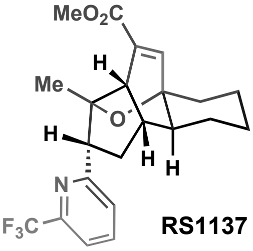

RS1137

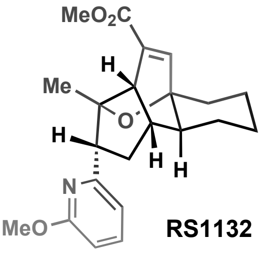

RS1132

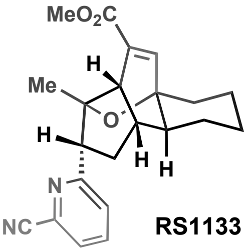

RS1133

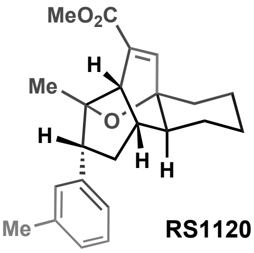

RS1120

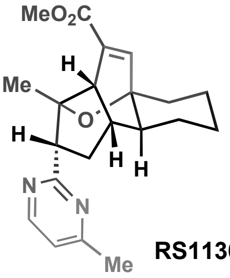

RS1136

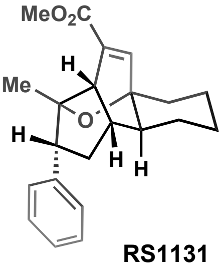

RS1131

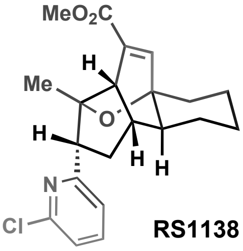

RS1138

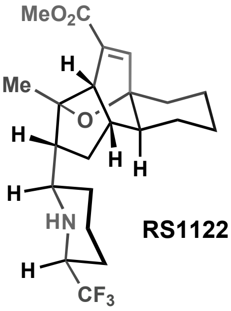

RS1122

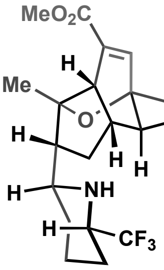

RS1123

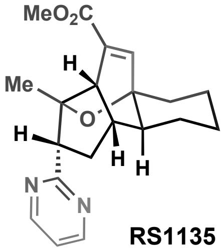

RS1135

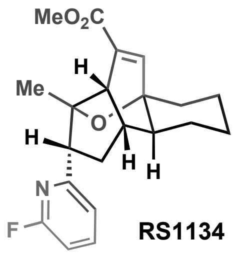

RS1134

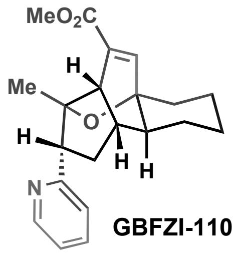

GBFZI-110

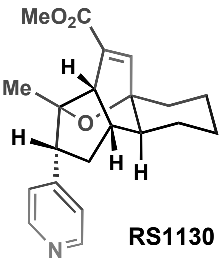

RS1130

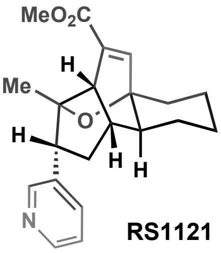

RS1121

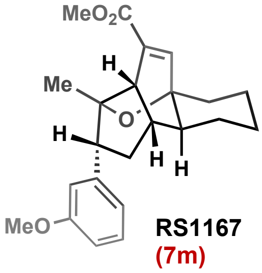

RS1167
(7m)

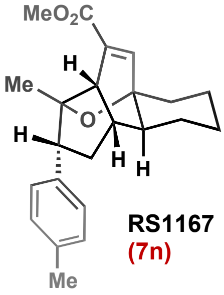

RS1167
(7n)

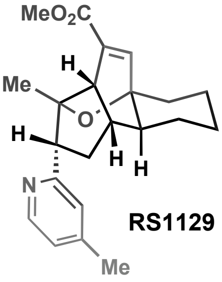

RS1129

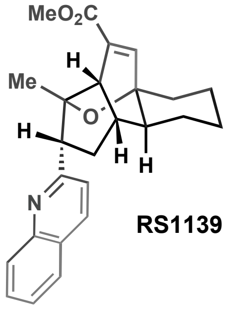

RS1139

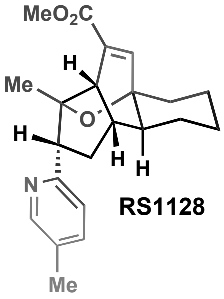

RS1128

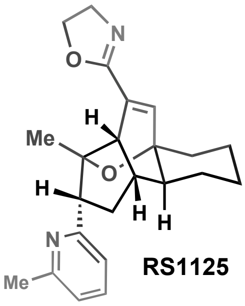

RS1125

General experimental

All reactions were carried out under positive pressure of argon unless otherwise noted. Glassware was oven-dried at 120 °C for a minimum of 12 hours, or flame-dried under vacuum (< 1 torr) unless using aqueous solvent or otherwise noted. Dry tetrahydrofuran (THF) and dichloromethane (CH_2Cl_2) were obtained by passing the previously degassed solvents through an activated alumina column, or from Sigma-Aldrich. All other solvents and reagents were obtained from commercial sources and used without further purification unless otherwise noted. Reactions were monitored by thin layer chromatography (TLC) using precoated silica gel plates from E. Merck (TLC Silica gel 60 F254, 250 μm thickness), using UV light (254 nm) as visualizing agent and an acidic ethanolic solution of *p*-anisaldehyde, or an acidic aqueous solution of ceric ammonium molybdate and ammonium molybdate (CAM), or an acidic solution of dinitrophenylhydrazine (DNP), or an aqueous solution of potassium permanganate ($KMnO_4$), and heat as developing agents. LCMS analysis was performed on Waters Acquity I-Class (SM-FTN) instruments with Waters PDA diode array detector and Waters QDa mass detector, each equipped with an ESI probe (CORTECS C18 column90 Å, 1.6 μm, 2.1 mm X 50 mm; mobile phase A, water + 0.1% formic acid; mobile phase B, acetonitrile + 0.06% formic acid). Compounds containing *N*-heterocycles could be monitored via TLCMS by isolating the desired band from analytical TLC plate (1:1 CH_2Cl_2/MeOH, syringe filtration), then subjecting acquired analytical solution to the forementioned LCMS instrument. Flash column chromatography was performed over silica gel 60 (particle size 0.04- 0.063 mm) from Fisher Scientific unless otherwise stated. Room temperature or ambient temperature for these studies was in the range 20-25 °C. NMR spectra were recorded on Bruker AVIII HD 600 MHz equipped with 5 mm CPQCI and 1.7 mm CPTCI CryoProbes, Bruker AVIII HD 600 MHz equipped with 5 mm CPDCH CryoProbe, Bruker AV NEO 500 MHz NMR equipped with 5 mm BBFO Probe, and Bruker AV NEO 399 MHz NMR equipped with 5 mm BBFO Probe, and calibrated against residual undeuterated solvent for ^{1}H NMR (δ_H($CDCl_3$) 7.26 ppm, δ_H(C_6D_6) 7.16 ppm, δ_H(CD_2Cl_2) 5.32 ppm, δ_H(DMSO-d_6) 2.50 ppm) and deuterated solvent for ^{13}C NMR (δ_C($CDCl_3$) 77.16 ppm, δ_C(C_6D_6) 128.06 ppm, δ_C(CD_2Cl_2) 53.84 ppm, δ_C(DMSO-d_6) 39.52 ppm) as an internal reference. The following abbreviations (or combinations thereof) were used to explain multiplicities: s = singlet, d = doublet, t = triplet, q = quartet, m = multiplet, b = broad, app = apparent. Optical rotations were obtained on an Anton Paar MCP100 modular circular polarimeter. Melting points were obtained on a Electrothermal 1101D Mel-Temp apparatus in open air capillaries and are uncorrected. Mass measurements for high-resolution mass spec (HRMS) were performed on a Waters Xevo G2-XS TOF calibrated against sodium formate clusters and using a LeuEnk lockmass. Expected monoisotopic masses were calculated using MassLynx 4.1 and the *m*/*z* values for calibrant and lockmass were MassLynx-default values. Quantitative ^{1}H NMR (qNMR) yield and purity determinations were performed with ns = 1, d1 = 30 s, integrating versus a known quantity of internal standard (e.g. 1,3,5-trimethoxybenzene). Isolated yields refer to > 95% pure material unless otherwise noted.

General purification notes for late-stage basic amine analogs

In general, analogs containing basic amines were purified using preparative TLC on SiO_2 plates with eluents containing 1-7% of 7N NH_3 in MeOH as the polar component, and a two-solvent non-polar component (e.g. 10-20% EtOAc/hexanes), though see also specific purification details for each compound. Triethylamine, isopropylamine, and ammonium hydroxide as basic additives typically induced very poor separation. At particularly low polarities of the non-polar component (typically lower than 10% EtOAc/hexanes) the mixed solvent system tended to phase separate slightly, and the TLC vessel (for analytical or preparative TLC) should be thoroughly shaken before running analytical or preparative TLC to minimize waviness in band shape. Due to NH_3 volatility/ adsorption to silica, eluent for TLC (analytical or preparative) should be made up immediately prior to running TLC and discarded after a plate is run to maximize reproducibility. Furthermore, calibrating the plates by running them through the desired solvent mixture prior to material loading significantly increased the reproducibility and waviness in band shape. This was also applied to analytical TLC plates.

For basic amines, oxazoles and oxazolines (and compounds containing two of these groups), pure product was recovered from TLC bands as follows unless otherwise stated. Products were eluted from silica following preparative TLC with <10% (7N NH_3 in MeOH)/CH_2Cl_2. This eluate was concentrated to dryness, then the residue (usually visibly containing traces of silica) was taken up in minimal CH_2Cl_2 and eluted through a pipette plug (2 cm) of 10% deactivated basic alumina with EtOAc. Concentration of this eluate gave pure material which consistently exhibited minimal NMR peak broadening (traces of acid, e.g. from silica or non-deacidified $CDCl_3$, significantly broadened and shifted NMR signals for basic compounds).

^{1}H NMR trends for attached-ring diasteromers

Throughout the syntheses of analogs, the bridge head protons H7 (benzylic) and H20 methyl groups has been observed to have significant difference in the two diastereomers. For *endo* compounds (e.g., ketone **7a** and vinyl triflate **SI-2**, see Figure S1 and Figure S4 respectively), the multiplicity of the bridgehead H7 is split as a doublet-of-doublet whereas the *exo* diastereomer appear as a triplet. Further, the H20 methyl group for the *endo* diastereomer is further downfield ca. 1.33 ppm. In contrast, the methyl group for the *exo* diastereomer is more upfield putatively due to the shielding by the proximal arene.

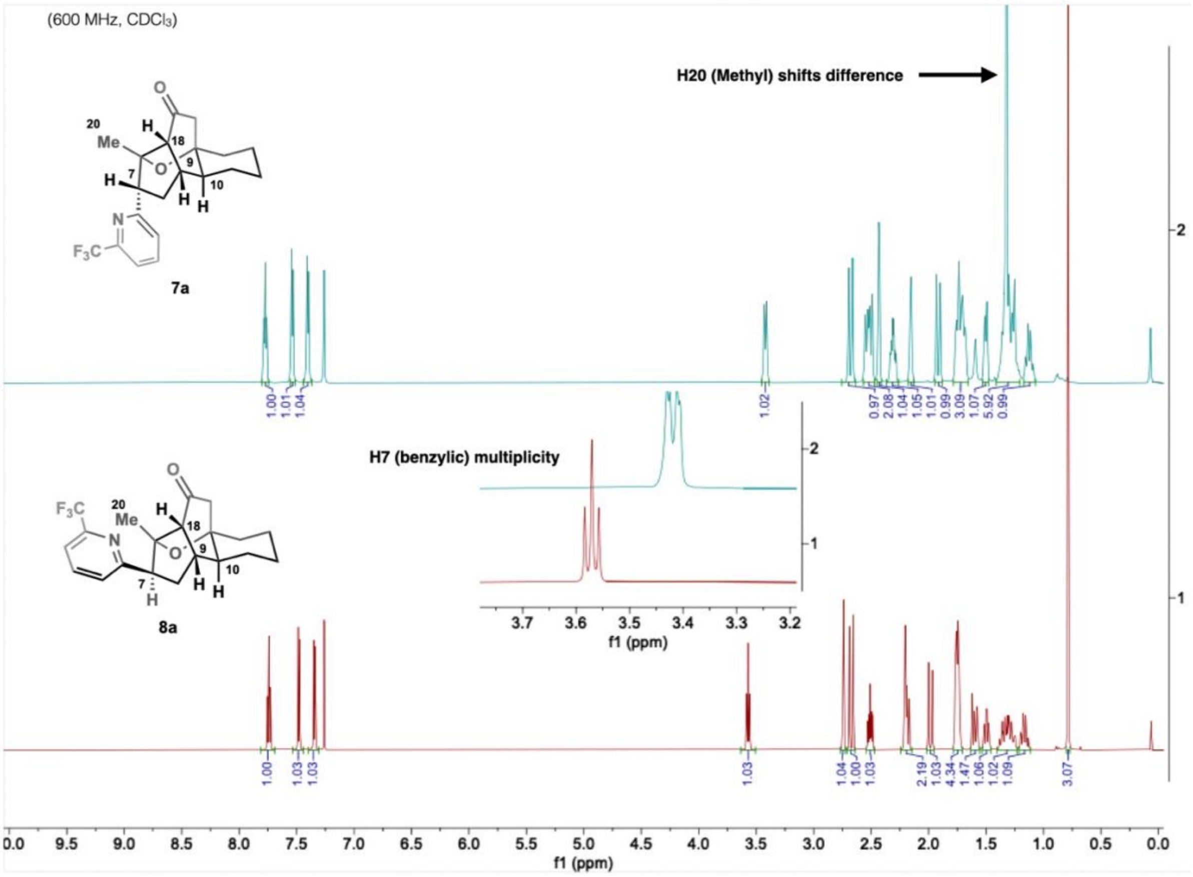

Figure S1. ^{1}H NMR comparison of ketone **7a** and **8a**

This fingerprint characteristic is consistent amongst all synthesized compounds. Though we have observed exceptions to this trend, for example ketone **7i** and **8i** (see Figure S2). However, shielding of H20 methyl groups of **7i** and **8i** are still consistent with our observation.

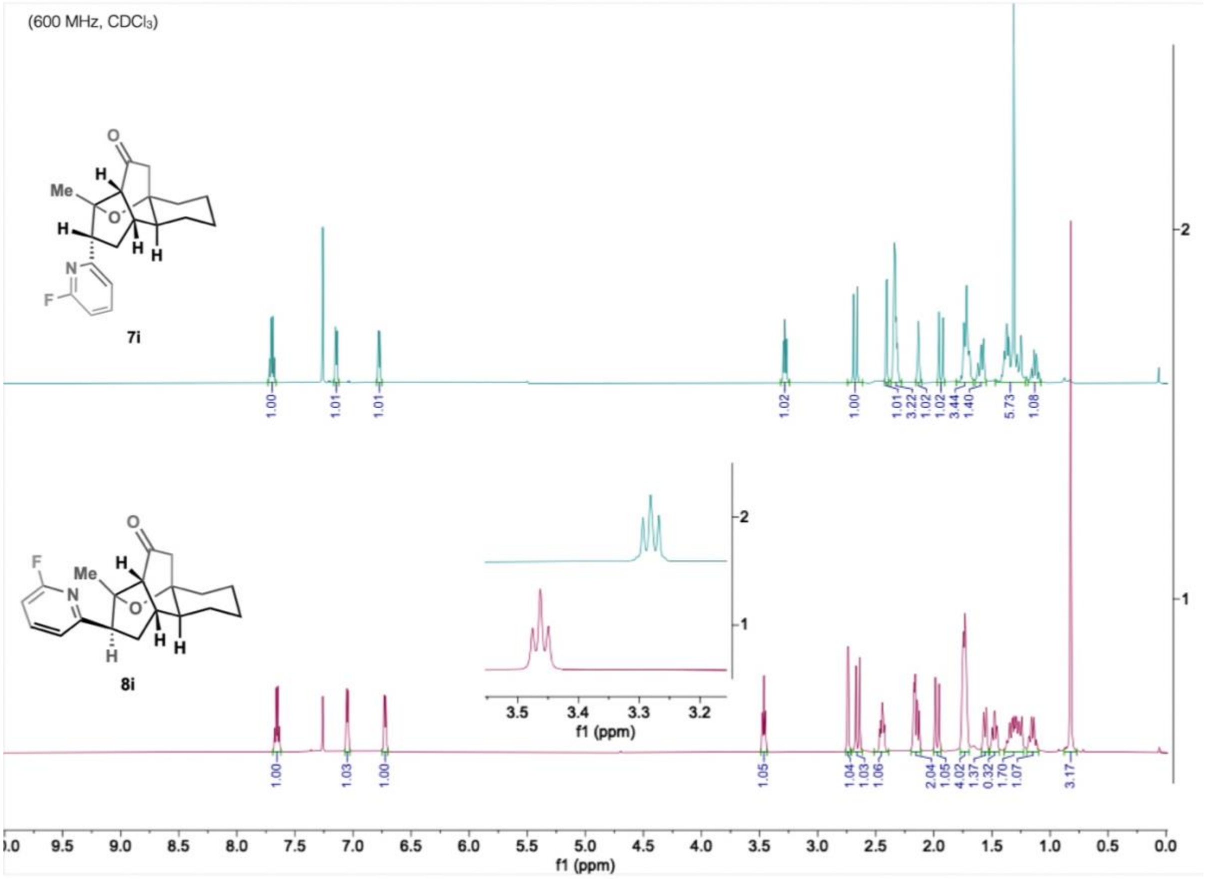

Figure S2. Ketone **7i** and **8i** show the same bridge head benzylic ^{1}H splitting

NOESY correlations between bridgehead H7 and H18 and H10 to the arene (see Figure S3) are diagnostic signs of *endo* stereochemistry. On the other hand, correlation between bridgehead H7 and H10 is indicative of *exo* stereochemistry.

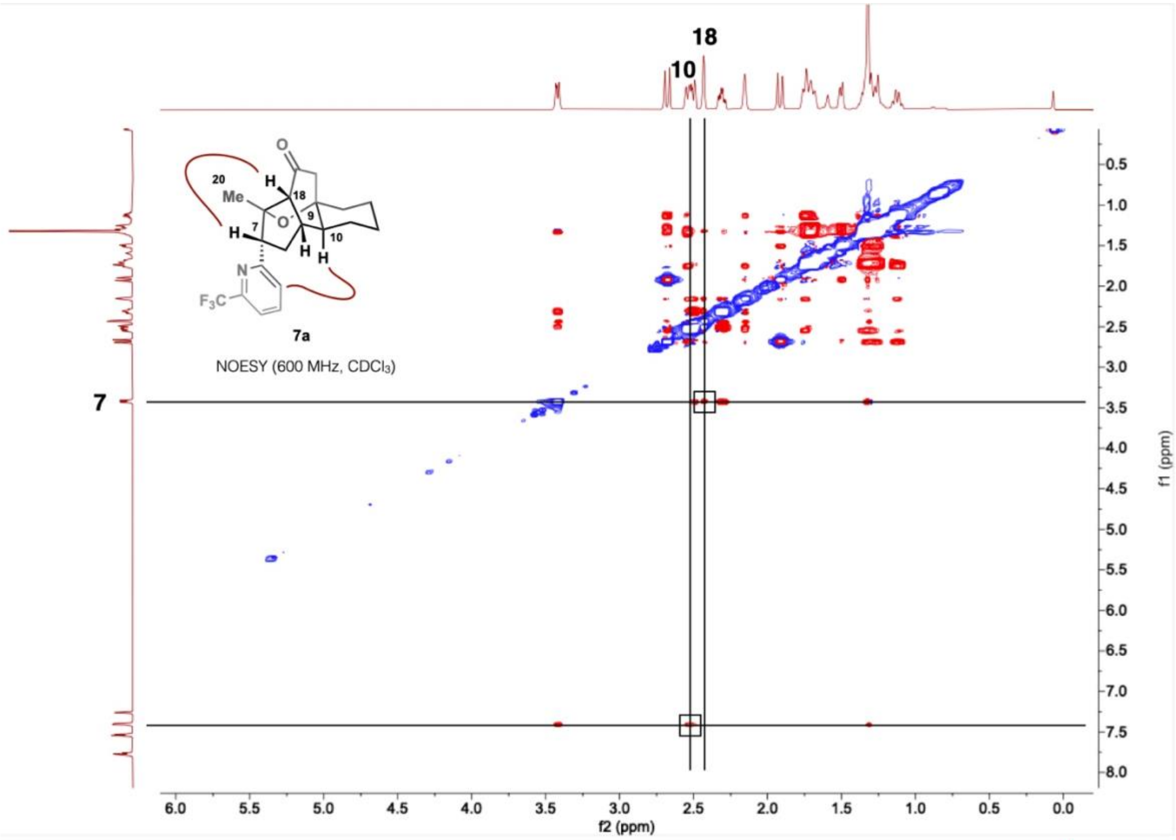

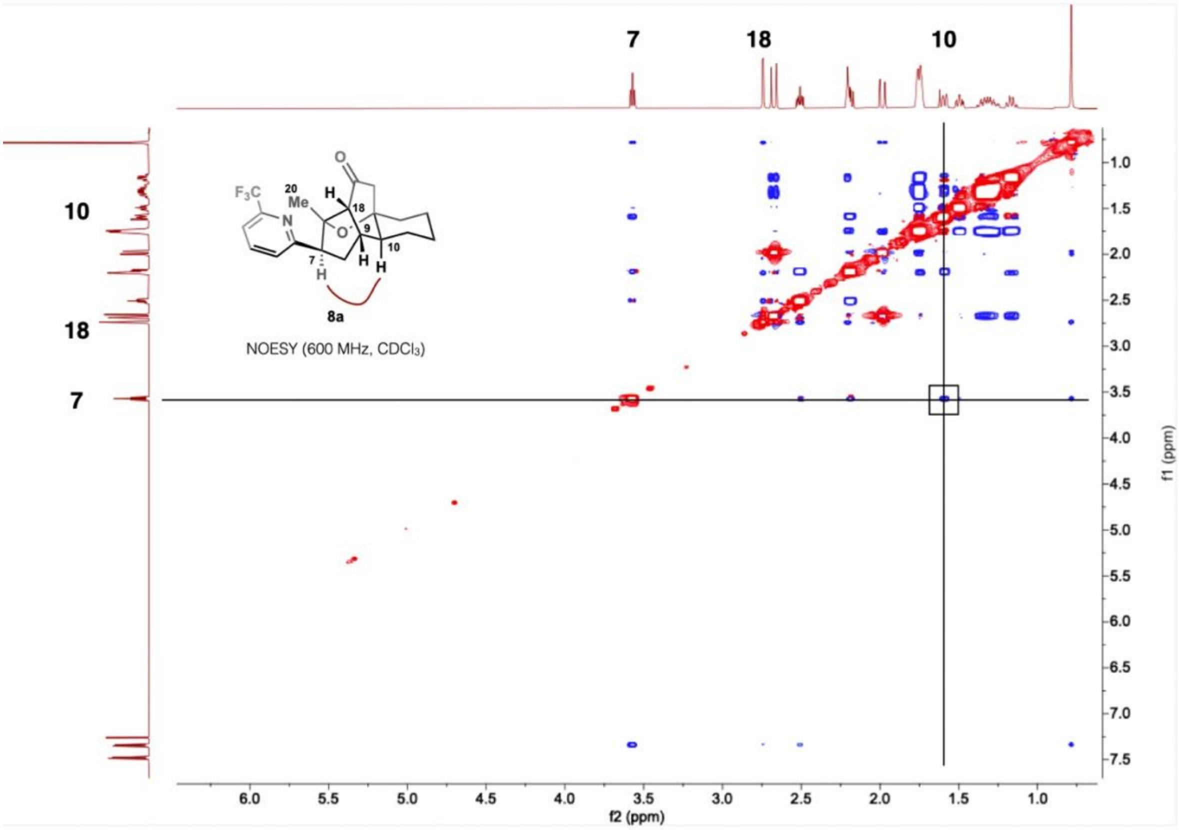

Figure S3. Key 2D NOESY correlation of ketone **7a** and **8a**

This fingerprint pattern is conserved independent of the ketone moeity (e.g., vinyl triflate **SI-2** and ***epi*-SI-2**, Figure S4).

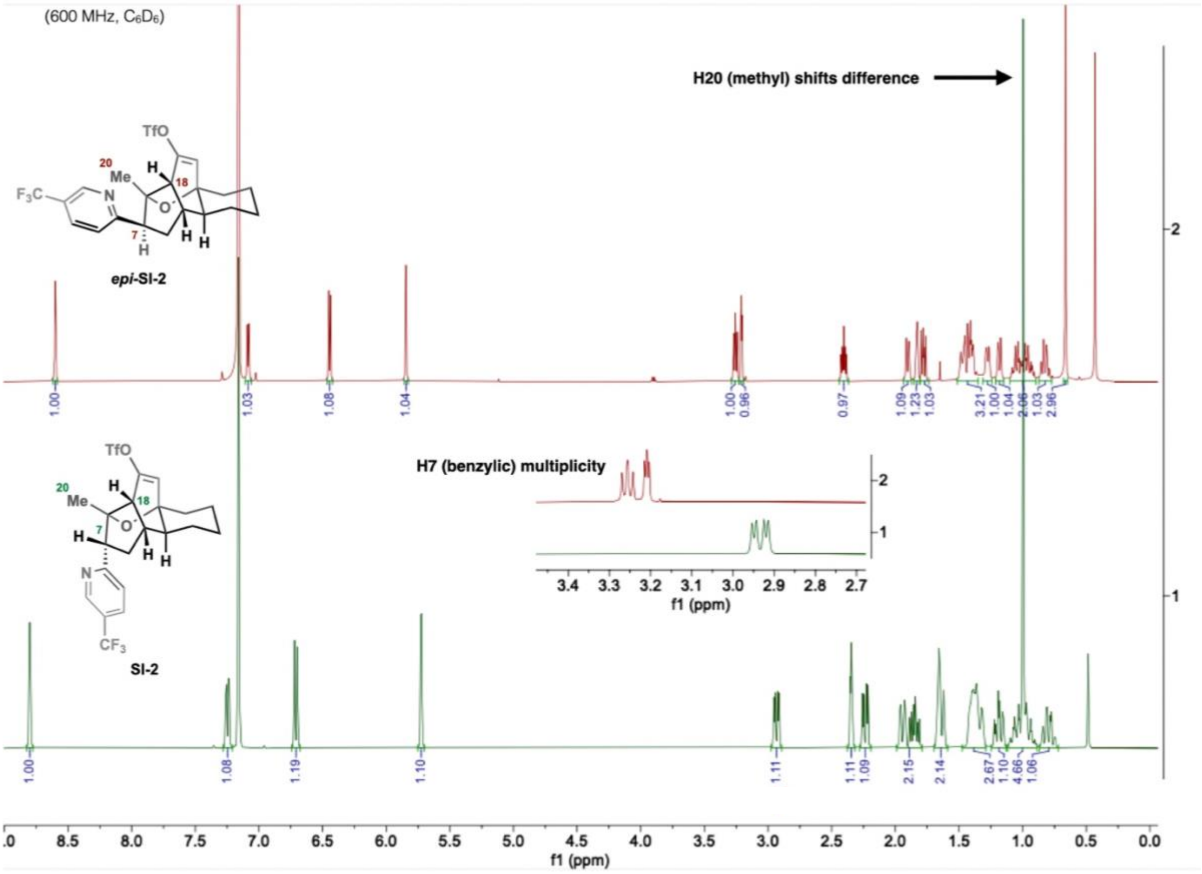

Figure S4. ^{1}H NMR comparison of vinyl triflate **SI-2** and ***epi*-SI-2**

Synthetic procedures and compound characterization

Notes on enantiopure/racemic intermediates for route scouting and biological evaluation

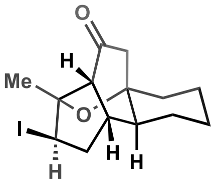

(–)-**4**
nat
[SFC separation]

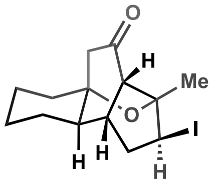

(+)-**4**
ent
[SFC separation]

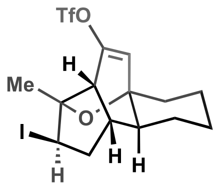

(–)-**9**
nat
[from enantiopure *(–)*-**4**]

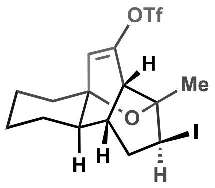

(+)-**9**
ent
[from enantiopure *(+)*-**4**]

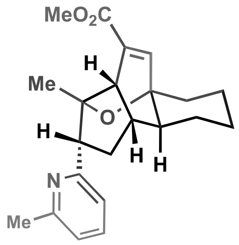

(–)-**2**
nat-pyr-GB18
[SFC separation]

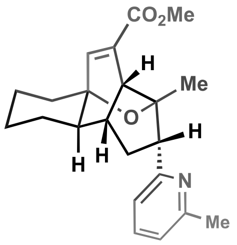

(+)-**2**
ent-pyr-GB18
[SFC separation]

Throughout these studies, racemic ketoiodide *rac*-**4**, *ent*-ketoiodide *(+)*-**4**, racemic vinyl triflate-iodide *rac*-**9**, *ent*-vinyl triflate-iodide *(+)*-**9**, racemic methyl enoate-iodide *rac*-**10**, *ent*-methyl enoate-iodide *(+)*-**10**, racemic pyr-GB18 *rac*-**2**, *ent*-pyr-GB18 *(+)*-**2** and their derivatives were used extensively as model systems to test reactivity. Material used for biological evaluation was exclusively derived from enantiopure *nat*-ketoiodide *(−)*-**4**, *nat*-methyl enoate-iodide *(−)*-**10**, or *nat*-pyr-GB18 *(−)*-**2** purified by preparative chiral SFC.

Chiral SFC separation of racemic ketoiodide (4)

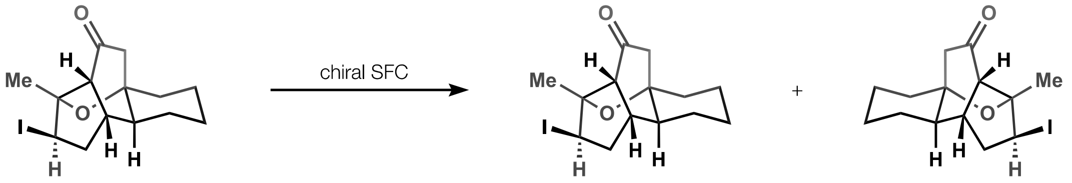

4 racemic | *(–)*-**4** nat | *(+)*-**4** ent

Racemic ketoiodide (**4**) was prepared as per our previous report.[1] The chiral separation procedure has been reproduced on scales between 6 – 9 g of racemate; a representative procedure follows. Due to the metastability of **4**, care should be taken throughout the purification to minimize exposure to light and duration of the material exposed to protic solvent (MeOH), particularly at high concentrations of **4**.
6.7g of recrystallized racemic ketoiodide **4** was subjected to prep SFC. The racemate was separated on a Waters Prep SFC 150 AP using a Diacel IG column (5 µm, 19x250 mm). The purification was run under isocratic conditions (35% MeOH / CO_2, 100 mL/min, 1600 psi backpressure) at 40 °C. Fractionation was triggered by UV light (220 nm). 2 x 3.0 g solid portions were each transferred into separate 21 mL scintillation vials, and dissolved in 19 mL PhMe/MeOH (2:1) immediately prior to injection. Remaining racemic sample was made up to same concentration for injection. (NB: this concentration is close to saturation in this solvent system; *brief* sonication helps dissolution.) Final concentration of injection sample is ~157 mg/mL (900 µL) injections which seems to give slightly poorer separation, relative to a smaller scale run with ~150 mg/mL (900 µL) injections, of a trace +203 impurity on the tail of the first product +347 peak, so a slightly more dilute injection sample may yield better results. Ca. 50 injections (900 µL each) were performed; the separation is done within a day.

Separated samples were concentrated within a few hours of SFC separation on a rotavap @ ≤ 30 °C and dried via PhMe azeotrope prior to storage. All manipulations were performed with minimal sample exposure to light (most lights turned off in vicinity of preparative SFC apparatus which was covered with foil, hood lights off, rotavap covered with foil).

Analytical SFC analysis (Figure S5) indicated >99% ee of separated enantiomers. Fast- and slow-moving enantiomers were assigned as *nat-(−)*-**4** and *ent-(+)*-**4** respectively based on single crystal X-ray diffraction (*vide infra*). NMR spectra for separated enantiomers were identical to those for the racemate.[1]
Pure, crystalline *(−)*-**4** and *(+)*-**4** can be stored under Ar at -20 °C in the absence of light for many months without appreciable degradation.

<u>Characterization data for ketoiodide *(−)*-**4**</u>
Description white crystals
optical rotation $[\alpha]_D^{20} = -63.7$ (c = 0.95, EtOAc)
mp 86 – 88 °C
X-ray crystals grown by slow evaporation from C_6H_6 (CCDC 2168687)

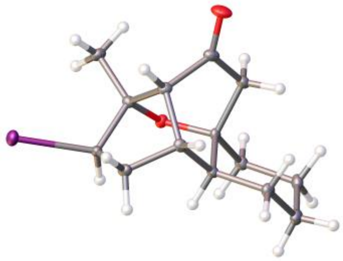

<u>Characterization data for ketoiodide *(+)*-**4**</u>

optical rotation $[\alpha]_D^{20}$= +63.2 (c = 1.14, EtOAc)
X-ray crystals grown by vapor diffusion of heptane against a solution of *(+)*-**4** in $^{i}Pr_2O$ (CCDC 2168686)

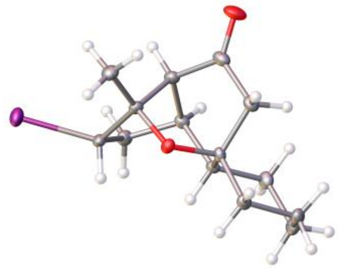

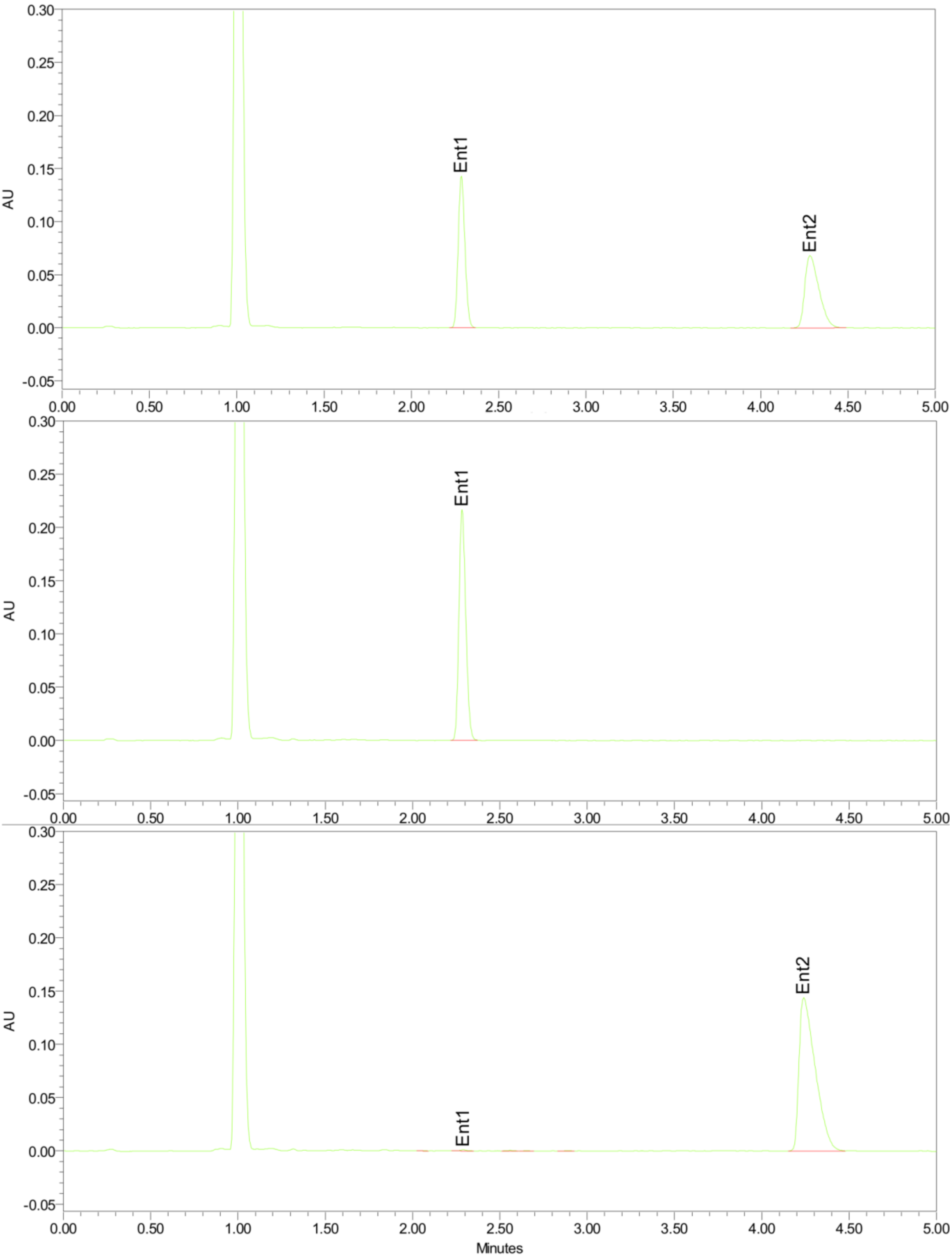

Figure S5. UV traces at 220 nm for SFC analysis of *(+)*-**4** (bottom, >99% ee), *(−)*-**4** (middle, >99% ee) and recrystallized *rac*-**4** (top). Peak at ca. 1.0 min is derived from PhMe loading solvent.

Synthesis of vinyl triflate-iodide and methyl enoate-iodide (9 and 10)

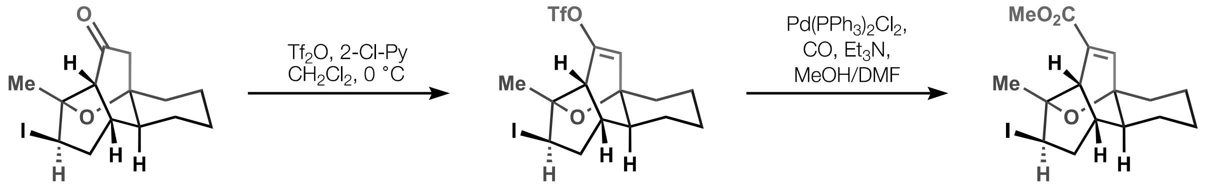

(–)-**4** *(–)*-**9** *(–)*-**10**

Triflation Enantiopure *(−)*-**4** (692 mg, 2.00 mmol) was charged to a flame-dried 100 mL round-bottom flask equipped with rubber septum and placed under Ar atmosphere (3x evacuate/backfill cycles). Anhydrous CH_2Cl_2 (4 mL) and 2-chloropyridine (700 µL, 7.40 mmol) were added. The reaction was cooled in an ice bath and Tf_2O (1 mL, 6.00 mmol) was added dropwise over 10 min (see note 1). After 10 min further stirring following complete addition of Tf_2O, the reaction was allowed to warm to rt and stirred for 30 min. TLC analysis indicated trace amounts of starting material remaining relative to product (see note 2). The reaction was cooled to 0 °C, then quenched with saturated aqueous $NaHCO_3$ (20 mL) (see note 3), diluted with saturated aqueous Na_2SO_3 (20 mL), and extracted with ether (3x). The combined organics were washed with water (3x) and brine, dried over Na_2SO_4 and concentrated to dryness under a stream of nitrogen. The residue (765 mg) was filtered through a SiO_2 plug (3 cm height x 3 cm diameter, Et_2O loading and elution) and the eluate concentrated to dryness. The hexanes-soluble residue obtained was purified by column chromatography (SiO_2, 22 cm x 3 cm, 0-4% EtOAc/hexanes) to give vinyl triflate-iodide *(−)*-**9** (714 mg, 75%) (see note 4). [Enantiopure *(+)*-**9** and racemic *rac*-**9** were obtained in similar fashion from the corresponding *(+)*-**4** and racemic *rac*-**4** respectively.]

Note 1 − The quality of Tf_2O is very important for this reaction; if old bottles were used large excesses of Tf_2O/2-Cl-pyridine were also required, which reduced the reaction's practicality on scale. Other bases (Hunig's base: decomposition; pyridine: no conversion; 2,6-di-*tert*-butyl-4-methylpyridine (with and without added DMAP): trace conversion; 2,6-di-*tert*-butylpyridine: modest conversion) gave poorer results compared to 2-Cl-pyridine (full conversion) on small scale.
Note 2 − A brown wet-looking stain/film/residue was observed forming on the neck of the flask near the rubber septum, possibly due to corrosion of the rubber by trace TfOH; minimizing the incorporation of this material into the reaction mixture may increase product purity and/or stability following chromatography.
Note 3 − Saturated aqueous $NaHCO_3$ was added dropwise at first due to vigorous bubbling from the evolution of CO_2 gas. If NaHCO3 was added too quickly, reaction mixture could overspill from the vessel.
Note 4 − This material exhibits sensitivity to ambient atmosphere and light, particularly when neat and when trace impurities are present. Material can be stored frozen in benzene at -20 °C away from light without appreciable decomposition over at least several weeks and concentrated to dryness immediately before use.

Carbonylation Vinyl triflate-iodide *(−)*-**9** (601 mg, 1.26 mmol) and $Pd(PPh_3)_2Cl_2$ (21.0 mg, 0.03 mmol) were charged to a flamed 250 mL round-bottom flask equipped with large egg-shaped stir-bar (see note 6). The headspace was purged with CO (caution: toxic! perform all procedures with CO in a well-ventilated fume hood) from a 3-layer balloon equipped with a 18G 3'' needle (3x evacuate/backfill cycles) (see note 5). Anhydrous DMF (pre-sparged with CO for 20 min, 4 mL) was added and the mixture was slurried. In rapid succession, MeOH

(pre-sparged with CO for 20 min, 4 mL) and Et_3N (1.76 mL) were added and the reaction headspace was purged with CO (20G vent needle) with rapid stirring. After 10 min, the vent needle was removed and the reaction flask was placed in a 50 °C oil bath. After 2.5 h, TLC analysis indicated approximately half conversion, and the reaction was heated to 54 °C. After 9 h at this temperature, replacing the CO balloon as necessary to maintain elevated partial pressure of CO, TLC analysis indicated trace amounts of starting material remaining (see note 6). The reaction was allowed to cool to room temperature and concentrated to dryness under a stream of nitrogen, giving a clear, dark oil. The crude oil (insoluble in PhMe or Et_2O) was loaded (CH_2Cl_2) onto a pre-equilibrated (Et_2O) SiO_2 plug (3 cm x 2.5 cm) and eluted with Et_2O (100 mL). The eluate was concentrated to near-dryness (small amount of Et_2O remaining assists loading) and the residue purified by column chromatography (SiO_2, 20 cm x 4 cm, PhMe loading, 0-5% EtOAc/hexanes). Fractions containing minimal impurities by TLC were pooled and evaporated. The product was recrystallized from minimal Et_2O at 0 °C and washed (3x) with minimal Et_2O/pentane (1:1) at 0 °C to afford pure *(−)*-**10** (320 mg, 65%) (see note 7) . The washings were slowly evaporated to afford pale yellowish crystals (96 mg, 20%) of lesser purity.

Note 5 – In other runs, the balloon may get deflated during the reaction timescale. It is recommended to use 5-layer CO balloon. However, reaction yields were stable regardless the layering amount.
Note 6 – A larger scale run (1.28 g *(+)*-**9**) with 5 mol% $Pd(PPh_3)_2Cl_2$ at 50 °C for 6 h gave full conversion of starting material and similar yields to the procedure above (82-90% including mixed fractions) but also produced small amounts of protodehalogenation byproduct and alkyl iodide epimerization (*endo*-I) byproduct (*m/z* = +389 $[M+H]^+$, +371 $[M+H-H_2O]^+$, inconsistent with potential Cl-I stereoinvertive displacement; exhibited similar NMR spectra [representative δ(^{1}H) (600 MHz, $CDCl_3$) 4.30 (dd, J = 10.9, 3.6 Hz, 1H), 3.12 (dd, J = 4.1, 1.6 Hz, 1H), 2.74 (ddd, J = 14.3, 10.9, 5.5 Hz, 1H)] to the analogous formal Br-I stereoinvertive displacement product [representative δ(^{1}H) (400 MHz, $CDCl_3$) 4.21 (dd, J = 10.7, 3.5 Hz, 1H), 3.06 (dd, J = 3.9, 1.7 Hz, 1H), 2.69 (ddd, J = 14.4, 10.7, 5.6 Hz, 1H)] which was formed by treatment of *(+)*-**9** with LiBr/DMSO at 120 °C [10% yield, *endo*-Br stereochemistry confirmed by NOESY] then vinyl triflate carbonylation) which were copolar with each other and difficult to separate from desired product.
Note 7 – Methyl enoate-iodide **10** exhibited greater qualitative stability than the other two alkyl iodide intermediates **4**/**9** (as racemates or either enantiomer); material can be left under ambient conditions for a couple days without noticeable degradation. Prolonged storage under Ar at -20 °C in the dark is advised.

<u>Characterization data for *rac*-vinyl triflate-iodide *rac*-**9**</u>
Description colorless oil, freezes at -20 °C into a white gum
TLC R_f = 0.54 (SiO_2, 20% EtOAc/hexanes); UV-active, stains indigo in anisaldehyde
^{1}H NMR (600 MHz, C_6D_6) δ 5.67 (d, J = 2.6 Hz, 1H), 4.07 (dd, J = 8.3, 7.3 Hz, 1H), 2.50 (dd, J = 4.0, 2.6 Hz, 1H), 2.07 (ddd, J = 13.8, 7.3, 5.6 Hz, 1H), 1.89 (dd, J = 13.7, 8.3 Hz, 1H), 1.77 – 1.68 (m, 1H), 1.43 (s, 3H), 1.41 – 1.31 (m, 1H), 1.30 – 1.21 (m, 2H), 1.21 – 1.12 (m, 1H), 1.00 (ddq, J = 12.7, 4.0, 2.0 Hz, 1H), 0.90 (qt, J = 13.4, 3.7 Hz, 1H), 0.80 (qq, J = 13.2, 4.4, 3.9 Hz, 1H), 0.72 (ddd, J = 13.1, 3.9, 2.4 Hz, 1H), 0.59 (qd, J = 13.0, 3.6 Hz, 1H).
^{13}C NMR (151 MHz, C_6D_6) δ 148.3, 121.5, 119.1 (q, J = 320 Hz), 84.3, 75.9, 52.3, 48.3, 46.0, 41.5, 33.8, 32.2, 31.5, 27.6, 25.5, 23.2.
^{19}F NMR (376 MHz, C_6D_6) δ -76.5.
2D NMR HSQC, HMBC, COSY
HRMS Calculated $C_{15}H_{17}F_3IO_3S$ $[M-OH]^+$ 460.9895 | Found: 460.9892

Characterization data for *nat*-vinyl triflate-iodide *(−)*-**9**
Optical rotation $[\alpha]_D^{20}$ = −63.8 (c = 0.65, EtOAc)

Characterization data for *ent*-vinyl triflate-iodide *(+)*-**9**
Optical rotation $[\alpha]_D^{20}$ = +67.9 (c = 0.64, EtOAc)

Characterization data for *rac*-methyl enoate-iodide *rac*-**10**
TLC R_f = 0.47 (SiO_2, 20% EtOAc/hexanes); UV-active, stains purple in anisaldehyde
^{1}H NMR (600 MHz, C_6D_6) δ 7.21 (d, *J* = 1.7 Hz, 1H), 4.23 (dd, *J* = 8.3, 7.3 Hz, 1H), 3.45 (s, 3H), 3.43 (dd, *J* = 4.0, 1.7 Hz, 1H), 2.25 (ddd, *J* = 13.6, 7.3, 5.6 Hz, 1H), 1.99 (dd, *J* = 13.5, 8.3 Hz, 1H), 1.94 – 1.81 (m, 1H), 1.51 (s, 3H), 1.46 – 1.40 (m, 1H), 1.32 – 1.20 (m, 2H), 1.12 – 1.02 (m, 2H), 1.02 – 0.94 (m, 1H), 0.90 – 0.79 (m, 2H), 0.43 (qd, *J* = 13.1, 3.7 Hz, 1H).
^{13}C NMR (151 MHz, C_6D_6) δ 165.1, 145.0, 133.1, 83.9, 73.9, 52.0, 51.5, 46.6, 44.1, 41.1, 33.8, 33.5, 32.0, 27.7, 25.7, 23.3.
2D NMR HSQC, HMBC, COSY, NOESY
HRMS Calculated $C_{16}H_{20}IO_2$ $[M-OH]^+$ 371.0508 | Found: 371.0513

Characterization data for *nat*-methyl enoate-iodide *(−)*-**10**
Description white crystalline solid
mp 97 – 99 °C
optical rotation $[\alpha]_D^{20}$ = −84.5 (c = 0.37, EtOAc)
^{1}H NMR (600 MHz, $CDCl_3$) δ 7.21 (d, *J* = 1.7 Hz, 1H), 4.34 (t, *J* = 7.7 Hz, 1H), 3.82 (s, 3H), 3.28 (dd, *J* = 4.2, 1.6 Hz, 1H), 2.55 – 2.40 (m, 2H), 2.01 (dt, *J* = 12.2, 3.5 Hz, 1H), 1.84 (d, *J* = 13.9 Hz, 1H), 1.61 (d, *J* = 13.5 Hz, 1H), 1.53 – 1.42 (m, 3H), 1.39 (td, *J* = 13.3, 12.9, 4.0 Hz, 1H), 1.31 (s, 3H), 1.25 (dt, *J* = 13.2, 3.3 Hz, 1H), 1.18 (qt, *J* = 13.1, 3.9 Hz, 1H), 0.73 (qd, *J* = 13.2, 3.8 Hz, 1H).

Characterization data for *ent*-methyl enoate-iodide *(+)*-**10**
optical rotation $[\alpha]_D^{20}$ = +85.7 (c = 0.52, EtOAc)

Syntheses of pyr-GB18 (2) and chiral SFC separation

Procedure A:

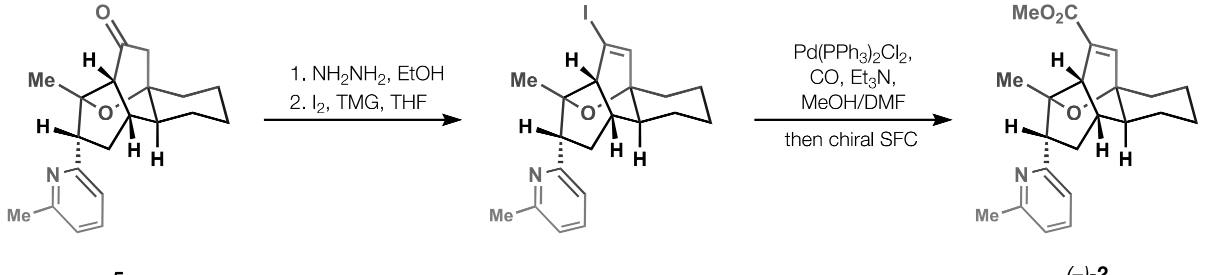

5 racemic **SI-1** (–)-**2** *nat*-Pyr-GB18

The ketone to vinyl iodide sequence from ref. 1 was adapted. Racemic *endo*-ketopyridine **5** was prepared as per ref. 1.

Hydrazone condensation In a 1 dram vial, racemic *endo*-pyridine **5** (25 mg, 0.08 mmol) was treated with EtOH (1.6 mL) and hydrazine hydrate (78 µL, 1.6 mmol) at 40 °C for 12 h. The reaction mixture was blown down under a stream of nitrogen and dried via PhMe azeotrope repeatedly (3x) to afford a white solid which was used for the next step without further purification.

Barton iodination To a flamed culture tube (Fisherbrand 14-957-76A, 100 mm length, 8 mL volume) under Ar with septum-lined screw cap (Chemglass CG-4910-15) was added iodine (45.0 mg, 0.18 mmol) and anhydrous, inhibitor-free THF (200 µL). The resulting dark red-brown solution was placed in an ice bath and 1,1,3,3-tetramethylguanidine (703 µL, 0.176 mmol) was added dropwise. A solution of the mixture of crude hydrazones from the previous reaction in THF (200 µL) was added dropwise, quantitating the transfer with THF (2 x 200 µL). Upon complete transfer of the mixture of crude hydrazones, the reaction turned clear yellow, and the ice bath was removed. After 40 min, the reaction was cooled to 0 °C and quenched with a mixture of saturated Na_2SO_3 (1 mL), saturated $NaHCO_3$ (1 mL) and water (1 mL). The mixture was extracted with EtOAc (3x) and the combined organics were dried over Na_2SO_4 and concentrated under reduced pressure giving a crude residue (285 mg) containing significant tetramethylguanidine. The crude residue was loaded (PhMe, quantitate with minimal CH_2Cl_2) onto a silica plug (3 cm x 15 mm) pre-equilibrated with Et_2O, and eluted with Et_2O to afford a solid residue (34 mg). Column chromatography (SiO_2, PhMe loading, 0-10% EtOAc/hexanes) afforded racemic vinyl iodide **SI-1** (33 mg, 98%).

Carbonylation Racemic vinyl iodide **SI-1** (31.0 mg, 0.075 mmol) from above was transferred to a culture tube (Fisherbrand 14-957-76A, 100 mm length, 8 mL volume) with minimal CH_2Cl_2 and dried via PhMe azeotrope (2x). An egg-shaped stir bar (should span most of the inner diameter of tube for optimal stirring) and $Pd(PPh_3)_2Cl_2$ (1.0 mg, 0.002 mmol) was added and the headspace was purged with CO (caution: toxic! perform all procedures with CO in a well-ventilated fume hood) from a 3-layer balloon equipped with a 22G 3'' needle (3x evacuate/backfill cycles). Anhydrous DMF (pre-sparged with CO for 20 min, 350 µL) was added and the mixture was slurried. In rapid succession, MeOH (pre-sparged with CO for 20 min, 350 µL) and Et_3N (105 µL) were added and the reaction headspace was purged with CO (20G vent needle) with rapid stirring. After 10 min, the vent needle was removed (reaction had clear pale-yellow appearance) and the reaction tube was placed in a 50 °C heating block and stirred at 1200 rpm under CO. After 2 h, the reaction appeared pale cherry-red. The reaction mixture was concentrated to dryness under a stream of nitrogen, and then dried via PhMe

azeotrope. The crude residue was loaded with minimal PhMe/a few drops of CH_2Cl_2 onto a silica plug (3 cm x 15 mm) pre-equilibrated with Et_2O, then eluted with Et_2O. The eluate was concentrated and further purified by two rounds of column chromatography (SiO_2, PhMe loading, 0-10-12-15% EtOAc/hexanes) to afford racemic pyr-GB18 **2** (19 mg, 72%) which was subjected to preparative SFC.

Procedure B:

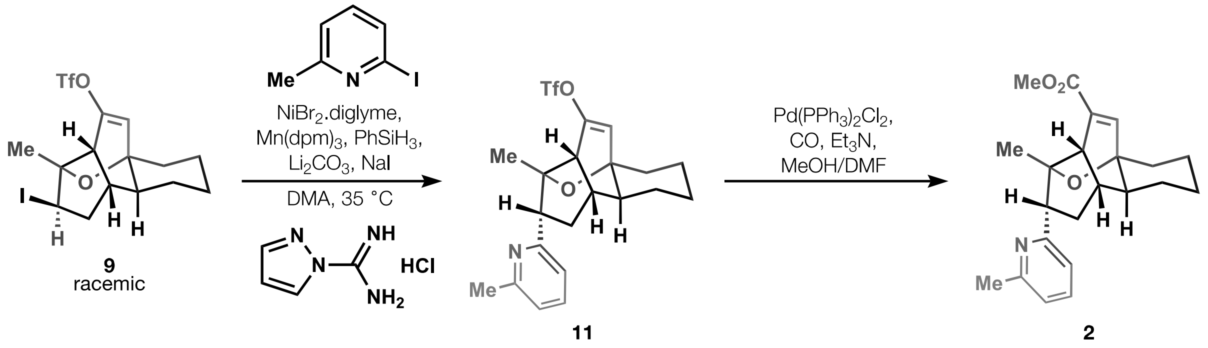

Preparation and notes for cross-electrophile coupling NaI (Oakwood Chemical) was dried in a 110 °C oven for at least 12 h before use. Li_2CO_3 (Sigma-Aldrich) was dried in a 110 °C oven for at least 12 h before use. $NiBr_2$·diglyme (Sigma-Aldrich) was used as received, and stored under argon atmosphere. $Mn(dpm)_3$ was synthesised and purified according to ref. S4. 1*H*-Pyrazole-1-carboxamidine, HCl salt (Combi-Blocks), was used as received. *N*,*N*-dimethylacetamide (DMA) (anhydrous, 99.9%, Sigma-Aldrich) was degassed by sparging with argon in an ultrasonication bath for at least 40 min prior to use for cross-electrophile coupling.

Cross-electrophile coupling A solution of *rac*-**9** (100.0 mg, 0.2 mmol, 1.0 mL, 0.20 M in PhH, 1 equiv.) was charged to a flame-dried culture tube (Fisherbrand 14-956-25E, 20 × 125 mm, 25 mL volume) and dried under positive pressure of N_2 then high vacuum. An oven-dried stir bar, $NiBr_2$·diglyme (221.0 mg,0.6 mmol, 3 equiv.), $Mn(dpm)_3$ (253.0 mg, 0.4 mmol, 2 equiv), oven-dried Li_2CO_3 (23.0 mg, 0.3 mmol, 1.5 equiv.), and oven-dried NaI (282.0 mg, 1.8 mmol, 9 equiv.) were added. The tube was placed under Ar (3 × evacuate/backfill cycles). 7.0 mL DMA (0.033 M of **25**) was added and the turbid blue mixture was sparged with Ar at 1500 rpm for 30 mins. A colloidal yellow-brown mixture was obtained. The septum was changed quickly under Ar, then $PhSiH_3$ (160 µL, 1.2 mmol, 6 equiv.) was added to the mixture. The Ar balloon was taken out and the septum was sealed with telfon tape, electrical tape, and parafilm (see note 1). The mixture was heated in a 35 °C water bath. After 16 hrs, TLC analysis indicated significant consumption of starting material. The mixture was then diluted with EtOAc (2 mL), and pre-chilled H_2O (2 mL) was added. The biphasic mixture was extracted with EtOAc (3 × 5 mL) (see note 2). The organic phase was washed with half-saturated brine, then back extract with EtOAc (2 × 5 mL). The organic phase was dried over Na_2SO_4, and concentrated under reduced pressure. The DMA-containing residue was blown down under positive pressure of N2 for several hours (see note 3). The remaining residue was purified by column chromatography (20% EtOAc/hexanes) to afford major **11** (55.0 mg, 59% yield) and minor, *epi*-**11** (8.6 mg, 9% yield) both as colorless waxy film.

Note 1 − As per original procedure in ref. 1, the presence of air/O_2 is either non-beneficial or detrimental. Here we found that sealing the pierced septum with the forementioned tapes helped with reproducibility on small-scale runs.

Note − 2 Significant formation of emulsion between the two phases could form. Small amounts of sat. aqueous LiCl solution could be added to the pre-shaken mixture to break down the emulsion without further homogenizing, and the phases were separated.
Note – 3 When not properly excluded, DMA could cause poor separation on SiO_2

Carbonylation A solution of vinyl triflate **11** (28 mg, 63.1 µmol, 0.5 mL PhH) was charged to a flame-dried culture tube (Fisherbrand 14-956-25E, 20 × 125 mm, 25 mL volume) and dried under positive pressure of N_2 then high vacuum. $Pd(PPh_3)_2Cl_2$ (21.0 mg, 0.03 mmol) and an oven-dried stir bar were added to the tube and the solids were placed under Ar (3 × evacuate/backfill cycles). Anhydrous DMF (pre-sparged with CO for 20 min, 0.25 mL) was added and the mixture was slurried. In rapid succession, MeOH (pre-sparged with CO for 20 min, 0.25 mL) and Et_3N (80 µL) were added and the reaction headspace was purged with CO (20G vent needle) with rapid stirring. After 10 min, the vent needle was removed and the septa was changed to a new one under CO. Then the reaction tube was placed in a 50 °C oil bath and equipped with 5-layer CO balloon (see note 4). At 5 h, TLC analysis indicated ca. half conversion. After 10 h, TLC analysis indicated trace amounts of starting material remaining. The reaction was allowed to cool room temperature and concentrated under a stream of N_2 for several hours (see note 5). The clear dark concentrate was loaded to a two 20 × 20 cm TLC plates, then eluted with 20% EtOAc/Hex to give racemic **2** (20.8 mg, 93%) as a white solid.

Note 4 – During the reaction timescale, the balloon may get deflated (but not always). To avoid fluctuation with the amount of CO is recommended to use 5-layer CO balloon. In our hands, 5-layer balloon remains inflated throughout the reaction time scale.
Note 5 – Significant amounts of DMF and Et_3N left in the concentrate may result in poor separation during chromatography.

Procedure C:

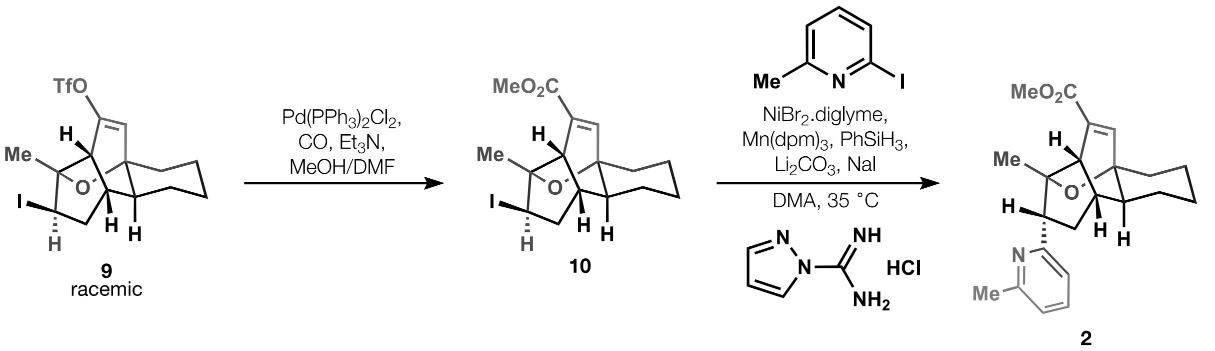

Carbonylation (see page S17).

Cross-electrophile coupling rac-Methyl enoate iodide **11** (20.0 mg, 51.3 µmol, 1 equiv.), an oven-dried stir bar, $NiBr_2$·diglyme (55.0 mg, 0.15 mmol, 3 equiv.), $Mn(dpm)_3$ (62.0 mg, 0.10 mmol, 2 equiv), oven-dried Li_2CO_3 (6.0 mg, 77.3 µmol, 1.5 equiv.), and oven-dried NaI (70.0 mg, 0.46 mmol, 9 equiv.) were charged to a flame-dried tube (Fisherbrand 14-956-25E, 20 × 125 mm, 25 mL volume). The tube was placed under Ar (3 × evacuate/backfill cycles). 0.17 mL DMA (0.033 M of **11**) was added and the turbid blue mixture was sparged with Ar at 1500 rpm for 30 mins. A colloidal yellow-brown mixture was obtained. The septum was changed quickly under Ar, then $PhSiH_3$ (20 µL, 151.0 µmol, 6 equiv.) was added to the mixture. The Ar balloon was taken out and the septum was sealed with telfon tape, electrical tape, and parafilm. The mixture was heated in a 35 °C water bath. After 4 hrs, TLC analysis indicated significant consumption of starting material. The mixture was then diluted with EtOAc (5 mL),

and pre-chilled H_2O (5 mL) was added. The biphasic mixture was extracted with EtOAc (3 × 10 mL) (see note 5). The organic phase was washed with half-saturated brine, then back extract with EtOAc (2 × 5 mL). The organic phase was dried over Na_2SO_4, and concentrated under reduced pressure. The DMA-containing residue was purified by column chromatography (20% EtOAc/Hex) to give a 7:1 mixture of **2** and *epi*-**2** (55% yield qNMR) as a yellow waxy film containing polymeric silane byproducts (see note 7 and 8).

Note – 7 Stereochemistry of **11** and *epi*-**11** was characterized by inference to page S8. Bridge head benzylic H overlapped with other signals.
Note – 8 When pure product is desired multiple we found that multiple chromatographic purification is sufficient (see page S57, 5-CF_3-pyr i.e., RS1163e). Alternatively, as per ref. 1, aqueous wash with pre-chilled 2 M HCl with basified CH_2Cl_2 was also found to be sufficient.

Preparative SFC Separation The racemate **2** was separated on a Waters Prep SFC 150 AP using a Diacel IG column (5 µm, 19x250 mm). The purification was run under isocratic conditions (5% MeOH containing 0.5 vol% 7M methanolic NH_3 / CO_2, 100 mL/min, 1600 psi backpressure) at 35 °C. Fractionation was triggered by mass spec (ESI+ on a Waters QDa, SIR channel m/z = 354.2).

Analytical SFC confirmed high ee for both enantiomers (Figure S6; fast-moving enantiomer, >99% ee; slow-moving enantiomer, >96% ee). Absolute stereochemistry was confirmed by single crystal X-ray diffraction of the fast-moving enantiomer (*vide infra*), assigning the fast-moving enantiomer as *ent*-pyr-GB18 *(+)*-**2**. By inference, the slow-moving enantiomer was assigned as *nat*-pyr-GB18 *(−)*-**2**.

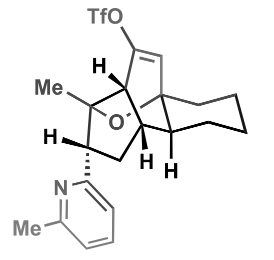

11

Characterization data for vinyl triflate **11**
Description colorless film
TLC R_f = 0.55 (SiO2, 20% EtOAc/hexanes); UV-active, stains purple in anisealdehyde
^{1}H NMR (600 MHz, C_6D_6) δ 7.08 (t, *J* = 7.6 Hz, 1H), 6.79 (d, *J* = 7.7 Hz, 1H), 6.62 (d, *J* = 7.6 Hz, 1H), 5.76 (d, *J* = 2.6 Hz, 1H), 3.05 (dd, *J* = 11.6, 4.2 Hz, 1H), 2.44 (s, 3H), 2.38 – 2.30 (m, 2H), 2.16 (ddd, *J* = 13.3, 3.9, 2.1 Hz, 1H), 1.94 (ddd, *J* = 12.9, 11.6, 5.8 Hz, 1H), 1.74 – 1.65 (m, 2H), 1.47 – 1.38 (m, 2H), 1.38 – 1.33 (m, 1H), 1.29 (td, *J* = 13.0, 4.0 Hz, 1H), 1.09 (s, 3H), 1.06 – 0.95 (m, 1H), 0.89 – 0.77 (m, 2H).
^{13}C NMR (151 MHz, C_6D_6) δ 159.85, 157.17, 148.61, 135.54, 122.58, 120.75, 120.47, 119.29 (d, *J* = 320.1 Hz), 85.29, 75.52, 54.25, 52.98, 51.55, 41.36, 35.70, 33.92, 31.98, 25.98, 24.68, 24.11, 23.48.
2D NMR HSQC, COSY, HMBC, NOESY
HRMS Calculated $C_{21}H_{24}F_3NO_4S$ $[M+H]^+$ 444.1456 | Found: 444.1453

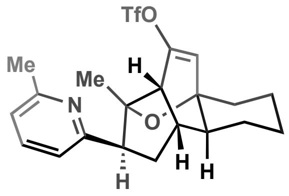

***epi*-11**

Characterization data for vinyl triflate ***epi*-11**
Description colorless film
TLC R_f = 0.60 (SiO2, 20% EtOAc/hexanes); UV-active, stains purple in anisealdehyde
^{1}H NMR (600 MHz, C_6D_6) δ 6.94 (t, *J* = 7.6 Hz, 1H), 6.60 (d, *J* = 7.5 Hz, 1H), 6.51 (d, *J* = 7.7 Hz, 1H), 5.85 (d, *J* = 2.7 Hz, 1H), 3.41 – 3.36 (m, 2H), 2.63 (ddd, *J* = 12.6, 7.2, 5.5 Hz, 1H), 2.31 (s, 3H), 1.95 – 1.85 (m, 3H), 1.45 (td, *J* = 13.3, 4.2 Hz, 2H), 1.38 (dt, *J* = 13.2, 3.1 Hz, 1H), 1.27 – 1.19 (m, 2H), 1.09 – 0.90 (m, 2H), 0.87 (s, 3H), 0.83 (td, *J* = 12.6, 3.7 Hz, 1H).
^{13}C NMR (151 MHz, C_6D_6) δ 162.50, 158.53, 149.03, 136.18, 121.66, 121.43, 120.80, 119.26 (d, *J* = 320.0 Hz), 86.80, 75.90, 56.73, 53.43, 50.51, 41.55, 38.62, 34.50, 31.93, 25.82, 24.61, 23.37, 22.54.
2D NMR HSQC, COSY, HMBC, NOESY
HRMS Calculated $C_{21}H_{24}F_3NO_4S$ $[M+H]^+$ 444.1456 | Found: 444.1465

Characterization data for *rac*-pyr-GB18 **2**
rac-pyrGB18
Description white solid
TLC R_f = 0.31 (SiO_2, 20% EtOAc/hexanes); UV-active, stains turquoise in anisaldehyde
HRMS Calculated $C_{22}H_{28}NO_3$ $[M+H]^+$ 354.2069 | Found: 354.2071

Characterization data for *ent*-pyr-GB18 *(+)*-**2**
optical rotation $[\alpha]_D^{20}$ = +30.0 (c = 0.030, $CHCl_3$)
^{1}H NMR (600 MHz, $CDCl_3$) δ 7.47 (t, *J* = 7.7 Hz, 1H), 7.24 (d, *J* = 1.6 Hz, 1H), 7.12 (d, *J* = 7.7 Hz, 1H), 6.96 (d, *J* = 7.6 Hz, 1H), 3.81 (s, 3H), 3.38 (dd, *J* = 11.8, 4.7 Hz, 1H), 3.19 (dd, *J* = 4.2, 1.8 Hz, 1H), 2.52 (s, 3H), 2.43 (td, *J* = 13.0, 11.8, 5.9 Hz, 1H), 2.10 (dd, *J* = 13.1, 4.7 Hz, 1H), 2.04 (dt, *J* = 13.4, 4.0, 2.1 Hz, 1H), 1.94 (dt, *J* = 12.3, 3.3 Hz, 1H), 1.86 – 1.80 (m, 1H), 1.68 – 1.62 (m, approx. 2H), 1.56 – 1.44 (m, 2H), 1.36 (td, *J* = 13.6, 12.2, 4.1 Hz, 1H), 1.32 – 1.21 (m, approx. 1H), 1.01 (s, 3H), 0.78 (qd, *J* = 13.1, 3.7 Hz, 1H).
^{13}C NMR (151 MHz, $CDCl_3$) δ 165.9, 160.4, 157.0, 145.7, 135.9, 132.6, 120.7, 120.6, 84.8, 73.5, 55.0, 52.3, 52.0, 48.5, 40.4, 37.5, 33.2, 32.3, 25.8, 24.8, 23.6, 23.5.
2D NMR HSQC, COSY
X-ray slow evaporation of a solution of *(+)*-**2** in pentane/C_6H_6 (CCDC 2171738)

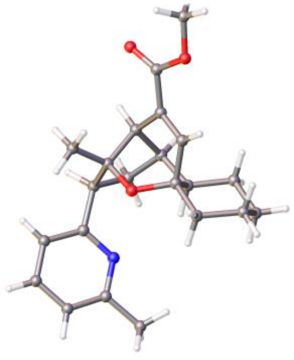

Characterization data for *nat*-pyr-GB18 *(−)*-**2**
NMR data (^{1}H and ^{13}C) matched those of enantiomer *ent*-pyr-GB18.

Optical rotation $[\alpha]_D^{20} = -20.0$ (c = 0.050, $CHCl_3$)

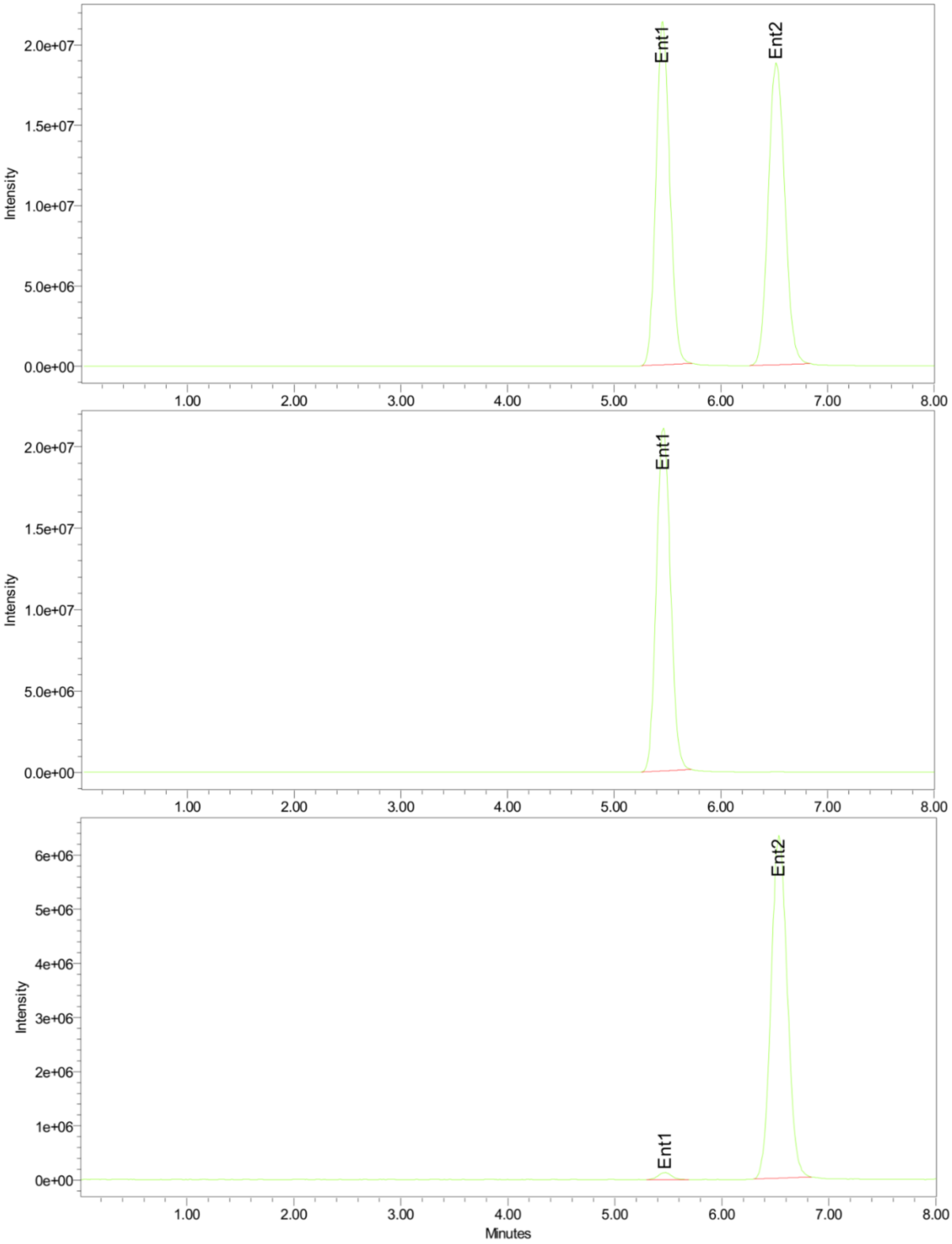

Figure S6. Extracted ion chromatograms at m/z = +354 for SFC analysis of *nat*-pyr-GB18 *(−)*-**2** (bottom, >96% ee), *ent*-pyr-GB18 *(+)*-**2** (middle, >99% ee), and racemic pyr-GB18 **2** (top).

Idiosyncratic Kumada cross-coupling and cross-electrophile coupling (13a)

Note – **13a** and ***epi*-13a** are the same as **7f** and **8f** respectively. (see page S85 for route to **RS1131**)

Procedure A:

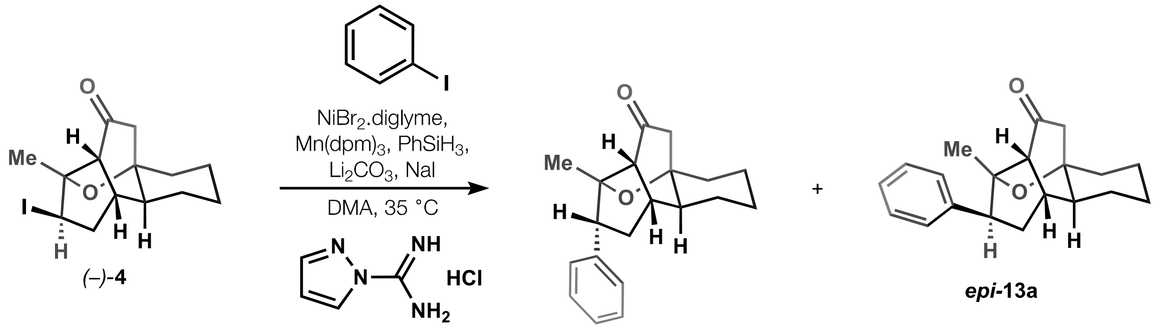

Cross-electrophile coupling (see page S21) A flame-dried 20 mL glass tube with threaded end was equipped with a stir bar and septum pierced with an needle fed by a balloon filled with argon was sequentially charged with praxadine.HCl (85.0 mg, 578 μmol, 2 equiv.), $NiBr_2$•diglyme (224 mg, 635 μmol, 2.2 equiv.), $Mn(dpm)_3$ (245 mg, 404 μmol, 1.4 equiv.), enantiopure *(−)-***4** (100 mg, 289 μmol, 1 equiv.), Li_2CO_3 (21.0 mg, 289 μmol, 1 equiv.), and NaI (260 mg, 1.73 mmol, 6 equiv.). The vial was evacuated and backfilled with argon (5x) and then charged with DMA (dry, 8.8 mL) using a syringe, phenyliodide (295 mg, 161 μL, 1.44 mmol, 5 equiv.) using a microsyringe, and the reaction mixture was degassed with an argon balloon and a vent needle while stirring for 45 min. The reaction mixture turned brown at the end of the degassing period. The vent needle was removed, the septum and argon balloon replaced. Silylbenzene (125 mg, 143 μL, 578 μmol, 4 equiv.) was added using a syringe, then the reaction mixture was placed into a preheated 35 °C water bath. The reaction underwent a series of changes in appearance similar to XEC with 2-iodo-6-methyl pyridine from turbid brown-yellow to turbid yellow-olive to turbid pastel green/light jade to turbid dark jade within a timespan of 15 – 30 min. The reaction was stirred for 7 h. Upon reaction completion (No SM mass found on LC-MS, faint SM spot remaining, dark forest green reaction mixture) EtOAc (8.8 mL) and ice-cold water (17 mL) were added. The layers were separated and the aqueous layer was extracted with EtOAc (6 x 2mL). The combined organic layers were washed with half saturated brine (2 x 8.8 mL) and brine (2 mL). The combined washes were back extracted with EtOAc (2 mL) and the back extract was washed with half saturated brine (2 x 2 mL), then combined with the organic layer, dried over $MgSO_4$, filtered and concentrated *in vacuo*. The crude product was purified and the diastereomers were separated by column chromatography (SiO_2, load in toluene, elute 5-10-15-20-25% EtOAc in hexanes). The product containing fractions were concentrated *in vacuo* and then further purified by column chromatography (SiO_2, load in toluene, elute 10% acetone in hexanes). The resulting product was further purified by preparative TLC (SiO_2, 20 x 20 cm, 0.5 mm thick, load in $CHCl_3$, elute 1.5% MeOH in DCM, total amount of product was split on two plates in total). The diastereomers were separated by preparative TLC (SiO_2, 20 x 20 cm, 0.5 mm thick, load in $CHCl_3$, elute 2.5% EtOAc in toluene, run 7 consecutive times) affording 36.1 mg (42%) of *epi*-**13a** as colorless oil 18.5 mg (22% yield) of **13a** as a colorless oil that formed a white solid upon storage in a -20°C freezer. **13a** from this batch was advanced to **RS1131** (see page S85).

Procedure B:

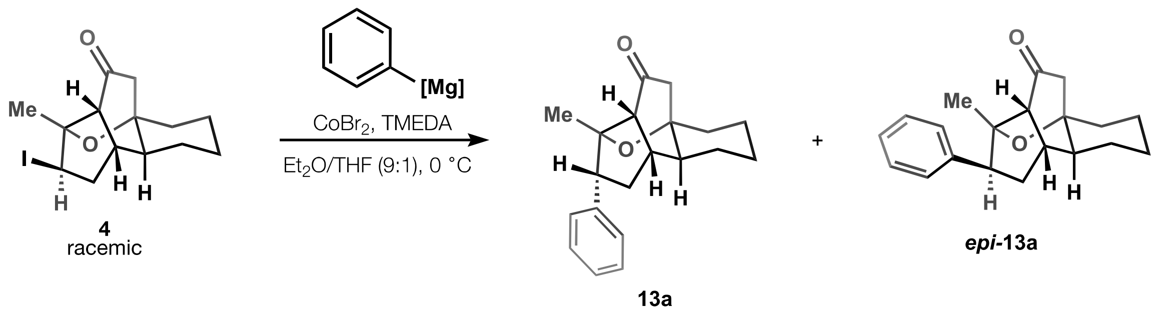

Aryl-Grignard preparation A flame-dried culture tube (Fisherbrand 14-959-25A, 13 × 100 mm, 8 mL volume) was charged with a stir bar and was placed under Ar (3 × evacuate/backfill cycles). Iodobenzene (0.2 mL, 1.73 mmol, 30 equiv.) was added to the tube. Turbo-Grignard (Sigma-Aldrich, 1.5 mL, 1.25 M in THF, 32 equiv.) was added dropwise at 22 °C. Then, the resulting clear dark brown mixture was stirred at the same temperature for at least 1 h before taken up to the next step (see note 1).
Kumada cross-coupling Into a flame-dried culture tube (Fisherbrand 14-956-25E, 20 × 125 mm, 25 mL volume) **4** (20.0 mg, 57.8 µmol, 1equiv.) and $CoBr_2$ (32.0 mg, 144.4 µmol, 2.5 equiv.) were added (see note 2). The solids were placed under Ar (3 × evacuate/backfill cycles). Anhydrous Et_2O (2 mL, 9 parts, 0.025 M), THF (0.2 mL, 1 part, 0.025 M), and TMEDA (20 µL, 2.5 equiv.) were charged to the mixture, resulting into a turbid light blue solution. The solution was cooled to 0 °C. The aryl-Grignard was added dropwise to the reaction mixture. Over the course of the addition, color changed was observed to deep dark blue. At 2 h, TLC analysis indicated trace amount of starting material. Sub-stoichiometric addition of the aryl-Grignard solution could be added (ca. 20 µL) to drive the reaction to completion. Upon complete starting material consumption (6 h), pre-chilled $NaHCO_3$ at 0 °C was added and diluted with Et_2O (5 mL). The biphasic mixture was taken out of the ice bath and extracted with Et_2O (3 × 5 mL) and washed with sat. NaCl solution. The sat. NaCl washings were back extract with Et_2O (2 × 5 mL). The combined organic phase was dried over Na_2SO_4 and concentrated under reduced pressure. The residue was loaded to a 20 × 20 cm TLC plate, then eluted with 10% EtOAc/toluene to give pure sample containing both **13a** and *epi*-**13a** (5.4 mg, 32% qNMR, 5:1 *epi*-**13a**/**13a**)

Note – 1 Only half (15 equiv.) of the solution was used in the Kumada coupling. As the solution was made and ready to be added to the mixture of **4**, all of it was taken up into a syringe and never stored.
Note – 2 $CoBr_2$ is a highly hygroscopic green powder. It may retain H_2O during the timescale of balancing the correct amount for the reaction. It was transferred quickly to the tube.

Characterization data for ketone *epi*-**13a (8f)**
Description colorless oil
TLC R_f = 0.66 (SiO_2, 30% EtOAc in hexanes); UV-active, stains initially grey then turns purple in anisaldehyde
^{1}H NMR (600 MHz, $CDCl_3$) δ 7.28 (t, *J* = 7.6 Hz, 2H), 7.22 – 7.19 (m, 1H), 7.17 – 7.12 (m, 2H), 3.43 (t, *J* = 8.3 Hz, 1H), 2.67 (d, *J* = 19.3 Hz, 1H), 2.44 (d, *J* = 3.9 Hz, 1H), 2.30 (dd, *J* = 13.1, 8.5 Hz, 1H), 2.18 (ddt, *J* = 6.5, 4.6, 2.6 Hz, 1H), 2.10 (ddd, *J* = 13.1, 8.1, 5.1 Hz, 1H), 1.99 (dd, *J* = 19.2, 1.6 Hz, 1H), 1.79 – 1.72 (m, 5H), 1.62 – 1.59 (m, 1H), 1.54 – 1.49 (m, 1H), 1.34 – 1.28 (m, 1H), 1.16 (qd, *J* = 13.0, 3.2 Hz, 1H).
^{13}C NMR (151 MHz, $CDCl_3$) δ 213.1, 142.8, 128.8, 128.6, 126.8, 87.3, 75.1, 59.5, 57.8,

49.6, 42.8, 42.7, 42.4, 36.3, 30.7, 25.8, 24.2, 23.0.
LC-MS m/z = 279.2 $[M-H_2O+H]^+$

Characterization data for ketone **13a (7f)**
Description White solid
TLC R_f = 0.59 (SiO_2, 30% EtOAc in hexanes); UV-active, stains grey in anisaldehyde
^{1}H NMR (600 MHz, $CDCl_3$) δ 7.35 – 7.30 (m, 4H), 7.27 – 7.23 (m, 1H), 3.02 (dd, *J* = 11.9, 5.5 Hz, 1H), 2.71 (d, *J* = 19.2 Hz, 1H), 2.64 (ddd, *J* = 13.5, 12.0, 6.3 Hz, 1H), 2.45 (d, *J* = 4.3 Hz, 1H), 2.17 (ddd, *J* = 6.2, 4.3, 1.7 Hz, 1H), 2.02 (dd, *J* = 19.2, 1.5 Hz, 1H), 1.91 – 1.84 (m, 2H), 1.83 – 1.69 (m, 4H), 1.54 (td, *J* = 12.8, 4.4 Hz, 1H), 1.43 – 1.33 (m, 2H), 1.22 – 1.13 (m, 4H).
^{13}C NMR (151 MHz, $CDCl_3$) δ 213.1, 139.1, 130.1, 128.1, 126.8, 84.9, 74.5, 61.2, 54.9, 52.5, 43.1, 42.7 (2C), 35.6, 30.4, 25.9, 24.5, 23.1.
2D NMR COSY, HSQC, HMBC, NOESY
LC-MS m/z = 298.2 $[M+H]^+$ and 279.2 $[M-H_2O+H]^+$

Idiosyncratic *endo*-selective Kumada cross-coupling (13b)

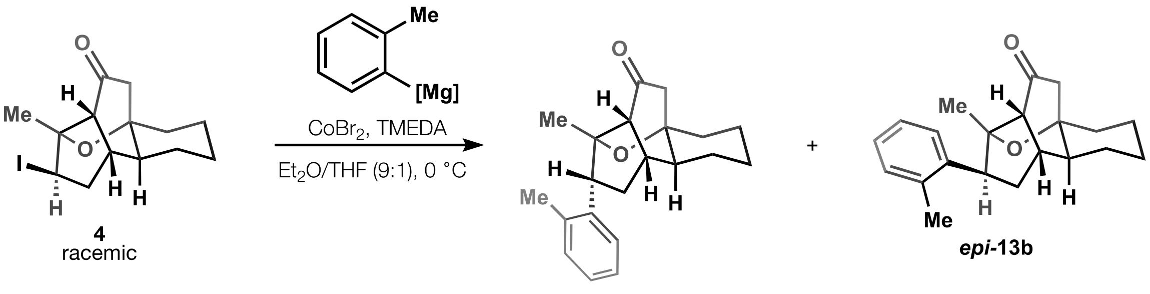

Aryl-Grignard preparation A flame-dried culture tube (Fisherbrand 14-959-25A, 13 × 100 mm, 8 mL volume) was charged with a stir bar and was placed under Ar (3 × evacuate/backfill cycles). 2-Iodotoluene (225.0 µL, 1.73 mmol, 30 equiv.) was added to the tube. Turbo-Grignard (Sigma-Aldrich, 1.5 mL, 1.25 M in THF, 32 equiv.) was added dropwise at 22 °C. Then, the resulting clear dark brown mixture was stirred at the same temperature for at least 1 h before taken up to the next step (see note 1).

Kumada cross-coupling Into a flame-dried culture tube (Fisherbrand 14-956-25E, 20 × 125 mm, 25 mL volume) **4** (20.0 mg, 57.8 µmol, 1equiv.) and $CoBr_2$ (32.0 mg, 144.4 µmol, 2.5 equiv.) were added (see note 2). The solids were placed under Ar (3 × evacuate/backfill cycles). Anhydrous Et_2O (2 mL, 9 parts, 0.025 M), THF (0.2 mL, 1 part, 0.025 M), and TMEDA (20 µL, 2.5 equiv.) were charged to the mixture, resulting into a turbid light blue solution. The solution was cooled to 0 °C. The aryl-Grignard was added dropwise to the reaction mixture. Over the course of the addition, color changed was observed to deep dark blue. At 2 h, TLC analysis indicated trace amount of starting material. Sub-stoichiometric addition of the aryl-Grignard solution could be added (ca. 20 µL) to drive the reaction to completion. Upon complete starting material consumption (6 h), pre-chilled $NaHCO_3$ at 0 °C was added and diluted with Et_2O (5 mL). The biphasic mixture was taken out of the ice bath and extracted with Et_2O (3 × 5 mL) and washed with sat. NaCl solution. The sat. NaCl washings were back extract with Et_2O (2 × 5 mL). The combined organic phase was dried over Na_2SO_4 and concentrated under reduced pressure. The residue was loaded to two 20 × 20 cm TLC plates, then eluted with 10% EtOAc/toluene to give pure sample containing both **13b** and *epi*-**13b** (7.0 mg, 39% yield, 1:4 *epi*-**13b**/**13b**). Then the mixture was loaded to two 20 × 20 cm TLC plates, then eluted with 5% EtOAc/PhH to separate the diastereomers (see note 3).

Note – 1 Only half (15 equiv.) of the solution was used in the Kumada coupling. As the solution was made and ready to be added to the mixture of **4**, all of it was taken up into a syringe and never stored.

Note – 2 $CoBr_2$ is a highly hygroscopic green powder. It may retain H_2O during the timescale of balancing the correct amount for the reaction. It was transferred quickly to the tube.

Note – 3 Consistent with ref. 1, new impurities appeared following the second round of preparative TLC that was not present in the pure diastereomeric mixture.

Characterization data for ketone *epi*-**13b**

Description White powder

TLC R_f = 0.50 (SiO_2, 10% EtOAc in toluene); UV-active, stains greyish blue in anisaldehyde

^{1}H NMR (600 MHz, $CDCl_3$) δ 7.19 – 7.12 (m, 2H), 7.10 (td, *J* = 7.4, 1.5 Hz, 1H), 7.06 – 7.01 (m, 1H), 3.78 (t, *J* = 8.4 Hz, 1H), 2.71 (dd, *J* = 33.6, 19.2 Hz, 1H), 2.46 – 2.39 (m, 4H), 2.27 – 2.15 (m, 3H), 2.10 (ddd, *J* = 13.0, 8.3, 4.9 Hz, 1H), 1.98 (dd, *J* = 19.3, 1.7 Hz, 1H),

1.82 – 1.70 (m, 3H), 1.65 (ddt, *J* = 13.2, 4.1, 2.0 Hz, 1H), 1.59 – 1.51 (m, 1H), 1.45 – 1.16 (m, 3H), 0.79 (s, 3H).
^{13}C NMR (151 MHz, CDCl3) δ 213.29, 141.03, 137.24, 130.55, 126.34, 61.45, 59.57, 52.72, 51.85, 49.36, 48.59, 42.85, 42.40, 42.22, 36.29, 30.79, 25.85, 23.76, 23.10, 23.04, 21.00.
2D NMR COSY, HSQC, HMBC, NOESY
HRMS Calculated $C_{21}H_{26}O_2$ $[M+H]^+$ 311.2011 | Found: 311.2019

<u>Characterization data for ketone **13b**</u>
Description White powder
TLC R_f = 0.45 (SiO_2, 10% EtOAc in Toluene); UV-active, stains greyish blue in anisaldehyde
^{1}H NMR (600 MHz, CDCl3) δ 7.61 – 7.56 (m, 1H), 7.22 (td, J = 7.3, 2.4 Hz, 1H), 7.18 – 7.09 (m, 2H), 3.36 (dd, J = 11.9, 5.9 Hz, 1H), 2.77 – 2.63 (m, 2H), 2.50 (d, J = 4.4 Hz, 1H), 2.34 (s, 3H), 2.19 (ddd, J = 6.3, 4.3, 1.7 Hz, 1H), 2.04 (dd, J = 19.2, 1.6 Hz, 1H), 1.90 (ddt, J = 13.4, 3.7, 1.7 Hz, 1H), 1.82 – 1.67 (m, 5H), 1.66 – 1.54 (m, 2H), 1.46 – 1.33 (m, 2H), 1.15 (s, 3H).
^{13}C NMR (151 MHz, CDCl3) δ 213.27, 137.72, 136.97, 130.31, 130.18, 126.50, 125.75, 85.65, 74.64, 61.44, 52.72, 48.58, 43.94, 43.07, 42.78, 35.57, 30.34, 25.84, 24.87, 23.09, 20.84.
2D NMR COSY, HSQC, HMBC, NOESY
HRMS Calculated $C_{21}H_{26}O_2$ $[M+H]^+$ 311.2011 | Found: 311.2021

Suzuki cross-coupling phenyl (14)

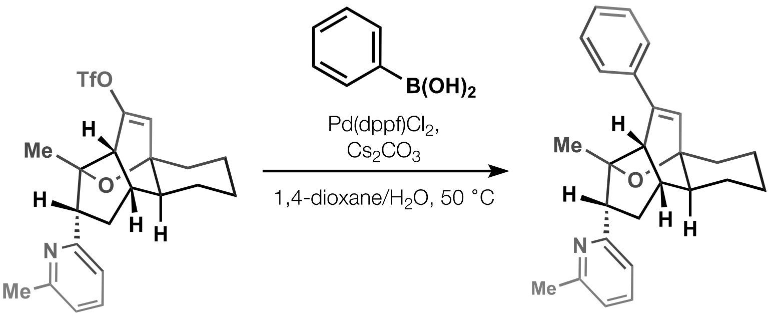

11 14

A solution of *rac*-vinyl triflate iodide **11** (125.0 µL, 12.5 mg, 28.2 µmol, 1 equiv., PhH solution) was added to a flame-dried culture tube (Fisherbrand 14-957-76A, 100 mm length, 8 mL volume) dried under N_2 then high vacuum. An oven-dried stir bar, Pd(dppf)Cl_2 (1.0 mg, 1.4 µmol, 5 mol%), phenylboronic acid (17.0 mg, 140.9 µmol, 5 equiv.), and Cs_2CO_3 (92.0 mg, 281.9 µmol, 10 equiv) were added to the tube. Then it was placed under Ar (3 × evacuate/backfill cycles). 2.0 mL pre-sparged dioxane and 500 µL miliQ-H_2O were added to the solids resulting in a clear deep red solution. The mixture was heated to 50 °C in a heating block. After 12 h, TLC analysis indicate full consumption of starting material. The reaction was filtered through Celite and Na_2SO_4 plug. The solution was concentrated under a positive stream of N_2 for several hours. The resulting yellowish-red waxy residue was purified by two rounds preparative TLC (one plate each run, 20 × 20 cm, 15% EtOAc/Hex then 20% EtOAc/Hex) to afford pure **14** (9.7 mg, 93% yield)

Characterization data for **14**
Description colorless oil
TLC R_f = 0.10 (SiO_2, 15% EtOAc in hexanes); UV-active, stains turquoise in anisealdehyde
^{1}H NMR (600 MHz, CD_2Cl_2) δ 7.53 – 7.48 (m, 3H), 7.37 (dd, *J* = 8.6, 6.9 Hz, 2H), 7.31 – 7.25 (m, 1H), 7.16 (d, *J* = 7.7 Hz, 1H), 6.99 (d, *J* = 7.6 Hz, 1H), 6.53 (d, *J* = 2.2 Hz, 1H), 3.39 (dd, *J* = 11.8, 4.8 Hz, 1H), 3.09 (dd, *J* = 4.1, 2.1 Hz, 1H), 2.51 (s, 3H), 2.43 (ddd, *J* = 13.1, 11.8, 5.9 Hz, 1H), 2.16 (dd, *J* = 13.0, 4.7 Hz, 1H), 2.04 – 1.98 (m, 1H), 1.87 (dt, *J* = 12.2, 4.0 Hz, 1H), 1.84 – 1.76 (m, 1H), 1.72 (ddd, *J* = 6.0, 4.1, 2.1 Hz, 1H), 1.68 – 1.49 (m, 3H), 1.36 – 1.21 (m, 2H), 1.00 (s, 3H), 0.91 (qd, *J* = 13.0, 3.7 Hz, 1H).
^{13}C NMR (151 MHz, CD_2Cl_2) δ 161.22, 157.19, 140.43, 139.52, 135.81, 130.50, 129.92, 128.89, 127.65, 125.48, 120.88, 120.52, 115.66, 84.55, 73.68, 55.40, 52.98, 52.01, 41.20, 37.55, 34.13, 32.71, 26.32, 24.68, 24.06, 23.97.
2D NMR HSQC, NOESY
HRMS Calculated $C_{26}H_{29}NO$ $[M+H]^+$ 372.2327 | Found: 372.2342

Suzuki cross-coupling 2-biphenyl (15)

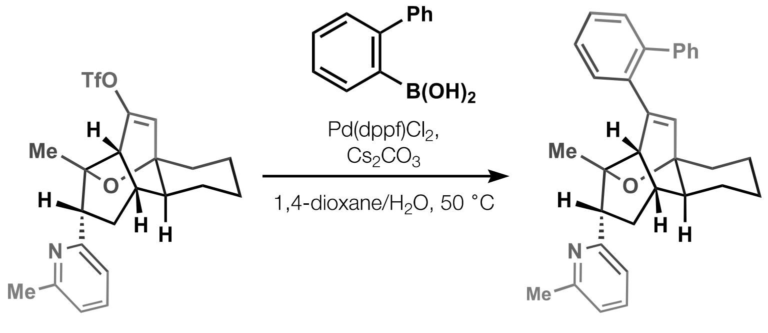

11 15

A solution of *rac*-vinyl triflate iodide **11** (150.0 µL, 13.8 mg, 31.1 µmol, 1 equiv., PhH solution) was added to a flame-dried culture tube (Fisherbrand 14-957-76A, 100 mm length, 8 mL volume) dried under N_2 then high vacuum. An oven-dried stir bar, Pd(dppf)Cl_2 (1.0 mg, 1.4 µmol, 5 mol%), 2-biphenylboronic acid (31.0 mg, 155.6 µmol, 5 equiv.), and Cs_2CO_3 (100.0 mg, 311.2 µmol, 10 equiv) were added to the tube. Then it was placed under Ar (3 × evacuate/backfill cycles). 2.0 mL pre-sparged dioxane and 500 µL miliQ-H_2O were added to the solids resulting in a clear deep red solution. The mixture was heated to 50 °C in a heating block. After 12 h, TLC analysis indicate full consumption of starting material. The reaction was filtered through Celite and Na_2SO_4 plug. The solution was concentrated under a positive stream of N_2 for several hours. The resulting yellowish-red waxy residue was purified by preparative TLC (one plate, 20 × 20 cm, 20% EtOAc/Hex) to afford pure **15** (6.2 mg, 45% yield).

Characterization data for **15**
Description white powder
TLC R_f = 0.15 (SiO_2, 20% EtOAc in hexanes); UV-active, stains turquoise in anisealdehyde
^{1}H NMR (600 MHz, CD_2Cl_2) δ 7.50 – 7.26 (m, 11H), 7.05 (d, *J* = 7.8 Hz, 1H), 6.94 (d, *J* = 7.6 Hz, 1H), 6.27 (d, *J* = 2.1 Hz, 1H), 3.07 (dd, *J* = 11.7, 4.9 Hz, 1H), 2.45 (s, 3H), 2.27 (dd, *J* = 4.0, 1.9 Hz, 1H), 1.93 (ddd, *J* = 13.1, 11.7, 6.2 Hz, 1H), 1.83 – 1.70 (m, 3H), 1.65 – 1.54 (m, 2H), 1.39 (qt, *J* = 13.6, 3.9 Hz, 2H), 1.28 – 1.19 (m, 2H), 0.92 (qd, *J* = 13.0, 3.7 Hz, 1H), 0.86 (s, 3H).
^{13}C NMR (151 MHz, CD_2Cl_2) δ 161.35, 157.05, 142.69, 142.63, 141.09, 140.70, 135.68, 135.02, 133.81, 130.68, 130.25, 129.68, 129.20, 129.09, 128.50, 127.79, 127.42, 127.23, 120.78, 120.41, 84.61, 73.75, 67.43, 55.18, 40.62, 37.67, 34.17, 32.52, 26.36, 24.62, 24.03, 23.93.
2D NMR HSQC, NOESY
HRMS Calculated $C_{32}H_{33}NO$ $[M+H]^+$ 448.2640| Found: 448.2652

Suzuki cross-coupling 2-trifluoromethyl phenyl (16)

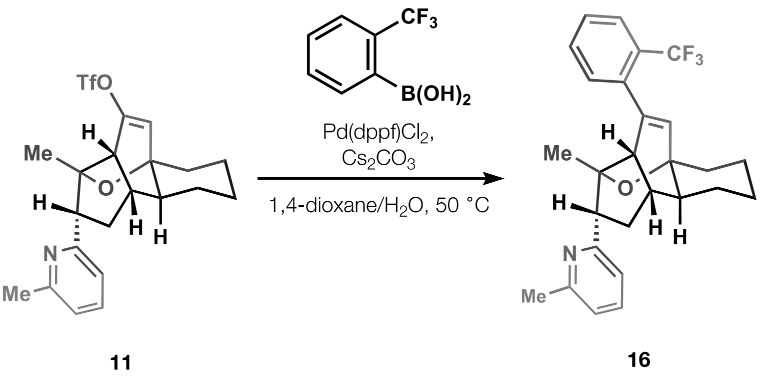

A solution of *rac*-vinyl triflate iodide **11** (150.0 µL, 13.8 mg, 31.1 µmol, 1 equiv., PhH solution) was added to a flame-dried culture tube (Fisherbrand 14-957-76A, 100 mm length, 8 mL volume) dried under N_2 then high vacuum. An oven-dried stir bar, $Pd(dppf)_2Cl_2$ (1.0 mg, 1.4 µmol, 5 mol%), 2-trifluoromethyl phenyl boronic acid (30.0 mg, 155.6 µmol, 5 equiv.), and Cs_2CO_3 (100.0 mg, 311.2 µmol, 10 equiv) were added to the tube. Then it was placed under Ar (3 × evacuate/backfill cycles). 2.0 mL pre-sparged dioxane and 500 µL miliQ-H_2O were added to the solids resulting in a clear deep red solution. The mixture was heated to 50 °C in a heating block. After 12 h, TLC analysis indicate full consumption of starting material. The reaction was filtered through Celite and Na_2SO_4 plug. The solution was concentrated under a positive stream of N_2 for several hours. The resulting yellowish-red waxy residue was purified by preparative TLC (one plate, 20 × 20 cm, 30% EtOAc/Hex) to afford pure **16** (12.7 mg, 93% yield).

Characterization data for **16**
Description white powder
TLC R_f = 0.42 (SiO_2, 30% EtOAc in hexanes); UV-active, stains turquoise in anisealdehyde
^{1}H NMR (600 MHz, CD_2Cl_2) δ 7.71 (dd, *J* = 8.2, 1.4 Hz, 1H), 7.56 (td, *J* = 7.6, 1.3 Hz, 1H), 7.50 (t, *J* = 7.6 Hz, 1H), 7.42 (dt, *J* = 7.7, 3.7 Hz, 2H), 7.15 (d, *J* = 7.7 Hz, 1H), 6.99 (d, *J* = 7.6 Hz, 1H), 6.29 (d, *J* = 2.1 Hz, 1H), 3.30 (dd, *J* = 11.8, 4.8 Hz, 1H), 2.81 (dd, *J* = 4.0, 2.0 Hz, 1H), 2.51 (s, 3H), 2.38 (ddd, *J* = 13.1, 11.7, 6.0 Hz, 1H), 2.12 (dd, *J* = 13.0, 4.8 Hz, 1H), 2.06 – 1.99 (m, 1H), 1.93 – 1.83 (m, 2H), 1.82 – 1.74 (m, 1H), 1.69 – 1.61 (m, 1H), 1.60 – 1.42 (m, 2H), 1.36 – 1.21 (m, 2H), 1.07 (s, 3H), 1.04 (dd, *J* = 13.1, 3.7 Hz, 1H).
^{13}C NMR (151 MHz, CD_2Cl_2) δ 161.16, 157.21, 140.89, 140.14, 135.81, 135.41, 132.26, 131.48, 127.51, 126.48, 125.78, 123.97, 120.92, 120.55, 84.58, 73.72, 67.43, 55.20, 52.89, 41.21, 37.36, 34.18, 31.96, 26.36, 24.68, 24.51, 23.95.
2D NMR HSQC, NOESY
HRMS Calculated $C_{27}H_{28}F_3NO$ $[M+H]^+$ 440.2201 | Found: 440.2219

Suzuki cross-coupling 2-butoxy phenyl (17)

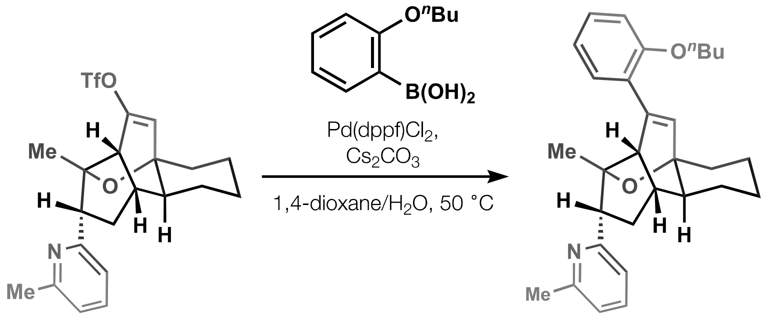

11 **17**

A solution of *rac*-vinyl triflate iodide **11** (150.0 µL, 13.8 mg, 31.1 µmol, 1 equiv., PhH solution) was added to a flame-dried culture tube (Fisherbrand 14-957-76A, 100 mm length, 8 mL volume) dried under N_2 then high vacuum. An oven-dried stir bar, Pd(dppf)$_2$Cl$_2$ (1.0 mg, 1.4 µmol, 5 mol%), 2-butoxy phenyl boronic acid (30.0 mg, 155.6 µmol, 5 equiv.), and Cs_2CO_3 (100.0 mg, 311.2 µmol, 10 equiv) were added to the tube. Then it was placed under Ar (3 × evacuate/backfill cycles). 2.0 mL pre-sparged dioxane and 500 µL miliQ-H_2O were added to the solids resulting in a clear deep red solution. The mixture was heated to 50 °C in a heating block. After 12 h, TLC analysis indicate full consumption of starting material. The reaction was filtered through Celite and Na_2SO_4 plug. The solution was concentrated under a positive stream of N_2 for several hours. The resulting yellowish-red waxy residue was purified by preparative TLC (one plate, 20 × 20 cm, 30% EtOAc/Hex) to afford pure **17** (12.7 mg, 93% yield).

Characterization data for **17**
Description white powder
TLC R_f = 0.42 (SiO_2, 30% EtOAc in hexanes); UV-active, stains turquoise in anisealdehyde
^{1}H NMR (600 MHz, CD_2Cl_2) δ 7.50 (t, *J* = 7.7 Hz, 1H), 7.29 (dd, *J* = 7.4, 1.9 Hz, 1H), 7.23 (td, *J* = 7.8, 1.9 Hz, 1H), 7.17 (d, *J* = 7.8 Hz, 1H), 6.95 – 6.88 (m, 2H), 6.38 (d, *J* = 1.9 Hz, 1H), 3.99 (t, *J* = 6.3 Hz, 2H), 3.30 (dd, *J* = 11.8, 4.8 Hz, 1H), 3.00 (dd, *J* = 4.1, 1.9 Hz, 1H), 2.50 (s, 3H), 2.40 (td, *J* = 12.4, 6.0 Hz, 1H), 2.09 (dd, *J* = 13.0, 4.9 Hz, 1H), 1.98 – 1.91 (m, 1H), 1.89 – 1.73 (m, 5H), 1.67 – 1.46 (m, 6H), 1.35 – 1.24 (m, 2H), 1.06 – 0.94 (m, 7H).
^{13}C NMR (151 MHz, CD_2Cl_2) δ 161.59, 157.12, 139.95, 136.92, 135.76, 132.83, 129.13, 128.73, 120.90, 120.67, 120.44, 111.70, 84.26, 73.56, 68.12, 55.57, 53.89, 53.59, 41.06, 37.68, 34.24, 32.74, 32.00, 26.39, 24.67, 24.17, 23.95, 19.97, 19.69, 14.05.
2D NMR HSQC, NOESY
HRMS Calculated $C_{30}H_{37}NO_2$ $[M+H]^+$ 444.2903 | Found: 444.2917

Suzuki cross-coupling 2-furan (18)

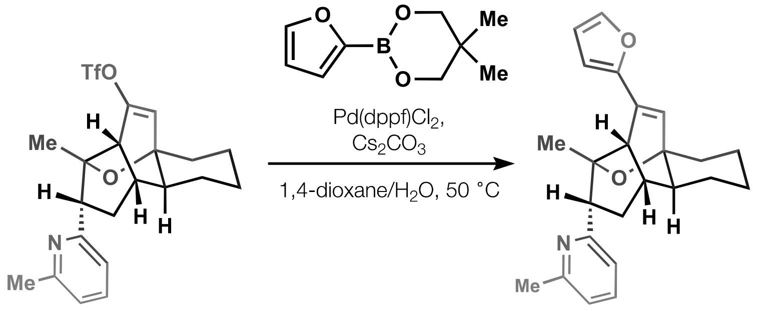

11 18

A solution of *rac*-vinyl triflate iodide **11** (100.0 µL, 12.5 mg, 28.19 µmol, 1 equiv., PhH solution) was added to a flame-dried culture tube (Fisherbrand 14-957-76A, 100 mm length, 8 mL volume) dried under N_2 then high vacuum. An oven-dried stir bar, Pd(dppf)Cl_2 (1.0 mg, 1.4 µmol, 5 mol%), 2-furanyl-Bnep (25.0 mg, 140.9 µmol, 5 equiv.), and Cs_2CO_3 (90.0 mg, 281.9 µmol, 10 equiv) were added to the tube. Then it was placed under Ar (3 × evacuate/backfill cycles). 2.0 mL pre-sparged dioxane and 500 µL miliQ-H_2O were added to the solids resulting in a clear deep red solution. The mixture was heated to 50 °C in a heating block. After 12 h, TLC analysis indicate full consumption of starting material. The reaction was filtered through Celite and Na_2SO_4 plug. The solution was concentrated under a positive stream of N_2 for several hours. The resulting yellowish-red waxy residue was purified by preparative TLC (one plate, 20 × 20 cm, 30% EtOAc/Hex) to afford pure **18** (7.9 mg, 78% yield).

Characterization data for **18**
Description white powder
TLC R_f = 0.42 (SiO_2, 30% EtOAc in hexanes); UV-active, stains deep blue in anisealdehyde
^{1}H NMR (600 MHz, CD_2Cl_2) δ 7.49 (t, *J* = 7.6 Hz, 1H), 7.41 (d, *J* = 2.4 Hz, 1H), 7.12 (d, *J* = 7.7 Hz, 1H), 6.98 (d, *J* = 7.6 Hz, 1H), 6.51 (d, *J* = 1.9 Hz, 1H), 6.43 (dd, *J* = 3.3, 1.9 Hz, 1H), 6.40 (d, *J* = 3.3 Hz, 1H), 3.36 (dd, *J* = 11.8, 4.7 Hz, 1H), 2.91 (dd, *J* = 4.1, 1.9 Hz, 1H), 2.50 (s, 3H), 2.39 (ddd, *J* = 13.1, 11.7, 5.9 Hz, 1H), 2.06 – 1.99 (m, 2H), 1.84 (dt, *J* = 12.2, 4.0 Hz, 1H), 1.81 – 1.73 (m, 0H), 1.70 (ddd, *J* = 6.0, 4.0, 2.1 Hz, 1H), 1.67 – 1.47 (m, 4H), 1.33 – 1.20 (m, 2H), 0.97 (s, 3H), 0.96 – 0.85 (m, 1H).
^{13}C NMR (151 MHz, CD_2Cl_2) δ 161.01, 157.22, 153.72, 142.43, 135.80, 130.60, 127.81, 120.84, 120.54, 111.60, 105.76, 84.38, 73.48, 55.36, 53.11, 50.46, 40.97, 37.36, 34.06, 32.64, 26.27, 24.69, 23.89, 23.83.
2D NMR HSQC, NOESY
HRMS Calculated $C_{24}H_{27}NO_2$ $[M+H]^+$ 362.2120 | Found: 362.2128

Suzuki cross-coupling 3-pyrridine (19)

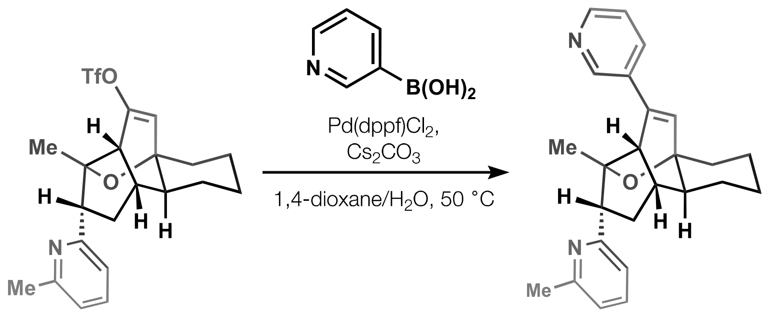

11 19

A solution of *rac*-vinyl triflate iodide **11** (125.0 µL, 14.2 mg, 32.02 µmol, 1 equiv., PhH solution) was added to a flame-dried culture tube (Fisherbrand 14-957-76A, 100 mm length, 8 mL volume) dried under N_2 then high vacuum. An oven-dried stir bar, Pd(dppf)Cl_2 (1.0 mg, 1.4 µmol, 5 mol%), 3-pyridinylboronic acid (20.0 mg, 160.1 µmol, 5 equiv.), and Cs_2CO_3 (105.0 mg, 320.2 µmol, 10 equiv) were added to the tube. Then it was placed under Ar (3 × evacuate/backfill cycles). 2.0 mL pre-sparged dioxane and 500 µL miliQ-H_2O were added to the solids resulting in a clear deep red solution. The mixture was heated to 50 °C in a heating block. After 12 h, TLC analysis indicate full consumption of starting material. The reaction was filtered through Celite and Na_2SO_4 plug. The solution was concentrated under a positive stream of N_2 for several hours. The resulting yellowish-red waxy residue was purified by preparative TLC (two plates, 20 × 20 cm, 70% EtOAc/Hex) to afford pure **19** (7.9 mg, 66% yield).

Characterization data for **19**
Description white powder
TLC R_f = 0.30 (SiO_2, 70% EtOAc in hexanes); UV-active, stains light turqouise in anisealdehyde
^{1}H NMR (600 MHz, CD_2Cl_2) δ 8.74 (d, *J* = 2.4 Hz, 1H), 8.48 (dd, *J* = 4.8, 1.7 Hz, 1H), 7.78 (dt, *J* = 8.0, 2.0 Hz, 1H), 7.51 (t, *J* = 7.6 Hz, 1H), 7.30 (dd, *J* = 8.0, 4.8 Hz, 1H), 7.14 (d, *J* = 7.8 Hz, 1H), 6.99 (d, *J* = 7.6 Hz, 1H), 6.62 (d, *J* = 1.9 Hz, 1H), 3.39 (dd, *J* = 11.8, 4.8 Hz, 1H), 3.07 (dd, *J* = 4.1, 1.9 Hz, 1H), 2.51 (s, 3H), 2.42 (td, *J* = 12.4, 5.9 Hz, 1H), 2.20 (dd, *J* = 13.0, 4.7 Hz, 1H), 2.07 (ddd, *J* = 13.2, 4.0, 2.0 Hz, 1H), 1.88 (dt, *J* = 12.2, 3.6 Hz, 1H), 1.81 (dq, *J* = 13.9, 2.5 Hz, 1H), 1.74 (td, *J* = 5.1, 2.0 Hz, 3H), 1.66 (dt, *J* = 13.2, 3.2 Hz, 1H), 1.55 (tt, *J* = 13.5, 6.7 Hz, 1H), 1.30 (dddd, *J* = 26.1, 12.8, 7.9, 3.3 Hz, 2H), 1.02 (s, 2H), 0.90 (qd, *J* = 13.1, 3.8 Hz, 1H).
^{13}C NMR (151 MHz, CD_2Cl_2) δ 160.94, 157.26, 148.75, 147.14, 137.79, 135.84, 134.84, 132.57, 132.56, 123.70, 120.86, 120.58, 84.62, 73.68, 55.26, 52.59, 51.75, 41.15, 37.32, 33.99, 32.69, 26.25, 24.69, 24.04, 23.93.
2D NMR HSQC, NOESY
HRMS Calculated $C_{25}H_{28}N_2O$ $[M+H]^+$ 373.2280 | Found: 373.2290

Suzuki cross-coupling 2-CO_2Me-pyridine (20)

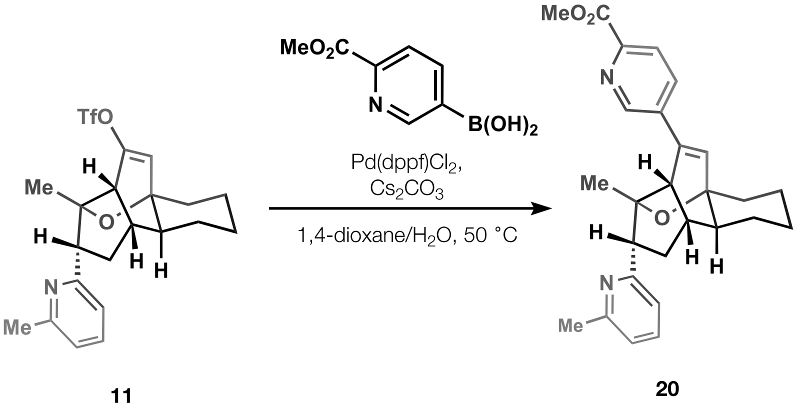

A solution of *rac*-vinyl triflate iodide **11** (150.0 µL, 16.25 mg, 36.64 µmol, 1 equiv., PhH solution) was added to a flame-dried culture tube (Fisherbrand 14-957-76A, 100 mm length, 8 mL volume) dried under N_2 then high vacuum. An oven-dried stir bar, Pd(dppf)Cl_2 (1.0 mg, 1.4 µmol, 4 mol%), 3-pyridinylboronic acid (33.0 mg, 183.20 µmol, 5 equiv.), and Cs_2CO_3 (120.0 mg, 366.4 µmol, 10 equiv.) were added to the tube. Then it was placed under Ar (3 × evacuate/backfill cycles). 2.0 mL pre-sparged dioxane and 500 µL miliQ-H_2O were added to the solids resulting in a clear deep red solution. The mixture was heated to 50 °C in a heating block. After 12 h, TLC analysis indicate full consumption of starting material. The reaction was filtered through Celite and Na_2SO_4 plug. The solution was concentrated under a positive stream of N_2 for several hours. The resulting yellowish-red waxy residue was purified by preparative TLC (two plates, 20 × 20 cm, 70% EtOAc/Hex) to afford pure **20** (7.2 mg, 46% yield).

Characterization data for **20**
Description white powder
TLC R_f = 0.54 (SiO_2, 2% MeOH in 50% EtOAc/Hex); UV-active and slightly fluorescent, stains turquoise in anisealdehyde
^{1}H NMR (600 MHz, CD_2Cl_2) δ 8.84 (d, *J* = 2.2 Hz, 1H), 8.09 (d, *J* = 8.1 Hz, 1H), 7.90 (dd, *J* = 8.2, 2.2 Hz, 1H), 7.50 (t, *J* = 7.6 Hz, 1H), 7.12 (d, *J* = 7.7 Hz, 1H), 6.77 (d, *J* = 1.9 Hz, 1H), 3.96 (d, *J* = 1.3 Hz, 3H), 3.41 (dd, *J* = 11.8, 4.7 Hz, 1H), 3.10 (dd, *J* = 4.0, 2.0 Hz, 1H), 2.52 (s, 3H), 2.43 (td, *J* = 12.4, 5.9 Hz, 1H), 2.23 (dd, *J* = 13.0, 4.6 Hz, 1H), 2.12 (ddd, *J* = 13.4, 4.0, 2.1 Hz, 1H), 1.92 – 1.79 (m, 3H), 1.78 – 1.73 (m, 1H), 1.69 – 1.63 (m, 1H), 1.54 (dp, *J* = 13.7, 4.0 Hz, 2H), 1.37 – 1.25 (m, 2H), 1.01 (s, 3H), 0.88 (qd, *J* = 13.0, 3.7 Hz, 1H).
^{13}C NMR (151 MHz, CD_2Cl_2) δ 165.92, 160.72, 157.32, 147.07, 146.82, 137.76, 137.13, 135.87, 135.11, 133.19, 125.28, 120.86, 120.62, 84.72, 73.76, 55.17, 52.88, 52.30, 51.55, 41.15, 37.15, 33.87, 32.66, 26.20, 24.70, 24.00, 23.90.
2D NMR HSQC, COSY
HRMS Calculated $C_{27}H_{30}N_2O_3$ $[M+H]^+$ 431.2335 | Found: 431.2351

Suzuki cross-coupling 2-OMe-pyrridine (21)

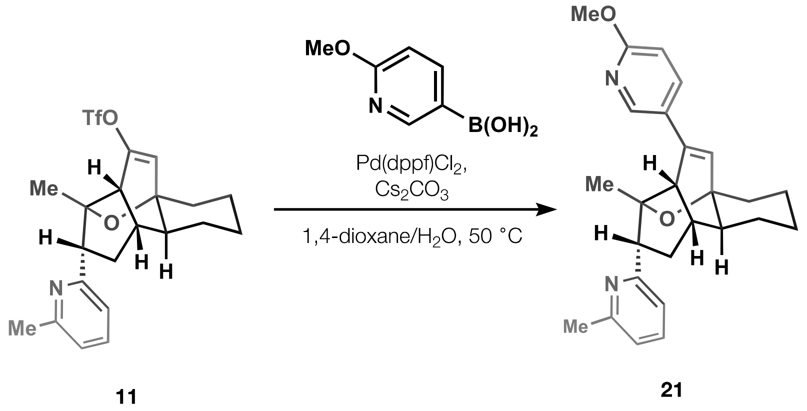

A solution of *rac*-vinyl triflate iodide **11** (125.0 µL, 12.5 mg, 36.64 µmol, 1 equiv., PhH solution) was added to a flame-dried culture tube (Fisherbrand 14-957-76A, 100 mm length, 8 mL volume) dried under N_2 then high vacuum. An oven-dried stir bar, Pd(dppf)Cl_2 (1.0 mg, 1.4 µmol, 4 mol%), 6-methoxypyridin-3-yl) boronic acid (22.0 mg, 140.9 µmol, 5 equiv.), and Cs_2CO_3 (90.0 mg, 281.9 µmol, 10 equiv.) were added to the tube. Then it was placed under Ar (3 × evacuate/backfill cycles). 2.0 mL pre-sparged dioxane and 500 µL miliQ-H_2O were added to the solids resulting in a clear deep red solution. The mixture was heated to 50 °C in a heating block. After 12 h, TLC analysis indicate full consumption of starting material. The reaction was filtered through Celite and Na_2SO_4 plug. The solution was concentrated under a positive stream of N_2 for several hours. The resulting yellowish-red waxy residue was purified by preparative TLC (two plates, 20 × 20 cm, 70% EtOAc/Hex) to afford pure **21** (7.5 mg, 66% yield)

Characterization data for **21**
Description white powder
TLC R_f = 0.30 (SiO_2, 40% EtOAc/Hex); UV-active, stains turquoise in anisealdehyde
^{1}H NMR (600 MHz, CD_2Cl_2) δ 8.29 – 8.25 (m, 1H), 7.73 (dd, *J* = 8.6, 2.6 Hz, 1H), 7.50 (t, *J* = 7.6 Hz, 1H), 7.14 (d, *J* = 7.7 Hz, 1H), 6.99 (d, *J* = 7.4 Hz, 1H), 6.75 (dd, *J* = 8.6, 0.8 Hz, 1H), 6.47 (d, *J* = 2.1 Hz, 1H), 3.92 (s, 3H), 3.38 (dd, *J* = 11.8, 4.7 Hz, 1H), 3.02 (dd, *J* = 4.1, 1.9 Hz, 1H), 2.51 (s, 3H), 2.42 (ddd, *J* = 13.1, 11.8, 6.0 Hz, 1H), 2.16 (dd, *J* = 13.0, 4.7 Hz, 1H), 2.05 – 1.99 (m, 1H), 1.83 (ddt, *J* = 35.6, 13.9, 2.8 Hz, 2H), 1.72 (t, *J* = 2.0 Hz, 1H), 1.69 – 1.61 (m, 1H), 1.59 – 1.48 (m, 2H), 1.35 – 1.20 (m, 2H), 1.00 (s, 3H), 0.90 (qd, *J* = 13.0, 3.6 Hz, 1H).
^{13}C NMR (151 MHz, CD_2Cl_2) δ 163.95, 161.09, 157.22, 143.76, 137.43, 135.97, 135.84, 129.93, 128.44, 120.87, 120.56, 110.85, 84.52, 73.68, 55.33, 53.76, 52.84, 51.81, 41.11, 37.47, 34.09, 32.71, 26.29, 24.67, 24.03, 23.95.
2D NMR HSQC, NOESY
HRMS Calculated $C_{26}H_{30}N_2O_2$ $[M+H]^+$ 403.2386 | Found: 403.2400

Suzuki cross coupling $O(CH_2)_4N$-pyrridyl (22)

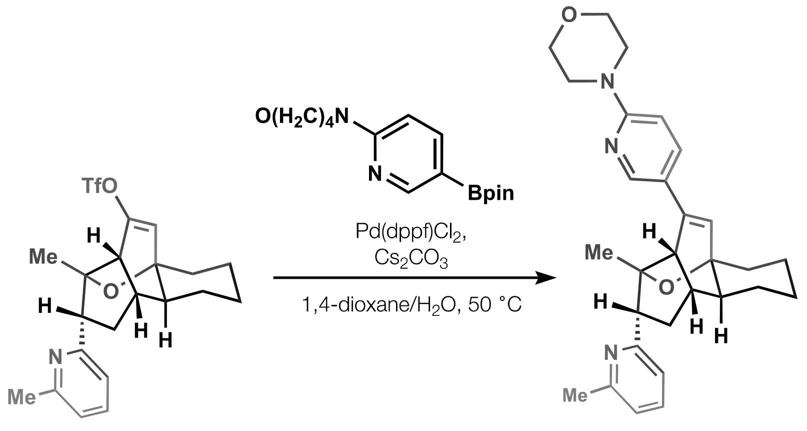

11 22

A solution of *rac*-vinyl triflate iodide **11** (200.0 µL, 18.4 mg, 36.64 µmol, 1 equiv., PhH solution) was added to a flame-dried culture tube (Fisherbrand 14-957-76A, 100 mm length, 8 mL volume) dried under N_2 then high vacuum. An oven-dried stir bar, Pd(dppf)Cl_2 (1.0 mg, 1.4 µmol, 3 mol%), 4-[5-(4,4,5,5-tetramethyl-1,3,2-dioxaborolan-2-yl)pyridin-2-yl]morpholine (60.0 mg, 270.0 µmol, 5 equiv.), and Cs_2CO_3 (135.0 mg, 415.0 µmol, 10 equiv.) were added to the tube. Then it was placed under Ar (3 × evacuate/backfill cycles). 2.0 mL pre-sparged dioxane and 500 µL miliQ-H_2O were added to the solids resulting in a clear deep red solution. The mixture was heated to 50 °C in a heating block. After 12 h, TLC analysis indicate full consumption of starting material. The reaction was filtered through Celite and Na_2SO_4 plug. The solution was concentrated under a positive stream of N_2 for several hours. The resulting yellowish-red waxy residue was purified by two rounds of preparative TLC (two plates each run, 20 × 20 cm, 50% EtOAc/Hex, then 40% EtOAc/CH_2Cl_2) to afford pure **22** (15.2 mg, 80% yield).

Characterization data for **22**
Description white powder
TLC R_f = 0.20 (SiO_2, 50% EtOAc/Hex); UV-active and slightly fluorescent, stains turquoise in anisealdehyde
^{1}H NMR (600 MHz, C_6D_6) δ 8.62 (d, *J* = 2.6 Hz, 1H), 7.50 (dd, *J* = 8.8, 2.5 Hz, 1H), 7.22 – 7.13 (m, 1H), 7.09 (d, *J* = 7.7 Hz, 1H), 6.67 (d, *J* = 7.4 Hz, 1H), 6.41 (d, *J* = 2.1 Hz, 1H), 6.30 (d, *J* = 8.7 Hz, 1H), 3.54 (dd, *J* = 5.5, 4.2 Hz, 4H), 3.40 – 3.29 (m, 6H), 2.87 (dd, *J* = 4.0, 2.0 Hz, 1H), 2.51 (s, 3H), 2.41 (dd, *J* = 13.0, 4.6 Hz, 1H), 2.31 – 2.17 (m, 2H), 2.02 (dt, *J* = 12.2, 3.6 Hz, 1H), 1.72 – 1.28 (m, 5H), 1.22 (s, 4H), 0.92 (qd, *J* = 13.0, 3.6 Hz, 1H).
^{13}C NMR (151 MHz, C_6D_6) δ 161.22, 159.28, 157.02, 145.51, 137.79, 135.49, 134.28, 128.44, 128.35, 125.04, 120.94, 120.32, 106.56, 84.45, 73.53, 66.72, 55.28, 52.76, 51.60, 45.89, 41.22, 37.31, 34.41, 32.75, 26.42, 24.95, 24.75, 24.40, 23.93.
2D NMR HSQC, NOESY
HRMS Calculated $C_{29}H_{35}N_3O_2$ $[M+H]^+$ 458.2808 | Found: 458.2811

Suzuki cross coupling CF_3-pyrazole (23)

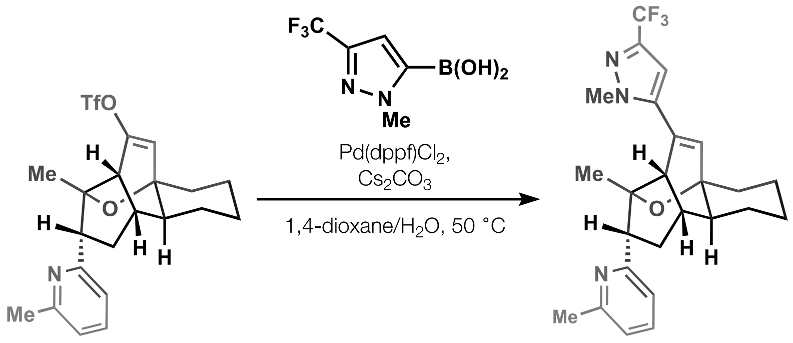

11 23

A solution of *rac*-vinyl triflate iodide **11** (125.0 µL, 12.5 mg, 28.19 µmol, 1 equiv., PhH solution) was added to a flame-dried culture tube (Fisherbrand 14-957-76A, 100 mm length, 8 mL volume) dried under N_2 then high vacuum. An oven-dried stir bar, Pd(dppf)Cl_2 (1.0 mg, 1.4 µmol, 3 mol%), [1-methyl-3-(trifluoromethyl)-1H-pyrazol-5-yl]boronic acid (30.0 mg, 140.9 µmol, 5 equiv.), and Cs_2CO_3 (92.0 mg, 281.9 µmol, 10 equiv.) were added to the tube. Then it was placed under Ar (3 × evacuate/backfill cycles). 2.0 mL pre-sparged dioxane and 500 µL miliQ-H_2O were added to the solids resulting in a clear deep red solution. The mixture was heated to 50 °C in a heating block. After 12 h, TLC analysis indicate full consumption of starting material. The reaction was filtered through Celite and Na_2SO_4 plug. The solution was concentrated under a positive stream of N_2 for several hours. The resulting yellowish-red waxy residue was purified by two rounds of preparative TLC (two plates each run, 20 × 20 cm, 50% EtOAc/Hex, then 40% EtOAc/CH_2Cl_2) to afford pure **23** (8.5 mg, 68% yield).

Characterization data for **23**
Description white powder
TLC R_f = 0.27 (SiO_2, 30% EtOAc/Hex); UV-active, stains weakly turquoise in anisealdehyde
^{1}H NMR (600 MHz, $CDCl_3$) δ 7.49 (t, J = 7.7 Hz, 1H), 7.15 (d, J = 7.7 Hz, 1H), 6.98 (d, J = 7.6 Hz, 1H), 6.49 – 6.45 (m, 2H), 4.01 (s, 3H), 3.39 (dd, J = 11.8, 4.8 Hz, 1H), 2.80 (dd, J = 4.2, 1.9 Hz, 1H), 2.53 (s, 3H), 2.45 (ddd, J = 13.2, 11.8, 5.9 Hz, 1H), 2.17 (dd, J = 13.2, 4.8 Hz, 1H), 2.09 (ddd, J = 13.2, 4.0, 2.1 Hz, 1H), 1.95 (dd, J = 11.7, 3.6 Hz, 1H), 1.90 – 1.83 (m, 1H), 1.77 (ddd, J = 6.0, 4.2, 2.1 Hz, 1H), 1.69 (dt, J = 13.2, 3.2 Hz, 1H), 1.62 (s, 1H), 1.52 – 1.23 (m, 3H), 1.09 (s, 3H), 0.88 (qd, J = 13.2, 3.8 Hz, 1H).
^{13}C NMR (151 MHz, $CDCl_3$) δ 160.11, 157.10, 143.37, 141.40 (d, J = 38.1 Hz)., 135.93, 135.91, 129.75, 120.79, 120.58, 103.44, 103.43, 84.47, 73.38, 54.90, 53.54, 52.20, 40.58, 39.11, 37.53, 33.50, 32.65, 25.82, 24.77, 23.95, 23.74.
2D NMR HSQC, NOESY
HRMS Calculated $C_{25}H_{28}F_3N_3O$ $[M+H]^+$ 444.2263 | Found: 444.2276

Suzuki cross-coupling N-Bn-pyrazole (24)

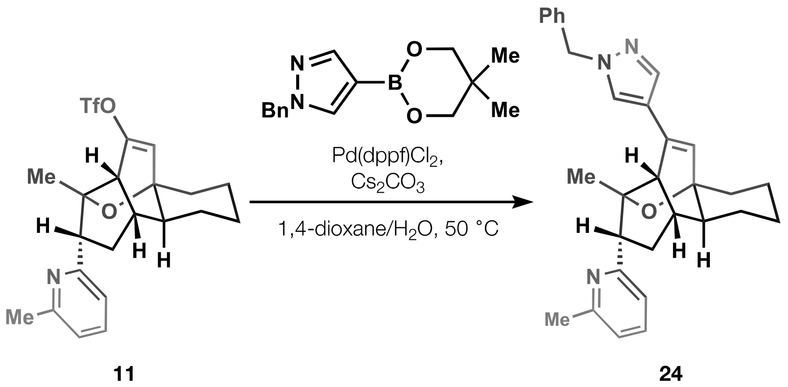

A solution of *rac*-vinyl triflate iodide **11** (125.0 µL, 12.5 mg, 28.19 µmol, 1 equiv., PhH solution) was added to a flame-dried culture tube (Fisherbrand 14-957-76A, 100 mm length, 8 mL volume) dried under N_2 then high vacuum. An oven-dried stir bar, Pd(dppf)Cl_2 (1.0 mg, 1.4 µmol, 5 mol%), N-Bn-pyrazole-4-Bnep (38.0 mg, 140.9 µmol, 5 equiv.), and Cs_2CO_3 (92.0 mg, 281.9 µmol, 10 equiv.) were added to the tube. Then it was placed under Ar (3 × evacuate/backfill cycles). 2.0 mL pre-sparged dioxane and 500 µL miliQ-H_2O were added to the solids resulting in a clear deep red solution. The mixture was heated to 50 °C in a heating block. After 12 h, TLC analysis indicate full consumption of starting material. The reaction was filtered through Celite and Na_2SO_4 plug. The solution was concentrated under a positive stream of N_2 for several hours. The resulting yellowish-red waxy residue was purified by two rounds of preparative TLC (two plates each run, 20 × 20 cm, 50% EtOAc/Hex, then 40% EtOAc/CH_2Cl_2) to afford pure **24** (8.5 mg, 68% yield).

Characterization data for **24**
Description white powder
TLC R_f = 0.30 (SiO_2, 40% EtOAc/Hex); UV-active, stains turquoise in anisealdehyde
^{1}H NMR (600 MHz, CD_2Cl_2) δ 7.65 (d, *J* = 0.8 Hz, 1H), 7.52 – 7.45 (m, 2H), 7.40 – 7.30 (m, 3H), 7.30 – 7.21 (m, 2H), 7.13 (d, *J* = 7.7 Hz, 1H), 6.98 (d, *J* = 7.6 Hz, 1H), 6.29 (d, *J* = 1.9 Hz, 1H), 5.28 (d, *J* = 3.7 Hz, 2H), 3.33 (dd, *J* = 11.7, 4.8 Hz, 1H), 2.78 (dd, *J* = 4.1, 1.8 Hz, 1H), 2.50 (s, 3H), 2.37 (ddd, *J* = 13.0, 11.7, 5.9 Hz, 1H), 2.11 (dd, *J* = 13.0, 4.8 Hz, 1H), 1.96 (ddd, *J* = 13.1, 3.9, 2.1 Hz, 1H), 1.83 – 1.72 (m, 2H), 1.68 (ddd, *J* = 6.2, 4.1, 2.2 Hz, 1H), 1.59 (s, 1H), 1.49 (dddd, *J* = 21.6, 9.6, 7.8, 3.9 Hz, 2H), 1.26 (dddd, *J* = 14.3, 13.1, 7.5, 3.6 Hz, 2H), 0.97 (s, 3H), 0.95 – 0.85 (m, 1H).
^{13}C NMR (151 MHz, CD_2Cl_2) δ 160.83, 156.77, 136.84, 136.32, 135.39, 131.86, 128.74, 128.00, 127.77, 126.38, 125.61, 122.43, 120.45, 120.11, 83.93, 73.20, 56.07, 55.05, 52.91, 52.44, 40.56, 37.17, 33.77, 32.27, 25.92, 24.29, 23.57, 23.52.
2D NMR HSQC, NOESY
HRMS Calculated $C_{30}H_{33}N_3O$ $[M+H]^+$ 452.2702 | Found: 452.2715

Syntheses of RS1176 (26)

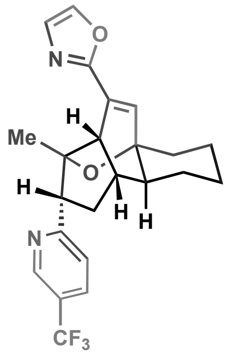

RS1176 (26)

Procedure A:

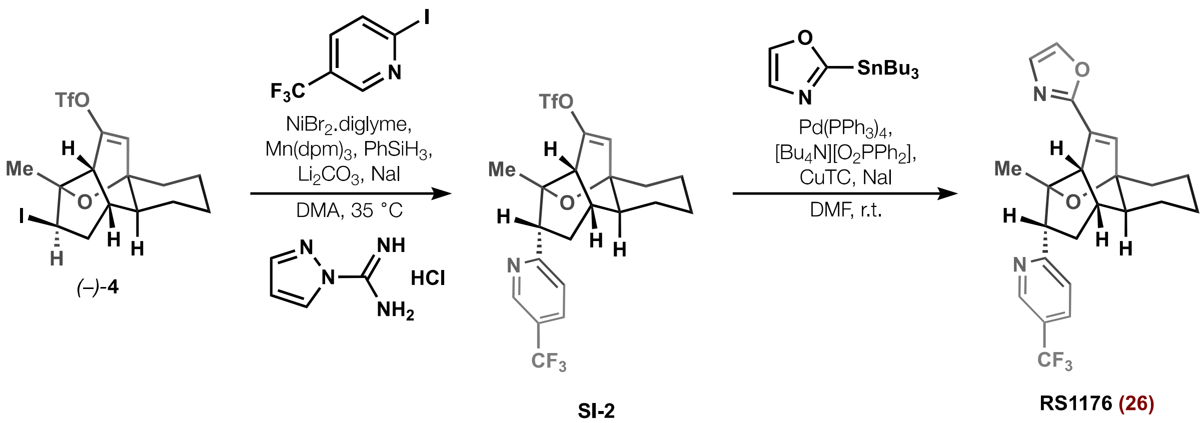

Cross-electrophile coupling (see page S21) *(−)*-**4** (100 mg, 0.21 mmol) was charged to a 100 mL round-bottom flask equipped with an egg-shaped stir bar, followed by oven-dried NaI (270 mg, 1.8 mmol), oven-dried Li_2CO_3 (22.0 mg, 0.30 mmol), 1*H*-pyrazole-1-carboxamidine hydrochloride (88 mg, 0.60 mmol), 2-iodo-5-trifluoromethylpyridine (246 mg, 0.90 mmol), $NiBr_2$·diglyme (233 mg, 0.66 mmol) and $Mn(dpm)_3$ (254 mg, 0.42 mmol). The flask was placed under Ar (3x evacuate/backfill cycles) and charged with dry, degassed DMA (9 mL). The reaction mixture was stirred at 1200 rpm with Ar sparging (20G Ar balloon inlet, 20G outlet), upon which a turbid light reddish-brown mixture was observed. After 30 min, the outlet needle was removed and $PhSiH_3$ (148 µL, 1.2 mmol) was added. The reaction flask was immediately placed in a pre-heated 35 °C water bath. After 1 h, the reaction mixture appeared black. After 3 h, TLC analysis indicated full conversion of starting material, and the reaction mixture was allowed to cool to room temperature, diluted with ice-cold water (20 mL) and extracted with EtOAc (5 x 20 mL). The combined organic extracts were washed with half-saturated brine (20 mL), dried over Na_2SO_4, and concentrated to dryness under reduced pressure then under a stream of nitrogen over several hours to remove high-boiling volatiles. The residue (270 mg) was subjected to column chromatography (SiO_2, 17 cm x 2.5 cm, minimal CH_2Cl_2/PhMe loading, 0-5% EtOAc/hexanes then 5% acetone/hexanes). The impure, separated diastereomers were each further purified by 2 rounds of preparative TLC (round 1: SiO_2, 20 cm x 20 cm x 0.5 mm, 10% acetone/hexanes; round 2: SiO_2, 20 cm x 20 cm x 0.5 mm, 30% Et_2O/hexanes) to give major, **SI-2** (21.2 mg, 20% yield) and minor, ***epi*-SI-2** (4.2 mg, 4% yield) (see note 1).

Modified Stille conditions from Corey/Fürstner and coworkers.[2,3]

Stille coupling **SI-2** (16.0 mg, 0.032 mmol) was charged to an 8 mL culture tube. An oven-dried stir bar, NaI (18.0 mg, 0.12 mmol), [Bu_4N][$OPOPh_2$] (28.0 mg, 0.06 mmol), CuTC (11.0 mg, 0.06 mmol) and $Pd(PPh_3)_4$ (7.0 mg, 0.006 mmol) were added and the tube was placed under Ar (3x evacuate/backfill cycles) (see note 2). Anhydrous, degassed DMF (1.5 mL) was added and a thick whitish slurry was obtained. After 1 min of stirring at 1200 rpm, 2-(tri-*n*-butylstannyl)oxazole (9.4 µL, 0.045 mmol) was added and a slurry with a light milk-chocolate appearance was obtained. After an additional 90 min stirring, LCMS and TLC analysis indicated full conversion of starting material. The reaction was diluted into water (20 mL) to give a voluminous precipitate. The mixture was extracted with EtOAc (4x), and the organic layers were filtered through a PTFE syringe filter, dried over Na_2SO_4 and concentrated (80 mg). Purification of the residue by 2 rounds of preparative TLC (round 1: SiO_2, 20 cm x 20 cm x 0.5 mm, 25% acetone/hexanes; round 2: SiO_2, 20 cm x 20 cm x 0.5 mm, 1% (7N NH_3/MeOH)/(10% EtOAc/hexanes)) afforded oxazole **26** (10.5 mg, 79%) (see note 3). **26** from this batch was advanced to biological experimentation (see page S134).

Note – 1 Stereochemistry of **SI-2** was characterized by analogy to page S8
Note 2 The precedent described the formation of a RCu(I) species as reactive transmetallation coupling partner under the reaction condition with minimal side reactivity e.g., *cine* substitution–a common major byproduct in Stille coupling with hindered substrates as follows,

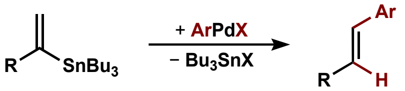

The referenced works proposed that the influence of Cu(I) will decrease with the increase of conversion due to the build-up of Bu_3SnX. They described the additive [Bu_4N][O_2PPh_2] as tin scavenger under Pd/Cu Stille coupling condition.
Note – 3 SiO_2 waste was disposed accordingly due to exposure of toxic organotin reagents.

Procedure B:

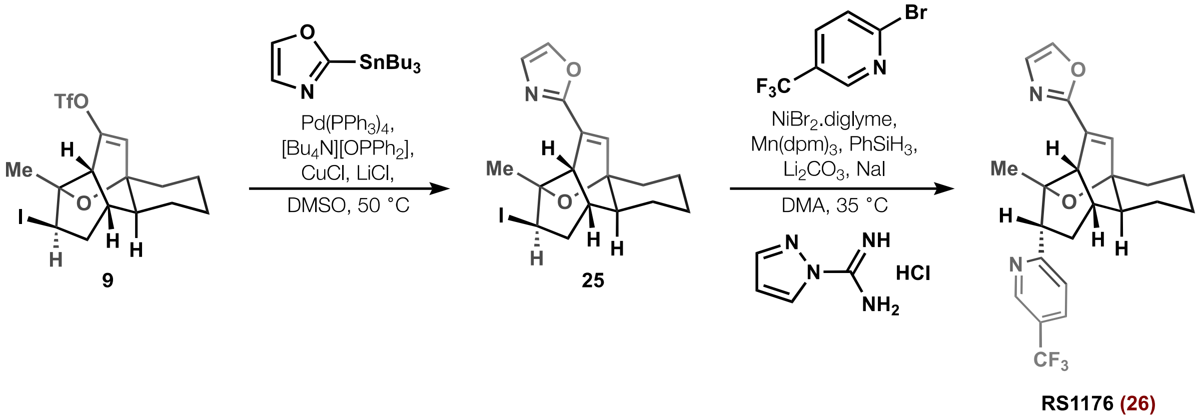

Stille A solution of *rac*-iodo-triflate **9** (54.0 mg, 113.0 µmol, 300 µL, 0.37 M PhH, 1 equiv.) and a separate solution of 2-(tributylstannyl)oxazole (202.0 mg, 565.0 µmol, 1.0 M PhH, 5 equiv.) was charged to a flame-dried culture tube (Fisherbrand 14-956-25E, 20 × 125 mm, 25 mL volume) and dried under a positive N_2 stream then high vacuum. An oven-dried samarium-cobalt stir bar (see note 4), [Bu_4N][O_2PPh_2] (520.0 mg, 10 equiv.), CuCl (112.0 mg, 10 equiv.), oven-dried LiCl (48.0 mg, 10 equiv.), and $Pd(PPh_3)_4$ (30.0 mg, 0.25 equiv.) were added and the tube was placed under Ar (3 × evacuate/backfill cycles). Degassed DMSO (11.3 mL, 10 mM) was added, and the colloidal light green mixture was stirred at 1500 rpm at 50 °C. After 3 hrs, TLC indicated full consumption of starting material. The mixture was cooled to room temperature, diluted with 25% aq. NH_4OH (see note 5), and extracted with EtOAc (3 × 1 equal

parts with respect to the volume added for 25% NH_4OH). The organic phase was washed with brine, then back extract with EtOAc (2 × 5 mL). The organic phase was dried over Na_2SO_4 and concentrated under reduced pressure. The residue was purified by column chromatography 5-10-20% EtOAc/Hex to afford *rac*-oxazole iodide **25** as a crystalline white solid (41.0 mg, 91%) (see note 3).

Cross-electrophile coupling A solution of *rac*-oxazole iodide **25** (10.0 mg, 25.2 µmol, 100 µL, 0.25 M in PhH, 1 equiv.) was charged to a flame-dried culture tube (Fisherbrand 14-959-25A, 13 × 100 mm, 8 mL volume) and dried under high vacuum. An oven-dried stir bar, $NiBr_2$·diglyme (30.0 mg, 75.5 µmol, 3 equiv.), $Mn(dpm)_3$ (30.0 mg, 50.3 µmol, 2 equiv), oven-dried Li_2CO_3 (3.0 mg, 37.7 µmol, 1.5 equiv.), oven-dried NaI (34.0 mg, 226.6 µmol, 9 equiv.), and 2-bromo-5-(trifluoromethyl)pyridine (14.0 mg, 63.0 µmol, 2.5 equiv.) were added. The tube was placed under Ar (3 × evacuate/backfill cycles). 800 µL DMA (0.033 M of **25**) was added and the turbid blue mixture was sparged with Ar at 1500 rpm for 30 mins. A colloidal yellow-brown mixture was obtained. The septum was changed quickly under Ar, then $PhSiH_3$ (20 µL, 151.0 µmol, 6 equiv.) was added to the mixture. The Ar balloon was taken out and the septum was sealed with telfon tape, electrical tape, and parafilm (see note 6). The mixture was heated in a 35 °C water bath. After 16 hrs, TLC analysis indicated significant consumption of starting material. The mixture was then diluted with EtOAc (2 mL), and pre-chilled H_2O (2 mL) was added. The biphasic mixture was extracted with EtOAc (3 × 5 mL) (see note 7). The organic phase was washed with half-saturated brine, then back extract with EtOAc (2 × 5 mL). The organic phase was dried over Na_2SO_4, and concentrated under reduced pressure. The DMA-containing residue was loaded to two 20 × 20 cm TLC plates, and then elute with 100% CH_2Cl_2 to give semipurified mixture containing both diastereomers of *rac*-**26** (26% qNMR yield, 1:5 exo/endo).

Note – 4 Samarium-cobalt stir bars i.e., rare Earth magnetic stir bars were chosen due to the heterogenous reaction mixture.
Note – 5 Typically, aq. 20% NH_4OH was added until the slurry/emulsion turned to a biphasic dark blue (aqueous phase) and clear with a tint of indigo (organic phase). Typically 10-15 mL was needed on this scale.
Note – 6 As per original procedure in ref. 1, the presence of air/O_2 is either non-beneficial or detrimental. Here we found that sealing the pierced septum with the forementioned tapes helped with reproducibility on small-scale runs.
Note – 7 Significant formation of emulsion between the two phases could form. Small amounts of sat. aqueous LiCl solution could be added to the pre-shaken mixture to break down the emulsion without further homogenizing, and the phases were separated.

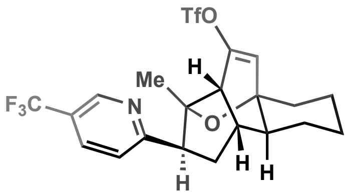

***epi*-SI-2**

<u>Characterization data for vinyl triflate *epi*-**SI-2**</u>
Description colorless film
TLC R_f = 0.59 (SiO_2, 10% acetone/hexanes); UV-active, stains olive/light khaki in anisaldehyde
^{1}H NMR (600 MHz, C_6D_6) δ 8.60 (d, J = 2.4 Hz, 1H), 7.08 (dd, J = 8.1, 2.4 Hz, 1H), 6.44 (d, J = 8.0 Hz, 1H), 5.84 (d, J = 2.7 Hz, 1H), 3.26 (dd, J = 8.8, 7.2 Hz, 1H), 3.21 (dd, J = 4.1, 2.7

Hz, 1H), 2.41 (ddd, *J* = 12.6, 7.2, 5.4 Hz, 1H), 1.96 – 1.87 (m, 1H), 1.87 – 1.80 (m, 1H), 1.78 (dd, *J* = 12.6, 8.8 Hz, 1H), 1.51 – 1.37 (m, 3H), 1.30 – 1.25 (m, 1H), 1.18 (ddd, *J* = 13.0, 4.0, 2.2 Hz, 1H), 1.10 – 0.90 (m, 2H), 0.82 (qd, *J* = 12.9, 3.6 Hz, 1H), 0.66 (s, 3H).
^{13}C NMR (151 MHz, C_6D_6) δ 167.2, 148.7, 146.7 (q, *J* = 4.0 Hz), 133.1 (q, *J* = 3.4 Hz), 124.34 (q, *J* = 270 Hz), 124.28 (q, *J* = 33 Hz), 124.0, 121.7, 119.3 (q, *J* = 320 Hz), 86.7, 76.0, 56.8, 53.4, 50.5, 41.5, 38.6, 34.4, 31.9, 25.8, 23.3, 22.5.
^{19}F NMR (376 MHz, C_6D_6) δ -64.7, -76.6.
2D NMR HSQC, HMBC, COSY, ^{1}H-^{19}F HOESY
HRMS Calculated $C_{21}H_{22}F_6NO_4S$ $[M+H]^+$ 498.1174 | Found: 498.1177

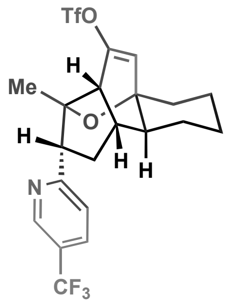

SI-2

Characterization data for vinyl triflate **SI-2**
Description colorless oil
TLC R*f* = 0.63 (SiO_2, 10% acetone/hexanes); UV-active, stains olive/light khaki in anisaldehyde
^{1}H NMR (600 MHz, C_6D_6) δ 8.85 – 8.76 (m, 1H), 7.25 (dd, *J* = 8.2, 2.5 Hz, 1H), 6.71 (d, *J* = 8.2 Hz, 1H), 5.72 (d, *J* = 2.6 Hz, 1H), 2.93 (dd, *J* = 11.5, 4.2 Hz, 1H), 2.35 (dd, *J* = 4.1, 2.6 Hz, 1H), 2.24 (dd, *J* =z 13.0, 4.2 Hz, 1H), 1.94 (ddd, *J* = 13.3, 3.9, 2.1 Hz, 1H), 1.85 (ddd, *J* = 13.0, 11.5, 5.8 Hz, 1H), 1.69 – 1.60 (m, 2H), 1.45 – 1.36 (m, 2H), 1.36 – 1.30 (m, 1H), 1.19 (td, *J* = 13.0, 4.1 Hz, 1H), 1.11 – 0.87 (m, 5H), 0.80 (qd, *J* = 13.0, 3.5 Hz, 1H).
^{13}C NMR (151 MHz, C_6D_6) δ 164.6, 148.3, 145.7 (q, *J* = 4.0 Hz), 132.4 (q, *J* = 3.4 Hz), 124.6 (q, *J* = 270 Hz), 124.2 (q, *J* = 33 Hz), 123.5, 122.7, 119.3 (q, *J* = 320 Hz), 85.3, 75.7, 54.4, 52.9, 51.7, 41.2, 35.3, 33.7, 31.9, 25.7, 24.0, 23.3.
^{19}F NMR (376 MHz, C_6D_6) δ -64.5, -76.6.
2D NMR HSQC, HMBC, COSY, NOESY, ^{1}H-^{19}F HOESY
HRMS Calculated $C_{21}H_{22}F_6NO_4S$ $[M+H]^+$ 498.1174 | Found: 498.1177

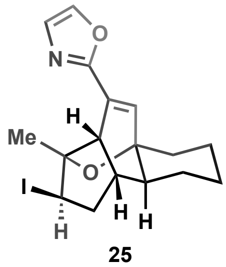

25

Characterization data for oxazole iodide **25**
Description white solid
TLC R*f* = 0.50 (SiO_2, 20% EtOAc/hexanes); UV-active, stains purple in anisaldehyde
^{1}H NMR (600 MHz, C_6D_6) δ 6.97 (d, *J* = 1.7 Hz, 1H), 6.94 (s, 1H), 6.88 (d, *J* = 0.8 Hz, 1H), 4.27 (dd, *J* = 8.3, 7.2 Hz, 1H), 3.74 (dd, *J* = 4.0, 1.7 Hz, 1H), 2.30 (ddd, *J* = 13.2, 7.1, 5.6 Hz, 1H), 2.02 (dd, *J* = 13.6, 8.3 Hz, 1H), 1.92 (dd, *J* = 12.6, 3.5 Hz, 1H), 1.54 (s, 3H), 1.49 – 1.42

(m, 1H), 1.34 – 1.26 (m, 2H), 1.14 (tt, J = 13.5, 3.3 Hz, 2H), 0.99 (ddd, J = 12.0, 4.8, 2.5 Hz, 1H), 0.93 – 0.76 (m, 2H), 0.53 (td, J = 13.1, 3.7 Hz, 1H).
^{13}C NMR (151 MHz, C_6D_6) δ 160.47, 138.22, 135.41, 128.89, 128.48, 83.43, 73.51, 52.16, 46.38, 44.42, 40.70, 33.72, 33.43, 31.62, 27.28, 25.40, 23.04.
2D NMR HSQC, HMBC, COSY, NOESY
HRMS Calculated $C_{17}H_{20}INO_2$ [M+H]$^+$ 398.0617 | Found: 398.0630

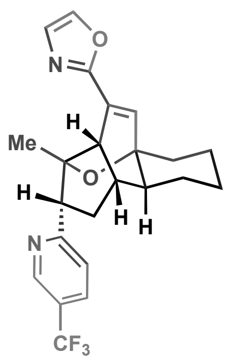

RS1176 (26)

Characterization data for oxazole **RS1176**
Description colorless oil
TLC R_f = 0.35 (SiO_2, 20% acetone/hexanes); UV-active, stains gold in anisaldehyde
optical rotation $[\alpha]_D^{20}$= +29 (c = 0.14, EtOAc)
^{1}H NMR (600 MHz, C_6D_6) δ 8.89 – 8.79 (m, 1H), 7.25 (dd, J = 8.2, 2.4 Hz, 1H), 7.07 (d, J = 1.7 Hz, 1H), 7.01 (d, J = 0.9 Hz, 1H), 6.95 (d, J = 0.9 Hz, 1H), 6.79 (d, J = 8.2 Hz, 1H), 3.64 (dd, J = 4.1, 1.7 Hz, 1H), 3.17 (dd, J = 11.5, 4.2 Hz, 1H), 2.35 (dd, J = 12.9, 4.2 Hz, 1H), 2.11 (ddd, J = 13.4, 4.0, 2.2 Hz, 1H), 2.06 (ddd, J = 12.8, 11.5, 5.8 Hz, 1H), 1.88 – 1.79 (m, 1H), 1.59 (ddd, J = 5.9, 4.0, 2.1 Hz, 1H), 1.55 – 1.46 (m, 1H), 1.46 – 1.38 (m, 1H), 1.38 – 1.27 (m, 2H), 1.26 – 1.08 (m, 5H), 0.75 (qd, J = 13.0, 3.7 Hz, 1H).
^{13}C NMR (151 MHz, C_6D_6) δ 165.5, 161.3, 145.6 (q, J = 4.0 Hz), 138.5, 137.1, 132.2 (q, J = 3.4 Hz), 129.1, 128.8, 124.8 (q, J = 270 Hz), 123.9 (q, J = 32 Hz), 123.6, 84.9, 73.5, 55.2, 52.0, 49.5, 40.9, 36.0, 33.7, 32.4, 26.0, 24.1, 23.6.
^{19}F NMR (376 MHz, C_6D_6) δ -64.5.
2D NMR HSQC, HMBC, COSY, NOESY, ^{1}H-^{19}F HOESY
HRMS Calculated $C_{23}H_{24}F_3N_2O_2$ [M+H]$^+$ 417.1790 | Found: 417.1799

Syntheses of RS1159 (27)

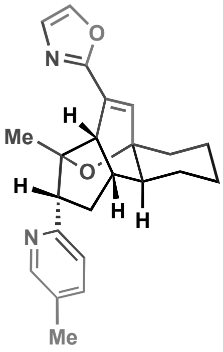

RS1159/RS1144 (27)

Note – **RS1144** and **RS1159** are the same compound i.e., **27**. Our internal records define resynthesized compounds that were submitted for biological experiments separately. Below, **RS1144** and **RS1159** will be defined as **27** for clarity.

Procedure A:

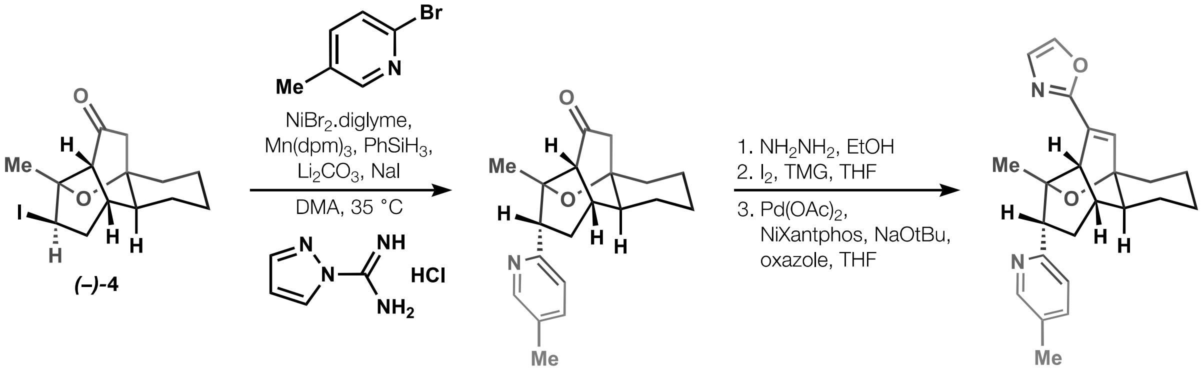

Cross-electrophile coupling (see page S21) A flame-dried 20 mL glass tube with threaded end was equipped with a stir bar and septum pierced with an needle fed by a balloon filled with argon. It was sequentially charged with praxadine.HCl (85.0 mg, 578 µmol, 2 equiv.), $NiBr_2$•diglyme (224 mg, 635 µmol, 2.2 equiv.), $Mn(dpm)_3$ (245 mg, 404 µmol, 1.4 equiv.), 2-bromo-5-methyl pyridine (149 mg, 867 µmol, 3 equiv), *(−)*-**4** (100 mg, 289 µmol, 1 equiv.), Li_2CO_3 (21.0 mg, 289 µmol, 1 equiv.), and NaI (260 mg, 1.73 mmol, 6 equiv.). The vial was evacuated and backfilled with argon (5x) and then charged with DMA (dry, 8.8 mL) using a syringe, and the reaction mixture was degassed with an argon balloon and a vent needle while stirring for 45 min. The reaction mixture turned brown at the end of the degassing period. The vent needle was removed, the septum and argon balloon replaced. Silylbenzene (125 mg, 143 µL, 578 µmol, 4 equiv.) was added using a syringe, then the reaction mixture was placed into a preheated 35 °C water bath. The reaction underwent a series of changes in appearance similar to XEC with 2-iodo-6-methyl pyridine from turbid brown-yellow to turbid yellow-olive to turbid pastel green/light jade to turbid dark jade within a timespan of 15 – 30 min. The reaction was stirred for 6 h. Upon reaction completion (No SM mass found on LC-MS, faint SM spot remaining, dark forest green colored reaction mixture) EtOAc (8.8 mL) and ice-cold water (17 mL) were added. The layers were separated and the aqueous layer was extracted with EtOAc (6 x 2mL). The combined organic layers were washed with half saturated brine (2 x 8.8 mL) and brine (2 mL). The combined washes were back extracted with EtOAc (2 mL) and the back extract was washed with half saturated brine (2 x 2 mL), then combined with the organic layer, dried over $MgSO_4$, filtered and concentrated *in vacuo*. The crude product was purified and diastereomers were separated by column chromatography (SiO_2, load in toluene, elute 10-15-20-25-30-35-40% EtOAc in hexanes).

Exo-diastereomer. **8q** The product containing fractions were concentrated *in vacuo* and resulting oil was further purified by two round of column chromatography (SiO_2, load in toluene, elute 10% acetone in hexanes) affording 2.7 mg (3% yield) of ketone **8q** as a colorless oil.
Endo-diastereomer. **7q** The product containing fractions were concentrated *in vacuo* and the resulting oil was further purified by column chromatography (SiO_2, load in toluene, elute 10% acetone in hexanes) affording 13 mg (14% yield) of ketone **7q** as a white solid.

Hydrazone condensation Cross-electrophile coupling product ketone **7q** (13 mg, 0.041 mmol) was dissolved in EtOH (500 µL) and $CHCl_3$ (50 µL) then charged with hydrazine hydrate (49 µL, 1 mmol). The mixture was heated to 40 °C for 21 h, concentrated to dryness under nitrogen, then dried with PhMe/MeOH azeotrope repeatedly until dry, then PhMe (3x) then hexanes (2x) and placed under high vacuum.

Barton iodination To a flamed culture tube (Fisherbrand 14-957-76A, 100 mm length, 8 mL volume) under Ar with septum-lined screw cap (Chemglass CG-4910-15) was added iodine (22.6 mg, 0.089 mmol) and anhydrous, inhibitor-free THF (80 µL). The resulting dark red-brown solution was placed in an ice bath and 1,1,3,3-tetramethylguanidine (356 µL, 2.84 mmol) was added dropwise. A solution of the crude hydrazone from the previous reaction in THF (200 µL) was added dropwise, quantitating the transfer with THF (3 x 200 µL). Upon complete transfer of the mixture of crude hydrazones, the reaction turned clear yellow, and the ice bath was removed. After 40 min, the reaction was cooled to 0 °C and quenched with a mixture of saturated Na_2SO_3 (3 mL) and saturated $NaCO_3$ (1 mL). The mixture was extracted with EtOAc (3x) and the combined organics were dried over Na_2SO_4 and concentrated under reduced pressure giving a crude residue (15 mg). Column chromatography (SiO_2, PhMe loading, 20% EtOAc/hexanes) afforded vinyl iodide (11.7 mg, 74%) (m/z = +422). This material was used without further purification for the next reaction.

CH-arylation Vinyl iodide (7.4 mg, 0.017 mmol, assume 100% purity) was charged to an 8 mL culture tube. $Pd(OAc)_2$ (1.0 mg, 0.0034 mmol) and NiXantphos (4.0 mg, 0.0068 mmol) were added, and the tube was placed under Ar (3x evacuate/backfill cycles). Oxazole (26 µL, 0.4 mmol) and KOtBu (1.0 M in THF solution, 800 µL) were simultaneously injected rapidly into bottom of the reaction tube at 22 °C, forming a clear orange-brown solution, which gradually went turbid dark green over the course of 10 min. The reaction was stirred for 11 h and a milk chocolate-like slurry was observed. LCMS analysis indicated complete conversion of starting material (m/z = +442). The reaction was quenched with a mixture of saturated aqueous $NaHCO_3$ (2 mL)/saturated aqueous Na_2CO_3 (2 mL) and extracted with EtOAc (3x). The combined organics were dried over Na_2SO_4 and concentrated to dryness to afford a blackish-dark purple residue (13 mg). The residue was purified by 2 rounds of preparative TLC (round 1: SiO_2, EtOAc; round 2: SiO_2, 4% (7N NH_3/MeOH)/(20% EtOAc/hexanes)) to give oxazole **27** (1.7 mg, 21% over 3 steps). **27** from this batch was advanced to biological experiments (see page S134).

Alternatively, higher yields were obtained by pre-formation of the Pd-NiXantphos complex:
In a 1 dram vial, $Pd(OAc)_2$ (4 mg, 0.017 mmol) and NiXantphos (14 mg, 0.025 mmol) were placed under Ar (3x evacuate/backfill cycles) and taken up in anhydrous degassed THF (1 mL). The resulting dark greenish-black mixture containing a clump of black solid was sonicated to give a fine red-brown "homogeneous suspension", solution A.
Separately, vinyl iodide (7.0 mg, 0.017 mmol, assume 100% purity) was charged to an 8 mL culture tube and placed under Ar (3x evacuate/backfill cycles). Oxazole (26 µL, 0.4 mmol) and

NaOtBu (1.0 M in THF solution, 800 µL) were simultaneously injected rapidly into bottom of the reaction tube at 22 °C. The resulting solution was then charged with solution A (200 µL, 0.0034 mmol Pd). The resulting faintly translucent orange solution was stirred at 22 °C under Ar for 15 h to give a clear red-orange solution, which was cooled to 0 °C, quenched with a mixture of saturated aqueous $NaHCO_3$ (1.5 mL)/saturated aqueous Na_2CO_3 (1.5 mL)/H_2O (0.5 mL) and extracted with EtOAc (3x). The combined organics were dried over Na_2SO_4 and concentrated to dryness to afford a residue, which was purified by 2 rounds of preparative TLC (SiO_2, 4% (7N NH_3/MeOH)/(20% EtOAc/hexanes)) to give oxazole **27** (2.3 mg, 27% over 3 steps). **27** from this batch was advanced to biological experiments (see page S134).

Produre B:

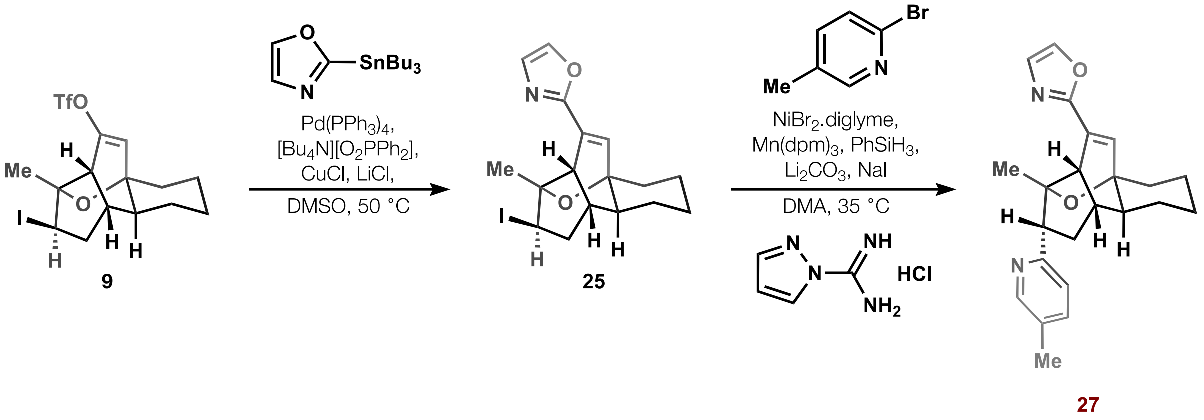

Stille coupling see page S42

Cross-electrophile coupling A solution of *rac*-oxazole iodide **25** (10.0 mg, 25.2 µmol, 100 µL, 0.25 M in PhH, 1 equiv.) was charged to a flame-dried culture tube (Fisherbrand 14-959-25A, 13 × 100 mm, 8 mL volume) and dried under high vacuum. An oven-dried stir bar, $NiBr_2$·diglyme (30.0 mg, 75.5 µmol, 3 equiv.), $Mn(dpm)_3$ (30.0 mg, 50.3 µmol, 2 equiv), oven-dried Li_2CO_3 (3.0 mg, 37.7 µmol, 1.5 equiv.), oven-dried NaI (34.0 mg, 226.6 µmol, 9 equiv.), and 2-bromo-5-methyl pyridine (10 mg, 63.0 µmol, 2.5 equiv) were added. The tube was placed under Ar (3 × evacuate/backfill cycles). 800 µL DMA (0.033 M of **25**) was added and the turbid blue mixture was sparged with Ar at 1500 rpm for 30 mins. A colloidal yellow-brown mixture was obtained. The septum was changed quickly under Ar, then $PhSiH_3$ (20 µL, 151.0 µmol, 6 equiv.) was added to the mixture. The Ar balloon was taken out and the septum was sealed with telfon tape, electrical tape, and parafilm (see note 1). The mixture was heated in a 35 °C water bath. After 16 hrs, TLC analysis indicated significant consumption of starting material. The mixture was then diluted with EtOAc (2 mL), and pre-chilled H_2O (2 mL) was added. The biphasic mixture was extracted with EtOAc (3 × 5 mL) (see note 2). The organic phase was washed with half-saturated brine, then back extract with EtOAc (2 × 5 mL). The organic phase was dried over Na_2SO_4, and concentrated under reduced pressure. The DMA-containing residue was loaded to two 20 × 20 cm TLC plates, and then elute with 100% CH_2Cl_2 to give semipurified diasteromeric mixture of *rac*-**27** (55% yield qNMR , 1:4 exo/endo).

Note-1: As per original procedure in ref. 1, the presence of air/O_2 is either non-beneficial or detrimental. Here we found that sealing the pierced septum with the forementioned tapes helped with reproducibility on small-scale runs.
Note-2: Significant formation of emulsion between the two phases could form. Small amounts of sat. aqueous LiCl solution could be added to the pre-shaken mixture to break down the emulsion without further homogenizing, and the phases were separated.

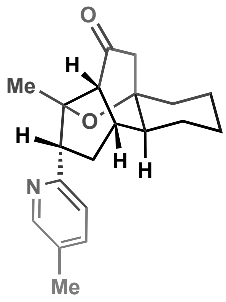

7q

<u>Characterization data for ketone **7q** (and **8q**)</u>

See page S125

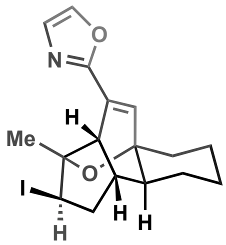

25

<u>Characterization data for oxazole iodide **25**</u>

See page S45

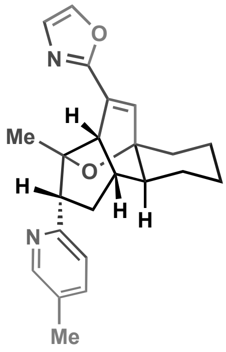

27

<u>Characterization data for **RS1144/RS1159 (27)**</u>

Description colorless film

TLC R_f = 0.35 (SiO_2, 4% (7N NH3 in MeOH)/(20% EtOAc/hexanes)), 0.29 (SiO_2, EtOAc); UV-active, stains blue in CAM

^{1}H NMR (600 MHz, $CDCl_3$) δ 8.36 (bd, J = 1.6 Hz, 1H), 7.64 (s, 1H), 7.42 (dd, J = 8.1, 2.3 Hz, 1H), 7.28 – 7.26 (m, 1H), 7.17 (s, 1H), 7.03 (bs, 1H), 3.47 – 3.41 (m, 2H), 2.52 (ddd, J = 13.2, 11.9, 6.0 Hz, 1H), 2.29 (s, 3H), 2.10 (dd, J = 13.2, 4.8 Hz, 1H), 1.98 (dt, J = 12.4, 3.5 Hz, 1H), 1.93 (ddd, J = 13.3, 3.9, 2.1 Hz, 1H), 1.87 – 1.80 (m, 1H), 1.79 – 1.74 (m, 1H), 1.65 (dt, J = 13.3, 3.4 Hz, 1H), 1.56 – 1.48 (m, 2H), 1.38 (td, J = 12.9, 4.2 Hz, 1H), 1.35 – 1.27 (m, 1H), 1.06 (s, 3H), 0.89 (qd, J = 13.0, 3.7 Hz, 1H).

^{13}C NMR (151 MHz, $CDCl_3$) δ 161.0, 158.3, 149.0, 138.6, 136.6, 136.4, 130.5, 128.7, 128.3, 123.2, 84.7, 73.5, 54.8, 53.4, 49.2, 40.3, 37.6, 33.5, 32.3, 25.7, 23.7, 23.6, 18.2.

2D NMR HSQC, HMBC, COSY

HRMS Calculated $C_{23}H_{27}N_2O_2$ $[M+H]^+$ 363.2073 | Found: 363.2072

Syntheses of RS1160 (28)

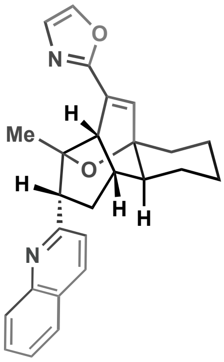

RS1160 (28)

Procedure A:

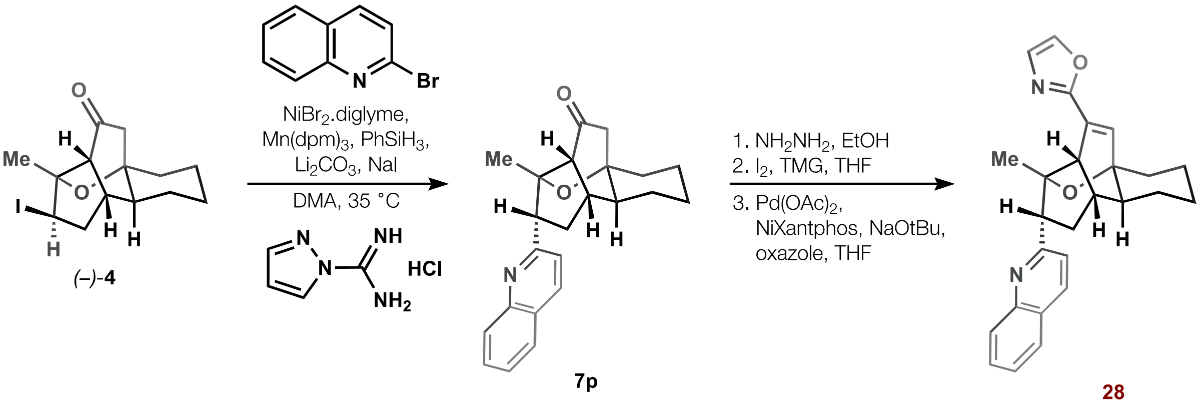

Cross-electrophile coupling (see page S21) A flame-dried 20 mL glass tube with threaded end was equipped with a stir bar and septum pierced with an needle fed by a balloon filled with argon. It was sequentially charged with praxadine.HCl (85 mg, 578 µmol, 2 equiv.), $NiBr_2$•diglyme (224 mg, 635 µmol, 2.2 equiv.), $Mn(dpm)_3$ (245 mg, 404 µmol, 1.4 equiv.), 2-bromo-quinoline (180 mg, 867 µmol, 3 equiv.), *(−)*-**4** (100 mg, 289 µmol, 1 equiv.), Li_2CO_3 (21 mg, 289 µmol, 1 equiv.), and NaI (260 mg, 1.73 mmol, 6 equiv.). The vial was evacuated and backfilled with argon (5x) and then charged with DMA (dry, 8.8 mL) using a syringe, and the reaction mixture was degassed with an argon balloon and a vent needle while stirring for 45 min. The reaction mixture turned brown at the end of the degassing period. The vent needle was removed, the septum and argon balloon replaced. Silylbenzene (125 mg, 143 µL, 578 µmol, 4 equiv.) was added using a syringe, then the reaction mixture was placed into a preheated 35 °C water bath. The reaction underwent a series of changes in appearance similar to XEC with 2-iodo-6-methyl pyridine from turbid brown-yellow to turbid yellow-olive to turbid pastel green/light jade to turbid dark jade within a timespan of 15 – 30 min. The reaction was stirred for 3 h. Upon reaction completion (No SM mass found on LC-MS, faint SM spot remaining, dark forest green reaction mixture) EtOAc (8.8 mL) and ice-cold water (17 mL) were added. The layers were separated and the aqueous layer was extracted with EtOAc (6 x 2mL). The combined organic layers were washed with half saturated brine (2 x 8.8 mL) and brine (2 mL). The combined washes were back extracted with EtOAc (2 mL) and the back extract was washed with half saturated brine (2 x 2 mL), then combined with the organic layer, dried over $MgSO_4$, filtered and concentrated *in vacuo*. The crude product was purified by column chromatography (40 g SiO_2, load in toluene, elute 10-15-20-25-30-35-40% EtOAc in hexanes, Combiflash purification system). The product containing fractions were concentrated *in vacuo* and and further purified by column chromatography (SiO_2, load in toluene, elute 2.5% MeOH in DCM). The diastereomers were separated by column chromatography (SiO_2, load in toluene, elute 5-7.5-10-15-30-50% EtOAc in hexanes).

Exo product. **8p** The product containing fractions were concentrated *in vacuo* and the resulting solid was further purified by preparative TLC (SiO_2, 10 x 10 cm, 0.25 mm thick, load in $CHCl_3$, elute 2.5% EtOAc in DCM) to afford 5.2 mg (5% yield) of ketone **8p** as a white solid.
Endo product. **7p** The product containing fractions were concentrated *in vacuo* and the resulting soldi was further purified by preparative TLC (SiO_2, 20 x 10 cm, 0.5 mm thick, load in $CHCl_3$ on long side, elute 10% EtOAc in hexanes) to afford 22.0 mg (22% yield) of ketone **7p** as a white solid.

Hydrazone condensation Cross-electrophile coupling product ketone **7p** (14 mg, 0.04 mmol) was dissolved in EtOH (500 µL) and $CHCl_3$ (50 µL) then charged with hydrazine hydrate (100 µL, 2 mmol). The mixture was heated to 40 °C for 21 h, concentrated to dryness under nitrogen, then dried with PhMe/MeOH azeotrope repeatedly until dry, then PhMe (3x) then hexanes (2x) and placed under high vacuum.

Barton iodination To a flamed culture tube (Fisherbrand 14-957-76A, 100 mm length, 8 mL volume) under Ar with septum-lined screw cap (Chemglass CG-4910-15) was added iodine (23 mg, 0.089 mmol) and anhydrous, inhibitor-free THF (80 µL). The resulting dark red-brown solution was placed in an ice bath and 1,1,3,3-tetramethylguanidine (350 µL, 2.84 mmol) was added dropwise. A solution of the crude hydrazone from the previous reaction in THF (200 µL) was added dropwise, quantitating the transfer with THF (3 x 200 µL). Upon complete transfer of the mixture of crude hydrazones, the reaction turned clear yellow, and the ice bath was removed. After 40 min, the reaction was cooled to 0 °C and quenched with a mixture of saturated Na_2SO_3 (3 mL) and saturated $NaCO_3$ (1 mL). The mixture was extracted with Et_2O (3x) and the combined organics were dried over Na_2SO_4 and concentrated under reduced pressure giving a crude residue (20 mg). Column chromatography (SiO_2, PhMe loading, 0-10% EtOAc/hexanes) afforded vinyl iodide (m/z = +458). This material was used without further purification for the next reaction.

Pre-complexation for CH arylation In a 1 dram vial, $Pd(OAc)_2$ (4 mg, 0.017 mmol) and NiXantphos (14 mg, 0.025 mmol) were placed under Ar (3x evacuate/backfill cycles) and taken up in anhydrous degassed THF (1 mL). The resulting dark greenish-black mixture containing a clump of black solid was sonicated to give a fine red-brown "homogeneous suspension", solution A.

CH arylation Separately, the crude vinyl iodide from above was charged to an 8 mL culture tube and placed under Ar (3x evacuate/backfill cycles). Oxazole (26 µL, 0.4 mmol) and NaOtBu (1.0 M in THF solution, 800 µL) were simultaneously injected rapidly into bottom of the reaction tube at 22 °C. The resulting solution was then charged with solution A (400 µL, 0.0068 mmol Pd). The resulting faintly translucent dark orange solution was stirred at 22 °C under Ar for 15 h to give a translucent dark brown-orange solution, which was cooled to 0 °C, quenched with a mixture of saturated aqueous $NaHCO_3$ (1.5 mL)/saturated aqueous Na_2CO_3 (1.5 mL)/H_2O (0.5 mL) and extracted with EtOAc (4x). The combined organics were dried over Na_2SO_4 and concentrated to dryness to afford a residue, which was purified by 2 rounds of preparative TLC (round 1: SiO_2, 5% (7N NH_3/MeOH)/(20% EtOAc/hexanes); round 2: SiO_2, 5.5% (7N NH_3/MeOH)/(20% EtOAc/hexanes)) to give oxazole **28** (3.0 mg, 19% over 3 steps). **28** from this batch was advanced to biological experiments (see page S134).

Procedure B:

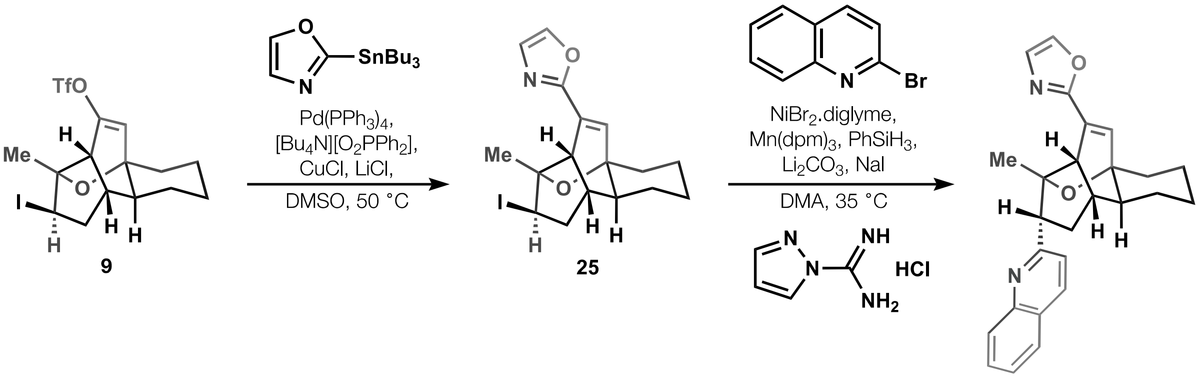

28

Stille coupling see page S42

Cross-electrophile coupling A solution of *rac*-oxazole iodide **25** (10.0 mg, 25.2 µmol, 100 µL, 0.25 M in PhH, 1 equiv.) was charged to a flame-dried culture tube (Fisherbrand 14-959-25A, 13 × 100 mm, 8 mL volume) and dried under high vacuum. An oven-dried stir bar, $NiBr_2$·diglyme (30.0 mg, 75.5 µmol, 3 equiv.), $Mn(dpm)_3$ (30.0 mg, 50.3 µmol, 2 equiv), oven-dried Li_2CO_3 (2.8 mg, 37.7 µmol, 1.5 equiv.), oven-dried NaI (34 mg, 226.6 µmol, 9 equiv.), and 2-bromo-quinoline (13.0 mg, 63.0 µmol, 2.5 equiv) were added. The tube was placed under Ar (3 × evacuate/backfill cycles). 800 µL DMA (0.033 M of **25**) was added and the turbid blue mixture was sparged with Ar at 1500 rpm for 30 mins. A colloidal yellow-brown mixture was obtained. The septum was changed quickly under Ar, then $PhSiH_3$ (20 µL, 151.0 µmol, 6 equiv.) was added to the mixture. The Ar balloon was taken out and the septum was sealed with telfon tape, electrical tape, and parafilm (see note 1). The mixture was heated in a 35 °C water bath. After 16 hrs, TLC analysis indicated significant consumption of starting material. The mixture was then diluted with EtOAc (2 mL), and pre-chilled H_2O (2 mL) was added. The biphasic mixture was extracted with EtOAc (3 × 5 mL) (see note 2). The organic phase was washed with half-saturated brine, then back extract with EtOAc (2 × 5 mL). The organic phase was dried over Na_2SO_4, and concentrated under reduced pressure. The DMA-containing residue was loaded to two 20 × 20 cm TLC plates, and then elute with 100% CH_2Cl_2 to give semipurified diasteromeric mixture of *rac*-**28** (15% yield qNMR , 1:5 exo/endo).

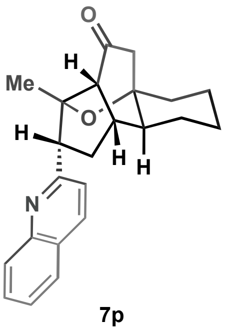

7p

Characterization data for ketone **7p** (and **8p)**
See page S123

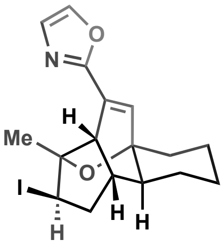

25

Characterization data for oxazole iodide **25**

See page S45

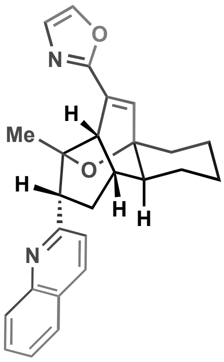

RS1160 (28)

Characterization data for oxazole **RS1160** **(28)**

Description colorless film

TLC R_f = 0.45 (SiO_2, 5% (7N NH_3/MeOH)/(20% EtOAc/hexanes)); UV-active, stains blue in CAM

^{1}H NMR (600 MHz, $CDCl_3$) δ 8.06 (dd, J = 8.4, 3.8 Hz, 2H), 7.78 (dd, J = 8.2, 1.4 Hz, 1H), 7.69 – 7.63 (m, 2H), 7.53 (d, J = 8.5 Hz, 1H), 7.47 (ddd, J = 8.1, 6.9, 1.2 Hz, 1H), 7.20 – 7.17 (m, 1H), 7.04 (bs, 1H), 3.67 (dd, J = 11.7, 4.7 Hz, 1H), 3.49 (dd, J = 4.2, 1.7 Hz, 1H), 2.55 (ddd, J = 13.3, 11.7, 5.9 Hz, 1H), 2.36 (dd, J = 13.3, 4.7 Hz, 1H), 2.24 – 2.15 (m, 1H), 1.93 (dt, J = 12.3, 3.4 Hz, 1H), 1.88 – 1.78 (m, 2H), 1.69 (dt, J = 13.1, 3.3 Hz, 1H), 1.63 – 1.59 (m, 1H), 1.57 – 1.49 (m, 1H), 1.41 – 1.30 (m, 2H), 1.13 (s, 3H), 0.92 (qd, J = 13.1, 3.6 Hz, 1H).

^{13}C NMR (151 MHz, $CDCl_3$) δ 161.7, 161.0, 147.9, 138.6, 136.8, 135.4, 129.3, 129.1, 128.6, 128.3, 127.5, 127.2, 125.7, 122.3, 85.0, 73.6, 56.0, 52.9, 49.4, 40.5, 37.3, 33.5, 32.4, 25.9, 23.9, 23.6.

2D NMR HSQC, HMBC, COSY, NOESY

HRMS Calculated $C_{26}H_{27}N_2O_2$ $[M+H]^+$ 399.2073 | Found: 399.2076

Synthesis of RS1166 (29)

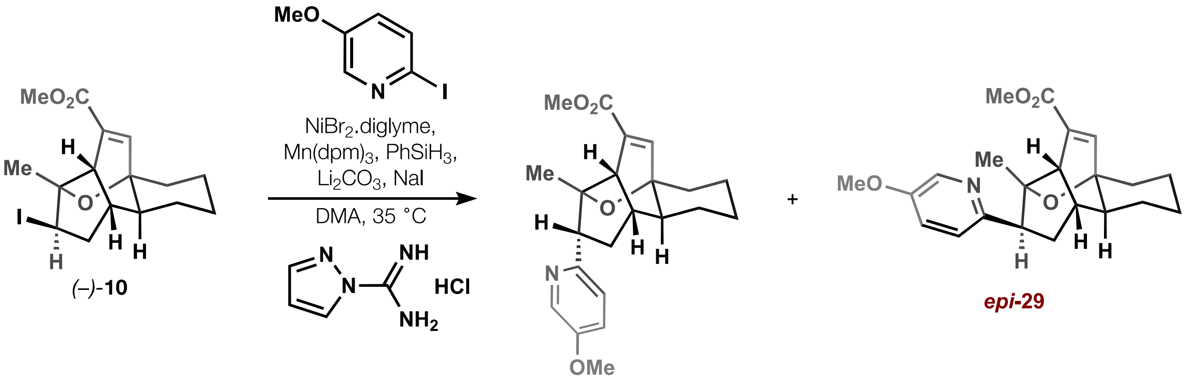

RS1166 (29)

(See page S21)
A flame-dried 20 mL glass tube with threaded end was equipped with a stir bar and septum pierced with an needle fed by a balloon filled with argon. It was sequentially charged with praxadine.HCl (15.1 mg, 103.0 µmol, 2 equiv.), $NiBr_2$•diglyme (40.0 mg, 113.3 µmol, 2.2 equiv.), $Mn(dpm)_3$ (44.0 mg, 72.1 µmol, 1.4 equiv.), 2-iodo-5-methoxypyridine (36.0 mg, 154.5 µmol, 3 equiv.), *(−)*-**10** (20.0 mg, 51.5 µmol, 1 equiv.), Li_2CO_3 (3.8 mg, 51.5 µmol, 1 equiv.), and NaI (46.3 mg, 309.1 µmol, 6 equiv.). The vial was evacuated and backfilled with argon (5x) and then charged with DMA (dry, 1.8 mL) using a syringe, and the reaction mixture was degassed with an argon balloon and a vent needle while stirring for 30 min. The reaction mixture turned brown at the end of the degassing period. The vent needle was removed, the septum and argon balloon replaced. Silylbenzene (22.3 mg, 25.5 µL, 206.1 µmol, 4 equiv.) was added using a syringe, then the reaction mixture was placed into a preheated 35 °C water bath. The reaction underwent a series of changes in appearance similar to XEC with 2-iodo-6-methyl pyridine from turbid brown-yellow to turbid yellow-olive to turbid pastel green/light jade to turbid dark jade within a timespan of 15 – 30 min. The reaction was stirred for 6 h. Upon reaction completion (No SM mass found on LC-MS, faint SM spot remaining, dark forest green reaction mixture) EtOAc (2 mL) and ice-cold water (4 mL) were added. The layers were separated and the aqueous layer was extracted with EtOAc (6 x 0.5 mL). The combined organic layers were washed with half saturated brine (2 x 2 mL) and brine (0.5 mL). The combined washes were back extracted with EtOAc (0.5 mL) and the back extract was washed with half saturated brine (2 x 0.5 mL), then combined with the organic layer, dried over $MgSO_4$, filtered and concentrated *in vacuo*. The crude product was purified by column chromatography (4 g SiO_2, load in toluene, elute 0-5-10-15-20-25-30-35-40-100% EtOAc in hexanes, Combiflash purification system). The product containing fractions were concentrated *in vacuo* and the product was further purified by column chromatography (SiO_2, load in toluene, elute 20% acetone in hexanes). The resulting product was purified by preparative TLC (SiO_2, 10 x 10 cm, 0.5 mm thick, load in $CHCl_3$, elute 30% EtOAc in hexanes) affording 9.5 mg (50% yield) of **29** as a colorless oil and single diastereomer.

Characterization data of methyl enoate **RS1166** **(29)**
Description colorless oil
TLC R_f = 0.27 (SiO_2, 30% EtOAc in hexanes); UV-active, stains green in anisaldehyde
^{1}H NMR (600 MHz, $CDCl_3$) δ 8.24 (d, *J* = 3.0 Hz, 1H), 7.26 (d, *J* = 8.7 Hz, 1H), 7.23 (d, *J* = 1.7 Hz, 1H), 7.15 (dd, *J* = 8.7, 3.0 Hz, 1H), 3.83 (s, 3H), 3.81 (s, 3H), 3.39 (dd, *J* = 11.9, 4.8 Hz, 1H), 3.20 (dd, *J* = 4.2, 1.8 Hz, 1H), 2.47 (ddd, *J* = 13.2, 11.9, 6.0 Hz, 1H), 2.05 (dd, *J* = 13.2, 4.8 Hz, 1H), 1.94 (dt, *J* = 12.3, 3.4 Hz, 1H), 1.88 (ddd, *J* = 13.4, 4.0, 2.1 Hz, 1H), 1.83

(dq, *J* = 14.3, 2.6 Hz, 1H), 1.67 – 1.60 (m, 2H), 1.55 – 1.44 (m, 2H), 1.36 (ddd, *J* = 13.5, 12.2, 4.2 Hz, 1H), 1.28 (dddd, *J* = 17.3, 13.3, 8.6, 4.0 Hz, 1H), 1.00 (s, 3H), 0.78 (qd, *J* = 13.1, 3.7 Hz, 1H).

^{13}C NMR (151 MHz, $CDCl_3$) δ 165.9, 154.2, 153.3, 145.5, 135.9, 132.6, 123.8, 120.9, 84.8, 73.5, 55.7, 54.2, 53.0, 52.0, 48.3, 40.2, 37.7, 33.2, 32.2, 25.7, 23.6, 23.5.

2D NMR COSY, HSQC, HMBC, NOESY

LC-MS m/z = 370.2 $[M+H]^+$

HRMS Calculated $C_{22}H_{28}NO_4$ $[M+H]^+$ = 370.2018 | Found: 370.2014

Synthesis of RS1163 (30)

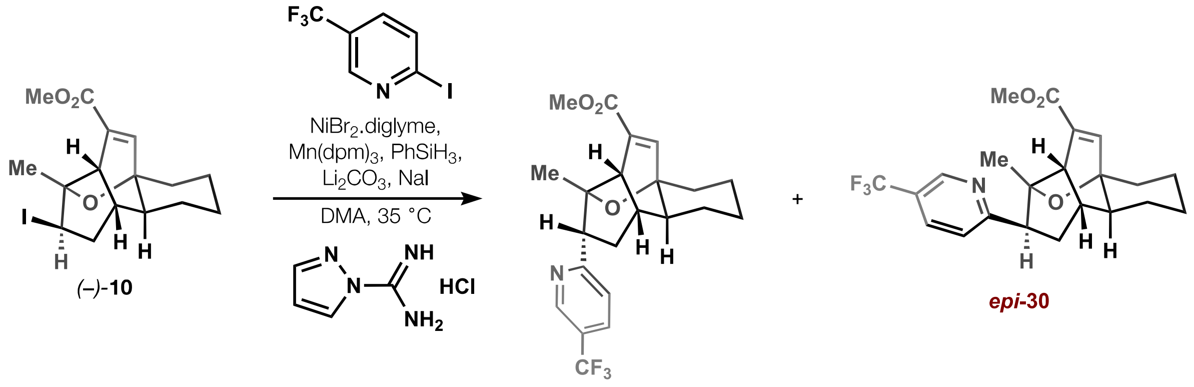

(See page S21)
A flame-dried 20 mL glass tube with threaded end was equipped with a stir bar and septum pierced with an needle fed by a balloon filled with argon. It was sequentially charged with praxadine.HCl (15.1 mg, 103.0 µmol, 2 equiv.), $NiBr_2$•diglyme (40.0 mg, 113.3 µmol, 2.2 equiv.), $Mn(dpm)_3$ (44.0 mg, 72.1 µmol, 1.4 equiv.), 2-iodo-5-(trifluoromethyl)-pyridine (42.2 mg, 154.5 µmol, 3 equiv.), *(−)*-**10** (20.0 mg, 51.5 µmol, 1 equiv.), Li_2CO_3 (4.0 mg, 51.5 µmol, 1 equiv.), and NaI (46.0 mg, 309.1 µmol, 6 equiv.). The vial was evacuated and backfilled with argon (5x) and then charged with DMA (dry, 1.8 mL) using a syringe, and the reaction mixture was degassed with an argon balloon and a vent needle while stirring for 30 min. The reaction mixture turned brown at the end of the degassing period. The vent needle was removed, the septum and argon balloon replaced. Silylbenzene (22.3 mg, 25.5 µL, 206.1 µmol, 4 equiv.) was added using a syringe, then the reaction mixture was placed into a preheated 35 °C water bath. The reaction underwent a series of changes in appearance similar to XEC with 2-iodo-6-methyl pyridine from turbid brown-yellow to turbid yellow-olive to turbid pastel green/light jade to turbid dark jade within a timespan of 15 – 30 min. The reaction was stirred for 6 h. Upon reaction completion (No SM mass found on LC-MS, faint SM spot remaining, dark forest green reaction mixture) EtOAc (2 mL) and ice-cold water (4 mL) were added. The layers were separated and the aqueous layer was extracted with EtOAc (6 x 0.5 mL). The combined organic layers were washed with half saturated brine (2 x 2 mL) and brine (0.5 mL). The combined washes were back extracted with EtOAc (0.5 mL) and the back extract was washed with half saturated brine (2 x 0.5 mL), then combined with the organic layer, dried over $MgSO_4$, filtered and concentrated *in vacuo*. The crude product was purified by column chromatography (4 g SiO_2, load in toluene, elute 0-5-10-15-20-25-30-35-40-100% acetone in hexanes, Combiflash purification system). The product containing fractions were concentrated *in vacuo* and further purified by column chromatography (SiO_2, load in toluene, elute 10% acetone in hexanes). The resulting product was purified by three rounds of preparative TLC (SiO_2, 10 x 10 cm, 0.25 mm thick, load in $CHCl_3$, elute 5% EtOAc in DCM, run 5 consecutive times), (SiO_2, 10 x 10 cm, 0.5 mm thick, load in $CHCl_3$, elute 10% acetone in hexanes) and (SiO_2, 10 x 10 cm, 0.5 mm thick, load in $CHCl_3$, elute 1.5% EtOAc in hexanes, run 2 consecutive times) affording 4.8 mg (23% yield) of **30** as a colorless oil and single diastereomer.

Characterization data for methyl enoate **RS1163** **(30)**
Description colorless oil
TLC R_f = 0.63 (SiO_2, 30% EtOAc in hexanes); UV-active, stains brown in anisaldehyde
^{1}H NMR (600 MHz, $CDCl_3$) δ 8.83 – 8.80 (m, 1H), 7.82 (dd, *J* = 8.2, 2.4 Hz, 1H), 7.43 (d, *J*

= 8.2 Hz, 1H), 7.24 (d, J = 1.7 Hz, 1H), 3.82 (s, 3H), 3.50 (dd, J = 11.6, 4.5 Hz, 1H), 3.25 (dd, J = 4.1, 1.7 Hz, 1H), 2.44 (ddd, J = 13.2, 11.7, 5.9 Hz, 1H), 2.20 (dd, J = 13.2, 4.5 Hz, 1H), 1.99 – 1.95 (m, 1H), 1.91 (dd, J = 12.3, 3.4 Hz, 1H), 1.87 – 1.79 (m, 1H), 1.69 (ddd, J = 6.1, 4.2, 2.0 Hz, 1H), 1.65 (dt, J = 13.2, 3.3 Hz, 1H), 1.55 – 1.45 (m, 2H), 1.34 – 1.27 (m, 2H), 1.04 (s, 3H), 0.79 (qd, J = 13.1, 3.6 Hz, 1H).
^{13}C NMR (151 MHz, $CDCl_3$) δ 165.8, 165.2 (q, ^{CF}J = 1.1 Hz) 145.7, 145.6 (q, ^{CF}J = 4.0 Hz), 132.7 (q, ^{CF}J = 3.5 Hz), 132.4, 124.3 (q, ^{CF}J = 32.9 Hz), 124.1 (q, ^{CF}J = 272.0 Hz), 123.5, 85.0, 73.8, 55.2, 52.3, 52.1, 48.4, 40.3, 36.8, 33.1, 32.2, 25.6, 23.8, 23.4.
^{19}F NMR (376 MHz, $CDCl_3$) δ -64.85.
2D NMR COSY, HSQC, HMBC, NOESY
LC-MS m/z = 408.2 $[M+H]^+$
HRMS Calculated $C_{22}H_{25}F_3NO_3$ $[M+H]^+$ = 408.1787 | Found: 408.1786

Synthesis of RS1165 (31)

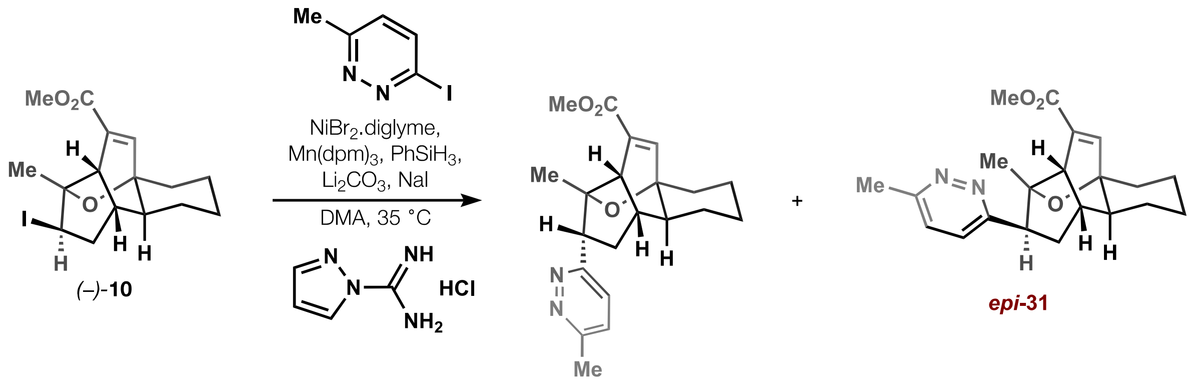

RS1165 (31)

(See page S21)

A flame-dried 20 mL glass tube with threaded end was equipped with a stir bar and septum pierced with an needle fed by a balloon filled with argon. It was sequentially charged with praxadine.HCl (15.1 mg, 103.0 µmol, 2 equiv.), $NiBr_2$•diglyme (40.0 mg, 113.3 µmol, 2.2 equiv.), $Mn(dpm)_3$ (44.0 mg, 72.1 µmol, 1.4 equiv.), 3-iodo-6-methylpyridazine (34.0 mg, 154.5 µmol, 3 equiv.), *(–)*-**10** (20.0 mg, 51.5 µmol, 1 equiv.), Li_2CO_3 (4.0 mg, 51.5 µmol, 1 equiv.), and NaI (46.0 mg, 309.1 µmol, 6 equiv.). The vial was evacuated and backfilled with argon (5x) and then charged with DMA (dry, 1.8 mL) using a syringe, and the reaction mixture was degassed with an argon balloon and a vent needle while stirring for 30 min. The reaction mixture turned brown at the end of the degassing period. The vent needle was removed, the septum and argon balloon replaced. Silylbenzene (22.3 mg, 25.5 µL, 206.1 µmol, 4 equiv.) was added using a syringe, then the reaction mixture was placed into a preheated 35 °C water bath. The reaction underwent a series of changes in appearance similar to XEC with 2-iodo-6-methyl pyridine from turbid brown-yellow to turbid yellow-olive to turbid pastel green/light jade to turbid dark jade within a timespan of 15 – 30 min. The reaction was stirred for 6 h. Upon reaction completion (No SM mass found on LC-MS, faint SM spot remaining, dark forest green reaction mixture) EtOAc (2 mL) and ice-cold water (4 mL) were added. The layers were separated and the aqueous layer was extracted with EtOAc (6 x 0.5 mL). The combined organic layers were washed with half saturated brine (2 x 2 mL) and brine (0.5 mL). The combined washes were back extracted with EtOAc (0.5 mL) and the back extract was washed with half saturated brine (2 x 0.5 mL), then combined with the organic layer, dried over $MgSO_4$, filtered and concentrated *in vacuo*. The crude product was purified by column chromatography (4 g SiO_2, load in toluene, elute 0-5-10-15-20-25-30-35-40-100% acetone in hexanes, Combiflash purification system). The product containing fractions were concentrated *in vacuo* and the product was further purified by column chromatography (SiO_2, load in toluene, elute 30% acetone in hexanes). The resulting product was purified by two rounds of preparative TLC (SiO_2, 10 x 10 cm, 0.5 mm thick, load in $CHCl_3$, elute 2.5% MeOH in DCM) and (SiO_2, 10 x 10 cm, 0.25 mm thick, load in $CHCl_3$, elute 2.5% MeOH in DCM, run two consecutive times) affording 1.5 mg (8% yield) of **31** as a colorless oil and single diastereomer.

Characterization data for methyl enoate **RS1165** (31)

Description colorless oil

TLC R_f = 0.06 (SiO_2, 30% EtOAc in hexanes); UV-active, stains green in anisaldehyde

^{1}H NMR (600 MHz, $CDCl_3$) δ 7.40 (d, *J* = 8.6 Hz, 1H), 7.24 (d, *J* = 1.7 Hz, 1H), 7.22 (d, *J* = 8.7 Hz, 1H), 3.82 (s, 3H), 3.62 (dd, *J* = 11.9, 4.8 Hz, 1H), 3.27 (dd, *J* = 4.2, 1.8 Hz, 1H), 2.67 (s, 3H), 2.51 (ddd, *J* = 13.4, 11.8, 6.0 Hz, 1H), 2.21 – 2.17 (m, 1H), 1.94 – 1.87 (m, 2H), 1.83

(d, *J* = 13.9 Hz, 1H), 1.73 – 1.68 (m, 1H), 1.66 – 1.62 (m, 1H), 1.55 – 1.44 (m, 2H), 1.39 – 1.35 (m, 1H), 1.35 – 1.29 (m, 1H), 1.01 (s, 3H), 0.79 (qd, *J* = 13.1, 3.7 Hz, 1H).
^{13}C NMR (151 MHz, $CDCl_3$) δ 165.8, 160.4, 158.1, 145.5, 132.5, 127.2, 126.4, 84.9, 73.8, 53.1, 53.0, 52.1, 48.3, 40.2, 37.2, 33.2, 32.2, 25.5, 23.6, 23.4, 22.1.
2D NMR COSY, HSQC, HMBC, NOESY
LC-MS m/z = 355.2 $[M+H]^+$
HRMS Calculated $C_{21}H_{27}N_2O_3$ $[M+H]^+$ = 355.2022 | Found: 355.2013

Synthesis of RS1161 (32)

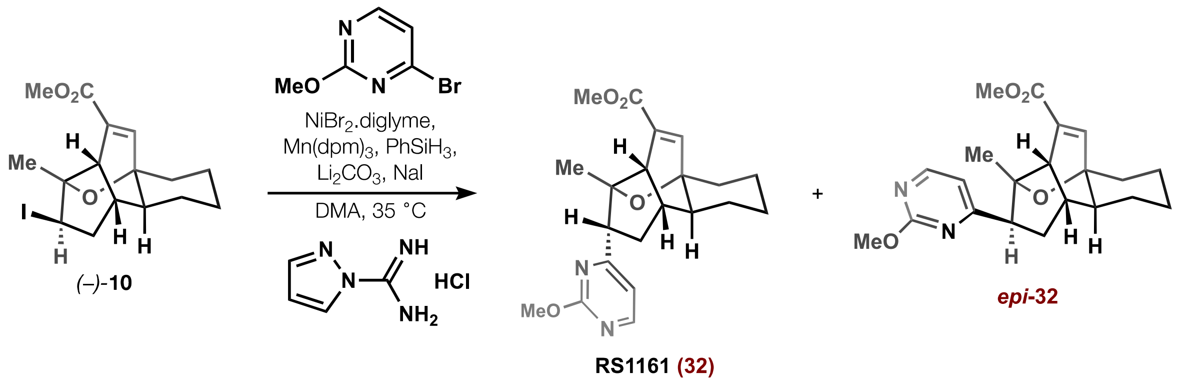

(See page S21)

A flame-dried 20 mL glass tube with threaded end was equipped with a stir bar and septum pierced with an needle fed by a balloon filled with argon was sequentially charged with praxadine.HCl (15.1 mg, 103.0 µmol, 2 equiv.), $NiBr_2$•diglyme (40.0 mg, 113.3 µmol, 2.2 equiv.), $Mn(dpm)_3$ (44.0 mg, 72.1 µmol, 1.4 equiv.), 4-bromo-2-methoxy-pyrimidine (49.0 mg, 257.6 µmol, 5 equiv.), *(−)*-**10** (20.0 mg, 51.5 µmol, 1 equiv.), Li_2CO_3 (4.0 mg, 51.5 µmol, 1 equiv.), and NaI (46.0 mg, 309.1 µmol, 6 equiv.). The vial was evacuated and backfilled with argon (5x) and then charged with DMA (dry, 1.8 mL) using a syringe, and the reaction mixture was degassed with an argon balloon and a vent needle while stirring for 30 min. The reaction mixture turned brown at the end of the degassing period. The vent needle was removed, the septum and argon balloon replaced. Silylbenzene (22.3 mg, 25.5 µL, 206.1 µmol, 4 equiv.) was added using a syringe, then the reaction mixture was placed into a preheated 35 °C water bath. The reaction underwent a series of changes in appearance similar to XEC with 2-iodo-6-methyl pyridine from turbid brown-yellow to turbid yellow-olive to turbid pastel green/light jade to turbid dark jade within a timespan of 15 – 30 min. The reaction was stirred for 5.5 h. Upon reaction completion (No SM mass found on LC-MS, faint SM spot remaining, dark forest green reaction mixture) EtOAc (2 mL) and ice-cold water (4 mL) were added. The layers were separated and the aqueous layer was extracted with EtOAc (6 x 0.5 mL). The combined organic layers were washed with half saturated brine (2 x 2 mL) and brine (0.5 mL). The combined washes were back extracted with EtOAc (0.5 mL) and the back extract was washed with half saturated brine (2 x 0.5 mL), then combined with the organic layer, dried over $MgSO_4$, filtered and concentrated *in vacuo*. The crude product was purified by column chromatography (4 g SiO_2, load in toluene, elute 0-5-10-15-20-25-30-35-40-100% EtOAc in hexanes, Combiflash purification system). The product containing fractions were concentrated *in vacuo* and the product was further purified by column chromatography (SiO_2, load in toluene, elute 30% acetone in hexanes). The diastereomers were separated by preparative TLC (SiO_2, 10 x 10 cm, 0.25 mm thick, load in $CHCl_3$, elute 30% EtOAc in hexanes, run 5 consecutive times) affording 0.8 mg (4% yield) of ***epi*-32** as a white solid and 2.9 mg (15% yield) of **32** as a colorless oil.

Characterization data for methyl enoate ***epi*-32**

Description White solid

TLC R_f = 0.24 (SiO_2, 30% EtOAc in hexanes); UV-active, stains brown in anisaldehyde

^{1}H NMR (600 MHz, $CDCl_3$) δ 8.38 (d, *J* = 4.9 Hz, 1H), 7.26 (s, 1H), 6.84 (d, *J* = 4.9 Hz, 1H), 4.03 (s, 3H), 3.79 (s, 3H), 3.48 (dd, *J* = 4.0, 1.8 Hz, 1H), 3.36 – 3.30 (m, 1H), 2.48 (ddd, *J* = 12.7, 7.5, 5.3 Hz, 1H), 2.06 – 1.98 (m, 2H), 1.86 (d, *J* = 13.7 Hz, 1H), 1.74 – 1.71 (m, 1H), 1.65 (d, *J* = 13.4 Hz, 1H), 1.55 – 1.38 (m, 4H), 1.22 (tt, *J* = 13.1, 4.3 Hz, 1H), 0.79 (qd, *J* = 13.2, 3.7 Hz, 1H), 0.62 (s, 3H).

^{13}C NMR (151 MHz, $CDCl_3$) δ 174.4, 165.8, 165.8, 159.1, 145.3, 132.7, 115.9, 85.8, 74.2,

57.0, 54.9, 52.3, 52.0, 46.1, 40.7, 37.9, 33.7, 32.3, 25.7, 23.4, 22.1.
2D NMR COSY, HSQC, HMBC, NOESY
LC-MS m/z = 371.2 $[M+H]^+$

Characterization data for methyl enoate **RS1161** (32)
Description colorless oil
TLC R_f = 0.17 (SiO_2, 30% EtOAc in hexanes); UV-active, stains brown in anisaldehyde
^{1}H NMR (600 MHz, $CDCl_3$) δ 8.37 (d, *J* = 5.0 Hz, 1H), 7.25 – 7.22 (m, 1H), 6.92 (d, *J* = 5.1 Hz, 1H), 4.02 (s, 3H), 3.82 (s, 3H), 3.31 (dd, *J* = 11.2, 4.6 Hz, 1H), 3.21 (dd, *J* = 4.1, 1.7 Hz, 1H), 2.37 – 2.23 (m, 2H), 1.98 (ddd, *J* = 13.5, 3.9, 2.2 Hz, 1H), 1.91 (dd, *J* = 12.4, 3.5 Hz, 1H), 1.86 – 1.79 (m, 1H), 1.69 – 1.61 (m, 2H), 1.55 – 1-43 (m, 2H), 1.34 – 1.17 (m, 2H), 1.08 (s, 3H), 0.77 (qd, *J* = 13.2, 3.7 Hz, 1H).
^{13}C NMR (151 MHz, $CDCl_3$) δ 172.3, 165.8, 165.3, 158.2, 145.8, 132.3, 115.1, 85.0, 73.7, 54.8, 54.6, 52.1, 51.7, 48.4, 40.5, 35.5, 33.2, 32.2, 25.8, 23.9, 23.4.
2D NMR COSY, HSQC, HMBC, NOESY
LC-MS m/z = 371.2 $[M+H]^+$
HRMS Calculated $C_{21}H_{27}N_2O_4$ $[M+H]^+$ = 371.1971 | Found: 371.1960

Synthesis of RS1162 (33)

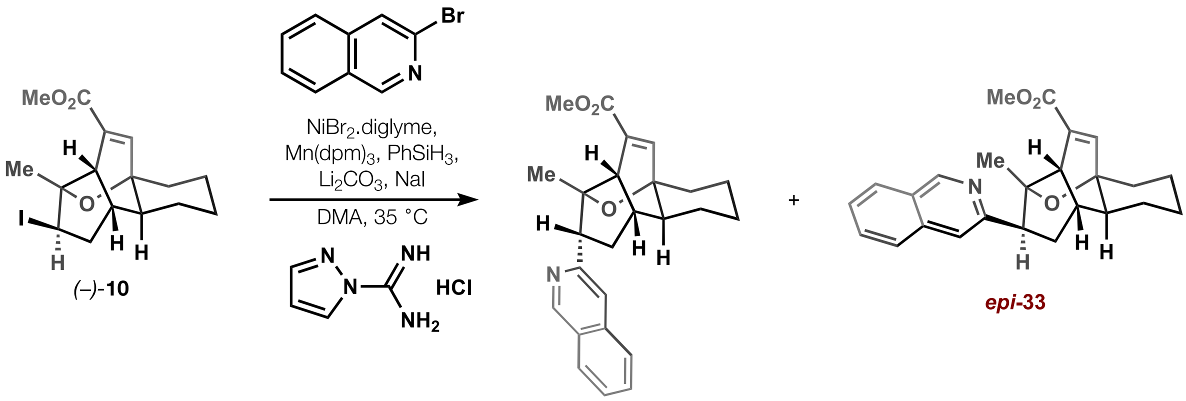

RS1162 (33)

(See page S21)

A flame-dried 20 mL glass tube with threaded end was equipped with a stir bar and septum pierced with an needle fed by a balloon filled with argon was sequentially charged with praxadine.HCl (15.1 mg, 103.0 µmol, 2 equiv.), $NiBr_2$•diglyme (40.0 mg, 113.3 µmol, 2.2 equiv.), $Mn(dpm)_3$ (43.6 mg, 72.1 µmol, 1.4 equiv.), 3-bromosioquinoline (53.6 mg, 257.6 µmol, 5 equiv.), *(−)*-**10** (20.0 mg, 51.5 µmol, 1 equiv.), Li_2CO_3 (4.0 mg, 51.5 µmol, 1 equiv.), and NaI (46.0 mg, 309.1 µmol, 6 equiv.). The vial was evacuated and backfilled with argon (5x) and then charged with DMA (dry, 1.8 mL) using a syringe, and the reaction mixture was degassed with an argon balloon and a vent needle while stirring for 30 min. The reaction mixture turned brown at the end of the degassing period. The vent needle was removed, the septum and argon balloon replaced. Silylbenzene (22.3 mg, 25.5 µL, 206.1 µmol, 4 equiv.) was added using a syringe, then the reaction mixture was placed into a preheated 35 °C water bath. The reaction underwent a series of changes in appearance similar to XEC with 2-iodo-6-methyl pyridine from turbid brown-yellow to turbid yellow-olive to turbid pastel green/light jade to turbid dark jade within a timespan of 15 – 30 min. The reaction was stirred for 2.5 h. Upon reaction completion (No SM mass found on LC-MS, faint SM spot remaining, dark forest green reaction mixture) EtOAc (2 mL) and ice-cold water (4 mL) were added. The layers were separated and the aqueous layer was extracted with EtOAc (6 x 0.5 mL). The combined organic layers were washed with half saturated brine (2 x 2 mL) and brine (0.5 mL). The combined washes were back extracted with EtOAc (0.5 mL) and the back extract was washed with half saturated brine (2 x 0.5 mL), then combined with the organic layer, dried over $MgSO_4$, filtered and concentrated *in vacuo*. The crude product was purified by column chromatography (4 g SiO_2, load in toluene, elute 10-15-20-25-30-35-40% EtOAc in hexanes, Combiflash purification system). The product containing fractions were concentrated *in vacuo* and the product was further purified by column chromatography (SiO_2, load in toluene, elute 15% EtOAc in hexanes). The resulting product was purified by preparative TLC (SiO_2, 10 x 10 cm, 0.25 mm thick, load in $CHCl_3$, elute 20% EtOAc in hexanes) affording 10.3 mg (51% yield) of **33** as a white solid and single diastereomer.

Characterization data for methyl enoate **RS1162** **(33)**

Description White solid

TLC R_f = 0.42 (SiO_2, 30% EtOAc in hexanes); UV-active, stains blue in anisaldehyde

^{1}H NMR (600 MHz, $CDCl_3$) δ 9.21 (s, 1H), 7.92 (d, *J* = 8.2 Hz, 1H), 7.77 (d, *J* = 8.3 Hz, 1H), 7.66 (s, 1H), 7.64 – 7.61 (m, 1H), 7.51 (t, *J* = 7.5 Hz, 1H), 7.25 (d, *J* = 1.9 Hz, 1H), 3.82 (s, 3H), 3.59 (dd, *J* = 11.8, 4.6 Hz, 1H), 3.27 (dd, *J* = 4.2, 1.8 Hz, 1H), 2.54 (td, *J* = 12.6, 5.9 Hz, 1H), 2.26 (dd, *J* = 13.2, 4.7 Hz, 1H), 2.13 – 2.07 (m, 1H), 1.91 (dt, *J* = 12.3, 3.3 Hz, 1H),

1.87 – 1.80 (m, 1H), 1.73 – 1.69 (m, 1H), 1.66 (dt, *J* = 13.4, 3.3 Hz, 1H), 1.59 – 1.54 (m, 1H), 1.50 (ddd, *J* = 17.5, 8.8, 3.8 Hz, 1H), 1.41 – 1.29 (m, 2H), 1.08 (s, 3H), 0.81 (qd, *J* = 13.1, 3.6 Hz, 1H).
^{13}C NMR (151 MHz, $CDCl_3$) δ 166.0, 154.6, 151.3, 145.8, 136.4, 132.6, 130.0, 127.5, 127.4, 126.6, 126.5, 119.6, 85.0, 73.6, 54.6, 52.4, 52.0, 48.5, 40.4, 37.5, 33.2, 32.3, 25.7, 23.8, 23.5.
2D NMR COSY, HSQC, HMBC, NOESY
LC-MS m/z = 390.2 $[M+H]^+$
HRMS Calculated $C_{25}H_{28}NO_3$ $[M+H]^+$ = 390.2069 | Found: 390.2064

Synthesis of RS1188

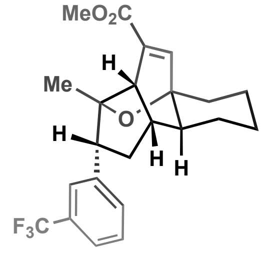

RS1188

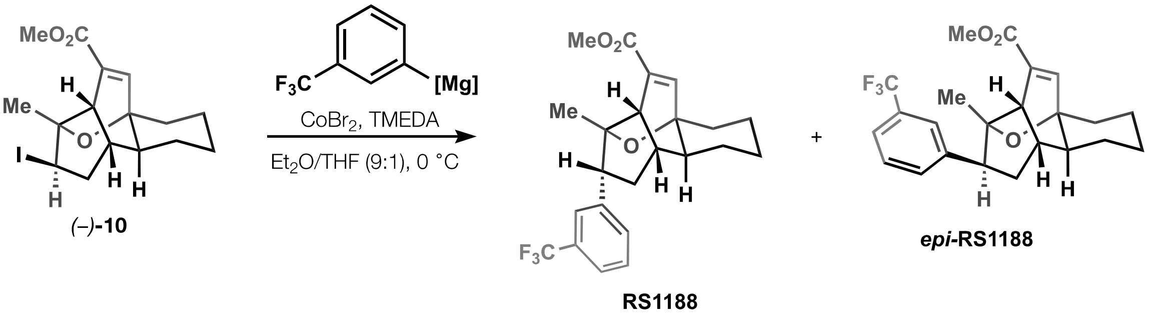

Aryl-Grignard preparation. A flame-dried 1.5 mL vial equipped with a stir bar and septum pierced with a needle fed by an argon balloon was charged with THF (dry, 100 µL), 3-iodo-trifluoromethyltoluene (210 mg, 111.4µL, 772 µmol, 15 equiv.). Turbo-Grignard (123 mg, 654 µL (1.3M solution in THF), 850 µmol, 16.5 equiv.) was added dropwise to the stirred reaction mixture at 22 °C using a syringe. The reaction mixture was stirred for 30 min before all of it was taken up in a syringe and used in the subsequent step.

Kumada coupling. An 8 mL flame-dried glass tube with threaded end was equipped with a stir bar and septum pierced with a needed fed by an argon balloon. It was charged with $CoBr_2$ (28.0 mg, 129 µmol, 2.5 equiv.) and *(–)*-**10** (20.0 mg, 51.5 µmol, 1 equiv.). The vial was evacuated and backfilled with argon (3x) and then charged with diethyl ether (dry, 1.8 mL), THF (dry, 0.2 mL) and TMEDA (15.0 mg, 19.3 µL, 129 µmol, 2.5 equiv.). An ice bath was equipped and the reaction was cooled to 0°C. The prepared aryl-Grignard was added dropwise to the reaction mixture. Upon complete starting material consumption, $NaHCO_3$ (saturated aqueous, ice-cold, 5 mL) was added and the aqueous layer was extracted with diethyl ether (2 x 1.5 mL). The combined organic layers were washed with brine (2 mL), dried over $MgSO_4$, filtered and concentrated *in vacuo*. The crude product was purified by column chromatography (4g SiO_2, load in toluene, elute 0-5-10-15-20-25-30-35-40-100% EtOAc in hexanes). The product containing fractions were concentrated *in vacuo* and then further purified by preparative TLC (SiO_2, 10 x 10 cm, 0.5 mm thick, load in $CHCl_3$, elute 5% EtOAc in hexanes). The product was further purified on a Waters Prep SFC 150 AP using a Chiralpak IG column (5 um, 21x250 mm). The purification was run under isocratic conditions (5% MeOH / CO_2, 100 mL/min, 1600 psi backpressure) at 35 °C. Fractionation was triggered by mass spec (ESI+ on a Waters QDa, SIR channel m/z = 389.2). Two products were obtained, fast-moving isomer ***epi*-RS1188** (2.6 mg, 12%) and slow-moving isomer **RS1188** (2.6 mg, 12%).

Characterization data for ***epi*-RS1188**

Description colorless film

TLC R_f = 0.33 (15% EtOAc in hexanes); UV-active, stains dark blue in anisaldehyde

^{1}H NMR (600 MHz, C_6D_6) δ 7.46 (s, 1H), 7.33 (d, *J* = 1.6 Hz, 1H), 7.22 (d, *J* = 7.7 Hz, 1H), 6.96 (d, *J* = 7.8 Hz, 1H), 6.85 (t, *J* = 7.7 Hz, 1H), 3.47 (s, 3H), 3.35 (dd, *J* = 4.0, 1.6 Hz, 1H), 3.26 (t, *J* = 8.7 Hz, 1H), 1.99 (dt, *J* = 12.5, 3.5 Hz, 1H), 1.83 (dd, *J* = 13.0, 9.0 Hz, 1H), 1.70 (ddd, *J* = 13.2, 8.5, 5.0 Hz, 1H), 1.59 – 1.49 (m, 1H), 1.47 (dt, *J* = 9.1, 4.1 Hz, 2H), 1.44 – 1.40 (m, 1H), 1.21 (dddd, *J* = 27.7, 13.4, 9.8, 4.1 Hz, 3H), 1.00 (dddd, *J* = 17.1, 13.2, 8.3, 3.8 Hz, 1H), 0.66 (s, 4H).
^{13}C NMR (151 MHz, C_6D_6) δ 165.46, 145.77, 145.35, 132.84, 131.81, 130.78 (q, *J* = 32.00 Hz), 129.22, 126.27 (q, *J* = 3.75 Hz), 125.00 (d, *J* = 272.20 Hz), 123.38 (q, *J* = 3.80 Hz), 85.28, 74.00, 56.88, 51.63, 51.44, 47.03, 40.76, 39.98, 34.05, 32.41, 30.48, 25.94, 23.37, 22.95.
^{19}F NMR (376 MHz, $CDCl_3$) δ -62.02.
HRMS Calculated $C_{23}H_{26}F_3O_3$ $[M+H]^+$ = 407.1834 | Found: 407.1826

Characterization data for **RS1188**
Description colorless film
TLC R_f = 0.35 (15% EtOAc in hexanes); UV-active, stains greyish-purple in anisaldehyde
^{1}H NMR (600 MHz, C_6D_6) δ 7.72 (s, 1H), 7.32 (d, *J* = 7.8 Hz, 1H), 7.24 – 7.19 (m, 2H), 6.99 (t, *J* = 7.7 Hz, 1H), 3.49 (s, 3H), 3.25 (dd, *J* = 4.3, 1.8 Hz, 1H), 2.69 (dd, *J* = 11.9, 5.5 Hz, 1H), 2.26 – 2.17 (m, 1H), 1.92 – 1.86 (m, 1H), 1.50 – 1.44 (m, 4H), 1.44 – 1.39 (m, 1H), 1.37 – 1.32 (m, 1H), 1.24 – 1.08 (m, 2H), 0.97 (qt, *J* = 13.3, 3.9 Hz, 1H), 0.84 (s, 3H), 0.64 (qd, *J* = 13.0, 3.7 Hz, 1H).
^{13}C NMR (151 MHz, C_6D_6) δ 165.38, 145.08, 143.04, 133.61, 132.93, 130.21 (q, *J* = 31.16 Hz), 127.61, 126.95 (q, *J* = 3.85 Hz), 125.38 (d, *J* = 272.20 Hz), 123.23 (q, *J* = 3.85 Hz), 123.22, 84.64, 73.44, 55.64, 53.13, 51.40, 48.35, 40.59, 39.61, 33.73, 32.25, 25.87, 23.37, 22.97.
^{19}F NMR (376 MHz, $CDCl_3$) δ -61.98.
2D NMR HSQC, NOESY
HRMS Calculated $C_{23}H_{26}F_3O_3$ $[M+H]^+$ = 407.1834 | Found: 407.1827

Synthesis of RS1137

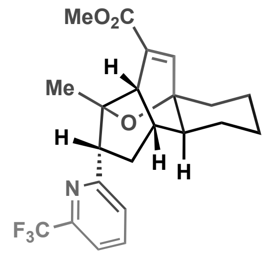

RS1137

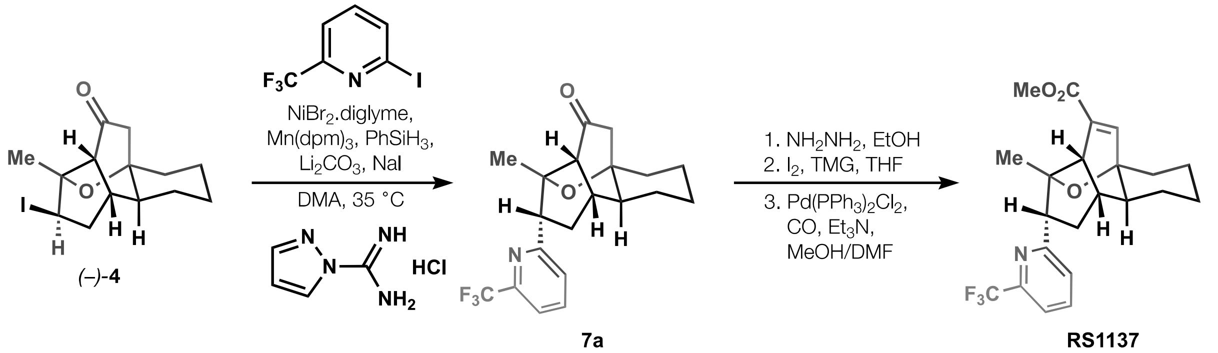

Cross-electrophile coupling (see page S21) A flame-dried 20 mL glass tube with threaded end was equipped with a stir bar and septum pierced with an needle fed by a balloon filled with argon. It was sequentially charged with praxadine.HCl (85.0 mg, 578 µmol, 2 equiv.), $NiBr_2$•diglyme (224 mg, 635 µmol, 2.2 equiv.), $Mn(dpm)_3$ (245 mg, 404 µmol, 1.4 equiv.), 2-iodo-6-(trifluoromethyl) pyridine (237 mg, 867 µmol, 3 equiv.) *(−)*-**4** (100 mg, 289 µmol, 1 equiv.), Li_2CO_3 (21.0 mg, 289 µmol, 1 equiv.), and NaI (260 mg, 1.73 mmol, 6 equiv.). The vial was evacuated and backfilled with argon (5x) and then charged with DMA (dry, 8.8 mL) using a syringe, and the reaction mixture was degassed with an argon balloon and a vent needle while stirring for 45 min. The reaction mixture turned brown at the end of the degassing period. The vent needle was removed, the septum and argon balloon replaced. Silylbenzene (125 mg, 143 µL, 578 µmol, 4 equiv.) was added using a syringe, then the reaction mixture was placed into a preheated 35 °C water bath. The reaction underwent a series of changes in appearance similar to XEC with 2-iodo-6-methyl pyridine from turbid brown-yellow to turbid yellow-olive to turbid pastel green/light jade to turbid dark jade within a timespan of 15 – 30 min. The reaction was stirred for 6.5 h. Upon reaction completion (No SM mass found on LC-MS, faint SM spot remaining, black reaction mixture that cleared up upon workup) EtOAc (8.8 mL) and ice-cold water (17 mL) were added. The layers were separated and the aqueous layer was extracted with EtOAc (6 x 2 mL). The combined organic layers were washed with half saturated brine (2 x 8.8 mL) and brine (2 mL). The combined washes were back extracted with EtOAc (2 mL) and the back extract was washed with half saturated brine (2 x 2 mL), then combined with the organic layer, dried over $MgSO_4$, filtered and concentrated *in vacuo*. The crude product was purified by flash column chromatography (40 g SiO_2, load in toluene, elute 10-15-20-25-30-35-40% EtOAc in hexanes, Combi-flash purification system). The product containing fractions were concentrated *in vacuo* and further purified by column chromatography (SiO_2, load in toluene, elute 10% acetone in hexanes). The diastereomers were separated by column chromatography (SiO_2, load in toluene, elute 1.25% EtOAc in DCM). The product containing fractions were concentrated *in vacuo* affording 41.3 mg (39% yield) of ketone **8a** as white crystals and 28.5 mg (27% yield) of ketone **7a** as white crystals.

Hydrazone condensation. An 8 mL glass vial equipped with a stir bar was charged with EtOH (0.5 mL) and hydrazine monohydrate (54.8 mg, 53.3 µL, 1.10 mmol, 40 equiv.). The resulting solution was placed in a 40 °C water bath with stirring, and a solution of ketone **7a** (10.0 mg, 27.4 µmol, 1 equiv.) in Chloroform (0.1 mL) was added. Upon full starting material consumption (4.5 h, monitored by TLC), the water bath was removed and the reaction mixture was blown down using nitrogen. The residue was dried with toluene/MeOH azeotrope (1:1, 1x), toluene (3x) and hexanes (3x) to afford a white solid.

Barton iodination. The crude mixture of hydrazones (assume 27.4 µmol) was charged with THF (dry, inhibitor-free, 0.1 mL) and cooled to 0 °C using an ice bath. A separate vessel was charged with iodine (14.0 mg, 56.1 µmol, 2.05 equiv.), TMG (221 mg, 240 µL, 1.92 mmol, 70 equiv.) and THF (dry, inhibitor-free, 250 µL). The iodine solution was taken up in a syringe and added dropwise to the cooled hydrazone solution giving an orange color that faded over time. Full consumption of productive intermediates was indicated once the orange color persisted and further addition of iodine solution only intensified the color and consistent with TLC data. Na_2SO_3 (saturated aqueous, 0.5 mL) was added, the ice-bath was removed, and the reaction mixture was diluted with EtOAc (1.25 mL) and minimal amount of water to dissolve all solids. The layers were separated, the aqueous layer was extracted with EtOAc (2 x 1.25 mL), the combined organic layers were washed with brine (2 mL). The brine wash was backextracted with EtOAc (3 x 2 mL). The combined organic layers were dried over $MgSO_4$, filtered and concentrated *in vacuo*. The resulting crude product was purified by column chromatography (SiO_2, load in toluene, elute 10-15-20-25% EtOAc in hexanes). The product containing fractions were concentrated *in vacuo* affording 8.4 mg (65% yield over two steps) of vinyl iodide a white solid that was taken up to the next step.

Carbonylation. A flame-dried 8 mL glass vial equipped with a stir bar and septum was charged with the white solid (8.4 mg, 18.0 µmol, 1 equiv.) obtained from the previous step and $PdCl_2(PPh_3)_2$ (4.0 mg, 5.3 µmol, 0.3 equiv.). The vial was evacuated (using a needle attached to the vacuum line, pump exhaust was vented into a well-ventilated fume hood) and back filled with CO gas (needle pierced through septum fed by a CO balloon, 3x). DMF (dry, 0.25 mL), MeOH (dry, 0.25 mL) and NEt_3 (0.14 mL) were added via syringes. The reaction mixture was degassed with a CO balloon and venting needle while stirring for 15 min resulting in a homogeneous reaction mixture. The CO balloon was replaced with a fresh one and the reaction was placed into a preheated 50°C water bath for 2 h. Upon full conversion, the headspace was purged with argon for 5 min. The reaction mixture was blown down using nitrogen gas and then dried with toluene azeotrope (5x). The crude mixture was purified by preparative TLC (SiO_2, 20 x 10 cm, 0.25 mm thick, load in $CHCl_3$ on long side, elute 10% EtOAc in hexanes, run 2 consecutive times). The obtained product was further purified by another round of preparative TLC (SiO_2, 10 x 10 cm, 0.25 mm thick, load in $CHCl_3$, elute 5% acetone in hexanes) affording 6.2 mg (86% yield) of **RS1137** as a white solid.

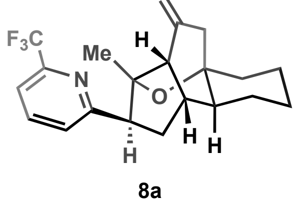

8a

Characterization data for ketone **8a**

Description White crystals

TLC R_f = 0.45 (SiO_2, 30% EtOAc in hexanes); UV-active, stains blue in anisaldehyde

^{1}H NMR (600 MHz, $CDCl_3$) δ 7.74 (t, J = 7.8 Hz, 1H), 7.48 (dd, J = 7.8, 1.0 Hz, 1H), 7.34 (d, J = 7.8 Hz, 1H), 3.57 (t, J = 7.8 Hz, 1H), 2.74 (d, J = 3.9 Hz, 1H), 2.67 (d, J = 19.2 Hz, 1H), 2.51 (ddd, J = 12.6, 7.5, 5.1 Hz, 1H), 2.23 – 2.15 (m, 2H), 1.98 (dd, J = 19.2, 1.6 Hz, 1H), 1.75 (dp, J = 9.6, 3.3 Hz, 4H), 1.59 (ddt, J = 13.2, 4.1, 2.0 Hz, 1H), 1.50 (td, J = 12.5, 4.0 Hz, 1H), 1.40 – 1.32 (m, 2H), 1.16 (qd, J = 13.2, 3.6 Hz, 1H), 0.79 (s, 3H).
^{13}C NMR (151 MHz, $CDCl_3$) δ 212.9, 163.2, 148.3 (q, $^{2}J_{CF}$ = 34.4 Hz), 137.5, 127.2, 121.5 (q, $^{1}J_{CF}$ = 274.4 Hz), 118.2 (q, $^{3}J_{CF}$ = 2.8 Hz), 87.4, 75.3, 58.7, 58.5, 49.9, 42.9, 42.8, 41.6, 36.2, 30.6, 25.8, 23.8, 23.0.
^{19}F NMR (376 MHz, $CDCl_3$) δ -70.88.
2D NMR COSY, HSQC, HMBC, NOESY
LC-MS m/z = 366.2 $[M+H]^+$

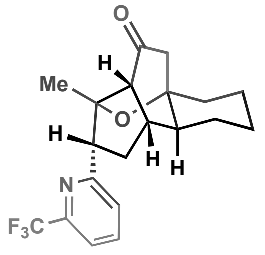

7a

Characterization data for ketone **7a**
Description white crystals
TLC R_f = 0.35 (SiO_2, (30% EtOAc in hexanes); UV-active, stains blue in anisaldehyde
^{1}H NMR (600 MHz, $CDCl_3$) δ 7.77 (t, J = 7.7 Hz, 1H), 7.54 (d, J = 7.7 Hz, 1H), 7.40 (d, J = 7.9 Hz, 1H), 3.42 (dd, J = 11.5, 3.8 Hz, 1H), 2.68 (d, J = 19.4 Hz, 1H), 2.52 (ddd, J = 16.5, 13.1, 7.5 Hz, 2H), 2.43 (d, J = 3.9 Hz, 1H), 2.31 (td, J = 12.0, 5.3 Hz, 1H), 2.18 – 2.13 (m, 1H), 1.91 (dd, J = 19.4, 1.8 Hz, 1H), 1.78 – 1.66 (m, 3H), 1.50 (dd, J = 11.8, 3.7 Hz, 1H), 1.40 – 1.21 (m, 6H), 1.12 (qd, J = 13.4, 3.9 Hz, 1H).
^{13}C NMR (151 MHz, $CDCl_3$) δ 213.2, 160.0, 147.3 (q, $^{2}J_{CF}$ = 34.3 Hz), 137.0, 126.8, 121.9 (q, $^{1}J_{CF}$ = 274.0 Hz), 118.1 (q, $^{3}J_{CF}$ = 2.9 Hz), 85.9, 75.0, 61.5, 55.8, 47.4, 43.4, 43.3, 38.3, 34.9, 30.6, 25.8, 25.4, 23.1.
^{19}F NMR (376 MHz, $CDCl_3$) δ -70.55.
2D NMR COSY, HSQC, HMBC, NOESY
LC-MS m/z = 366.2 $[M+H]^+$

Characterization data for hydrazone product
Description white solid
TLC R_f = 0.19 (SiO_2, 30% EtOAc in hexanes); UV-active, stains green in anisaldehyde

Characterization data for vinyl iodide
Description White solid
TLC R_f = 0.74 (SiO_2, 30% EtOAc in hexanes); UV-active, stains purple in anisaldehyde
LC-MS (no ionization observed)

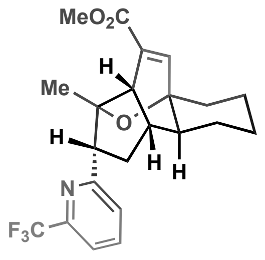

RS1137

<u>Characterization data for **RS1137**</u>

Description white solid

TLC R_f = 0.57 (SiO_2, 30% EtOAc in hexanes); UV-active, stains blue in anisaldehyde

^{1}H NMR (600 MHz, $CDCl_3$) δ 7.73 (t, J = 7.8 Hz, 1H), 7.49 (d, J = 7.7 Hz, 1H), 7.40 (d, J = 7.8 Hz, 1H), 7.24 (d, J = 1.7 Hz, 1H), 3.81 (s, 3H), 3.52 – 3.45 (m, 1H), 3.22 (dd, J = 4.1, 1.9 Hz, 1H), 2.37 – 2.31 (m, 2H), 2.26 (dt, J = 13.1, 3.2 Hz, 1H), 1.85 – 1.78 (m, 2H), 1.67 (d, J = 4.0 Hz, 1H), 1.63 (d, J = 13.6 Hz, 1H), 1.54 (d, J = 17.1 Hz, 1H), 1.45 (qt, J = 13.4, 3.9 Hz, 1H), 1.34 – 1.23 (m, 2H), 1.03 (s, 3H), 0.75 (qd, J = 13.2, 3.6 Hz, 1H).

^{13}C NMR (151 MHz, $CDCl_3$) δ 165.9, 161.6, 147.3 (q, $^2J_{CF}$ = 34.2 Hz), 146.3, 136.8, 132.3, 126.5, 122.0 (q, $^1J_{CF}$ = 274.2 Hz), 117.8 (q, $^3J_{CF}$ = 2.6 Hz), 84.8, 73.9, 54.8, 52.0, 50.4, 48.5, 40.8, 36.1, 32.7, 32.4, 25.7, 23.9, 23.6.

^{19}F NMR (376 MHz, $CDCl_3$) δ -70.62.

2D NMR HSQC

LC-MS m/z = 408.2 $[M+H]^+$

HRMS Calculated $C_{22}H_{25}F_3NO_3$ $[M+H]^+$ = 408.1787 | Found: 408.1784

Synthesis of RS1132

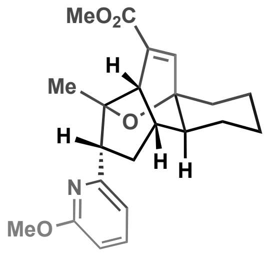

RS1132

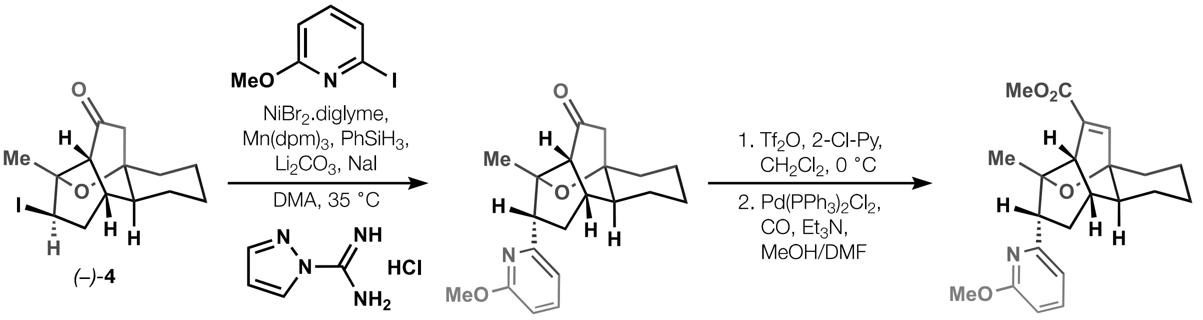

7b **RS1132**

Cross-electrophile coupling (see page S21) A flame-dried 20 mL glass tube with threaded end was equipped with a stir bar and septum pierced with an needle fed by a balloon filled with argon was sequentially charged with praxadine.HCl (85.0 mg, 578 µmol, 2 equiv.), $NiBr_2$•diglyme (224 mg, 635 µmol, 2.2 equiv.), $Mn(dpm)_3$ (245 mg, 404 µmol, 1.4 equiv.), 3-iodo-6-methoxy pyridine (204 mg, 867 µmol, 3 equiv.) *(−)*-**4** (100 mg, 289 µmol, 1 equiv.), Li_2CO_3 (21.0 mg, 289 µmol, 1 equiv.), and NaI (260 mg, 1.73 mmol, 6 equiv.). The vial was evacuated and backfilled with argon (5x) and then charged with DMA (dry, 8.8 mL) using a syringe, and the reaction mixture was degassed with an argon balloon and a vent needle while stirring for 45 min. The reaction mixture turned brown at the end of the degassing period. The vent needle was removed, the septum and argon balloon replaced. Silylbenzene (125 mg, 143 µL, 578 µmol, 4 equiv.) was added using a syringe, then the reaction mixture was placed into a preheated 35 °C water bath. The reaction underwent a series of changes in appearance similar to XEC with 2-iodo-6-methyl pyridine from turbid brown-yellow to turbid yellow-olive to turbid pastel green/light jade to turbid dark jade within a timespan of 15 – 30 min. The reaction was stirred for 7 h. Upon reaction completion (No SM mass found on LC-MS, faint SM spot remaining, black reaction mixture that became clear orange up during workup) EtOAc (8.8 mL) and ice-cold water (17 mL) were added. The layers were separated and the aqueous layer was extracted with EtOAc (6 x 2mL). The combined organic layers were washed with half saturated brine (2 x 8.8 mL) and brine (2 mL). The combined washes were back extracted with EtOAc (2 mL) and the back extract was washed with half saturated brine (2 x 2 mL), then combined with the organic layer, dried over $MgSO_4$, filtered and concentrated *in vacuo*. The crude product was purified and the diastereomers were separated by column chromatography (SiO_2, load in toluene, elute 10-15-20-25-30-35-40% EtOAc in hexanes).

Exo product. **8b** The product containing fractions were concentrated *in vacuo* and then further purified by column chromatography (SiO_2, load in toluene, elute 10% acetone in hexanes) affording 2.9 mg (3% yield) of ketone **8b** as a white solid containing an unknown impurity as an inseparable mixture.

Endo product. **7b** The product containing fractions were concentrated *in vacuo* and then further purified by column chromatography (SiO_2, load in toluene, elute 10% acetone in hexanes) affording 19.9 mg (21% yield) of ketone **7b** as white crystals.

Triflation. A flame-dried 8 mL vial equipped with a stir bar and septum pierced with an argon balloon was charged with ketone **7b** (5.0 mg, 15.3 µmol, 1 equiv.), DCM (dry, 200 µL) and 2-chloropyridine (13 mg, 10.8 µL, 115 µmol, 7.5 equiv.). The reaction mixture was cooled to 0°C using an ice bath then triflic anhydride (21.5 mg, 12.9 µL, 76.4 µmol, 5 equiv.) was added dropwise. Upon complete addition (~5 min), the ice bath was removed, stirring was stopped and the reaction mixture was allowed to warm to room temperature. The stirring was occasionally turned on (300 rpm) for 5 sec. Upon completion (1 h), the reaction mixture was cooled to 0 °C using an ice bath, $NaHCO_3$ (saturated aqueous 3 mL) and Na_2SO_3 (saturated aqueous, 3 mL) were added. The reaction mixture was extracted with diethyl ether (3 x 3 mL) and the combined organic layers were washed with water (3 x 2 mL), brine (2 mL), dried over $MgSO_4$, filtered and concentrated *in vacuo*. The crude product was purified by column chromatography (SiO_2, load in toluene, elute 10% EtOAc in hexanes) affording 3.3 mg (47% yield) of vinyl triflate as a white solid.

Carbonylation. A flame-dried 8 mL glass vial equipped with a stir bar and septum was charged with vinyl triflate (3.3 mg, 7.2 µmol, 1 equiv.) and $PdCl_2(PPh_3)_2$ (1.0 mg, 2.2 µmol, 0.3 equiv.). The vial was evacuated (using a needle attached to the vacuum line, pump exhaust was vented into a well-ventilated fume hood) and back filled with CO gas (needle pierced through septum fed by a CO balloon, 3x). DMF (dry, 0.25 mL), MeOH (dry, 0.25 mL) and NEt_3 (0.14 mL) were added via syringes. The reaction mixture was degassed with a CO balloon and venting needle while stirring for 15 min resulting in a homogeneous reaction mixture. The CO balloon was replaced with a fresh one and the reaction was placed into a preheated 50 °C water bath for 2 h. Upon full conversion, the headspace was purged with argon for 5 min. The reaction mixture was blown down using nitrogen gas and then dried with toluene (5x) azeotrope. The crude mixture was purified by preparative TLC (SiO_2, 10 x 10 cm, 0.25 mm thick, load in $CHCl_3$, elute 10% EtOAc in hexanes). The obtained product was further purified by another round of preparative TLC (SiO_2, 10 x 10 cm, 0.25 mm thick, load in $CHCl_3$, elute 5% acetone in hexanes) affording 2.2 mg (83% yield) of **RS1132** as a colorless oil.

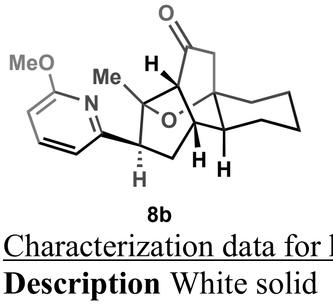

8b

Characterization data for ketone **8b** (10% impurity present)

Description White solid

TLC R_f = 0.59 (SiO_2, 30% EtOAc in hexanes); UV-active, stains first blue, then turns purple in anisaldehyde

LC-MS m/z = 328.2 $[M+H]^+$

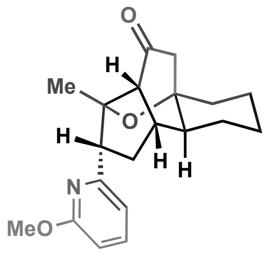

7b

<u>Characterization data for ketone **7b**</u>
Description White crystals
TLC R_f = 0.52 (SiO_2, 30% EtOAc in hexanes); UV-active, stains blue in anisaldehyde
^{1}H NMR (600 MHz, $CDCl_3$) δ 7.49 (dd, *J* = 8.2, 7.2 Hz, 1H), 6.80 (d, *J* = 7.2 Hz, 1H), 6.59 (dd, *J* = 8.2, 0.8 Hz, 1H), 3.99 (s, 3H), 3.25 (dd, *J* = 11.5, 4.0 Hz, 1H), 2.66 (d, *J* = 19.3 Hz, 1H), 2.49 (dd, *J* = 12.8, 4.0 Hz, 1H), 2.43 (ddt, *J* = 13.6, 4.0, 1.8 Hz, 1H), 2.40 (d, *J* = 3.9 Hz, 1H), 2.27 (ddd, *J* = 12.7, 11.5, 5.6 Hz, 1H), 2.12 (ddd, *J* = 5.8, 3.8, 2.2 Hz, 1H), 1.93 (dd, *J* = 19.3, 1.6 Hz, 1H), 1.79 – 1.67 (m, 3H), 1.60 (dt, *J* = 9.7, 1.8 Hz, 1H), 1.37 – 1.23 (m, 6H), 1.14 (qd, *J* = 12.9, 3.6 Hz, 1H).
^{13}C NMR (151 MHz, $CDCl_3$) δ 213.4, 163.3, 156.6, 138.4, 117.0, 108.4, 86.0, 74.6, 61.5, 55.6, 53.4, 48.3, 43.3 (2C), 38.1, 35.4 30.5, 26.1, 25.4, 23.1.
2D NMR COSY, HSQC, HMBC, NOESY
LC-MS m/z = 328.2 $[M+H]^+$

<u>Characterization data for vinyl triflate</u>
Description White solid
TLC R_f = 0.83 (SiO_2, 30% EtOAc in hexanes); UV-active, stains blue in anisaldehyde
LC-MS m/z = 460.2 $[M+H]^+$

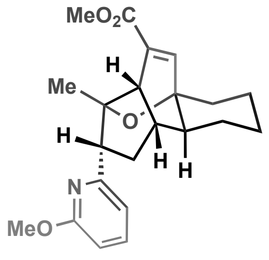

RS1132

<u>Characterization data for **RS1132**</u>
Description colorless oil
TLC R_f = 0.73 (SiO_2, 30% EtOAc in hexanes); UV-active, stains blue in anisaldehyde
^{1}H NMR (600 MHz, $CDCl_3$) δ 7.45 (t, *J* = 7.7 Hz, 1H), 7.23 (d, *J* = 1.7 Hz, 1H), 6.79 (d, *J* = 7.2 Hz, 1H), 6.56 (d, *J* = 8.2 Hz, 1H), 3.97 (s, 3H), 3.81 (s, 3H), 3.31 (dd, *J* = 11.4, 4.4 Hz, 1H), 3.18 (dd, *J* = 4.1, 1.8 Hz, 1H), 2.35 (dd, *J* = 12.8, 4.4 Hz, 1H), 2.28 (td, *J* = 12.1, 5.6 Hz, 1H), 2.20 (dt, *J* = 13.3, 3.3 Hz, 1H), 1.91 (dt, *J* = 12.4, 3.4 Hz, 1H), 1.81 (d, *J* = 14.2 Hz, 1H), 1.67 – 1.62 (m, 2H), 1.56 – 1.42 (m, 2H), 1.32 (td, *J* = 13.0, 4.1 Hz, 1H), 1.18 (tt, *J* = 12.3, 3.5 Hz, 1H), 1.05 (s, 3H), 0.77 (qd, *J* = 13.2, 3.7 Hz, 1H).
^{13}C NMR (151 MHz, $CDCl_3$) δ 166.0, 163.2, 158.3, 145.9, 138.3, 132.6, 116.8, 108.0, 84.9, 73.5, 54.5, 53.4, 52.07, 51.1, 48.5, 40.7, 35.8, 33.2, 32.3, 26.0, 23.8, 23.5.
2D NMR HSQC
LC-MS m/z = 370.2 $[M+H]^+$
HRMS Calculated $C_{22}H_{28}NO_4$ $[M+H]^+$ = 370.2018 | Found: 370.2018

Synthesis of RS1133

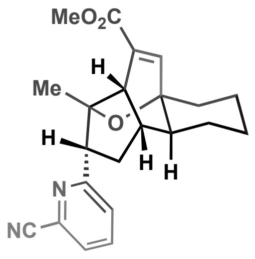

RS1133

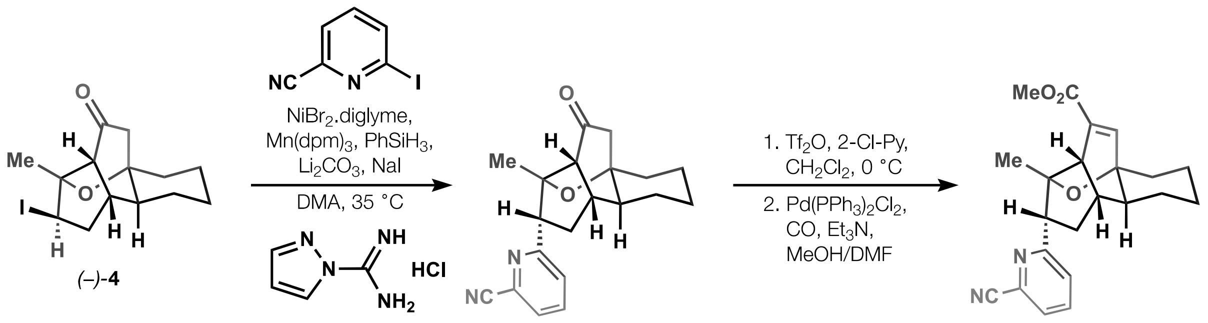

(–)-**4** **7c** **RS1133**

Cross-electrophile coupling (see page S21) A flame-dried 20 mL glass tube with threaded end was equipped with a stir bar and septum pierced with an needle fed by a balloon filled with argon. It was sequentially charged with praxadine.HCl (85.0 mg, 578 µmol, 2 equiv.), $NiBr_2$•diglyme (224 mg, 635 µmol, 2.2 equiv.), $Mn(dpm)_3$ (245 mg, 404 µmol, 1.4 equiv.), 6-bromo-picolinonitrile (159 mg, 867 µmol, 3 equiv.) *(−)*-**4** (100 mg, 289 µmol, 1 equiv.), Li_2CO_3 (21.0 mg, 289 µmol, 1 equiv.), and NaI (260 mg, 1.73 mmol, 6 equiv.). The vial was evacuated and backfilled with argon (5x) and then charged with DMA (dry, 8.8 mL) using a syringe, and the reaction mixture was degassed with an argon balloon and a vent needle while stirring for 45 min. The reaction mixture turned brown at the end of the degassing period. The vent needle was removed, the septum and argon balloon replaced. Silylbenzene (125 mg, 143 µL, 578 µmol, 4 equiv.) was added using a syringe, then the reaction mixture was placed into a preheated 35 °C water bath. The reaction underwent a series of changes in appearance similar to XEC with 2-iodo-6-methyl pyridine from turbid brown-yellow to turbid yellow-olive to turbid pastel green/light jade to turbid dark jade within a timespan of 15 – 30 min. The reaction was stirred for 4.5 h. Upon reaction completion (No SM mass found on LC-MS, faint SM spot remaining, dark forest green colored reaction mixture) EtOAc (8.8 mL) and ice-cold water (17 mL) were added. The layers were separated and the aqueous layer was extracted with EtOAc (6 x 2mL). The combined organic layers were washed with half saturated brine (2 x 8.8 mL) and brine (2 mL). The combined washes were back extracted with EtOAc (2 mL) and the back extract was washed with half saturated brine (2 x 2 mL), then combined with the organic layer, dried over $MgSO_4$, filtered and concentrated *in vacuo*. The crude product was purified by column chromatography (SiO_2, load in toluene with traces of methanol to dissolve everything, elute 10-15-20-25-30-35-40% EtOAc in hexanes). The product containing fractions were concentrated *in vacuo* and the diastereomers were separated by column chromatography (SiO_2, load in toluene, elute 5% EtOAc in DCM). Mixed fractions were concentrated *in vacuo* and the diastereomer were separated by preparative TLC (SiO_2, 20 x 20 cm, 0.5 mm thick, load in $CHCl_3$, elute 5% EtOAc in DCM) affording in total 35.6 mg (38% yield) of ketone **8c** as a white solid and 37.9 mg of endo ketone **7c** (41% yield) as white crystals.

Triflation. A flame-dried 8 mL vial equipped with a stir bar and septum pierced with an argon balloon was charged with ketone **7c** (10 mg, 31.02 µmol, 1 equiv.), DCM (dry, 200 µL) and 2-chloropyridine (50 mg, 42 µL, 0.44 mmol, 14 equiv.). The reaction mixture was cooled to 0°C using an ice bath then triflic anhydride (50 µL) was added dropwise. Upon complete addition (~5 min), the ice bath was removed, stirring was stopped and the reaction mixture was allowed to warm to room temperature. The stirring was occasionally turned on (300 rpm) for 5 sec. Upon completion (1 h), the reaction mixture was cooled to 0 °C using an ice bath, $NaHCO_3$ (saturated aqueous 3 mL) and Na_2SO_3 (saturated aqueous, 3 mL) were added. The reaction mixture was extracted with diethyl ether (3 x 3 mL) and the combined organic layers were washed with water (3 x 2 mL), brine (2 mL), dried over $MgSO_4$, filtered and concentrated *in vacuo*. The crude product was purified by column chromatography (SiO_2, load in toluene, elute 10% acetone in hexanes). Impure product fractions were concentrated *in vacuo* and further purified by preparative TLC (SiO_2, 10 x 10 cm, 0.25 mm thick, load in $CHCl_3$, elute 10% acetone in hexanes) affording in total 5.8 mg (41% yield) of vinyl triflate as a white solid.

Carbonylation. A flame-dried 8 mL glass vial equipped with a stir bar and septum was charged with vinyl triflate (5.8 mg, 13.0 µmol, 1 equiv.) and $PdCl_2(PPh_3)_2$ (3.0 mg, 3.8 µmol, 0.3 equiv.). The vial was evacuated (using a needle attached to the vacuum line, pump exhaust was vented into a well-ventilated fume hood) and back filled with CO gas (needle pierced through septum fed by a CO balloon, 3x). DMF (dry, 0.25 mL), MeOH (dry, 0.25 mL) and NEt_3 (0.14 mL) were added via syringes. The reaction mixture was degassed with a CO balloon and venting needle while stirring for 15 min resulting in a homogeneous reaction mixture. The CO balloon was replaced with a fresh one and the reaction was placed into a preheated 50 °C water bath for 3 h. Upon full conversion, the headspace was purged with argon for 5 min. The reaction mixture was blown down using nitrogen gas and then dried with toluene azeotrope (5x). The crude mixture was purified by preparative TLC (SiO_2, 10 x 10 cm, 0.25 mm thick, load in $CHCl_3$, elute 20% EtOAc in hexanes, run 2 consecutive times). The obtained product was further purified by another round of preparative TLC (SiO_2, 10 x 10 cm, 0.25 mm thick, load in $CHCl_3$, elute 10% acetone in hexanes, run 2 consecutive times) affording 3.7 mg (80% yield) of **RS1133** as a white solid.

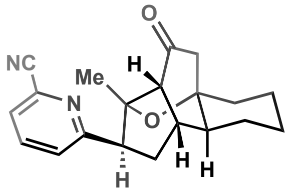

8c

Characterization data for ketone **8c**

Description White solid

TLC R_f = 0.30 (SiO_2, 30% EtOAc in hexanes); UV-active, stains red in anisaldehyde

^{1}H NMR (600 MHz, $CDCl_3$) δ 7.72 (t, *J* = 7.8 Hz, 1H), 7.53 – 7.49 (m, 1H), 7.38 (dd, *J* = 7.9, 1.0 Hz, 1H), 3.54 (t, *J* = 7.8 Hz, 1H), 2.73 (d, *J* = 3.9 Hz, 1H), 2.67 (d, *J* = 19.3 Hz, 1H), 2.49 (ddd, *J* = 12.6, 7.5, 5.1 Hz, 1H), 2.23 – 2.15 (m, 2H), 1.98 (dd, *J* = 19.3, 1.7 Hz, 1H), 1.78 – 1.71 (m, 4H), 1.57 (ddt, *J* = 13.1, 4.0, 1.9 Hz, 1H), 1.48 (td, *J* = 12.2, 3.7 Hz, 1H), 1.40 – 1.23 (m, 3H), 1.16 (qd, *J* = 13.1, 3.7 Hz, 1H), 0.78 (s, 3H).

^{13}C NMR (151 MHz, $CDCl_3$) δ 212.5, 164.6, 137.2, 133.8, 128.1, 126.4, 117.4, 87.3, 75.3, 58.6, 58.5, 50.0, 42.7 (2C), 41.5, 36.2, 30.6, 25.8, 23.9, 23.0.

2D NMR COSY, HSQC, HMBC, NOESY

LC-MS m/z = 323.2 $[M+H]^+$

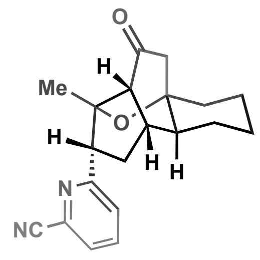

7c

Characterization data for ketone **7c**

Description White crystals

TLC R_f = 0.20 (SiO_2, 30% EtOAc in hexanes); UV-active, stains red in anisaldehyde

^{1}H NMR (600 MHz, $CDCl_3$) δ 7.74 (t, J = 7.8 Hz, 1H), 7.57 (d, J = 7.6 Hz, 1H), 7.47 (d, J = 7.9 Hz, 1H), 3.38 (dd, J = 11.1, 4.3 Hz, 1H), 2.68 (d, J = 19.3 Hz, 1H), 2.43 (d, J = 4.0 Hz, 1H), 2.42 – 2-41 (m, 3H), 2.16 (q, J = 3.6 Hz, 1H), 1.93 (dd, J = 19.3, 1.6 Hz, 1H), 1.77 – 1.69 (m, 3H), 1.61 – 1.55 (m, 1H), 1.45 – 1.26 (m, 6H), 1.13 (qd, J = 12.8, 3.4 Hz, 1H).

^{13}C NMR (151 MHz, $CDCl_3$) δ 212.8, 161.5, 136.7, 133.1, 127.5, 126.3, 117.7, 85.9, 75.0, 61.3, 56.0, 48.3, 43.3, 43.2, 38.6, 35.3, 30.5, 25.8, 25.4, 23.1.

2D NMR COSY, HSQC, HMBC, NOESY

LC-MS m/z = 323.2 $[M+H]^+$

Characterization data for vinyl triflate-X

Description White solid

TLC R_f = 0.49 (SiO_2, 30% EtOAc in hexanes); UV-active, stains blue in anisaldehyde

LC-MS m/z = 455.2 $[M+H]^+$

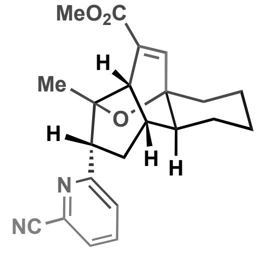

RS1133

Characterization data for **RS1133**

Description white solid

TLC R_f = 0.37 (SiO_2, 30% EtOAc in hexanes); UV-active, stains green in anisaldehyde

^{1}H NMR (600 MHz, $CDCl_3$) δ 7.71 (t, J = 7.8 Hz, 1H), 7.53 (d, J = 7.6 Hz, 1H), 7.48 (d, J = 8.0 Hz, 1H), 7.24 (d, J = 1.7 Hz, 1H), 3.82 (s, 3H), 3.45 (dd, J = 11.5, 4.2 Hz, 1H), 3.23 (dd, J = 4.1, 1.8 Hz, 1H), 2.42 – 2.33 (m, 1H), 2.23 (dd, J = 13.1, 4.3 Hz, 1H), 2.10 – 2.05 (m, 1H), 1.91 – 1.81 (m, 2H), 1.70 – 1.62 (m, 2H), 1.53 – 1.43 (m, 2H), 1.36 – 1.26 (m, 2H), 1.02 (s, 3H), 0.77 (qd, J = 13.2, 3.7 Hz, 1H).

^{13}C NMR (151 MHz, $CDCl_3$) δ 165.8, 163.2, 146.0, 136.5, 133.0, 132.3, 127.3, 126.1, 117.9, 84.9, 73.9, 55.0, 52.0, 51.4, 48.4, 40.5, 36.4, 33.1, 32.3, 25.7, 23.8, 23.5.

2D NMR HSQC

LC-MS m/z = 365.2 $[M+H]^+$

HRMS Calculated $C_{22}H_{25}N_2O_3$ $[M+H]^+$ = 365.1865 | Found: 365.1856

Synthesis of RS1120

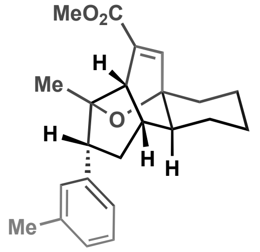

RS1120

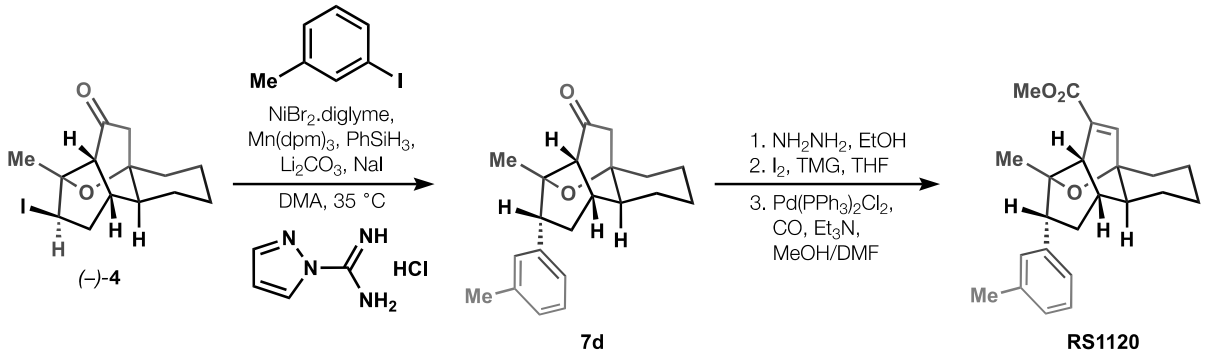

Cross-electrophile coupling (see page S21) A flame-dried 20 mL glass tube with threaded end was equipped with a stir bar and septum pierced with a needle fed by a balloon filled with argon was sequentially charged with praxadine.HCl (42.0 mg, 289 µmol, 2 equiv.), $NiBr_2$•diglyme (112 mg, 318 µmol, 2.2 equiv.), $Mn(dpm)_3$ (122 mg, 202 µmol, 1.4 equiv.) *(−)*-**4** (50 mg, 144 µmol, 1 equiv.), Li_2CO_3 (11.0 mg, 144 µmol, 1 equiv.), and NaI (130 mg, 867 µmol, 6 equiv.). The vial was evacuated and backfilled with argon (5x) and then charged with DMA (dry, 4.4 mL) using a syringe. 3-iodotoluene (157 mg, 92.7 µL, 722 µmol, 5 equiv.) were added using a micro-syringe and the reaction mixture was degassed with an argon balloon and a vent needle while stirring for 30 min. The reaction mixture turned brown at the end of the degassing period. The vent needle was removed, the septum and argon balloon replaced. Silylbenzene (62.5 mg, 71.6 µL, 578 µmol, 4 equiv.) was added using a micro-syringe, then the reaction mixture was placed into a preheated 35 °C water bath. The reaction underwent a series of changes in appearance similar to XEC with 2-iodo-6-methyl pyridine from turbid brown-yellow to turbid yellow-olive to turbid pastel green/light jade to turbid dark jade within a timespan of 15 – 30 min. The reaction was stirred for 3.5 h. Upon reaction completion (No SM mass found on LC-MS, faint SM spot remaining, dark forest green reaction mixture) EtOAc (4.25 mL) and ice-cold water (8.5 mL) were added. The layers were separated and the aqueous layer was extracted with EtOAc (6 x 1mL). The combined organic layers were washed with half saturated brine (2 x 4.25 mL) and brine (1 mL). The combined washes were back extracted with EtOAc (1 mL) and the back extract was washed with half saturated brine (2 x 1 mL), then combined with the organic layer, dried over $MgSO_4$, filtered and concentrated *in vacuo*. The crude product was purified and diastereomers were separated by column chromatography (SiO_2, load in toluene, elute 10-15-20-20-20% EtOAc in hexanes). The product containing fractions were concentrated *in vacuo* and then further purified by three rounds of column chromatography (SiO_2, load in toluene, elute 0.5% MeOH in DCM), (SiO_2, load in toluene, elute 10% acetone in hexane), and (SiO_2, load in toluene, elute 10-15-20-25% EtOAc in hexanes). The diastereomers were separated by preparative TLC (SiO_2, 20 x 20 cm, 0.5 mm thick, load in $CHCl_3$, elute 2.5% EtOAc in benzene) affording 12.0 mg (13% yield) of ketone **8d** as a colorless oil containing starting material as an inseparable mixture and 11.8 mg

(13% yield) of ketone **7d** as a colorless oil that formed a white solid upon storage in a -20°C freezer.

Hydrazone condensation. An 8 mL glass vial equipped with a stir bar was charged with EtOH (0.5 mL) and hydrazine monohydrate (54.8 mg, 53.3 µL, 1.10 mmol, 40 equiv.). The resulting solution was placed in a 40 °C water bath with stirring, and a solution of ketone **7d** (8.0 mg, 27.4 µmol, 1 equiv.) in Chloroform (0.1 mL) was added. Upon full starting material consumption (3 h, monitored by TLC), the water bath was removed and the reaction mixture was blown down using nitrogen. The residue was dried with toluene/MeOH azeotrope (1:1, 1x), toluene (3x) and hexanes (3x) to afford a white solid.

Barton iodination. The crude mixture of hydrazones (assume 27.4 µmol) was charged with THF (dry, inhibitor-free, 0.1 mL) and cooled to 0 °C using an ice bath. A separate vessel was charged with iodine (14.0 mg, 56.2 µmol, 2.05 equiv.), TMG (221 mg, 240 µL, 1.92 mmol, 70 equiv.) and THF (dry, inhibitor-free, 250 µL). The iodine solution was taken up in a syringe and added dropwise to the cooled hydrazone solution giving an orange color that faded over time. Full consumption of productive intermediates was indicated once the orange color persisted and further addition of iodine solution only intensified the color and consistent with TLC data. Na_2SO_3 (saturated aqueous, 0.5 mL) was added, the ice-bath was removed, and the reaction mixture was diluted with EtOAc (1.25 mL) and minimal amount of water to dissolve all solids. The layers were separated, the aqueous layer was extracted with EtOAc (2 x 1.25 mL), the combined organic layers were washed with brine (2 mL). The brine wash was backextracted with EtOAc (3 x 2 mL). The combined organic layers were dried over $MgSO_4$, filtered and concentrated *in vacuo*. The resulting crude product was purified by column chromatography (SiO_2, load in toluene, elute 10-15-20% EtOAc in hexanes). The product containing fractions were concentrated *in vacuo* affording 6.9 mg (60% yield over two steps) of vinyl iodide as a white solid.

Carbonylation. A flame-dried 8 mL glass vial equipped with a stir bar and septum was charged with vinyl iodide (6.9 mg, 16.0 µmol, 1 equiv.) and $PdCl_2(PPh_3)_2$ (2.0 mg, 2.5 µmol, 0.15 equiv.). The vial was evacuated (using a needle attached to the vacuum line, pump exhaust was vented into a well-ventilated fume hood) and back filled with CO gas (needle pierced through septum fed by a CO balloon, 3x). DMF (dry, 0.25 mL), MeOH (dry, 0.25 mL) and NEt_3 (0.14 mL) were added via syringes. The reaction mixture was degassed with a CO balloon and venting needle while stirring for 15 min resulting in a homogeneous reaction mixture. The CO balloon was replaced with a fresh one and the reaction was placed into a preheated 50 °C water bath for 4 h. Upon full conversion, the headspace was purged with argon for 5 min. The reaction mixture was blown down using nitrogen gas and then dried with toluene azeotrope (5x). The crude mixture was purified by preparative TLC (SiO_2, 20 x 10 cm, 0.25 mm thick, load in $CHCl_3$ on long side, elute 20% EtOAc in hexanes, run 2 consecutive times). The obtained product was further purified by another round of preparative TLC (SiO_2, 20 x 10 cm, 0.25 mm thick, load in $CHCl_3$ on long side, elute 30% EtOAc in hexanes) affording 4.2 mg (73% yield) of **RS1120** as a colorless oil.

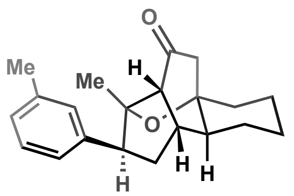

8d

Characterization data for ketone **8d** [in separable with *(−)*-**4**]
Description colorless oil
TLC R_f = 0.43 (SiO_2, 5% EtOAc in benzene); UV-active, stains initially grey then turns purple in anisaldehyde
LC-MS m/z = 293.2 $[M-H_2O+H]^+$

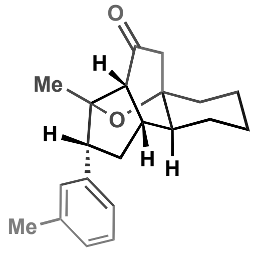

7d

Characterization data for ketone **7d**
Description White solid
TLC R_f = 0.33 (SiO_2, 5% EtOAc in benzene); UV-active, stains grey in anisaldehyde
^{1}H NMR (600 MHz, $CDCl_3$) δ 7.22 (t, *J* = 7.5 Hz, 1H), 7.15 – 7.11 (m, 2H), 7.07 (ddd, *J* = 7.2, 2.0, 1.1 Hz, 1H), 2.99 (dd, *J* = 11.9, 5.5 Hz, 1H), 2.71 (d, *J* = 19.2 Hz, 1H), 2.62 (ddd, *J* = 13.5, 11.9, 6.3 Hz, 1H), 2.44 (d, *J* = 4.3 Hz, 1H), 2.36 (s, 3H), 2.18 – 2-15 (m, 1H), 2.01 (dd, *J* = 19.2, 1.5 Hz, 1H), 1.91 – 1.84 (m, 2H), 1.82 – 1.68 (m, 4H), 1.52 (td, *J* = 12.2, 3.5 Hz, 1H), 1.44 – 1.34 (m, 2H), 1.22 – 1-14 (m, 4H).
^{13}C NMR (151 MHz, $CDCl_3$) δ 213.2, 139.0, 137.4, 131.0, 128.0, 127.6, 127.2, 84.9, 74.5, 61.2, 54.8, 52.4, 43.1, 42.7, 42.6, 35.6, 30.4, 25.9, 24.5, 23.1, 21.8.
2D NMR COSY, HQSC, HMBC, NOESY
LC-MS m/z = 293.2 $[M-H_2O+H]^+$

Characterization data for hydrazone product
Description white solid
TLC R_f = 0.37 and 0.27 (SiO_2, 30% EtOAc in hexanes, presumably E/Z hydrazones); UV-active, stains yellow in anisaldehyde

Characterization data for vinyl iodide
Description White solid
TLC R_f = 0.86 (SiO_2, 30% EtOAc in hexanes); UV-active, stains grey in anisaldehyde
LC-MS (no ionization observed)

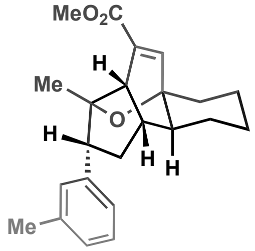

RS1120

Characterization data for methyl enoate **RS1120**
Description colorless oil
TLC R_f = 0.80 (SiO_2, 30% EtOAc in hexanes); UV-active, stains purple in anisaldehyde
^{1}H NMR (600 MHz, $CDCl_3$) δ 7.23 (s, 1H), 7.18 (t, *J* = 7.5 Hz, 1H), 7.12 – 7.08 (m, 2H), 7.02 (d, *J* = 7.5 Hz, 1H), 3.81 (s, 3H), 3.21 (d, *J* = 4.2 Hz, 1H), 3.08 (dd, *J* = 12.0, 5.6 Hz,

1H), 2.59 (td, *J* = 12.8, 6.3 Hz, 1H), 2.34 (s, 3H), 2.02 (d, *J* = 11.1 Hz, 1H), 1.90 – 1.84 (m, 1H), 1.78 (dd, *J* = 13.6, 5.6 Hz, 1H), 1.74 – 1.65 (m, 3H), 1.54 – 1-45 (m 3H), 1.37 – 1.27 (m, 1H), 0.88 (s, 3H), 0.82 (qd, , *J* = 13.2, 3.5 Hz, 1H).
^{13}C NMR (151 MHz, $CDCl_3$) δ 165.9, 145.1, 141.1, 137.3, 132.7, 130.9, 127.9, 127.2, 127.0, 84.6, 73.4, 55.2, 53.4, 52.0, 48.3, 40.4, 39.5, 33.5, 32.2, 25.8, 23.5, 23.0, 21.8.
2D NMR HSQC
LC-MS m/z = 335 $[M-H_2O+H]^+$
HRMS Calculated $C_{23}H_{27}O_2$ $[M+H]^+$ = 335.2011 | Found: 335.2006

Synthesis of RS1136

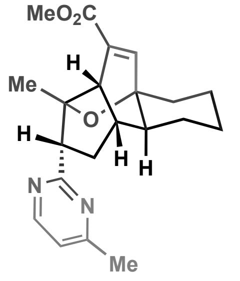

RS1136

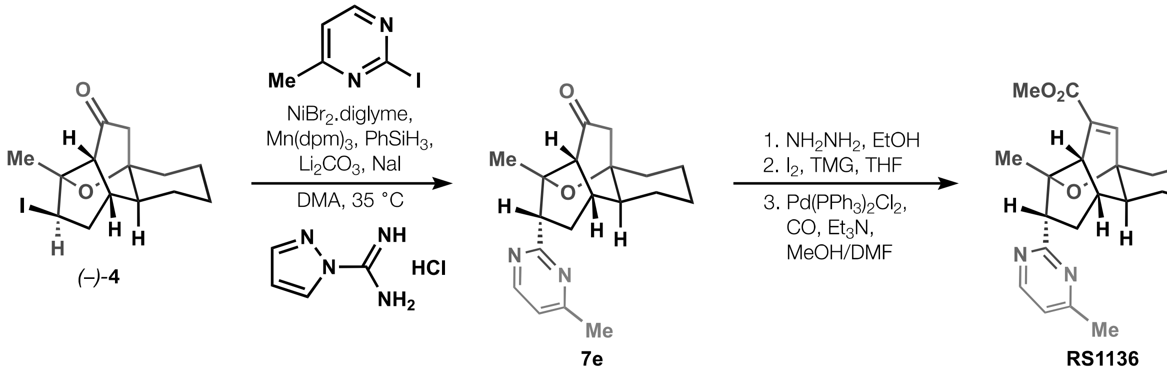

Cross-electrophile coupling (see page S21) A flame-dried 20 mL glass tube with threaded end was equipped with a stir bar and septum pierced with an needle fed by a balloon filled with argon. It was sequentially charged with praxadine.HCl (85.0 mg, 578 µmol, 2 equiv.), $NiBr_2$•diglyme (224 mg, 635 µmol, 2.2 equiv.), $Mn(dpm)_3$ (245 mg, 404 µmol, 1.4 equiv.), 2-bromo-4-methyl pyrimidine (150 mg, 867 µmol, 3 equiv.) *(−)*-**4** (100 mg, 289 µmol, 1 equiv.), Li_2CO_3 (21.0 mg, 289 µmol, 1 equiv.), and NaI (260 mg, 1.73 mmol, 6 equiv.). The vial was evacuated and backfilled with argon (5x) and then charged with DMA (dry, 8.8 mL) using a syringe, and the reaction mixture was degassed with an argon balloon and a vent needle while stirring for 45 min. The reaction mixture turned brown at the end of the degassing period. The vent needle was removed, the septum and argon balloon replaced. Silylbenzene (125 mg, 143 µL, 578 µmol, 4 equiv.) was added using a syringe, then the reaction mixture was placed into a preheated 35 °C water bath. The reaction underwent a series of changes in appearance similar to XEC with 2-iodo-6-methyl pyridine from turbid brown-yellow to turbid yellow-olive to turbid pastel green/light jade to turbid dark jade within a timespan of 15 – 30 min. The reaction was stirred for 2 h. Upon reaction completion (No SM mass found on LC-MS, faint SM spot remaining, dark forest green colored reaction mixture) EtOAc (8.8 mL) and ice-cold water (17 mL) were added. The layers were separated and the aqueous layer was extracted with EtOAc (6 x 2mL). The combined organic layers were washed with half saturated brine (2 x 8.8 mL) and brine (2 mL). The combined washes were back extracted with EtOAc (2 mL) and the back extract was washed with half saturated brine (2 x 2 mL), and then combined with the organic layer. Significant amount of product was remaining in the aqueous layer and washes. The aqueous layer was extracted with EtOAc (2 x 5 mL) and the combined washes were extracted with EtOAc (5 x 5 mL). All combined organic layers were dried over $MgSO_4$, filtered and concentrated *in vacuo*. The crude product was purified by column chromatography (SiO_2, load in toluene, elute 10-15-20-25-30-35-40% acetone in hexanes). The product containing fractions were concentrated *in vacuo* and the diastereomers were separated by column chromatography (SiO_2, load in $CHCl_3$, elute 10% acetone in DCM). Mixed fractions were concentrated *in vacuo* and the diastereomers were separated by preparative TLC (SiO_2, 20 x 20 cm, 0.5 mm thick, load in $CHCl_3$, elute 5% acetone in diisopropyl ether, run 3 consecutive times) affording 8.8

mg (10% yield) of ketone **8e** as a white solid and 26 mg (29% yield) of ketone **7e** as a white solid in total.

Hydrazone condensation. An 8 mL glass vial equipped with a stir bar was charged with EtOH (0.5 mL) and hydrazine monohydrate (64.1 mg, 62.4 µL, 1.28 mmol, 40 equiv.). The resulting solution was placed in a 40 °C water bath with stirring, and a solution of ketone **7e** (10.0 mg, 32.0 µmol, 1 equiv.) in Chloroform (0.1 mL) was added. Upon full starting material consumption (1.5 h, monitored by TLC), the water bath was removed and the reaction mixture was blown down using nitrogen. The residue was dried with toluene/MeOH azeotrope (1:1, 1x), toluene (3x) and hexanes (3x) to afford a white solid.

Barton iodination. The crude mixture of hydrazones (assume 32.0 µmol) was charged with THF (dry, inhibitor-free, 0.1 mL) and cooled to 0 °C using an ice bath. A separate vessel was charged with iodine (17.0 mg, 65.6 µmol, 2.05 equiv.), TMG (258 mg, 281 µL, 2.24 mmol, 70 equiv.) and THF (dry, inhibitor-free, 300 µL). The iodine solution was taken up in a syringe and added dropwise to the cooled hydrazone solution giving an orange color that faded over time. Full consumption of productive intermediates was indicated once the orange color persisted and further addition of iodine solution only intensified the color and consistent with TLC data. Na_2SO_3 (saturated aqueous, 0.5 mL) was added, the ice-bath was removed, and the reaction mixture was diluted with EtOAc (1.25 mL) and minimal amount of water to dissolve all solids. The layers were separated, the aqueous layer was extracted with EtOAc (2 x 1.25 mL), the combined organic layers were washed with brine (2 mL). The brine wash was backextracted with EtOAc (3 x 2 mL). The combined organic layers were dried over $MgSO_4$, filtered and concentrated *in vacuo*. The resulting crude product was purified by column chromatography (SiO_2, load in toluene, elute 10-15-20-25% acetone in hexanes). The product containing fractions were concentrated *in vacuo* affording 9.2 mg (68% yield over two steps) of vinyl iodide as a white solid.

Carbonylation. A flame-dried 8 mL glass vial equipped with a stir bar and septum was charged with vinyl iodide (9.2 mg, 22.0 µmol, 1 equiv.) and $PdCl_2(PPh_3)_2$ (5.0 mg, 6.5 µmol, 0.3 equiv.). The vial was evacuated (using a needle attached to the vacuum line, pump exhaust was vented into a well-ventilated fume hood) and back filled with CO gas (needle pierced through septum fed by a CO balloon, 3x). DMF (dry, 0.25 mL), MeOH (dry, 0.25 mL) and NEt_3 (0.14 mL) were added via syringes. The reaction mixture was degassed with a CO balloon and venting needle while stirring for 15 min resulting in a homogeneous reaction mixture. The CO balloon was replaced with a fresh one and the reaction was placed into a preheated 50 °C water bath for 2.5 h. Upon full conversion, the headspace was purged with argon for 5 min. The reaction mixture was blown down using nitrogen gas and then dried with toluene azeotrope (5x). The crude mixture was purified by preparative TLC (SiO_2, 10 x 10 cm, 0.25 mm thick, load in $CHCl_3$, elute 20% EtOAc in hexanes, run 2 consecutive times). The obtained product was further purified by another round of preparative TLC (SiO_2, 10 x 10 cm, 0.25 mm thick, load in $CHCl_3$, elute 10% acetone in hexanes, run 2 consecutive times) affording 5.7 mg (76% yield) of **RS1136** as a white solid.

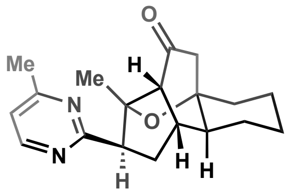

8e

Characterization data for ketone **8e**
Description White solid
TLC R_f = 0.29 (SiO_2, 20% acetone in hexanes); UV-active, stains red in anisaldehyde (slowly fades to orange over time)
^{1}H NMR (600 MHz, $CDCl_3$) δ 8.47 (d, *J* = 5.1 Hz, 1H), 6.95 (d, *J* = 5.1 Hz, 1H), 3.71 (t, *J* = 7.9 Hz, 1H), 2.76 (d, *J* = 3.9 Hz, 1H), 2.66 (d, *J* = 19.2 Hz, 1H), 2.54 – 2.44 (m, 4H), 2.24 – 2.15 (m, 2H), 1.98 (dd, *J* = 19.3, 1.6 Hz, 1H), 1.58 (ddt, *J* = 13.4, 4.1, 2.0 Hz, 1H), 1.48 (tt, *J* = 11.3, 5.2 Hz, 1H), 1.38 – 1.26 (m, 2H), 1.15 (qd, *J* = 13.0, 3.6 Hz, 1H), 0.83 (s, 3H).
^{13}C NMR (151 MHz, $CDCl_3$) δ 213.7, 171.6, 166.9, 156.5, 118.3, 87.5, 75.0, 60.1, 58.7, 49.9, 43.1, 42.9, 41.2, 36.1, 30.7, 25.9, 24.4, 23.3, 23.0.
2D NMR COSY, HSQC, HMBC, NOESY
LC-MS m/z = 313.2 $[M+H]^+$

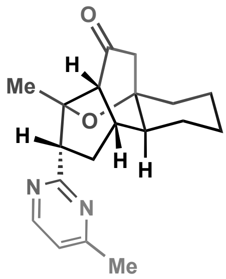

7e

Characterization data for ketone **7e**
Description White solid
TLC R_f = 0.29 (SiO_2, 20% acetone in hexanes); UV-active, stains red in anisaldehyde (slowly fades to orange over time)
^{1}H NMR (600 MHz, $CDCl_3$) δ 8.53 (d, *J* = 5.1 Hz, 1H), 6.99 (d, *J* = 5.1 Hz, 1H), 3.53 (dd, *J* = 11.3, 3.3 Hz, 1H), 2.70 (dd, *J* = 12.6, 3.4 Hz, 1H), 2.64 (d, *J* = 19.3 Hz, 1H), 2.51 (s, 4H), 2.40 (d, *J* = 3.7 Hz, 1H), 2.18 (td, *J* = 11.9, 5.2 Hz, 1H), 2.14 – 2.11 (m, 1H), 1.91 (d, *J* = 19.3 Hz, 1H), 1.76 – 1.69 (m, 2H), 1.67 – 1.64 (m, 1H), 1.49 (m, 4H), 1.35 – 1.26 (m, 3H), 1.16 – 1.06 (m, 2H).
^{13}C NMR (151 MHz, $CDCl_3$) δ 213.4, 168.6, 166.2, 156.0, 118.2, 87.0, 74.7, 61.6, 56.7, 46.7, 43.6, 43.3, 36.7, 35.3, 30.6, 25.9, 25.9, 24.5, 23.1.
2D NMR COSY, HSQC, HMBC, NOESY
LC-MS m/z = 313.2 $[M+H]^+$

Characterization data for hydrazone product
Description white solid
TLC R_f = 0.60 (SiO_2, 50% acetone in hexanes); UV-active, stains yellow in anisaldehyde

Characterization data for vinyl iodide X
Description White solid
TLC R_f = 0.69 (SiO_2, 50% acetone in hexanes); UV-active, stains red in anisaldehyde
LC-MS m/z = 423.2 $[M+H]^+$

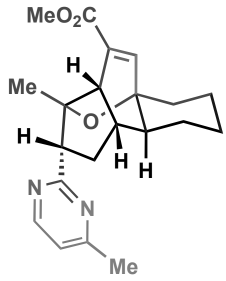

RS1136

<u>Characterization data for methyl enoate **RS1136**</u>

Description White solid

TLC R_f = 0.69 (SiO_2, 50% acetone in hexanes); UV-active, stains red in anisaldehyde

^{1}H NMR (600 MHz, $CDCl_3$) δ 8.50 (d, *J* = 5.1 Hz, 1H), 7.23 (d, *J* = 1.7 Hz, 1H), 6.95 (d, *J* = 5.1 Hz, 1H), 3.81 (s, 3H), 3.59 (dd, *J* = 11.4, 3.4 Hz, 1H), 3.19 (dd, *J* = 4.0, 1.9 Hz, 1H), 2.56 (dd, *J* = 12.7, 3.5 Hz, 1H), 2.49 (s, 2H), 2.25 (dt, *J* = 13.5, 3.2 Hz, 1H), 2.20 (dt, *J* = 12.0, 6.1 Hz, 1H), 1.84 – 1.79 (m, H), 1.76 (d, *J* = 14.4 Hz, 1H), 1.65 – 1.60 (m, 2H), 1.52 (d, *J* = 13.3 Hz, 1H), 1.48 – 1.40 (m, 1H), 1.29 – 1.22 (m, 1H) 1.20 (s, 3H), 1.13 (td, *J* = 13.0, 4.2 Hz, 1H), 0.73 (qd, *J* = 13.2, 3.6 Hz, 1H).

^{13}C NMR (151 MHz, $CDCl_3$) δ 169.8, 166.1, 166.0, 155.9, 146.2, 132.5, 118.0, 85.5, 73.6, 55.7, 52.0, 49.3, 48.6, 41.1, 34.1, 33.0, 32.3, 25.7, 24.5, 24.4, 23.5.

2D NMR HSQC

LC-MS m/z = 355.2 $[M+H]^+$

HRMS Calculated $C_{21}H_{27}N_2O_3$ $[M+H]^+$ = 355.2022 | Found: 355.2020

Synthesis of RS1131

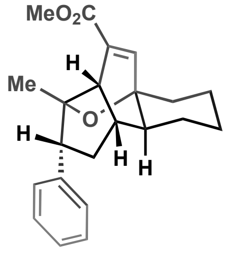

RS1131

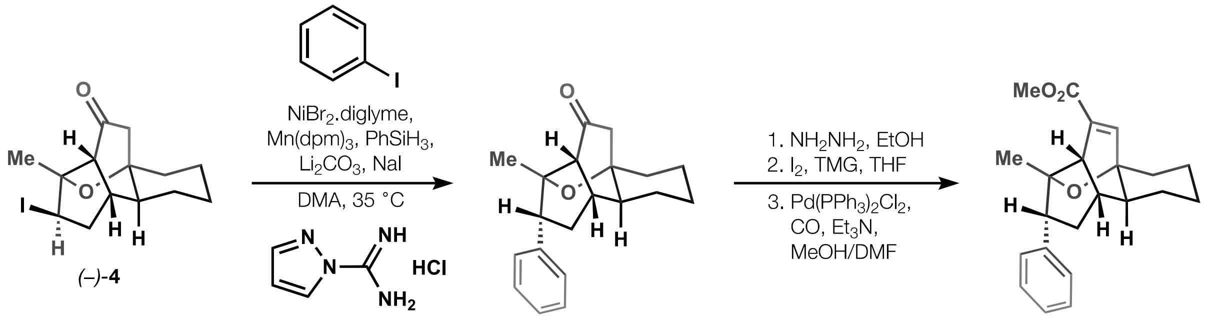

Note: ketone **7f** and **8f** has been synthesized alternatively via Kumada coupling (see page S27) and defined as **13a** and ***epi*-13a** respectively.

Cross-electrophile coupling (see page S26)

Hydrazone condensation. An 8 mL glass vial equipped with a stir bar was charged with EtOH (0.5 mL) and hydrazine monohydrate (67.6 mg, 65.7 µL, 1.35 mmol, 40 equiv.). The resulting solution was placed in a 40 °C water bath with stirring, and a solution of ketone **7f** (10.0 mg, 33.7 µmol, 1 equiv., derived from *(−)*-**4**) in Chloroform (0.1 mL) was added. Upon full starting material consumption (2 h, monitored by TLC), the water bath was removed and the reaction mixture was blown down using nitrogen. The residue was dried with toluene/MeOH azeotrope (1:1, 1x), toluene (3x) and hexanes (3x) to afford a white solid.

Barton iodination. The crude mixture of hydrazones (assume 33.7 µmol) was charged with THF (dry, inhibitor-free, 0.1 mL) and cooled to 0 °C using an ice bath. A separate vessel was charged with iodine (18.0 mg, 69.3 µmol, 2.05 equiv.), TMG (273 mg, 297 µL, 2.37 mmol, 70 equiv.) and THF (dry, inhibitor-free, 300 µL). The iodine solution was taken up in a syringe and added dropwise to the cooled hydrazone solution giving an orange color that faded over time. Full consumption of productive intermediates was indicated once the orange color persisted and further addition of iodine solution only intensified the color and consistent with TLC data. Na_2SO_3 (saturated aqueous, 0.5 mL) was added, the ice-bath was removed, and the reaction mixture was diluted with EtOAc (1.25 mL) and minimal amount of water to dissolve all solids. The layers were separated, the aqueous layer was extracted with EtOAc (2 x 1.25 mL), the combined organic layers were washed with brine (2 mL). The brine wash was backextracted with EtOAc (3 x 2 mL). The combined organic layers were dried over $MgSO_4$, filtered and concentrated *in vacuo*. The resulting crude product was purified by column chromatography (SiO_2, load in toluene, elute 0-5-10-15% EtOAc in hexanes). The product containing fractions were concentrated *in vacuo* affording 8.7 mg (63% yield over two steps) of vinyl iodide as a white solid.

Carbonylation. A flame-dried 8 mL glass vial equipped with a stir bar and septum was charged with vinyl iodide (8.7 mg, 21.0 µmol, 1 equiv.) and $PdCl_2(PPh_3)_2$ (4.0 mg, 6.4 µmol, 0.3 equiv.). The vial was evacuated (using a needle attached to the vacuum line, pump exhaust was vented into a well-ventilated fume hood) and back filled with CO gas (needle pierced through septum fed by a CO balloon, 3x). DMF (dry, 0.25 mL), MeOH (dry, 0.25 mL) and NEt_3 (0.14 mL) were added via syringes. The reaction mixture was degassed with a CO balloon and venting needle while stirring for 15 min resulting in a homogeneous reaction mixture. The CO balloon was replaced with a fresh one and the reaction was placed into a preheated 50 °C water bath for 1.5 h. Upon full conversion, the headspace was purged with argon for 5 min. The reaction mixture was blown down using nitrogen gas and then dried with toluene azeotrope (5x). The crude mixture was purified by preparative TLC (SiO_2, 20 x 10 cm, 0.25 mm thick, load in $CHCl_3$ on long side, elute 10% EtOAc in hexanes, run 2 consecutive times). The obtained product was further purified by another round of preparative TLC (SiO_2, 10 x 10 cm, 0.25 mm thick, load in $CHCl_3$, elute 5% acetone in hexanes) affording 5.6 mg (77% yield) of **RS1131** as a colorless oil.

Characterization data for hydrazone product
Description white solid
TLC R_f = 0.04 (SiO_2, 30% EtOAc in hexanes); UV-active, stains brown in anisaldehyde

Characterization data for vinyl iodide X
Description White solid
TLC R_f = 0.81 (SiO_2, 30% EtOAc in hexanes); UV-active, stains grey in anisaldehyde
LC-MS m/z = 407.2 $[M+H]^+$ and 389.2 $[M-H_2O+H]^+$

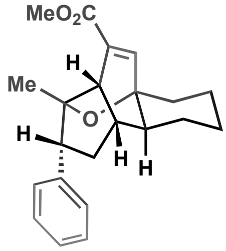

RS1131

Characterization data for **RS1131**
Description colorless oil
TLC R_f = 0.77 (SiO_2, 30% EtOAc in hexanes); UV-active, stains purple/blueish in anisaldehyde
^{1}H NMR (600 MHz, $CDCl_3$) δ 7.32 – 7.27 (m, 4H), 7.23 (d, J = 1.7 Hz, 1H), 7.21 (td, J = 5.9, 2.9 Hz, 1H), 3.82 (s, 3H), 3.22 (dd, J = 4.4, 1.8 Hz, 1H), 3.11 (dd, J = 12.0, 5.6 Hz, 1H), 2.61 (ddd, J = 13.5, 12.0, 6.3 Hz, 1H), 2.02 (dd, J = 11.8, 3.1 Hz, 1H), 1.87 (d, J = 13.0 Hz, 1H), 1.78 (dd, J = 13.6, 5.7 Hz, 1H), 1.73 – 1.69 (m, 1H), 1.69 – 1.64 (m, 2H), 1.59 – 1.46 (m, 4H), 1.31 (dt, J = 13.1, 4.2 Hz, 1H), 0.88 (s, 3H), 0.83 (qd, J = 13.2, 3.7 Hz, 1H).
^{13}C NMR (151 MHz, $CDCl_3$) δ 165.9, 145.1, 141.3, 132.7, 130.0, 128.0, 126.4, 84.7, 73.4, 55.2, 53.5, 52.0, 48.3, 40.6, 39.5, 33.4, 32.2, 25.7, 23.5, 22.9.
2D NMR HSQC
LC-MS m/z = 339.2 $[M+H]^+$ and 321.2 $[M-H_2O+H]^+$
HRMS Calculated $C_{22}H_{25}O_2$ $[M-H_2O+H]^+$ = 321.1855 | Found: 321.1852

Synthesis of RS1138

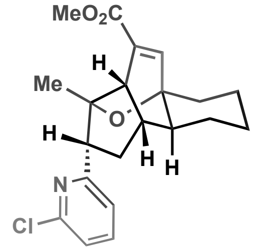

RS1138

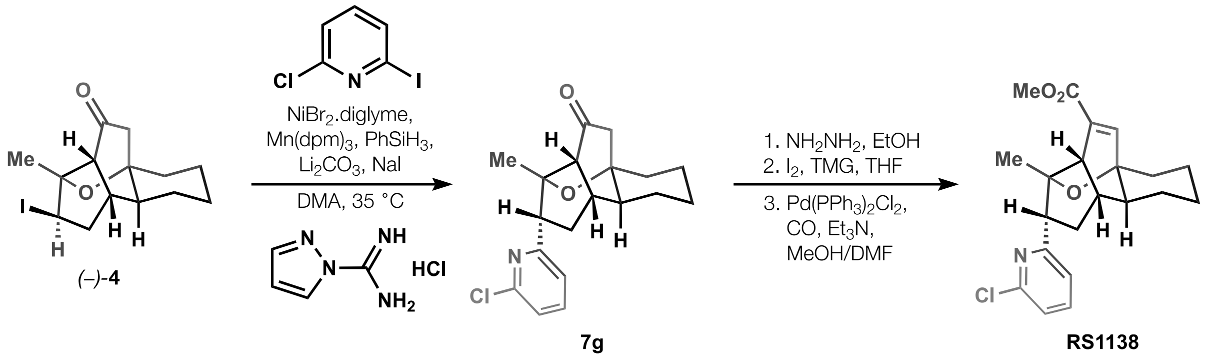

Cross-electrophile coupling (see page S21) A flame-dried 20 mL glass tube with threaded end was equipped with a stir bar and septum pierced with an needle fed by a balloon filled with argon. It was sequentially charged with praxadine.HCl (85.0 mg, 578 µmol, 2 equiv.), $NiBr_2$•diglyme (224 mg, 635 µmol, 2.2 equiv.), $Mn(dpm)_3$ (245 mg, 404 µmol, 1.4 equiv.), 2-chloro-6-iodopyridine (207 mg, 867 µmol, 3 equiv.) *(–)*-**4** (100 mg, 289 µmol, 1 equiv.), Li_2CO_3 (21.0 mg, 289 µmol, 1 equiv.), and NaI (260 mg, 1.73 mmol, 6 equiv.). The vial was evacuated and backfilled with argon (5x) and then charged with DMA (dry, 8.8 mL) using a syringe, and the reaction mixture was degassed with an argon balloon and a vent needle while stirring for 45 min. The reaction mixture turned brown at the end of the degassing period. The vent needle was removed, the septum and argon balloon replaced. Silylbenzene (125 mg, 143 µL, 578 µmol, 4 equiv.) was added using a syringe, then the reaction mixture was placed into a preheated 35 °C water bath The reaction underwent a series of changes in appearance similar to XEC with 2-iodo-6-methyl pyridine from turbid brown-yellow to turbid yellow-olive to turbid pastel green/light jade to turbid dark jade within a timespan of 15 – 30 min. The reaction was stirred for 4.5 h. Upon reaction completion (No SM mass found on LC-MS, faint SM spot remaining, dark forest green reaction mixture) EtOAc (8.8 mL) and ice-cold water (17 mL) were added. The layers were separated and the aqueous layer was extracted with EtOAc (6 x 2mL). The combined organic layers were washed with half saturated brine (2 x 8.8 mL) and brine (2 mL). The combined washes were back extracted with EtOAc (2 mL) and the back extract was washed with half saturated brine (2 x 2 mL), then combined with the organic layer, dried over $MgSO_4$, filtered and concentrated *in vacuo*. The crude product was purified by column chromatography (40 g SiO_2, load in toluene, elute 10-15-20-25-30-35-40% EtOAc in hexanes, Combiflash purification system). The product containing fractions were concentrated *in vacuo* and the diastereomers were separated by column chromatography (SiO_2, load in toluene, elute 2.5% EtOAc in hexanes) affording 17.5 mg (18.3% yield) of ketone **8g** as a colorless oil and 10.2 mg (11 % yield) of ketone **7g** as a white solid.

Hydrazone condensation. An 8 mL glass vial equipped with a stir bar was charged with EtOH (0.5 mL) and hydrazine monohydrate (48.3 mg, 47.0 µL, 964 µmol, 40 equiv.). The resulting

solution was placed in a 40 °C water bath with stirring, and a solution of ketone **7g** (8.0 mg, 24.1 μmol, 1 equiv.) in Chloroform (0.1 mL) was added. Upon full starting material consumption (1 h, monitored by TLC), the water bath was removed and the reaction mixture was blown down using nitrogen. The residue was dried with toluene/MeOH azeotrope (1:1, 1x), toluene (3x) and hexanes (3x) to afford a white solid.

Barton iodination. The crude mixture of hydrazones (assume 24.1 μmol) was charged with THF (dry, inhibitor-free, 0.1 mL) and cooled to 0 °C using an ice bath. A separate vessel was charged with iodine (12.5 mg, 49.4 μmol, 2.05 equiv.), TMG (194 mg, 212 μL, 1.69 mmol, 70 equiv.) and THF (dry, inhibitor-free, 210 μL). The iodine solution was taken up in a syringe and added dropwise to the cooled hydrazone solution giving an orange color that faded over time. Full consumption of productive intermediates was indicated once the orange color persisted and further addition of iodine solution only intensified the color and consistent with TLC data. Na_2SO_3 (saturated aqueous, 0.5 mL) was added, the ice-bath was removed, and the reaction mixture was diluted with EtOAc (1.25 mL) and minimal amount of water to dissolve all solids. The layers were separated, the aqueous layer was extracted with EtOAc (2 x 1.25 mL), the combined organic layers were washed with brine (2 mL). The brine wash was backextracted with EtOAc (3 x 2 mL). The combined organic layers were dried over $MgSO_4$, filtered and concentrated *in vacuo*. The resulting crude product was purified by column chromatography (SiO_2, load in toluene, elute 10-15-20-25% EtOAc in hexanes). The product containing fractions were concentrated *in vacuo* affording 7.7 mg (72% yield over two steps) of vinyl iodide as a white solid.

Carbonylation. A flame-dried 8 mL glass vial equipped with a stir bar and septum was charged with vinyl iodide (7.7 mg, 17.0 μmol, 1 equiv.) and $PdCl_2(PPh_3)_2$ (4.0 mg, 5.2 μmol, 0.3 equiv.). The vial was evacuated (using a needle attached to the vacuum line, pump exhaust was vented into a well-ventilated fume hood) and back filled with CO gas (needle pierced through septum fed by a CO balloon, 3x). DMF (dry, 0.25 mL), MeOH (dry, 0.25 mL) and NEt_3 (0.14 mL) were added via syringes. The reaction mixture was degassed with a CO balloon and venting needle while stirring for 15 min resulting in a homogeneous reaction mixture. The CO balloon was replaced with a fresh one and the reaction was placed into a preheated 50 °C water bath for 2 h. Upon full conversion, the headspace was purged with argon for 5 min. The reaction mixture was blown down using nitrogen gas and then dried with toluene azeotrope (5x). The crude mixture was purified by preparative TLC (SiO_2, 20 x 10 cm, 0.25 mm thick, load in $CHCl_3$ on long side, elute 10% EtOAc in hexanes, run 2 consecutive times). The obtained product was further purified by another round of preparative TLC (SiO_2, 10 x 10 cm, 0.25 mm thick, load in $CHCl_3$, elute 5% acetone in hexanes) affording 4.6 mg (71% yield) of **RS1138** as a white solid.

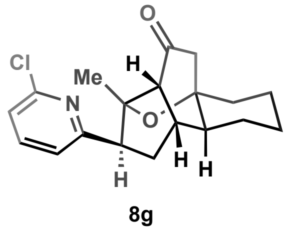

8g

Characterization data for ketone **8g**

Description colorless oil

TLC R_f = 0.46 (SiO_2, 30% EtOAc in hexanes); UV-active, stains grey in anisaldehyde

^{1}H NMR (600 MHz, $CDCl_3$) δ 7.52 (t, *J* = 7.7 Hz, 1H), 7.12 (dd, *J* = 8.0, 0.8 Hz, 1H), 7.08 (dd, *J* = 7.5, 0.8 Hz, 1H), 3.46 (t, *J* = 7.8 Hz, 1H), 2.75 (d, *J* = 3.9 Hz, 1H), 2.66 (d, *J* = 19.2

Hz, 1H), 2.47 (ddd, J = 12.5, 7.4, 5.1 Hz, 1H), 2.21 – 2.12 (m, 2H), 1.98 (dd, J = 19.2, 1.6 Hz, 1H), 1.78 – 1.70 (m, 4H), 1.57 (ddt, J = 13.2, 4.1, 1.9 Hz, 1H), 1.53 – 1.44 (m, 1H), 1.39 – 1.24 (m, 2H), 1.16 (qd, J = 13.3, 3.7 Hz, 1H), 0.83 (s, 3H).
^{13}C NMR (151 MHz, $CDCl_3$) δ 213.1, 163.3, 151.3, 138.7, 123.0, 122.2, 87.4, 75.2, 58.5, 58.4, 50.0, 42.8, 42.8, 41.4, 36.2, 30.6, 25.8, 23.8, 23.0.
2D NMR COSY, HSQC, HMBC, NOESY
LC-MS m/z = 332.2 and 334.2 $[M+H]^+$

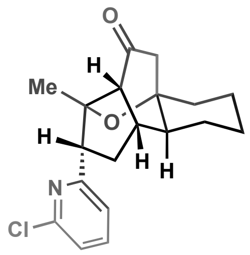

7g

Characterization data for ketone **7g**
Description white solid
TLC 0.39 (SiO_2, 30% EtOAc in hexanes); UV-active, stains grey in anisaldehyde
^{1}H NMR (600 MHz, $CDCl_3$) δ 7.56 (t, J = 7.7 Hz, 1H), 7.19 – 7.16 (m, 2H), 3.34 – 3.27 (m, 1H), 2.68 (d, J = 19.3 Hz, 1H), 2.43 – 2.38 (m, 2H), 2.38 – 2.30 (m, 2H), 2.14 (tt, J = 3.9, 1.7 Hz, 1H), 1.94 (dd, J = 19.3, 1.6 Hz, 1H), 1.76 – 1.70 (m, 3H), 1.61 – 1.58 (m, 1H), 1.44 – 1.26 (m, 6H), 1.20 – 1.07 (m, 1H).
^{13}C NMR (151 MHz, $CDCl_3$) δ 213.1, 160.3, 150.5, 138.4, 122.4, 122.0, 85.8, 74.9, 61.4, 55.7, 48.2, 43.4, 43.3, 39.0, 35.2, 30.6, 25.9, 25.3, 23.1.
2D NMR COSY, HSQC, HMBC, NOESY
LC-MS m/z = 332.2 and 334.2 $[M+H]^+$

Characterization data for hydrazone product
Description white solid
TLC R_f = 0.52 (SiO_2, 30% acetone in hexanes); UV-active, stains yellow in anisaldehyde

Characterization data for vinyl iodide X
Description White solid
TLC R_f = 0.77 (SiO_2, 30% EtOAc in hexanes), 0.72 (SiO_2, 30% acetone in hexanes); UV-active, stains green in anisaldehyde
LC-MS (no ionization observed)

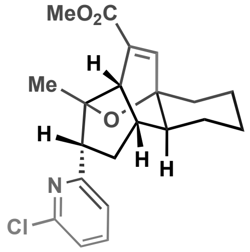

RS1138

Characterization data for methyl enoate **RS1138**
Description White solid
TLC R_f = 0.65 (SiO_2, 30% EtOAc in hexanes); UV-active, stains green in anisaldehyde
^{1}H NMR (600 MHz, $CDCl_3$) δ 7.53 (t, J = 7.7 Hz, 1H), 7.25 (d, J = 1.7 Hz, 1H), 7.20 (dd, J =

7.6, 0.8 Hz, 1H), 7.14 (dd, *J* = 7.9, 0.8 Hz, 1H), 3.81 (s, 3H), 3.38 (dd, *J* = 11.6, 4.4 Hz, 1H), 3.20 (dd, *J* = 4.1, 1.8 Hz, 1H), 2.39 (ddd, *J* = 13.2, 11.7, 5.9 Hz, 1H), 2.14 (dd, *J* = 13.2, 4.4 Hz, 1H), 2.07 (ddd, *J* = 13.4, 4.0, 2.2 Hz, 1H), 1.91 (dd, *J* = 12.1, 3.5 Hz, 1H), 1.88 – 1.80 (m, 1H), 1.68 – 1.61 (m, 2H), 1.54 – 1.44 (m, 2H), 1.39 (ddd, *J* = 13.6, 12.1, 4.0 Hz, 1H), 1.30 (dddd, *J* = 19.4, 15.4, 9.7, 5.2 Hz, 1H), 1.01 (s, 3H), 0.77 (qd, *J* = 13.1, 3.7 Hz, 1H).
^{13}C NMR (151 MHz, $CDCl_3$) δ 165.9, 162.2, 150.3, 146.0, 138.2, 132.4, 122.2, 121.7, 84.8, 73.8, 54.7, 52.0, 51.6, 48.4, 40.5, 37.0, 33.0, 32.3, 25.7, 23.7, 23.5.
2D NMR HSQC
LC-MS m/z = 374.2 and 376.2 $[M+H]^+$
HRMS Calculated $C_{21}H_{25}{}^{35}ClNO_3$ $[M+H]^+$ = 374.1523 | Found: 374.1516

Synthesis of RS1122 and RS1123

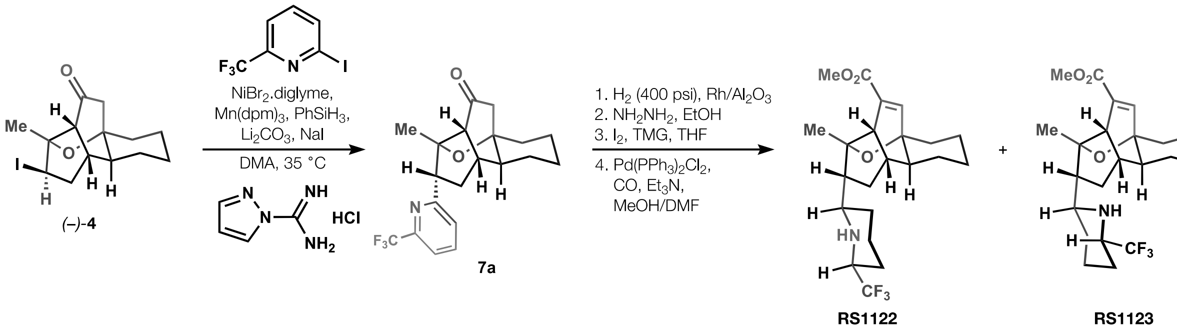

Cross electrophile coupling (see page S67)

Ketone **7a** (10 mg, 0.027 mmol) and Rh/Al_2O_3 (5%, Oakwood, batch #104864N19W) (40 mg) were charged to a test tube (Kimble 73500-1275, 6 mL capacity, 75 mm x 12 mm) and CH_2Cl_2 (2 mL) was added. The reaction tube was subjected to hydrogenation in a Freeslate Junior OSR (Unchained Labs) apparatus (600 rpm, 25 °C, 395-405 H_2 pressure deadband range) for 18 h. The reaction mixture was slurried briefly with celite (100 mg) and filtered through 10% deactivated basic alumina pre-equilibrated with EtOAc. The eluate was concentrated to afford a mixture of crude CF_3-piperidine diastereomers (R_f = 0.61 (major), 0.74 (minor) (50% EtOAc/CH_2Cl_2, UV-inactive, stains purple in anisaldehyde); major, slow-moving spot is copolar with starting material (black-grey in anisaldehyde, UV-active); 2.3:1 dr by NMR analysis; $\delta(^1H)$ (400 MHz, $CDCl_3$) 2.19 (d, J = 3.8 Hz, 1H, minor), 2.17 (d, J = 3.8 Hz, 1H, major); $\delta(^{19}F)$ (376 MHz, $CDCl_3$) -77.4 (d, J = 6.8 Hz, minor), -77.8 (d, J = 6.8 Hz, major)) which was used for the next reaction without further purification.

The crude residue from the previous step was transferred to a 1 dram vial with minimal CH_2Cl_2 and concentrated to dryness, then dissolved in $CHCl_3$ (50 µL) and EtOH (500 µL) (note: residue completely dissolved after stirring at 40 °C for 2 min). Hydrazine hydrate (49 µL, 1 mmol) was added. The vial was sealed with a solid PTFE-lined screw cap (Chemglass CG-4911-10) and placed in a 40 °C heating block for 14 h. The reaction was blown down under nitrogen and dried with copious PhMe/MeOH azeotrope until all hydrazine was removed (4x or more), then hexanes (3x) giving a foamy residue upon subjection to high vacuum. The mixture of crude hydrazones thus obtained was used for the next reaction without further purification.

To a flamed culture tube (Fisherbrand 14-957-76A, 100 mm length, 8 mL volume) under Ar with septum-lined screw cap (Chemglass CG-4910-15) was added iodine (15.0 mg, 0.060 mmol) and anhydrous, inhibitor-free THF (55 µL). The resulting dark red-brown solution was placed in an ice bath and 1,1,3,3-tetramethylguanidine (241 µL, 1.92 mmol) was added dropwise. A solution of the mixture of crude hydrazones from the previous reaction in THF (300 µL) was added dropwise, quantitating the transfer with THF (2 x 300 µL). Upon complete transfer of the mixture of crude hydrazones, the reaction turned clear yellow, and the ice bath was removed. After stirring for 90 min, additional iodine (50 µL of a solution containing iodine (7 mg) in THF (200 µL)) was added. After an additional 20 min stirring, minimal change in TLC profile was observed, and the reaction was quenched with a mixture of saturated Na_2SO_3 (3 mL) and saturated Na_2CO_3 (1 mL). The mixture was extracted with EtOAc (3x) and the combined organics were dried over Na_2SO_4 and concentrated under reduced pressure giving a crude residue (90 mg) containing significant tetramethylguanidine. The crude residue was loaded with minimal CH_2Cl_2 onto a silica plug (3 cm x 15 mm) pre-equilibrated with 20% EtOAc/hexanes, and eluted with 20% EtOAc/hexanes to afford a crude mixture of vinyl iodide

diastereomers (R_f = 0.67 (major), 0.78 (minor) (20% EtOAc/hexanes, UV-active, stains with anisaldehyde); 2.3:1 dr by NMR analysis; δ(^{1}H) (600 MHz, $CDCl_3$) 6.82 (d, J = 1.9 Hz, 1H, major), 6.74 (d, J = 1.9 Hz, 1H, minor); δ(^{19}F) (376 MHz, $CDCl_3$) -80.2 (s, minor), -80.6 (s, major)) which was used for the next reaction without further purification. Additional purification at this stage may simplify purification in the next step.

The crude vinyl iodides were transferred to a culture tube (Fisherbrand 14-957-76A, 100 mm length, 8 mL volume) with minimal CH_2Cl_2 and dried with PhMe azeotrope (2x). An egg-shaped stir bar (should span most of the inner diameter of tube for optimal stirring) and $Pd(PPh_3)_2Cl_2$ (1.0 mg, 0.0014 mmol) was added and the headspace was purged with CO (caution: toxic! perform all procedures with CO in a well-ventilated fume hood) from a 3-layer balloon equipped with a 22G 3'' needle (3x evacuate/backfill cycles). Anhydrous DMF (pre-sparged with CO for 20 min, 300 μL) was added and the mixture was slurried. In rapid succession, MeOH (pre-sparged with CO for 20 min, 300 μL) and Et_3N (100 μL) were added and the reaction headspace was purged with CO (20G vent needle) with rapid stirring. After 10 min, the vent needle was removed and the reaction tube was placed in a 40 °C heating block and stirred at 1200 rpm under CO. After 30 min at this temperature, the stir plate heating the block was set to 50 °C. A dark magenta, slightly purple solution was observed. After an additional 30 min at 50 °C, TLC analysis indicated complete conversion of starting material. The reaction mixture was concentrated to dryness under a stream of nitrogen, and then dried with PhMe azeotrope. The crude residue was loaded with minimal PhMe/a few drops of CH_2Cl_2 onto a silica plug (3 cm x 15 mm) pre-equilibrated with 50% EtOAc/hexanes, then eluted with 50% EtOAc/hexanes. The eluate was concentrated and further purified by 3 rounds of preparative TLC (round 1: SiO_2, 5% EtOAc/hexanes x 2 passes; round 2: SiO_2, 3% (7N NH_3/MeOH)/(10% Et_2O/hexanes); round 3: 1:4:5 $Et_2O/CHCl_3$/hexanes x 2 passes). The product-containing bands (see TLC data below) were separately collected and individually subjected to further column chromatography (minor, fast-moving diastereomer A: SiO_2, 3% (7N NH_3/MeOH)/(10% Et_2O/hexanes); major, slow-moving diastereomer B: SiO_2, 3.2% (7N NH_3/MeOH)/(10% Et_2O/hexanes)) to afford diastereomer A **RS1122** (2.3 mg, 21%), diastereomer B **RS1123** (1.3 mg, 12%) with 5.4 mg of diastereomers A and B in mixed fractions (mostly diastereomer B). A small portion of diastereomer A was crystallized (see description below) to assign its relative stereochemistry (same as GB18); therefore, the relative stereochemistry of diastereomer B was assigned by analogy to that observed in the heteroarene hydrogenation to synthesise GB18 (same as 2-*epi*-6-*epi*-GB18; see ref. S1. NOESY spectra are provided for each diastereomer.

Characterization data for **RS1122**

Description colorless film; or, crystallized by slow evaporation from 2 drops of C_6H_6/3 μL Bu_2O to give a single big chunky crystal

TLC R_f = 0.45 (SiO_2, 1:4:5 $Et_2O/CHCl_3$/hexanes); UV-active, stains blue-grey in anisaldehyde

optical rotation $[\alpha]_D^{24.6}$= +24.6 (c = 0.057, CH_2Cl_2).

^{1}H NMR (600 MHz, $CDCl_3$) δ 7.18 (s, 1H), 3.79 (s, 3H), 3.21 – 3.12 (m, 1H), 3.07 (bs, 1H), 2.98 (d, J = 3.3 Hz, 1H), 2.93 (d, J = 11.2 Hz, 1H), 1.99 – 1.88 (m, 3H), 1.88 – 1.78 (m, 3H), 1.78 – 1.73 (m, 1H), 1.60 (m, 2H), 1.52 – 1.48 (m, 1H), 1.48 – 1.39 (m, 3H), 1.39 – 1.32 (m, 3H), 1.25 – 1.12 (m, 2H), 0.97 (s, 3H), 0.69 (qd, J = 13.1, 3.6 Hz, 1H).

^{13}C NMR (151 MHz, $CDCl_3$) δ 165.9, 144.7, 132.5, 126.4 (q, J = 280 Hz), 85.0, 73.7, 57.8 (q, J = 28 Hz), 54.6, 52.0, 51.9, 51.4, 48.0, 39.4, 33.2, 32.3, 31.7, 31.6, 25.6, 24.6, 23.64, 23.60, 23.4.

^{19}F NMR (376 MHz, $CDCl_3$) δ -80.2.

2D NMR HSQC, HMBC, COSY, NOESY

HRMS Calculated $C_{22}H_{31}F_3NO_3$ $[M+H]^+$ 414.2256 | Found: 414.2254

X-ray see above (CCDC 2248063)

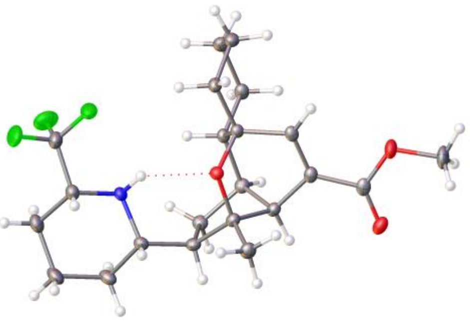

<u>Characterization data for **RS1123**</u>

Description colorless film

TLC R_f = 0.21 (UV-active, stains blue-grey in anisaldehyde)

optical rotation $[\alpha]_D^{23.7} = -20.9$ (c = 0.067, CH_2Cl_2).

^{1}H NMR (600 MHz, $CDCl_3$) δ 7.25 (d, J = 1.7 Hz, 1H), 3.79 (s, 3H), 3.08 – 3.00 (m, 1H), 2.97 (dd, J = 4.0, 1.7 Hz, 1H), 2.73 (bs, 1H), 2.60 – 2.52 (m, 1H), 2.07 – 2.00 (m, 2H), 1.94 – 1.86 (m, 2H), 1.86 – 1.74 (m, 3H), 1.65 – 1.59 (m, 1H), 1.54 – 1.40 (m, 4H), 1.40 – 1.28 (m, 4H), 1.23 – 1.09 (m, 2H), 1.08 (s, 3H), 0.71 (qd, J = 13.2, 3.7 Hz, 1H).

^{13}C NMR (151 MHz, $CDCl_3$) δ 165.9, 145.8, 132.5, 126.1 (q, J = 277 Hz), 83.2, 73.6, 60.6, 58.6 (q, J = 28 Hz), 52.3, 52.0 (CH_3 + CH), 48.7, 40.2, 35.7, 33.5, 32.4, 31.0, 26.6, 25.7, 24.6, 23.5, 23.4.

^{19}F NMR (376 MHz, $CDCl_3$) δ -80.6.

2D NMR HSQC, HMBC, COSY, NOESY

HRMS Calculated $C_{22}H_{31}F_3NO_3$ $[M+H]^+$ 414.2256 | Found: 414.2254

Synthesis of RS1135

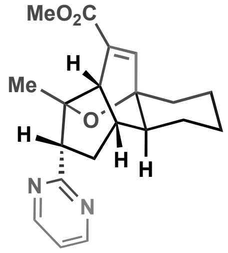

RS1135

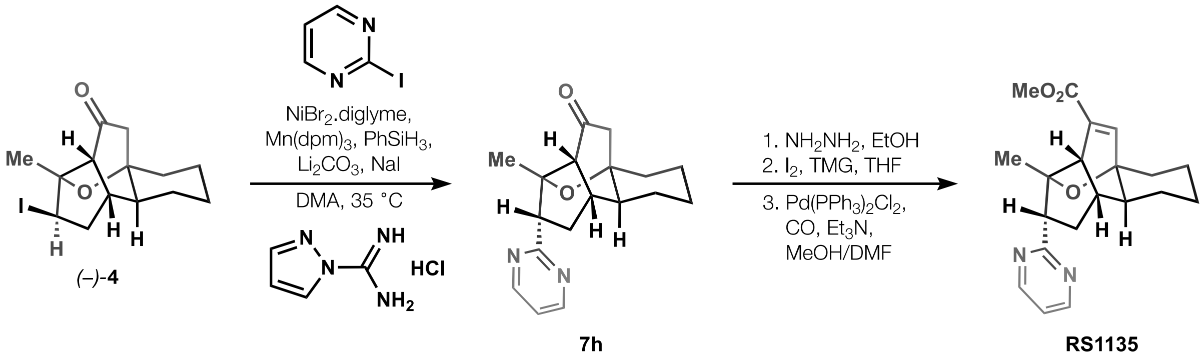

Cross-electrophile coupling (see page S21) A flame-dried 20 mL glass tube with threaded end was equipped with a stir bar and septum pierced with an needle fed by a balloon filled with argon. It was sequentially charged with praxadine.HCl (85.0 mg, 578 µmol, 2 equiv.), $NiBr_2$•diglyme (224 mg, 635 µmol, 2.2 equiv.), $Mn(dpm)_3$ (245 mg, 404 µmol, 1.4 equiv.), 2-iodopyrimidine (178 mg, 867 µmol, 3 equiv.) *(−)*-**4** (100 mg, 289 µmol, 1 equiv.), Li_2CO_3 (21.0 mg, 289 µmol, 1 equiv.), and NaI (260 mg, 1.73 mmol, 6 equiv.). The vial was evacuated and backfilled with argon (5x) and then charged with DMA (dry, 8.8 mL) using a syringe, and the reaction mixture was degassed with an argon balloon and a vent needle while stirring for 45 min. The reaction mixture turned brown at the end of the degassing period. The vent needle was removed, the septum and argon balloon replaced. Silylbenzene (125 mg, 143 µL, 578 µmol, 4 equiv.) was added using a syringe, then the reaction mixture was placed into a preheated 35 °C water bath. The reaction underwent a series of changes in appearance similar to XEC with 2-iodo-6-methyl pyridine from turbid brown-yellow to turbid yellow-olive to turbid pastel green/light jade to turbid dark jade within a timespan of 15 – 30 min. The reaction was stirred for 4 h. Upon reaction completion (No SM mass found on LC-MS, faint SM spot remaining, dark forest green colored reaction mixture) EtOAc (8.8 mL) and ice-cold water (17 mL) were added. The layers were separated and the aqueous layer was extracted with EtOAc (6 x 2mL). The combined organic layers were washed with half saturated brine (2 x 8.8 mL) and brine (2 mL). The combined washes were back extracted with EtOAc (2 mL) and the back extract was washed with half saturated brine (2 x 2 mL), then combined with the organic layer, dried over $MgSO_4$, filtered and concentrated *in vacuo*. The crude product was purified by column chromatography (SiO_2, load in toluene, elute 10-15-20-25-30-35-40% EtOAc in hexanes). The product containing fractions were concentrated *in vacuo* and the product was further purified by another round of column chromatography (SiO_2, load in toluene, elute 25% acetone in hexanes) affording 7 mg (8% yield) of ketone **7h** as a white solid (see note).

Hydrazone condensation. An 8 mL glass vial equipped with a stir bar was charged with EtOH (0.5 mL) and hydrazine monohydrate (40.3 mg, 39.2 µL, 804 µmol, 40 equiv.). The resulting solution was placed in a 40 °C water bath with stirring, and a solution of ketone **7h** (6.0 mg,

20.1 μmol, 1 equiv.) in Chloroform (0.1 mL) was added. Upon full starting material consumption (1 h, monitored by TLC), the water bath was removed and the reaction mixture was blown down using nitrogen. The residue was dried with toluene/MeOH azeotrope (1:1, 1x), toluene (3x) and hexanes (3x) to afford a white solid.

Barton iodination. The crude mixture of hydrazones (assume 20.1 μmol) was charged with THF (dry, inhibitor-free, 0.1 mL) and cooled to 0 °C using an ice bath. A separate vessel was charged with iodine (10.0 mg, 41.2 μmol, 2.05 equiv.), TMG (162 mg, 177 μL, 1.41 mmol, 70 equiv.) and THF (dry, inhibitor-free, 200 μL). The iodine solution was taken up in a syringe and added dropwise to the cooled hydrazone solution giving an orange color that faded over time. Full consumption of productive intermediates was indicated once the orange color persisted and further addition of iodine solution only intensified the color and consistent with TLC data. Na_2SO_3 (saturated aqueous, 0.5 mL) was added, the ice-bath was removed, and the reaction mixture was diluted with EtOAc (1.25 mL) and minimal amount of water to dissolve all solids. The layers were separated, the aqueous layer was extracted with EtOAc (2 x 1.25 mL), the combined organic layers were washed with brine (2 mL), The brine wash was backextracted with EtOAc (3 x 2 mL). The combined organic layers were dried over $MgSO_4$, filtered and concentrated *in vacuo*. The resulting crude product was purified by column chromatography (SiO_2, load in toluene, elute 10-15-20-25% acetone in hexanes). The product containing fractions were concentrated *in vacuo* affording 4.2 mg (51% yield over two steps) of vinyl iodide as a colorless oil.

Carbonylation. A flame-dried 8 mL glass vial equipped with a stir bar and septum was charged with vinyl iodide (4.2 mg, 10.0 μmol, 1 equiv.) and $PdCl_2(PPh_3)_2$ (2.0 mg, 3.1 μmol, 0.3 equiv.). The vial was evacuated (using a needle attached to the vacuum line, pump exhaust was vented into a well-ventilated fume hood) and back filled with CO gas (needle pierced through septum fed by a CO balloon, 3x). DMF (dry, 0.25 mL), MeOH (dry, 0.25 mL) and NEt_3 (0.14 mL) were added via syringes. The reaction mixture was degassed with a CO balloon and venting needle while stirring for 15 min resulting in a homogeneous reaction mixture. The CO balloon was replaced with a fresh one and the reaction was placed into a preheated 50 °C water bath for 2 h. Upon full conversion, the headspace was purged with argon for 5 min. The reaction mixture was blown down using nitrogen gas and then dried with toluene azeotrope (5x). The crude mixture was purified by preparative TLC (SiO_2, 10 x 10 cm, 0.25 mm thick, load in $CHCl_3$, elute 20% EtOAc in hexanes, run 2 consecutive times). The obtained product was further purified by another round of preparative TLC (SiO_2, 10 x 10 cm, 0.25 mm thick, load in $CHCl_3$, elute 10% acetone in hexanes) affording 2.3 mg (66% yield) of **RS1135** as a white solid.

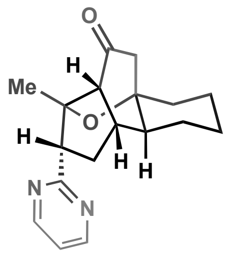

7h

Characterization data for ketone **7h**

Description White solid

TLC R_f = 0.21 (SiO_2, 20% acetone in hexanes)

^{1}H NMR (600 MHz, $CDCl_3$) δ 8.71 (d, *J* = 4.8 Hz, 2H), 7.14 (t, *J* = 4.9 Hz, 1H), 3.59 (dd, *J* =

11.3, 3.3 Hz, 1H), 2.74 (dd, J = 12.7, 3.3 Hz, 1H), 2.64 (d, J = 19.3 Hz, 1H), 2.42 (d, J = 3.8 Hz, 1H), 2.36 (ddt, J = 13.5, 4.0, 1.9 Hz, 1H), 2.20 (ddd, J = 12.7, 11.2, 5.3 Hz, 1H), 2.16 – 2.12 (m, 1H), 1.91 (dd, J = 19.2, 1.6 Hz, 1H), 1.77 – 1.67 (m, 2H), 1.66 – 1.60 (m, 1H), 1.52 (s, 3H), 1.49 (dt, J = 13.0, 3.2 Hz, 1H), 1.36 – 1.21 (m, 2H), 1.16 – 1.05 (m, 2H).
^{13}C NMR (151 MHz, $CDCl_3$) δ 213.3, 169.3, 156.4, 118.8, 87.0, 74.7, 61.6, 56.9, 47.0, 43.6, 43.3, 36.4, 35.2, 30.6, 26.0, 25.7, 23.0.
2D NMR COSY, HSQC, HMBC, NOESY
LC-MS m/z = 299.2 $[M+H]^+$

<u>Characterization data for hydrazone product</u>
Description white solid
TLC R_f = 0.54 (SiO_2, 50% acetone in hexanes); UV-active, stains yellow in anisaldehyde

<u>Characterization data for vinyl iodide</u>
Description Colorless oil
TLC R_f = 0.63(SiO_2, 30% acetone in hexanes); UV-active, stains brown in anisaldehyde
LC-MS m/z = 409.2 $[M+H]^+$

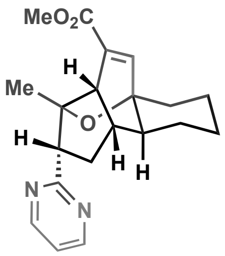

RS1135

<u>Characterization data for methyl enoate **RS1135**</u>
Description White solid
TLC R_f = 0.48 (SiO_2, 30% acetone in hexanes); UV-active, stains brown in anisaldehyde
^{1}H NMR (600 MHz, $CDCl_3$) δ 8.68 (d, J = 4.9 Hz, 2H), 7.23 (d, J = 1.7 Hz, 1H), 7.11 (t, J = 4.9 Hz, 1H), 3.81 (s, 3H), 3.64 (dd, J = 11.3, 3.5 Hz, 1H), 3.21 (dd, J = 4.0, 1.7 Hz, 1H), 2.59 (dd, J = 12.8, 3.5 Hz, 1H), 2.26 – 2.16 (m, 1H), 2.13 (dt, J = 13.0, 3.2 Hz, 1H), 1.80 (dt, J = 12.2, 3.4 Hz, 1H), 1.78 – 1.73 (m, 1H), 1.67 – 1.62 (m, 1H), 1.55 – 1.49 (m, 1H), 1.44 (qt, J = 13.7, 3.8 Hz, 1H), 1.31 – 1.20 (m, 4H), 1.15 – 1.07 (m, 1H), 0.73 (qd, J = 13.2, 3.7 Hz, 1H).
^{13}C NMR (151 MHz, $CDCl_3$) δ 170.3, 165.9, 156.3 (2C), 146.2, 132.5, 118.6, 85.6, 73.6, 55.8, 52.0, 49.6, 48.6, 41.1, 33.9, 33.0, 32.3, 25.6, 24.4, 23.4.
2D NMR HSQC
LC-MS m/z = 341.2 $[M+H]^+$
HRMS Calculated $C_{20}H_{25}N_2O_3$ $[M+H]^+$ = 341.1865 | Found: 341.1862

Synthesis of RS1134

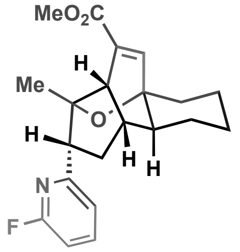

RS1134

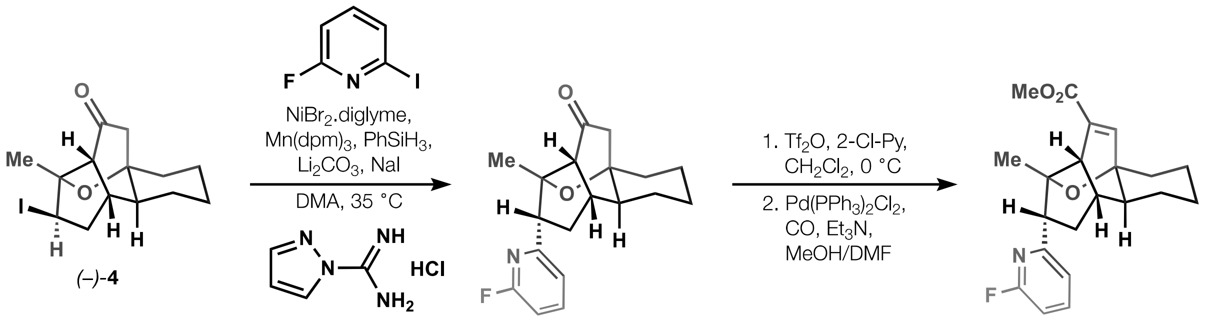

Cross-electrophile coupling (see page S21) A flame-dried 20 mL glass tube with threaded end was equipped with a stir bar and septum pierced with an needle fed by a balloon filled with argon. It was sequentially charged with praxadine.HCl (85.0 mg, 578 µmol, 2 equiv.), $NiBr_2$•diglyme (224 mg, 635 µmol, 2.2 equiv.), $Mn(dpm)_3$ (245 mg, 404 µmol, 1.4 equiv.), 2-bromo-6-fluoropyridine (152 mg, 867 µmol, 3 equiv.) *(−)*-**4** (100 mg, 289 µmol, 1 equiv.), Li_2CO_3 (21.0 mg, 289 µmol, 1 equiv.), and NaI (260 mg, 1.73 mmol, 6 equiv.). The vial was evacuated and backfilled with argon (5x) and then charged with DMA (dry, 8.8 mL) using a syringe, and the reaction mixture was degassed with an argon balloon and a vent needle while stirring for 45 min. The reaction mixture turned brown at the end of the degassing period. The vent needle was removed, the septum and argon balloon replaced. Silylbenzene (125 mg, 143 µL, 578 µmol, 4 equiv.) was added using a syringe, then the reaction mixture was placed into a preheated 35 °C water bath. The reaction underwent a series of changes in appearance similar to XEC with 2-iodo-6-methyl pyridine from turbid brown-yellow to turbid yellow-olive to turbid pastel green/light jade to turbid dark jade within a timespan of 15 – 30 min. The reaction was stirred for 5 h. Upon reaction completion (No SM mass found on LC-MS, faint SM spot remaining, dark forest green reaction mixture) EtOAc (8.8 mL) and ice-cold water (17 mL) were added. The layers were separated and the aqueous layer was extracted with EtOAc (6 x 2mL). The combined organic layers were washed with half saturated brine (2 x 8.8 mL) and brine (2 mL). The combined washes were back extracted with EtOAc (2 mL) and the back extract was washed with half saturated brine (2 x 2 mL), then combined with the organic layer, dried over $MgSO_4$, filtered and concentrated *in vacuo*. The crude product was purified by column chromatography (40 g SiO_2, load in toluene, elute 10-15-20-25-30-35-40% EtOAc in hexanes, Combiflash purification system). The diastereomers were separated by column chromatography (SiO_2, load in toluene, elute 2.5% EtOAc in DCM). Mixed fractions were concentrated *in vacuo* and the diastereomers were separated by preparative TLC (SiO_2, 20 x 10 cm, 0.25 mm thick, load in $CHCl_3$ on long side, elute 5% EtOAc in DCM, run two consecutive times) in total affording 40.5 mg (45% yield) of ketone **8i** as white crystals and 20.4 mg (23% yield) of ketone **7i** as white crystals.

Triflation. A flame-dried 8 mL vial equipped with a stir bar and septum pierced with an argon balloon was charged with ketone **7i** (10 mg, 31.7 µmol, 1 equiv.), DCM (dry, 200 µL) and 2-chloropyridine (50 mg, 42 µL, 0.44 mmol, 14 equiv.). The reaction mixture was cooled to 0 °C using an ice bath then triflic anhydride (50 µL) was added dropwise. Upon complete addition (~5 min), the ice bath was removed, stirring was stopped and the reaction mixture was allowed to warm to room temperature. The stirring was occasionally turned on (300 rpm) for 5 sec. Upon completion (1 h), the reaction mixture was cooled to 0°C using an ice bath, $NaHCO_3$ (saturated aqueous 3 mL) and Na_2SO_3 (saturated aqueous, 3 mL) were added. The reaction mixture was extracted with diethyl ether (3 x 3 mL) and the combined organic layers were washed with water (3 x 2 mL), brine (2 mL), dried over $MgSO_4$, filtered and concentrated *in vacuo*. The crude product was purified by column chromatography (SiO_2, load in toluene, elute 5-10-15-20% EtOAc in hexanes) affording 7.3 mg (51% yield) of vinyl triflate as a white solid.

Carbonylation. A flame-dried 8 mL glass vial equipped with a stir bar and septum was charged with vinyl triflate (7.3 mg, 16.0 µmol, 1 equiv.) and $PdCl_2(PPh_3)_2$ (3.0 mg, 4.9 µmol, 0.3 equiv.). The vial was evacuated (using a needle attached to the vacuum line, pump exhaust was vented into a well-ventilated fume hood) and back filled with CO gas (needle pierced through septum fed by a CO balloon, 3x). DMF (dry, 0.25 mL), MeOH (dry, 0.25 mL) and NEt_3 (0.14 mL) were added via syringes. The reaction mixture was degassed with a CO balloon and venting needle while stirring for 15 min resulting in a homogeneous reaction mixture. The CO balloon was replaced with a fresh one and the reaction was placed into a preheated 50 °C water bath for 2 h. Upon full conversion, the headspace was purged with argon for 5 min. The reaction mixture was blown down using nitrogen gas and then dried with toluene azeotrope (5x). The crude mixture was purified by preparative TLC (SiO_2, 10 x 10 cm, 0.25 mm thick, load in $CHCl_3$, elute 10% EtOAc in hexanes, run 3 consecutive times). The obtained product was further purified by another round of preparative TLC (SiO_2, 10 x 10 cm, 0.25 mm thick, load in $CHCl_3$, elute 10% acetone in hexanes, run 2 consecutive times) affording 4.0 mg (69% yield) of **RS1134** as a colorless oil.

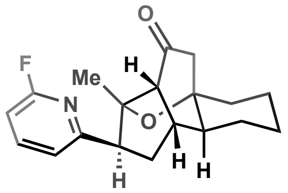

8i

Characterization data for ketone **8i**

Description White crystals

TLC R_f = 0.45 (SiO_2, 30% EtOAc in hexanes); UV-active, stains blue in anisaldehyde

^{1}H NMR (600 MHz, $CDCl_3$) δ 7.65 (q, J = 8.0 Hz, 1H), 7.05 (dd, J = 7.4, 2.3 Hz, 1H), 6.72 (dd, J = 8.2, 2.9 Hz, 1H), 3.46 (t, J = 7.8 Hz, 1H), 2.74 (d, J = 3.9 Hz, 1H), 2.66 (d, J = 19.2 Hz, 1H), 2.44 (ddd, J = 12.5, 7.4, 5.2 Hz, 1H), 2.19 – 2.11 (m, 2H), 1.97 (dd, J = 19.2, 1.7 Hz, 1H), 1.77 – 1.70 (m, 4H), 1.56 (ddt, J = 13.1, 4.0, 1.9 Hz, 1H), 1.48 (td, J = 12.4, 3.8 Hz, 1H), 1.39 – 1.24 (m, 2H), 1.15 (qd, J = 13.2, 3.7 Hz, 1H), 0.82 (s, 3H).

^{13}C NMR (151 MHz, $CDCl_3$) δ 212.88, 163.4 (d, $^1J_{CF}$ = 238.9 Hz), 161.4 (d, $^3J_{CF}$ = 12.5 Hz), 141.1 (d, $^3J_{CF}$ = 7.7 Hz), 121.9 (d, $^4J_{CF}$ = 4.0 Hz), 107.4 (d, $^2J_{CF}$ = 37.8 Hz), 87.31, 75.17, 58.50, 58.02, 50.03, 42.76, 42.71, 41.25, 36.19, 30.60, 25.82, 23.61, 22.99.

^{19}F NMR (376 MHz, $CDCl_3$) δ -69.06.

2D NMR COSY, HSQC, HMBC, NOESY

LC-MS m/z = 366.2 $[M+H]^+$

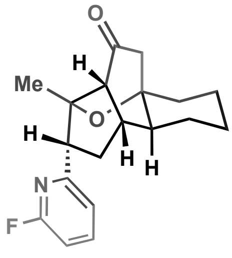

7i

Characterization data for ketone **7i**
Description White crystals
TLC R_f = 0.35 (SiO_2, 30% EtOAc in hexanes); UV-active, stains blue in anisaldehyde
^{1}H NMR (600 MHz, $CDCl_3$) δ 7.70 (q, J = 7.9 Hz, 1H), 7.14 (dd, J = 7.4, 2.4 Hz, 1H), 6.77 (dd, J = 8.2, 3.0 Hz, 1H), 3.28 (dd, J = 8.8, 6.8 Hz, 1H), 2.68 (d, J = 19.3 Hz, 1H), 2.41 (d, J = 3.9 Hz, 1H), 2.36 – 2.31 (m, 3H), 1.94 (dd, J = 19.2, 1.6 Hz, 1H), 1.72 (m, 3H), 1.58 (dt, J = 12.4, 3.1 Hz, 1H), 1.42 – 1.27 (m, 6H), 1.13 (qd, J = 12.8, 3.4 Hz, 1H).
^{13}C NMR (151 MHz, $CDCl_3$) δ 213.1, 162.9 (d, $^1J_{CF}$ = 236.7 Hz), 158.7 (d, $^3J_{CF}$ = 13.3 Hz), 140.6 (d, $^3J_{CF}$ = 7.5 Hz), 121.3 (d, $^4J_{CF}$ = 4.2 Hz), 107.0 (d, $^2J_{CF}$ = 37.9 Hz), 85.8, 74.8, 61.4, 55.4, 48.4, 43.3, 43.2, 38.7, 35.2, 30.5, 25.7, 25.3, 23.1.
^{19}F NMR (376 MHz, $CDCl_3$) δ -70.52.
2D NMR COSY, HSQC, HMBC, NOESY
LC-MS m/z = 366.2 $[M+H]^+$

Characterization data for vinyl triflate
Description White solid
TLC R_f = 0.72 (SiO_2, 30% EtOAc in hexanes); UV-active, stains green in anisaldehyde
LC-MS m/z = 448.2 $[M+H]^+$

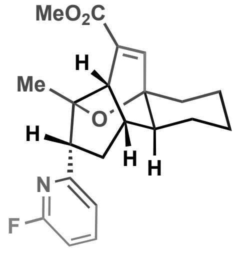

RS1134

Characterization data for **RS1134**
Description colorless oil
TLC R_f = 0.57 (SiO_2, 30% EtOAc in hexanes); UV-active, stains grey in anisaldehyde
^{1}H NMR (600 MHz, $CDCl_3$) δ 7.67 (q, J = 8.0 Hz, 1H), 7.24 (s, 1H), 7.16 (dd, J = 7.5, 2.3 Hz, 1H), 6.74 (dd, J = 8.1, 2.8 Hz, 1H), 3.81 (s, 3H), 3.35 (dd, J = 11.7, 4.4 Hz, 1H), 3.20 (dd, J = 4.2, 1.9 Hz, 1H), 2.38 (td, J = 12.5, 5.8 Hz, 1H), 2.17 (dd, J = 13.1, 4.5 Hz, 1H), 2.03 (dt, J = 12.7, 3.0 Hz, 1H), 1.90 (d, J = 11.8 Hz, 1H), 1.83 (d, J = 14.2 Hz, 1H), 1.67 – 1.60 (m, 2H), 1.55 – 1.43 (m, 2H), 1.39 – 1.27 (m, 2H), 1.03 (s, 3H), 0.77 (qd, J = 13.2, 3.6 Hz, 1H).
^{13}C NMR (151 MHz, $CDCl_3$) δ 165.9, 162.8 (d, $^1J_{CF}$ = 236.2 Hz), 160.5 (d, $^3J_{CF}$ = 13.2 Hz) 145.9, 140.5 (d, $^3J_{CF}$ = 7.6 Hz), 132.4, 121.1 (d, $^4J_{CF}$ = 4.0 Hz), 106.7 (d, $^2J_{CF}$ = 37.9 Hz) 84.9, 73.7, 54.4, 52.0, 51.7, 48.4, 40.4, 36.6, 33.0, 32.3, 25.6, 23.8, 23.5.
^{19}F NMR (376 MHz, $CDCl_3$) δ -70.83.
2D NMR HSQC

LC-MS m/z = 358.2 $[M+H]^+$
HRMS Calculated $C_{21}H_{25}FNO_3$ $[M+H]^+$ = 358.1818| Found: 358.1807

Synthesis of GBFZI-110

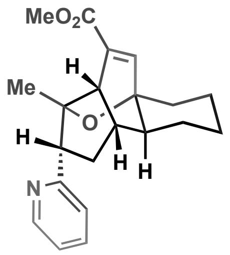

GBFZI-110

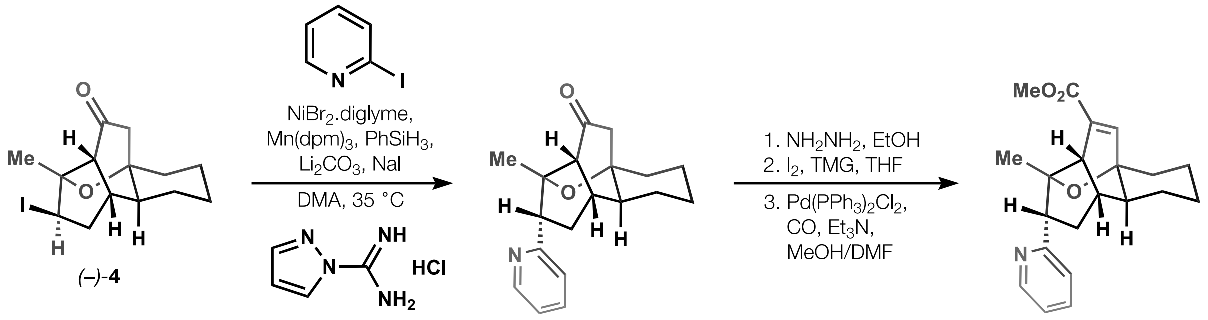

Cross-electrophile coupling (see page S21) A flame-dried 20 mL glass tube with threaded end was equipped with a stir bar and septum pierced with a needle fed by a balloon filled with argon. It was sequentially charged with praxadine.HCl (42.0 mg, 289 µmol, 2 equiv.), $NiBr_2$•diglyme (112 mg, 318 µmol, 2.2 equiv.), $Mn(dpm)_3$ (122 mg, 202 µmol, 1.4 equiv.) *(−)*-**4** (50 mg, 144 µmol, 1 equiv.), Li_2CO_3 (11.0 mg, 144 µmol, 1 equiv.), and NaI (130 mg, 867 µmol, 6 equiv.). The vial was evacuated and backfilled with argon (5x) and then charged with DMA (dry, 4.4 mL) using a syringe. 2-iodopyridine (88.8 mg, 46.0 µL, 433 µmol, 3 equiv.) were added using a micro-syringe and the reaction mixture was degassed with an argon balloon and a vent needle while stirring for 30 min. The reaction mixture turned brown at the end of the degassing period. The vent needle was removed, the septum and argon balloon replaced. Silylbenzene (62.5 mg, 71.6 µL, 578 µmol, 4 equiv.) was added using a micro-syringe, then the reaction mixture was placed into a preheated 35 °C water bath. The reaction underwent a series of changes in appearance similar to XEC with 2-iodo-6-methyl pyridine from turbid brown-yellow to turbid yellow-olive to turbid pastel green/light jade to turbid dark jade within a timespan of 15 – 30 min. The reaction was stirred for 3h. Upon reaction completion (No SM mass found on LC-MS, faint SM spot remaining, dark forest green colored reaction mixture) EtOAc (4.25 mL) and ice-cold water (8.5 mL) were added. The layers were separated and the aqueous layer was extracted with EtOAc (6 x 1mL). The combined organic layers were washed with half saturated brine (2 x 4.25 mL) and brine (1 mL). The combined washes were back extracted with EtOAc (1 mL) and the back extract was washed with half saturated brine (2 x 1 mL), then combined with the organic layer, dried over $MgSO_4$, filtered and concentrated *in vacuo*. The crude product was purified and diastereomers were separated by column chromatography (SiO_2, load in toluene, elute 10-15-20-20-20% EtOAc in hexanes).

Exo diastereomer. **8j** The product containing fractions were concentrated *in vacuo* and further purified by column chromatography (SiO_2, load in toluene, elute 10% acetone in hexanes). The product containing fractions were concentrated *in vacuo* and further purified by reverse phase preparative HPLC (dissolve in acetonitrile/methanol 1:1, elute acetonitrile/water 0 – 100% with 0.1% formic acid). The product containing fractions were combined and the acetonitrile was

removed *in vacuo*. The aqueous phase was basified with solid $NaHCO_3$ until pH 7-8 was reached, and then extracted with DCM (6 x 15 mL). The combined organic layers were washed with brine (15 mL), dried over $MgSO_4$, filtered and concentrated *in vacuo* to afford 1.0 mg (2 % yield) of ketone **8j** as a pale-yellow solid.
Endo diastereomer. **7j** The product containing fractions were concentrated *in vacuo* and further purified by column chromatography (SiO_2, load in toluene, elute 10% acetone in hexanes). The product containing fractions were concentrated *in vacuo* and further purified by reverse phase preparative HPLC (dissolve in acetonitrile/methanol 1:1, elute acetonitrile/water 0 – 100% with 0.1% formic acid). The product containing fractions were combined and the acetonitrile was removed *in vacuo*. The remaining aqueous phase was basified with solid $NaCO_3$ until pH 7-8 was reached, and then extracted with DCM (6 x 15 mL). The combined organic layers were washed with brine (15 mL), dried over $MgSO_4$, filtered and concentrated *in vacuo* to afford 17.5 mg (41% yield) of ketone **7j** as a pale-yellow oil.

Hydrazone condensation. An 8 mL glas vial equipped with a stir bar was charged with EtOH (0.5 mL) and hydrazine monohydrate (57.2 mg, 55.7 µL, 1.14 mmol, 40 equiv.). The resulting solution was placed in a 40 °C water bath with stirring, and a solution of ketone **7j** (8.0 mg, 28.6 µmol, 1 equiv.) in Chloroform (0.1 mL) was added. Upon full starting material consumption (3h, monitored by TLC), the water bath was removed and the reaction mixture was blown down using nitrogen. The residue was dried with toluene/MeOH azeotrope (1:1, 1x), toluene (3x) and hexanes (3x) to afford a white solid

Barton iodination. The crude mixture of hydrazones (assume 28.6 µmol) was charged with THF (dry, inhibitor-free, 0.1 mL) and cooled to 0 °C using an ice bath. A separate vessel was charged with iodine (15 mg, 58.6 µmol, 2.05 equiv.), TMG (230 mg, 250 µL, 2.00 mmol, 70 equiv.) and THF (dry, inhibitor-free, 250 µL). The iodine solution was taken up in a syringe and added dropwise to the cooled hydrazone solution giving an orange color that faded over time. Full consumption of productive intermediates was indicated once the orange color persisted and further addition of iodine solution only intensified the color and consistent with TLC data. Na_2SO_3 (saturated aqueous, 0.5 mL) was added, the ice-bath was removed, and the reaction mixture was diluted with EtOAc (1.25 mL) and minimal amount of water to dissolve all solids. The layers were separated, the aqueous layer was extracted with EtOAc (2 x 1.25 mL), the combined organic layers were washed with brine (2 mL), dried over $MgSO_4$, filtered and concentrated *in vacuo*. The resulting crude product was purified by column chromatography (SiO_2, load in toluene, elute 10-15-20% EtOAc in hexanes). The product containing fractions were concentrated *in vacuo* affording 6.3 mg (52% yield over two steps) of vinyl iodide as a white solid.

Carbonylation. A flame-dried 8 mL glass vial equipped with a stir bar and septum was charged with vinyl iodide (6.3 mg, 15 µmol, 1 equiv.) and $PdCl_2(PPh_3)_2$ (1.0 mg, 2 µmol, 0.13 equiv.). The vial was evacuated (using a needle attached to the vacuum line, pump exhaust was vented into a well-ventilated fume hood) and back filled with CO gas (needle pierced through septum fed by a CO balloon, 3x). DMF (dry, 0.25 mL), MeOH (dry, 0.25 mL) and NEt_3 (0.14 mL) were added via syringes. The reaction mixture was degassed with a CO balloon and venting needle while stirring for 15 min resulting in a homogeneous reaction mixture. The CO balloon was replaced with a fresh one and the reaction was placed into a preheated 50 °C water bath for 1.5 h. Upon full conversion, the headspace was purged with argon for 5 min. The reaction mixture was blown down using nitrogen gas and then dried with toluene azeotrope (5x). The crude mixture was purified by preparative TLC (SiO_2, 20 x 10 cm, 0.25 mm thick, load in $CHCl_3$ on long side, elute 20% EtOAc in hexanes). The obtained orange oil was further purified

by another round of preparative TLC (SiO_2, 20 x 20 cm, 0.25 mm thick, load in $CHCl_3$, elute 30% EtOAc in hexanes) affording 3.5 mg (67% yield) of **GBFZI-110** as a colorless oil.

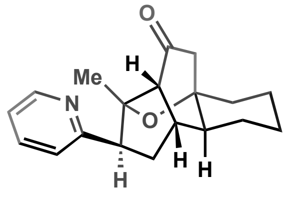

8j

<u>Characterization data for ketone **8j**</u>

Description pale-yellow solid

TLC R_f = 0.36 (SiO_2, 30% EtOAc in hexanes) UV-active, stains grey in anisaldehyde

^{1}H NMR (600 MHz, $CDCl_3$) δ 8.55 – 8.50 (m, 1H), 7.56 (td, J = 7.6, 1.9 Hz, 1H, 7.16 (d, J = 7.7 Hz, 1H), 7.09 (ddd, J = 7.7, 4.8, 1.2 Hz, 1H), 3.49 (t, J = 7.8 Hz, 1H), 2.77 (d, J = 3.9 Hz, 1H), 2.67 (d, J = 19.2 Hz, 1H), 2.54 (ddd, J = 12.5, 7.4, 5.2 Hz, 1H), 2.23 – 2.14 (m, 2H), 1.98 (dd, J = 19.2, 1.6 Hz, 1H), 1.78 – 1.71 (m, 4H), 1.67 – 1.57 (m, 1H), 1.54 – 1.46 (m, 1H), 1.39 – 1.27 (m, 2H), 1.16 (qd, J = 13.1, 3.5 Hz, 1H), 0.79 (s, 3H).

^{13}C NMR (151 MHz, $CDCl_3$) δ 213.3, 162.3, 149.7, 136.1, 124.8, 121.6, 87.5, 75.1, 58.7, 58.6, 50.1, 42.8 (2C), 41.4, 36.3, 30.7, 25.9, 23.7, 23.1.

2D-NMR COSY, HSQC, HMBC, NOESY

LC-MS 298.1 $[M+H]^+$

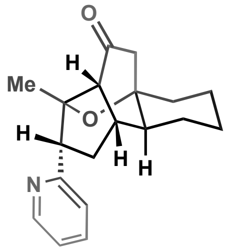

7j

<u>Characterization data for ketone **7j**</u>

Description pale yellow oil

TLC R_f = 0.18 (SiO_2, 30% EtOAc in hexanes); UV-active, stains grey with anisaldehyde

^{1}H NMR (600 MHz, $CDCl_3$) δ 8.59 – 8.55 (m, 1H), 7.62 (td, J = 7.7, 1.8 Hz, 1H), 7.33 (d, J = 7.9 Hz, 1H), 7.14 (ddd, J = 7.5, 4.9, 1.1 Hz, 1H), 3.35 (dd, J = 11.6, 4.5 Hz, 1H), 2.68 (d, J = 19.3 Hz, 1H), 2.46 – 2.38 (m, 2H), 2.32 (dd, J = 13.1, 4.5 Hz, 1H), 2.22 – 2.19 (m, 1H), 2.15 (ddd, J = 5.9, 4.0, 2.1 Hz, 1H), 1.95 (dd, J = 19.3, 1.6 Hz, 1H), 1.77 – 1.66 (m, 3H), 1.64 – 1.58 (m, 1H), 1.38 – 1.29 (m, 5H), 1.19 – 1.09 (m, 1H).

^{13}C NMR (151 MHz, $CDCl_3$) δ 213.2, 148.8, 1358, 124.0, 121.6, 85.7, 74.7, 61.4, 56.2, 49.4, 43.2, 43.2, 39.2, 35.3, 30.5, 25.8, 25.3, 23.1.

2D NMR COSY, HSQC, HMBC, NOESY

LC-MS 298.1 $[M+H]^+$

<u>Characterization data for hydrazone product</u>

Description white solid

TLC R_f = 0.46 and 0.29 (SiO_2, 7.5% (7N NH_3 in MeOH) in DCM); UV-active, stains green in anisaldehyde (presumably E and Z hydrazones

<u>Characterization data for vinyl iodide</u>

Description White solid

TLC R_f = 0.41 (SiO_2, 30% EtOAc in hexanes); UV-active, stains grey with anisaldehyde
LC-MS m/z = 408 $[M+H]^+$

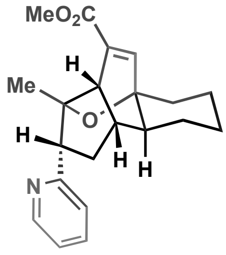

GBFZI-110

Characterization data for methyl enoate **GBFZI-110**
Description colorless oil
TLC R_f = 0.30 (SiO_2, 30% EtOAc in hexanes); UV-active, stains grey in anisaldehyde
^{1}H NMR (600 MHz, $CDCl_3$) δ 8.53 (ddd, *J* = 4.9, 1.8, 0.9 Hz, 1H), 7.60 (td, *J* = 7.7, 1.9 Hz, 1H), 7.34 (dt, *J* = 8.0, 1.1 Hz, 1H), 7.24 (d, *J* = 1.7 Hz, 1H), 7.11 (ddd, *J* = 7.5, 4.9, 1.1 Hz, 1H), 3.81 (s, 3H), 3.44 (dd, *J* = 11.8, 4.8 Hz, 1H), 3.22 (dd, *J* = 4.2, 1.8 Hz, 1H), 2.47 (ddd, *J* = 13.2, 11.8, 6.0 Hz, 1H), 2.12 (dd, *J* = 13.2, 4.8 Hz, 1H), 1.96 – 1.88 (m, 2H), 1.87 – 1.79 (m, 1H), 1.69 – 1.60 (m, 2H), 1.58 – 1.41 (m, 2H), 1.38 – 1.33 (m, 1H), 1.33 – 1.26 (m, 1H), 1.03 (s, 3H), 0.79 (qd, *J* = 13.2, 3.7 Hz, 1H).
^{13}C NMR (151 MHz, $CDCl_3$) δ 165.9, 161.2, 148.6, 145.6, 135.7, 132.6, 123.8, 121.4, 84.9, 73.6, 55.1, 52.8, 52.0, 48.4, 40.2, 37.3, 33.2, 32.3, 25.6, 23.7, 23.5.
2D NMR HSQC
LC-MS m/z = 340.1 $[M+H]^+$
HRMS Calculated $C_{21}H_{26}NO_3$ $[M+H]^+$ = 340.1913 | Found: 340.1922

Synthesis of RS1130

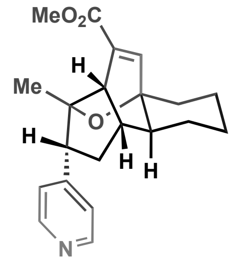

RS1130

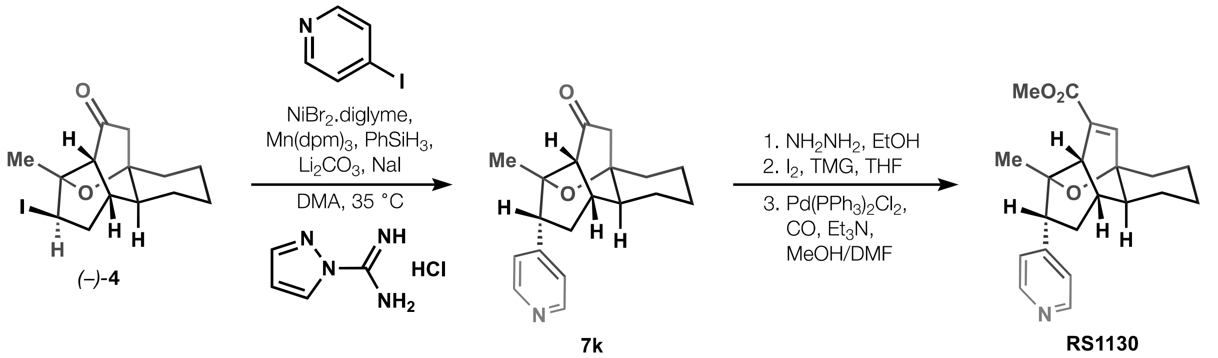

Cross-electrophile coupling (see page S21) A flame-dried 20 mL glass tube with threaded end was equipped with a stir bar and septum pierced with an needle fed by a balloon filled with argon. It was sequentially charged with praxadine.HCl (85.0 mg, 578 µmol, 2 equiv.), $NiBr_2$•diglyme (224 mg, 635 µmol, 2.2 equiv.), $Mn(dpm)_3$ (245 mg, 404 µmol, 1.4 equiv.), 4-iodopyridine (178 mg, 867 µmol, 3 equiv), *(−)*-**4** (100 mg, 289 µmol, 1 equiv.), Li_2CO_3 (21.0 mg, 289 µmol, 1 equiv.), and NaI (260 mg, 1.73 mmol, 6 equiv.). The vial was evacuated and backfilled with argon (5x) and then charged with DMA (dry, 8.8 mL) using a syringe, and the reaction mixture was degassed with an argon balloon and a vent needle while stirring for 45 min. The reaction mixture turned brown at the end of the degassing period. The vent needle was removed, the septum and argon balloon replaced. Silylbenzene (125 mg, 143 µL, 578 µmol, 4 equiv.) was added using a syringe, then the reaction mixture was placed into a preheated 35 °C water bath. The reaction underwent a series of changes in appearance similar to XEC with 2-iodo-6-methyl pyridine from turbid brown-yellow to turbid yellow-olive to turbid pastel green/light jade to turbid dark jade within a timespan of 15 – 30 min. The reaction was stirred for 5.5 h. Upon reaction completion (No SM mass found on LC-MS, faint SM spot remaining, dark forest green reaction mixture) EtOAc (8.8 mL) and ice-cold water (17 mL) were added. The layers were separated and the aqueous layer was extracted with EtOAc (6 x 2mL). The combined organic layers were washed with half saturated brine (2 x 8.8 mL) and brine (2 mL). The combined washes were back extracted with EtOAc (2 mL) and the back extract was washed with half saturated brine (2 x 2 mL), then combined with the organic layer, dried over $MgSO_4$, filtered and concentrated *in vacuo*. The crude product was purified by column chromatography (SiO_2, load in toluene, elute 10-15-20-25-30-35-40-45-50-100% acetone in hexanes). The diastereomers barely separated leaving lots of mixed fractions. The product containing fractions were combined and concentrated *in vacuo* and then further purified by column chromatography (SiO_2, load in toluene, elute 50% acetone in hexanes). The product containing fractions were concentrated *in vacuo* and the diastereomers were separated by preparative TLC (SiO_2, 20 x 20 cm, 0.5 mm thick, load in $CHCl_3$, elute 70% EtOAc in hexanes, 2 plates with half amount of product each, run 4 consecutive times each) affording 24.5 mg

(29% yield) of ketone **8k** as a colorless oil and 26.1 mg (30% yield) of ketone **7k** as a white solid.

Hydrazone condensation. An 8 mL glass vial equipped with a stir bar was charged with EtOH (0.5 mL) and hydrazine monohydrate (67.33 mg, 65.50 µL, 1.35 mmol, 40 equiv.). The resulting solution was placed in a 40 °C water bath with stirring, and a solution of ketone **7k** (10 mg, 33.6 µmol, 1 equiv.) in Chloroform (0.1 mL) was added. Upon full starting material consumption (5.5 h, monitored by TLC), the water bath was removed and the reaction mixture was blown down using nitrogen. The residue was dried with toluene/MeOH azeotrope (1:1, 1x), toluene (3x) and hexanes (3x) to afford a white solid.

Barton iodination. The crude mixture of hydrazones (assume 33.6 µmol) was charged with THF (dry, inhibitor-free, 0.1 mL) and cooled to 0 °C using an ice bath. A separate vessel was charged with iodine (17.0 mg, 68.9 µmol, 2.05 equiv.), TMG (271 mg, 296 µL, 2.35 mmol, 70 equiv.) and THF (dry, inhibitor-free, 300 µL). The iodine solution was taken up in a syringe and added dropwise to the cooled hydrazone solution giving an orange color that faded over time. Full consumption of productive intermediates was indicated once the orange color persisted and further addition of iodine solution only intensified the color and consistent with TLC data. Na_2SO_3 (saturated aqueous, 0.5 mL) was added, the ice-bath was removed, and the reaction mixture was diluted with EtOAc (1.25 mL) and minimal amount of water to dissolve all solids. The layers were separated, the aqueous layer was extracted with EtOAc (2 x 1.25 mL), the combined organic layers were washed with brine (2 mL), dried over $MgSO_4$, filtered and concentrated *in vacuo*. The resulting crude product was purified by column chromatography (SiO_2, load in toluene, elute 20% acetone in hexanes). The product containing fractions were concentrated *in vacuo* affording 8.5 mg (62% yield over two steps) of vinyl iodide as a white solid.

Carbonylation. A flame-dried 8 mL glass vial equipped with a stir bar and septum was charged with vinyl iodide (8.5 mg, 21 µmol, 1 equiv.) and $PdCl_2(PPh_3)_2$ (4.0 mg, 3.76 µmol, 0.3 equiv.). The vial was evacuated (using a needle attached to the vacuum line, pump exhaust was vented into a well-ventilated fume hood) and back filled with CO gas (needle pierced through septum fed by a CO balloon, 3x). DMF (dry, 0.25 mL), MeOH (dry, 0.25 mL) and NEt_3 (0.14 mL) were added via syringes. The reaction mixture was degassed with a CO balloon and venting needle while stirring for 15 min resulting in a homogeneous reaction mixture. The CO balloon was replaced with a fresh one and the reaction was placed into a preheated 50 °C water bath for 6 h. Upon full conversion, the headspace was purged with argon for 5 min. The reaction mixture was blown down using nitrogen gas and then dried with toluene azeotrope (5x). The crude mixture was purified by preparative TLC (SiO_2, 20 x 10 cm, 0.25 mm thick, load in $CHCl_3$ on long side, elute 30% acetone in hexanes, run 2 consecutive times). The obtained product was further purified by another round of preparative TLC (SiO_2, 10 x 10 cm, 0.25 mm thick, load in $CHCl_3$, elute 2.5% MeOH in DCM) affording 4.3 mg (61% yield) of **RS1130** as a colorless oil.

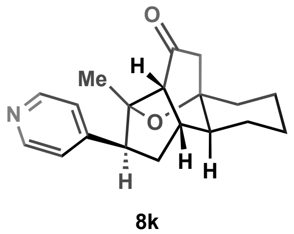

8k

Characterization data for ketone **8k**
Description colorless oil
TLC R_f = 0.14 (SiO_2, 20% acetone in hexanes); UV-active, stains blue in anisaldehyde
^{1}H NMR (600 MHz, $CDCl_3$) δ 8.51 (d, *J* = 5.4 Hz, 2H), 7.07 (d, *J* = 5.9 Hz, 2H), 3.39 (t, *J* = 8.2 Hz, 1H), 2.68 (d, *J* = 19.3 Hz, 1H), 2.41 (d, *J* = 3.9 Hz, 1H), 2.30 (dd, *J* = 13.2, 8.5 Hz, 1H), 2.21 (dt, *J* = 6.7, 3.1 Hz, 1H), 2.05 (ddd, *J* = 13.1, 8.1, 5.0 Hz, 1H), 2.00 (dd, *J* = 19.3, 1.6 Hz, 1H), 1.79 – 1.71 (m, 4H), 1.60 (ddt, *J* = 13.2, 4.0, 1.9 Hz, 1H), 1.50 (td, *J* = 12.2, 3.7 Hz, 1H), 1.38 – 1.26 (m, 2H), 1.22 – 1.12 (m, 1H), 0.82 (s, 3H).
^{13}C NMR (151 MHz, $CDCl_3$) δ 212.1, 151.6, 149.9, 123.9, 86.7, 75.2, 59.4, 57.2, 49.4, 42.5, 42.3, 42.2, 36.0, 30.5, 25.6, 24.1, 22.8.
2D NMR COSY, HSQC, HMBC, NOESY
LC-MS m/z = 298.1 $[M+H]^+$

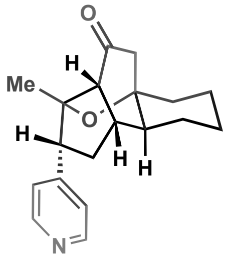

7k

Characterization data for ketone **7k**
Description white solid
TLC R_f = 0.11 (SiO_2, 20% acetone in hexanes); UV-active, stains blue with anisaldehyde
^{1}H NMR (600 MHz, $CDCl_3$) δ 8.56 – 8.50 (m, 2H), 7.26 – 7.22 (m, 2H), 2.99 (dd, *J* = 11.8, 5.3 Hz, 1H), 2.71 (d, *J* = 19.3 Hz, 1H), 2.62 (ddd, *J* = 13.7, 11.8, 6.3 Hz, 1H), 2.45 (d, *J* = 4.2 Hz, 1H), 2.19 (ddd, *J* = 6.1, 4.2, 1.7 Hz, 1H), 2.01 (dd, *J* = 19.3, 1.5 Hz, 1H), 1.86 (dd, *J* = 13.7, 5.3 Hz, 1H), 1.82 – 1.68 (m, 5H), 1.50 (td, *J* = 12.7, 4.0 Hz, 1H), 1.36 (dddd, *J* = 13.1, 11.2, 6.1, 2.8 Hz, 2H), 1.18 (s, 4H).
^{13}C NMR (151 MHz, $CDCl_3$) δ 212.2, 149.4 (2C), 148.6, 125.4 (2C), 85.0, 74.8, 61.0, 54.2, 52.5, 43.0, 42.7, 42.1, 35.5, 30.3, 25.8, 24.6, 23.0.
2D NMR COSY, HSQC, HMBC, NOESY
LC-MS m/z = 298.1 $[M+H]^+$

Characterization data for hydrazone product
Description white solid
TLC R_f = 0.13 (SiO_2, 20% acetone in hexanes); UV-active, stains brown in anisaldehyde

Characterization data for vinyl iodide
Description White solid
TLC R_f = 0.31 (SiO_2, 20% acetone in hexanes); UV-active, stains blue with anisaldehyde
LC-MS m/z = 408 $[M+H]^+$

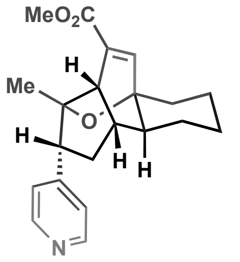

RS1130

Characterization data for methyl enoate **RS1130**
Description colorless oil
TLC $R_f = 0.14$ (SiO_2, 20% acetone in hexanes); UV-active, stains blue in anisaldehyde
^{1}H NMR (600 MHz, $CDCl_3$) δ 8.52 – 8.48 (m, 2H), 7.25 – 7.20 (m, 3H), 3.82 (s, 3H), 3.24 (dd, J = 4.3, 1.8 Hz, 1H), 3.09 (dd, J = 11.9, 5.4 Hz, 1H), 2.61 (ddd, J = 13.7, 11.9, 6.3 Hz, 1H), 2.02 (dt, J = 12.0, 3.4 Hz, 1H), 1.88 (ddd, J = 13.1, 6.3, 3.3 Hz, 1H), 1.76 (dd, J = 13.7, 5.5 Hz, 1H), 1.72 – 1.64f (m, 3H), 1.59 – 1.43 (m, 3H), 1.35 – 1.26 (m, 1H), 0.90 (s, 3H), 0.83 (qd, J = 13.3, 3.8 Hz, 1H).
^{13}C NMR (151 MHz, $CDCl_3$) δ 165.7, 150.6, 149.3, 145.1, 132.5, 125.3, 84.7, 73.7, 55.2, 53.0, 52.1, 48.2, 39.9, 39.5, 33.4, 32.2, 25.7, 23.4, 23.0.
LC-MS m/z = 340.2 $[M+H]^+$
HRMS Calculated $C_{21}H_{26}NO_3$ $[M+H]^+$ = 340.1913 | Found: 340.1917

Synthesis of RS1121

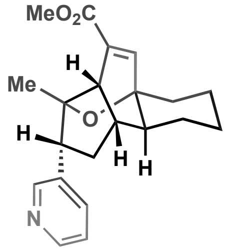

RS1121

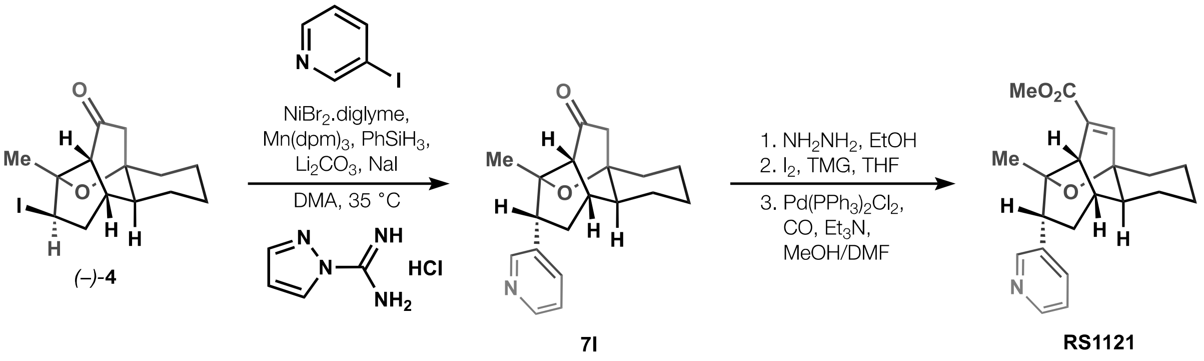

Cross-electrophile coupling (see page S21) A flame-dried 20 mL glass tube with threaded end was equipped with a stir bar and septum pierced with a needle fed by a balloon filled with argon. It was sequentially charged with praxadine.HCl (85.0 mg, 578 µmol, 2 equiv.), $NiBr_2$•diglyme (224 mg, 635 µmol, 2.2 equiv.), $Mn(dpm)_3$ (245 mg, 404 µmol, 1.4 equiv.), 3-iodopyridine (178 mg, 867 µmol, 3 equiv), *(−)*-**4** (100 mg, 289 µmol, 1 equiv.), Li_2CO_3 (21.0 mg, 289 µmol, 1 equiv.), and NaI (260 mg, 1.73 mmol, 6 equiv.). The vial was evacuated and backfilled with argon (5x) and then charged with DMA (dry, 8.8 mL) using a syringe, and the reaction mixture was degassed with an argon balloon and a vent needle while stirring for 45 min. The reaction mixture turned brown at the end of the degassing period. The vent needle was removed, the septum and argon balloon replaced. Silylbenzene (125 mg, 143 µL, 578 µmol, 4 equiv.) was added using a syringe, then the reaction mixture was placed into a preheated 35 °C water bath. The reaction underwent a series of changes in appearance similar to XEC with 2-iodo-6-methyl pyridine from turbid brown-yellow to turbid yellow-olive to turbid pastel green/light jade to turbid dark jade within a timespan of 15 – 30 min. The reaction was stirred for 3h. Upon reaction completion (No SM mass found on LC-MS, faint SM spot remaining, dark forest green colored reaction mixture) EtOAc (8.8 mL) and ice-cold water (17 mL) were added. The layers were separated and the aqueous layer was extracted with EtOAc (6 x 2mL). The combined organic layers were washed with half saturated brine (2 x 8.8 mL) and brine (2 mL). The combined washes were back extracted with EtOAc (2 mL) and the back extract was washed with half saturated brine (2 x 2 mL), then combined with the organic layer, dried over $MgSO_4$, filtered and concentrated *in vacuo*. The crude product was purified by column chromatography (SiO_2, load in toluene, elute 10-15-20-25-30-35-40-45-50-100% EtOAc in hexanes). The diastereomers barely separated leaving lots of mixed fractions. The product containing fractions were combined and concentrated *in vacuo* and then further purified by column chromatography (SiO_2, load in toluene, elute 40% acetone in hexanes). The product containing fractions were concentrated *in vacuo* and the diastereomers were separated by preparative TLC (SiO_2, 20 x 20 cm, 0.5 mm thick, load in $CHCl_3$, elute 50% EtOAc in hexanes, 2 plates with half amount of product each, run 4 consecutive times each) affording 14.7 mg (17% yield) of ketone **8l** as a white solid and 39.5 mg (46% yield) of ketone **7l** as a colorless oil.

Hydrazone condensation. An 8 mL glas vial equipped with a stir bar was charged with EtOH (0.5 mL) and hydrazine monohydrate (67.33 mg, 65.50 µL, 1.345 mmol, 40 equiv.). The resulting solution was placed in a 40 °C water bath with stirring, and a solution of ketone **7l** (10 mg, 33.6 µmol, 1 equiv.) in Chloroform (0.1 mL) was added. Upon full starting material consumption (2 h, monitored by TLC), the water bath was removed and the reaction mixture was blown down using nitrogen. The residue was dried with toluene/MeOH azeotrope (1:1, 1x), toluene (3x) and hexanes (3x) to afford a white solid

Barton iodination. The crude mixture of hydrazones (assume 33.6 µmol) was charged with THF (dry, inhibitor-free, 0.1 mL) and cooled to 0 °C using an ice bath. A separate vessel was charged with iodine (17.0 mg, 68.9 µmol, 2.05 equiv.), TMG (271 mg, 296 µL, 2.35 mmol, 70 equiv.) and THF (dry, inhibitor-free, 300 µL). The iodine solution was taken up in a syringe and added dropwise to the cooled hydrazone solution giving an orange color that faded over time. Full consumption of productive intermediates was indicated once the orange color persisted and further addition of iodine solution only intensified the color and consistent with TLC data. Na_2SO_3 (saturated aqueous, 0.5 mL) was added, the ice-bath was removed, and the reaction mixture was diluted with EtOAc (1.25 mL) and minimal amount of water to dissolve all solids. The layers were separated, the aqueous layer was extracted with EtOAc (2 x 1.25 mL), the combined organic layers were washed with brine (2 mL), dried over $MgSO_4$, filtered and concentrated *in vacuo*. The resulting crude product was purified by column chromatography (SiO_2, load in toluene, elute 20% acetone in hexanes). The product containing fractions were concentrated *in vacuo* affording 10.2 mg (75% yield over two steps) of vinyl iodide as an orange solid.

Carbonylation. A flame-dried 8 mL glass vial equipped with a stir bar and septum was charged with vinyl iodide (10.2 mg, 25 µmol, 1 equiv.) and $PdCl_2(PPh_3)_2$ (3.0 mg, 3.76 µmol, 0.15 equiv.). The vial was evacuated (using a needle attached to the vacuum line, pump exhaust was vented into a well-ventilated fume hood) and back filled with CO gas (needle pierced through septum fed by a CO balloon, 3x). DMF (dry, 0.25 mL), MeOH (dry, 0.25 mL) and NEt_3 (0.14 mL) were added via syringes. The reaction mixture was degassed with a CO balloon and venting needle while stirring for 15 min resulting in a homogeneous reaction mixture. The CO balloon was replaced with a fresh one and the reaction was placed into a preheated 50 °C water bath for 2.5 h. Upon full conversion, the headspace was purged with argon for 5 min. The reaction mixture was blown down using nitrogen gas and then dried with toluene azeotrope (5x). The crude mixture was purified by preparative TLC (SiO_2, 20 x 10 cm, 0.25 mm thick, load in $CHCl_3$ on long side, elute 30% acetone in hexanes, run 5 consecutive times). The obtained product was further purified by another round of preparative TLC (SiO_2, 10 x 10 cm, 0.25 mm thick, load in $CHCl_3$, elute DCM) affording 3.8 mg (45% yield) of **RS1121** as a colorless oil.

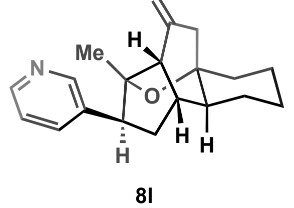

8l

Characterization data for ketone **8l**

Description white solid

TLC $R_f = 0.22$ (SiO_2, 20% acetone in hexanes); UV-active, stains blue in anisaldehyde

¹H NMR (600 MHz, $CDCl_3$) δ 8.51 – 8.4i0 (m, 2H), 7.44 (d, *J* = 7.8 Hz, 1H), 7.23 (t, *J* = 6.3 Hz, 1H), 3.44 (t, *J* = 8.5 Hz, 1H), 2.68 (d, *J* = 19.3 Hz, 1H), 2.41 (d, *J* = 3.5 Hz, 1H), 2.36 – 2.29 (m, 1H), 2.20 (s, 1H), 2.07 – 1.96 (m, 2H), 1.76 (d, *J* = 11.7 Hz, 4H), 1.61 (d, *J* = 13.3 Hz, 1H), 1.51 (t, *J* = 12.5 Hz, 1H), 1.32 (q, *J* = 15.2 Hz, 2H), 1.17 (q, *J* = 12.9 Hz, 1H), 0.81 (s, 3H).
¹³C NMR (151 MHz, $CDCl_3$) δ 212.4, 150.5, 148.3, 138.2, 135.5, 123.5, 87.0, 75.2, 59.5, 55.2, 49.4, 42.7 (2C), 42.4, 36.2, 30.7, 25.8, 24.5, 23.0.
2D NMR COSY, HSQC, HMBC, NOESY
LC-MS m/z = 298.0 $[M+H]^+$

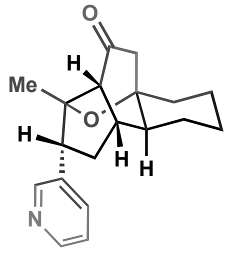

7l

Characterization data for ketone **7l**
Description colorless oil
TLC R_f = 0.15 (SiO_2, 20% acetone in hexanes); UV-active, stains blue in anisaldehyde
¹H NMR (600 MHz, $CDCl_3$) δ 8.54 (d, *J* = 2.2 Hz, 1H), 8.49 (dd, *J* = 4.8, 1.7 Hz, 1H), 7.68 (dt, *J* = 7.9, 2.0 Hz, 1H), 7.26 (dd, *J* = 7.9, 4.8 Hz, 1H), 3.01 (dd, *J* = 12.0, 5.4 Hz, 1H), 2.72 (d, *J* = 19.3 Hz, 1H), 2.66 (ddd, *J* = 13.6, 12.0, 6.3 Hz, 1H), 2.47 (d, *J* = 4.2 Hz, 1H), 2.20 (ddd, *J* = 6.1, 4.2, 1.7 Hz, 1H), 2.02 (dd, *J* = 19.3, 1.5 Hz, 1H), 1.86 – 1.68 (m, 6H), 1.58 – 1.50 (m, 1H), 1.43 – 1.29 (m, 2H), 1.17 (qd, *J* = 13.3, 3.4 Hz, 1H), 1.15 (s, 3H).
¹³C NMR (151 MHz, $CDCl_3$) δ 212.4, 151.3, 148.2, 137.4, 134.8, 123.0, 84.8, 74.8, 61.0, 52.7, 52.3, 43.0, 42.7, 42.6, 35.5, 30.3, 25.8, 24.4, 23.0.
LC-MS m/z = 298.2 $[M+H]^+$

Characterization data for hydrazone product
Description white solid
TLC R_f = 0.25 (SiO_2, 20% acetone in hexanes); UV-active, stains grey/brown in anisaldehyde

Characterization data for vinyl iodide
Description Orange solid
TLC R_f = 0.35 (SiO_2, 20% acetone in hexanes); UV-active, stains blue with anisaldehyde
LC-MS m/z = 408 $[M+H]^+$

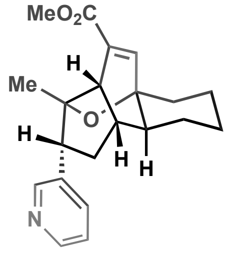

RS1121

Characterization data for methyl enoate **RS1121**
Description colorless oil

TLC R_f = 0.14 (SiO_2, 20% acetone in hexanes); UV-active, stains blue in anisaldehyde
^{1}H NMR (600 MHz, $CDCl_3$) δ 8.52 (s, 1H), 8.45 (d, *J* = 4.7 Hz, 1H), 7.68 – 7.63 (m, 1H), 7.23 (s, 2H), 3.82 (d, *J* = 2.2 Hz, 3H), 3.27 – 3.23 (m, 1H), 3.11 (dd, *J* = 12.3, 5.5 Hz, 1H), 2.64 (td, *J* = 12.9, 6.5 Hz, 1H), 2.02 (d, *J* = 10.1 Hz, 1H), 1.88 (d, *J* = 12.4 Hz, 1H), 1.75 – 1.64 (m, 4H), 1.57 – 1.47 (m, 3H), 1.29 (d, *J* = 17.3 Hz, 2H), 0.88 (d, *J* = 2.1 Hz, 3H), 0.86 – 0.79 (m, 1H).
^{13}C NMR (151 MHz, $CDCl_3$) δ 165.7, 151.2, 147.8, 145.0, 137.2, 136.8, 132.6, 123.1, 84.6, 73.6, 55.4, 52.1, 51.0, 48.1, 40.4, 39.5, 33.4, 32.2, 25.7, 23.4, 22.9.
2D NMR HSQC
LC-MS m/z = 340 $[M+H]^+$
HRMS Calculated $C_{21}H_{26}NO_3$ $[M+H]^+$ = 340.1913 | Found: 340.1908

Synthesis of RS1187 (7m)

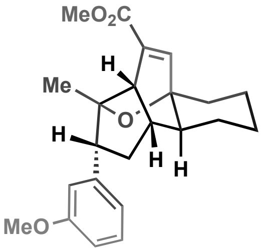

RS1167
(7m)

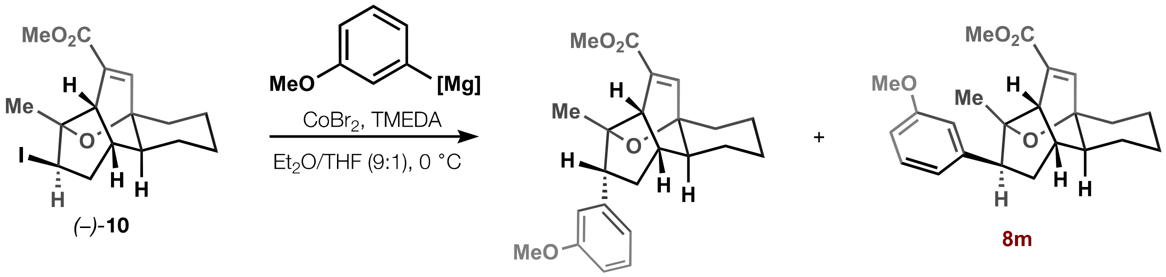

RS1167
(7m)

Aryl-Grignard preparation. A flame-dried 1.5 mL vial equipped with a stir bar and septum pierced with a needle fed by an argon balloon was charged with THF (dry, 100 µL), 3-iodoanisole (120 mg, 63.4 µL, 515 µmol, 10 equiv.) using micro syringes. Turbo-Grignard (82.3 mg, 436 µL (1.3M solution in THF), 567 µmol, 11 equiv.) was added dropwise to the stirred reaction mixture at 22 °C using a syringe. The reaction mixture was stirred for 30 min before all of it was taken up in a syringe and used in the subsequent step.

Kumada coupling. An 8 mL flame-dried glass tube with threaded end was equipped with a stir bar and septum pierced with a needed fed by an argon balloon. It was charged with $CoBr_2$ (28.0 mg, 129 µmol, 2.5 equiv.) and *(−)*-**10** (20.0 mg, 51.5 µmol, 1 equiv.). The vial was evacuated and backfilled with argon (3x) and then charged with diethyl ether (dry, 1.8 mL), THF (dry, 0.2 mL) and TMEDA (15.0 mg, 19.3 µL, 129 µmol, 2.5 equiv.). An ice bath was equipped and the reaction was cooled to 0 °C. The prepared aryl-Grignard was added dropwise to the reaction mixture. Upon complete starting material consumption, $NaHCO_3$ (saturated aqueous, ice-cold, 5 mL) was added and the aqueous layer was extracted with diethyl ether (2 x 1.5 mL). The combined organic layers were washed with brine (2 mL), dried over $MgSO_4$, filtered and concentrated *in vacuo*. The crude product was purified by column chromatography (4g SiO_2, load in toluene, elute 0-5-10-15-20-25-30-35-40-100% EtOAc in hexanes). The product containing fractions were concentrated *in vacuo* and then further purified by preparative TLC (SiO_2, 10 x 10 cm, 0.5 mm thick, load in $CHCl_3$, elute 5% EtOAc in hexanes, run 2 consecutive times). The diastereomers were separated by preparative TLC (SiO_2, 10 x 10 cm, 0.5 mm thick, load in toluene, elute 1% EtOAc in $CHCl_3$, run two consecutive times). The desired endo product was further purified by another round of preparative TLC (SiO_2, 10 x 10 cm, 0.5 mm thick, load in toluene, elute 1% EtOAc in $CHCl_3$, run two consecutive times) affording 3.9 mg (21% yield) of methyl enoate **8m** as a colorless oil and 2.2 mg (12% yield) of methyl enoate **7m** (**RS1167**) as a colorless oil.

Characterization data for methyl enoate **8m**
Description colorless oil

TLC R_f = 0.37 (SiO_2, 2.5% EtOAc in $CHCl_3$); UV-active, stains blue in anisaldehyde
^{1}H NMR (600 MHz, $CDCl_3$) δ 7.26 (s, 1H), 7.22 (t, *J* = 7.9 Hz, 1H), 6.85 (d, *J* = 7.6 Hz, 1H), 6.80 (d, *J* = 2.2 Hz, 1H), 6.75 (dd, *J* = 8.2, 2.5 Hz, 1H), 3.80 (s, 6H), 3.30 (t, *J* = 8.6 Hz, 1H), 3.19 (q, *J* = 1.6 Hz, 1H), 2.16 (dd, *J* = 13.0, 9.0 Hz, 1H), 2.11 – 2.00 (m, 2H), 1.86 (d, *J* = 13.2 Hz, 1H), 1.70 (d, *J* = 5.3 Hz, 1H), 1.68 – 1.62 (m, 1H), 1.55 – 1.40 (m, 4H), 1.25 – 1.19 (m, 1H), 0.79 (qd, *J* = 13.2, 3.7 Hz, 1H), 0.57 (s, 3H).
^{13}C NMR (151 MHz, $CDCl_3$) δ 165.9, 159.6, 145.6, 145.5, 132.7, 129.4, 121.3, 115.1, 111.6, 85.7, 74.0, 56.6, 55.3, 52.0, 51.8, 46.4, 40.4, 39.8, 33.8, 32.4, 25.7, 23.4, 22.6.
2D NMR COSY, HSQC, HMBC, NOESY
LC-MS m/z = 369.2 $[M+H]^+$ and 351.2 $[M-H_2O+H]^+$

Characterization data for methyl enoate **7m** (**RS1167**)
Description colorless oil
TLC R_f = 0.27 (SiO_2, 2.5% EtOAc in $CHCl_3$); UV-active, stains blue in anisaldehyde
^{1}H NMR (600 MHz, $CDCl_3$) δ 7.23 (d, *J* = 1.8 Hz, 1H), 7.20 (t, *J* = 8.1 Hz, 1H), 6.91 – 6.83 (m, 2H), 6.83 – 6.73 (m, 1H), 3.81 (s, 3H), 3.80 (s, 3H), 3.21 (dd, *J* = 4.3, 1.8 Hz, 1H), 3.09 (dd, *J* = 12.0, 5.6 Hz, 1H), 2.60 (ddd, *J* = 13.5, 11.9, 6.3 Hz, 1H), 2.06 – 2.00 (m, 1H) 1.87 (d, *J* = 13.4 Hz, 1H), 1.77 (dd, *J* = 13.6, 5.6 Hz, 1H), 1.73 – 1.61 (m, 3H), 1.54 – 1.45 (m, 3H), 1.35 – 1.26 (m, 1H), 0.89 (s, 3H), 0.82 (qd, *J* = 13.1, 3.8 Hz, 1H).
^{13}C NMR (151 MHz, $CDCl_3$) δ 165.9, 159.3, 145.1, 143.0, 132.7, 128.8, 122.6, 116.1, 111.5, 84.7, 73.5, 55.2(9), 55.2(7), 53.5, 52.0, 48.3, 40.5, 39.5, 33.5, 32.2, 25.8, 23.5, 23.0.
2D NMR COSY, HSQC, HMBC, NOESY
LC-MS m/z = 369.2 $[M+H]^+$ and 351.2 $[M-H_2O+H]^+$
HRMS Calculated $C_{23}H_{28}O_4$ $[M+H]^+$ = 369.2066 | Found: 369.2062

Synthesis of RS1187 (7n)

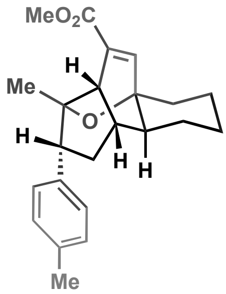

RS1187
(7n)

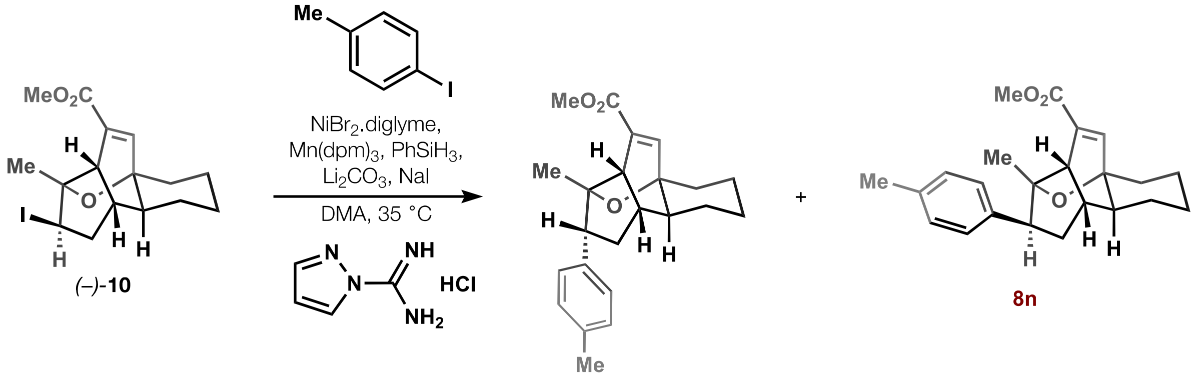

(See page S21)
A flame-dried 20 mL glass tube with threaded end was equipped with a stir bar and septum pierced with an needle fed by a balloon filled with argon. It was sequentially charged with praxadine.HCl (15.0 mg, 103.0 µmol, 2 equiv.), $NiBr_2$•diglyme (40.0 mg, 113.3 µmol, 2.2 equiv.), $Mn(dpm)_3$ (44.0 mg, 72.1 µmol, 1.4 equiv.), 4-iodotoluene (56.0 mg, 257 µmol, 5 equiv.), *(−)*-**10** (20.0 mg, 51.5 µmol, 1 equiv.), Li_2CO_3 (4.0 mg, 51.5 µmol, 1 equiv.), and NaI (46.0 mg, 309.1 µmol, 6 equiv.). The vial was evacuated and backfilled with argon (5x) and then charged with DMA (dry, 1.8 mL) using a syringe, and the reaction mixture was degassed with an argon balloon and a vent needle while stirring for 30 min. The reaction mixture turned brown at the end of the degassing period. The vent needle was removed, the septum and argon balloon replaced. Silylbenzene (22.3 mg, 25.5 µL, 206.1 µmol, 4 equiv.) was added using a syringe, then the reaction mixture was placed into a preheated 35°C water bath. The reaction underwent a series of changes in appearance similar to XEC with 2-iodo-6-methyl pyridine from turbid brown-yellow to turbid yellow-olive to turbid pastel green/light jade to turbid dark jade within a timespan of 15 – 30 min. The reaction was stirred for 20 h (still SM remaining and productive intermediates at 6 h into the reaction. Left overnight with additional 4-iodotoluene (5 equiv.) and silylbenzene (4 equiv.)). Upon reaction completion (No SM mass found on LC-MS, faint SM spot remaining, dark forest green reaction mixture) EtOAc (2 mL) and ice-cold water (4 mL) were added. The layers were separated and the aqueous layer was extracted with EtOAc (6 x 0.5 mL). The combined organic layers were washed with half saturated brine (2 x 2 mL) and brine (0.5 mL). The combined washes were back extracted with EtOAc (0.5 mL) and the back extract was washed with half saturated brine (2 x 0.5 mL), then combined with the organic layer, dried over $MgSO_4$, filtered and concentrated *in vacuo*. The crude product was purified by column chromatography (4 g SiO_2, load in toluene, elute 0-5-10-15-20-25-30-35-40-100% acetone in hexanes, Combiflash purification system). The product containing fractions were concentrated *in vacuo* and further purified by column chromatography (SiO_2, load in toluene, elute 10% acetone in hexanes). The resulting product

was further purified by column chromatography (SiO_2, load in toluene, elute 0-10% EtOAc in DCM). Subsequently, the product was further purified on a Waters Prep SFC 150 AP using a Phenomenex CEL-1 column (5 um, 21x250 mm). The purification was run under isocratic conditions (5% IPA / CO2, 100 mL/min, 1600 psi backpressure) at 35 °C. Fractionation was triggered by mass spec (ESI+ on a Waters QDa, SIR channel m/z = 335.2). Two products were obtained, fast-moving isomer mentyl enoate **7n** (3.3 mg, 19%) (**RS1187**) and slow-moving isomer methyl enoate **8n** (3.5 mg, 10%).

Characterization data for methyl enoate **7n** (**RS1187**)
Description colorless film
TLC R_f = 0.38 (SiO_2, 15% EtOAc in hexanes); UV-active, stains greyish purple in anisaldehyde
^{1}H NMR (600 MHz, C_6D_6) δ 7.28 (d, *J* = 1.7 Hz, 1H), 7.25 (d, *J* = 7.9 Hz, 2H), 7.09 (d, *J* = 7.8 Hz, 2H), 3.50 (s, 3H), 3.31 (dd, *J* = 4.3, 1.8 Hz, 1H), 2.85 (dd, *J* = 12.0, 5.6 Hz, 1H), 2.33 (ddd, *J* = 13.4, 12.0, 6.4 Hz, 1H), 2.19 (s, 3H), 1.95 – 1.90 (m, 1H), 1.68 (dd, *J* = 13.4, 5.7 Hz, 1H), 1.57 (dt, *J* = 12.9, 3.1 Hz, 1H), 1.53 – 1.41 (m, 3H), 1.36 (s, 1H), 1.27 – 1.13 (m, 2H), 0.98 (s, 4H), 0.68 (qd, *J* = 13.1, 3.7 Hz, 1H).
^{13}C NMR (151 MHz, C_6D_6) δ 165.52, 145.19, 138.82, 135.57, 133.21, 130.28, 128.73, 84.78, 73.26, 55.54, 53.18, 51.35, 48.52, 40.67, 39.75, 33.83, 32.29, 25.92, 23.46, 23.20, 21.09.
2D NMR HSQC, HMBC, NOESY
LCMS m/z +335 $[M\text{-}H_2O+H]^+$
HRMS Calculated $C_{23}H_{27}O_2$ $[M\text{-}H_2O+H]^+$ = 335.2011 | Found: 335.2010

Characterization data for methyl enoate **8n**
Description white film
TLC R_f = 0.38 (SiO_2, 15% EtOAc in hexanes); UV-active, stains purple in anisaldehyde
^{1}H NMR (600 MHz, C_6D_6) δ 7.38 (d, *J* = 1.7 Hz, 1H), 7.03 (d, *J* = 7.7 Hz, 2H), 6.93 (d, *J* = 7.7 Hz, 2H), 3.49 (d, *J* = 0.9 Hz, 4H), 3.42 (t, *J* = 8.7 Hz, 1H), 2.12 (s, 3H), 2.05 – 1.91 (m, 2H), 1.53 (d, *J* = 19.0 Hz, 3H), 1.48 (dd, *J* = 12.9, 4.2 Hz, 1H), 1.40 (s, 1H), 1.33 – 1.16 (m, 3H), 1.06 – 0.93 (m, 1H), 0.85 (s, 3H), 0.67 (qd, *J* = 12.9, 3.6 Hz, 1H).
^{13}C NMR (151 MHz, C_6D_6) δ 165.61, 145.79, 141.25, 135.75, 133.14, 129.43, 129.43, 129.03, 85.66, 73.92, 56.67, 51.93, 51.39, 46.95, 40.83, 40.16, 34.16, 32.48, 25.98, 23.44, 23.08, 21.00.
2D NMR HSQC, HMBC, NOESY
LCMS m/z +335 $[M\text{-}H_2O+H]^+$
HRMS Calculated $C_{23}H_{29}O_3$ $[M+H]^+$ = 353.2117 | Found: 353.2109

Synthesis of RS1129

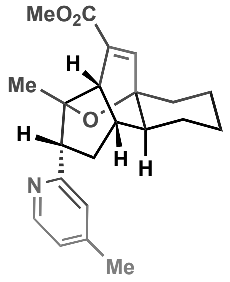

RS1129

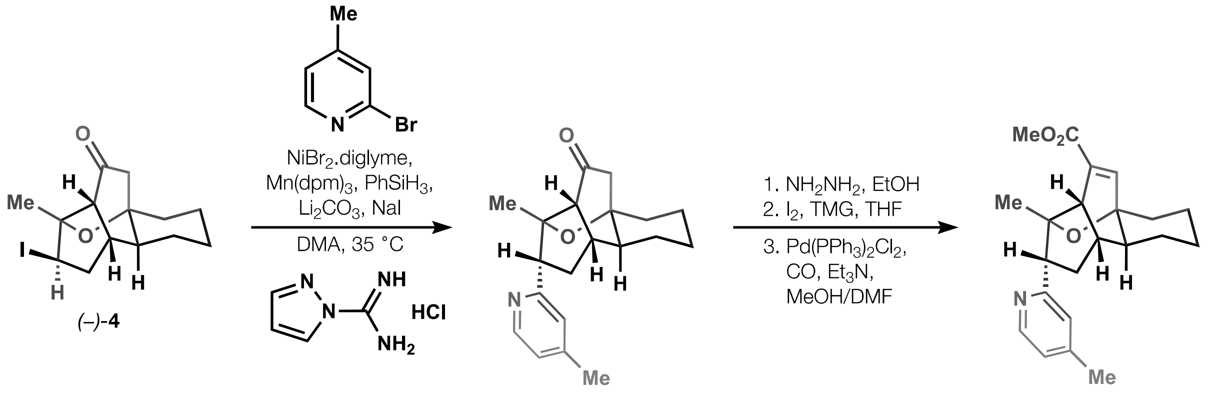

Cross-electrophile coupling (see page S21) A flame-dried 20 mL glass tube with threaded end was equipped with a stir bar and septum pierced with an needle fed by a balloon filled with argon. It was sequentially charged with praxadine.HCl (85.0 mg, 578 µmol, 2 equiv.), $NiBr_2$•diglyme (224 mg, 635 µmol, 2.2 equiv.), $Mn(dpm)_3$ (245 mg, 404 µmol, 1.4 equiv.), *(−)*-**4** (100 mg, 289 µmol, 1 equiv.), Li_2CO_3 (21.0 mg, 289 µmol, 1 equiv.), and NaI (260 mg, 1.73 mmol, 6 equiv.). The vial was evacuated and backfilled with argon (5x) and then charged with DMA (dry, 8.8 mL) using a syringe, 2-bromo-4-methyl pyridine (149 mg, 96.5 µL, 867 µmol, 3 equiv.) using a micro syringe, and the reaction mixture was degassed with an argon balloon and a vent needle while stirring for 45 min. The reaction mixture turned brown at the end of the degassing period. The vent needle was removed, the septum and argon balloon replaced. Silylbenzene (125 mg, 143 µL, 578 µmol, 4 equiv.) was added using a syringe, then the reaction mixture was placed into a preheated 35 °C water bath. The reaction underwent a series of changes in appearance similar to XEC with 2-iodo-6-methyl pyridine from turbid brown-yellow to turbid yellow-olive to turbid pastel green/light jade to turbid dark jade within a timespan of 15 – 30 min. The reaction and stirred for 4 h. Upon reaction completion (No SM mass found on LC-MS, faint SM spot remaining, dark forest green colored reaction mixture) EtOAc (8.8 mL) and ice-cold water (17 mL) were added. The layers were separated and the aqueous layer was extracted with EtOAc (6 x 2mL). The combined organic layers were washed with half saturated brine (2 x 8.8 mL) and brine (2 mL). The combined washes were back extracted with EtOAc (2 mL) and the back extract was washed with half saturated brine (2 x 2 mL), then combined with the organic layer, dried over $MgSO_4$, filtered and concentrated *in vacuo*. The crude product was purified and diastereomers were separated by column chromatography (SiO_2, load in toluene, elute 10-15-20-25-30-35-40% EtOAc in hexanes).

Exo diastereomer. **8o** The product containing fractions were concentrated *in vacuo* and further purified by column chromatography (SiO_2, load in toluene, elute 10% acetone in hexanes) affording 1.7 mg (2% yield) of ketone **8o** as a clear oil.

Endo diastereomer. **7o** The product containing fractions were concentrated *in vacuo* and further purified by column chromatography (SiO_2, load in toluene, elute 10% acetone in hexanes) affording 14.2 mg (15.8% yield) of ketone **7o** as a white solid.

Hydrazone condensation. An 8 mL glass vial equipped with a stir bar was charged with EtOH (0.5 mL) and hydrazine monohydrate (39.9 mg, 38.8 µL, 796 µmol, 40 equiv.). The resulting solution was placed in a 40 °C water bath with stirring, and a solution of ketone **7o** (6.2 mg, 19.9 µmol, 1 equiv.) in Chloroform (0.1 mL) was added. Upon full starting material consumption (3 h, monitored by TLC), the water bath was removed and the reaction mixture was blown down using nitrogen. The residue was dried with toluene/MeOH azeotrope (1:1, 1x), toluene (3x) and hexanes (3x) to afford a white solid.

Barton iodination. The crude mixture of hydrazones (assume 19.9 µmol) was charged with THF (dry, inhibitor-free, 0.1 mL) and cooled to 0 °C using an ice bath. A separate vessel was charged with iodine (10.0 mg, 40.8 µmol, 2.05 equiv.), TMG (160 mg, 175 µL, 1.39 mmol, 70 equiv.) and THF (dry, inhibitor-free, 180 µL). The iodine solution was taken up in a syringe and added dropwise to the cooled hydrazone solution giving an orange color that faded over time. Full consumption of productive intermediates was indicated once the orange color persisted and further addition of iodine solution only intensified the color and consistent with TLC data. Na_2SO_3 (saturated aqueous, 0.5 mL) was added, the ice-bath was removed, and the reaction mixture was diluted with EtOAc (1.25 mL) and minimal amount of water to dissolve all solids. The layers were separated, the aqueous layer was extracted with EtOAc (2 x 1.25 mL), the combined organic layers were washed with brine (2 mL), dried over $MgSO_4$, filtered and concentrated *in vacuo*. The resulting crude product was purified by column chromatography (SiO_2, load in toluene, elute 10-15-20-25-30% EtOAc in hexanes). The product containing fractions were concentrated *in vacuo* affording 3.8 mg (45% yield over two steps) of vinyl iodide as a colorless gum.

Carbonylation. A flame-dried 8 mL glass vial equipped with a stir bar and septum was charged with vinyl iodide (3.8 mg, 9.0 µmol, 1 equiv.) and $PdCl_2(PPh_3)_2$ (2.0 mg, 2.7 µmol, 0.3 equiv.). The vial was evacuated (using a needle attached to the vacuum line, pump exhaust was vented into a well-ventilated fume hood) and back filled with CO gas (needle pierced through septum fed by a CO balloon, 3x). DMF (dry, 0.25 mL), MeOH (dry, 0.25 mL) and NEt_3 (0.14 mL) were added via syringes. The reaction mixture was degassed with a CO balloon and venting needle while stirring for 15 min resulting in a homogeneous reaction mixture. The CO balloon was replaced with a fresh one and the reaction was placed into a preheated 50 °C water bath for 2.5 h. Upon full conversion, the headspace was purged with argon for 5 min. The reaction mixture was blown down using nitrogen gas and then dried with toluene azeotrope (5x). The crude mixture was purified by preparative TLC (SiO_2, 20 x 10 cm, 0.25 mm thick, load in $CHCl_3$ on long side, elute 30% EtOAc in hexanes). The obtained product was further purified by another round of preparative TLC (SiO_2, 10 x 10 cm, 0.25 mm thick, load in $CHCl_3$, elute 20% acetone in hexanes) affording 2.5 mg (78% yield) of **RS1129** as a colorless oil.

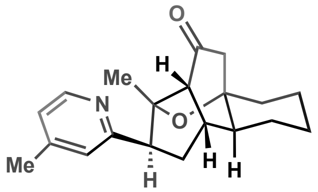

8o

Characterization data for ketone **8o**
Description colorless oil
TLC R_f = 0.38 (SiO_2, 30% EtOAc in hexanes); UV-active, stains blue in anisaldehyde
^{1}H NMR (600 MHz, $CDCl_3$) δ 8.37 (d, *J* = 4.9 Hz, 1H), 6.99 (s, 1H), 6.91 (d, *J* = 4.9 Hz, 1H), 3.43 (t, *J* = 8.0 Hz, 1H), 2.75 (t, *J* = 3.1 Hz, 1H), 2.66 (d, *J* = 19.2 Hz, 1H), 2.51 (dt, *J* = 13.3, 6.2 Hz, 1H), 2.31 (s, 3H), 2.19 – 2.11 (m, 2H), 1.97 (d, *J* = 19.1 Hz, 1H), 1.77 – 1.71 (m, 4H), 1.61 – 1.57 (m, 1H), 1.53 – 1.46 (m, 1H), 1.38 – 1.31 (m, 2H), 1.19 – 1.12 (m, 1H), 0.79 (s 3H).
^{13}C NMR (151 MHz, $CDCl_3$) δ 213.4, 162.0, 149.4, 147.2, 125.7, 122.6, 87.5, 75.1, 58.7, 58.6, 50.1, 42.8, 42.8, 41.3, 36.3, 30.7, 25.9, 23.7, 23.1, 21.1.
2D NMR COSY, HSQC, HMBC, NOESY
LC-MS m/z = 312.1 $[M+H]^+$

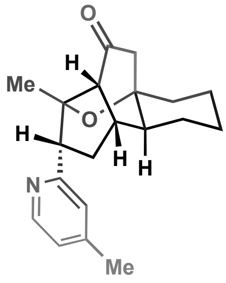

7o

Characterization data for ketone **7o**
Description white solid
TLC R_f = 0.21 (SiO_2, 30% acetone in hexanes); UV-active, stains blue in anisaldehyde
^{1}H NMR (600 MHz, $CDCl_3$) δ 8.42 (d, *J* = 5.0 Hz, 1H), 7.12 (d, *J* = 1.6 Hz, 1H), 6.96 (dd, *J* = 5.2, 1.6 Hz, 1H), 3.30 (dd, *J* = 11.3, 4.7 Hz, 1H), 2.67 (d, *J* = 19.3 Hz, 1H), 2.43 (d, *J* = 4.0 Hz, 1H), 2.42 – 2.31 (m, 5H), 2.26 (ddt, *J* = 13.5, 3.9, 1.8 Hz, 1H), 2.17 – 2.12 (m, 1H), 1.95 (dd, *J* = 19.2, 1.6 Hz, 1H), 1.76 – 1.70 (m, 3H), 1.65 – 1.59 (m, 1H), 1.43 – 1.27 (m, 6H), 1.19 – 1.09 (m, 1H).
^{13}C NMR (151 MHz, $CDCl_3$) δ 213.3, 159.0, 148.6, 146.6, 125.0, 122.7, 85.8, 74.7, 61.4, 56.0, 49.1, 43.2, 43.1, 39.0, 35.4, 30.5, 25.7, 25.3, 23.1, 21.3.
2D NMR COSY, HSQC, HMBC, NOESY
LC-MS m/z = 312.2 $[M+H]^+$

Characterization data for hydrazone product
Description white solid
TLC R_f = 0.42 (SiO_2, 30% EtOAc in hexanes); UV-active, stains grey in anisaldehyde

Characterization data for vinyl iodide
Description Colorless oil
TLC R_f = 0.55 (SiO_2, 30% EtOAc in hexanes); UV-active, stains blue with anisaldehyde
LC-MS m/z = 422.1 $[M+H]^+$

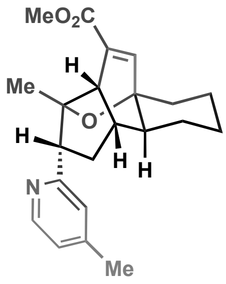

RS1129

<u>Characterization data for methyl enoate **RS1129**</u>

Description colorless oil

TLC R_f = 0.21 (SiO_2, 30% EtOAc in hexanes); UV-active, stains blue in anisaldehyde

^{1}H NMR (600 MHz, $CDCl_3$) δ 8.39 (d, J = 5.1 Hz, 1H), 7.24 (d, J = 1.7 Hz, 1H), 7.15 – 7.12 (m, 1H), 6.95 – 6.91 (m, 1H), 3.81 (s, 3H), 3.39 (dd, J = 11.8, 4.7 Hz, 1H), 3.21 (dd, J = 4.1, 1.8 Hz, 1H), 2.44 (ddd, J = 13.2, 11.8, 6.0 Hz, 1H), 2.33 (s, 3H), 2.13 (dd, J = 13.2, 4.8 Hz, 1H), 1.95 (ddt, J = 15.1, 11.2, 2.8 Hz, 2H), 1.86 – 1.80 (m, 1H), 1.68 – 1.61 (m, 2H), 1.55 – 1.44 (m, 2H), 1.37 – 1.28 (m, 3H), 1.02 (s, 3H), 0.78 (qd, J = 13.2, 3.7 Hz, 1H).

^{13}C NMR (151 MHz, $CDCl_3$) δ 165.9, 160.8, 148.4, 145.6, 132.6, 124.8, 122.5, 84.9, 73.6, 54.9, 52.6, 52.00, 48.5, 40.3, 37.2, 33.2, 32.2, 25.6, 23.7, 23.5, 21.4. (one carbon not observed)

LC-MS m/z = 354.1 $[M+H]^+$

HRMS Calculated $C_{22}H_{28}NO_3$ $[M+H]^+$ = 354.2069 | Found: 354.2061

Synthesis of RS1139

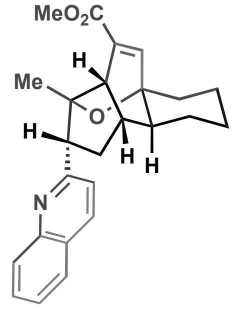

RS1139

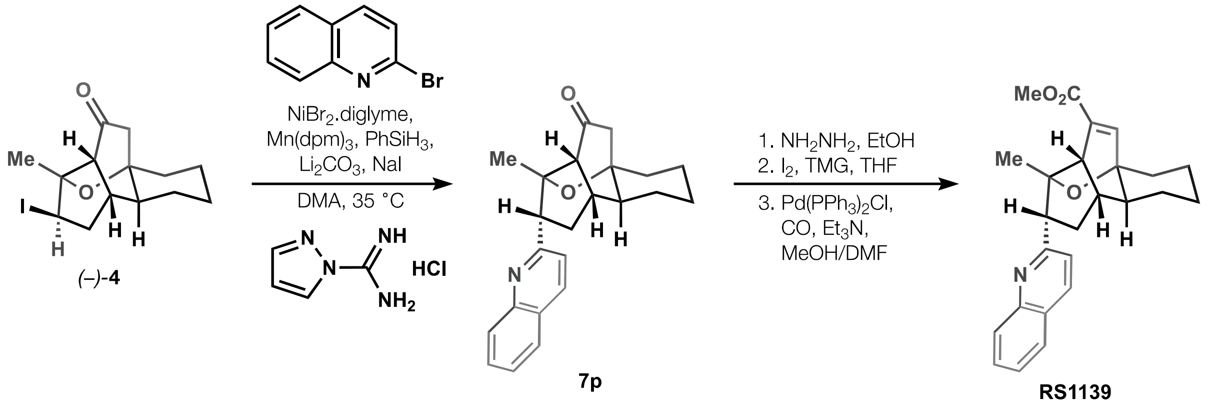

Cross-electrophile coupling (see page S21) A flame-dried 20 mL glass tube with threaded end was equipped with a stir bar and septum pierced with an needle fed by a balloon filled with argon. It was sequentially charged with praxadine.HCl (85.0 mg, 578 µmol, 2 equiv.), $NiBr_2$•diglyme (224 mg, 635 µmol, 2.2 equiv.), $Mn(dpm)_3$ (245 mg, 404 µmol, 1.4 equiv.), 2-bromo-quinoline (180 mg, 867 µmol, 3 equiv.) *(−)*-**4** (100 mg, 289 µmol, 1 equiv.), Li_2CO_3 (21.0 mg, 289 µmol, 1 equiv.), and NaI (260 mg, 1.73 mmol, 6 equiv.). The vial was evacuated and backfilled with argon (5x) and then charged with DMA (dry, 8.8 mL) using a syringe, and the reaction mixture was degassed with an argon balloon and a vent needle while stirring for 45 min. The reaction mixture turned brown at the end of the degassing period. The vent needle was removed, the septum and argon balloon replaced. Silylbenzene (125 mg, 143 µL, 578 µmol, 4 equiv.) was added using a syringe, then the reaction mixture was placed into a preheated 35 °C water bath. The reaction underwent a series of changes in appearance similar to XEC with 2-iodo-6-methyl pyridine from turbid brown-yellow to turbid yellow-olive to turbid pastel green/light jade to turbid dark jade within a timespan of 15 – 30 min. The reaction was stirred for 3 h. Upon reaction completion (No SM mass found on LC-MS, faint SM spot remaining, dark forest green reaction mixture) EtOAc (8.8 mL) and ice-cold water (17 mL) were added. The layers were separated and the aqueous layer was extracted with EtOAc (6 x 2mL). The combined organic layers were washed with half saturated brine (2 x 8.8 mL) and brine (2 mL). The combined washes were back extracted with EtOAc (2 mL) and the back extract was washed with half saturated brine (2 x 2 mL), then combined with the organic layer, dried over $MgSO_4$, filtered and concentrated *in vacuo*. The crude product was purified by column chromatography (40 g SiO_2, load in toluene, elute 10-15-20-25-30-35-40% EtOAc in hexanes, Combiflash purification system). The product containing fractions were concentrated *in vacuo* and and further purified by column chromatography (SiO_2, load in toluene, elute 2.5% MeOH in DCM). The diastereomers were separated by column chromatography (SiO_2, load in toluene, elute 5-7.5-10-15-30-50% EtOAc in hexanes).

Exo product. **8p** The product containing fractions were concentrated *in vacuo* and the resulting solid was further purified by preparative TLC (SiO_2, 10 x 10 cm, 0.25 mm thick, load in $CHCl_3$, elute 2.5% EtOAc in DCM) to afford 5.2 mg (5% yield) of ketone **8p** as a white solid.
Endo product. **7p** The product containing fractions were concentrated *in vacuo* and the resulting soldi was further purified by preparative TLC (SiO_2, 20 x 10 cm, 0.5 mm thick, load in $CHCl_3$ on long side, elute 10% EtOAc in hexanes) to afford 22.0 mg (22% yield) of ketone **7p** as a white solid.

Hydrazone condensation. An 8 mL glass vial equipped with a stir bar was charged with EtOH (0.5 mL) and hydrazine monohydrate (57.6 mg, 56.0 µL, 1.15 mmol, 40 equiv.). The resulting solution was placed in a 40 °C water bath with stirring, and a solution of ketone **7p** (10.0 mg, 28.8 µmol, 1 equiv.) in Chloroform (0.1 mL) was added. Upon full starting material consumption (1.5 h, monitored by TLC), the water bath was removed and the reaction mixture was blown down using nitrogen. The residue was dried with toluene/MeOH azeotrope (1:1, 1x), toluene (3x) and hexanes (3x) to afford a white solid.

Barton iodination. The crude mixture of hydrazones (assume 28.8 µmol) was charged with THF (dry, inhibitor-free, 0.1 mL) and cooled to 0 °C using an ice bath. A separate vessel was charged with iodine (15.0 mg, 59.0 µmol, 2.05 equiv.), TMG (232 mg, 253 µL, 2.01 mmol, 70 equiv.) and THF (dry, inhibitor-free, 250 µL). The iodine solution was taken up in a syringe and added dropwise to the cooled hydrazone solution giving an orange color that faded over time. Full consumption of productive intermediates was indicated once the orange color persisted and further addition of iodine solution only intensified the color and consistent with TLC data. Na_2SO_3 (saturated aqueous, 0.5 mL) was added, the ice-bath was removed, and the reaction mixture was diluted with EtOAc (1.25 mL) and minimal amount of water to dissolve all solids. The layers were separated, the aqueous layer was extracted with EtOAc (2 x 1.25 mL), the combined organic layers were washed with brine (2 mL). The brine wash was backextracted with EtOAc (3 x 2 mL). The combined organic layers were dried over $MgSO_4$, filtered and concentrated *in vacuo*. The resulting crude product was purified by column chromatography (SiO_2, load in toluene, elute 10-15-20-25% EtOAc in hexanes). The product containing fractions were concentrated *in vacuo* affording 7.7 mg (59% yield over two steps) of vinyl iodide as a white solid.

Carbonylation. A flame-dried 8 mL glass vial equipped with a stir bar and septum was charged with vinyl iodide (7.7 mg, 17.0 µmol, 1 equiv.) and $PdCl_2(PPh_3)_2$ (3.0 mg, 5.1 µmol, 0.3 equiv.). The vial was evacuated (using a needle attached to the vacuum line, pump exhaust was vented into a well-ventilated fume hood) and back filled with CO gas (needle pierced through septum fed by a CO balloon, 3x). DMF (dry, 0.25 mL), MeOH (dry, 0.25 mL) and NEt_3 (0.14 mL) were added via syringes. The reaction mixture was degassed with a CO balloon and venting needle while stirring for 15 min resulting in a homogeneous reaction mixture. The CO balloon was replaced with a fresh one and the reaction was placed into a preheated 50 °C water bath for 2 h. Upon full conversion, the headspace was purged with argon for 5 min. The reaction mixture was blown down using nitrogen gas and then dried with toluene azeotrope (5x). The crude mixture was purified by preparative TLC (SiO_2, 20 x 10 cm, 0.25 mm thick, load in $CHCl_3$ on long side, elute 10% EtOAc in hexanes, run 4 consecutive times). The obtained product was further purified by another round of preparative TLC (SiO_2, 10 x 10 cm, 0.25 mm thick, load in $CHCl_3$, elute 5% acetone in hexanes, run 2 consecutive times) affording 5.5 mg (84% yield) of **RS1139** as a white solid.

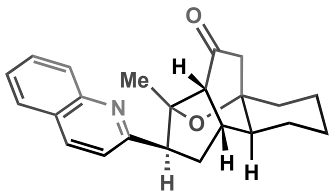

8p

<u>Characterization data for ketone **8p**</u>

Description White solid

TLC R_f = 0.54 (SiO_2, 30% EtOAc in hexanes); UV-active, stains blue in anisaldehyde

^{1}H NMR (600 MHz, $CDCl_3$) δ 8.04 (d, *J* = 8.4 Hz, 1H), 7.99 (d, *J* = 8.5 Hz, 1H), 7.78 – 7.73 (m, 1H), 7.67 (ddd, *J* = 8.5, 6.8, 1.5 Hz, 1H), 7.51 – 7.45 (m, 1H), 7.29 (d, *J* = 8.4 Hz, 1H), 3.71 (t, *J* = 7.8 Hz, 1H), 2.90 (d, *J* = 3.9 Hz, 1H), 2.76 (ddd, *J* = 12.6, 7.3, 5.3 Hz, 1H), 2.70 (d, *J* = 19.2 Hz, 1H), 2.28 – 2.21 (m, 2H), 2.00 (dd, *J* = 19.2, 1.6 Hz, 1H), 1.80 – 1.74 (m, 4H), 1.68 – 1.62 (m, 1H), 1.54 (td, *J* = 12.7, 4.2 Hz, 1H), 1.42 – 1.27 (m, 2H), 1.24 – 1.14 (m, 1H), 0.82 (s, 3H).

^{13}C NMR (151 MHz, $CDCl_3$) δ 213.6, 162.7, 148.2, 136.0, 129.5(3), 129.4(7) (br), 127.5 (br), 127.0, 126.1 (br), 123.1 (br), 87.8, 75.2, 59.2 (br), 58.8 (br) 50.1, 43.0 (br), 42.8 (br), 41.5 (br), 36.3, 30.7, 25.9, 23.9, 23.1.

2D NMR COSY, HSQC, HMBC, NOESY

LC-MS m/z = 348.2 $[M+H]^+$

Notes: broad carbon signals obtained are attributed to quinoline rotamers

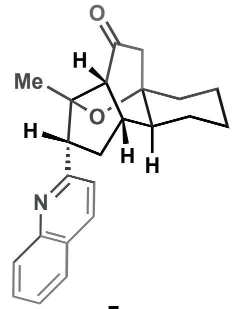

7p

<u>Characterization data for ketone **7p**</u>

Description white solid

TLC R_f = 0.38 (SiO_2, 30% EtOAc in hexanes); UV-active, stains blue in anisaldehyde

^{1}H NMR (600 MHz, $CDCl_3$) δ 8.10 – 8-04 (m, 2H), 7.79 (dd, *J* = 8.1, 1.5 Hz, 1H), 7.68 (ddd, *J* = 8.4, 6.8, 1.5 Hz, 1H), 7.52 – 7.44 (m, 2H), 3.56 (dd, *J* = 11.5, 4.3 Hz, 1H), 2.69 (d, *J* = 19.2 Hz, 1H), 2.55 (dd, *J* = 13.1, 4.3 Hz, 1H), 2.53 – 2.48 (m, 2H), 2.44 (ddd, *J* = 13.1, 11.5, 5.7 Hz, 1H), 2.23 – 2.18 (m, 1H), 1.95 (dd, *J* = 19.3, 1.6 Hz, 1H), 1.83 – 1.74 (m, 2H), 1.72 – 1.67 (m, 1H), 1.56 – 1.53 (m, 1H), 1.38 (s, 3H), 1.31 – 1.24 (m, 3H), 1.17 (qd, *J* = 13.3, 3.9 Hz, 1H).

^{13}C NMR (151 MHz, $CDCl_3$) δ 213.2, 159.6, 147.8, 135.4, 129.1 (2C), 127.6 (br), 127.0, 125.9 (br), 122.3 (br), 86.0, 74.7, 61.6 (br), 56.9 (br), 48.9 (br), 43.3 (br, 2C), 39.0 (br), 35.2, 30.5, 25.9, 25.6 (br), 23.0.

2D NMR COSY, HSQC, HMBC, NOESY

LC-MS m/z = 348.2 $[M+H]^+$

<u>Characterization data for hydrazone product</u>

Description white solid

TLC R_f = 0.00 (SiO_2, 30% EtOAc in hexanes), 0.64 and 0.43 (30% acetone in hexanes, presumably E/Z hydrazones); UV-active, stains yellow in anisaldehyde

Characterization data for vinyl iodide
Description White solid
TLC R_f = 0.71 (SiO_2, 30% EtOAc in hexanes), 0.56 (SiO_2, 30% acetone in hexanes); UV-active, stains green in anisaldehyde
LC-MS m/z = 458.2 $[M+H]^+$

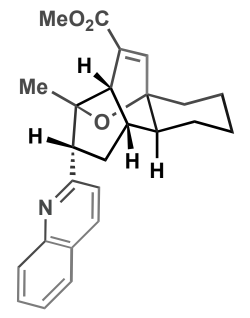

RS1139

Characterization data for **RS1139**
Description White solid
TLC R_f = 0.61 (SiO_2, 30% EtOAc in hexanes); UV-active, stains green in anisaldehyde
^{1}H NMR (600 MHz, $CDCl_3$) δ 8.07 – 8.02 (m, 2H), 7.77 (dd, *J* = 8.1, 1.5 Hz, 1H), 7.65 (ddd, *J* = 8.4, 6.8, 1.5 Hz, 1H), 7.51 – 7.42 (m, 2H), 7.25 (d, *J* = 1.8 Hz, 1H), 3.82 (s, 3H), 3.63 (dd, *J* = 11.6, 4.6 Hz, 1H), 3.27 (dd, *J* = 4.2, 1.8 Hz, 1H), 2.50 (ddd, *J* = 13.2, 11.7, 5.9 Hz, 1H), 2.34 (dd, *J* = 13.2, 4.6 Hz, 1H), 2.22 – 2.14 (m, 1H), 1.89 (dt, *J* = 12.3, 3.4 Hz, 1H), 1.85 – 1.80 (m, 1H), 1.72 (ddd, *J* = 6.1, 4.1, 2.0 Hz, 1H), 1.67 (dt, *J* = 13.5, 3.2 Hz, 1H), 1.59 – 1.55 (m, 1H), 1.50 (qt, *J* = 13.6, 3.9 Hz, 1H), 1.38 – 1.27 (m, 2H), 1.08 (s, 3H), 0.91 – 0.77 (m, 1H).
^{13}C NMR (151 MHz, $CDCl_3$) δ 165.9, 161.6, 147.9, 145.8, 135.4, 132.5, 129.3, 129.1, 127.6, 127.1, 125.7, 122.2, 85.2, 73.7, 55.9, 52.3, 52.0, 48.6, 40.5, 37.1, 33.2, 32.3, 25.8, 23.9, 23.5.
2D NMR HSQC
LC-MS m/z = 390.2 $[M+H]^+$
HRMS Calculated $C_{25}H_{28}NO_3$ $[M+H]^+$ = 390.2069 | Found: 390.2068

Synthesis of RS1128

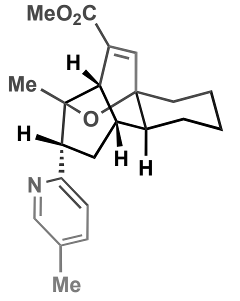

RS1128

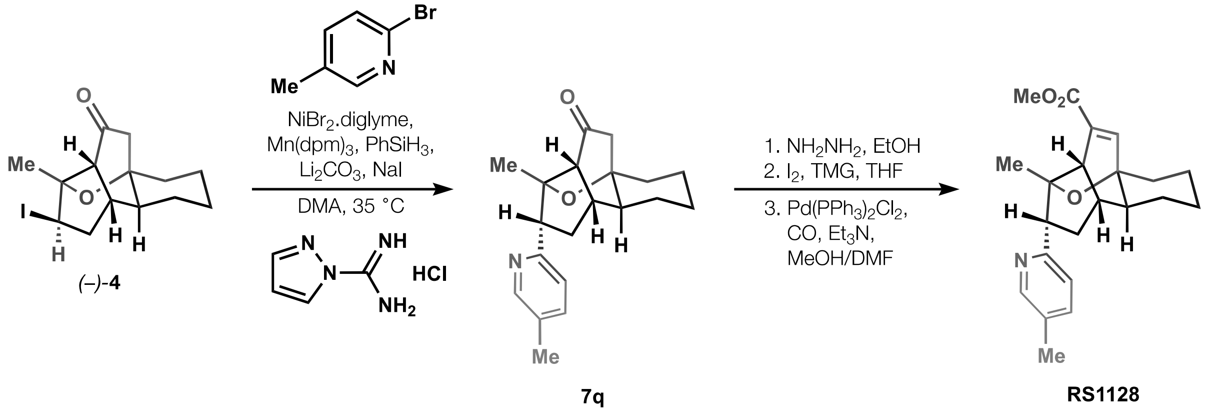

Cross-electrophile coupling (see page S21) A flame-dried 20 mL glass tube with threaded end was equipped with a stir bar and septum pierced with an needle fed by a balloon filled with argon. It was sequentially charged with praxadine.HCl (85.0 mg, 578 µmol, 2 equiv.), $NiBr_2$•diglyme (224 mg, 635 µmol, 2.2 equiv.), $Mn(dpm)_3$ (245 mg, 404 µmol, 1.4 equiv.), 2-bromo-5-methyl pyridine (149 mg, 867 µmol, 3 equiv), *(−)*-**4** (100 mg, 289 µmol, 1 equiv.), Li_2CO_3 (21.0 mg, 289 µmol, 1 equiv.), and NaI (260 mg, 1.73 mmol, 6 equiv.). The vial was evacuated and backfilled with argon (5x) and then charged with DMA (dry, 8.8 mL) using a syringe, and the reaction mixture was degassed with an argon balloon and a vent needle while stirring for 45 min. The reaction mixture turned brown at the end of the degassing period. The vent needle was removed, the septum and argon balloon replaced. Silylbenzene (125 mg, 143 µL, 578 µmol, 4 equiv.) was added using a syringe, then the reaction mixture was placed into a preheated 35 °C water bath. The reaction underwent a series of changes in appearance similar to XEC with 2-iodo-6-methyl pyridine from turbid brown-yellow to turbid yellow-olive to turbid pastel green/light jade to turbid dark jade within a timespan of 15 – 30 min. The reaction was stirred for 6 h. Upon reaction completion (No SM mass found on LC-MS, faint SM spot remaining, dark forest green colored reaction mixture) EtOAc (8.8 mL) and ice-cold water (17 mL) were added. The layers were separated and the aqueous layer was extracted with EtOAc (6 x 2mL). The combined organic layers were washed with half saturated brine (2 x 8.8 mL) and brine (2 mL). The combined washes were back extracted with EtOAc (2 mL) and the back extract was washed with half saturated brine (2 x 2 mL), then combined with the organic layer, dried over $MgSO_4$, filtered and concentrated *in vacuo*. The crude product was purified and diastereomers were separated by column chromatography (SiO_2, load in toluene, elute 10-15-20-25-30-35-40% EtOAc in hexanes).

Exo-diastereomer. **8q** The product containing fractions were concentrated *in vacuo* and resulting oil was further purified by two round of column chromatography (SiO_2, load in

toluene, elute 10% acetone in hexanes) affording 2.7 mg (3% yield) of ketone **8q** as a colorless oil.
Endo-diastereomer. **7q** The product containing fractions were concentrated *in vacuo* and the resulting oil was further purified by column chromatography (SiO_2, load in toluene, elute 10% acetone in hexanes) affording 13 mg (14% yield) of ketone **7q** as a white solid.

Hydrazone condensation. An 8 mL glass vial equipped with a stir bar was charged with EtOH (0.5 mL) and hydrazine monohydrate (32.1 mg, 31.2 µL, 642 µmol, 40 equiv.). The resulting solution was placed in a 40 °C water bath with stirring, and a solution of ketone **7q** (5 mg, 16.1 µmol, 1 equiv.) in Chloroform (0.1 mL) was added. Upon full starting material consumption (4 h, monitored by TLC), the water bath was removed and the reaction mixture was blown down using nitrogen. The residue was dried with toluene/MeOH azeotrope (1:1, 1x), toluene (3x) and hexanes (3x) to afford a white solid.

Barton iodination. The crude mixture of hydrazones (assume 16.1 µmol) was charged with THF (dry, inhibitor-free, 0.1 mL) and cooled to 0 °C using an ice bath. A separate vessel was charged with iodine (8.0 mg, 32.9 µmol, 2.05 equiv.), TMG (130 mg, 141 µL, 1.12 mmol, 70 equiv.) and THF (dry, inhibitor-free, 150 µL). The iodine solution was taken up in a syringe and added dropwise to the cooled hydrazone solution giving an orange color that faded over time. Full consumption of productive intermediates was indicated once the orange color persisted and further addition of iodine solution only intensified the color and consistent with TLC data. Na_2SO_3 (saturated aqueous, 0.5 mL) was added, the ice-bath was removed, and the reaction mixture was diluted with EtOAc (1.25 mL) and minimal amount of water to dissolve all solids. The layers were separated, the aqueous layer was extracted with EtOAc (2 x 1.25 mL), the combined organic layers were washed with brine (2 mL), dried over $MgSO_4$, filtered and concentrated *in vacuo*. The resulting crude product was purified by column chromatography (SiO_2, load in toluene, elute 10-15-20-25-30% EtOAc in hexanes). The product containing fractions were concentrated *in vacuo* affording 3.5 mg (52% yield over two steps) of vinyl iodide as a colorless gum.

Carbonylation. A flame-dried 8 mL glass vial equipped with a stir bar and septum was charged with vinyl iodide (3.5 mg, 8.3 µmol, 1 equiv.) and $PdCl_2(PPh_3)_2$ (2.0 mg, 2.5 µmol, 0.3 equiv.). The vial was evacuated (using a needle attached to the vacuum line, pump exhaust was vented into a well-ventilated fume hood) and back filled with CO gas (needle pierced through septum fed by a CO balloon, 3x). DMF (dry, 0.25 mL), MeOH (dry, 0.25 mL) and NEt_3 (0.14 mL) were added via syringes. The reaction mixture was degassed with a CO balloon and venting needle while stirring for 15 min resulting in a homogeneous reaction mixture. The CO balloon was replaced with a fresh one and the reaction was placed into a preheated 50 °C water bath for 4 h. Upon full conversion, the headspace was purged with argon for 5 min. The reaction mixture was blown down using nitrogen gas and then dried with toluene azeotrope (5x). The crude mixture was purified by preparative TLC (SiO_2, 20 x 10 cm, 0.25 mm thick, load in $CHCl_3$ on long side, elute 30% EtOAc in hexanes). The obtained product was further purified by another round of preparative TLC (SiO_2, 10 x 10 cm, 0.25 mm thick, load in $CHCl_3$, elute 30% acetone in hexanes) affording 1.7 mg (58% yield) of **RS1128** as a colorless oil.

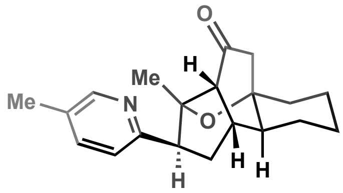

8q

Characterization data for ketone **8q**
Description colorless oil
TLC R_f = 0.41 (SiO_2, 30% EtOAc in hexanes); UV-active, stains green in anisaldehyde
^{1}H NMR (600 MHz, $CDCl_3$) δ 8.35 (d, *J* = 2.3 Hz, 1H), 7.37 (dd, *J* = 7.9, 2.4 Hz, 1H), 7.05 (d, *J* = 7.8 Hz, 1H), 3.46 (t, *J* = 7.8 Hz, 1H), 2.74 (d, *J* = 3.9 Hz, 1H), 2.66 (d, *J* = 19.1 Hz, 1H), 2.50 (ddd, *J* = 12.5, 7.8, 5.0 Hz, 1H), 2.28 (s, 3H), 2.16 (tt, *J* = 12.6, 5.6 Hz, 2H), 1.97 (dd, *J* = 19.2, 1.6 Hz, 1H), 1.77 – 1.70 (m, 4H), 1.61 – 1.55 (m, 2H), 1.50 (td, *J* = 12.4, 3.7 Hz, 1H), 1.35 – 1.27 (m, 2H), 1.20 – 1.12 (m, 1H), 0.78 (s, 3H).
^{13}C NMR (151 MHz, $CDCl_3$) δ 213.4, 159.2, 150.1, 136.7, 130.9, 124.2, 87.5, 75.1, 58.7, 58.2, 50.1, 42.8, 42.8, 41.3, 36.2, 30.7, 25.9, 23.7, 23.1, 18.2.
2D NMR COSY, HSQC, HMBC, NOESY
LC-MS m/z = 312.2 $[M+H]^+$

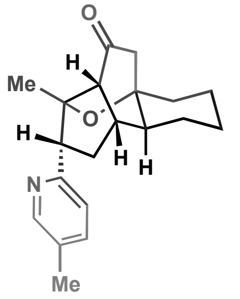

7q

Characterization data for ketone **7q**
Description White solid
TLC R_f = 0.22 (SiO_2, 30% EtOAc in hexanes); UV-active, stains green in anisaldehyde
^{1}H NMR (600 MHz, $CDCl_3$) δ 8.42 (s, 1H), 7.46 (d, *J* = 7.9 Hz, 1H), 7.25 (d, *J* = 8.0 Hz, 1H), 3.34 (dt, *J* = 11.8, 3.8 Hz, 1H), 2.70 (dd, *J* = 19.2, 2.2 Hz, 1H), 2.48 – 2.40 (m, 2H), 2.33 (s, f3H), 2.31 (s, 0H), 2.21 (d, *J* = 13.6 Hz, 1H), 2.16 (d, *J* = 5.2 Hz, 1H), 1.97 (d, *J* = 19.3 Hz, 1H), 1.79 – 1.70 (m, 3H), 1.64 (d, *J* = 10.9 Hz, 1H), 1.43 – 1.30 (m, 6H), 1.16 (q, *J* = 12.8 Hz, 1H).
^{13}C NMR (151 MHz, $CDCl_3$) δ 213.2, 156.2, 149.1, 136.3, 130.7, 123.3, 85.6, 74.5, 61.3, 55.6, 49.4, 43.1, 43.0, 39.2, 35.2, 30.4, 25.6, 25.1, 22.9, 18.1.
2D NMR COSY, HSQC, HMBC, NOESY
LC-MS m/z = 312.2 $[M+H]^+$

Characterization data for hydrazone product
Description white solid
TLC R_f = 0.32 (SiO_2, 30% EtOAc in hexanes); UV-active, stains blue in anisaldehyde

Characterization data for vinyl iodide
Description Colorless gum
TLC R_f = 0.60 (SiO_2, 30% EtOAc in hexanes); UV-active, stains green with anisaldehyde
LC-MS m/z = 422.1 $[M+H]^+$

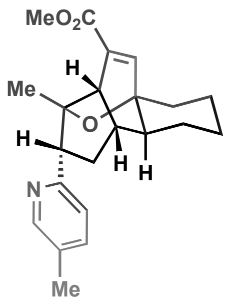

RS1128

Characterization data for **RS1128**

Description Colorless oil

TLC R_f = 0.41 (SiO_2, 30% EtOAc in hexanes); UV-active, stains green in anisaldehyde

^{1}H NMR (600 MHz, $CDCl_3$) δ 8.40 – 8.37 (m, 1H), 7.43 (dd, *J* = 8.1, 2.3 Hz, 1H), 7.28 – 7.24 (m, 2H), 3.84 (s, 3H), 3.43 (dd, *J* = 11.8, 4.8 Hz, 1H), 3.23 (dd, *J* = 4.3, 1.8 Hz, 1H), 2.53 – 2.45 (m, 1H), 2.31 (s, 3H), 2.10 (dd, *J* = 13.1, 4.9 Hz, 1H), 1.99 – 1.89 (m, 2H), 1.85 (d, *J* = 14.2 Hz, 1H), 1.71 – 1.63 (m, 2H), 1.57 – 1.46 (m, 2H), 1.42 – 1.29 (m, 2H), 1.03 (s, 3H), 0.81 (qd, *J* = 13.1, 3.6 Hz, 1H).

^{13}C NMR (151 MHz, $CDCl_3$) δ 165.8, 158.0, 148.9, 145.5, 136.3, 132.5, 130.4, 123.1, 84.7, 73.4, 54.4, 52.7, 51.9, 48.3, 40.1, 37.3, 33.1, 32.1, 25.5, 23.5, 23.3, 18.0.

LC-MS m/z = 354.2 $[M+H]^+$

HRMS Calculated $C_{22}H_{28}NO_3$ $[M+H]^+$ = 354.2069 | Found: 354.2067

Synthesis of RS1125

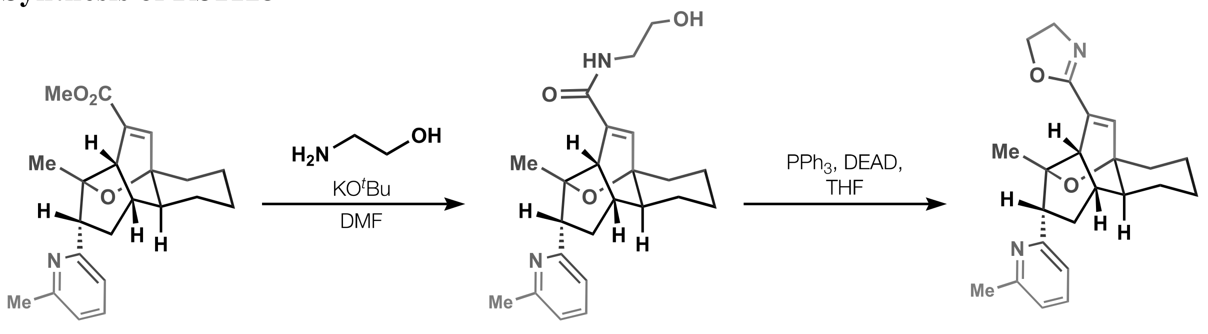

(–)-**2**

Nat-pyr-GB18 *(−)*-**2** (5 mg, 0.014 mmol) was transferred to an 8 mL culture tube and dried with PhMe azeotrope. The headspace was placed under Ar (3x evacuate/backfill cycles), then anhydrous DMF (200 µL) and ethanolamine (300 µL) were added. The mixture was sonicated to ensure full dissolution (note: A fully clear pale-yellow solution should be observed. Full dissolution of starting material prior to KOtBu addition is essential, otherwise significant methyl ester saponification is observed.). KOtBu (solid, ca. 100 mg, excess) was quickly added in one portion and dissolved over the course of 30 s – 1 min. The reaction tube was heated to 50 °C for 15 h, then cooled to 0 °C and quenched with a mixture of saturated aqueous $NaHCO_3$ (2 mL) and saturated aqueous Na_2CO_3 (2 mL) to give a final pH of ~11-12. The mixture was extracted with EtOAc (5x) and the combined organics were dried over Na_2SO_4. Concentration gave an oily residue (11 mg) (NB: partly insoluble in CH_2Cl_2!) the soluble portion of which was then filtered through Na_2SO_4 with Et_2O/minimal CH_2Cl_2, concentrated to dryness and dried with PhMe azeotrope in an 8 mL culture tube. The resulting residue was used directly for the subsequent reaction.

The residue from above containing the putative (2-hydroxyethyl)amide intermediate (*m/z* = +383) was charged with an oven-dried stir bar and PPh_3 (16.0 mg, 0.06 mmol). The tube was placed under Ar (3x evacuate/backfill cycles) and anhydrous THF (600 µL) was added. The reaction was placed in an ice bath and diethyl azodicarboxylate (40 wt% in PhMe, 27.3 µL, 0.06 mmol) was added, giving a pale yellow solution. After 2 h at 0 °C, additional PPh_3 (16.0 mg, 0.06 mmol) and diethyl azodicarboxylate (40 wt% in PhMe, 27.3 µL, 0.06 mmol) were added. After an additional 4 h at 0 °C, the entire reaction mixture was passed through a plug of pre-equilibrated (EtOAc) deactivated basic alumina (3 cm x 15 mm), eluting with EtOAc. The eluate was concentrated to give a solid residue (45 mg), which was purified by column chromatography (SiO_2, 0-10-30-50% acetone/CH_2Cl_2, product elutes in 50% acetone/CH_2Cl_2). Additional purification by two rounds of preparative TLC (round 1: SiO_2, 30% acetone/CH_2Cl_2 x 2 passes; round 2: SiO_2, EtOAc) afforded oxazoline **RS1125** (1.8 mg, 35%).

Characterization data for oxazoline **RS1125**

Description colorless film

TLC R_f = 0.20 (SiO_2, 30% acetone/CH_2Cl_2); UV-active, stains turquoise in anisaldehyde

^{1}H NMR (600 MHz, $CDCl_3$) δ 7.47 (t, *J* = 7.7 Hz, 1H), 7.14 (d, *J* = 7.8 Hz, 1H), 6.95 (d, *J* = 7.6 Hz, 1H), 6.94 – 6.92 (bs, 1H), 4.39 – 4.31 (m, 2H), 4.06 – 3.93 (m, 2H), 3.38 (dd, *J* = 11.8, 4.7 Hz, 1H), 3.24 (dd, *J* = 4.1, 1.7 Hz, 1H), 2.52 (s, 3H), 2.44 (ddd, *J* = 13.1, 11.8, 5.9 Hz, 1H), 2.07 (dd, *J* = 13.1, 4.7 Hz, 1H), 2.02 (ddd, *J* = 13.4, 3.9, 2.1 Hz, 1H), 1.93 (dt, *J* = 12.3, 3.4 Hz, 1H), 1.85 – 1.78 (m, 1H), 1.70 (ddd, *J* = 6.1, 4.0, 2.0 Hz, 1H), 1.67 – 1.61 (m, 1H), 1.55 – 1.44 (m, 2H), 1.35 (ddd, *J* = 13.7, 12.2, 4.2 Hz, 1H), 1.32 – 1.18 (m, 1H), 1.05 (s, 3H), 0.84 (qd, *J* = 13.1, 3.6 Hz, 1H).

^{13}**C NMR** (151 MHz, $CDCl_3$) δ 163.6, 160.6, 157.0, 140.6, 135.8, 129.4, 120.6, 84.6, 73.3, 67.5, 55.1, 54.9, 52.7, 49.5, 40.4, 37.6, 33.4, 32.3, 25.9, 24.8, 23.7, 23.5.
2D NMR HSQC, HMBC, COSY, NOESY
HRMS Calculated $C_{23}H_{29}N_2O_2$ $[M+H]^+$ 365.2229 | Found: 365.2230

Exo attached-ring product as poor KOR agonist

KOR agonism of **SI-2 (RS1177)** and ***epi*-SI-2 (RS1178)** (diastereomeric intermediates *en route* to **RS1176**) have been investigated as controls.

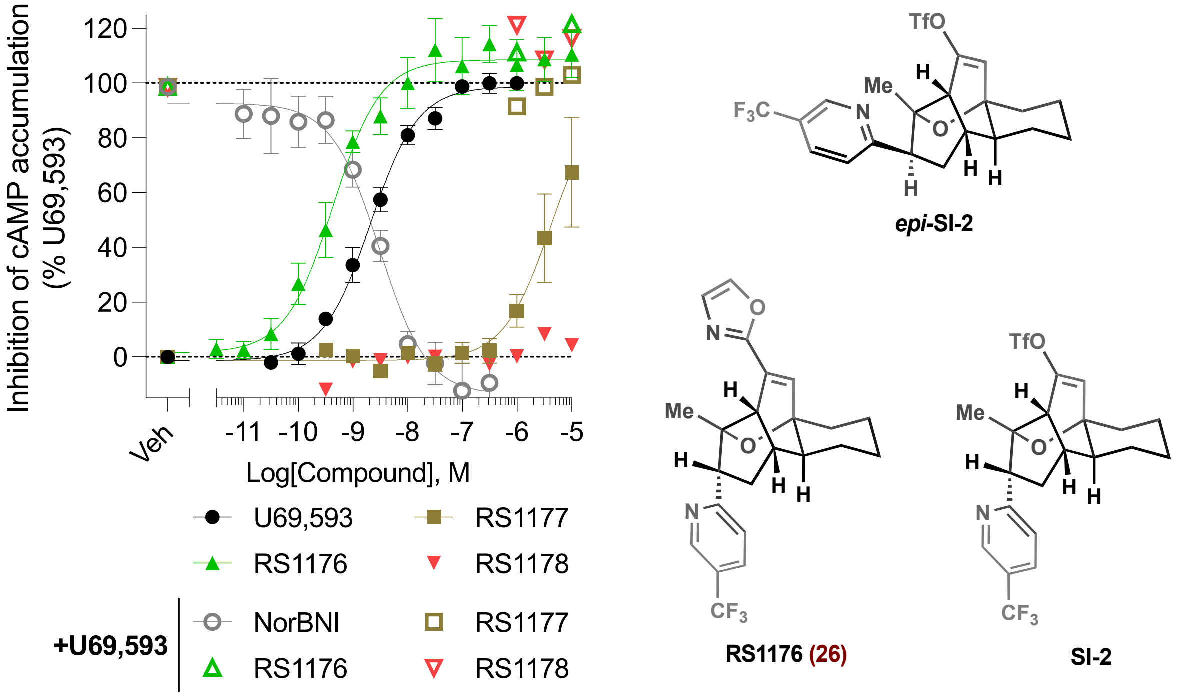

Figure S7. Forskolin-stimulated cAMP accumulation potencies as % max U69,593 response (solid) and antagonism (testing for partial agonism) with norBNI and U69,593-mixed samples Shown are the mean values of replicates performed in duplicate: RS1176 (n=4 for as agonist, n=1 as antagonist); RS1177 (n=3 agonist, n=1 antagonist; RS1178 (n=1 for each); U69 (n=4) and norBNI (n=3).

Methods and materials for pharmacological experiments

Chemicals and drugs U69,593 are from Sigma-Aldrich (St. Louis, MO); Tween 80 and dimethyl sulfoxide (DMSO) (certified grade reference material pharmaceutical secondary standard, >=99.7% purity) from Fisher Scientific (Pittsburg, PA); and sterile 0.9% saline from VWR (Radnor, PA). For all in vitro assays, compounds were prepared in DMSO at concentrations spanning from 32 nM to 10 mM, for dilutions with the final DMSO concentration of less than 1% in any assay. ^{35}S-GTPγS is from Revvity, (Waltham, MA) (with specific activity of 1250 Ci/mmol) and ^{3}H-Naloxone was provided by the NIDA Drug Supply Program (specific activity 48.19 Ci/mmol).

Cell lines and cell culture Chinese hamster ovary (CHO) cells expressing human kappa opioid receptor (hKOR) complementary DNA (cDNA) including three haemagglutinin tags on the N-terminus (3×HA-hKOR, cDNA.org) were generated and used as previously described; the DiscoveRx PathHunterTM U2OS hKOR (U2OS-hKOR-βarrestin2-EFC) were purchased from DiscoveRx Corp. (Fremont, CA) and are used as previously described.[5,6]

The hMOR-CHO cell line has also been used to determine affinity and the hMOR DiscoveRx PathHunterTM U2OS hKOR (U2OS-hKOR-βarrestin2-EFC) was used to determine potency as previously described.[8]

Radioligand binding assays Membranes were prepared from CHO cell lines that stably express MOR or KOR as previously described.[8] Briefly, 10 μg membrane protein was incubated in the presence of 2nM 3H-Naloxone and increasing concentrations of GB18 derivative for 2 hours at room temperature in 10 mM Tris, pH 7.4. The reaction was stopped by rapid filtration, washing and drying of membranes and application of scintillation fluid allowed for counting of retained radioactivity (MicroscintTM-20, from Revvity). Nonspecific binding was determined in the presence of 1 μM unlabeled naloxone. The pKd for naloxone was determined by homologous competition for hMOR: 9.1 ± 0.22 and hKOR: 8.6 ± 0.03. Nonlinear regression analysis was used to determine pKi using Graphpad Prism (v. 10.2) software.

Signaling assays Cell membranes are prepared from CHO-hKOR cells and assessed for ^{35}S-GTPγS binding following treatment with KOR agonists according to previously published protocols.[5,6] Briefly, 15 μg membranes were incubated in assay buffer (50 mM Tris pH 7.4, 100 mM NaCl, 5 mM MgCl2, 1 mM EDTA, 3 μM GDP) containing 0.1 nM ^{35}SGTPγS from Revvity, (Waltham, MA) (with specific activity of 1250 Ci/mmol) with agonist at a total reaction volume of 200 μl for 1 hour at 25 °C. Termination of the reaction is achieved by rapid filtration and washing with ice cold water through a GF/B glass microfiber filter (Whatman® glass microfiber filters, Grade GF/B from Sigma Aldrich) using a 96-well plate harvester from Brandel Inc. (Gaithersburg, MD). Filters were punched into shallow 96-well plates compatible with the TopCount NXT Microplate Scintillation and Luminescence Counter from PerkinElmer Life Sciences (Waltham, MA) and air-dried filters are counted following addition of scintillation fluid (MicroscintTM-20, from Revvity). Two technical replicates were included in each individual experiment (n≥ 3).

Forskolin-stimulated cAMP accumulation assay was conducted using the CISBIO homogeneous time-resolved florescence (HTRF) cAMP kit (CISBIO HTRF cAMP Gs HiRange from Revvity) was used to assess cAMP levels as previously described in detail.[5,6] HTRF ratios (fluorescence at 665 nm/ fluorescence at 620 nm) were determined using a BioTek Synergy Neo2 Hybrid Multimode reader (Agilent, Santa Clara, CA) as a measure of inhibition of forskolin (20 μM) cAMP accumulation. Three technical replicates were included in each individual experiment (n≥ 3).

The βarrestin2 recruitment (DiscoverX PathHunter™) from MilliporeSigma (Burlington, MA) assay was performed in PathHunter U2OS OPRK1 β-Arrestin or PathHunterU2OS OPRM1 β-Arrestin cells according to manufacturer's protocol and as previously described.[5,8]

Luminescence values are determined by using a BioTek Synergy Neo2 Hybrid Multimode reader. All compounds were run in triplicate per assay and normalized to vehicle-treated cells; three technical replicates were included in each individual experiment (n≥ 3).

Animals Male C57BL/6J mice were purchased from The Jackson Laboratory. Adult mice are used between 10 and 15 weeks of age and are only used once per assay. Mice are kept on a 12-hour light/dark cycle in a temperature-controlled room and are group-housed (three to five mice per cage). All behavioral tests were performed during the light cycle between 8am-6pm. For drug treatments mice are injected with compound or vehicle at 10 μl/g body weight. Injections were administered intraperitoneal (i.p.). Experimenters conducting injections and performing measurements are blinded to the treatment groups. The vehicle for animal studies is DMSO, Tween 80, and 0.9% sterile saline (1:1:8). To facilitate solubility, the compounds are prepared by dissolving drug powder in DMSO, adding warm (50°C) Tween80, then 0.9% saline and immediately vortexing; solutions were made and used immediately before each study. All experiments conducted with mice are approved by the Institutional Animal Care and Use Committee at The Herbert Wertheim UF Scripps Institute for Biomedical Innovation & Technology (Jupiter, FL) and adhere to the National Institutes of Health Animal Care guidelines.

Locomotor activity The Versamax Animal Activity Monitoring System (20 cm × 20 cm x 30.5 cm) from Accuscan Instruments (Columbus, OH) was used to assess open-field locomotor activity as previously described.[6,7] The system consists of a photocell-equipped automated open field chamber contained inside sound-mitigating boxes to record locomotor activity. The Versadat software from Accuscan Instruments records the overall activity (distance traveled, beam breaks, center time, and center distance) in 5-minute intervals. Without habituation, mice are injected with vehicle or KOR agonist (injection indicated in figures) and placed immediately into the locomotor motor activity box after injection to record spontaneous locomotor activity over 60 minutes.

Statistical and data analyses (*in vitro*) Concentration–response curves were analyzed with GraphPad Prism 10 software (GraphPad Software Inc., San Diego, CA) using a three-parameter, nonlinear regression analysis with data normalized to vehicle and maximal U96,593 stimulation. Individual experiments were performed in duplicate and normalized to vehicle-treated cells; data are presented as means of 3 or more individual experiments (mean with S.E.M. data are graphed; EC50 values with 95% CI are presented in the tables). Bias analysis was carried out according by fitting to the operational model as we have described in detail.[5,6,8]

Statistical and data analyses (*in vivo*) Comparison of potency values are determined by applying an extra sum-of-squares F test (p=0.05) between two curves within the nonlinear regression analysis using Graphpad Prism. In the figures, raw data are presented as the individual animals and with means ± S.E.M.; effects are compared to vehicle by one-way ordinary ANOVA with a Dunnett's post-hoc comparison; comparison between drugs is made by Tukey post-hoc comparison, significance was set at $p<0.05$. Animal numbers are indicated in the figure legends.

Radio ligand binding Membranes were prepared from KOR- or MOR- expressing stable CHO-K1 cell lines as previously described in Schmid et al., 2017.[8] 3H-Naloxone was provided by the National Institute on Drug Abuse Drug Supply Program and was used at 2 nM per assay with a specific activity of 48.19 ci/mmol.

ED50 Calculation For normalization, the mean sum of vehicle activity measures defined the bottom while the mean of the sum of the activity measures produced by 15 mg/kg U50,488H defined the maximum response. Normalized data were fit to a hyperbolic nonlinear regression analysis constraining the maximum response to 100% and the minimum to 0%. Data are presented as the mean with 95% CI from the curve analysis.

Pharmacology Data

Table S1. cAMP inhibition potencies as % max U69,593 response. Note *a*: ^{35}GTPγgS binding assay.

Name	pEC50, M		Emax, %U69		n	A	B	Full Structure
	#	S.E.M	#	S.E.M				
RS1188	5.52	0.22	96	18	3	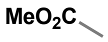	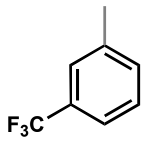	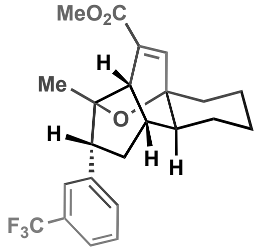
RS1137	5.59	0.19	54	8	3	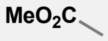	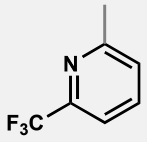	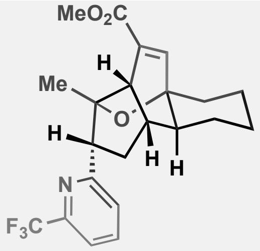
RS1132	5.72	0.19	48	7	3	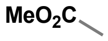	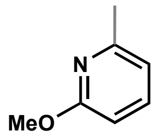	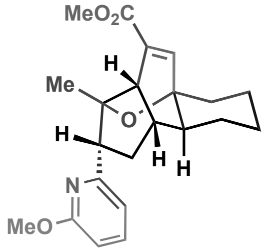
RS1133	5.77	0.40	29	7	3	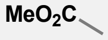	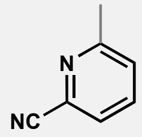	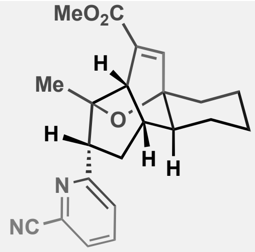
RS1120	5.78	0.12	76	5	6	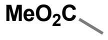	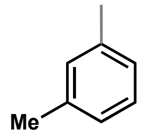	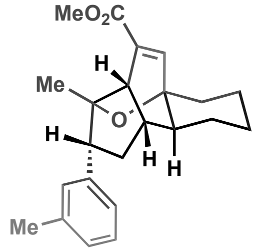
RS1136	5.81	0.18	59	7	3	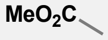	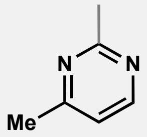	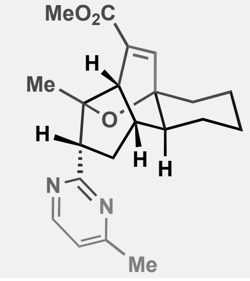

RS1131	5.83	0.11	65	5	3	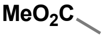	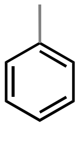	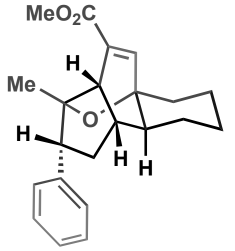
RS1138	5.91	0.19	63	8	3	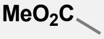	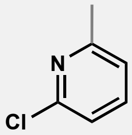	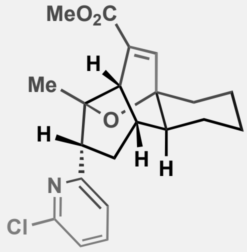
RS1122	5.92	0.11	43	3	2	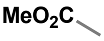	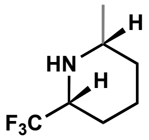	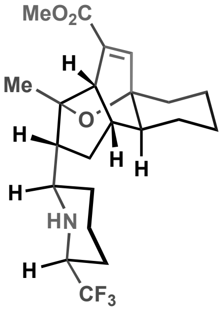
RS1135	5.93	0.32	26	5	3	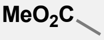	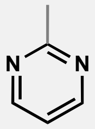	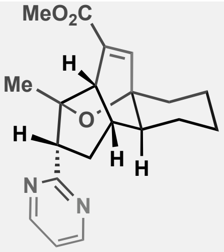
RS1161	5.95	0.19	49	6	5	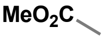	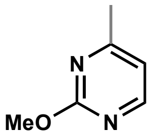	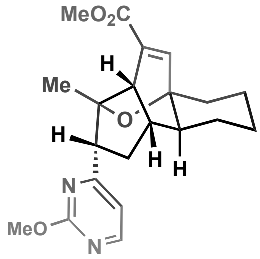
RS1123	5.98	0.16	66	6	6	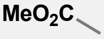	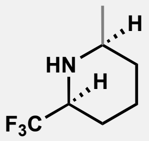	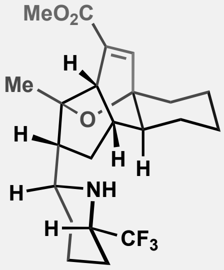
RS1134	6.20	0.37	22	4	3	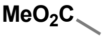	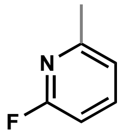	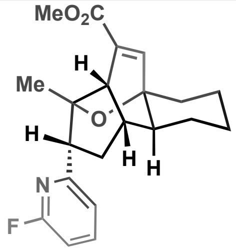

RS1165	6.31	0.19	79	8	3	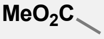	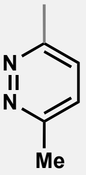	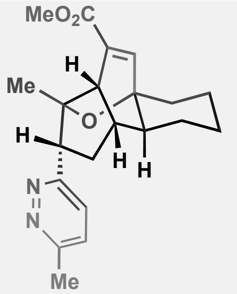
GBFZI-110[a]	6.64		21		1	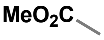	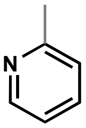	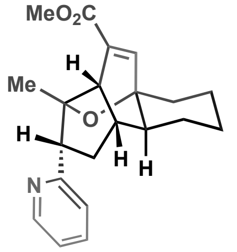
RS1130	6.33	0.26	50	6	5	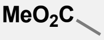	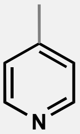	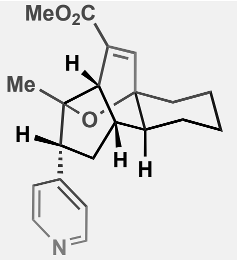
nat-pyr-GB18	6.32	0.29	56	8	5	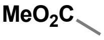	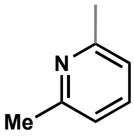	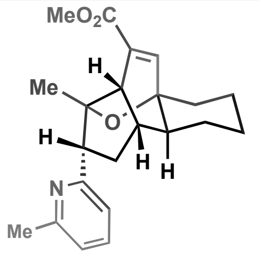
RS1121	6.46	0.22	18	2	2	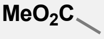	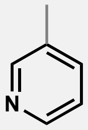	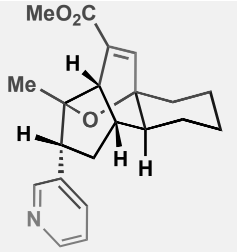
RS1167	6.51	0.28	69	9	3	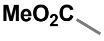	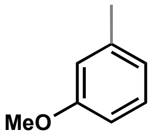	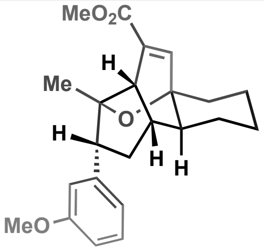

RS1187	6.92	0.18	92	7	3	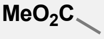	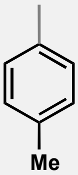	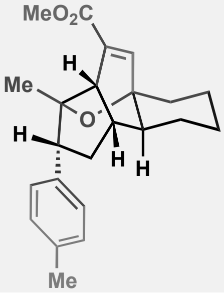
RS1168	7.01	0.17	99	6	3	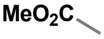	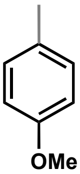	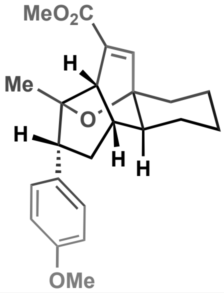
RS1129	7.06	0.19	64	4	6	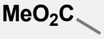	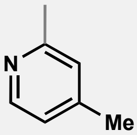	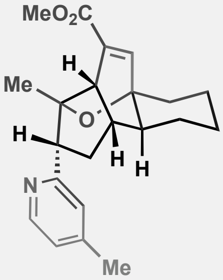
RS1139	7.39	0.10	107	4	4	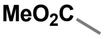	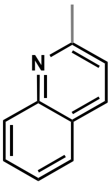	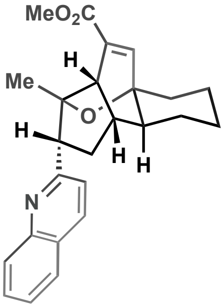
RS1166 (29)	7.42	0.16	96	5	3	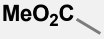	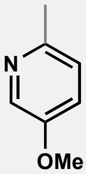	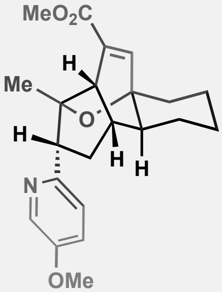
RS1128	7.70	0.11	101	3	6	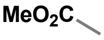	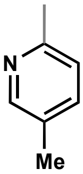	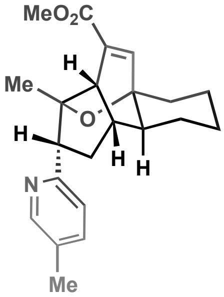

RS1162 (33)	8.43	0.13	102	4	3	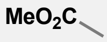	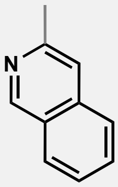	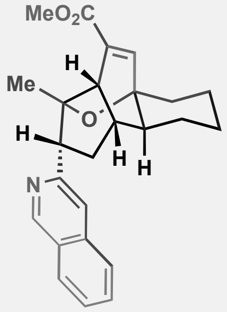
RS1163 (30)	8.56	0.14	102	4	3	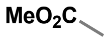	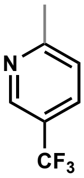	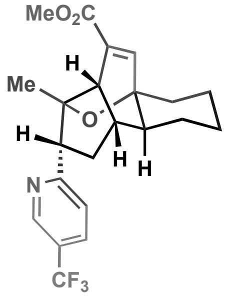
RS1125	6.67	0.19	72	6	5	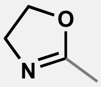	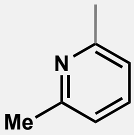	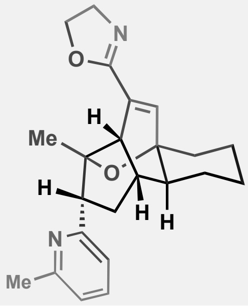
RS1160 (28)	8.37	0.11	109	3	3	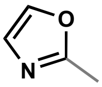	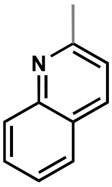	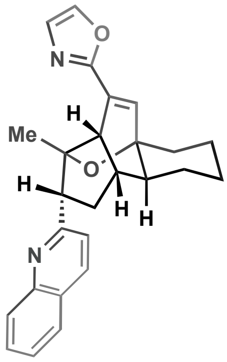
RS1159 (27)	9.17	0.09	107	3	9	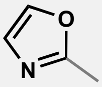	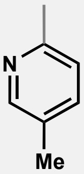	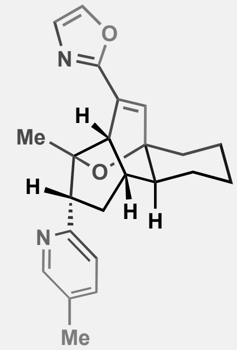

RS1176 (26)	9.36	0.10	109	3	4	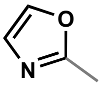	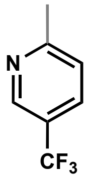	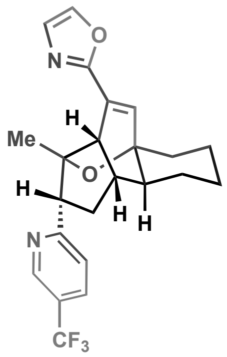

Table S2. Spontaneous locomotor activity assessment in the open field.

TIMECOURSE (Fig 6a) Two-way RM ANOVA

Figure	Treatment	Compared to	Time	F (DFn, DFd) time x dose	P-Value	F (DFn, DFd) drug effect	P-Value	treatment n
6a	**all RS1176**	Vehicle	5 – 60	F (22, 176) = 1.748	**0.0254**	F (2, 16) = 9.555	**0.0019**	
	0.2 RS1176	Vehicle	5 – 60	F (11, 121) = 1.597	0.1078	F (1, 11) = 2.775	0.1239	6
	1 RS1176	Vehicle	5 – 60	F (11, 121) = 2.307	**0.0133**	F (1, 11) = 14.42	**0.0030**	8
	**all RS1162**	Vehicle	5 – 60	F (33, 253) = 1.323	0.1208	F (3, 23) = 0.3431	0.7944	
	1 RS1162	Vehicle	5 – 60	F (11, 121) = 0.5436	0.8699	F (1, 11) = 0.2848	0.6042	6
	3 RS1162	Vehicle	5 – 60	F (11, 143) = 1.172	0.3117	F (1, 13) = 0.8838	0.3643	8
	15 RS1162	Vehicle	5 – 60	F (11, 121) = 2.643	**0.0046**	F (1, 11) = 0.07394	0.7907	6
	**all RS1139**	Vehicle	5 – 60	F (33, 253) = 0.9118	0.6104	F (3, 23) = 1.315	0.2935	
	1 RS1139	Vehicle	5 – 60	F (11, 121) = 1.004	0.4474	F (1, 11) = 0.5087	0.4906	6
	3 RS1139	Vehicle	5 – 60	F (11, 143) = 0.4232	0.9438	F (1, 13) = 0.5596	0.4678	8
	15 RS1139	Vehicle	5 – 60	F (11, 121) = 1.271	0.2493	F (1, 11) = 1.466	0.2514	6

CENTER TIME (Fig 6b) Two-way RM ANOVA

Figure	Treatment	Compared to	Time	F (DFn, DFd) time x dose	P-Value	F (DFn, DFd) drug effect	P-Value	treatment n
6b	**all RS1176**	Vehicle	5 – 60	F (22, 176) = 0.9098	0.5823	F (2, 16) = 19.09	**0.0001**	
	0.2 RS1176	Vehicle	5 – 60	F (11, 121) = 0.5644	0.8543	F (1, 11) = 22.73	**0.0006**	6
	1 RS1176	Vehicle	5 – 60	F (11, 121) = 1.246	0.2643	F (1, 11) = 34.21	**0.0001**	8
	**all RS1162**	Vehicle	5 – 60	F (33, 253) = 1.232	0.1885	F (3, 23) = 12.30	**0.0001**	
	1 RS1162	Vehicle	5 – 60	F (11, 121) = 0.6127	0.8151	F (1, 11) = 10.60	**0.0077**	6
	3 RS1162	Vehicle	5 – 60	F (11, 143) = 1.494	0.1398	F (1, 13) = 22.68	**0.0004**	8
	15 RS1162	Vehicle	5 – 60	F (11, 121) = 1.876	**0.0490**	F (1, 11) = 29.64	**0.0002**	6
	**all RS1139**	Vehicle	5 – 60	F (33, 253) = 1.371	0.0934	F (3, 23) = 5.527	**0.0052**	
	1 RS1139	Vehicle	5 – 60	F (11, 121) = 1.492	0.1430	F (1, 11) = 16.62	**0.0018**	6
	3 RS1139	Vehicle	5 – 60	F (11, 143) = 1.335	0.2112	F (1, 13) = 6.282	**0.0263**	8
	15 RS1139	Vehicle	5 – 60	F (11, 121) = 0.7832	0.6563	F (1, 11) = 27.99	**0.0003**	6

Figure S8. KOR agonists effects on distance travelled in the center of the open-field and horizontal activity (beam breaks). Distance travelled in the center of the open-field over 1-hour did not differ between vehicle and drug treated mice. Data are presented as mean ± S.E.M; n=6 to 8 C57BL6/J mice per treatment; injections were administered intraperitoneal (i.p.)., dose indicated on x-axis. The sum of activity or time spent over 1 hour is presented in the bar charts showing statistical comparisons of dose vs. vehicle using ordinary one-way ANOVA with Dunnett's post-hoc test (**p<0.05, **p<0.01, ***p<0.001, ****p<0.000*).

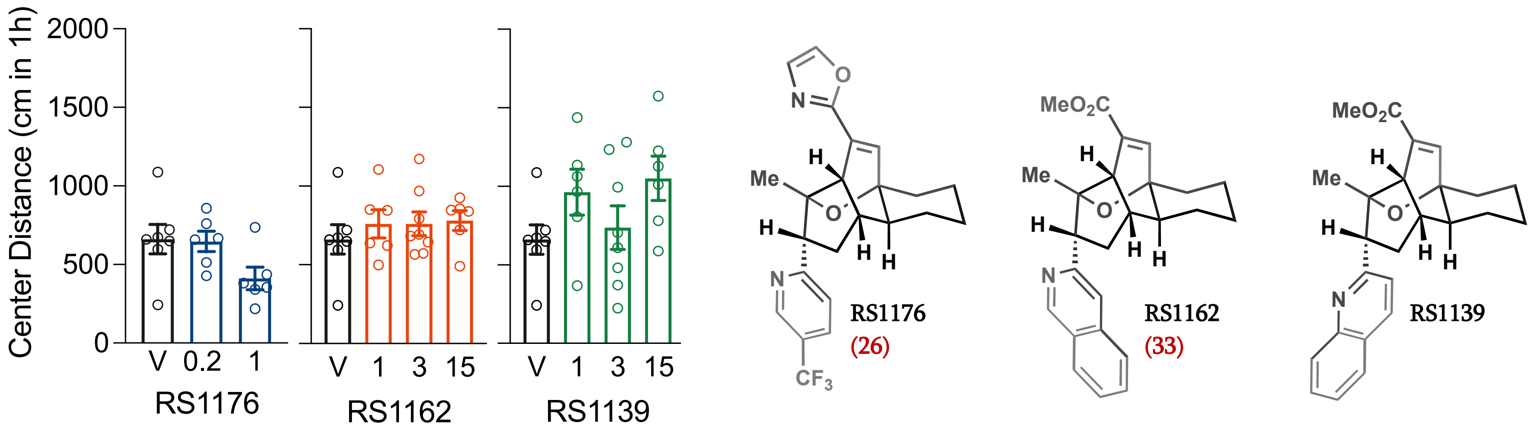

Figure S9. Radioligand competition binding in hKOR and hMOR cells. Membrane protein (10µg/well) were incubated with 2nM 3H-Naloxone and increasing concentrations of test ligands (see Table S4 for pKi) for 2 hours at room temperature; 1 µM Naloxone was used to define the nonspecific binding.

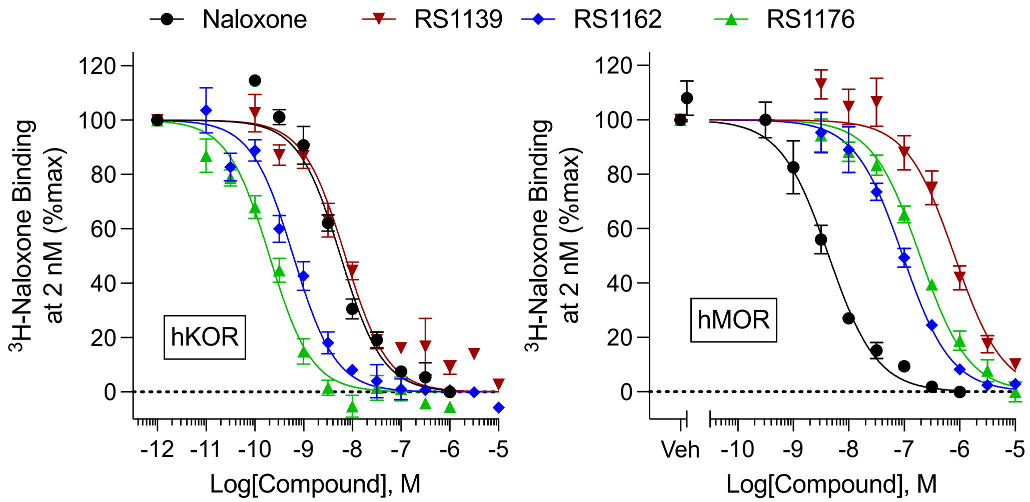

Table S3. **MOR agonist and antagonist potencies.**

	Antagonist MOR GTP vs 1 μM DAMGO				Agonist MOR βarr2 DX					Antagonist MOR Barr2 vs 1 μM DAMGO				Agonist MOR cAMP HTRF					Antagonist MOR cAMP vs 1uM DAMGO				Antagonist MOR cAMP vs 10nM DAMGO			
	IC50 (nM)	low 95% CI	high 95% CI	n	EC50 (nM)	low 95% CI	high 95% CI	Emax (%DAMGO)	n	IC50 (nM)	low 95% CI	high 95% CI	n	EC50 (nM)	low 95% CI	high 95% CI	Emax (%DAMGO)	n	IC50 (nM)	low 95% CI	high 95% CI	n	IC50 (nM)	low 95% CI	high 95% CI	n
U69593 or damgo	-	-	-		358.92	307.2	419.3	100	9	-	-	-	-	8.69	6.5	11.8	100	9	-	-	-	-	-	-	-	-
NorBNI or naloxone	45.8	30.6	68.7	3	-	-	-	-	-	4.1	3.0	5.5	9	-	-	-	-	-	220.1	120.4	405.4	8	8.3	5.1	13.6	6
RS1188	-	-	-	-	nc	-	-	-	1	nc	-	-	2	nc	-	-	-	1	-	-	-	-	nc	-	-	1
RS1137	-	-	-	-	nc	-	-	-	1	3600.3	-	-	2	nc	-	-	-	1	nc	-	-	1	-	-	-	-
RS1132	-	-	-	-	nc	-	-	-	1	>10⁴	poor fit	-	1	nc	-	-	-	1	nc	-	-	1	-	-	-	-
RS1133	-	-	-	-	nc	-	-	-	1	665.2	-	-	2	nc	-	-	-	1	>10⁴	poor fit	-	1	-	-	-	-
RS1120	-	-	-	-	nc	-	-	-	1	nc	-	-	1	nc	-	-	-	2	nc	-	-	1	-	-	-	-
RS1136	-	-	-	-	nc	-	-	-	1	nc	-	-	1	nc	-	-	-	1	nc	-	-	1	-	-	-	-
RS1131	-	-	-	-	nc	-	-	-	1	nc	-	-	1	nc	-	-	-	1	nc	-	-	1	-	-	-	-
RS1138	-	-	-	-	nc	-	-	-	1	1163.3	-	-	2	nc	-	-	-	1	nc	-	-	1	-	-	-	-
RS1122	-	-	-	-	nc	-	-	-	1	>10⁴	poor fit	-	3	nc	-	-	-	1	nc	-	-	2	-	-	-	-
RS1135	-	-	-	-	nc	-	-	-	1	nc	-	-	1	nc	-	-	-	1	nc	-	-	1	-	-	-	-
RS1161	-	-	-	-	nc	-	-	-	1	1486.9	1090.4	2038.2	3	nc	-	-	-	1	nc	-	-	1	-	-	-	-
RS1123	-	-	-	-	nc	-	-	-	1	>10⁴	poor fit	-	3	nc	-	-	-	1	nc	-	-	2	-	-	-	-
RS1134	-	-	-	-	nc	-	-	-	1	2878.3	-	-	2	nc	-	-	-	1	nc	-	-	1	-	-	-	-
RS1165	-	-	-	-	nc	-	-	-	1	207.7	104.2	414.2	3	nc	-	-	-	1	6187.2	poor fit	-	1	129.9	46.1	354.2	3
GBFZ1-110	>10⁴	-	-	1	nc	-	-	0*	1	3667.2	1718.9	9072.6	1	not tested	-	-	-	-	not tested	-	-	-	-	-	-	-
NAT-pyrGB18	>10⁴	poor fit	-	5	nc	-	-	-	1	4501.1	2359.9	10013.7	3	nc	-	-	-	2	nc	-	-	2	-	-	-	-
RS1130	-	-	-	-	nc	-	-	-	1	2118.2	1206.3	3860.0	3	nc	-	-	-	1	nc	-	-	2	-	-	-	-
RS1121	-	-	-	-	nc	-	-	-	1	2085.2	1316.5	3394.6	3	nc	-	-	15	1	nc	-	-	1	-	-	-	-
RS1167	-	-	-	-	nc	-	-	-	1	nc	-	-	1	nc	-	-	-	1	nc	-	-	1	-	-	-	-
RS1187	-	-	-	-	nc	-	-	-	1	nc	-	-	2	nc	-	-	-	1	-	-	-	-	nc	-	-	1
RS1168	-	-	-	-	nc	-	-	-	1	>10⁴	poor fit	-	1	nc	-	-	-	1	nc	-	-	1	-	-	-	-
RS1129	-	-	-	-	nc	-	-	-	1	4038.9	1966.9	9954.2	3	nc	-	-	-	1	nc	-	-	2	-	-	-	-
RS1139	-	-	-	-	nc	-	-	-	1	1075.9	-	-	2	nc	-	-	-	3	nc	-	-	2	-	-	-	-
RS1166	-	-	-	-	nc	-	-	-	1	935.6	-	-	2	nc	-	-	-	1	nc	-	-	1	-	-	-	-
RS1128	-	-	-	-	nc	-	-	-	1	2629.9	1361.3	5522.1	3	nc	-	-	-	1	nc	-	-	2	-	-	-	-
RS1162	-	-	-	-	nc	-	-	-	1	278.4	188.8	407.8	3	nc	-	-	-	1	nc	-	-	1	-	-	-	-
RS1163	-	-	-	-	nc	-	-	-	1	1142.2	801.9	1630.8	3	nc	-	-	-	1	nc	-	-	1	-	-	-	-
RS1125	-	-	-	-	nc	-	-	-	1	1632.1	922.1	2981.3	3	nc	-	-	-	1	nc	-	-	2	-	-	-	-
RS1160	-	-	-	-	nc	-	-	-	1	524.2	391.7	699.8	3	nc	-	-	-	1	nc	-	-	1	-	-	-	-
RS1144 RS1159	-	-	-	-	nc	-	-	-	3	647.8	521.7	804.6	6	nc	-	-	-	4	nc	-	-	3	-	-	-	-
RS1176	-	-	-	-	nc	-	-	-	1	1102.9	770.0	1585.0	2	nc	-	-	-	1	-	-	-	-	588.2	350.0.0	994.0	3

Table S4. Radioligand displacement assay at KOR and MOR for **RS1176**, **RS1162**, and **RS1139**; and pIC50 potencies (MOR βarr2 vs 1 μM DAMGO). The radioligand binding assay was performed in cell membranes from the same cell lines that were used in the functional assays. ^{3}H-Naloxone (2nM) was used for the competition curves, 10 μM cold naloxone was used to define the non-specific binding. Shown are the mean with S.E.M of three independent experiments performed in duplicate (average of the K_i or K_d).

	KOR		MOR			
	K_d or K_i (M)	S.E.M	K_d or K_i (M)	S.E.M	pIC50 (M)	S.E.M
**Naloxone**	8.68	0.030	9.16	0.225	8.42	0.03
**RS1176**	9.85	0.083	7.20	0.195	5.97	0.22
**RS1162**	9.46	0.097	7.72	0.190	6.61	0.06
**RS1139**	8.59	0.098	6.88	0.240	5.94	0.05

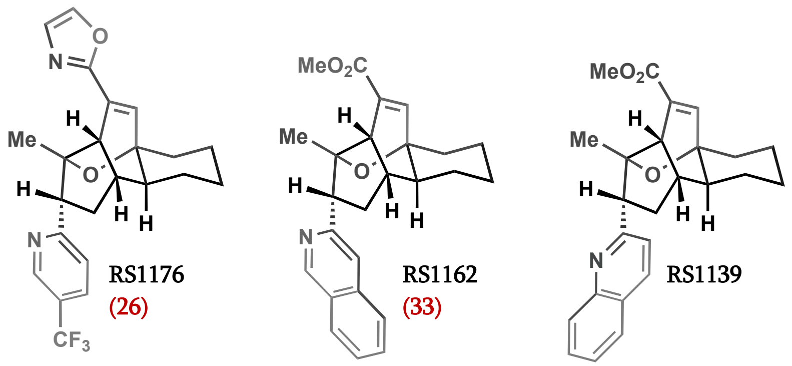

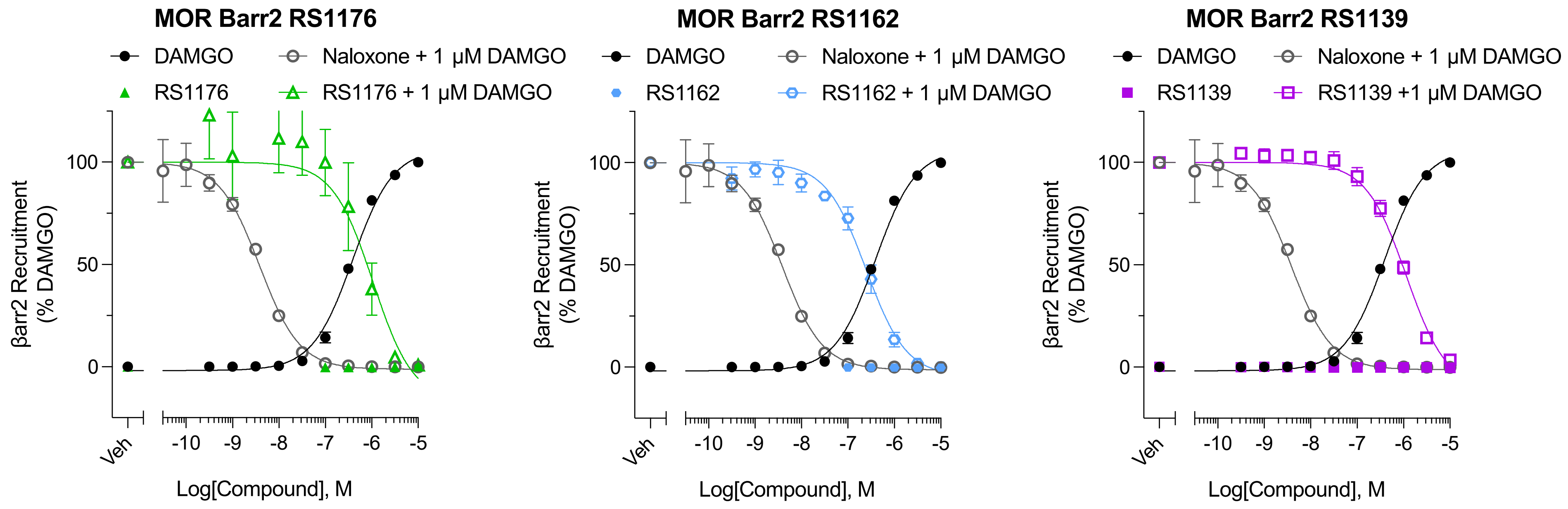

PDSP's PRESTO-Tango GPCRome

Well-information (OPRK1, KOR; PDSP assay book)

2	CALCRb	44	SSTR5	86	GAL1	127	FFA1	169	CASR	211	NMUR2	253	GPR75	295	MRGPRX2
3	CALCRL	45	Empty	87	SSTR2	128	CYSLTR2	170	LPA4	212	S1PR1	254	GPRC5A	296	GPR82
4	GPR151	46	GRM5	88	CHRM4	129	GPER	171	CMKLR1	213	CXCR7	255	GPR123	297	GPR87
5	GAL2	47	ADRB3	89	AVPR1A	130	NMUR1	172	LPAR5	214	GPR32	256	HTR1D	298	P2RY8
6	LHCGR	48	CRHR1	90	PK1	131	F2R-L1	173	FFA3	215	GPR22	257	GPR153	299	GPR6
7	CX3CR1	49	Empty	91	GPR119	132	CNR1	174	GPR174	216	HTR2CVGV	258	BB3	300	GPR35
8	QRFP	50	NPFF1	92	MC2R	133	SSTR4	175	GLP1R	217	OXGR1	259	CHRM2	301	GPR17
9	GPRC5B	51	GPR20	93	ADORA2A	134	GPR113	176	MRGPRX1	218	ADRA2A	260	GPR68	302	GPR125
10	CCR6	52	AVPR1B	94	MC1R	135	HCA1	177	CCKAR	219	MC5R	261	GPR4	303	LPAR1
11	GPR148	53	GPR176	95	CNR2	136	F2RL3	178	CCR8	220	NPY5R	262	P2RY4	304	AGTR2
12	FPR2	54	GPR149	96	PTGER2	137	ADRB1	179	GPRC5D	221	GPR116	263	GPR12	305	HTR2C INI
13	RXFP3	55	GPR126	97	PTGDR	138	AGTR1	180	ADRA1A	222	EDNRA	264	S1PR2	306	GPR65
14	TAAR5	56	GPR162	98	GPR15	139	P2RY12	181	TACR1	223	GRM4	265	GPR182	307	F2RL2
15	GPR27	57	GPR61	99	ADRA1D	140	PRRP	182	NMBR	224	MTNR1A	266	GPR85	308	P2RY14
16	KISSPEPTIN	58	MLNR	100	DRD5	141	SCTR	183	P2RY10	225	HTR7	267	GPR63	309	FPR3
17	GPR114	59	MAS1L	101	CCR5	142	RXFP2	184	GPR158	226	NPBW2	268	GPR1	310	MAS1
18	MRGPRX3	60	C3AR1	102	TAAR2	143	FFA2	185	TAAR9	227	NPY2R	269	OPN5	311	GPR115
19	GPR120	61	OPN3	103	ADORA2B	144	HTR2C VNV	186	GRPR	228	ADCYAP1R1	270	GPR56	312	TAAR6
20	S1PR5	62	CHRM1	104	HRH2	145	CCKBR	187	HTR4	229	MRGPRF	271	GPRC6A	313	HTR2B
21	UTS2R	63	CXCR3	105	PTH1R	146	CRHR2	188	MTNR1B	230	GPBA	272	GPR64	314	P2RY13
22	HCTR2	64	MCHR1R	106	OXER1	147	GPR171	189	GPR3	**231**	**OPRK1**	273	CCR3	315	CXCR2
23	EDNRB	65	RXFP4	107	GRM7	148	NPY1R	190	LPAR6	232	TACR2	274	P2RY2	316	PTAFR
24	GPR62	66	CCRL2	108	GPR78	149	DRD4	191	CCR10	233	CHRM3	275	GPR55	317	HRH1
25	CXCR6	67	TBXA2R	109	MC3Rb	150	CCR2	192	TAAR8	234	GPR37L1	276	HCA2	318	GHSR
26	HCTR1	68	GPR21	110	HCA3	151	GPR142	193	FSHR	235	PTGER4	277	GPR19	319	HTR2C VSV
27	GABBR1	69	GRM8	111	VIPR2	152	GPR156	194	CXCR1	236	CXCR4	278	HTR1A	320	CHRM5
28	OPRL1	70	GPR25	112	NTSR2	153	NPFF2	195	OPRD1	237	GPR110	279	GPR77	321	HTR1E
29	ADRA2C	71	GPR150	113	CYSLTR1	154	GPR135	196	HRH4	238	HTR2A	280	GPR141		
30	P2RY11	72	SSTR3	114	GPRC5C	155	TSHR	197	GPR83	239	GLP2R	281	HTR1F		
31	BDKBR1	73	GRM6	115	CCR7	156	GPR45	198	BDKBR2	240	LTB4R2B	282	GPR132		
32	DRD3	74	SUCNR1	116	S1PR3	157	GPR50	199	GPR26	241	ADORA1	283	P2RY6		
33	PTGFR	75	OXTR	117	GPR97	158	LPAR2	200	MC4R	242	MRGPRG	284	ELTD1		
34	GIPR	76	ADRB2	118	OPRM1	159	CD97	201	GPR18	243	GPR152	285	GPR37		
35	PTGIR	77	GRM1	119	DRD1	160	VIPR1	202	ADRA1B	244	TA1	286	TACR3		
36	GHRHR	78	MCHR2R	120	PTGER1	161	GPR143	203	GPR124	245	AVPR2	287	GPR161		
37	NPY4R	79	GCGR	121	ADRA2B	162	PTH2R	204	P2RY1	246	PTGER3	288	HTR1B		
38	GNRHR	80	GPR157	122	HTR6	163	MRGPRX4	205	GPR34	247	GPR160	289	GPR84		
39	GPR111	81	GMR2	123	NPS	164	GPR39	206	SSTR1	248	GPR52	290	NTSR1		
40	HRH3	82	GAL3	124	NPBW1	165	FPR1	207	GPR44	249	ADORA3	291	S1PR4		
41	CCR4	83	PK2	125	GPR88	166	MRGPRD	208	APJ	250	GPR133	292	HTR5		
42	GPR146	84	GPR173	126	DRD2	167	GPR183	209	CXCR5	251	C5A	293	GPR31		
43	GPR144	85	LTB4R	127	RXFP1	168	CASR	210	MRGPRE	252	F2R	294	GPR101		

Primary Binding Assay for *nat*-Pyr-GB18 **2**

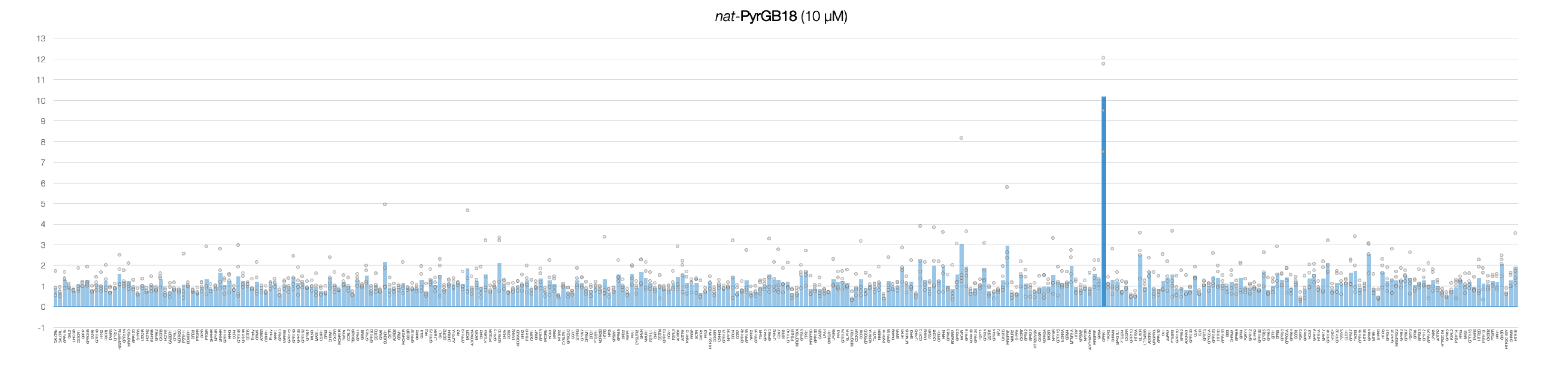

Fold over basal (average of triplicates, see S144)

2-21	22-41	42-61	62-81	82-101	102-121	122-141	142-161	162-181	182-201	202-221	222-241	242-261	262-281	282-301	302-321
0.93	0.78	1.50	1.45	1.31	0.90	1.36	1.16	1.22	1.22	0.73	1.17	0.91	0.93	1.19	1.09
0.72	1.04	1.26	1.08	0.72	1.03	0.72	0.67	0.67	0.48	0.90	1.23	0.87	0.91	1.13	1.31
1.31	0.84	1.08	0.81	1.34	1.10	0.83	0.77	0.71	1.25	1.06	1.97	1.25	0.96	1.22	1.03
0.99	1.34	0.97	1.21	1.06	1.28	1.57	1.16	1.52	0.93	1.85	0.97	1.41	0.84	1.67	0.70
0.79	0.72	1.23	1.02	1.56	0.89	1.02	0.75	1.73	1.02	0.76	0.84	1.59	1.69	1.76	0.55
1.11	0.78	0.87	0.62	0.88	1.04	0.68	1.08	0.86	1.93	0.73	0.99	0.88	0.82	0.93	0.83
1.07	0.92	0.77	1.32	1.17	1.39	1.60	0.91	0.89	1.10	0.77	0.95	0.95	1.02	1.03	0.95
1.30	0.76	1.06	0.99	1.10	0.73	0.95	1.01	0.89	1.24	1.28	1.59	0.82	1.67	2.56	1.31
0.79	1.08	1.15	1.51	1.13	1.28	1.69	1.49	0.76	0.56	2.97	1.39	0.81	1.31	0.92	1.07
1.11	1.05	0.66	0.82	1.11	1.12	1.41	0.77	0.71	2.30	0.71	10.20	1.51	1.42	0.52	1.19
1.07	0.77	1.07	1.07	1.87	0.51	1.18	0.88	1.35	1.29	0.67	1.14	0.67	0.94	1.71	0.96
1.26	0.71	1.00	0.77	0.95	1.02	0.90	1.29	1.21	0.96	1.55	1.31	1.13	1.23	1.25	1.40
0.80	1.01	1.45	2.17	1.27	0.74	0.96	0.68	1.26	2.01	1.16	1.19	1.01	0.45	1.34	1.16
0.94	1.35	1.17	0.95	0.97	0.69	0.89	0.73	1.16	1.31	0.83	0.82	1.50	0.87	1.27	1.11
1.62	0.85	1.17	0.89	1.59	1.30	0.87	0.97	0.43	1.96	0.76	1.03	1.40	1.38	1.31	1.27
1.27	0.96	0.99	0.95	0.82	1.18	1.16	1.06	0.97	1.02	0.93	0.51	1.15	1.62	1.49	1.19
1.27	1.66	1.00	1.17	1.00	0.86	1.45	1.59	1.34	0.87	1.01	0.57	0.87	0.95	1.41	2.07
0.97	1.05	0.97	0.88	2.12	0.81	1.62	1.32	0.91	1.58	0.99	2.53	1.19	1.39	1.06	0.69
0.67	1.26	0.76	0.93	1.12	1.01	1.18	1.33	1.09	3.07	1.56	0.98	0.98	2.14	1.22	1.29
1.06	0.86	0.74	0.91	0.79	0.87	1.08	1.19	0.96	1.96	1.19	1.74	1.41	0.84	1.30	1.94

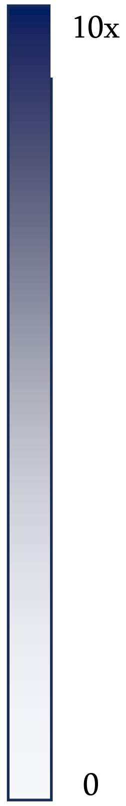

Secondary Binding Assay for *nat*-Pyr-GB18 **2**

**log[drug]**	**[^{3}H]-U69593 remaining binding, replicate no. / CPM**					
		***nat*-pyr-GB18**			**Salvinorin A**	
	1	2	3	1	2	3
-11.00	744	707	658	575	515	679
-10.00	686	888	741	682	618	678
-9.52	660	817	804	632	592	645
-9.00	701	722	745	746	695	714
-8.52	724	746	643	421	446	533
-8.00	633	671	628	283	288	286
-7.52	1001	478	483	105	118	101
-7.00	252	251	310	57	56	31
-6.52	104	112	87	17	22	48
-6.00	16	0	16	34	34	43
-5.52	12	35	35	37	25	23
-5.00	3	0	3	41	28	30

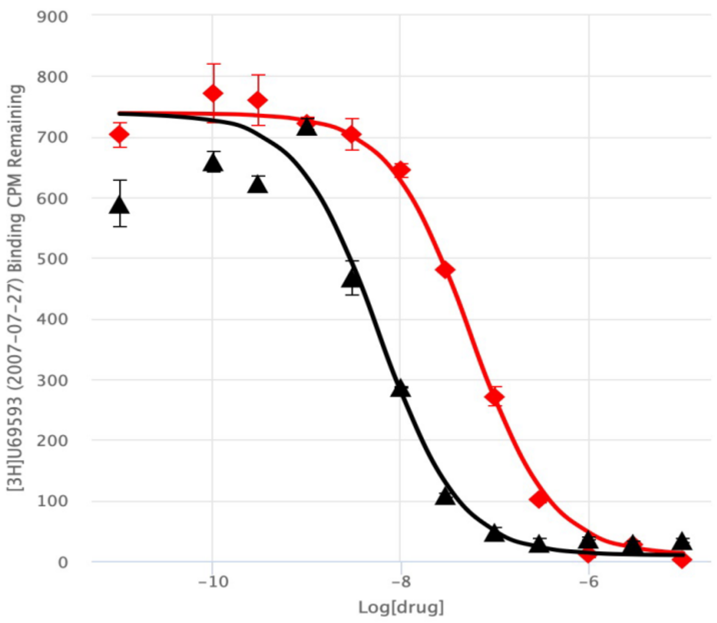

Primary Binding Assay for *ent*-pyr-GB18 **3**

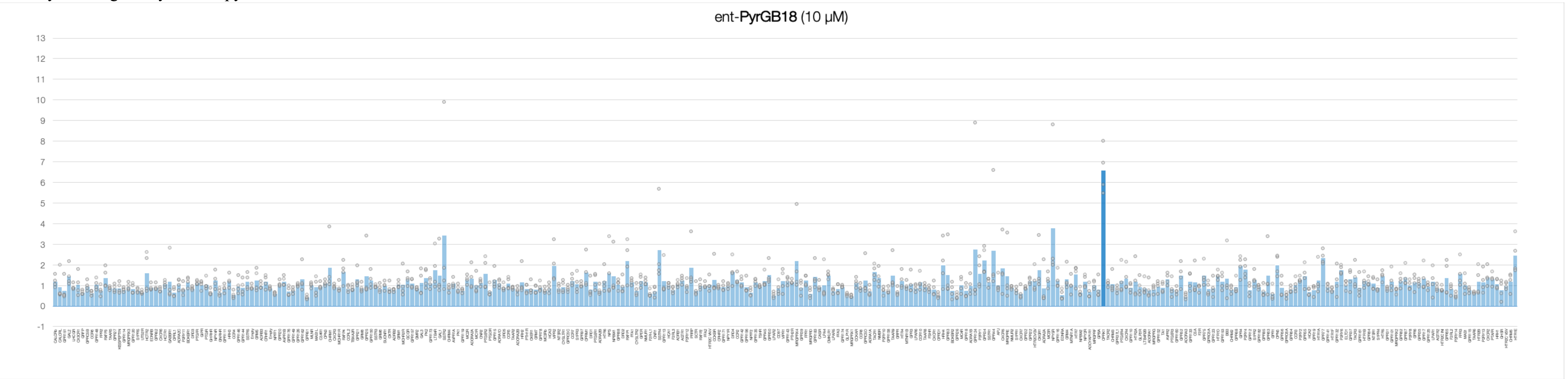

Fold over basal (average of triplicates, see S144)

2-21	22-41	42-61	62-81	82-101	102-121	122-141	142-161	162-181	182-201	202-221	222-241	242-261	262-281	282-301	302-321
1.19	1.62	0.97	1.87	1.13	0.88	1.21	0.76	1.23	1.37	0.98	0.56	0.81	1.78	1.19	0.97
0.93	0.90	0.91	0.94	1.36	0.75	1.61	1.00	1.16	0.74	2.76	1.30	0.95	0.79	1.75	1.19
0.77	0.91	1.18	0.90	1.12	1.16	1.47	0.99	2.20	0.75	1.58	1.01	0.91	1.29	1.03	0.88
1.46	0.78	0.90	1.66	1.75	0.83	1.06	1.04	0.90	1.47	2.23	1.54	1.30	0.93	1.30	0.82
0.92	1.08	1.26	0.85	1.49	0.85	0.97	1.28	1.24	0.68	1.17	0.67	0.84	0.72	1.40	1.37
1.04	1.19	1.00	0.87	3.44	0.80	2.20	0.92	0.62	1.22	2.69	1.19	0.73	1.50	0.89	0.84
0.65	0.66	1.18	1.30	0.85	0.91	1.18	0.93	1.43	0.98	1.02	0.69	1.50	0.61	1.21	0.53
0.96	1.08	0.87	0.88	1.10	0.76	0.75	0.99	0.94	1.07	1.85	1.03	0.47	1.98	1.17	1.57
0.64	0.80	0.60	1.47	0.83	0.86	1.22	1.67	1.02	0.88	1.47	0.82	1.20	0.90	1.11	1.03
1.04	1.17	1.07	1.26	0.75	1.97	1.18	1.28	1.50	1.15	0.90	6.59	1.15	0.52	0.90	0.77
0.75	0.85	1.21	0.95	1.28	0.86	0.60	1.16	0.73	0.93	0.76	1.08	0.88	0.80	1.46	0.63
1.37	1.15	0.73	0.85	1.34	0.94	0.71	0.92	0.83	0.82	0.62	1.08	1.48	1.01	0.83	1.20
0.86	1.02	0.75	0.99	1.02	0.83	2.74	0.66	0.93	0.81	0.87	0.80	0.85	1.27	1.21	1.17
0.86	1.06	1.02	0.85	1.15	1.23	1.24	1.17	0.54	0.51	1.23	1.23	0.87	1.47	0.74	1.41
0.88	0.65	1.30	0.77	1.58	1.07	1.02	0.96	0.49	2.00	1.23	1.34	1.48	0.68	1.22	1.31
0.79	1.24	0.41	1.04	0.72	1.03	0.79	1.22	1.14	1.52	1.75	0.89	1.19	0.79	1.40	1.08
0.94	0.67	1.00	1.06	1.27	1.62	1.06	1.52	0.89	0.74	0.87	1.20	1.35	1.26	1.01	0.78
0.83	0.85	0.93	1.31	1.10	0.81	1.26	0.52	1.24	0.50	1.26	0.93	0.82	2.36	1.16	1.00
0.76	1.21	0.97	1.09	0.89	1.20	0.96	0.87	0.72	1.03	3.80	0.88	0.92	0.94	1.13	1.20
0.66	0.54	1.10	0.86	1.09	0.77	1.86	1.23	1.66	0.74	1.26	0.78	1.95	0.79	1.34	2.46

10x

0

Secondary Binding Assay for *ent*-pyr-GB18 **3**

log[drug]	[³H]-U69593 remaining binding, replicate no. / CPM					
	ent-pyr-GB18			Salvinorin A		
	1	2	3	1	2	3
–11.00	698	882	614	575	515	679
–10.00	754	871	959	682	618	678
–9.52	699	875	798	632	592	645
–9.00	647	719	704	746	695	714
–8.52	707	690	801	421	446	533
–8.00	628	701	712	283	288	286
–7.52	712	690	763	105	118	101
–7.00	706	645	735	57	56	31
–6.52	538	638	663	17	22	48
–6.00	449	390	500	34	34	43
–5.52	197	221	342	37	25	23
–5.00	90	96	101	41	28	30

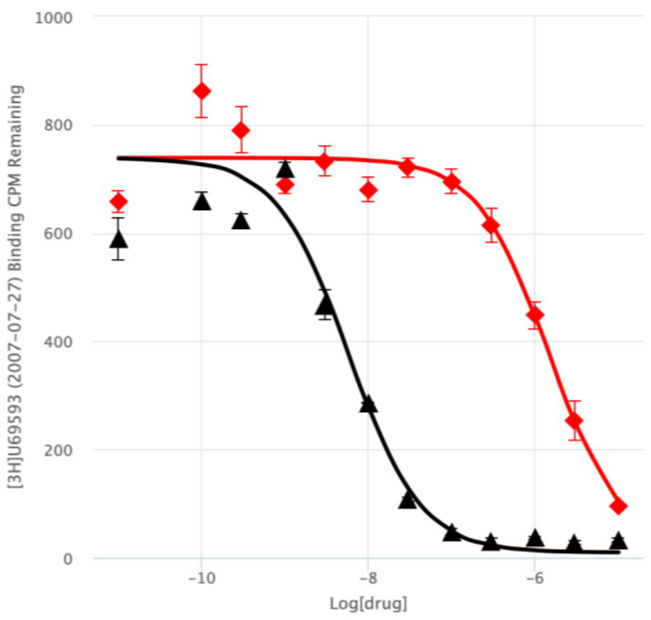

Reference

[1] Woo, S., & Shenvi, R. A. Synthesis and target annotation of the alkaloid GB18. *Nature* **2022**, *606* (7916), 917-921.

[2] Han, X.; Stoltz, B. M.; Corey, E. J. Cuprous Chloride Accelerated Stille Reactions. A General and Effective Coupling System for Sterically Congested Substrates and for Enantioselective Synthesis. *J. Am. Chem. Soc.* **1999**, *121*, 33, 7600–7605.

[3] Furstner, A.; Funel, J.-A.; Tremblay, M.; Bouchez, L. C.; Nevado, C.; Waser, M.; Ackerstaff, J.; Stimson, C. C. A Versatile Protocol for Stille–Migita Cross Coupling Reactions. *Chem. Commun*. **2008**, 2873–2875.

[4] Green, S. A.; Huffman, T. R.; McCourt, R. O.; van der Puyl, V.; Shenvi. R. A. Hydroalkylation of Olefins to Form Quaternary Carbons. *J. Am. Chem. Soc*. **2019**, *141*, 7709-7714.

[5] Brust, T. F.; Morgenweck, J.; Kim, S.A.; Rose, J. H.; Locke, J. L,; Schmid, C. L.; Zhou, L.; Stahl, E. L.; Cameron, M. D.; Scarry, S. M.; Aubé, J.; Jones, S. R.; Martin, T. J.; Bohn, L. M. Biased agonists of the kappa opioid receptor suppress pain and itch without causing sedation or dysphoria. *Sci. Signal.* **2016**, *9* (456), ra117.

[6] Volf, A.; Brust, T. F.; Kopbhylski, R. R.; Czekner, K. M.; Stahl, E. L.; Cameron, M. D.; Trojniak, A. E.; Aubé, J.; Bohn, L. M. "Triazole 187 is a biased KOR agonist that suppresses itch without sedation and induces anxiolytic-like behaviors in mice" *BioRxiv* DOI: 10.1101/2025.02.17.638680.

[7] Ho, J. H.; Stahl, E. L.; Schmid, C. L.; Scarry, S. M.; Aubé, J.; Bohn, L. M. G protein signaling- biased agonism at the kappa-opioid receptor is maintained in striatal neurons. *Sci. Signal.* **2018**, *11* (542), aar4309.

[8] Schmid, C. L.; Kennedy, N. M.; Ross, N. C.; Lovell, K. M.; Yue, Z.; Morgenweck, J.; Cameron, M. D.; Bannister, T. D.; Bohn, L. M. Bias factor and therapeutic window correlate to predict safer opioid analgesics. *Cell* **2017**, *171* (5), 1165-1175.

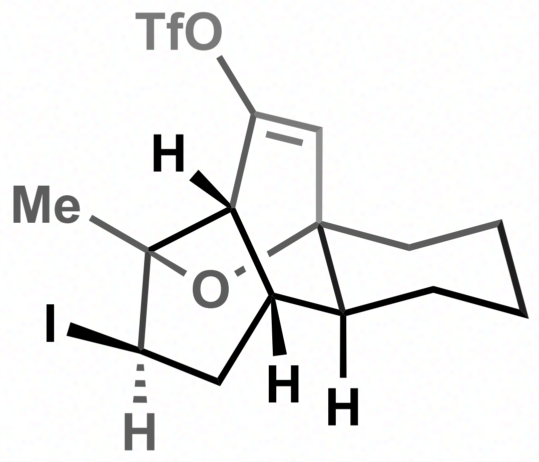

9
racemic
(600 MHz, C_6D_6)

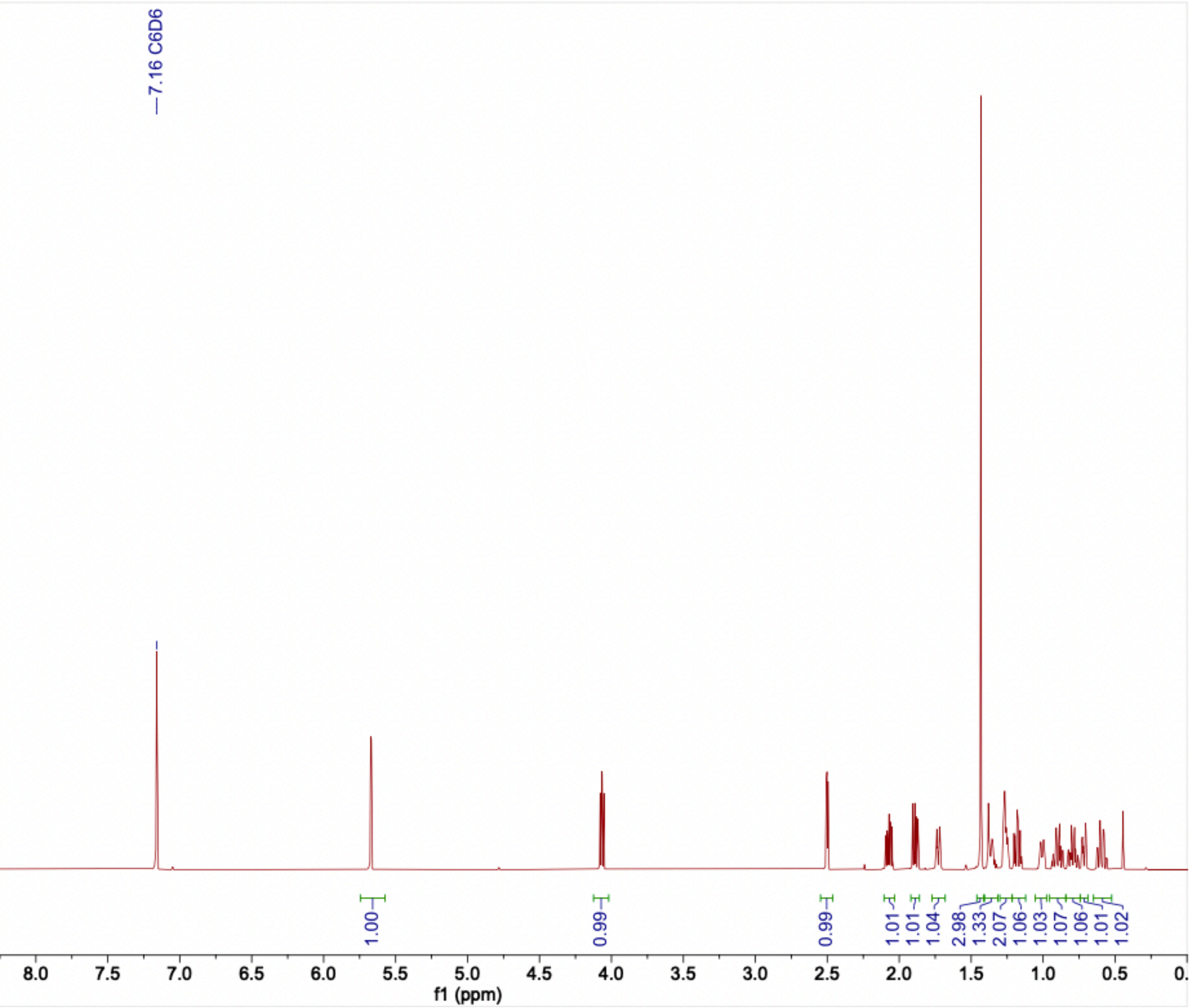

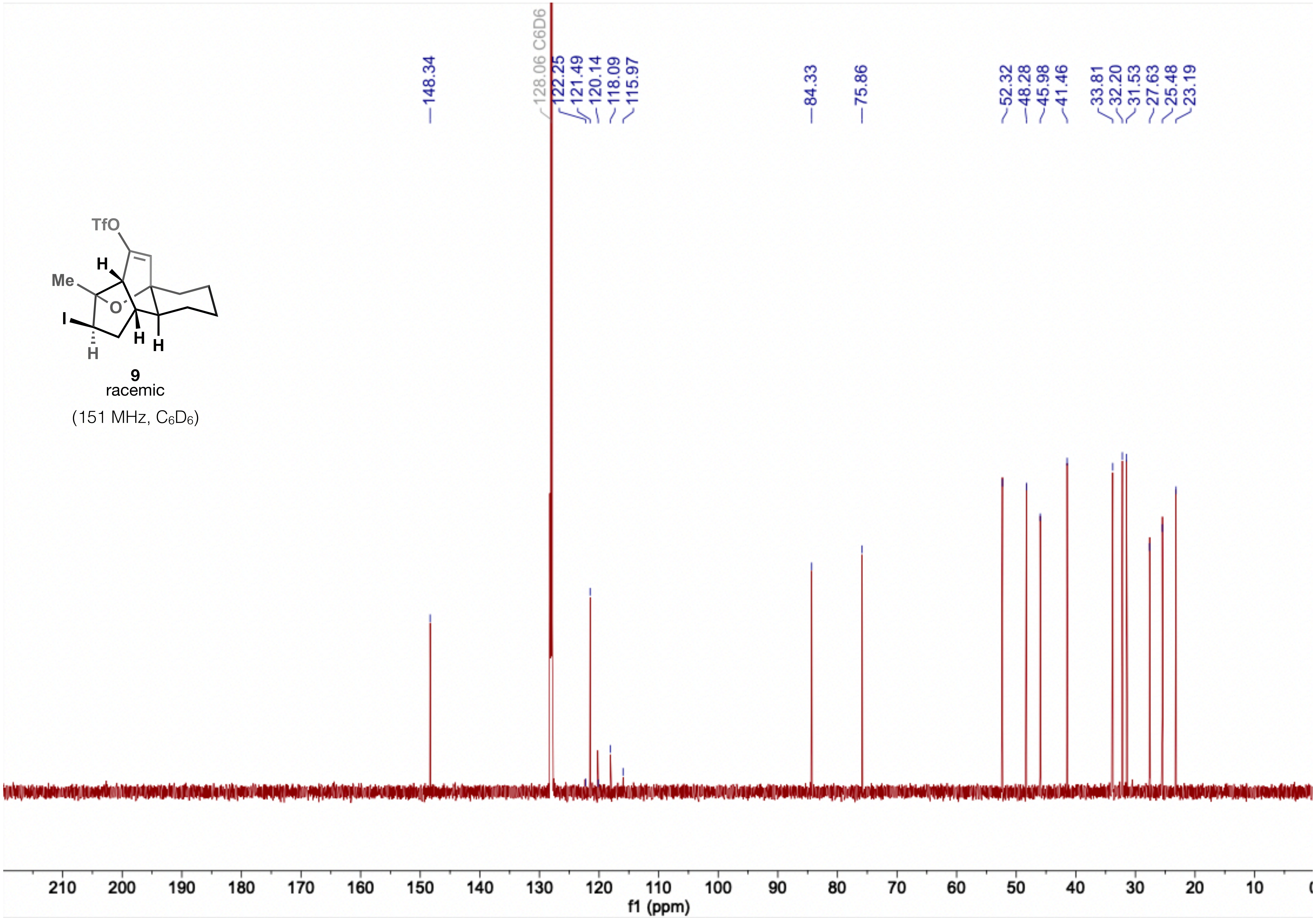

148.34
128.06 C6D6
122.25
121.49
120.14
118.09
115.97
84.33
75.86
52.32
48.28
45.98
41.46
33.81
32.20
31.53
27.63
25.48
23.19
TfO
H
Me
O
I
H
H
H
9
racemic
(151 MHz, C6D6)
210
200
190
180
170
160
150
140
130
120
110
100
90
80
70
60
50
40
30
20
10
f1 (ppm)

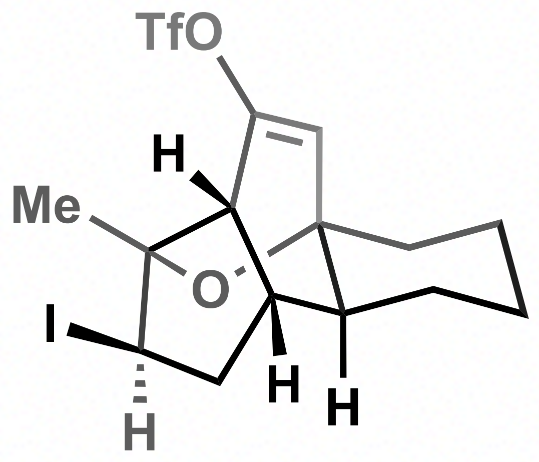

9
racemic
(376 MHz, C_6D_6)

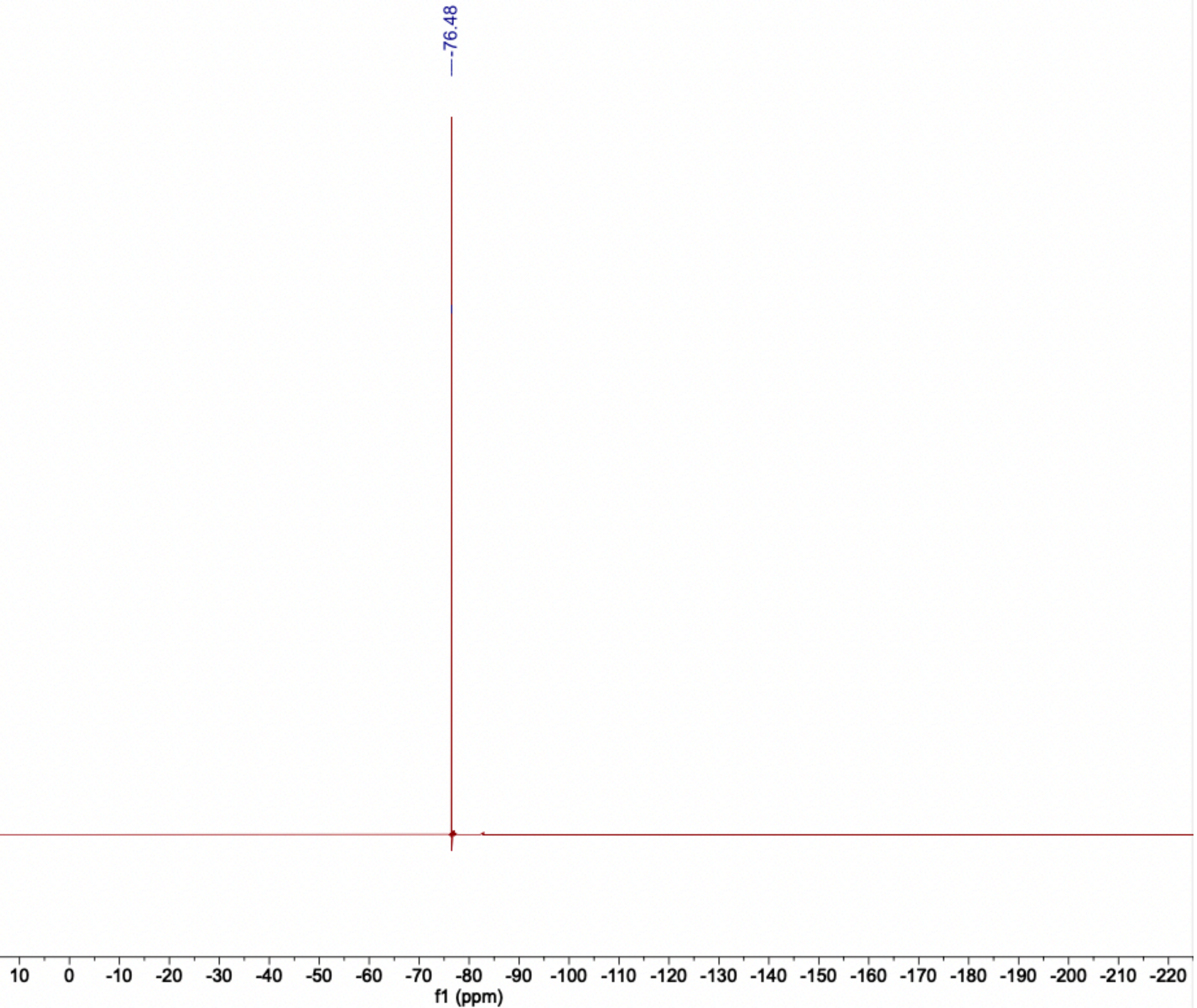

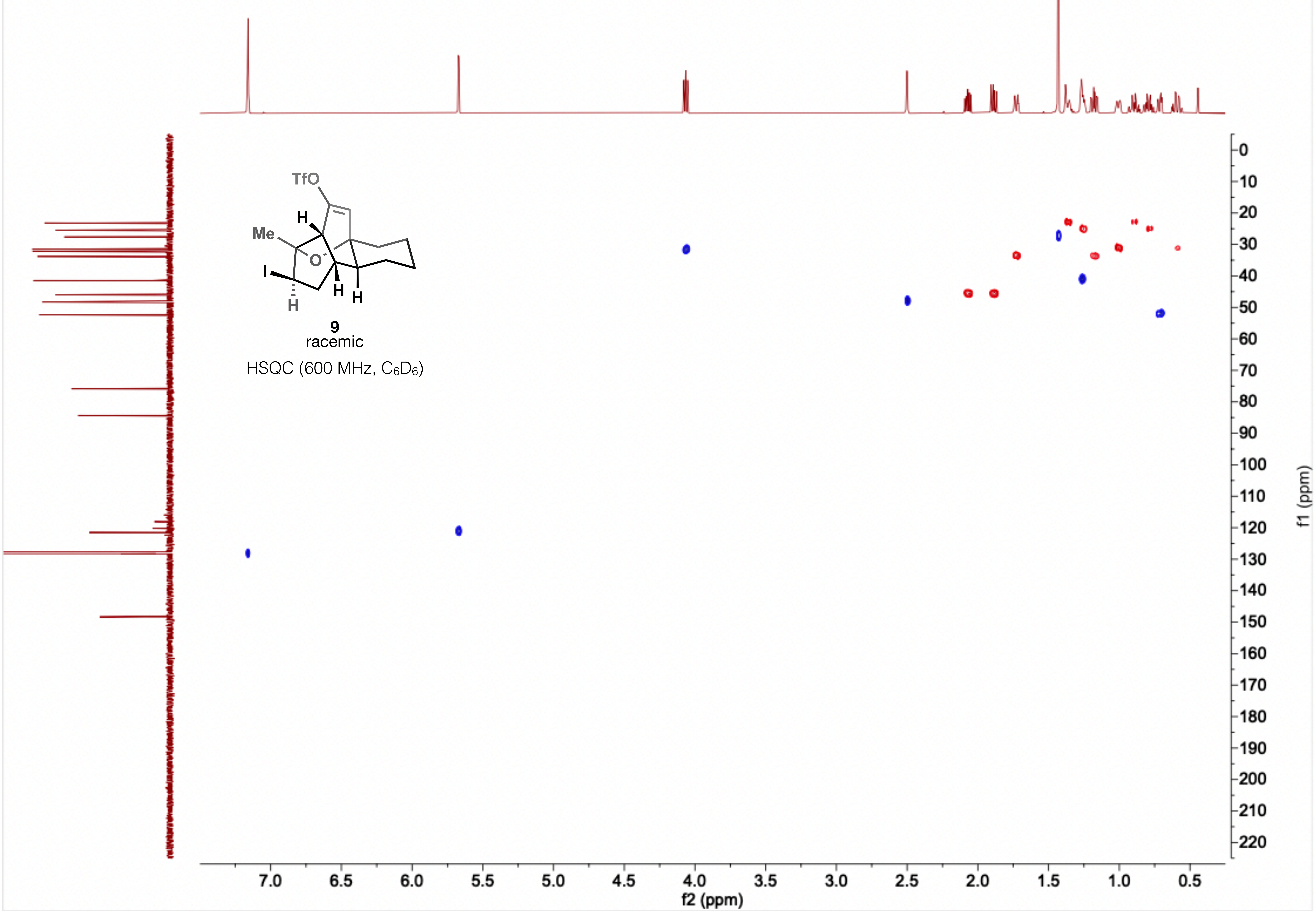
TfO
H
Me
O
I
H
H
H
9
racemic
HSQC (600 MHz, C6D6)
f1 (ppm)
f2 (ppm)
7.0 6.5 6.0 5.5 5.0 4.5 4.0 3.5 3.0 2.5 2.0 1.5 1.0 0.5
0 10 20 30 40 50 60 70 80 90 100 110 120 130 140 150 160 170 180 190 200 210 220

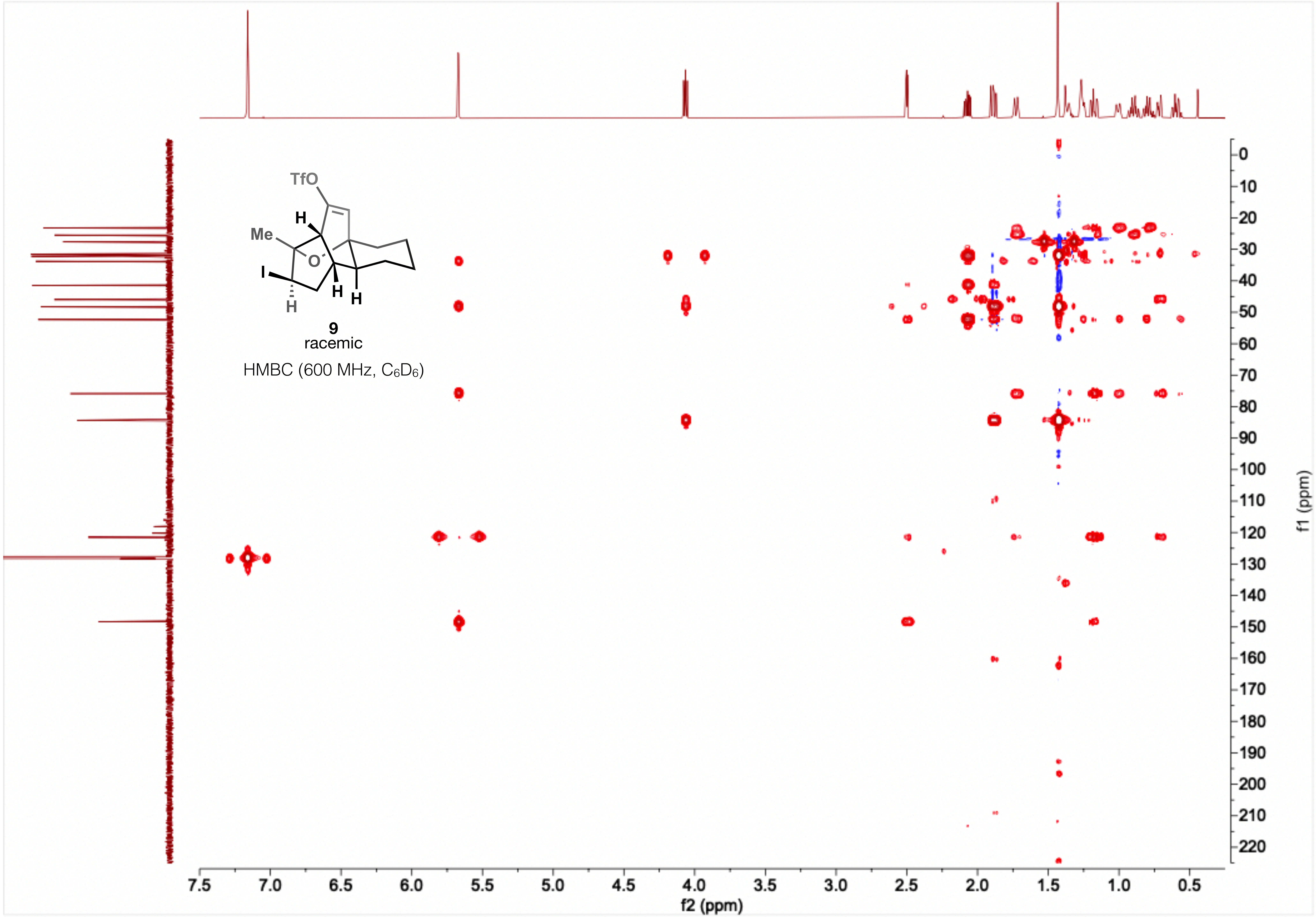

TfO
Me
O
I
H
9
racemic
HMBC (600 MHz, C6D6)
f2 (ppm)
f1 (ppm)

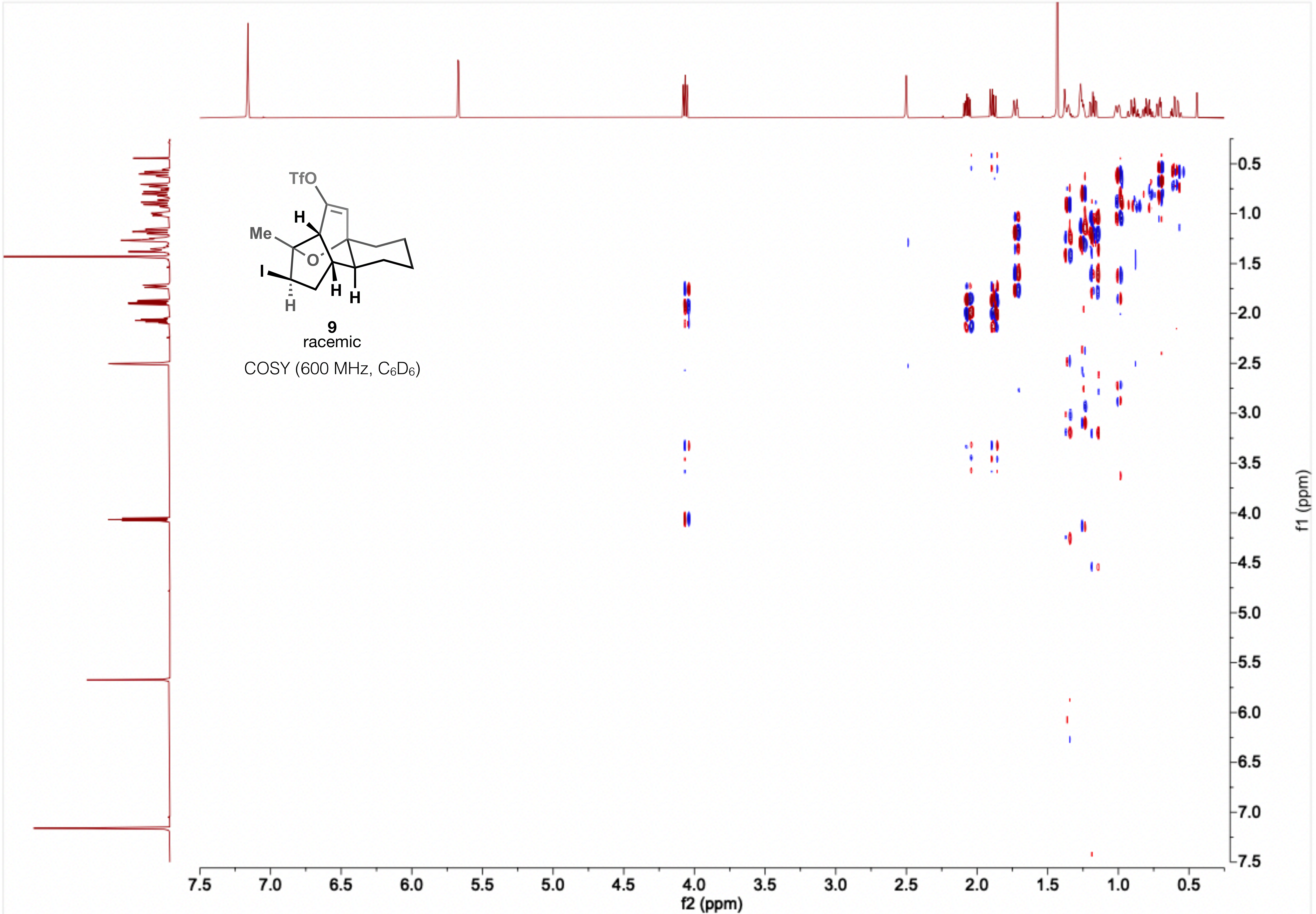
TfO
Me
H
O
I
H
H
H
9
racemic
COSY (600 MHz, C_6D_6)
f2 (ppm)
f1 (ppm)

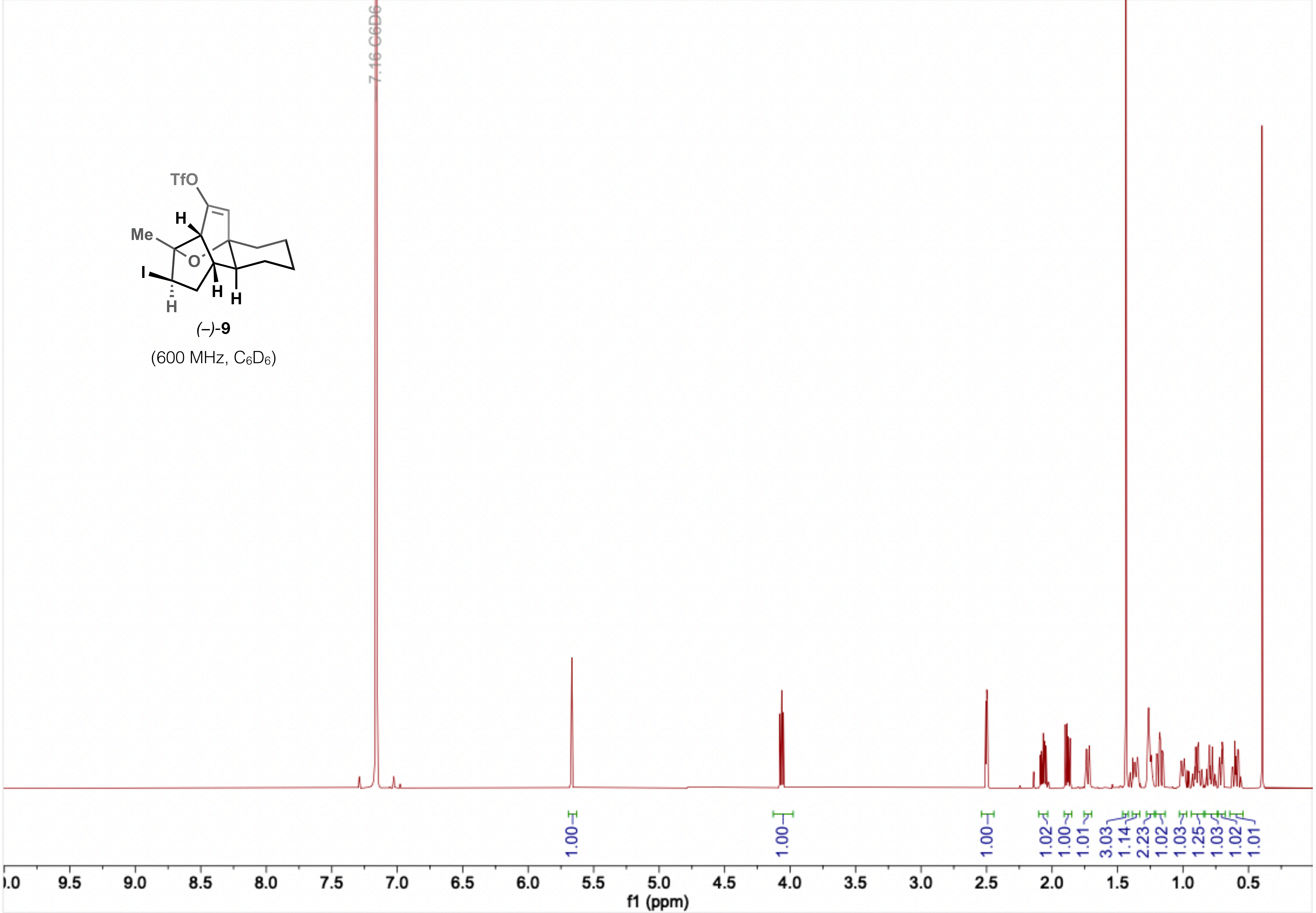

(–)-**9**

(600 MHz, C_6D_6)

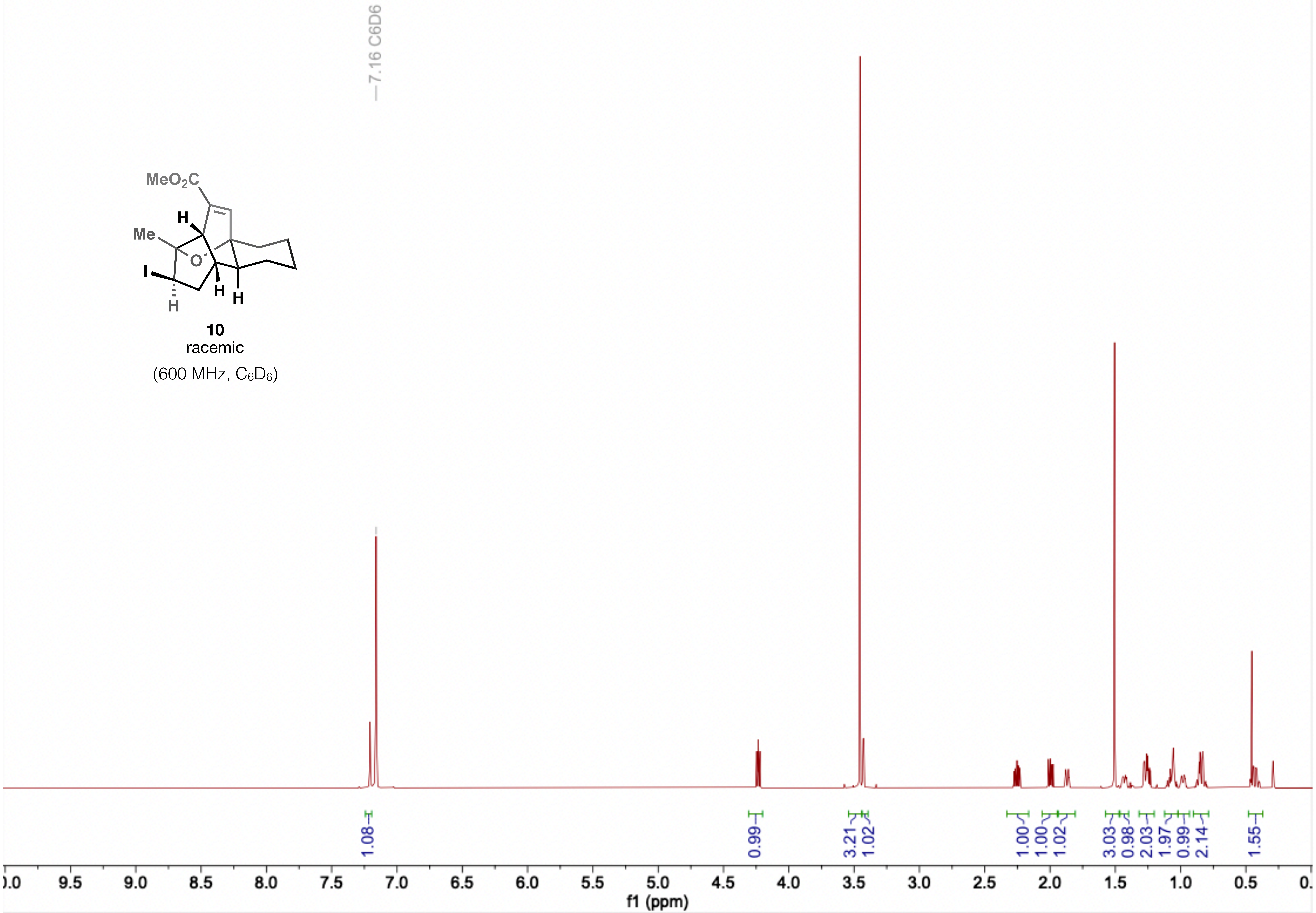
MeO2C
H
Me
O
I
H
H
H
10
racemic
(600 MHz, C6D6)
7.16 C6D6
1.08
0.99
3.21
1.02
1.00
1.00
1.02
3.03
0.98
2.03
1.97
0.99
2.14
1.55
9.5 9.0 8.5 8.0 7.5 7.0 6.5 6.0 5.5 5.0 4.5 4.0 3.5 3.0 2.5 2.0 1.5 1.0 0.5
f1 (ppm)

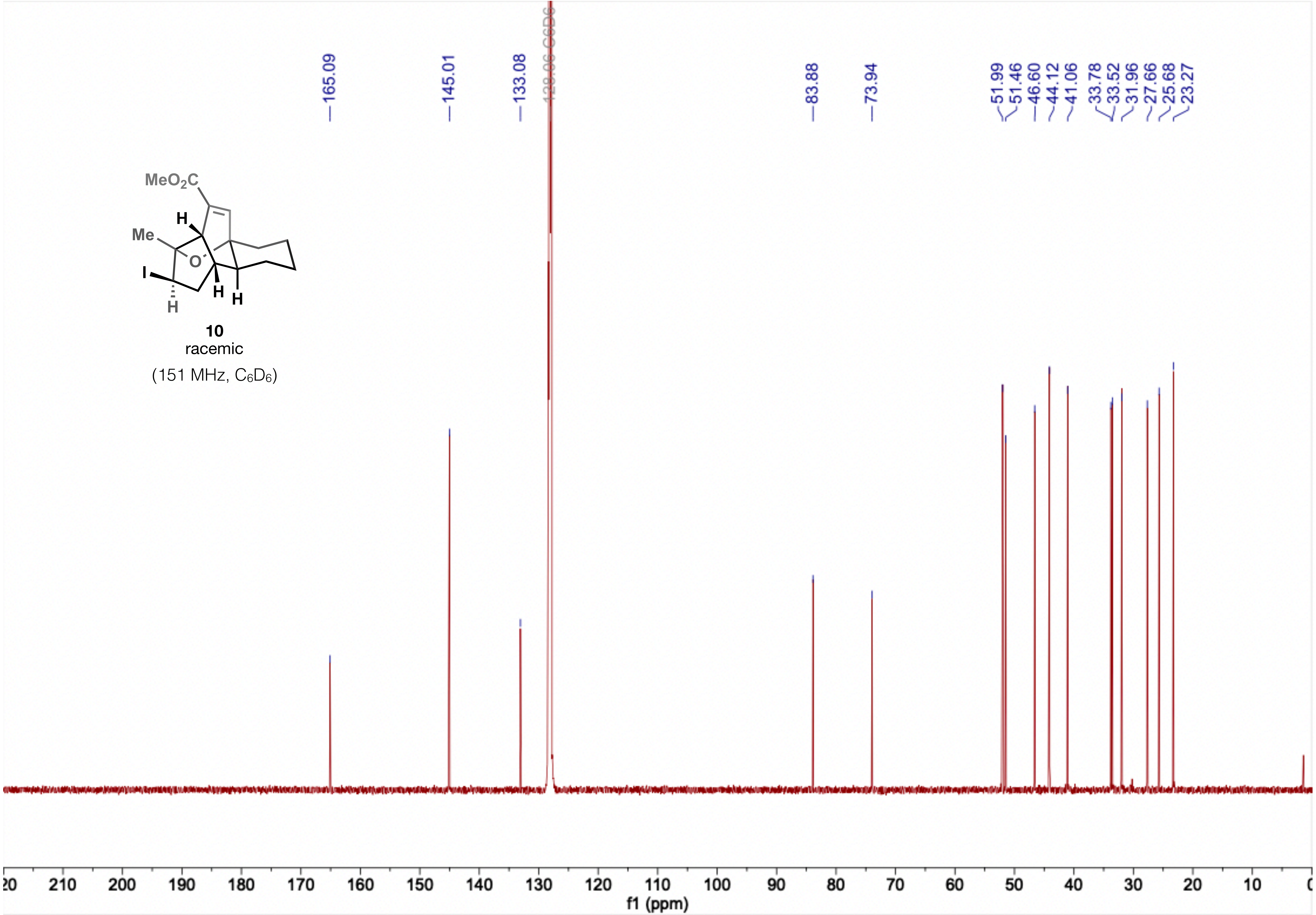

MeO2C
H
Me
O
I
H
H
H
10
racemic
(151 MHz, C6D6)
165.09
145.01
133.08
128.06 C6D6
83.88
73.94
51.99
51.46
46.60
44.12
41.06
33.78
33.52
31.96
27.66
25.68
23.27
220 210 200 190 180 170 160 150 140 130 120 110 100 90 80 70 60 50 40 30 20 10 0
f1 (ppm)

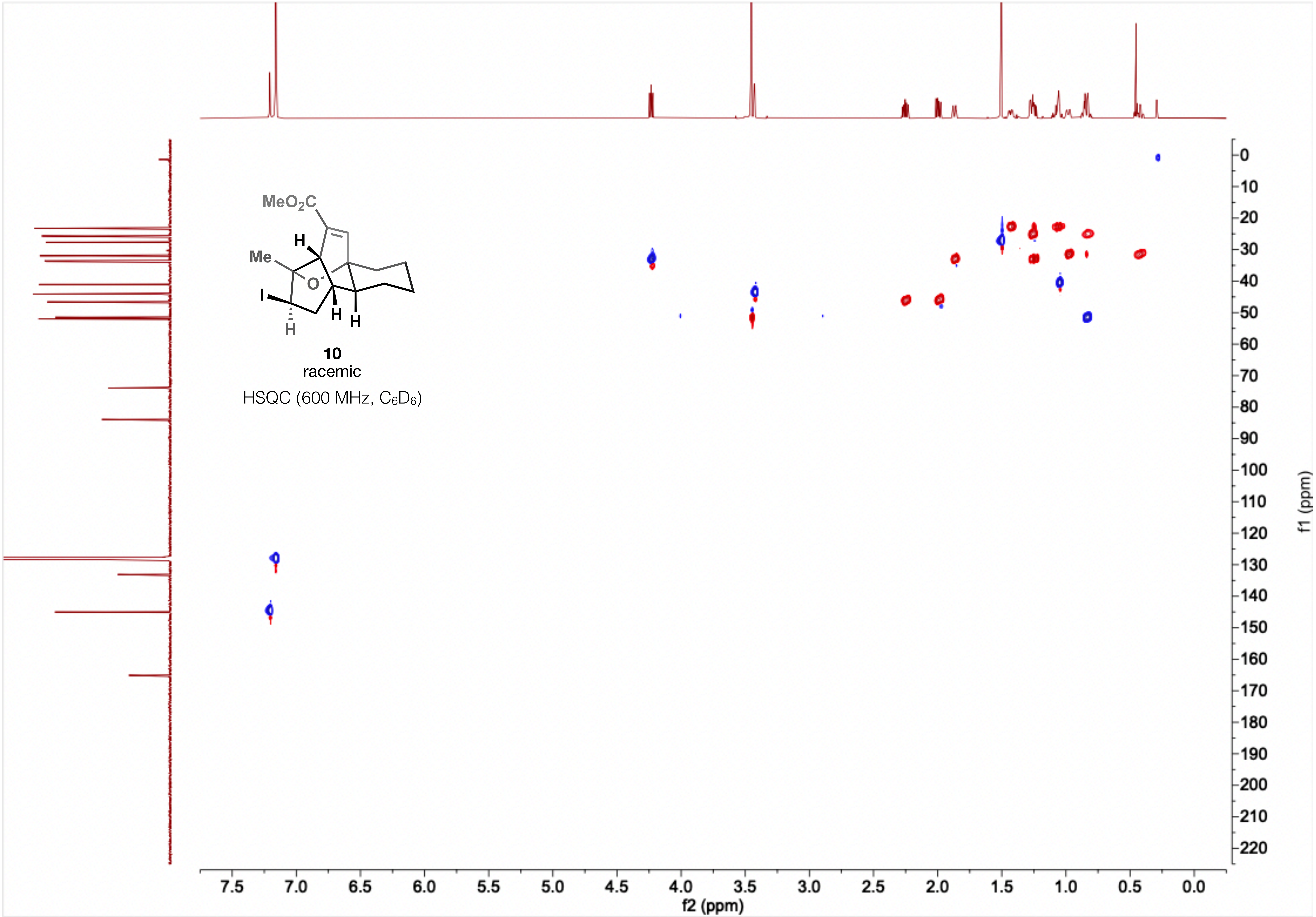
MeO2C
Me
H
O
I
H
H
H
10
racemic
HSQC (600 MHz, C6D6)
f2 (ppm)
f1 (ppm)

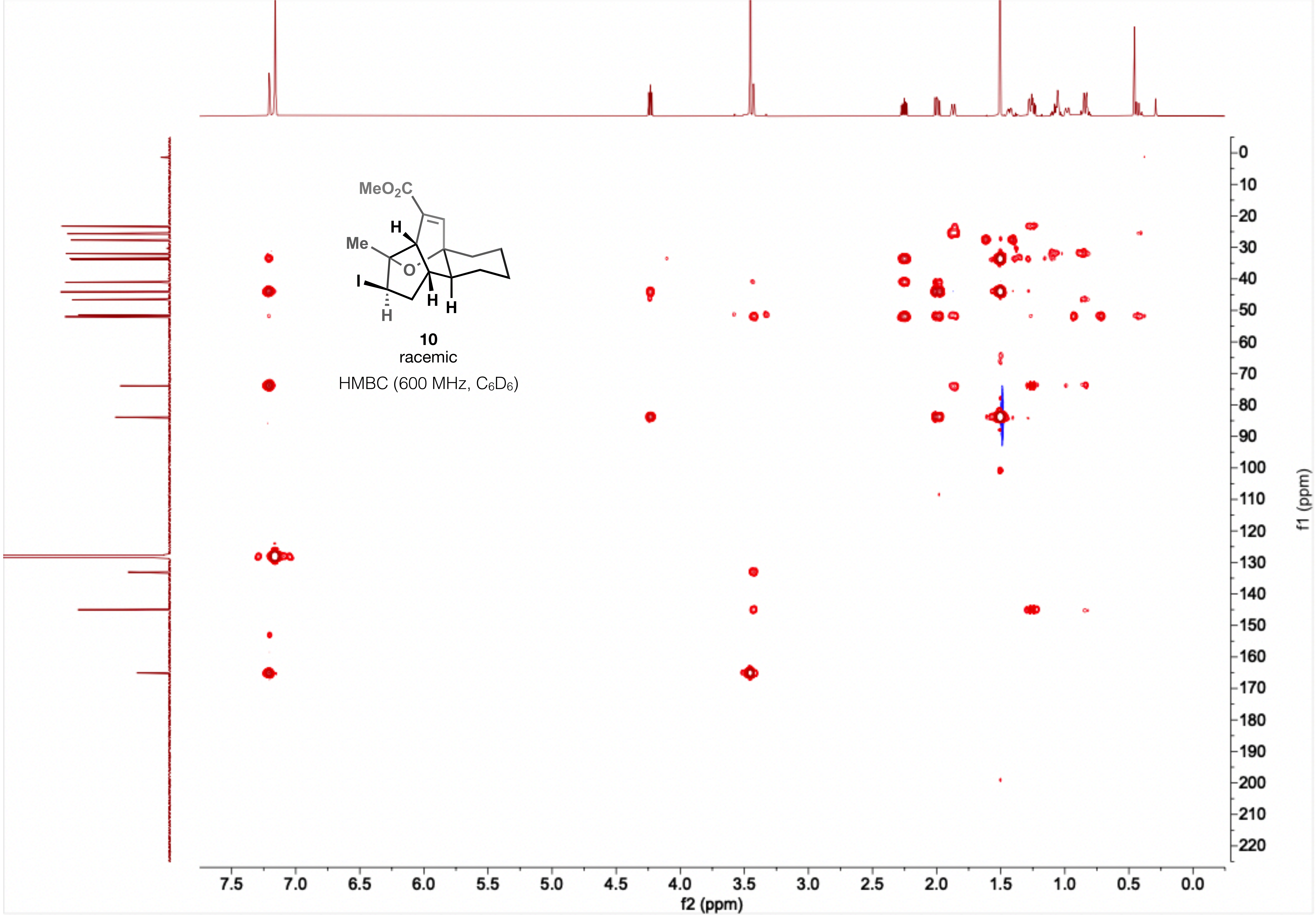
MeO2C
Me
H
O
I
H
H
H
10
racemic
HMBC (600 MHz, C6D6)
f2 (ppm)
f1 (ppm)

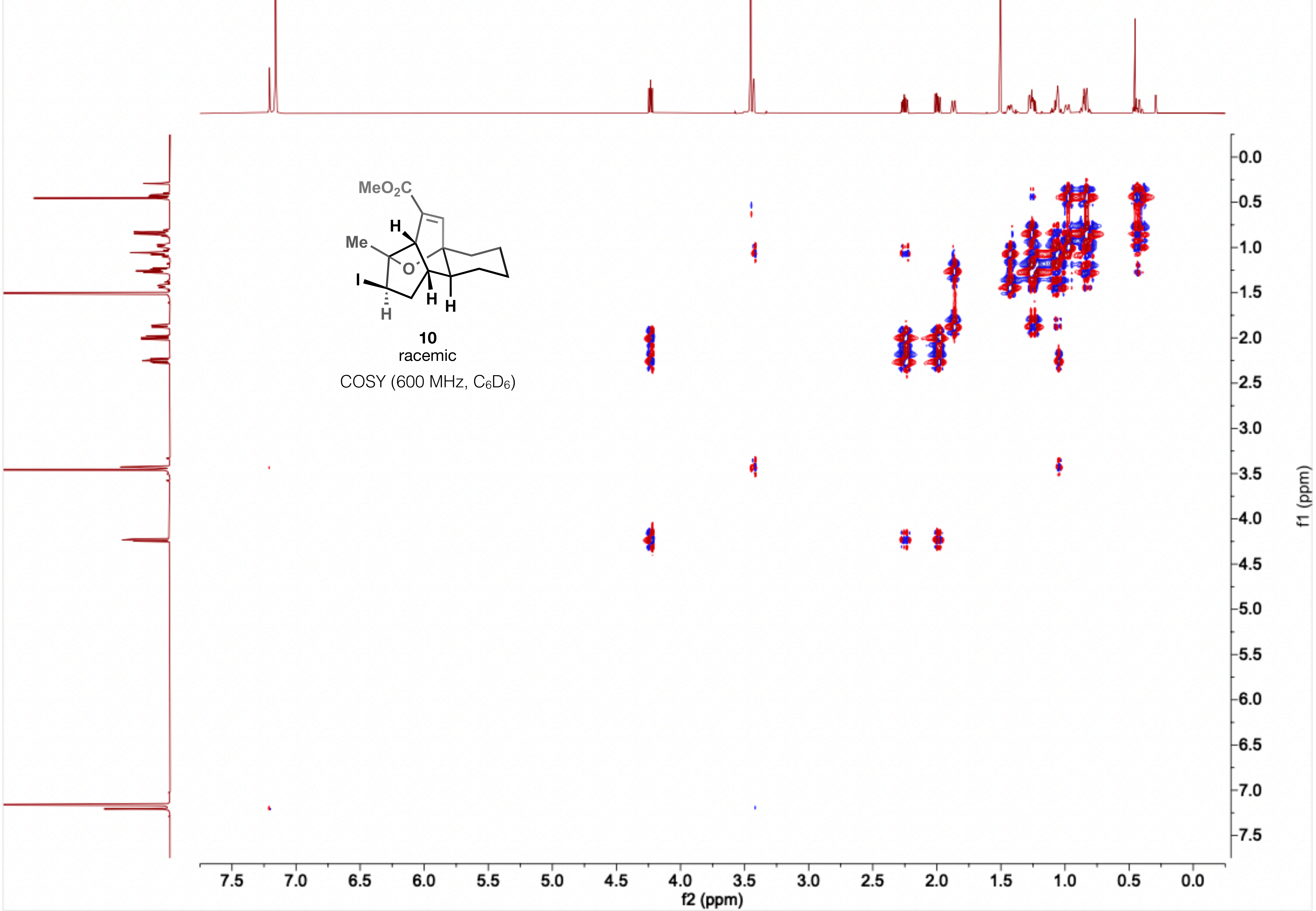

MeO2C
Me
O
I
H
10
racemic
COSY (600 MHz, C6D6)
f2 (ppm)
f1 (ppm)

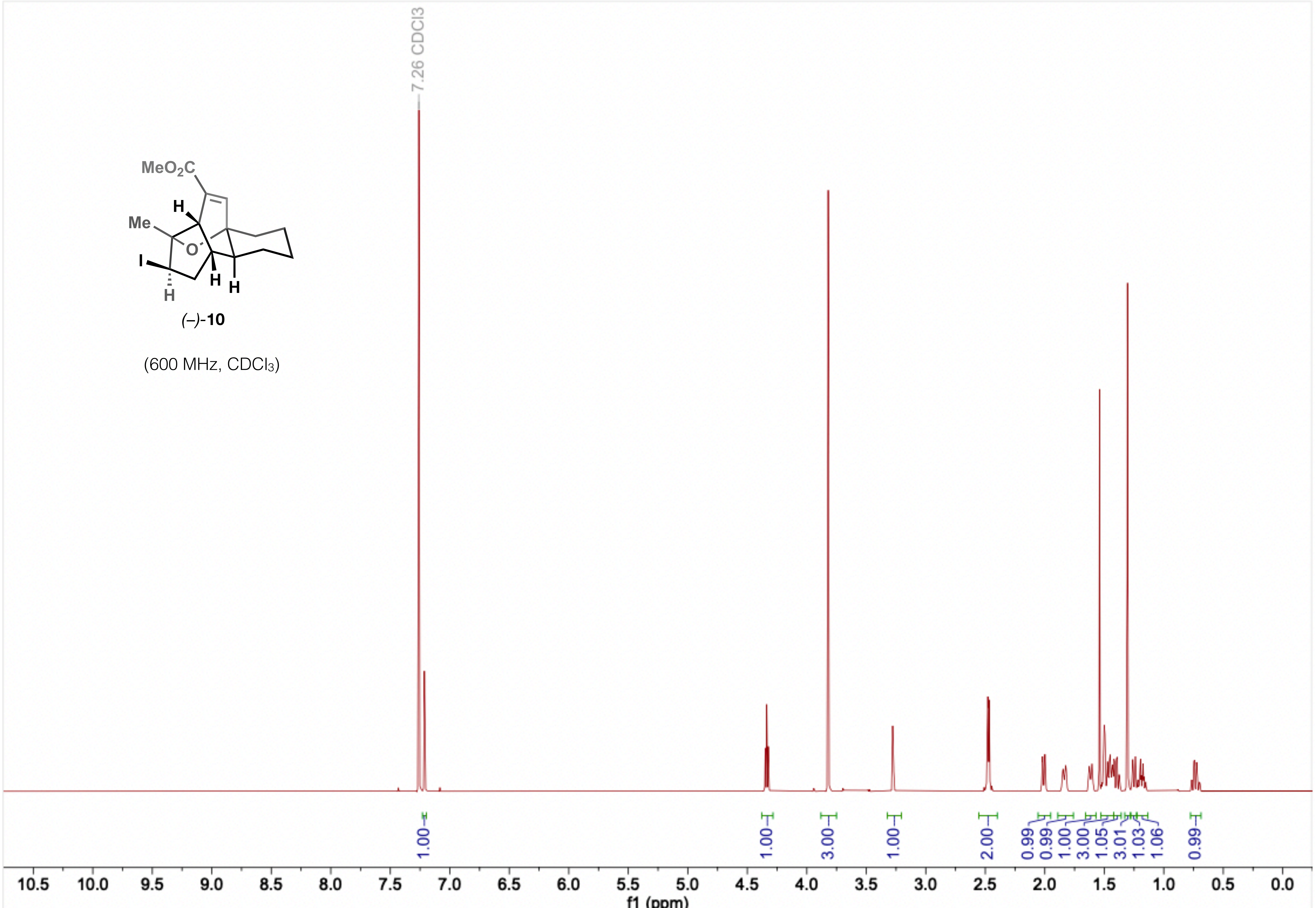
7.26 CDCl3
MeO2C
Me
O
I
H
(–)-10
(600 MHz, CDCl3)
1.00
1.00
3.00
1.00
2.00
0.99
0.99
1.00
3.00
1.05
3.01
1.03
1.06
0.99
10.5 10.0 9.5 9.0 8.5 8.0 7.5 7.0 6.5 6.0 5.5 5.0 4.5 4.0 3.5 3.0 2.5 2.0 1.5 1.0 0.5 0.0
f1 (ppm)

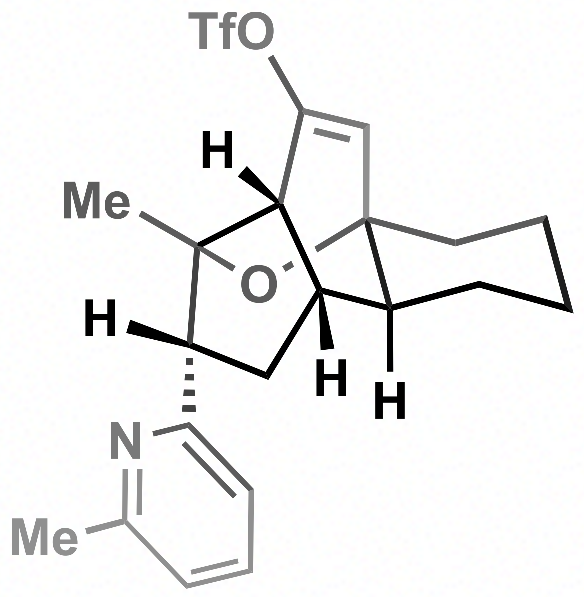

11

(600 MHz, C_6D_6)

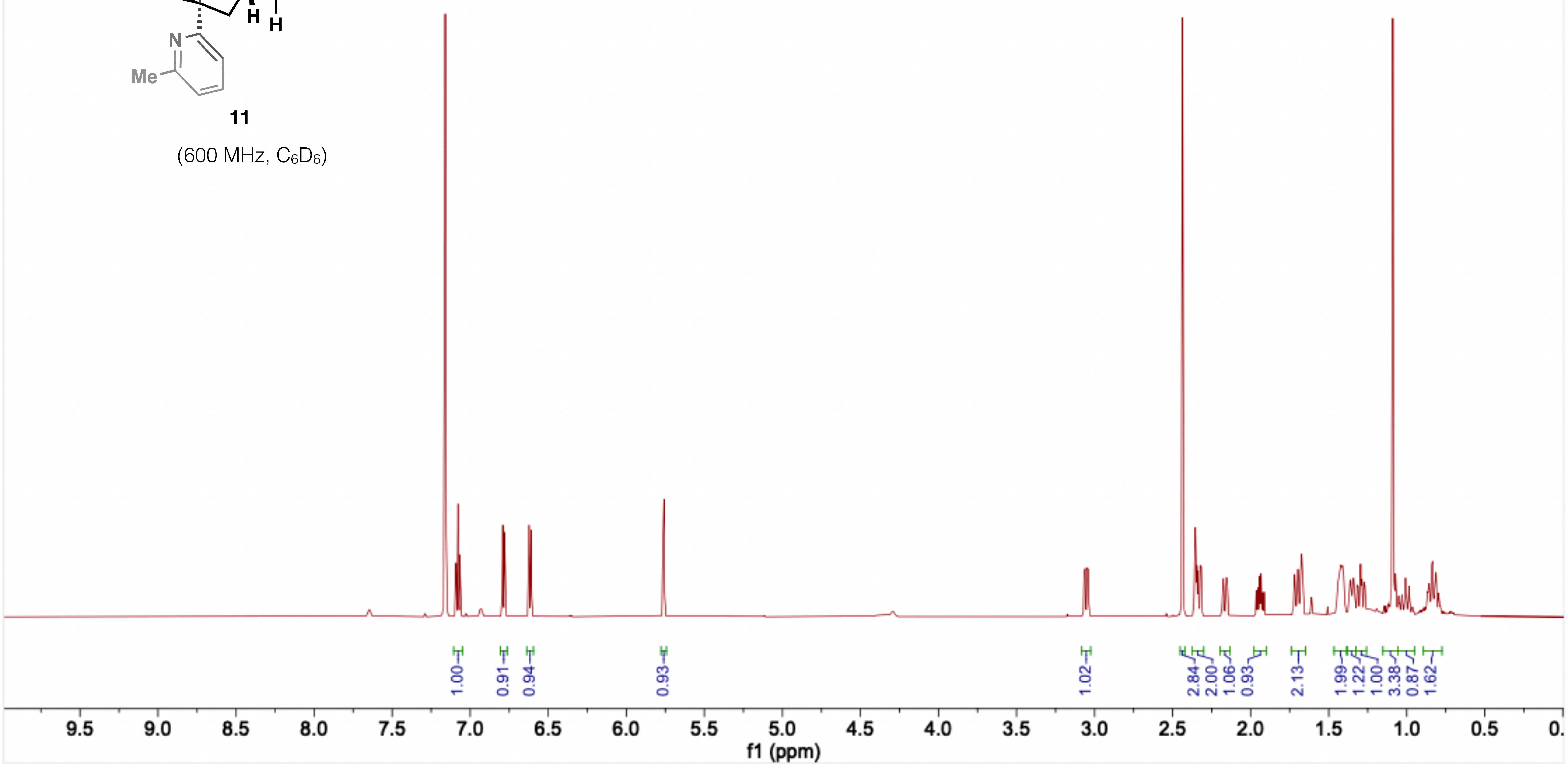

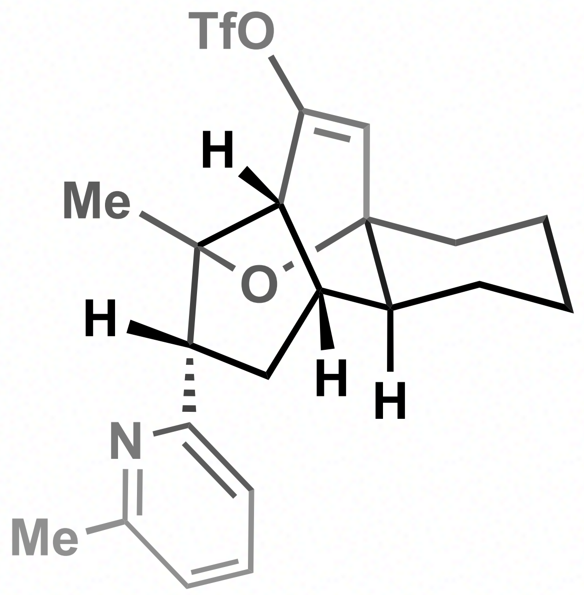

11

(151 MHz, C_6D_6)

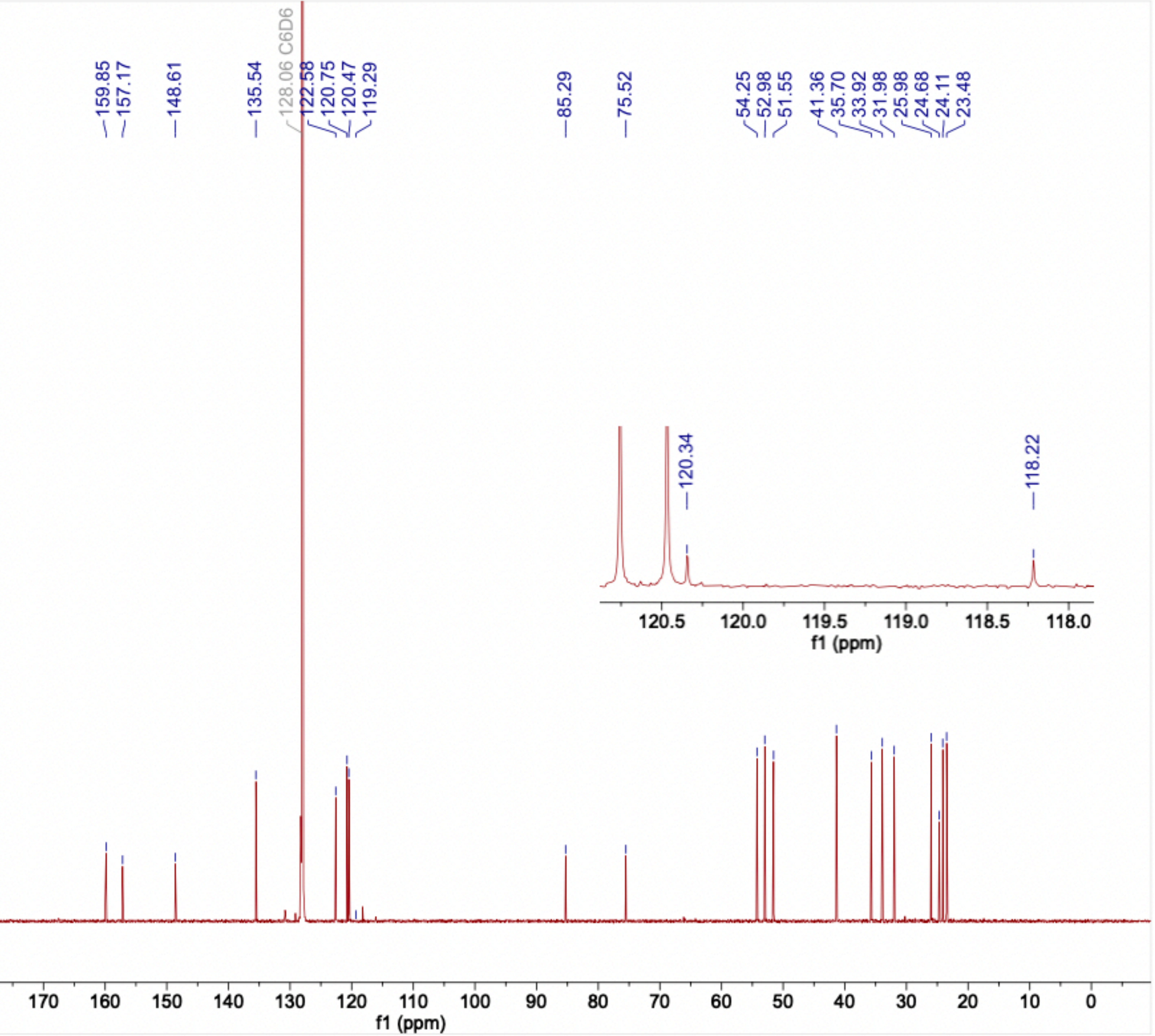

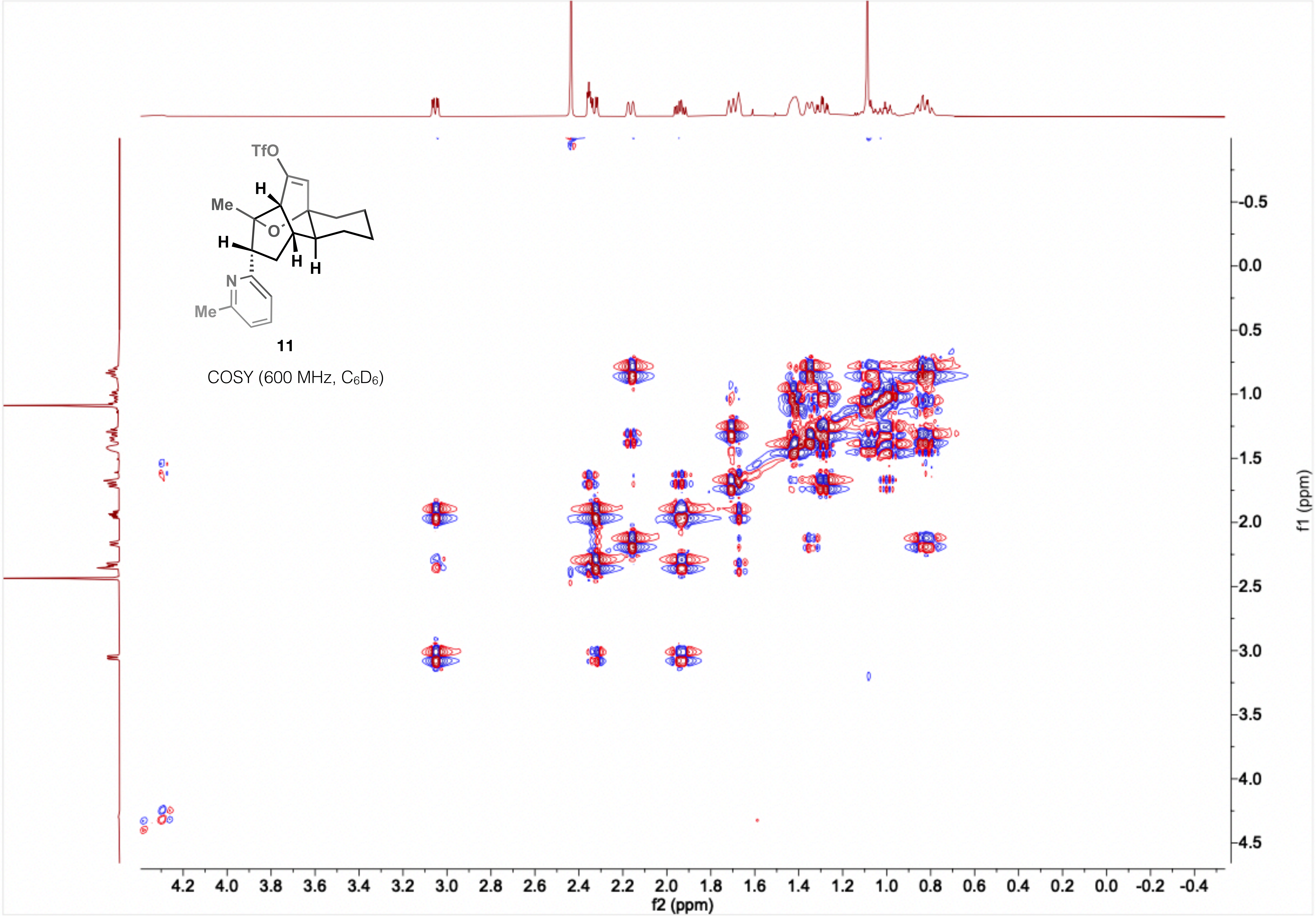

TfO
H
Me
O
H
H
H
N
Me
11
COSY (600 MHz, C6D6)
f2 (ppm)
f1 (ppm)

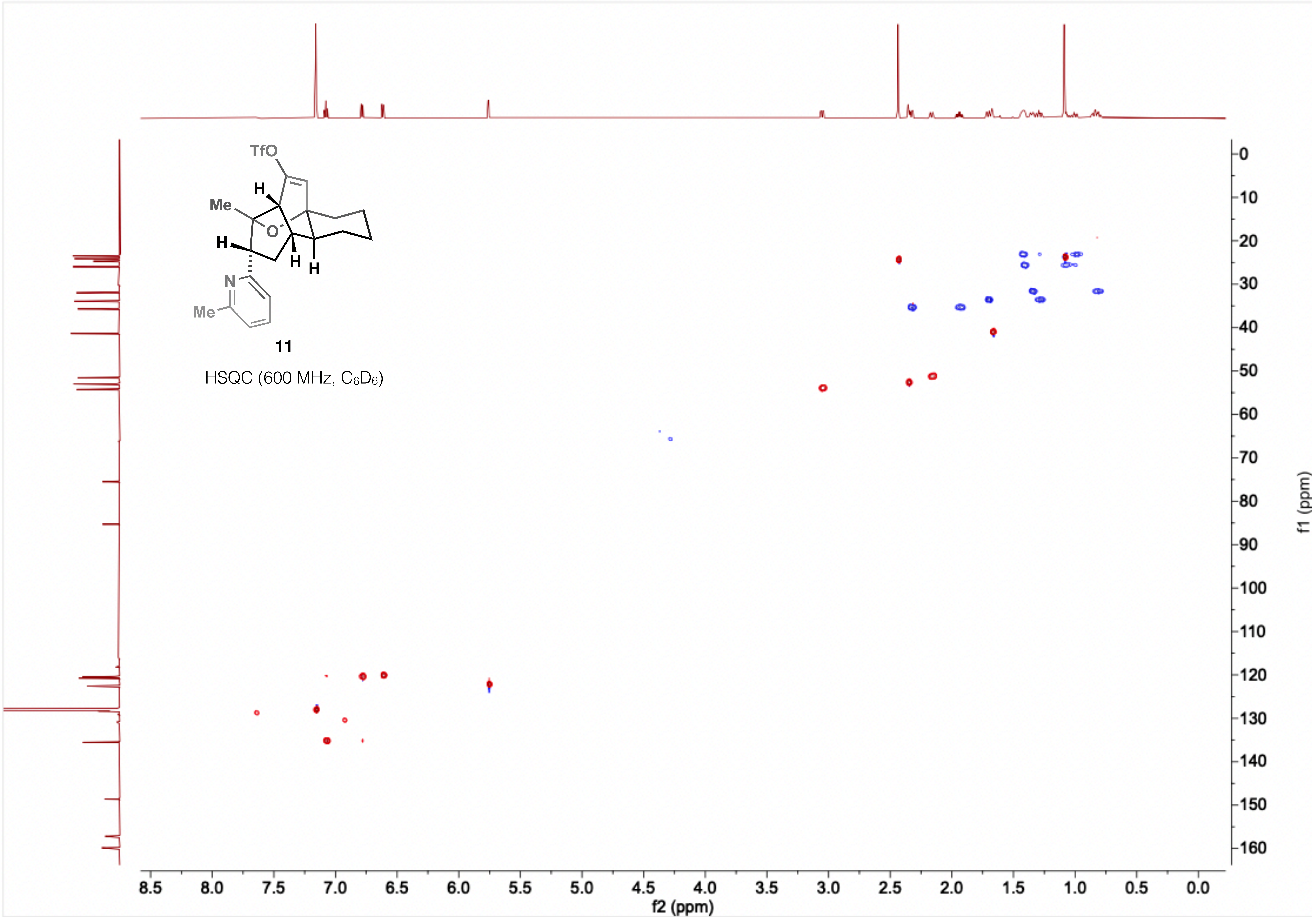

TfO
Me
H
O
H
H
H
N
Me
11
HSQC (600 MHz, C6D6)
f2 (ppm)
f1 (ppm)

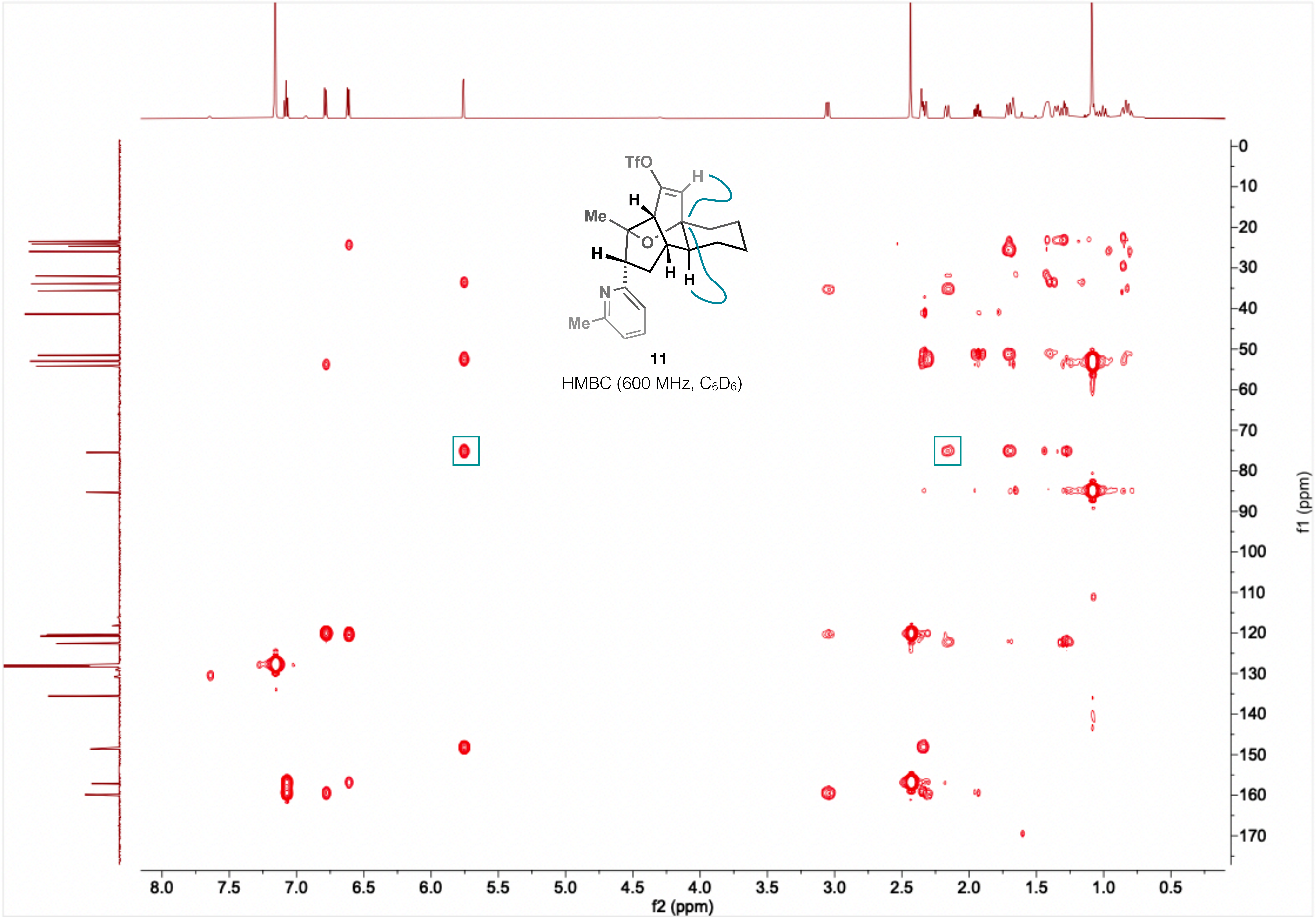

TfO
H
Me
O
H
H
H
H
N
Me
11
HMBC (600 MHz, C6D6)
f2 (ppm)
f1 (ppm)

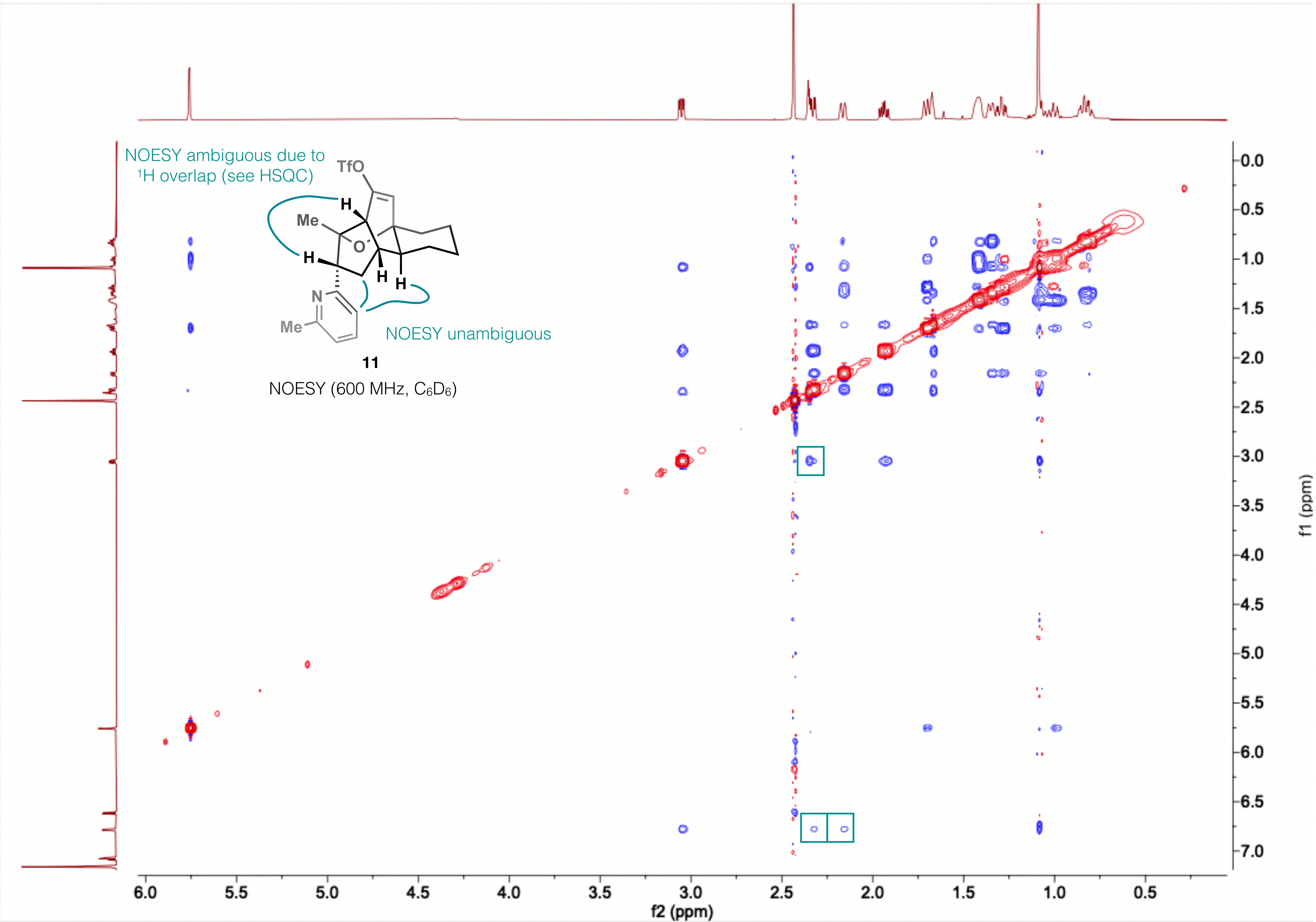

NOESY ambiguous due to
1H overlap (see HSQC)
TfO
Me
O
H
N
Me
NOESY unambiguous
11
NOESY (600 MHz, C6D6)
f2 (ppm)
f1 (ppm)

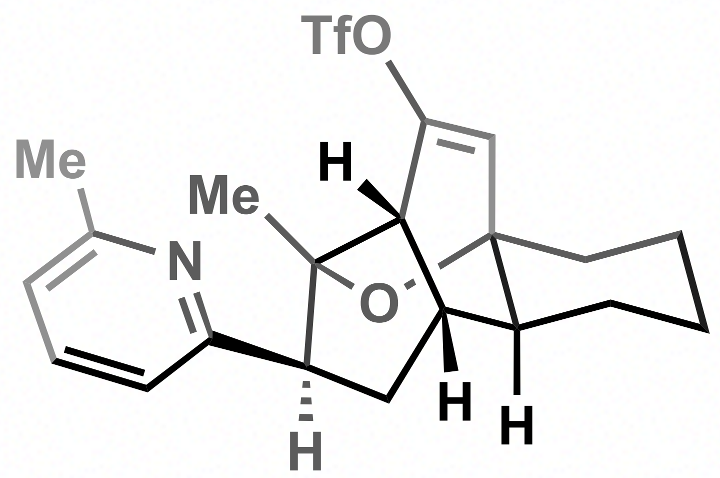

***epi*-11**

(600 MHz, C_6D_6)

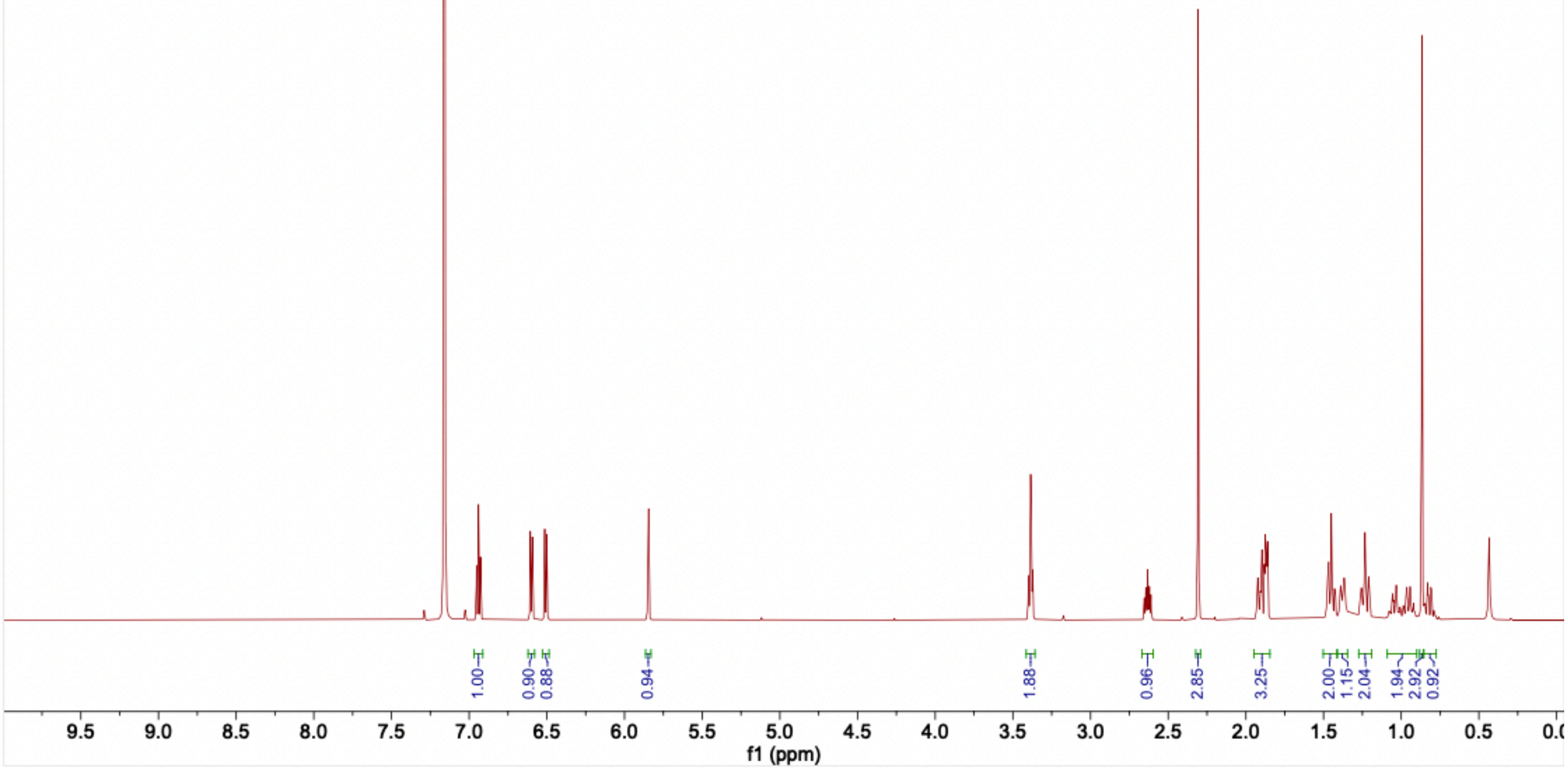

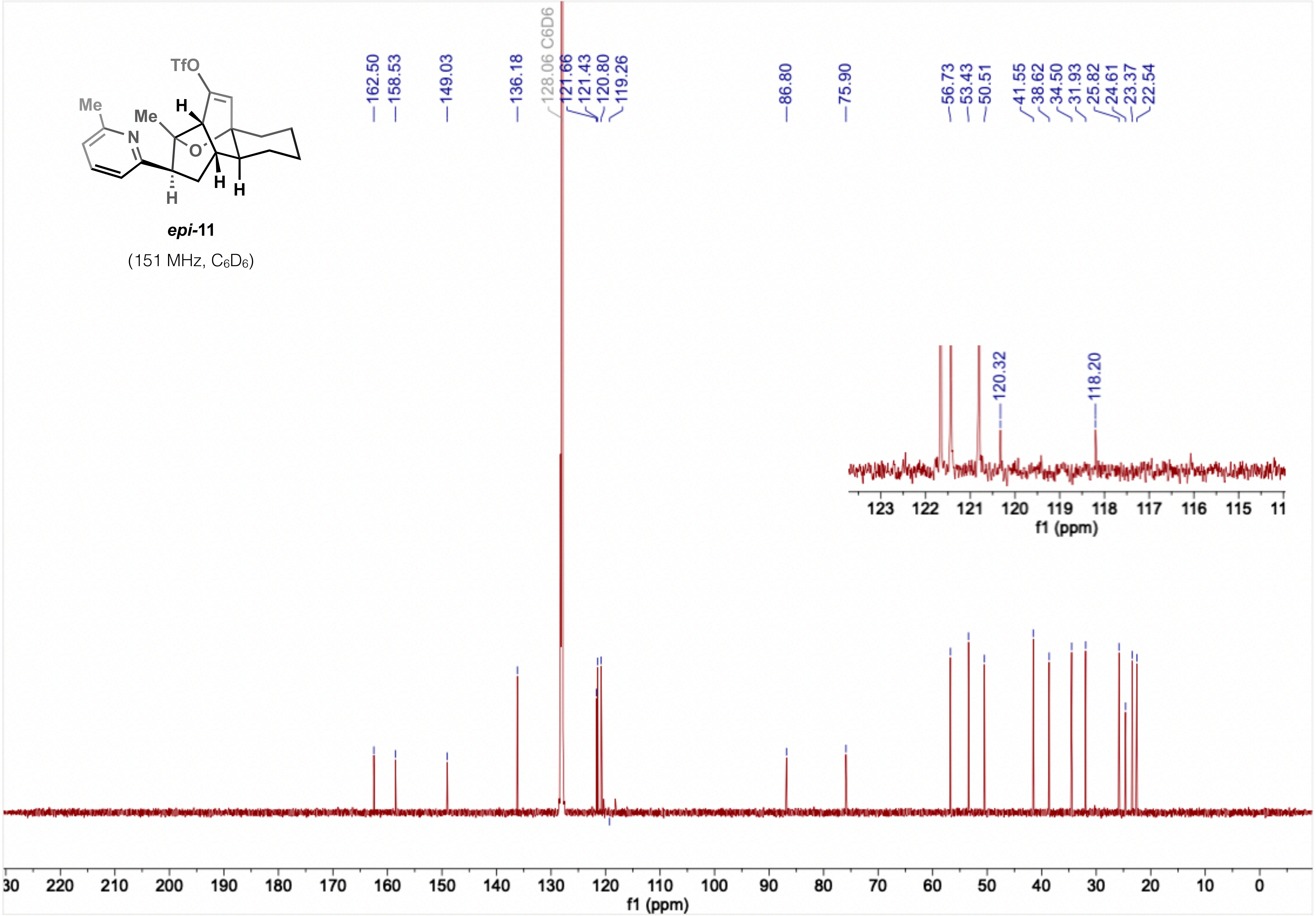
TfO
Me
N
Me
H
O
H
H
H
epi-11
(151 MHz, C_6D_6)
162.50
158.53
149.03
136.18
128.06 C6D6
121.66
121.43
120.80
119.26
86.80
75.90
56.73
53.43
50.51
41.55
38.62
34.50
31.93
25.82
24.61
23.37
22.54
120.32
118.20
f1 (ppm)

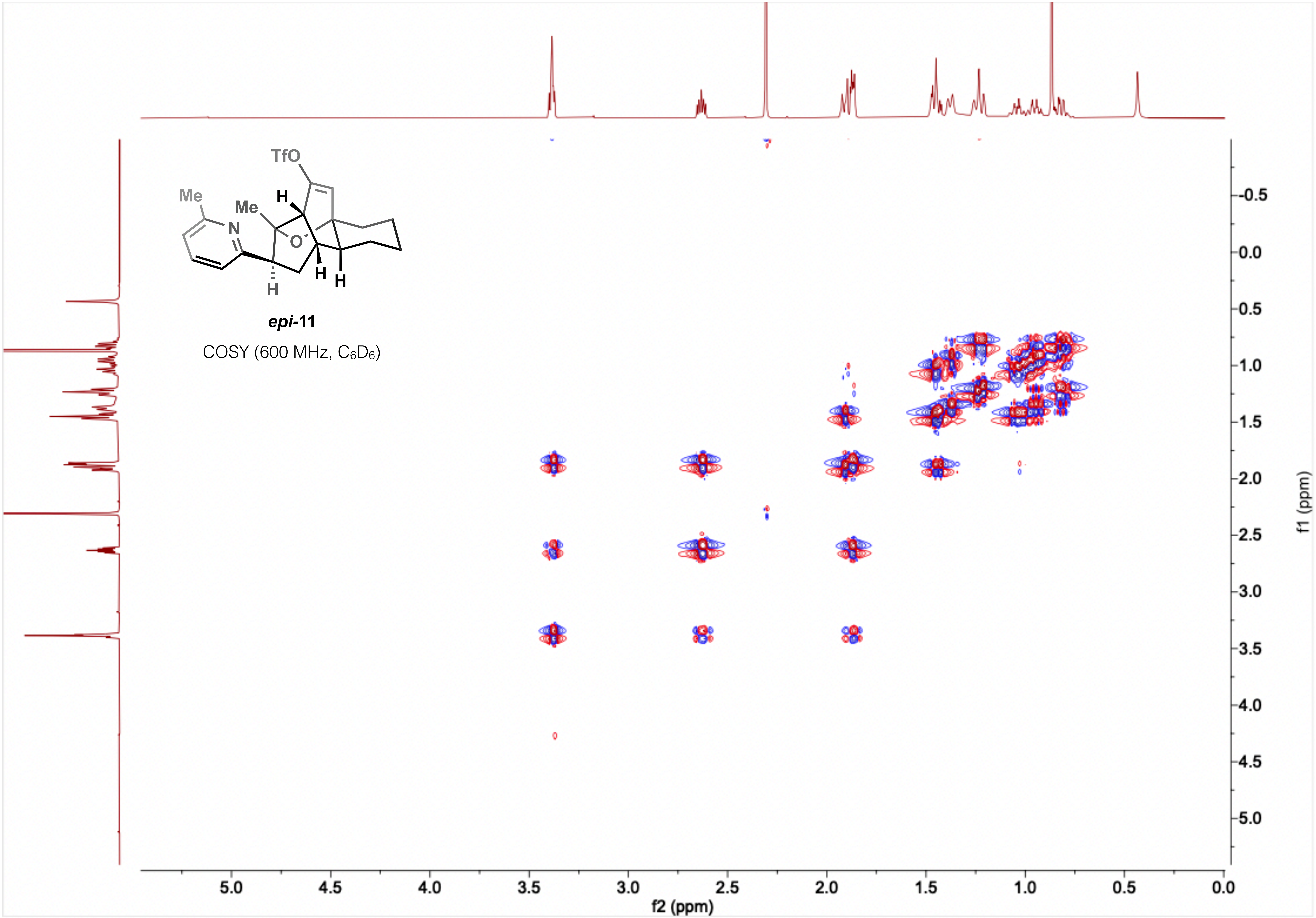
TfO
Me
Me
N
O
H
H
H
H
epi-11
COSY (600 MHz, C_6D_6)
f2 (ppm)
f1 (ppm)
5.0
4.5
4.0
3.5
3.0
2.5
2.0
1.5
1.0
0.5
0.0
-0.5

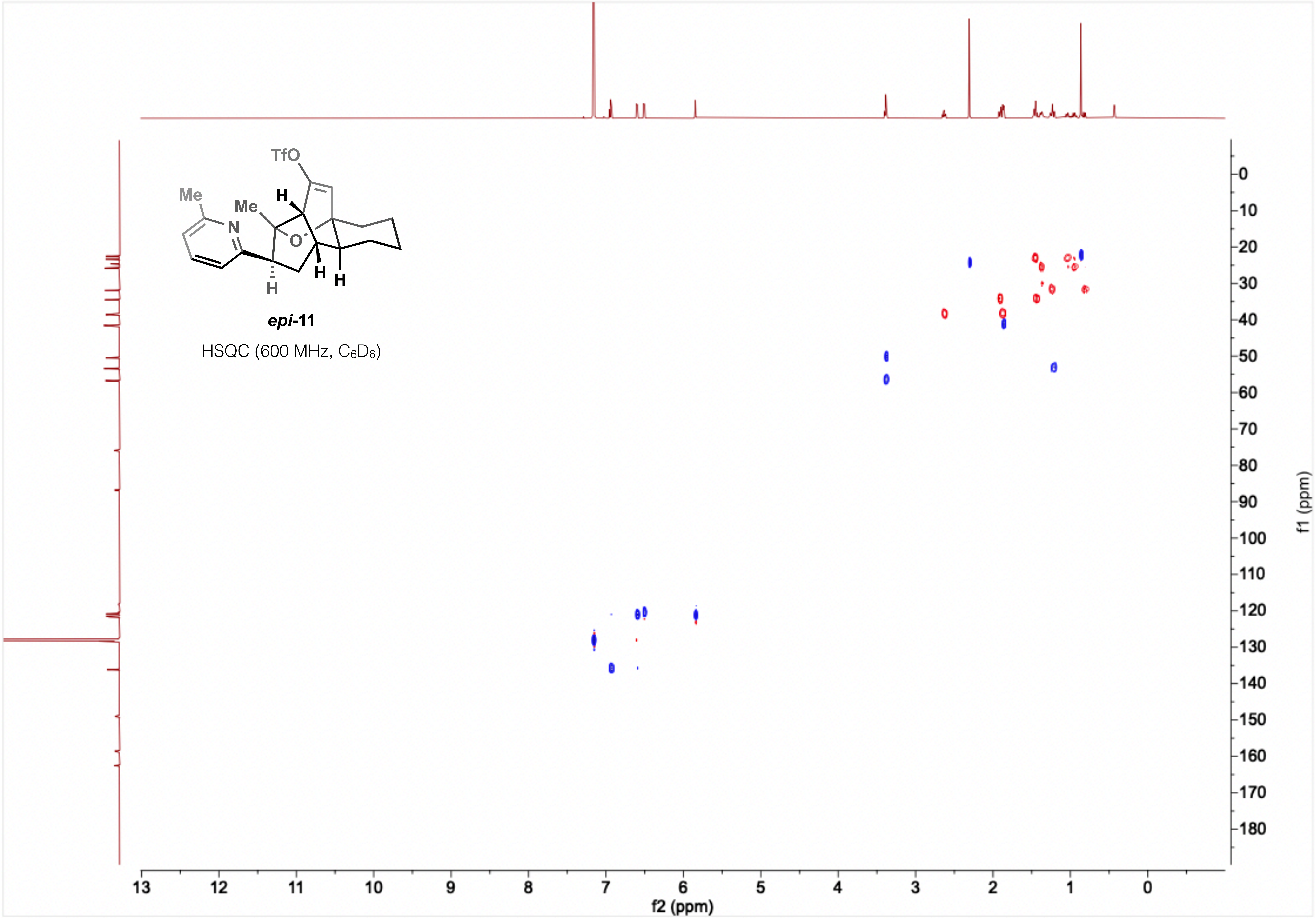

TfO
Me
Me
N
O
H
H
H
H
epi-11
HSQC (600 MHz, C_6D_6)
f2 (ppm)
f1 (ppm)

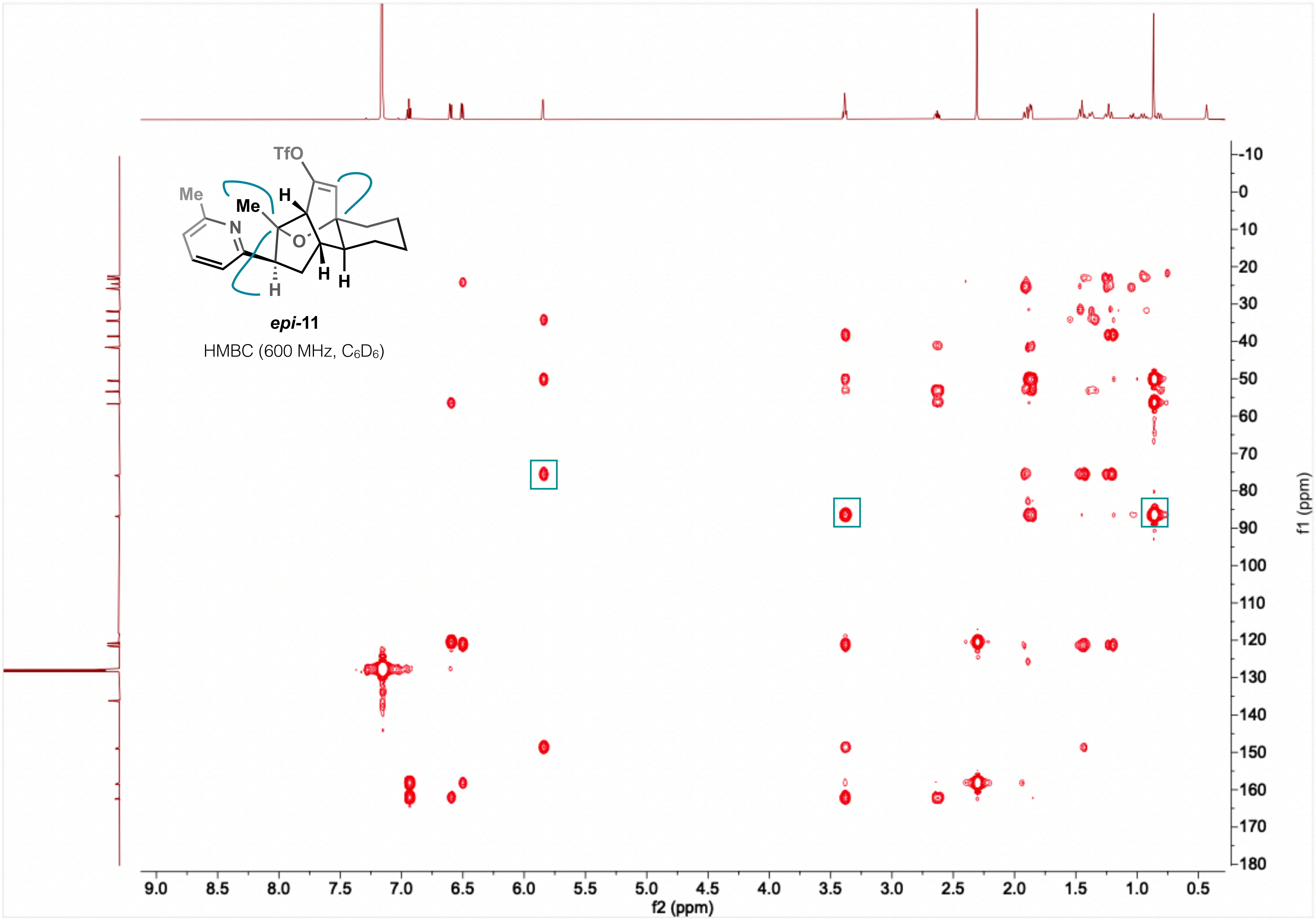

***epi*-11**

HMBC (600 MHz, C_6D_6)

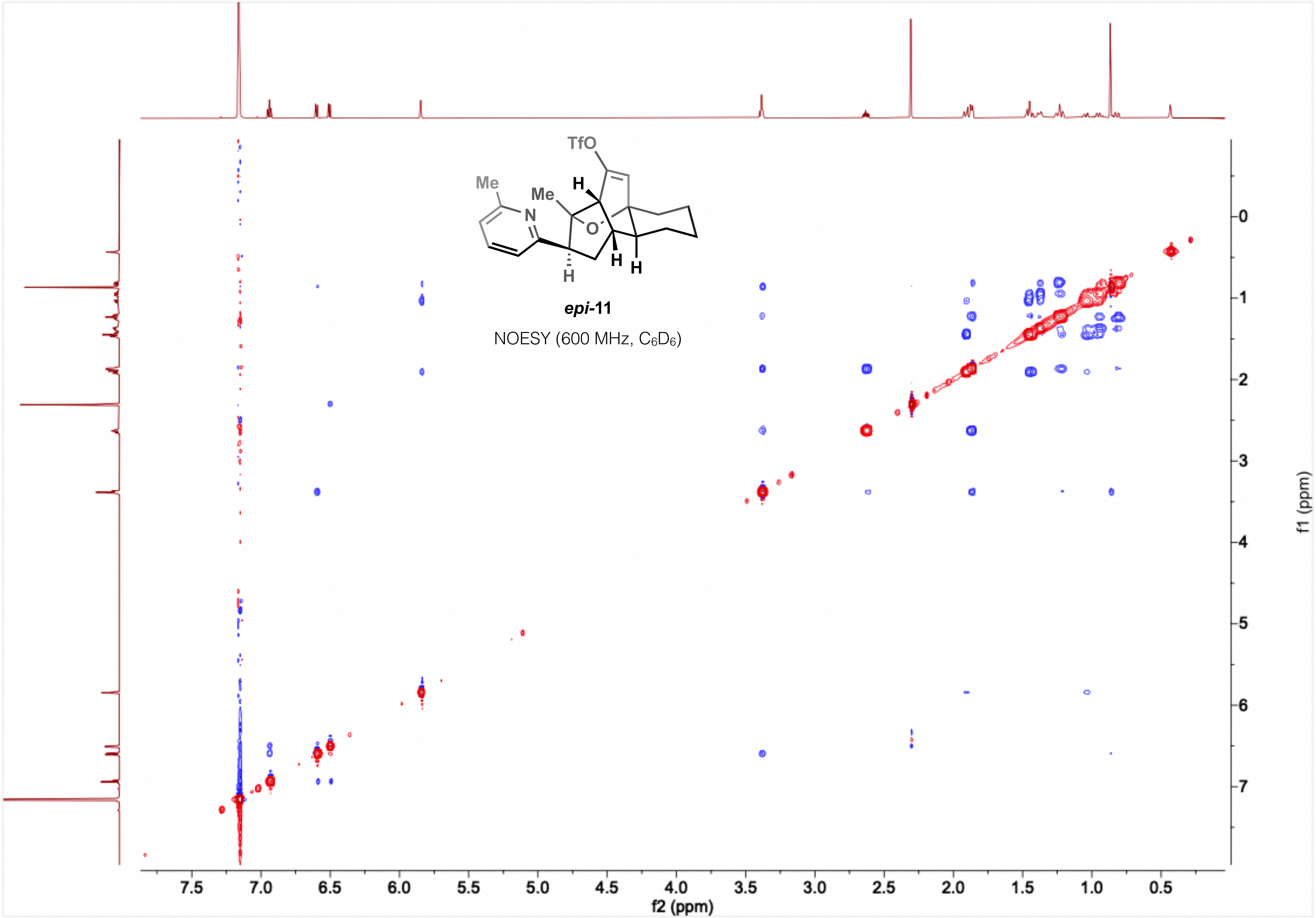

TfO
Me
Me
N
O
H
H
H
H
epi-11
NOESY (600 MHz, C6D6)
f1 (ppm)
f2 (ppm)

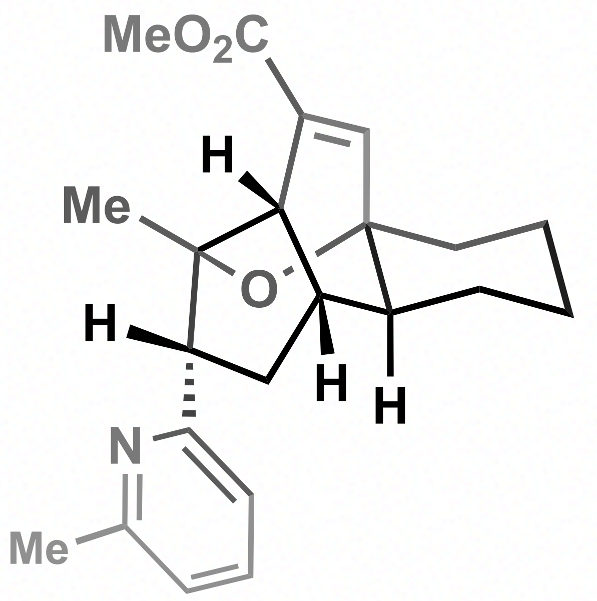

(–)-**2**
nat-Pyr-GB18
(600 MHz, $CDCl_3$)

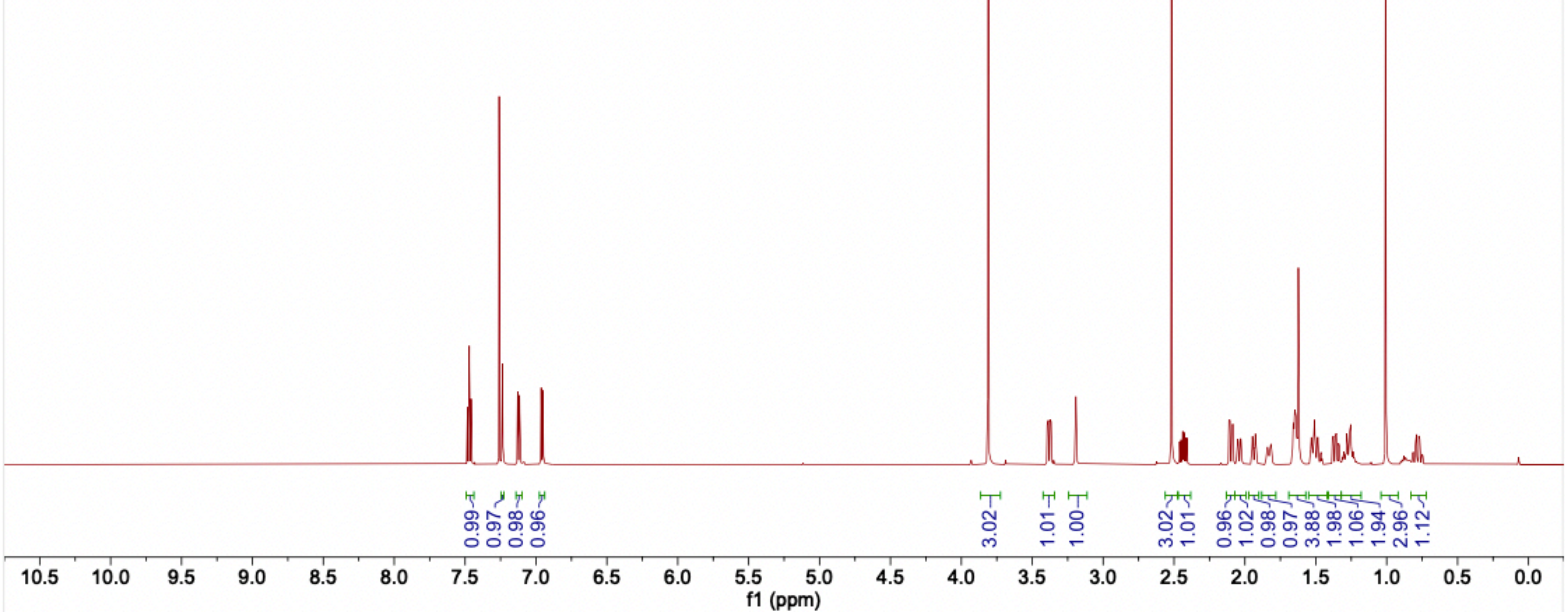

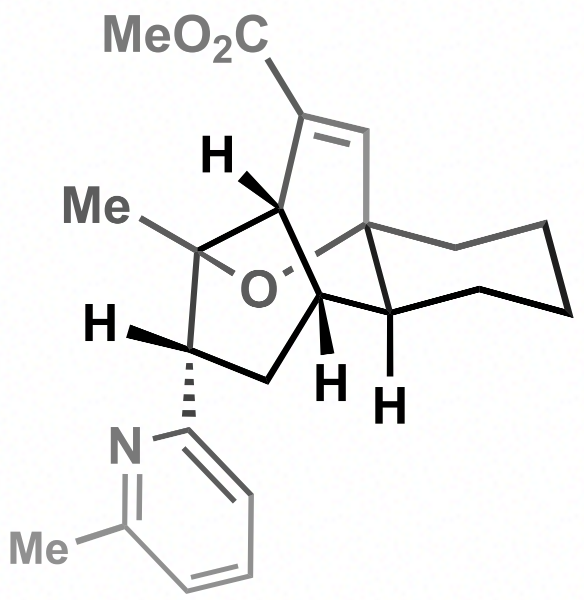

(–)-**2**
nat-Pyr-GB18
(151 MHz, $CDCl_3$)

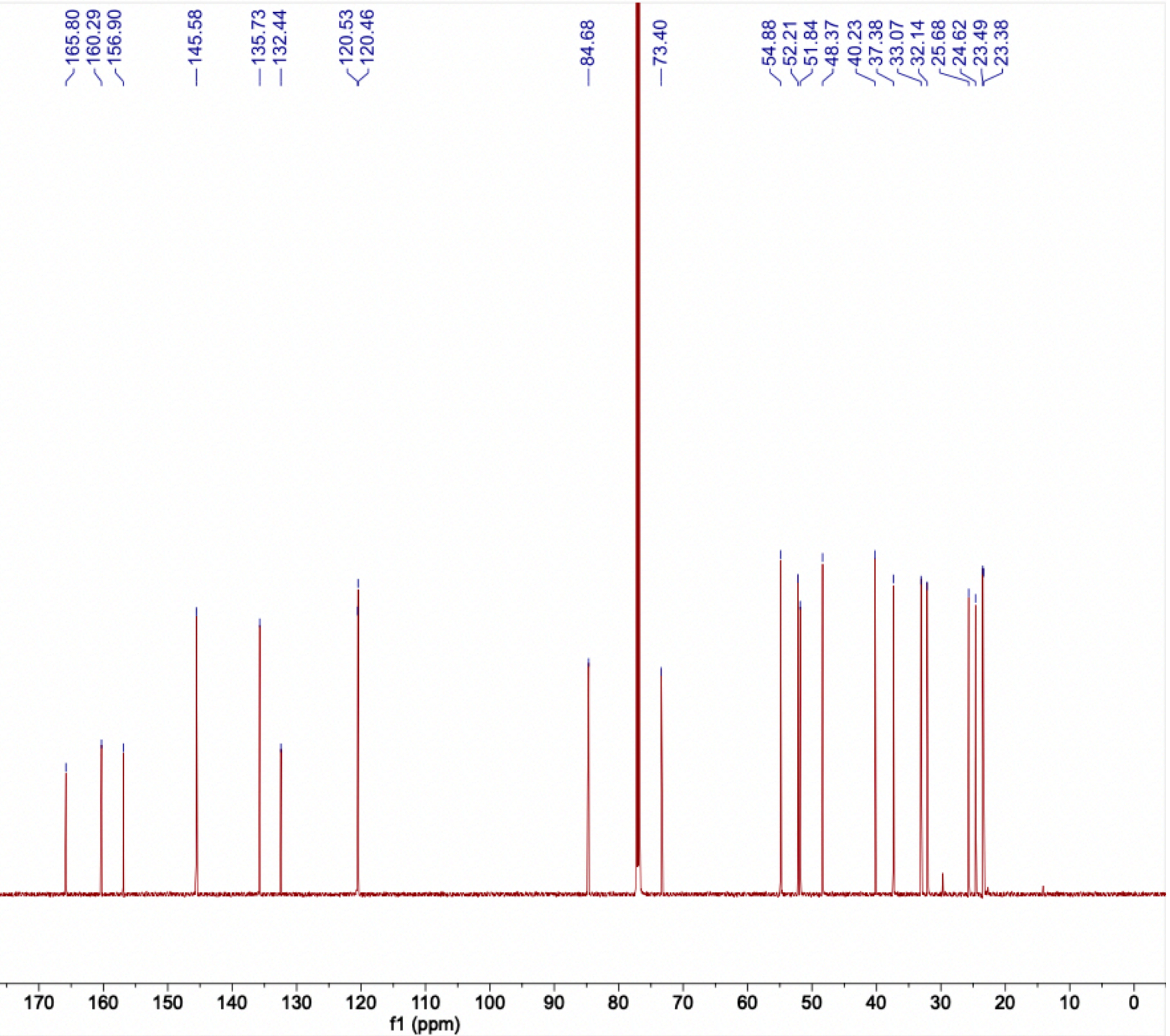

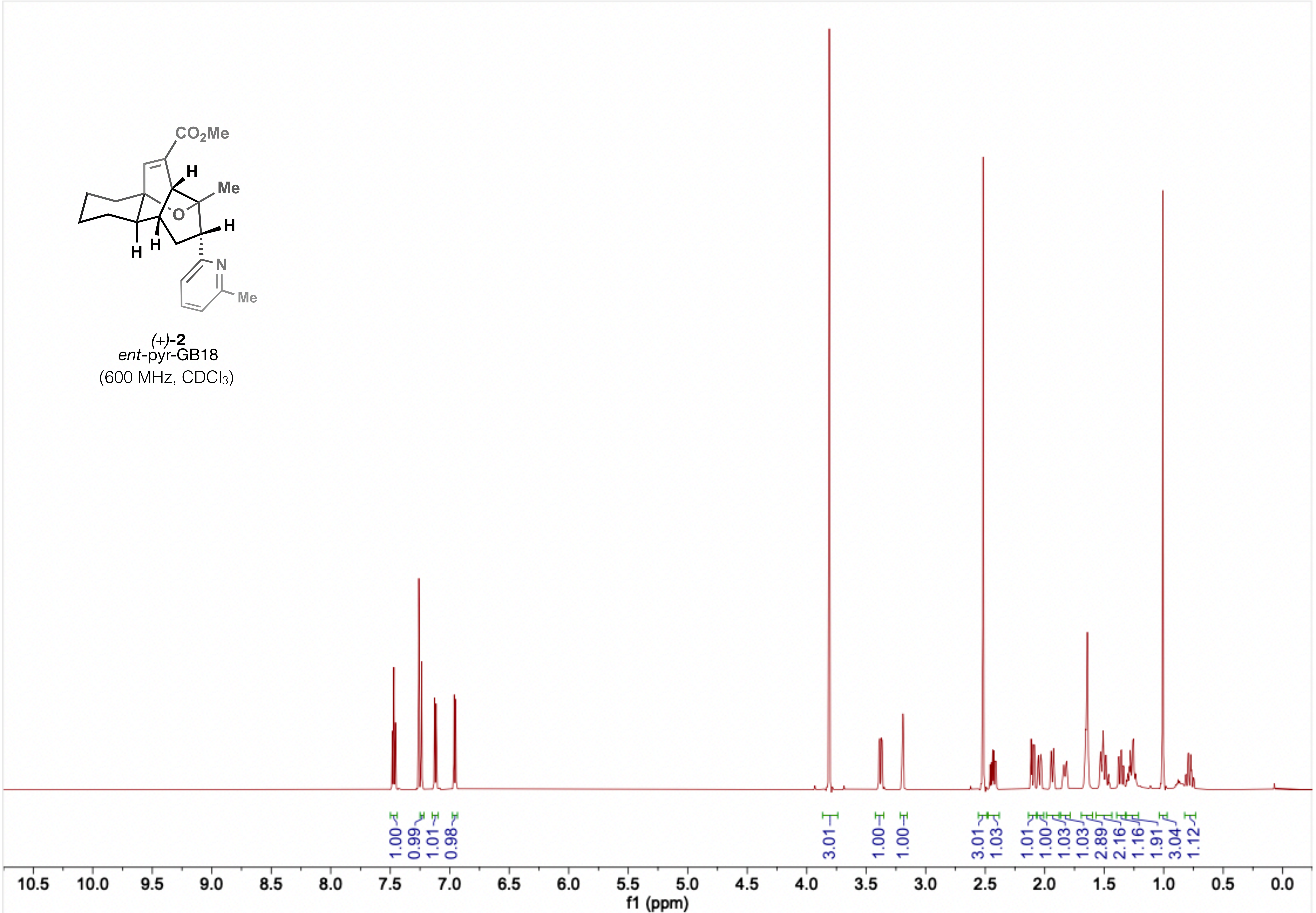

CO_2Me
H
Me
O
H
H
H
N
Me
(+)-**2**
ent-pyr-GB18
(600 MHz, $CDCl_3$)
1.00
0.99
1.01
0.98
3.01
1.00
1.00
3.01
1.03
1.01
1.00
1.03
1.03
2.89
2.16
1.16
1.91
3.04
1.12
10.5 10.0 9.5 9.0 8.5 8.0 7.5 7.0 6.5 6.0 5.5 5.0 4.5 4.0 3.5 3.0 2.5 2.0 1.5 1.0 0.5 0.0
f1 (ppm)

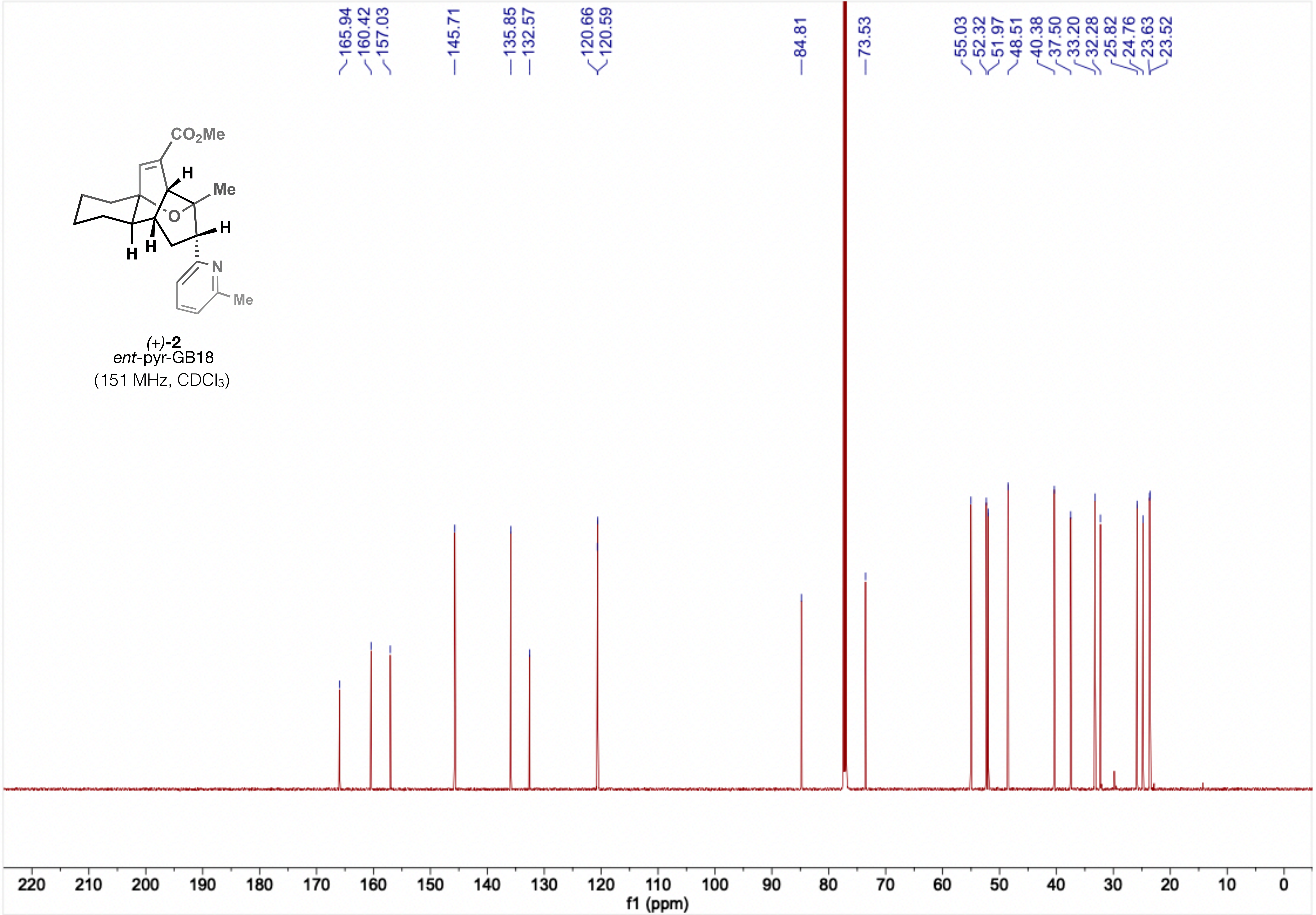
165.94
160.42
157.03
145.71
135.85
132.57
120.66
120.59
84.81
73.53
55.03
52.32
51.97
48.51
40.38
37.50
33.20
32.28
25.82
24.76
23.63
23.52
CO2Me
H
Me
O
H
H
H
N
Me
(+)-2
ent-pyr-GB18
(151 MHz, CDCl3)
220 210 200 190 180 170 160 150 140 130 120 110 100 90 80 70 60 50 40 30 20 10 0
f1 (ppm)

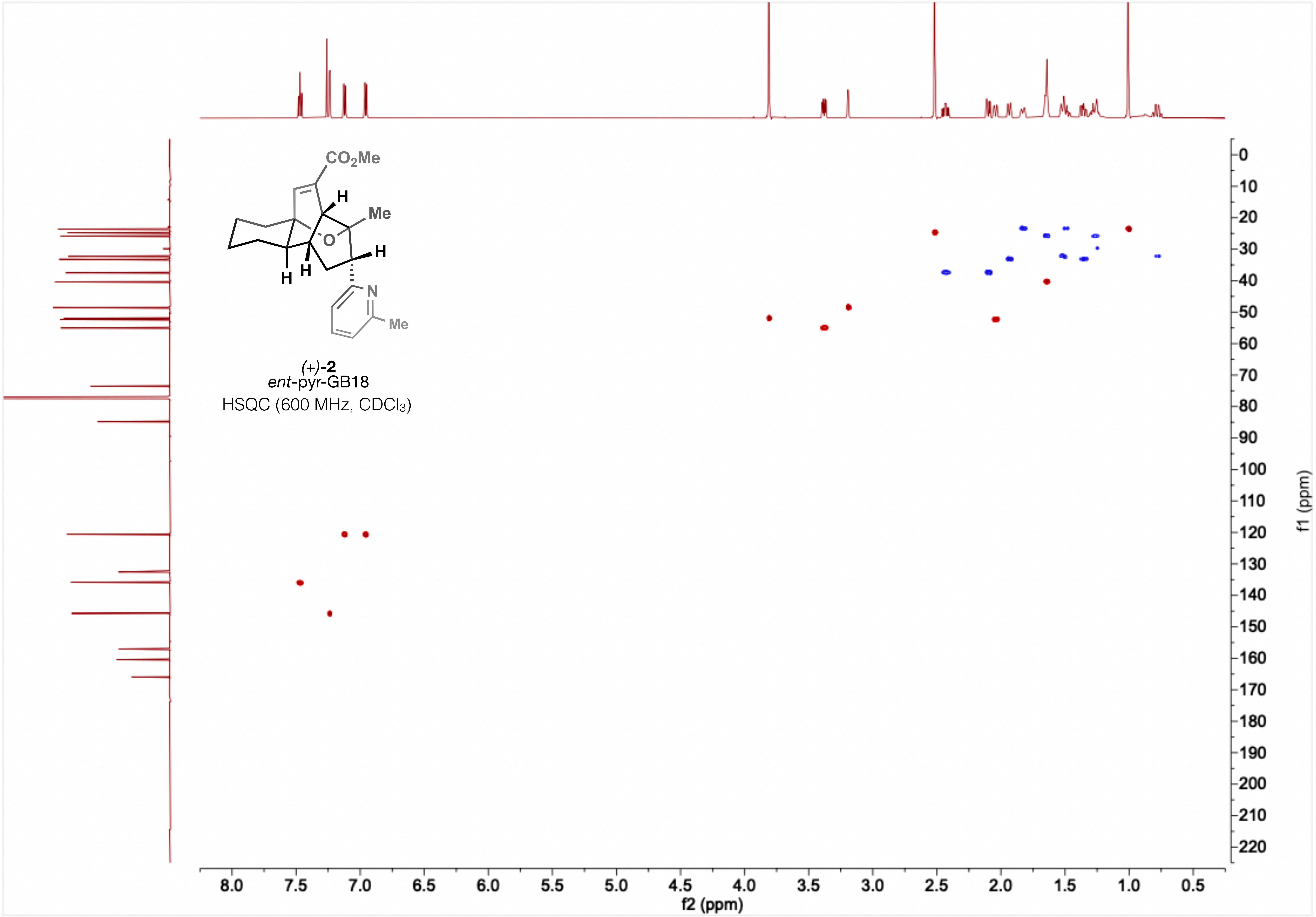

CO2Me
H
Me
O
H
H
H
N
Me
(+)-2
ent-pyr-GB18
HSQC (600 MHz, CDCl3)
f2 (ppm)
f1 (ppm)

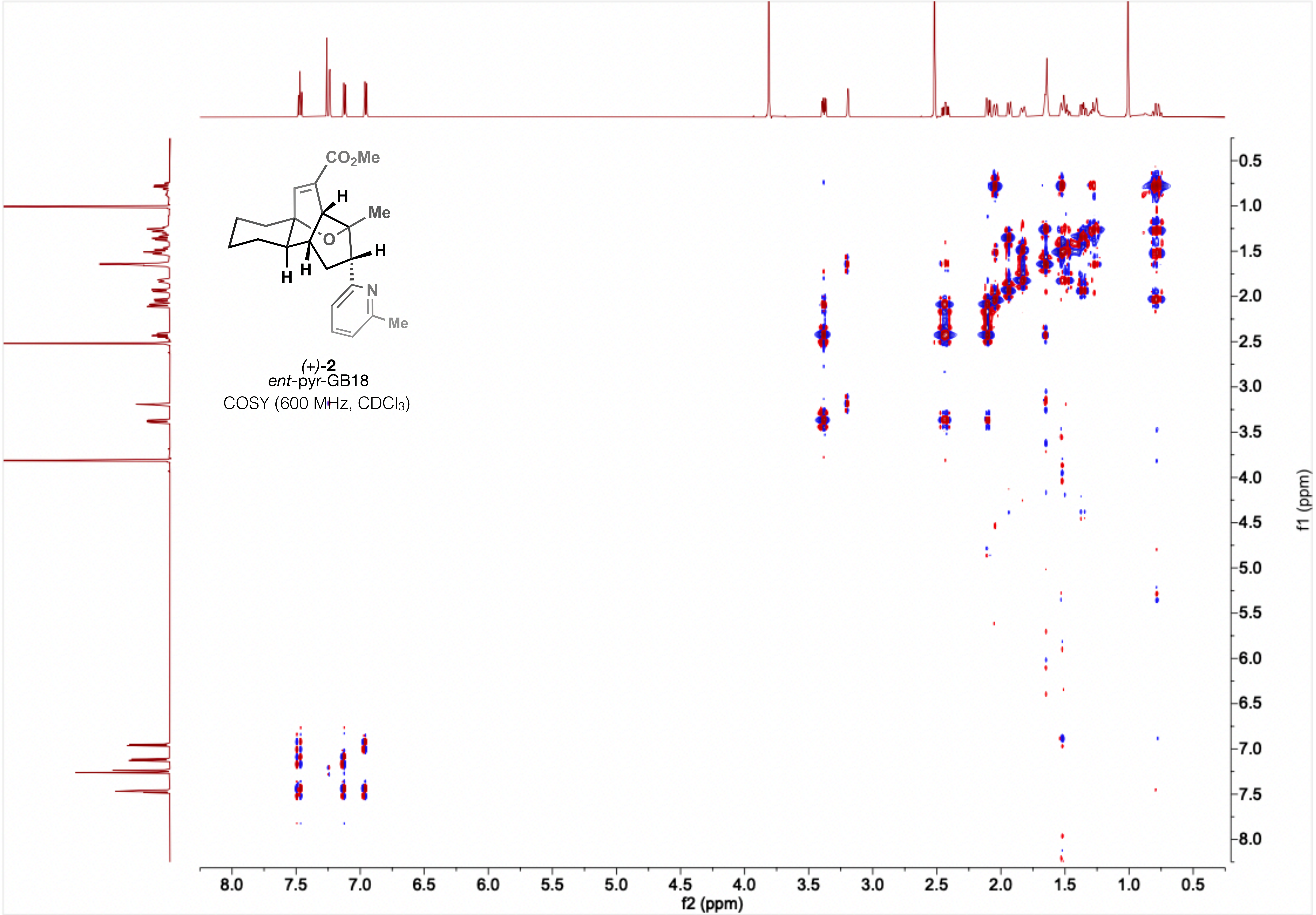

CO2Me
H
Me
O
H
H
H
N
Me
(+)-2
ent-pyr-GB18
COSY (600 MHz, CDCl3)
f1 (ppm)
f2 (ppm)
8.0
7.5
7.0
6.5
6.0
5.5
5.0
4.5
4.0
3.5
3.0
2.5
2.0
1.5
1.0
0.5

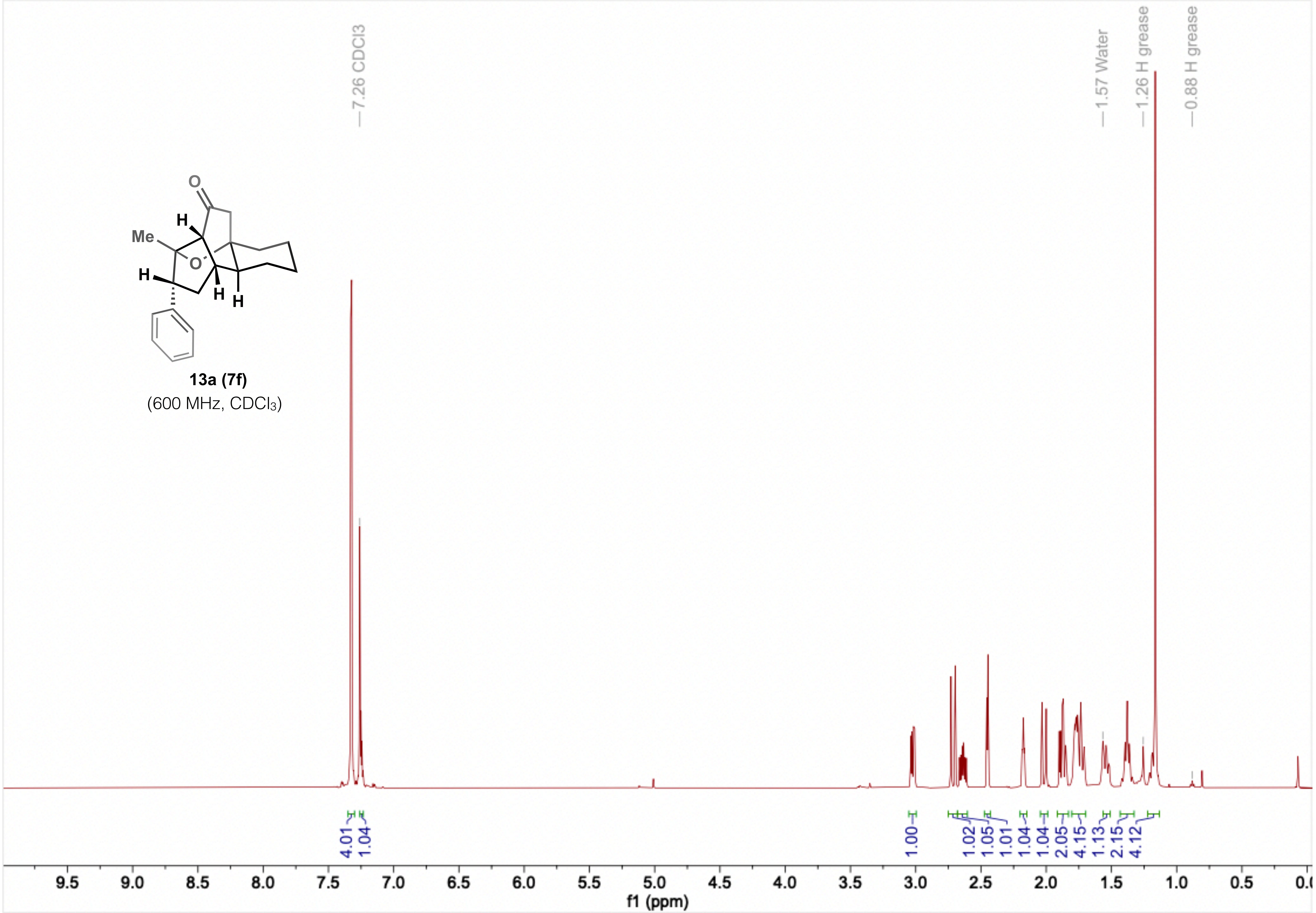

7.26 CDCl3
1.57 Water
1.26 H grease
0.88 H grease
Me
H
O
13a (7f)
(600 MHz, CDCl3)
4.01
1.04
1.00
1.02
1.05
1.01
1.04
1.04
2.05
4.15
1.13
2.15
4.12
9.5 9.0 8.5 8.0 7.5 7.0 6.5 6.0 5.5 5.0 4.5 4.0 3.5 3.0 2.5 2.0 1.5 1.0 0.5 0.0
f1 (ppm)

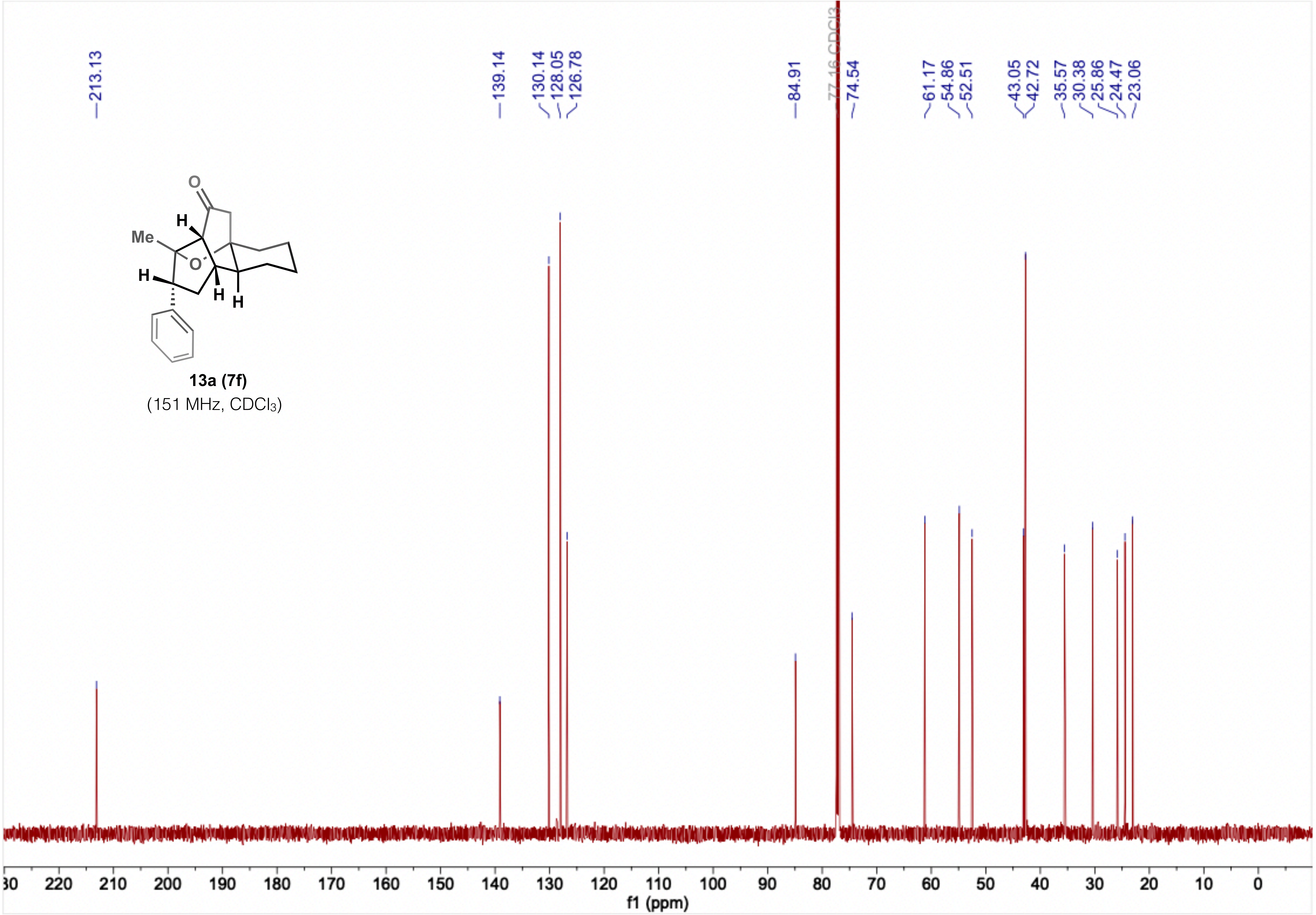

213.13
139.14
130.14
128.05
126.78
84.91
77.16 CDCl3
74.54
61.17
54.86
52.51
43.05
42.72
35.57
30.38
25.86
24.47
23.06
O
H
Me
O
H
H
H
13a (7f)
(151 MHz, CDCl3)
30 220 210 200 190 180 170 160 150 140 130 120 110 100 90 80 70 60 50 40 30 20 10 0
f1 (ppm)

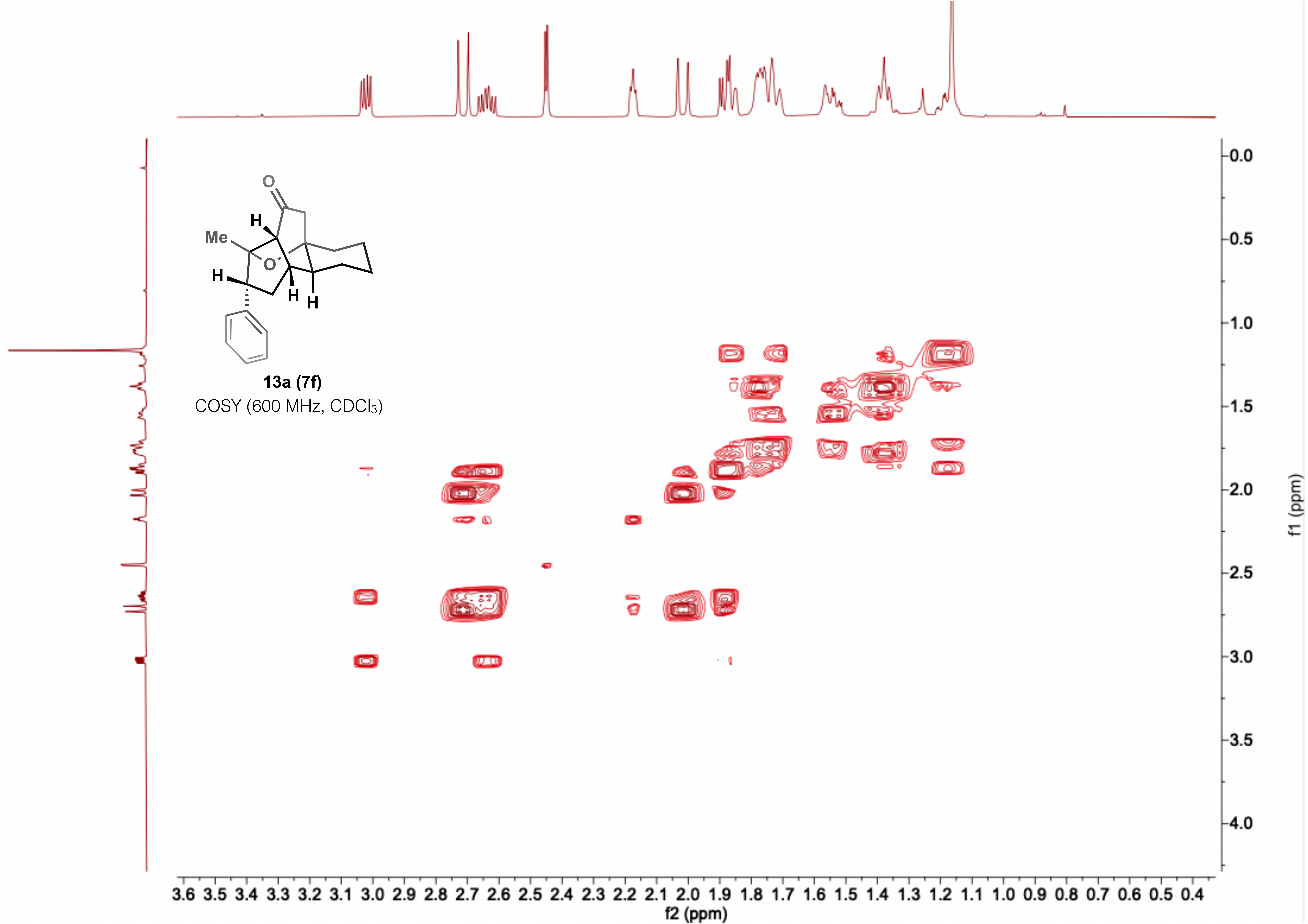
O
H
Me
O
H
H
H
13a (7f)
COSY (600 MHz, CDCl3)
0.0
0.5
1.0
1.5
2.0
2.5
3.0
3.5
4.0
f1 (ppm)
3.6 3.5 3.4 3.3 3.2 3.1 3.0 2.9 2.8 2.7 2.6 2.5 2.4 2.3 2.2 2.1 2.0 1.9 1.8 1.7 1.6 1.5 1.4 1.3 1.2 1.1 1.0 0.9 0.8 0.7 0.6 0.5 0.4
f2 (ppm)

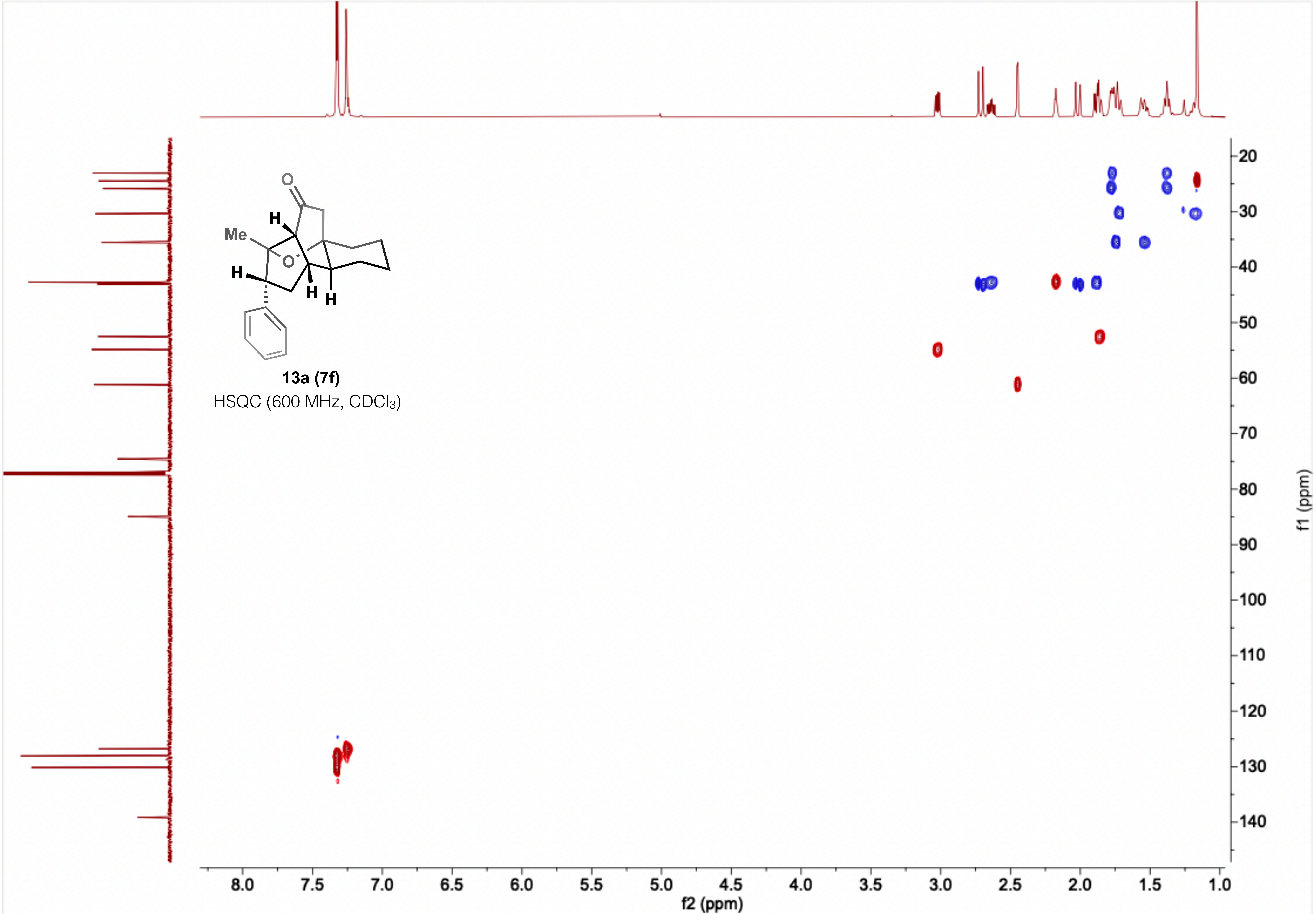
13a (7f)
HSQC (600 MHz, CDCl3)
f1 (ppm)
f2 (ppm)

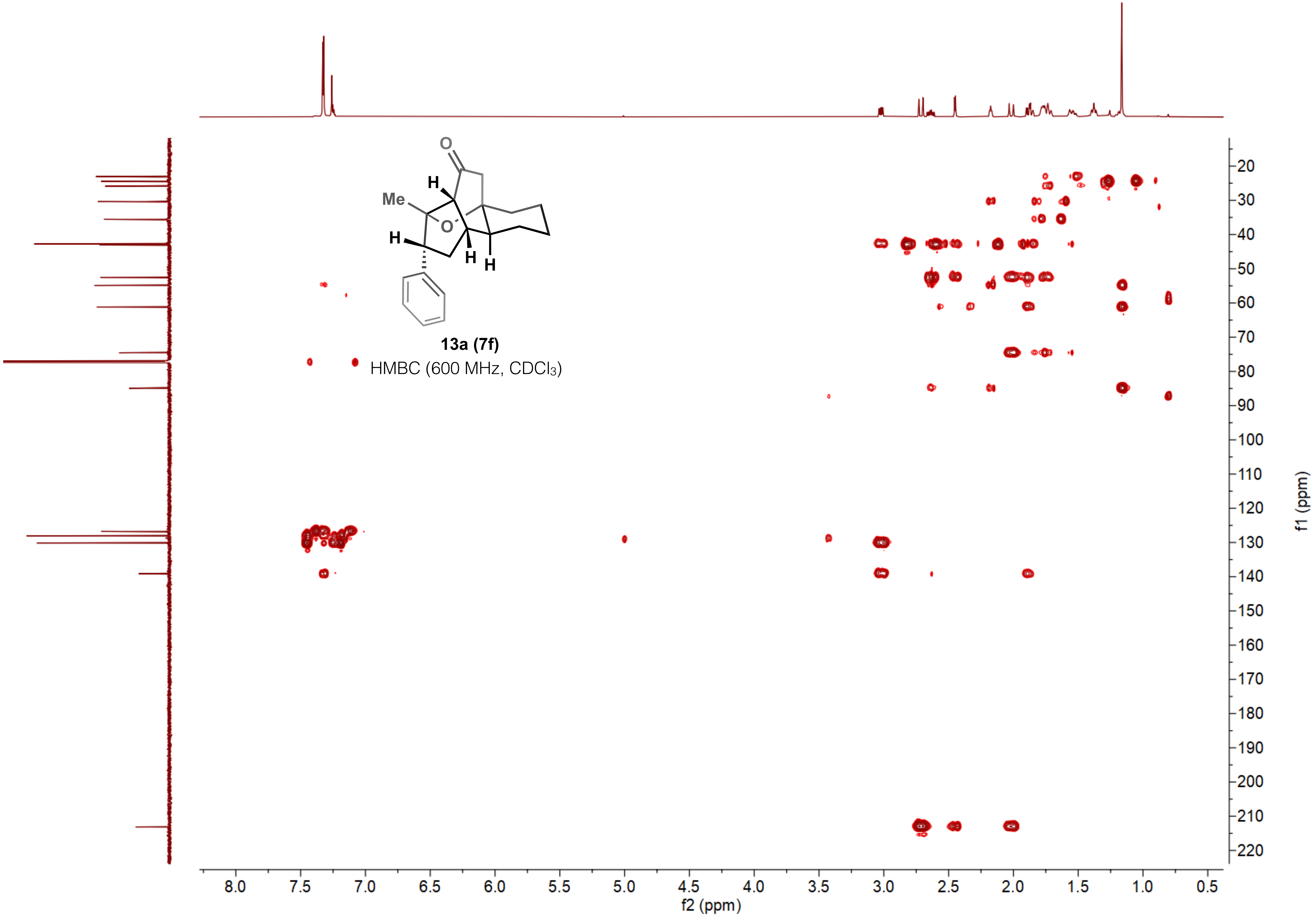

O
H
Me
O
H
H
H
13a (7f)
HMBC (600 MHz, CDCl$_3$)
f2 (ppm)
f1 (ppm)
8.0 7.5 7.0 6.5 6.0 5.5 5.0 4.5 4.0 3.5 3.0 2.5 2.0 1.5 1.0 0.5
20 30 40 50 60 70 80 90 100 110 120 130 140 150 160 170 180 190 200 210 220

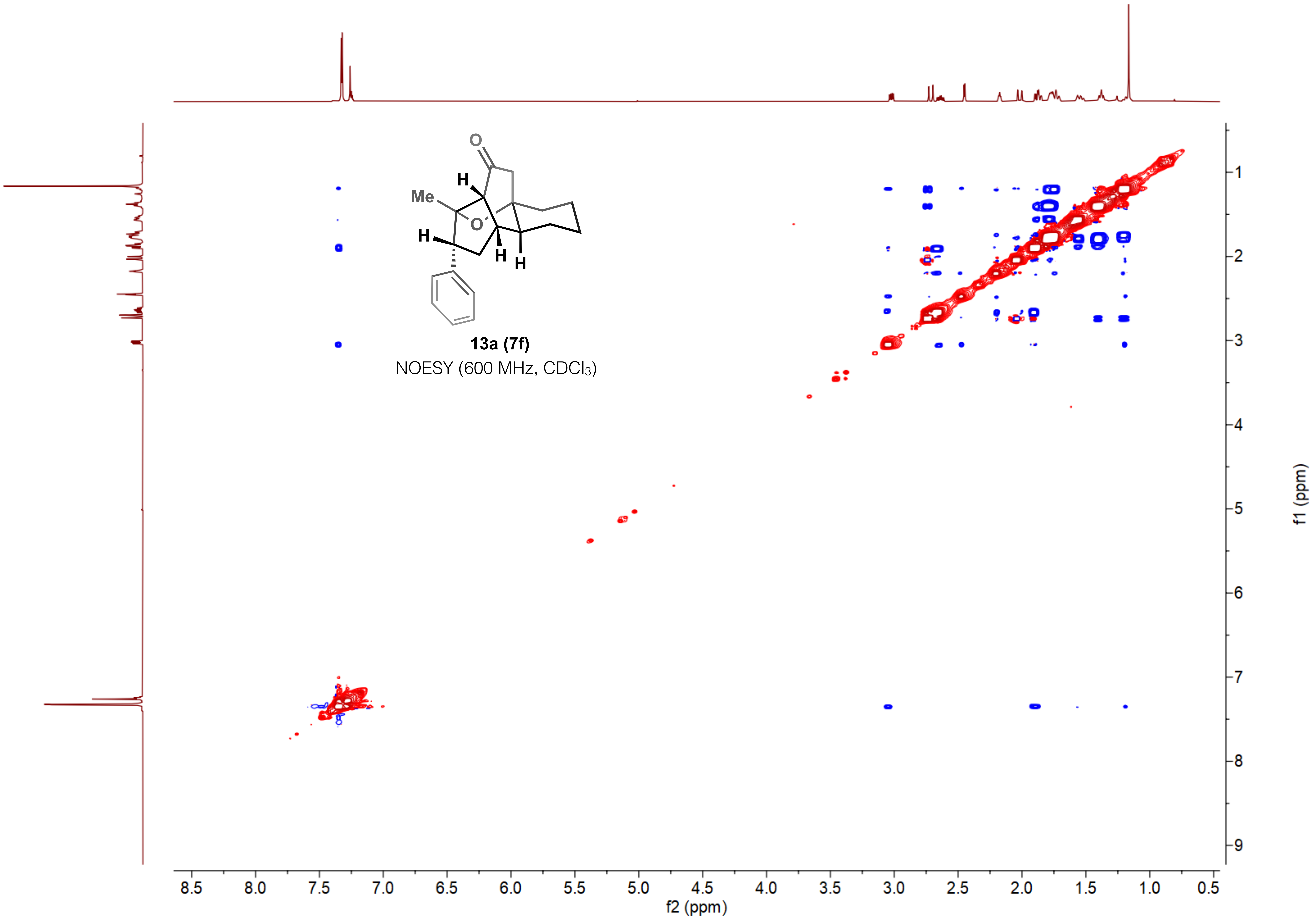
13a (7f)
NOESY (600 MHz, CDCl3)
Me
O
H
f2 (ppm)
f1 (ppm)

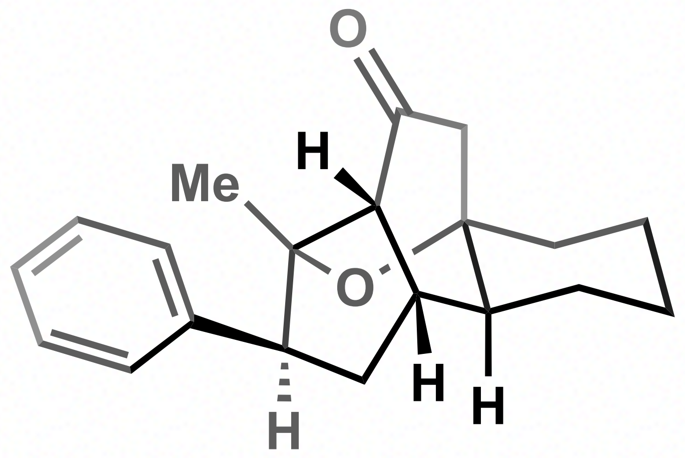

***epi*-13a (8f)**

(600 MHz, $CDCl_3$)

NB: ca. 5% inseparable impurities present

NB: compound was not probed for KOR agonism

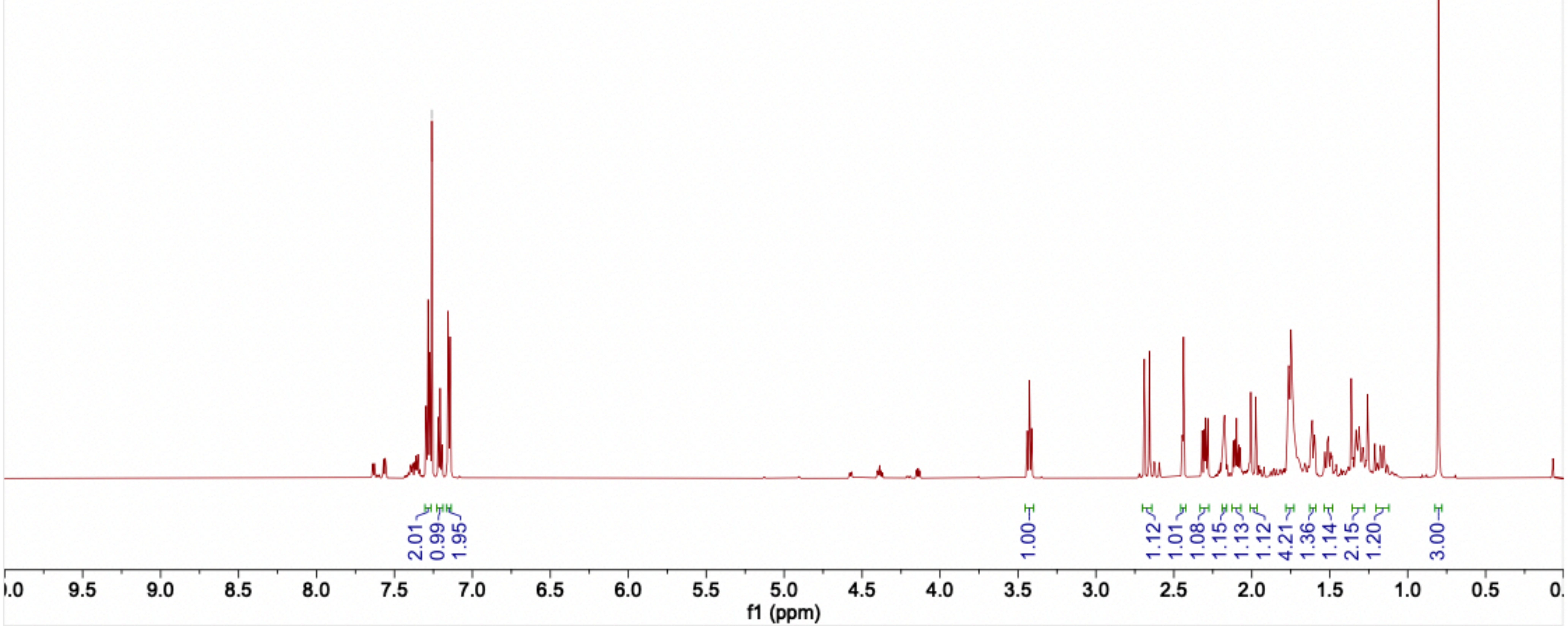

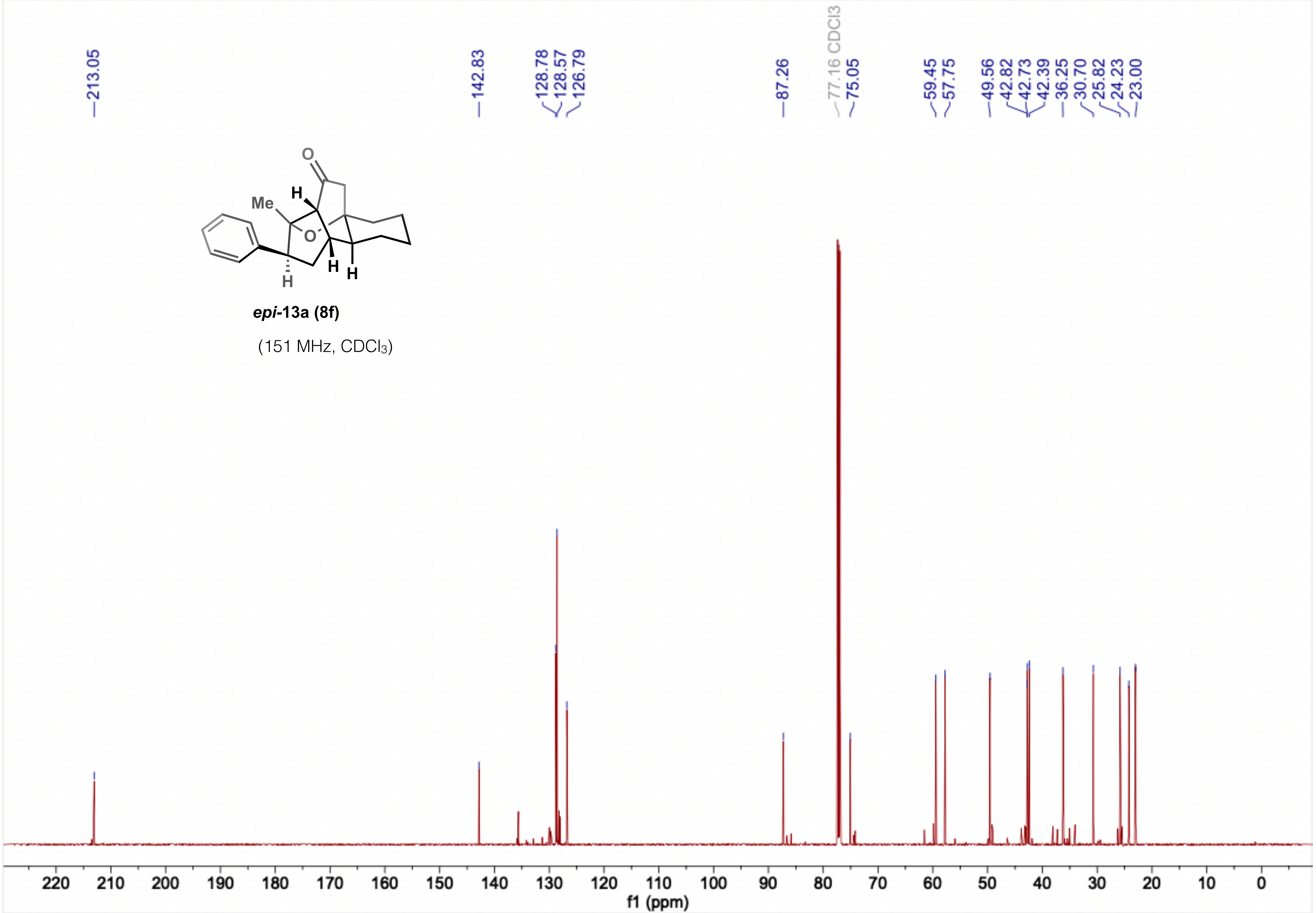
213.05
142.83
128.78
128.57
126.79
87.26
77.16 CDCl3
75.05
59.45
57.75
49.56
42.82
42.73
42.39
36.25
30.70
25.82
24.23
23.00
Me
H
O
H
H
H
epi-13a (8f)
(151 MHz, CDCl3)
220 210 200 190 180 170 160 150 140 130 120 110 100 90 80 70 60 50 40 30 20 10 0
f1 (ppm)

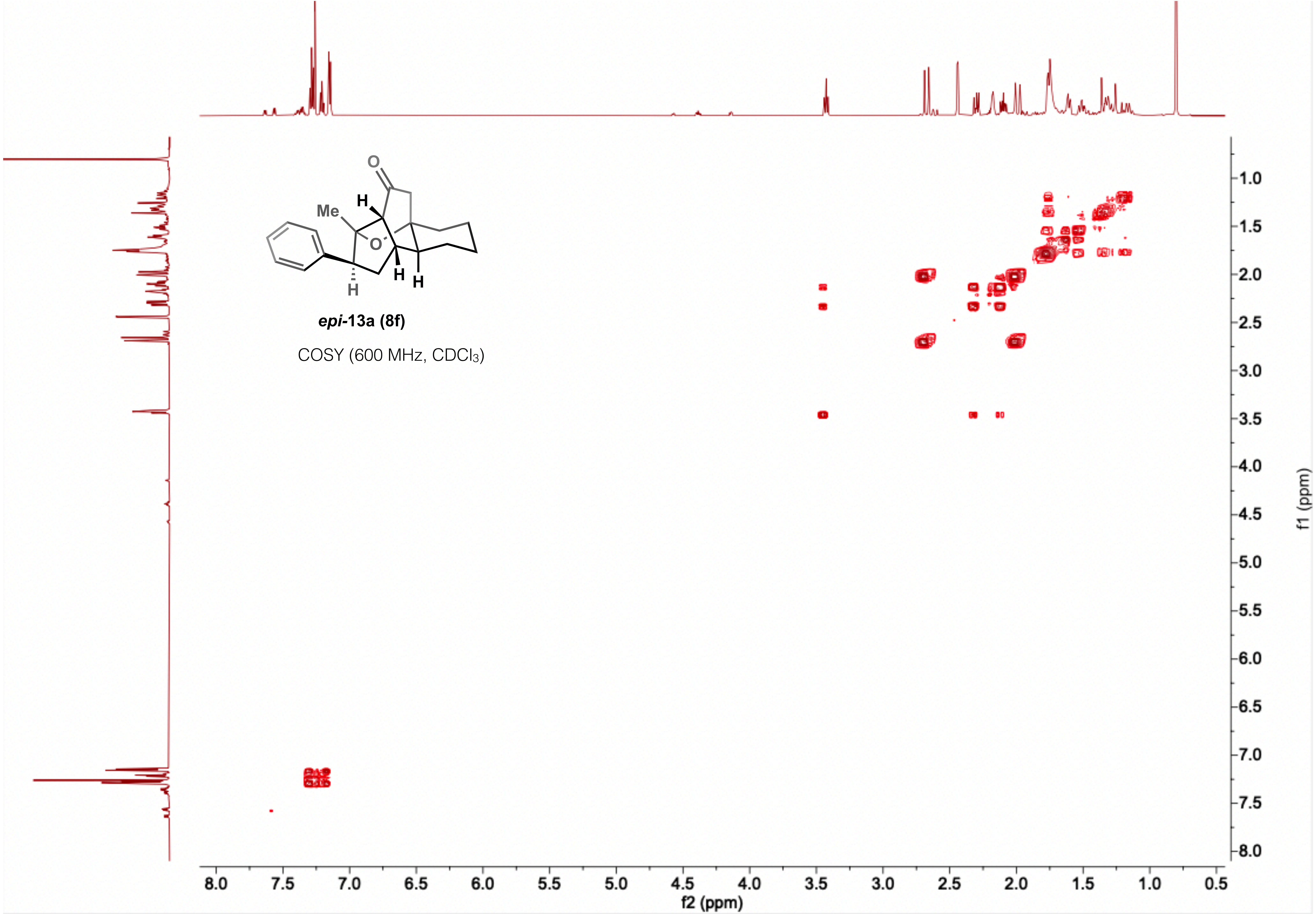
epi-13a (8f)
COSY (600 MHz, CDCl3)
f2 (ppm)
f1 (ppm)

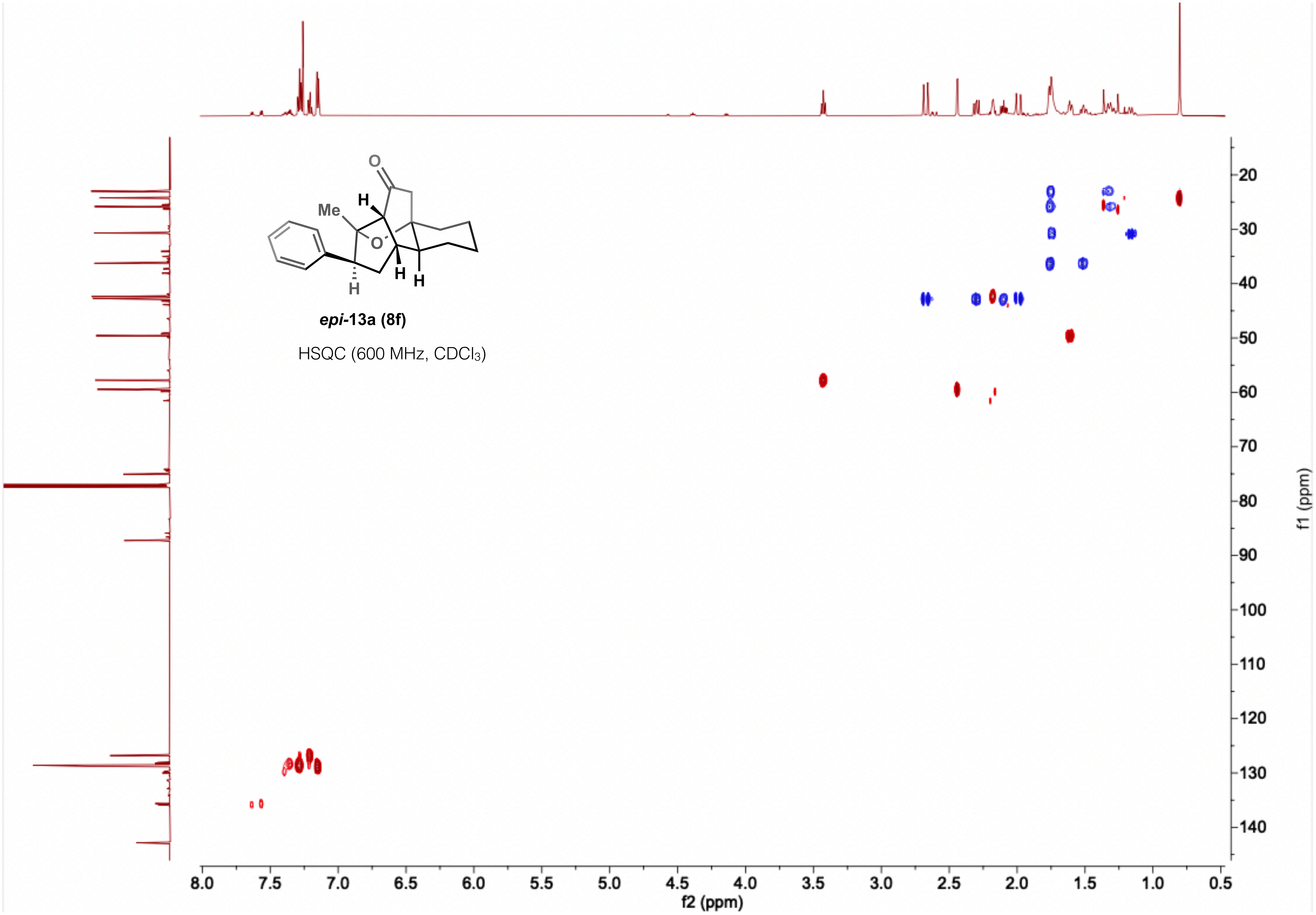
O
Me
H
O
H
H
H
epi-13a (8f)
HSQC (600 MHz, CDCl3)
f2 (ppm)
f1 (ppm)
8.0 7.5 7.0 6.5 6.0 5.5 5.0 4.5 4.0 3.5 3.0 2.5 2.0 1.5 1.0 0.5
20 30 40 50 60 70 80 90 100 110 120 130 140

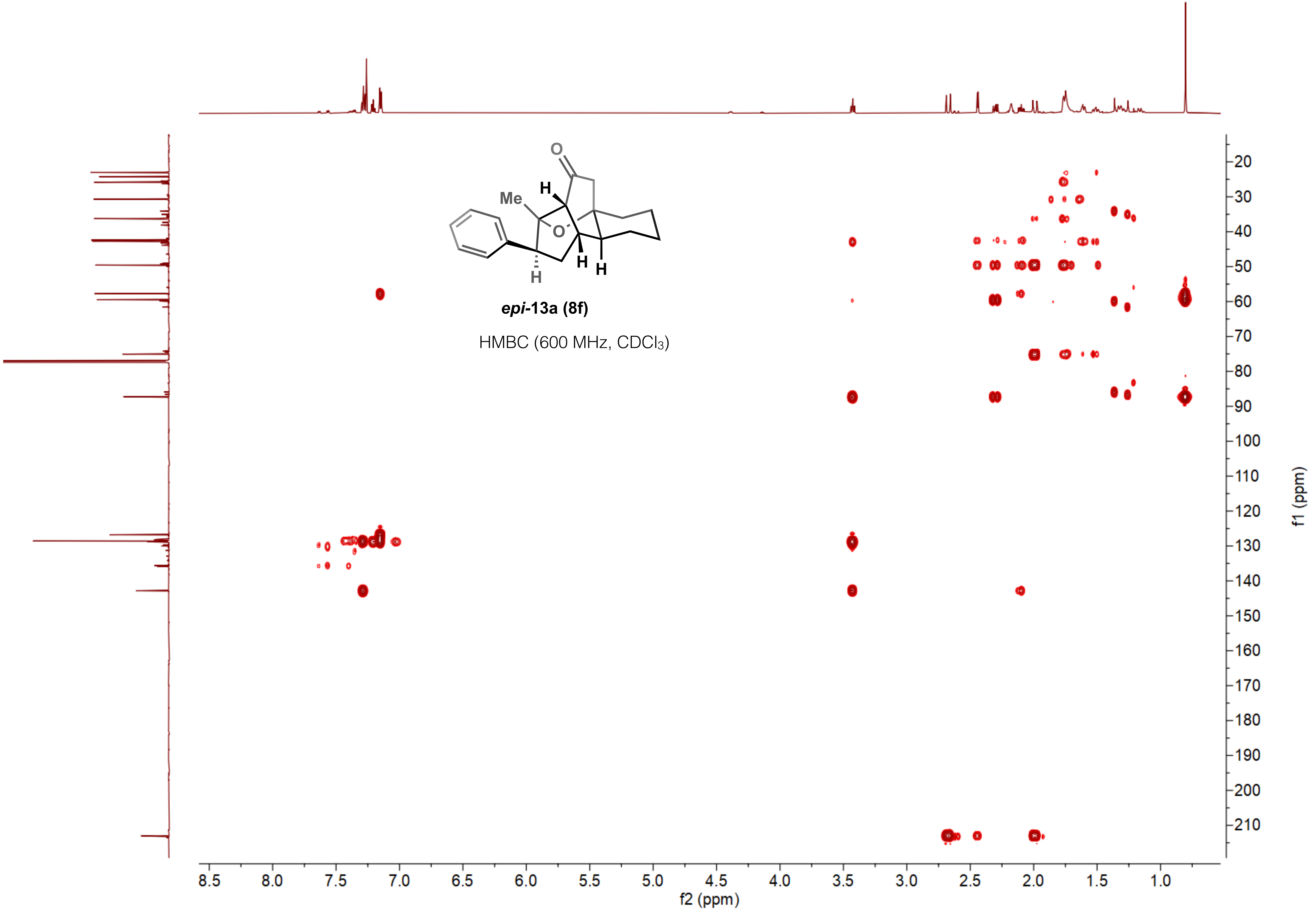

epi-13a (8f)
HMBC (600 MHz, CDCl3)
f2 (ppm)
f1 (ppm)

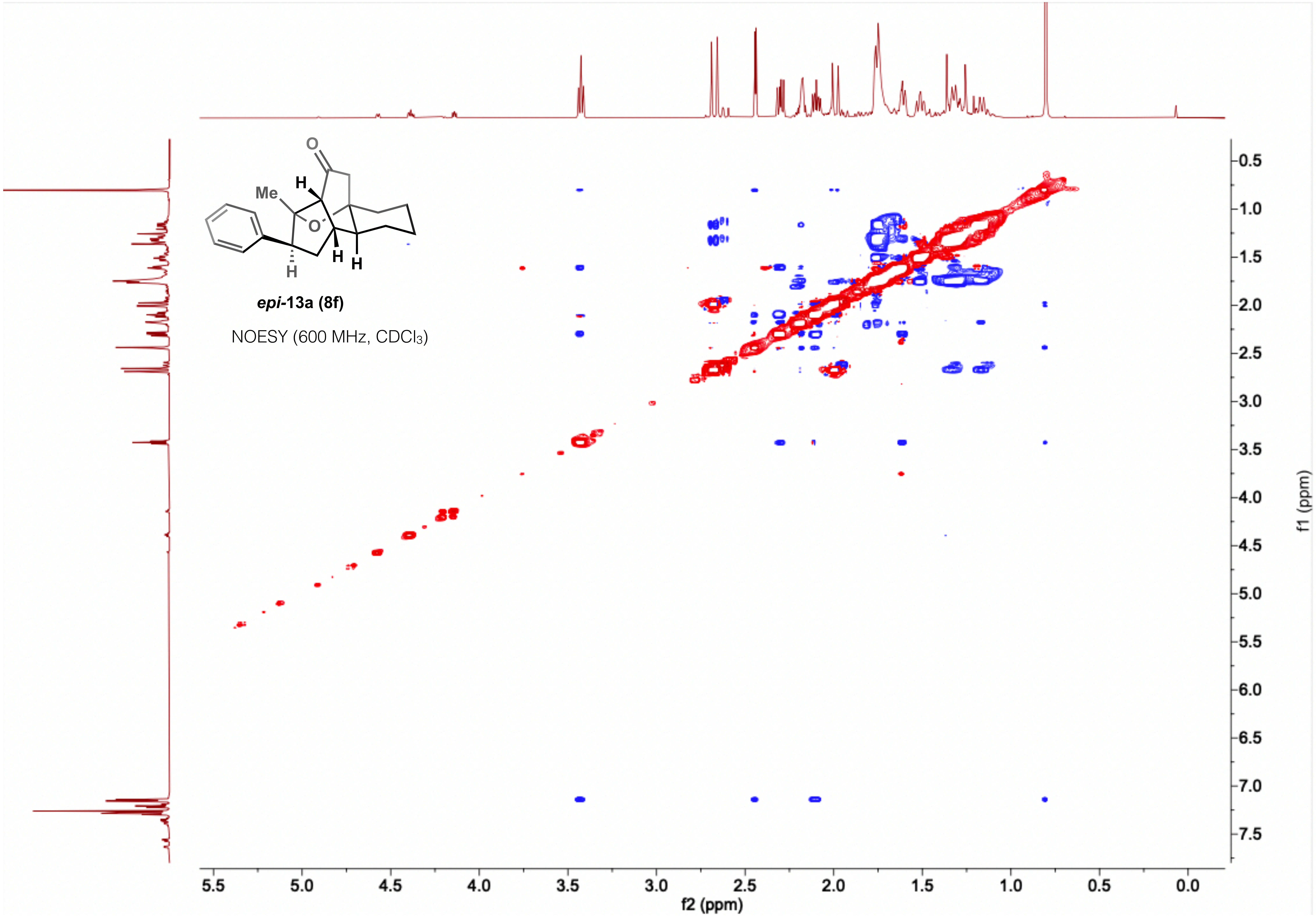

O
Me
H
O
H
H
H
epi-13a (8f)
NOESY (600 MHz, $CDCl_3$)
f2 (ppm)
f1 (ppm)

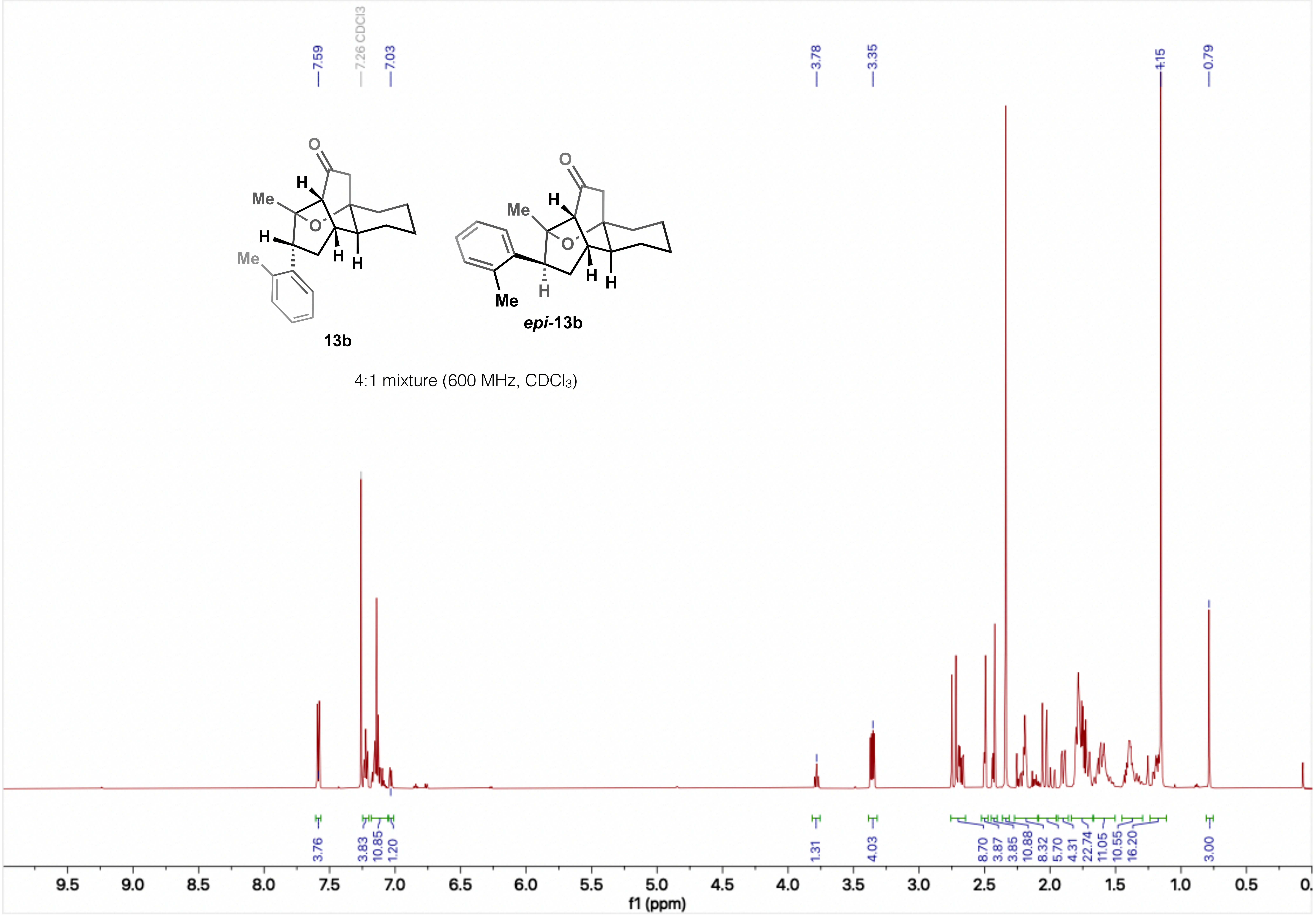
7.59
7.26 CDCl3
7.03
3.78
3.35
1.15
0.79
13b
epi-13b
4:1 mixture (600 MHz, CDCl3)
3.76
3.83
10.85
1.20
1.31
4.03
8.70
3.87
3.85
10.88
8.32
5.70
4.31
22.74
11.05
10.55
16.20
3.00
f1 (ppm)

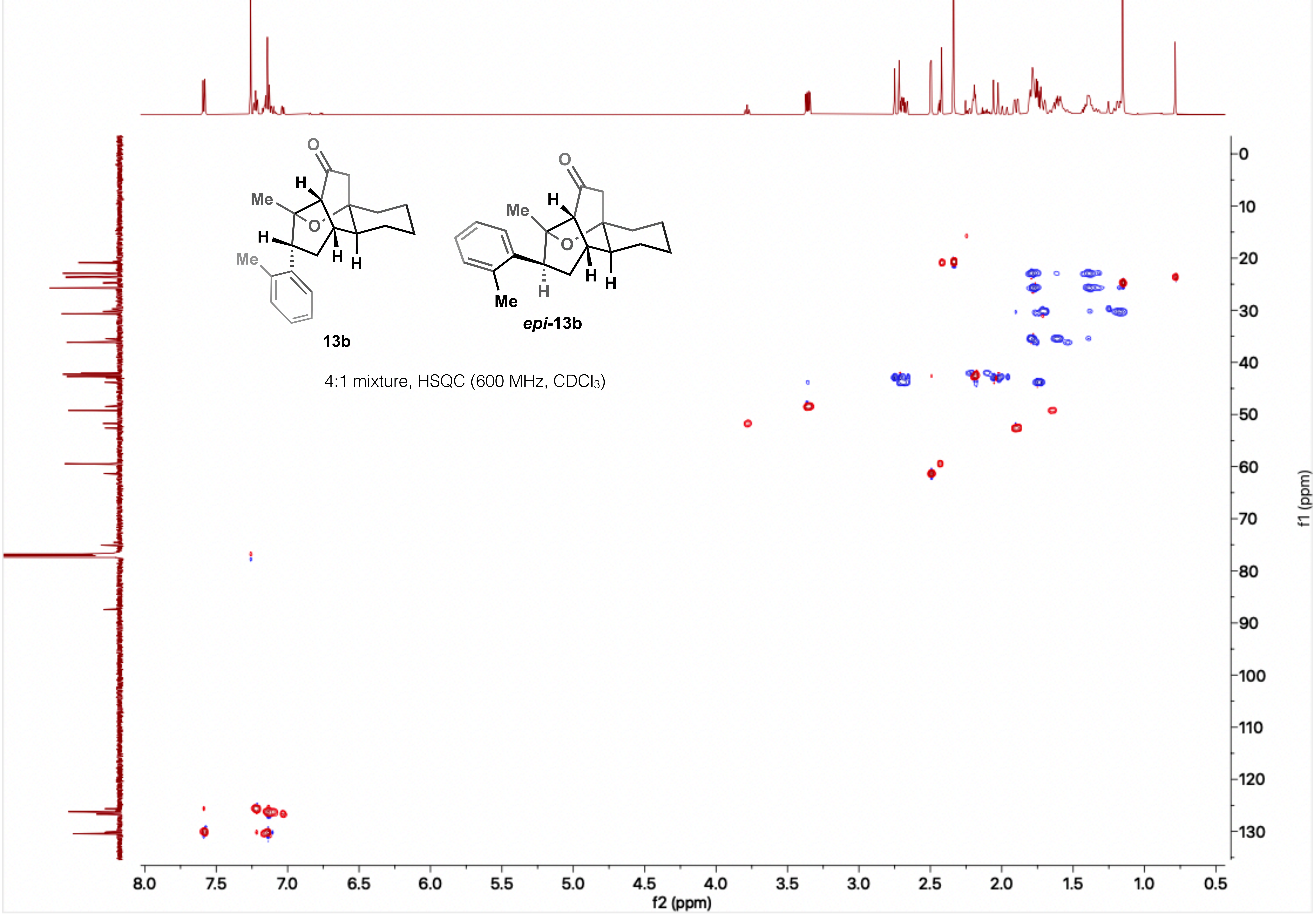

13b
epi-13b
4:1 mixture, HSQC (600 MHz, CDCl3)
f2 (ppm)
f1 (ppm)

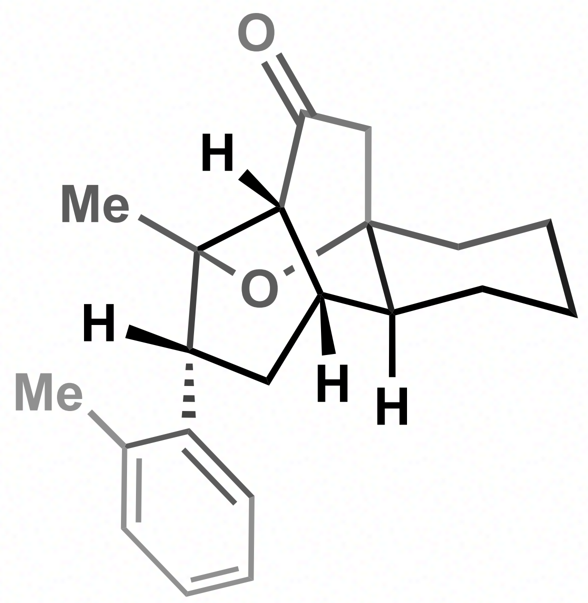

13b

(600 MHz, $CDCl_3$)

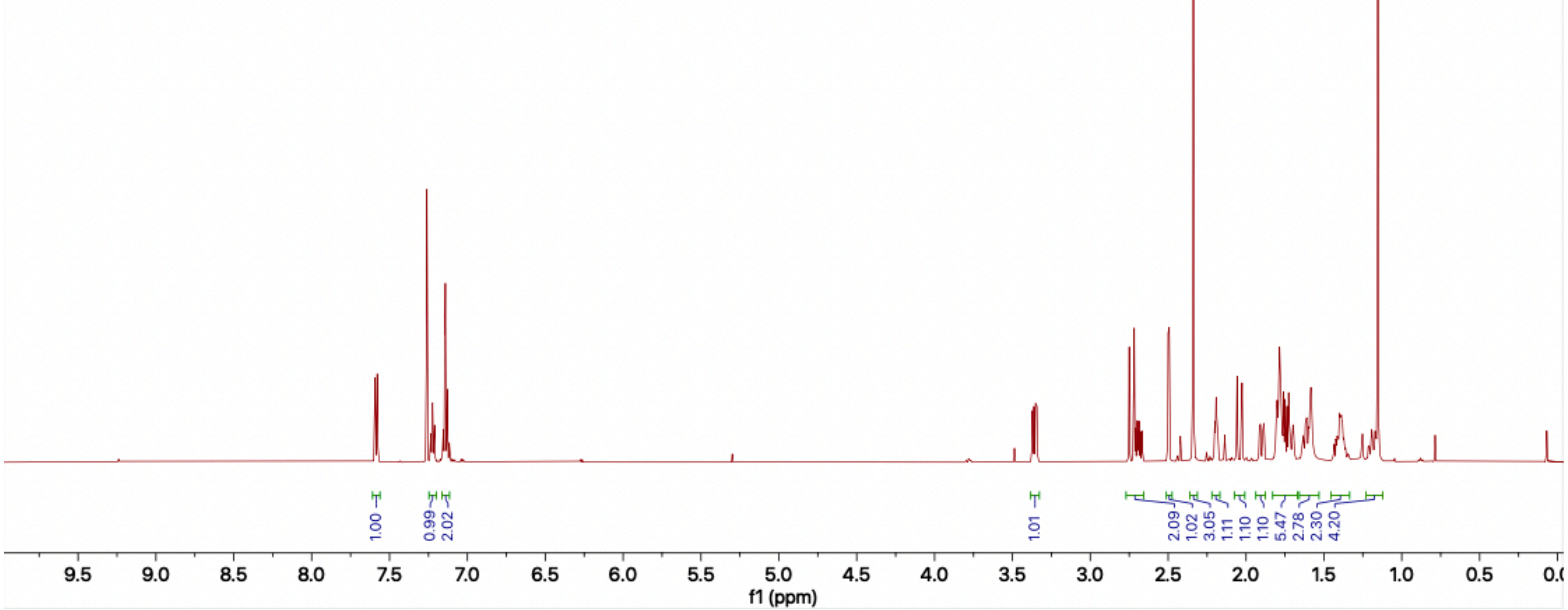

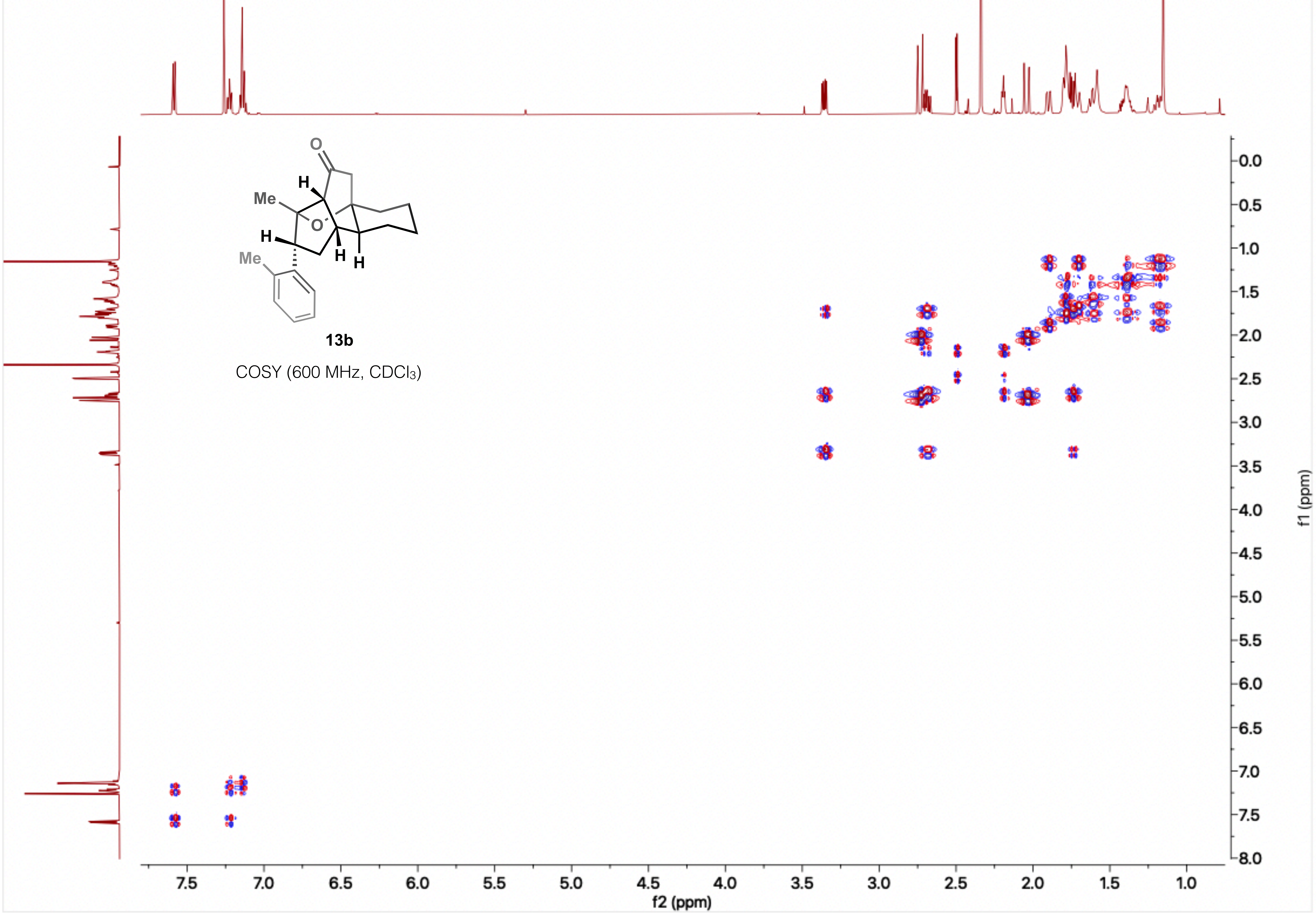

O
H
Me
O
H
Me
H
H
13b
COSY (600 MHz, CDCl3)
0.0
0.5
1.0
1.5
2.0
2.5
3.0
3.5
4.0
4.5
5.0
5.5
6.0
6.5
7.0
7.5
8.0
f1 (ppm)
7.5
7.0
6.5
6.0
5.5
5.0
4.5
4.0
3.5
3.0
2.5
2.0
1.5
1.0
f2 (ppm)

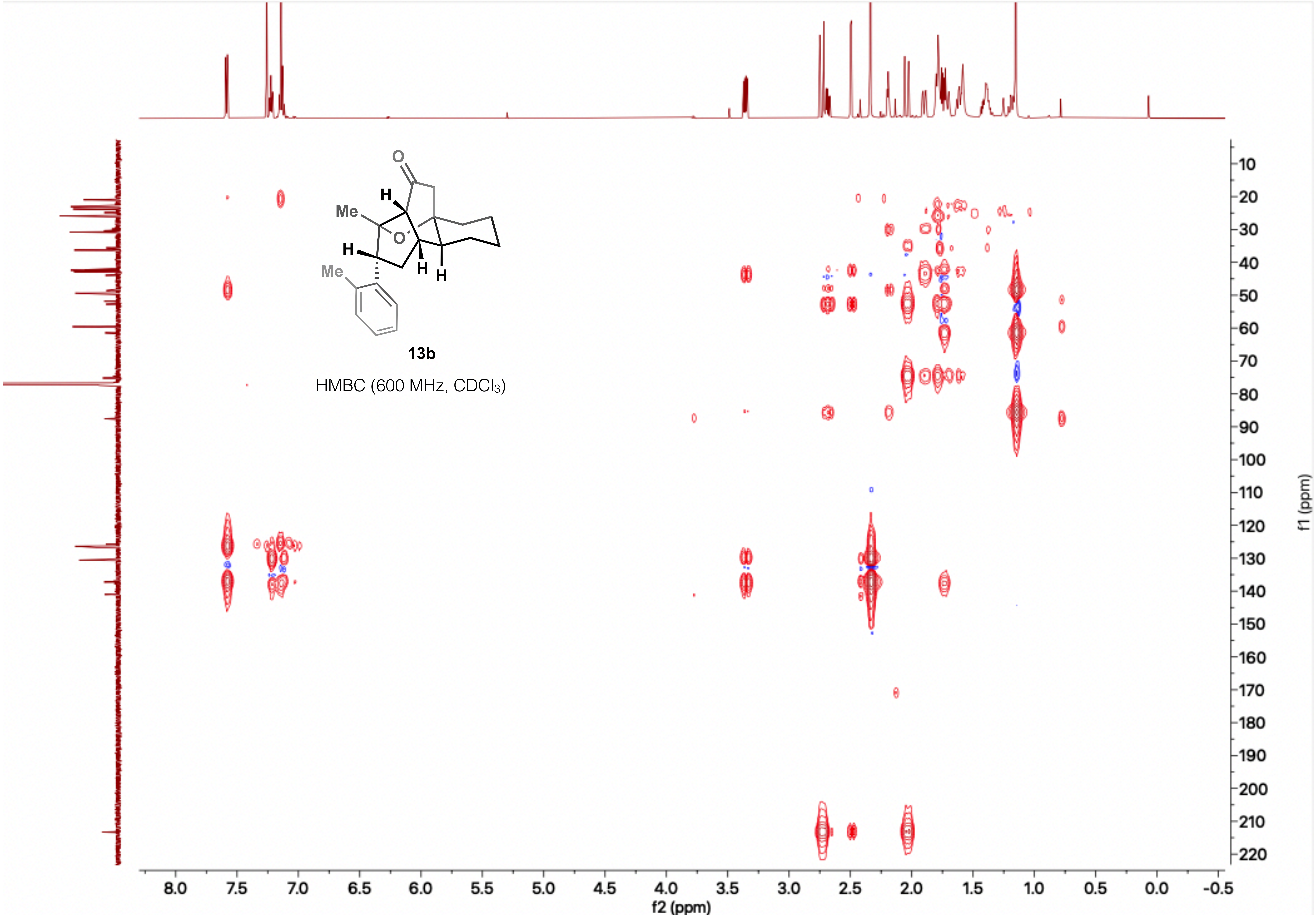

O
H
Me
O
H
H
H
Me
13b
HMBC (600 MHz, CDCl3)
f2 (ppm)
f1 (ppm)

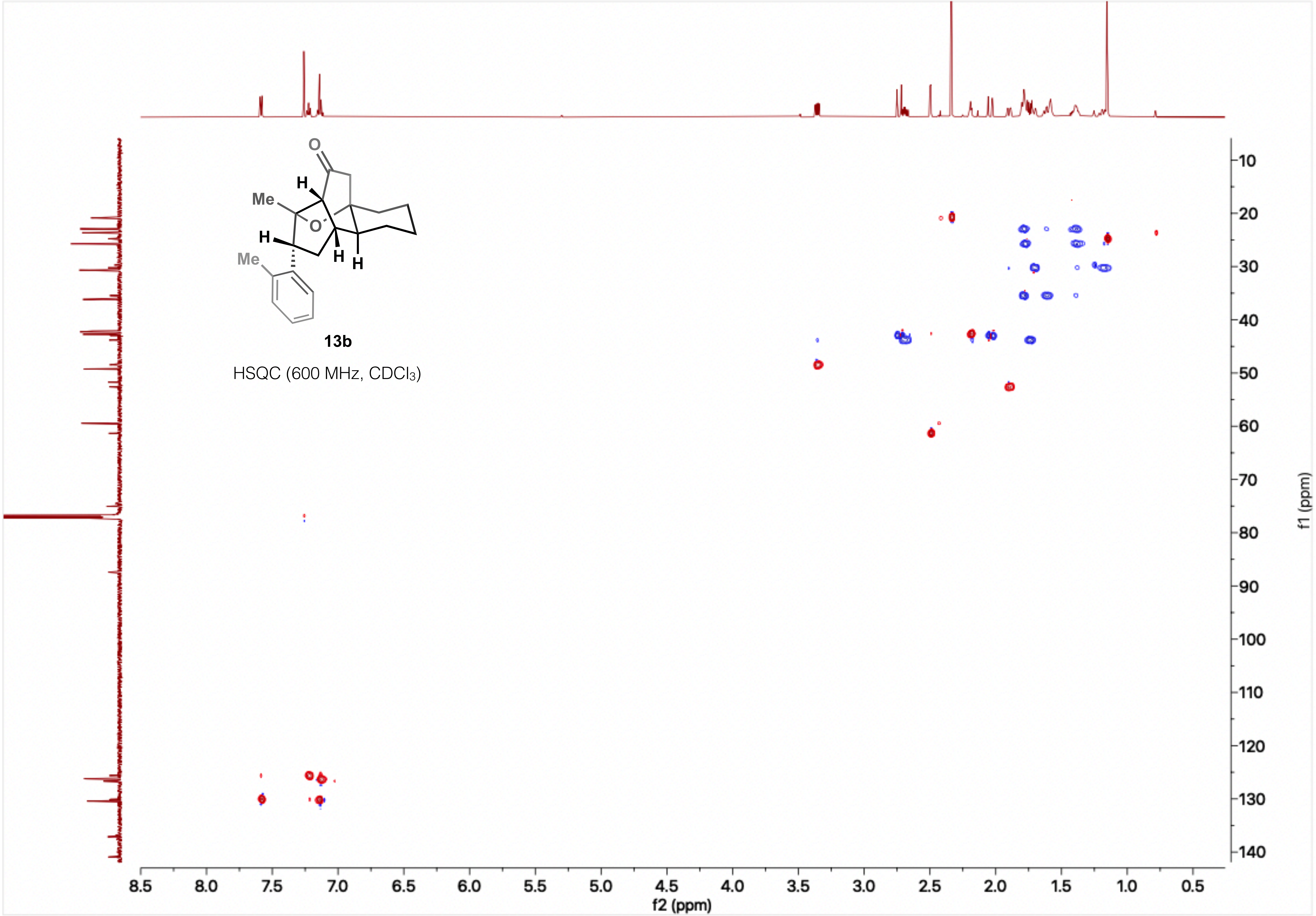

O
H
Me
O
H
Me
H
H
13b
HSQC (600 MHz, CDCl3)
f2 (ppm)
f1 (ppm)

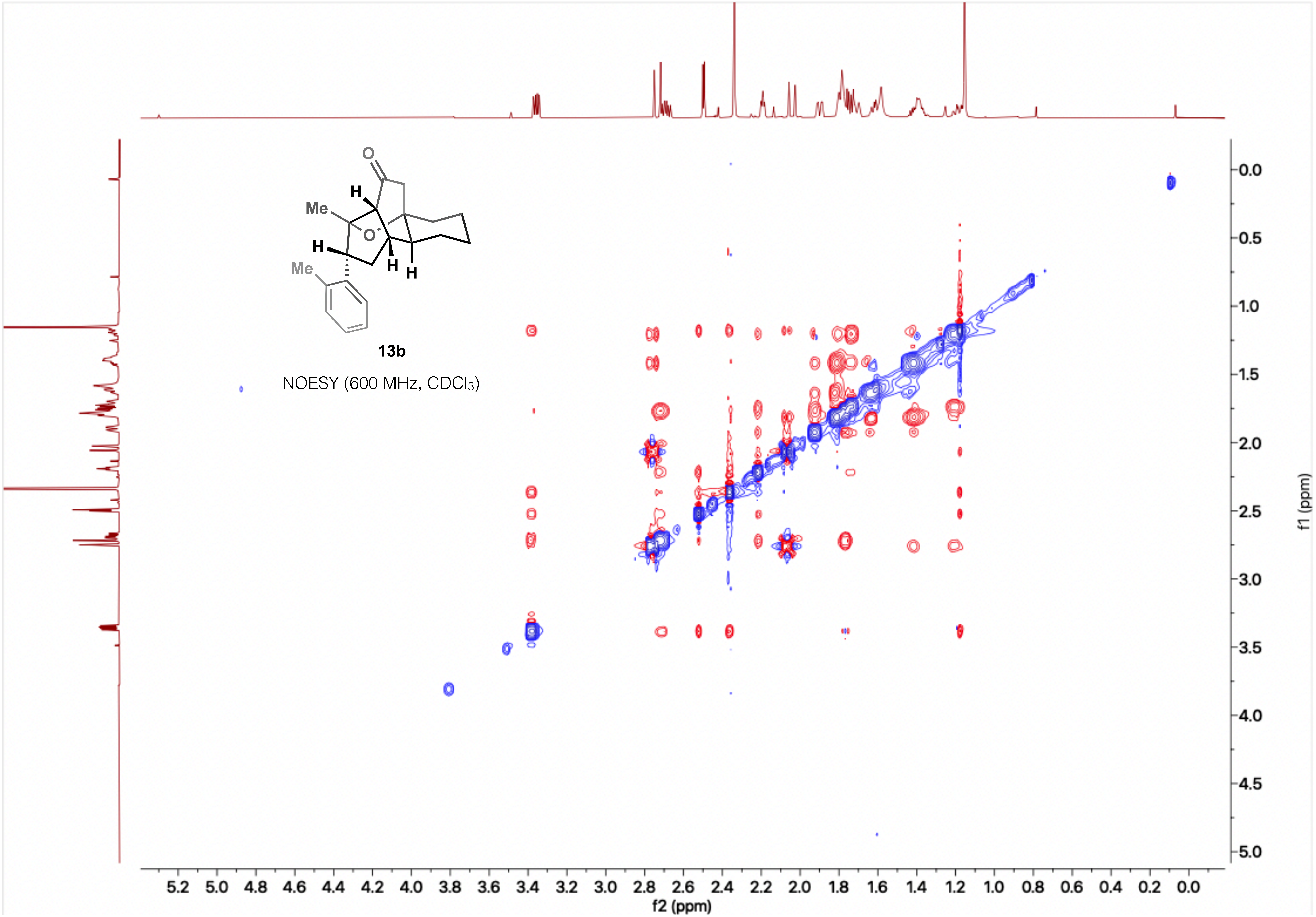

O
H
Me
O
H
Me
H
H
13b
NOESY (600 MHz, CDCl$_3$)
f2 (ppm)
f1 (ppm)

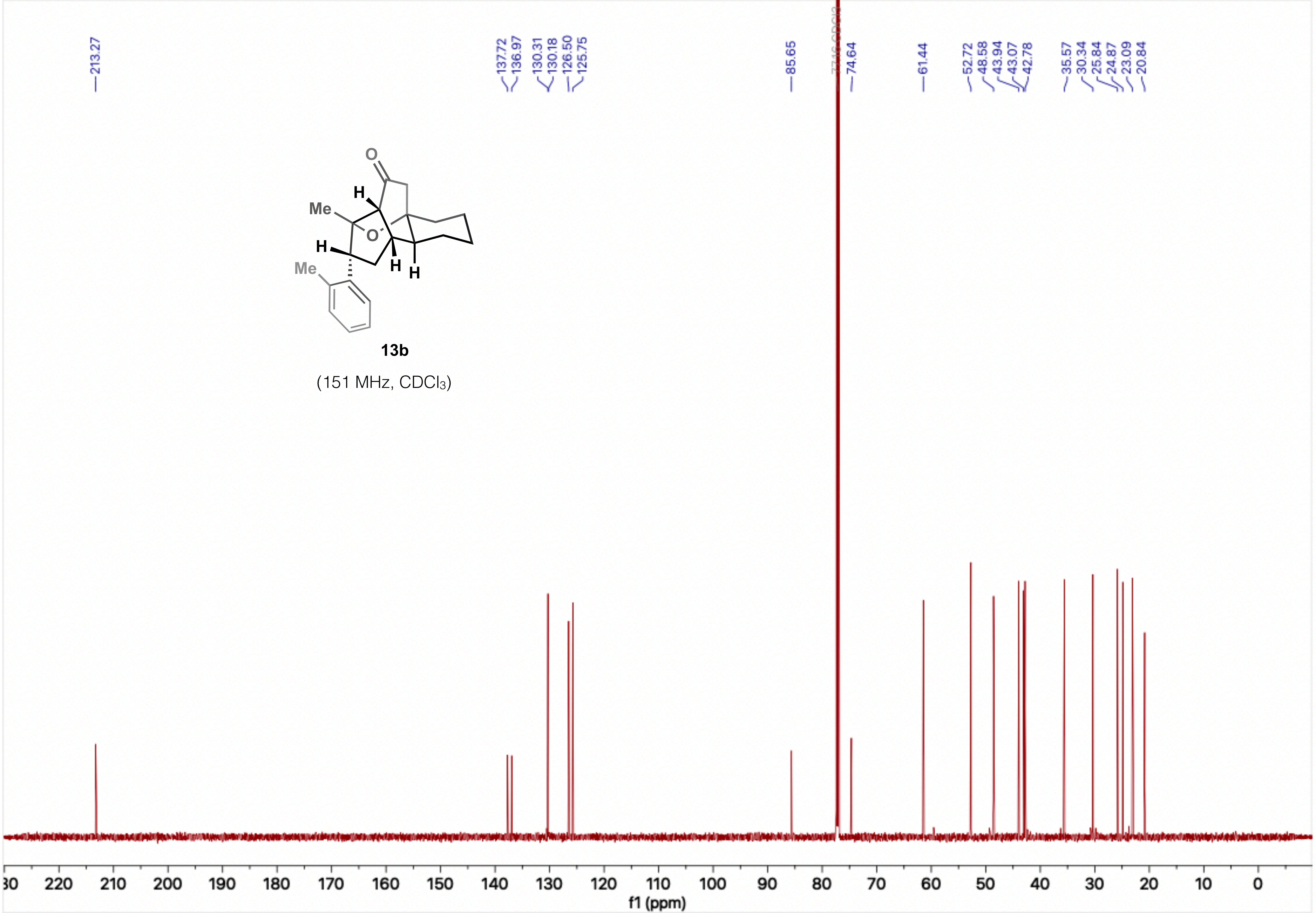

213.27
137.72
136.97
130.31
130.18
126.50
125.75
85.65
74.64
61.44
52.72
48.58
43.94
43.07
42.78
35.57
30.34
25.84
24.87
23.09
20.84
O
H
Me
O
H
Me
H
H
13b
(151 MHz, CDCl3)
30 220 210 200 190 180 170 160 150 140 130 120 110 100 90 80 70 60 50 40 30 20 10 0
f1 (ppm)

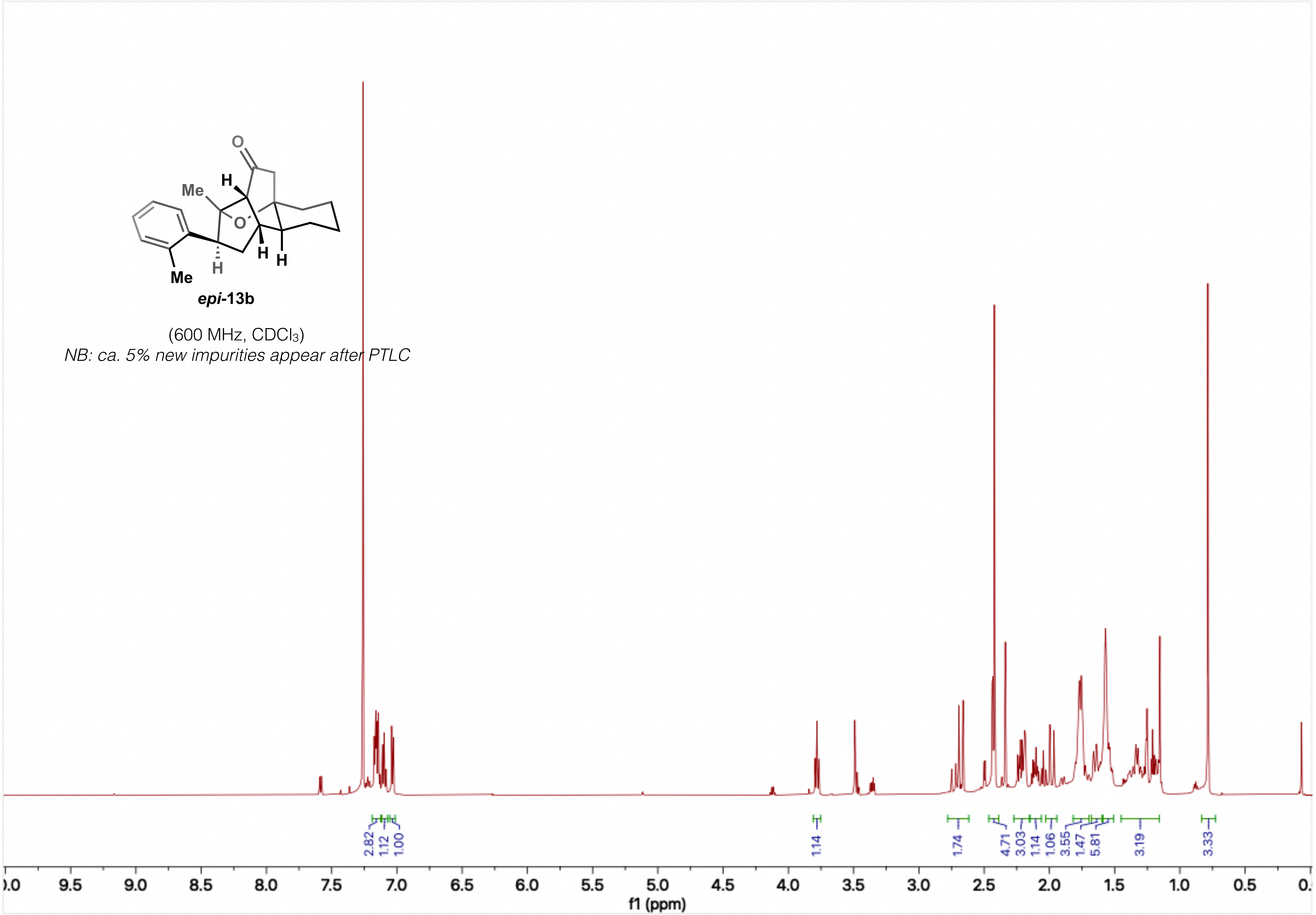
O
H
Me
O
H
H
H
Me
epi-13b
(600 MHz, CDCl3)
NB: ca. 5% new impurities appear after PTLC
2.82
1.12
1.00
1.14
1.74
4.71
3.03
1.14
1.06
3.55
1.47
5.81
3.19
3.33
9.5
9.0
8.5
8.0
7.5
7.0
6.5
6.0
5.5
5.0
4.5
4.0
3.5
3.0
2.5
2.0
1.5
1.0
0.5
f1 (ppm)

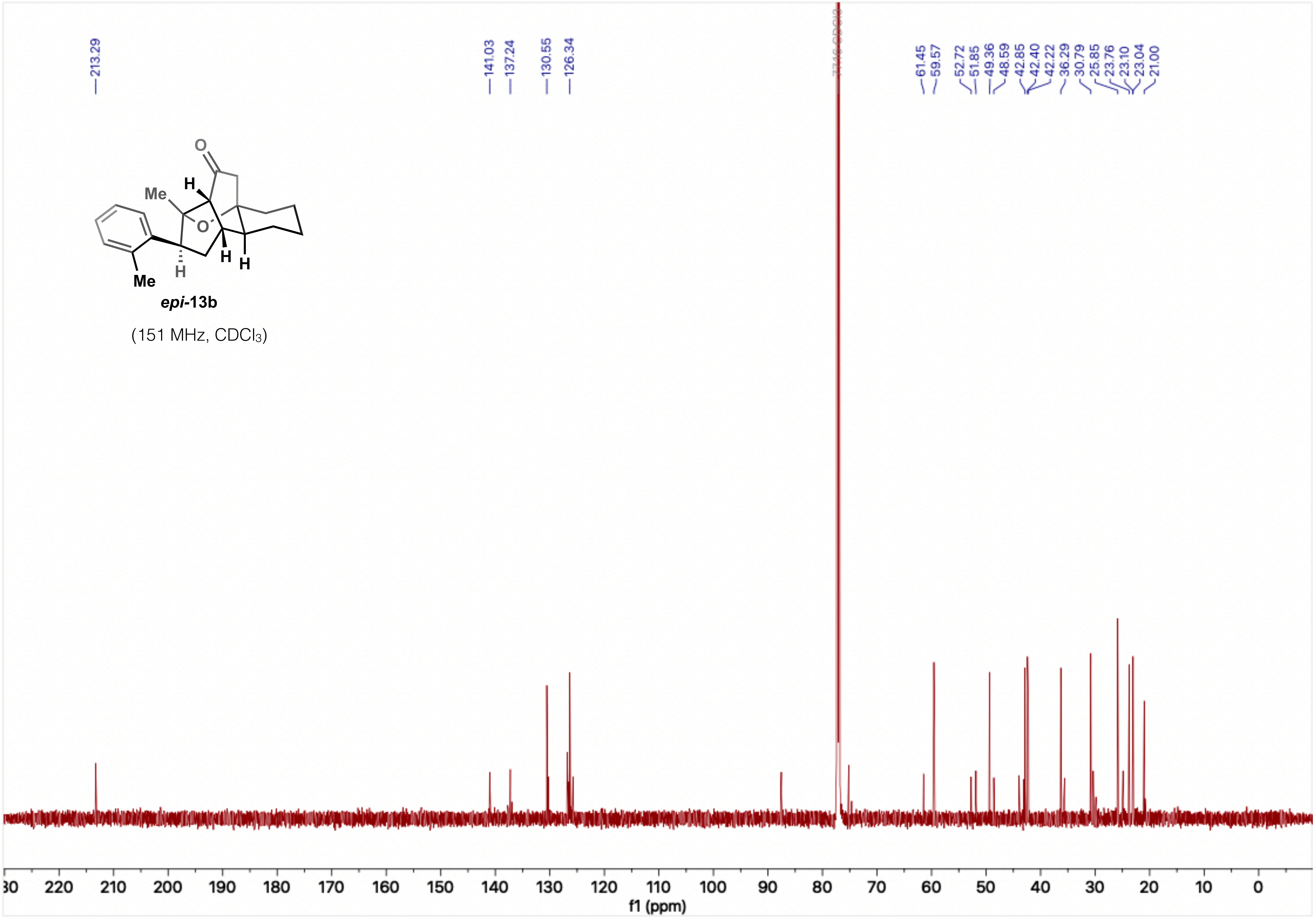
213.29
141.03
137.24
130.55
126.34
61.45
59.57
52.72
51.85
49.36
48.59
42.85
42.40
42.22
36.29
30.79
25.85
23.76
23.10
23.04
21.00
O
Me
H
O
H
H
H
Me
epi-13b
(151 MHz, CDCl3)
30
220
210
200
190
180
170
160
150
140
130
120
110
100
90
80
70
60
50
40
30
20
10
0
f1 (ppm)

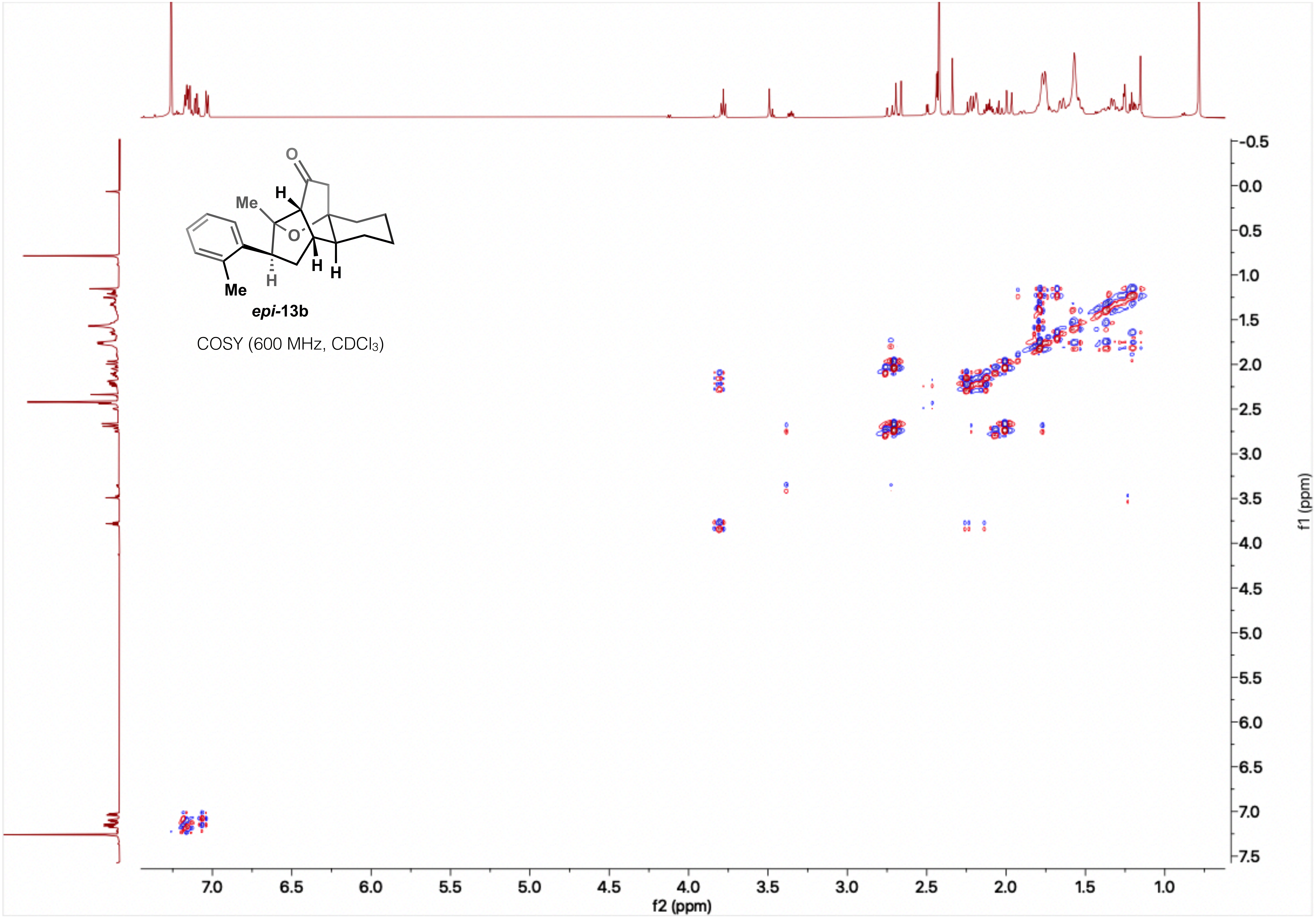

epi-13b
COSY (600 MHz, CDCl3)
f2 (ppm)
f1 (ppm)

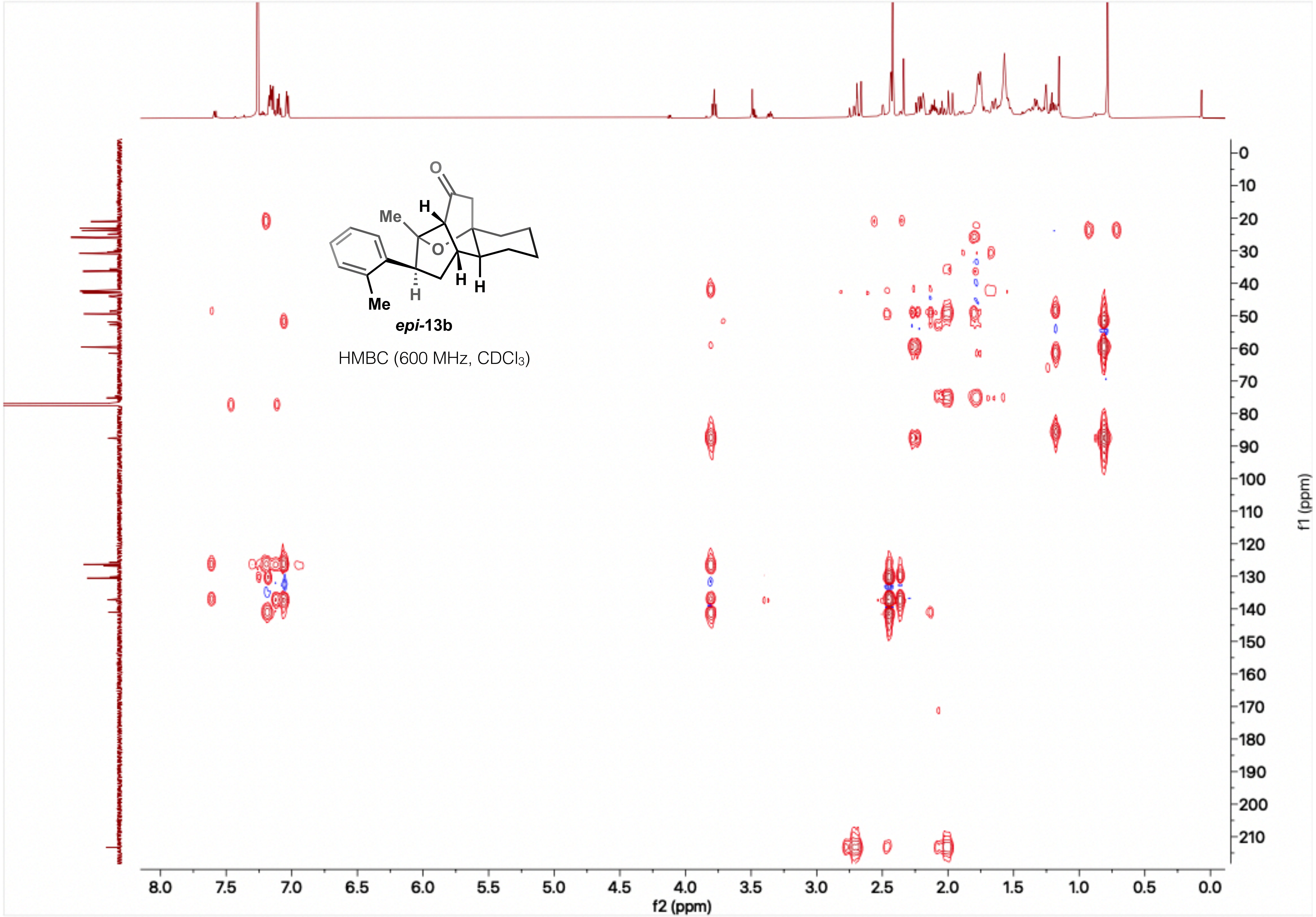

O
Me
H
O
H
H
H
Me
epi-13b
HMBC (600 MHz, CDCl3)
f2 (ppm)
f1 (ppm)

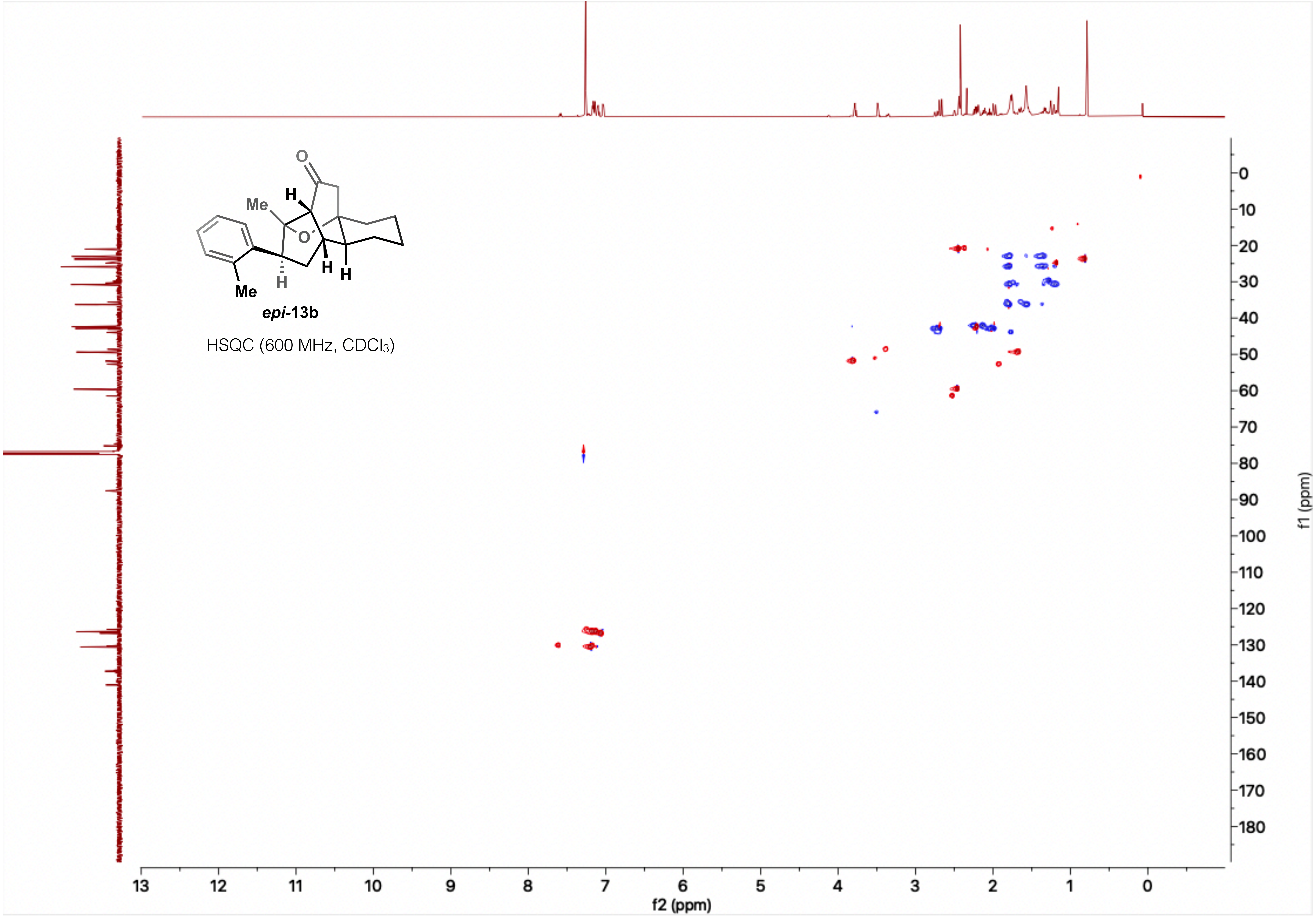
O
Me
H
O
H
H
Me
H
epi-13b
HSQC (600 MHz, CDCl3)
f2 (ppm)
f1 (ppm)
13 12 11 10 9 8 7 6 5 4 3 2 1 0
0 10 20 30 40 50 60 70 80 90 100 110 120 130 140 150 160 170 180

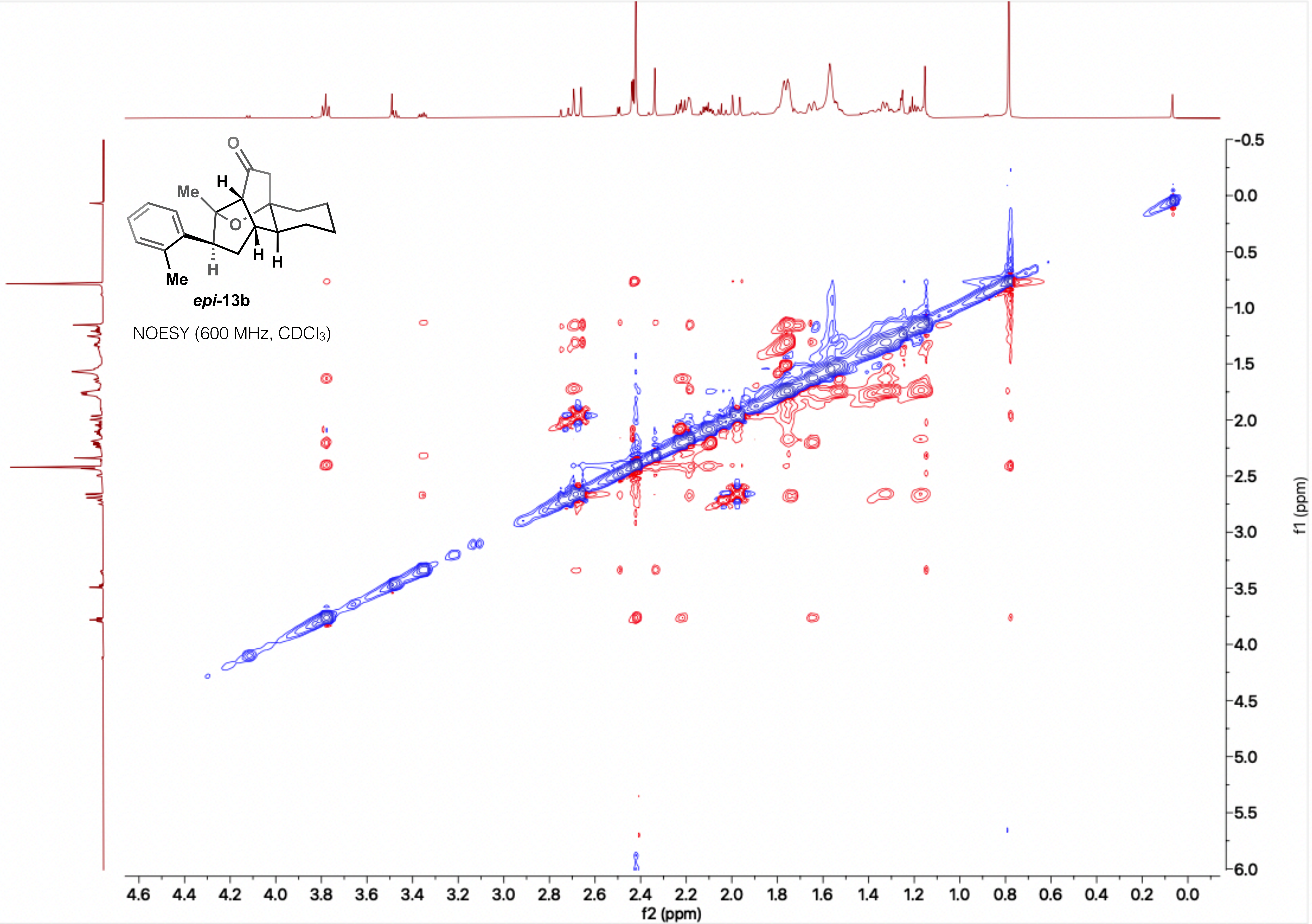
O
H
Me
O
H
H
H
Me
epi-13b
NOESY (600 MHz, CDCl3)
f1 (ppm)
f2 (ppm)

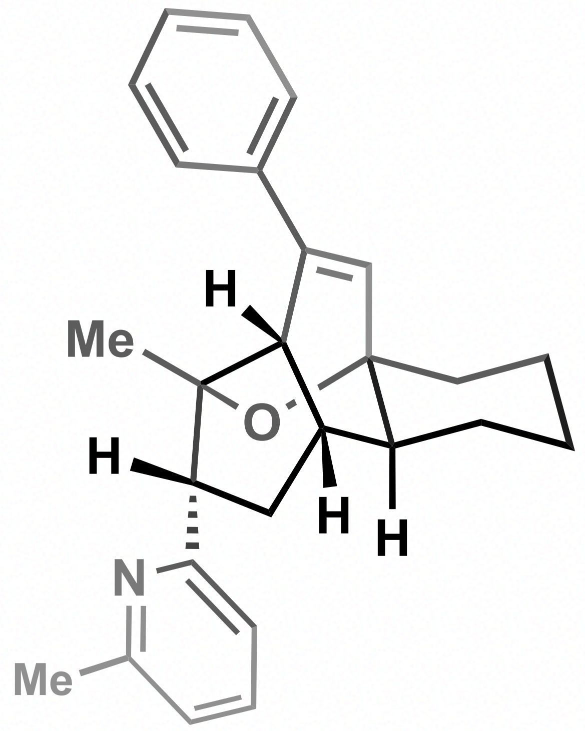

14

(600 MHz, CD_2Cl_2)

4 mol% EtOAc

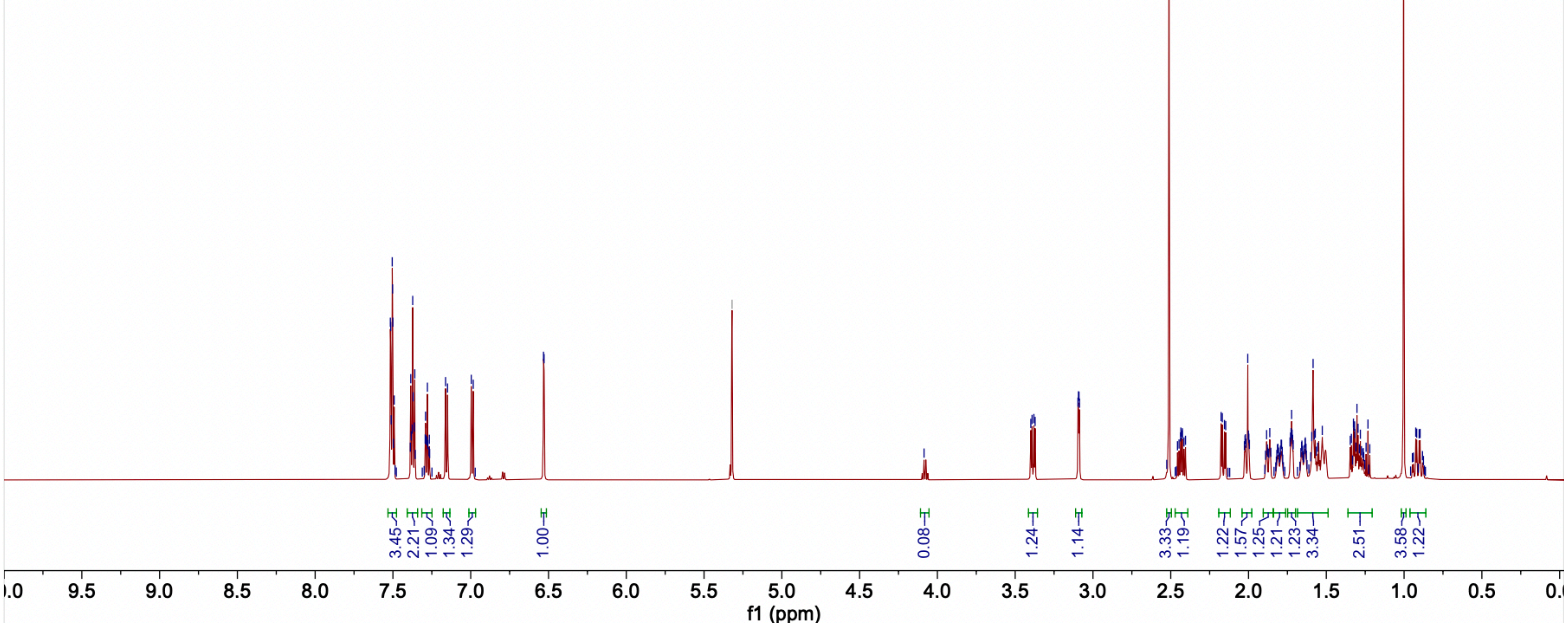

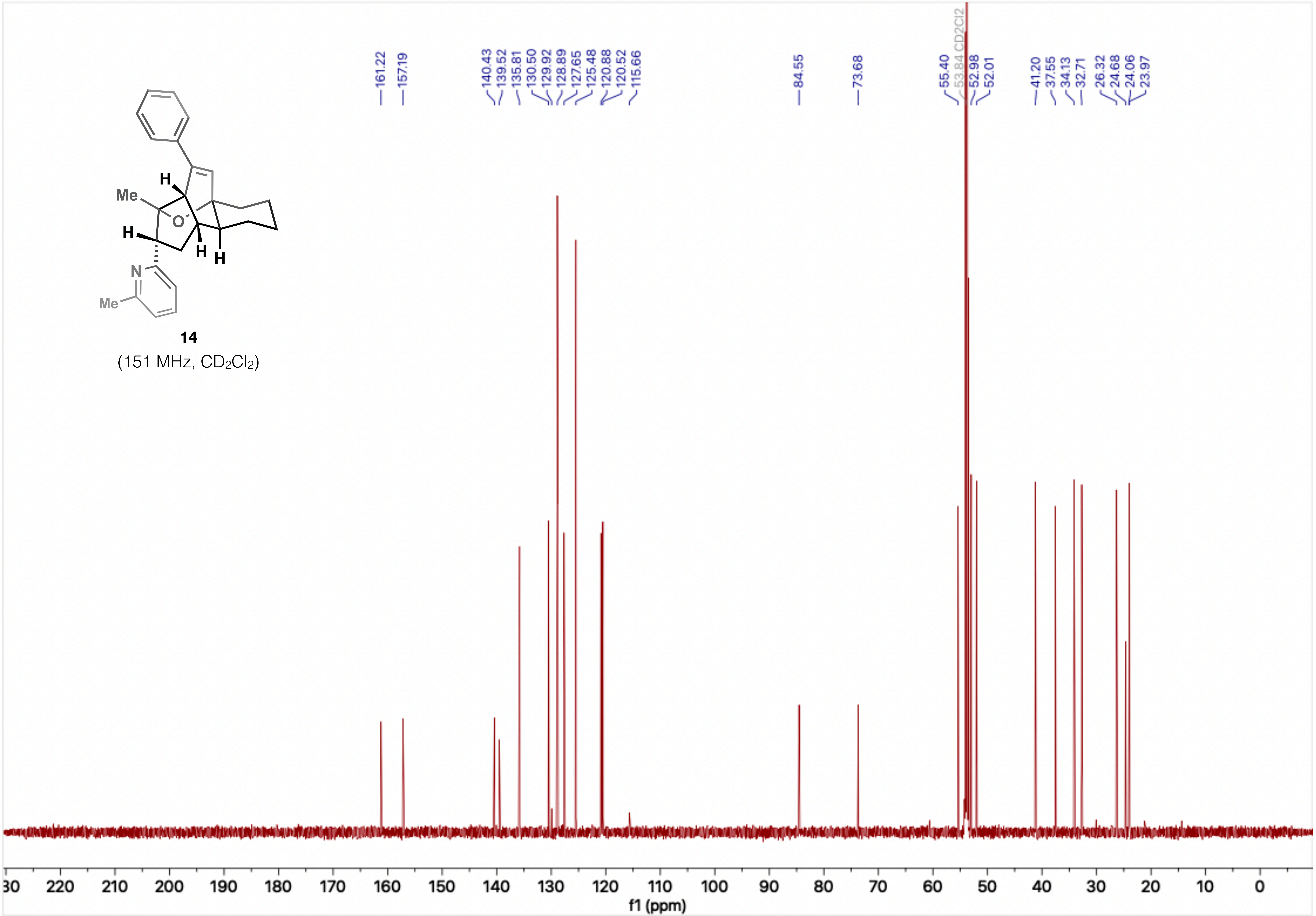

14

(151 MHz, CD_2Cl_2)

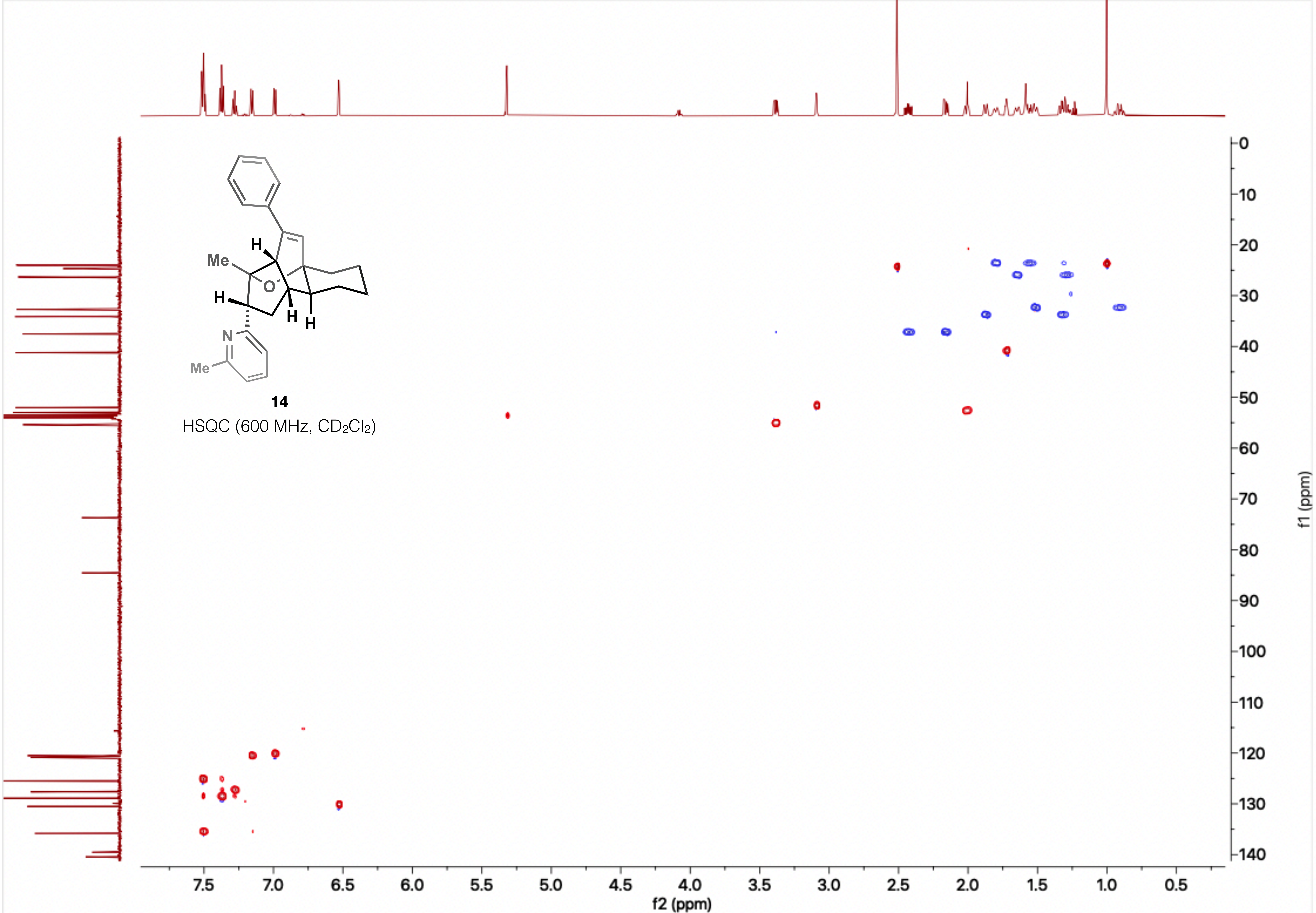

14

HSQC (600 MHz, CD_2Cl_2)

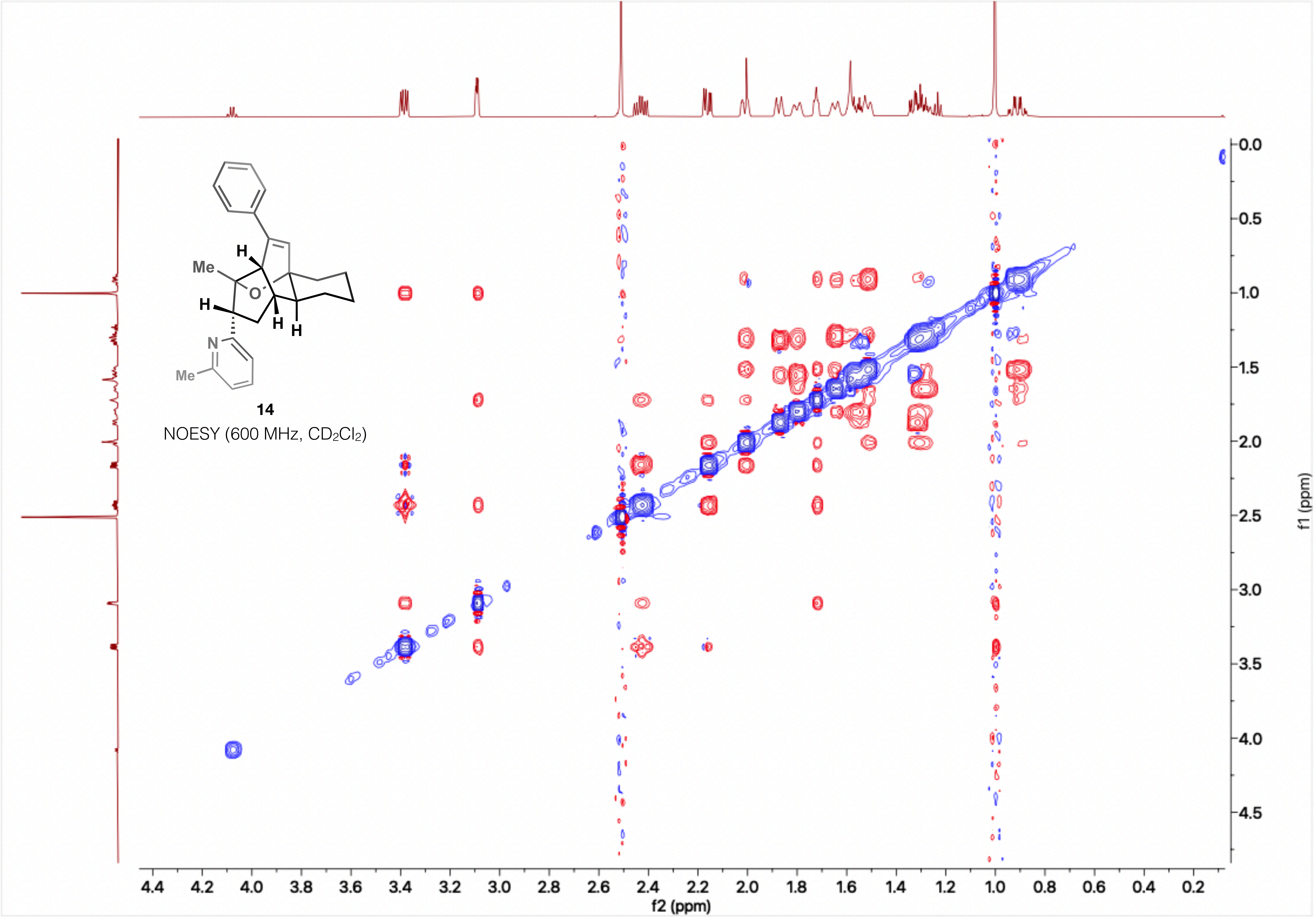

Me
H
O
H
H
H
N
Me
14
NOESY (600 MHz, CD2Cl2)
f2 (ppm)
f1 (ppm)

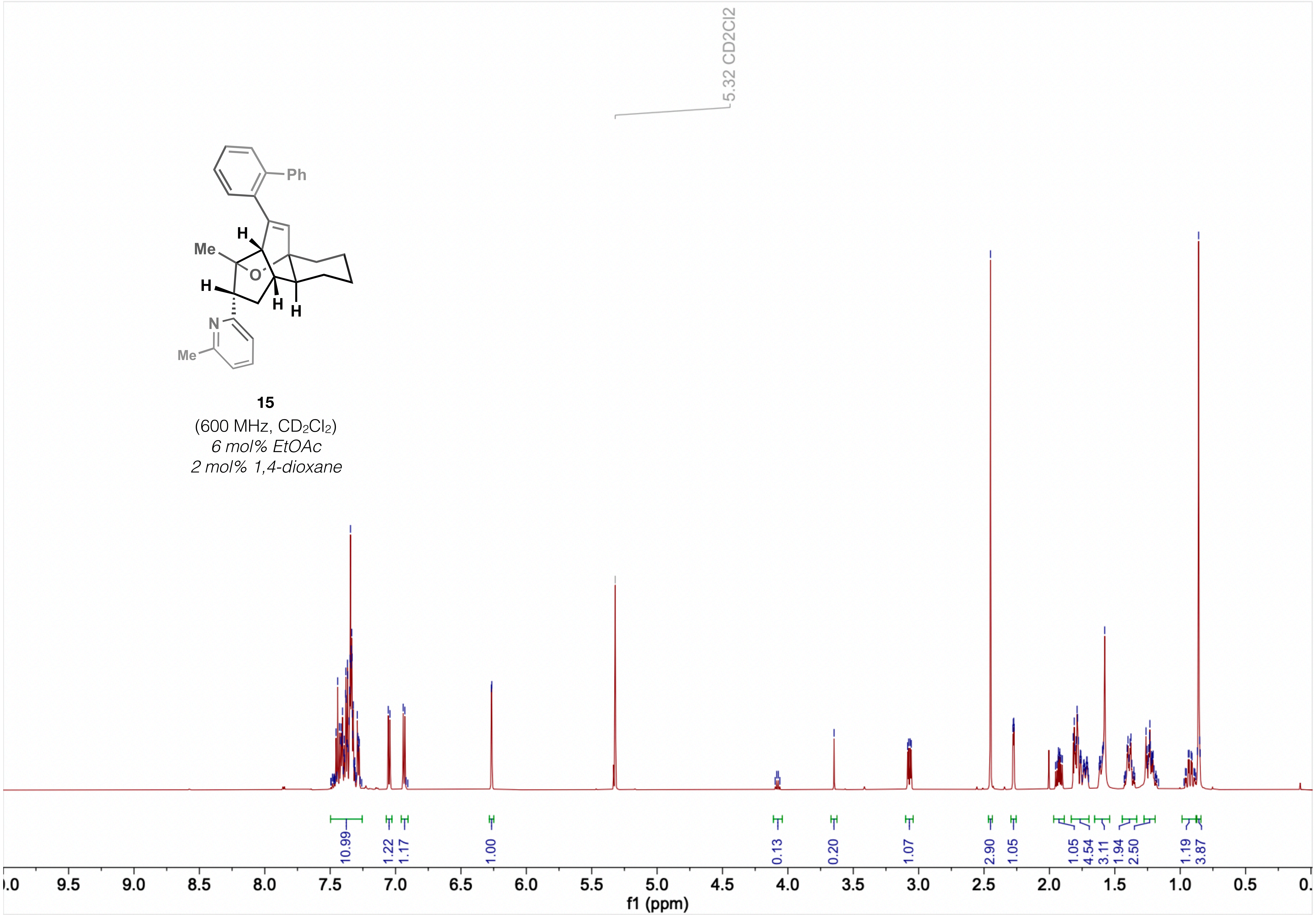
5.32 CD2Cl2
Ph
Me
H
O
N
15
(600 MHz, CD2Cl2)
6 mol% EtOAc
2 mol% 1,4-dioxane
10.99
1.22
1.17
1.00
0.13
0.20
1.07
2.90
1.05
1.05
4.54
3.11
1.94
2.50
1.19
3.87
9.5
9.0
8.5
8.0
7.5
7.0
6.5
6.0
5.5
5.0
4.5
4.0
3.5
3.0
2.5
2.0
1.5
1.0
0.5
f1 (ppm)

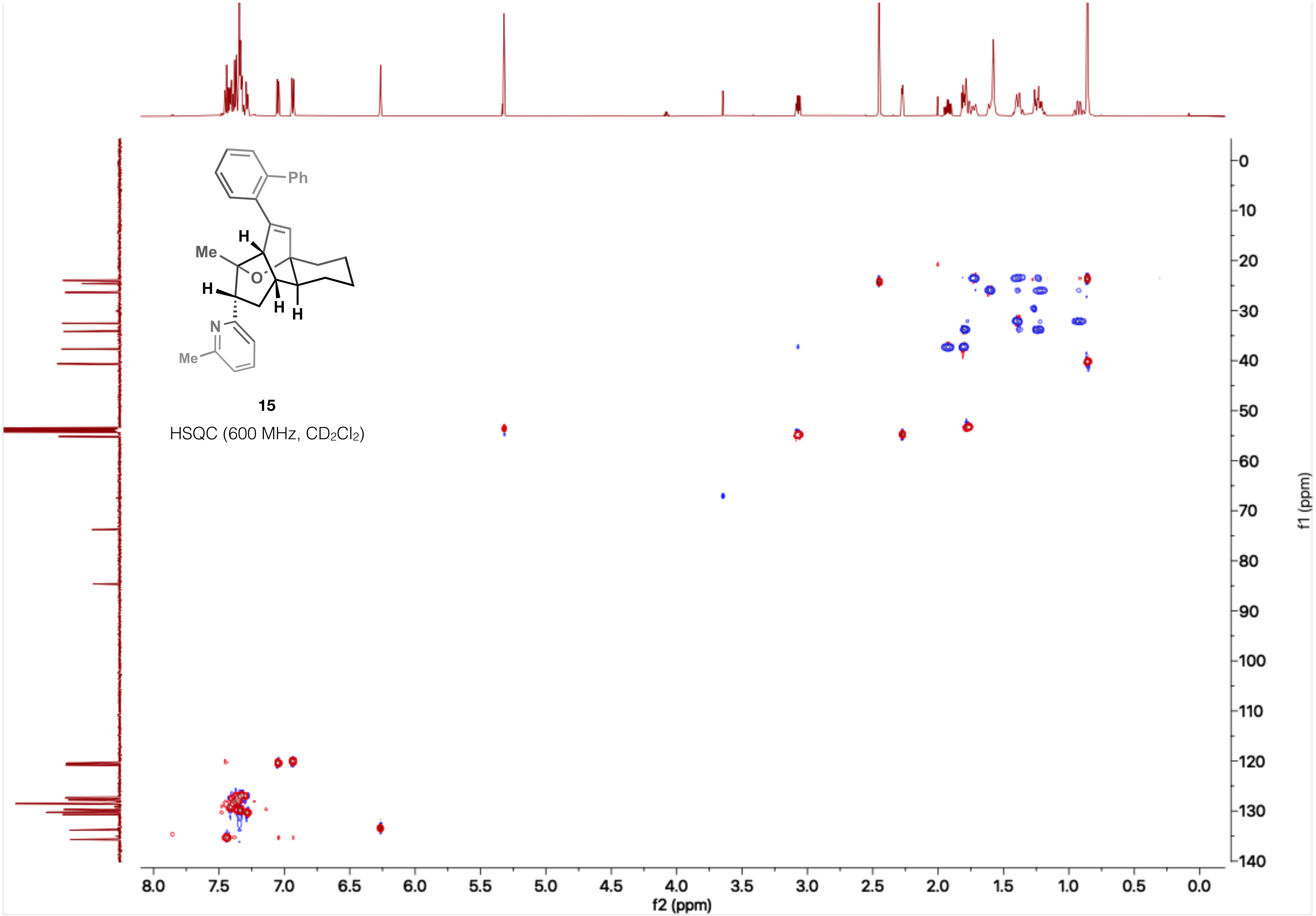
Ph
Me
H
O
H
H
H
N
Me
15
HSQC (600 MHz, CD2Cl2)
f2 (ppm)
f1 (ppm)
8.0
7.5
7.0
6.5
6.0
5.5
5.0
4.5
4.0
3.5
3.0
2.5
2.0
1.5
1.0
0.5
0.0
0
10
20
30
40
50
60
70
80
90
100
110
120
130
140

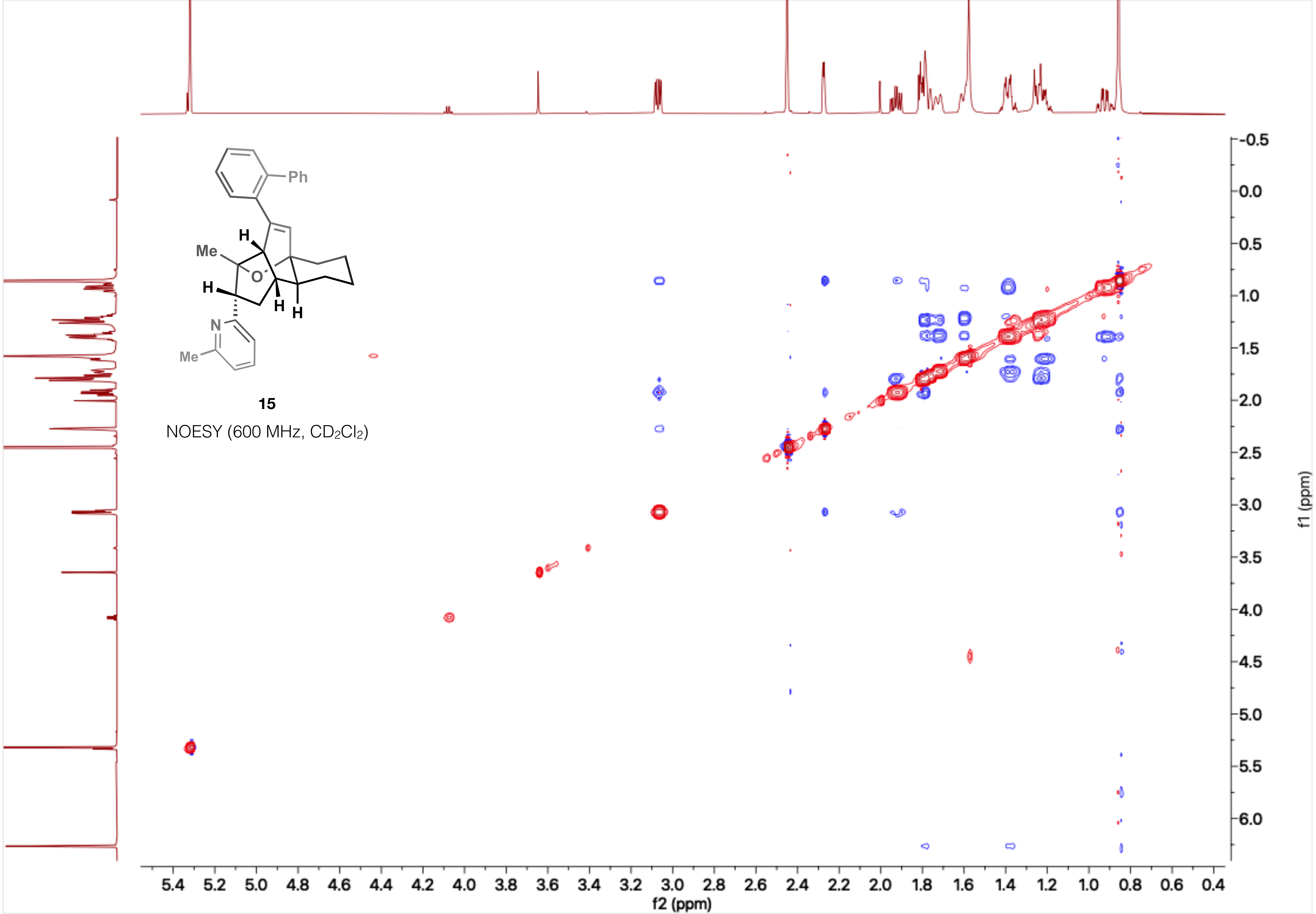
Ph
Me
H
O
H
H
H
N
Me
15
NOESY (600 MHz, CD2Cl2)
f2 (ppm)
f1 (ppm)

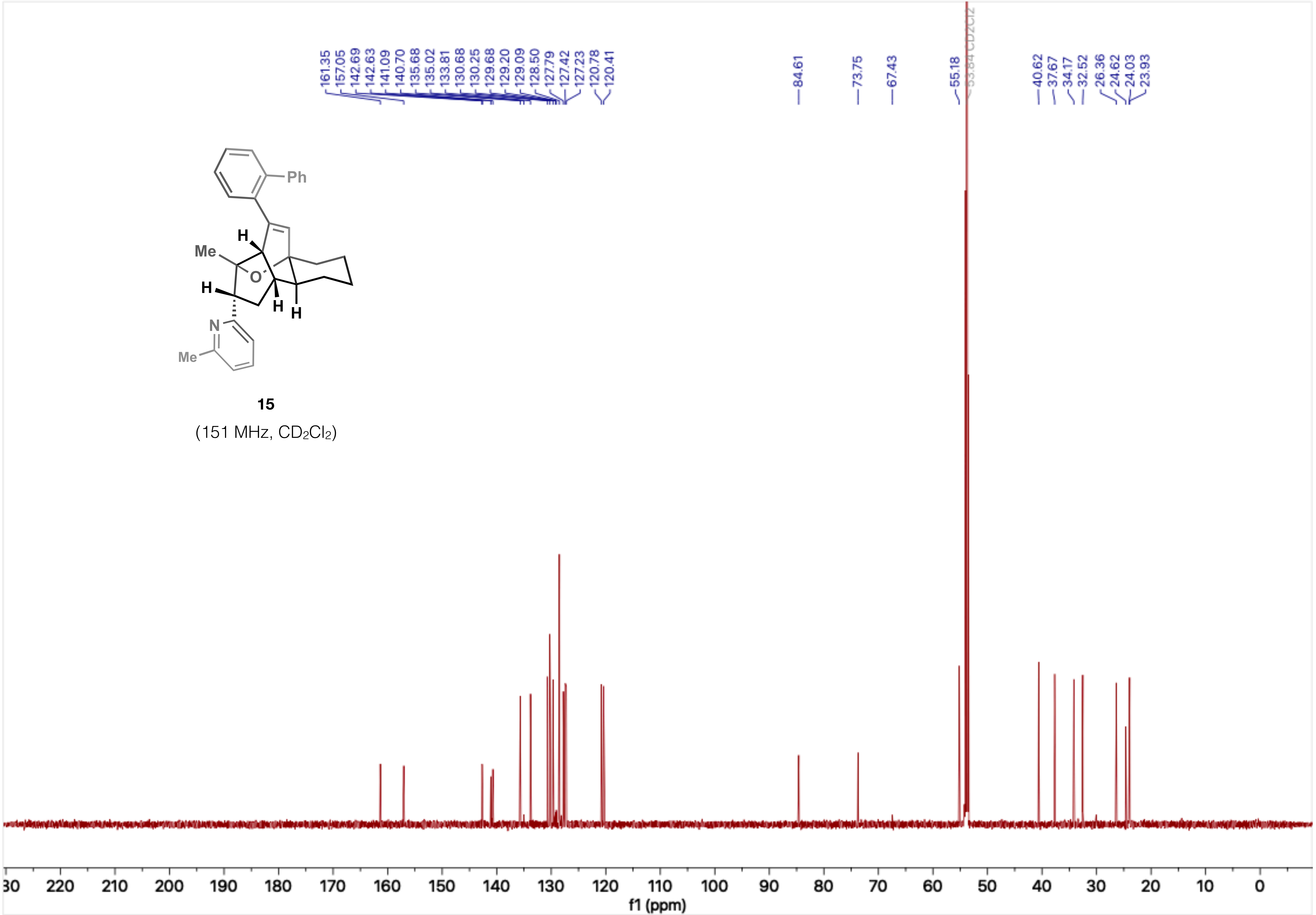
161.35
157.05
142.69
142.63
141.09
140.70
135.68
135.02
133.81
130.68
130.25
129.68
129.20
129.09
128.50
127.79
127.42
127.23
120.78
120.41
84.61
73.75
67.43
55.18
53.84 CD2Cl2
40.62
37.67
34.17
32.52
26.36
24.62
24.03
23.93
Ph
Me
H
O
H
H
H
N
Me
15
(151 MHz, CD2Cl2)
f1 (ppm)

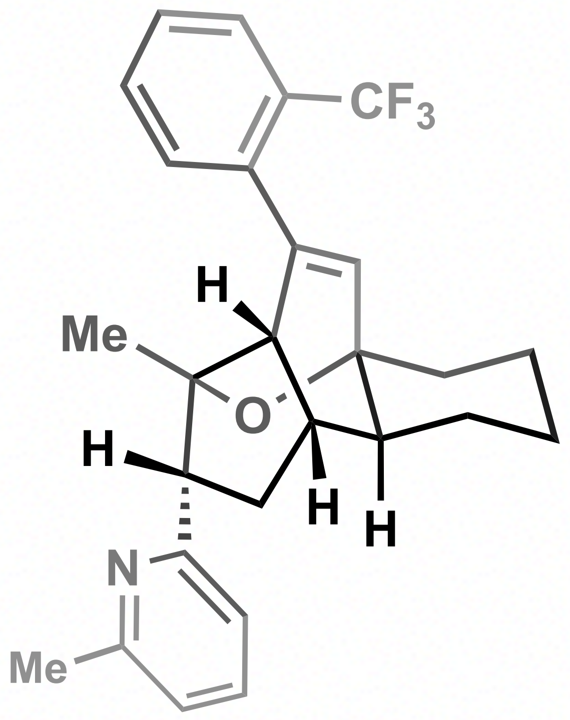

16

(600 MHz, CD_2Cl_2)

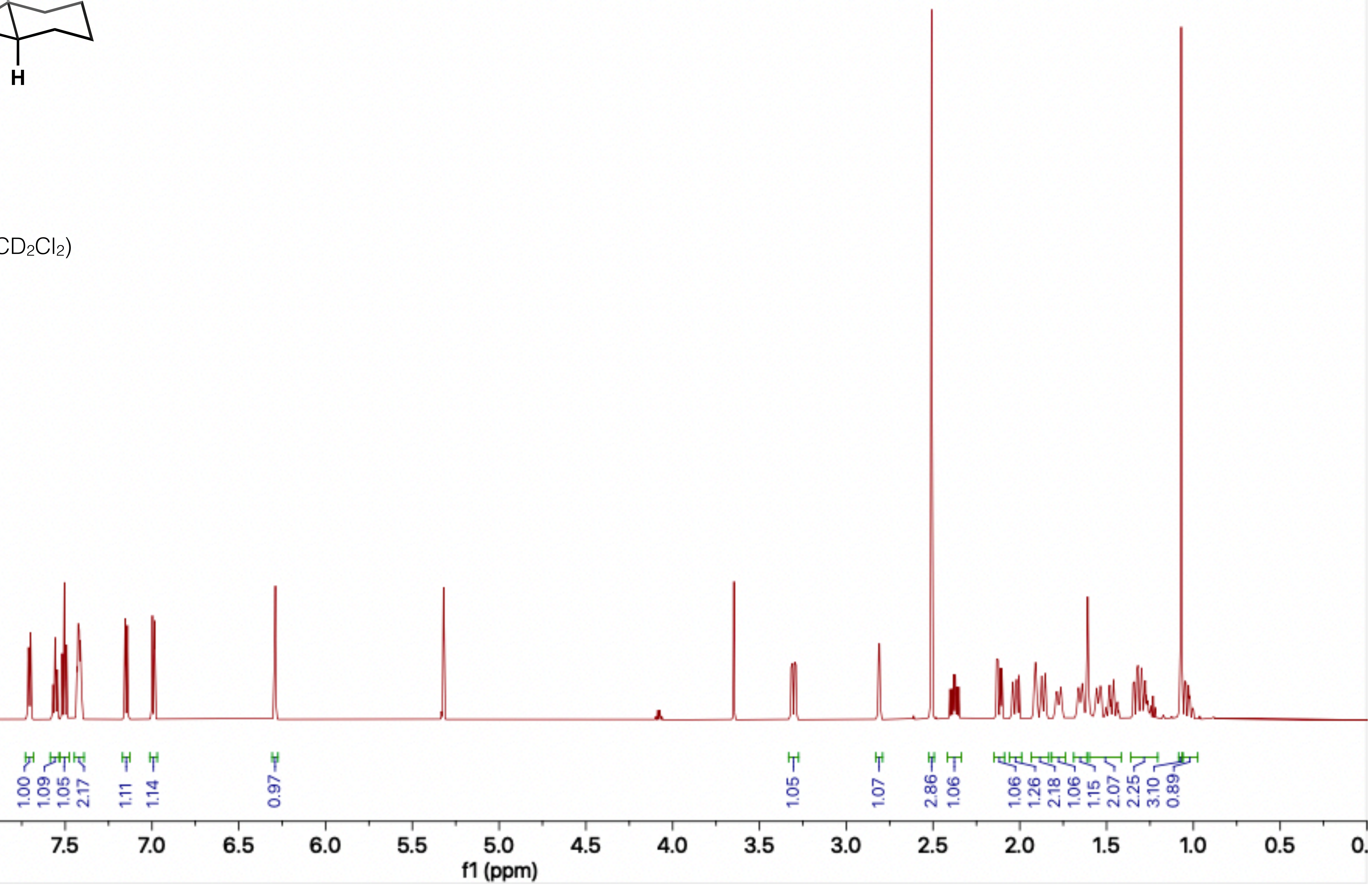

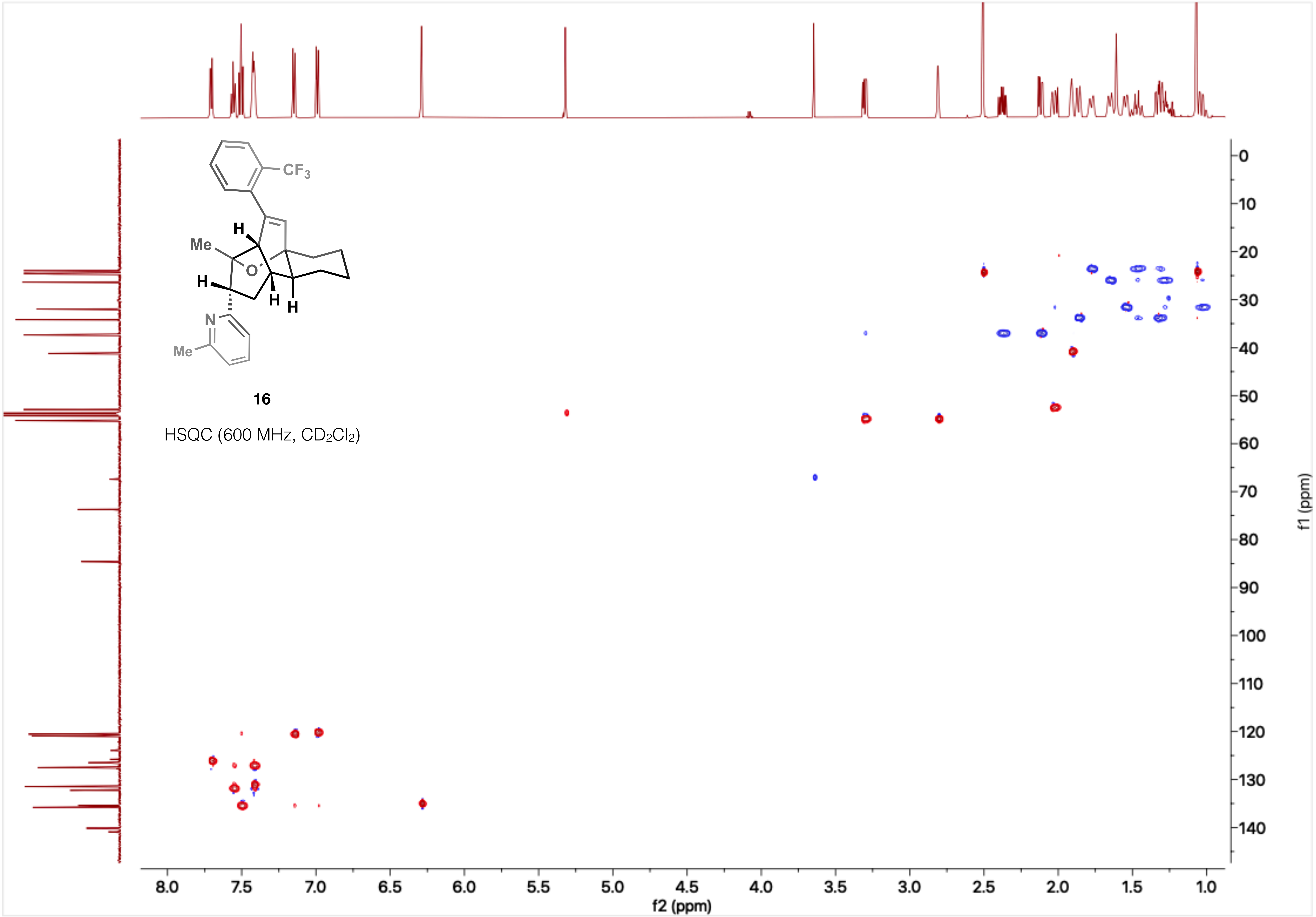

CF3
H
Me
O
H
H H
N
Me
16
HSQC (600 MHz, CD2Cl2)
f1 (ppm)
f2 (ppm)

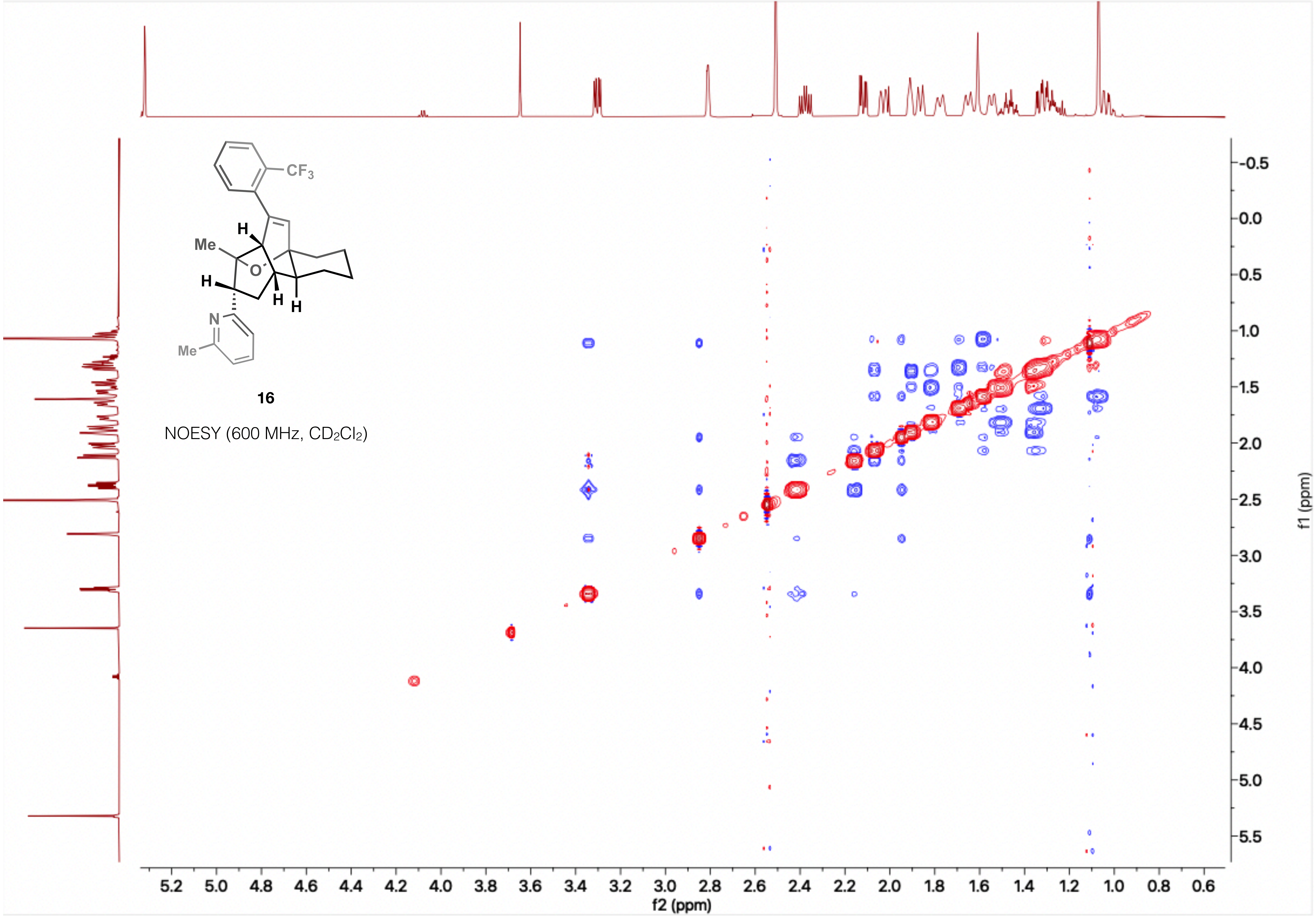
CF3
H
Me
O
H
H
H
N
Me
16
NOESY (600 MHz, CD2Cl2)
f2 (ppm)
f1 (ppm)

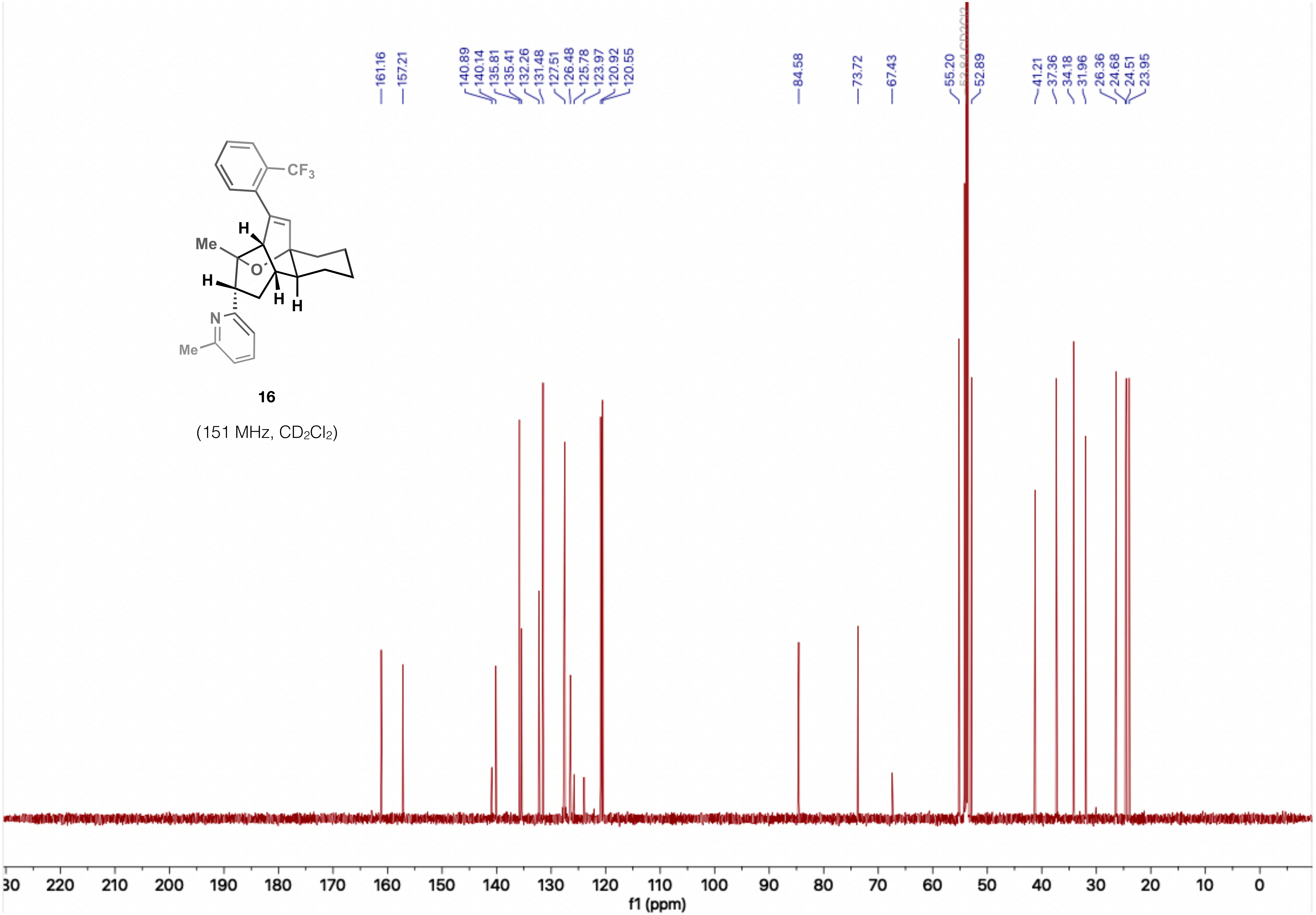

16

(151 MHz, CD_2Cl_2)

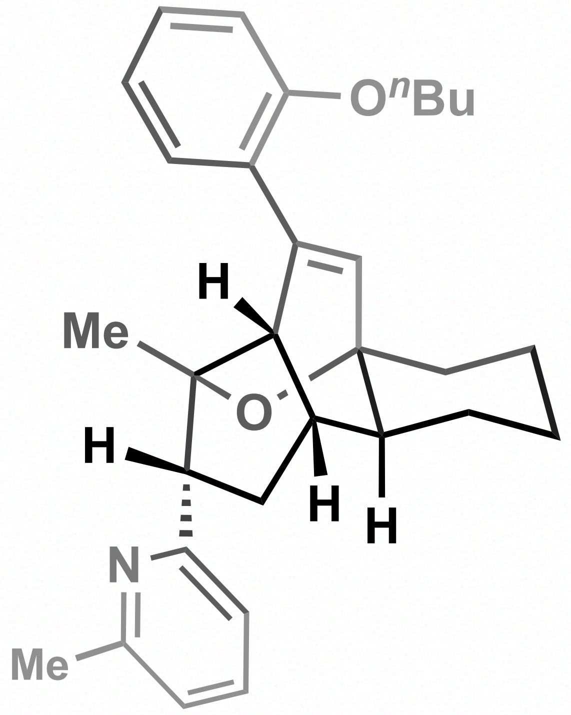

17

(600 MHz, CD_2Cl_2)

3 mol% boronic acid-derived impurities

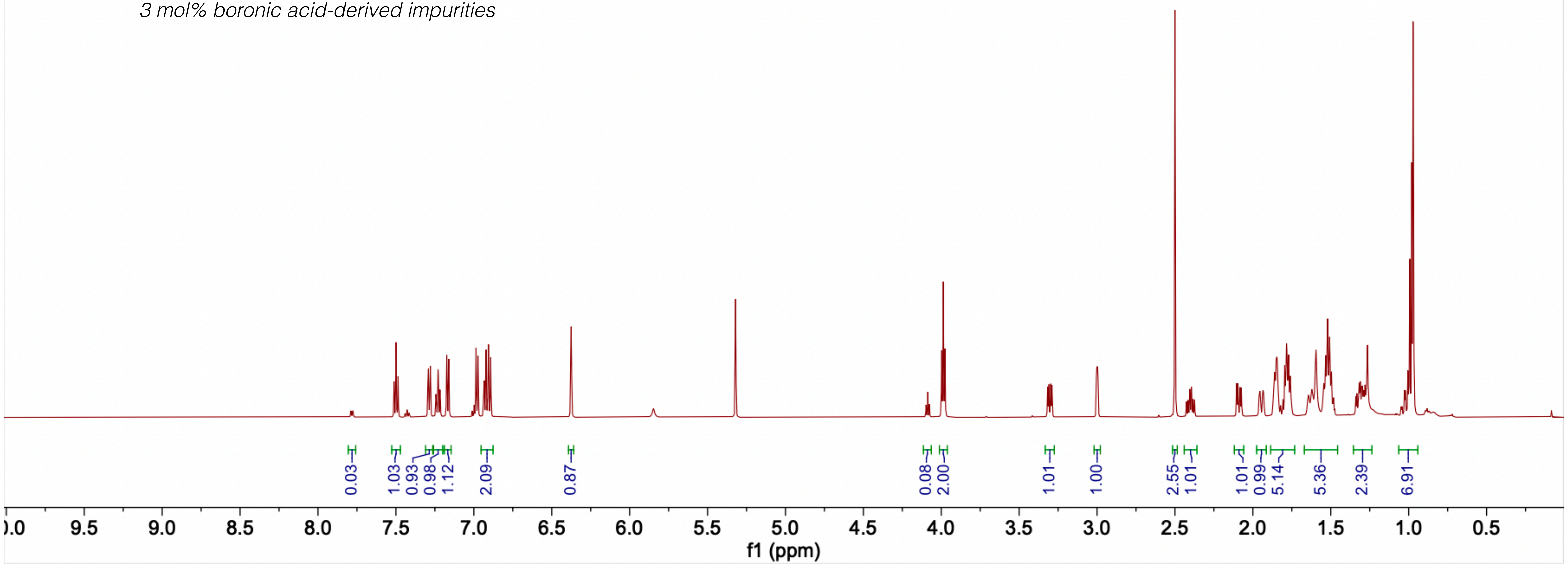

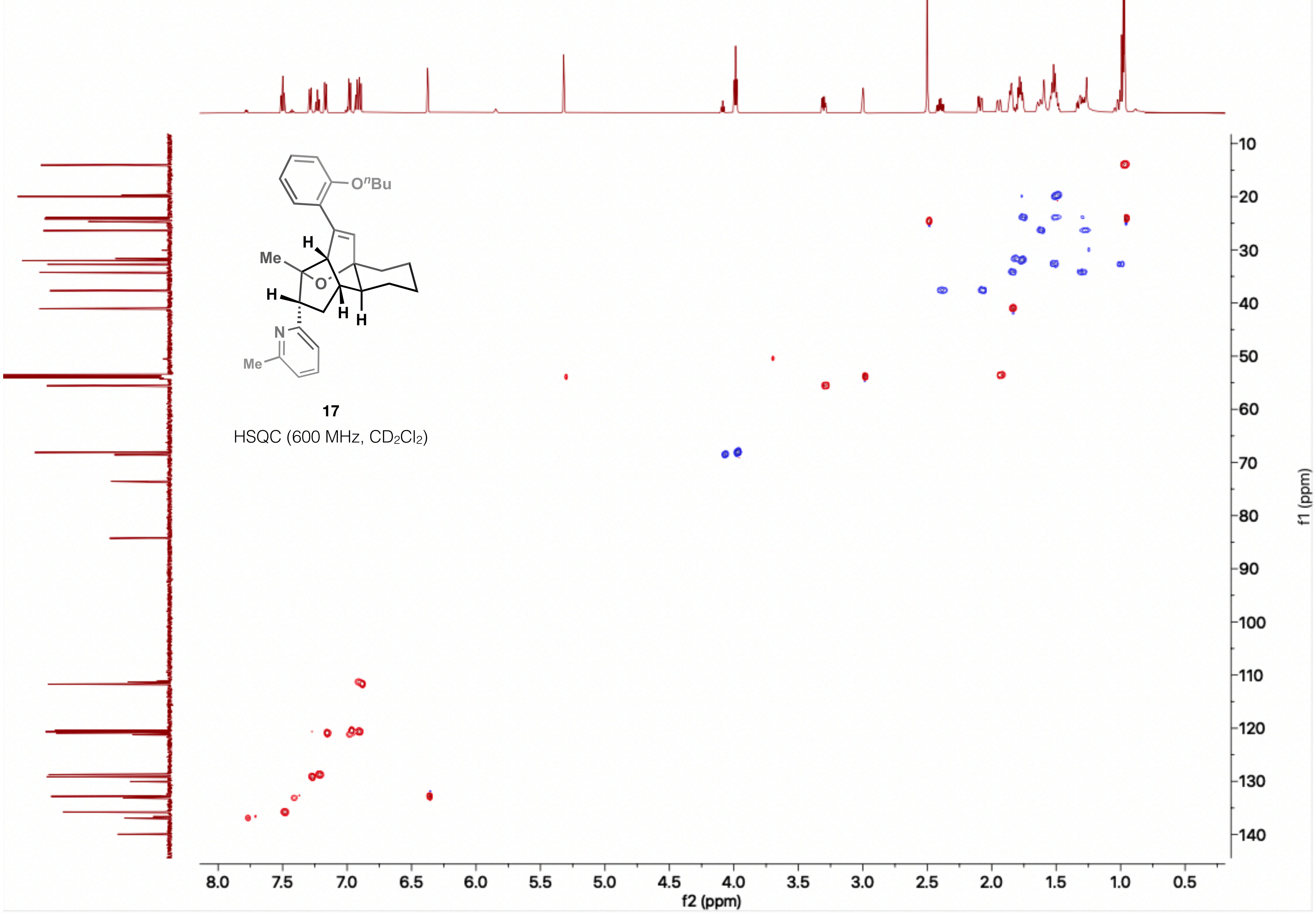

O^{n}Bu
Me
H
O
H
H
H
N
Me
17
HSQC (600 MHz, CD_2Cl_2)
f1 (ppm)
f2 (ppm)

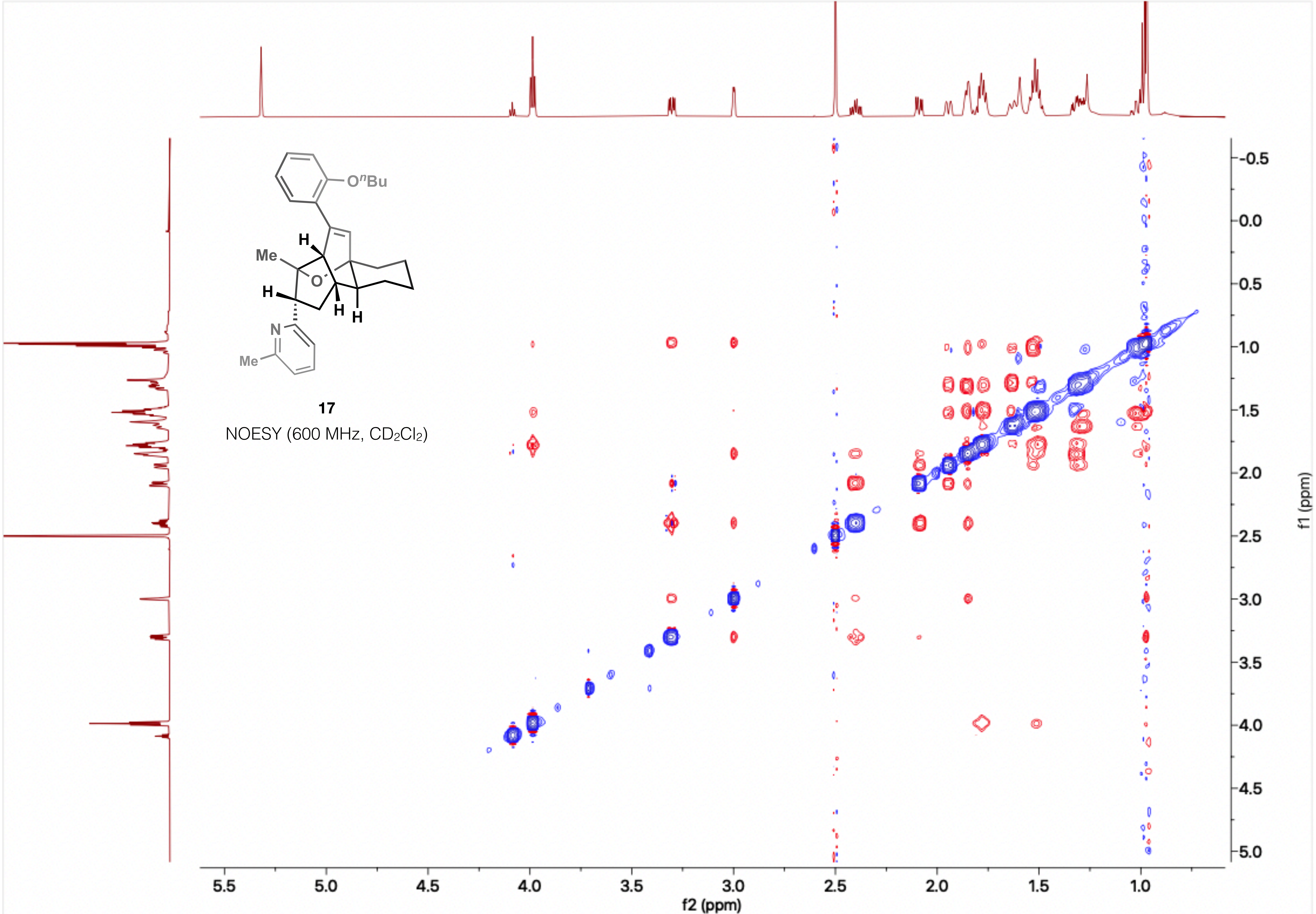

O^{n}Bu
Me
H
O
H
H
H
N
Me
17
NOESY (600 MHz, CD_2Cl_2)
f2 (ppm)
f1 (ppm)

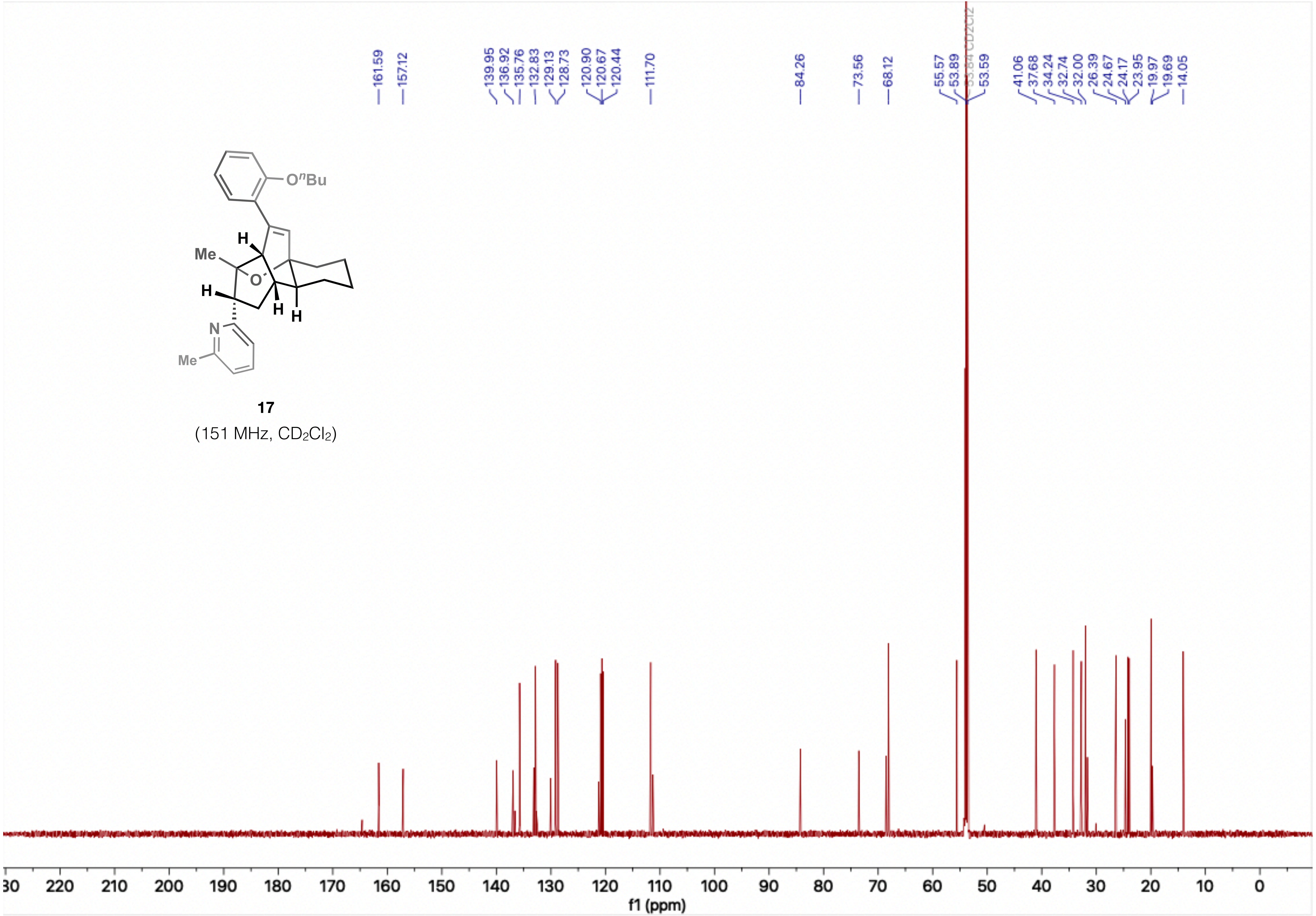

17

(151 MHz, CD_2Cl_2)

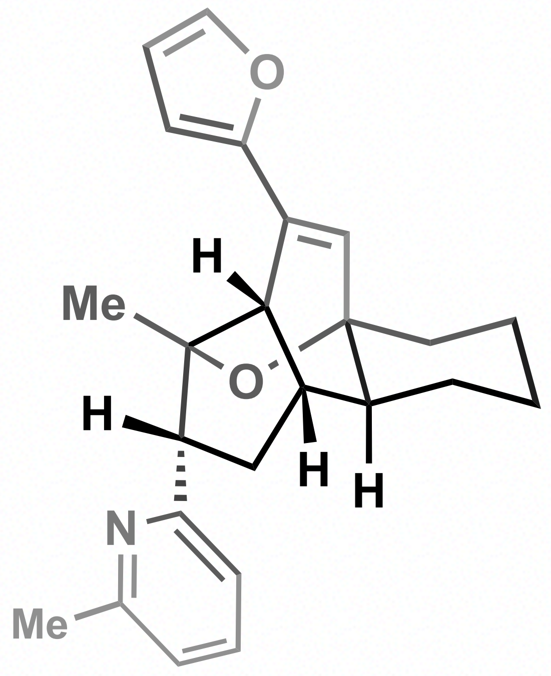

18

(600 MHz, CD_2Cl_2)

6 mol% EtOAc

1 mol% 1,4-dioxane

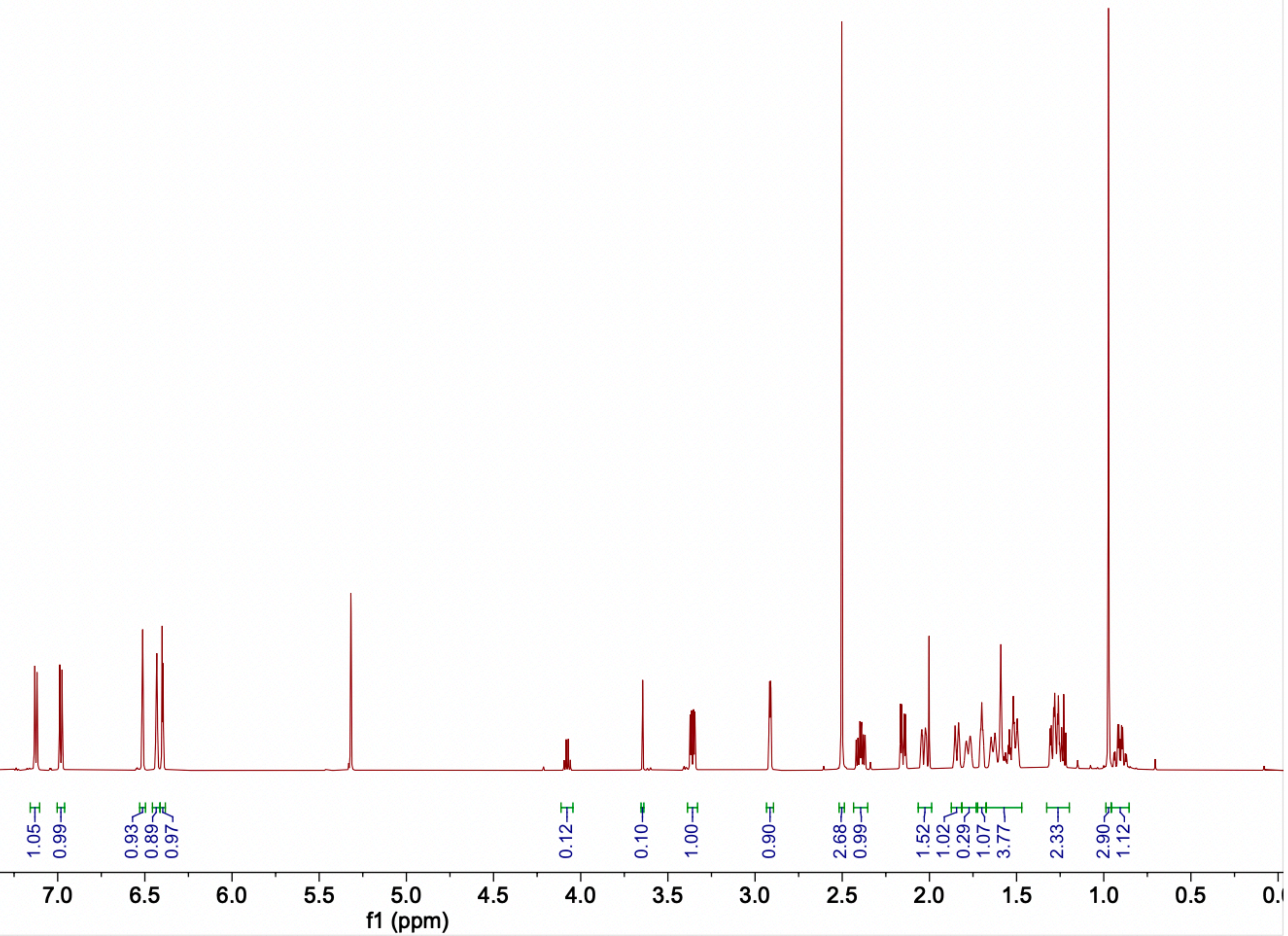

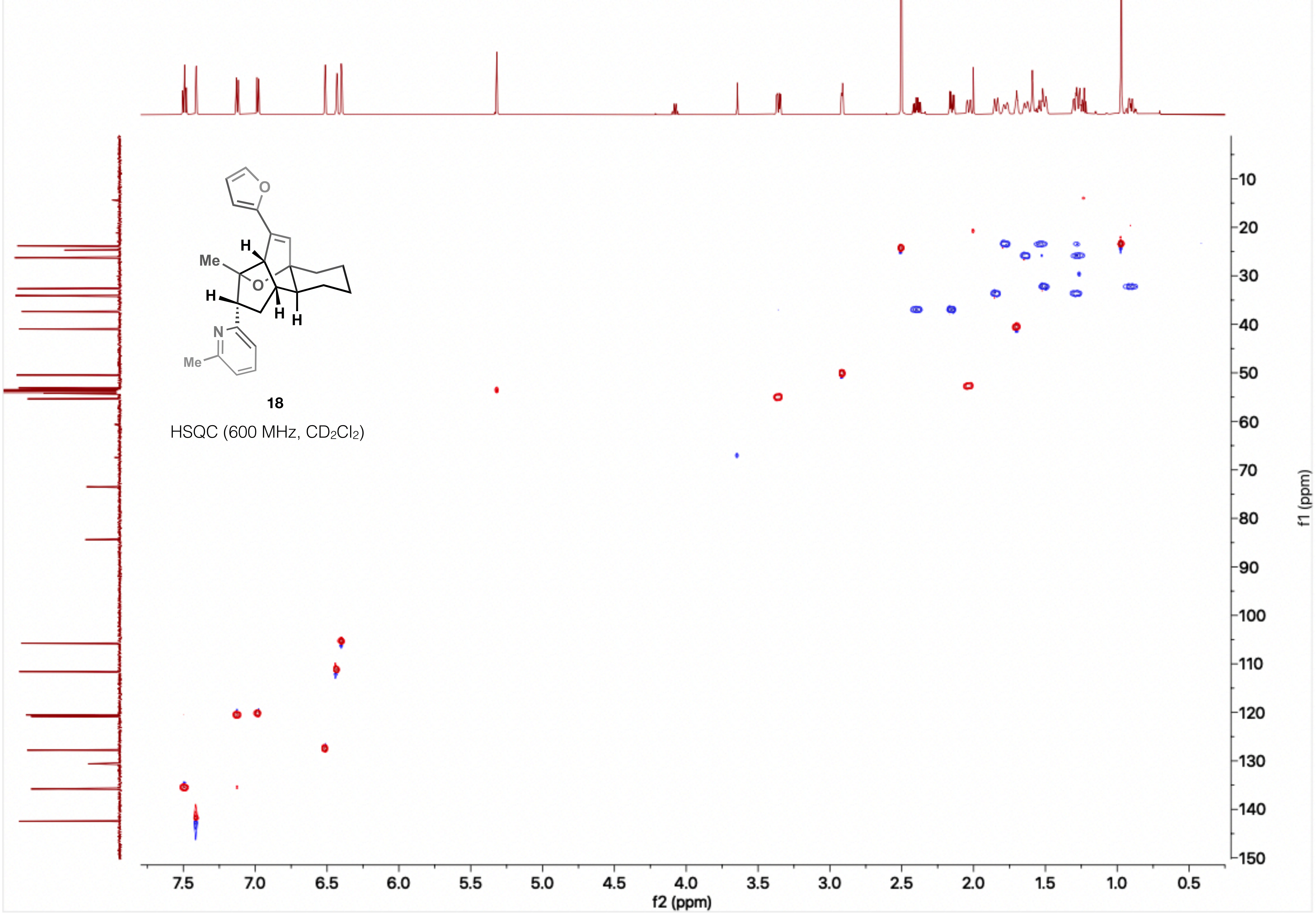
Me
H
O
H
N
Me
H
H
18
HSQC (600 MHz, CD2Cl2)
f2 (ppm)
f1 (ppm)
7.5
7.0
6.5
6.0
5.5
5.0
4.5
4.0
3.5
3.0
2.5
2.0
1.5
1.0
0.5
10
20
30
40
50
60
70
80
90
100
110
120
130
140
150

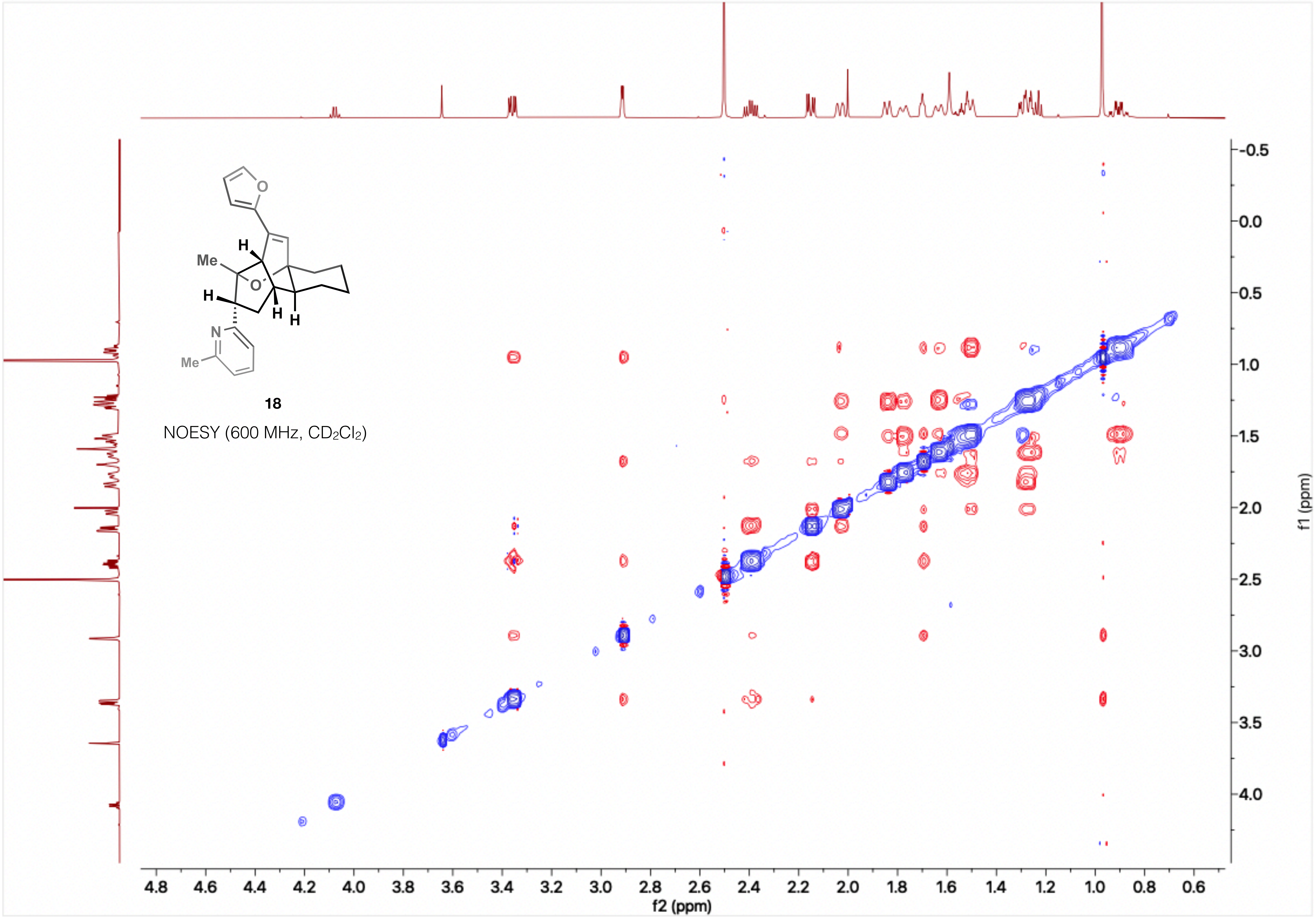
Me
O
H
H
H
H
N
Me
O
18
NOESY (600 MHz, CD₂Cl₂)
f2 (ppm)
f1 (ppm)

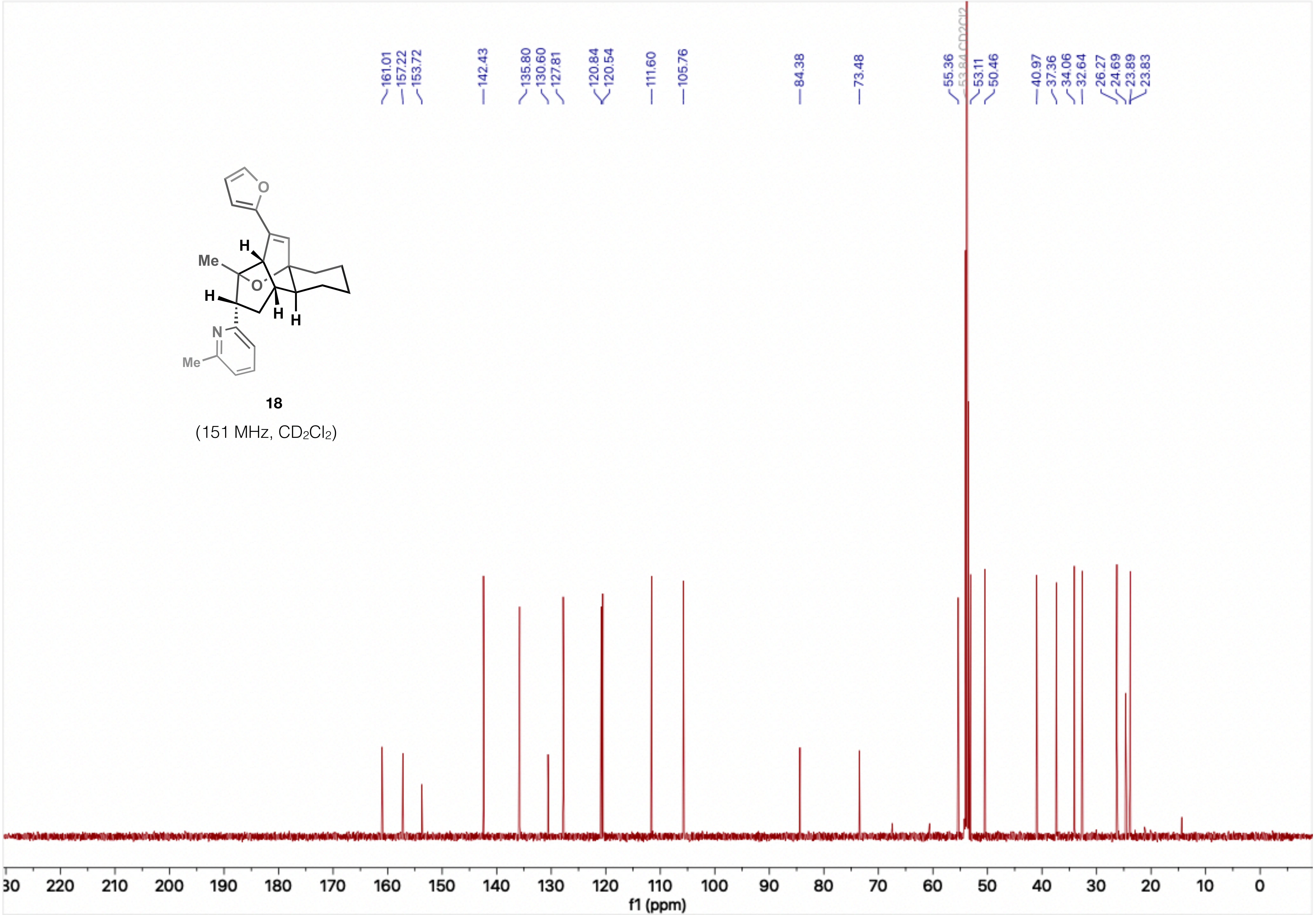
161.01
157.22
153.72
142.43
135.80
130.60
127.81
120.84
120.54
111.60
105.76
84.38
73.48
55.36
53.84 CD2Cl2
53.11
50.46
40.97
37.36
34.06
32.64
26.27
24.69
23.89
23.83
Me
H
O
H
H
H
N
Me
18
(151 MHz, CD_2Cl_2)
f1 (ppm)

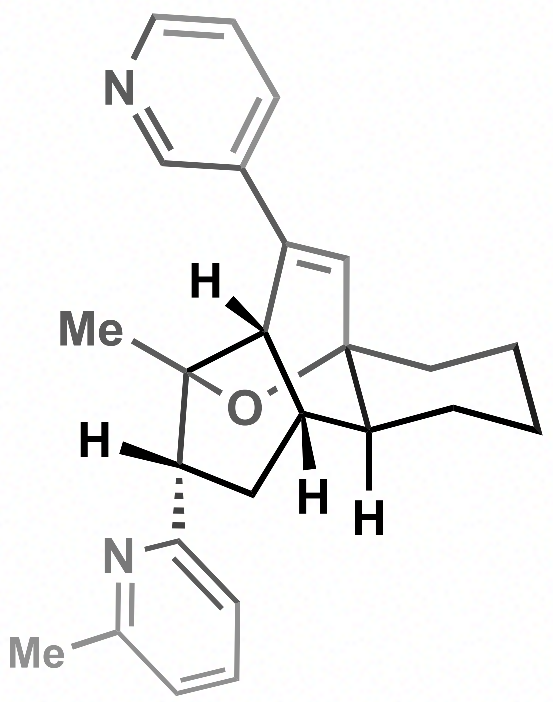

19

(600 MHz, CD_2Cl_2)

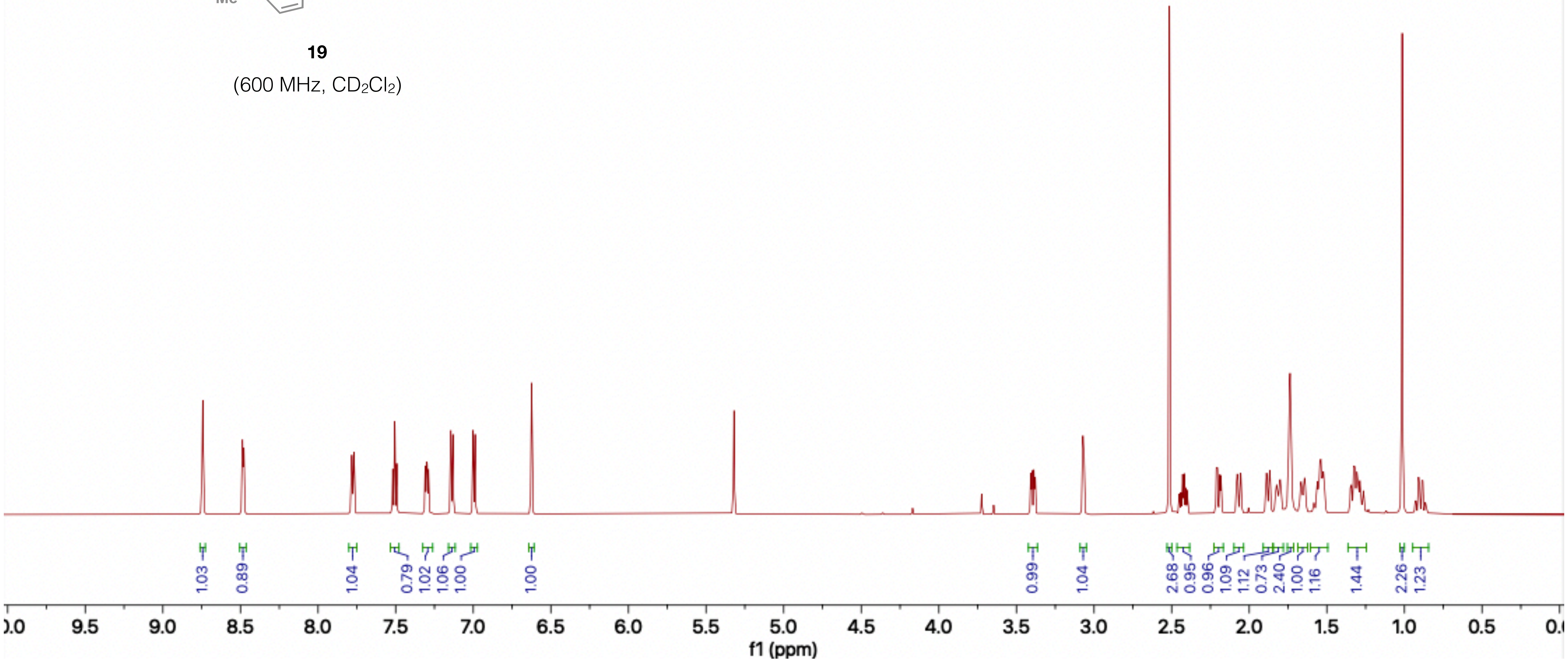

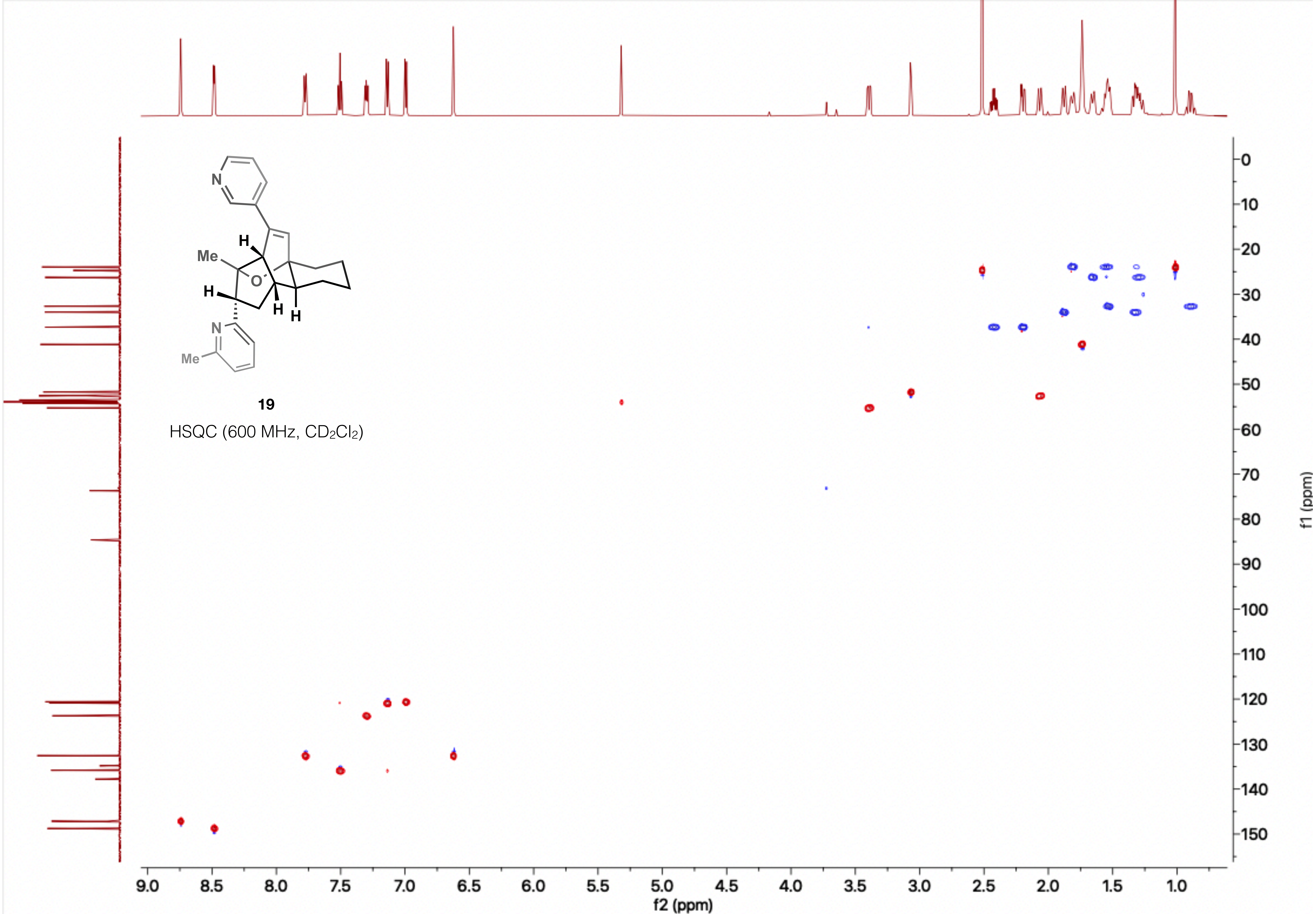
19
HSQC (600 MHz, CD2Cl2)
f1 (ppm)
f2 (ppm)

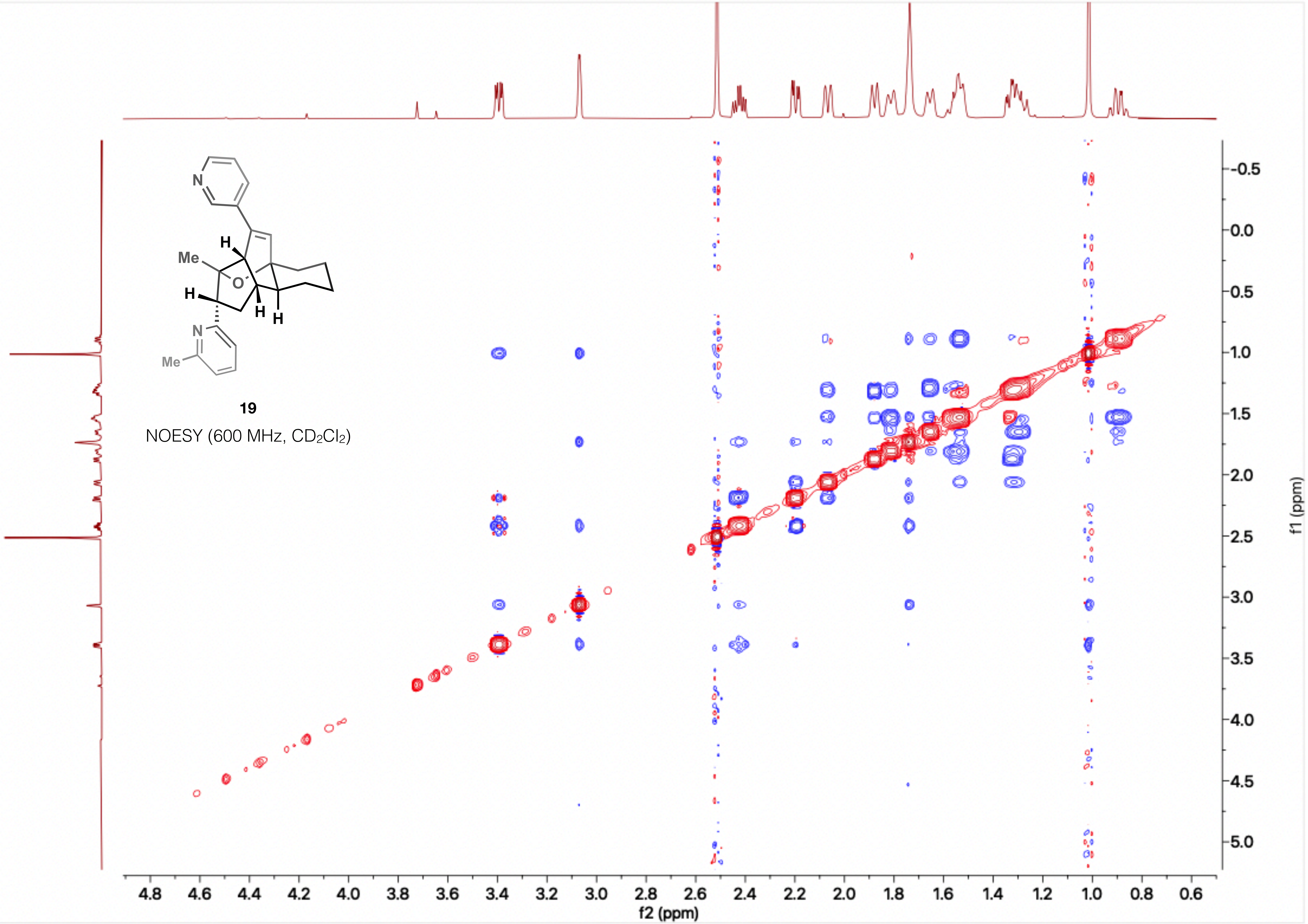

19

NOESY (600 MHz, CD_2Cl_2)

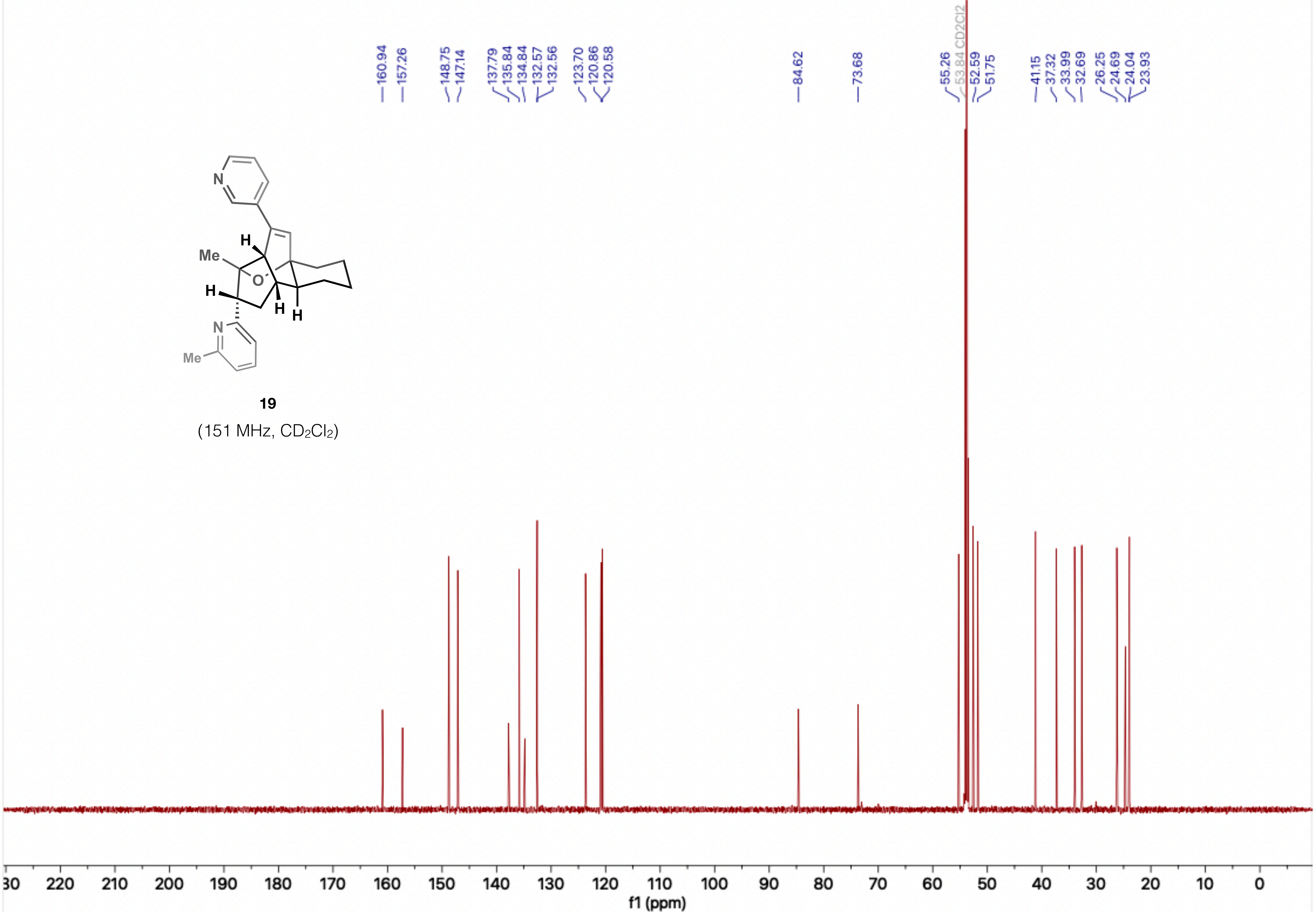

19

(151 MHz, CD_2Cl_2)

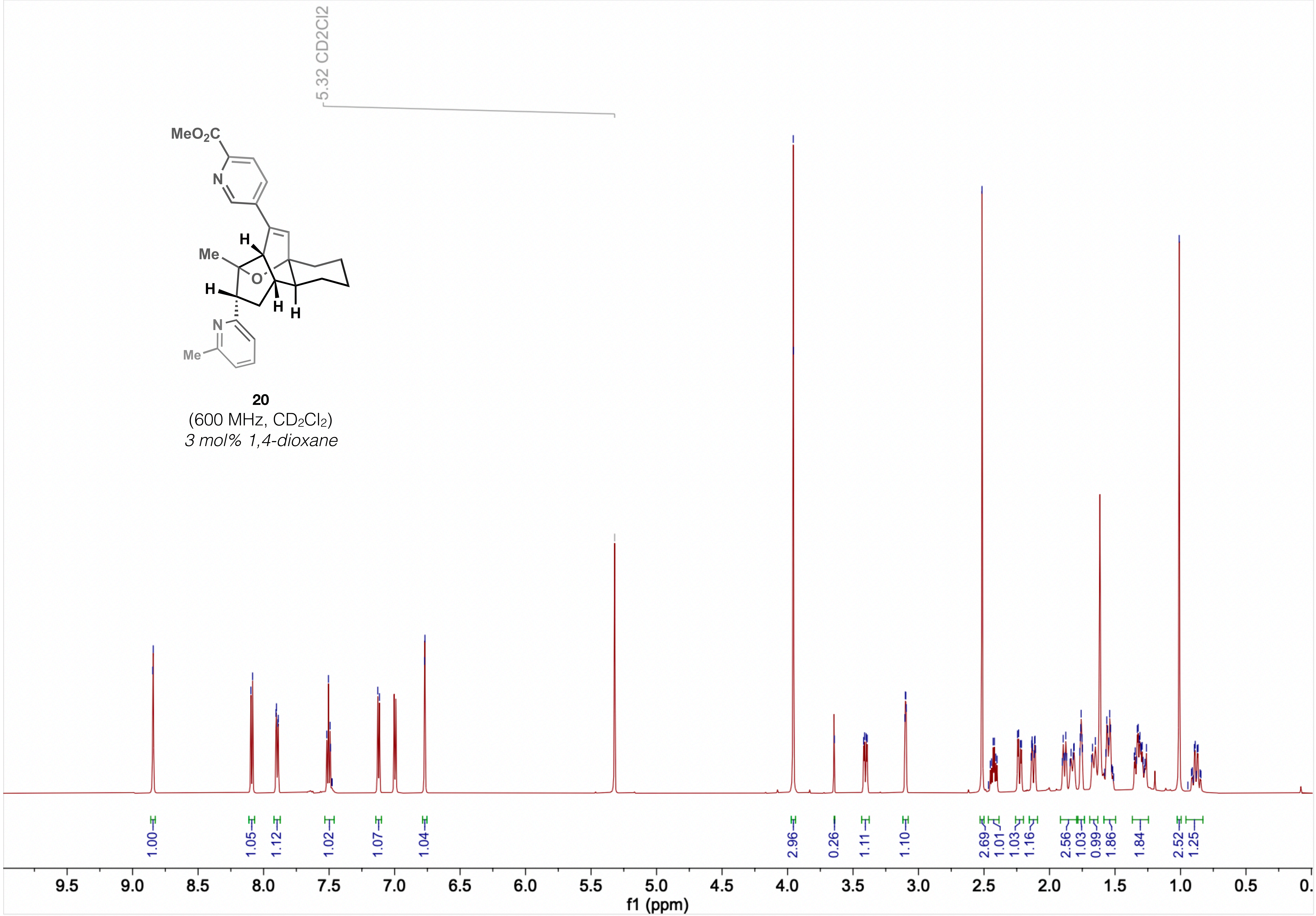

5.32 CD2Cl2
MeO2C
N
H
Me
O
H
H
H
N
Me
20
(600 MHz, CD2Cl2)
3 mol% 1,4-dioxane
1.00
1.05
1.12
1.02
1.07
1.04
2.96
0.26
1.11
1.10
2.69
1.01
1.03
1.16
2.56
1.03
0.99
1.86
1.84
2.52
1.25
9.5 9.0 8.5 8.0 7.5 7.0 6.5 6.0 5.5 5.0 4.5 4.0 3.5 3.0 2.5 2.0 1.5 1.0 0.5 0.
f1 (ppm)

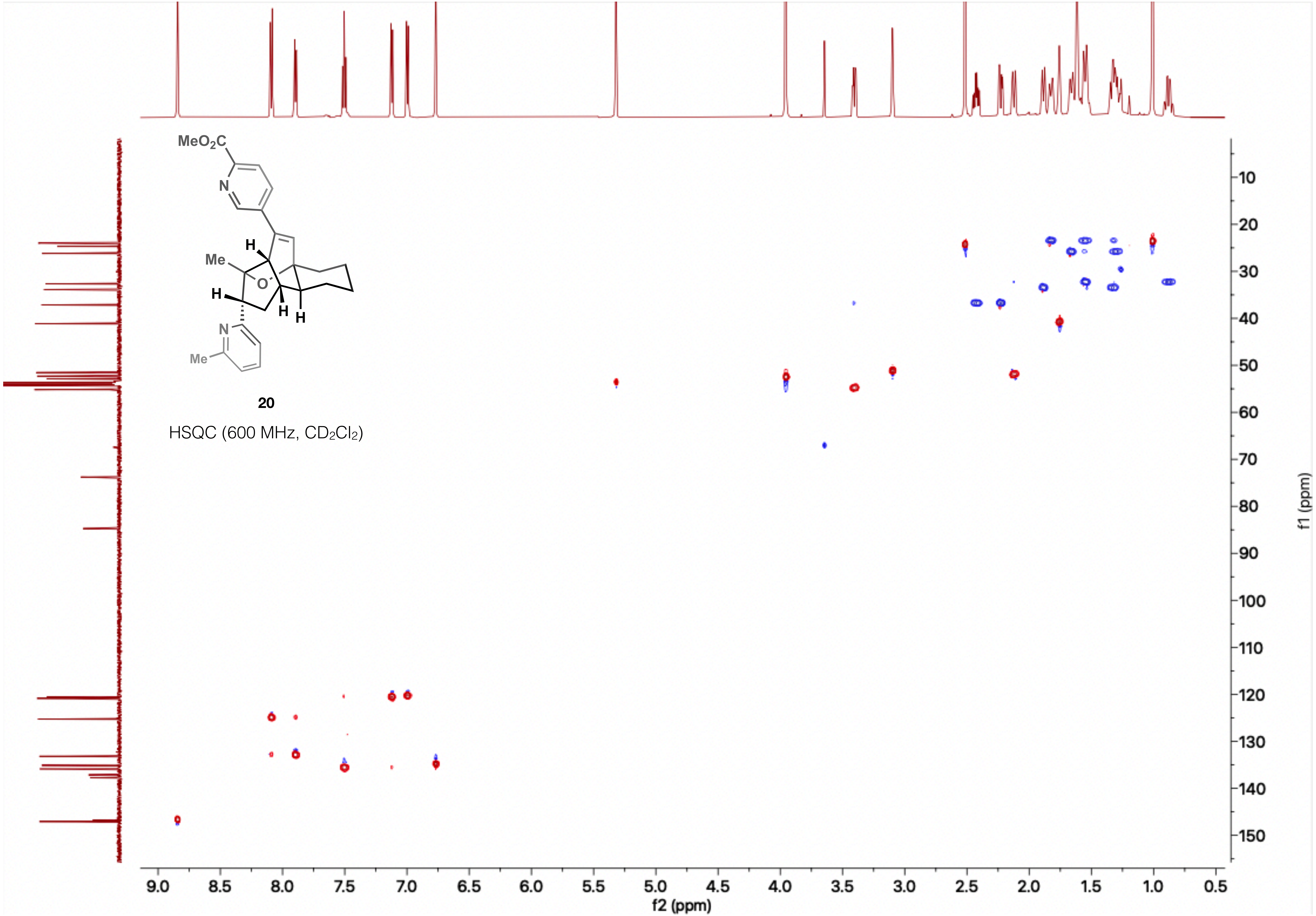
MeO2C
N
H
Me
O
H
H
H
N
Me
20
HSQC (600 MHz, CD2Cl2)
f2 (ppm)
f1 (ppm)

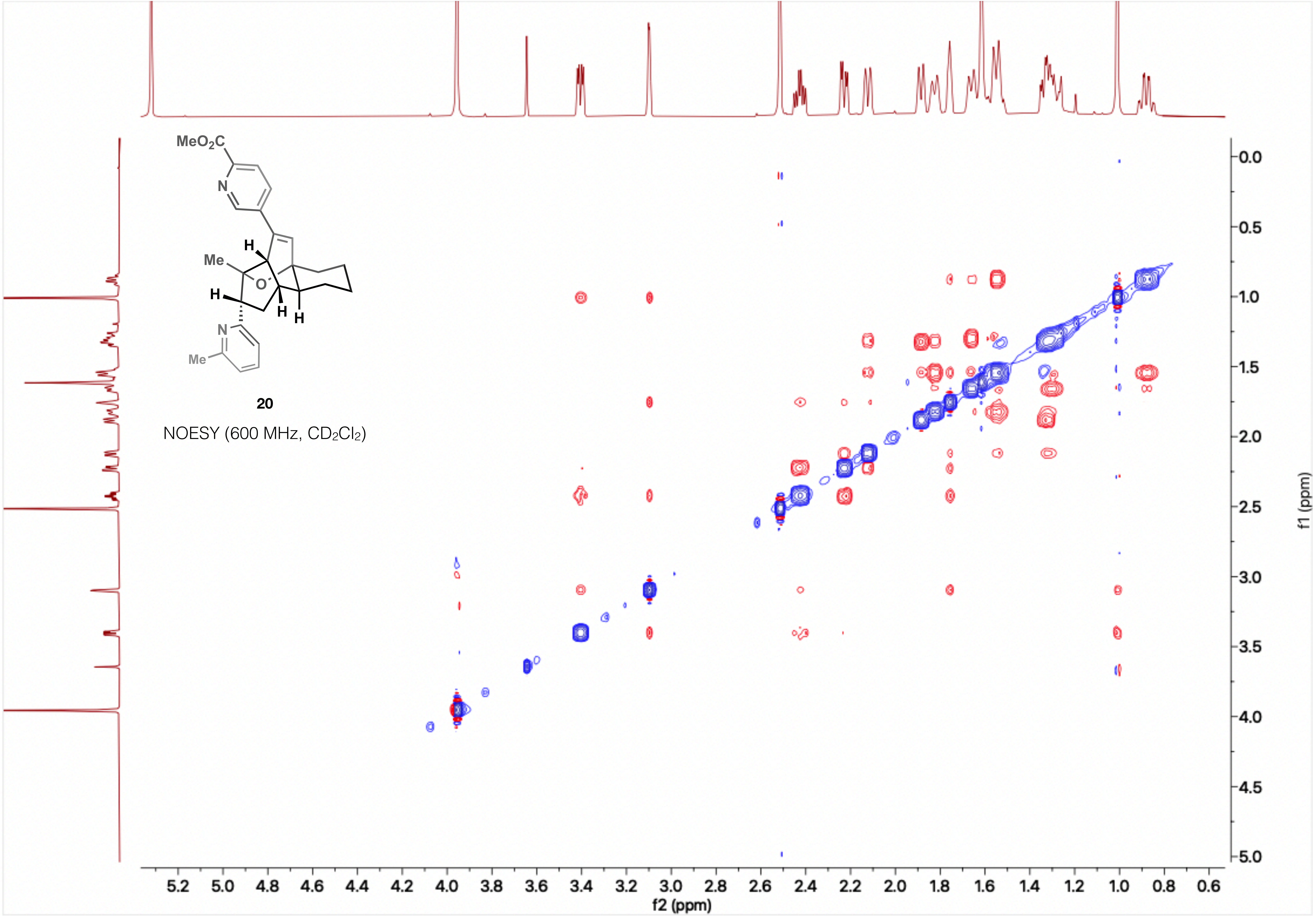
MeO2C
N
Me
H
O
H
H
H
N
Me
20
NOESY (600 MHz, CD2Cl2)
f2 (ppm)
f1 (ppm)

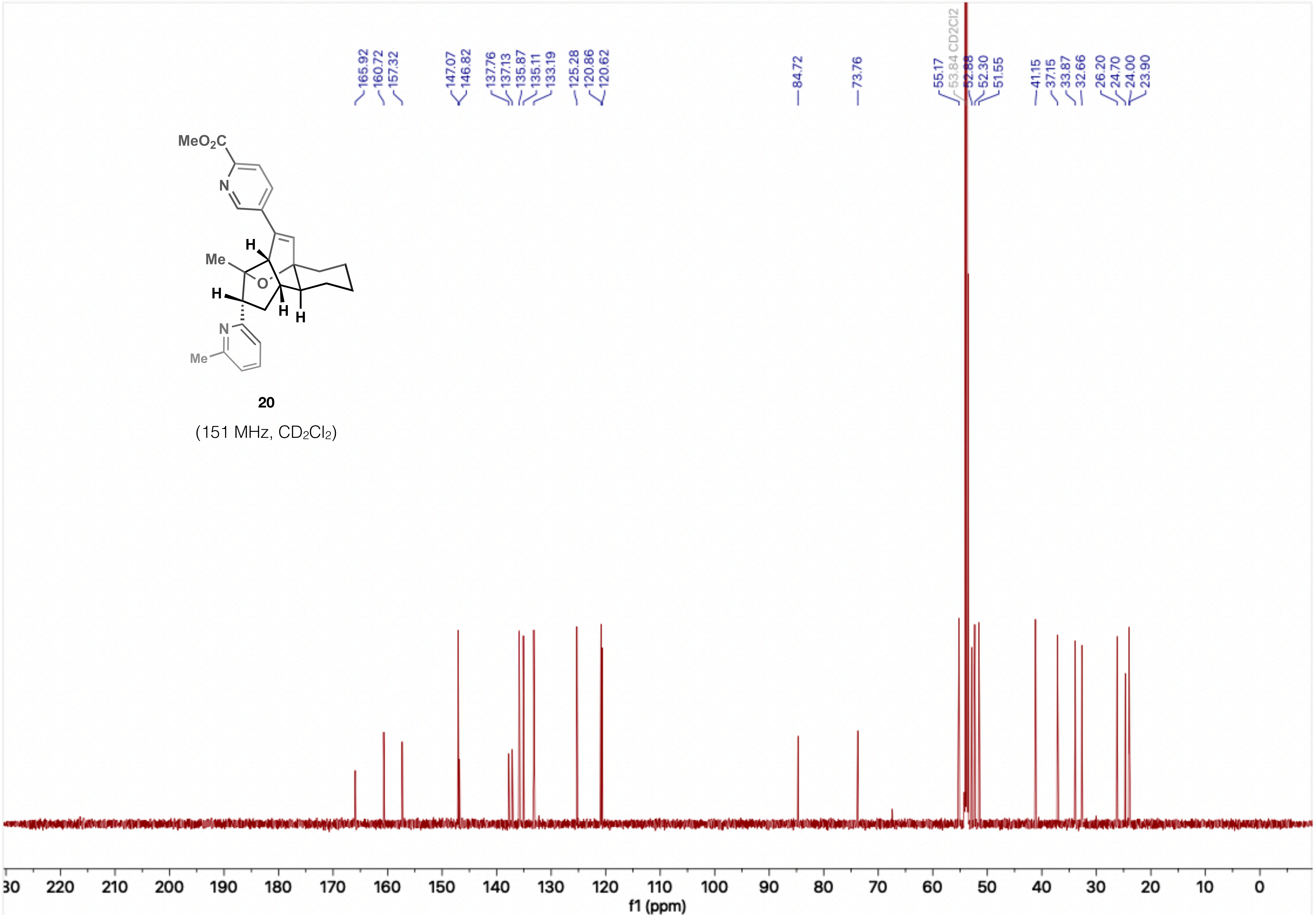
165.92
160.72
157.32
147.07
146.82
137.76
137.13
135.87
135.11
133.19
125.28
120.86
120.62
84.72
73.76
55.17
53.84 CD2Cl2
52.88
52.30
51.55
41.15
37.15
33.87
32.66
26.20
24.70
24.00
23.90
MeO2C
N
H
Me
O
H
H
H
N
Me
20
(151 MHz, CD2Cl2)
30 220 210 200 190 180 170 160 150 140 130 120 110 100 90 80 70 60 50 40 30 20 10 0
f1 (ppm)

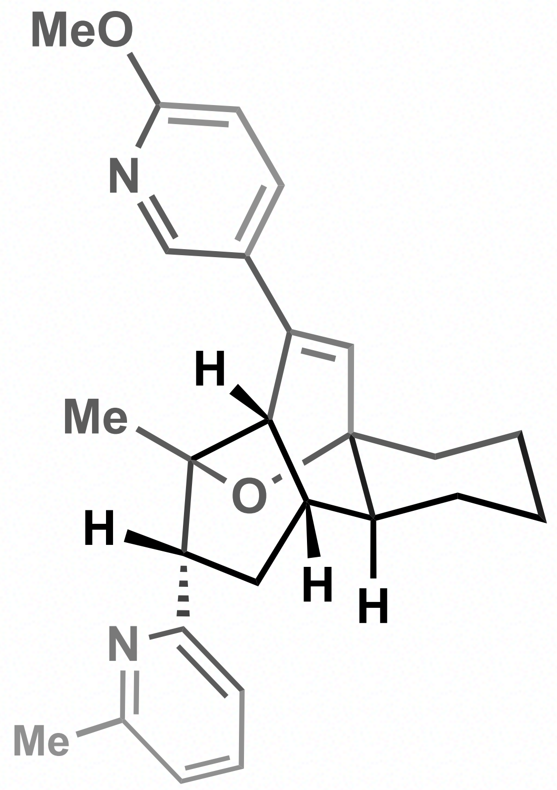

21
(600 MHz, CD_2Cl_2)
4 mol% EtOAc
1 mol% dichloroethane

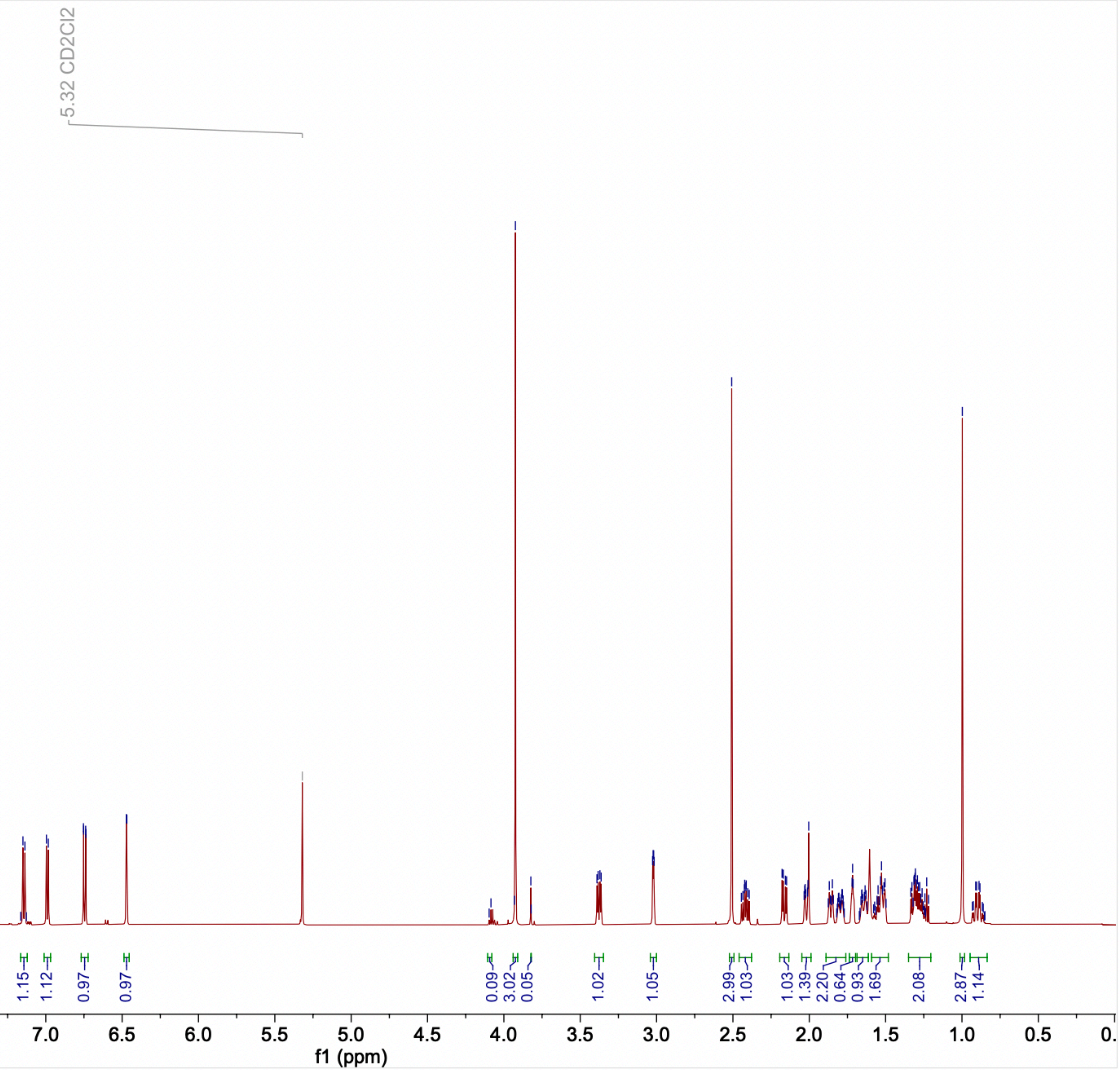

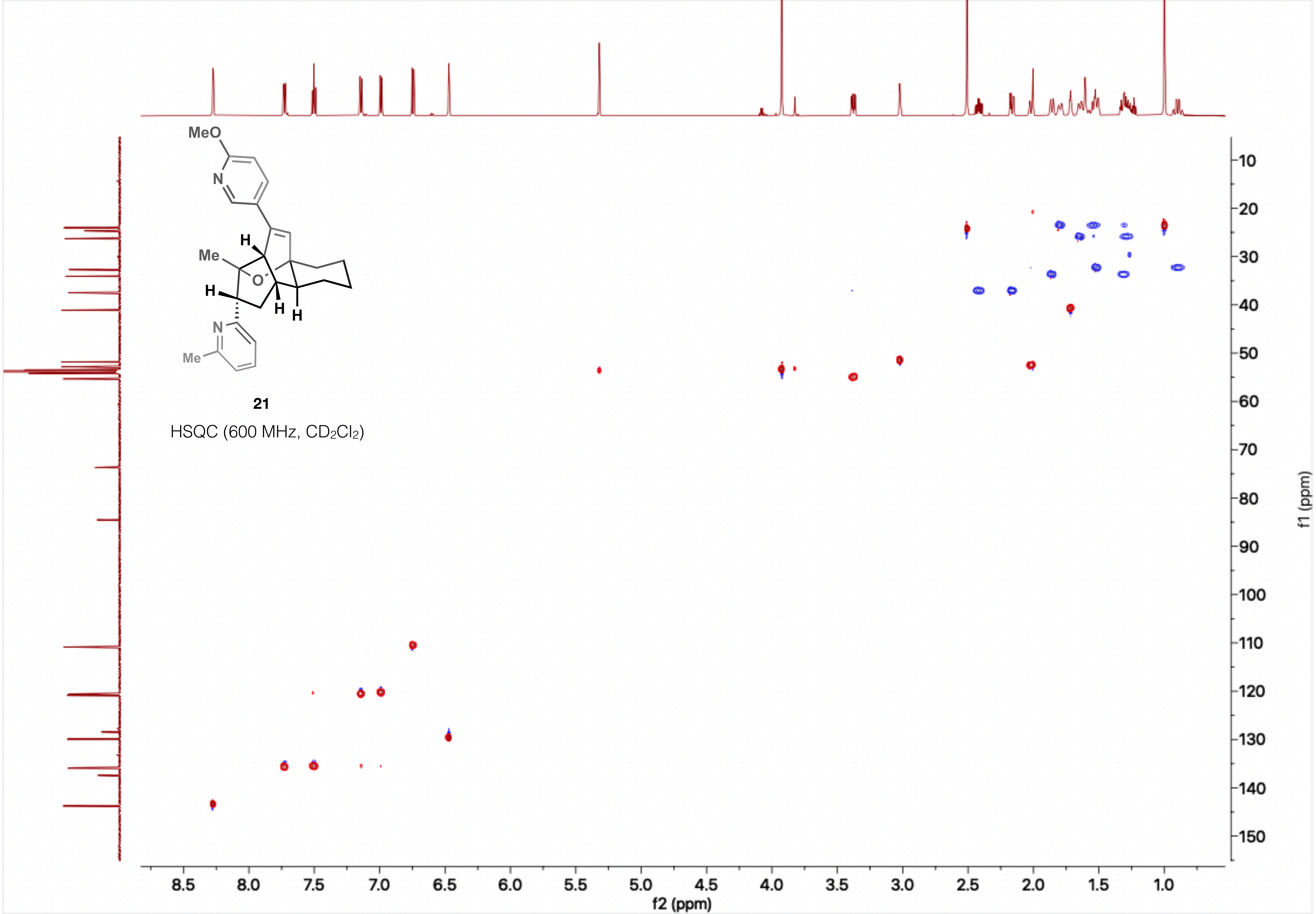

21

HSQC (600 MHz, CD_2Cl_2)

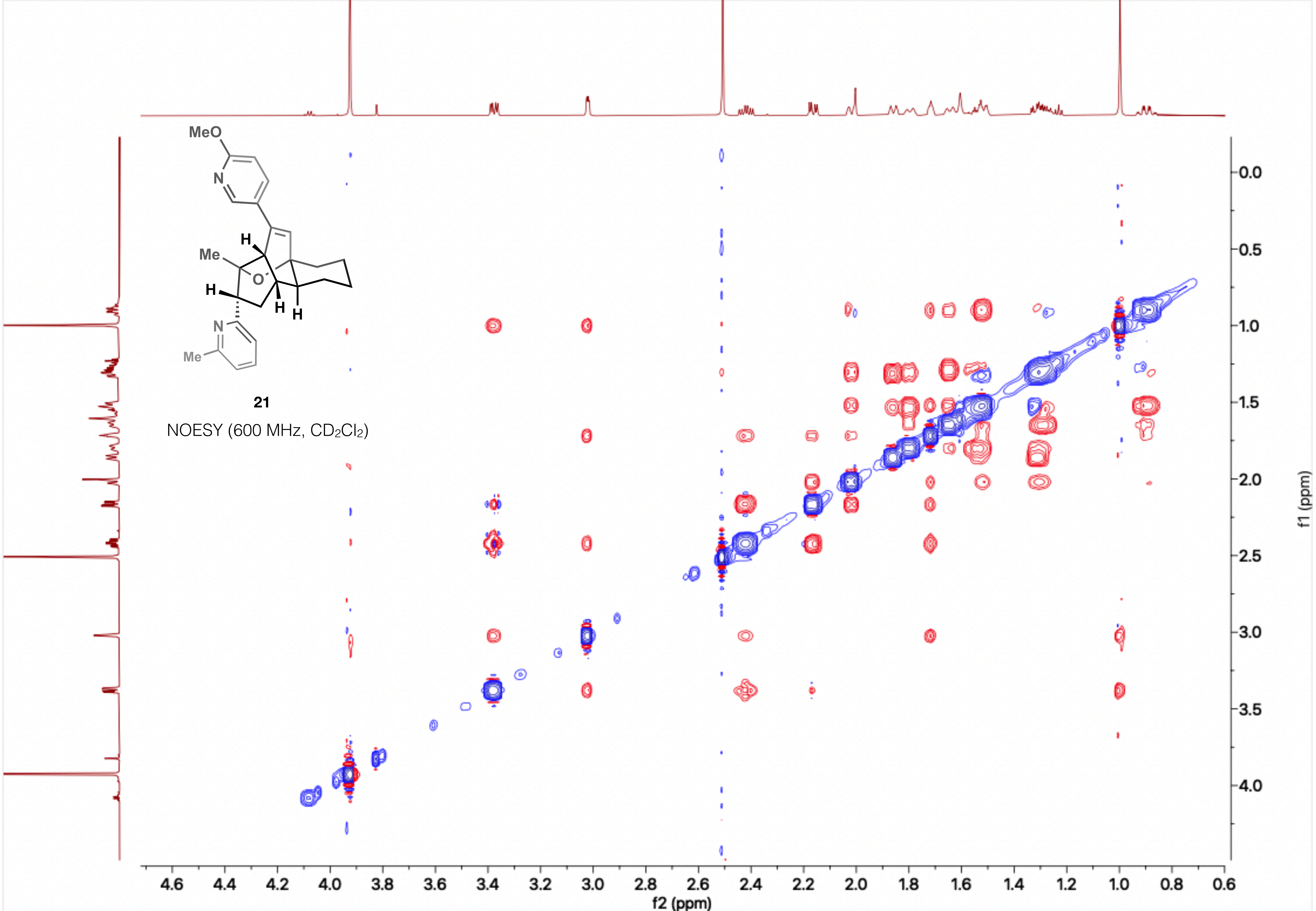

21

NOESY (600 MHz, CD_2Cl_2)

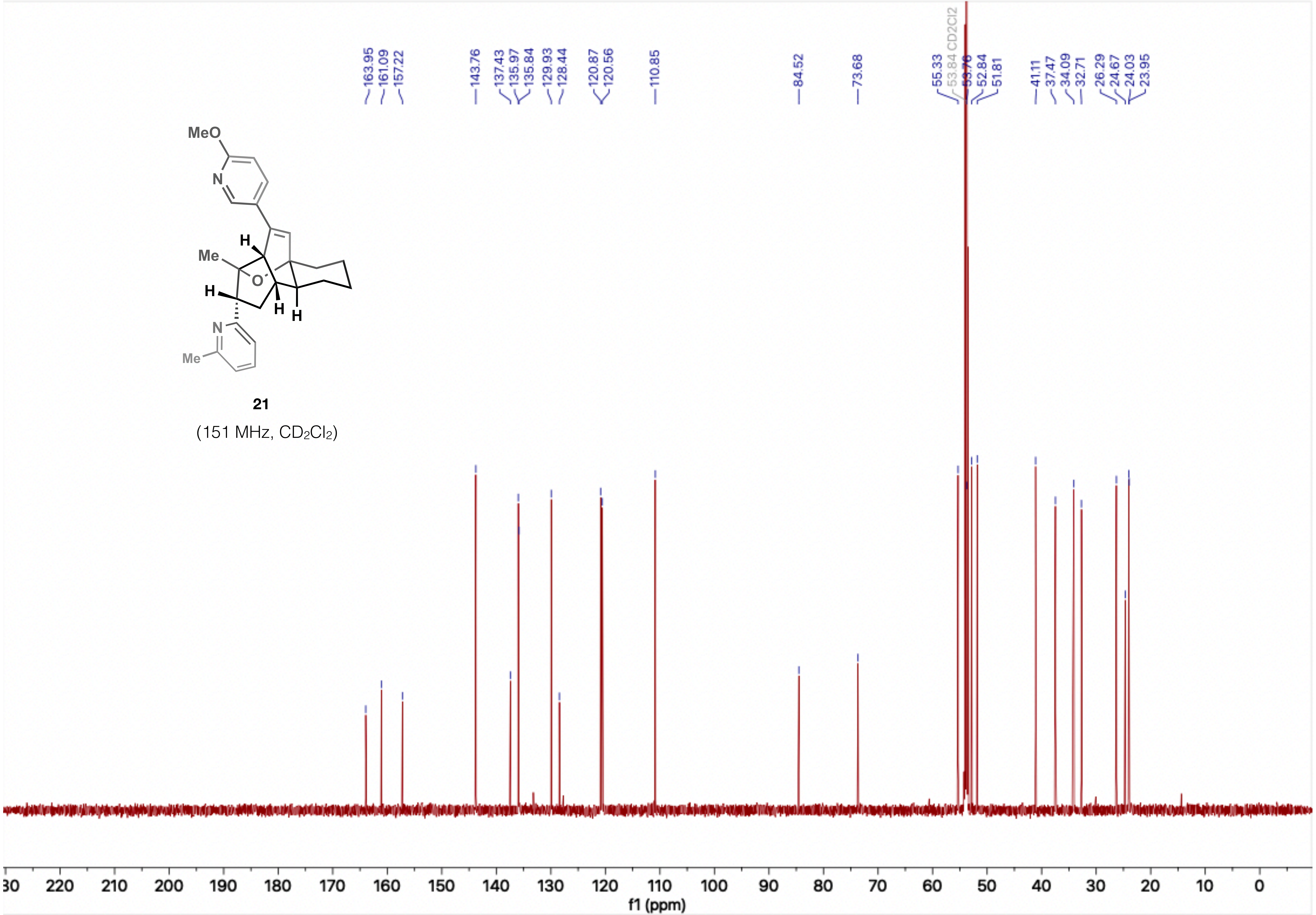

21

(151 MHz, CD_2Cl_2)

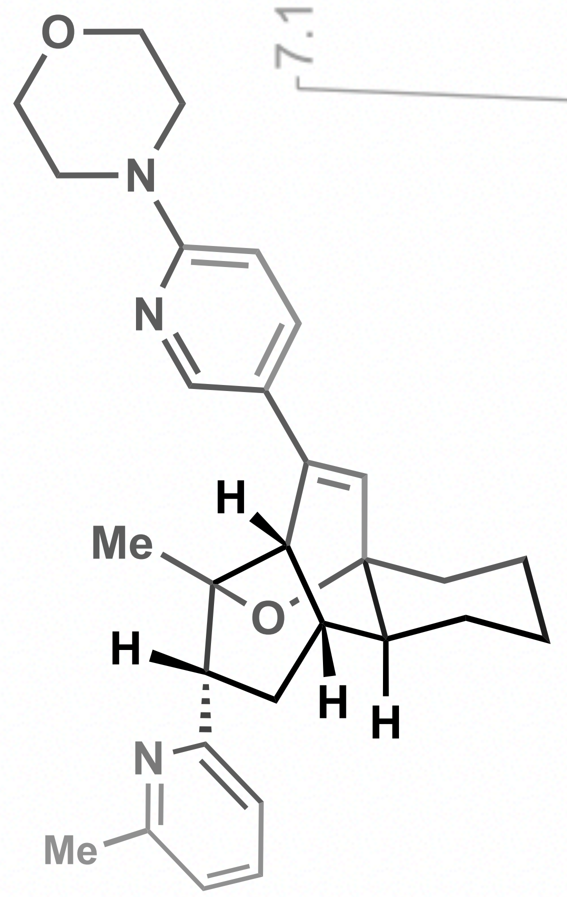

22
(600 MHz, C_6D_6)
5 mol% boronic ester-derived impurities

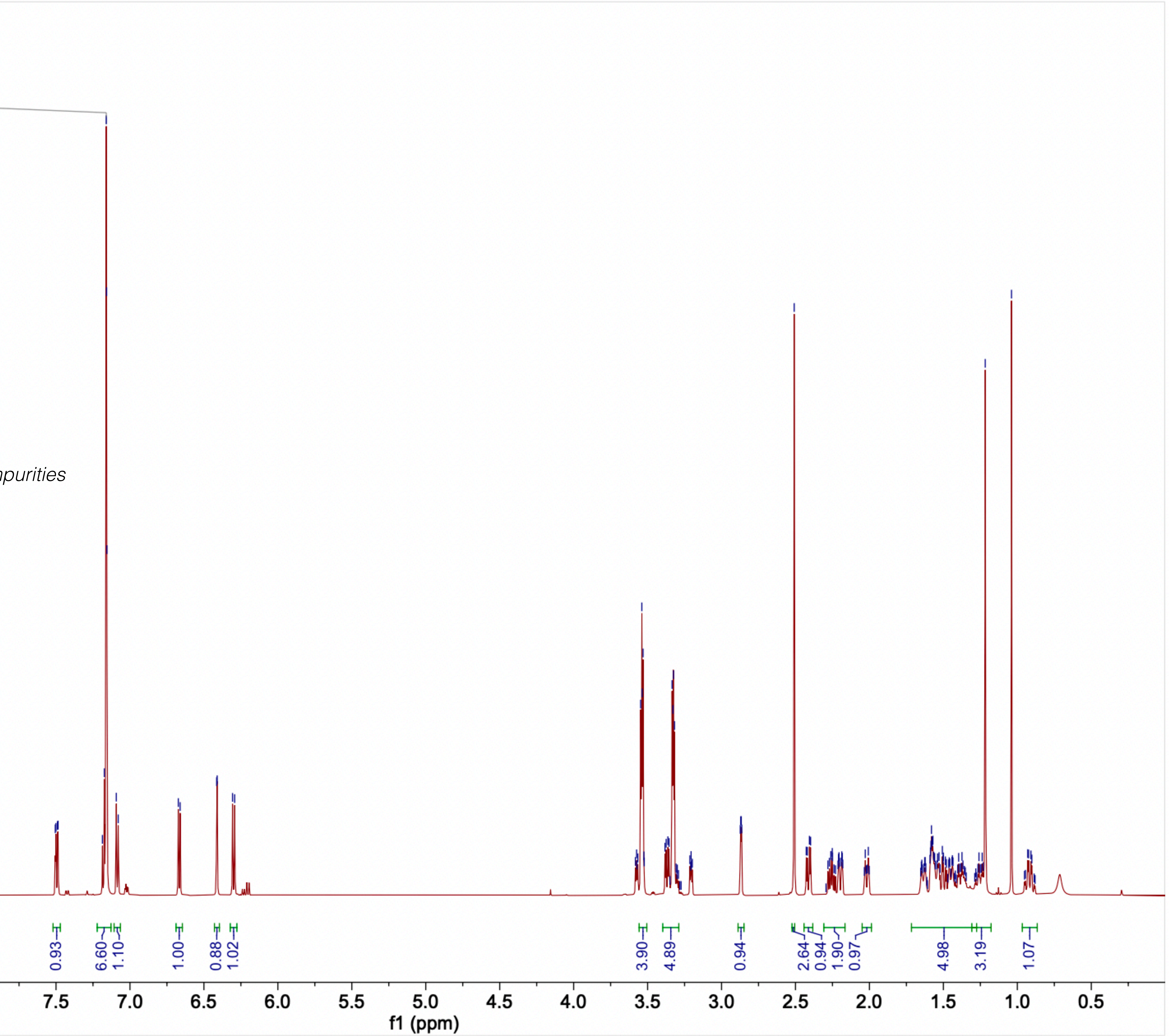

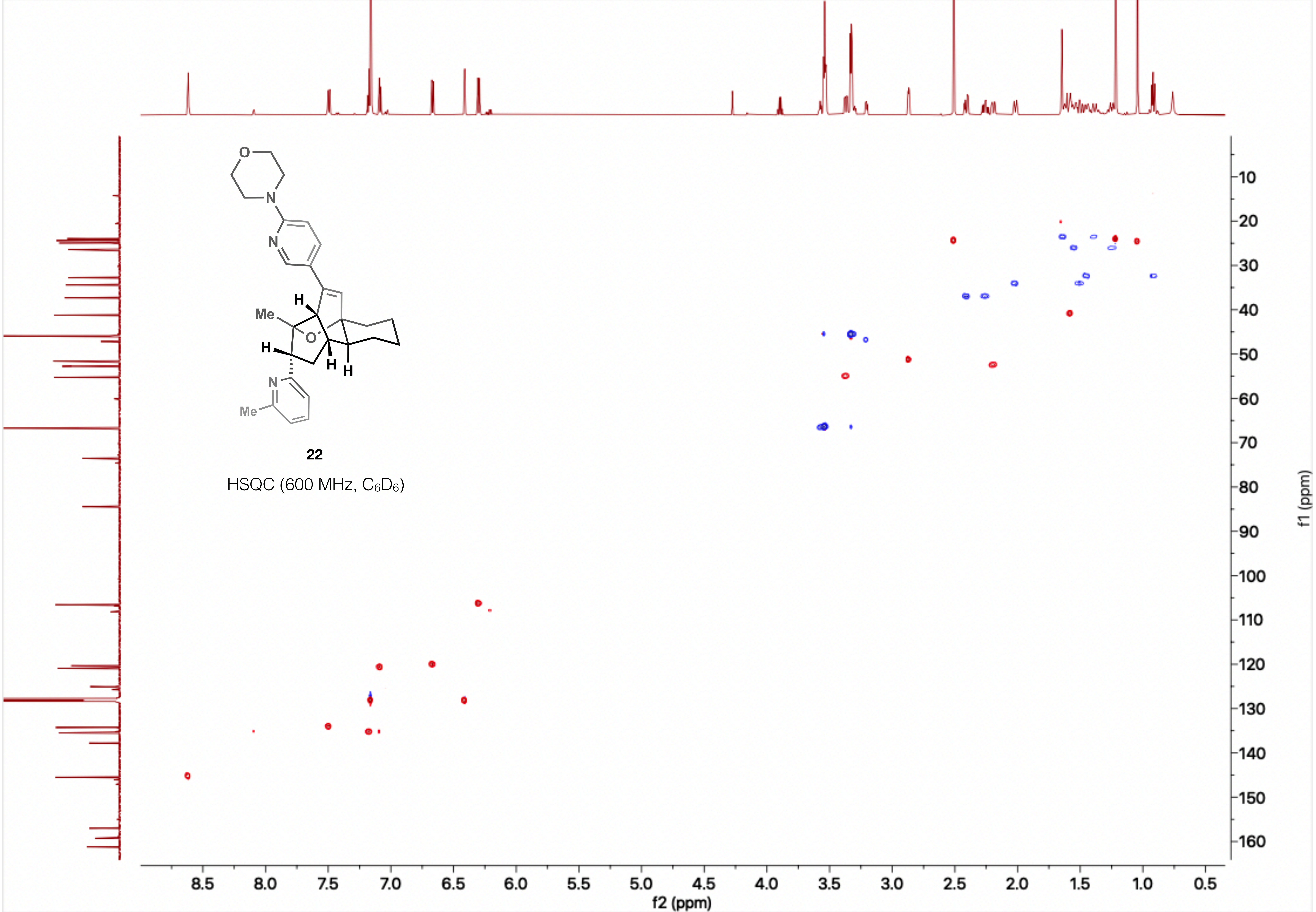

22

HSQC (600 MHz, C_6D_6)

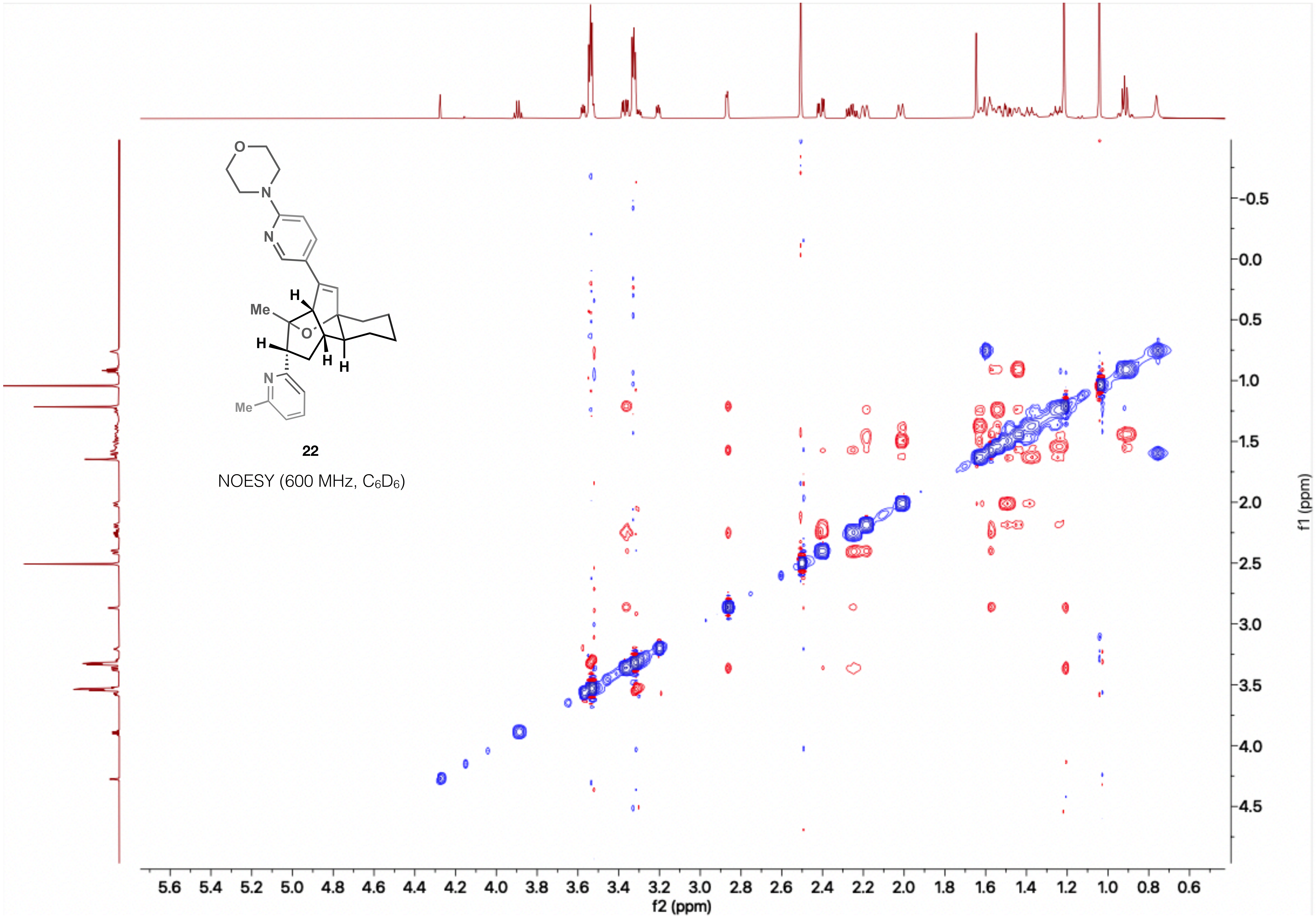
22
NOESY (600 MHz, C_6D_6)
f2 (ppm)
f1 (ppm)

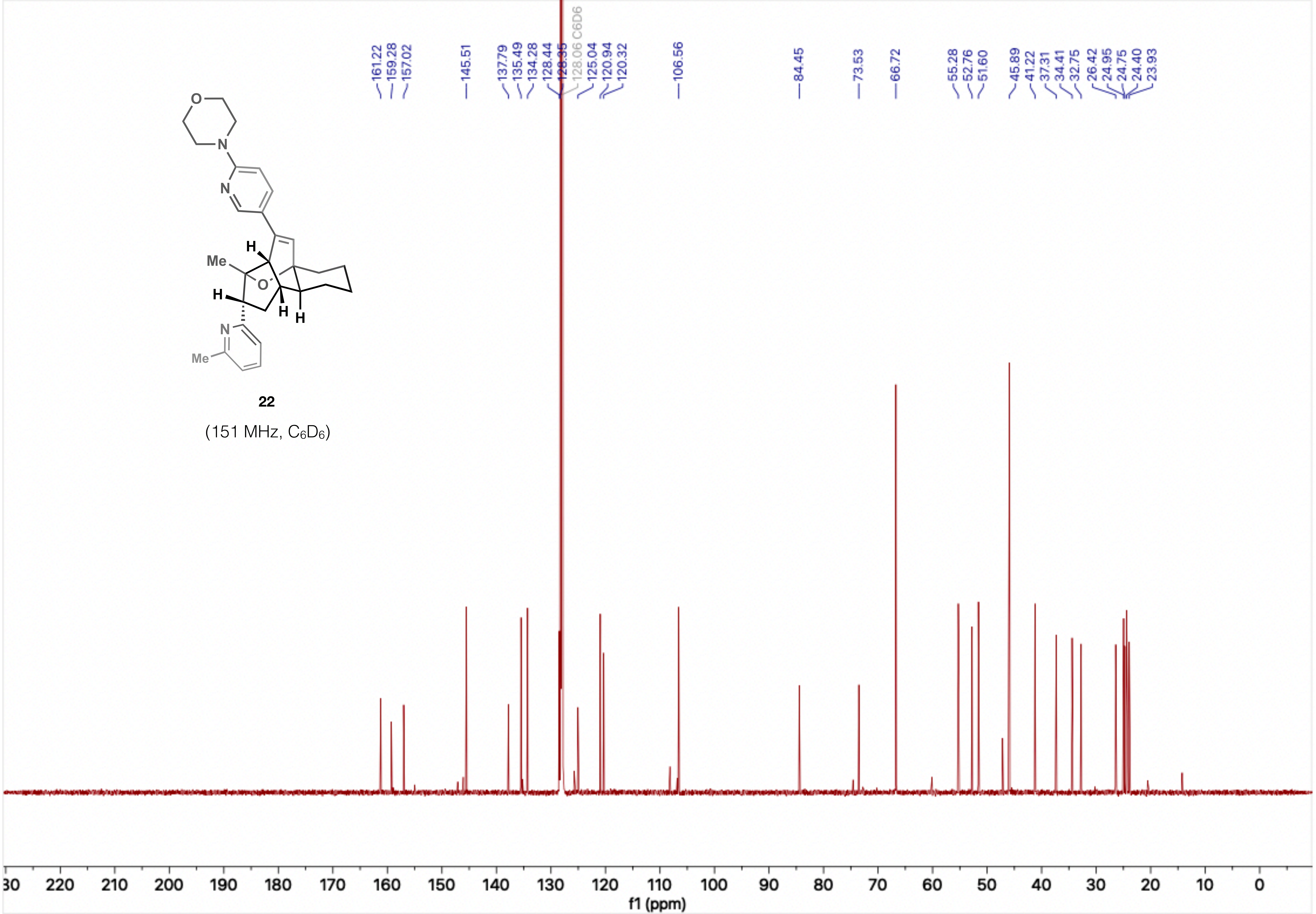

161.22
159.28
157.02
145.51
137.79
135.49
134.28
128.44
128.35
128.06 C6D6
125.04
120.94
120.32
106.56
84.45
73.53
66.72
55.28
52.76
51.60
45.89
41.22
37.31
34.41
32.75
26.42
24.95
24.75
24.40
23.93
Me
H
O
N
22
(151 MHz, C_6D_6)
f1 (ppm)

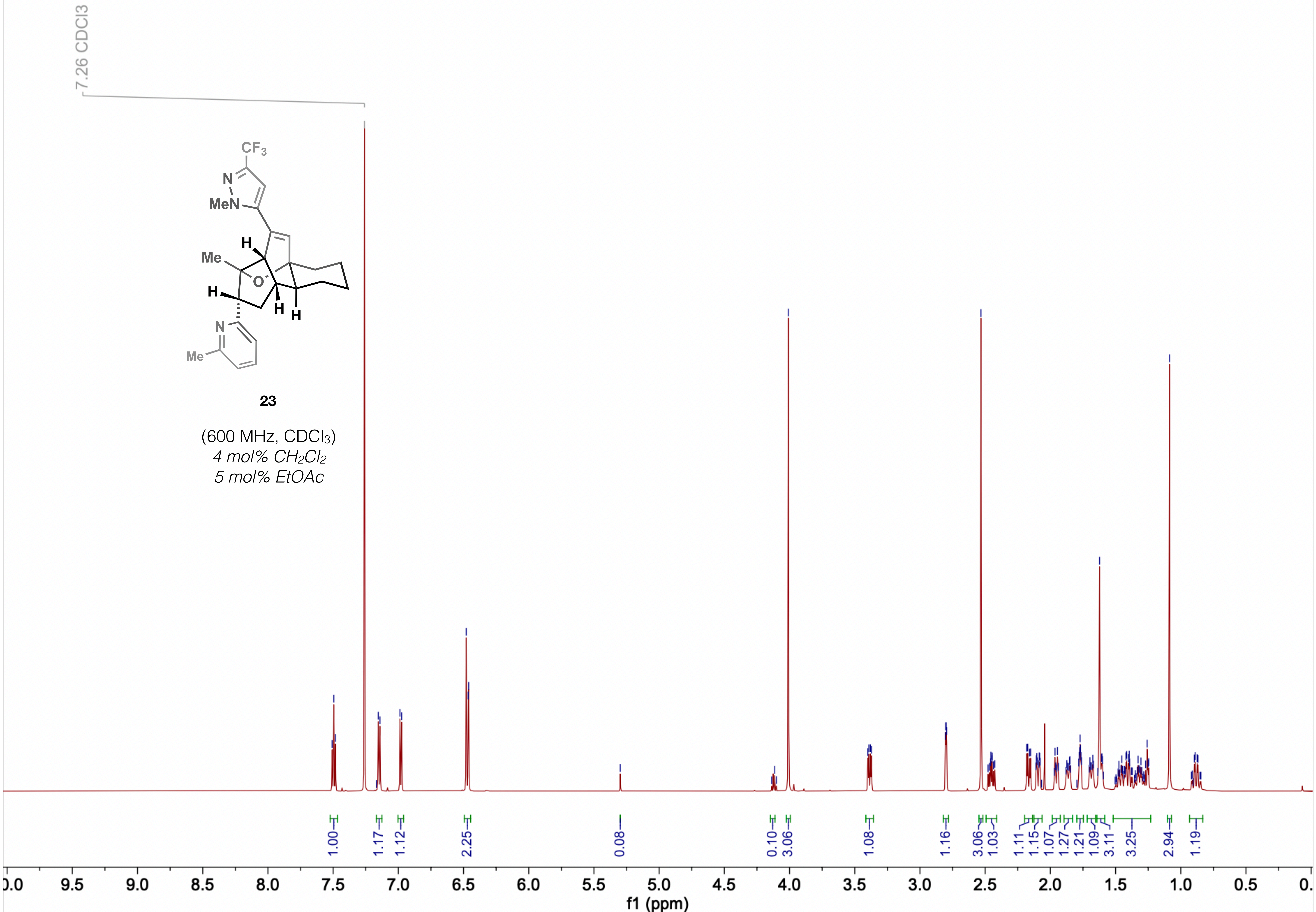

7.26 CDCl3
CF3
N
MeN
H
Me
O
H
H H
N
Me
23
(600 MHz, CDCl3)
4 mol% CH2Cl2
5 mol% EtOAc
1.00
1.17
1.12
2.25
0.08
0.10
3.06
1.08
1.16
3.06
1.03
1.11
1.15
1.07
1.27
1.21
1.09
3.11
3.25
2.94
1.19
f1 (ppm)

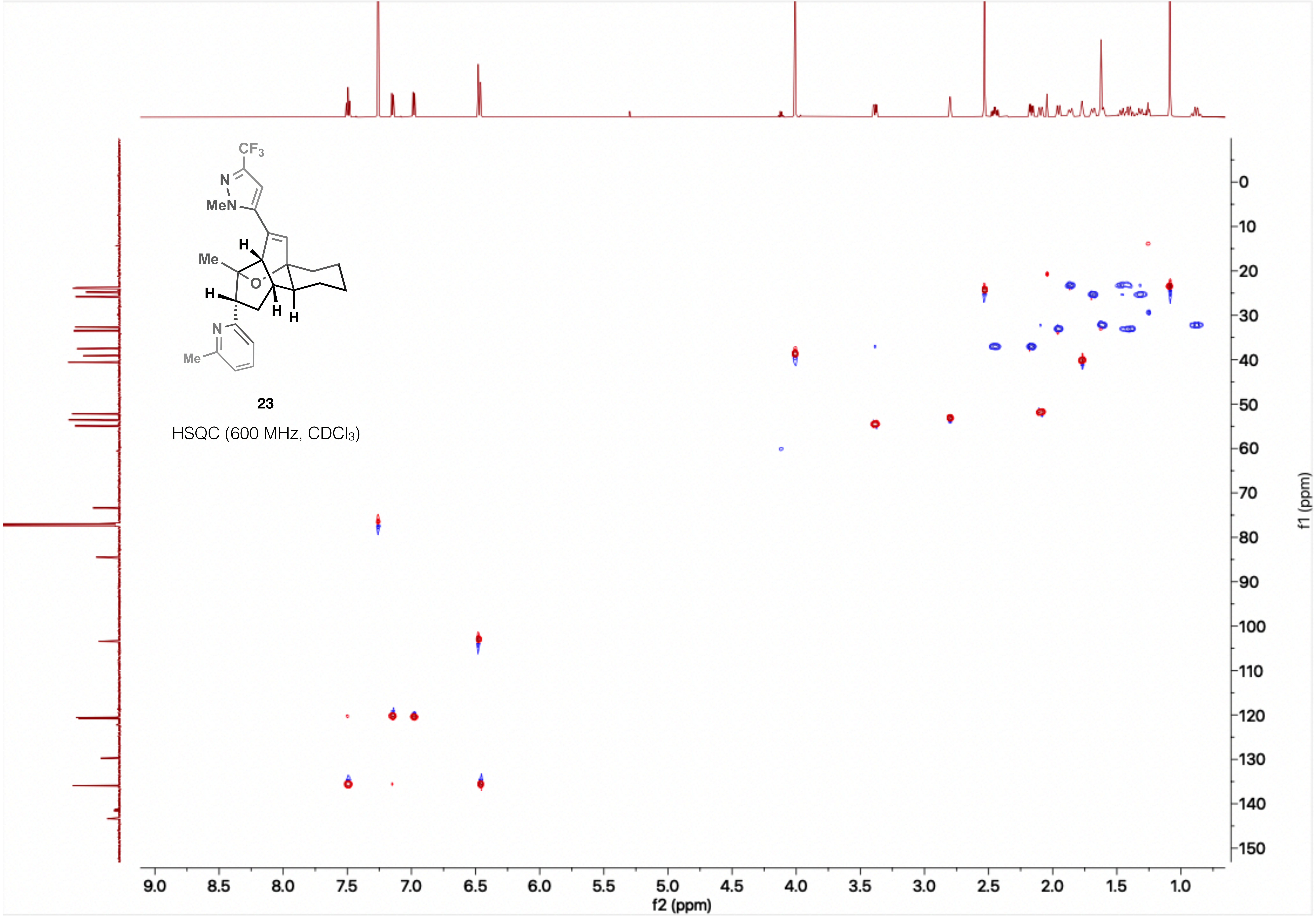
CF3
N
MeN
H
Me
O
H
H
H
N
Me
23
HSQC (600 MHz, CDCl3)
f2 (ppm)
f1 (ppm)
9.0 8.5 8.0 7.5 7.0 6.5 6.0 5.5 5.0 4.5 4.0 3.5 3.0 2.5 2.0 1.5 1.0
0 10 20 30 40 50 60 70 80 90 100 110 120 130 140 150

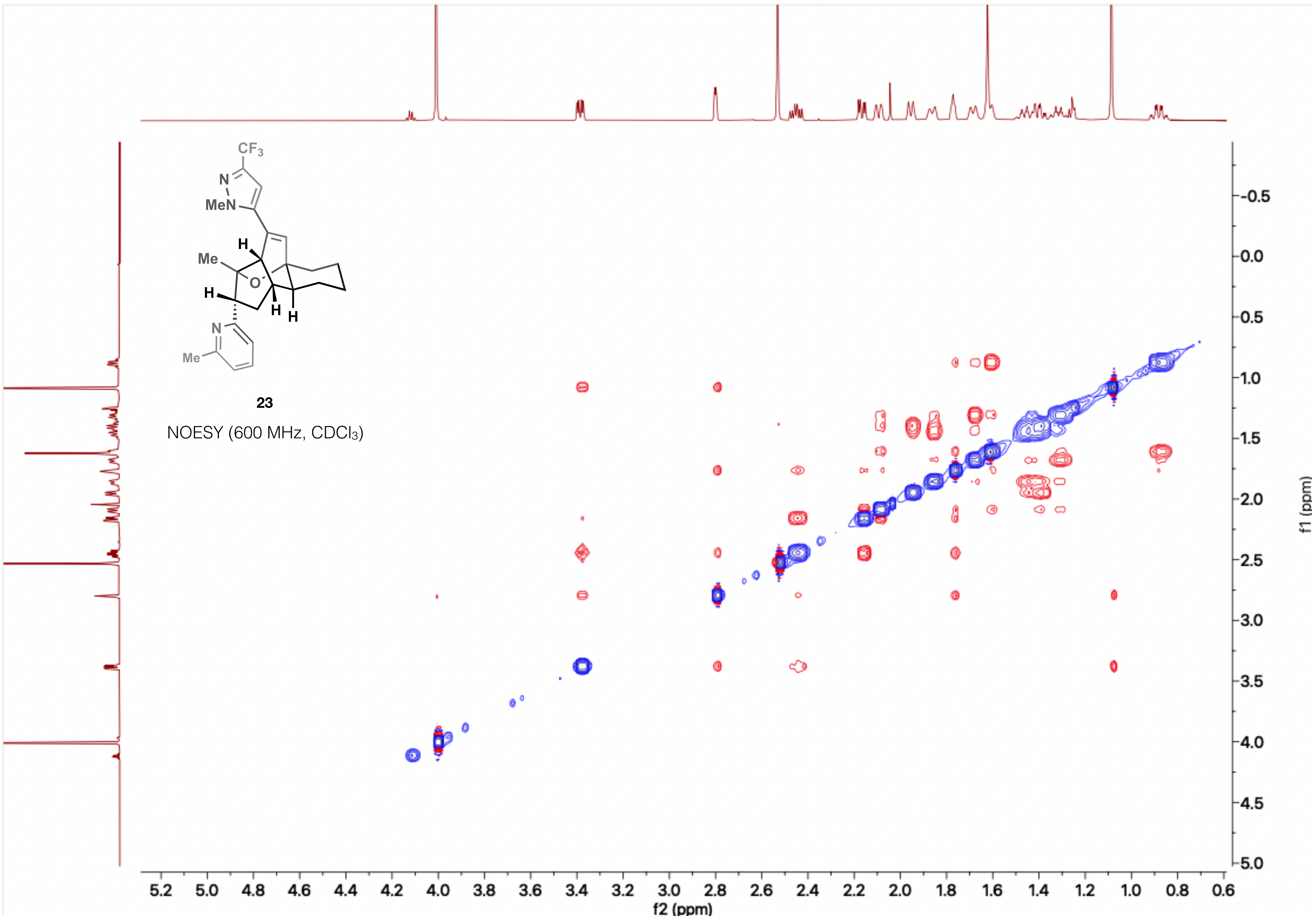

CF3
N
MeN
H
Me
O
H
H
H
N
Me
23
NOESY (600 MHz, CDCl3)
f2 (ppm)
5.2 5.0 4.8 4.6 4.4 4.2 4.0 3.8 3.6 3.4 3.2 3.0 2.8 2.6 2.4 2.2 2.0 1.8 1.6 1.4 1.2 1.0 0.8 0.6
f1 (ppm)
-0.5 0.0 0.5 1.0 1.5 2.0 2.5 3.0 3.5 4.0 4.5 5.0

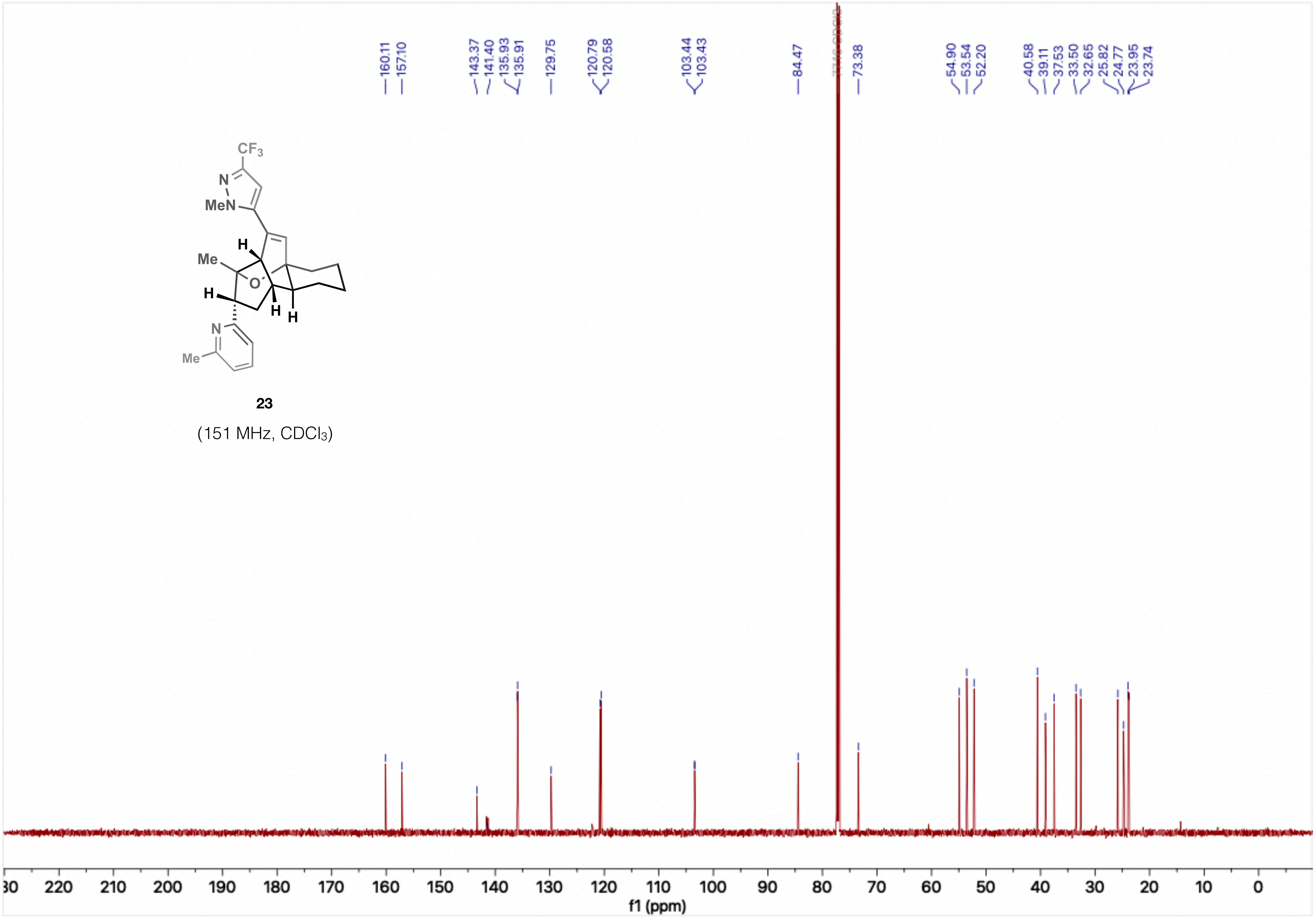

23

(151 MHz, $CDCl_3$)

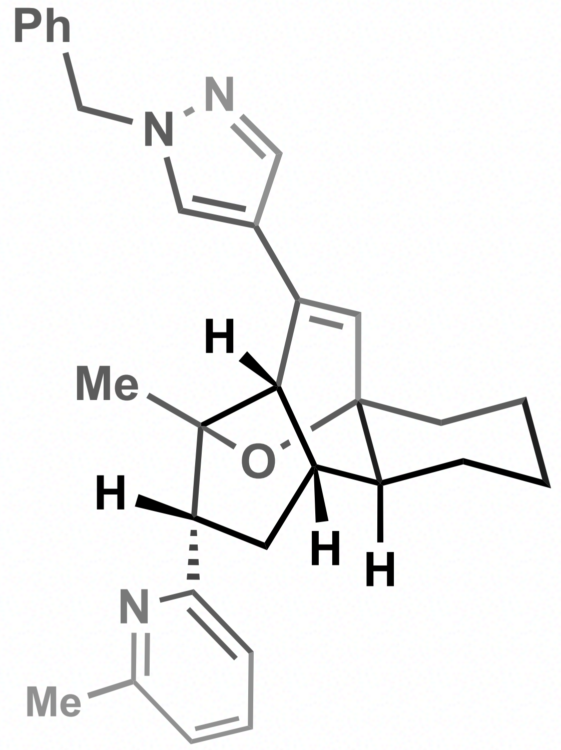

24
(600 MHz, CD_2Cl_2)
4 mol% EtOAc
1 mol% 1,4-dioxane

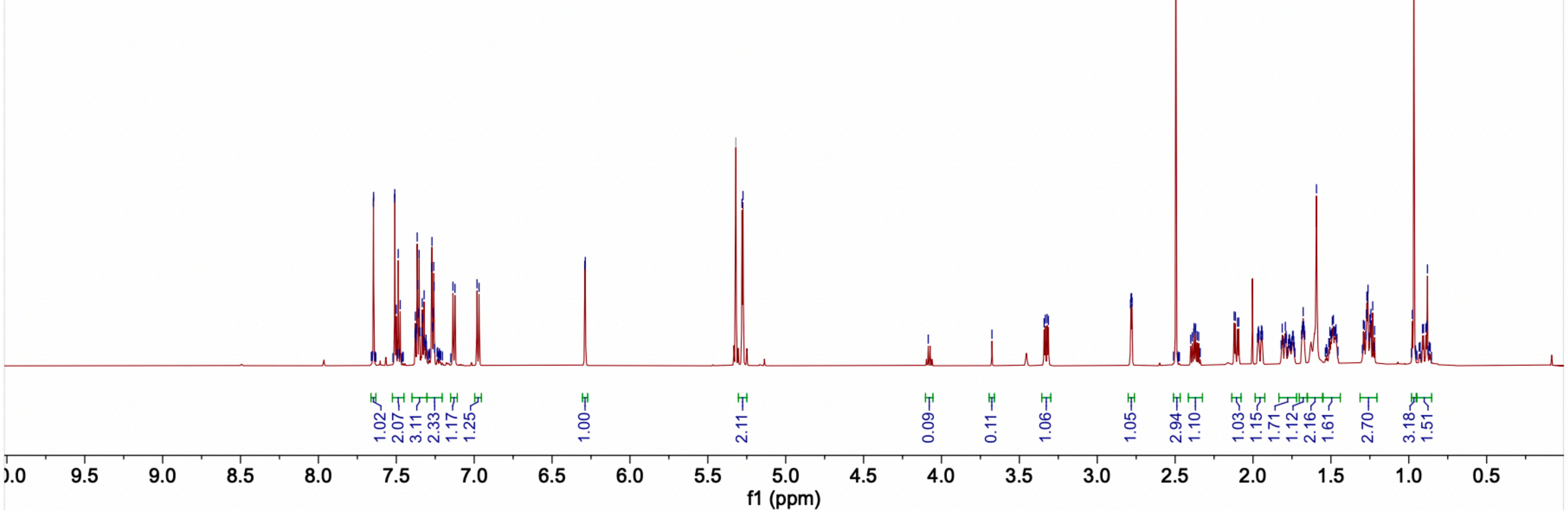

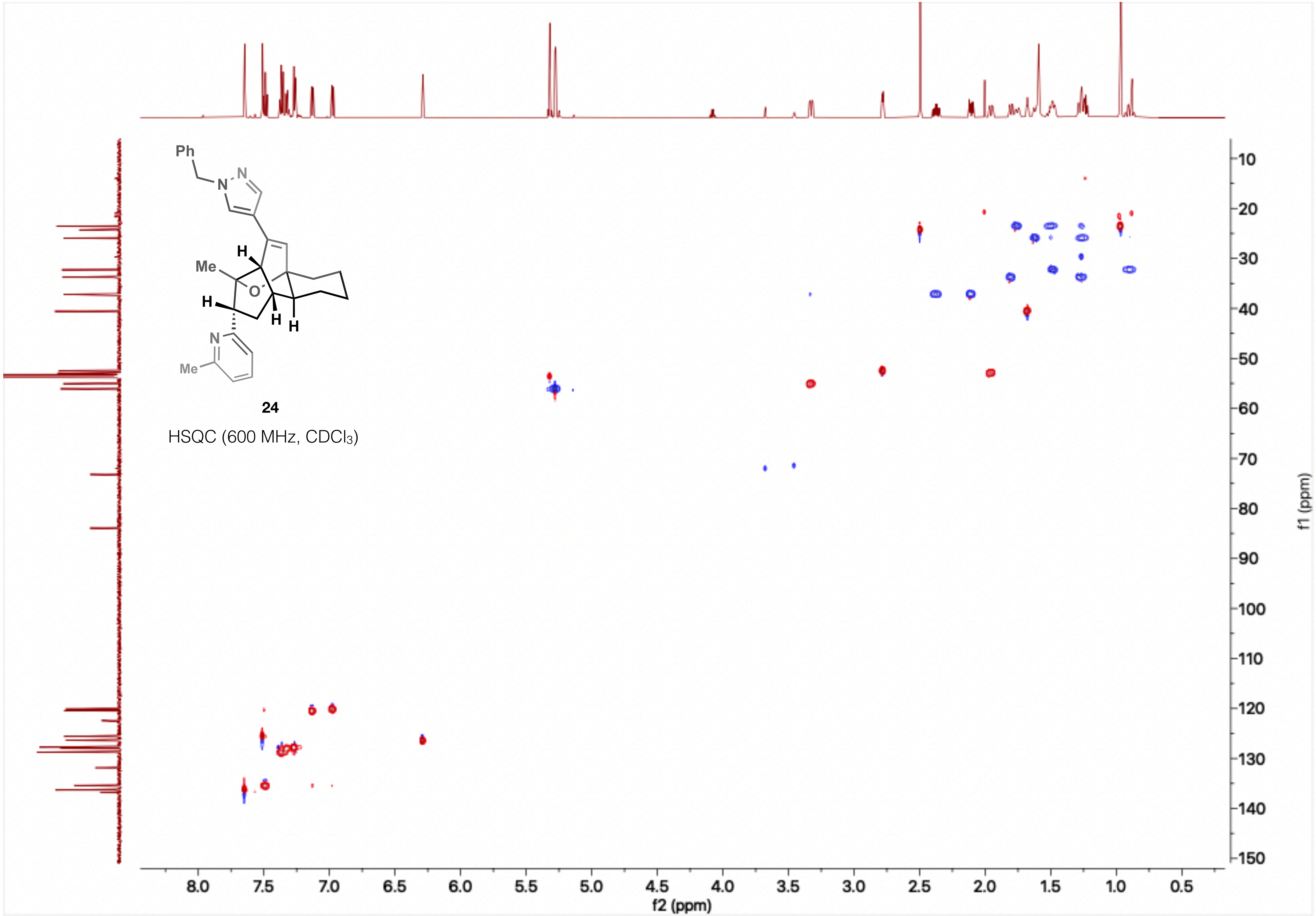

Ph
N
N
Me
H
O
H
H
H
N
Me
24
HSQC (600 MHz, CDCl3)
f1 (ppm)
f2 (ppm)

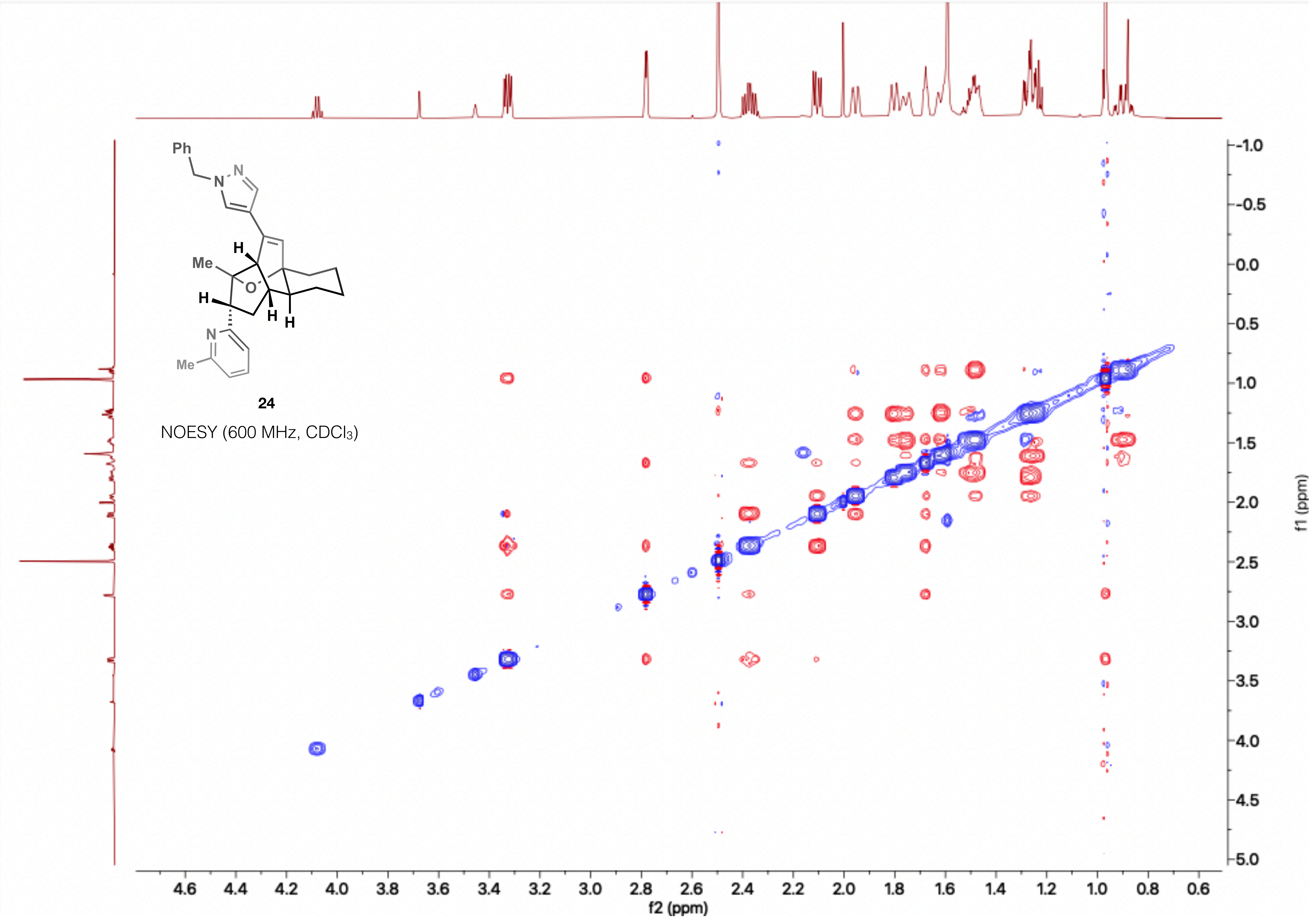

24

NOESY (600 MHz, CDCl3)

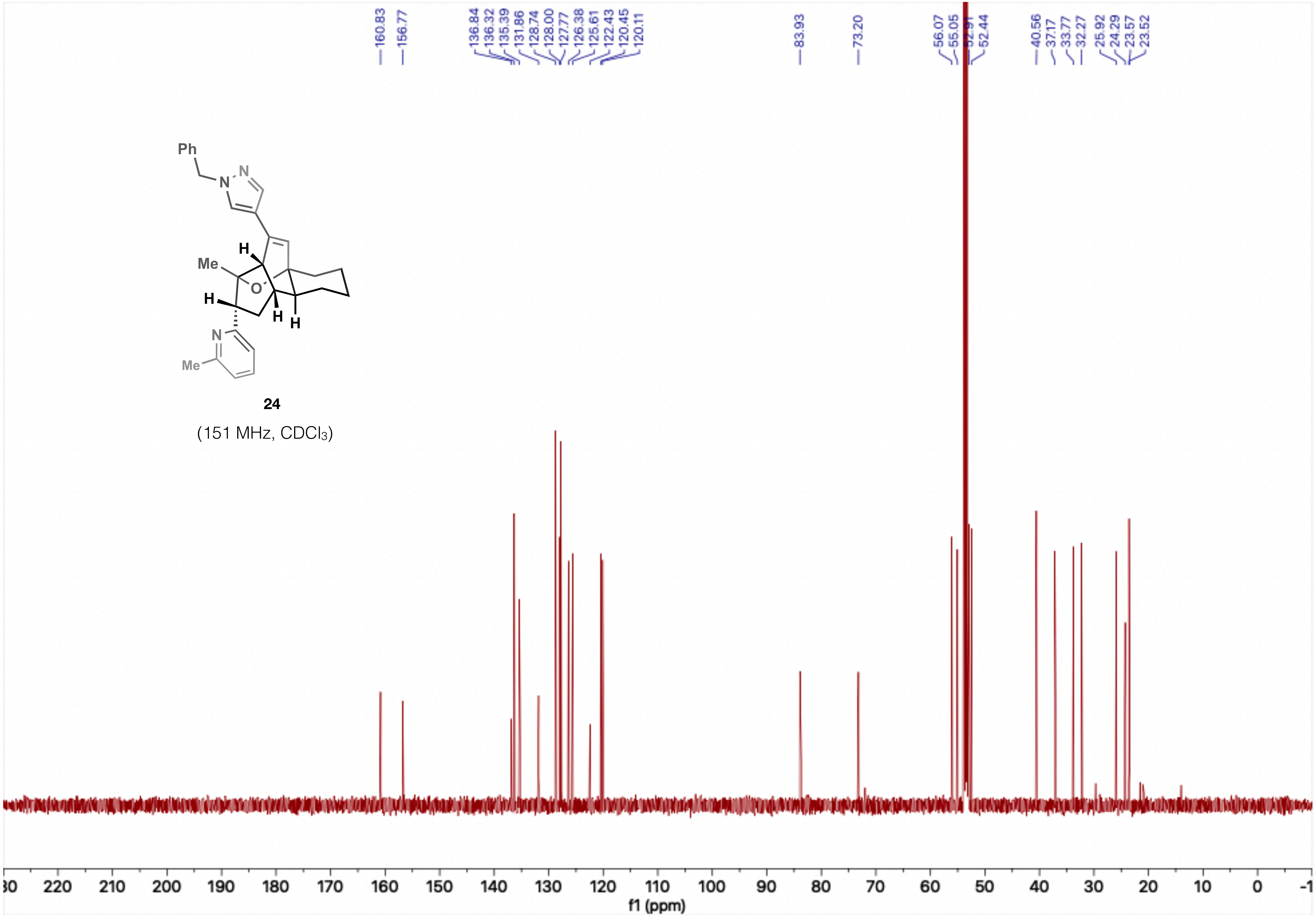
160.83
156.77
136.84
136.32
135.39
131.86
128.74
128.00
127.77
126.38
125.61
122.43
120.45
120.11
83.93
73.20
56.07
55.05
52.91
52.44
40.56
37.17
33.77
32.27
25.92
24.29
23.57
23.52
Ph
N
N
Me
H
O
H
H
H
N
Me
24
(151 MHz, CDCl3)
30
220
210
200
190
180
170
160
150
140
130
120
110
100
90
80
70
60
50
40
30
20
10
0
-1
f1 (ppm)

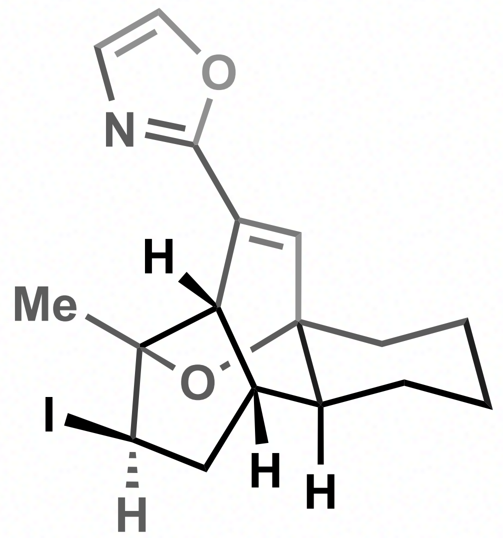

25

(600 MHz, C_6D_6)

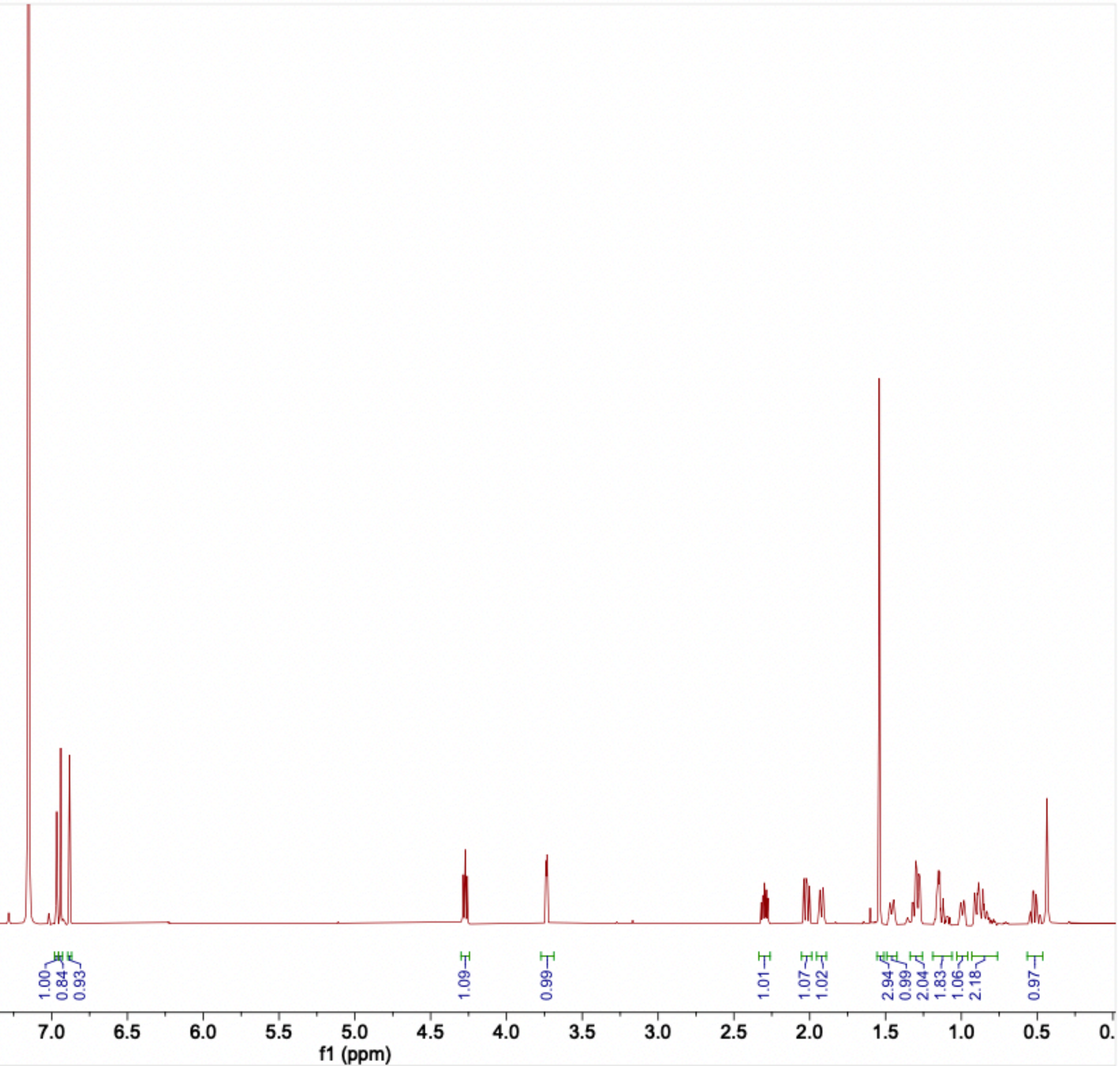

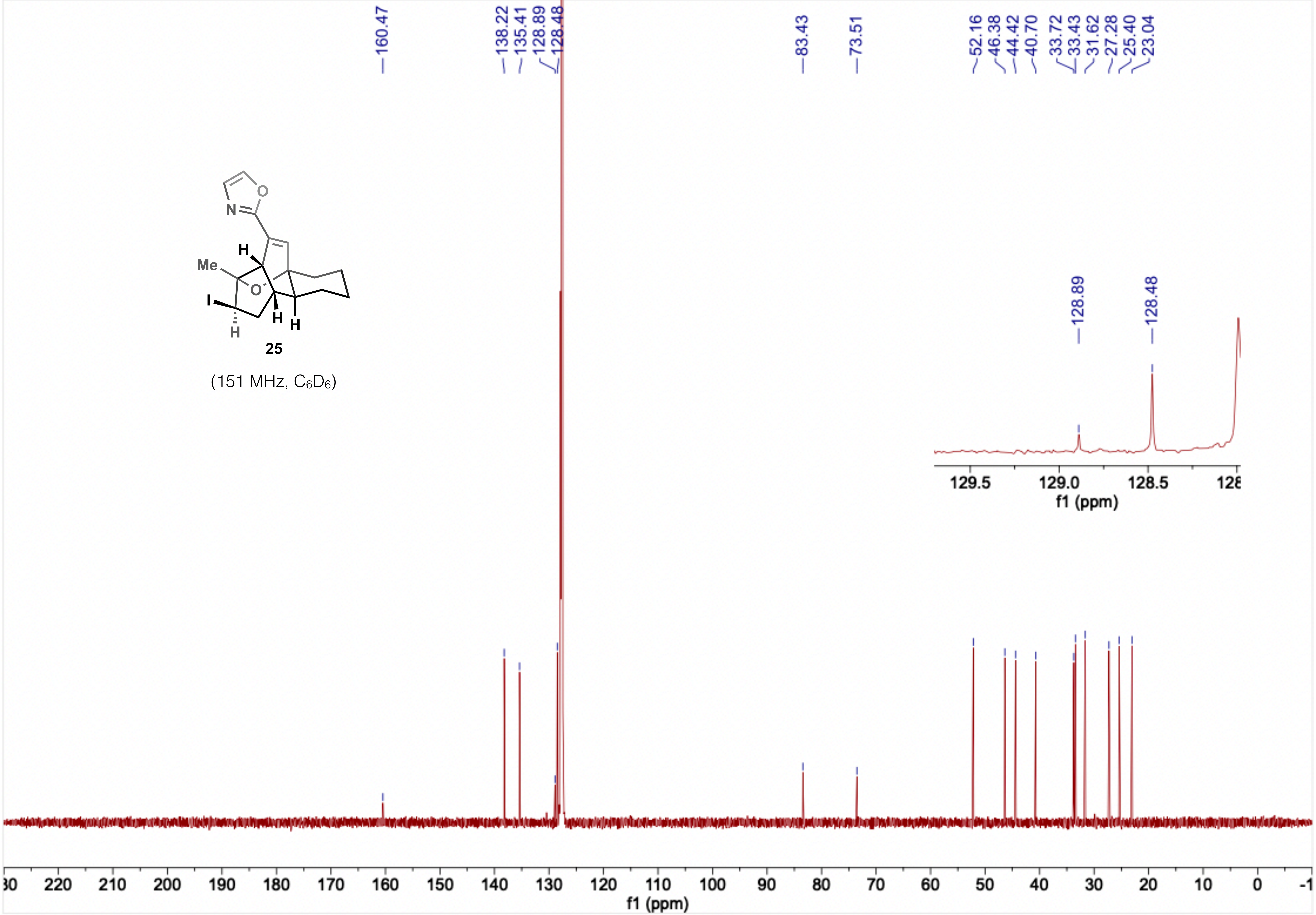

25

(151 MHz, C_6D_6)

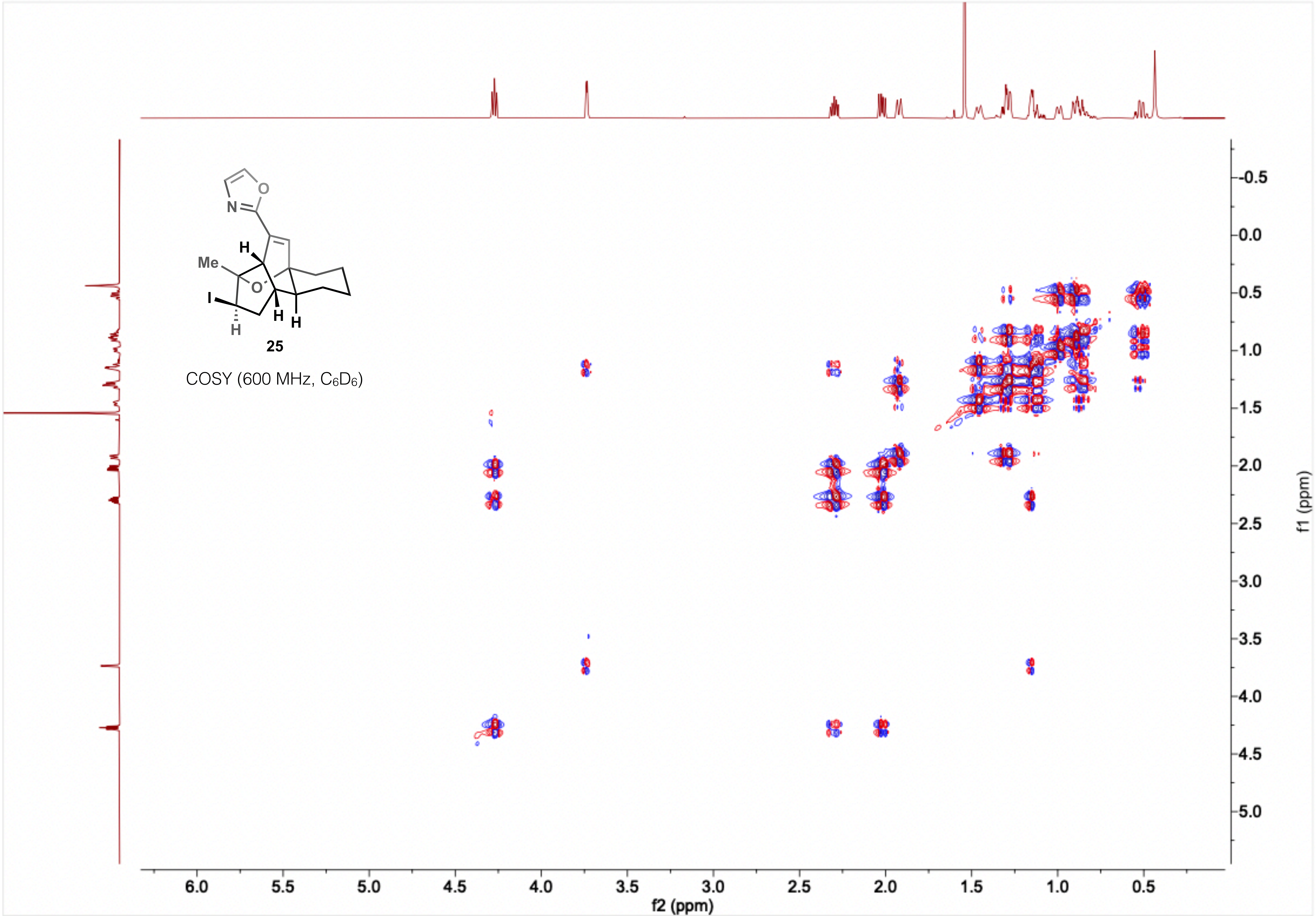

25
COSY (600 MHz, C_6D_6)
f2 (ppm)
f1 (ppm)

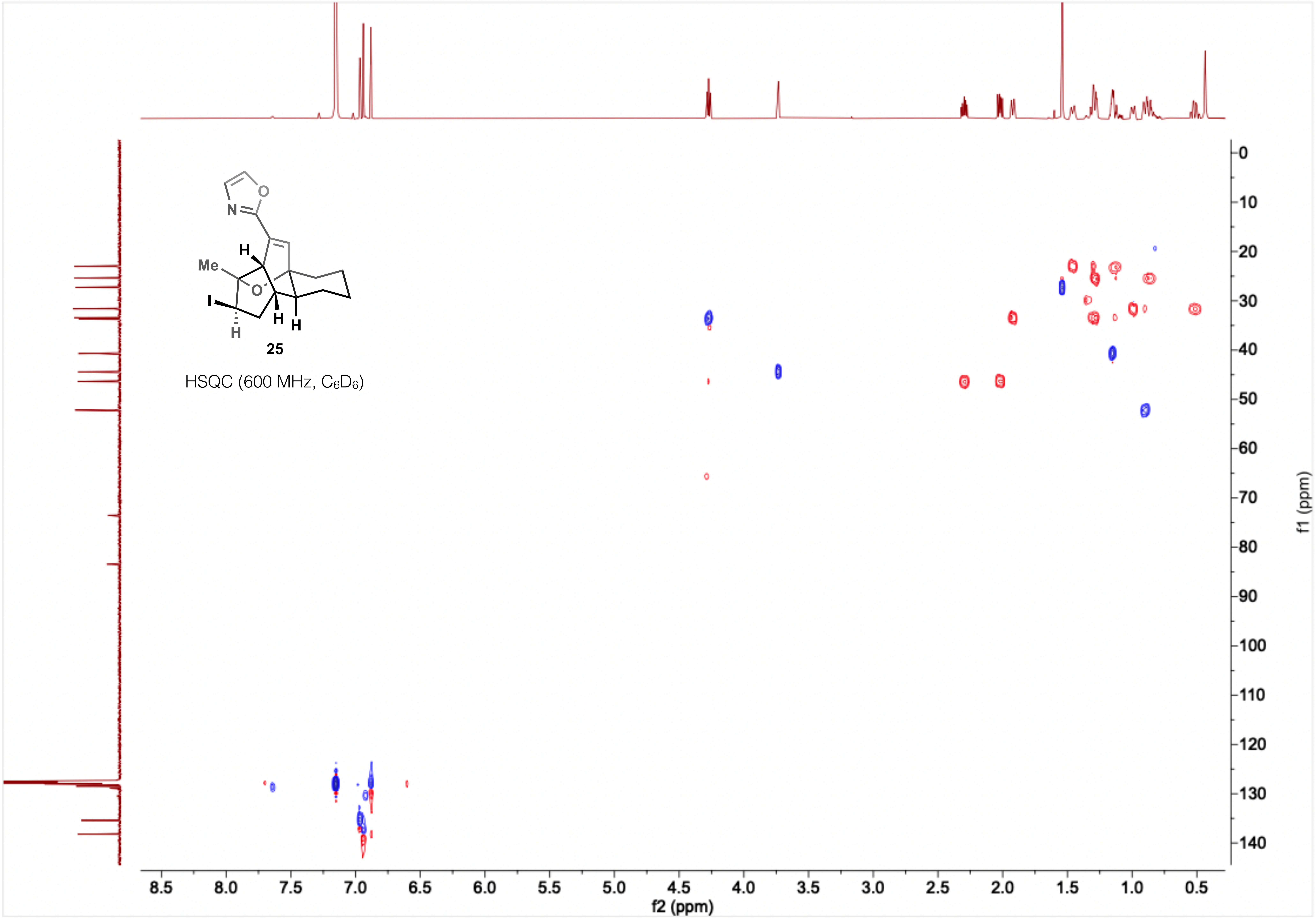

25
HSQC (600 MHz, C6D6)
f2 (ppm)
f1 (ppm)
8.5 8.0 7.5 7.0 6.5 6.0 5.5 5.0 4.5 4.0 3.5 3.0 2.5 2.0 1.5 1.0 0.5
0 10 20 30 40 50 60 70 80 90 100 110 120 130 140

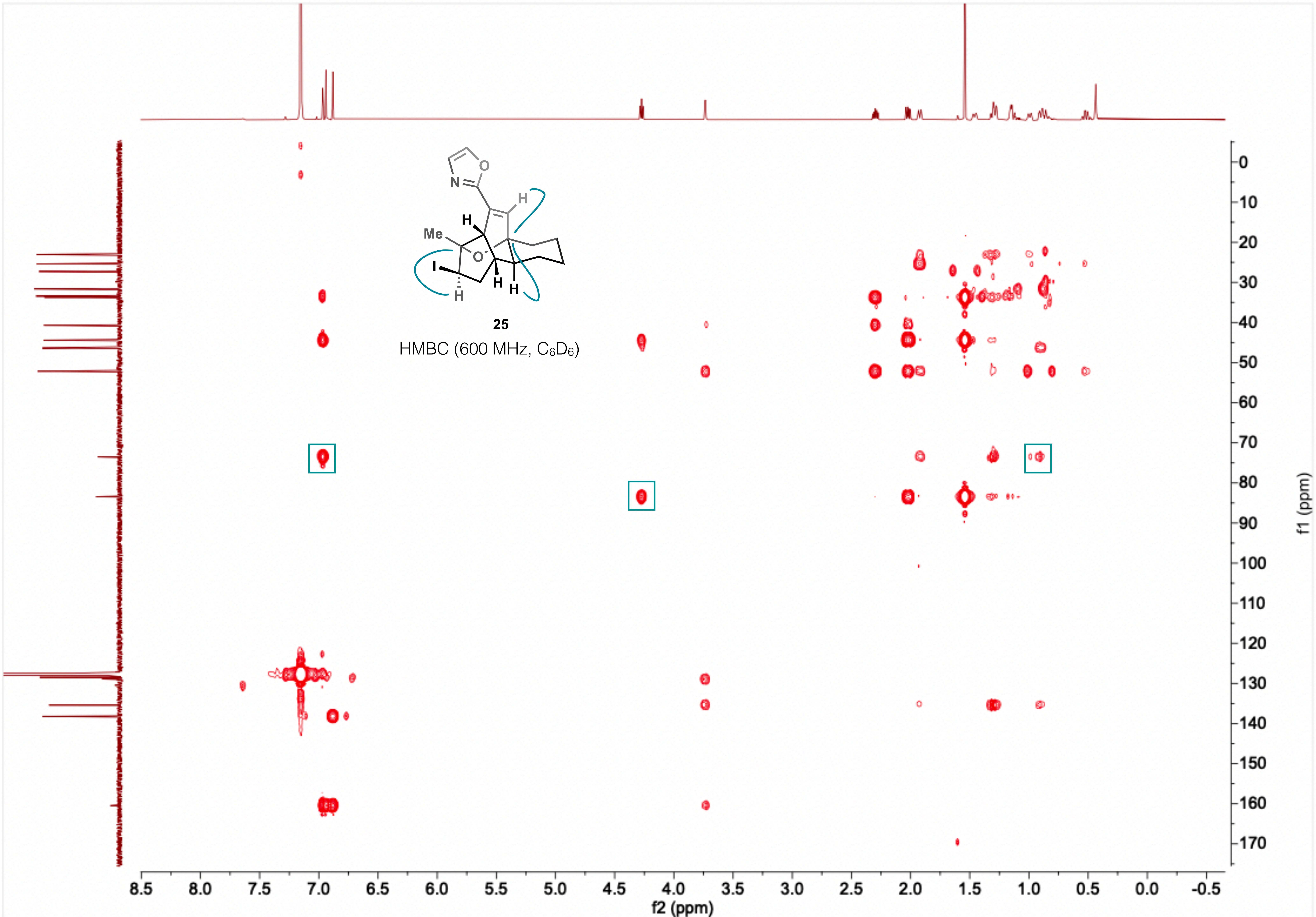

25
HMBC (600 MHz, C_6D_6)
f1 (ppm)
f2 (ppm)

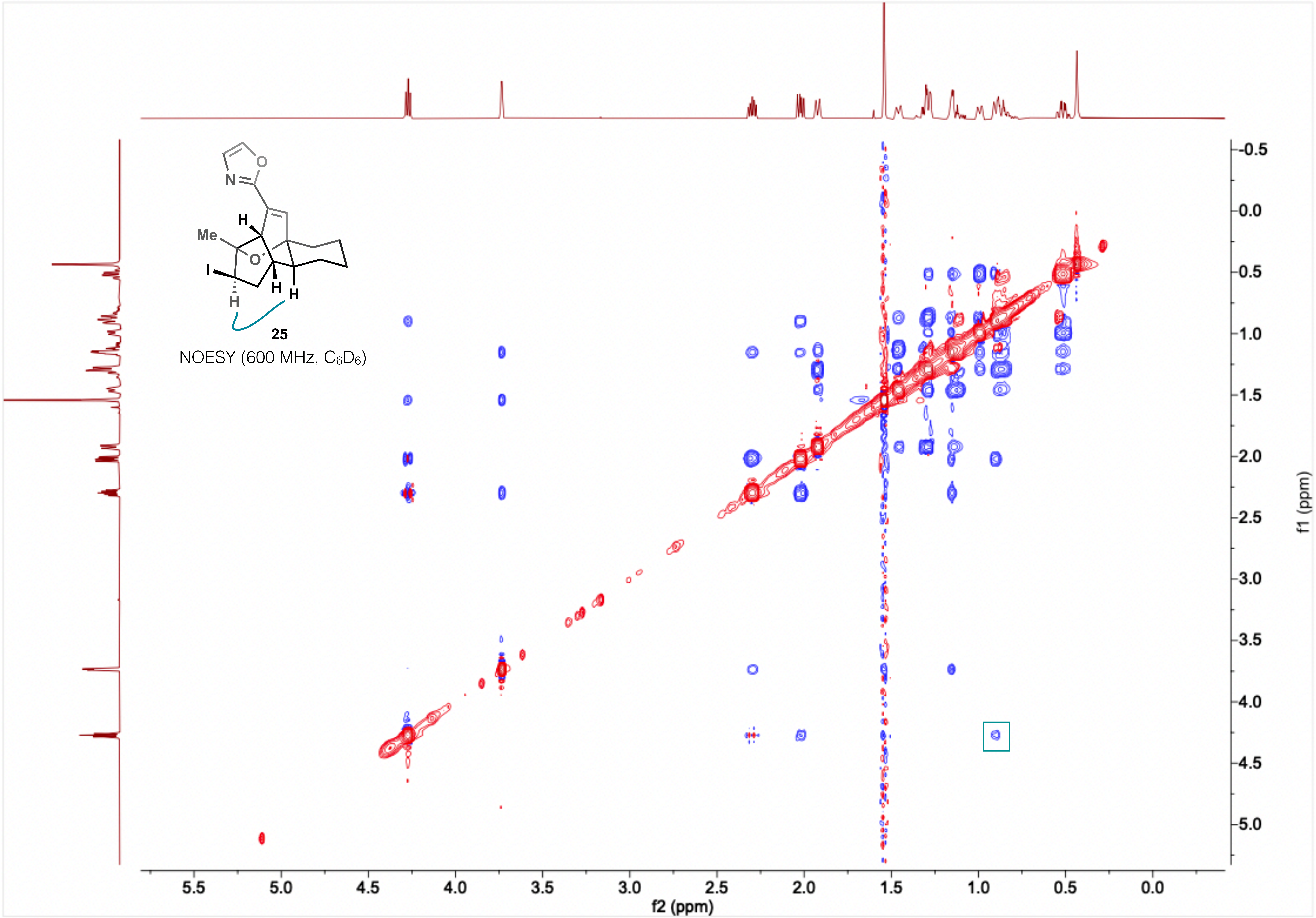

Me
O
I
H
N
25
NOESY (600 MHz, C6D6)
f2 (ppm)
f1 (ppm)

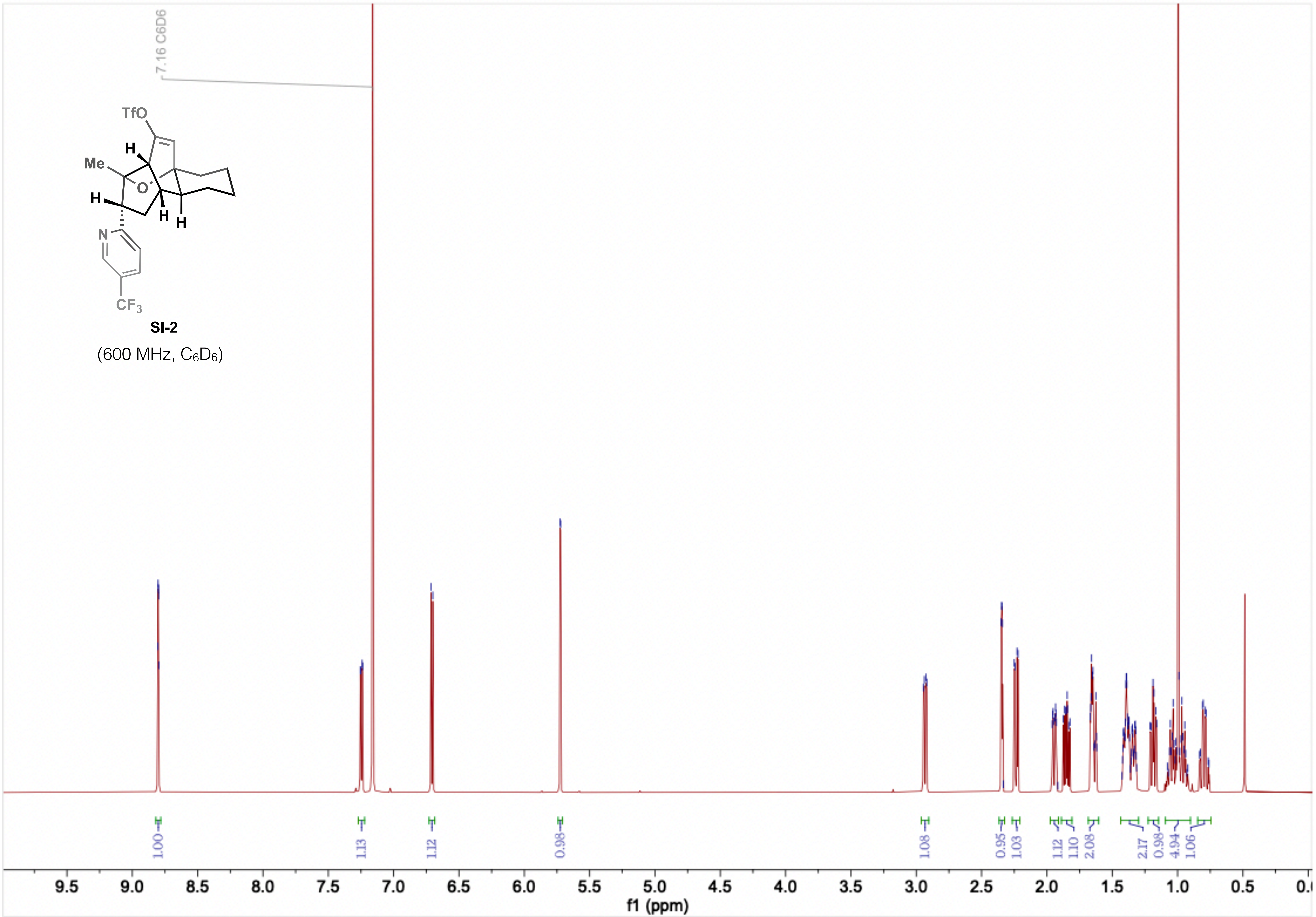
7.16 C6D6
TfO
Me
H
O
H
H
H
N
CF3
SI-2
(600 MHz, C6D6)
1.00
1.13
1.12
0.98
1.08
0.95
1.03
1.12
1.10
2.08
2.17
0.98
4.94
1.06
9.5 9.0 8.5 8.0 7.5 7.0 6.5 6.0 5.5 5.0 4.5 4.0 3.5 3.0 2.5 2.0 1.5 1.0 0.5
f1 (ppm)

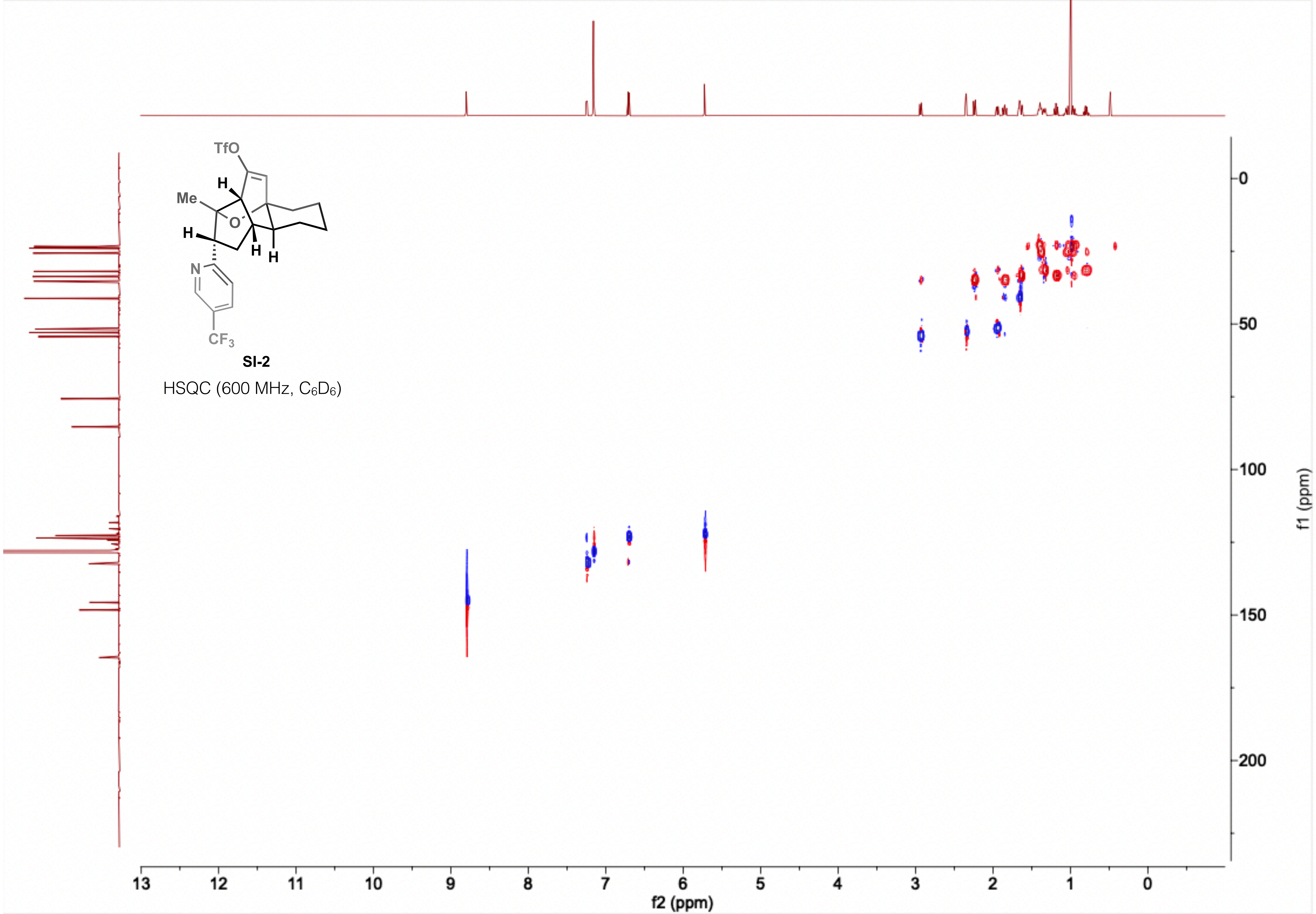
TfO
H
Me
O
H
H
H
N
CF3
SI-2
HSQC (600 MHz, C6D6)
f2 (ppm)
f1 (ppm)

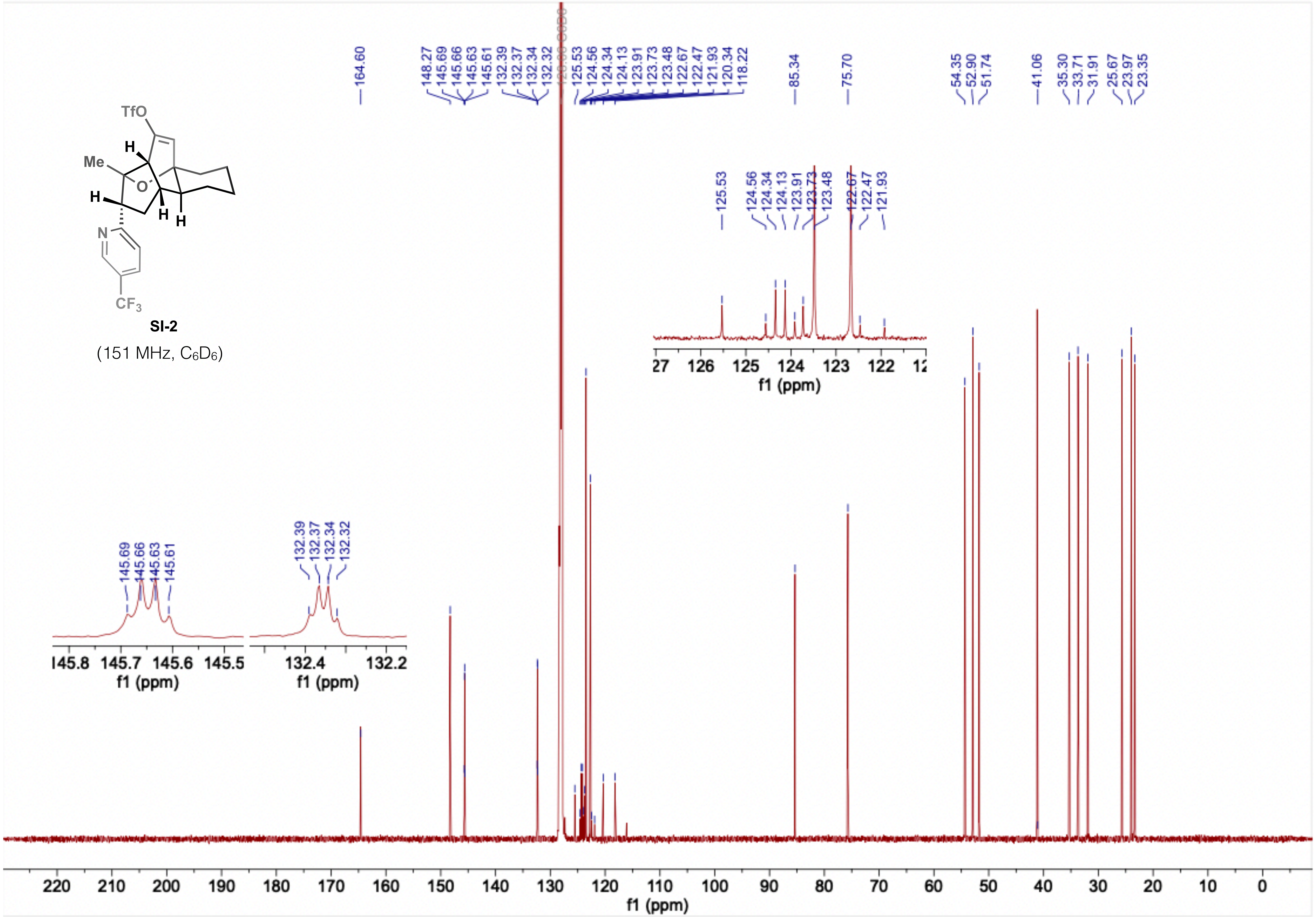
164.60
148.27
145.69
145.66
145.63
145.61
132.39
132.37
132.34
132.32
125.53
124.56
124.34
124.13
123.91
123.73
123.48
122.67
122.47
121.93
120.34
118.22
85.34
75.70
54.35
52.90
51.74
41.06
35.30
33.71
31.91
25.67
23.97
23.35
TfO
Me
H
O
N
CF3
SI-2
(151 MHz, C6D6)
f1 (ppm)

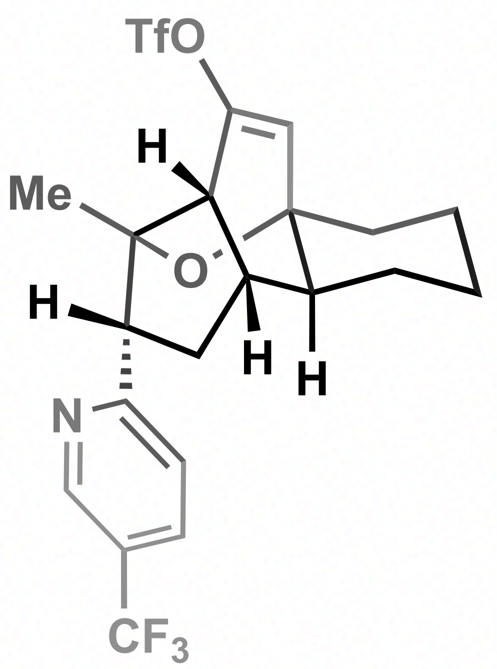

SI-2

19F (376 MHz, C_6D_6)

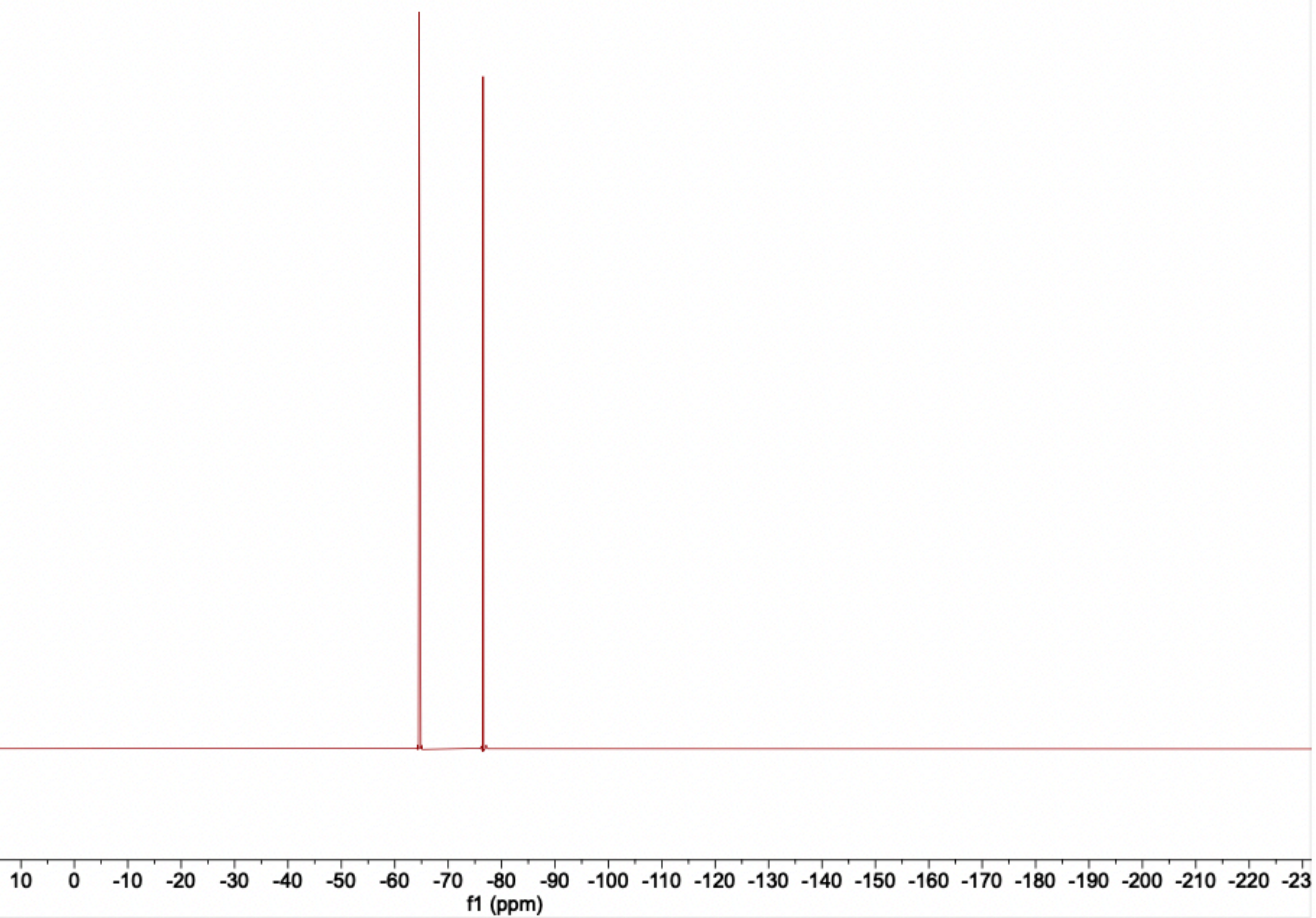

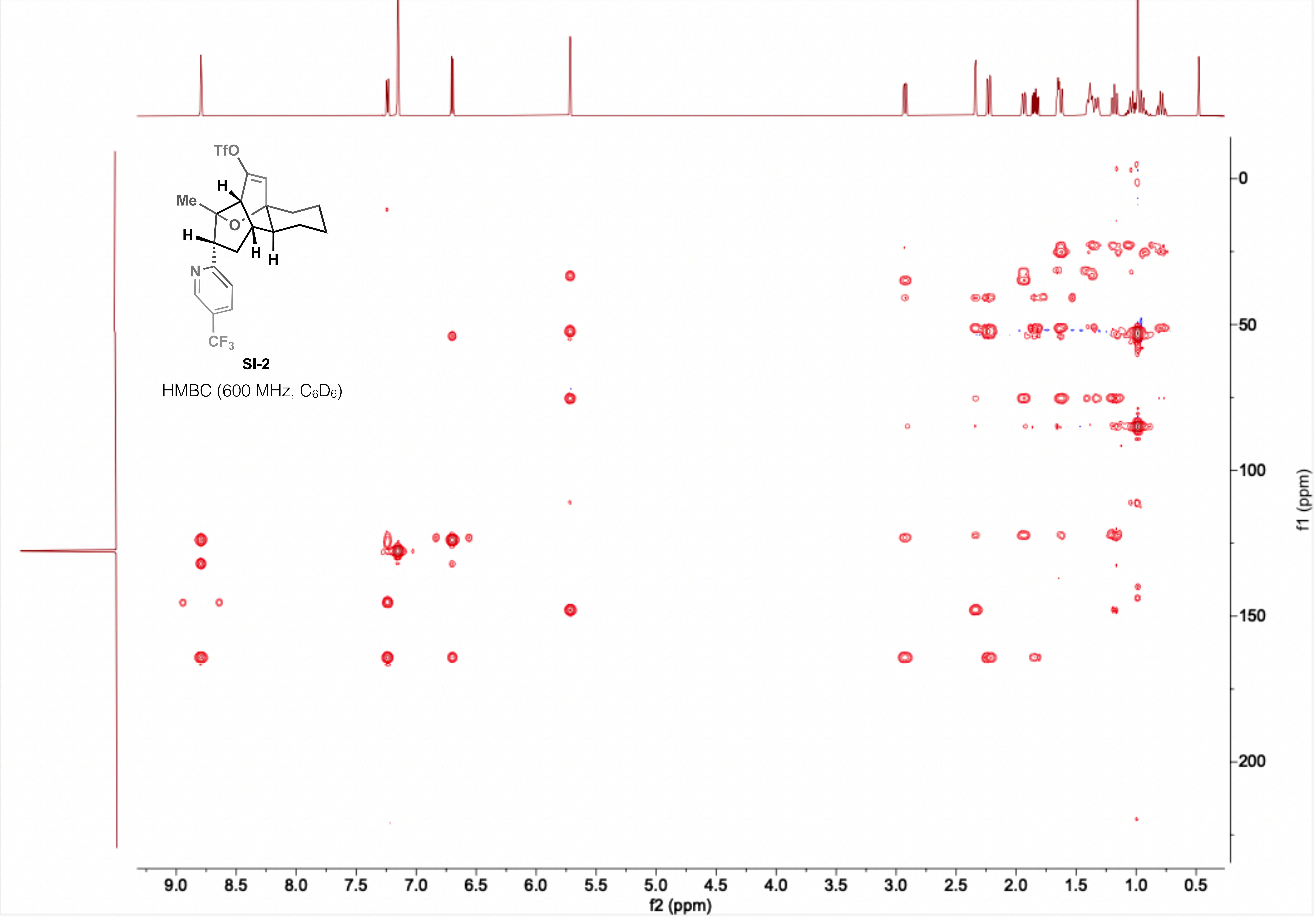
TfO
Me
H
O
N
CF_3
SI-2
HMBC (600 MHz, C_6D_6)
f2 (ppm)
f1 (ppm)

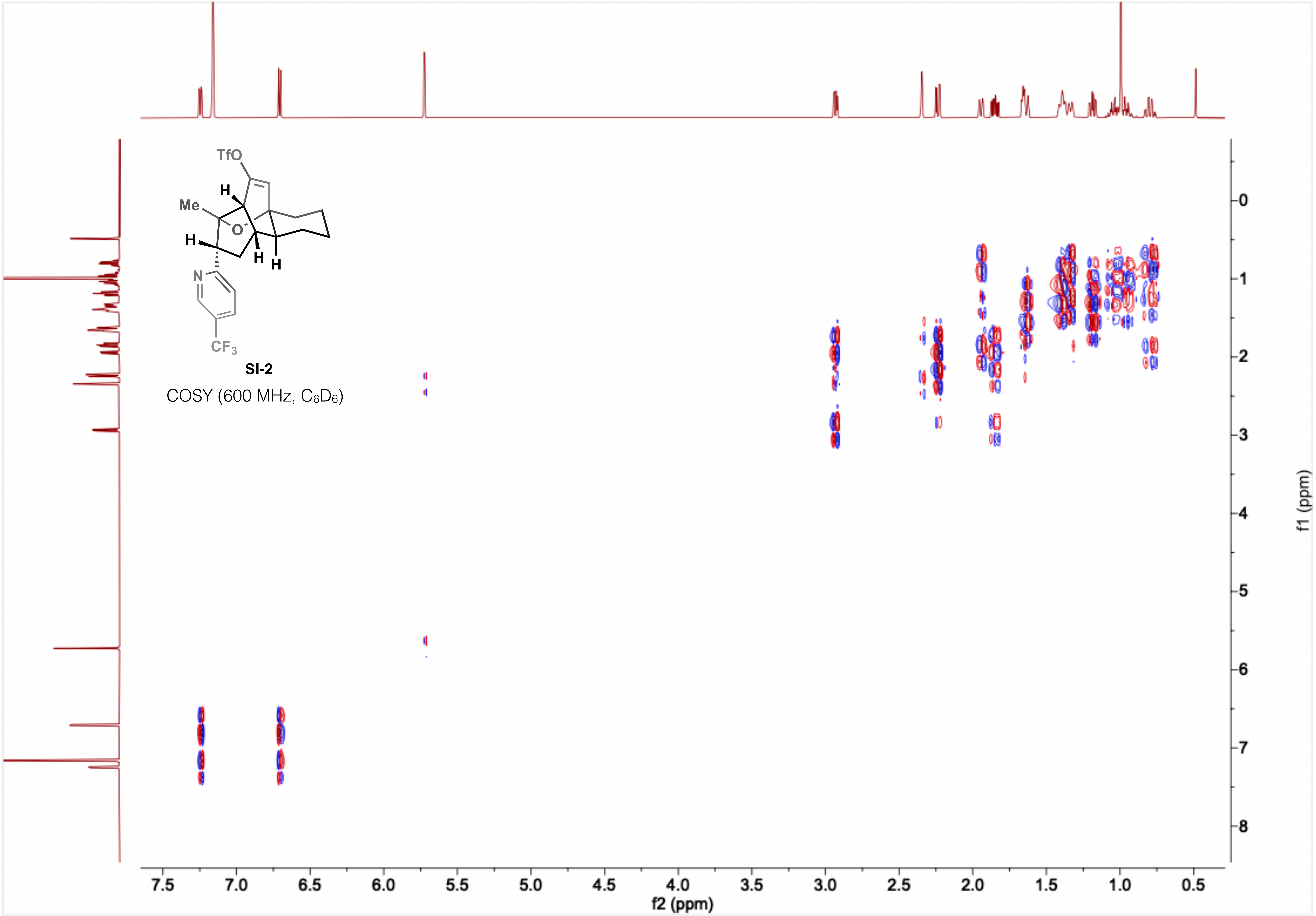
TfO
Me
H
O
H
H
H
N
CF3
SI-2
COSY (600 MHz, C6D6)
f2 (ppm)
f1 (ppm)

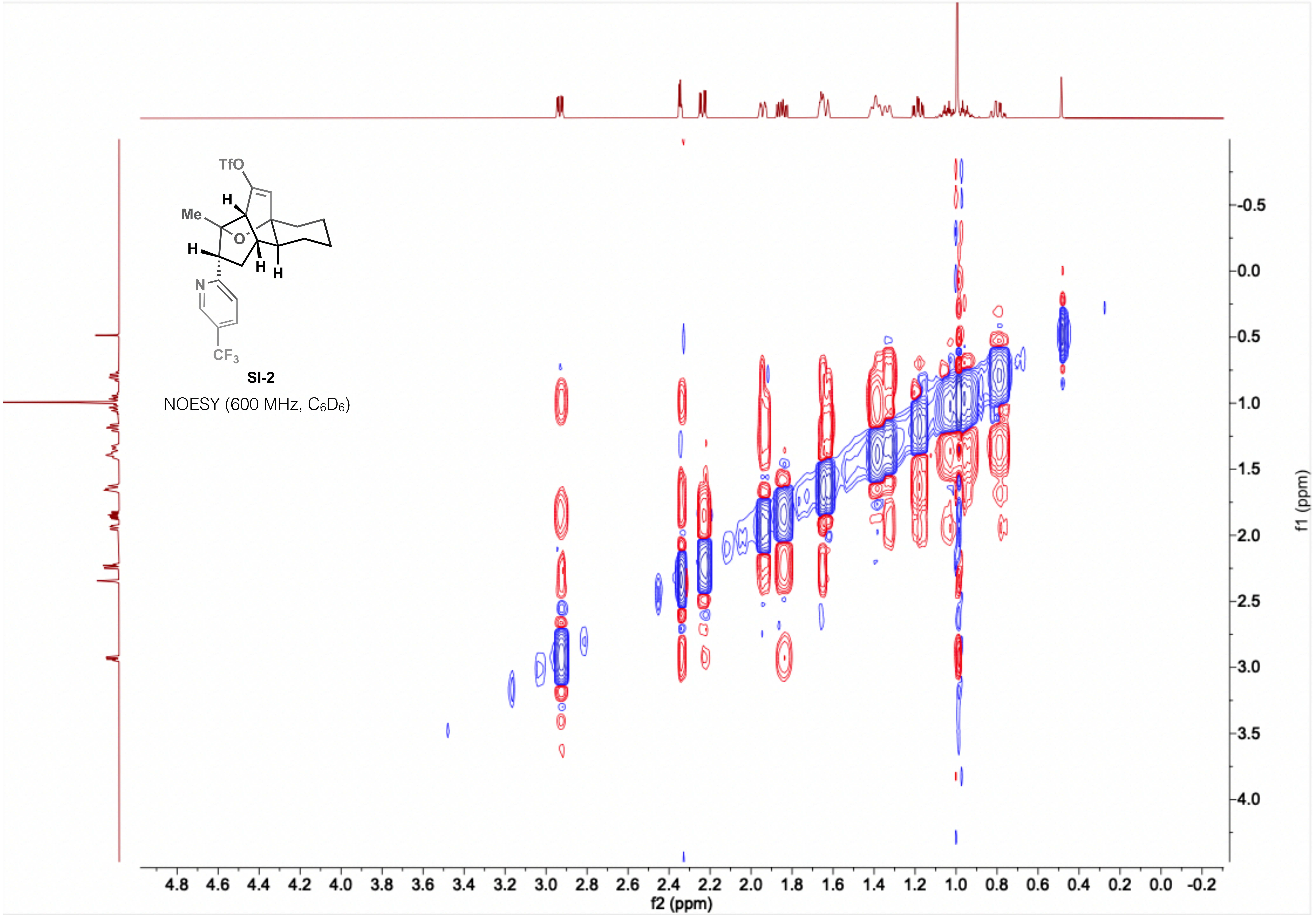

SI-2

NOESY (600 MHz, C_6D_6)

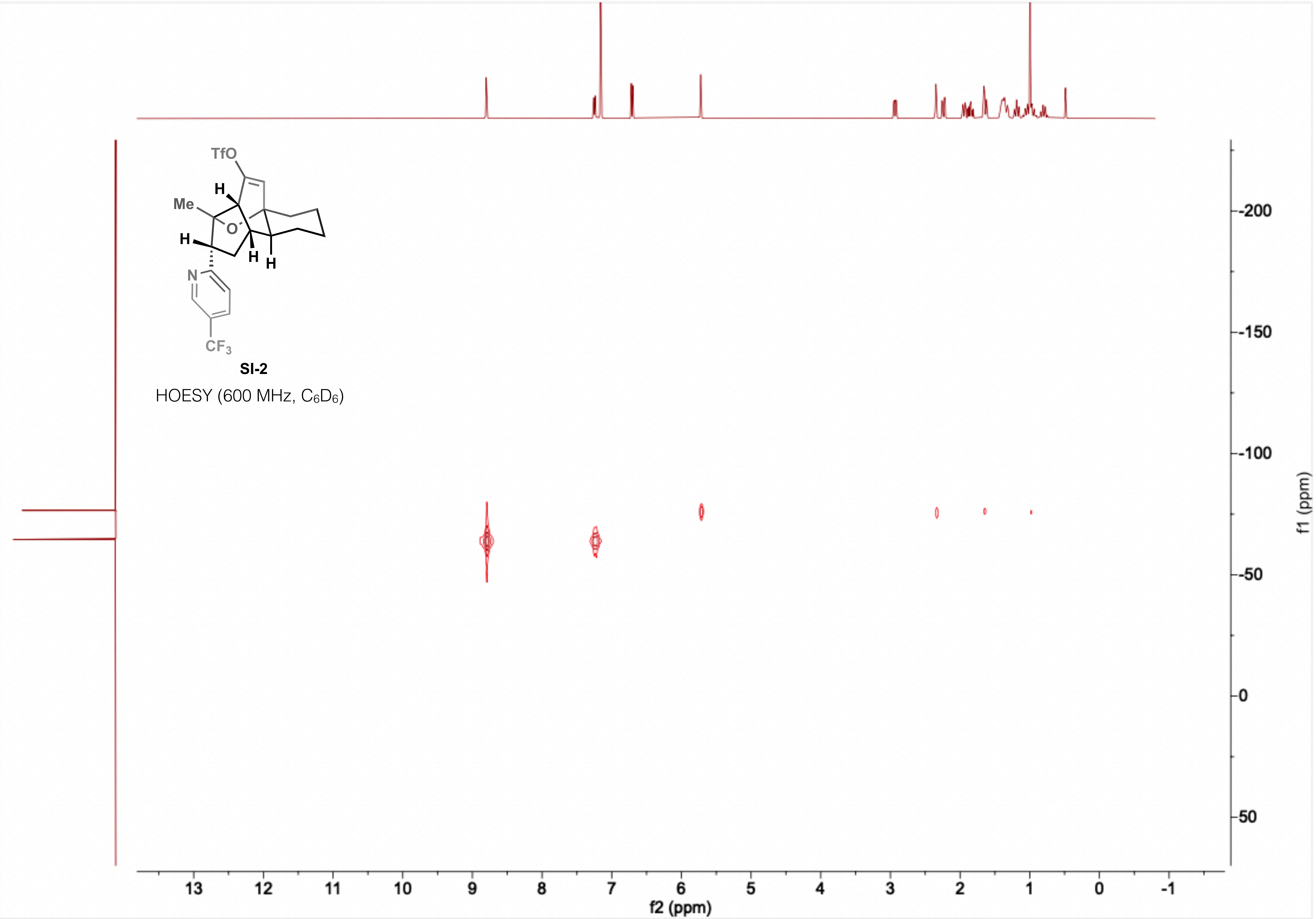

SI-2

HOESY (600 MHz, C_6D_6)

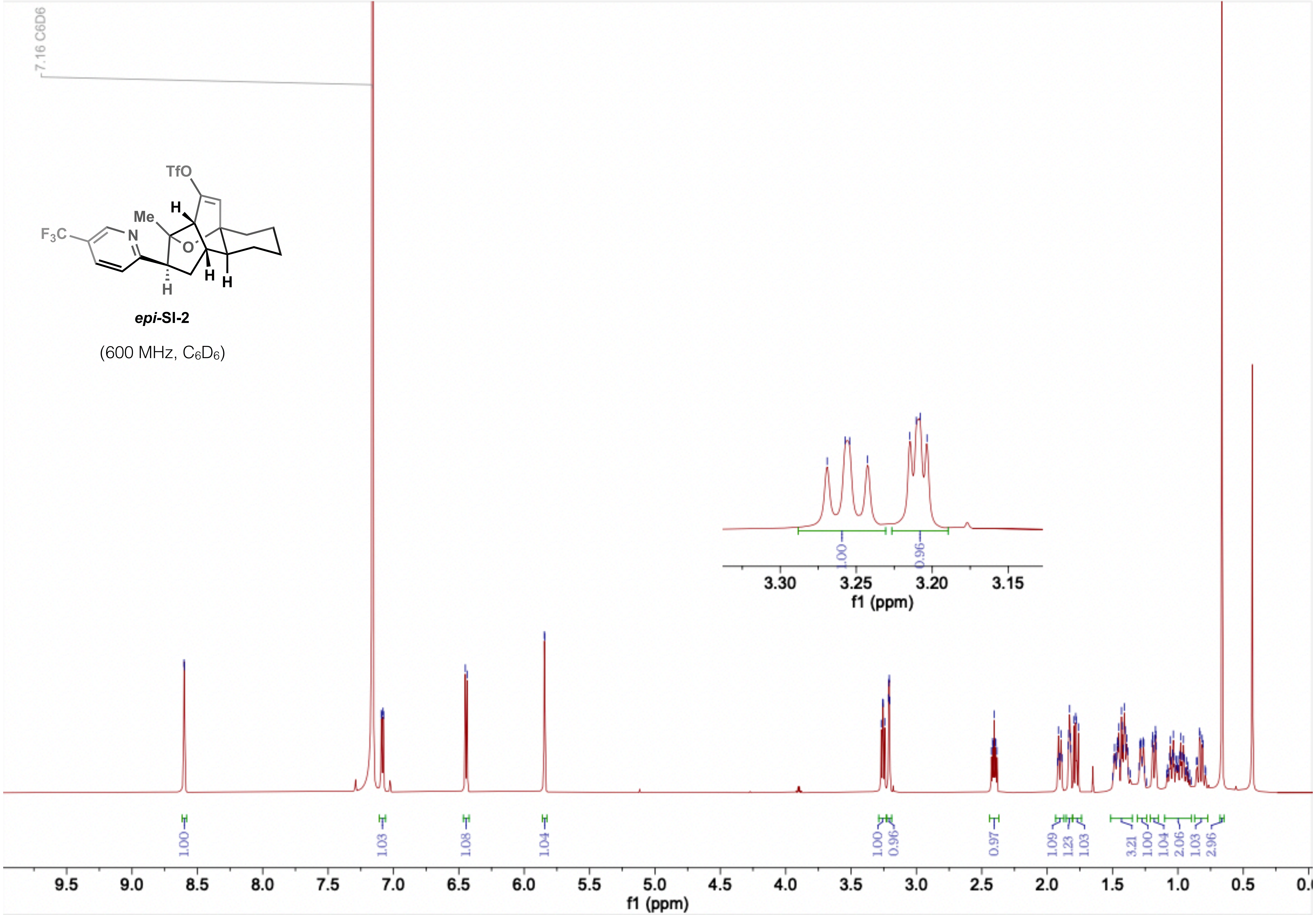

7.16 C6D6
TfO
Me
H
F3C
N
O
H
H
H
epi-SI-2
(600 MHz, C6D6)
1.00
0.96
3.30
3.25
3.20
3.15
f1 (ppm)
1.00
1.03
1.08
1.04
1.00
0.96
0.97
1.09
1.23
1.03
3.21
1.00
1.04
2.06
1.03
2.96
9.5
9.0
8.5
8.0
7.5
7.0
6.5
6.0
5.5
5.0
4.5
4.0
3.5
3.0
2.5
2.0
1.5
1.0
0.5
f1 (ppm)

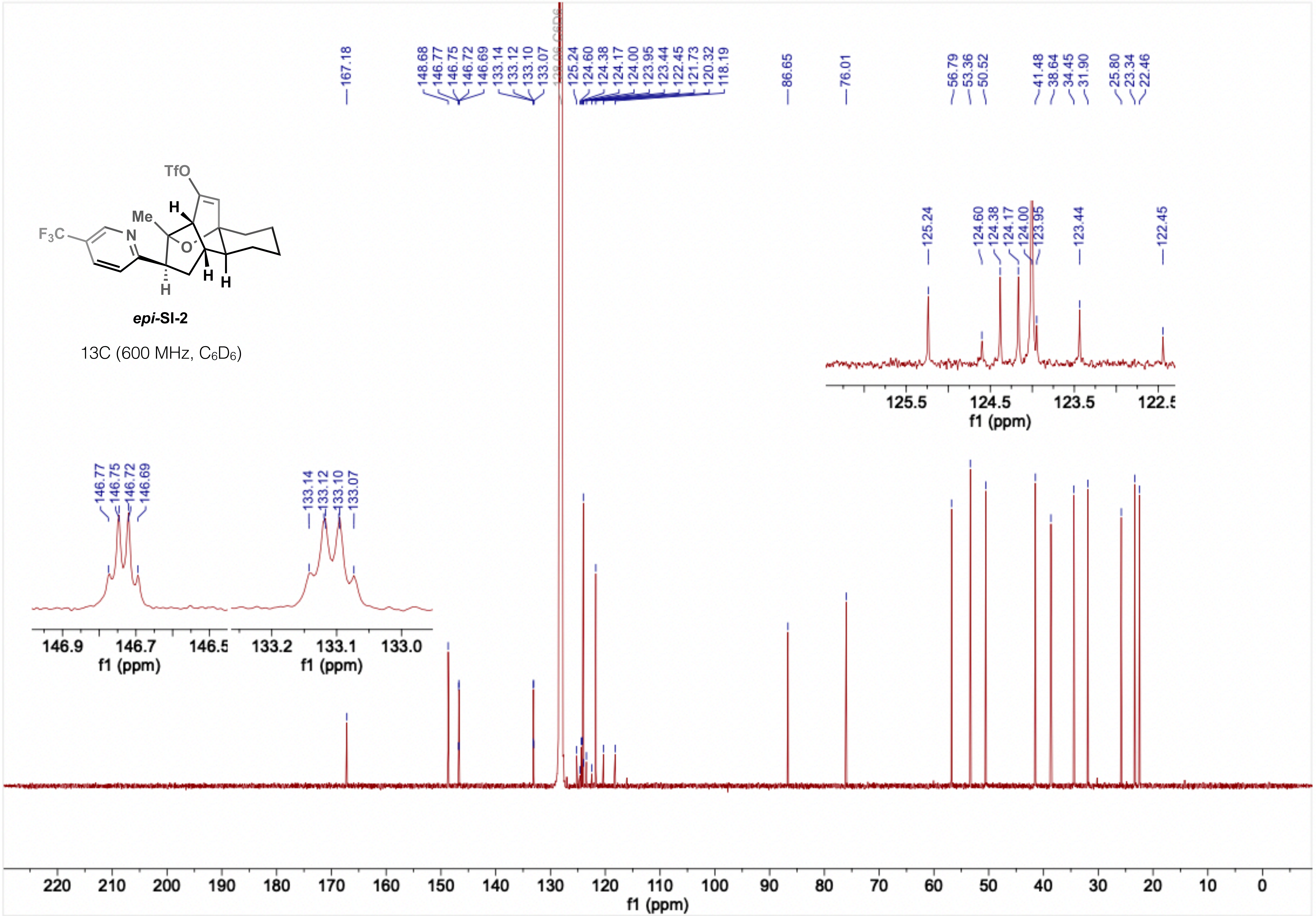

TfO
Me
H
F3C
N
O
H
H
H
epi-SI-2
13C (600 MHz, C6D6)
167.18
148.68
146.77
146.75
146.72
146.69
133.14
133.12
133.10
133.07
125.24
124.60
124.38
124.17
124.00
123.95
123.44
122.45
121.73
120.32
118.19
86.65
76.01
56.79
53.36
50.52
41.48
38.64
34.45
31.90
25.80
23.34
22.46
f1 (ppm)

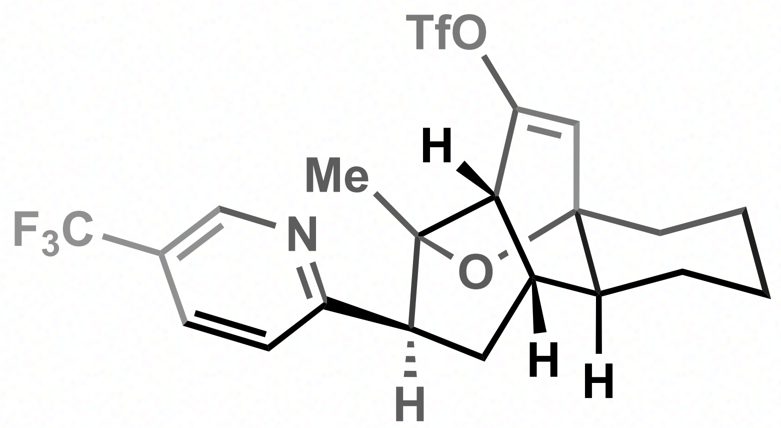

***epi*-SI-2**

19F (600 MHz, C_6D_6)

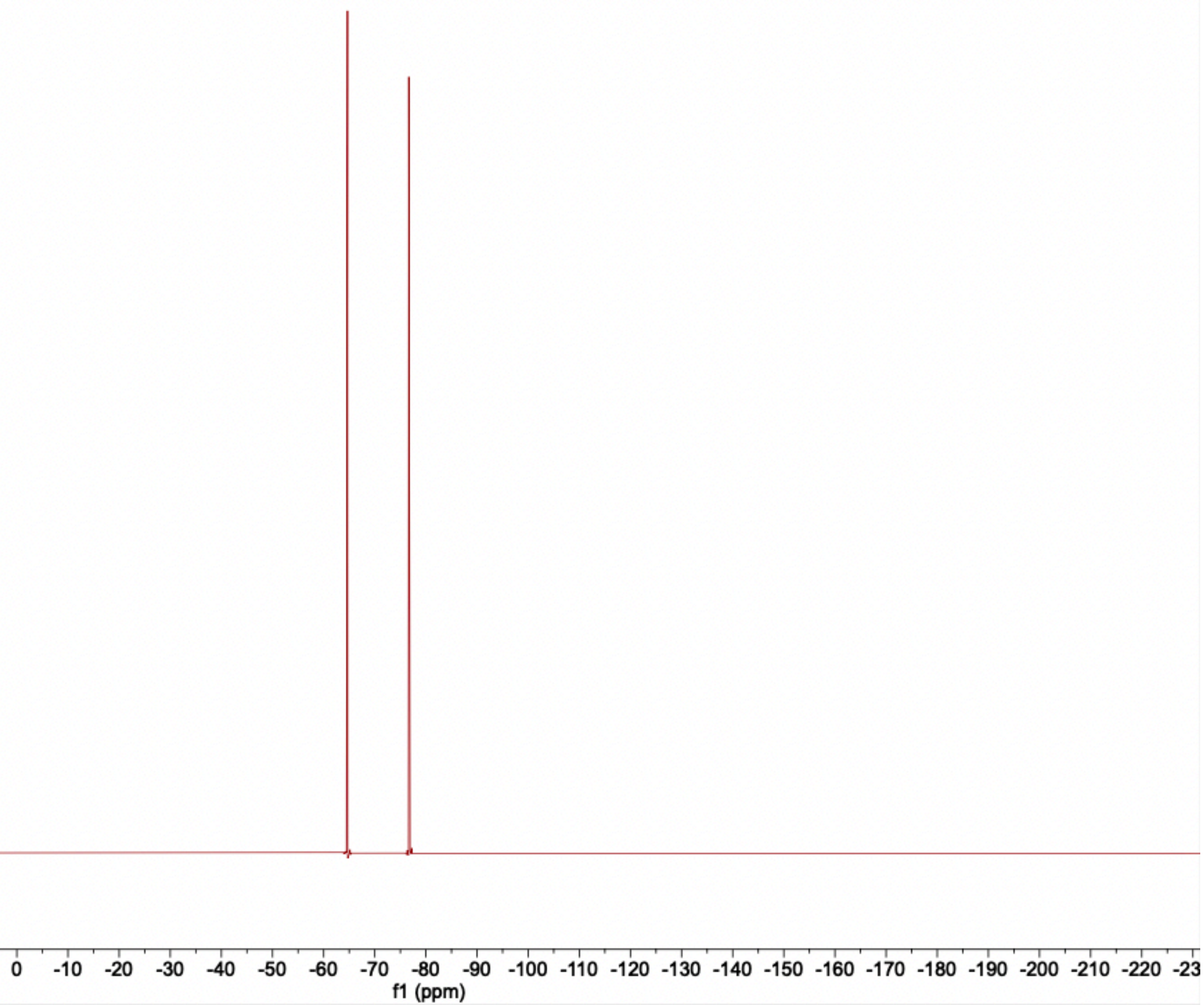

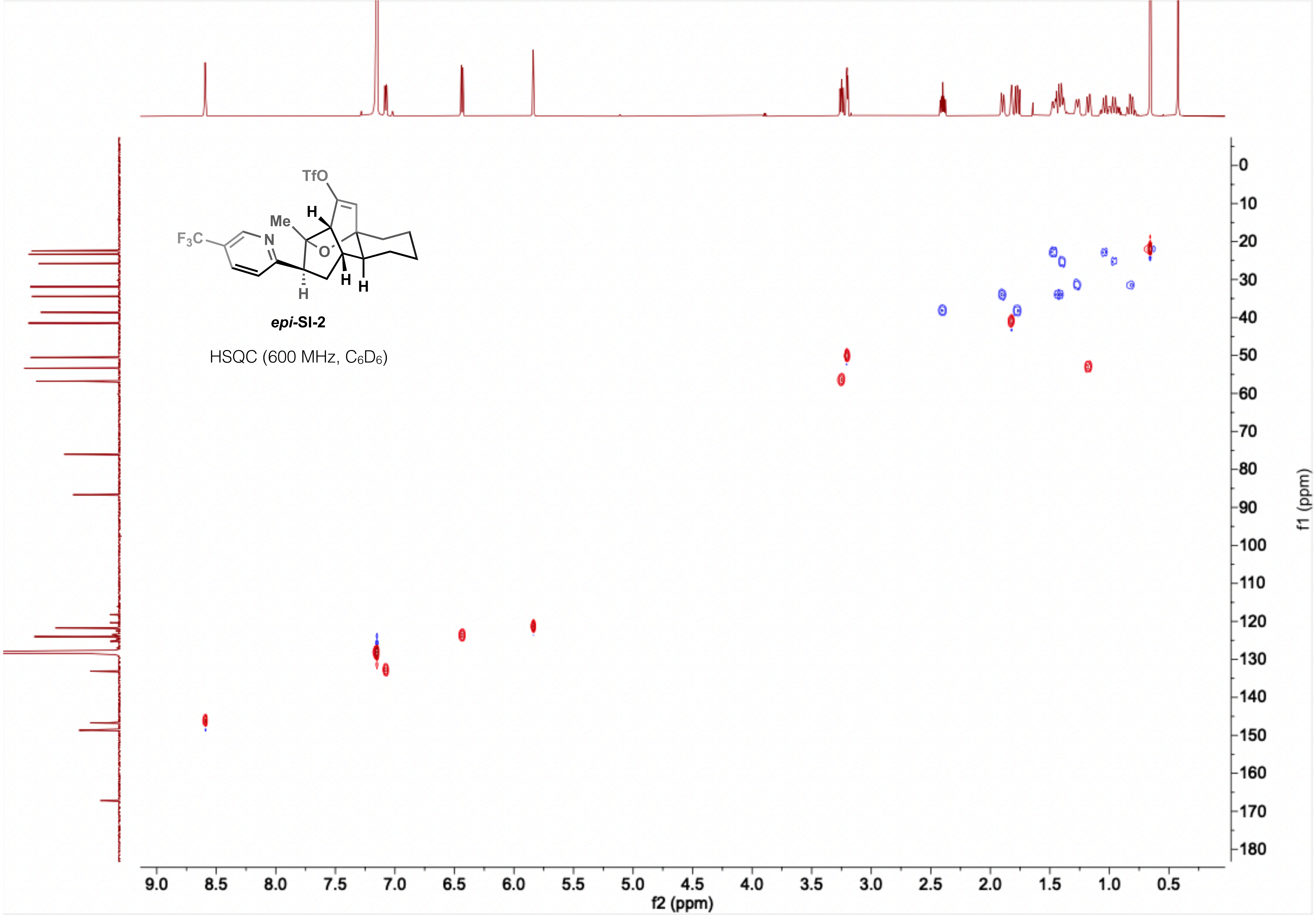

TfO
Me
H
F3C
N
O
H
H
H
epi-SI-2
HSQC (600 MHz, C6D6)
f2 (ppm)
f1 (ppm)

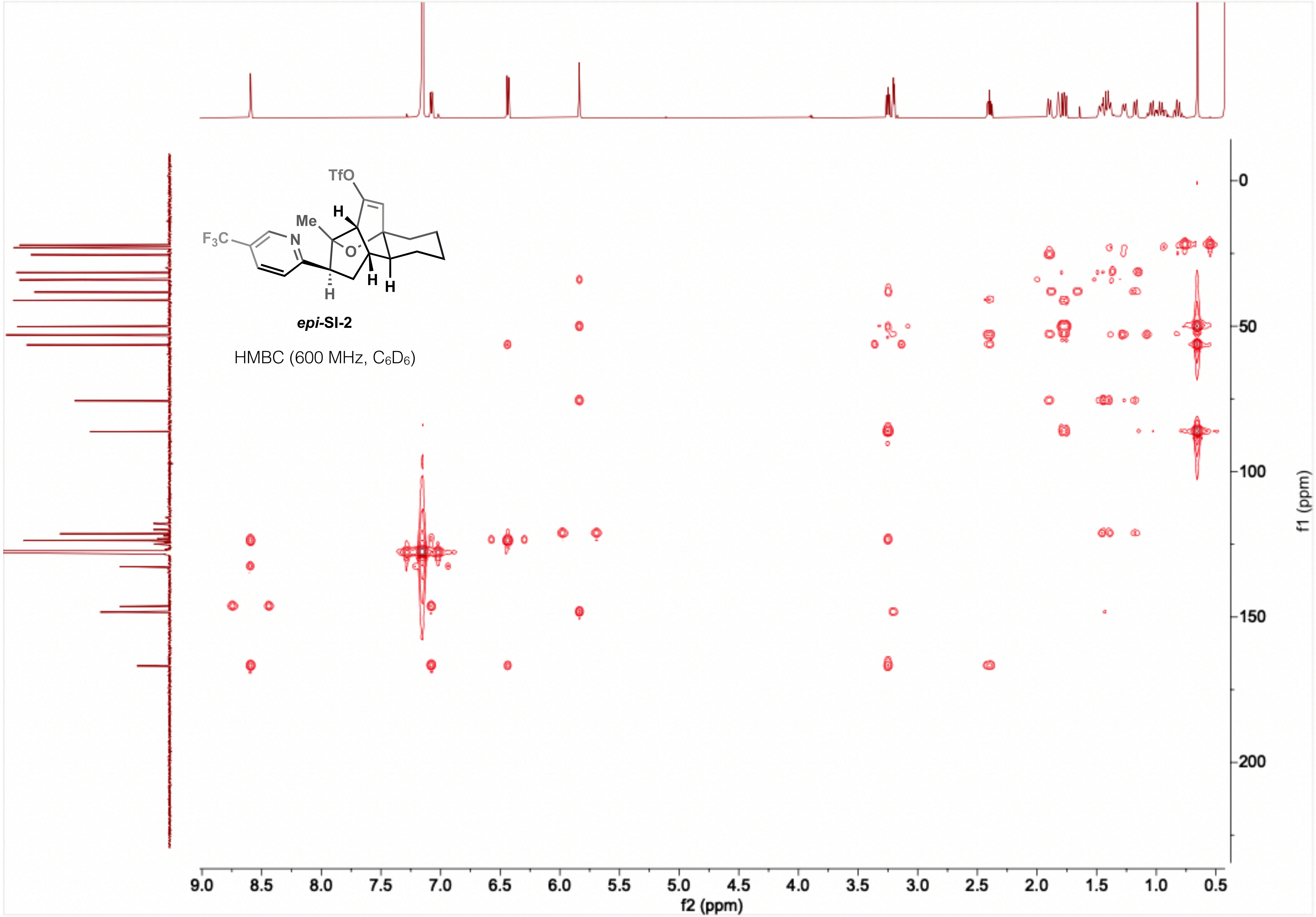

TfO
Me
N
F3C
O
H
H
H
H
epi-SI-2
HMBC (600 MHz, C6D6)
f1 (ppm)
f2 (ppm)
0
50
100
150
200
9.0
8.5
8.0
7.5
7.0
6.5
6.0
5.5
5.0
4.5
4.0
3.5
3.0
2.5
2.0
1.5
1.0
0.5

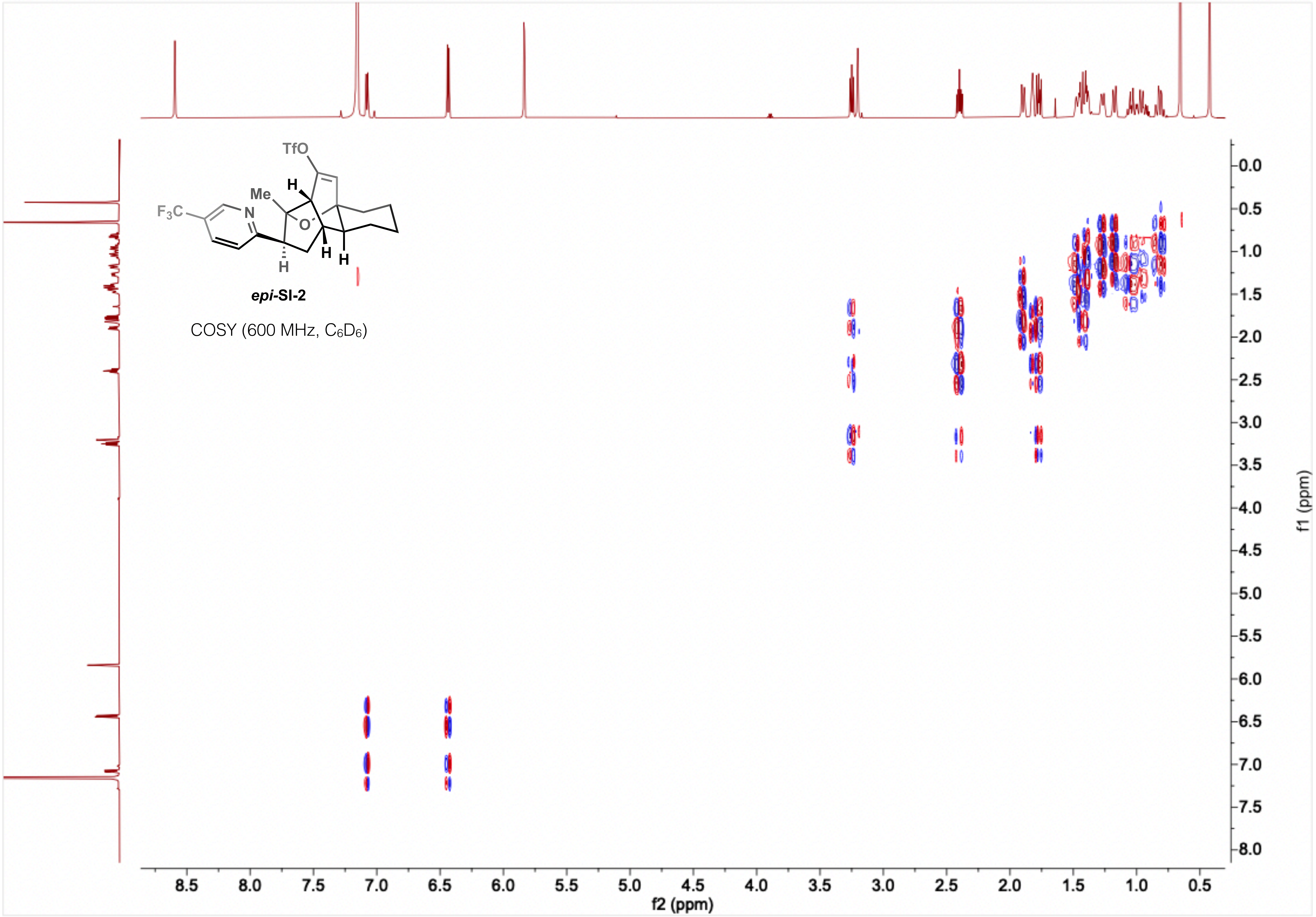
TfO
Me
H
F3C
N
O
H
H
H
epi-SI-2
COSY (600 MHz, C6D6)
f2 (ppm)
f1 (ppm)

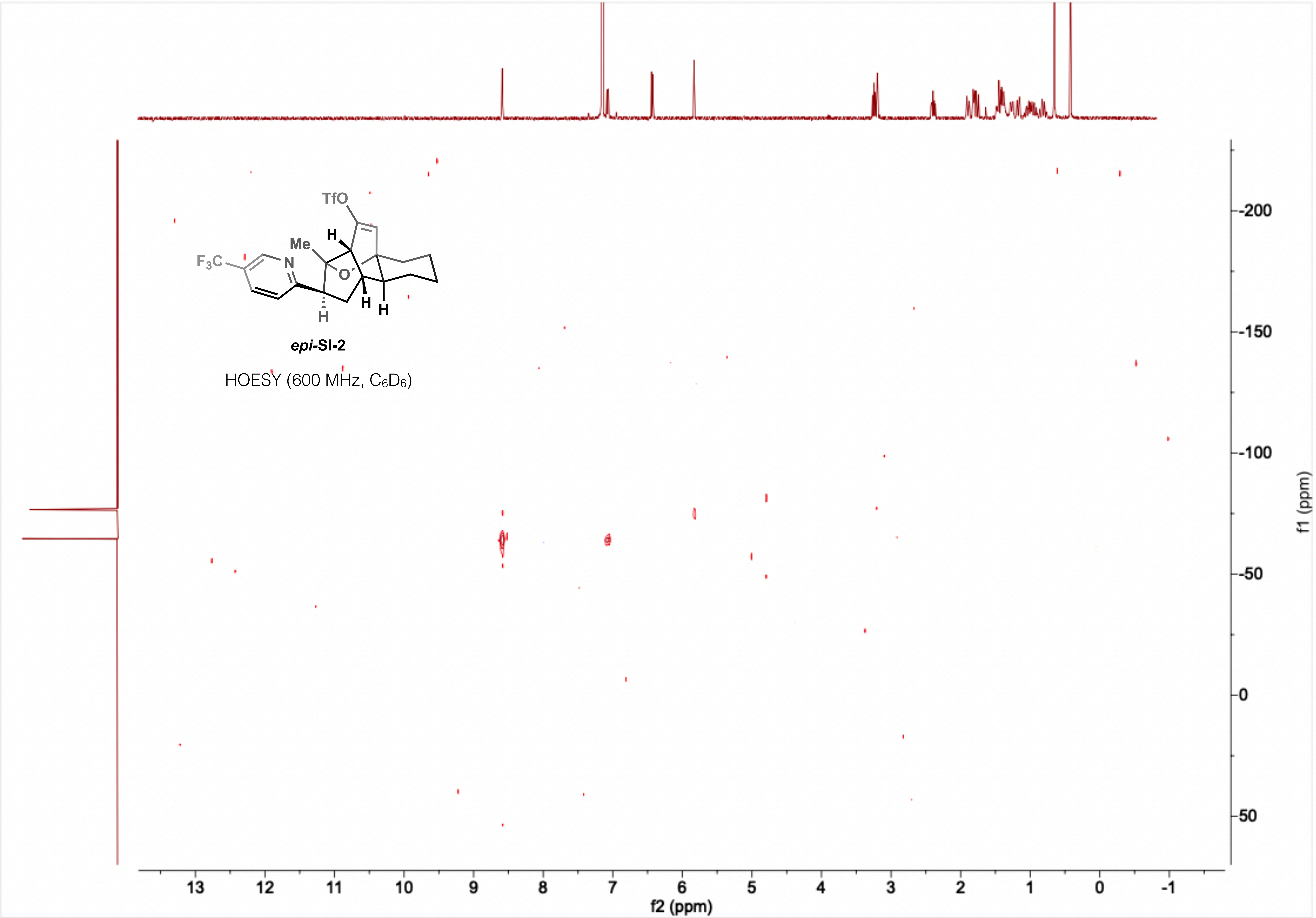
TfO
Me
H
F3C
N
O
H
H
H
epi-SI-2
HOESY (600 MHz, C6D6)
-200
-150
-100
-50
0
50
f1 (ppm)
13
12
11
10
9
8
7
6
5
4
3
2
1
0
-1
f2 (ppm)

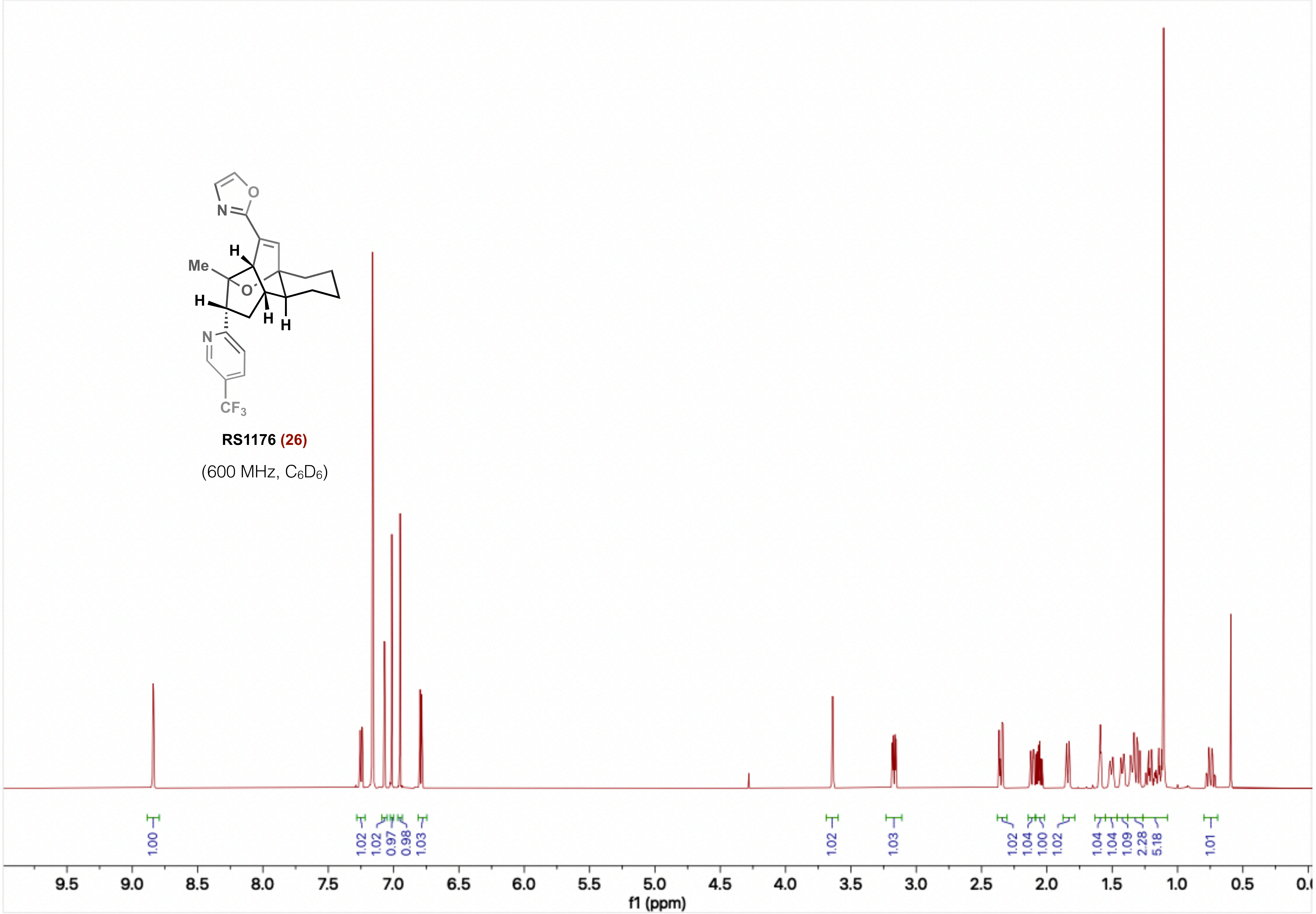

RS1176 (26)
(600 MHz, C6D6)
Me
O
N
CF3
1.00
1.02
1.02
0.97
0.98
1.03
1.02
1.03
1.02
1.04
1.00
1.02
1.04
1.04
1.09
2.28
5.18
1.01
9.5 9.0 8.5 8.0 7.5 7.0 6.5 6.0 5.5 5.0 4.5 4.0 3.5 3.0 2.5 2.0 1.5 1.0 0.5
f1 (ppm)

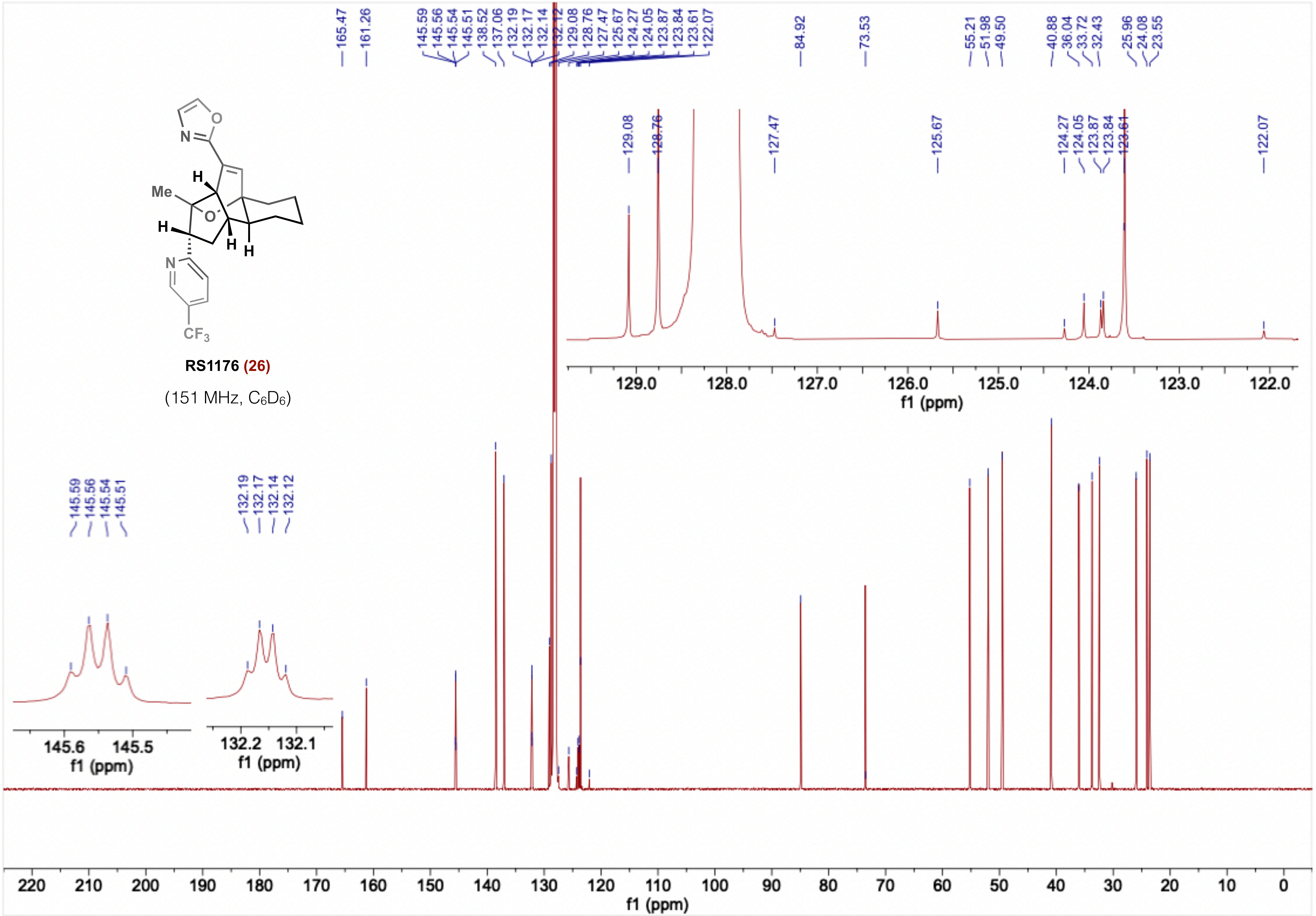
RS1176 (26)
(151 MHz, C6D6)
165.47
161.26
145.59
145.56
145.54
145.51
138.52
137.06
132.19
132.17
132.14
132.12
129.08
128.76
127.47
125.67
124.27
124.05
123.87
123.84
123.61
122.07
84.92
73.53
55.21
51.98
49.50
40.88
36.04
33.72
32.43
25.96
24.08
23.55
f1 (ppm)

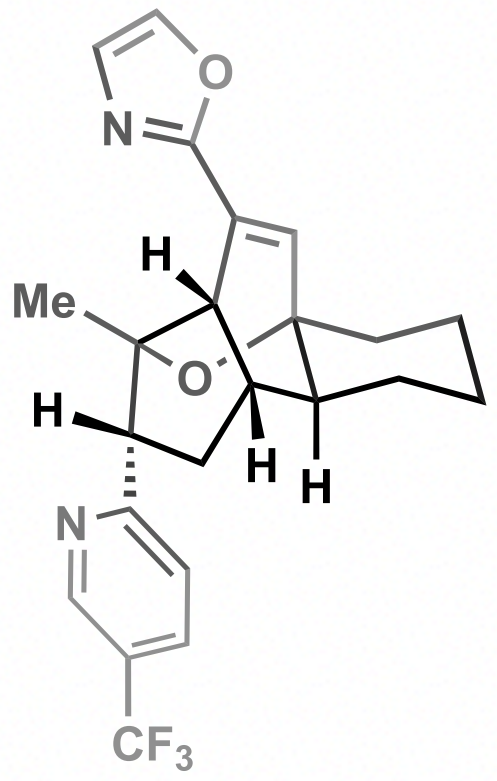

RS1176 (26)

19F (376 MHz, C_6D_6)

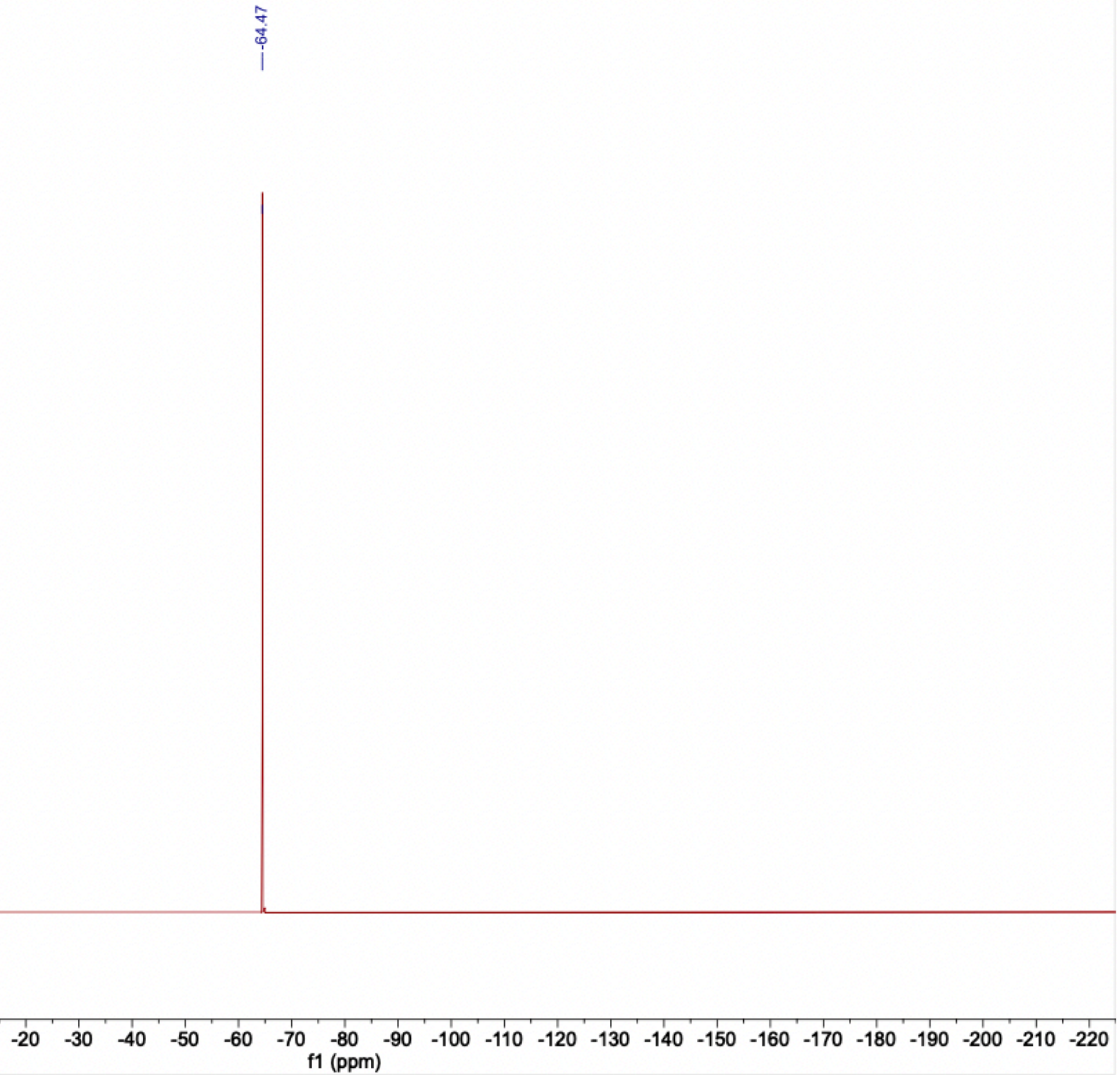

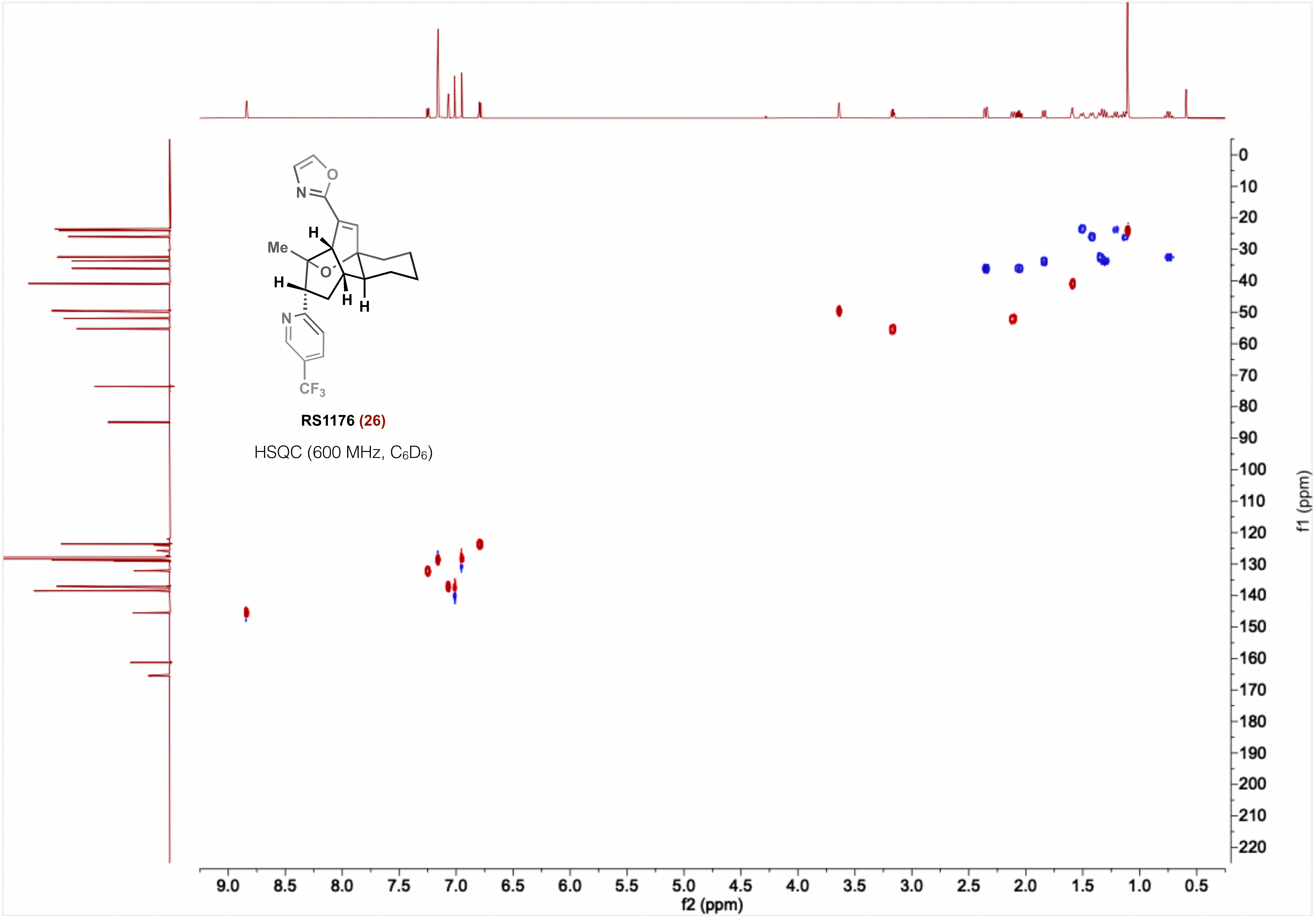

Me
O
N
H
H
H
H
CF3
RS1176 (26)
HSQC (600 MHz, C6D6)
f2 (ppm)
f1 (ppm)

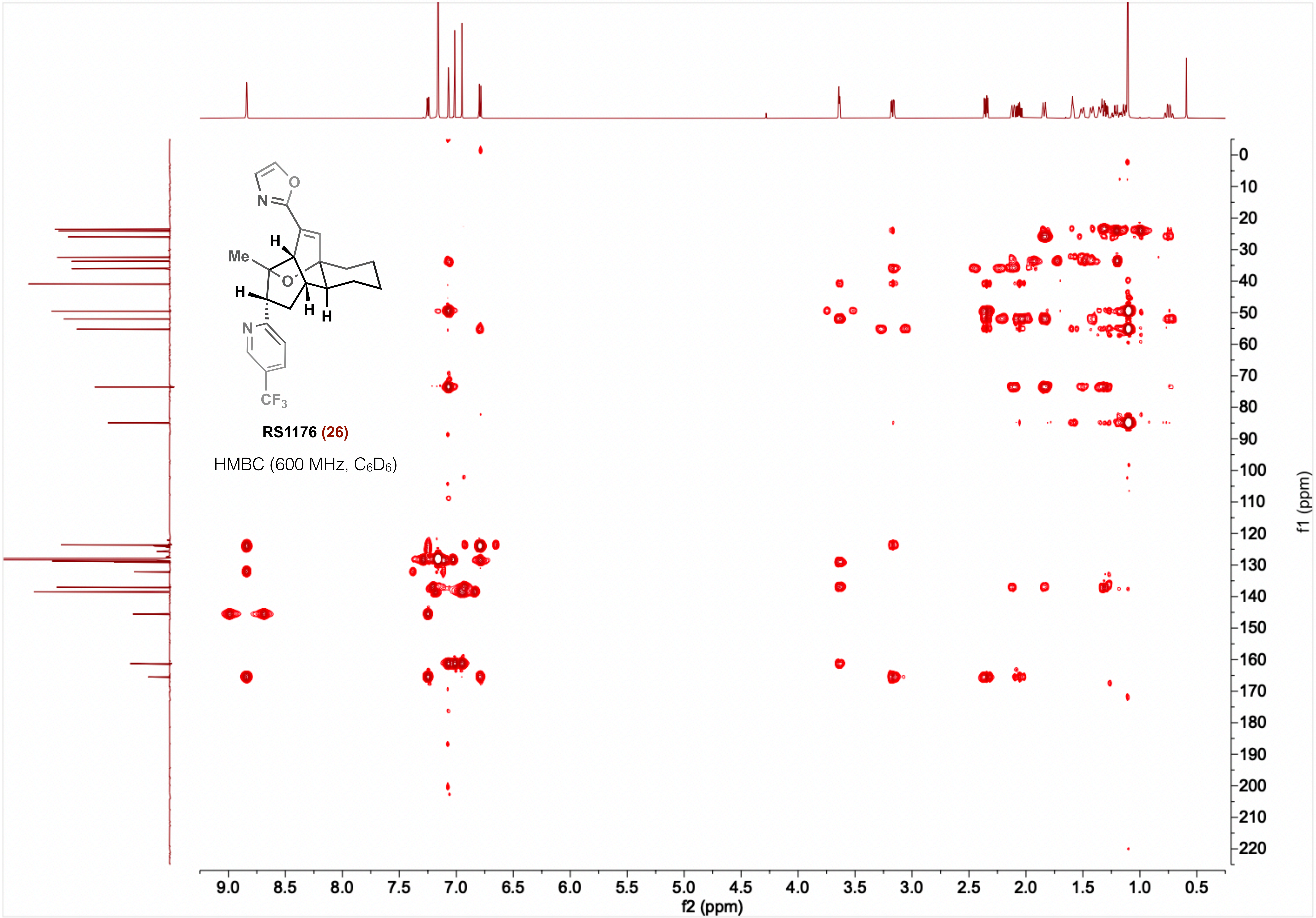

RS1176 **(26)**

HMBC (600 MHz, C_6D_6)

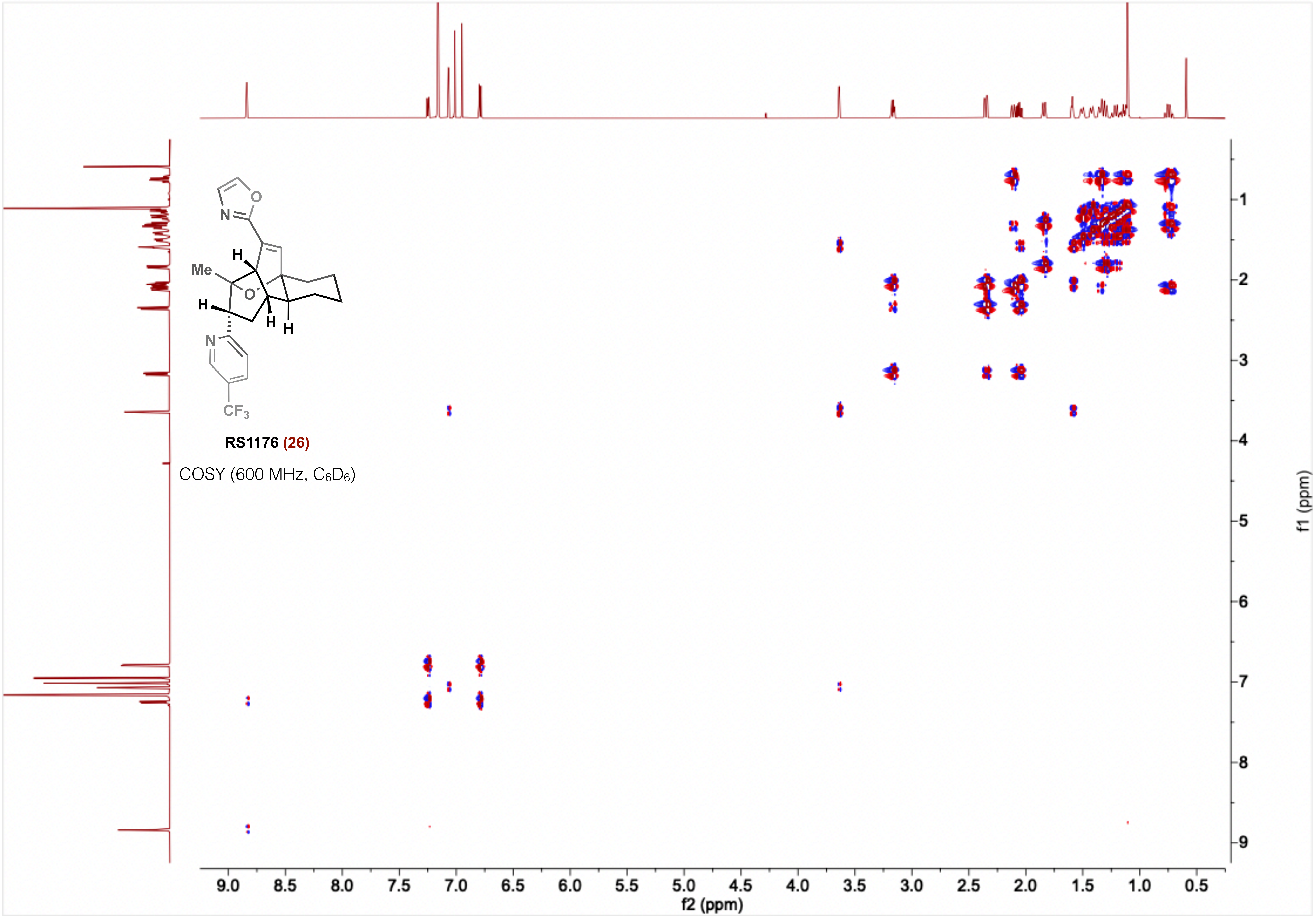

RS1176 (26)

COSY (600 MHz, C_6D_6)

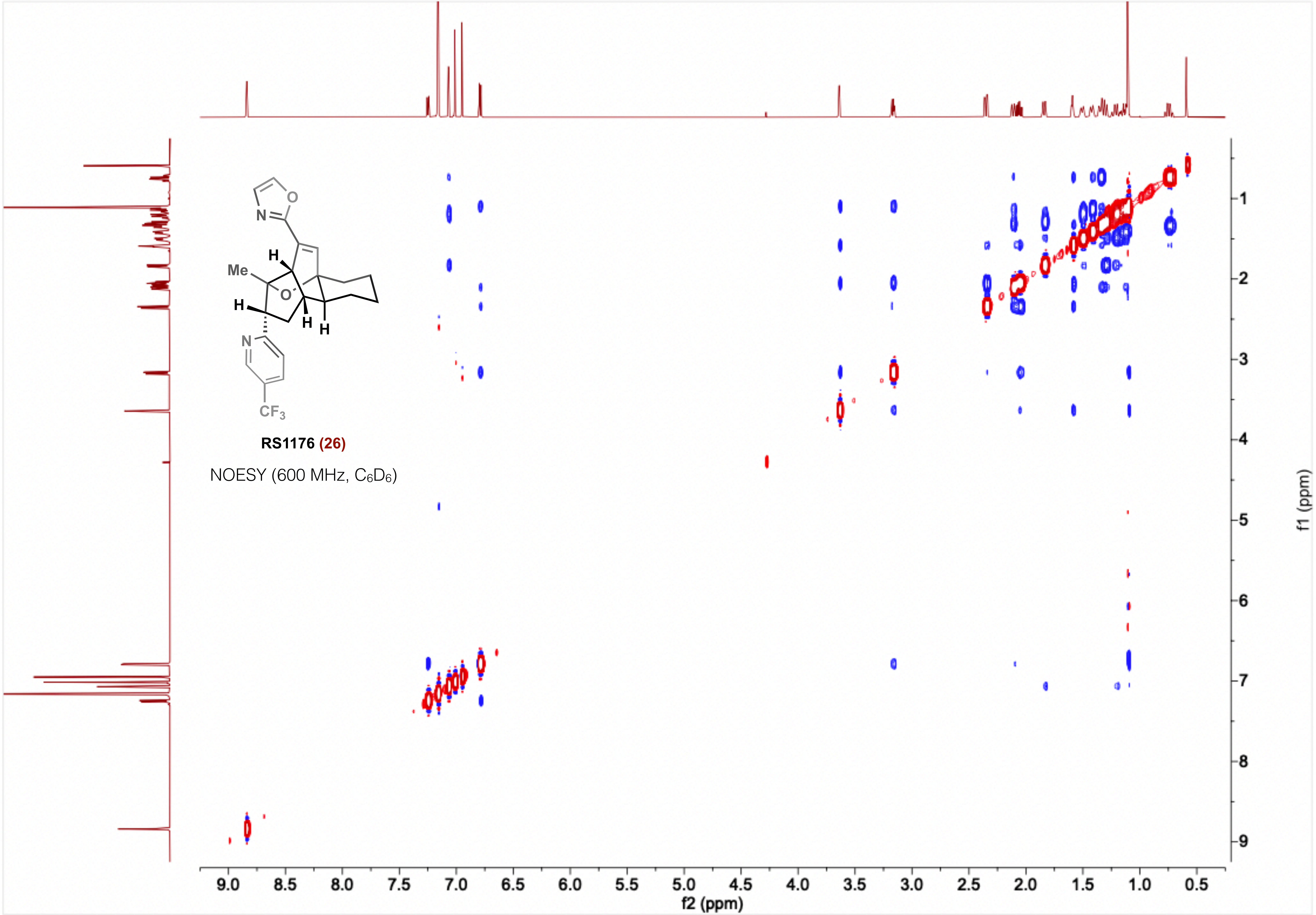

RS1176 **(26)**

NOESY (600 MHz, C_6D_6)

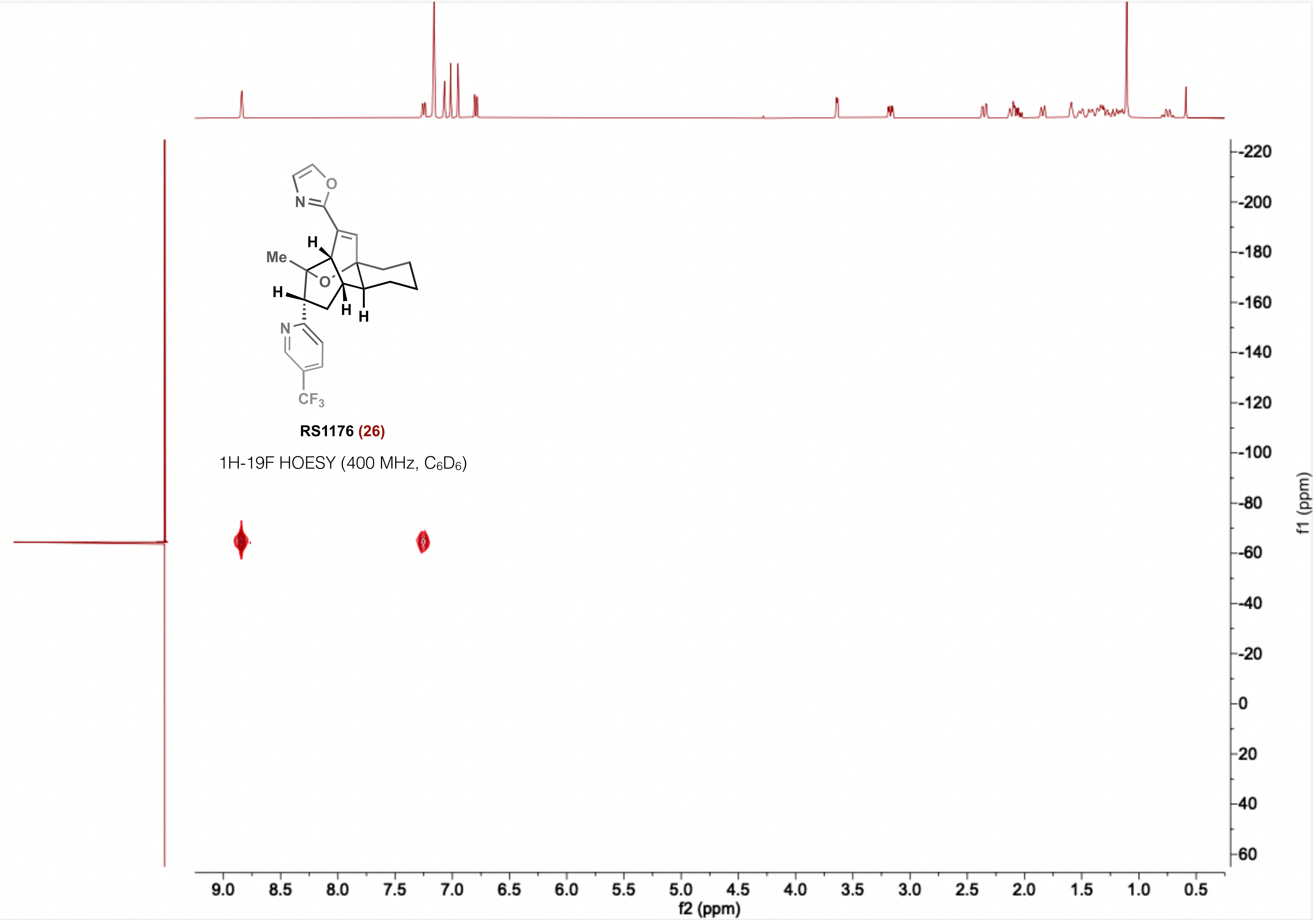

O
N
Me
H
O
H
H
H
N
CF3
RS1176 (26)
1H-19F HOESY (400 MHz, C6D6)
-220
-200
-180
-160
-140
-120
-100
-80
-60
-40
-20
0
20
40
60
f1 (ppm)
9.0
8.5
8.0
7.5
7.0
6.5
6.0
5.5
5.0
4.5
4.0
3.5
3.0
2.5
2.0
1.5
1.0
0.5
f2 (ppm)

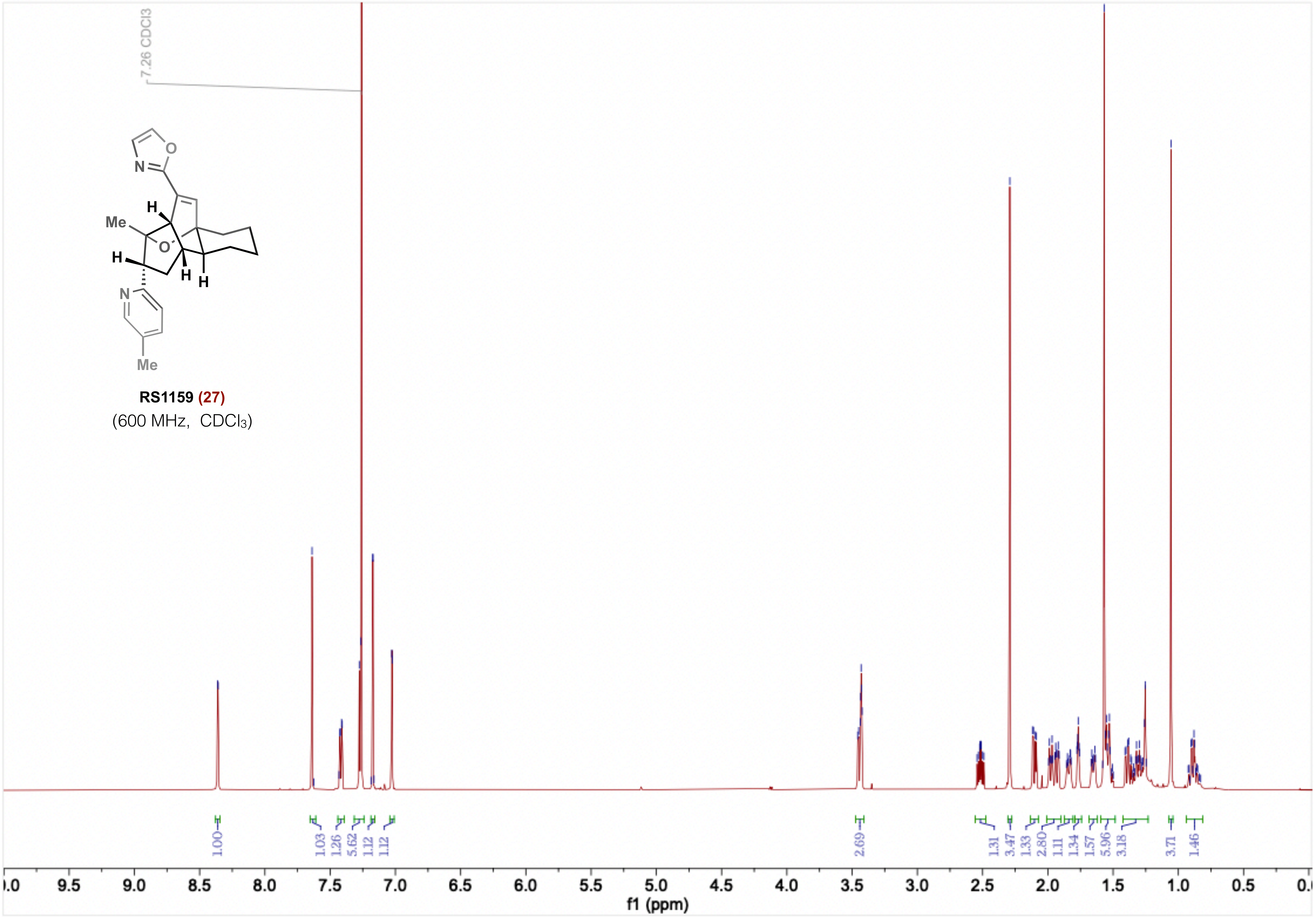
7.26 CDCl3
Me
H
O
H
H
H
N
O
N
Me
Me
RS1159 (27)
(600 MHz, CDCl3)
1.00
1.03
1.26
5.62
1.12
1.12
2.69
1.31
3.47
1.33
2.80
1.11
1.34
1.57
5.96
3.18
3.71
1.46
.0
9.5
9.0
8.5
8.0
7.5
7.0
6.5
6.0
5.5
5.0
4.5
4.0
3.5
3.0
2.5
2.0
1.5
1.0
0.5
0.
f1 (ppm)

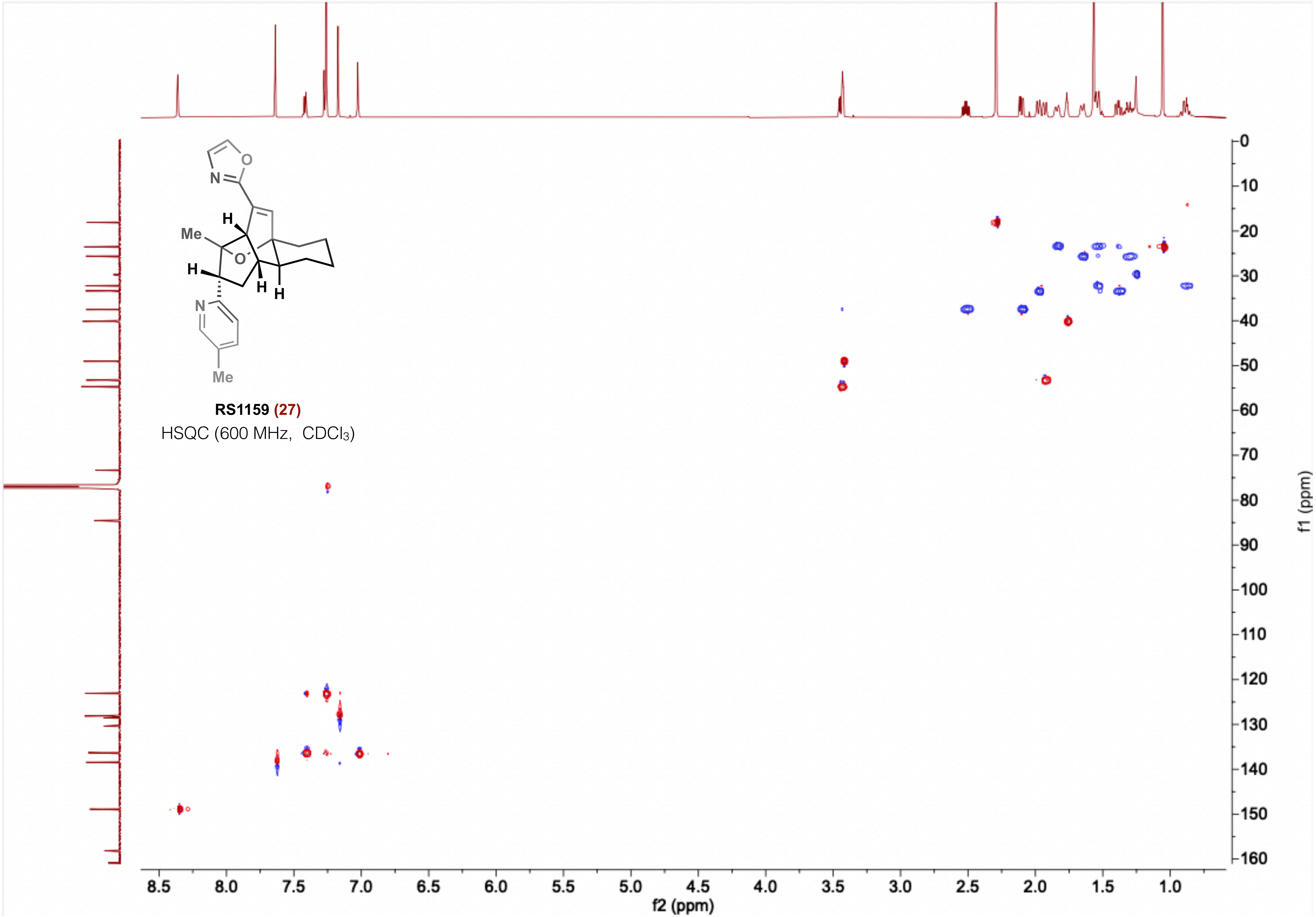

Me
H
O
H
H
H
N
O
N
Me
RS1159 (27)
HSQC (600 MHz, CDCl3)
f2 (ppm)
f1 (ppm)

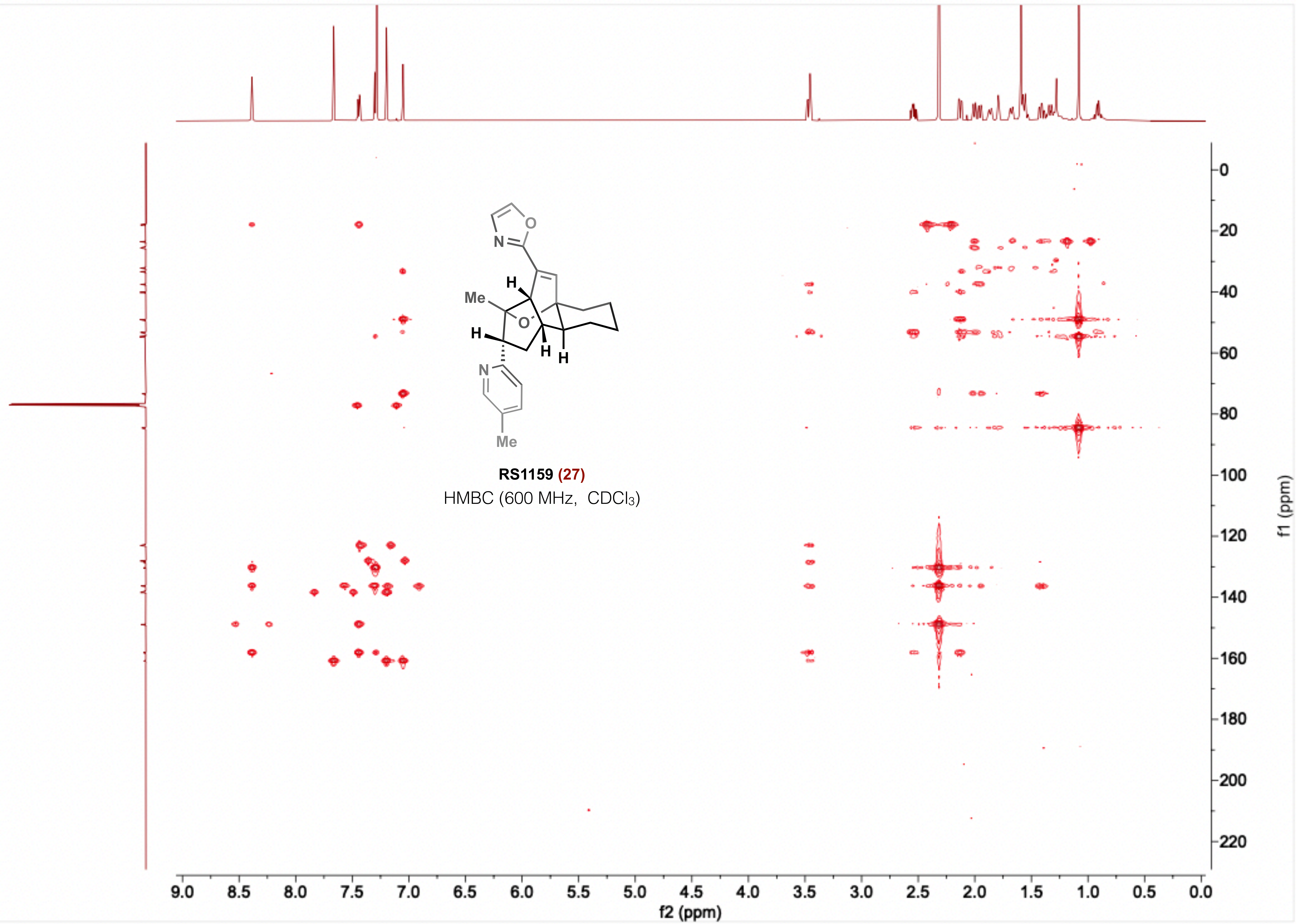
RS1159 (27)
HMBC (600 MHz, CDCl3)
f1 (ppm)
f2 (ppm)
Me
Me
O
N
N
H

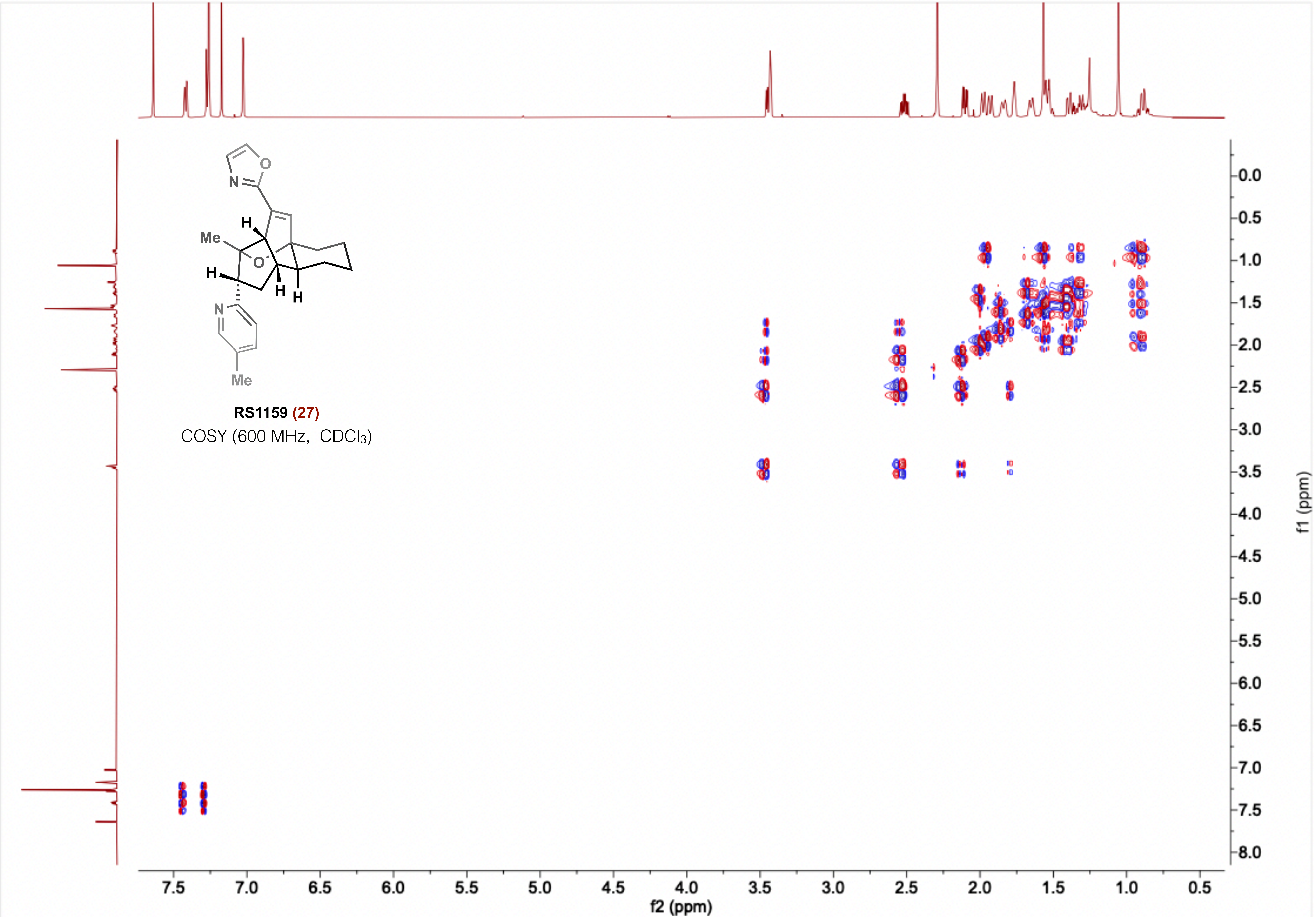
Me
O
H
H
H
H
N
O
N
Me
RS1159 (27)
COSY (600 MHz, CDCl3)
f2 (ppm)
f1 (ppm)

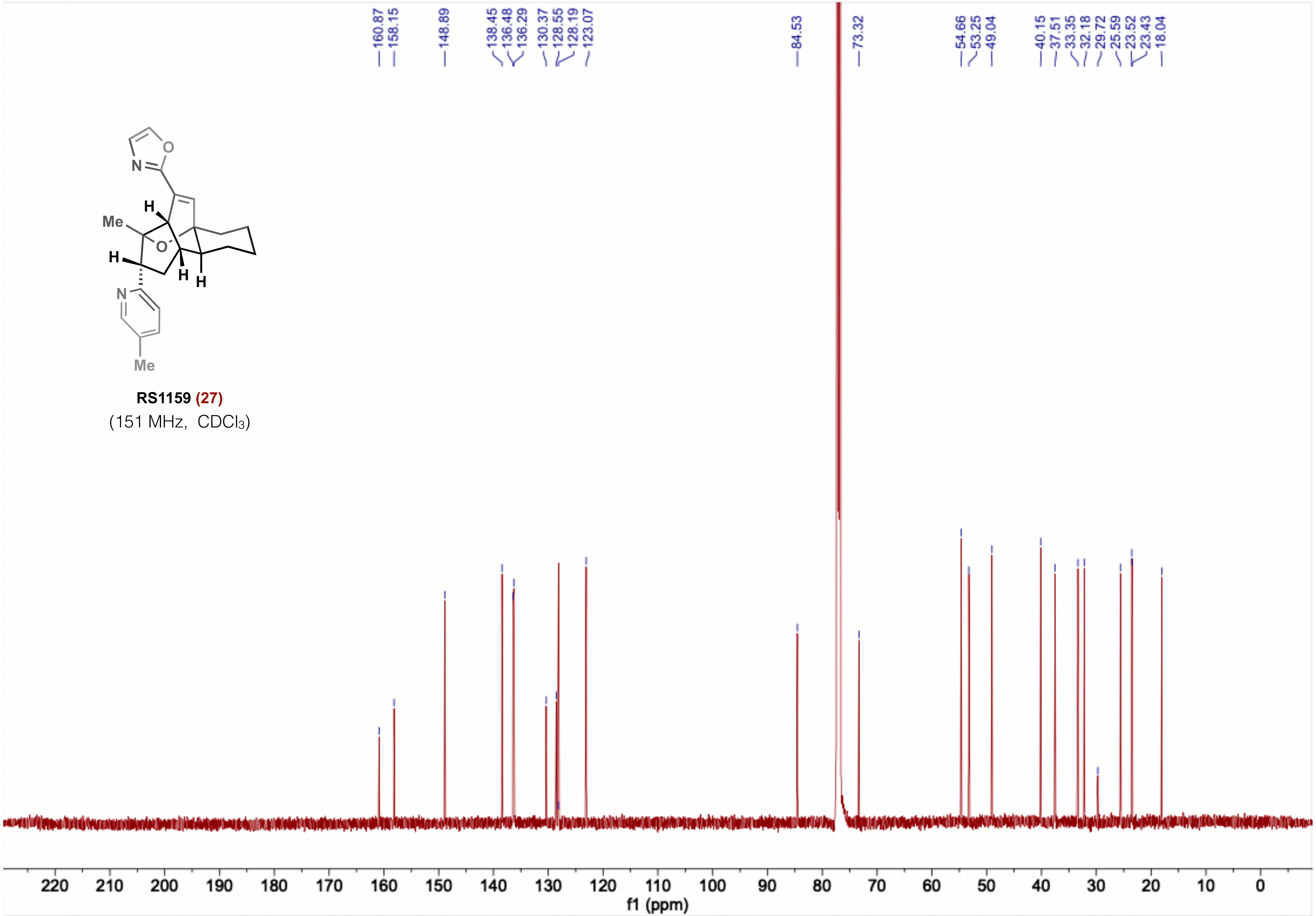
160.87
158.15
148.89
138.45
136.48
136.29
130.37
128.55
128.19
123.07
84.53
73.32
54.66
53.25
49.04
40.15
37.51
33.35
32.18
29.72
25.59
23.52
23.43
18.04
N
O
H
Me
O
H
H
H
N
Me
RS1159 (27)
(151 MHz, CDCl3)
220 210 200 190 180 170 160 150 140 130 120 110 100 90 80 70 60 50 40 30 20 10 0
f1 (ppm)

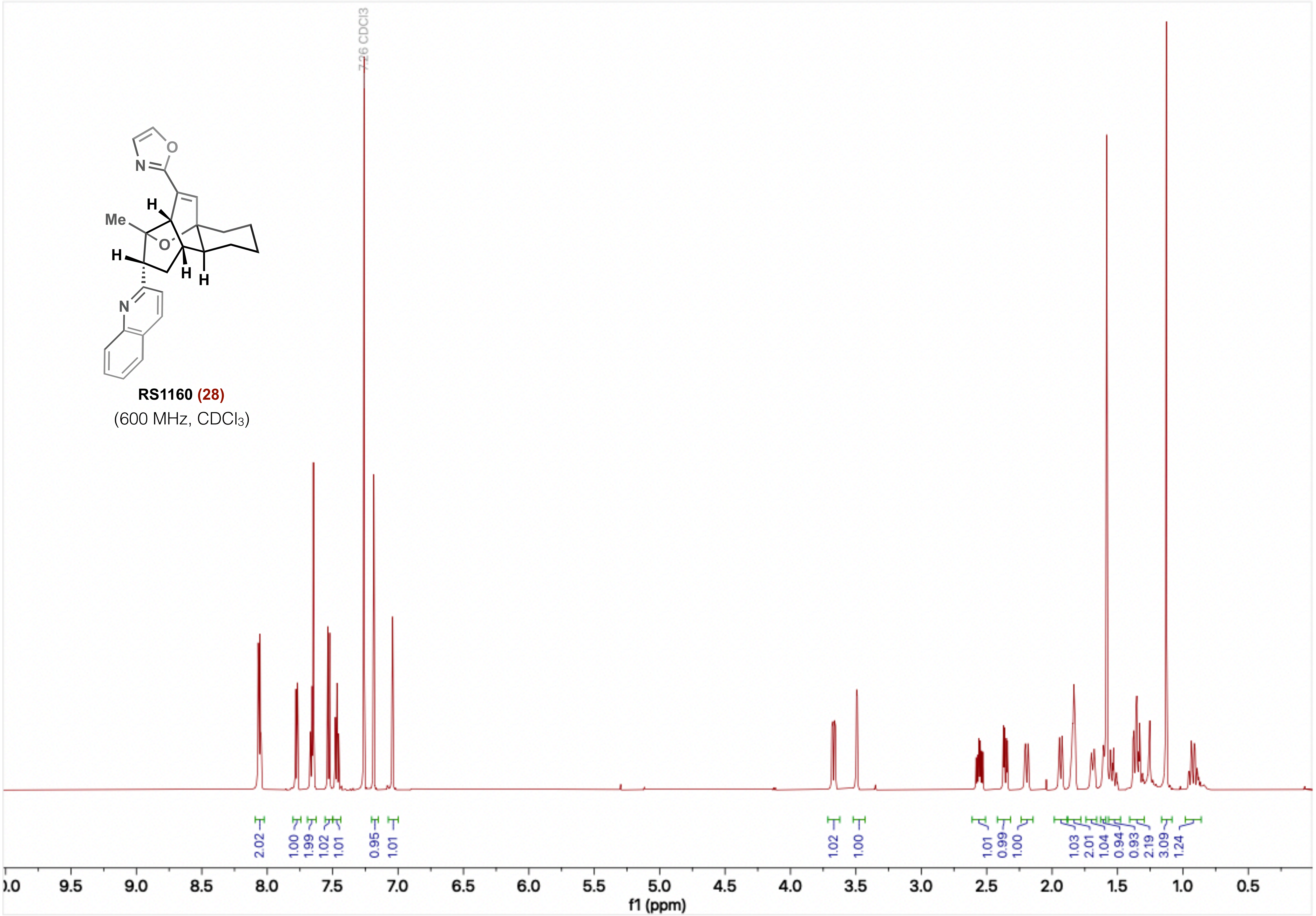
7.26 CDCl3
Me
H
O
N
RS1160 (28)
(600 MHz, CDCl3)
2.02
1.00
1.99
1.02
1.01
0.95
1.01
1.02
1.00
1.01
0.99
1.00
1.03
2.01
1.04
0.94
0.93
2.19
3.09
1.24
9.5
9.0
8.5
8.0
7.5
7.0
6.5
6.0
5.5
5.0
4.5
4.0
3.5
3.0
2.5
2.0
1.5
1.0
0.5
f1 (ppm)

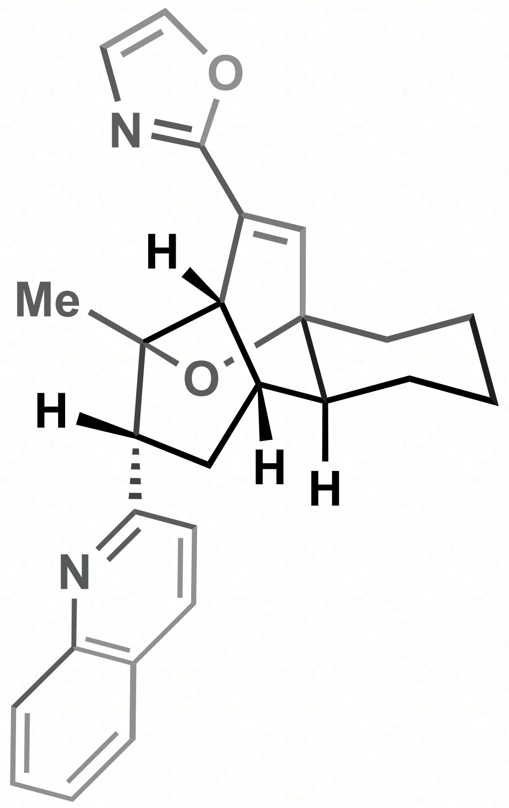

RS1160 (28)

(151 MHz, $CDCl_3$)

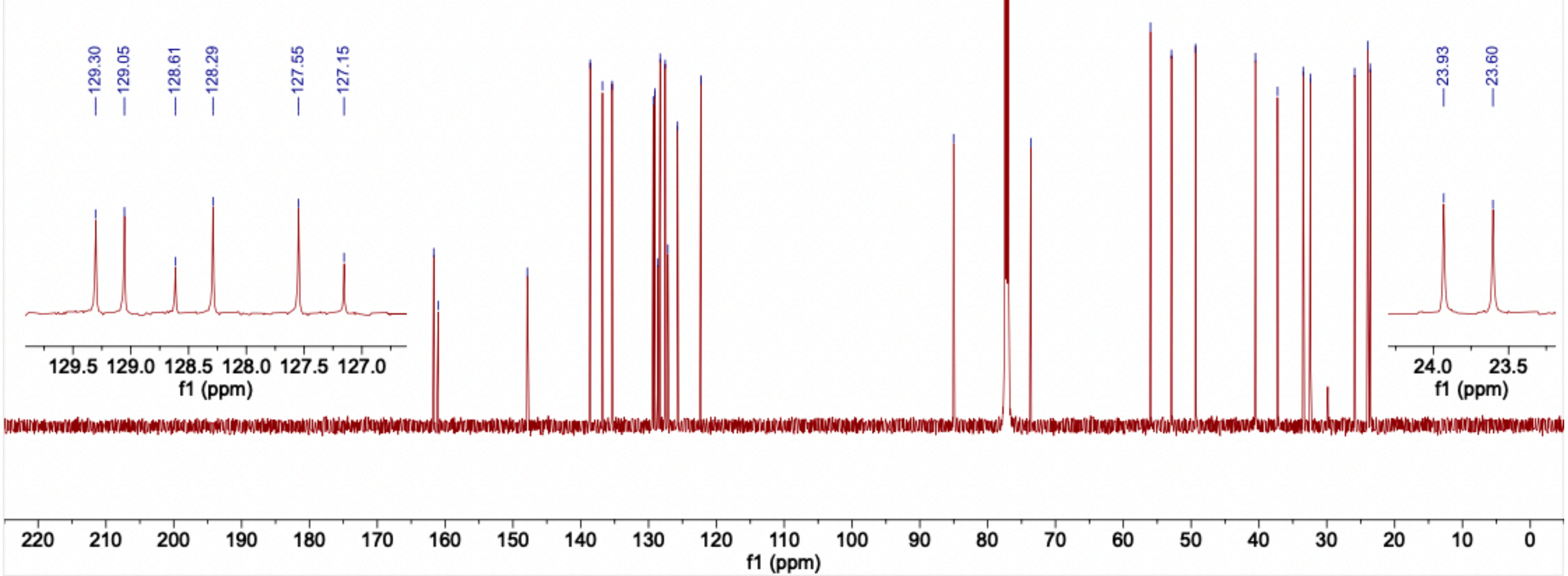

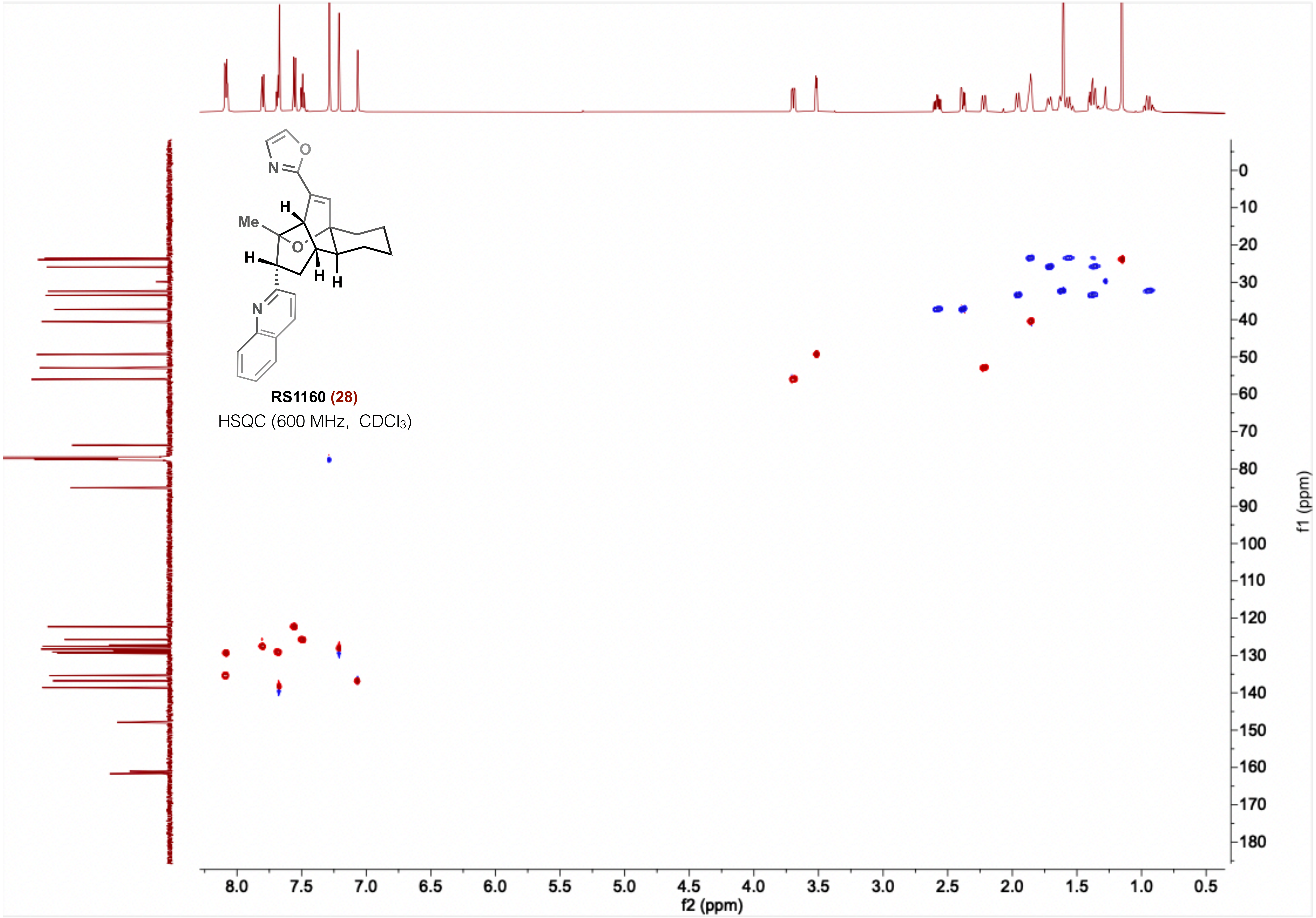

RS1160 (28)

HSQC (600 MHz, $CDCl_3$)

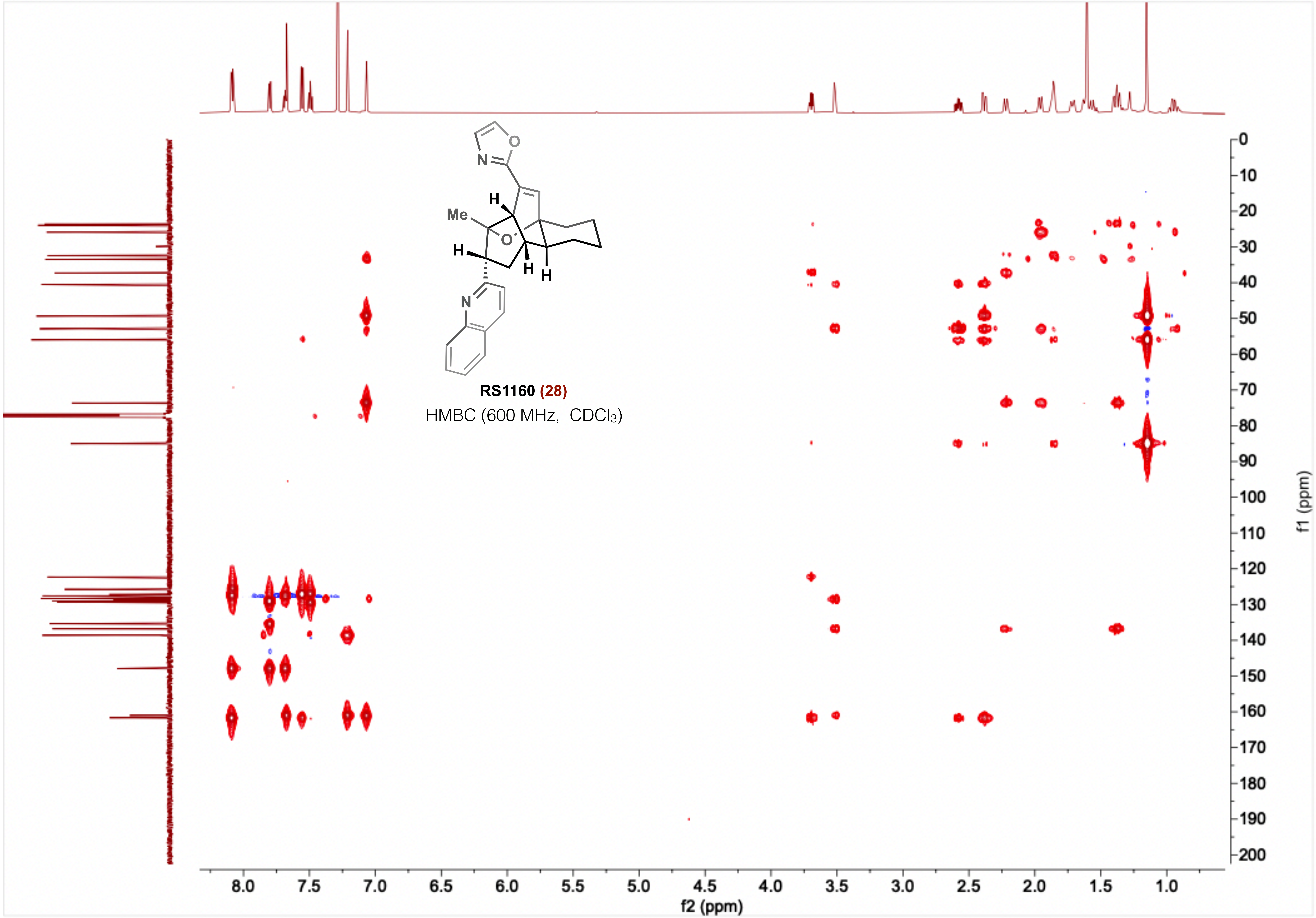

Me
H
H
H
H
O
N
O
N
RS1160 (28)
HMBC (600 MHz, CDCl3)
f2 (ppm)
f1 (ppm)

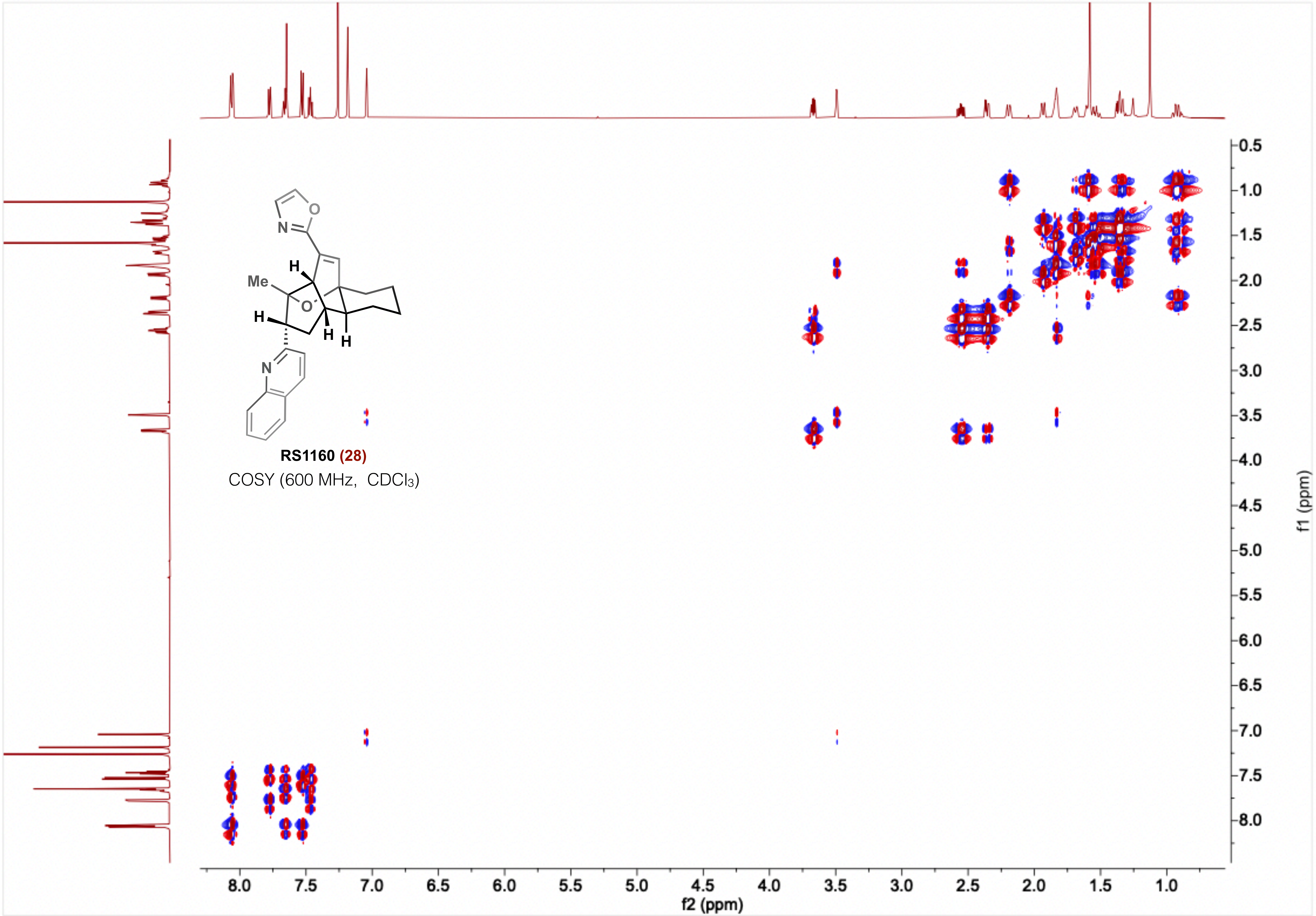
Me
O
H
H
H
H
N
O
N
RS1160 (28)
COSY (600 MHz, CDCl3)
f2 (ppm)
f1 (ppm)

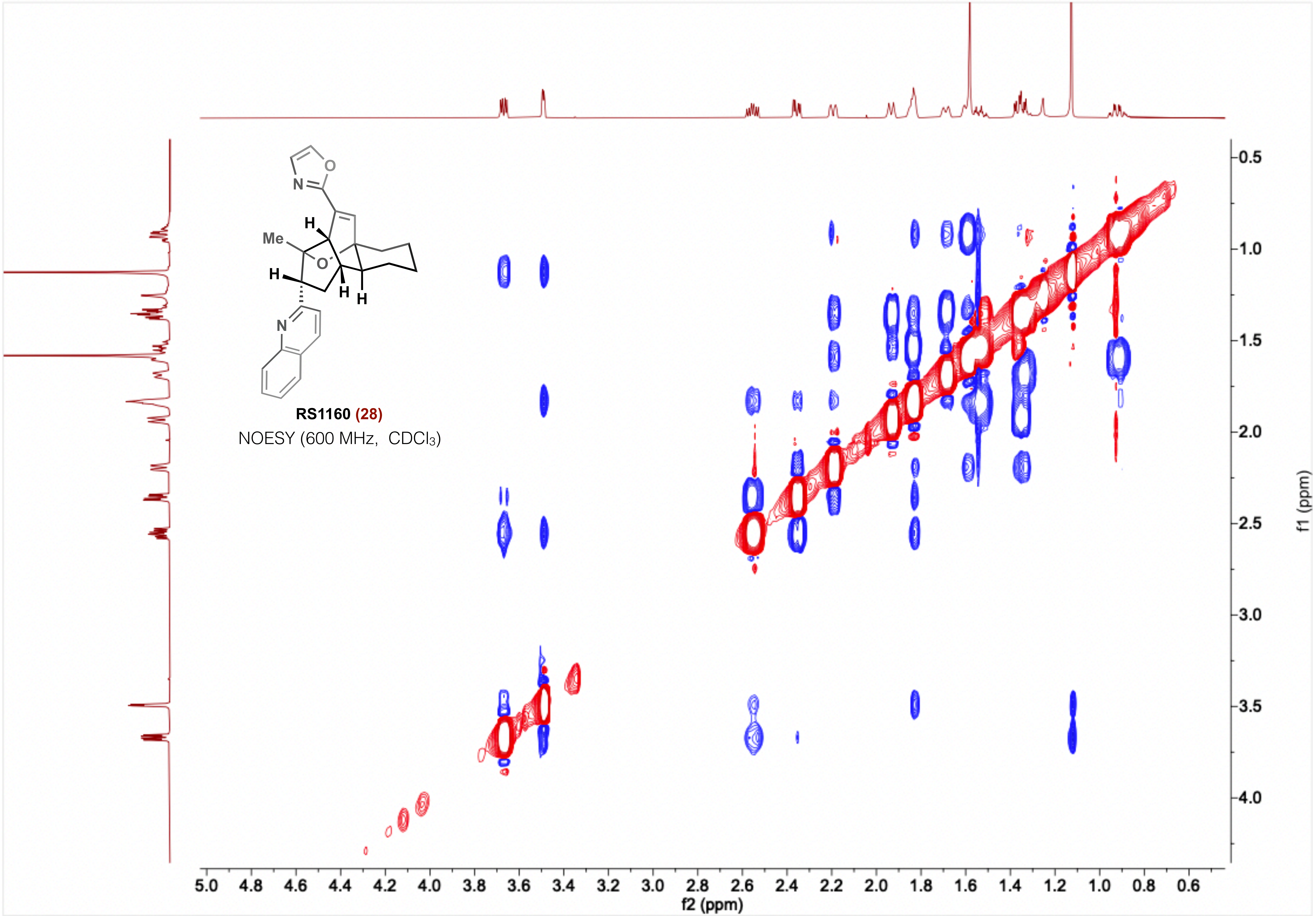

Me
H
O
N
RS1160 (28)
NOESY (600 MHz, CDCl3)
f1 (ppm)
f2 (ppm)

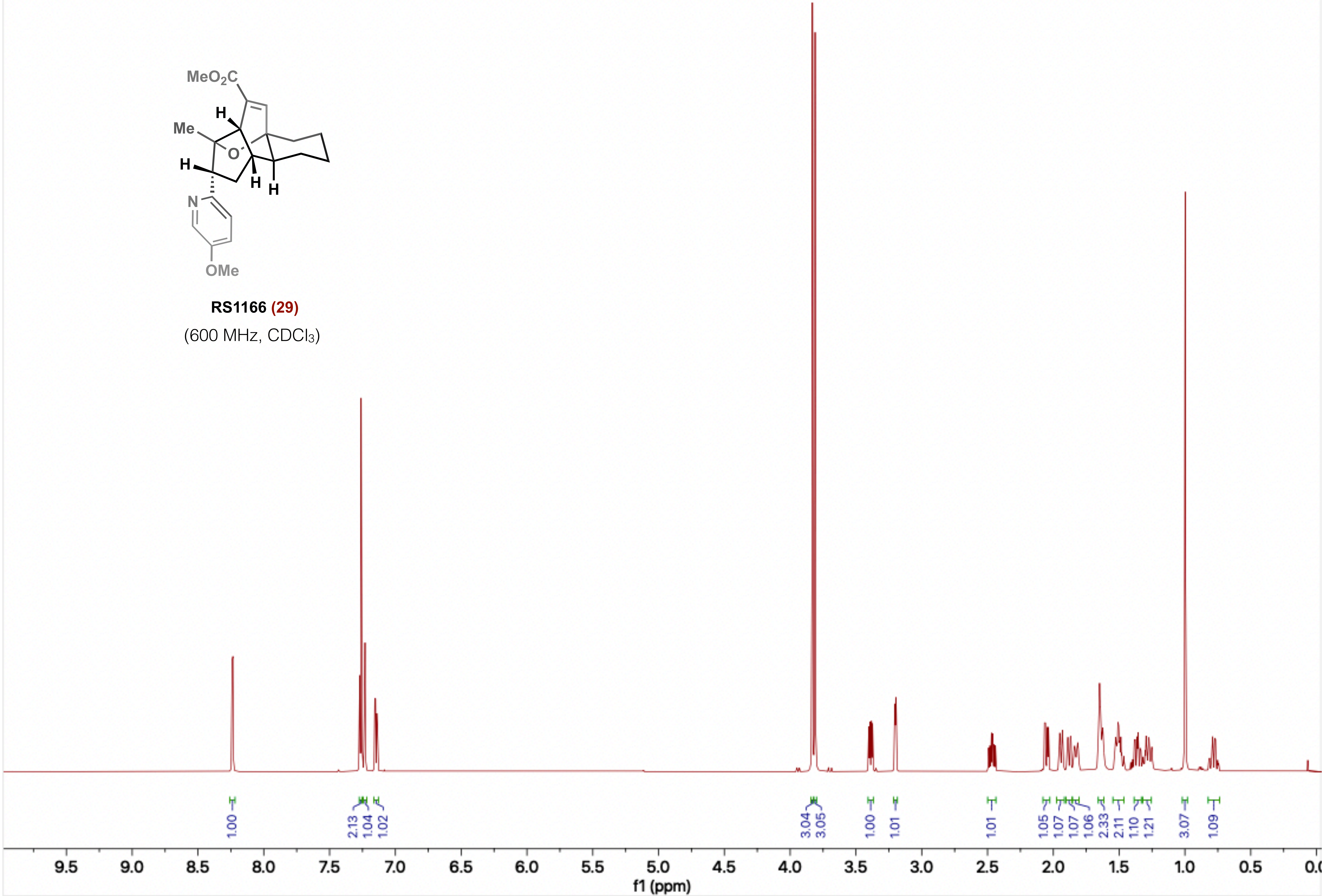

RS1166 **(29)**

(600 MHz, $CDCl_3$)

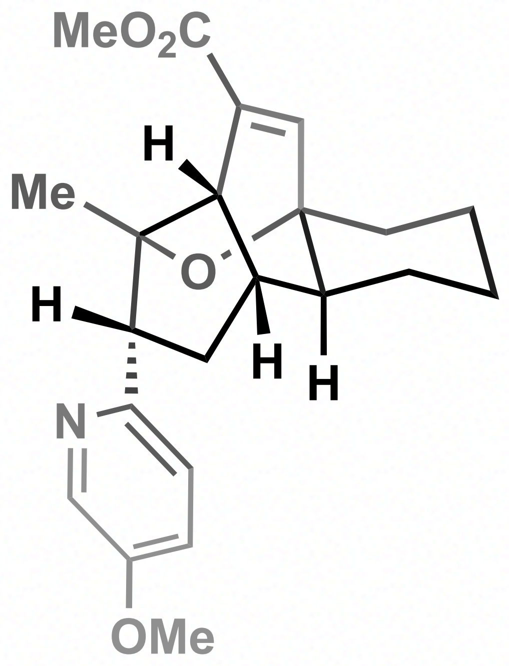

RS1166 (29)

(151 MHz, $CDCl_3$)

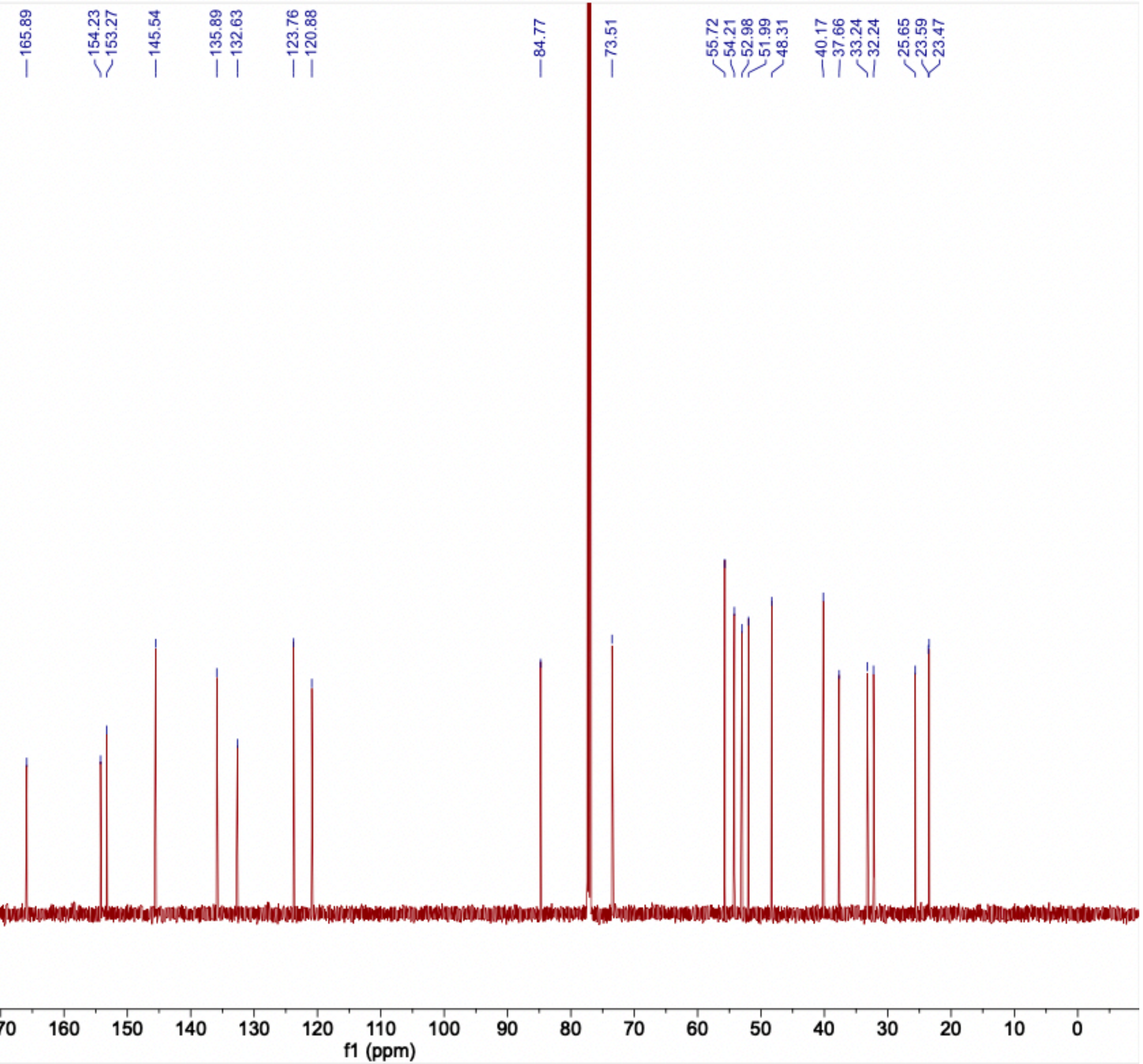

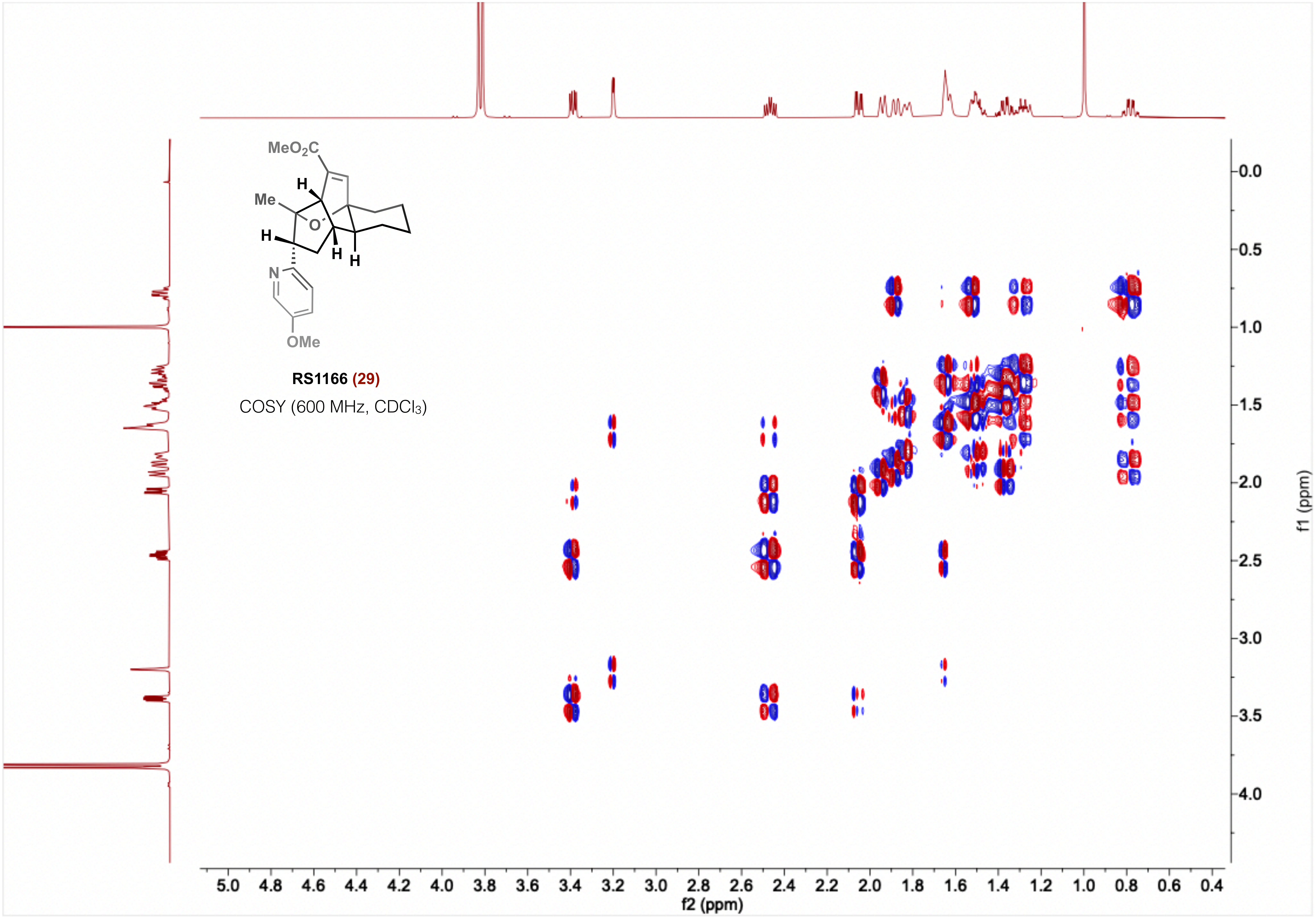
MeO2C
H
Me
O
H
H
H
N
OMe
RS1166 (29)
COSY (600 MHz, CDCl3)
f2 (ppm)
f1 (ppm)
5.0 4.8 4.6 4.4 4.2 4.0 3.8 3.6 3.4 3.2 3.0 2.8 2.6 2.4 2.2 2.0 1.8 1.6 1.4 1.2 1.0 0.8 0.6 0.4
0.0 0.5 1.0 1.5 2.0 2.5 3.0 3.5 4.0

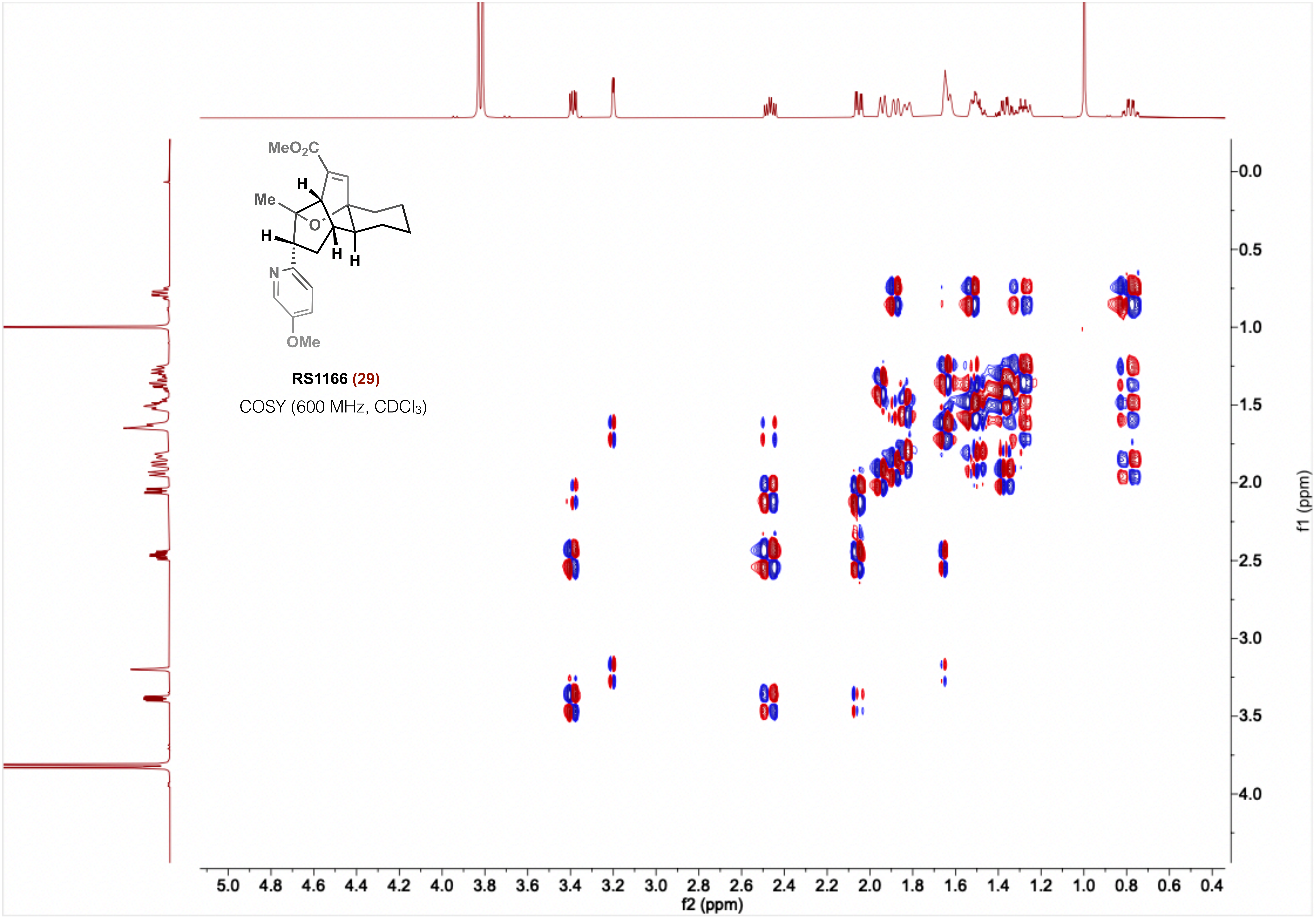

MeO2C
H
Me
O
H
H
H
N
OMe
RS1166 (29)
COSY (600 MHz, CDCl3)
f1 (ppm)
f2 (ppm)

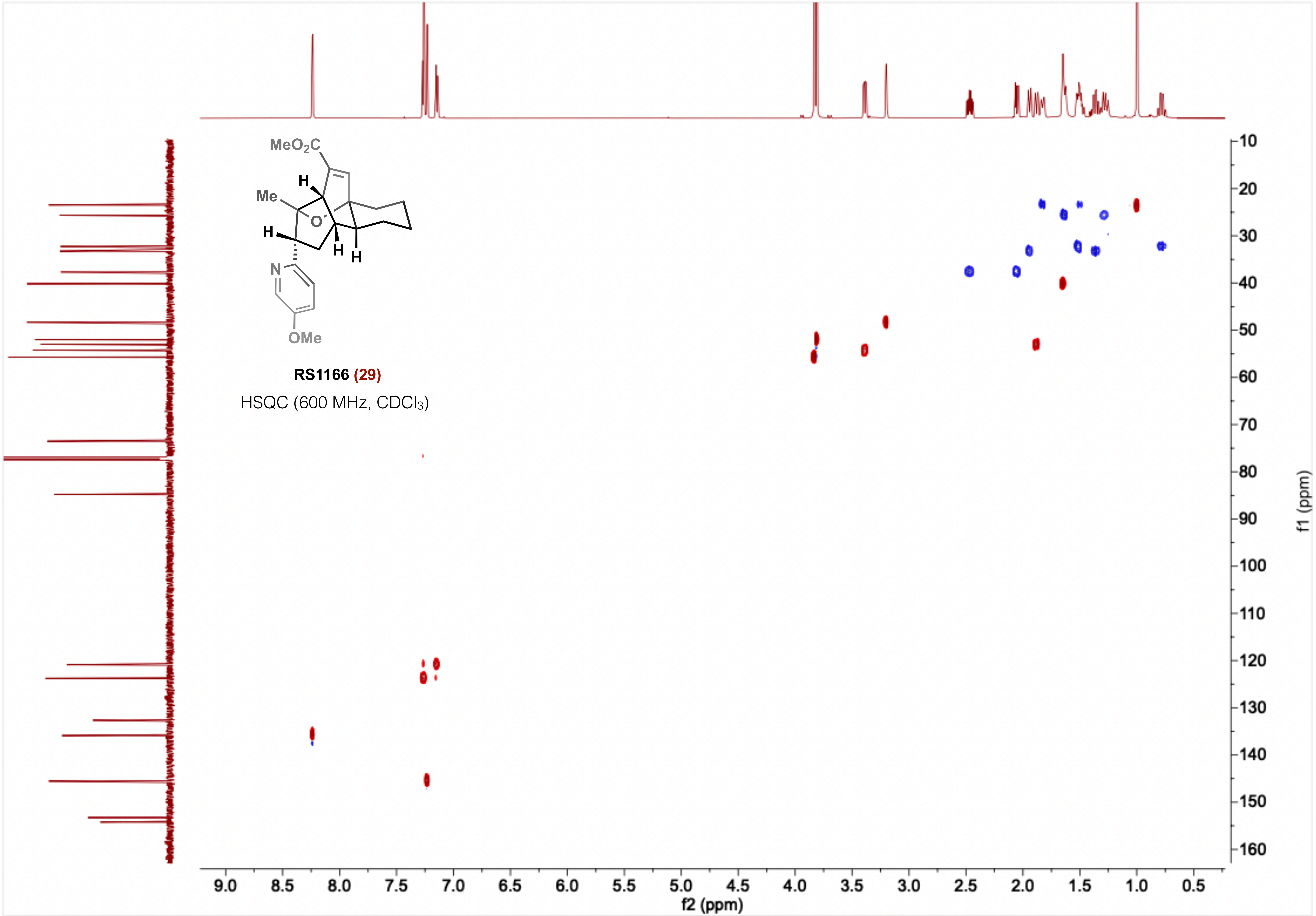
MeO2C
Me
H
O
H
H
H
N
OMe
RS1166 (29)
HSQC (600 MHz, CDCl3)
f2 (ppm)
f1 (ppm)
9.0 8.5 8.0 7.5 7.0 6.5 6.0 5.5 5.0 4.5 4.0 3.5 3.0 2.5 2.0 1.5 1.0 0.5
10 20 30 40 50 60 70 80 90 100 110 120 130 140 150 160

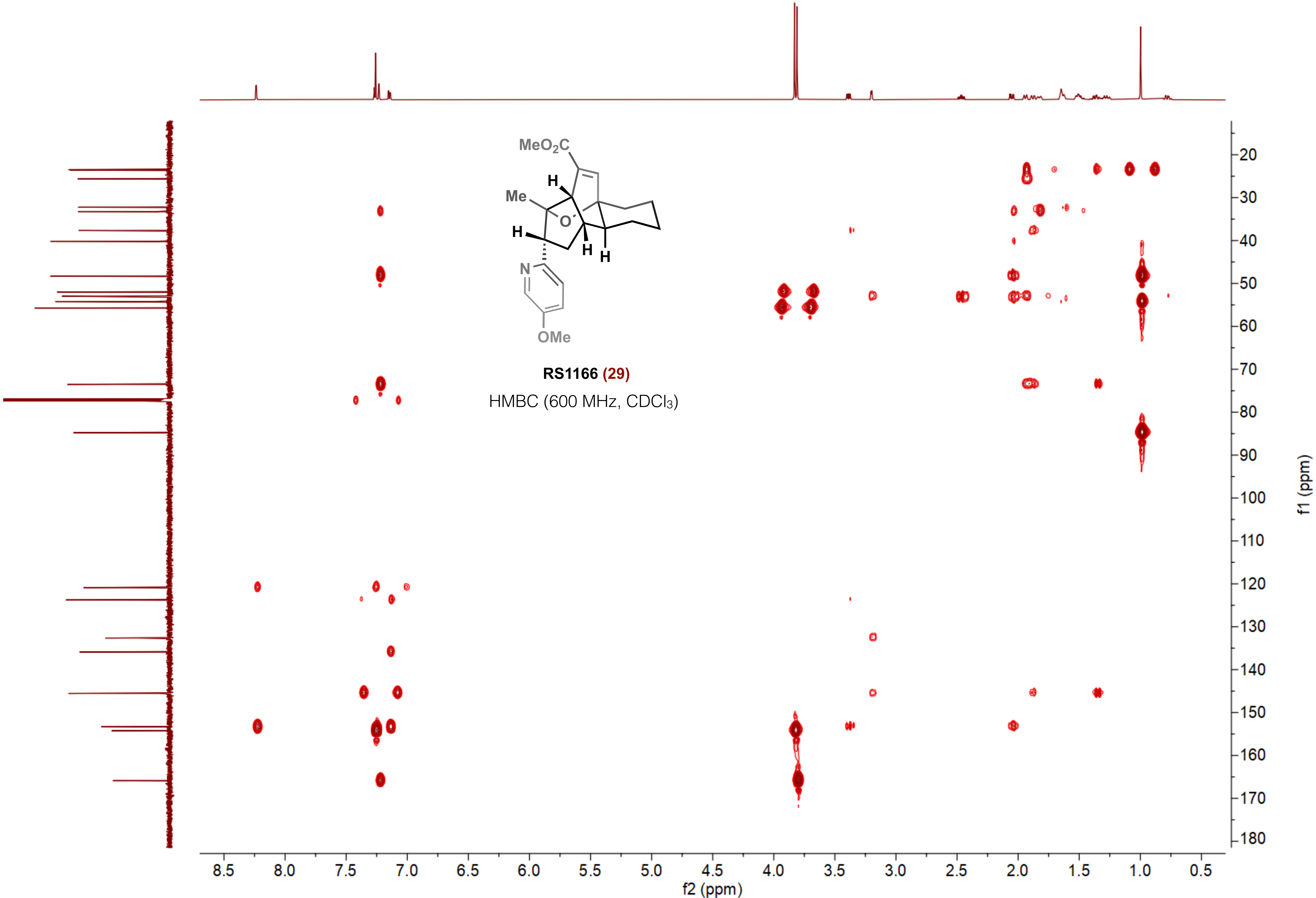

RS1166 (29)

HMBC (600 MHz, $CDCl_3$)

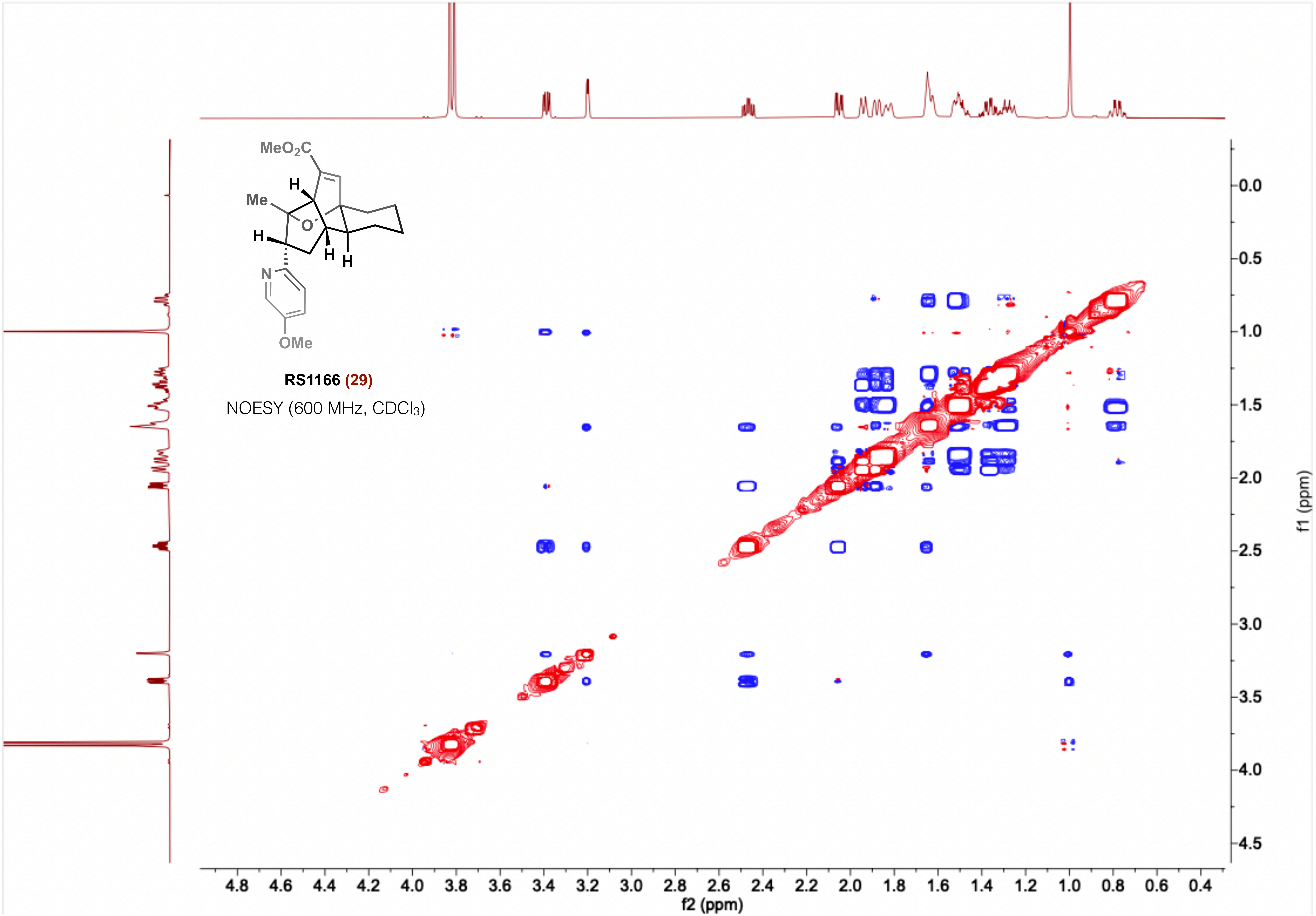

MeO2C
H
Me
O
H
H
H
N
OMe
RS1166 (29)
NOESY (600 MHz, CDCl3)
f1 (ppm)
f2 (ppm)

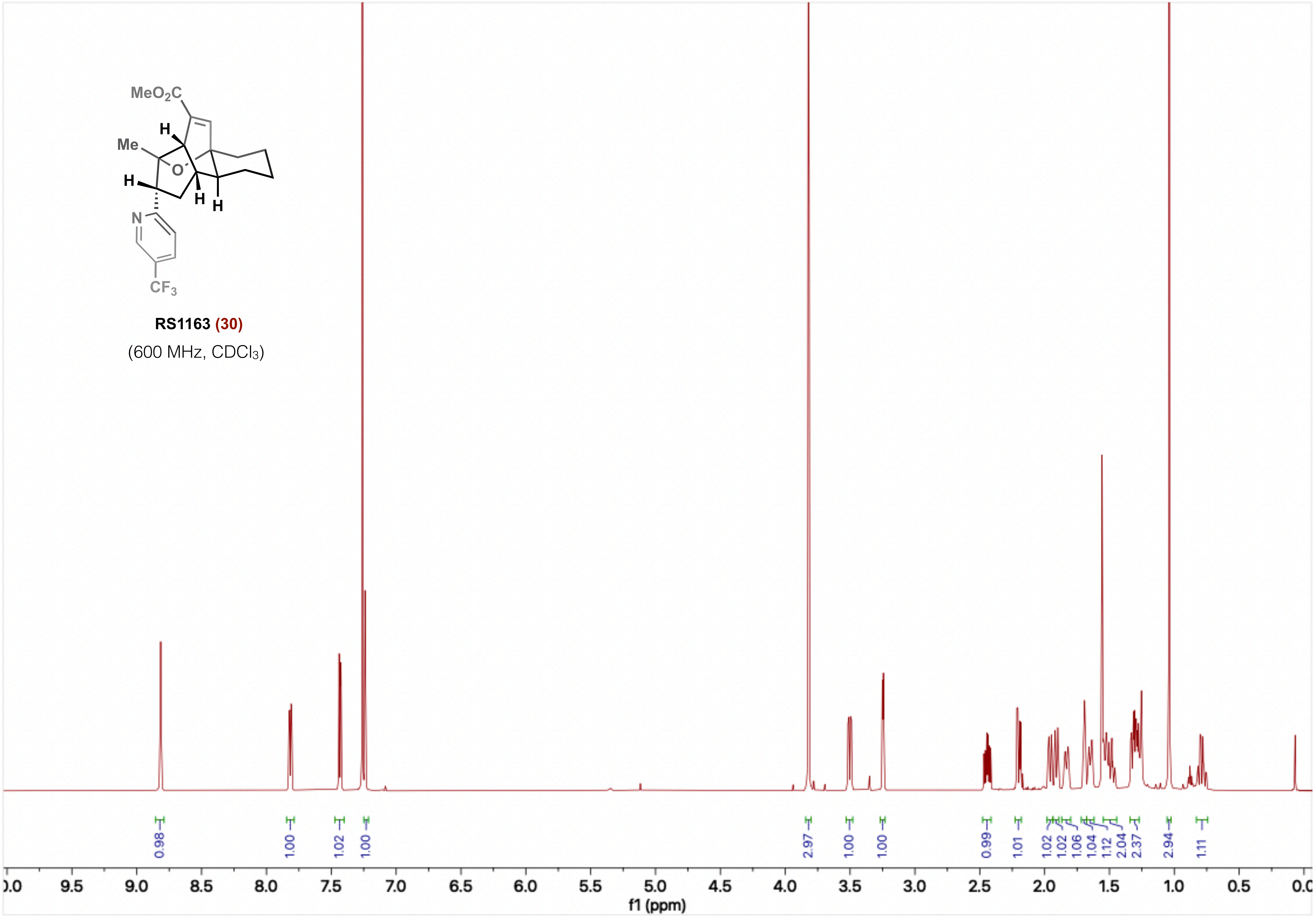

MeO2C
Me
H
O
N
CF3
H
H
H
RS1163 (30)
(600 MHz, CDCl3)
0.98
1.00
1.02
1.00
2.97
1.00
1.00
0.99
1.01
1.02
1.02
1.06
1.04
1.12
2.04
2.37
2.94
1.11
f1 (ppm)

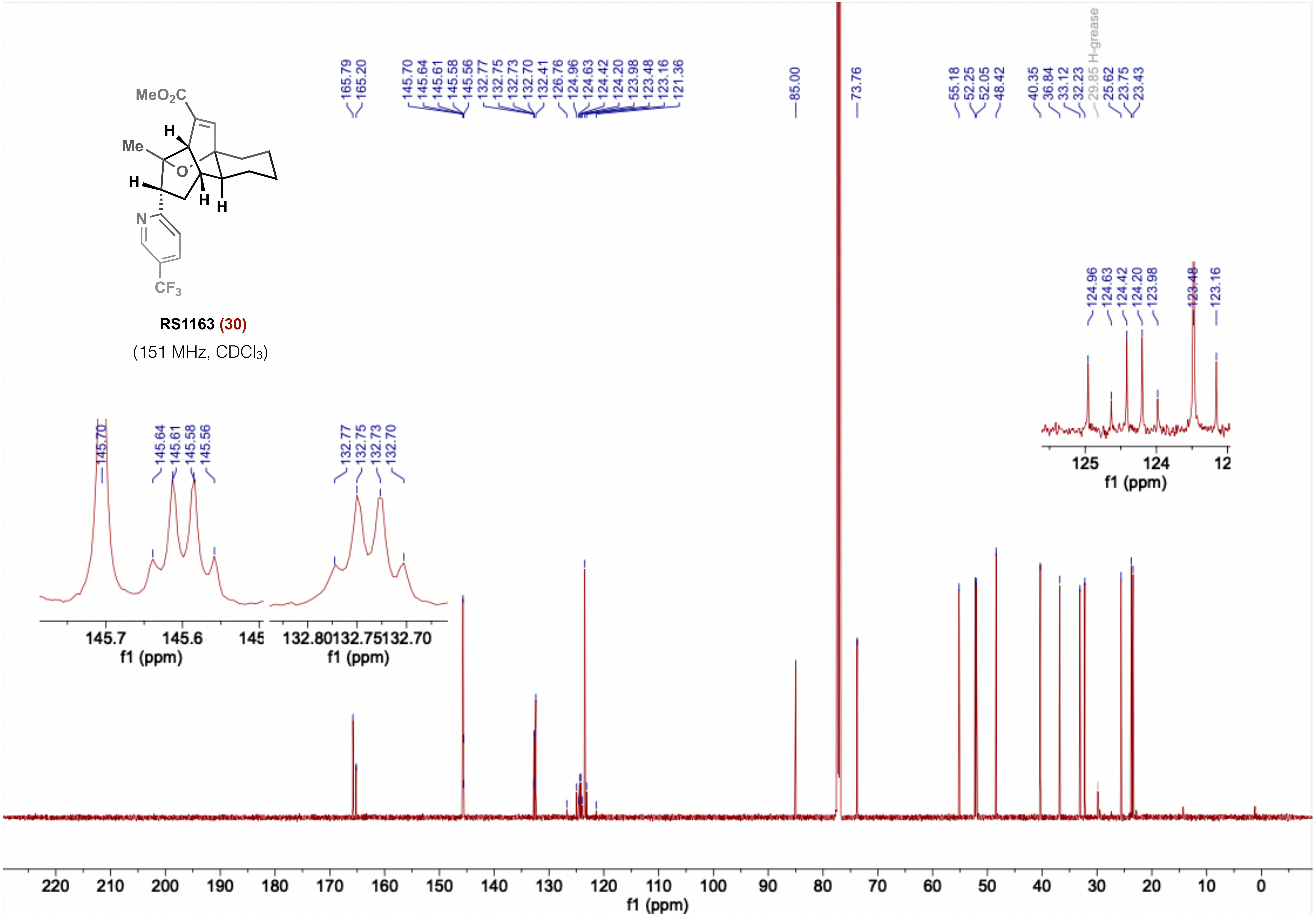

MeO2C
Me
H
O
N
CF3
H
H
H
RS1163 (30)
(151 MHz, CDCl3)
165.79
165.20
145.70
145.64
145.61
145.58
145.56
132.77
132.75
132.73
132.70
132.41
126.76
124.96
124.63
124.42
124.20
123.98
123.48
123.16
121.36
85.00
73.76
55.18
52.25
52.05
48.42
40.35
36.84
33.12
32.23
29.85 H-grease
25.62
23.75
23.43
f1 (ppm)

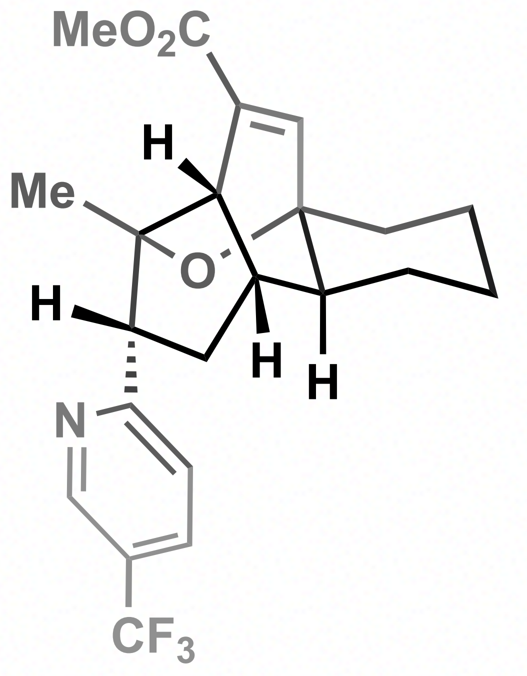

RS1163 **(30)**

19F (376 MHz, $CDCl_3$)

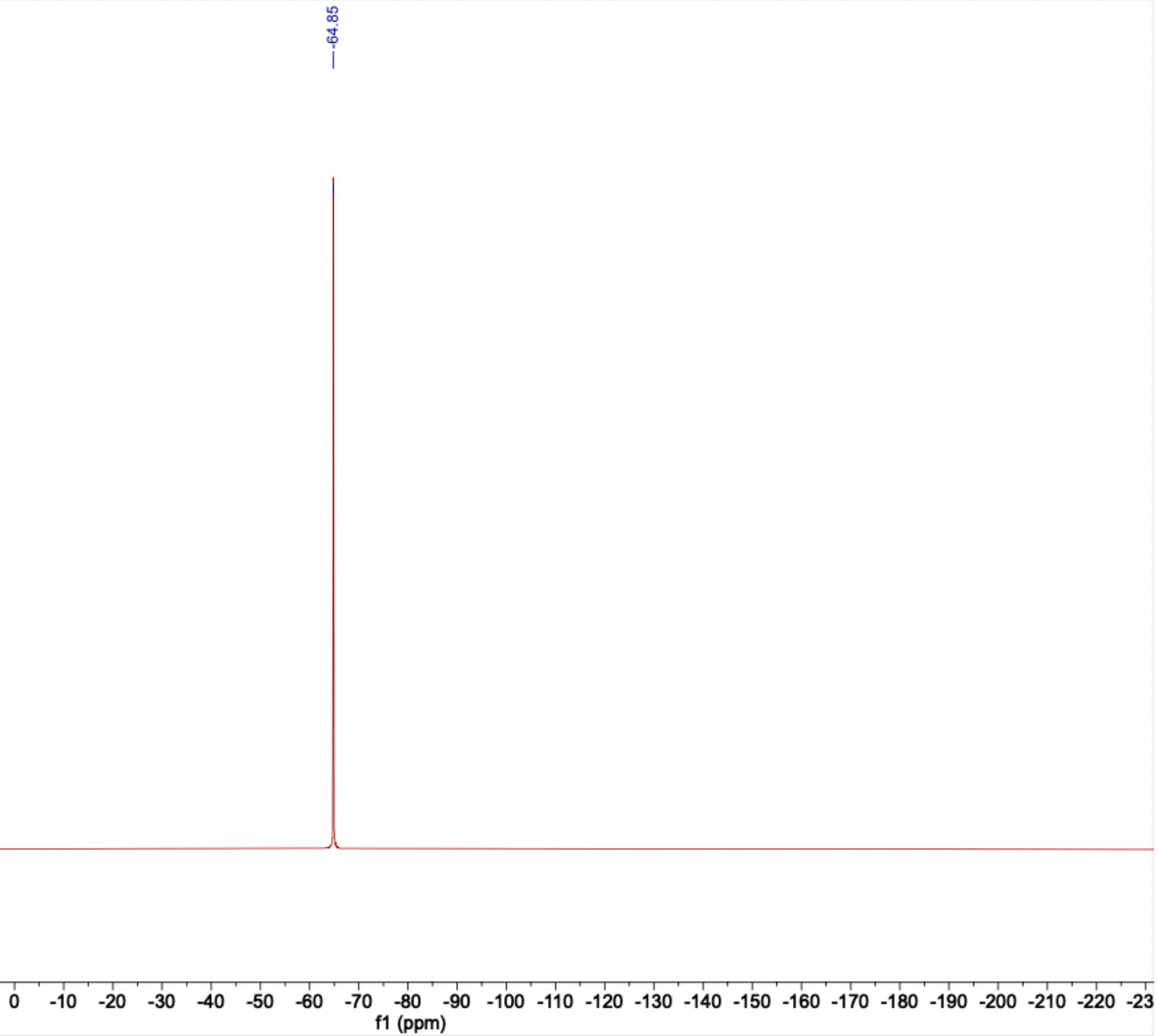

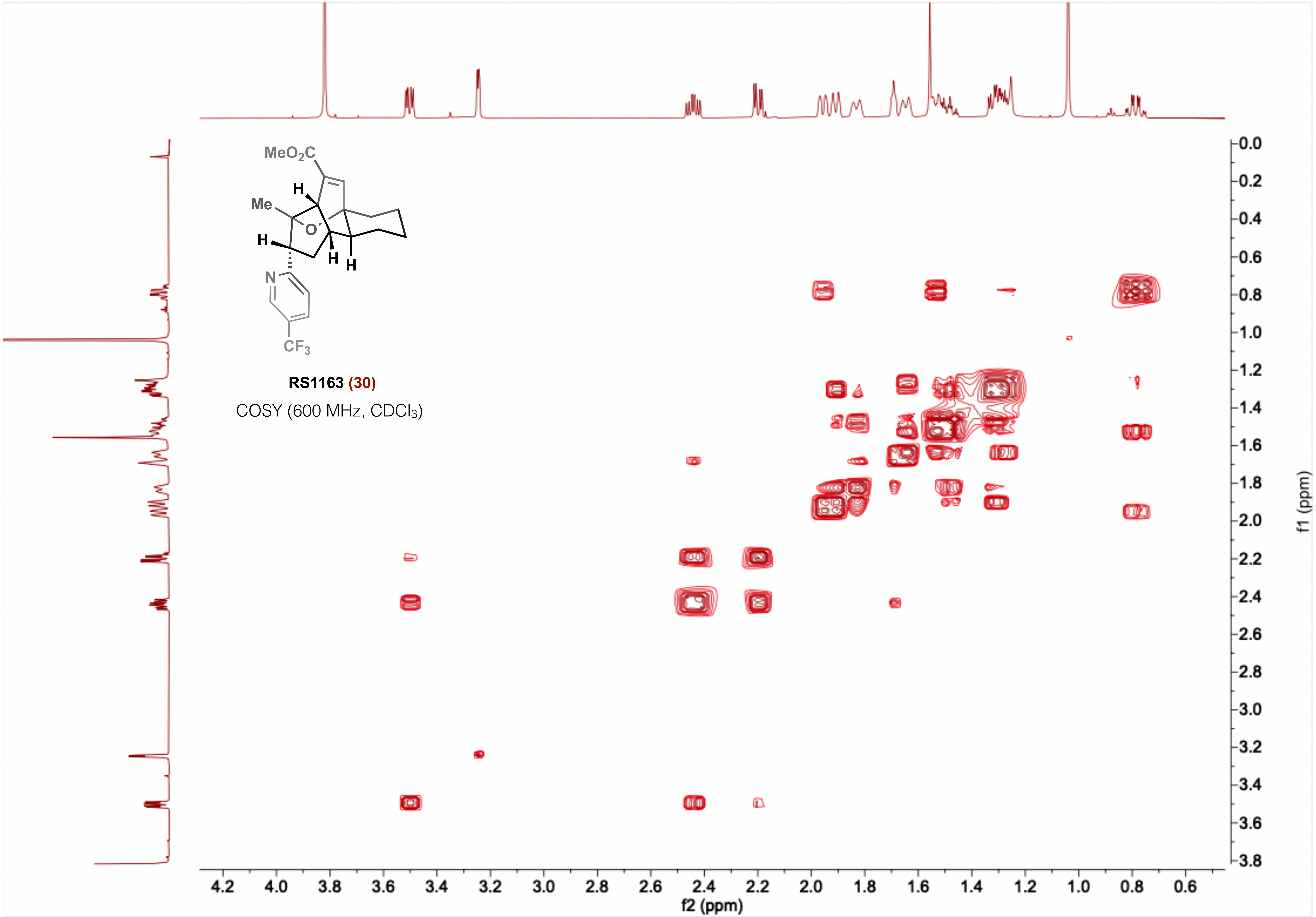

MeO2C
Me
H
O
N
CF3
RS1163 (30)
COSY (600 MHz, CDCl3)
f2 (ppm)
4.2 4.0 3.8 3.6 3.4 3.2 3.0 2.8 2.6 2.4 2.2 2.0 1.8 1.6 1.4 1.2 1.0 0.8 0.6
f1 (ppm)
0.0 0.2 0.4 0.6 0.8 1.0 1.2 1.4 1.6 1.8 2.0 2.2 2.4 2.6 2.8 3.0 3.2 3.4 3.6 3.8

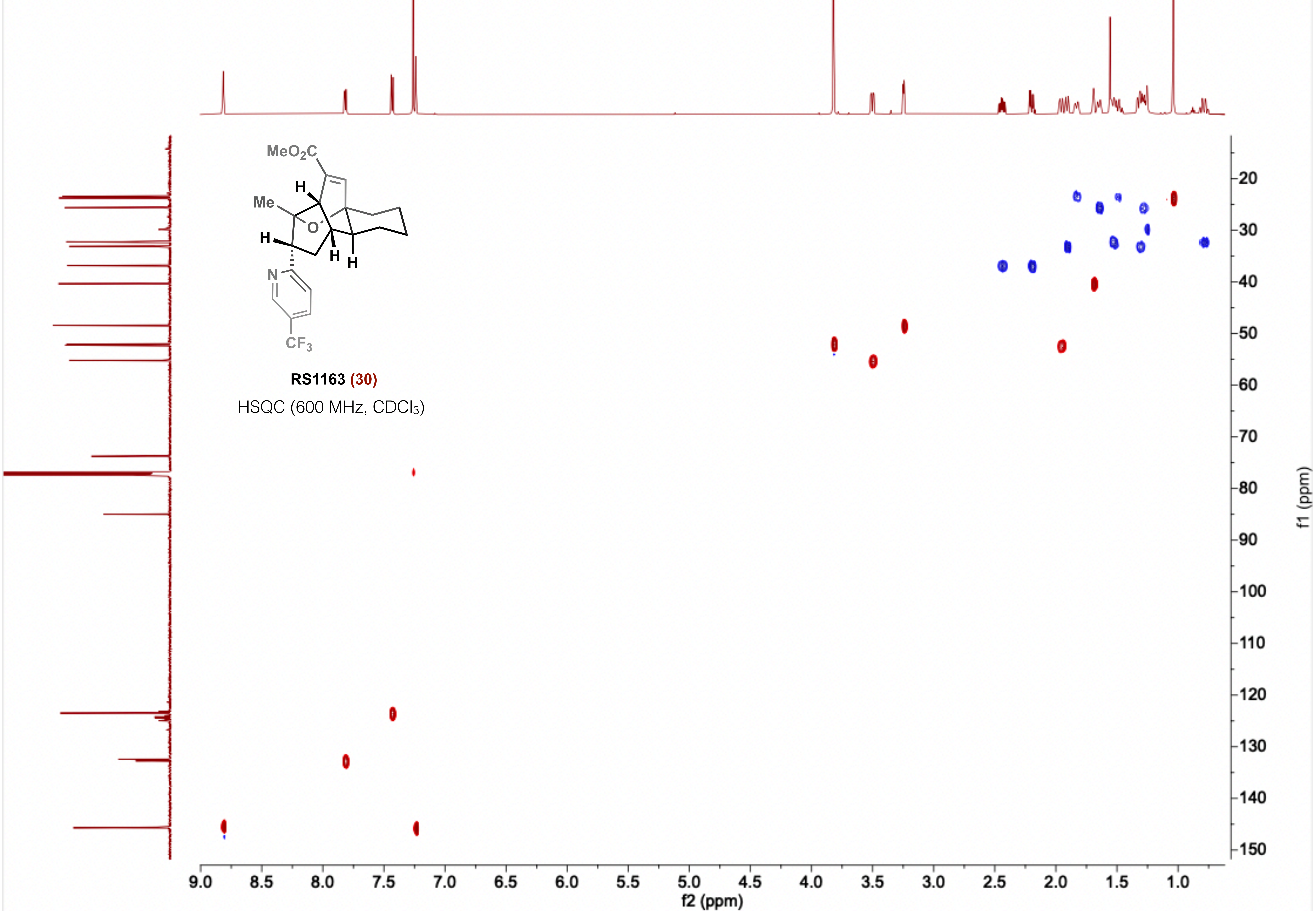

RS1163 (30)

HSQC (600 MHz, $CDCl_3$)

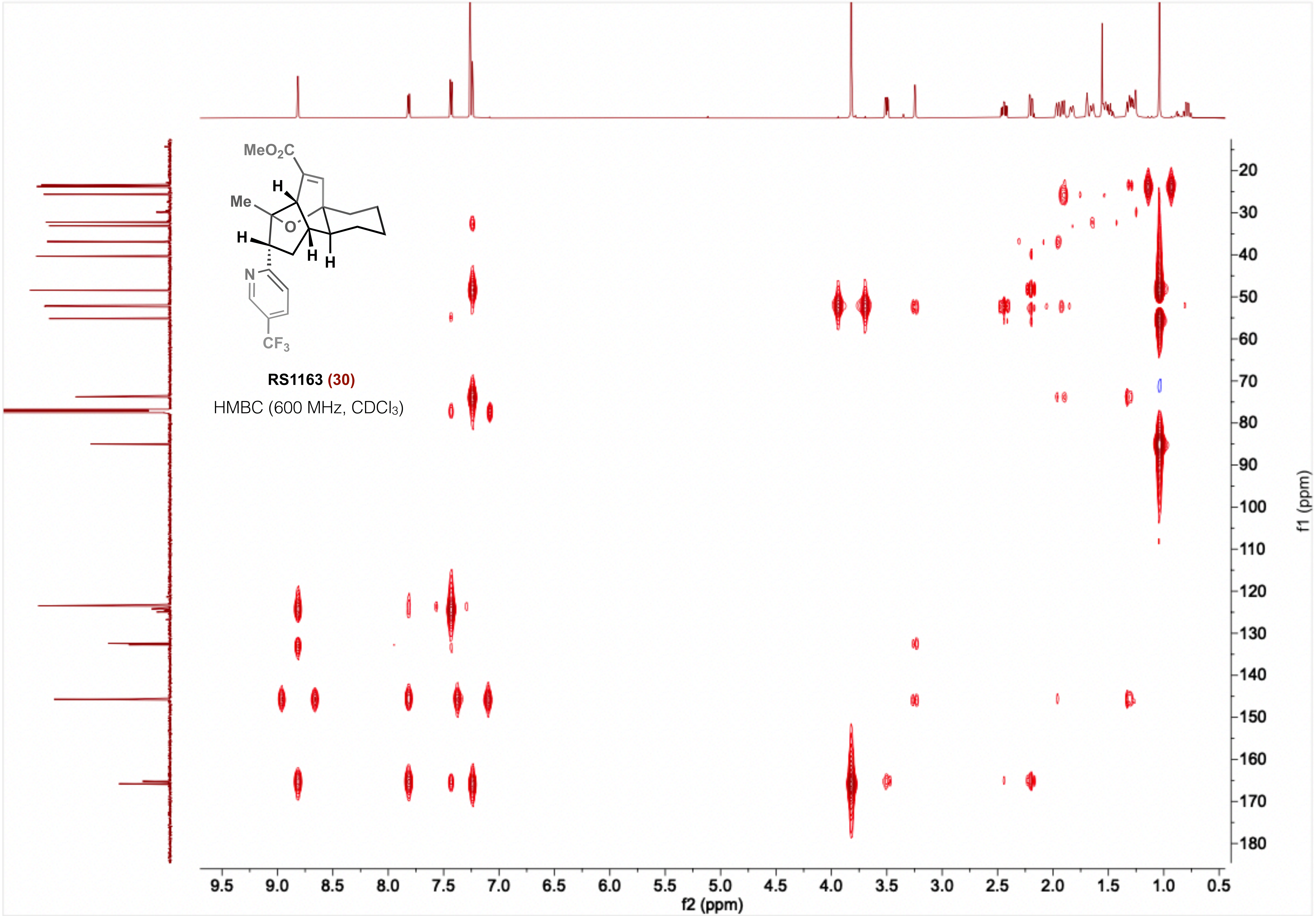
MeO2C
Me
H
O
H
H
H
N
CF3
RS1163 (30)
HMBC (600 MHz, CDCl3)
f1 (ppm)
f2 (ppm)
9.5 9.0 8.5 8.0 7.5 7.0 6.5 6.0 5.5 5.0 4.5 4.0 3.5 3.0 2.5 2.0 1.5 1.0 0.5
20 30 40 50 60 70 80 90 100 110 120 130 140 150 160 170 180

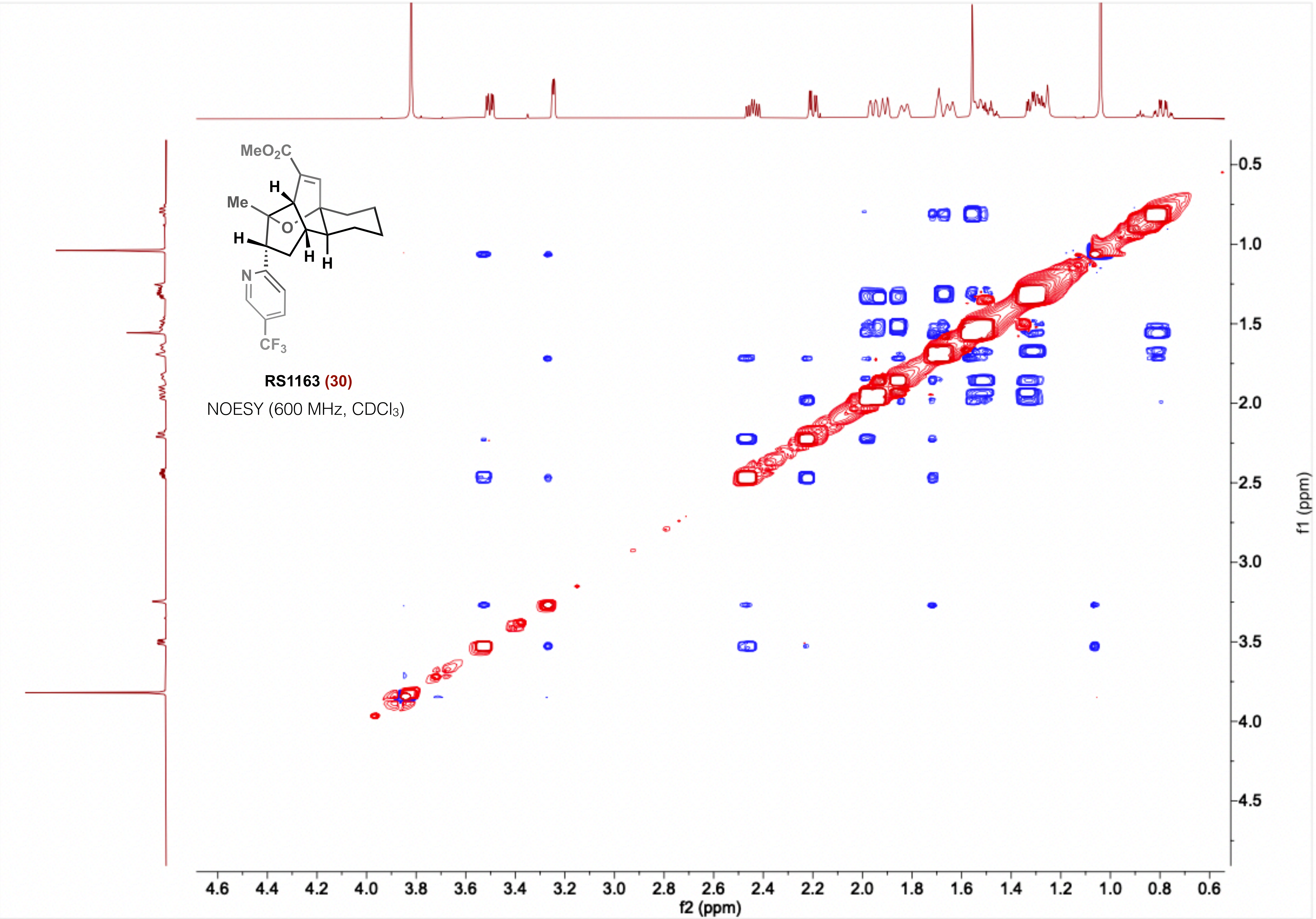

MeO2C
Me
H
O
H
H H
N
CF3
RS1163 (30)
NOESY (600 MHz, CDCl3)
f1 (ppm)
f2 (ppm)
4.6 4.4 4.2 4.0 3.8 3.6 3.4 3.2 3.0 2.8 2.6 2.4 2.2 2.0 1.8 1.6 1.4 1.2 1.0 0.8 0.6
0.5 1.0 1.5 2.0 2.5 3.0 3.5 4.0 4.5

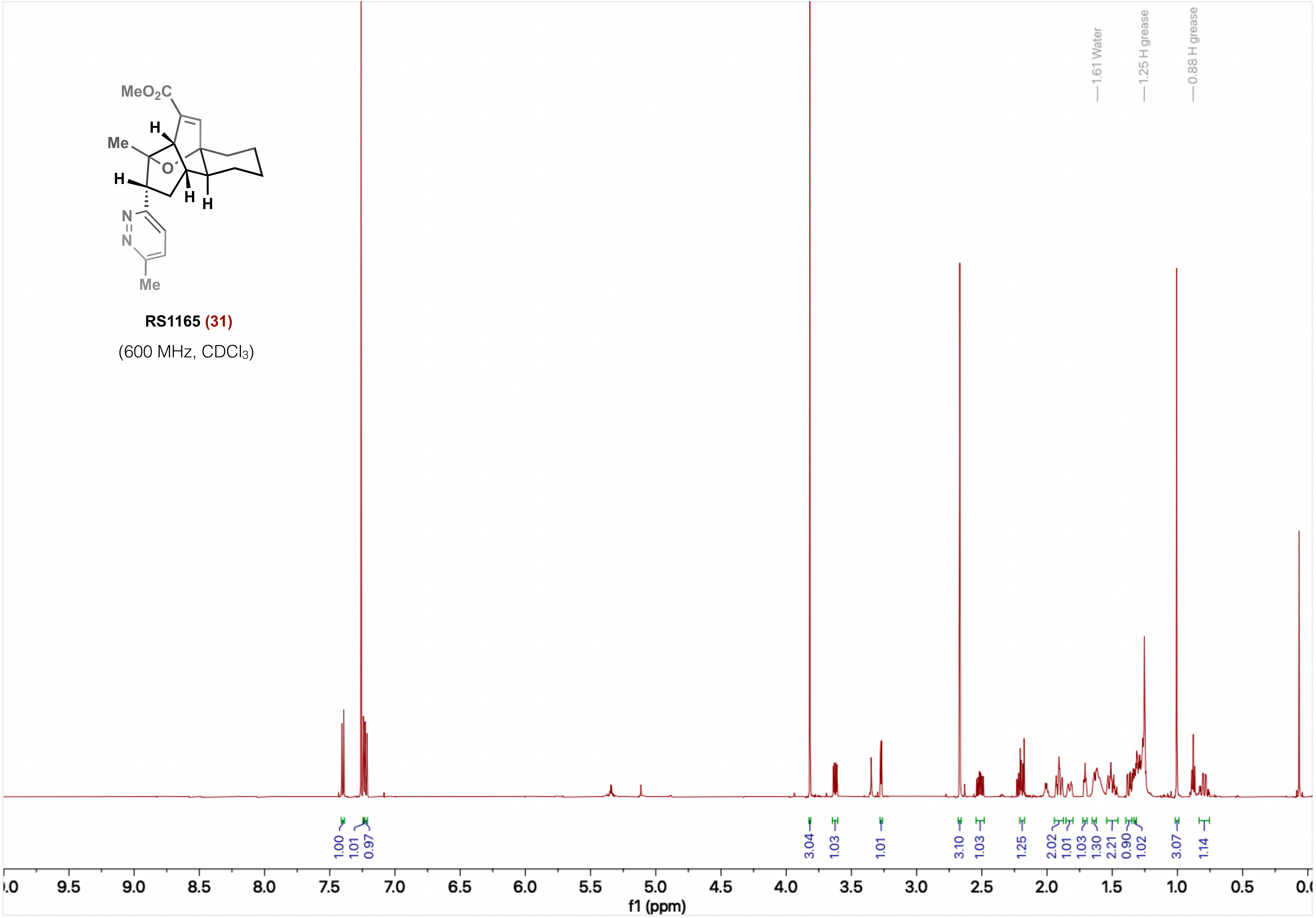

MeO2C
Me
O
H
N
N
Me
RS1165 (31)
(600 MHz, CDCl3)
1.61 Water
1.25 H grease
0.88 H grease
1.00
1.01
0.97
3.04
1.03
1.01
3.10
1.03
1.25
2.02
1.01
1.03
1.30
2.21
0.90
1.02
3.07
1.14
9.5
9.0
8.5
8.0
7.5
7.0
6.5
6.0
5.5
5.0
4.5
4.0
3.5
3.0
2.5
2.0
1.5
1.0
0.5
f1 (ppm)

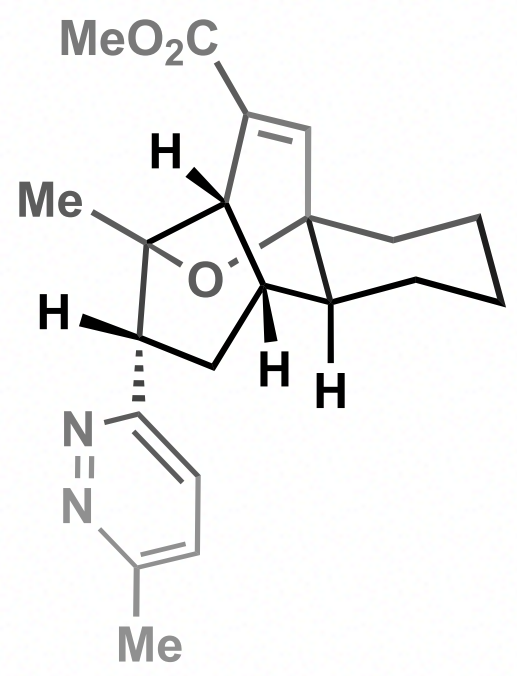

RS1165 **(31)**

(151 MHz, $CDCl_3$)

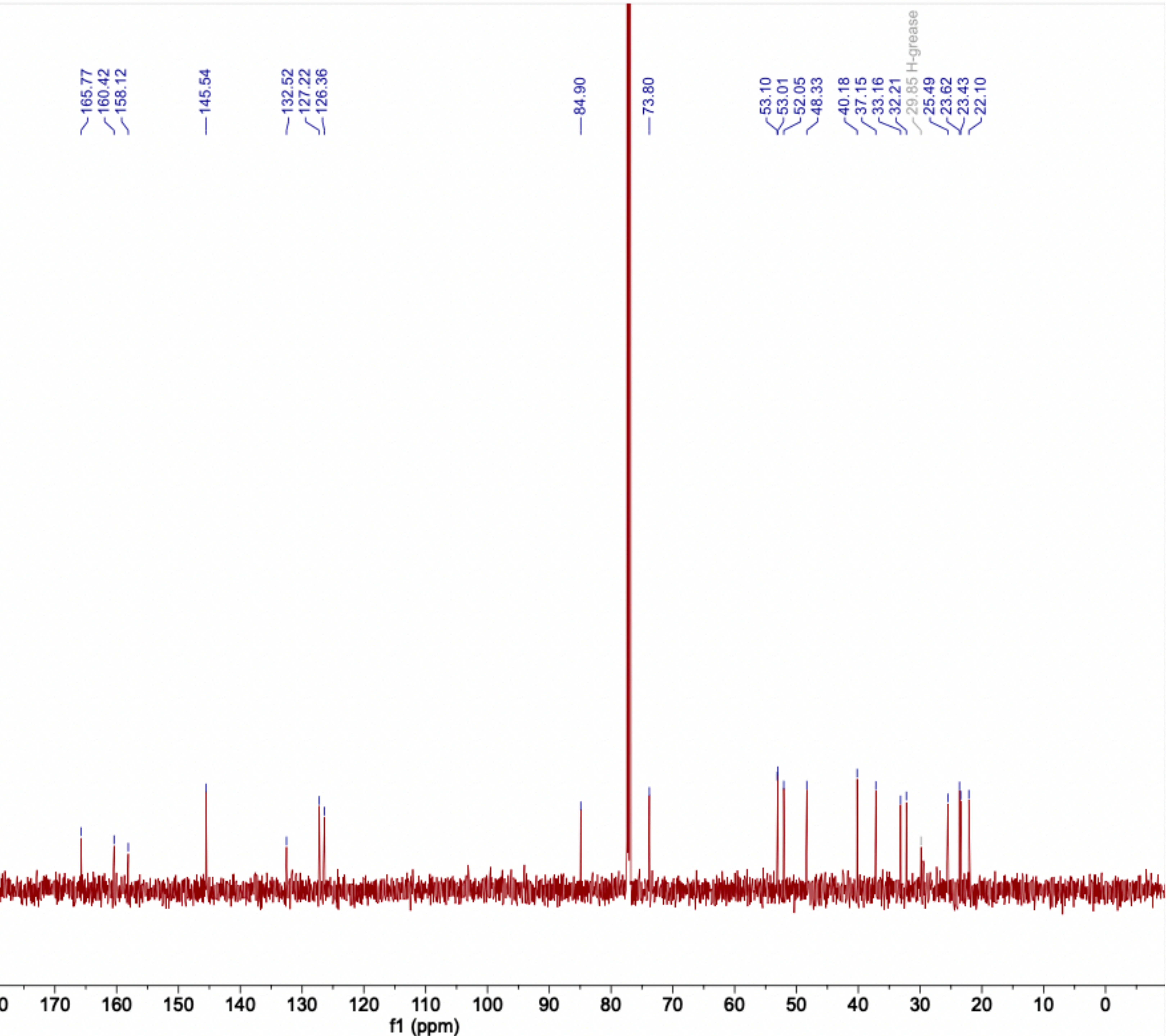

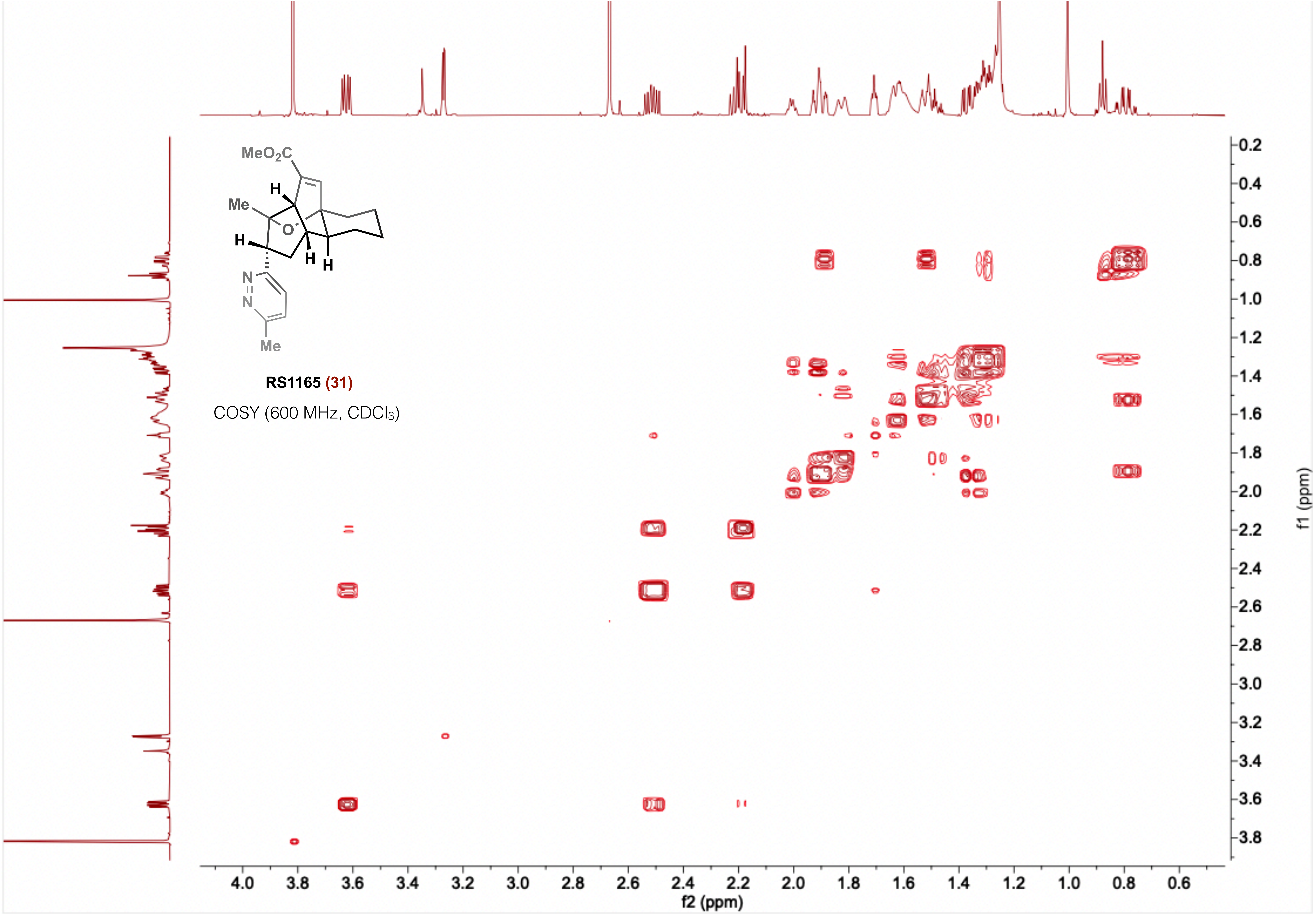
MeO2C
Me
H
O
H
H
H
N
N
Me
RS1165 (31)
COSY (600 MHz, CDCl3)
f2 (ppm)
f1 (ppm)

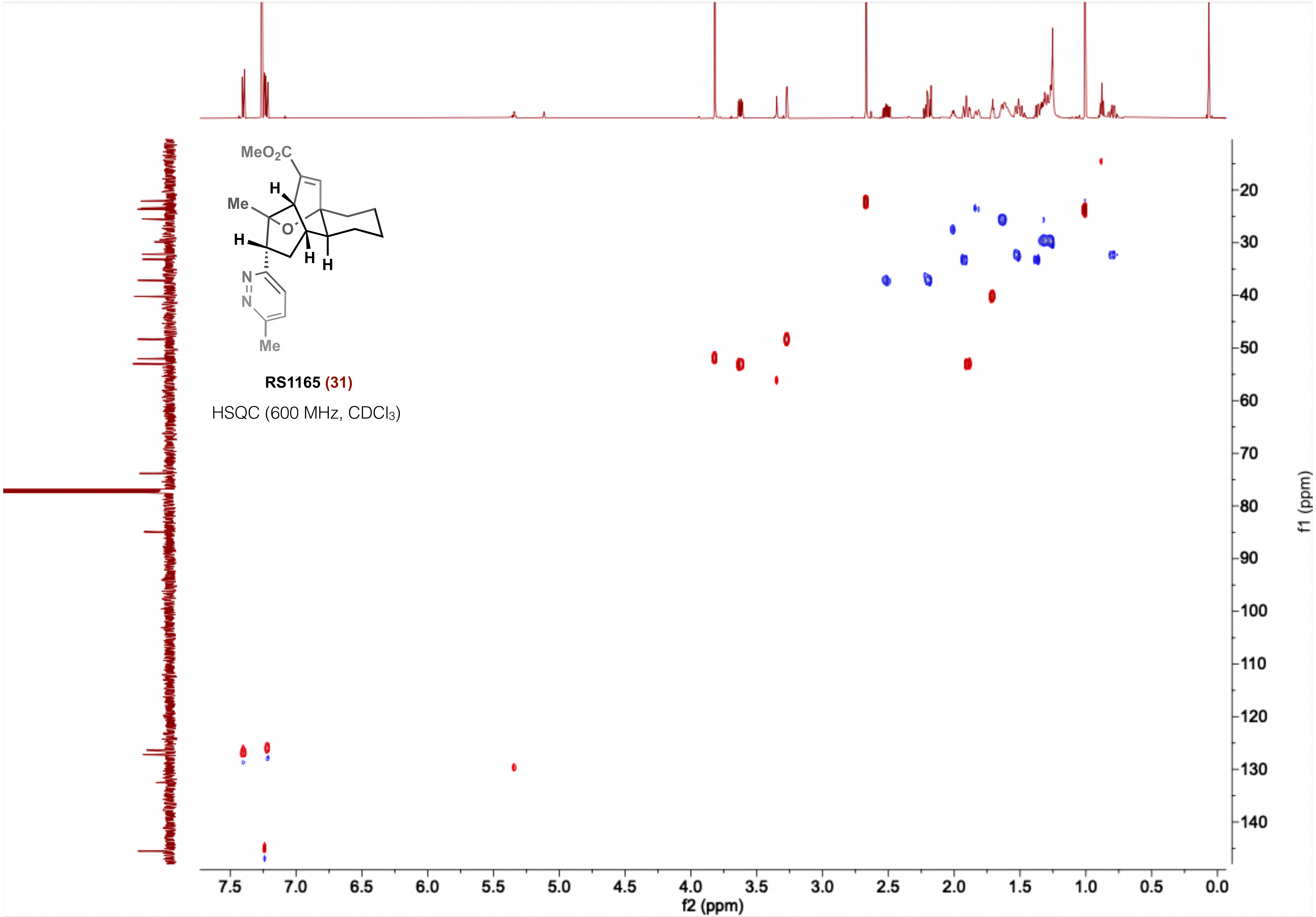

RS1165 (31)

HSQC (600 MHz, $CDCl_3$)

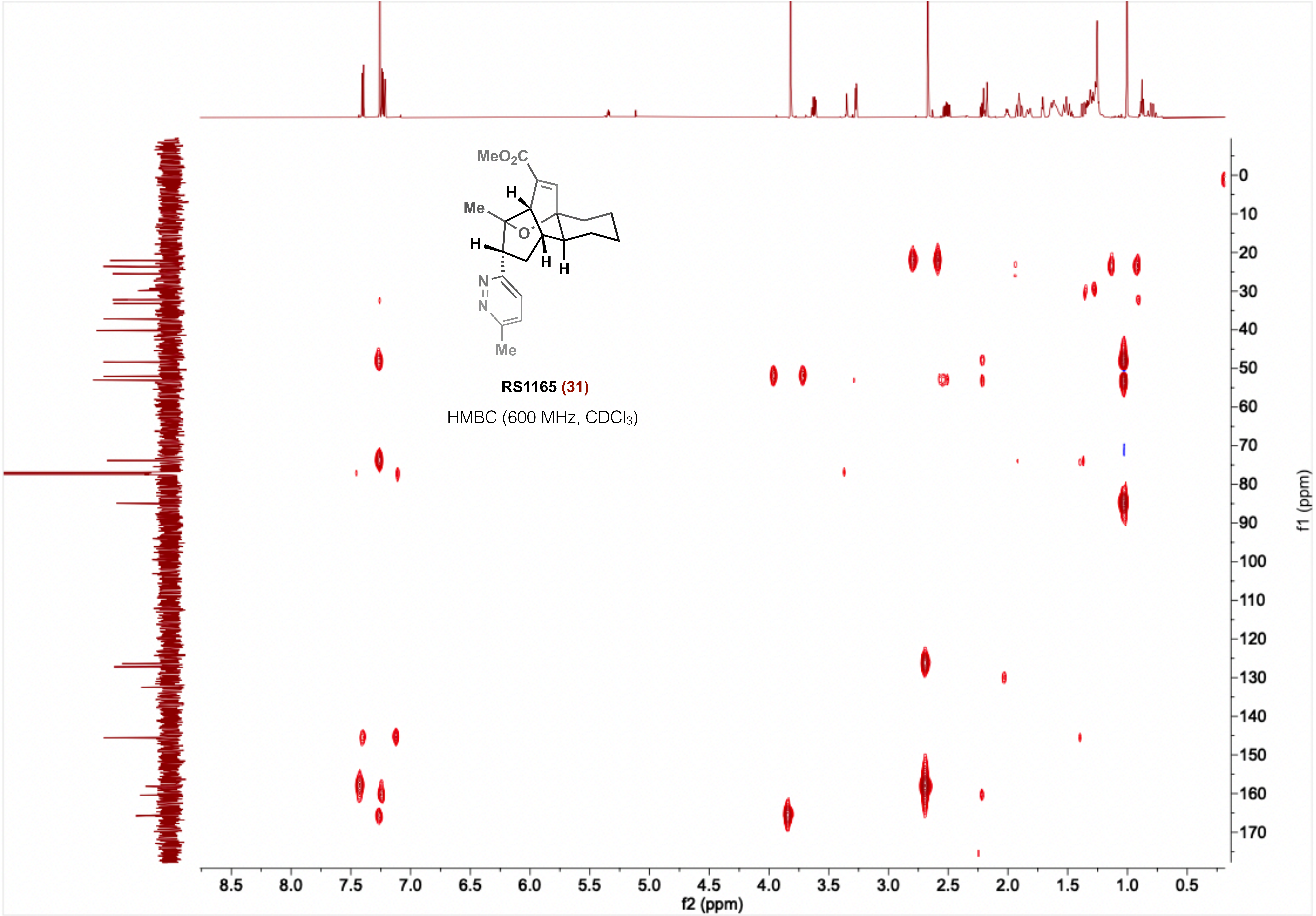
MeO2C
H
Me
O
H
H
H
N
N
Me
RS1165 (31)
HMBC (600 MHz, CDCl3)
f2 (ppm)
f1 (ppm)
8.5
8.0
7.5
7.0
6.5
6.0
5.5
5.0
4.5
4.0
3.5
3.0
2.5
2.0
1.5
1.0
0.5
0
10
20
30
40
50
60
70
80
90
100
110
120
130
140
150
160
170

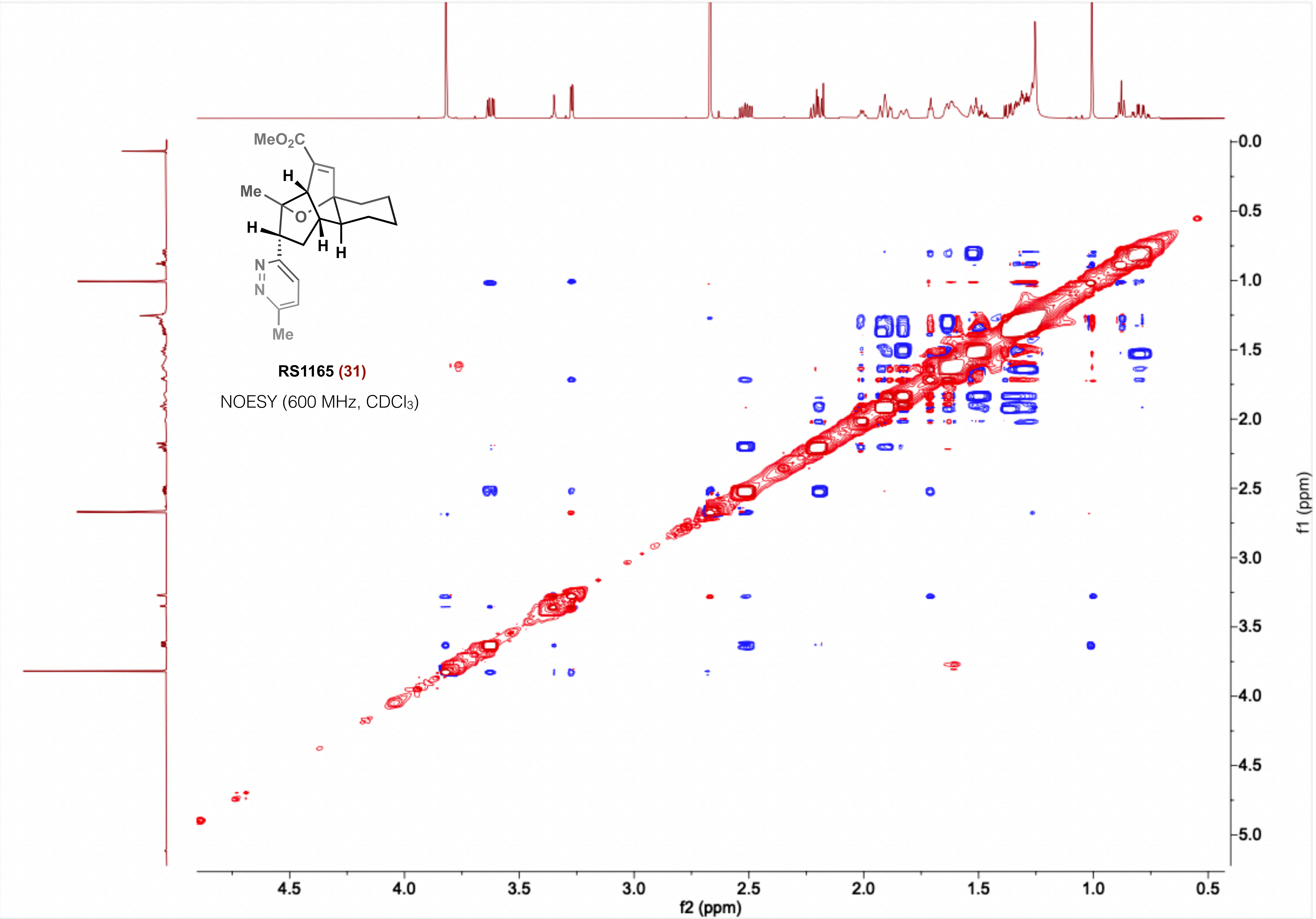

MeO2C
Me
H
O
H
H
H
N
N
Me
RS1165 (31)
NOESY (600 MHz, CDCl3)
f2 (ppm)
f1 (ppm)

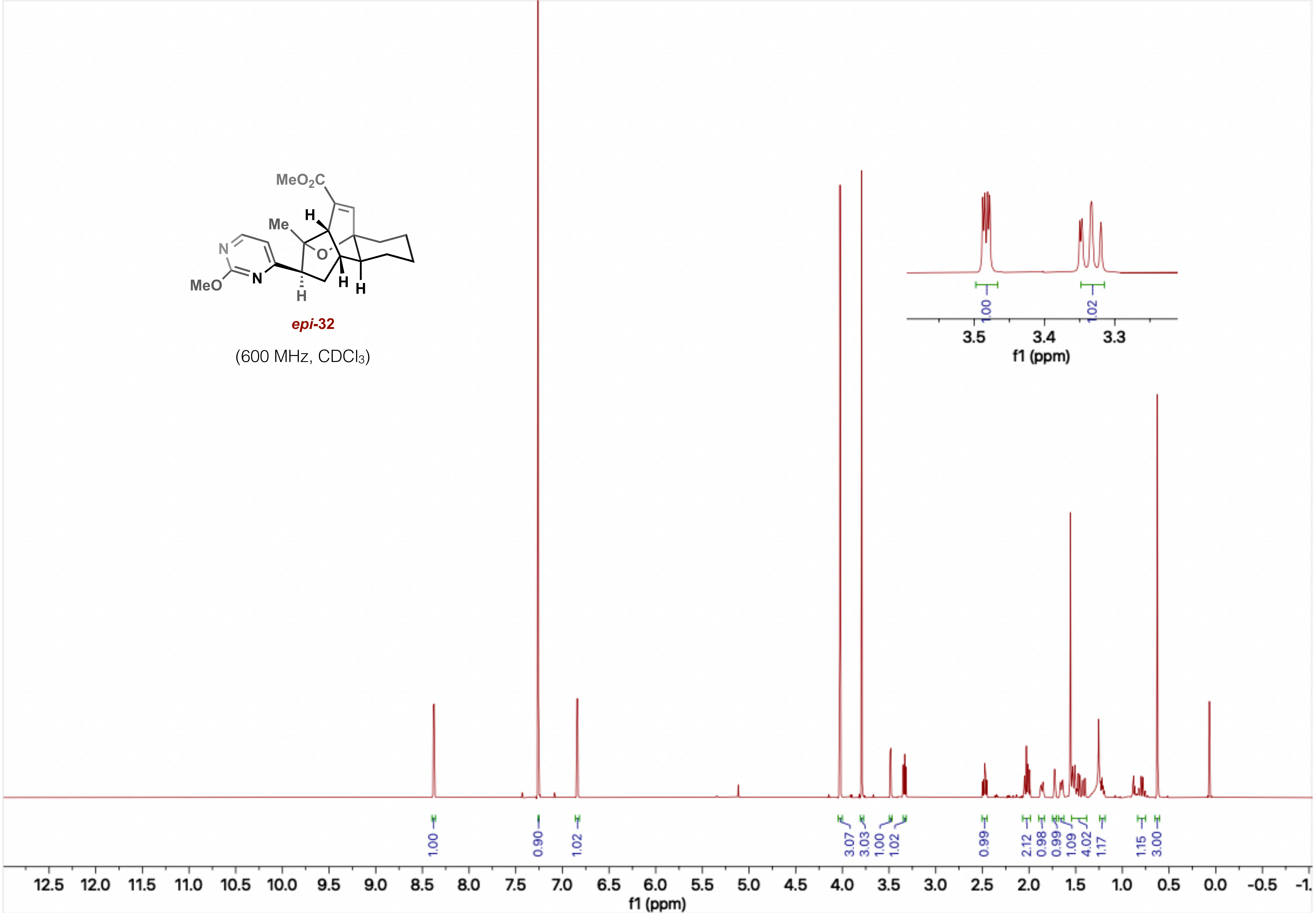

MeO2C
Me
H
O
N
MeO
N
H
H
H
epi-32
(600 MHz, CDCl3)
3.5
3.4
3.3
f1 (ppm)
1.00
1.02
1.00
0.90
1.02
3.07
3.03
1.00
1.02
0.99
2.12
0.98
0.99
1.09
4.02
1.17
1.15
3.00
12.5 12.0 11.5 11.0 10.5 10.0 9.5 9.0 8.5 8.0 7.5 7.0 6.5 6.0 5.5 5.0 4.5 4.0 3.5 3.0 2.5 2.0 1.5 1.0 0.5 0.0 -0.5 -1.
f1 (ppm)

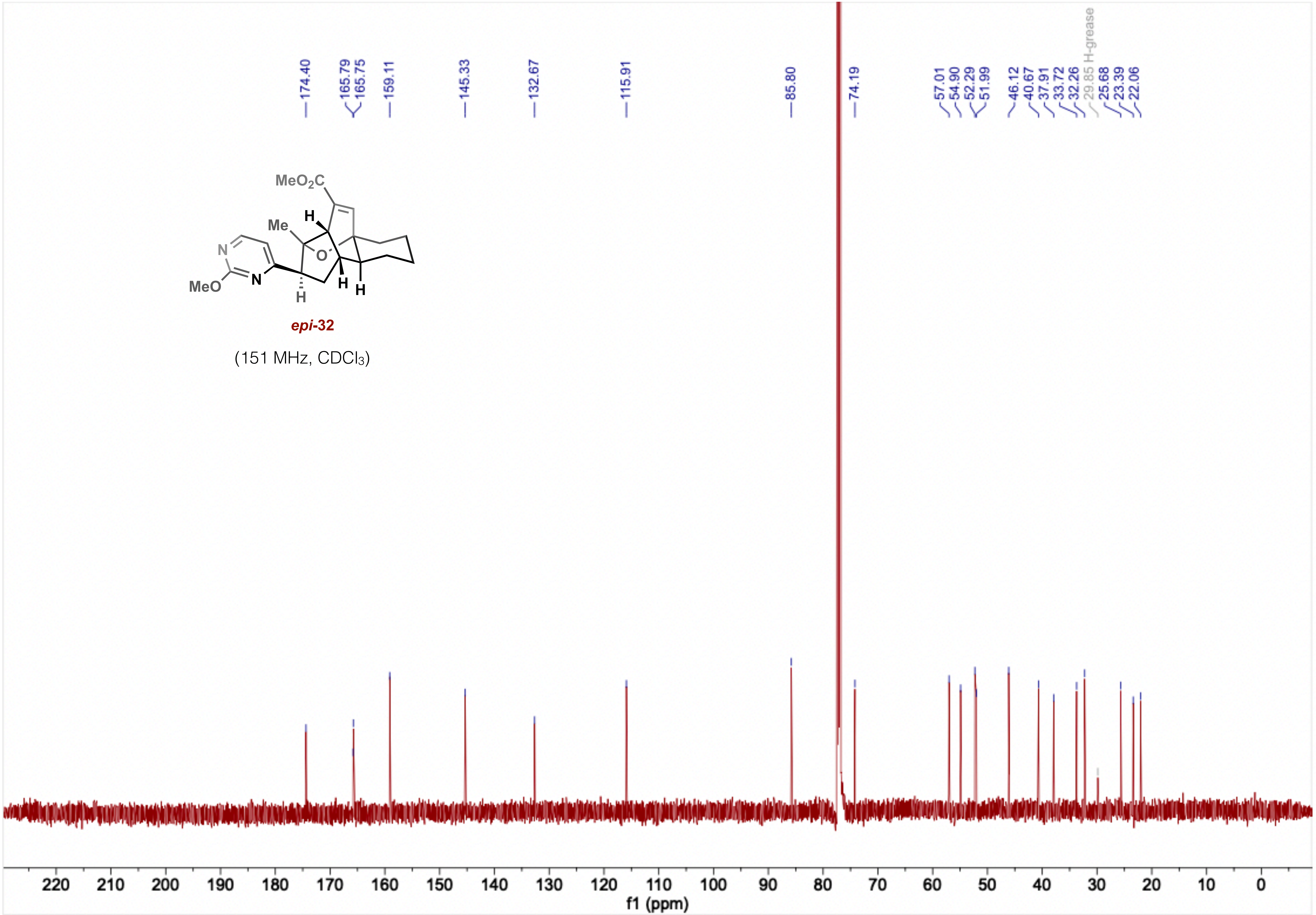

174.40
165.79
165.75
159.11
145.33
132.67
115.91
85.80
74.19
57.01
54.90
52.29
51.99
46.12
40.67
37.91
33.72
32.26
29.85 H-grease
25.68
23.39
22.06
MeO2C
Me
H
N
O
MeO
N
H
H
H
epi-32
(151 MHz, CDCl3)
220 210 200 190 180 170 160 150 140 130 120 110 100 90 80 70 60 50 40 30 20 10 0
f1 (ppm)

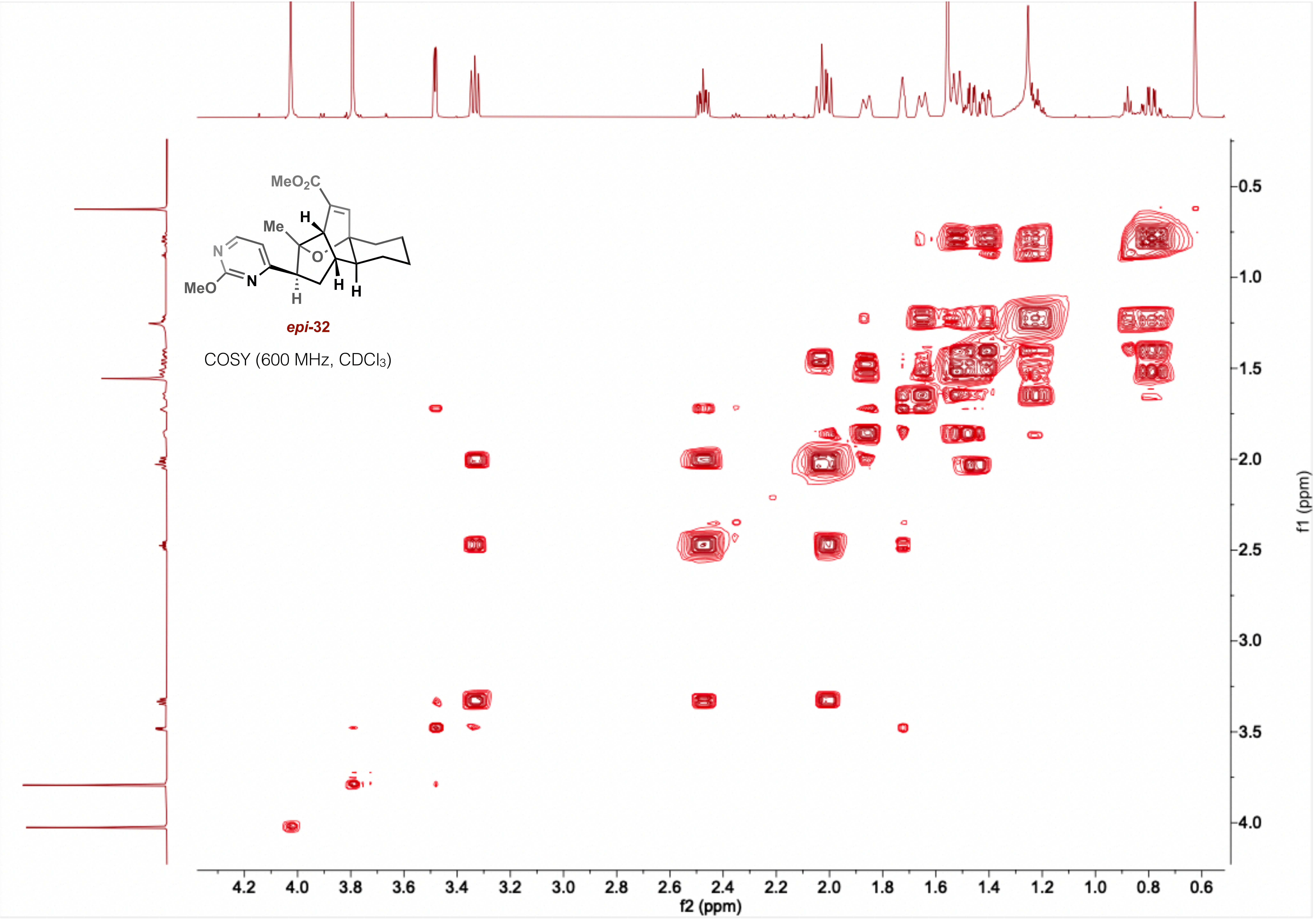
MeO2C
Me
H
N
O
MeO
N
H
H
H
epi-32
COSY (600 MHz, CDCl3)
f1 (ppm)
f2 (ppm)
4.2
4.0
3.8
3.6
3.4
3.2
3.0
2.8
2.6
2.4
2.2
2.0
1.8
1.6
1.4
1.2
1.0
0.8
0.6
0.5
1.0
1.5
2.0
2.5
3.0
3.5
4.0

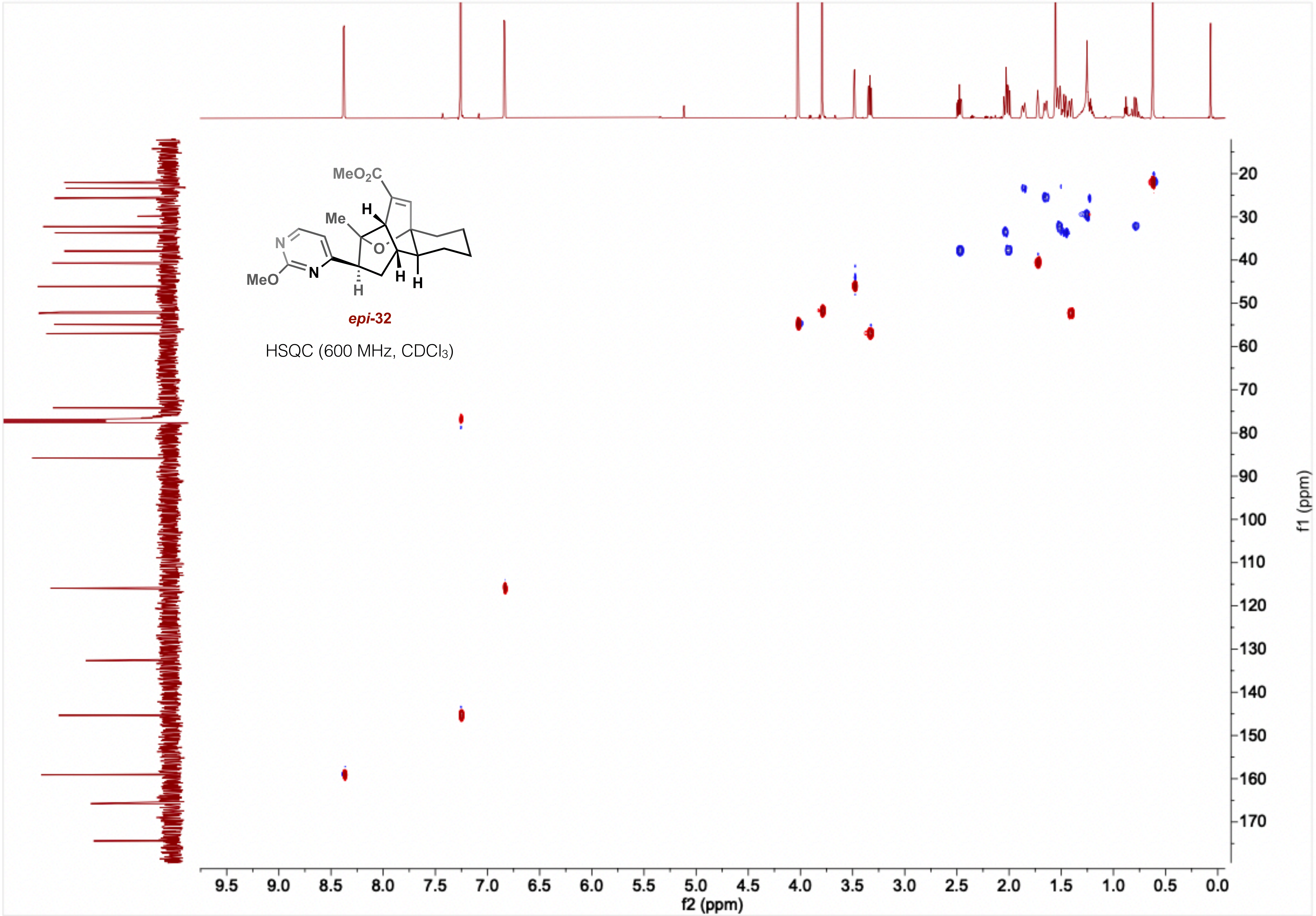

MeO2C
Me
H
N
MeO
N
O
H
H
H
epi-32
HSQC (600 MHz, CDCl3)
f1 (ppm)
f2 (ppm)

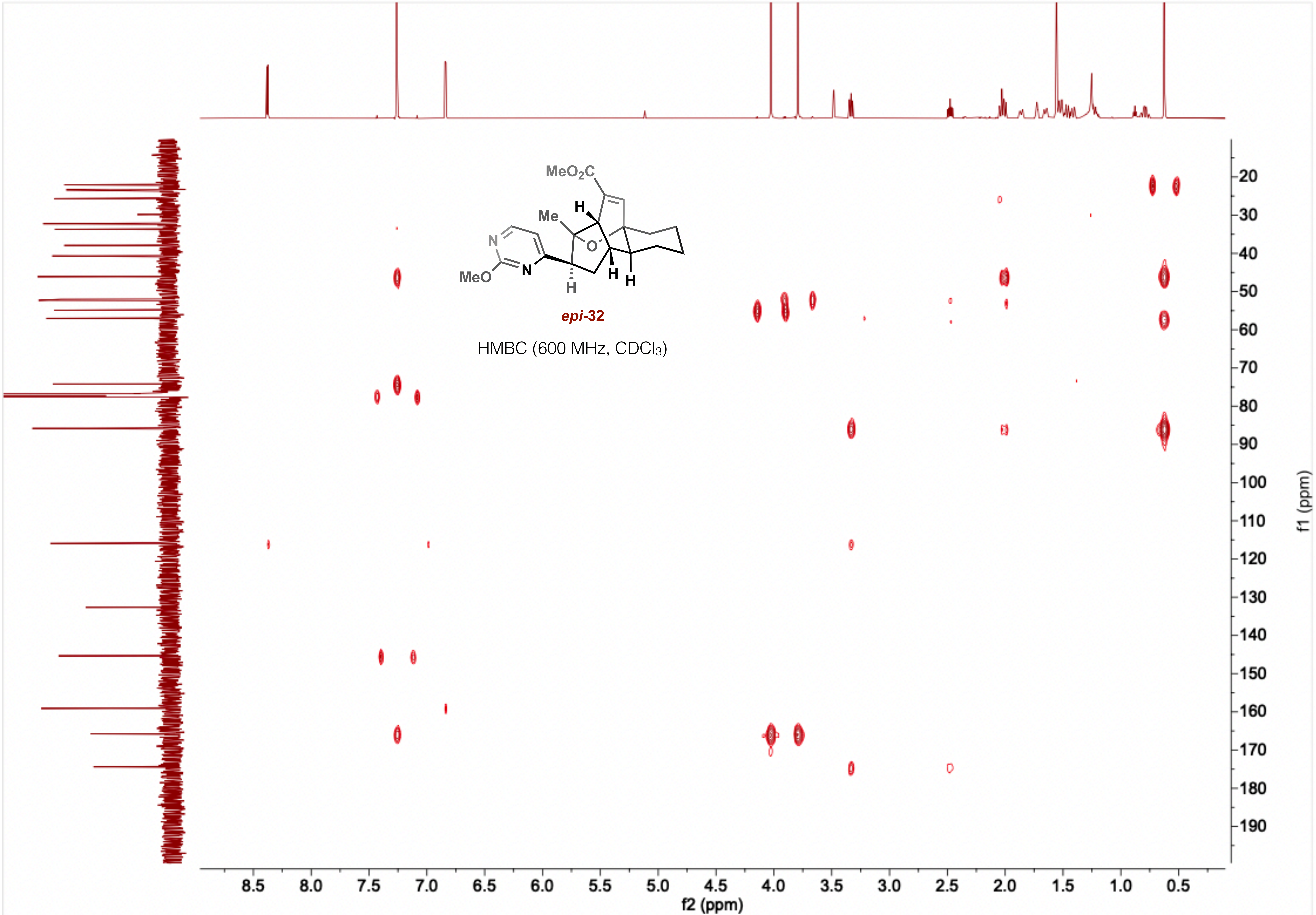
MeO2C
Me
H
N
O
MeO
N
H
H
H
epi-32
HMBC (600 MHz, CDCl3)
f2 (ppm)
f1 (ppm)

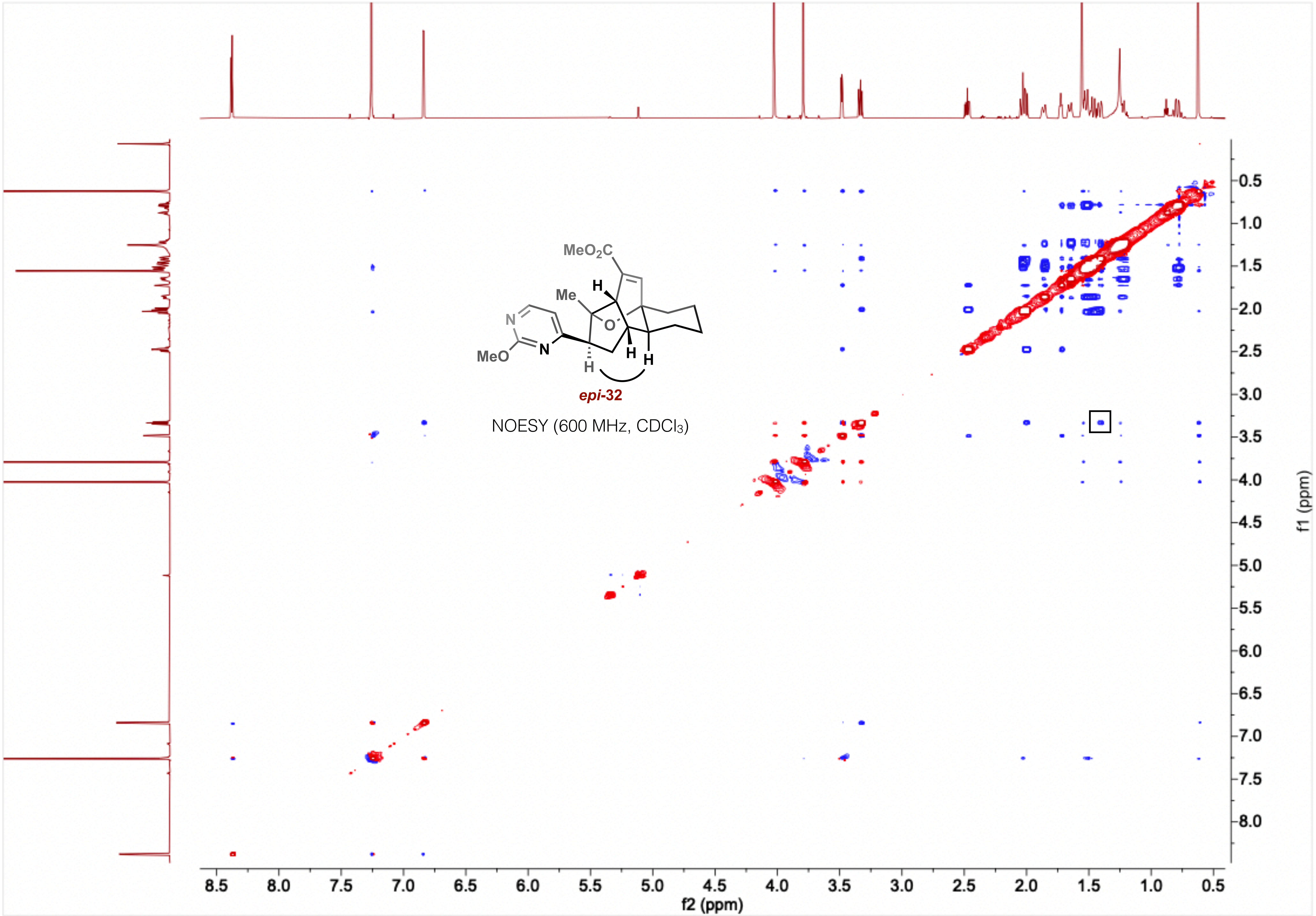

MeO2C
Me
H
O
N
MeO
N
H
H
H
epi-32
NOESY (600 MHz, CDCl3)
f2 (ppm)
f1 (ppm)

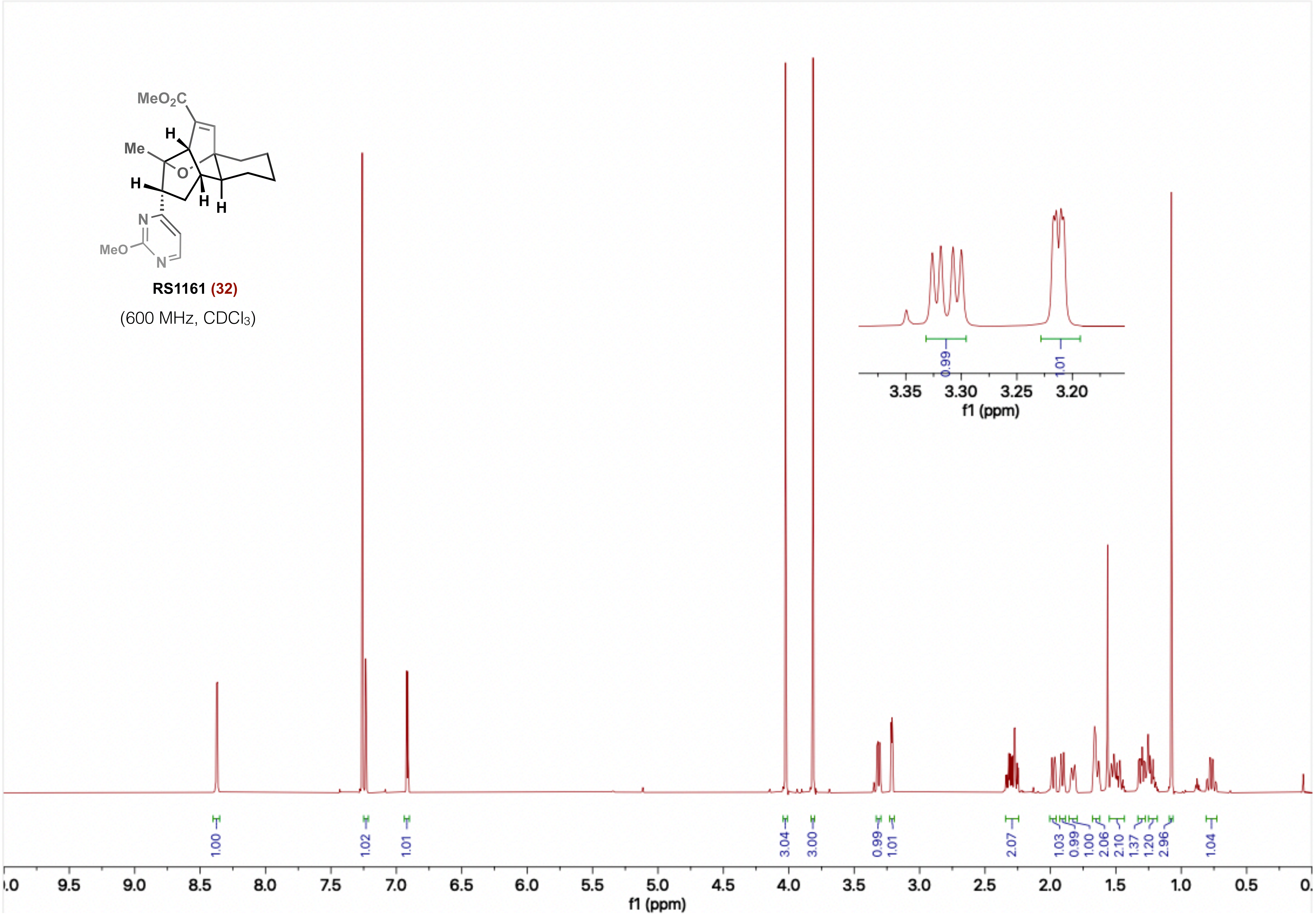
MeO2C
Me
H
H
O
H
H
N
MeO
N
RS1161 (32)
(600 MHz, CDCl3)
3.35 3.30 3.25 3.20
f1 (ppm)
0.99
1.01
1.00
1.02
1.01
3.04
3.00
0.99
1.01
2.07
1.03
0.99
1.00
2.06
2.10
1.37
1.20
2.96
1.04
9.5 9.0 8.5 8.0 7.5 7.0 6.5 6.0 5.5 5.0 4.5 4.0 3.5 3.0 2.5 2.0 1.5 1.0 0.5
f1 (ppm)

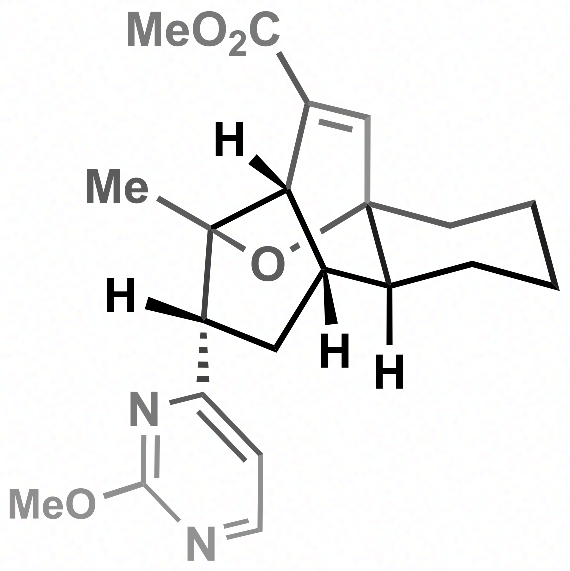

RS1161 **(32)**

(151 MHz, $CDCl_3$)

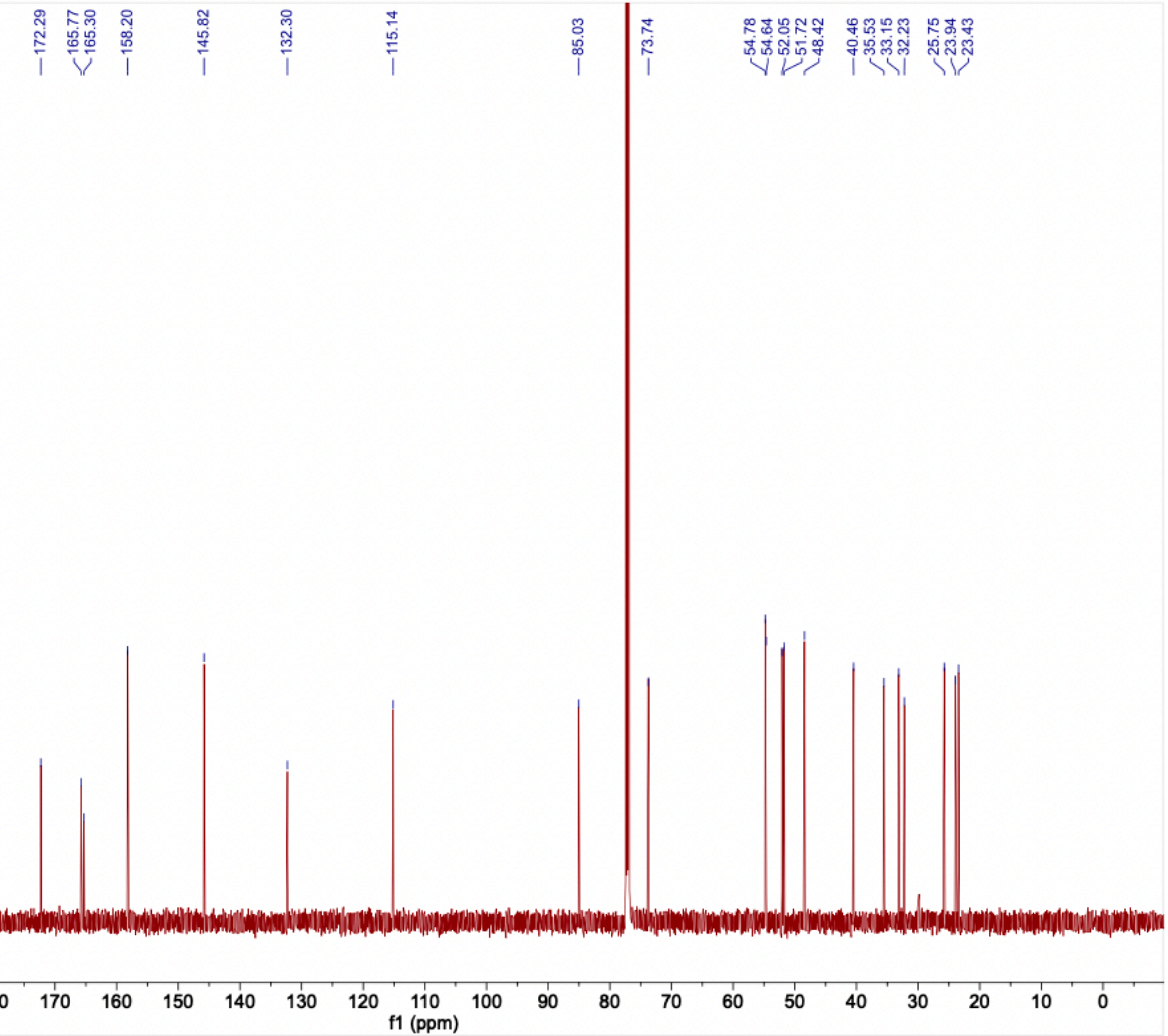

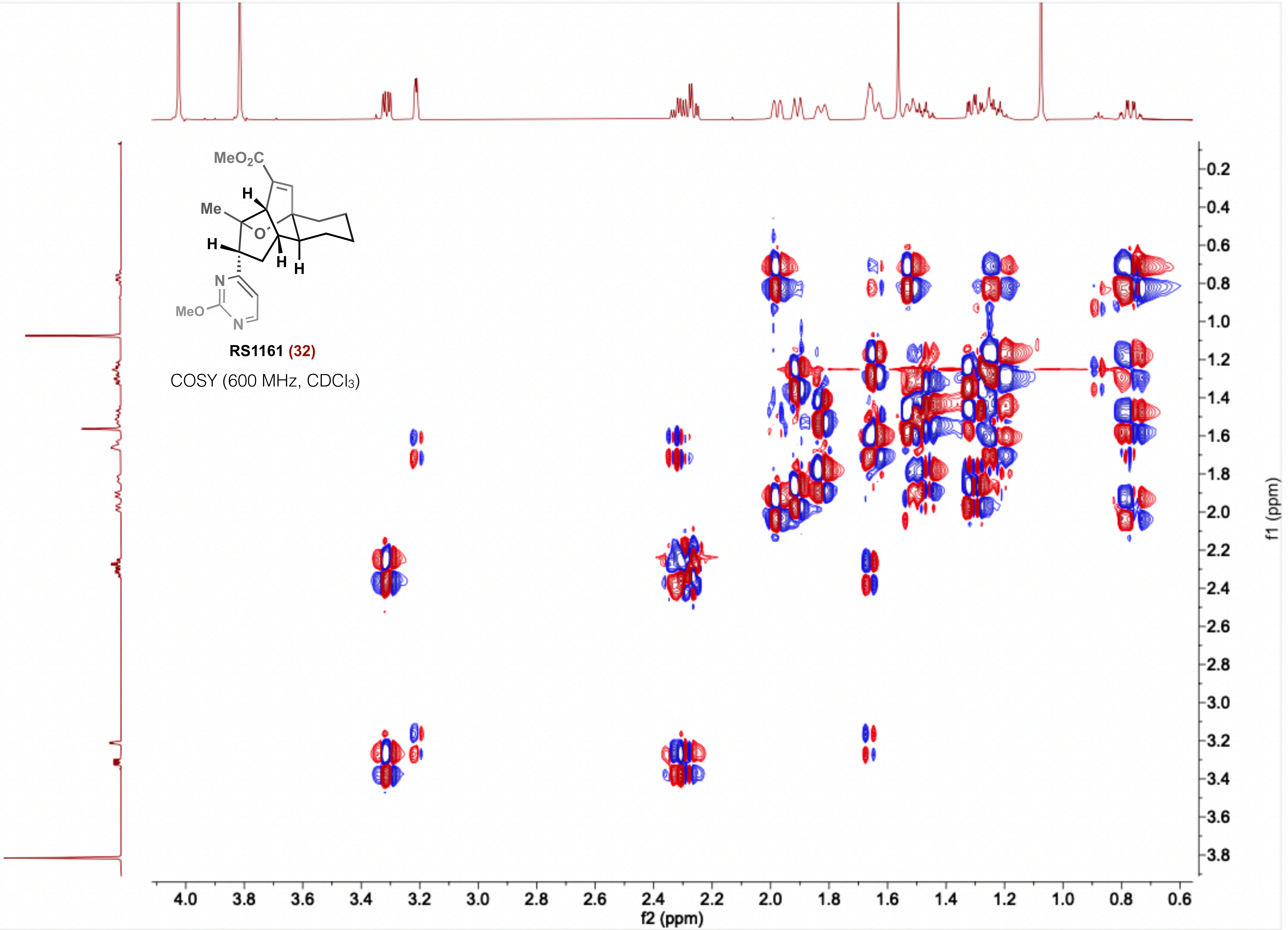

MeO2C
Me
H
O
H
H
H
N
MeO
N
RS1161 (32)
COSY (600 MHz, CDCl3)
f1 (ppm)
f2 (ppm)
4.0
3.8
3.6
3.4
3.2
3.0
2.8
2.6
2.4
2.2
2.0
1.8
1.6
1.4
1.2
1.0
0.8
0.6
0.2
0.4

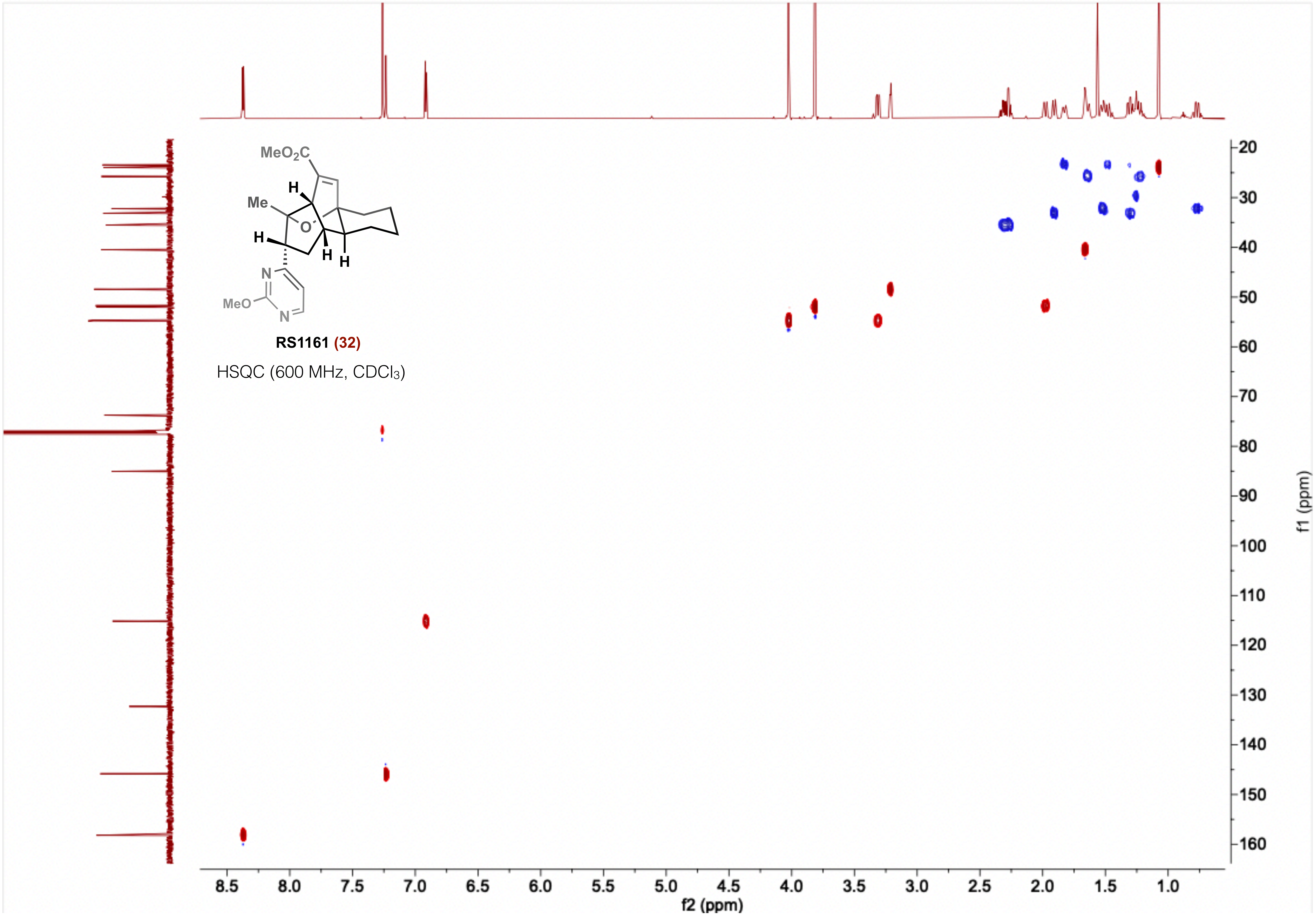

MeO2C
H
Me
O
H
H
H
N
MeO
N
RS1161 (32)
HSQC (600 MHz, CDCl3)
f1 (ppm)
f2 (ppm)

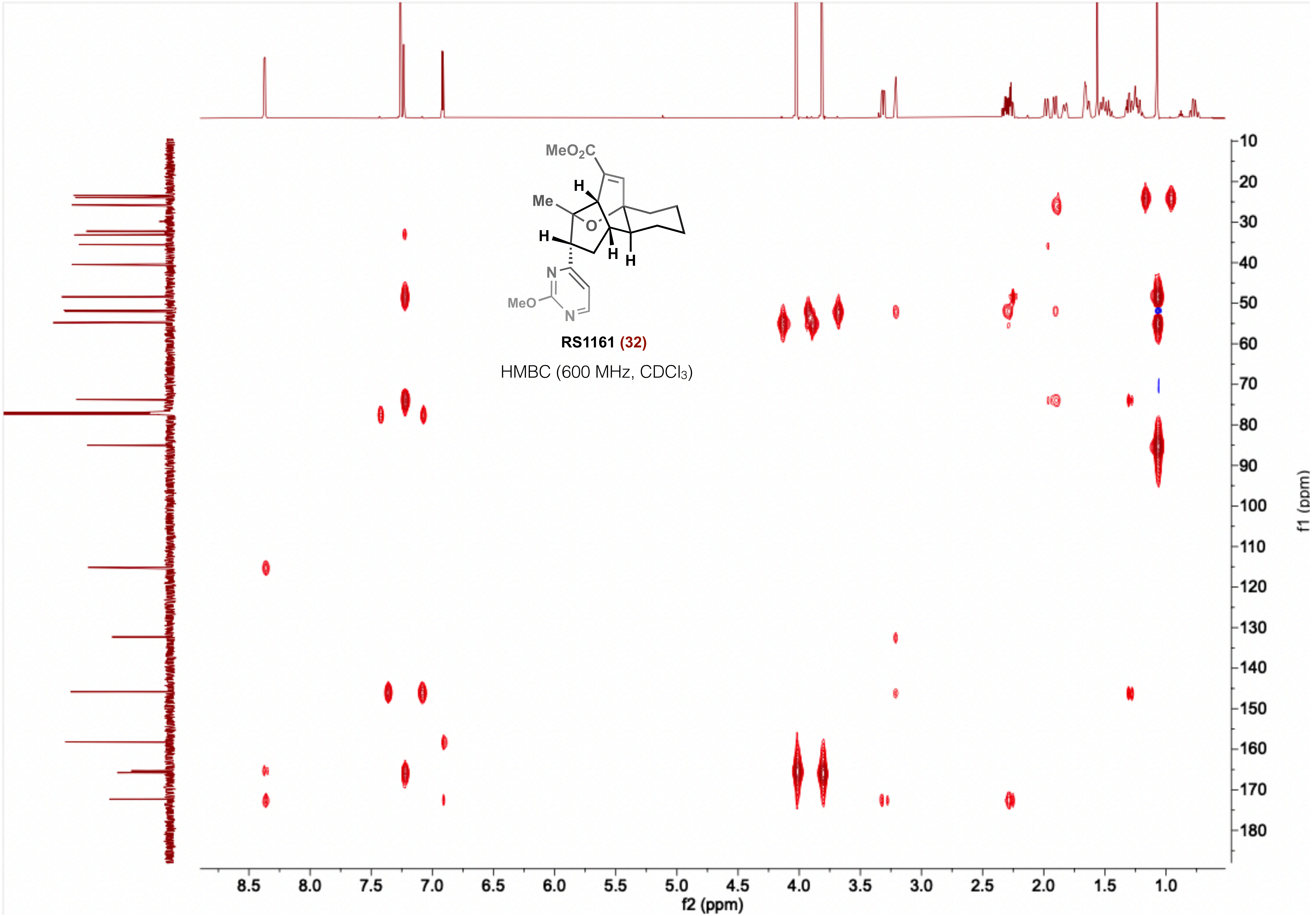
MeO2C
H
Me
O
H
H
H
N
MeO
N
RS1161 (32)
HMBC (600 MHz, CDCl3)
f2 (ppm)
f1 (ppm)

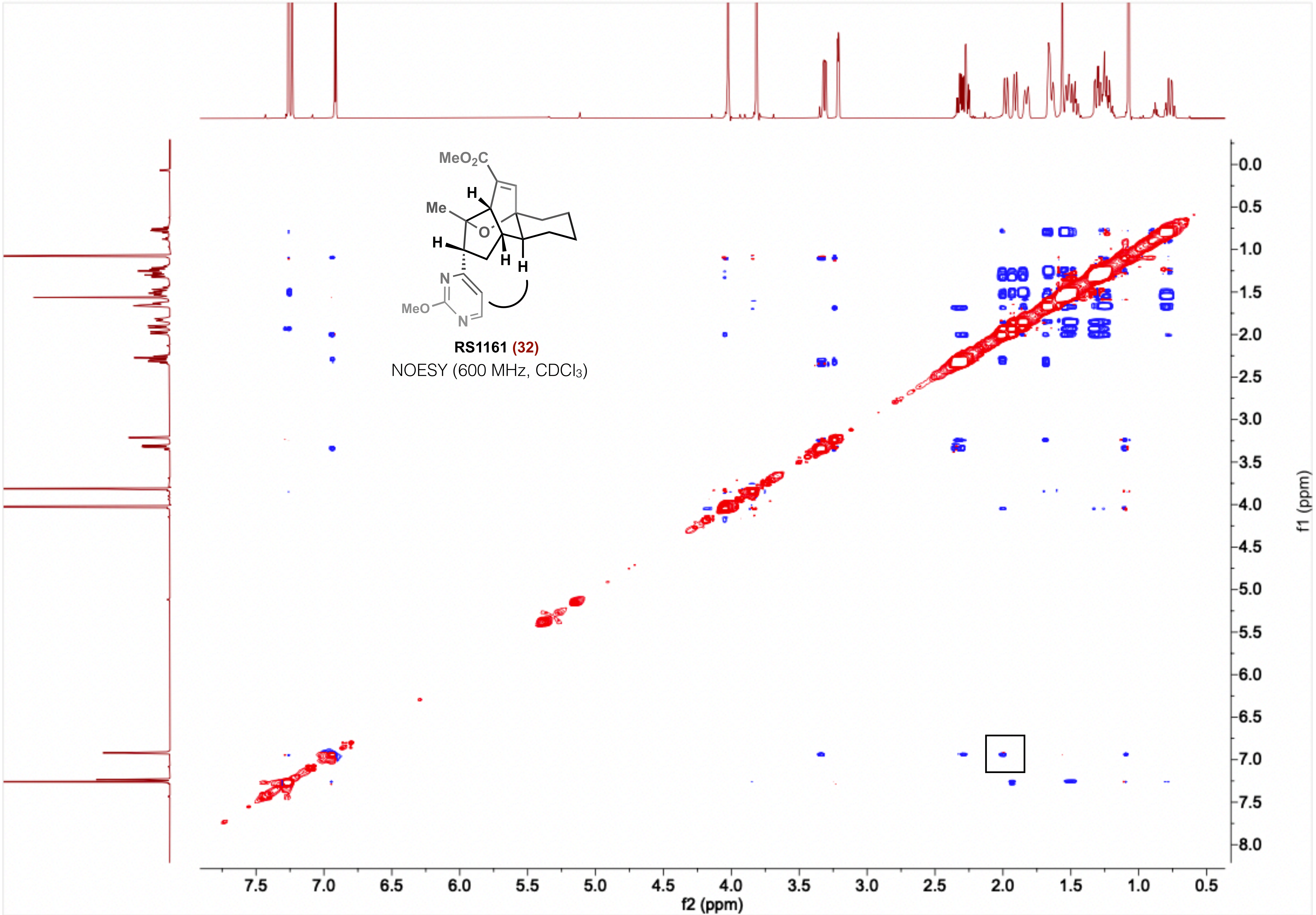
MeO2C
Me
H
O
H
H
H
N
MeO
N
RS1161 (32)
NOESY (600 MHz, CDCl3)
f2 (ppm)
f1 (ppm)

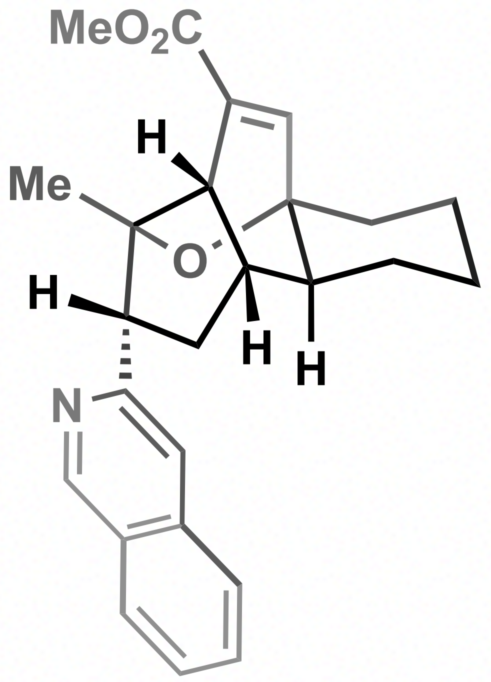

RS1162 **(33)**

(600 MHz, CDCl3)

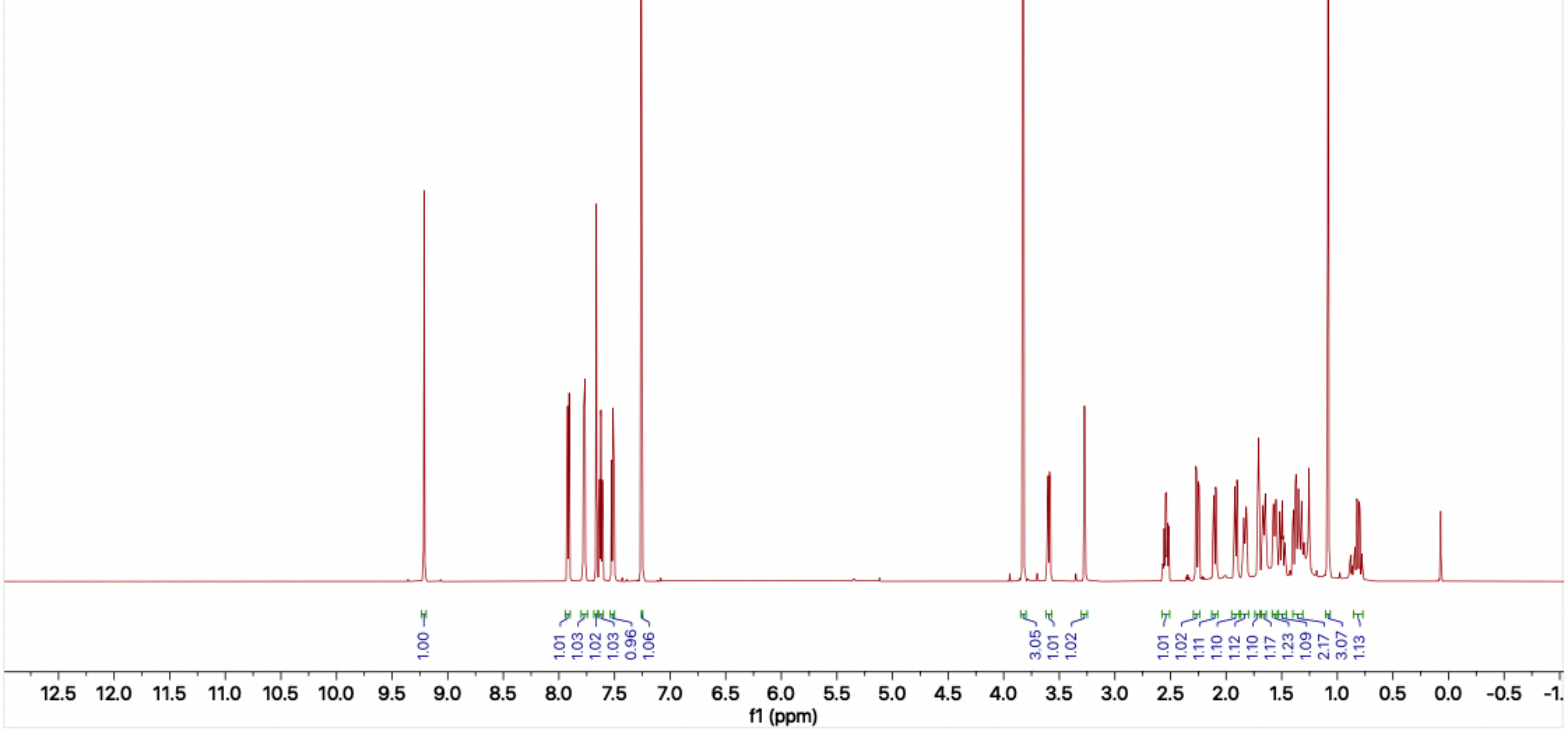

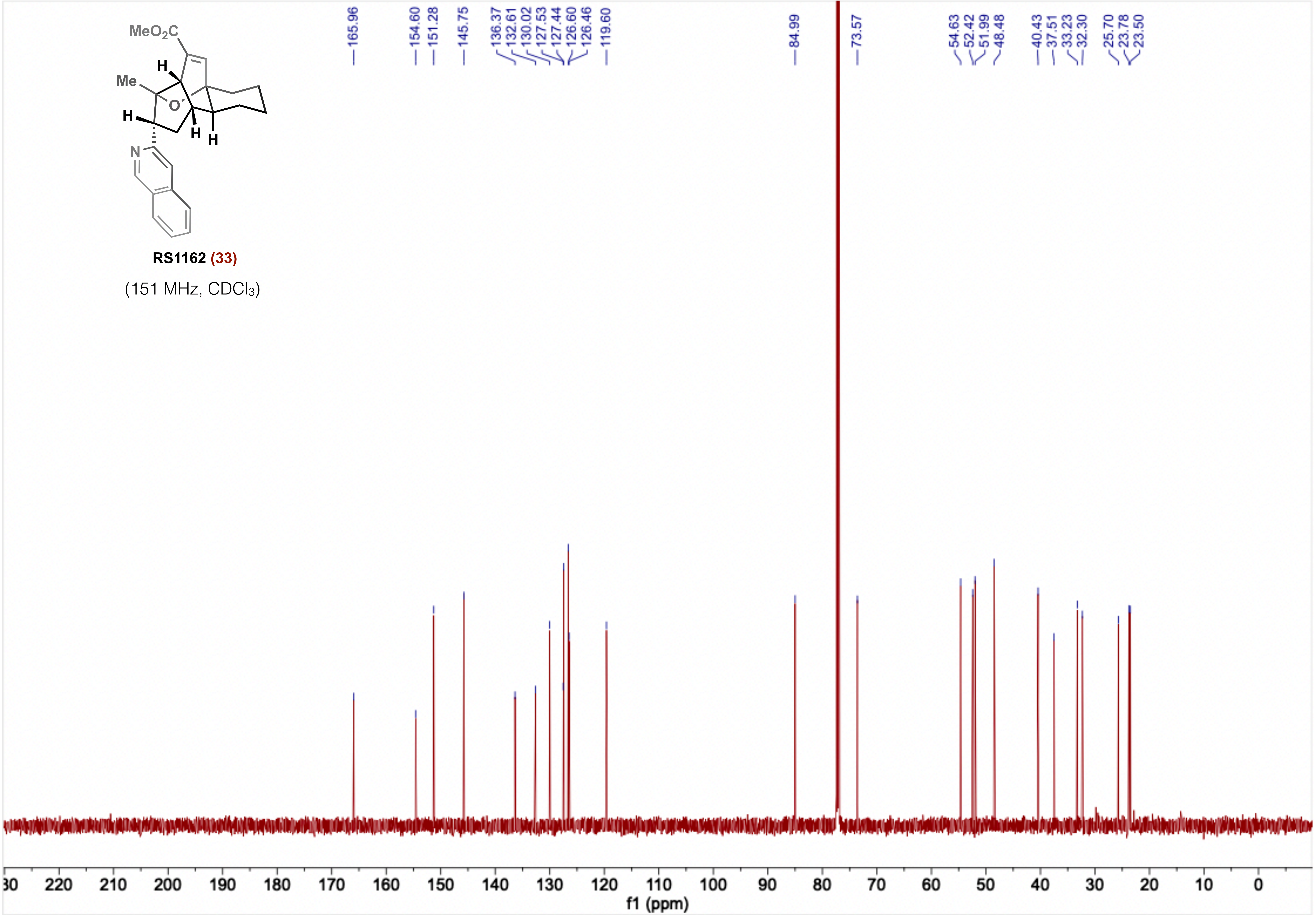

RS1162 (33)

(151 MHz, $CDCl_3$)

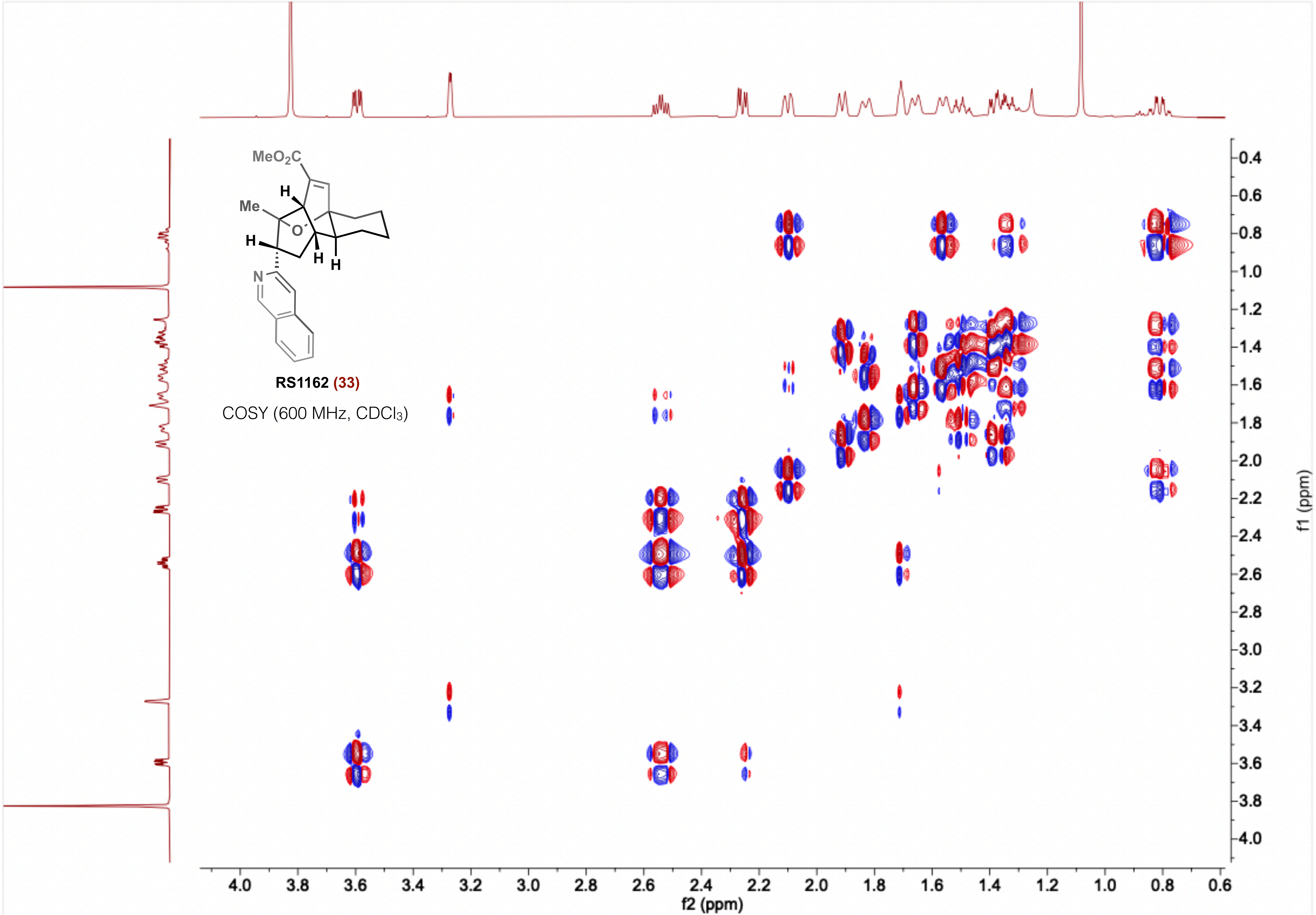

MeO2C
Me
H
O
N
RS1162 (33)
COSY (600 MHz, CDCl3)
f2 (ppm)
f1 (ppm)

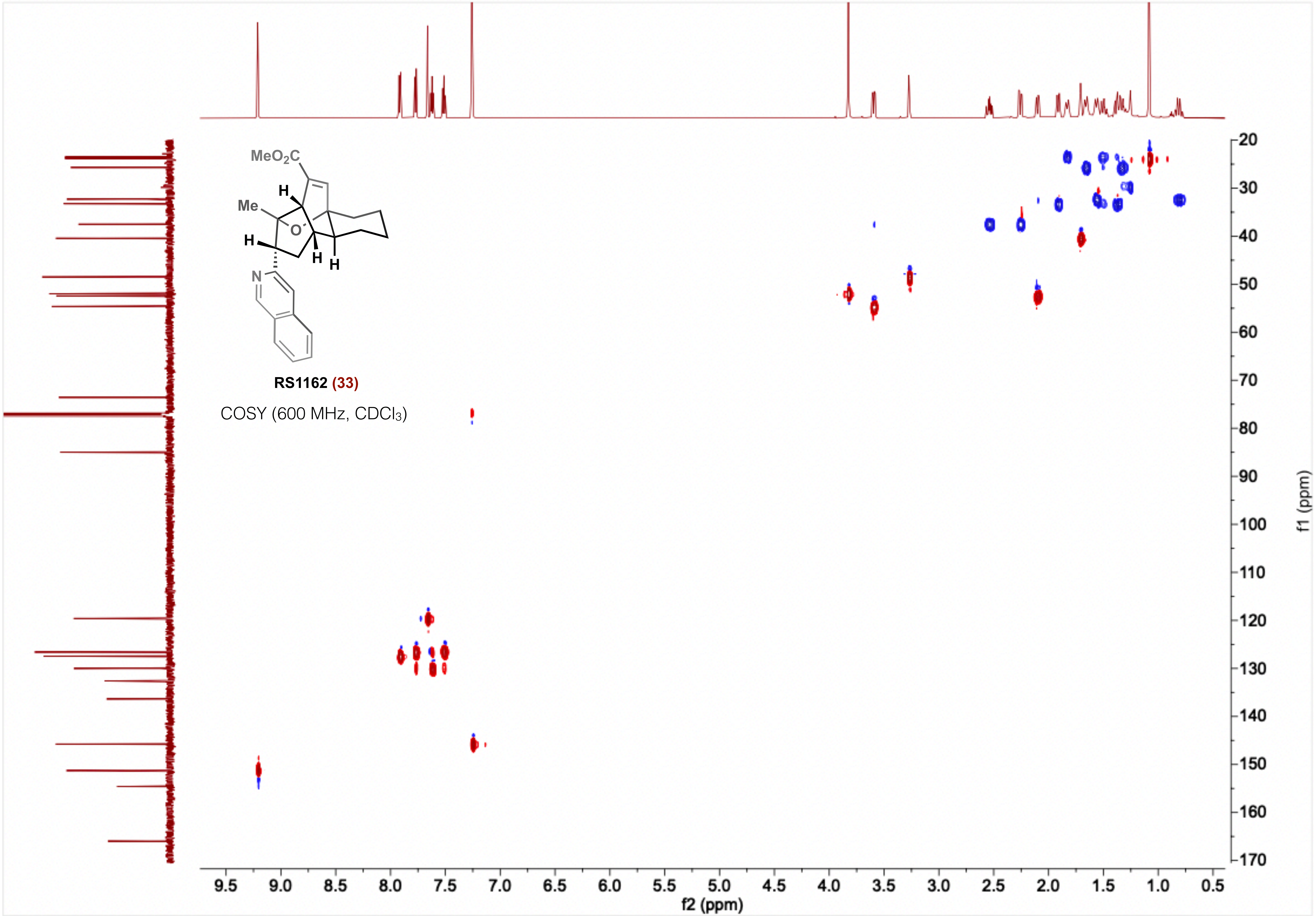

RS1162 (33)

COSY (600 MHz, $CDCl_3$)

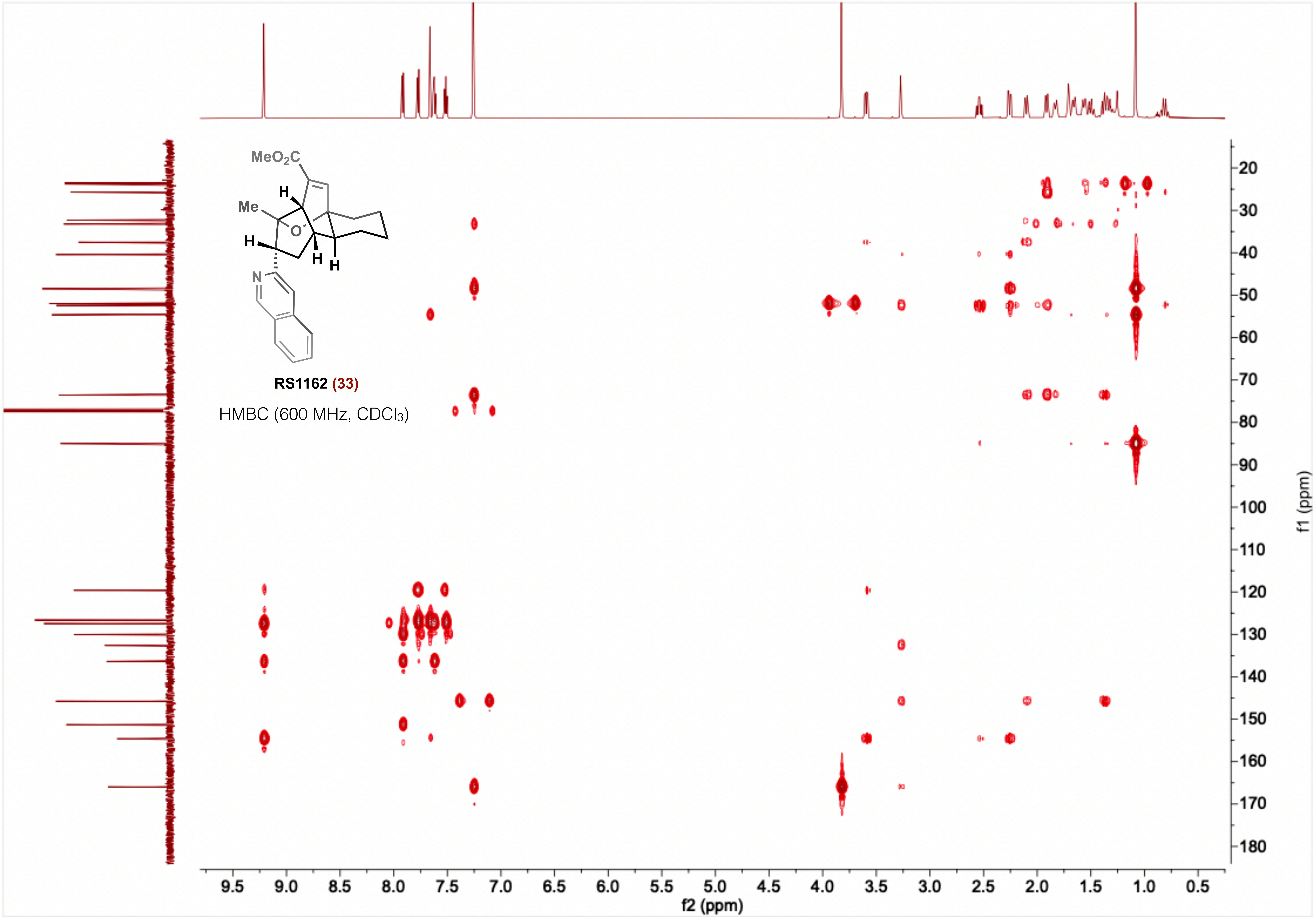
MeO2C
H
Me
O
H
H
H
N
RS1162 (33)
HMBC (600 MHz, CDCl3)
f2 (ppm)
f1 (ppm)

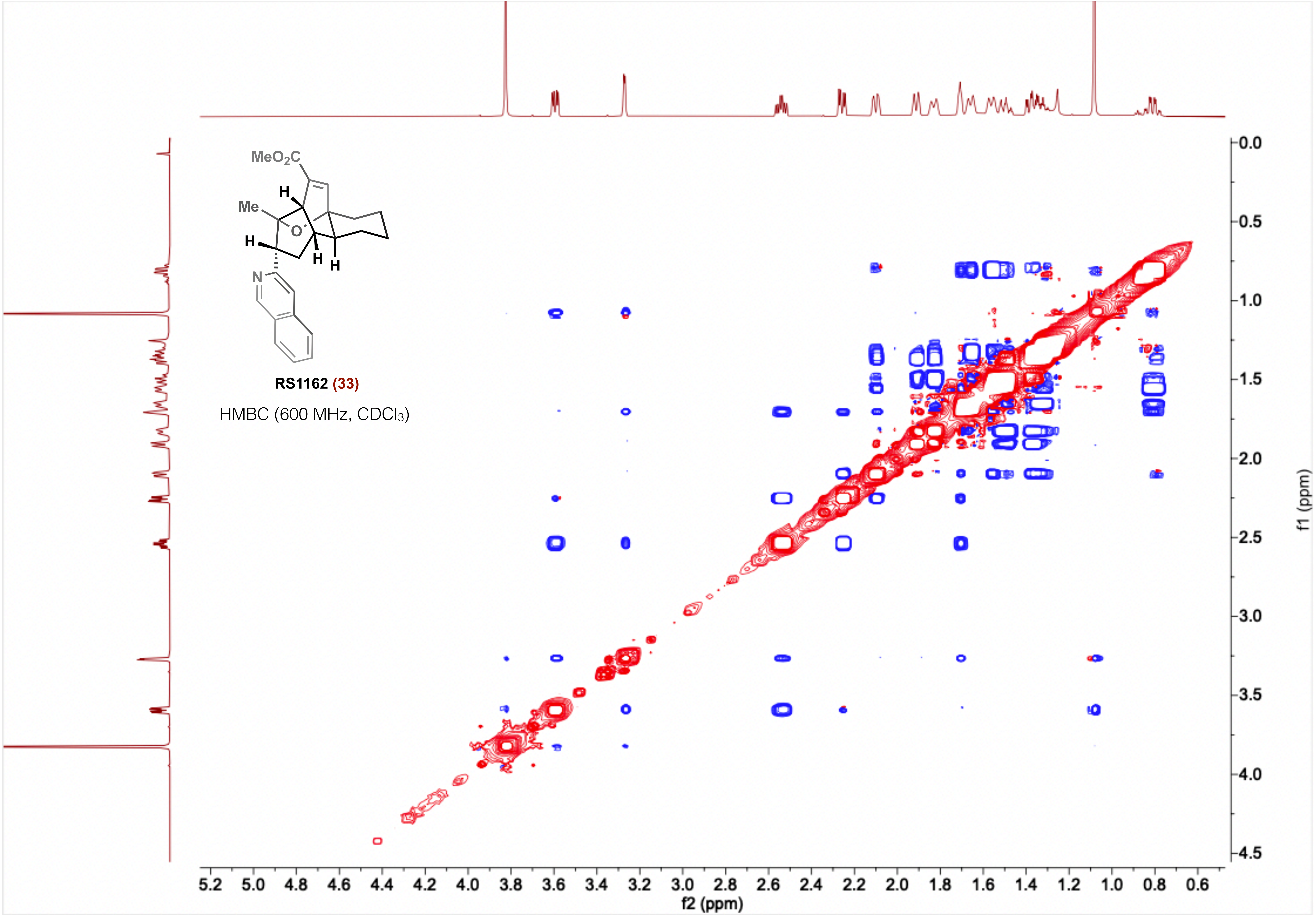
MeO2C
Me
H
O
H
H
H
N
RS1162 (33)
HMBC (600 MHz, CDCl3)
f2 (ppm)
f1 (ppm)

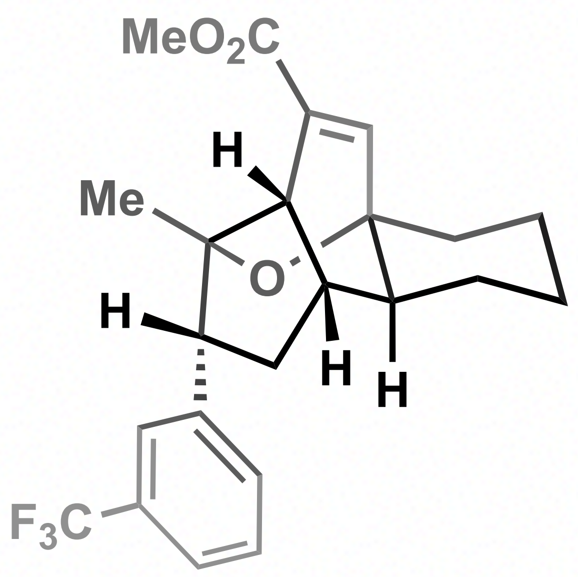

RS1188

(600 MHz, C_6D_6)

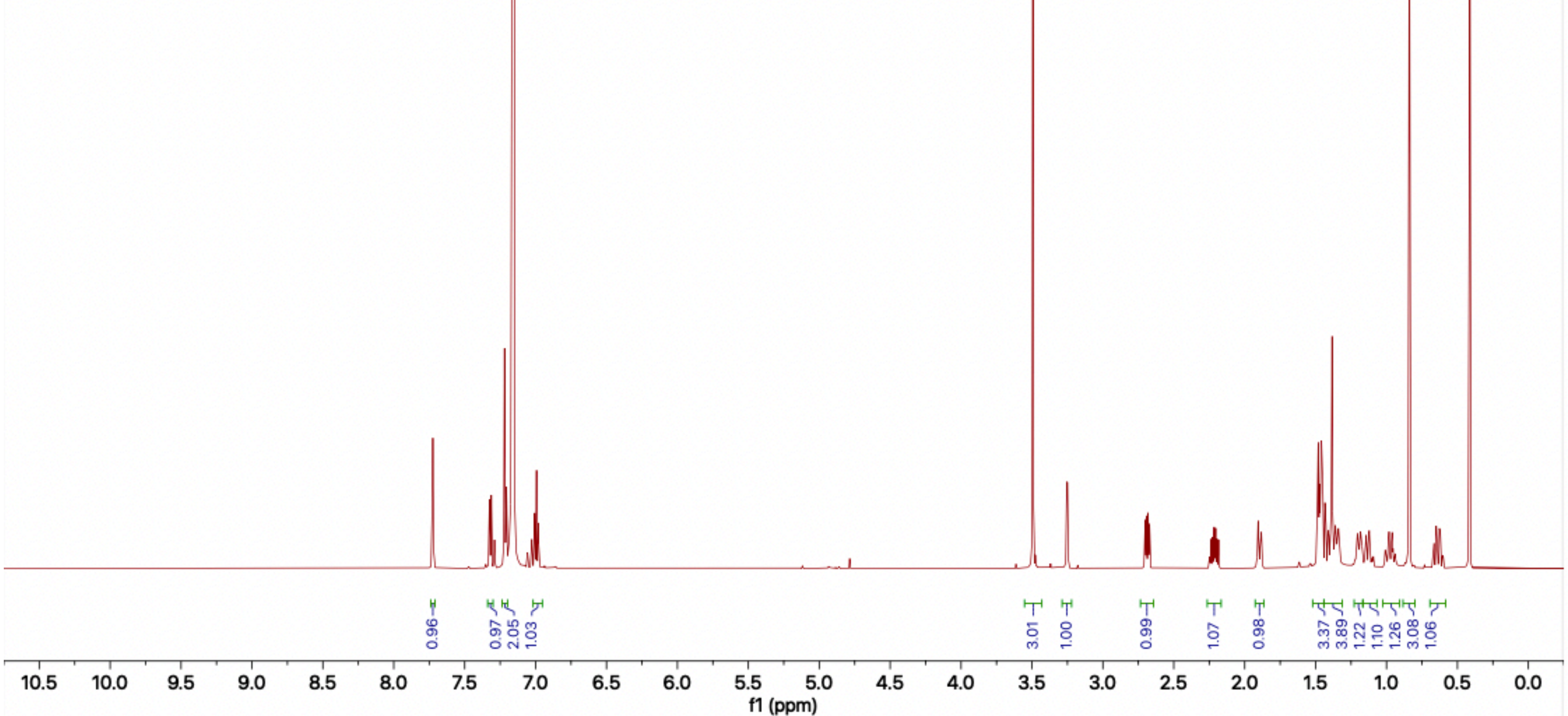

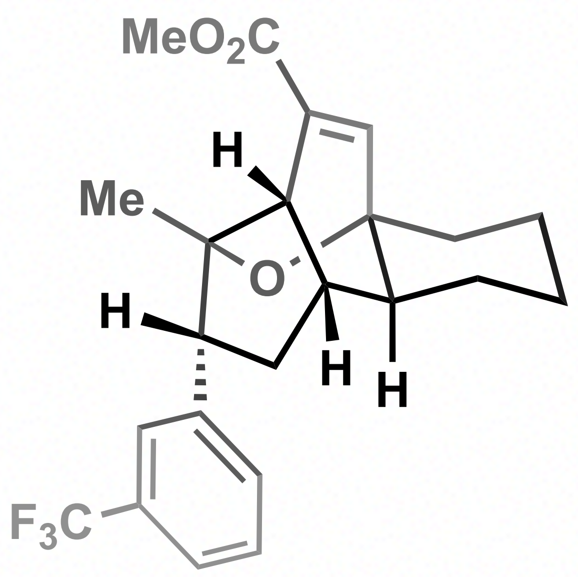

RS1188

(151 MHz, C_6D_6)

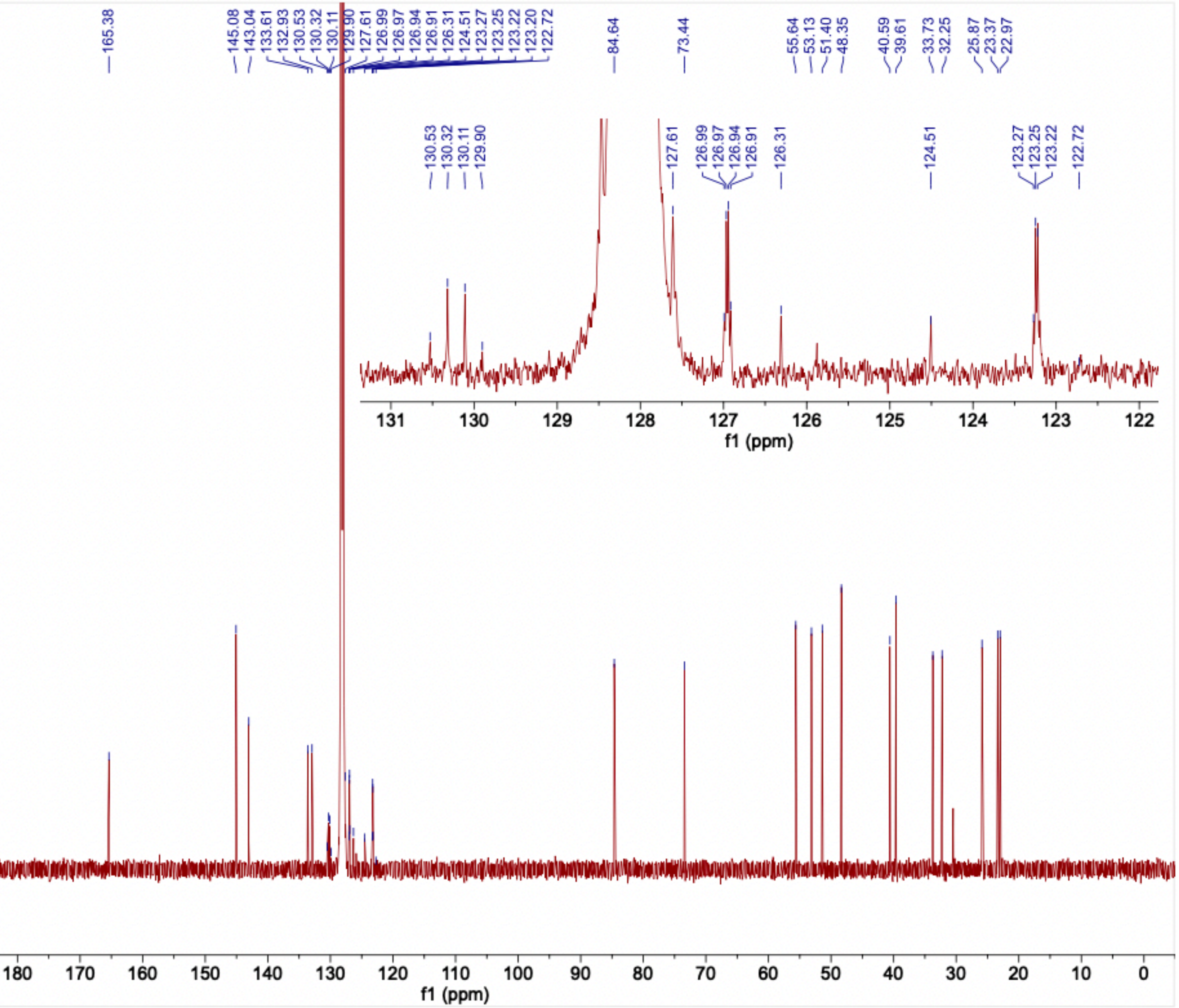

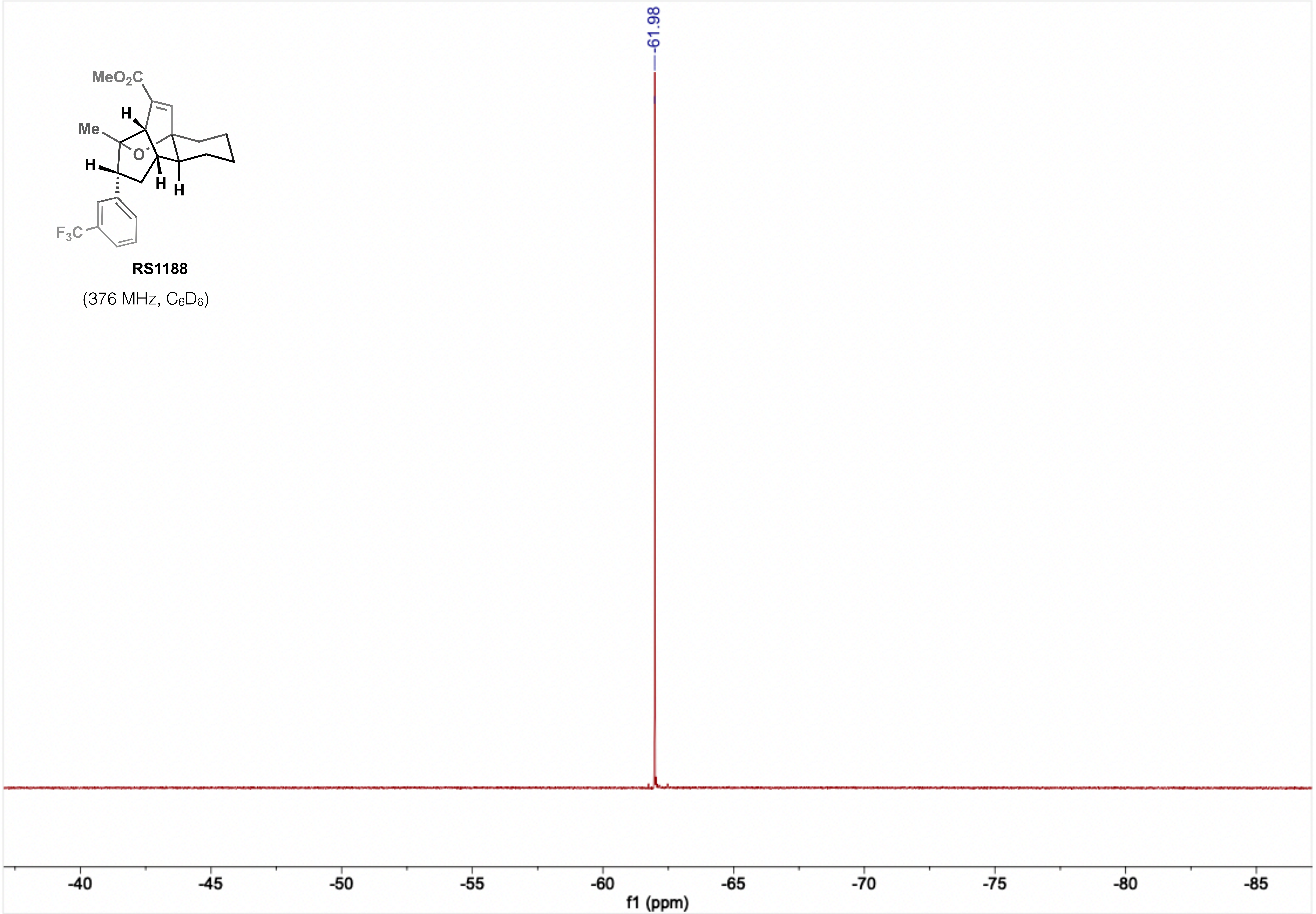

-61.98
MeO2C
Me
H
O
H
H
H
F3C
RS1188
(376 MHz, C6D6)
-40
-45
-50
-55
-60
-65
-70
-75
-80
-85
f1 (ppm)

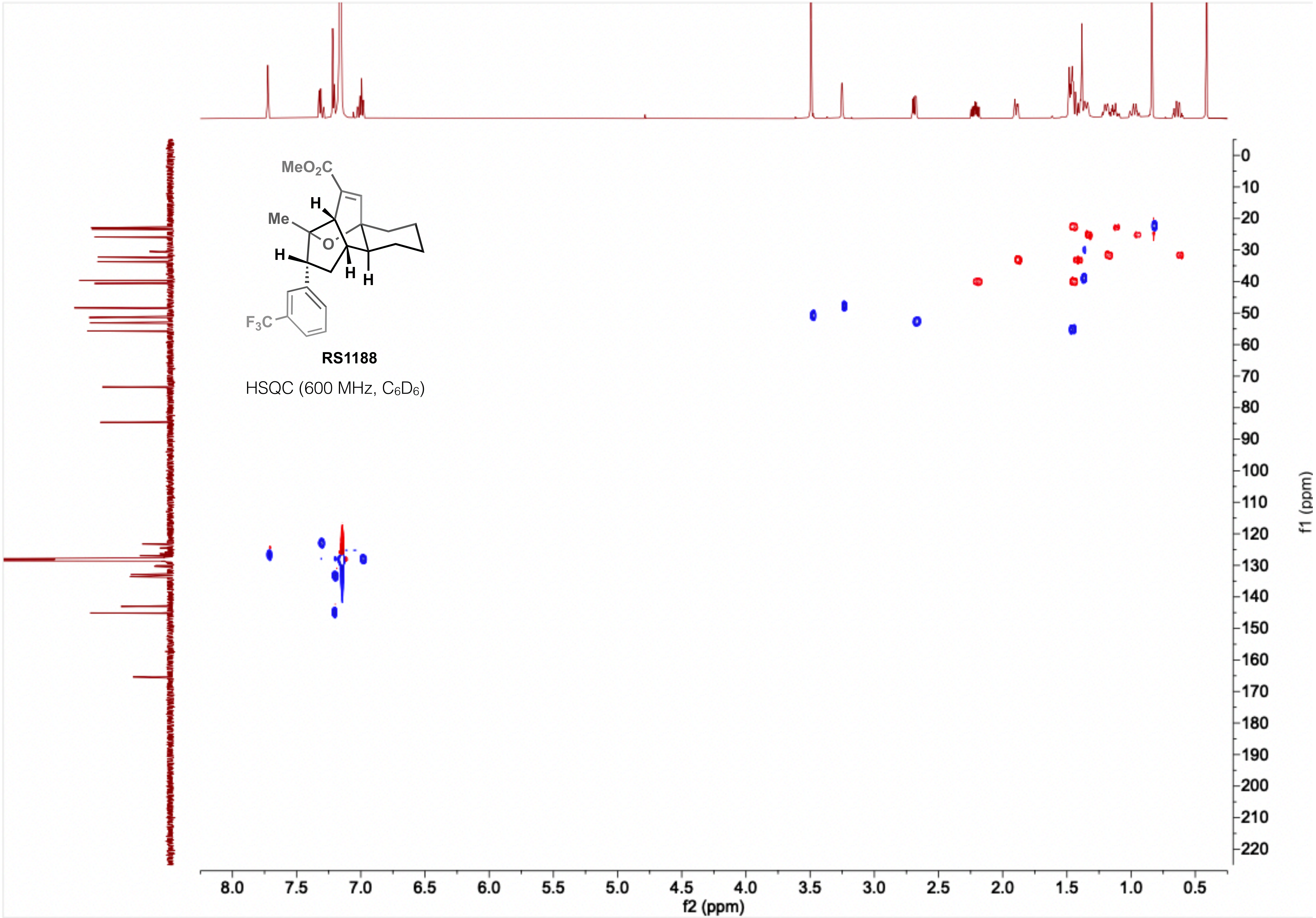
MeO2C
Me
H
O
H
H
H
F3C
RS1188
HSQC (600 MHz, C6D6)
f2 (ppm)
f1 (ppm)

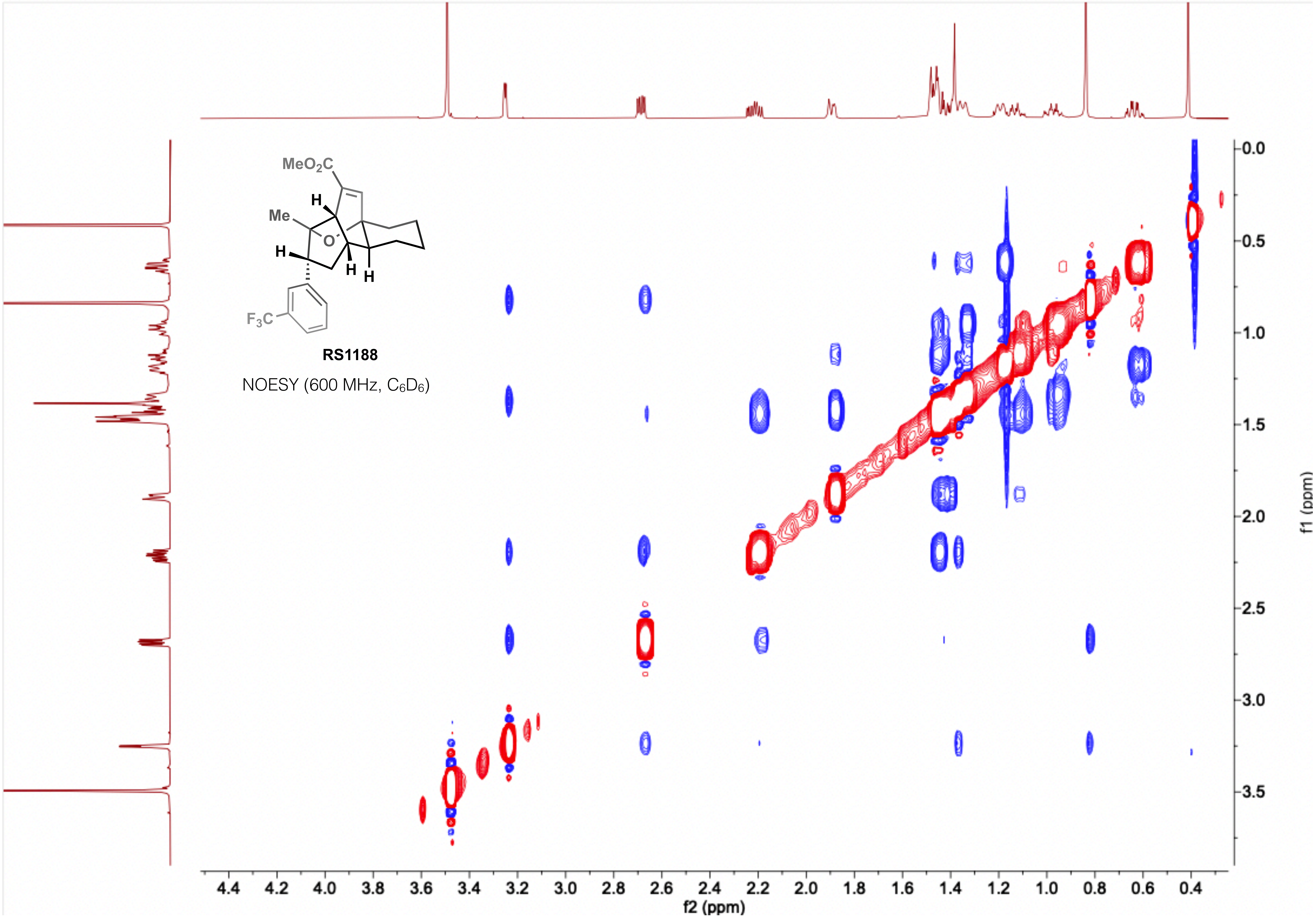
MeO2C
Me
H
O
H
H
H
F3C
RS1188
NOESY (600 MHz, C6D6)
f1 (ppm)
f2 (ppm)
0.0
0.5
1.0
1.5
2.0
2.5
3.0
3.5
4.4
4.2
4.0
3.8
3.6
3.4
3.2
3.0
2.8
2.6
2.4
2.2
2.0
1.8
1.6
1.4
1.2
1.0
0.8
0.6
0.4

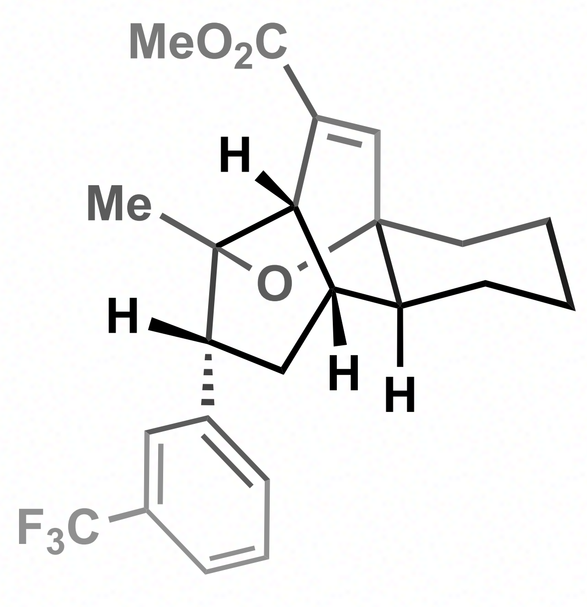

RS1188

(600 MHz, C_6D_6)

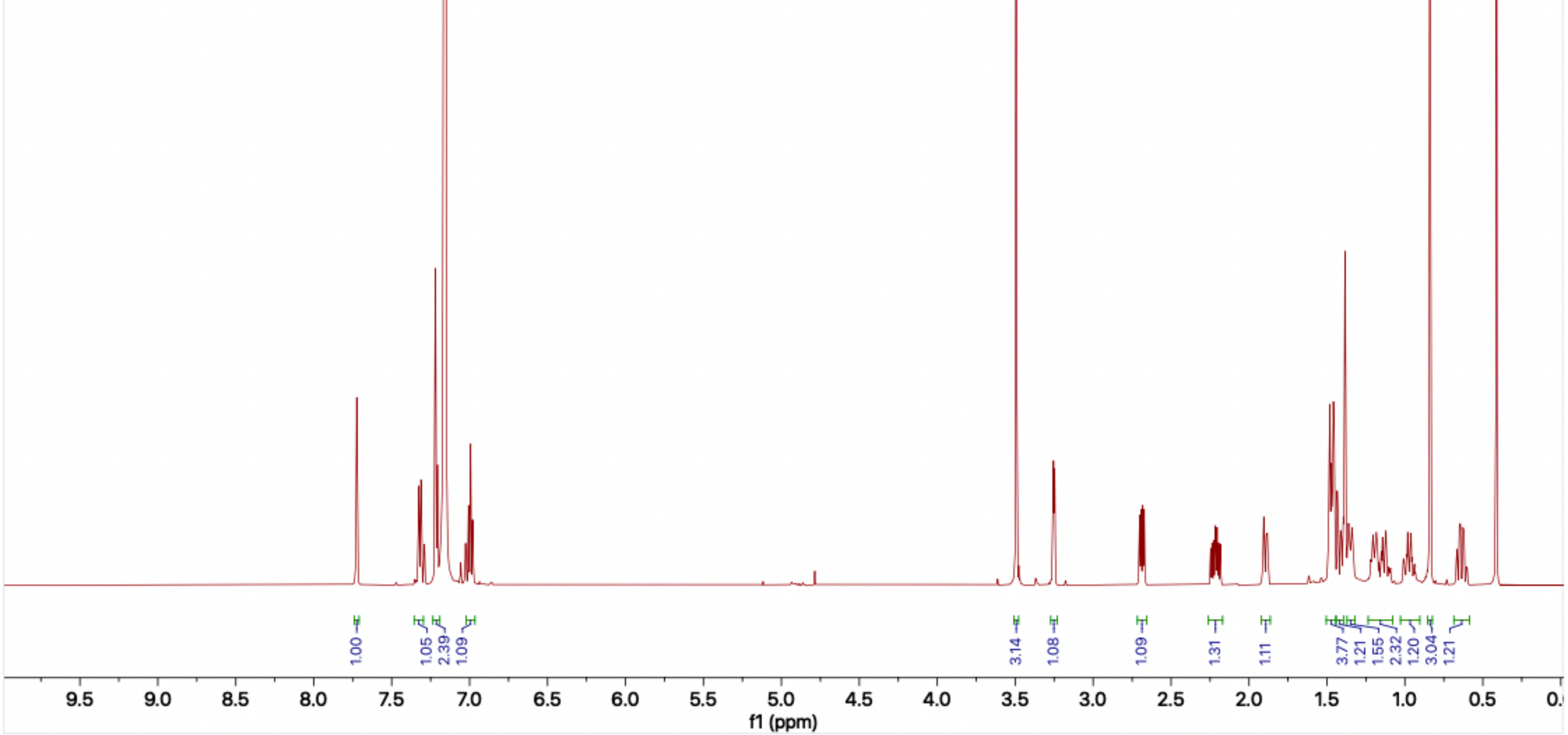

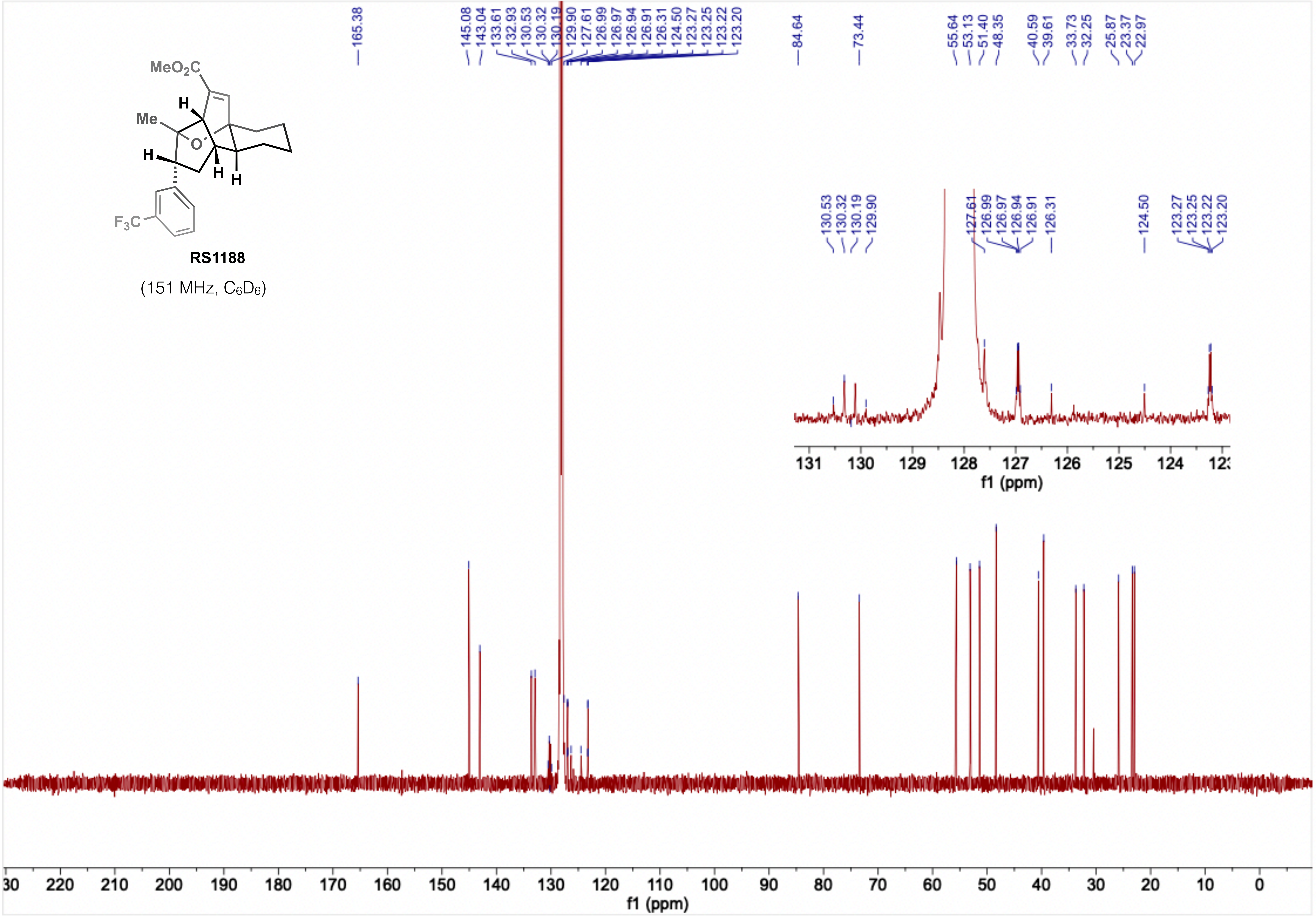
MeO2C
Me
H
O
H
H
H
F3C
RS1188
(151 MHz, C6D6)
165.38
145.08
143.04
133.61
132.93
130.53
130.32
130.19
129.90
127.61
126.99
126.97
126.94
126.91
126.31
124.50
123.27
123.25
123.22
123.20
84.64
73.44
55.64
53.13
51.40
48.35
40.59
39.61
33.73
32.25
25.87
23.37
22.97
f1 (ppm)

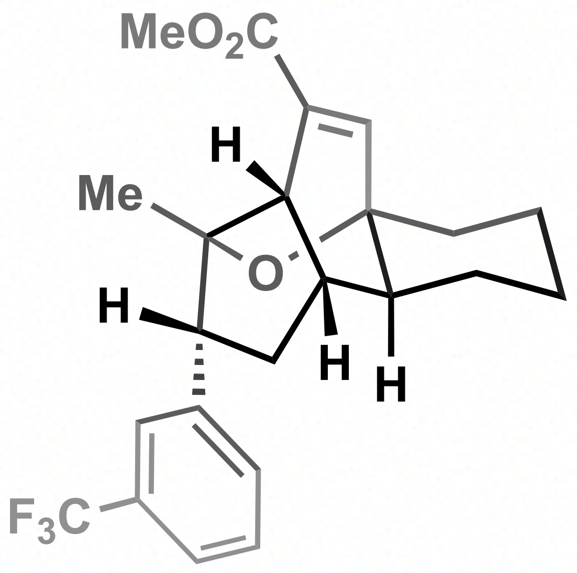

RS1188

19F (376 MHz, C_6D_6)

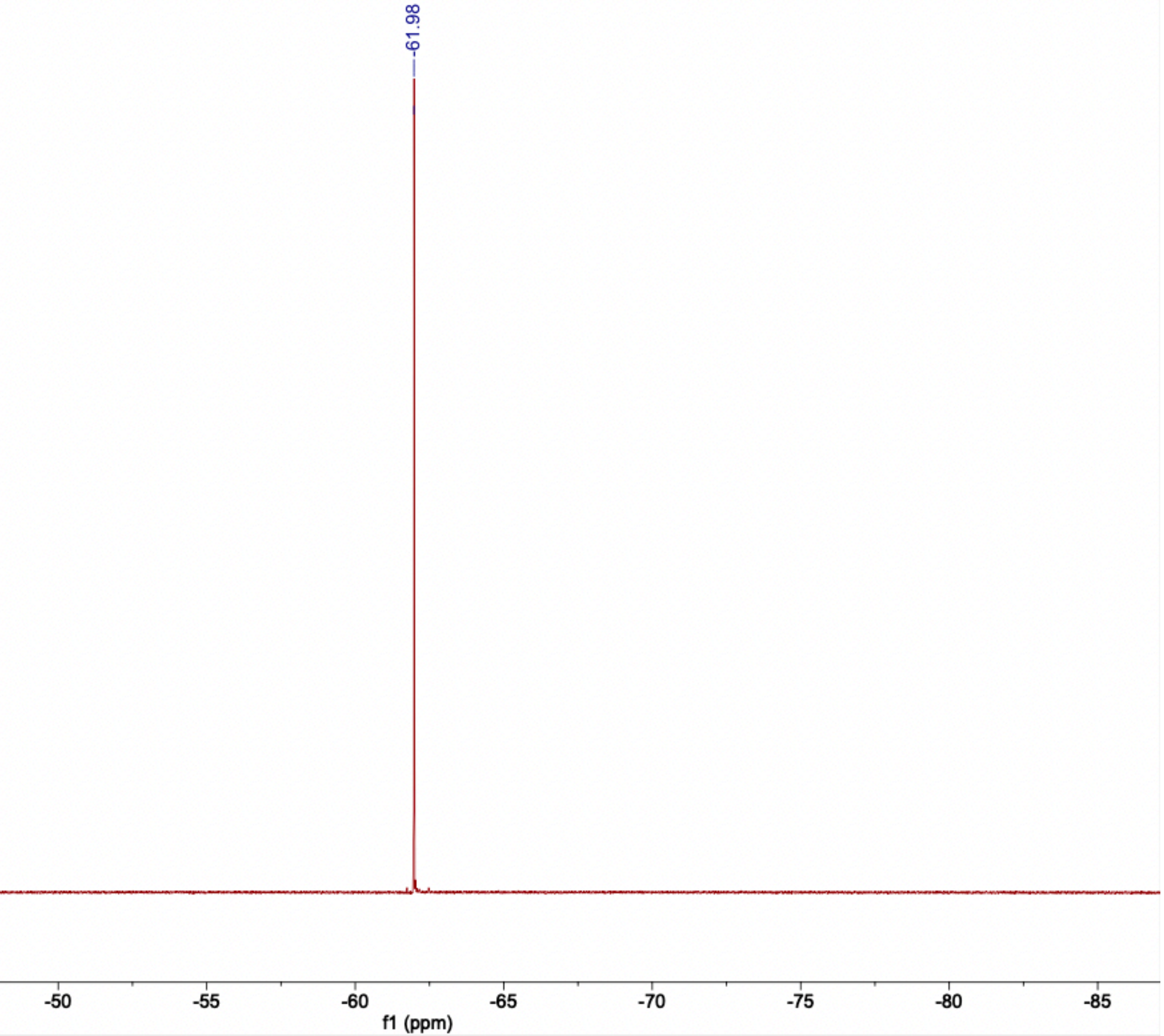

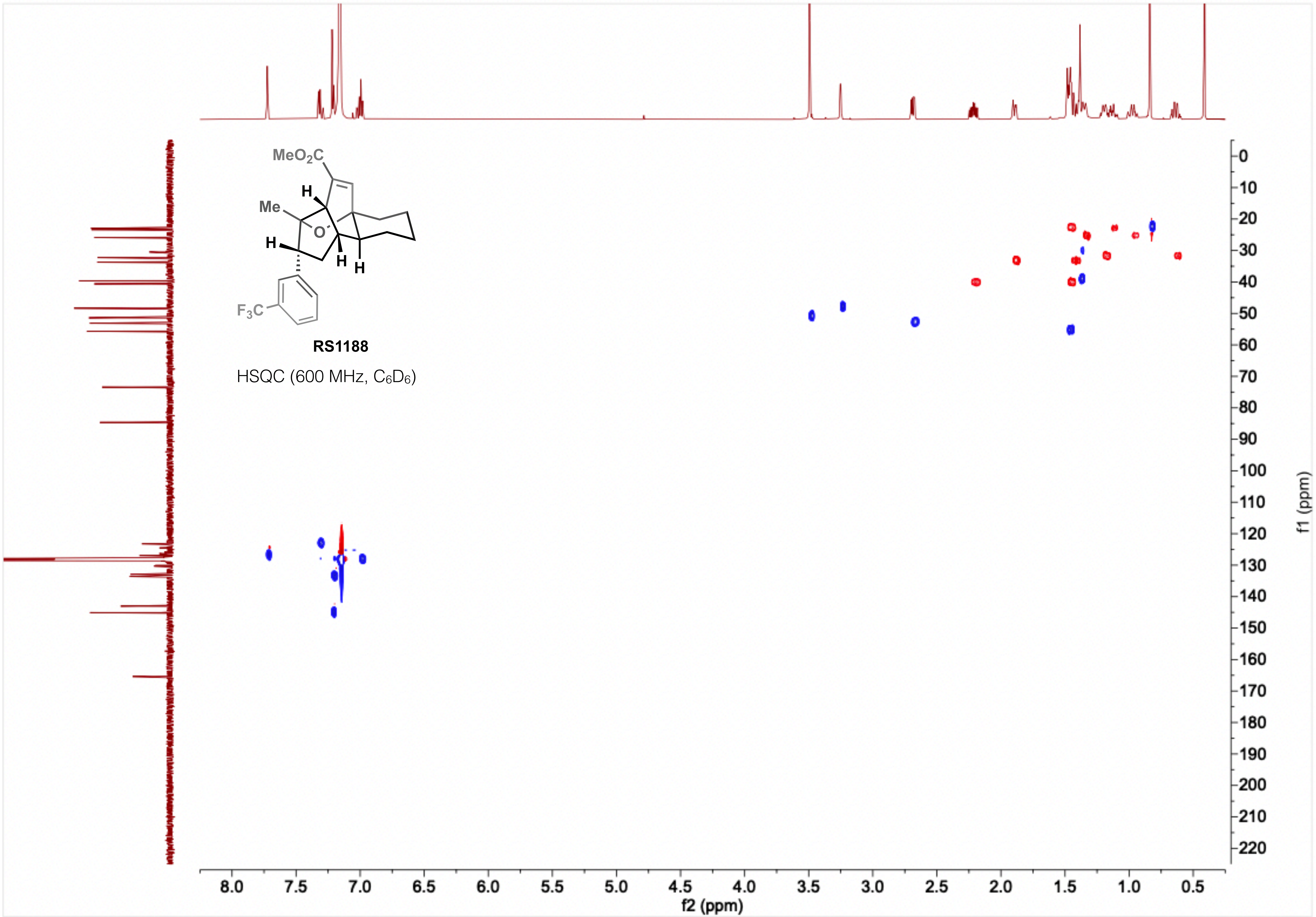

MeO2C
Me
O
H
F3C
RS1188
HSQC (600 MHz, C6D6)
f2 (ppm)
f1 (ppm)

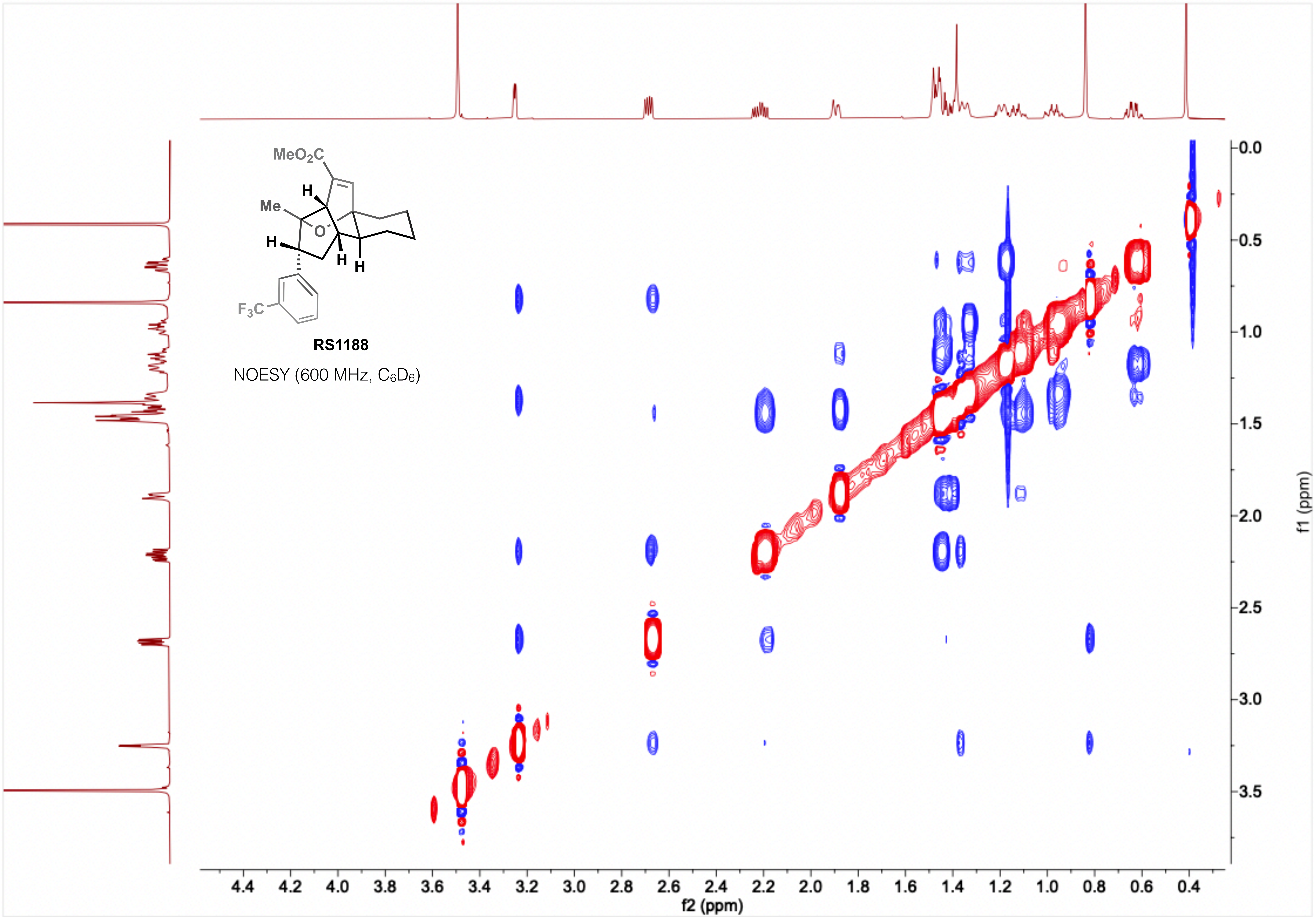
MeO2C
Me
H
O
H
H
H
F3C
RS1188
NOESY (600 MHz, C6D6)
f2 (ppm)
f1 (ppm)

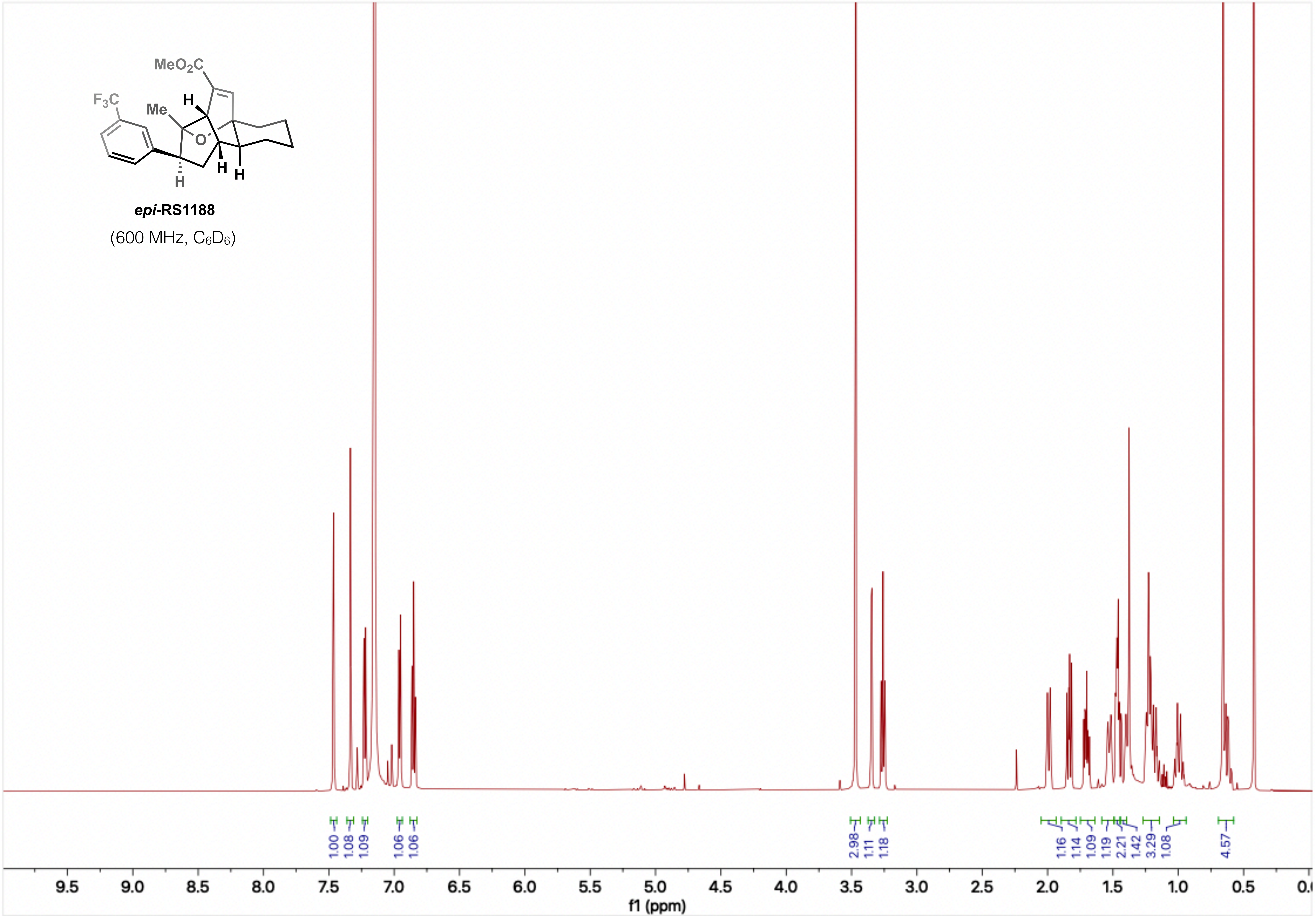

MeO2C
F3C
Me
H
O
H
H
H
epi-RS1188
(600 MHz, C6D6)
1.00
1.08
1.09
1.06
1.06
2.98
1.11
1.18
1.16
1.14
1.09
1.19
2.21
1.42
3.29
1.08
4.57
9.5
9.0
8.5
8.0
7.5
7.0
6.5
6.0
5.5
5.0
4.5
4.0
3.5
3.0
2.5
2.0
1.5
1.0
0.5
f1 (ppm)

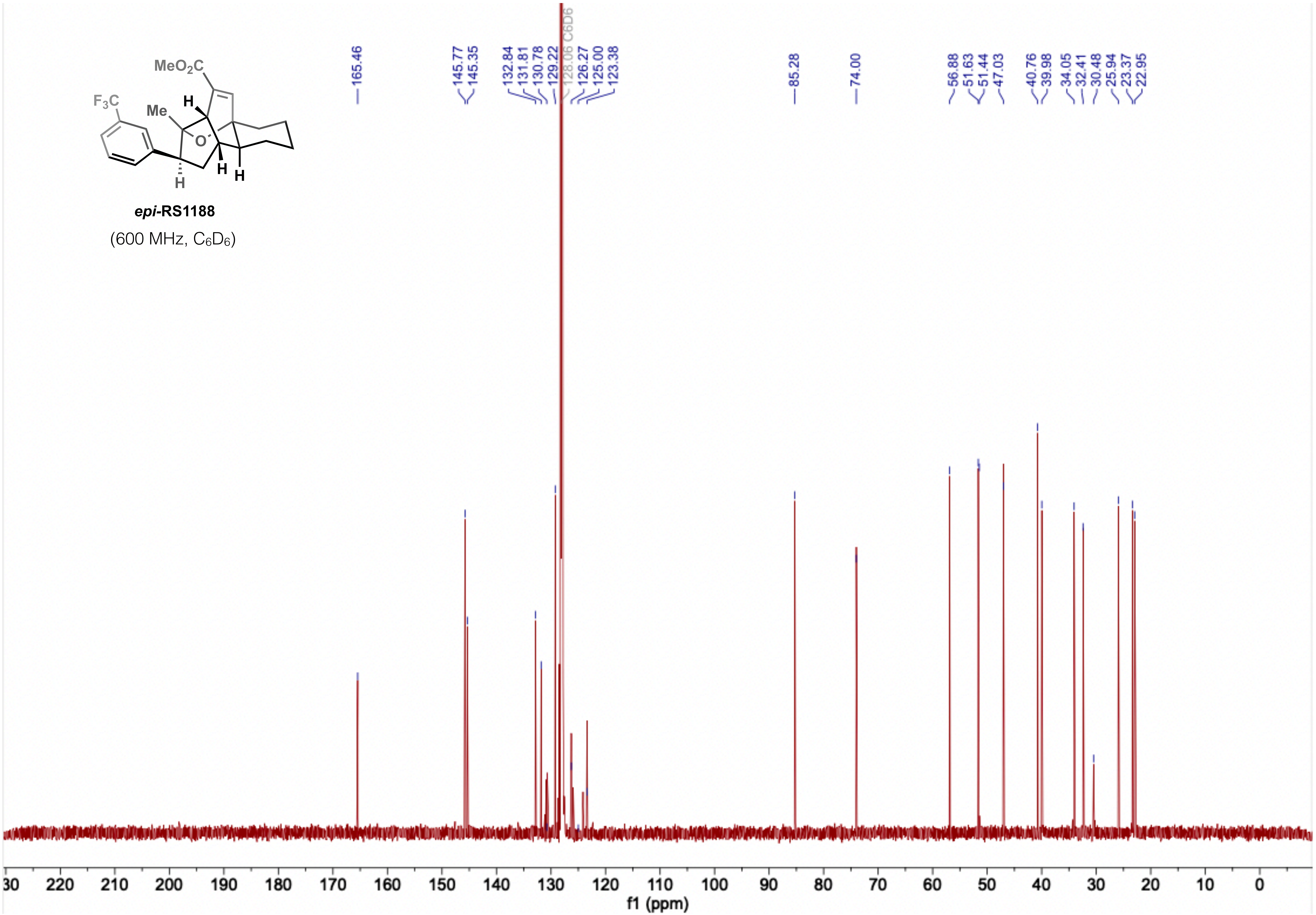

***epi*-RS1188**

(600 MHz, C_6D_6)

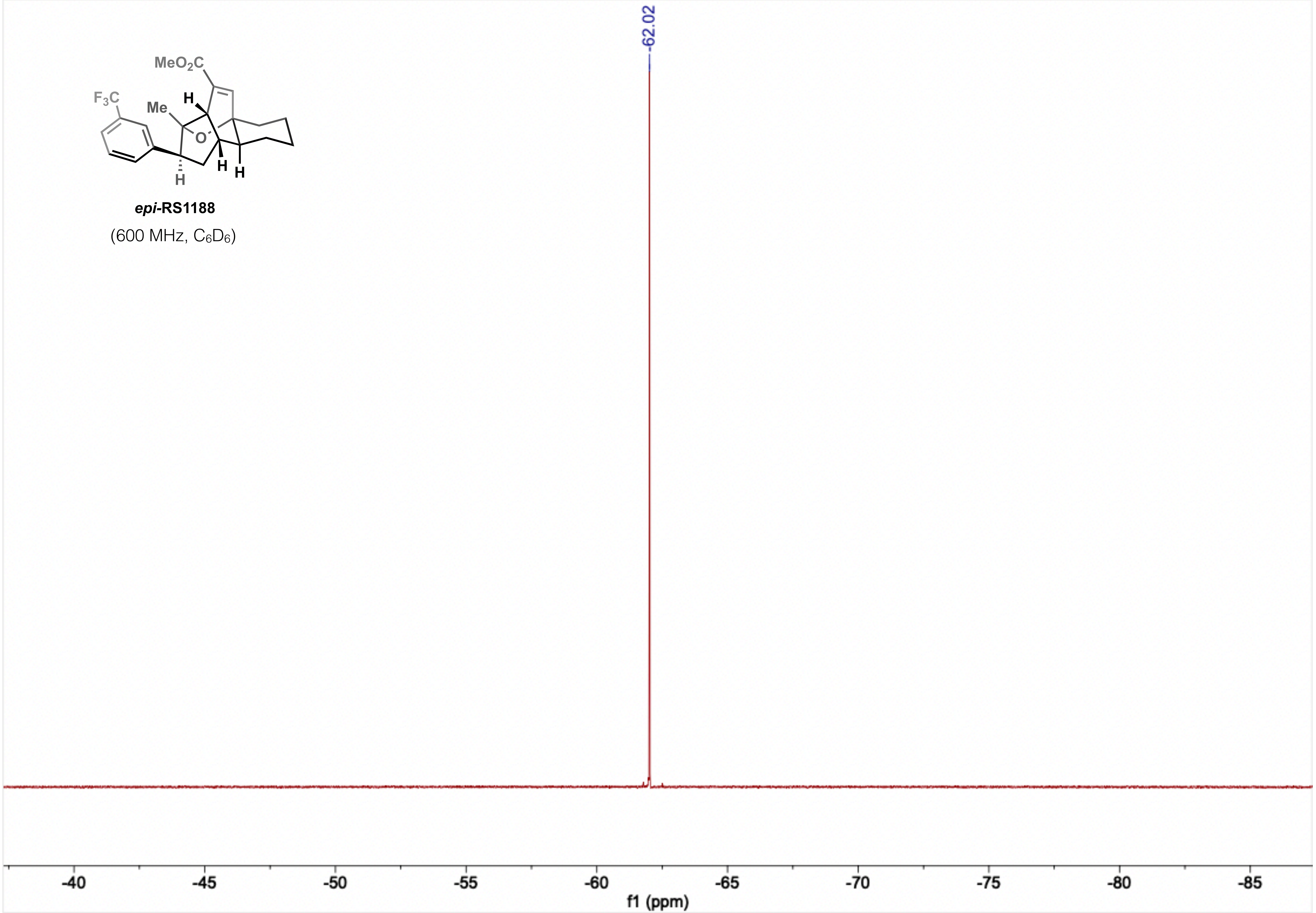

-62.02
MeO2C
F3C
Me
H
O
H
H
H
epi-RS1188
(600 MHz, C6D6)
-40
-45
-50
-55
-60
-65
-70
-75
-80
-85
f1 (ppm)

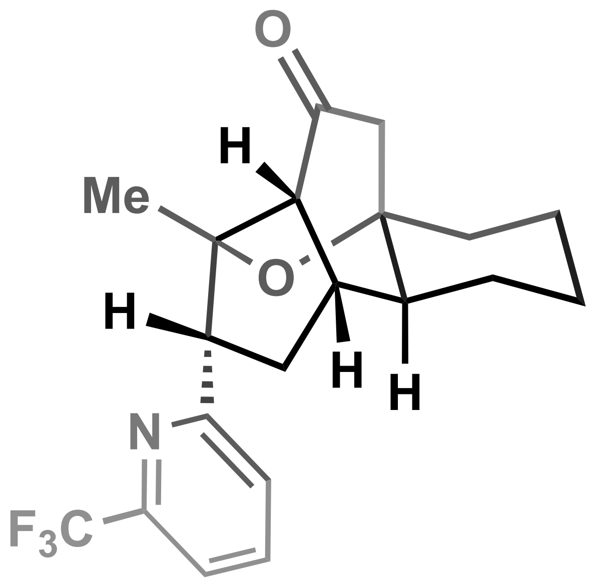

7a

(600 MHz, CDCl3)

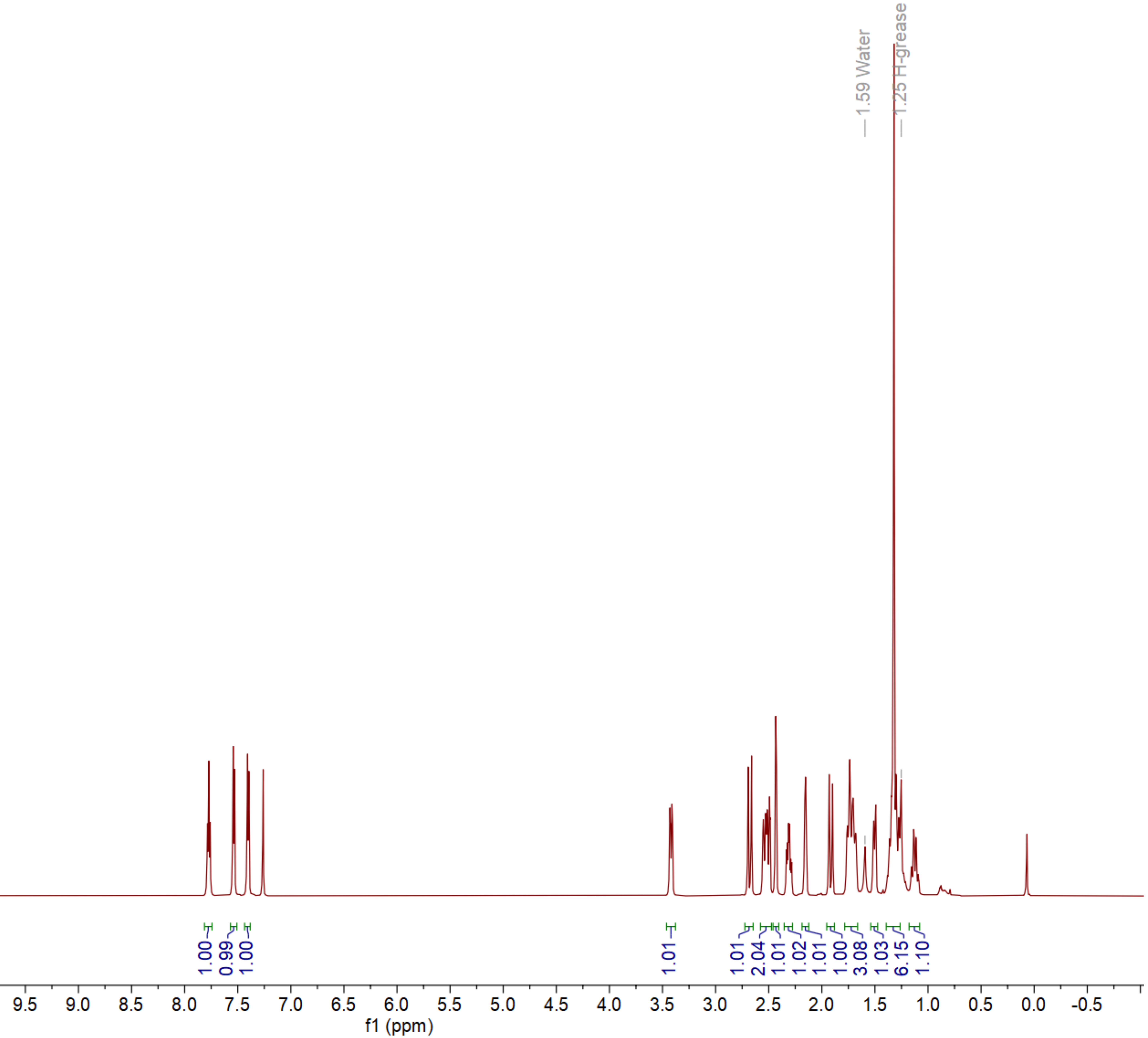

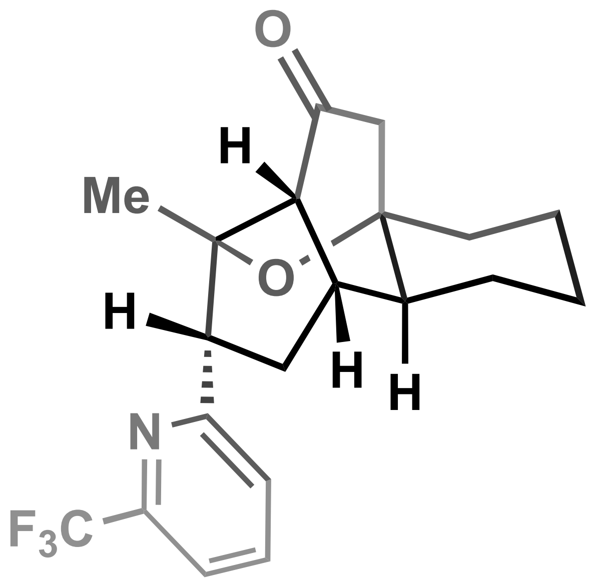

7a

(151 MHz, $CDCl_3$)

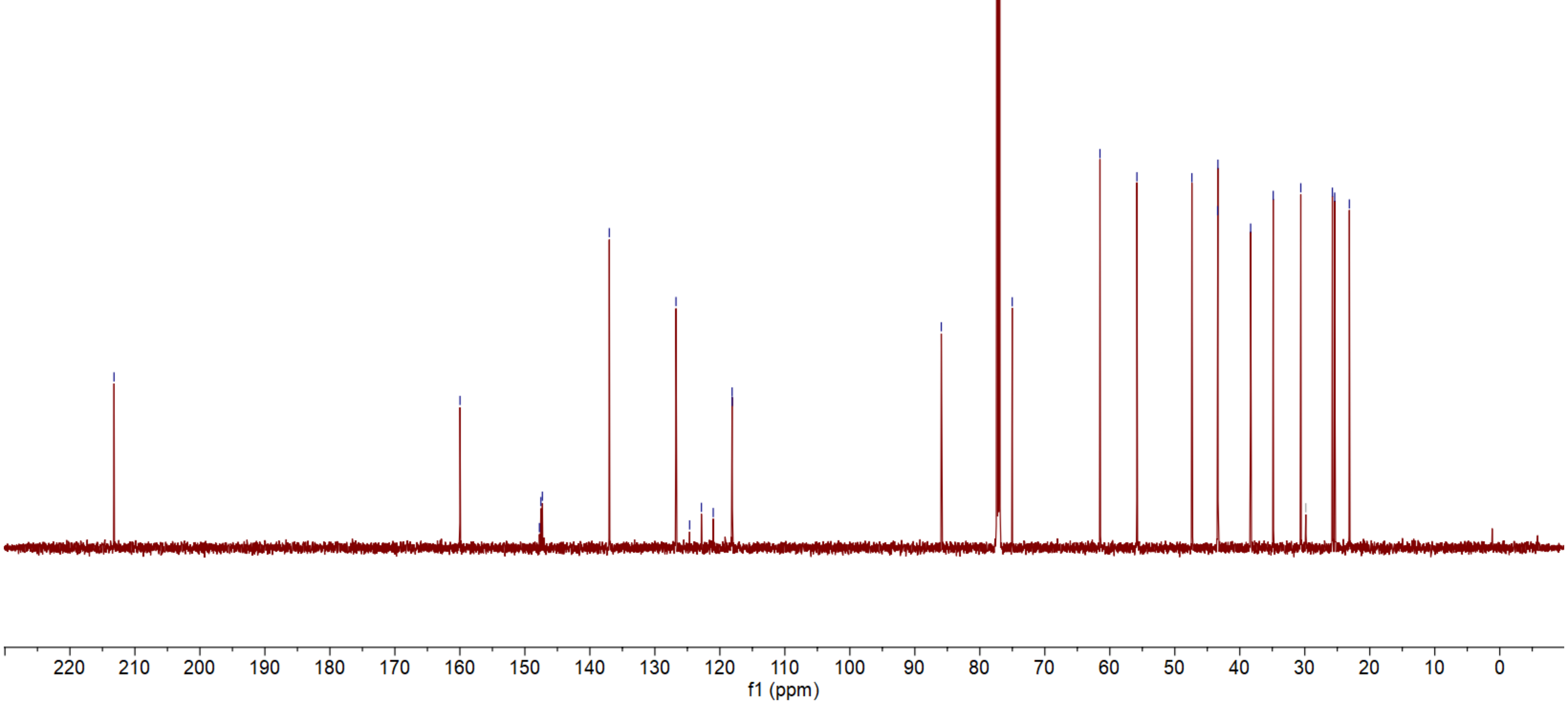

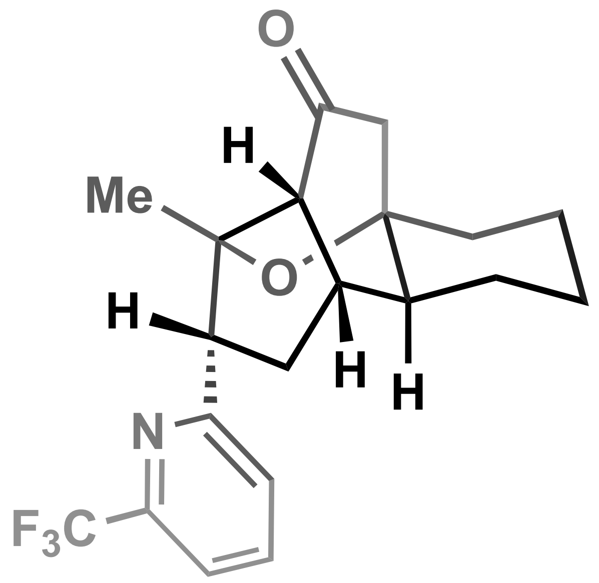

7a

COSY (600 MHz, $CDCl_3$)

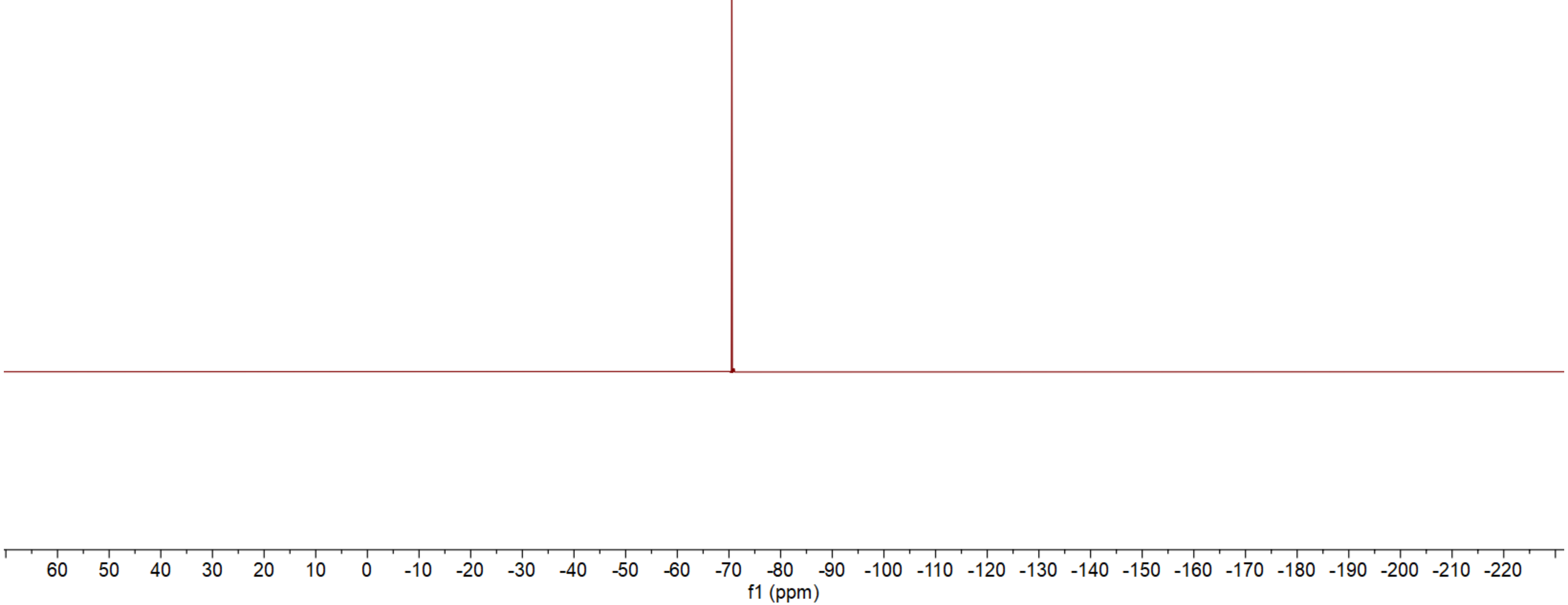

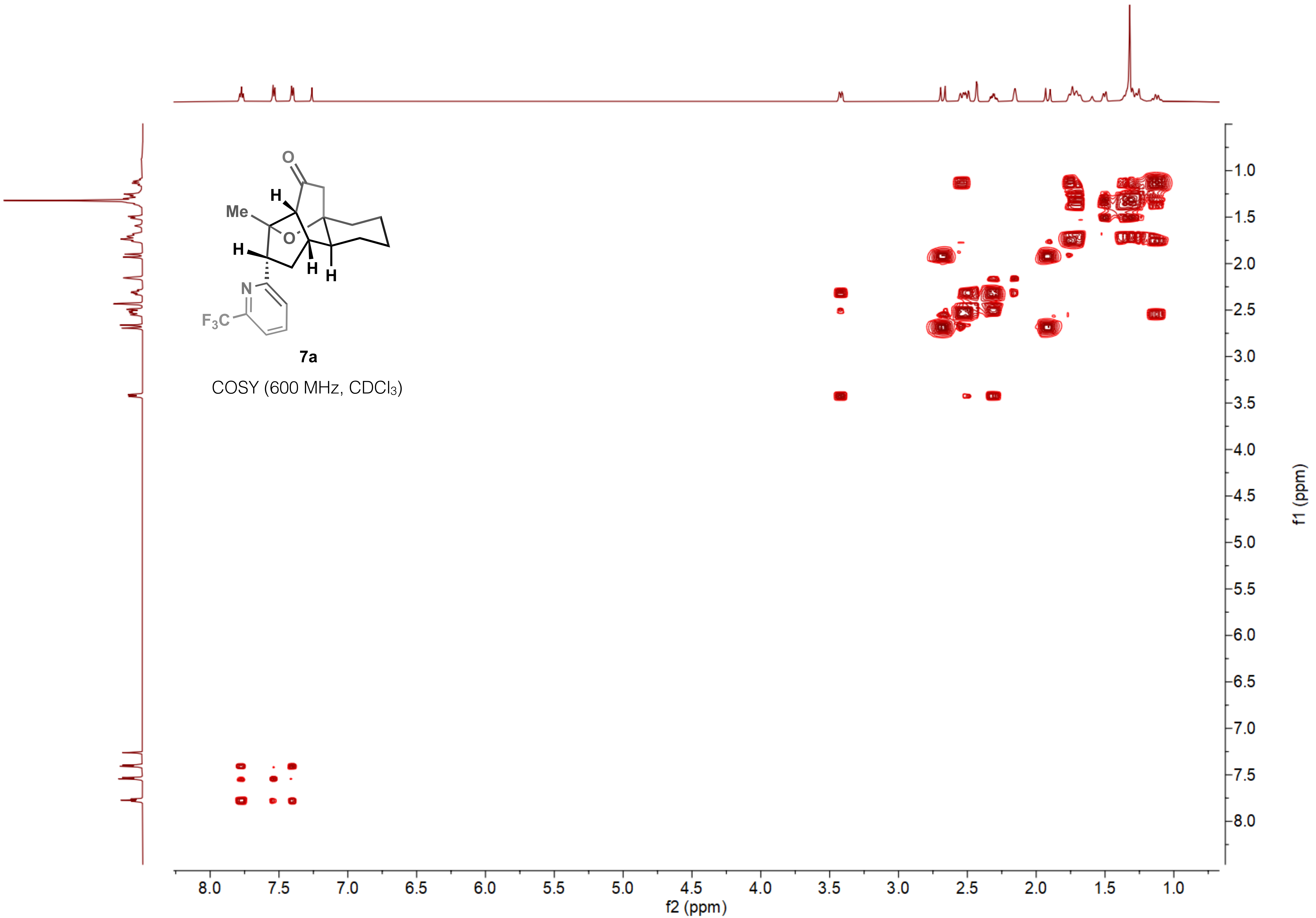
O
H
Me
O
H
H
H
N
F3C
7a
COSY (600 MHz, CDCl3)
f2 (ppm)
f1 (ppm)

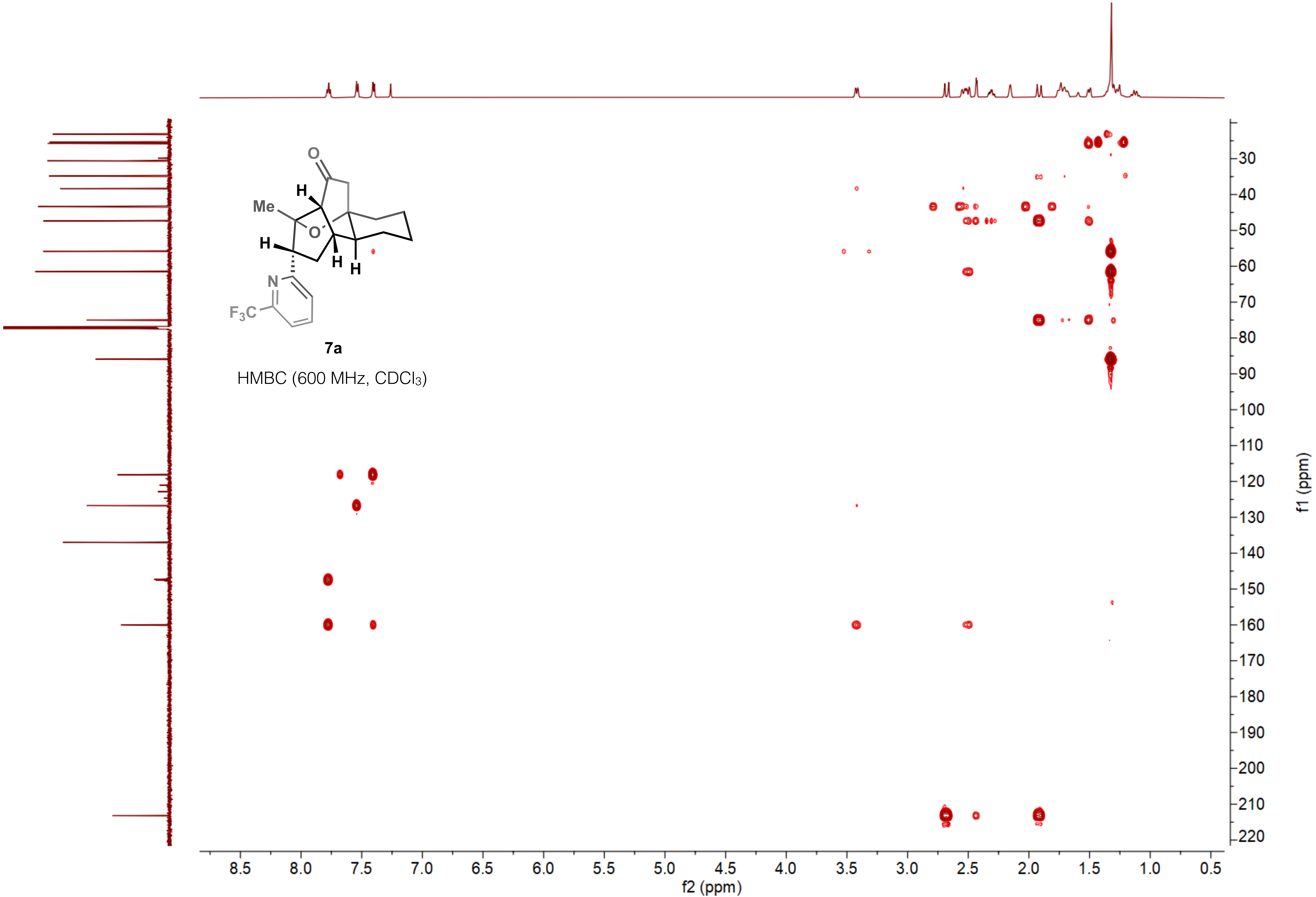

O
H
Me
O
H
H
H
N
F3C
7a
HMBC (600 MHz, CDCl3)
f2 (ppm)
f1 (ppm)

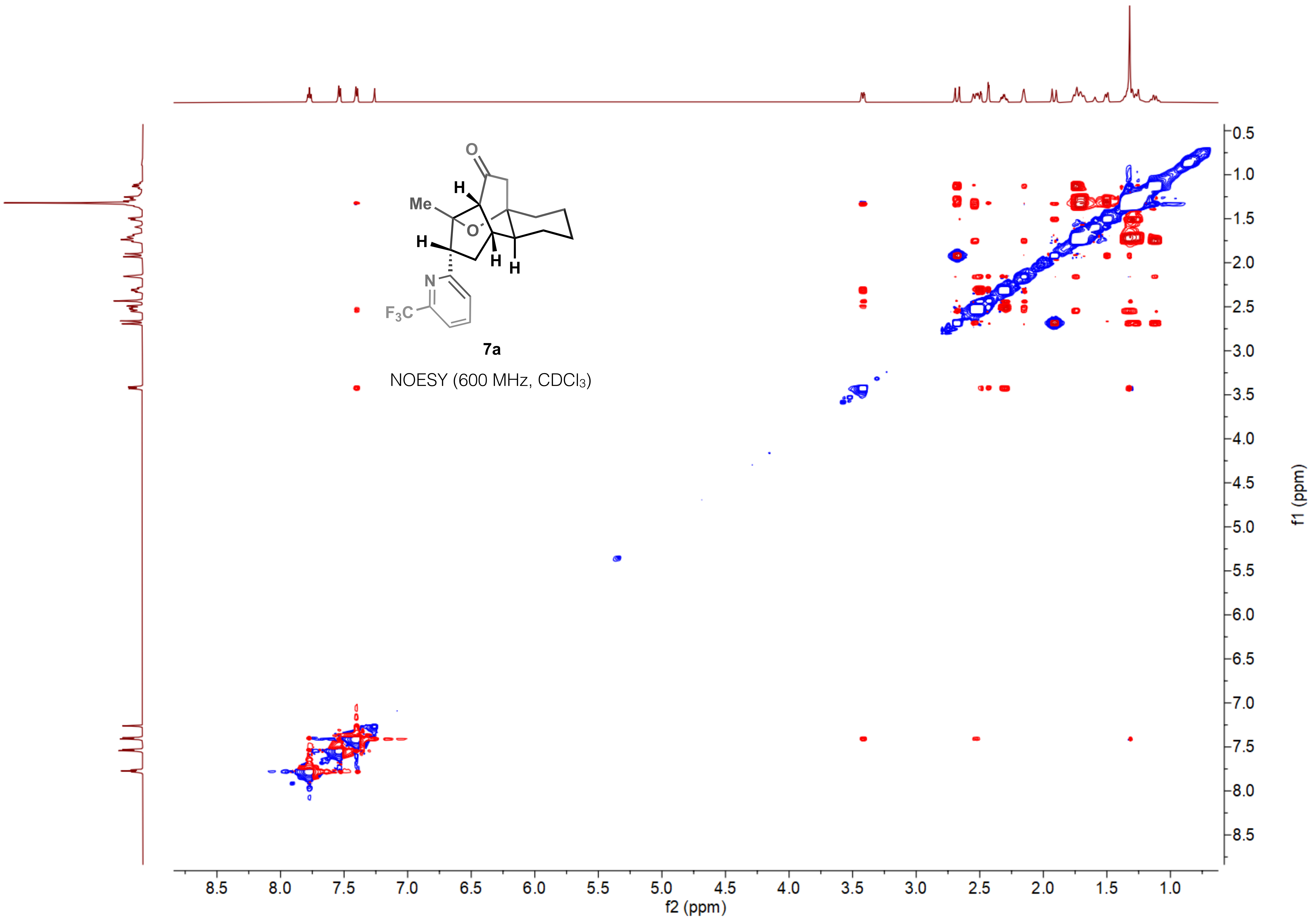

O
H
Me
O
H
H
H
N
F3C
7a
NOESY (600 MHz, CDCl3)
f2 (ppm)
f1 (ppm)

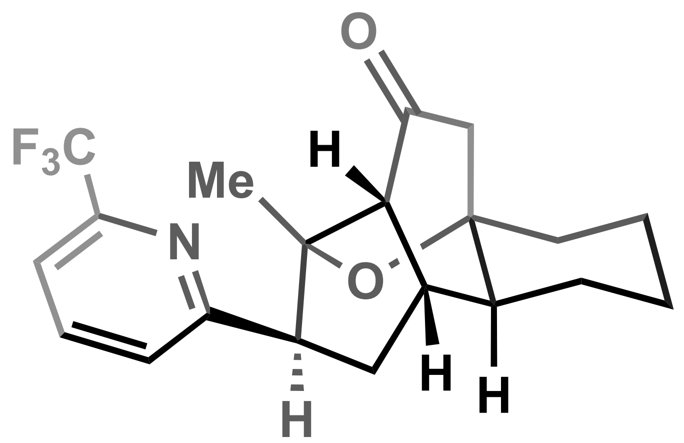

8a

(600 MHz, $CDCl_3$)

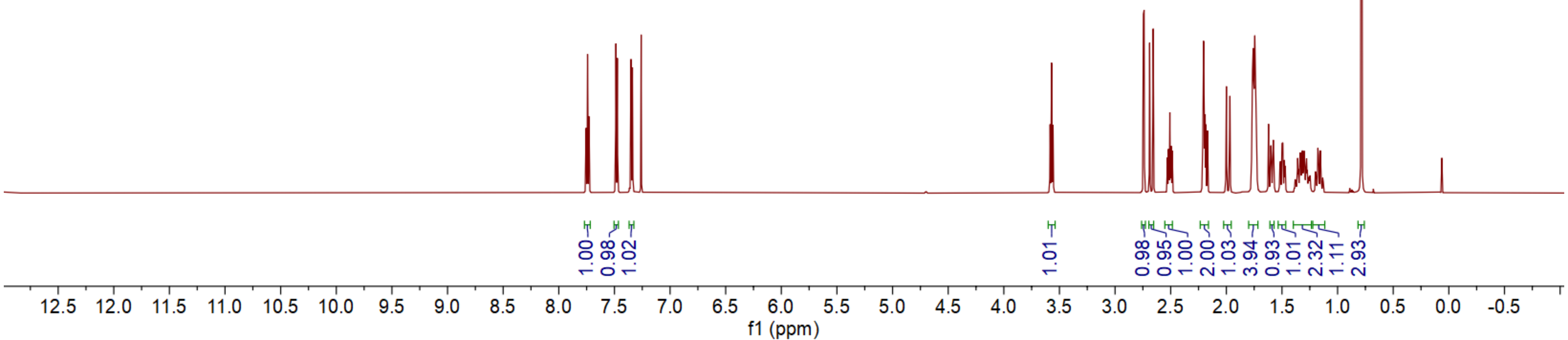

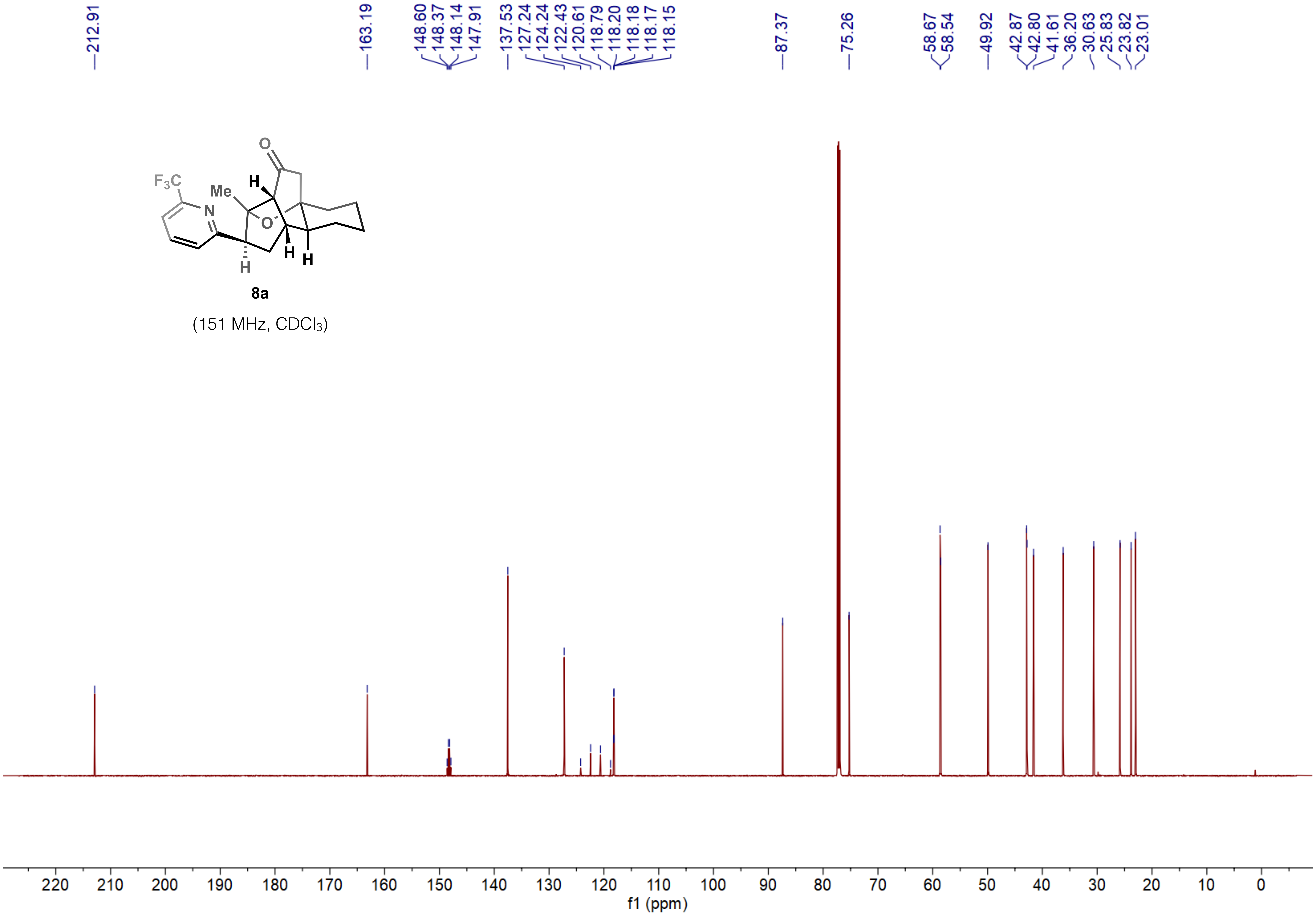

212.91
163.19
148.60
148.37
148.14
147.91
137.53
127.24
124.24
122.43
120.61
118.79
118.20
118.18
118.17
118.15
87.37
75.26
58.67
58.54
49.92
42.87
42.80
41.61
36.20
30.63
25.83
23.82
23.01
F_3C
Me
N
O
H
8a
(151 MHz, $CDCl_3$)
220 210 200 190 180 170 160 150 140 130 120 110 100 90 80 70 60 50 40 30 20 10 0
f1 (ppm)

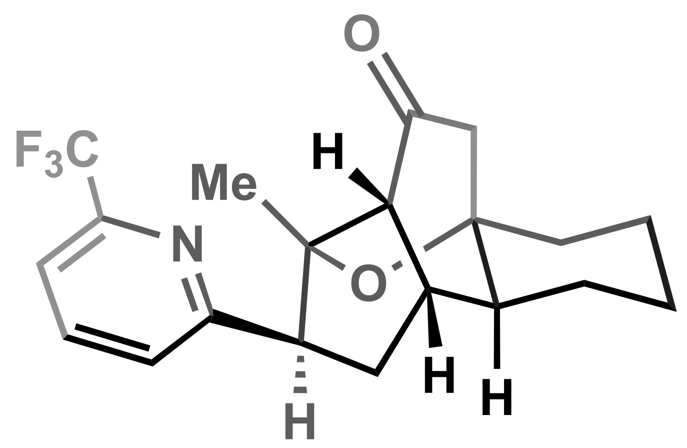

8a

(376 MHz, $CDCl_3$)

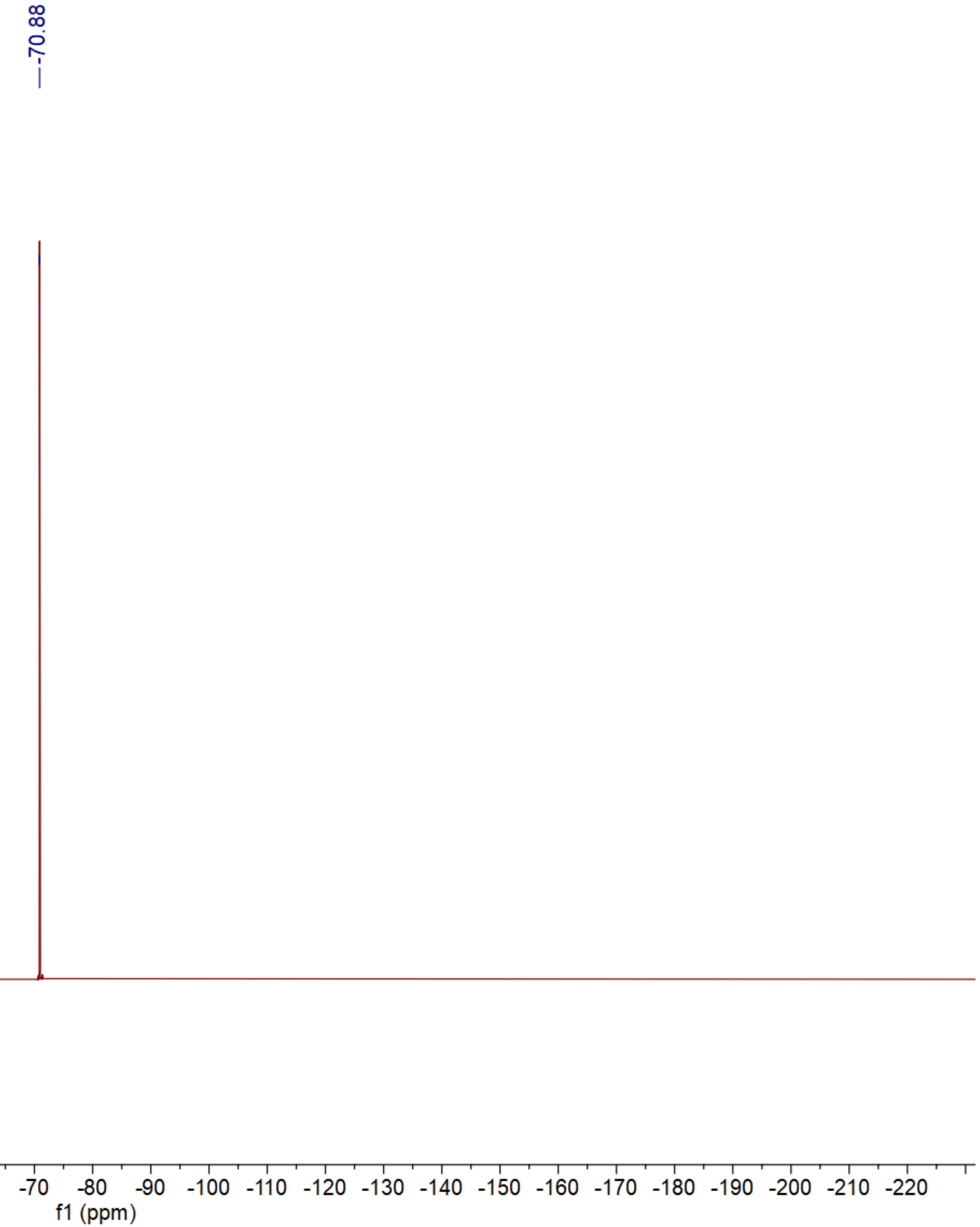

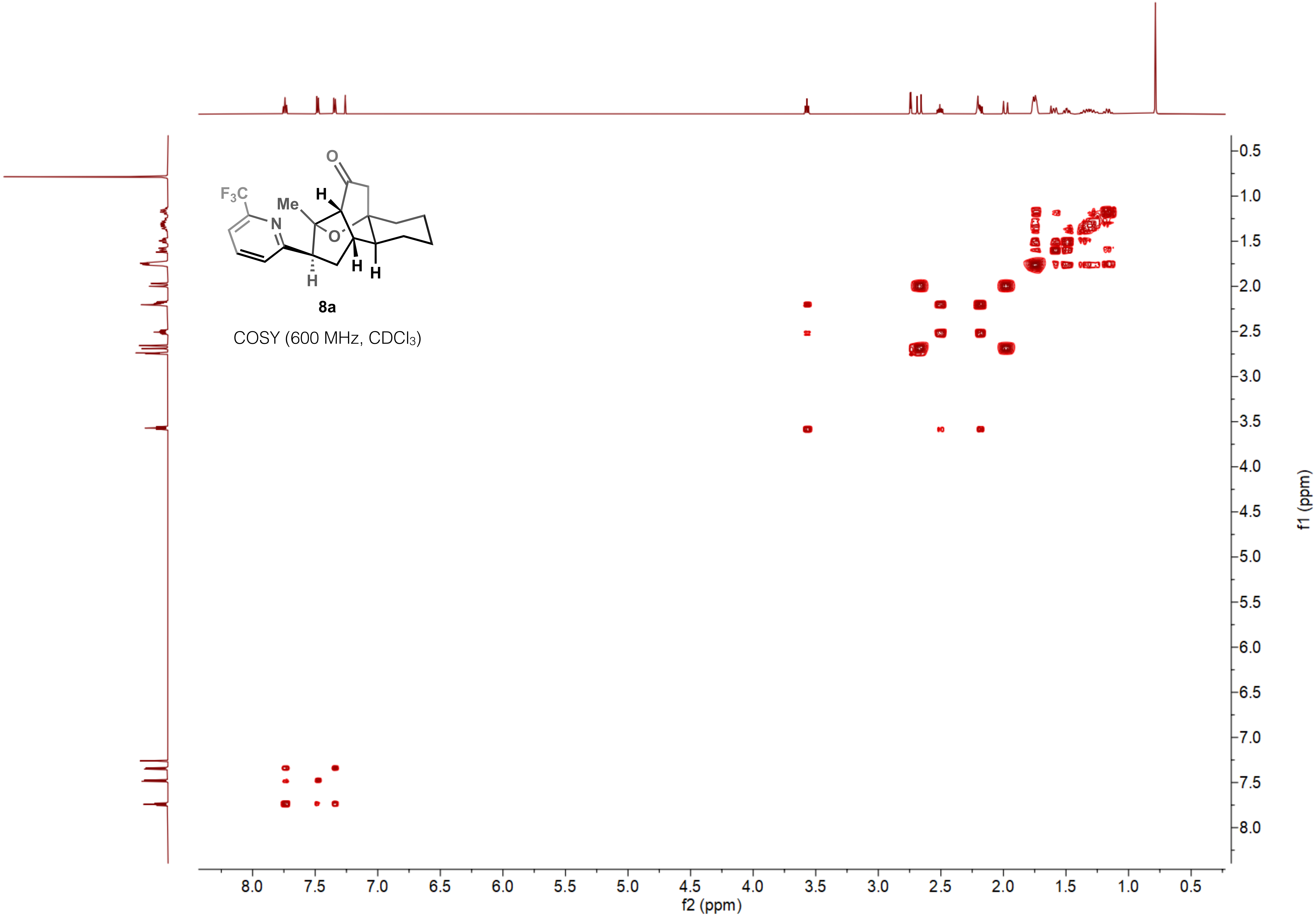
F3C
N
Me
O
H
H
H
H
8a
COSY (600 MHz, CDCl3)
f2 (ppm)
f1 (ppm)

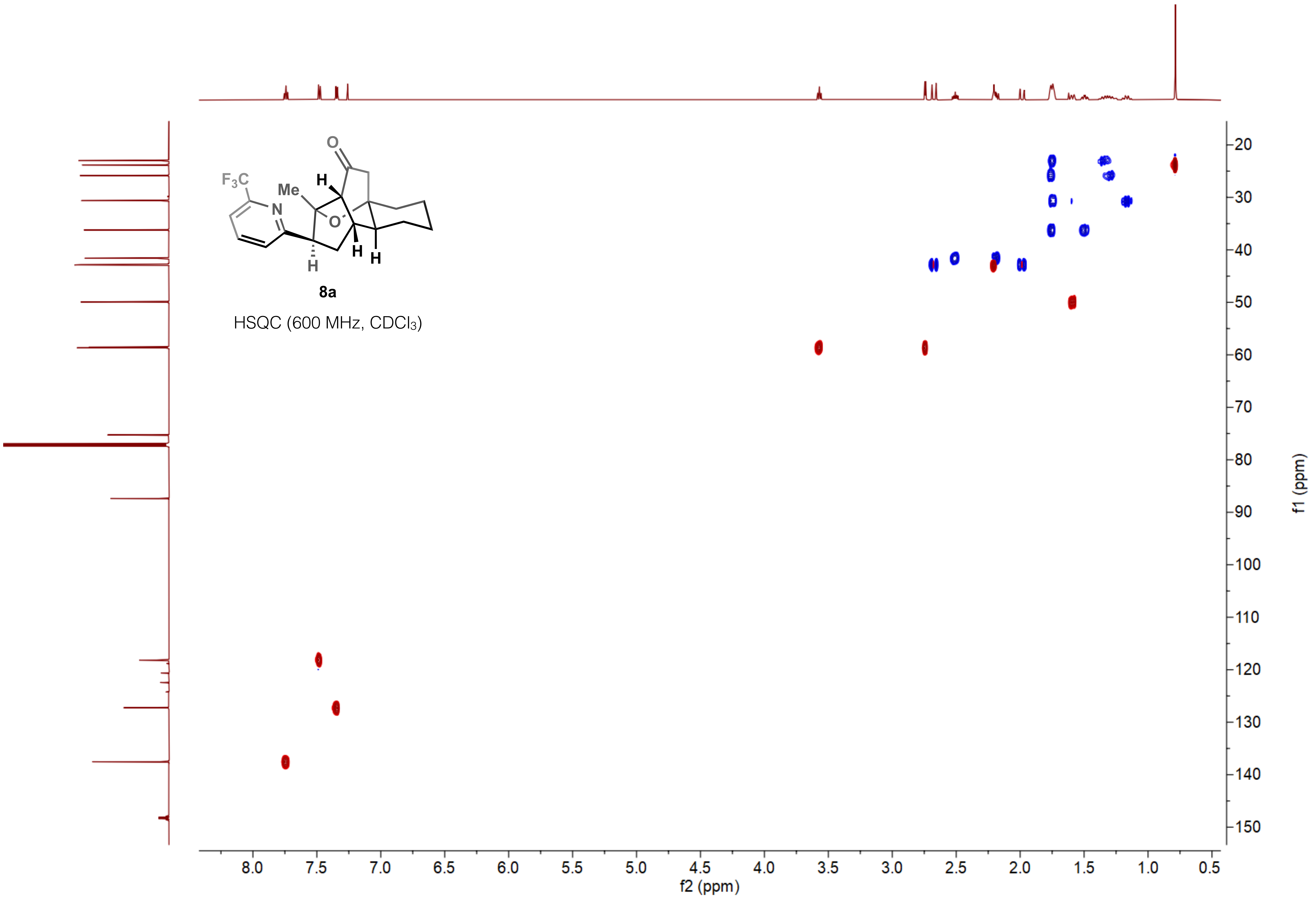

F3C
Me
N
O
H
H
H
H
8a
HSQC (600 MHz, CDCl3)
f2 (ppm)
f1 (ppm)
8.0 7.5 7.0 6.5 6.0 5.5 5.0 4.5 4.0 3.5 3.0 2.5 2.0 1.5 1.0 0.5
20 30 40 50 60 70 80 90 100 110 120 130 140 150

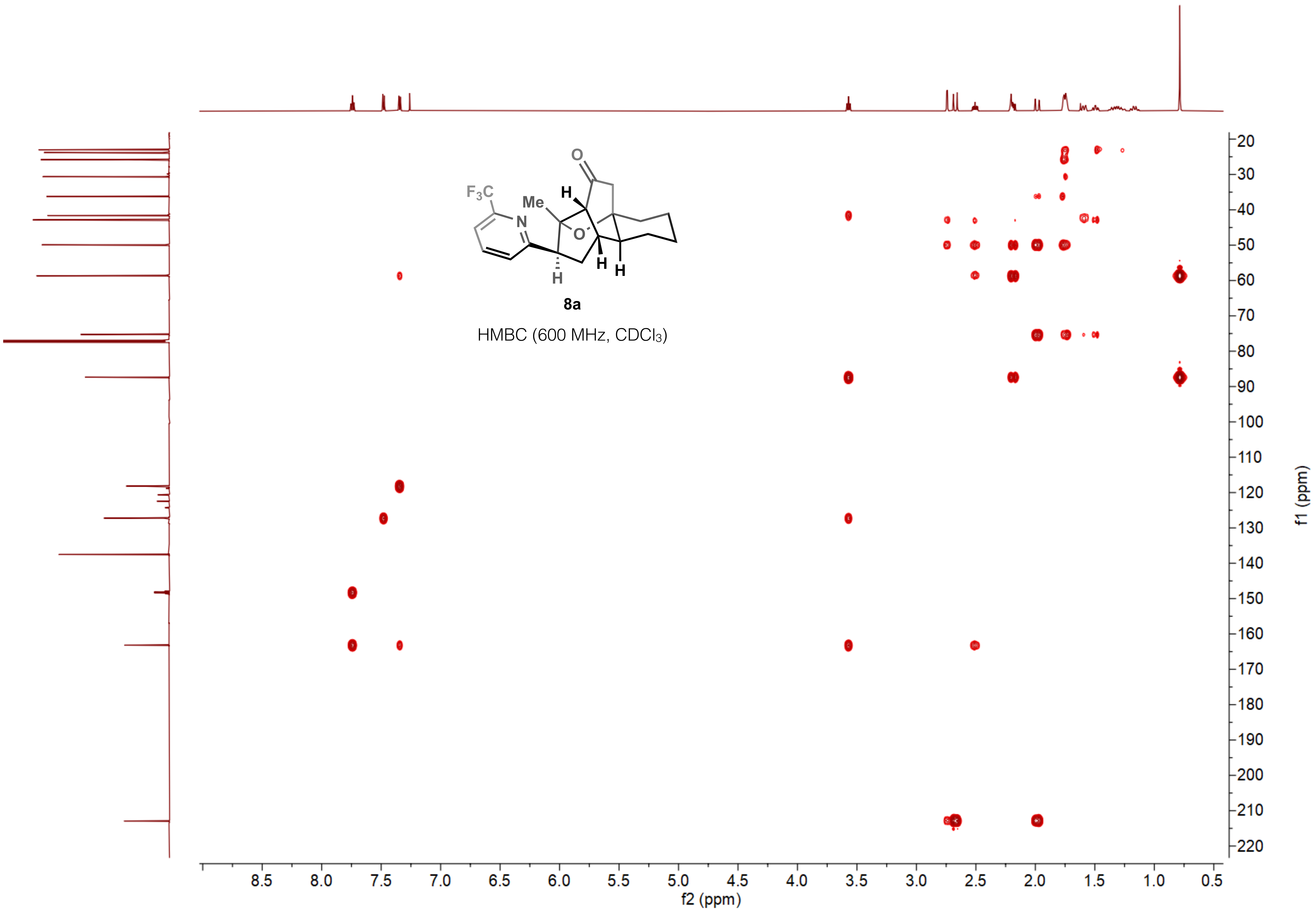
F3C
N
Me
O
H
H
H
H
8a
HMBC (600 MHz, CDCl3)
f2 (ppm)
f1 (ppm)

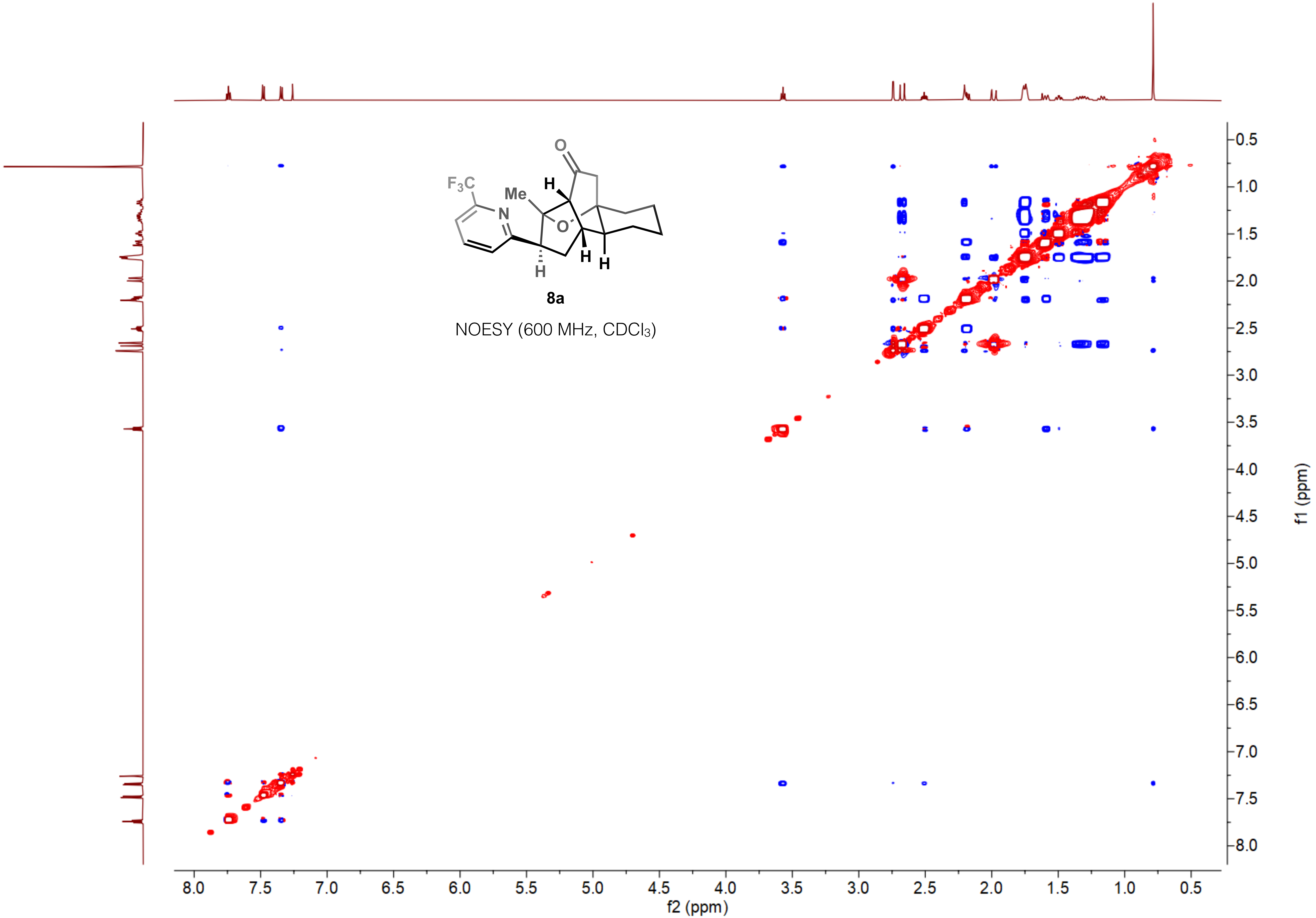
F3C
N
Me
O
H
H
H
H
O
8a
NOESY (600 MHz, CDCl3)
f2 (ppm)
f1 (ppm)

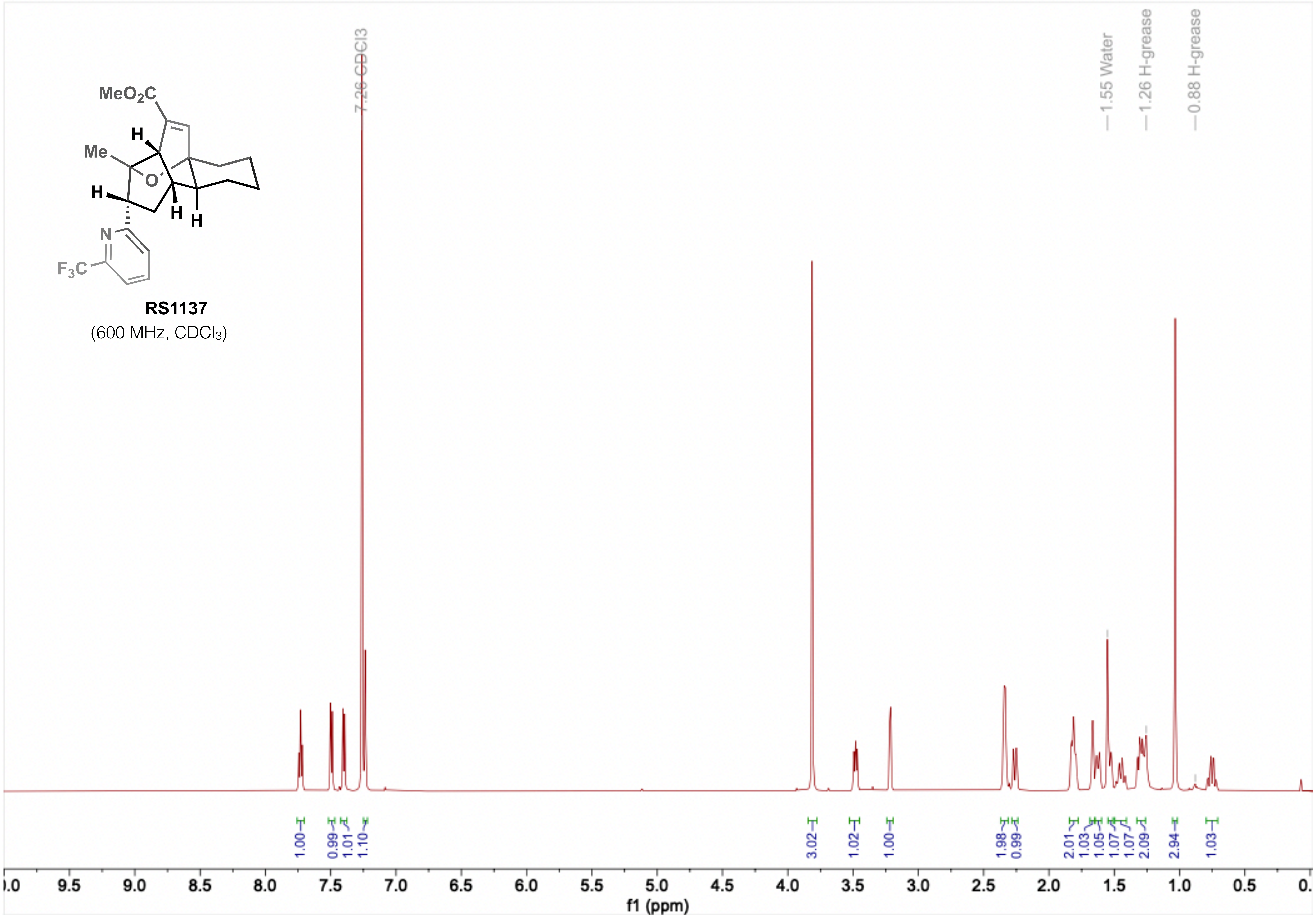

RS1137
(600 MHz, CDCl3)
MeO2C
Me
O
H
N
F3C
7.26 CDCl3
1.55 Water
1.26 H-grease
0.88 H-grease
1.00
0.99
1.01
1.10
3.02
1.02
1.00
1.98
0.99
2.01
1.03
1.05
1.07
1.07
2.09
2.94
1.03
9.5
9.0
8.5
8.0
7.5
7.0
6.5
6.0
5.5
5.0
4.5
4.0
3.5
3.0
2.5
2.0
1.5
1.0
0.5
f1 (ppm)

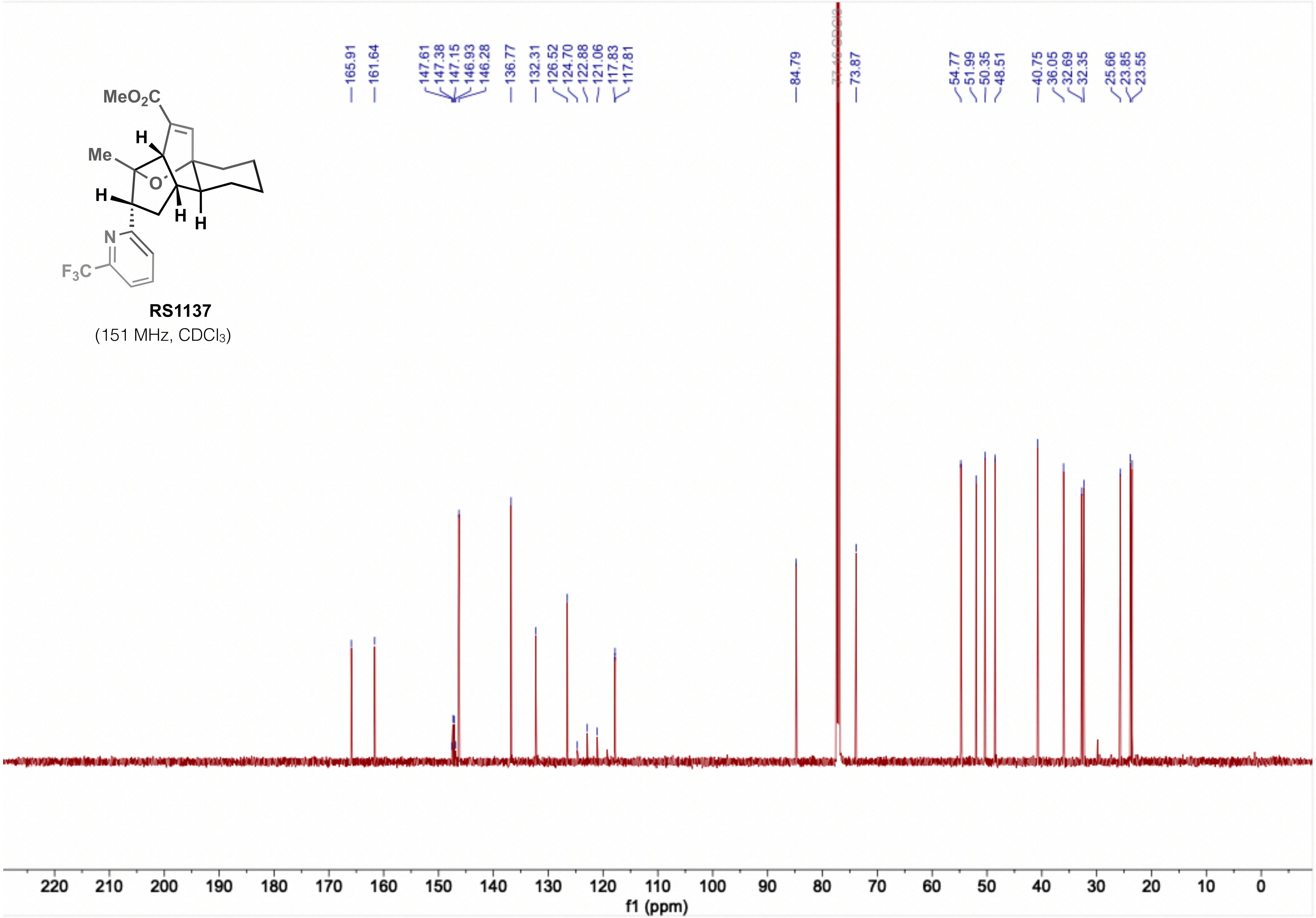
MeO2C
Me
H
O
H
H
H
N
F3C
RS1137
(151 MHz, CDCl3)
165.91
161.64
147.61
147.38
147.15
146.93
146.28
136.77
132.31
126.52
124.70
122.88
121.06
117.83
117.81
84.79
77.16 CDCl3
73.87
54.77
51.99
50.35
48.51
40.75
36.05
32.69
32.35
25.66
23.85
23.55
220
210
200
190
180
170
160
150
140
130
120
110
100
90
80
70
60
50
40
30
20
10
0
f1 (ppm)

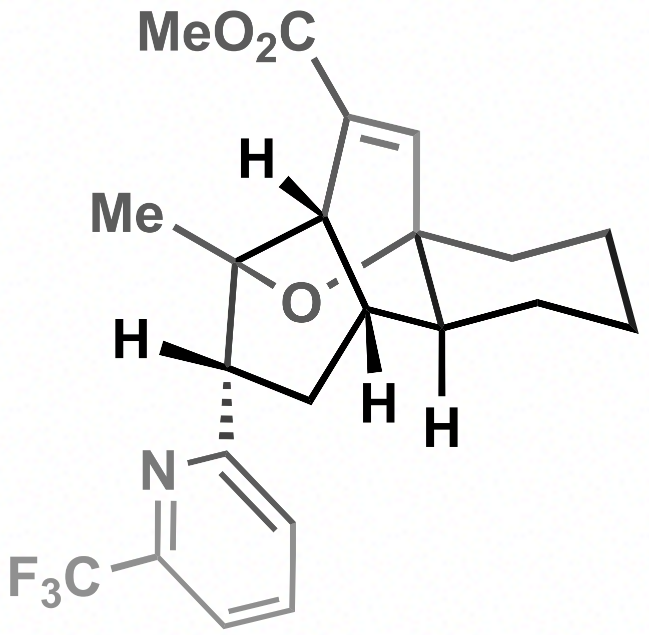

RS1137

19F (376 MHz, $CDCl_3$)

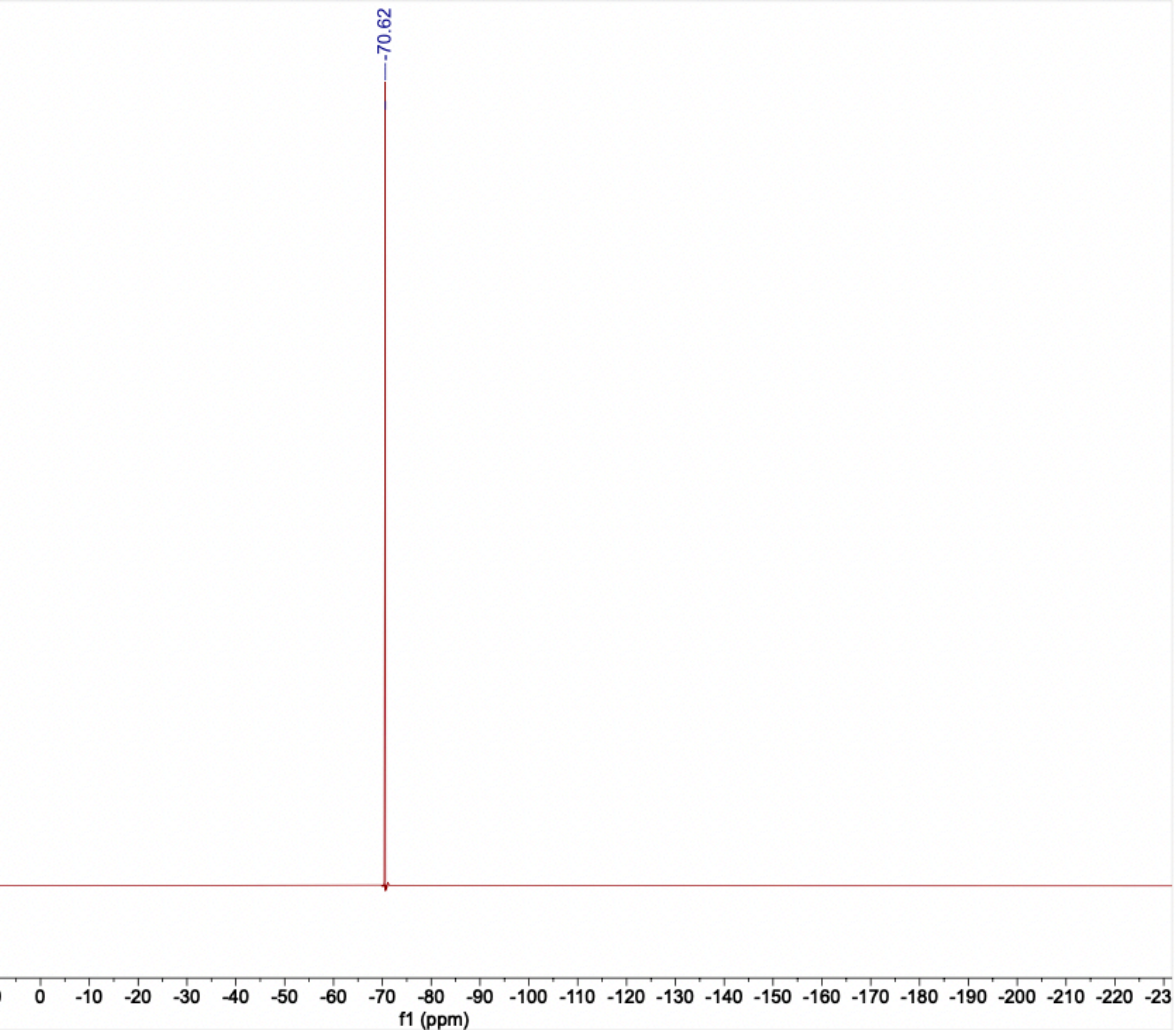

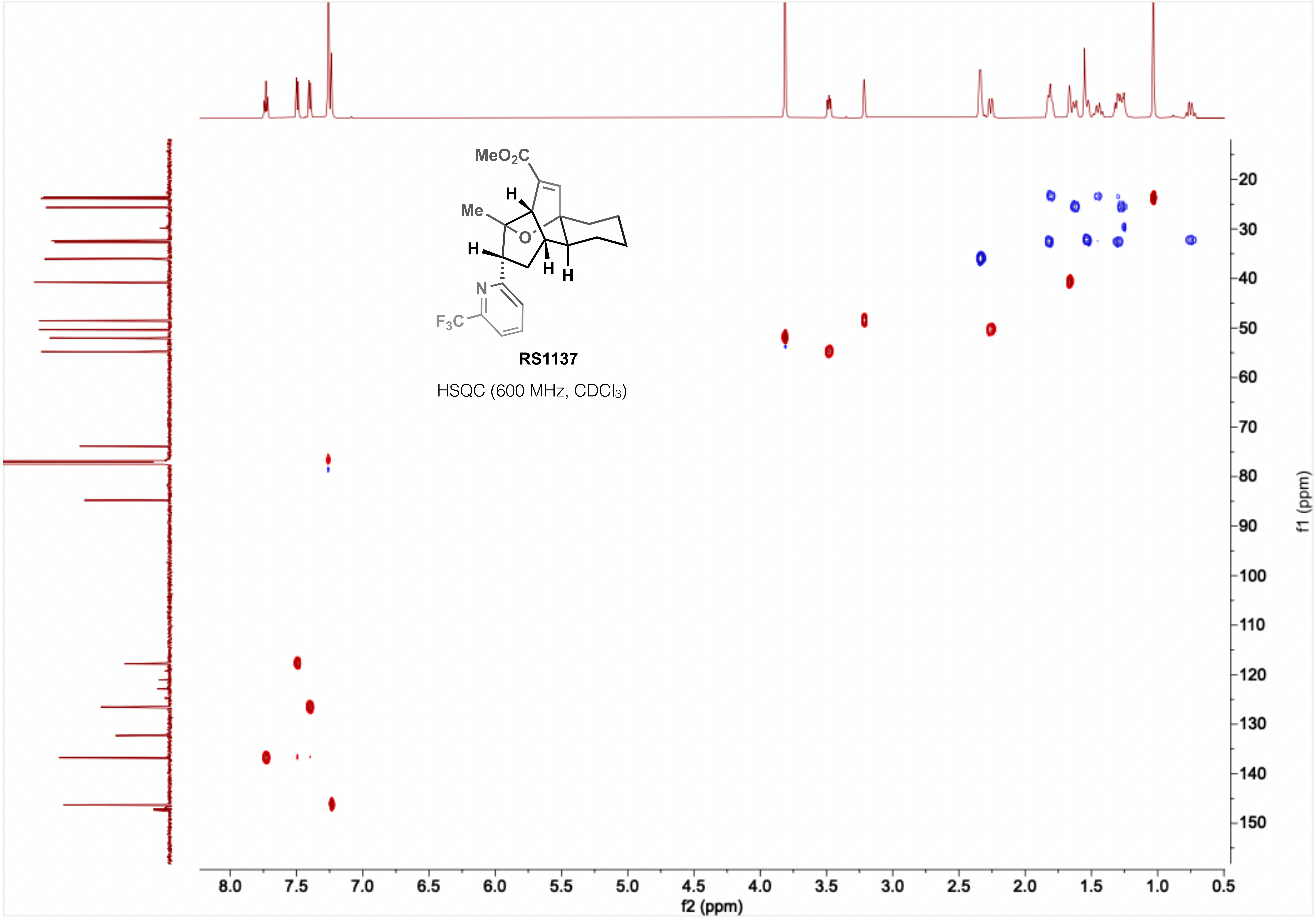
MeO2C
Me
O
H
H
H
H
N
F3C
RS1137
HSQC (600 MHz, CDCl3)
f2 (ppm)
f1 (ppm)

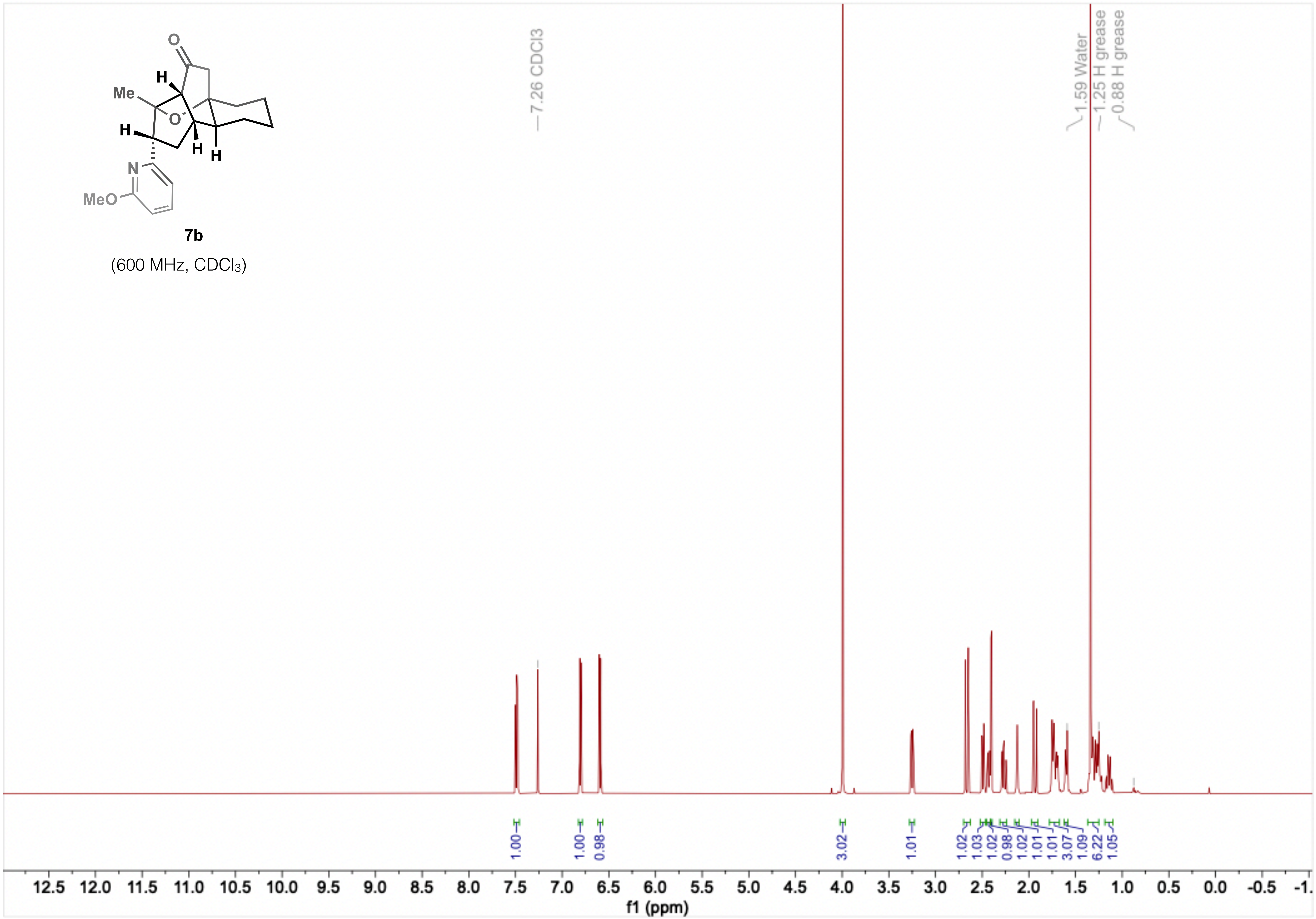

O
H
Me
O
H
H
H
N
MeO
7b
(600 MHz, CDCl$_3$)
7.26 CDCl3
1.59 Water
1.25 H grease
0.88 H grease
1.00
1.00
0.98
3.02
1.01
1.02
1.03
1.02
0.98
1.02
1.01
1.01
3.07
1.09
6.22
1.05
12.5 12.0 11.5 11.0 10.5 10.0 9.5 9.0 8.5 8.0 7.5 7.0 6.5 6.0 5.5 5.0 4.5 4.0 3.5 3.0 2.5 2.0 1.5 1.0 0.5 0.0 -0.5
f1 (ppm)

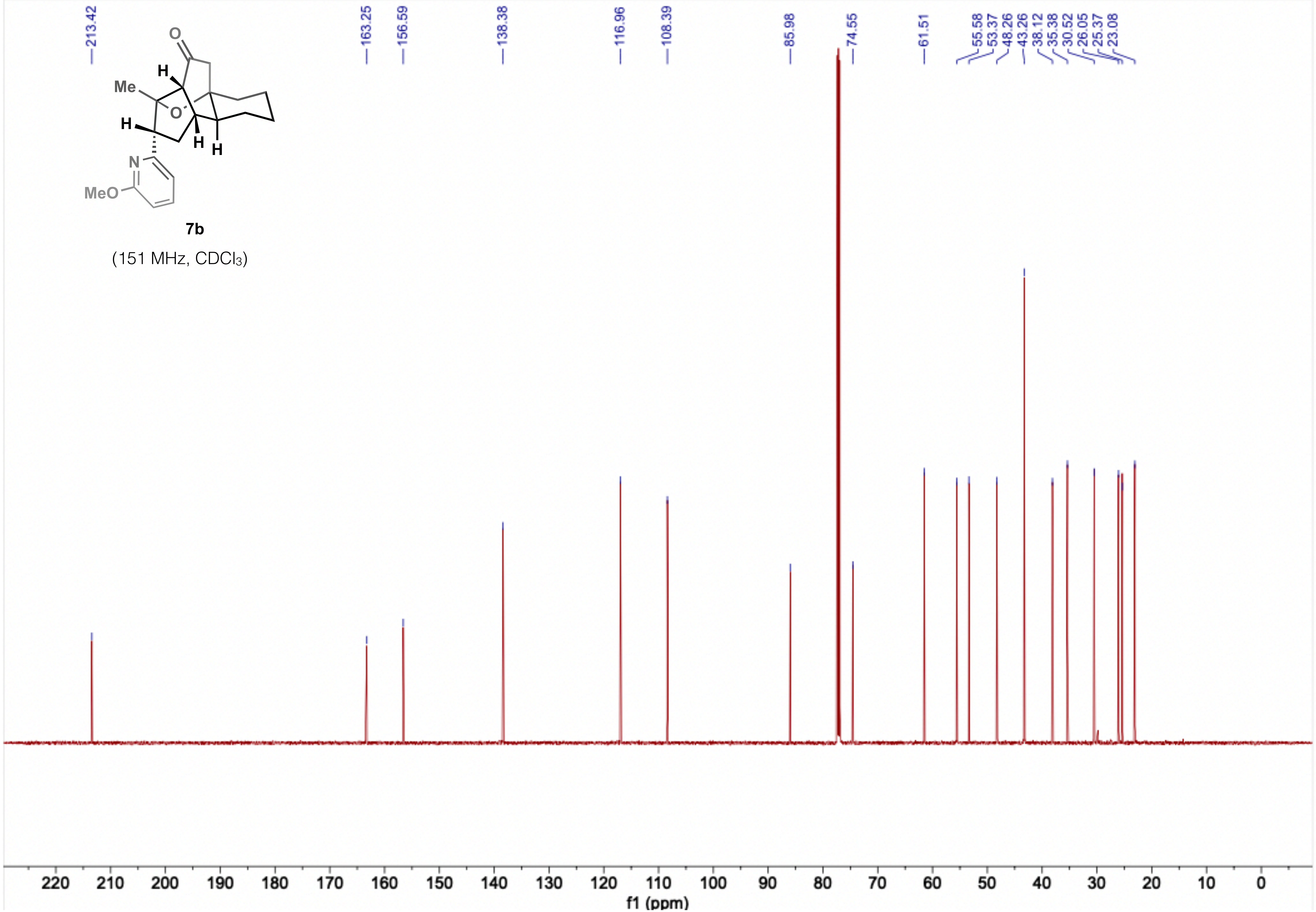

213.42
163.25
156.59
138.38
116.96
108.39
85.98
74.55
61.51
55.58
53.37
48.26
43.26
38.12
35.38
30.52
26.05
25.37
23.08
Me
O
H
N
MeO
7b
(151 MHz, CDCl3)
220 210 200 190 180 170 160 150 140 130 120 110 100 90 80 70 60 50 40 30 20 10 0
f1 (ppm)

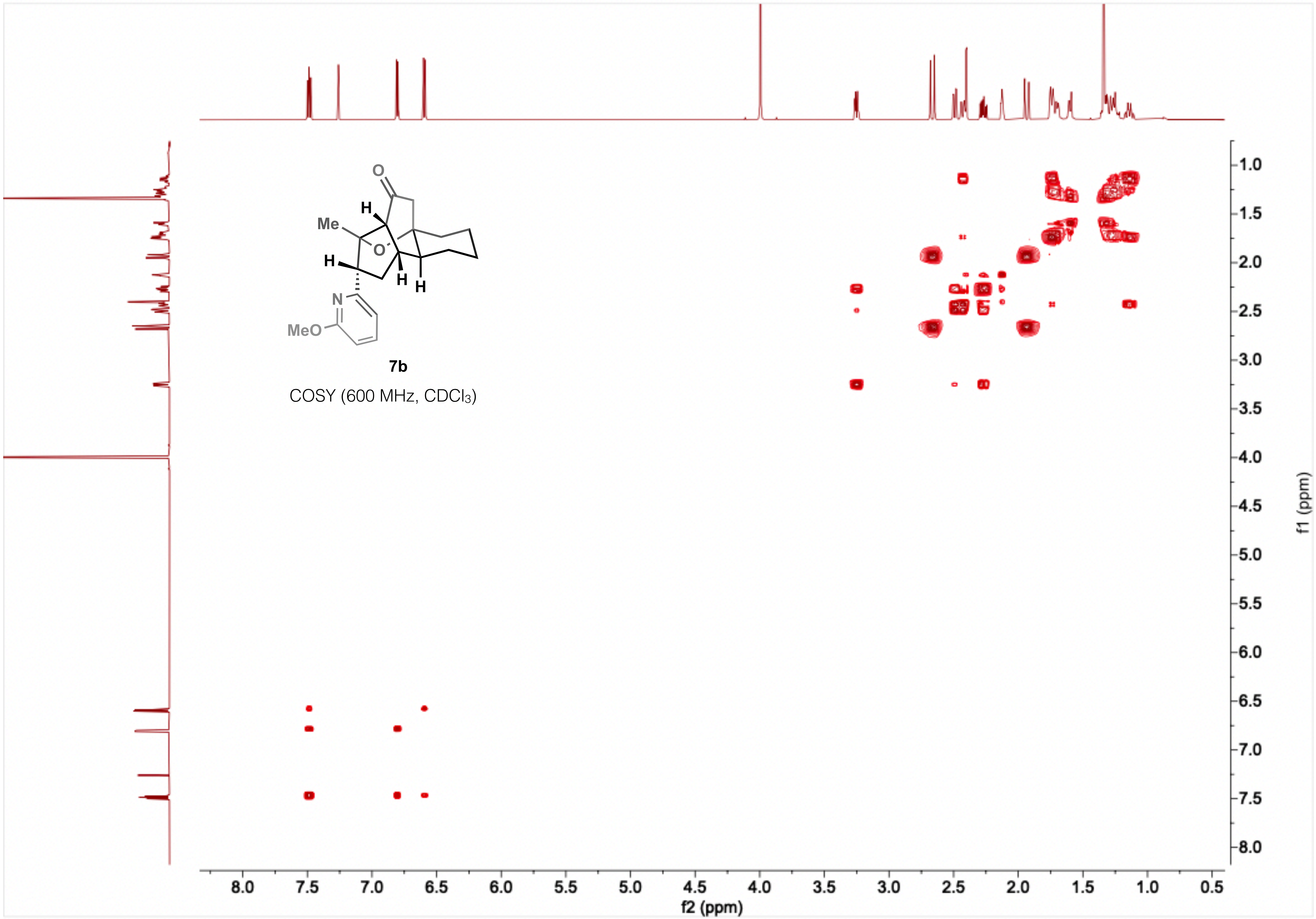

7b

COSY (600 MHz, $CDCl_3$)

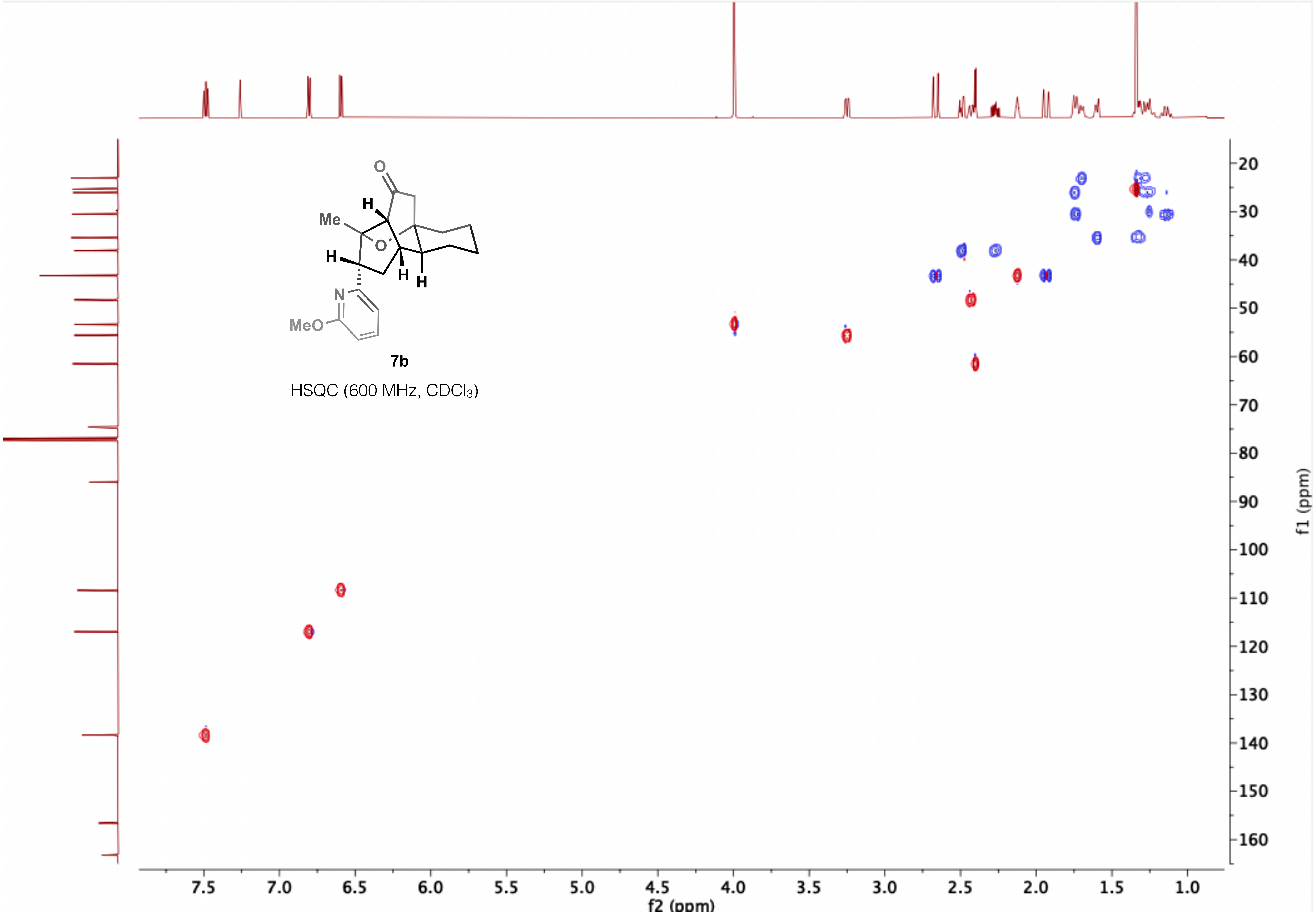

O
H
Me
O
H
H
H
N
MeO
7b
HSQC (600 MHz, CDCl3)
f2 (ppm)
f1 (ppm)

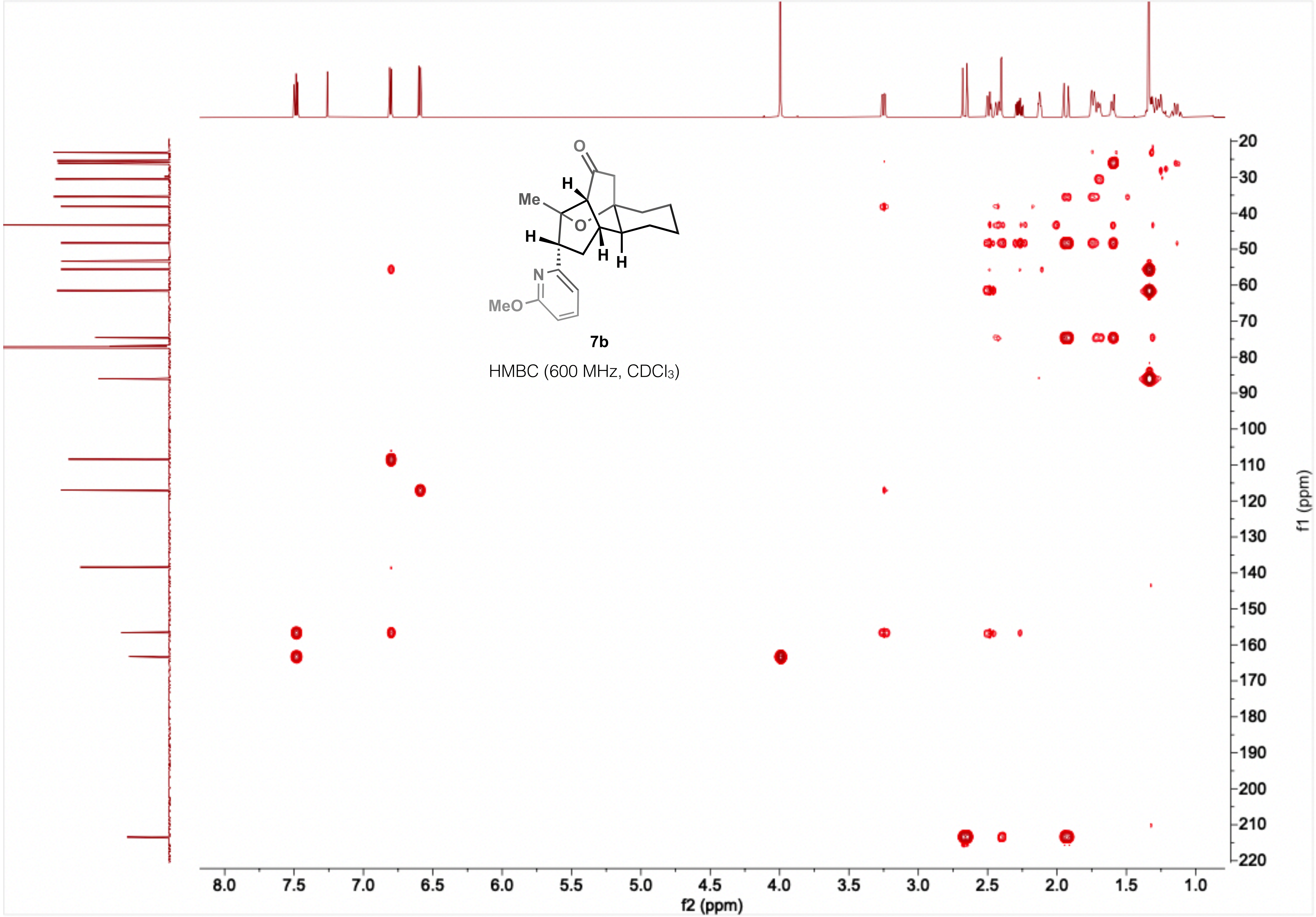
7b
HMBC (600 MHz, CDCl3)
f2 (ppm)
f1 (ppm)
Me
O
H
N
MeO

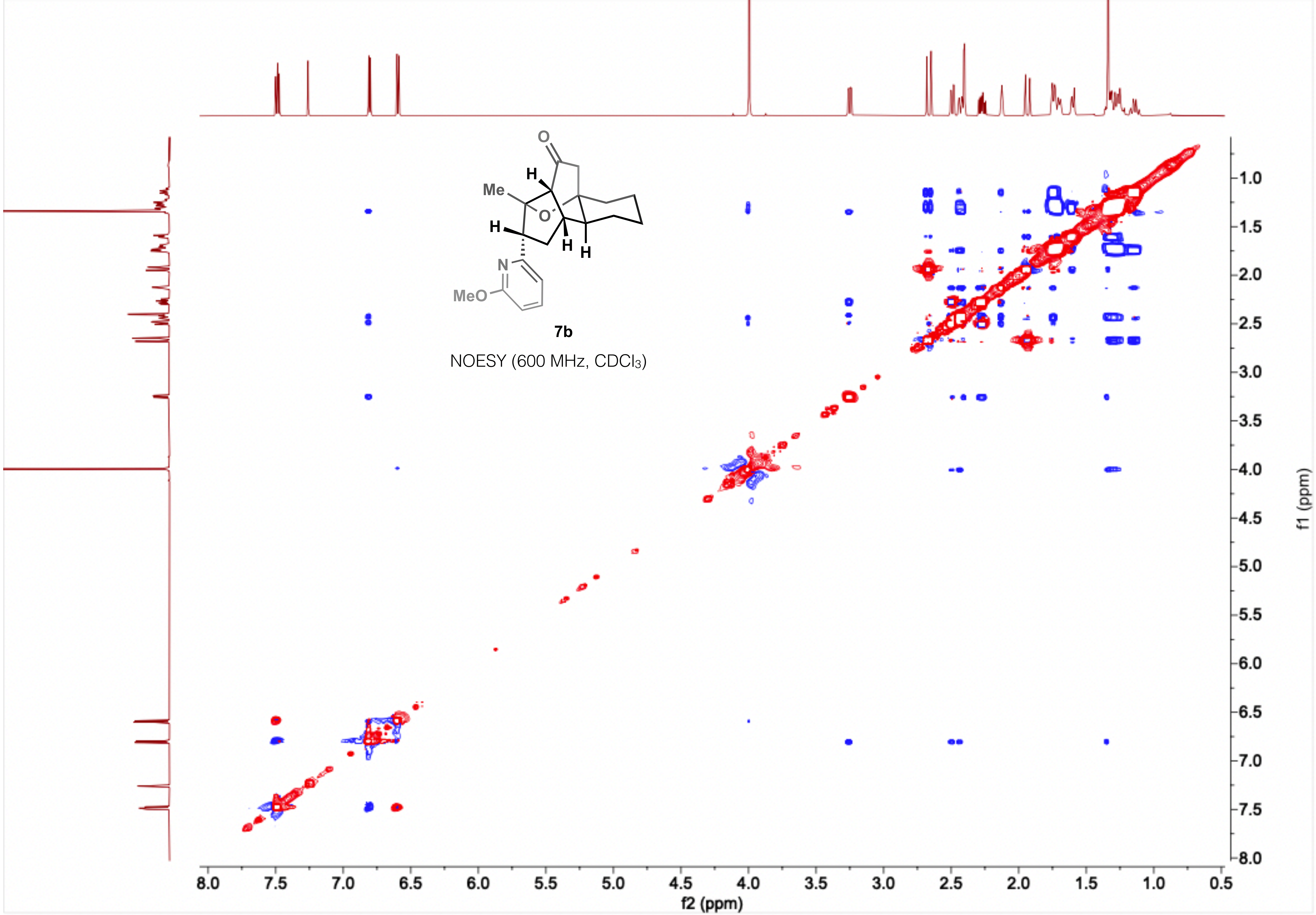
7b
NOESY (600 MHz, CDCl3)
f2 (ppm)
f1 (ppm)
Me
MeO
O
N
H

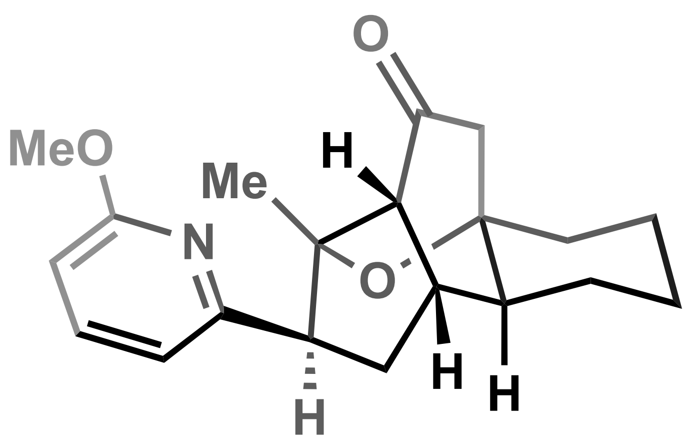

8b

(600 MHz, $CDCl_3$)

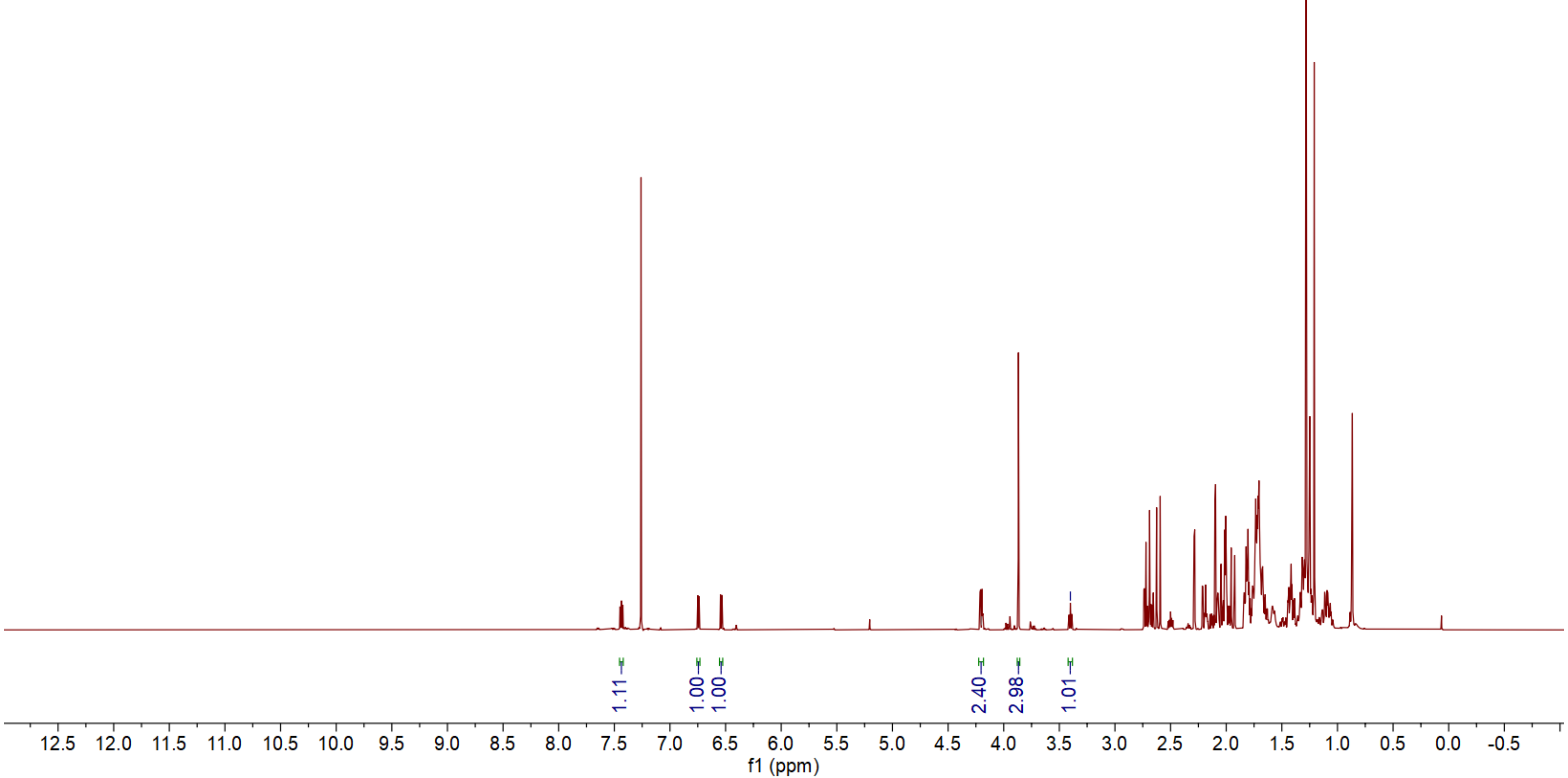

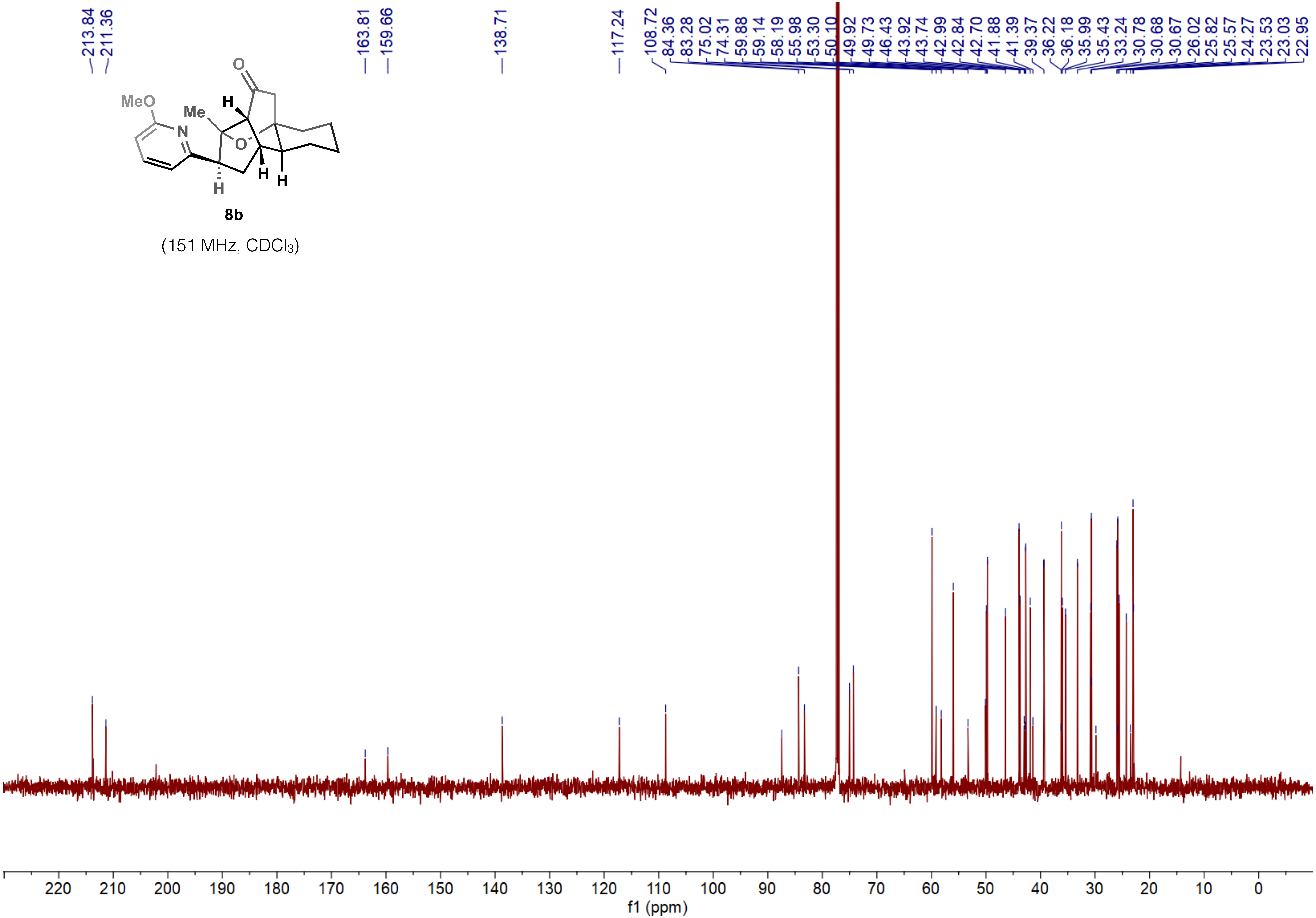
213.84
211.36
163.81
159.66
138.71
117.24
108.72
84.36
83.28
75.02
74.31
59.88
59.14
58.19
55.98
53.30
50.10
49.92
49.73
46.43
43.92
43.74
42.99
42.84
42.70
41.88
41.39
39.37
36.22
36.18
35.99
35.43
33.24
30.78
30.68
30.67
26.02
25.82
25.57
24.27
23.53
23.03
22.95
MeO
N
Me
H
O
H
H
H
8b
(151 MHz, CDCl3)
220 210 200 190 180 170 160 150 140 130 120 110 100 90 80 70 60 50 40 30 20 10 0
f1 (ppm)

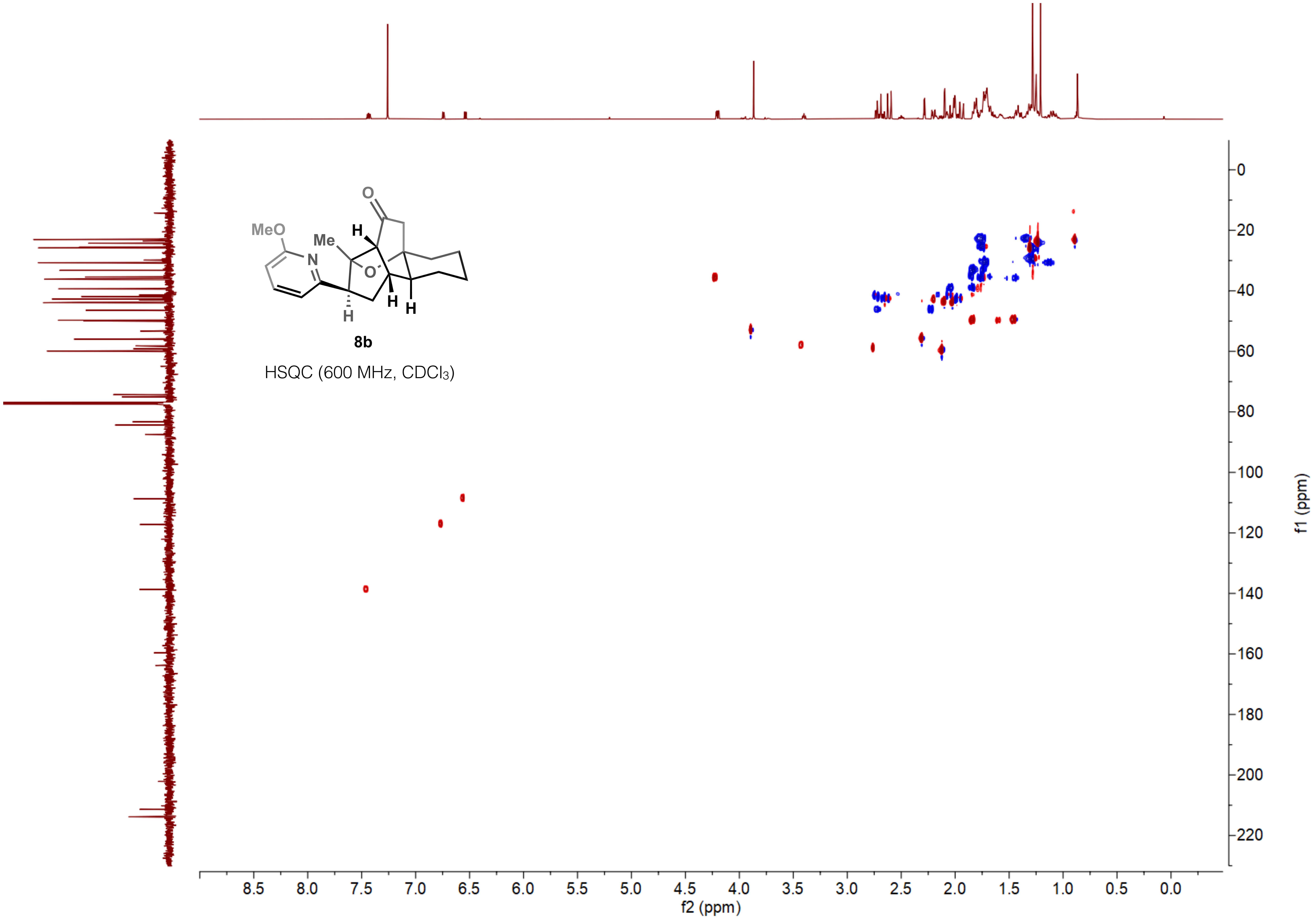

MeO
N
Me
O
H
H
H
H
8b
HSQC (600 MHz, CDCl3)
f1 (ppm)
f2 (ppm)

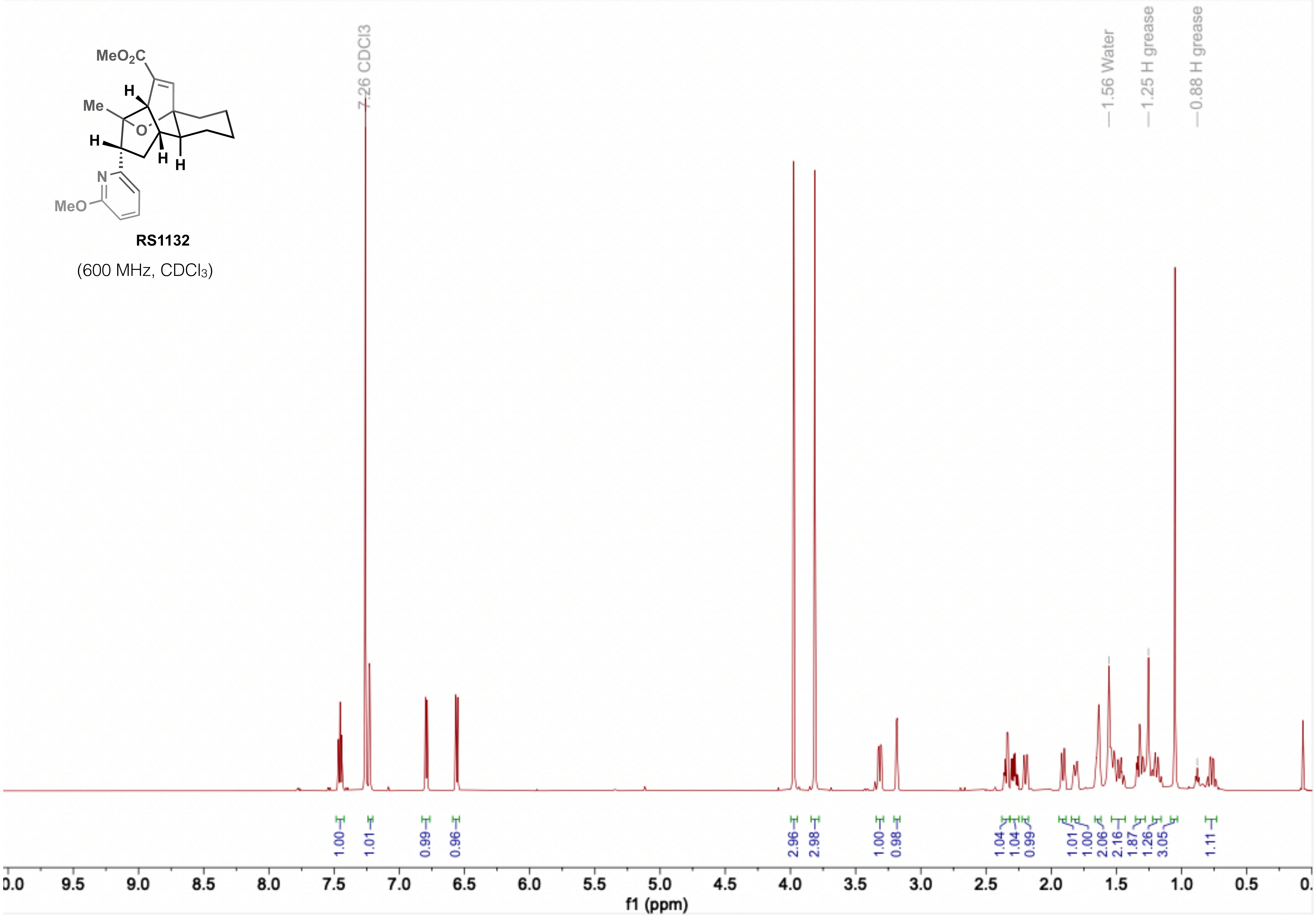

MeO2C
Me
H
O
H
H
H
N
MeO
RS1132
(600 MHz, CDCl3)
7.26 CDCl3
1.56 Water
1.25 H grease
0.88 H grease
1.00
1.01
0.99
0.96
2.96
2.98
1.00
0.98
1.04
1.04
0.99
1.01
1.00
2.06
2.16
1.87
1.26
3.05
1.11
f1 (ppm)

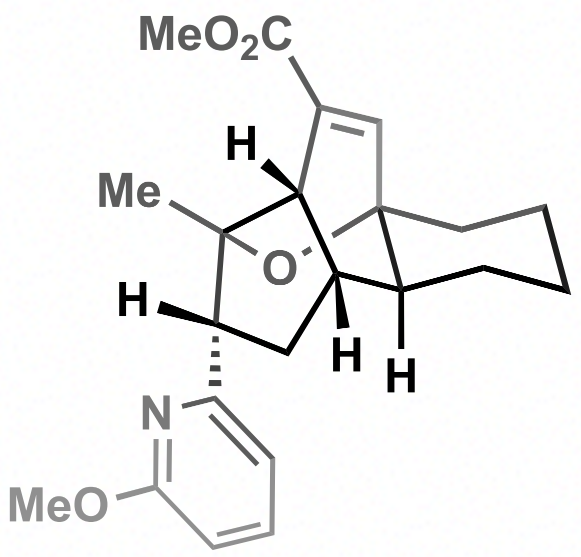

RS1132

(151 MHz, $CDCl_3$)

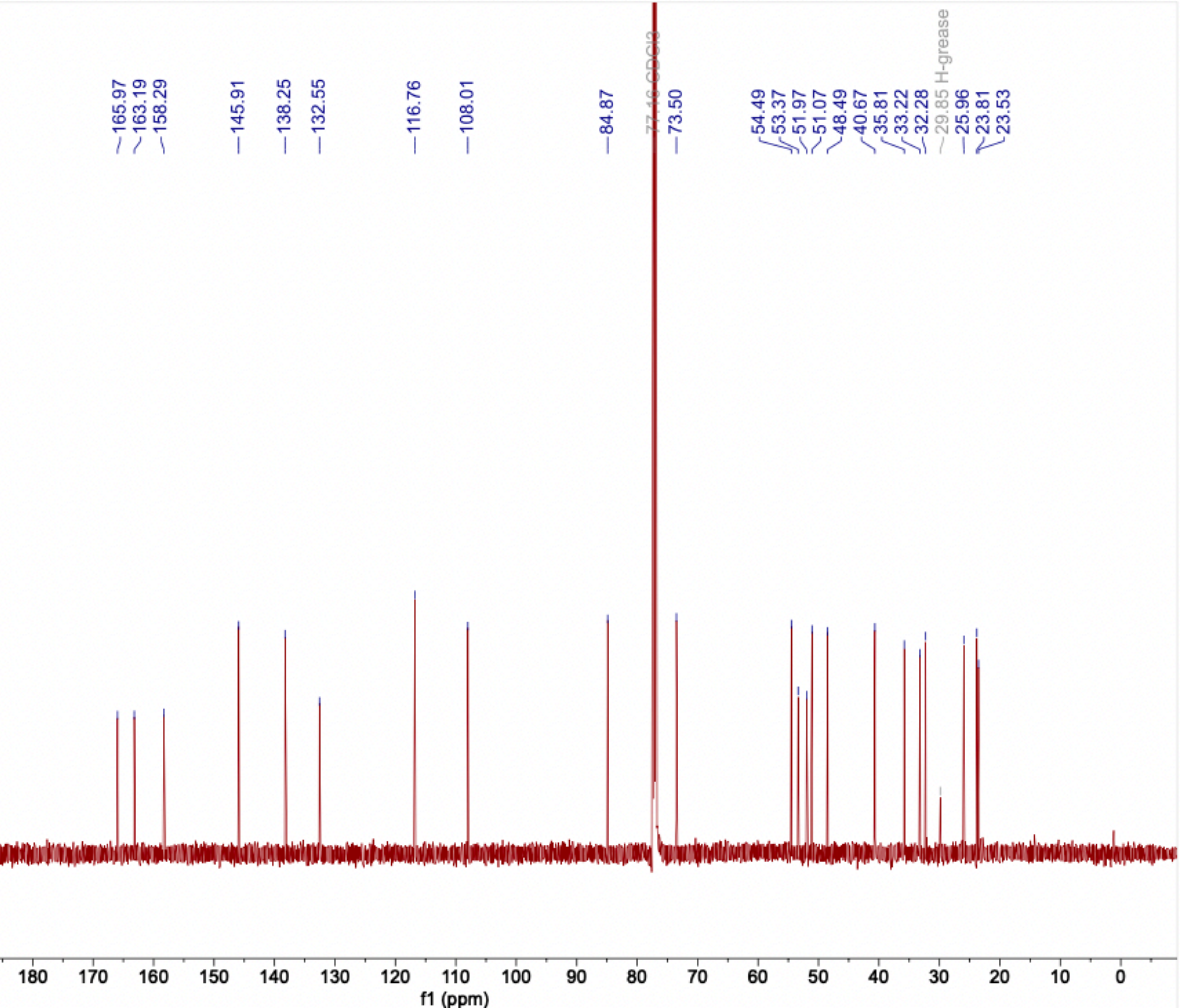

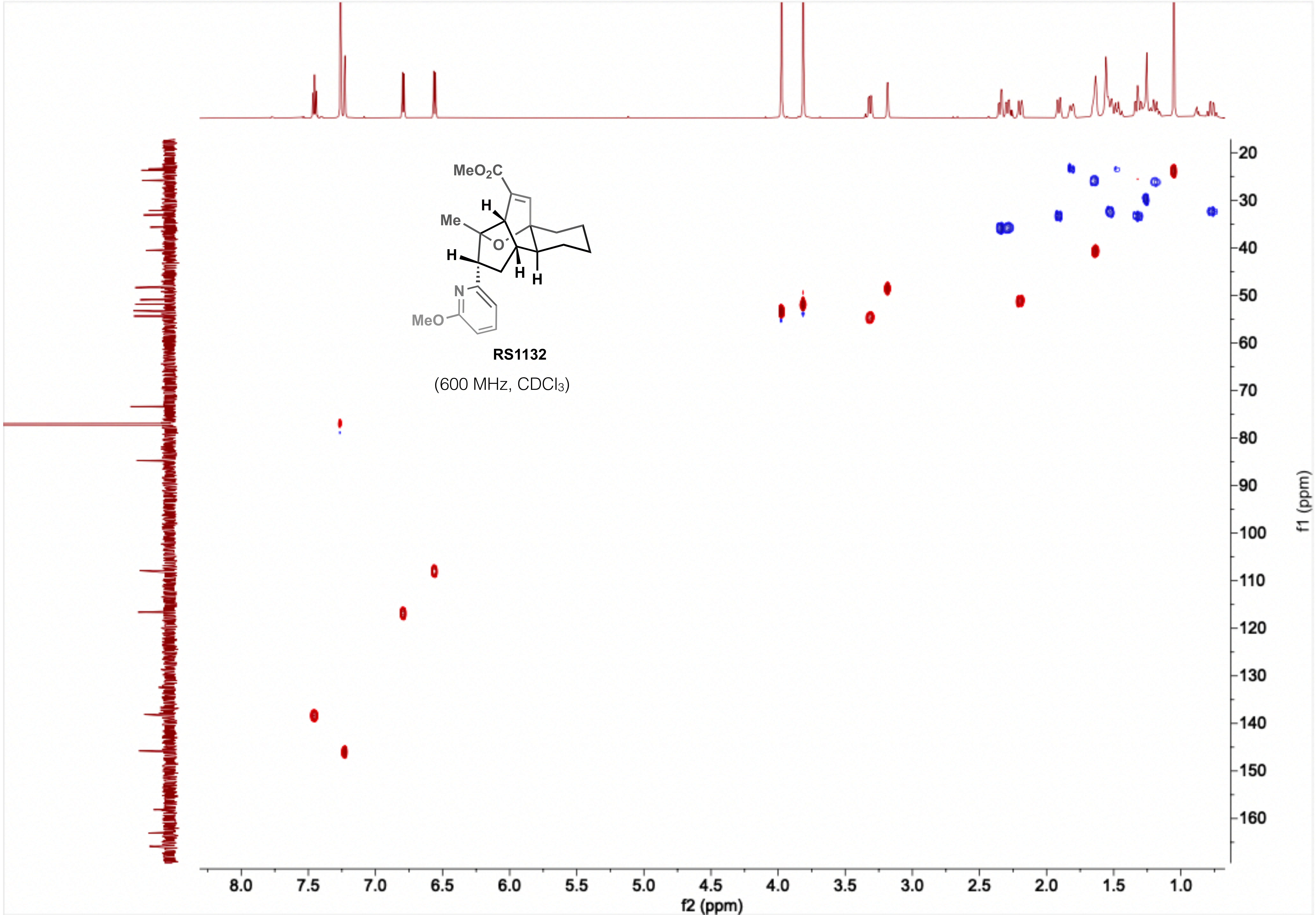

MeO2C
Me
H
O
H
H
H
N
MeO
RS1132
(600 MHz, CDCl3)
f2 (ppm)
f1 (ppm)

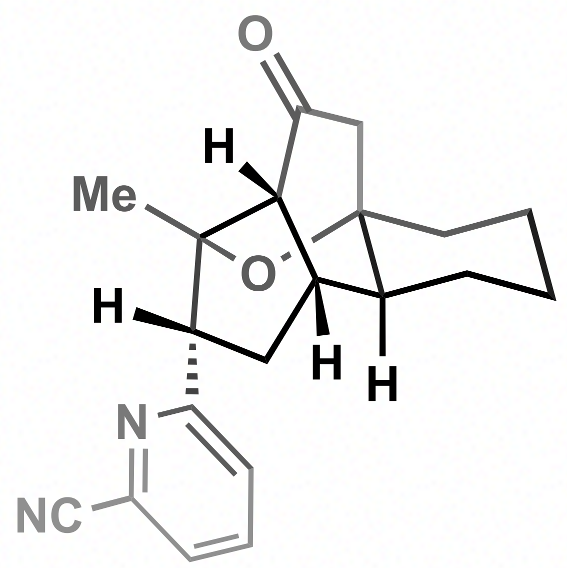

7c

(600 MHz, $CDCl_3$)

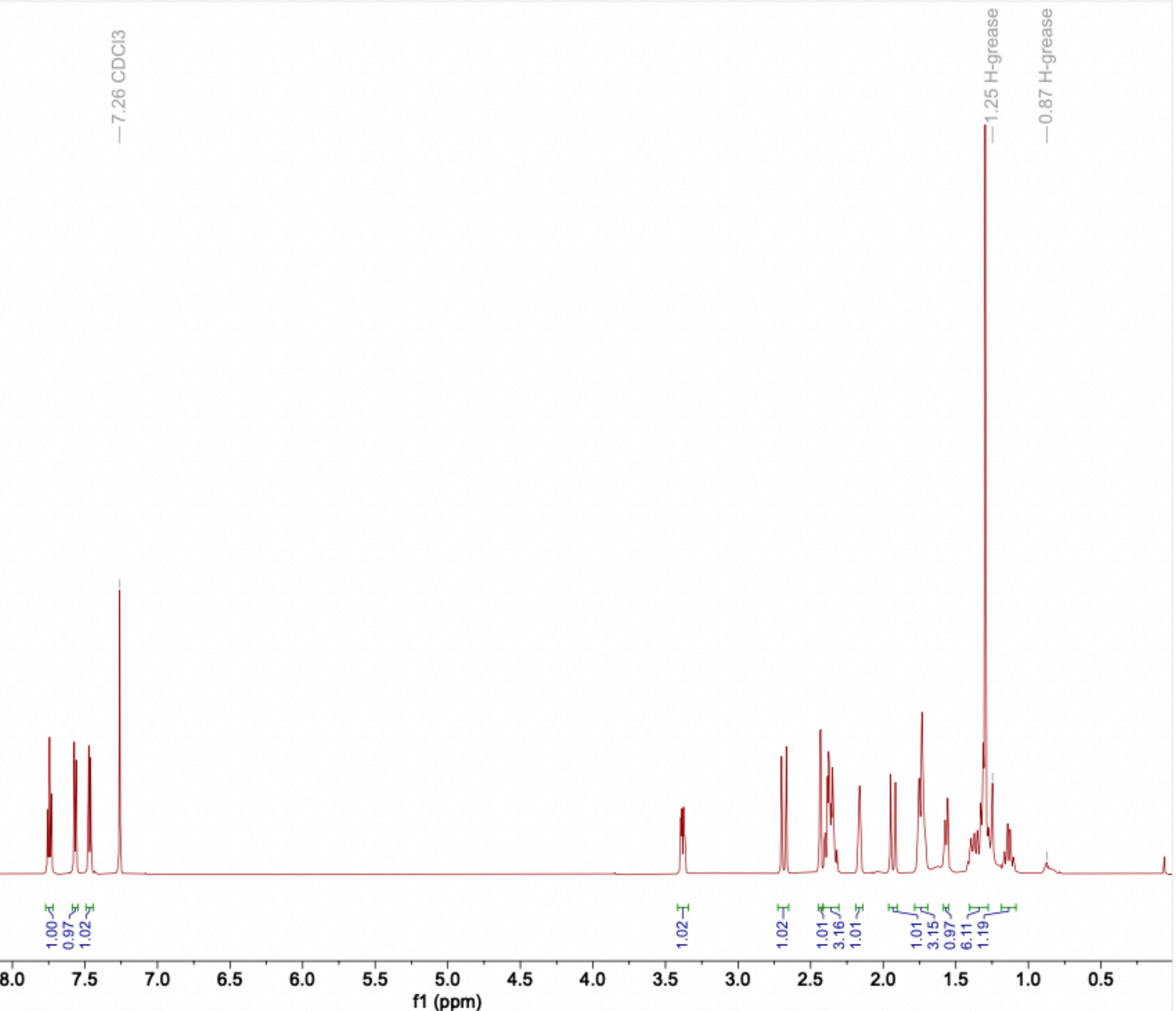

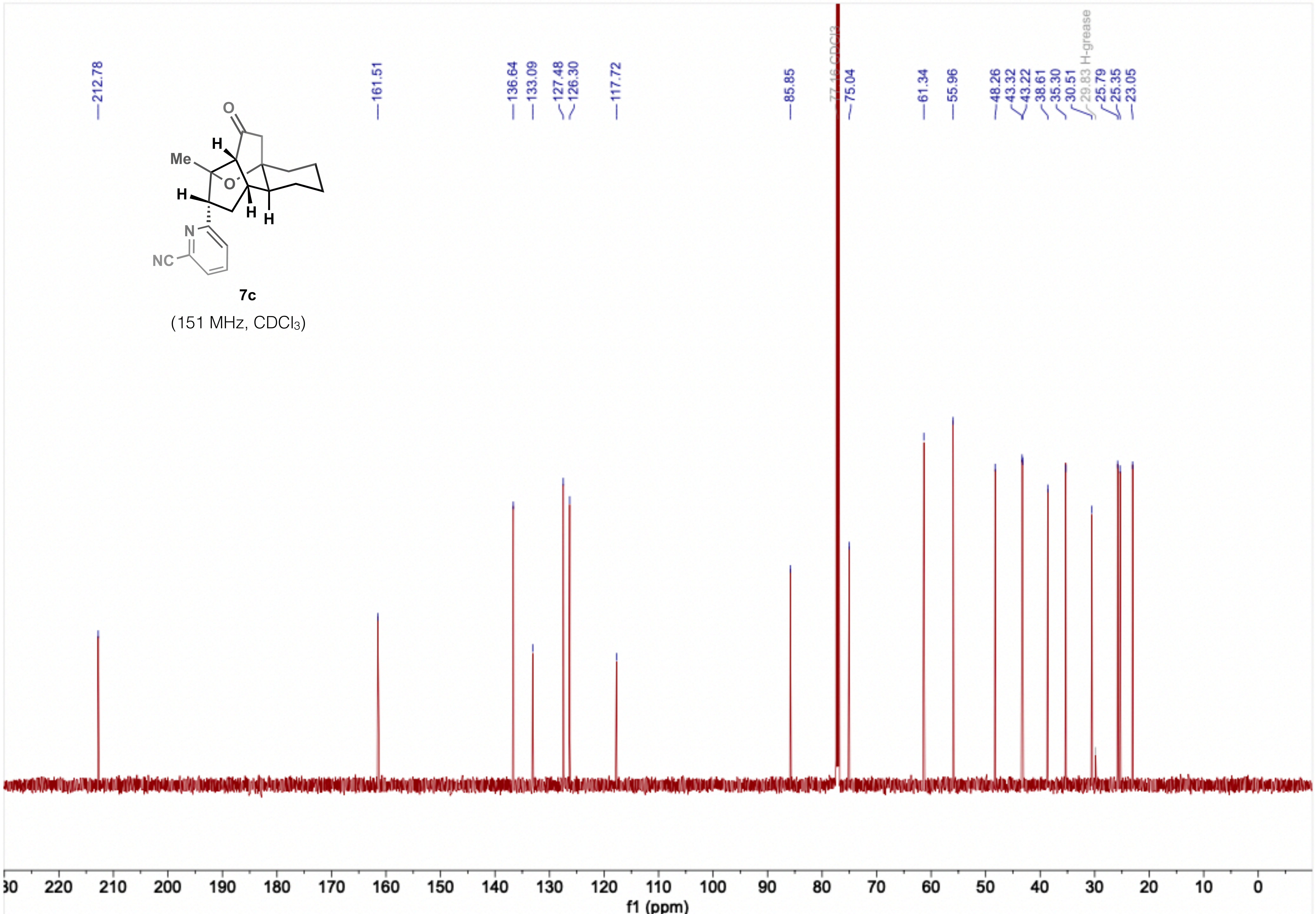

212.78
161.51
136.64
133.09
127.48
126.30
117.72
85.85
77.16 CDCl3
75.04
61.34
55.96
48.26
43.32
43.22
38.61
35.30
30.51
29.83 H-grease
25.79
25.35
23.05
O
H
Me
O
H
H
H
N
NC
7c
(151 MHz, CDCl3)
230
220
210
200
190
180
170
160
150
140
130
120
110
100
90
80
70
60
50
40
30
20
10
0
f1 (ppm)

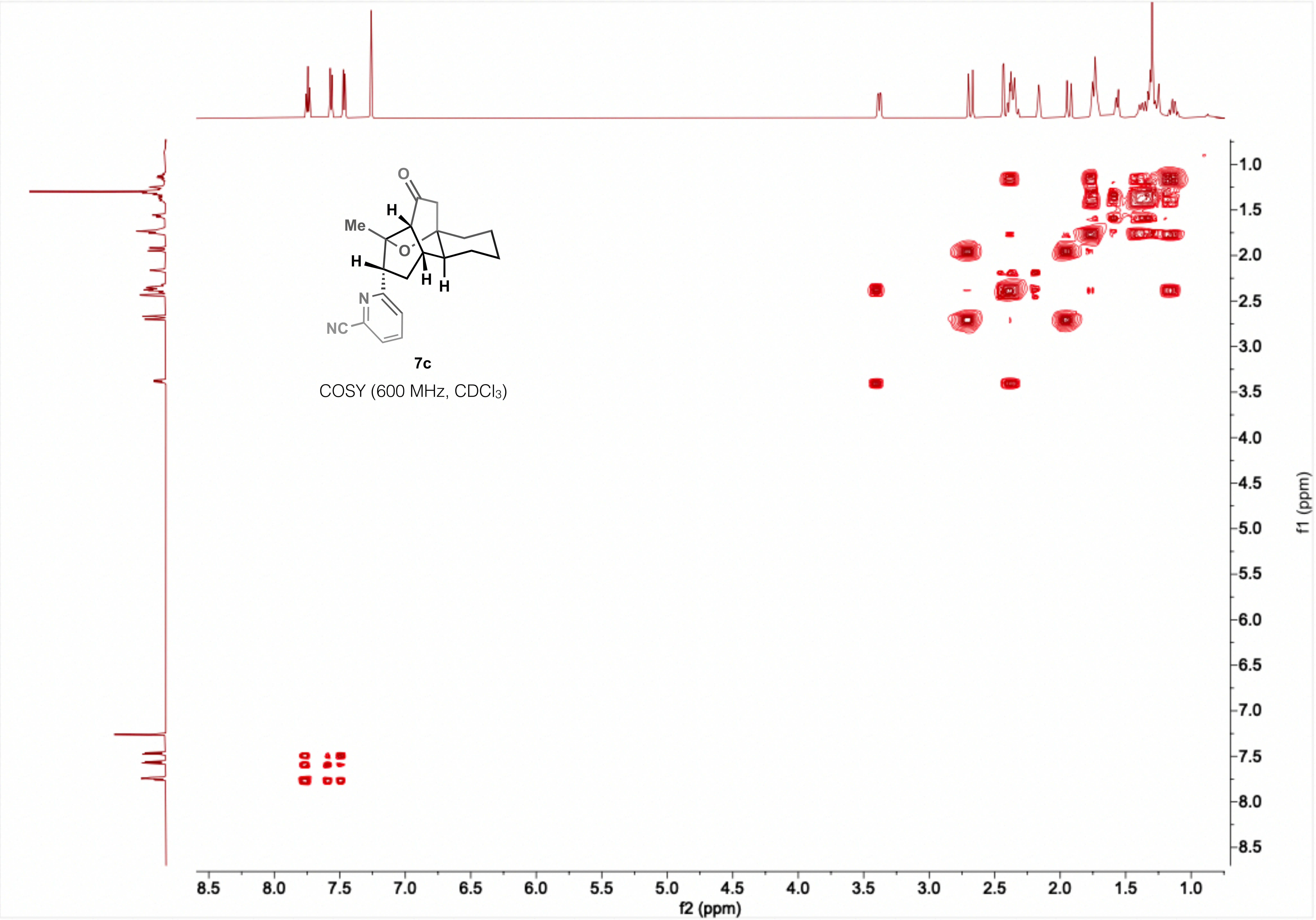

7c

COSY (600 MHz, $CDCl_3$)

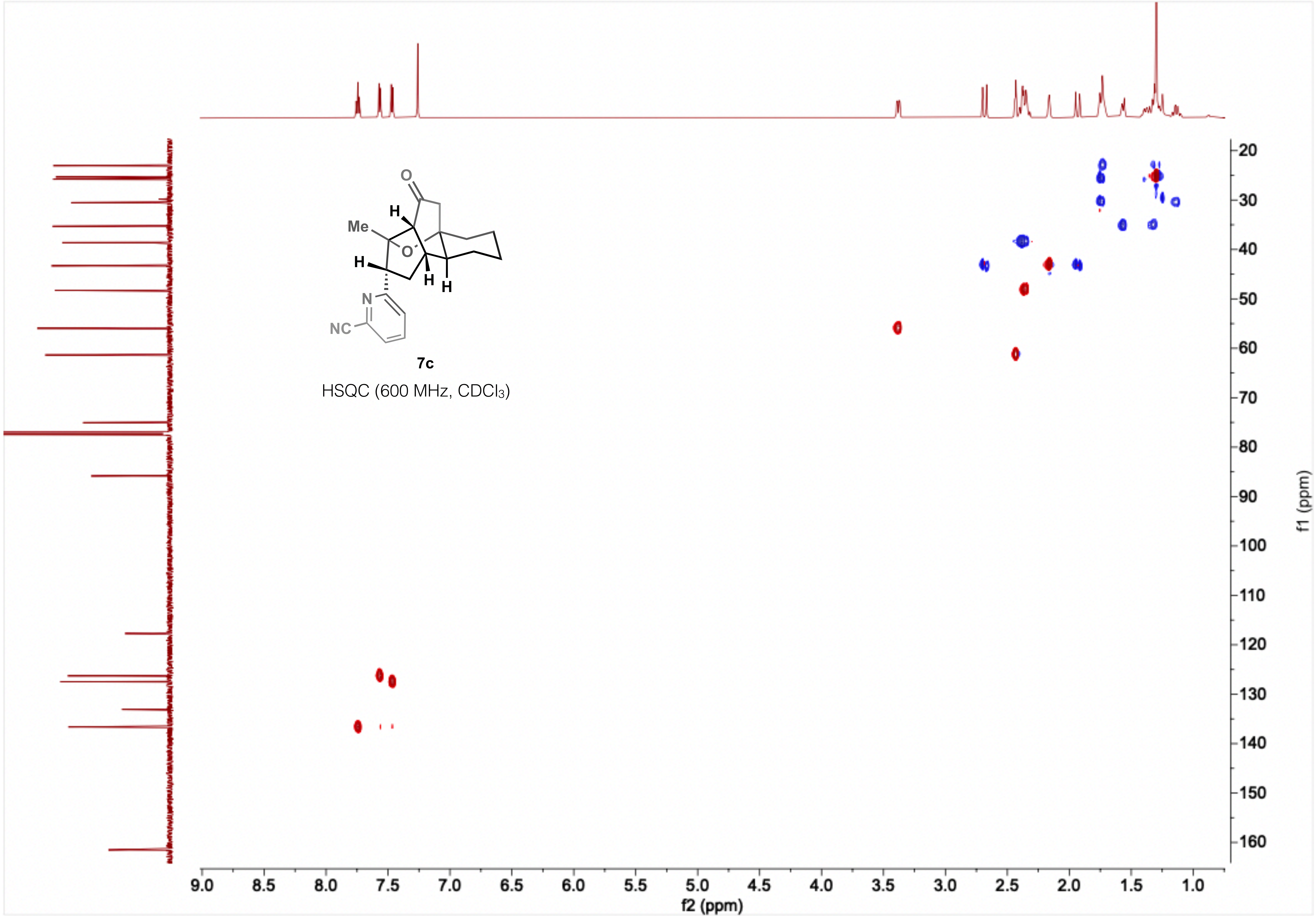

O
H
Me
O
H
H
H
N
NC
7c
HSQC (600 MHz, CDCl3)
f2 (ppm)
f1 (ppm)
9.0 8.5 8.0 7.5 7.0 6.5 6.0 5.5 5.0 4.5 4.0 3.5 3.0 2.5 2.0 1.5 1.0
20 30 40 50 60 70 80 90 100 110 120 130 140 150 160

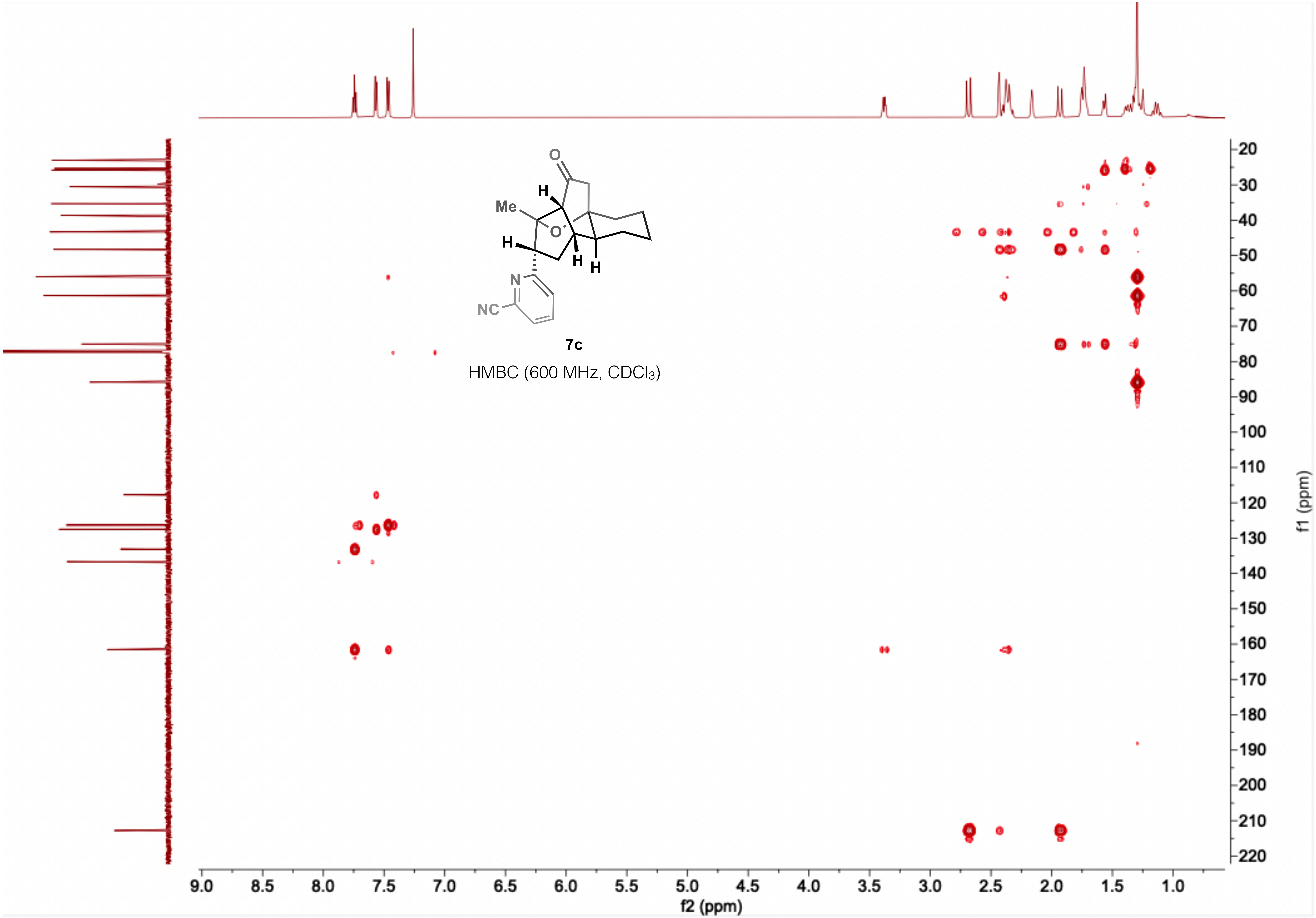

O
H
Me
O
H
H
H
N
NC
7c
HMBC (600 MHz, CDCl3)
f2 (ppm)
f1 (ppm)

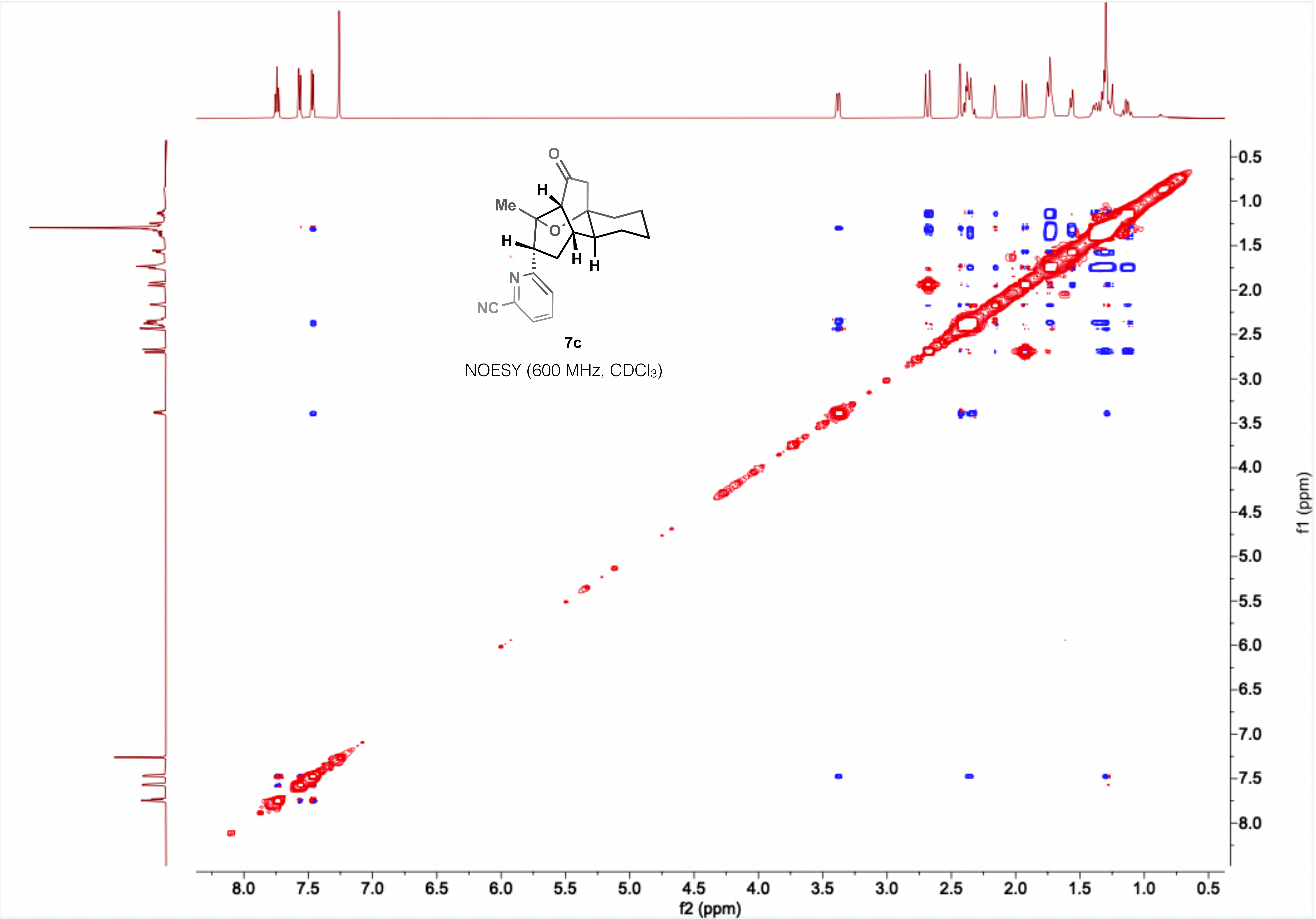

O
H
Me
O
H
H
H
N
NC
7c
NOESY (600 MHz, CDCl3)
f1 (ppm)
f2 (ppm)

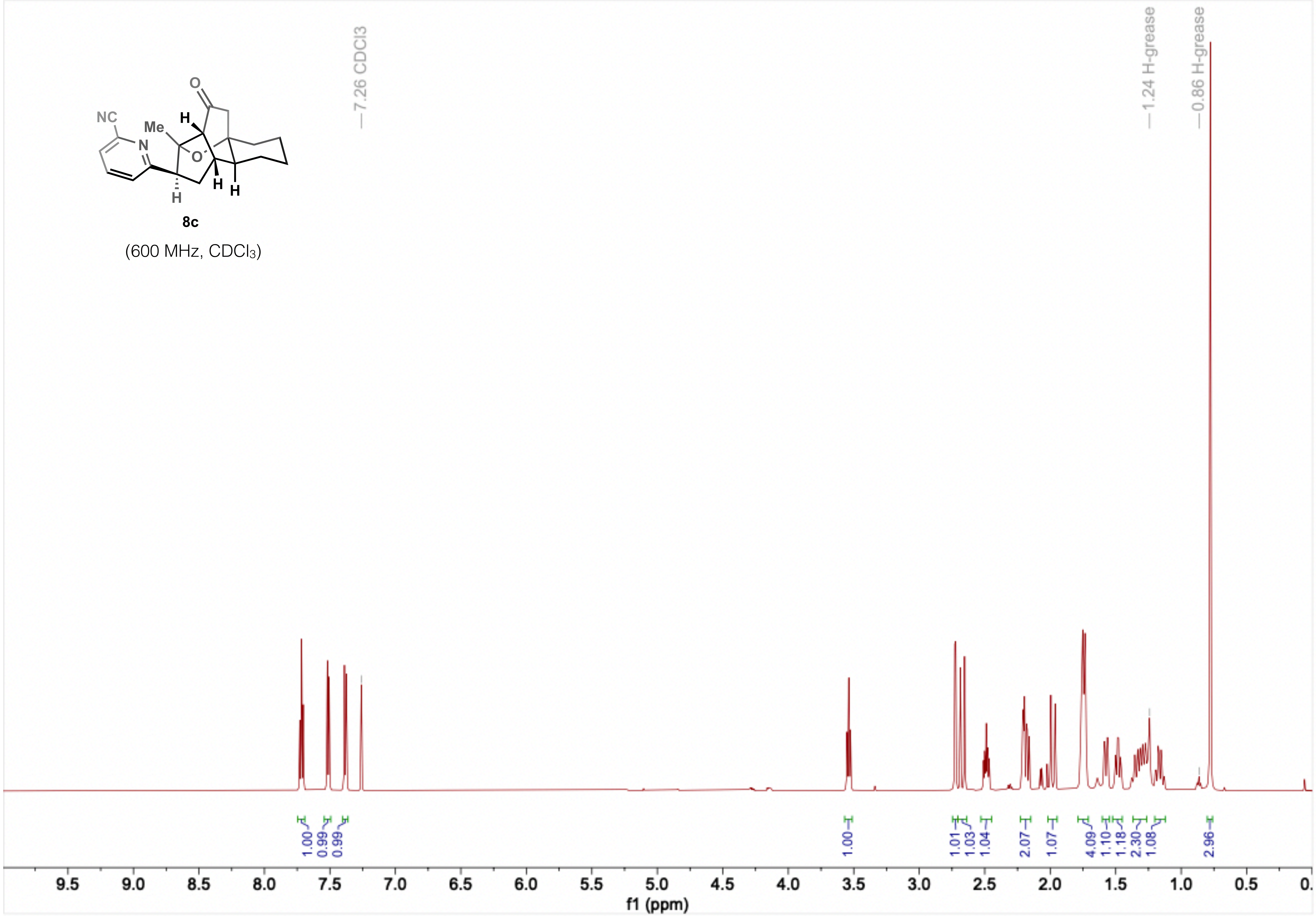

NC
Me
N
O
H
8c
(600 MHz, CDCl3)
7.26 CDCl3
1.24 H-grease
0.86 H-grease
1.00
0.99
0.99
1.00
1.01
1.03
1.04
2.07
1.07
4.09
1.10
1.18
2.30
1.08
2.96
9.5
9.0
8.5
8.0
7.5
7.0
6.5
6.0
5.5
5.0
4.5
4.0
3.5
3.0
2.5
2.0
1.5
1.0
0.5
0.
f1 (ppm)

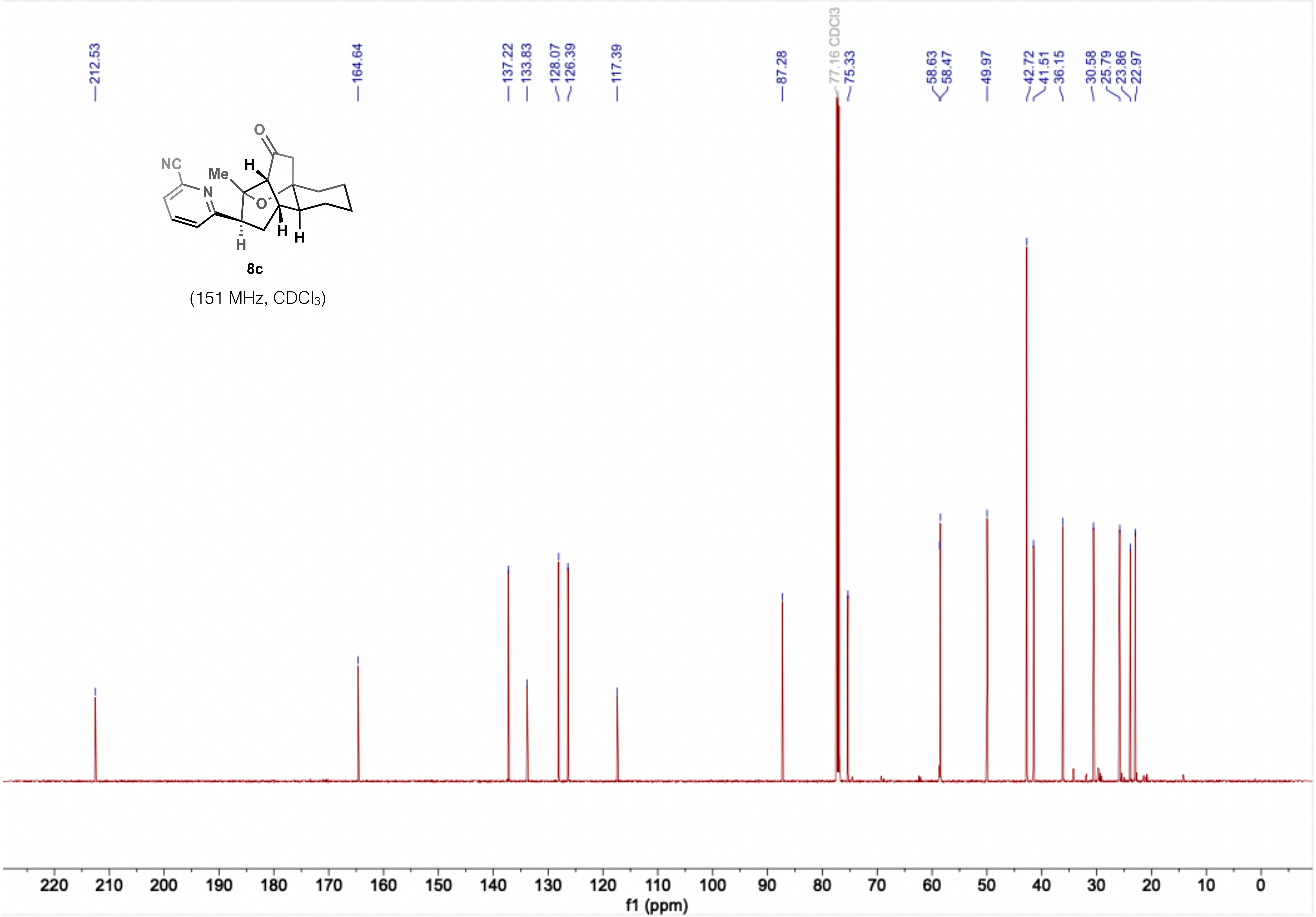
212.53
164.64
137.22
133.83
128.07
126.39
117.39
87.28
77.16 CDCl3
75.33
58.63
58.47
49.97
42.72
41.51
36.15
30.58
25.79
23.86
22.97
NC
N
Me
H
O
O
H
H
H
8c
(151 MHz, CDCl3)
220
210
200
190
180
170
160
150
140
130
120
110
100
90
80
70
60
50
40
30
20
10
0
f1 (ppm)

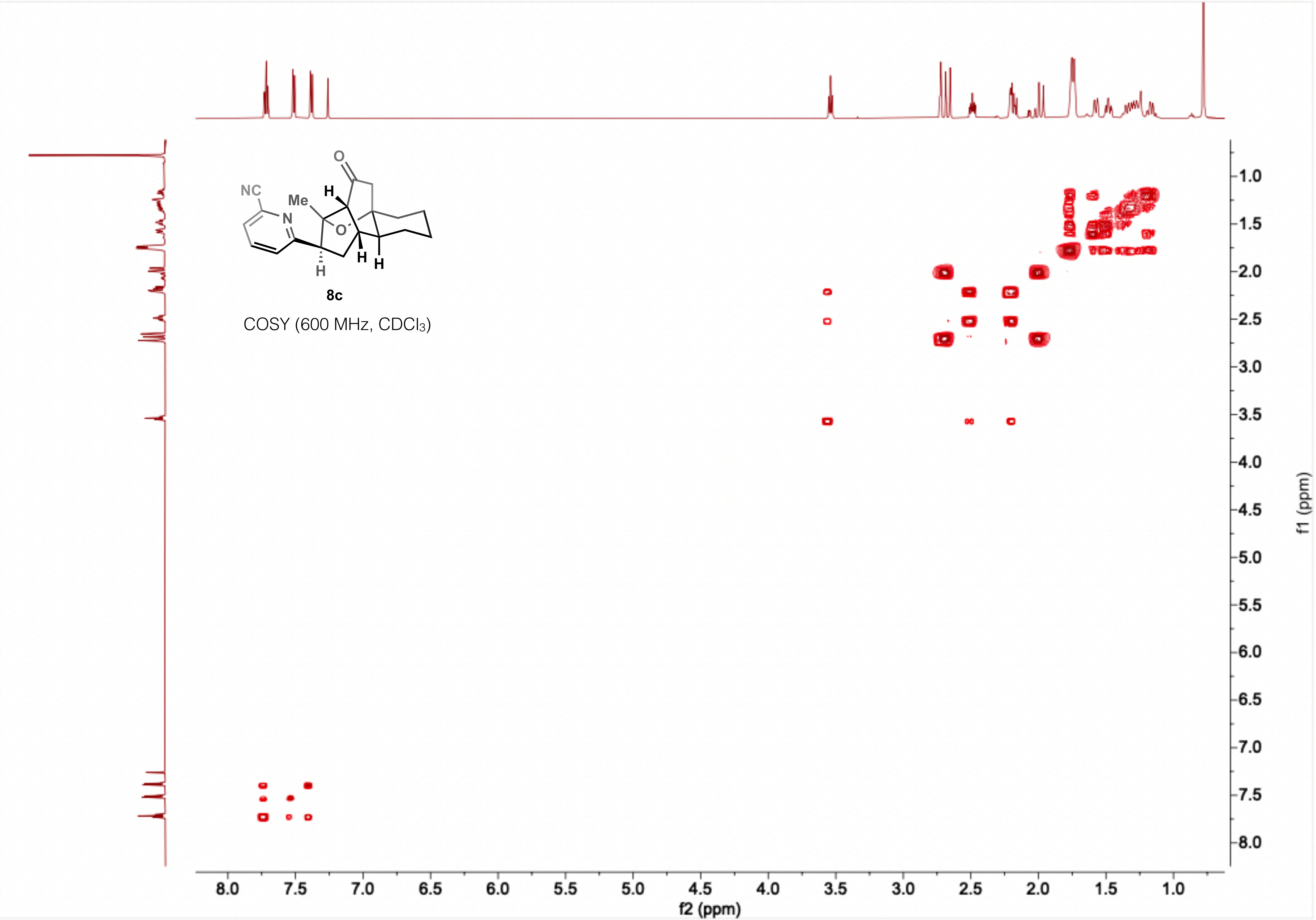
NC
Me
H
O
N
O
H
H
H
8c
COSY (600 MHz, CDCl3)
f2 (ppm)
f1 (ppm)
8.0 7.5 7.0 6.5 6.0 5.5 5.0 4.5 4.0 3.5 3.0 2.5 2.0 1.5 1.0
1.0 1.5 2.0 2.5 3.0 3.5 4.0 4.5 5.0 5.5 6.0 6.5 7.0 7.5 8.0

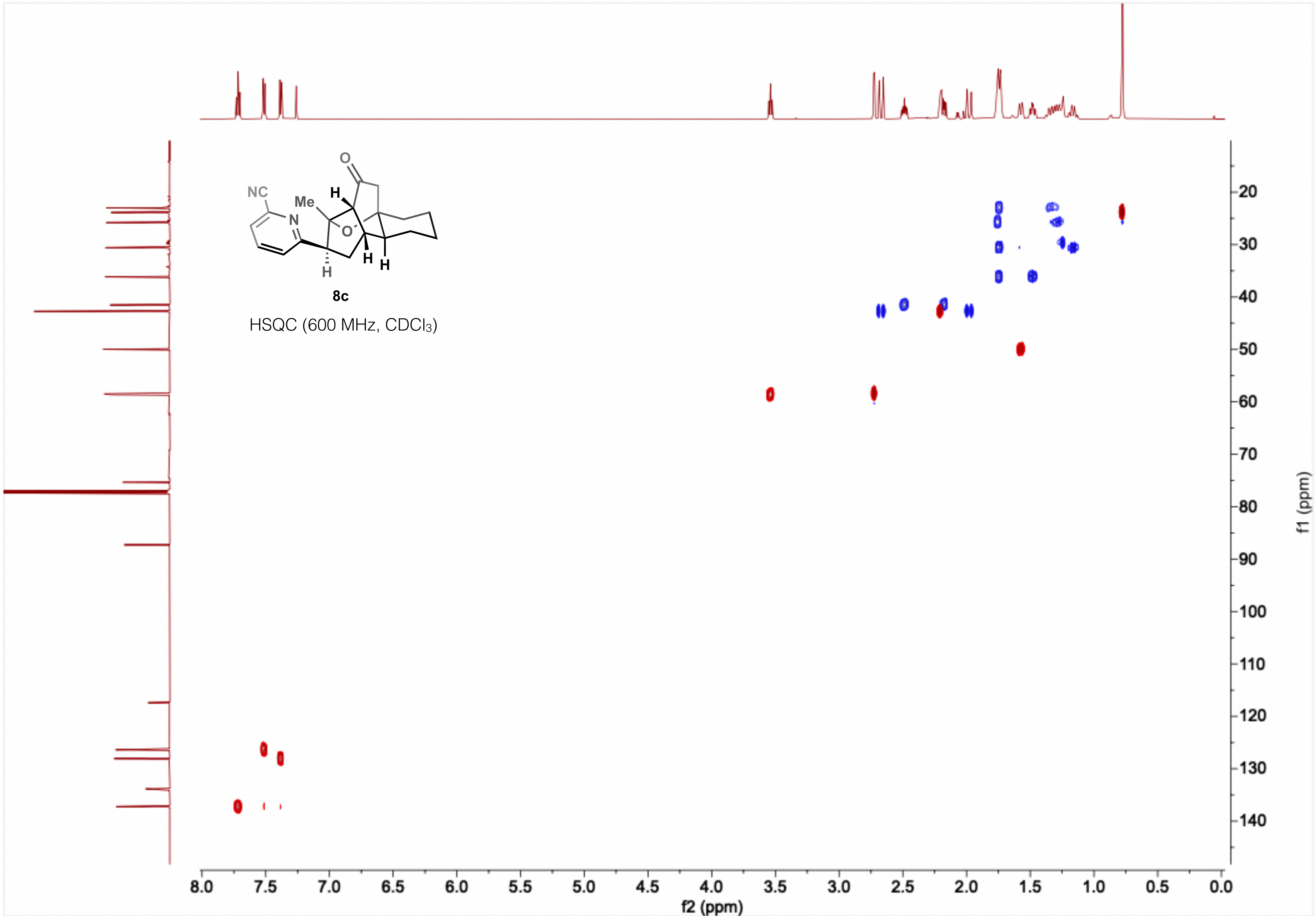
NC
Me
N
O
H
O
H
H
H
8c
HSQC (600 MHz, CDCl3)
f1 (ppm)
f2 (ppm)
20
30
40
50
60
70
80
90
100
110
120
130
140
8.0
7.5
7.0
6.5
6.0
5.5
5.0
4.5
4.0
3.5
3.0
2.5
2.0
1.5
1.0
0.5
0.0

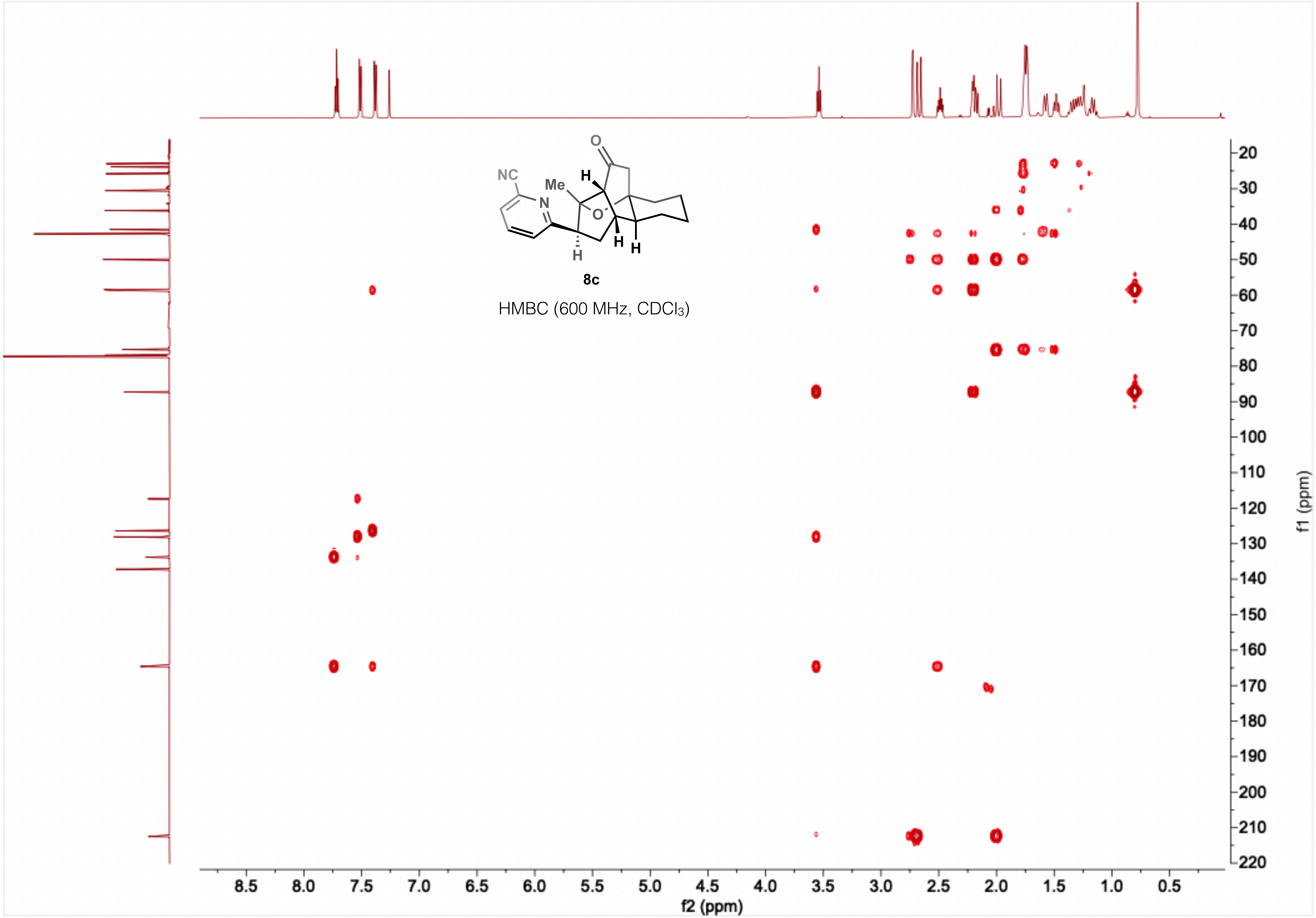

NC
N
Me
H
O
O
H
H
H
8c
HMBC (600 MHz, CDCl3)
f2 (ppm)
f1 (ppm)

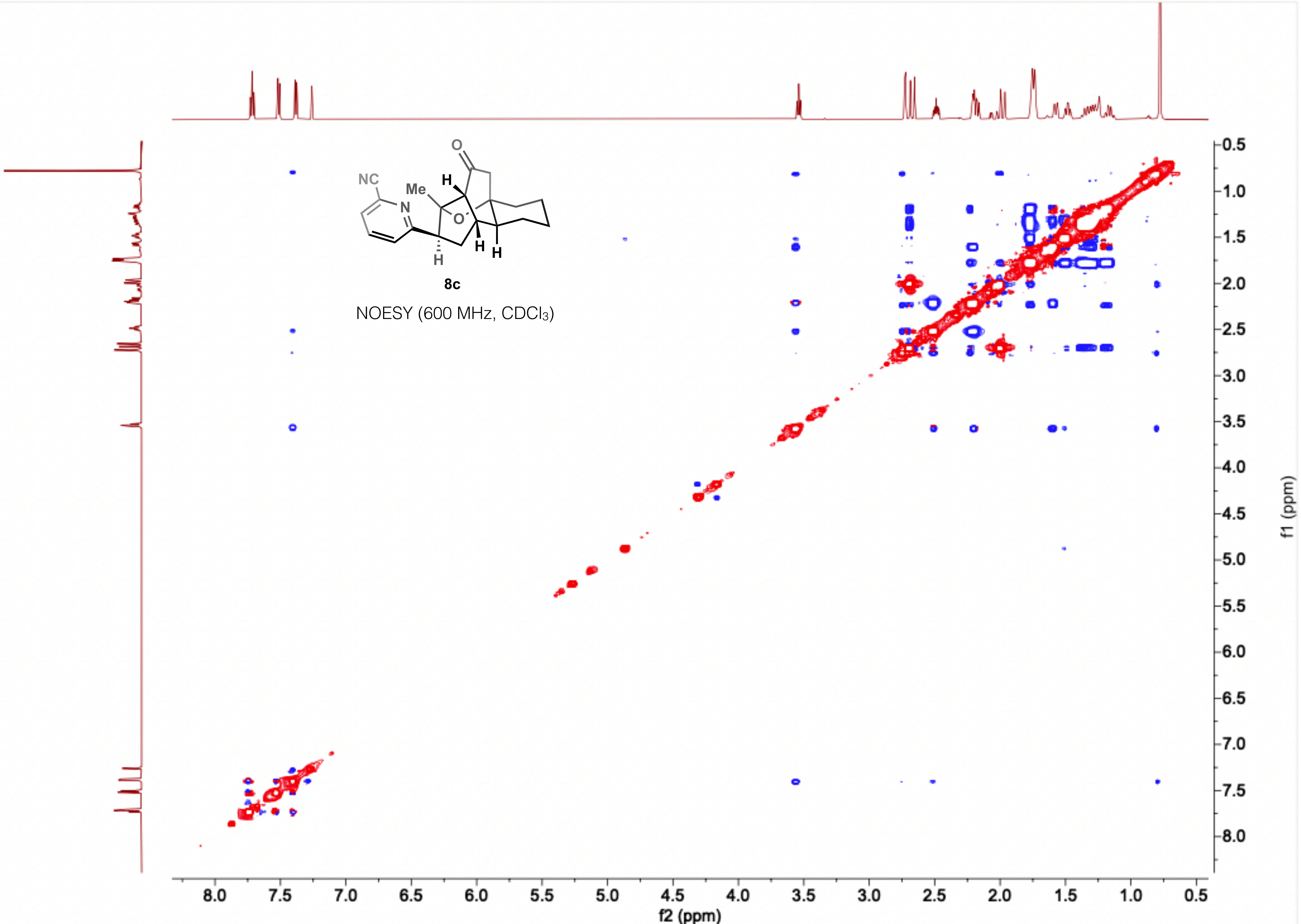

NC
Me
N
O
O
H
H
H
H
8c
NOESY (600 MHz, CDCl3)
f2 (ppm)
f1 (ppm)

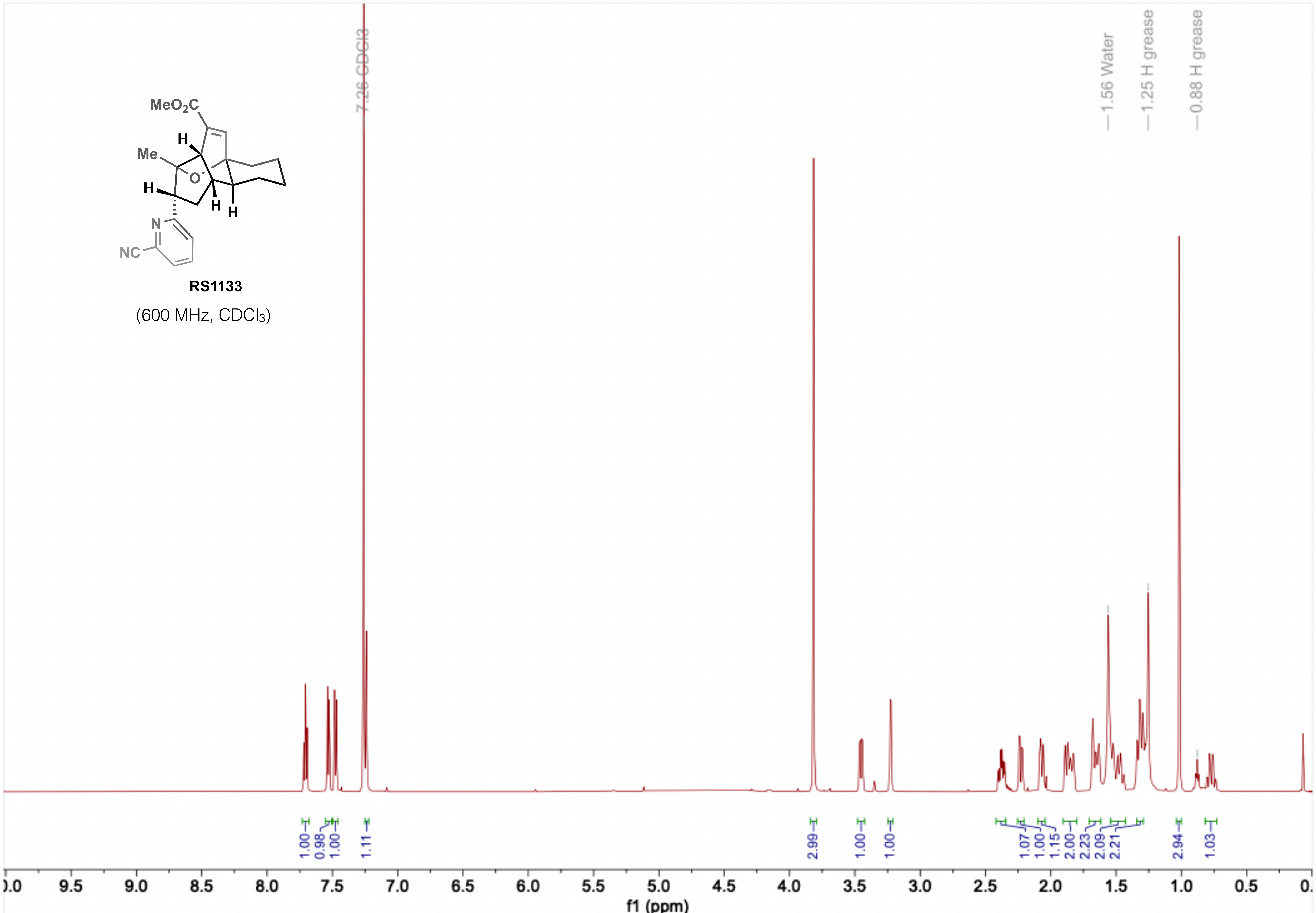

MeO2C
Me
H
O
H
H
H
N
NC
RS1133
(600 MHz, CDCl3)
7.26 CDCl3
1.56 Water
1.25 H grease
0.88 H grease
1.00
0.98
1.00
1.11
2.99
1.00
1.00
1.07
1.00
1.15
2.00
2.23
2.09
2.21
2.94
1.03
9.5
9.0
8.5
8.0
7.5
7.0
6.5
6.0
5.5
5.0
4.5
4.0
3.5
3.0
2.5
2.0
1.5
1.0
0.5
f1 (ppm)

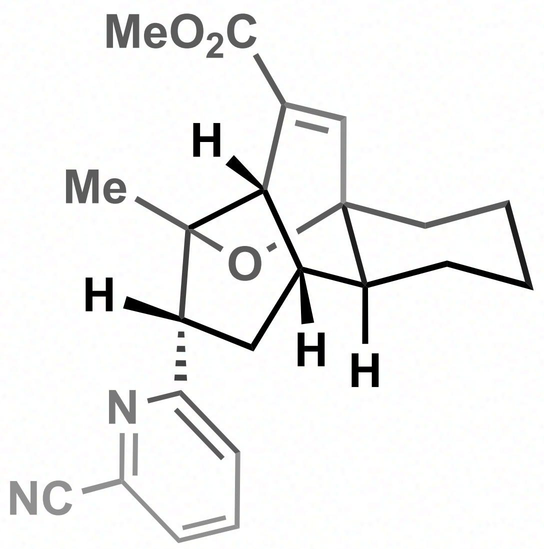

RS1133

(151 MHz, $CDCl_3$)

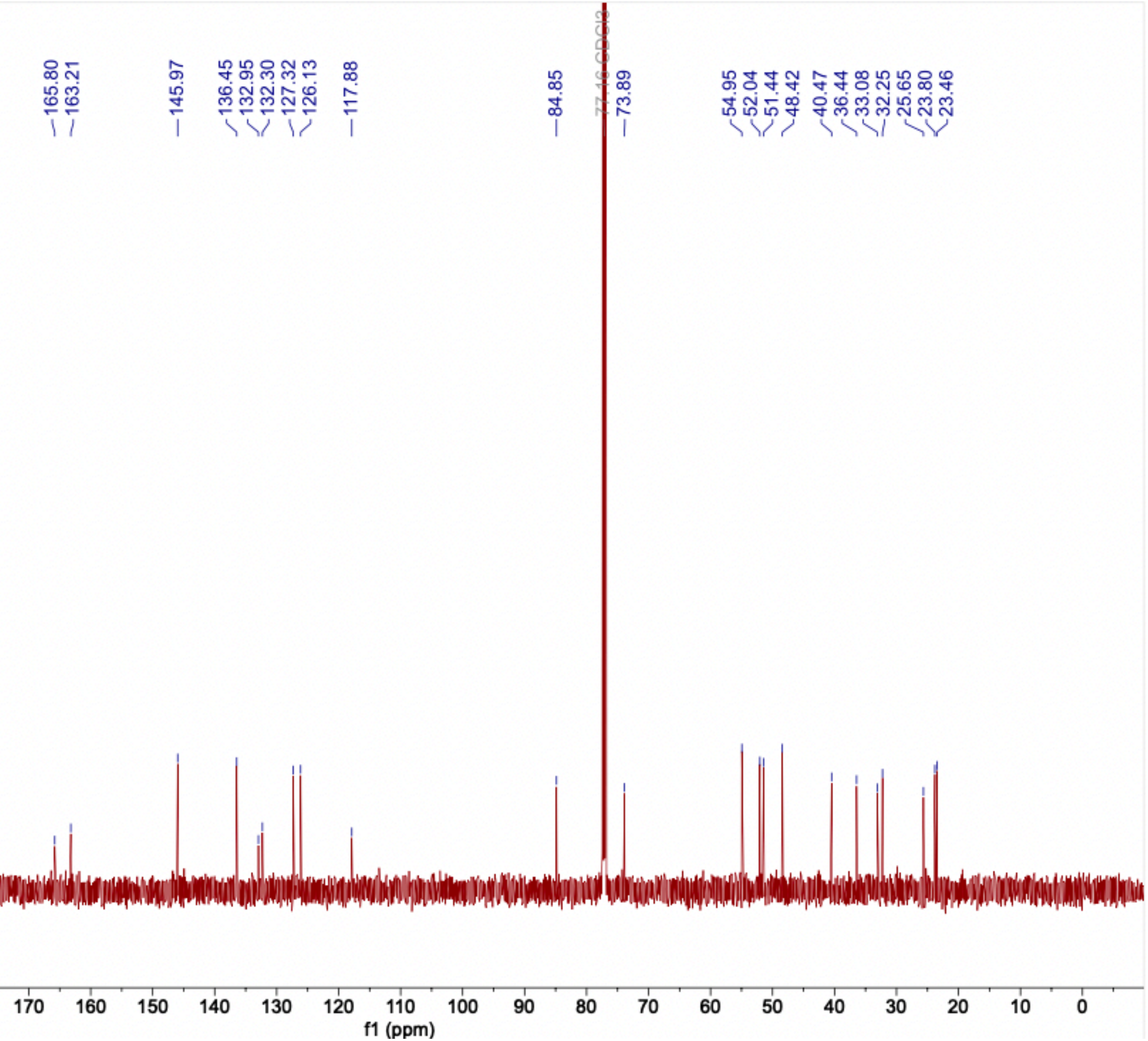

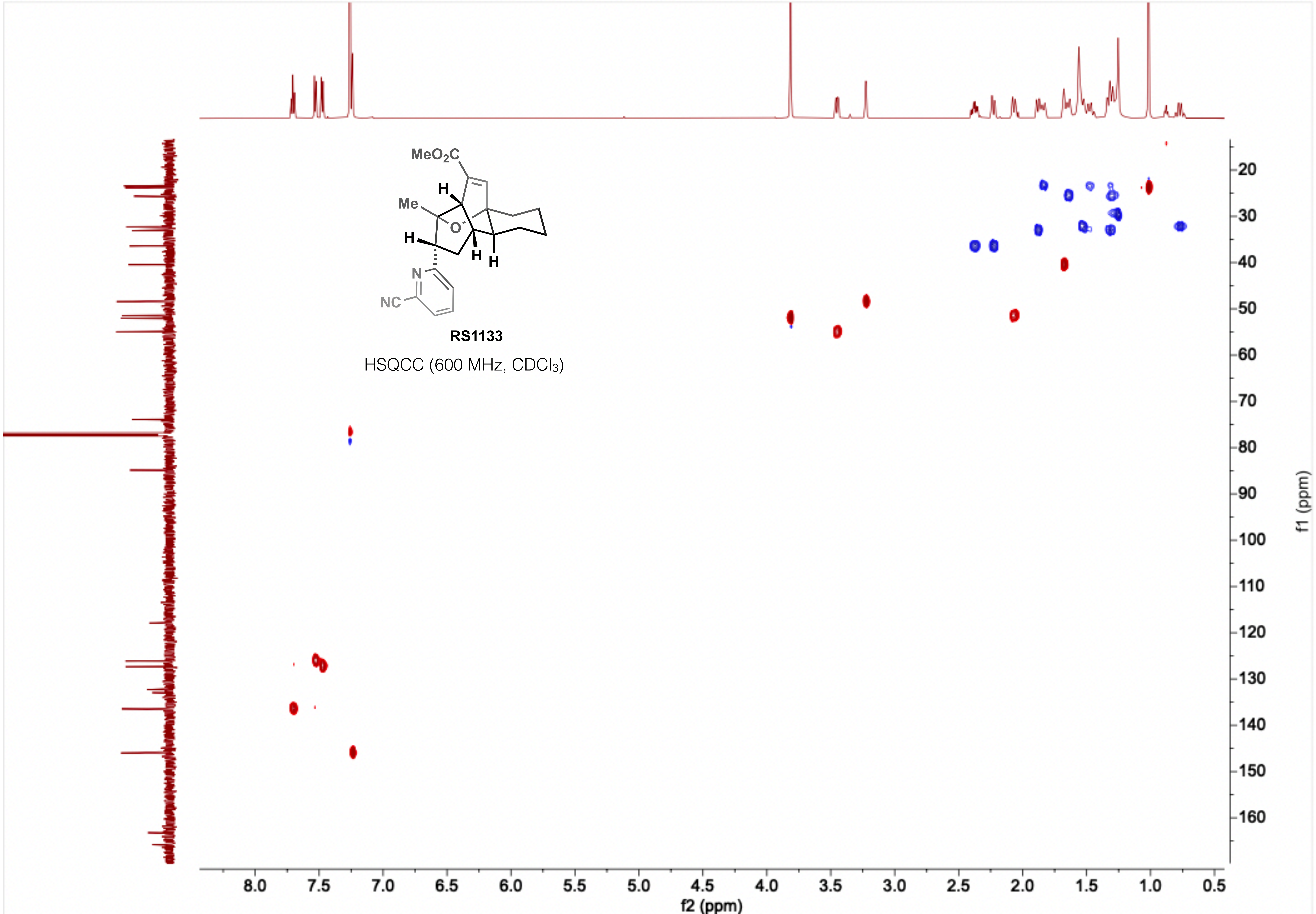
MeO2C
Me
H
O
H
H
H
N
NC
RS1133
HSQCC (600 MHz, CDCl3)
f1 (ppm)
f2 (ppm)

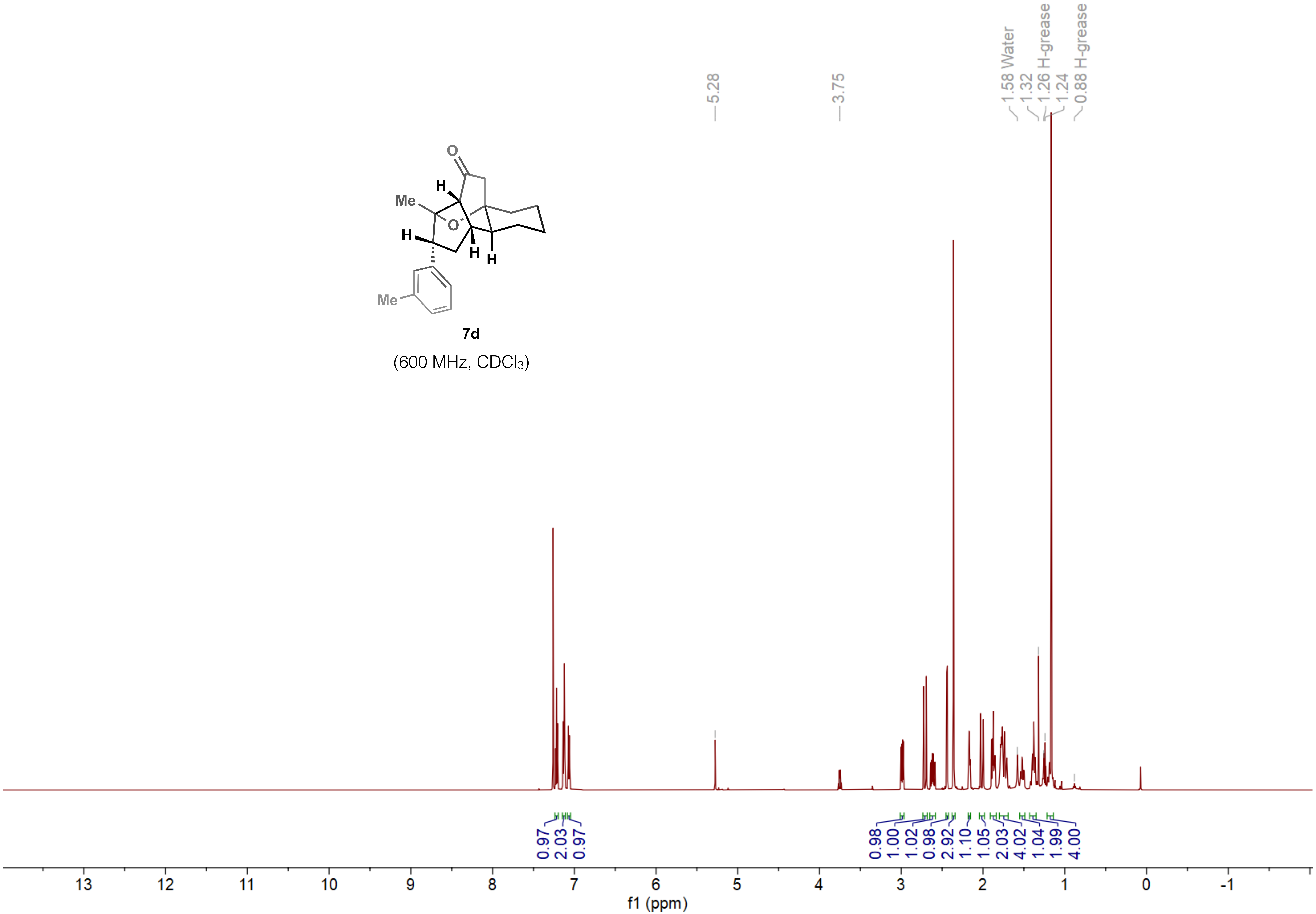

7d

(600 MHz, $CDCl_3$)

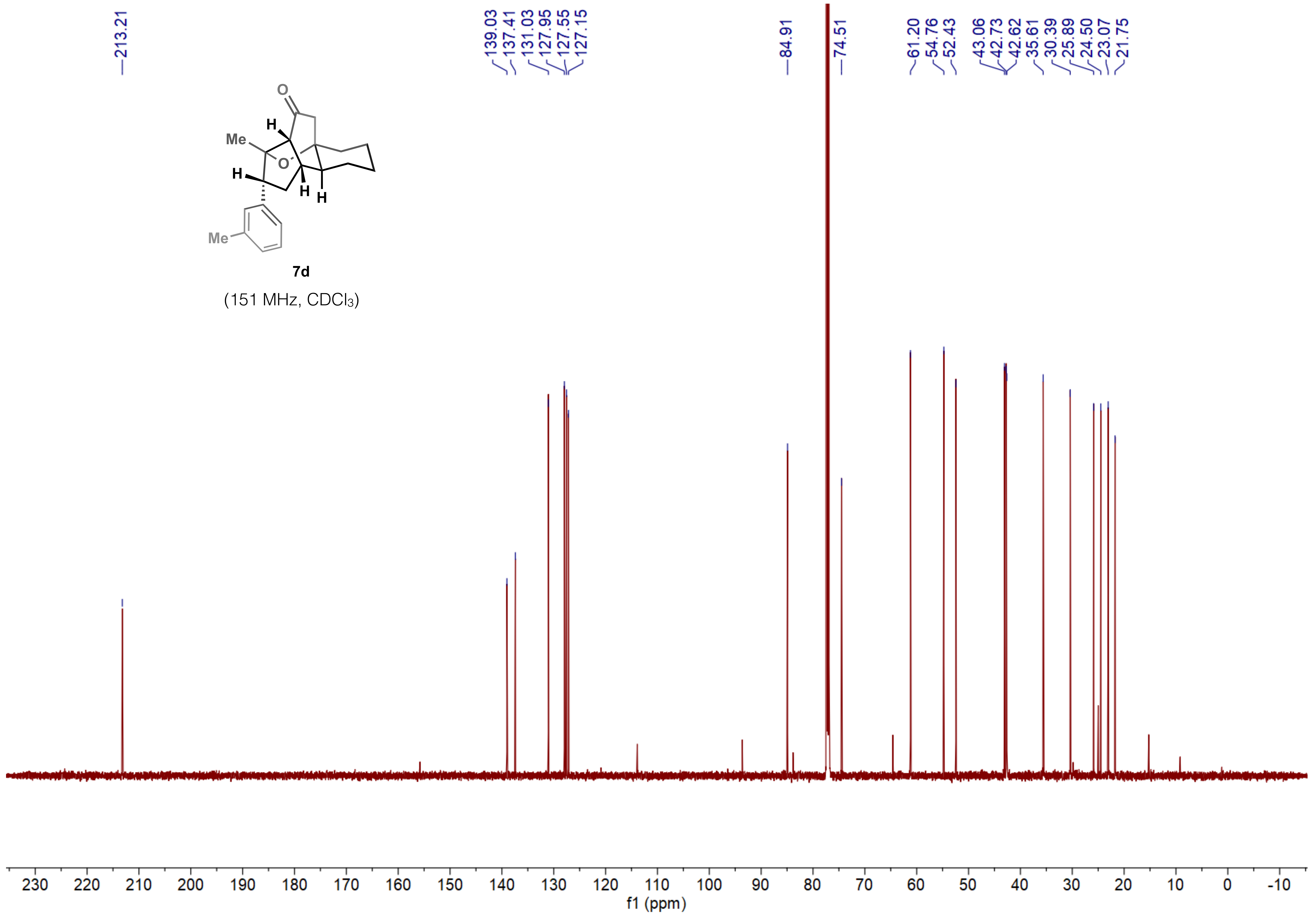
213.21
139.03
137.41
131.03
127.95
127.55
127.15
84.91
74.51
61.20
54.76
52.43
43.06
42.73
42.62
35.61
30.39
25.89
24.50
23.07
21.75
O
H
Me
O
H
H
H
Me
7d
(151 MHz, CDCl3)
230
220
210
200
190
180
170
160
150
140
130
120
110
100
90
80
70
60
50
40
30
20
10
0
-10
f1 (ppm)

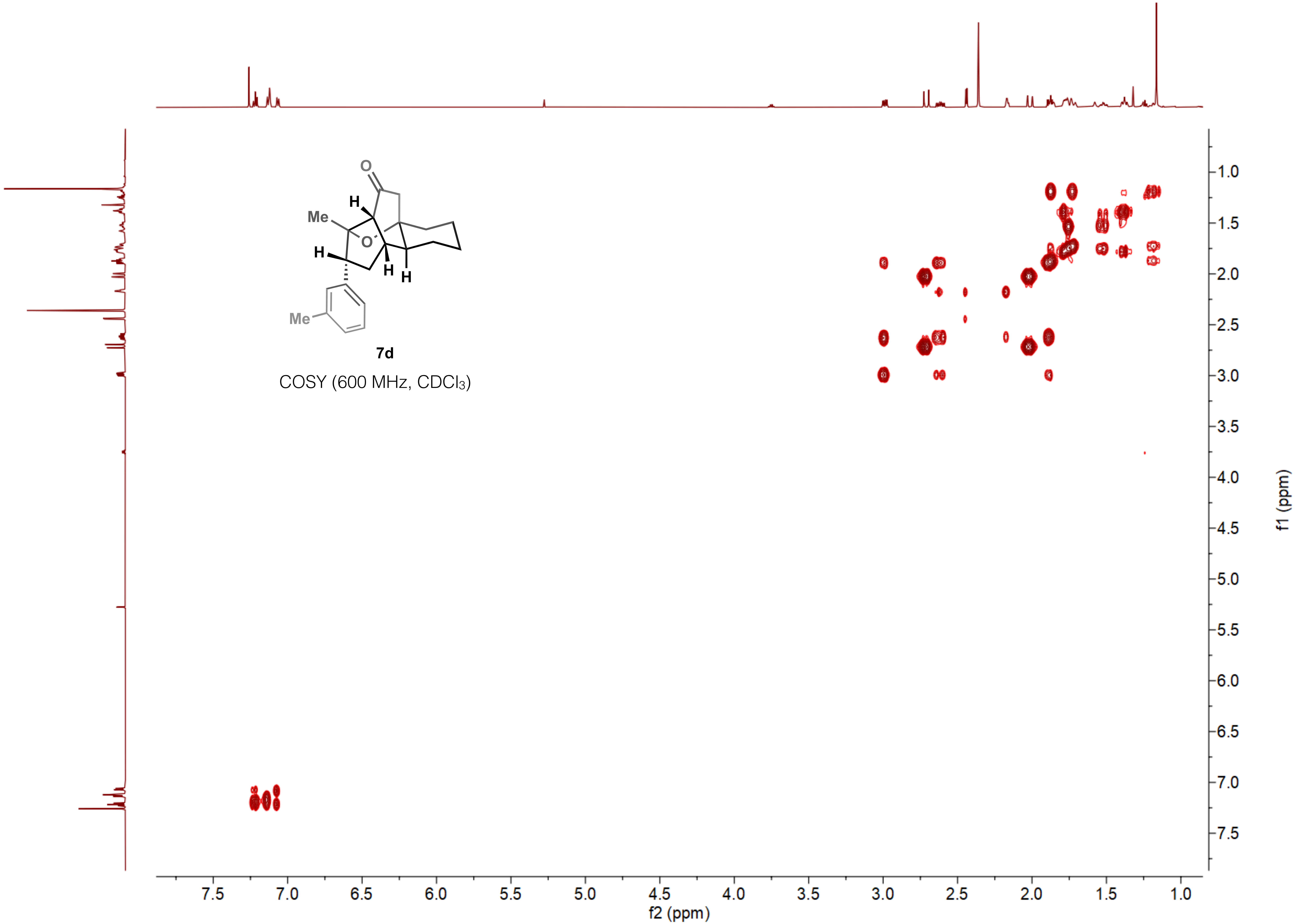

O
H
Me
O
H
H
H
Me
7d
COSY (600 MHz, CDCl3)
f2 (ppm)
f1 (ppm)
7.5
7.0
6.5
6.0
5.5
5.0
4.5
4.0
3.5
3.0
2.5
2.0
1.5
1.0

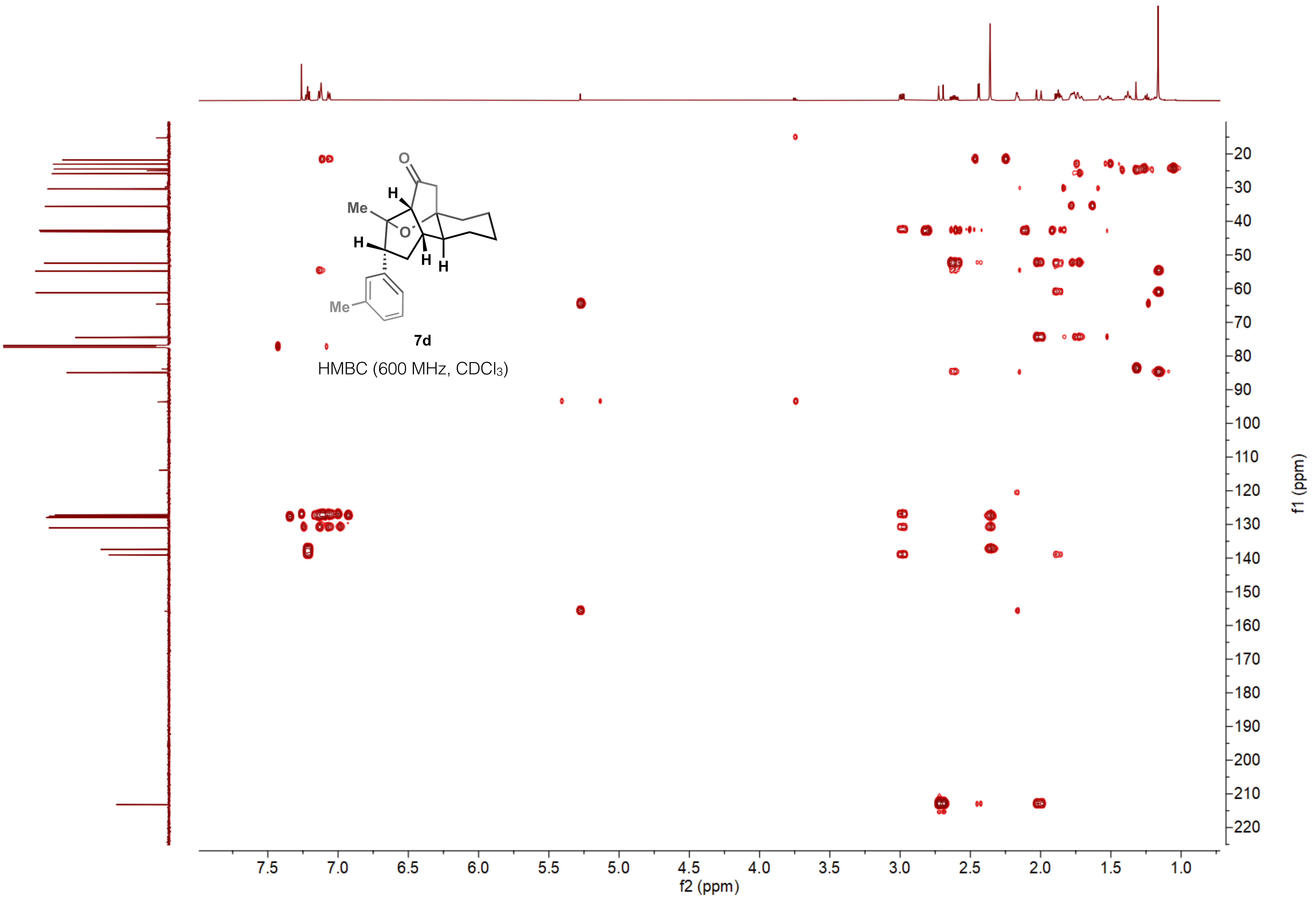

O
H
Me
O
H
H
H
Me
7d
HMBC (600 MHz, CDCl3)
f2 (ppm)
f1 (ppm)

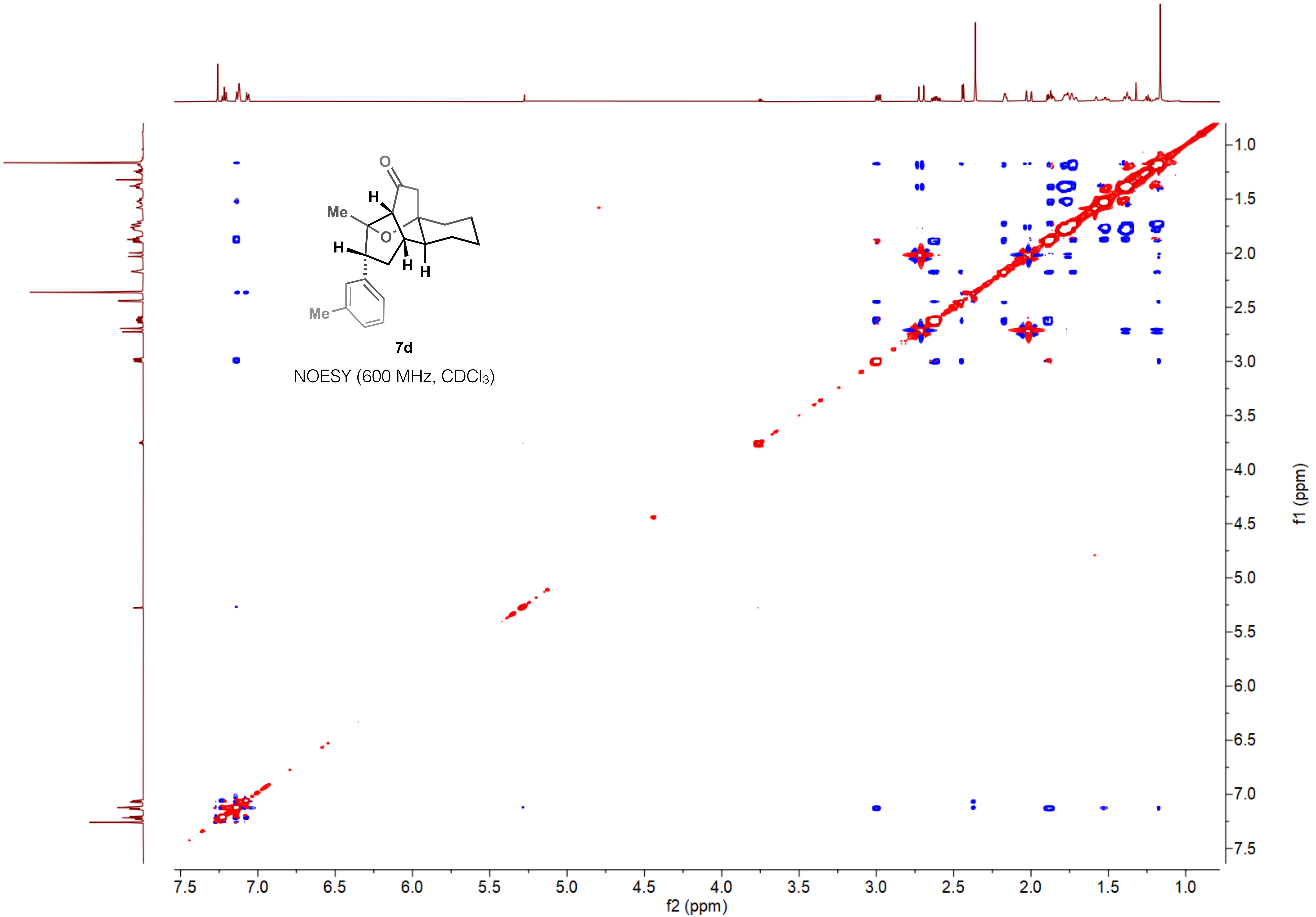

O
H
Me
O
H
H
H
Me
7d
NOESY (600 MHz, CDCl3)
f2 (ppm)
f1 (ppm)

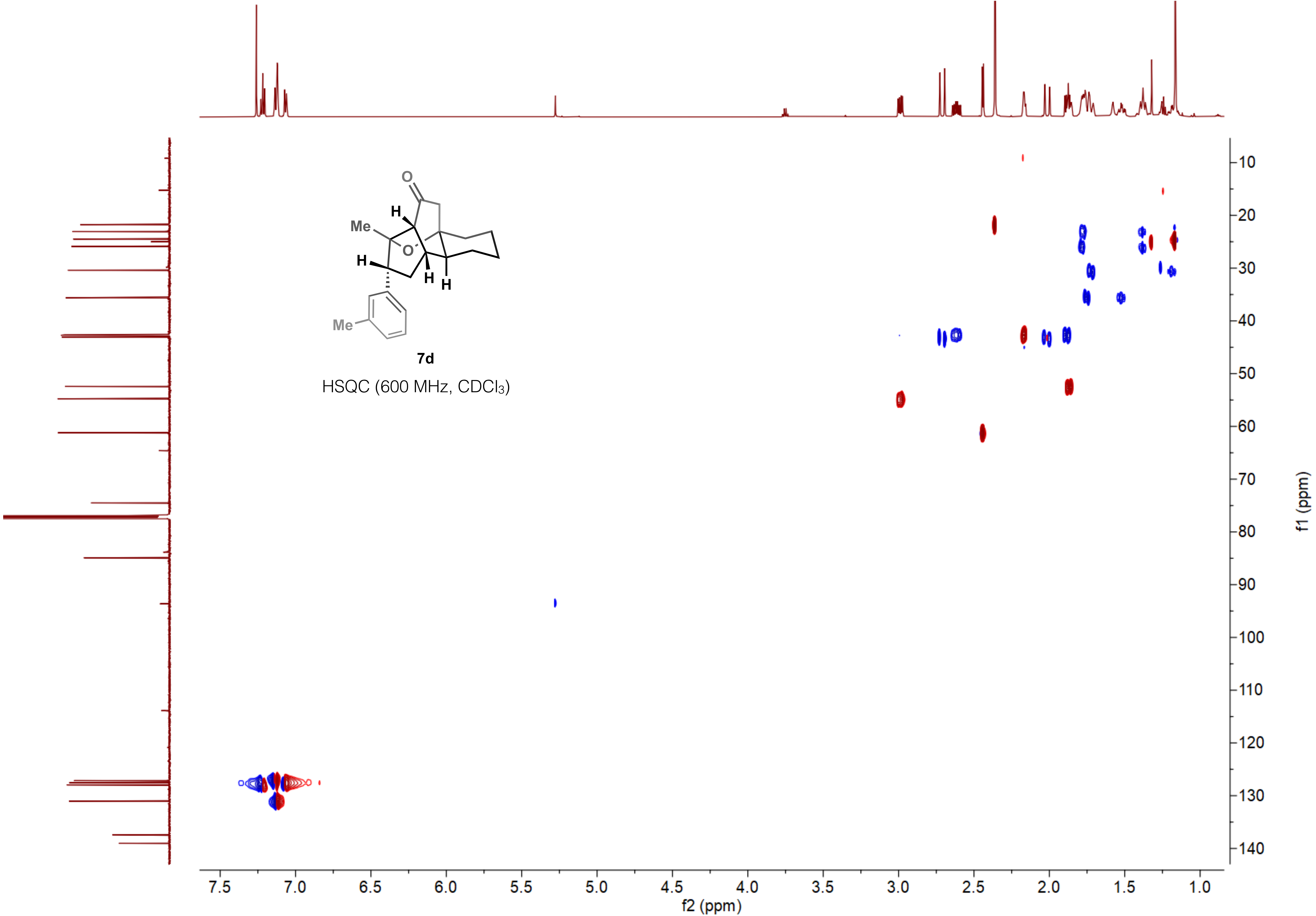

O
H
Me
O
H
H
H
Me
7d
HSQC (600 MHz, CDCl3)
f2 (ppm)
f1 (ppm)

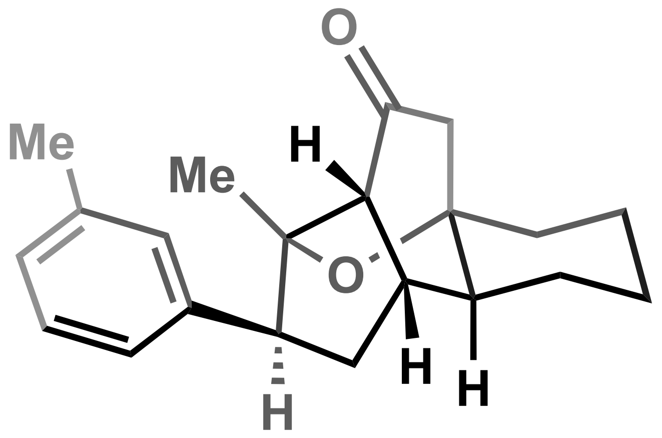

8d

(600 MHz, $CDCl_3$)

NB: 28% iodoether **4** as inseparable mixture

NB: not used to probe KOR agonism

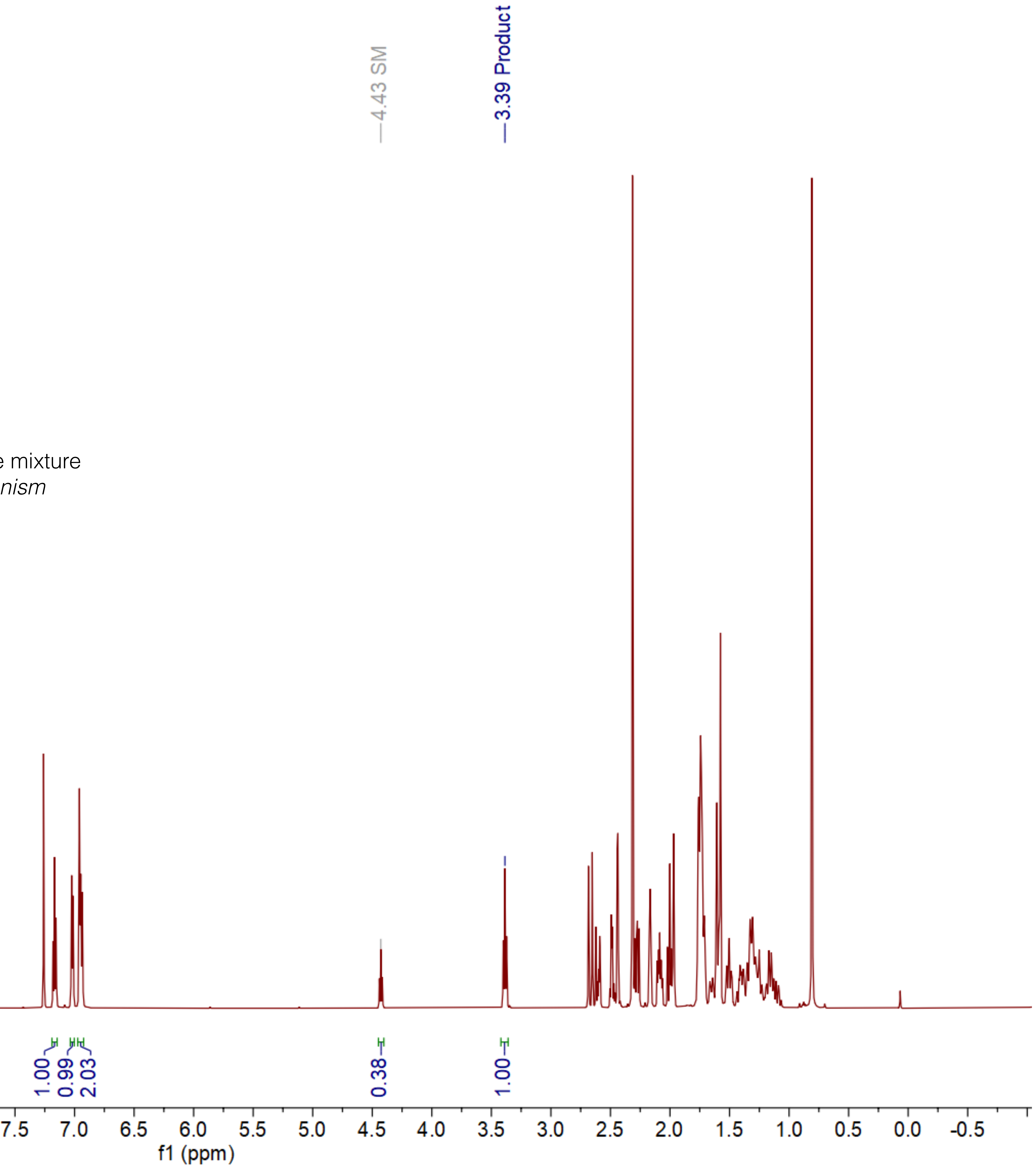

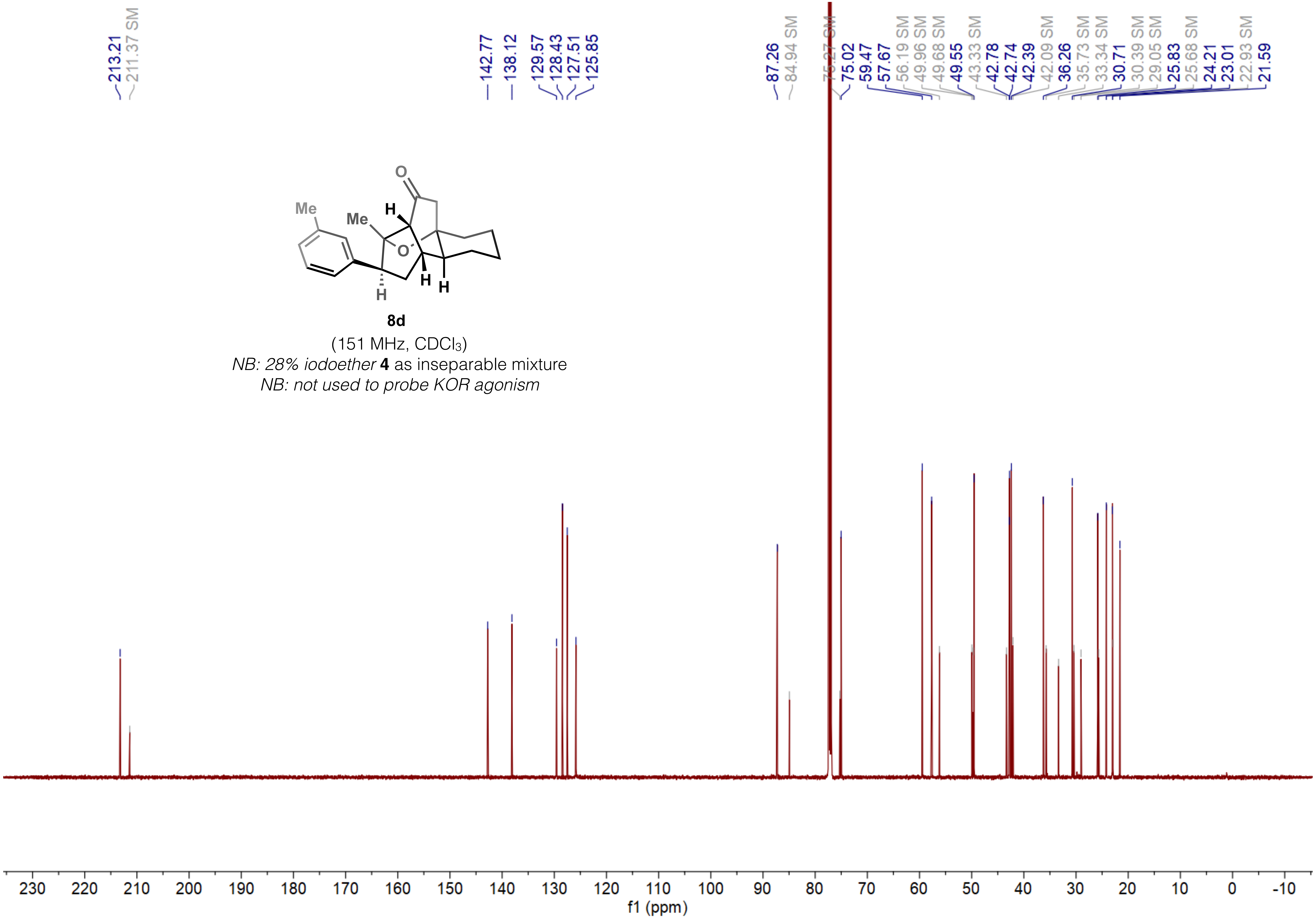

8d

(151 MHz, $CDCl_3$)

NB: 28% iodoether **4** as inseparable mixture

NB: not used to probe KOR agonism

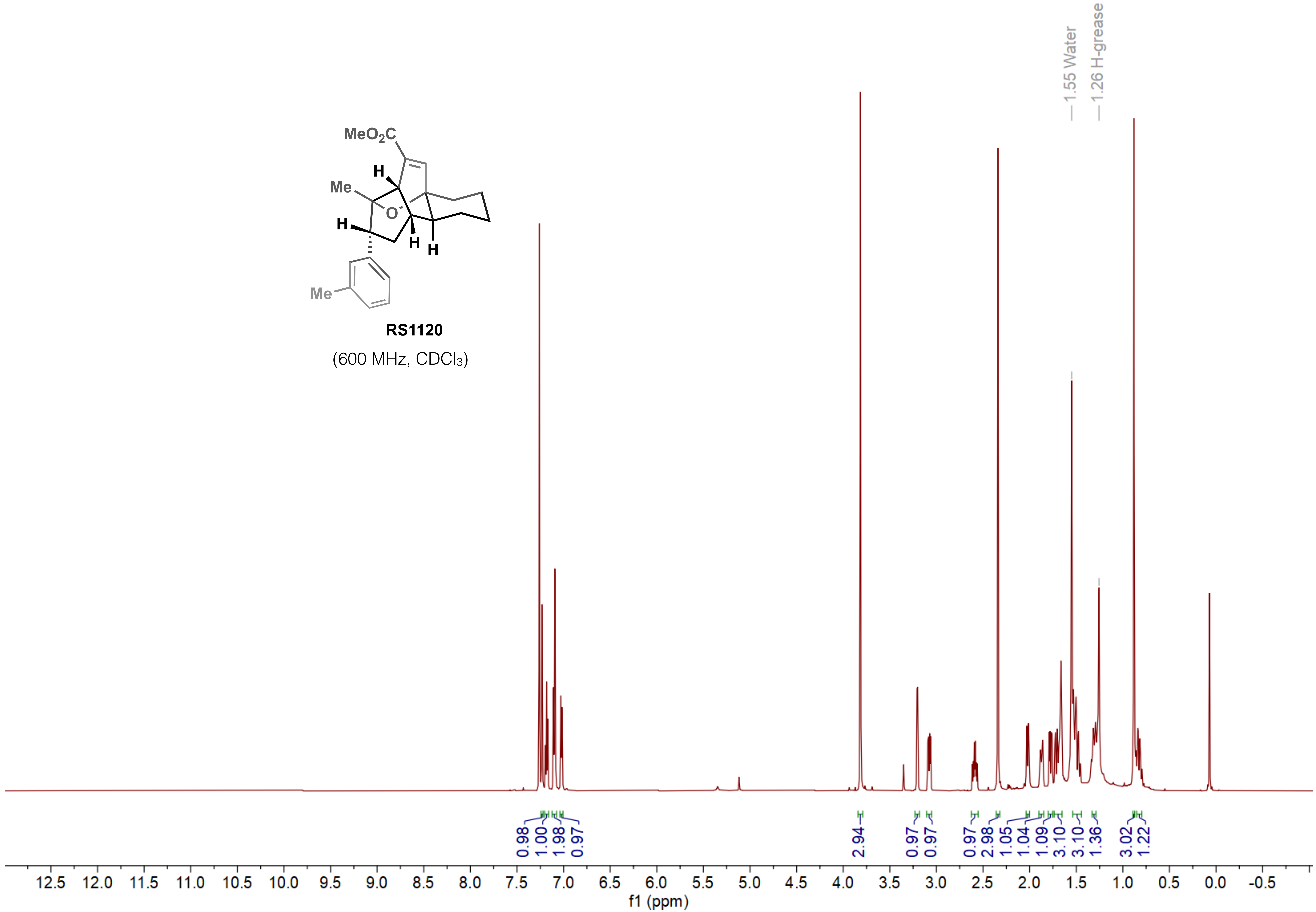
MeO2C
Me
H
O
H
H
H
Me
RS1120
(600 MHz, CDCl3)
1.55 Water
1.26 H-grease
0.98
1.00
1.98
0.97
2.94
0.97
0.97
0.97
2.98
1.05
1.04
1.09
3.10
3.10
1.36
3.02
1.22
12.5 12.0 11.5 11.0 10.5 10.0 9.5 9.0 8.5 8.0 7.5 7.0 6.5 6.0 5.5 5.0 4.5 4.0 3.5 3.0 2.5 2.0 1.5 1.0 0.5 0.0 -0.5
f1 (ppm)

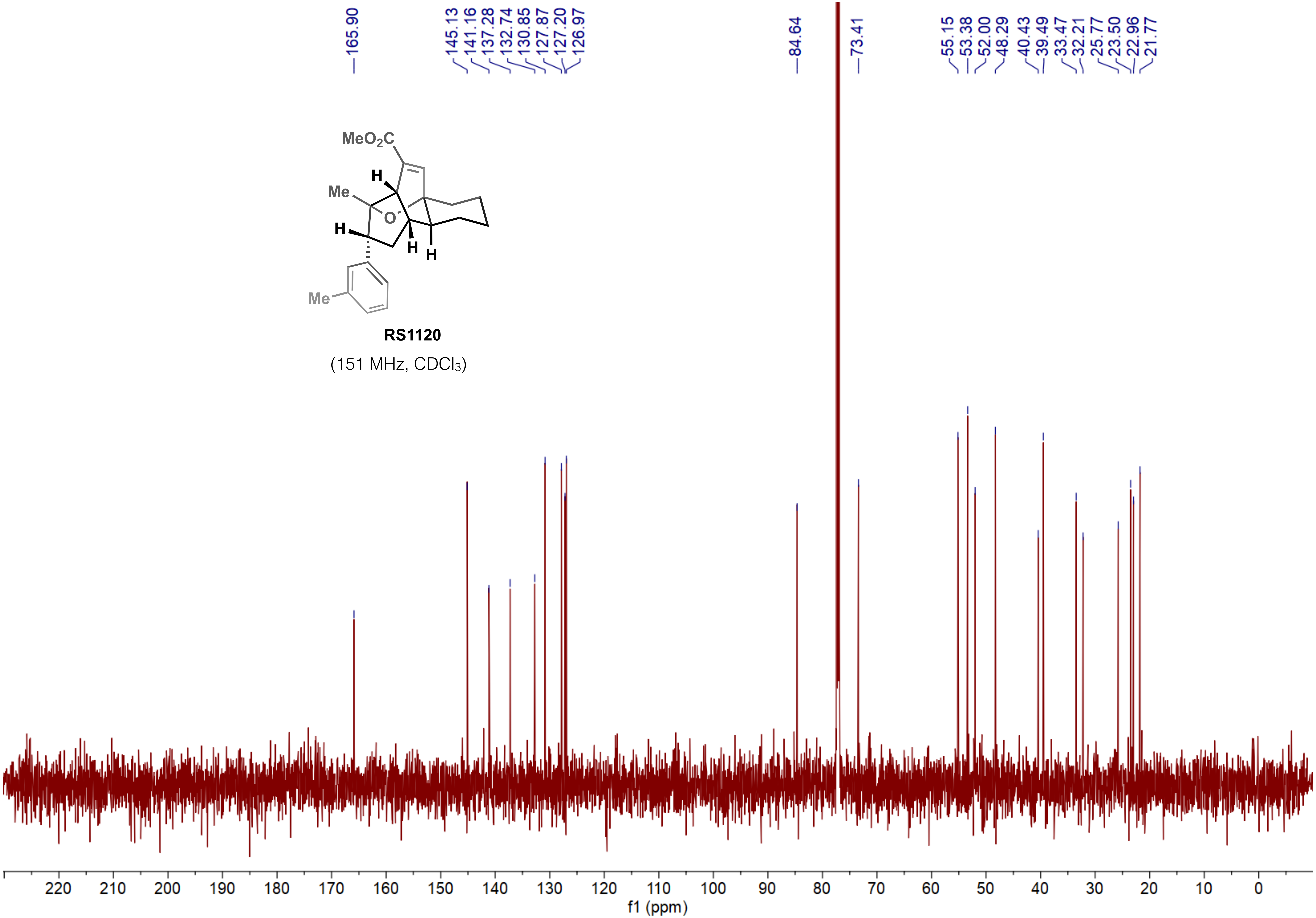

RS1120

(151 MHz, $CDCl_3$)

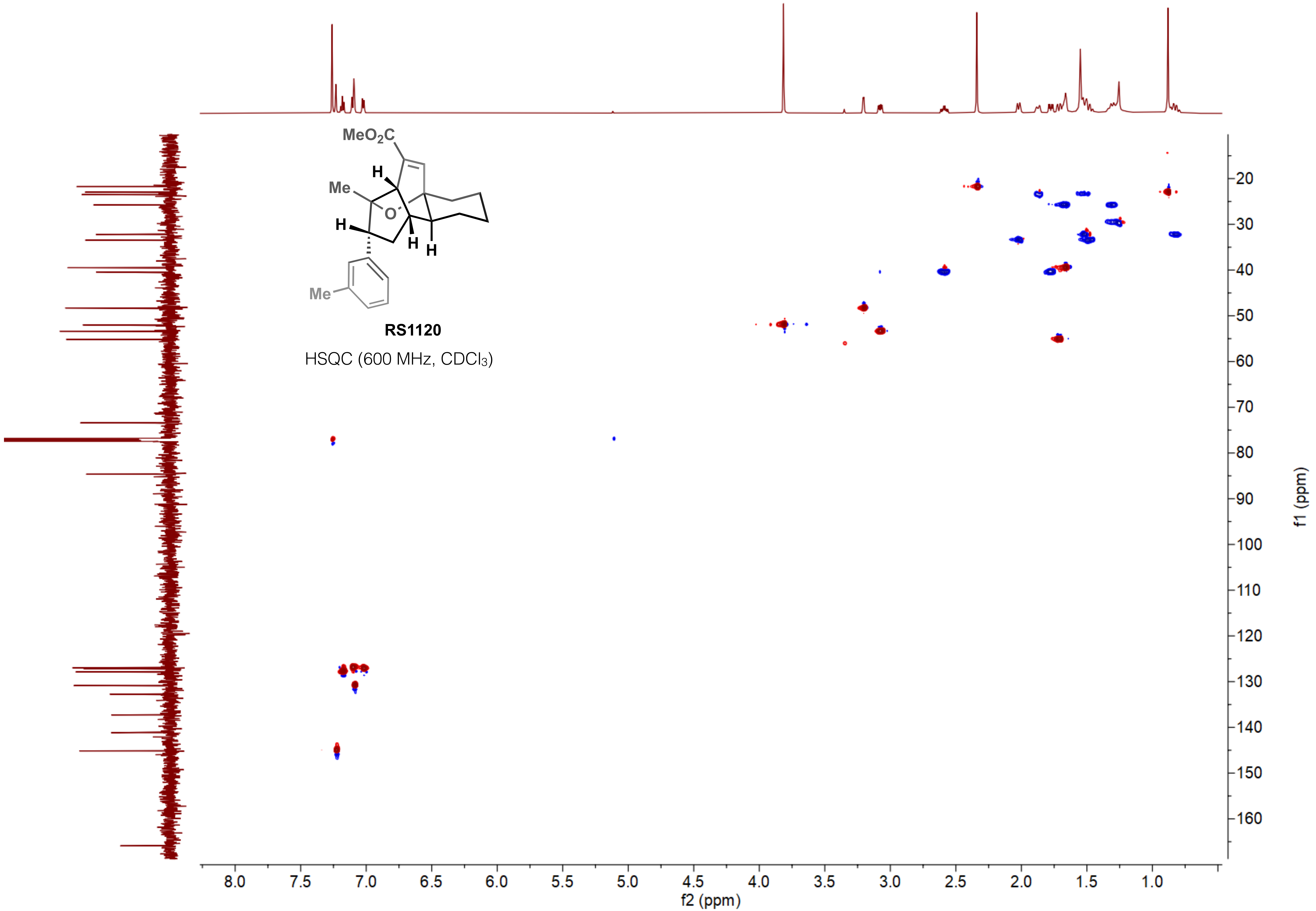
MeO2C
Me
H
O
H
H
H
Me
RS1120
HSQC (600 MHz, CDCl3)
f1 (ppm)
f2 (ppm)
8.0
7.5
7.0
6.5
6.0
5.5
5.0
4.5
4.0
3.5
3.0
2.5
2.0
1.5
1.0
20
30
40
50
60
70
80
90
100
110
120
130
140
150
160

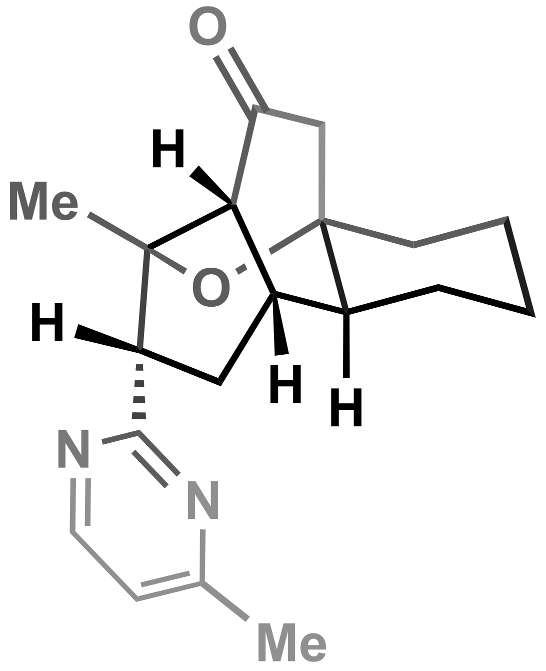

7e

(600 MHz, $CDCl_3$)

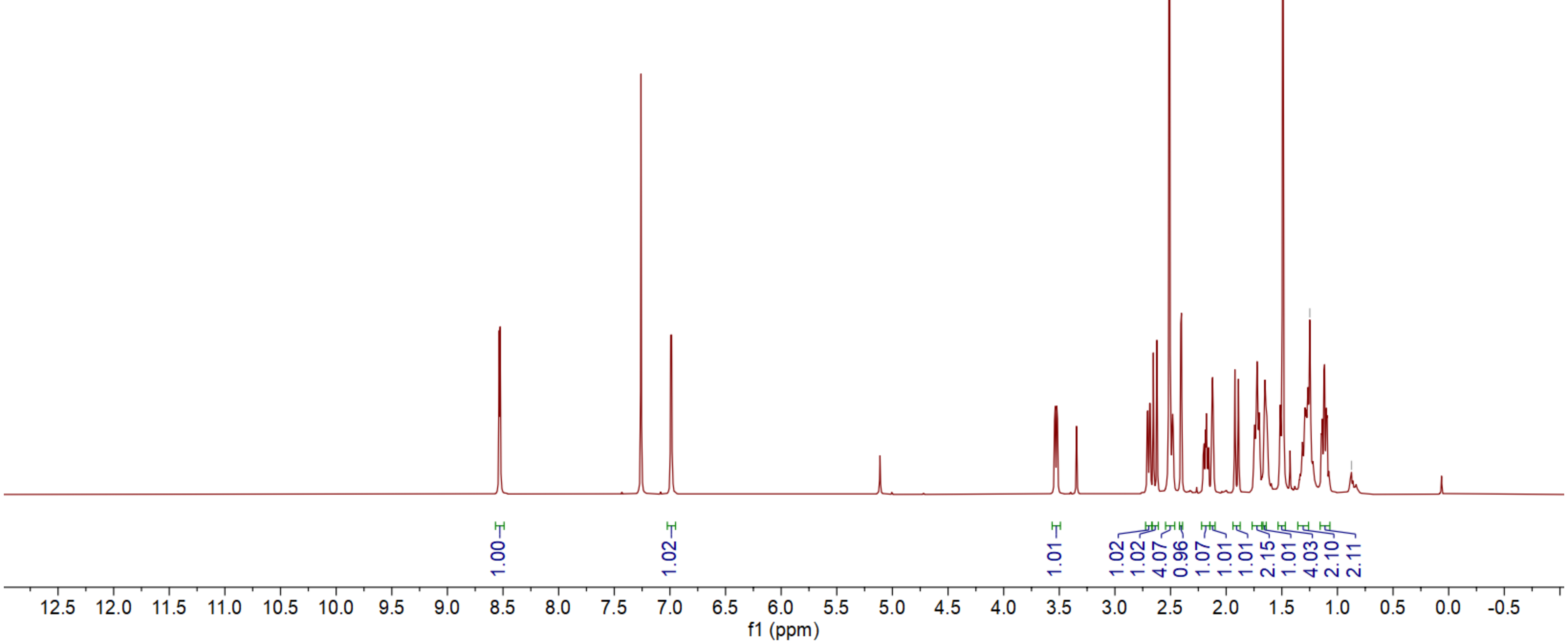

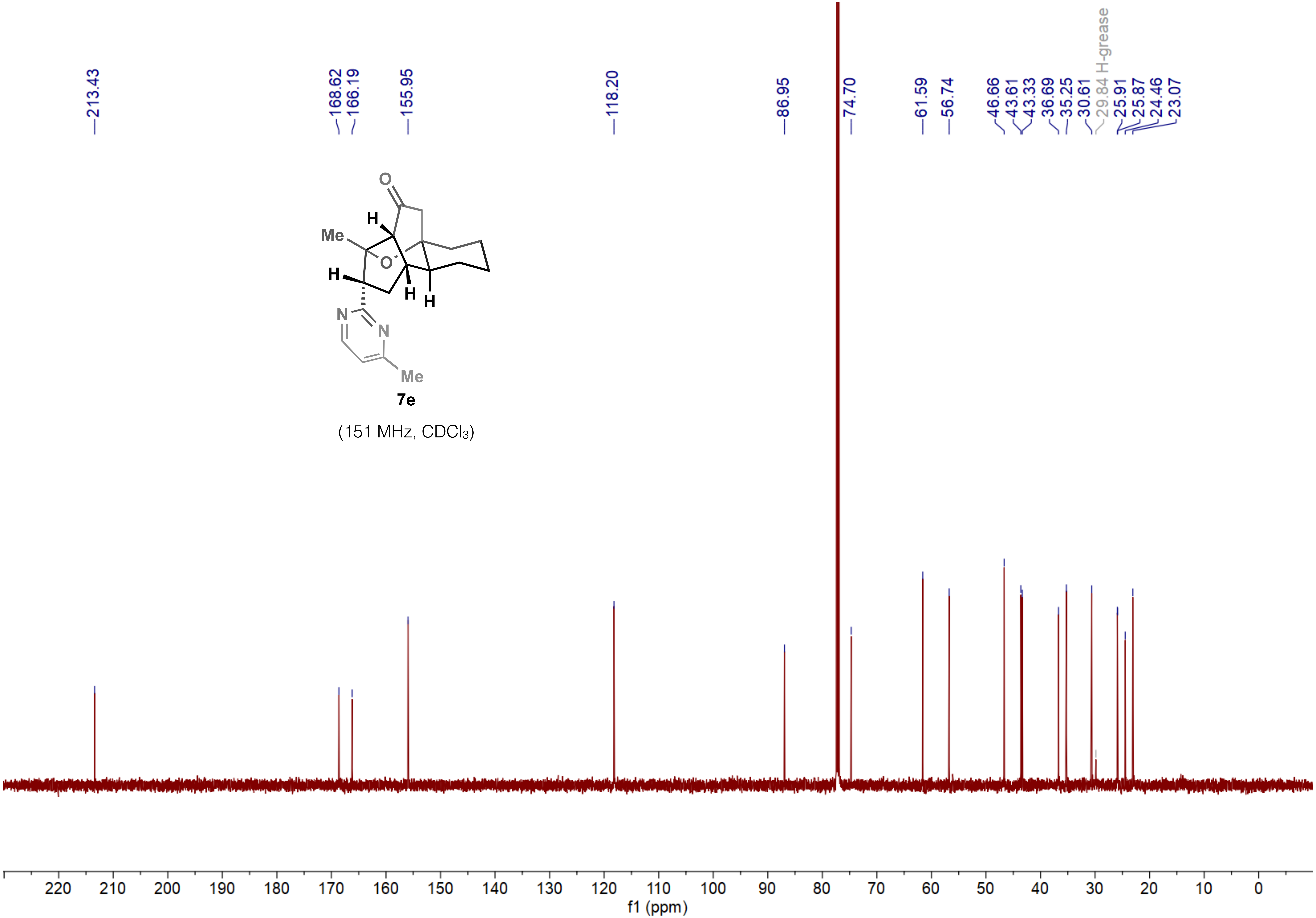
213.43
168.62
166.19
155.95
118.20
86.95
74.70
61.59
56.74
46.66
43.61
43.33
36.69
35.25
30.61
29.84 H-grease
25.91
25.87
24.46
23.07
O
H
Me
O
H
H
H
N
N
Me
7e
(151 MHz, CDCl3)
220 210 200 190 180 170 160 150 140 130 120 110 100 90 80 70 60 50 40 30 20 10 0
f1 (ppm)

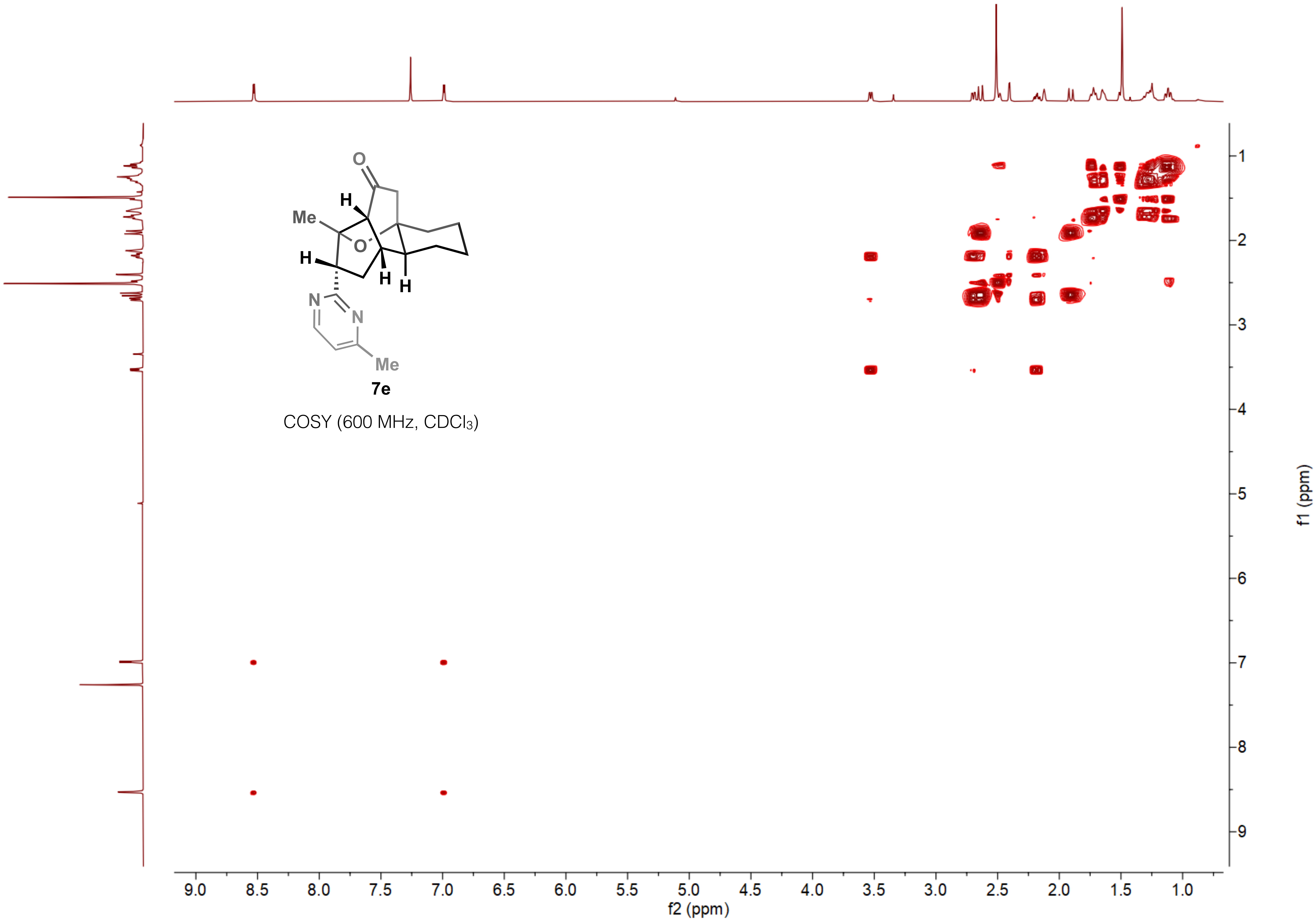

O
H
Me
O
H
H
H
N
N
Me
7e
COSY (600 MHz, CDCl3)
9.0 8.5 8.0 7.5 7.0 6.5 6.0 5.5 5.0 4.5 4.0 3.5 3.0 2.5 2.0 1.5 1.0
f2 (ppm)
1 2 3 4 5 6 7 8 9
f1 (ppm)

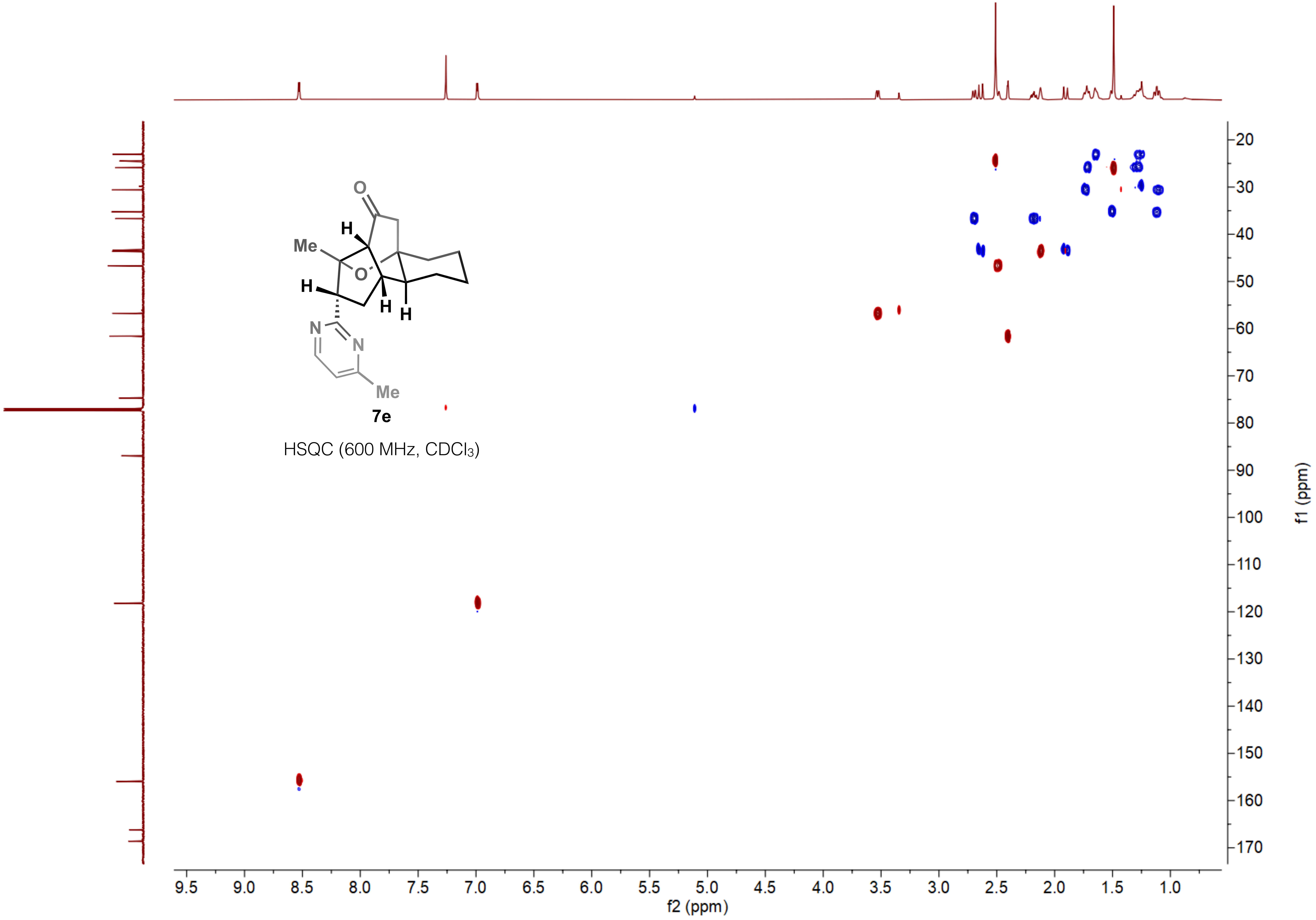

7e

HSQC (600 MHz, $CDCl_3$)

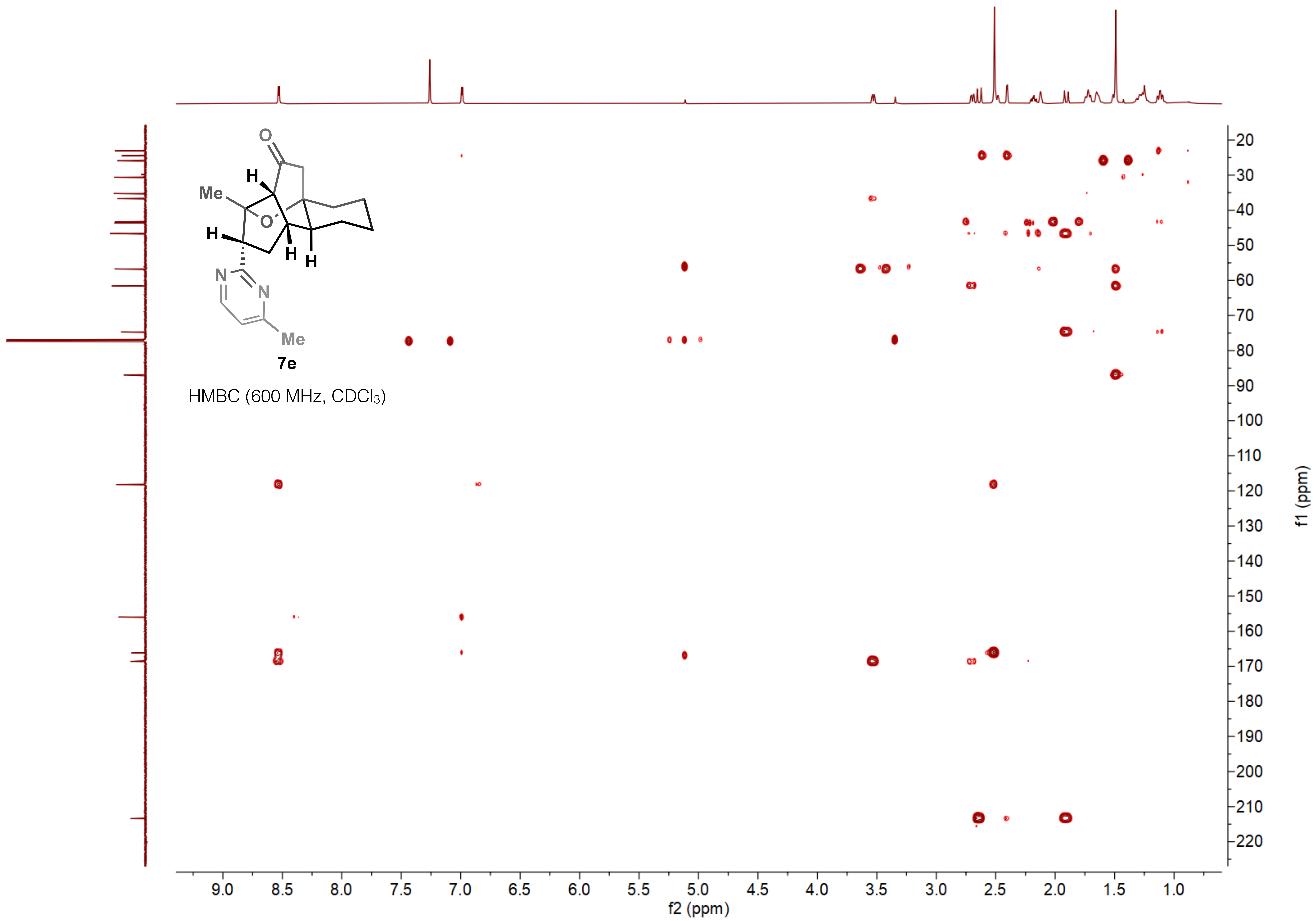

O
H
Me
O
H
H
H
N
N
Me
7e
HMBC (600 MHz, CDCl3)
9.0
8.5
8.0
7.5
7.0
6.5
6.0
5.5
5.0
4.5
4.0
3.5
3.0
2.5
2.0
1.5
1.0
f2 (ppm)
f1 (ppm)
20
30
40
50
60
70
80
90
100
110
120
130
140
150
160
170
180
190
200
210
220

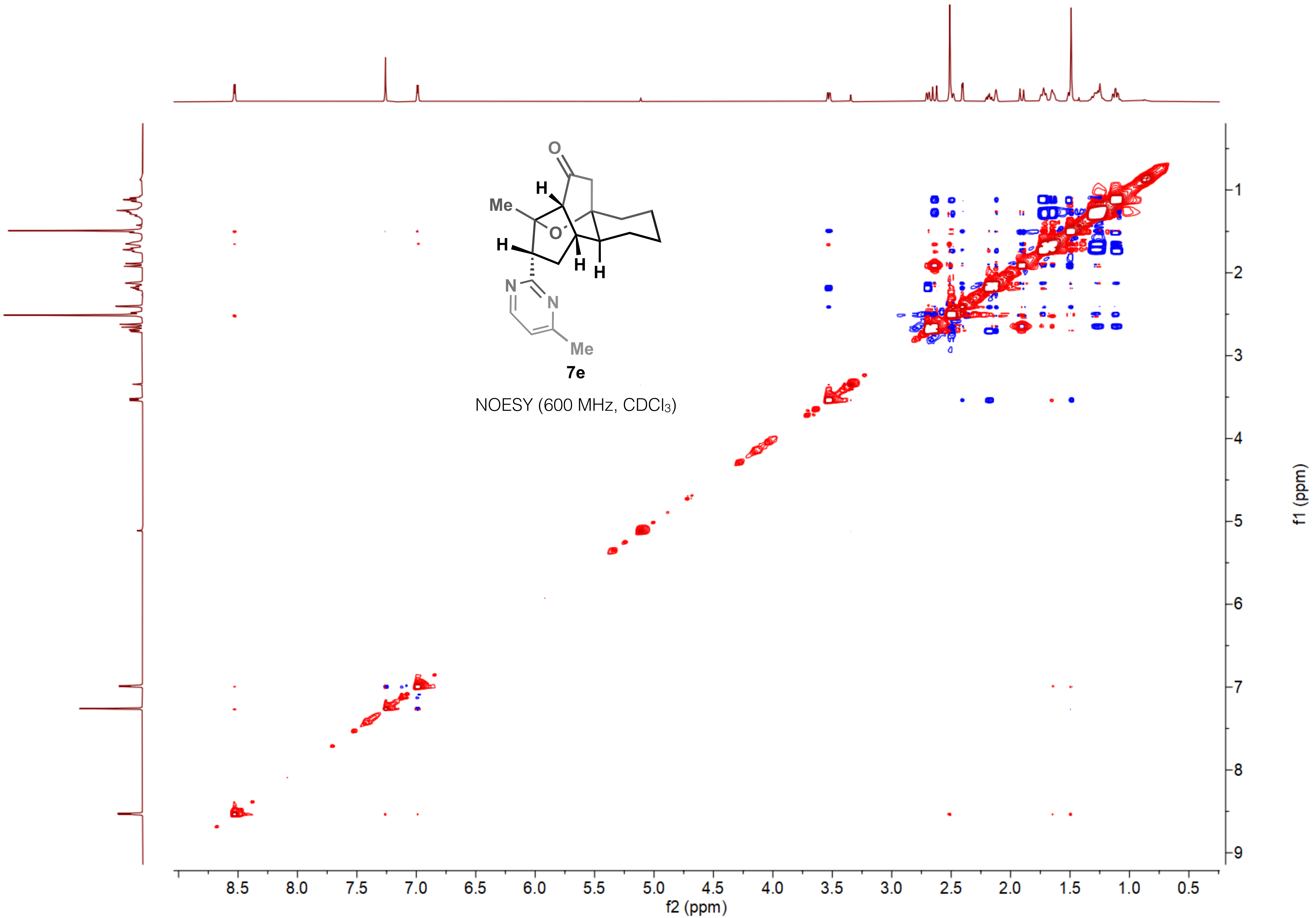

Me
H
O
H
H
H
N
N
Me
7e
NOESY (600 MHz, CDCl3)
f2 (ppm)
f1 (ppm)
8.5
8.0
7.5
7.0
6.5
6.0
5.5
5.0
4.5
4.0
3.5
3.0
2.5
2.0
1.5
1.0
0.5
1
2
3
4
5
6
7
8
9

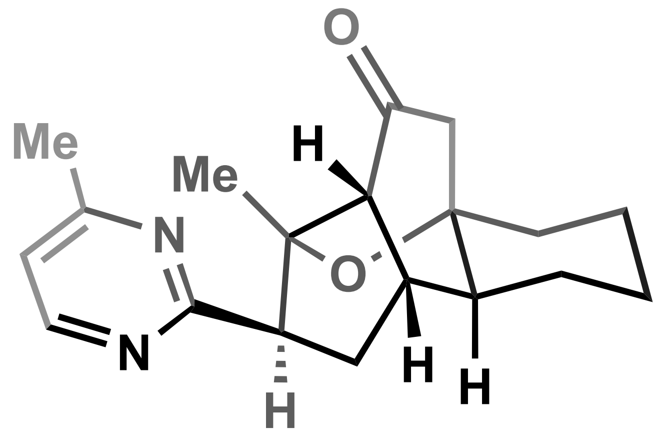

8e

(600 MHz, $CDCl_3$)

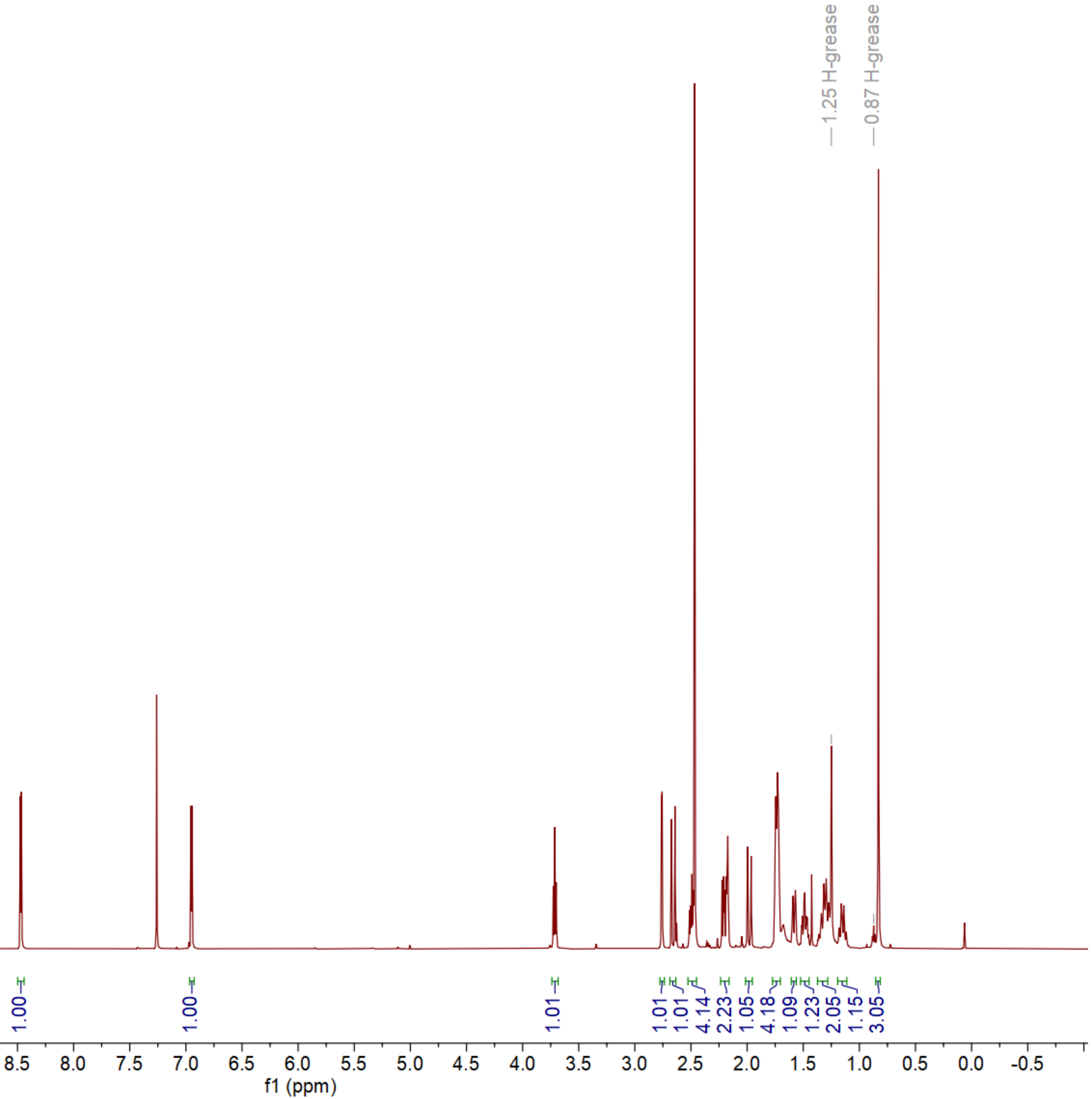

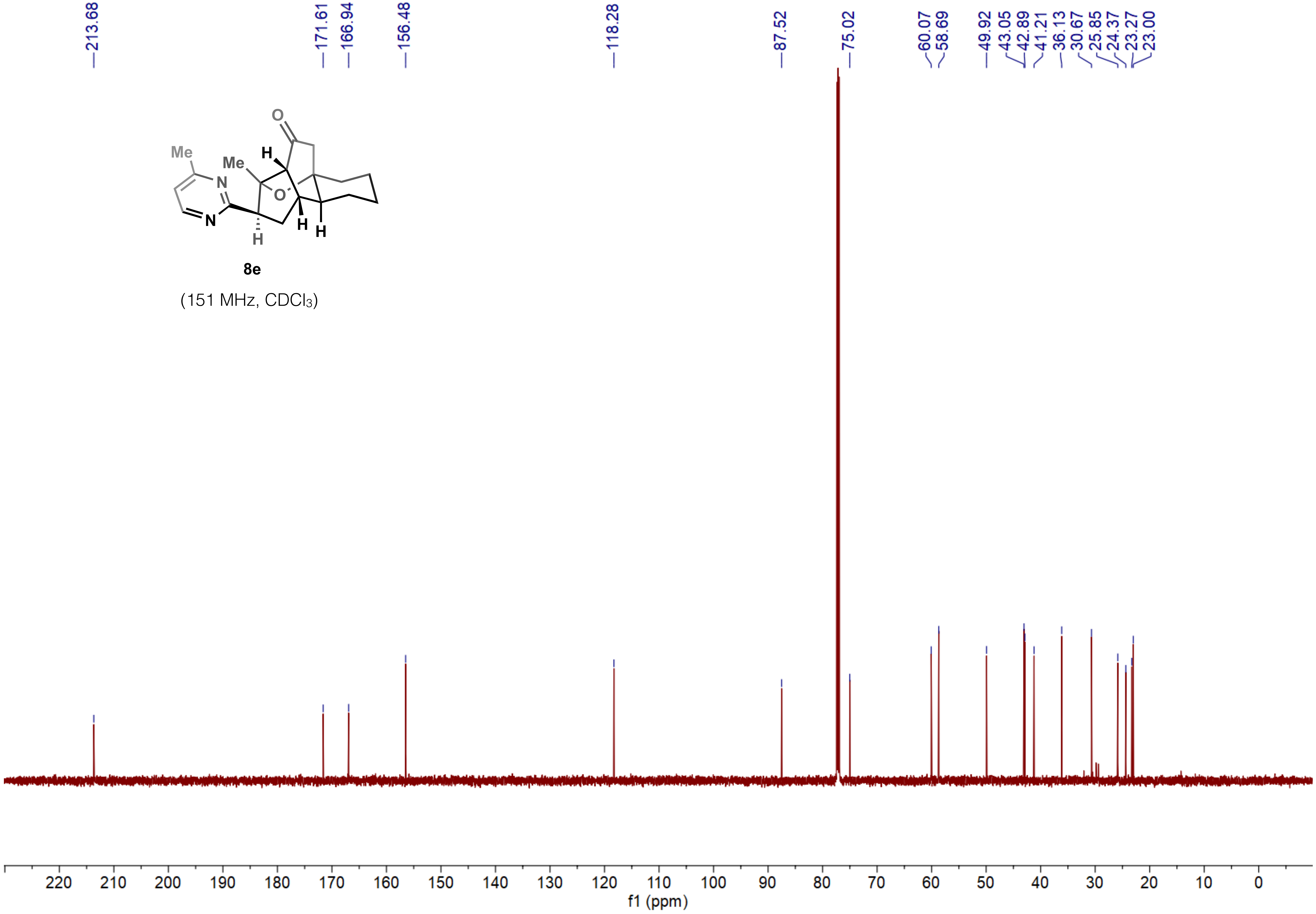

8e

(151 MHz, $CDCl_3$)

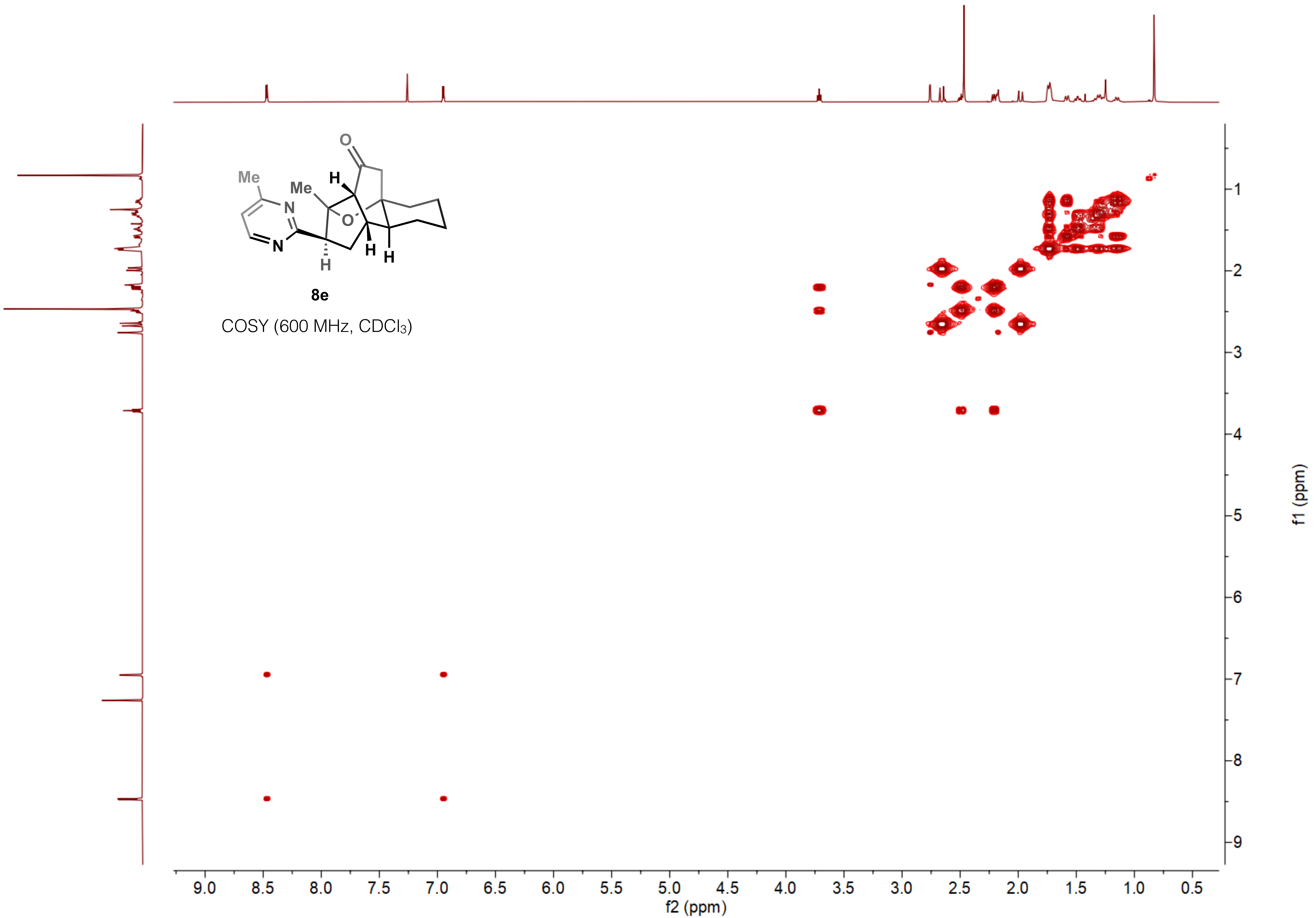

Me
Me
N
N
O
O
H
H
H
H
8e
COSY (600 MHz, CDCl3)
f1 (ppm)
f2 (ppm)

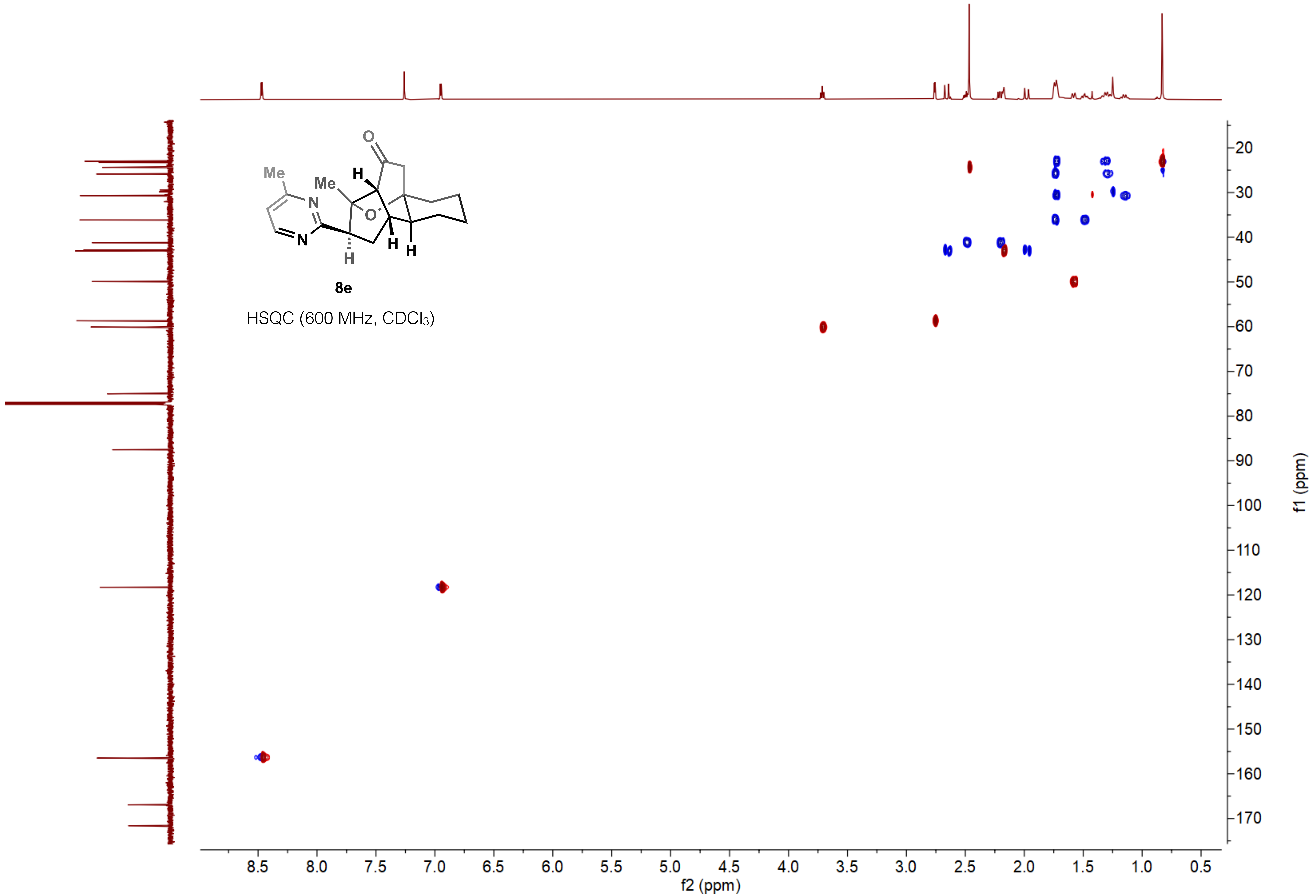

Me
Me
N
N
O
O
H
H
H
H
8e
HSQC (600 MHz, CDCl3)
f2 (ppm)
f1 (ppm)
8.5 8.0 7.5 7.0 6.5 6.0 5.5 5.0 4.5 4.0 3.5 3.0 2.5 2.0 1.5 1.0 0.5
20 30 40 50 60 70 80 90 100 110 120 130 140 150 160 170

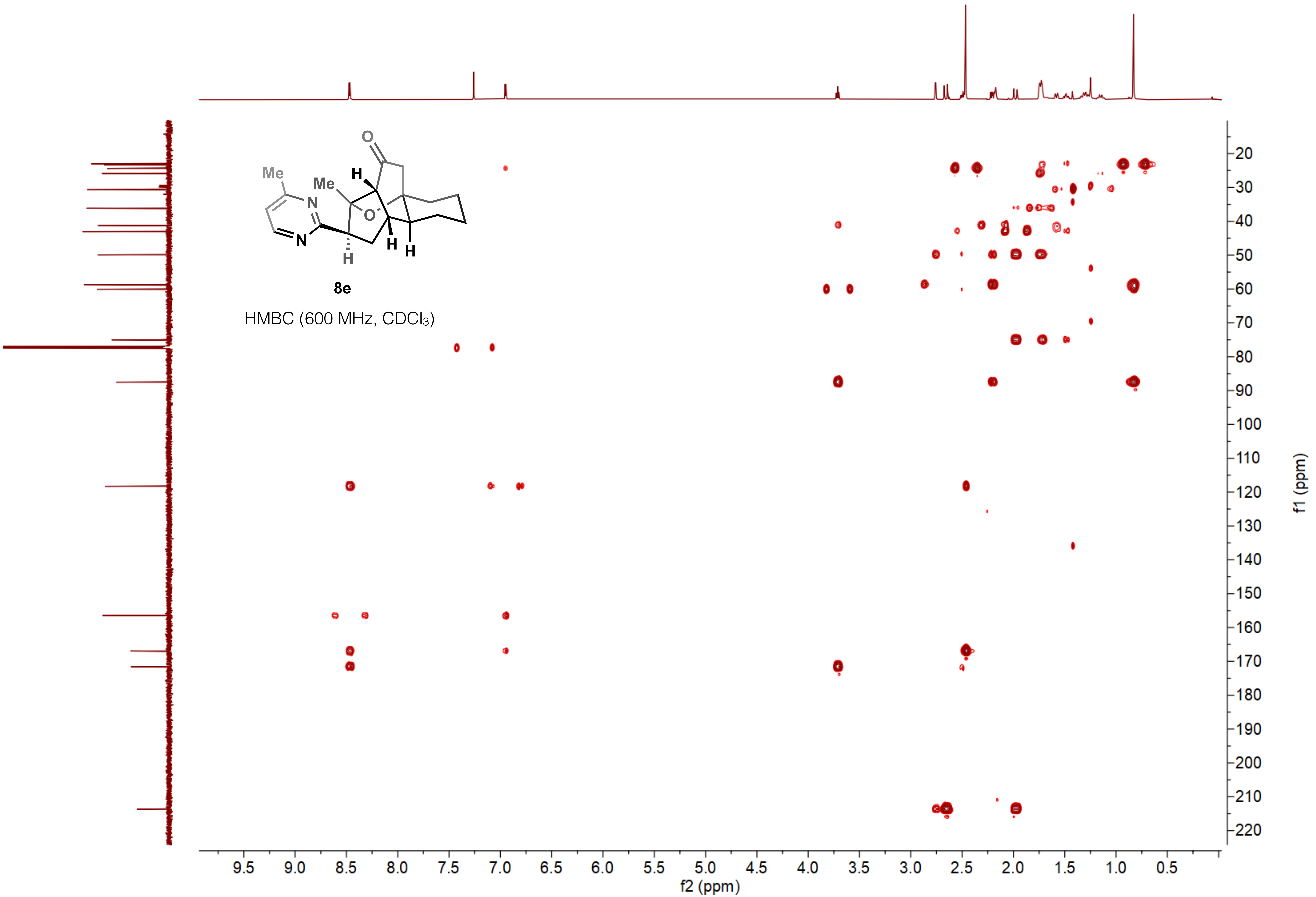
Me
Me
N
N
O
O
H
H
H
H
8e
HMBC (600 MHz, CDCl3)
f2 (ppm)
f1 (ppm)

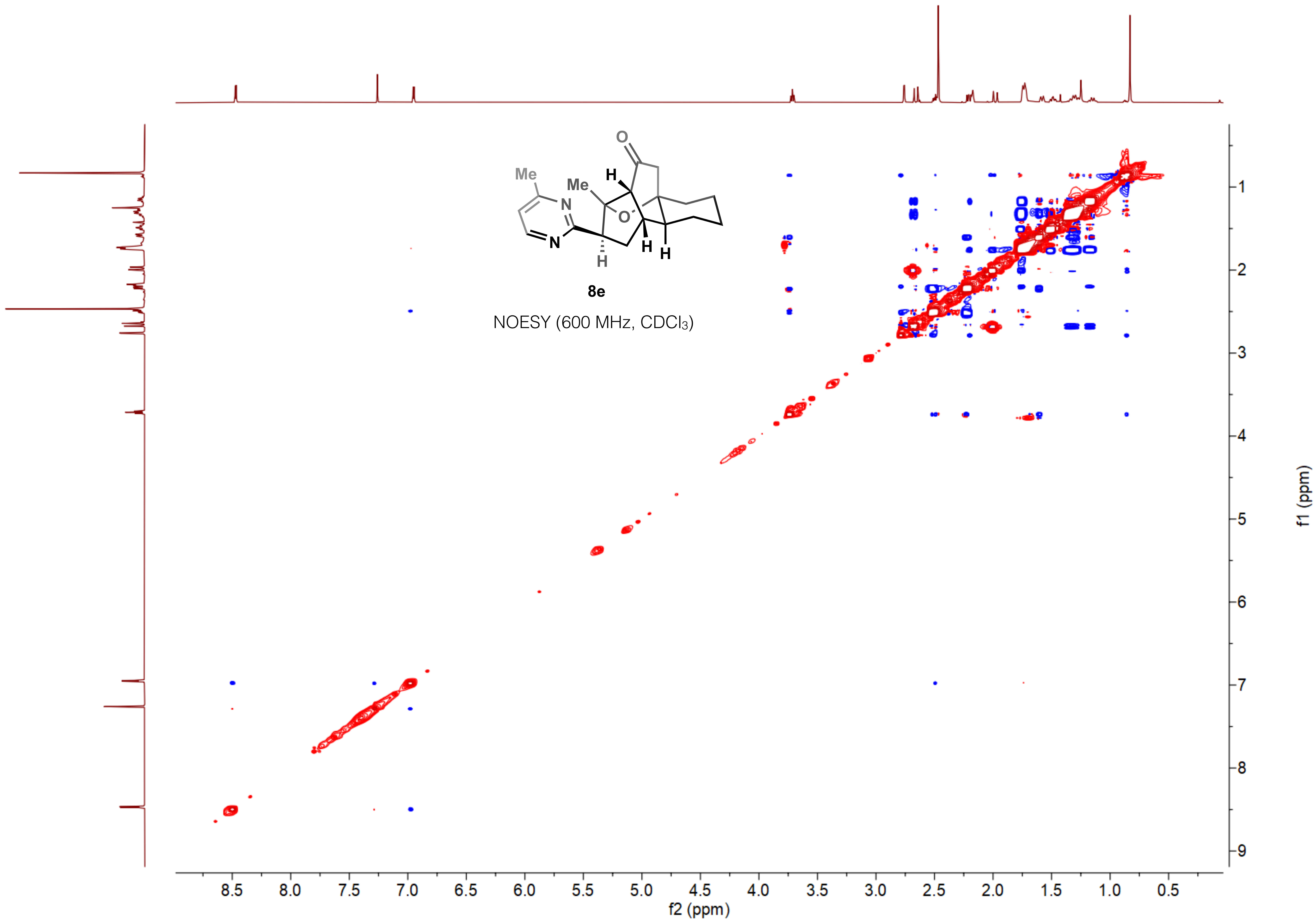

Me
Me
N
N
O
O
H
H
H
H
8e
NOESY (600 MHz, CDCl3)
f2 (ppm)
f1 (ppm)

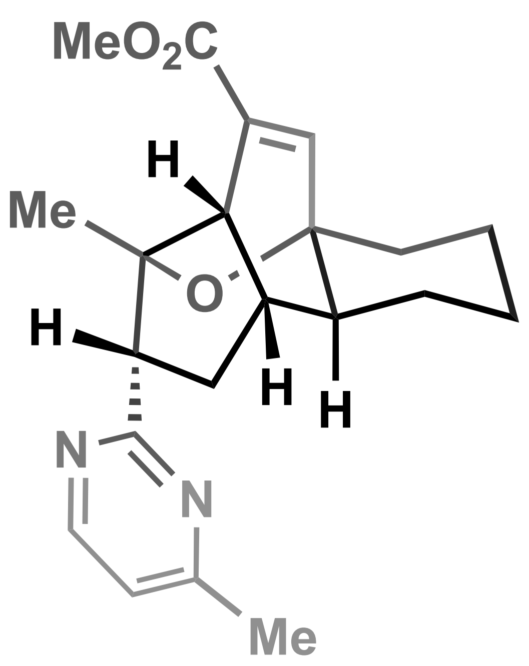

RS1136

(600 MHz, $CDCl_3$)

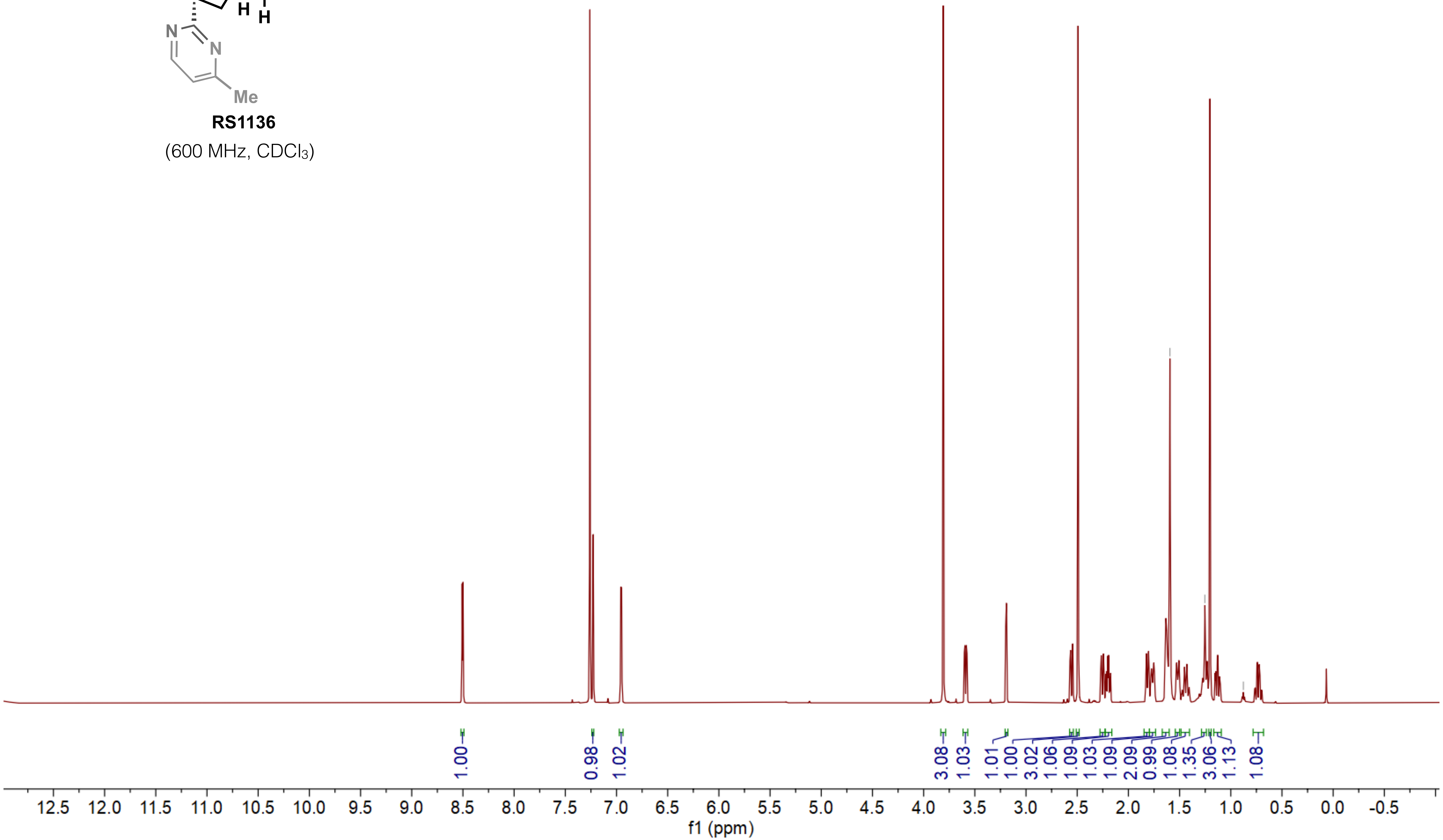

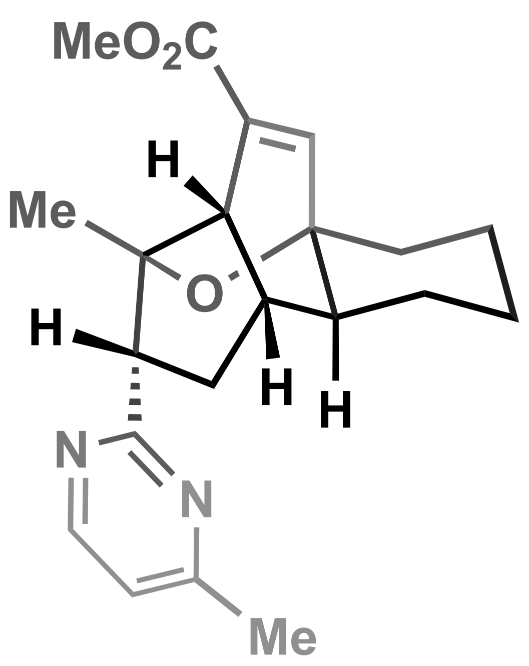

RS1136

(151 MHz, $CDCl_3$)

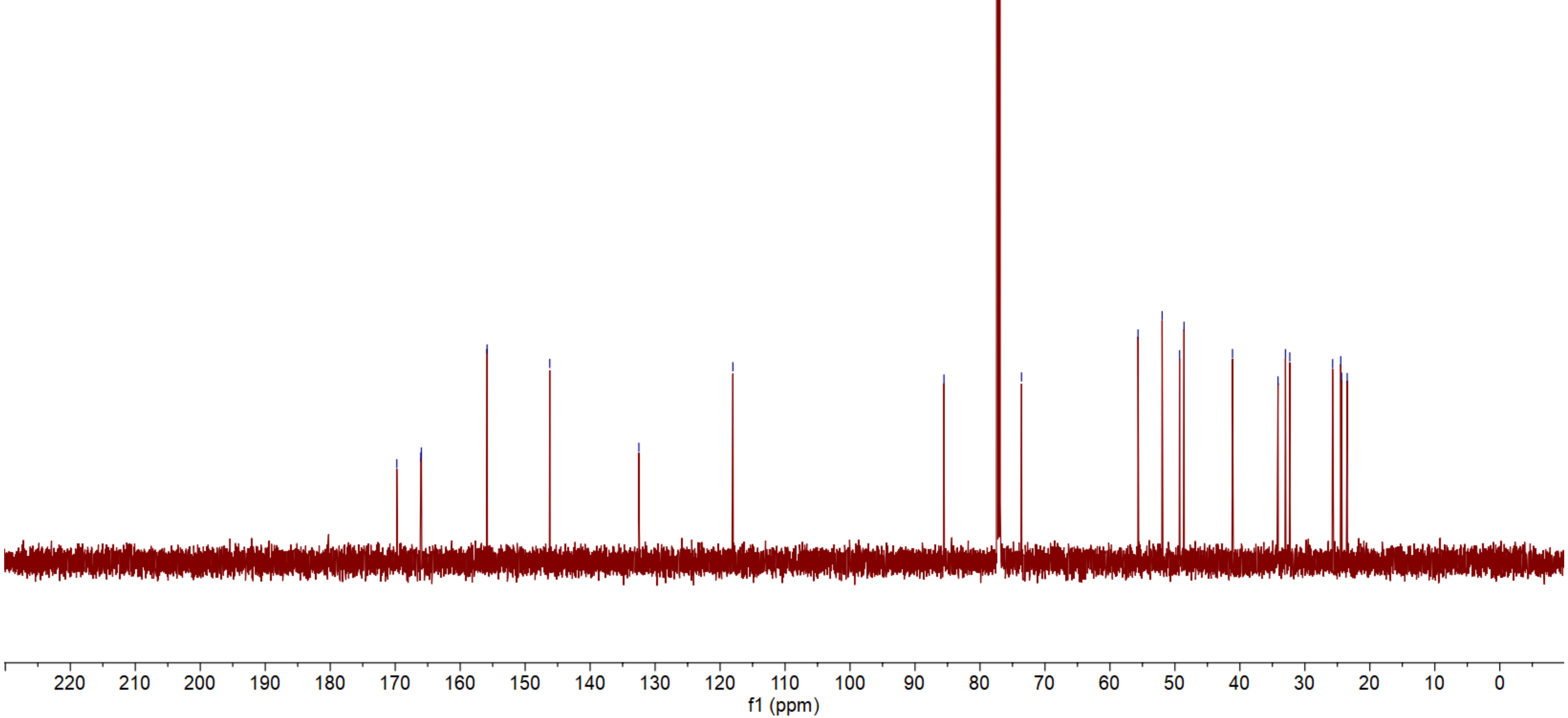

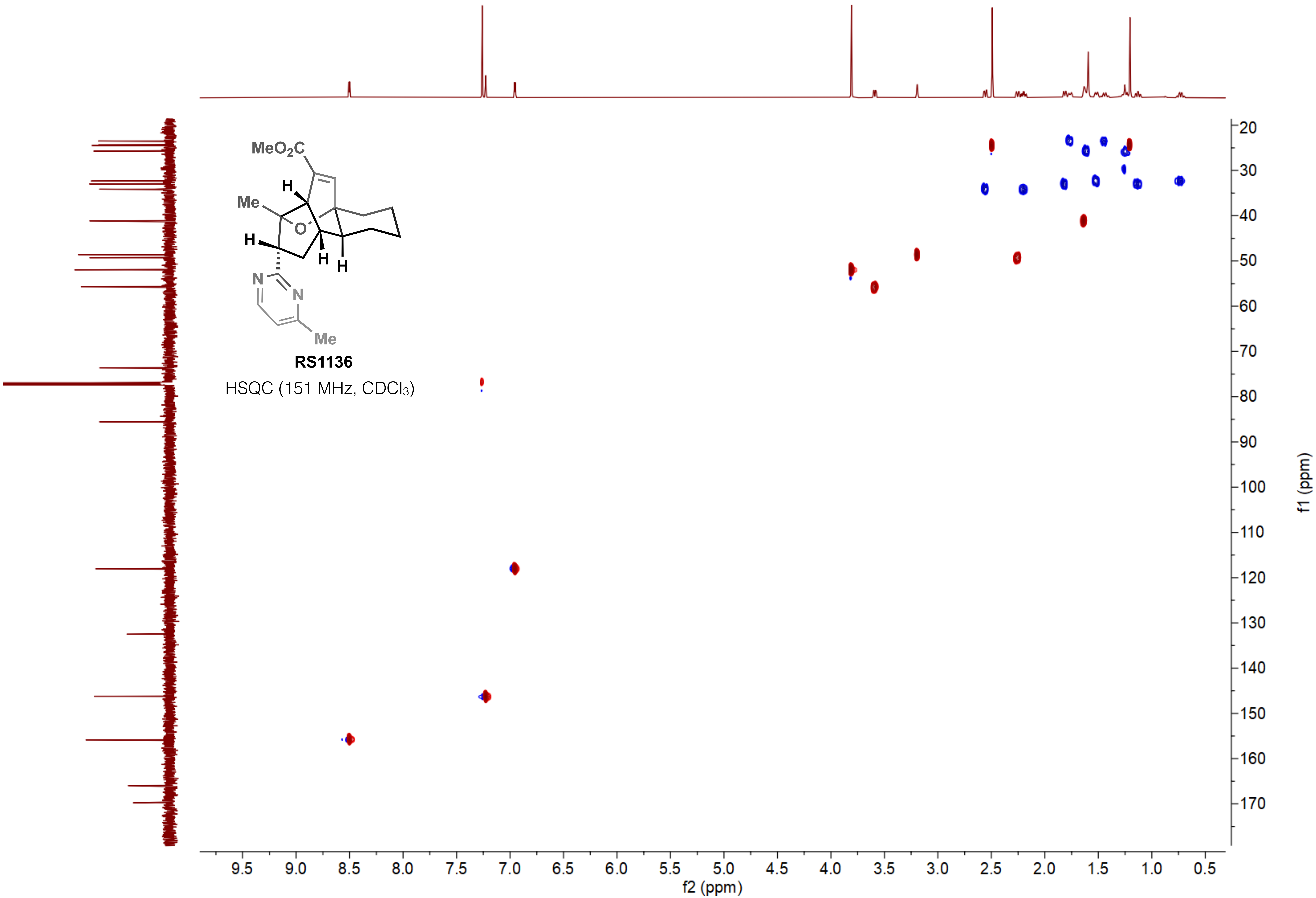
MeO2C
Me
H
O
H
H
H
N
N
Me
RS1136
HSQC (151 MHz, CDCl3)
f2 (ppm)
f1 (ppm)

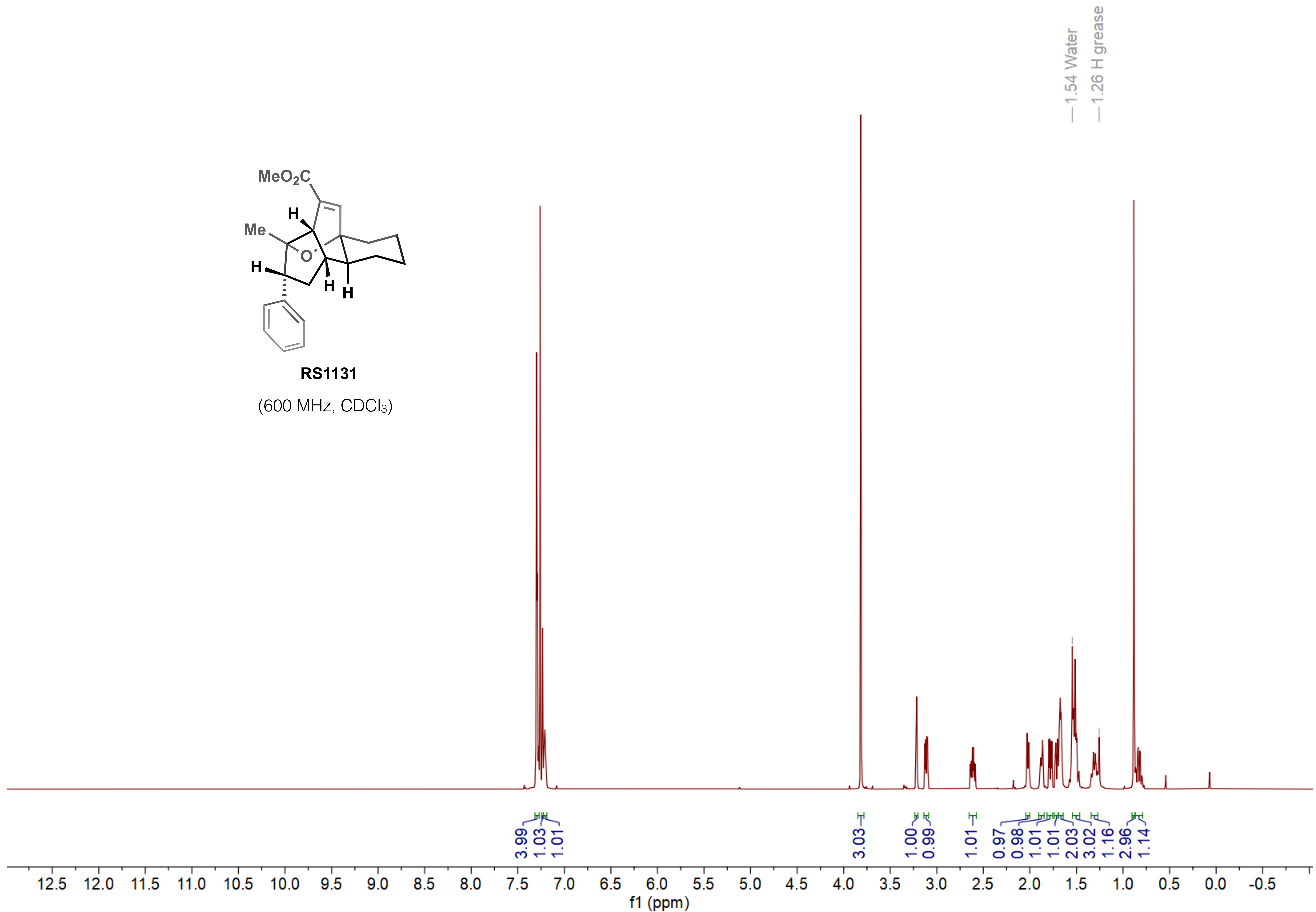
MeO2C
Me
O
H
H
H
H
RS1131
(600 MHz, CDCl3)
1.54 Water
1.26 H grease
3.99
1.03
1.01
3.03
1.00
0.99
1.01
0.97
0.98
1.01
1.01
2.03
3.02
1.16
2.96
1.14
f1 (ppm)

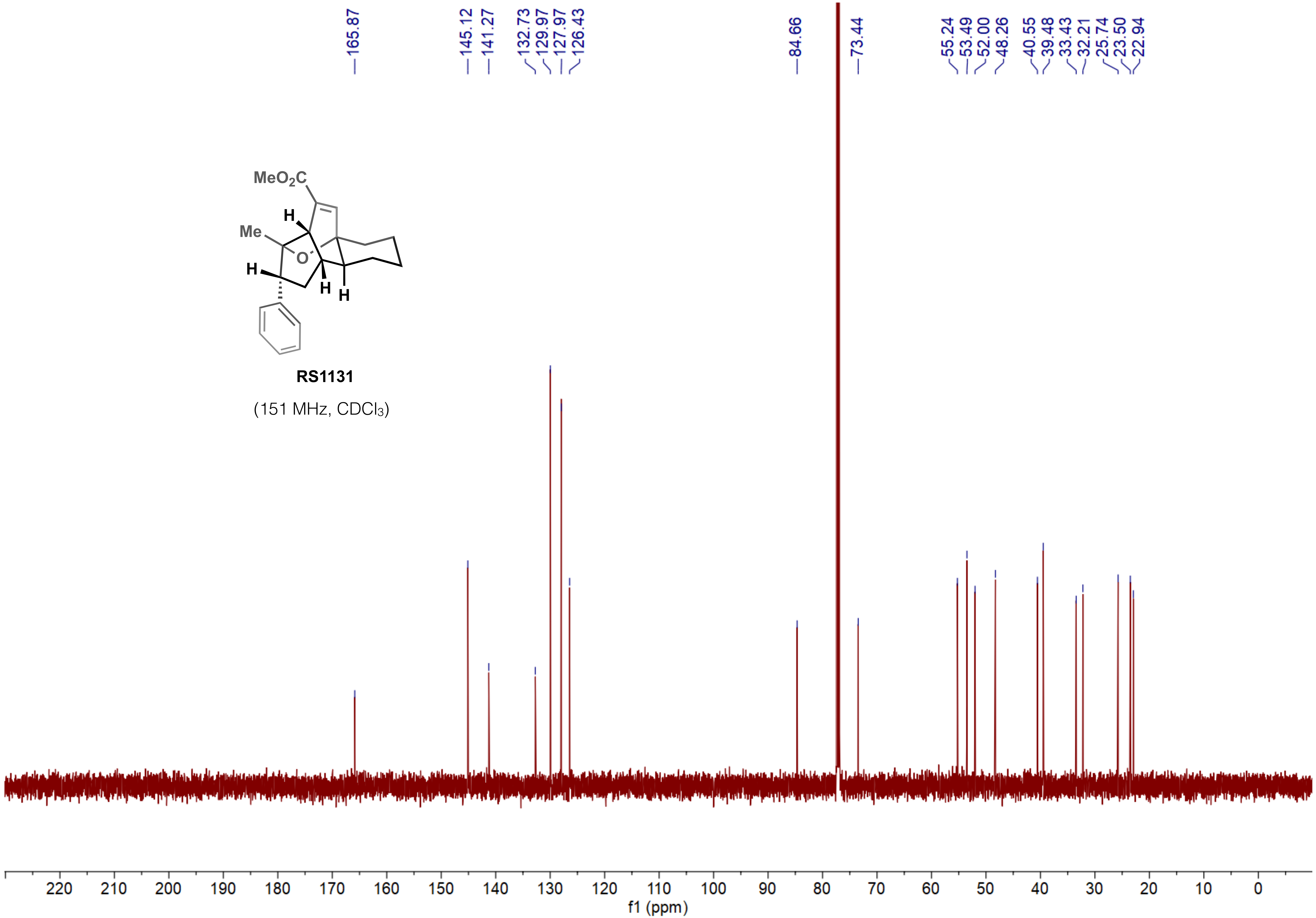
165.87
145.12
141.27
132.73
129.97
127.97
126.43
84.66
73.44
55.24
53.49
52.00
48.26
40.55
39.48
33.43
32.21
25.74
23.50
22.94
MeO2C
Me
H
O
H
H
H
RS1131
(151 MHz, CDCl3)
220
210
200
190
180
170
160
150
140
130
120
110
100
90
80
70
60
50
40
30
20
10
0
f1 (ppm)

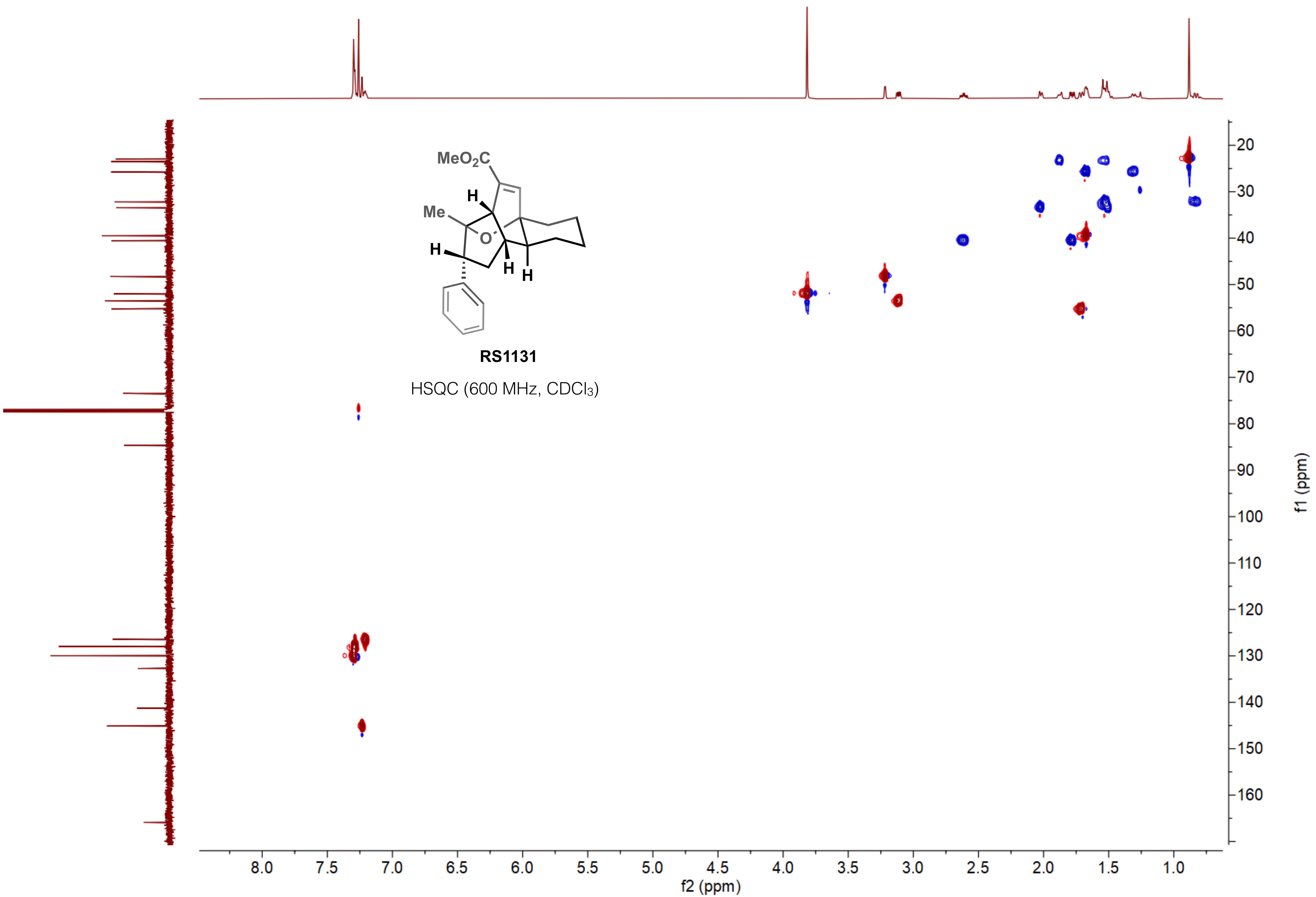

MeO2C
H
Me
O
H
H
H
RS1131
HSQC (600 MHz, CDCl3)
f2 (ppm)
f1 (ppm)

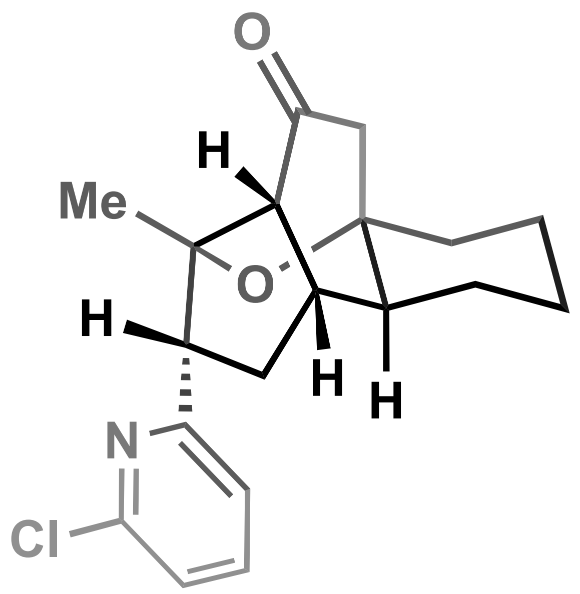

7g

(600 MHz, $CDCl_3$)

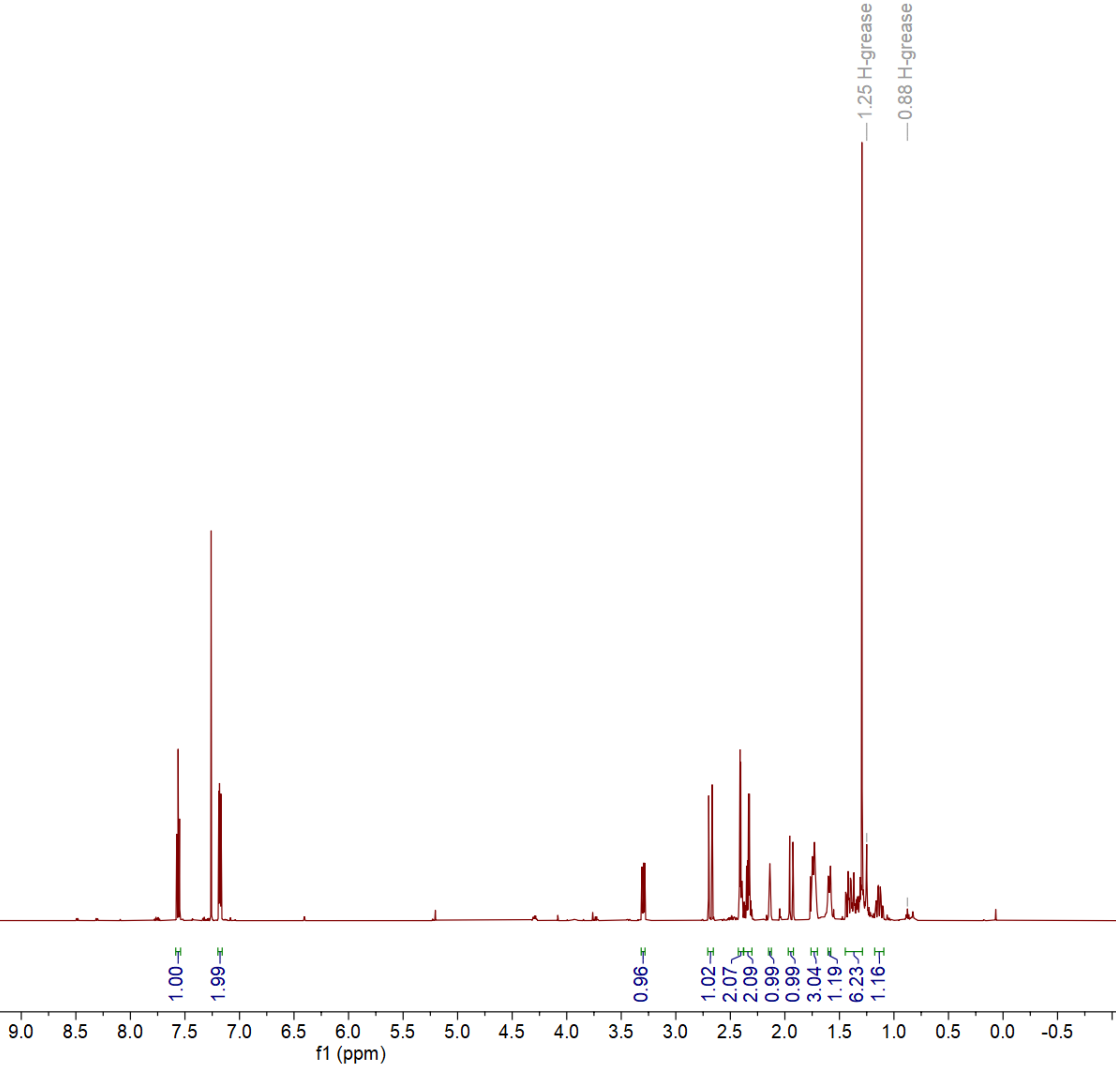

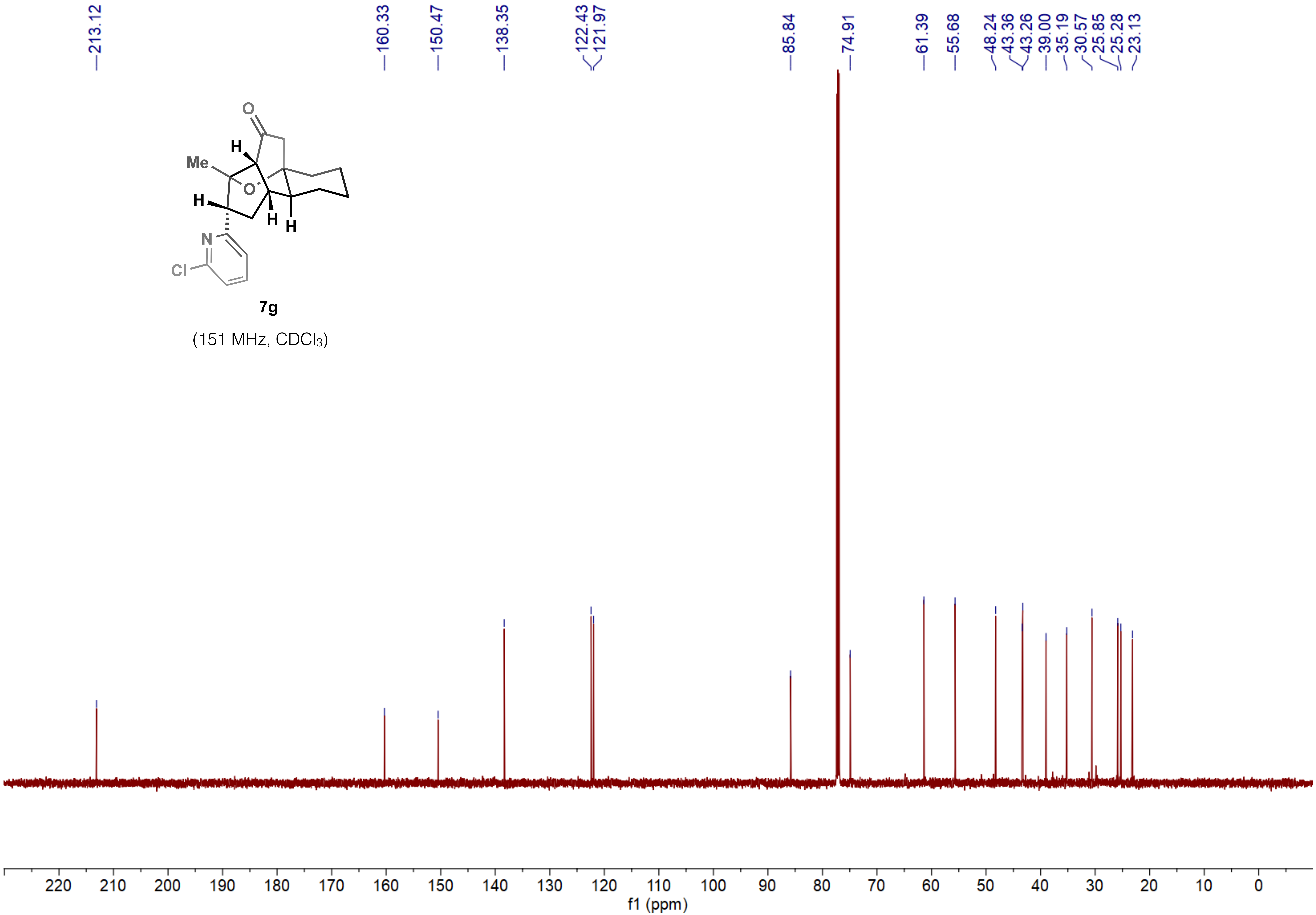
213.12
160.33
150.47
138.35
122.43
121.97
85.84
74.91
61.39
55.68
48.24
43.36
43.26
39.00
35.19
30.57
25.85
25.28
23.13
O
H
Me
O
H
H
H
N
Cl
7g
(151 MHz, $CDCl_3$)
220
210
200
190
180
170
160
150
140
130
120
110
100
90
80
70
60
50
40
30
20
10
0
f1 (ppm)

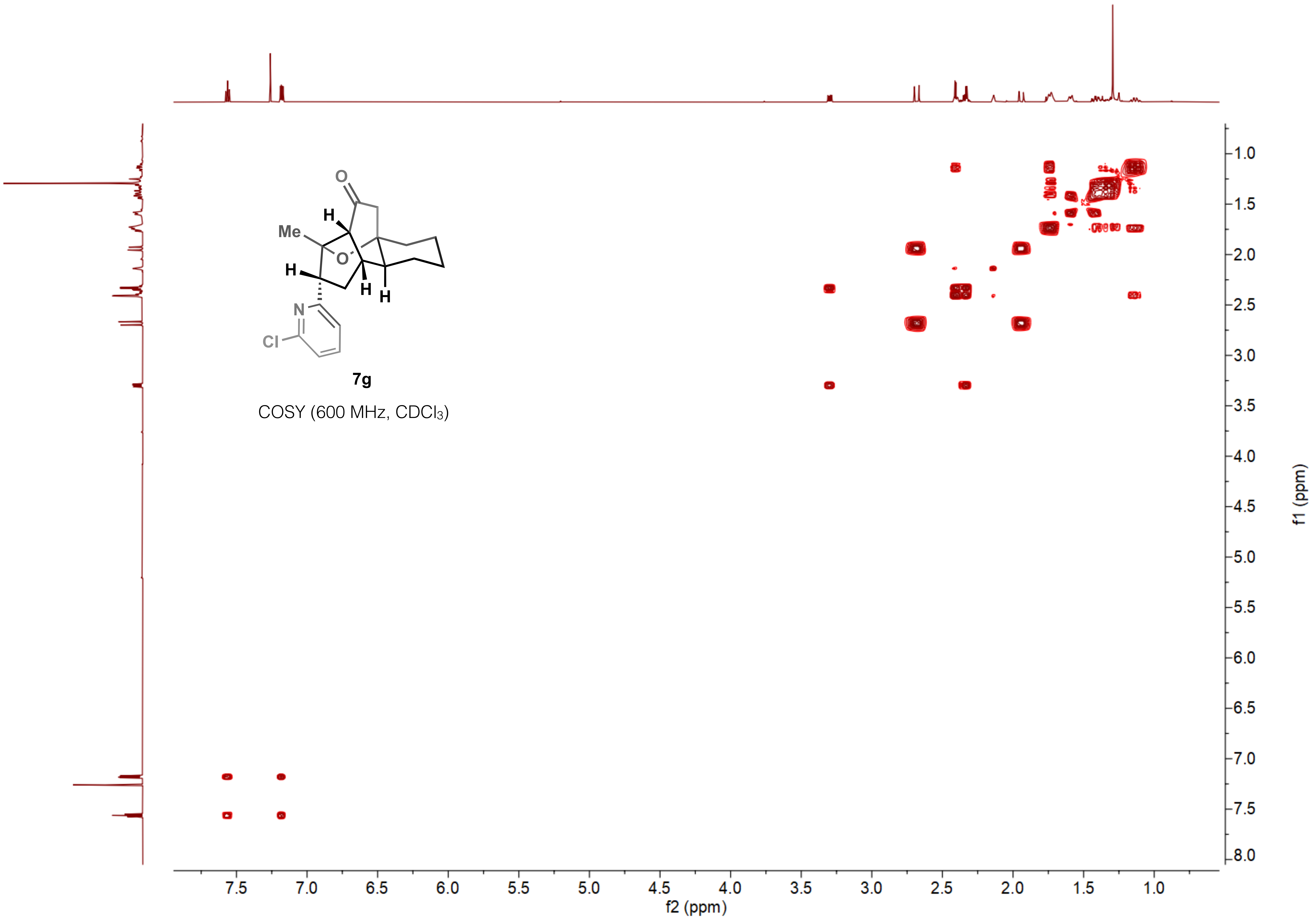
O
H
Me
O
H
H
H
N
Cl
7g
COSY (600 MHz, CDCl3)
f2 (ppm)
f1 (ppm)
7.5
7.0
6.5
6.0
5.5
5.0
4.5
4.0
3.5
3.0
2.5
2.0
1.5
1.0
8.0

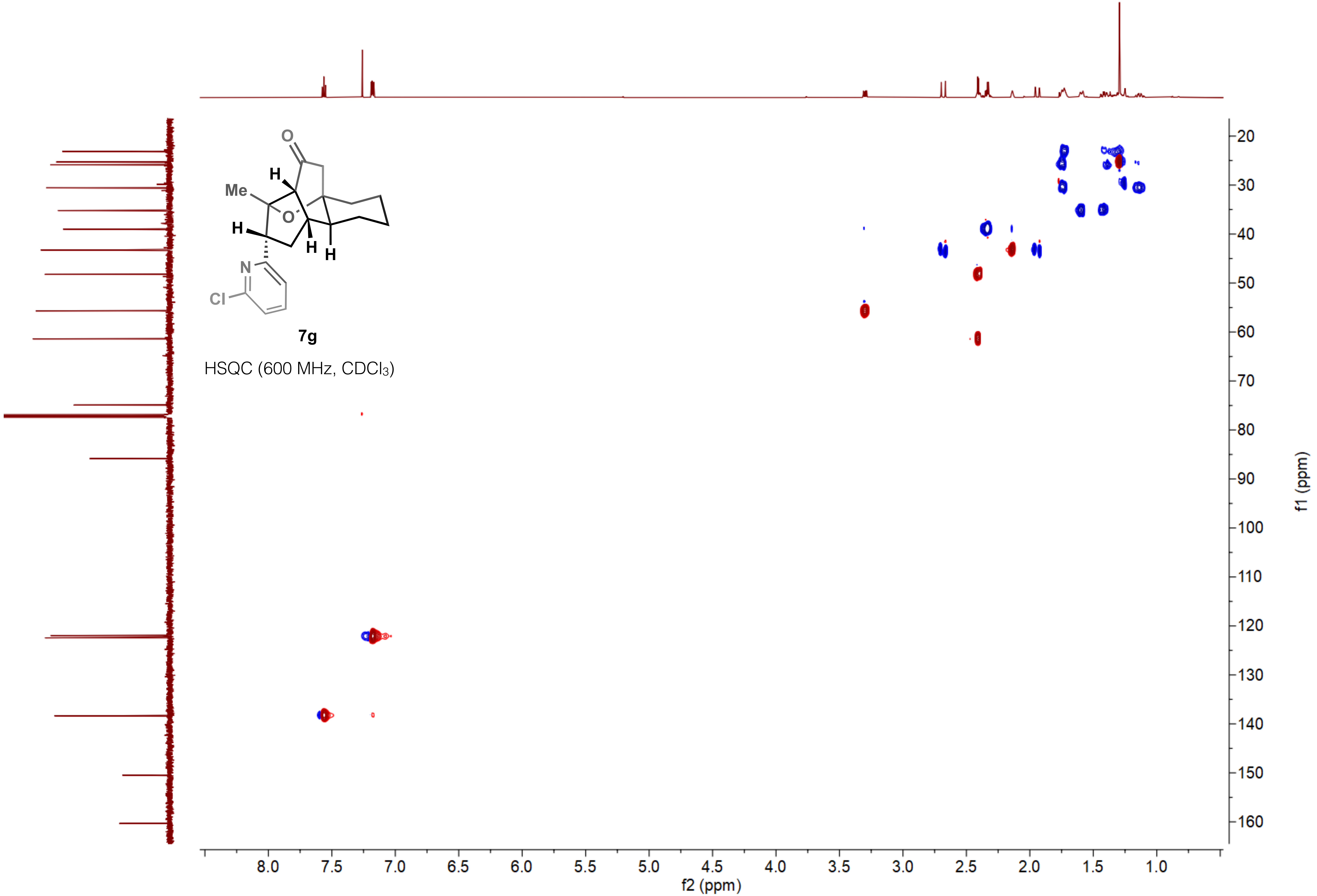

7g
HSQC (600 MHz, CDCl3)
f2 (ppm)
f1 (ppm)

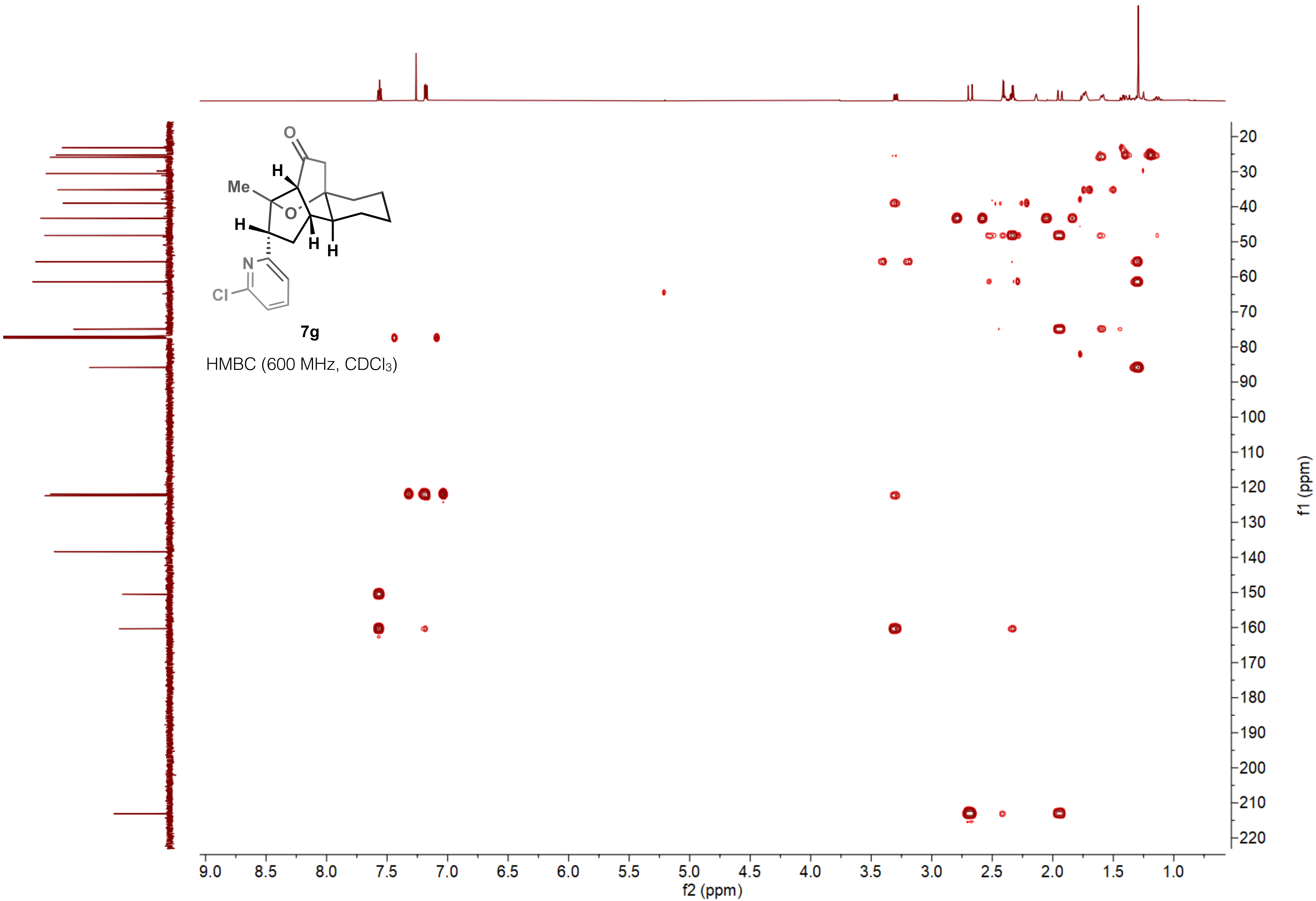
O
H
Me
O
H
H
H
N
Cl
7g
HMBC (600 MHz, CDCl3)
f2 (ppm)
f1 (ppm)
9.0 8.5 8.0 7.5 7.0 6.5 6.0 5.5 5.0 4.5 4.0 3.5 3.0 2.5 2.0 1.5 1.0
20 30 40 50 60 70 80 90 100 110 120 130 140 150 160 170 180 190 200 210 220

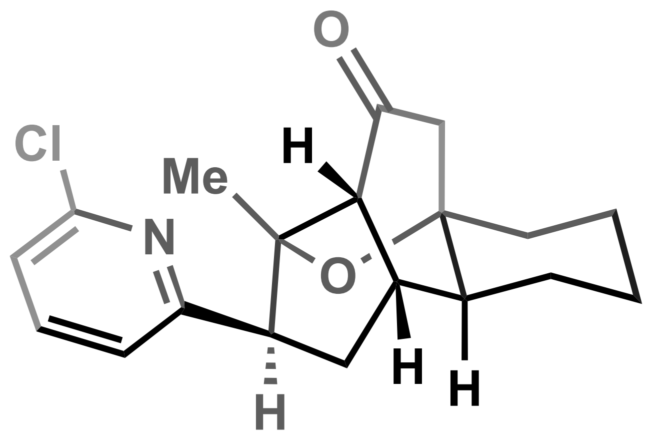

8g

(600 MHz, $CDCl_3$)

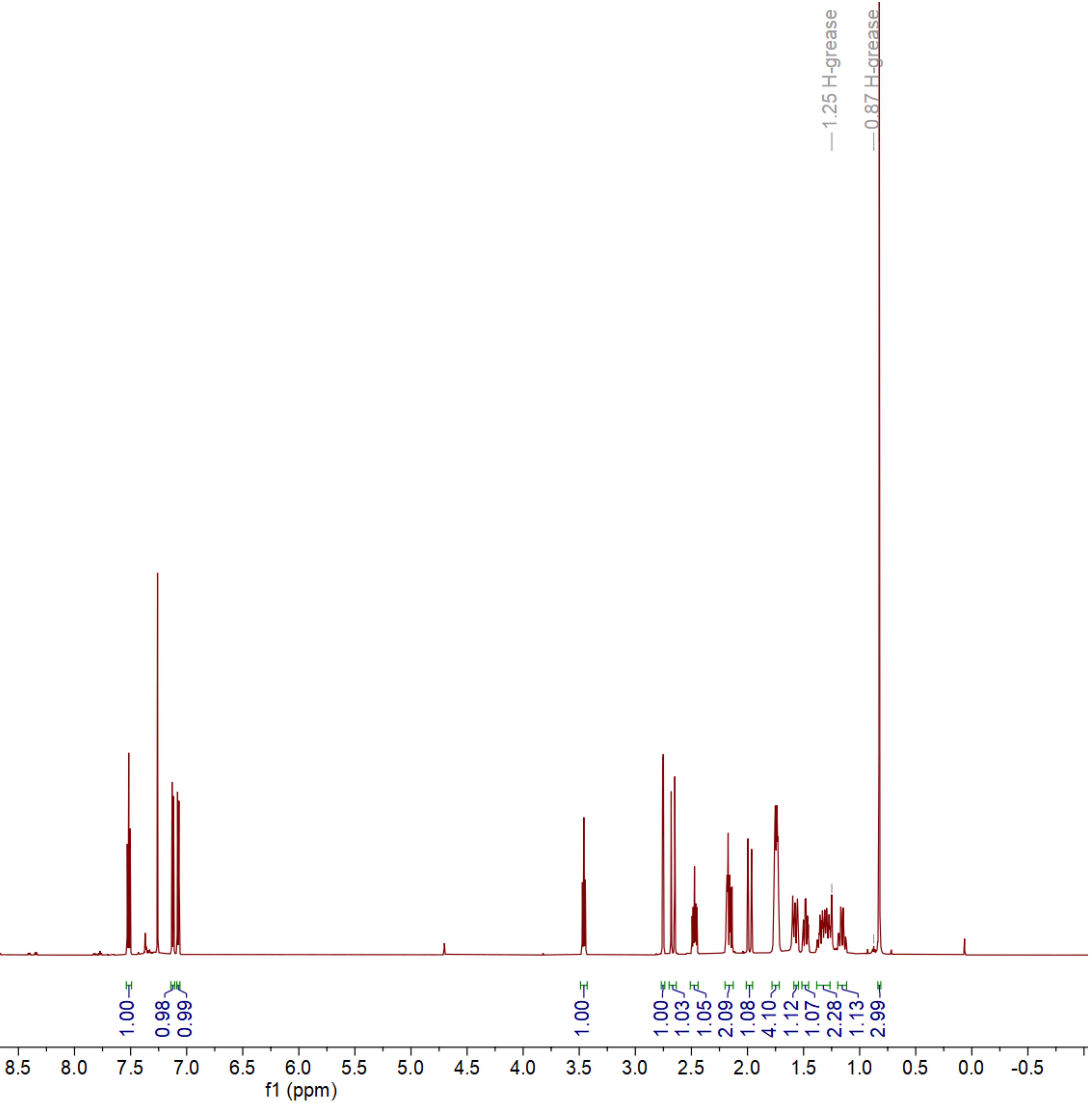

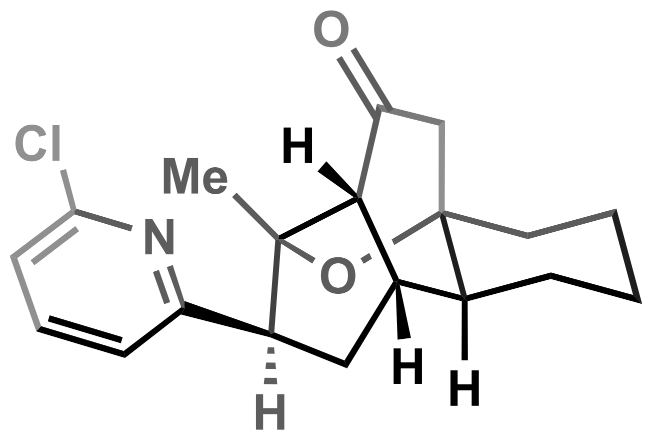

8g

(151 MHz, $CDCl_3$)

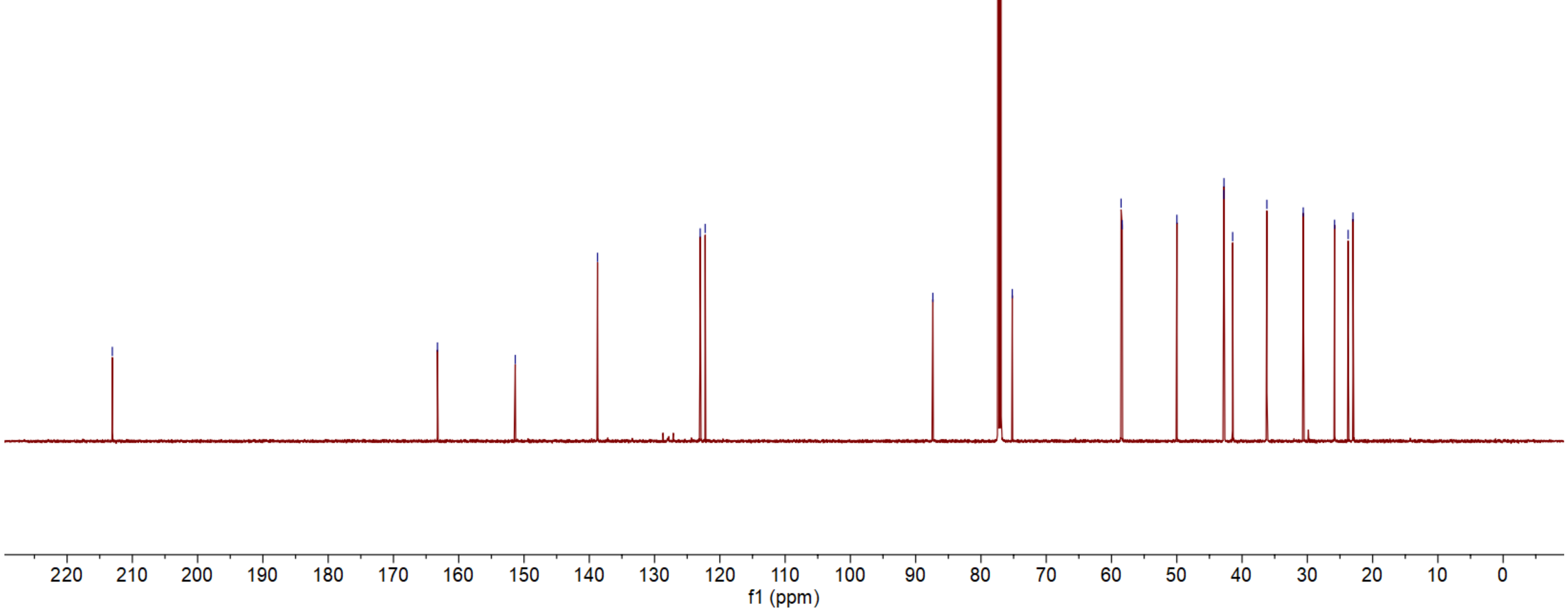

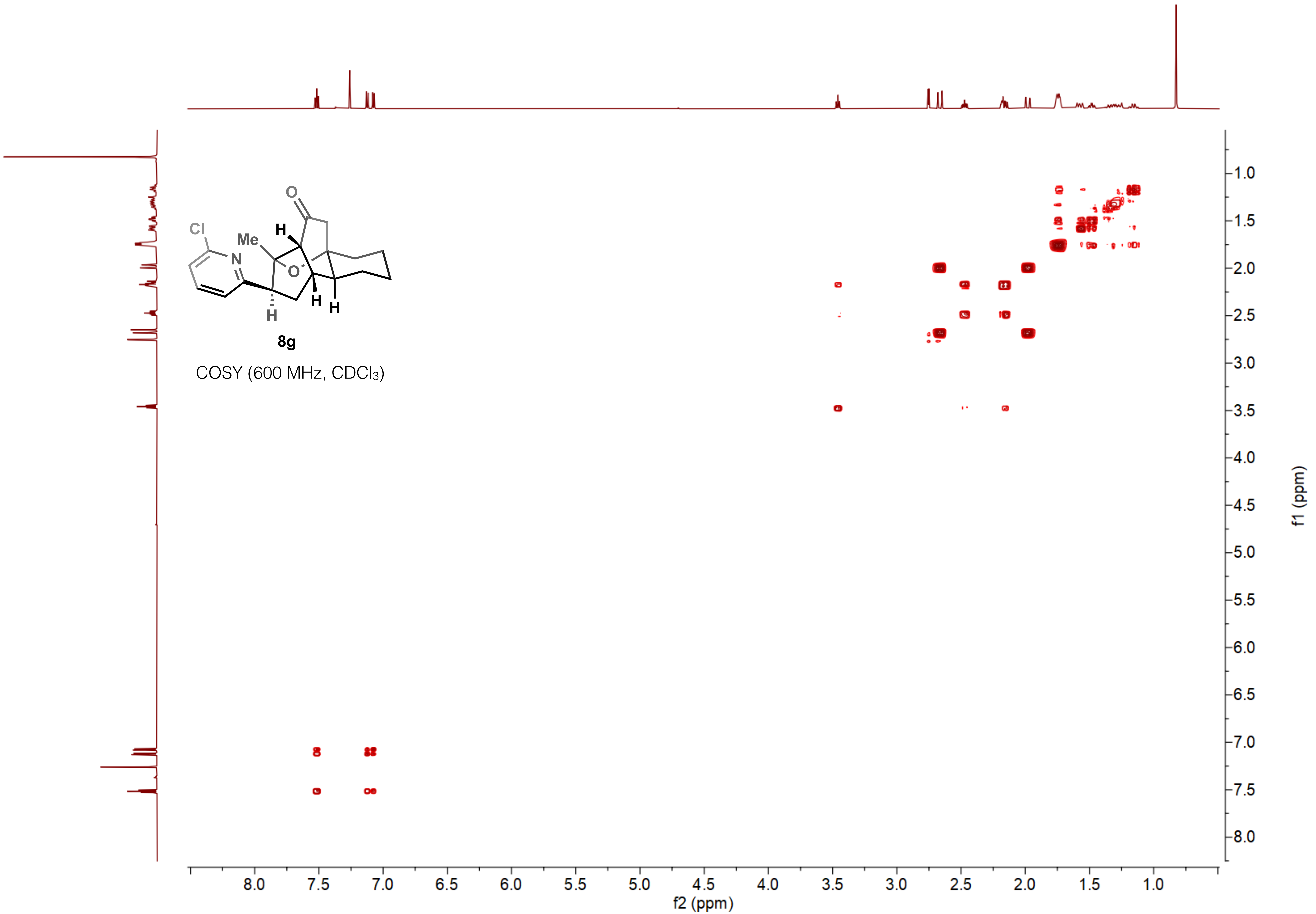
Cl
N
Me
H
O
O
H
H
H
8g
COSY (600 MHz, CDCl3)
f2 (ppm)
f1 (ppm)

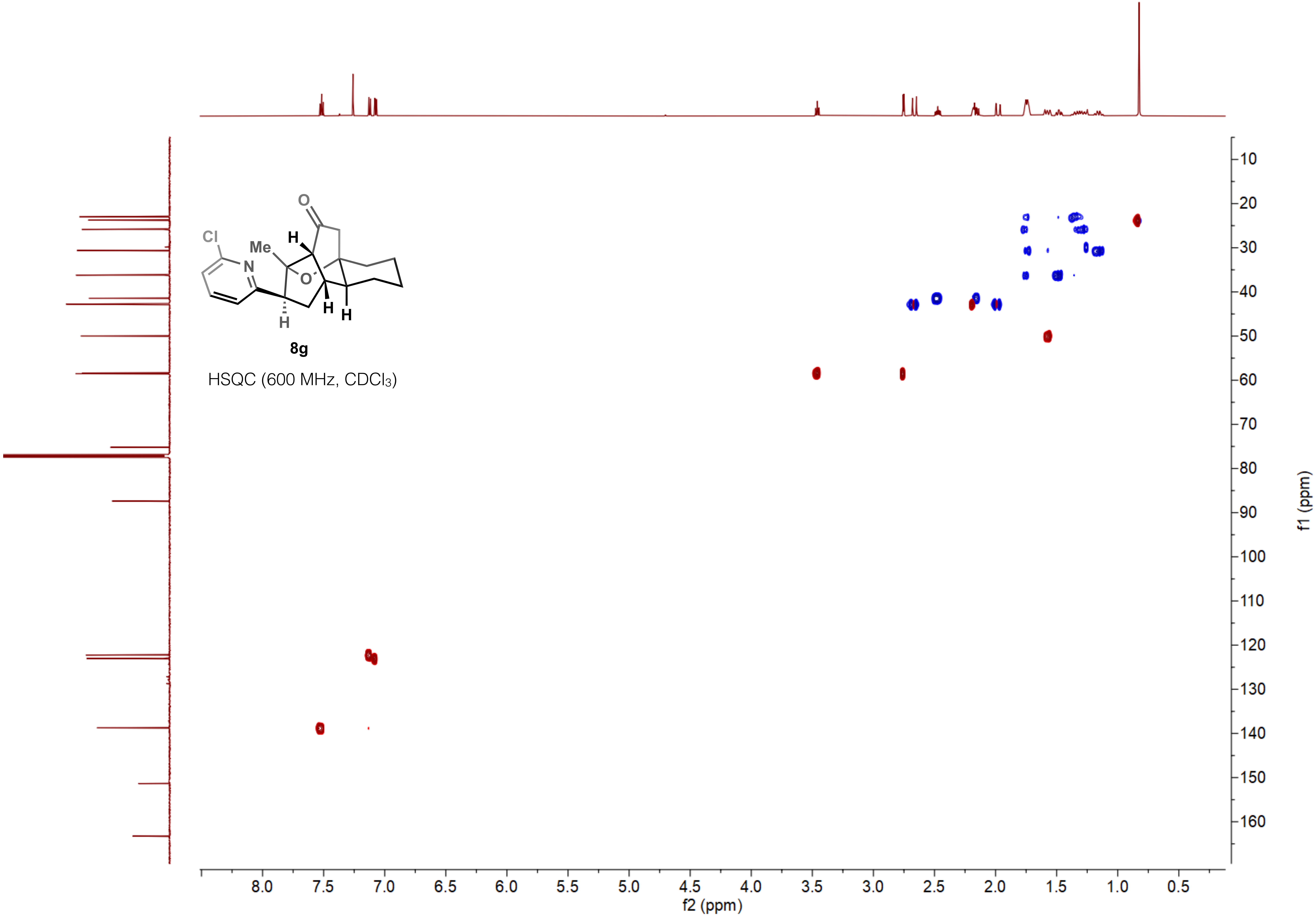
8g
HSQC (600 MHz, CDCl3)
Cl
N
Me
H
O
H
H
H
f2 (ppm)
f1 (ppm)

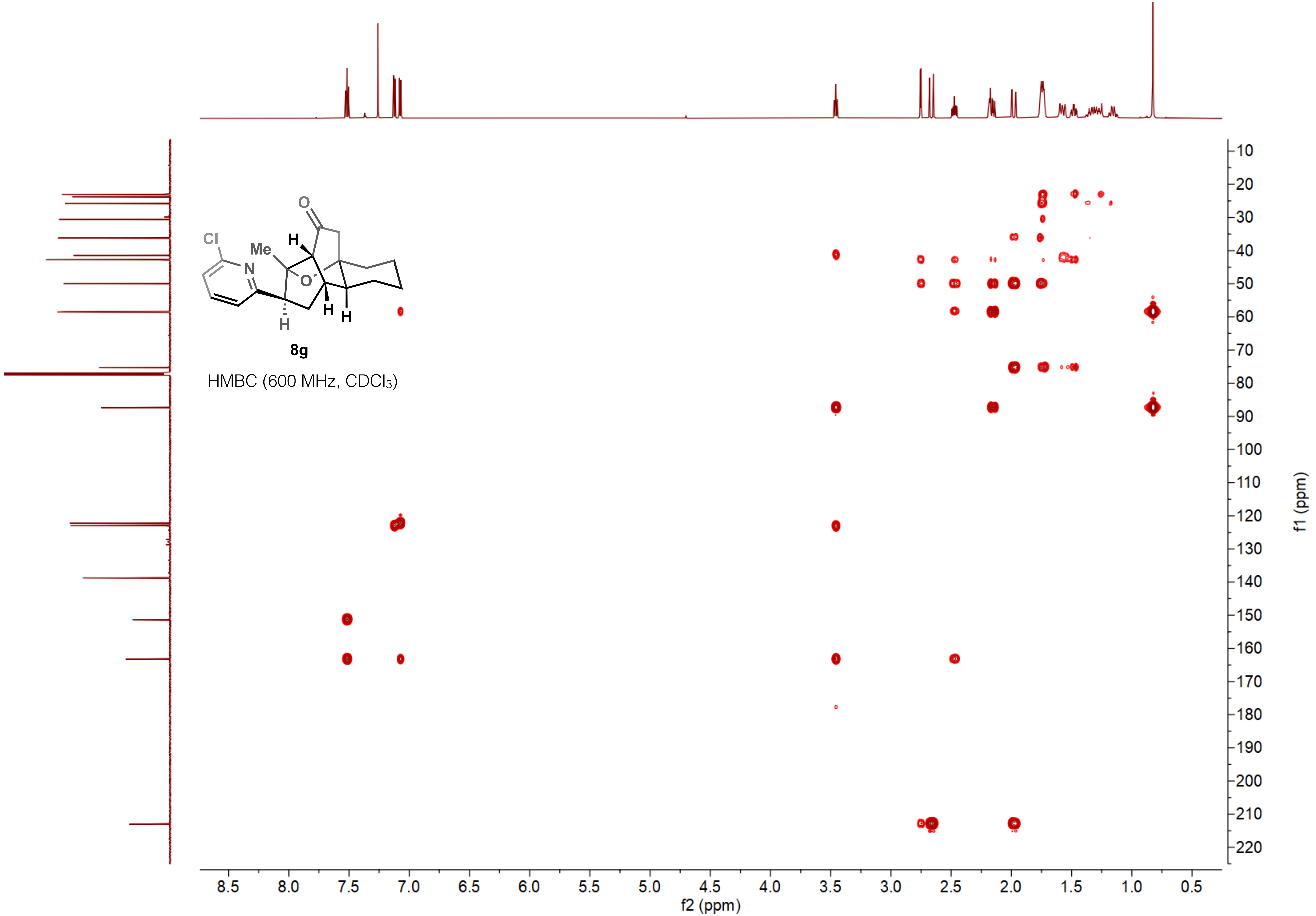
Cl
N
Me
H
O
O
H
H
H
8g
HMBC (600 MHz, CDCl3)
f2 (ppm)
f1 (ppm)

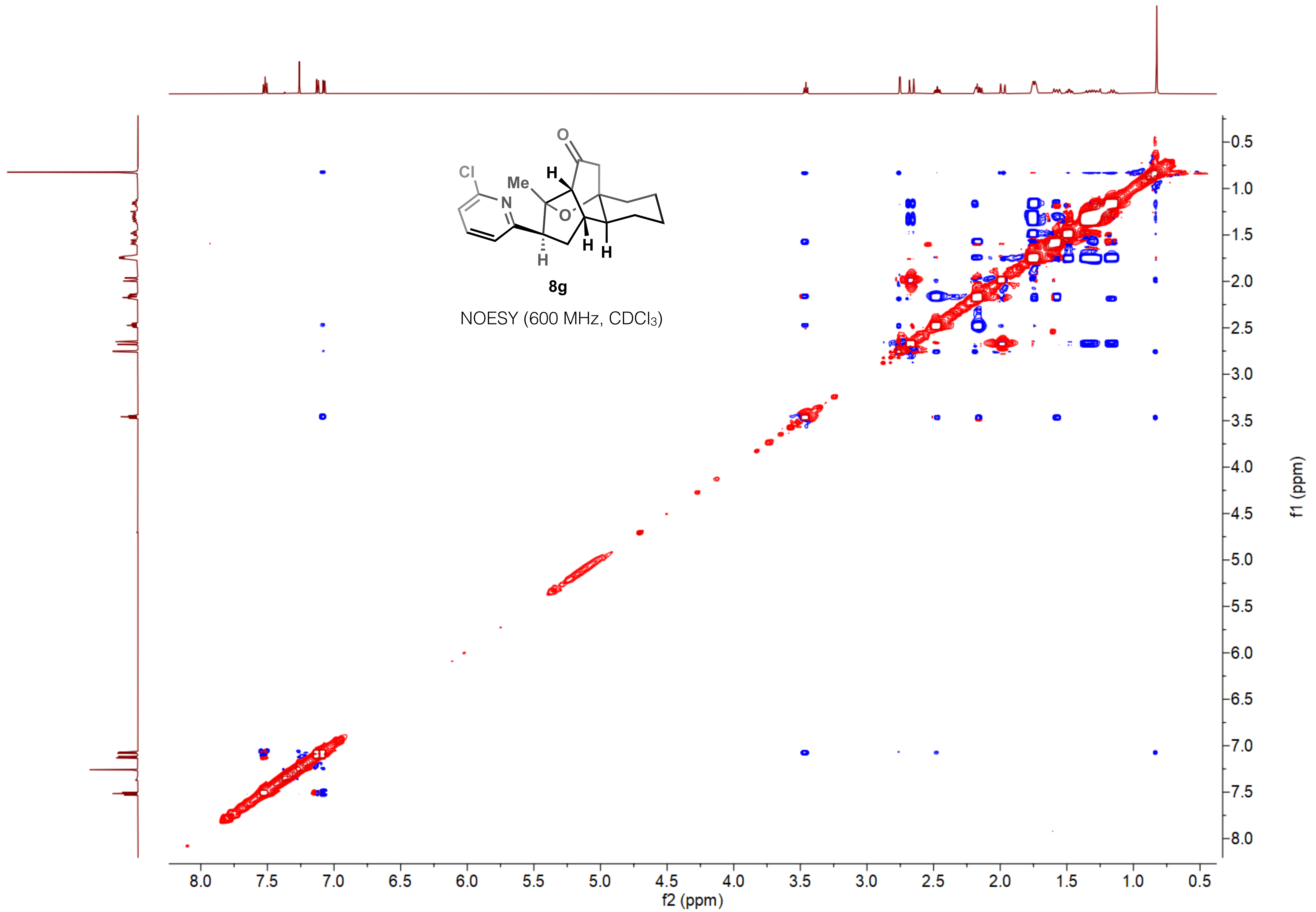

Cl
N
Me
O
H
H
H
H
8g
NOESY (600 MHz, CDCl3)
f2 (ppm)
f1 (ppm)

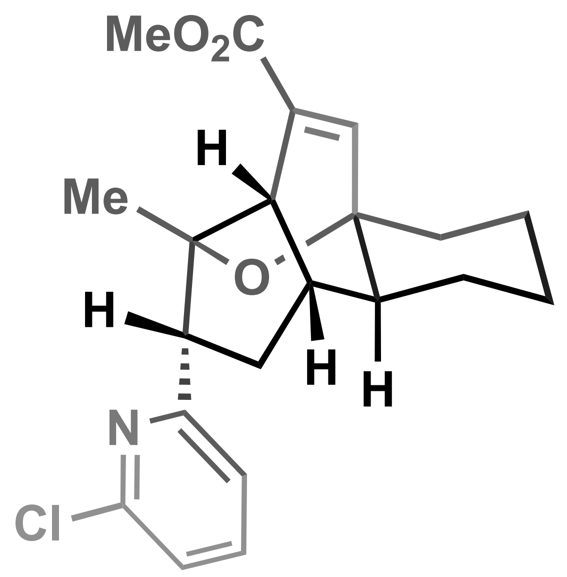

RS1138

(600 MHz, $CDCl_3$)

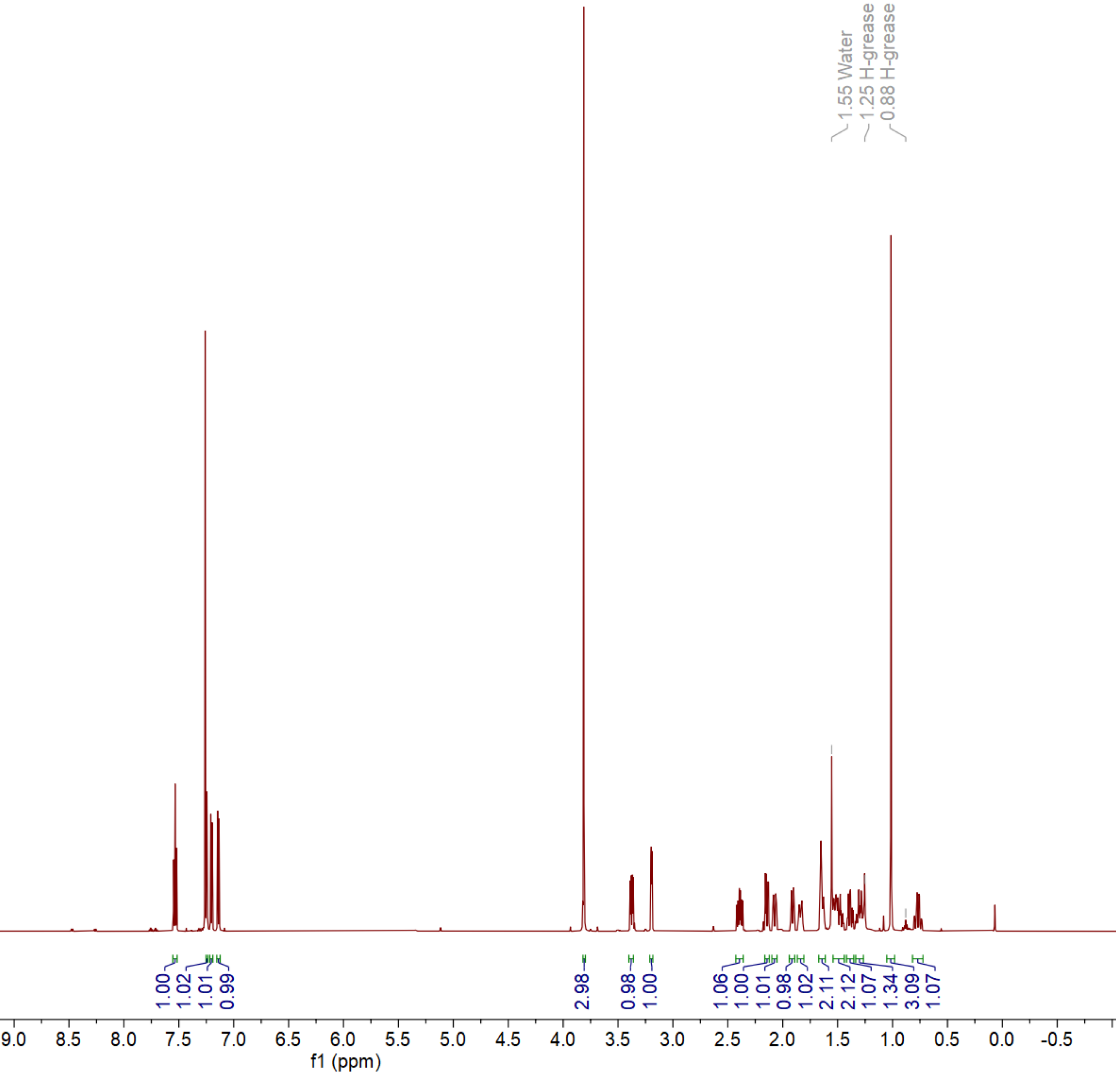

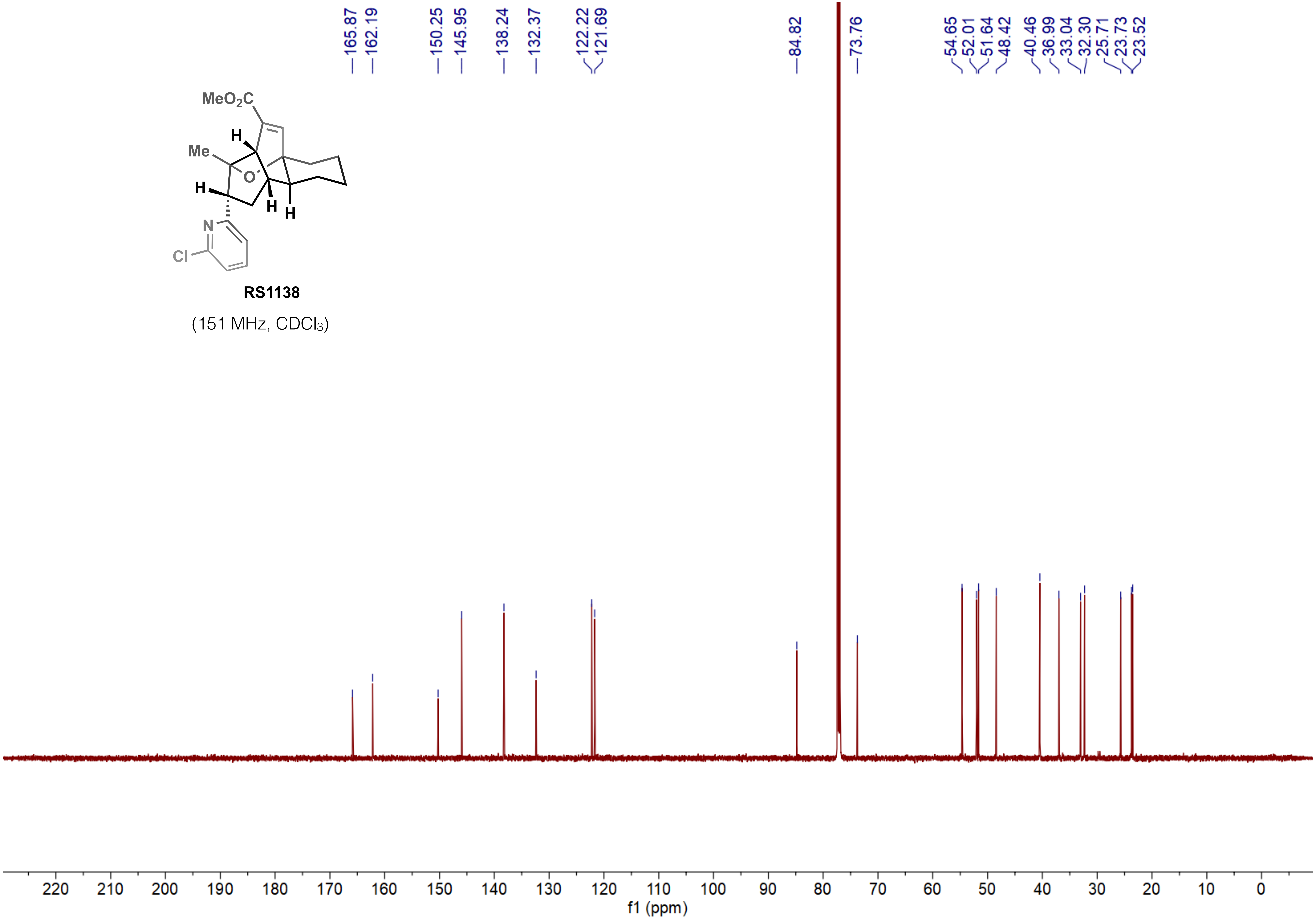

RS1138

(151 MHz, $CDCl_3$)

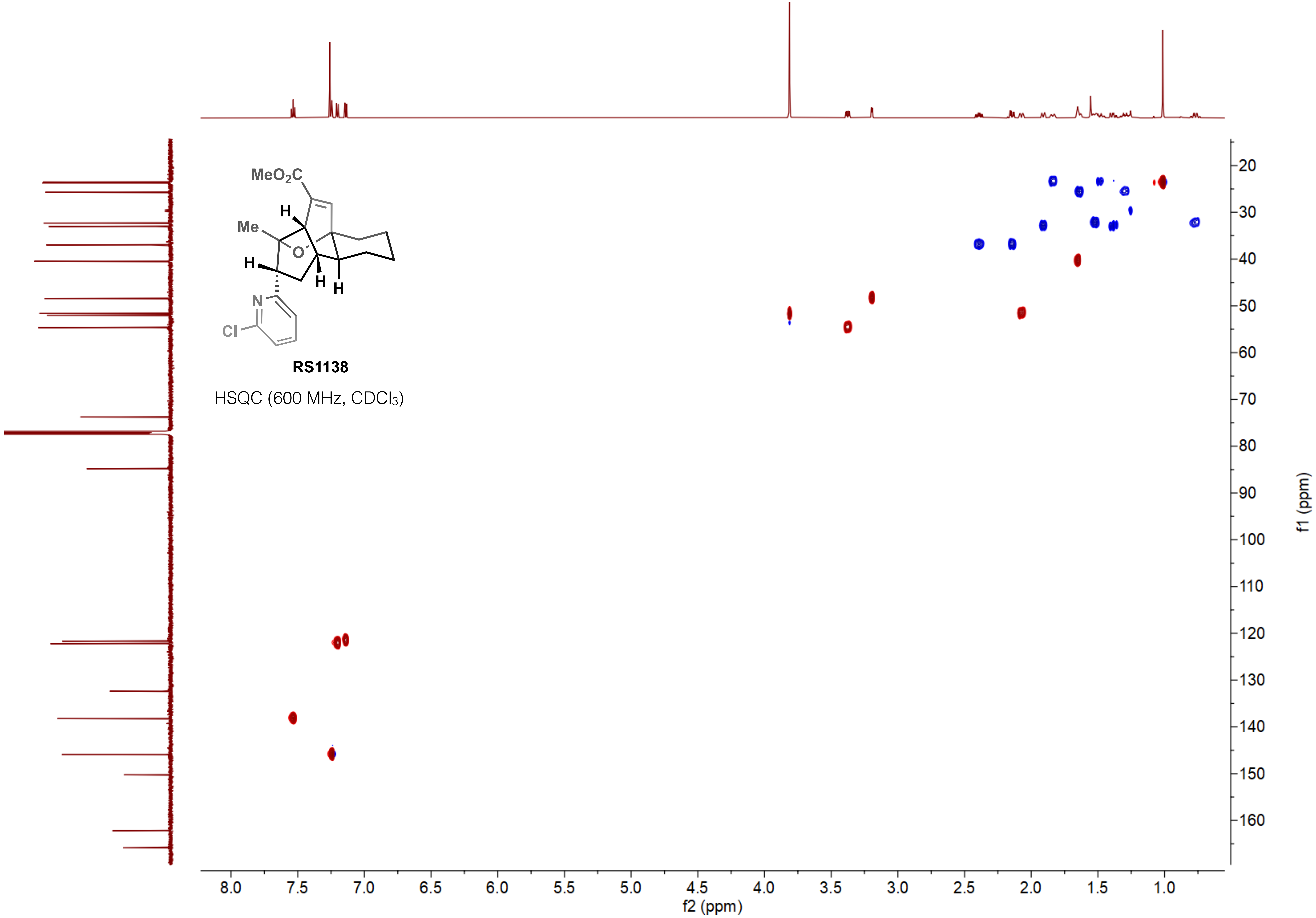

MeO2C
Me
H
O
H
H
H
N
Cl
RS1138
HSQC (600 MHz, CDCl3)
f2 (ppm)
f1 (ppm)

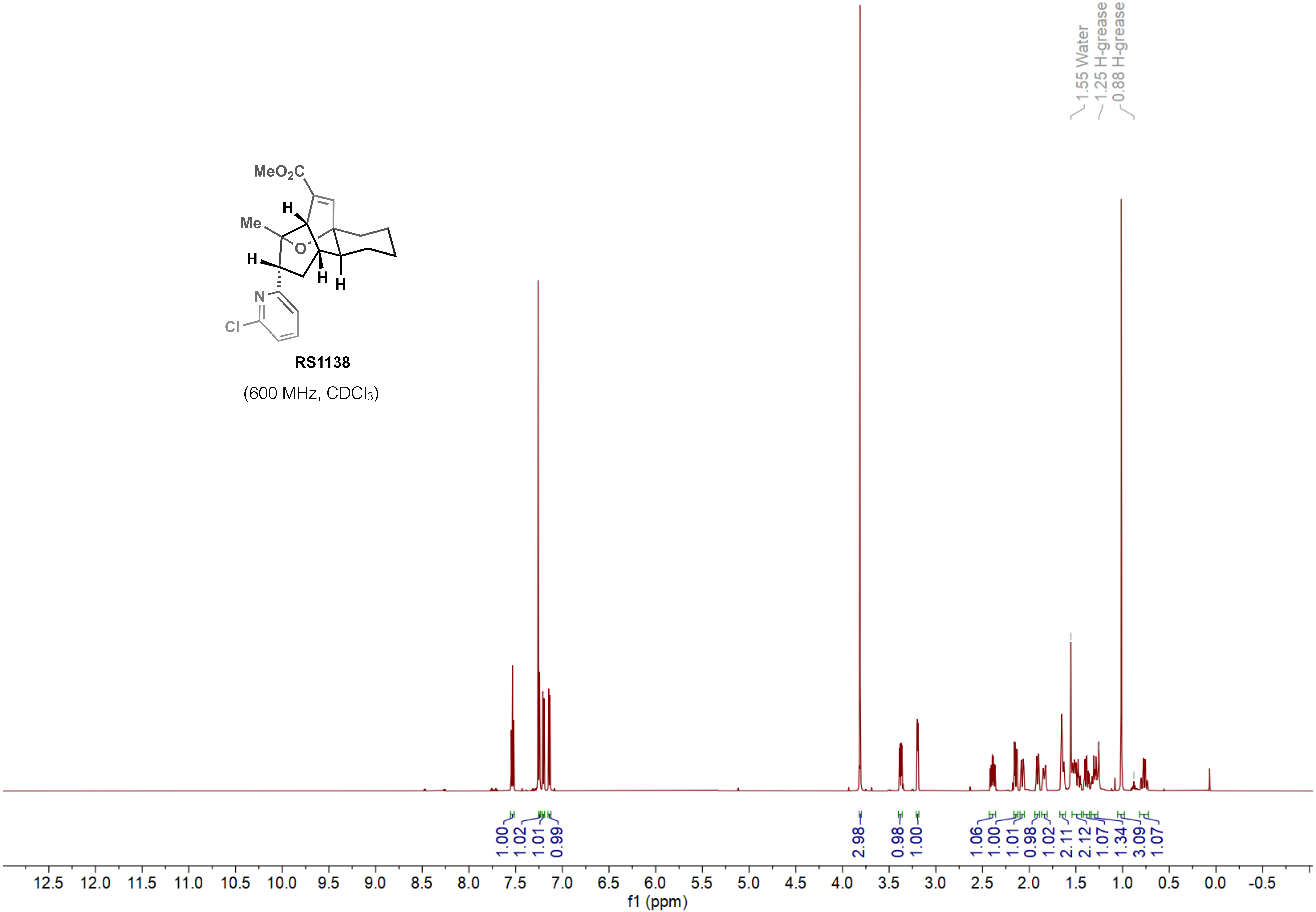

RS1138

(600 MHz, $CDCl_3$)

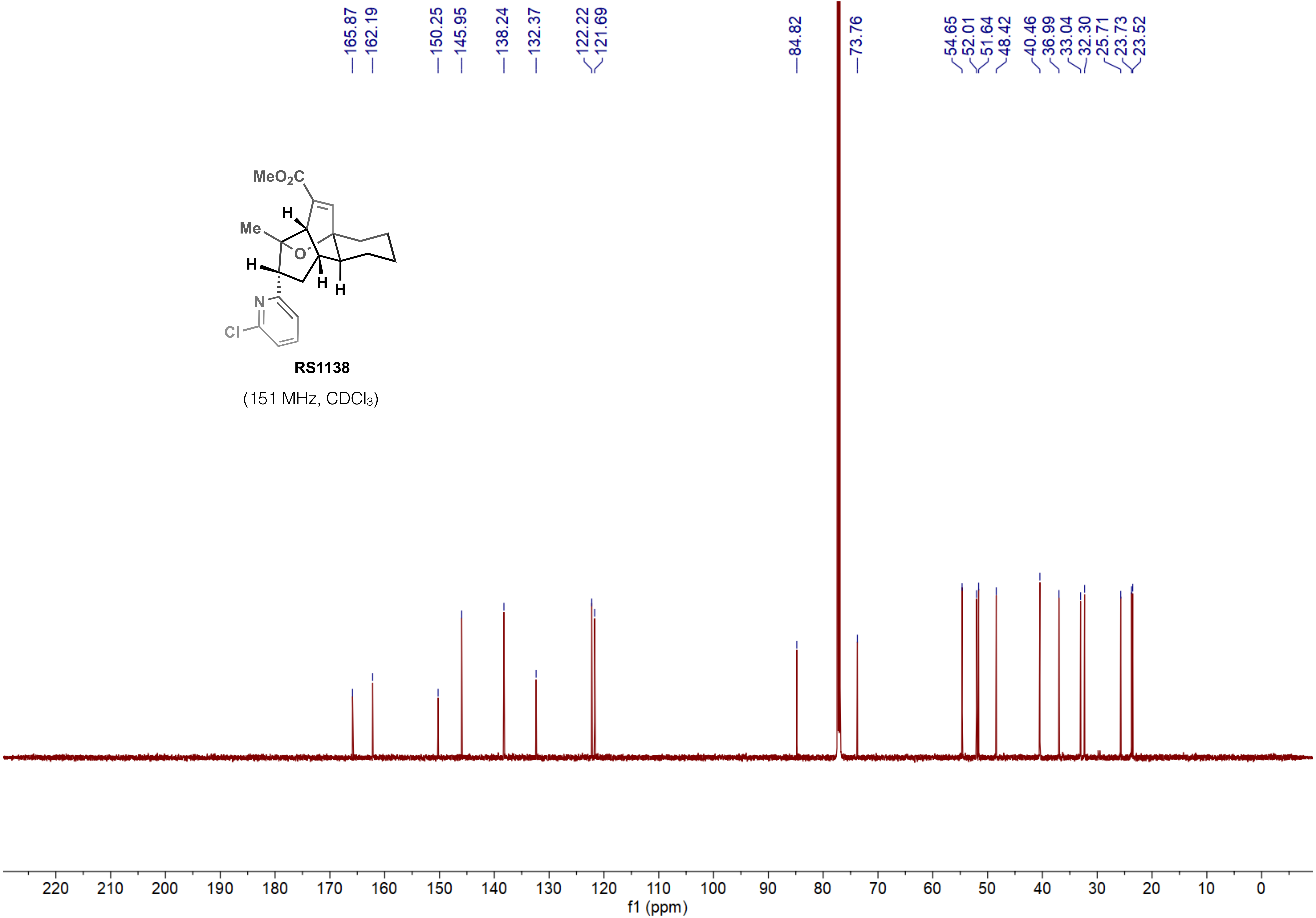

RS1138

(151 MHz, $CDCl_3$)

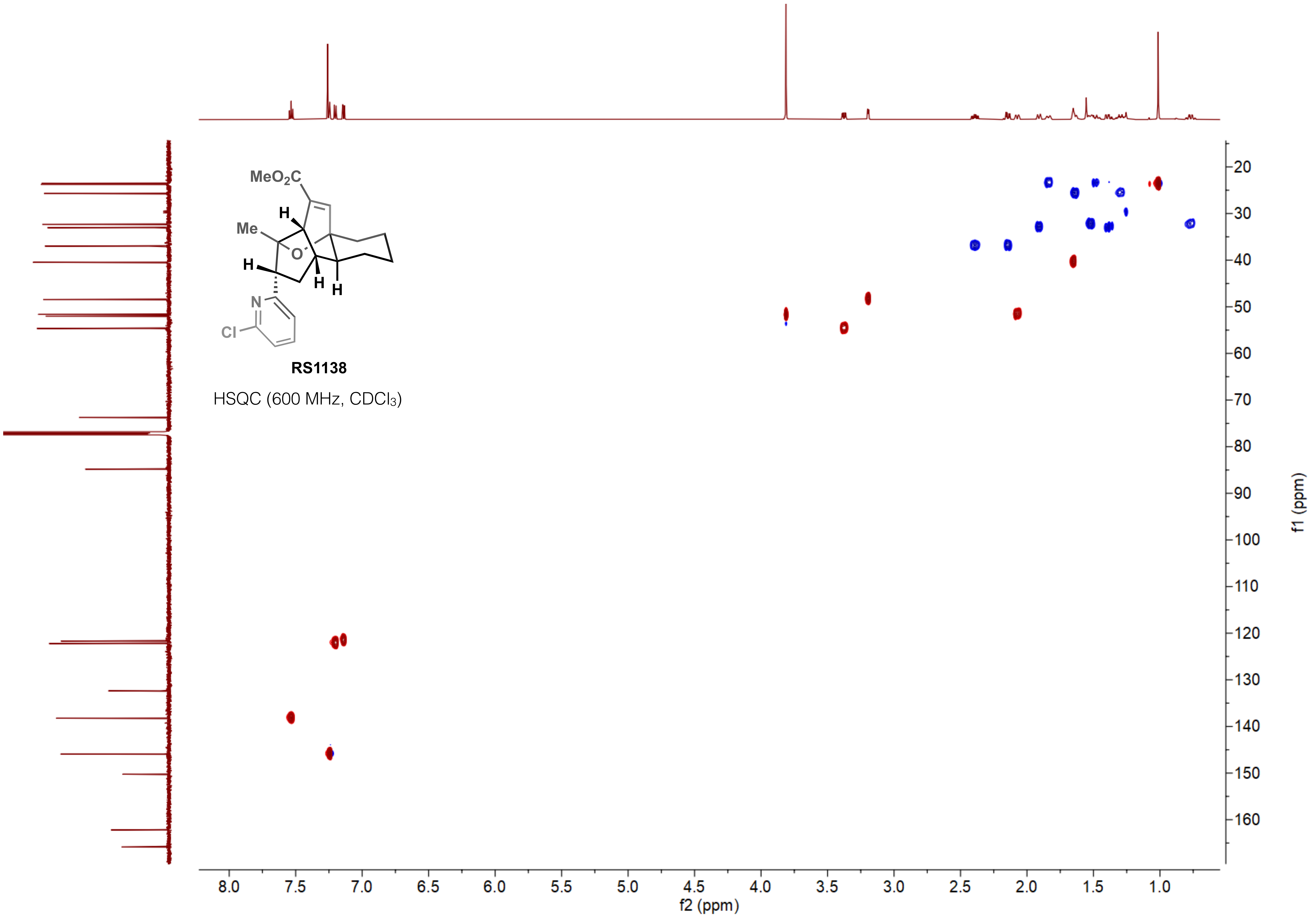
MeO2C
Me
H
O
H
H
H
N
Cl
RS1138
HSQC (600 MHz, CDCl3)
f2 (ppm)
f1 (ppm)

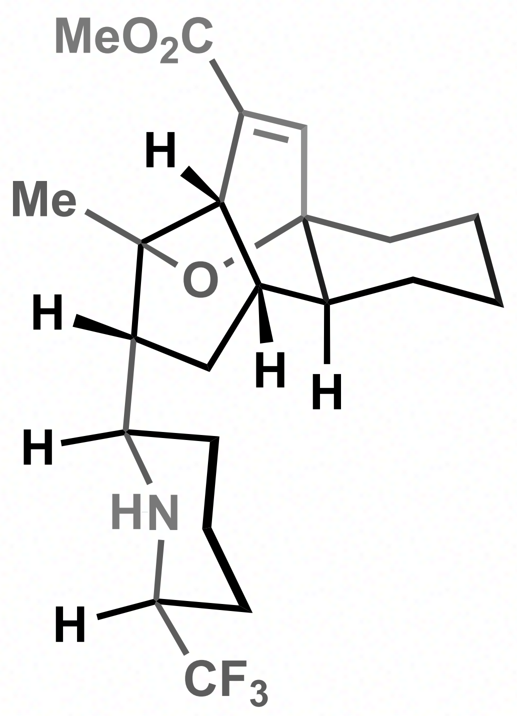

RS1122

(600 MHz, CDCl3)

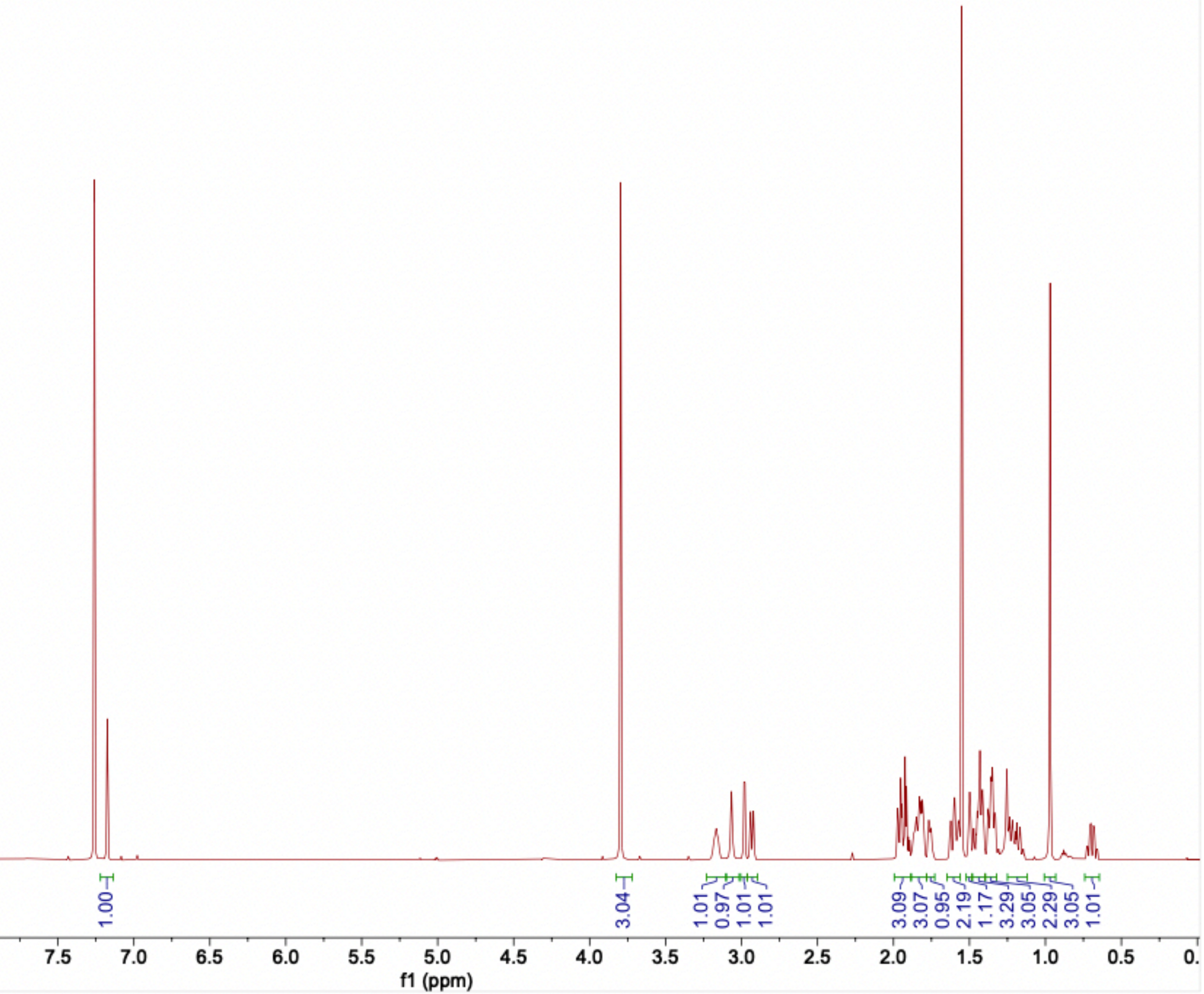

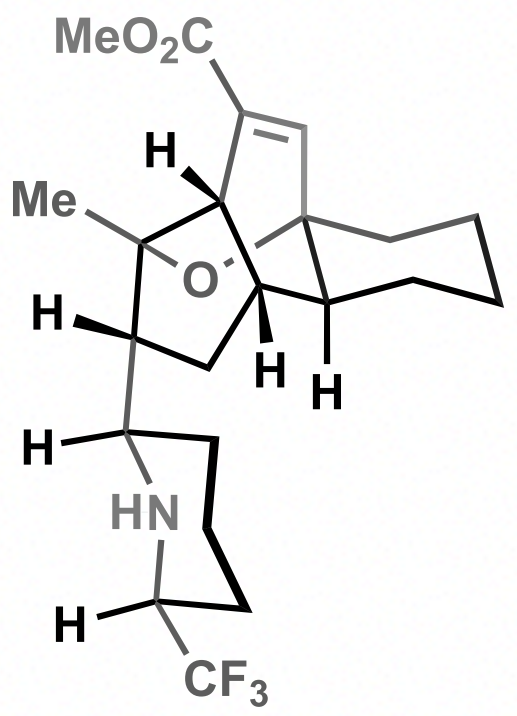

RS1122

(151 MHz, $CDCl_3$)

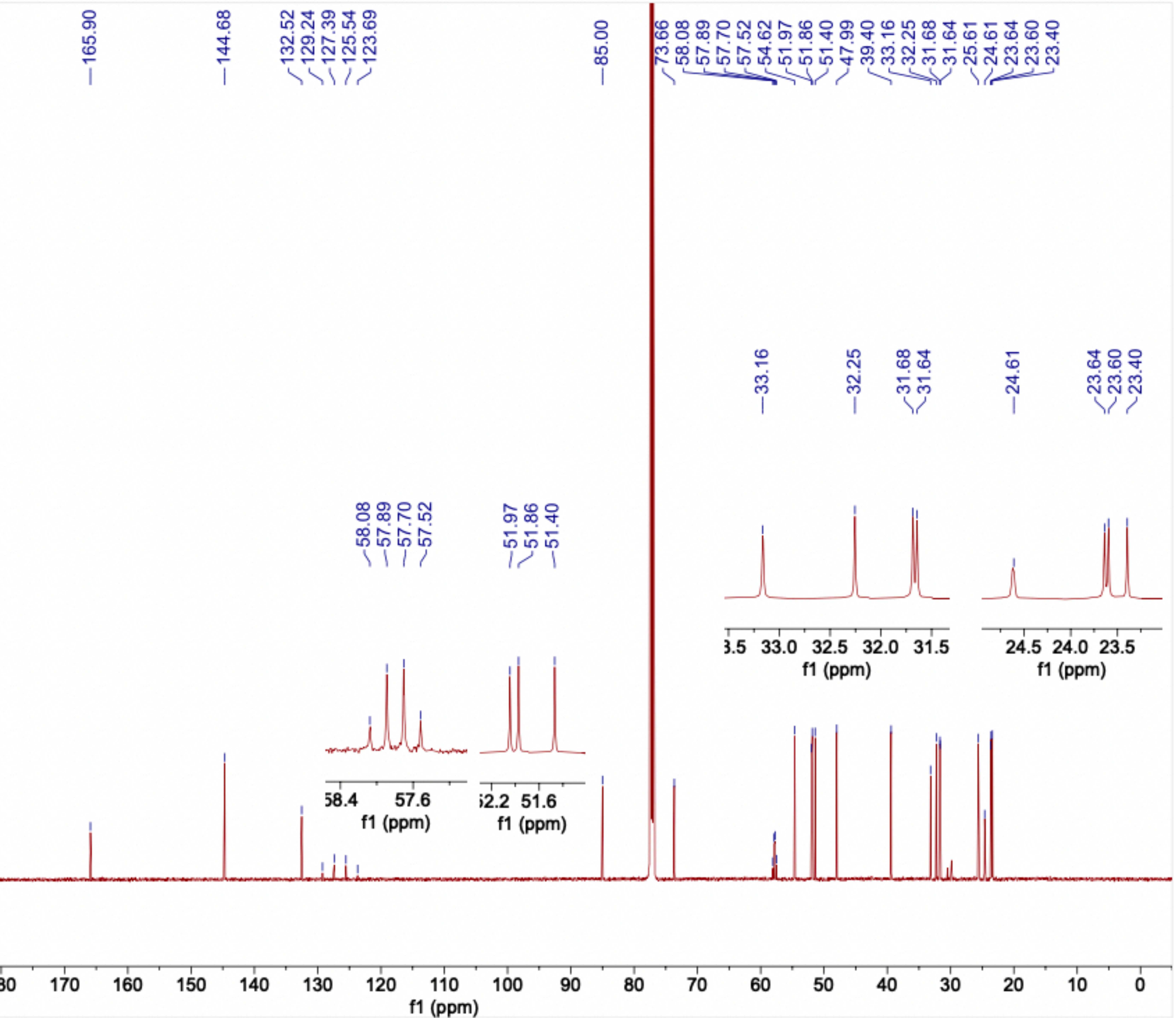

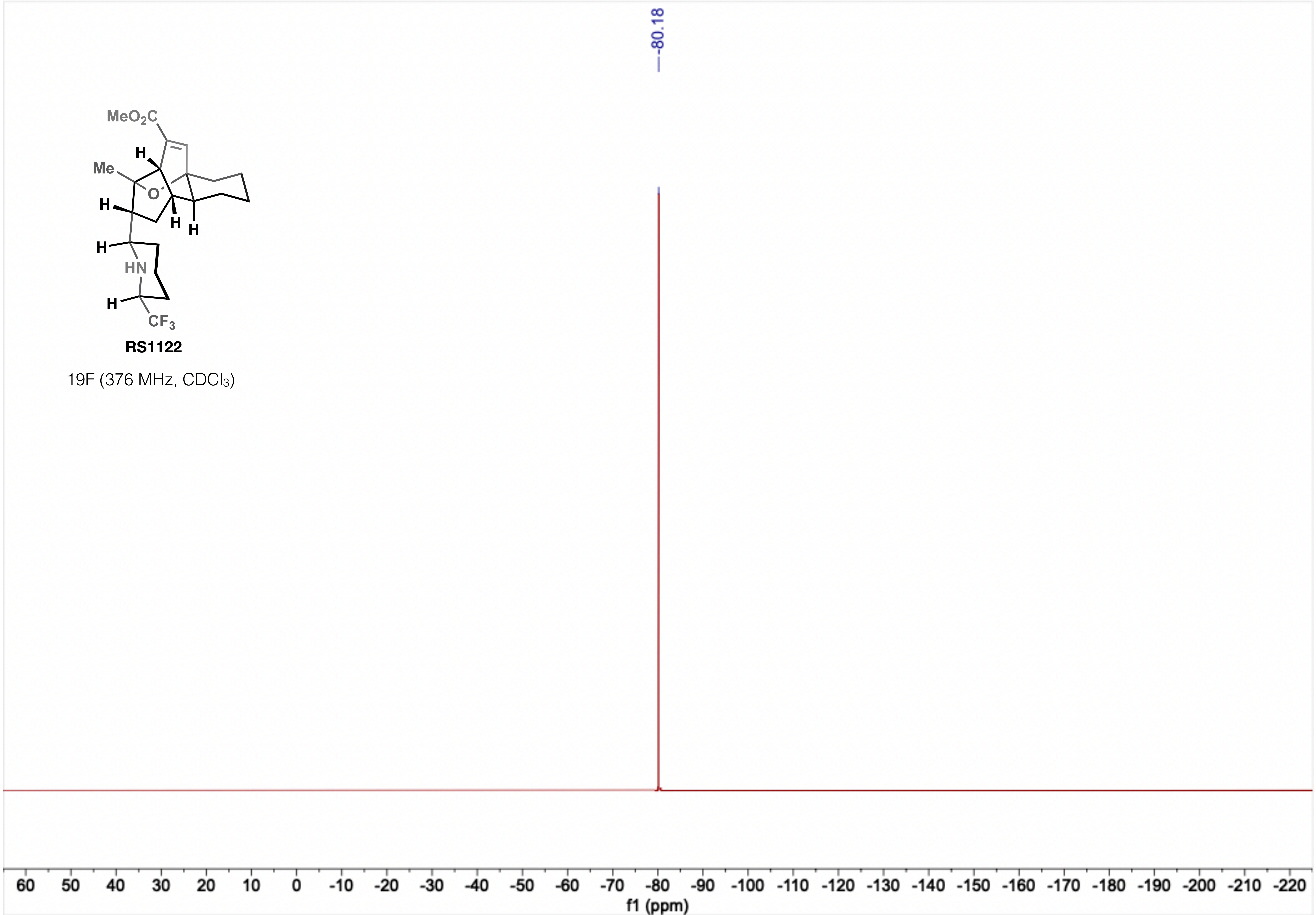

RS1122

19F (376 MHz, $CDCl_3$)

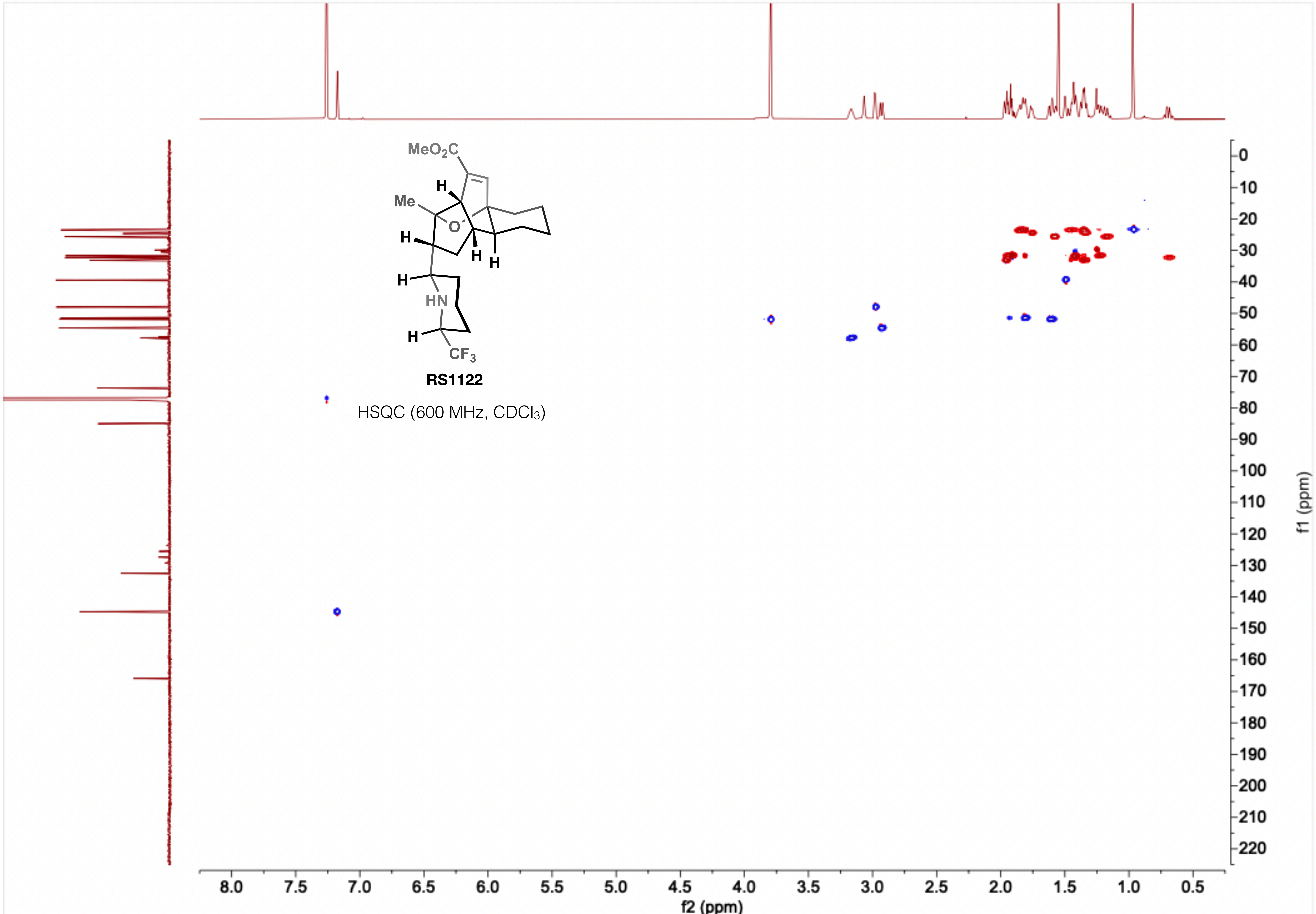
MeO2C
Me
O
H
HN
CF3
RS1122
HSQC (600 MHz, CDCl3)
f2 (ppm)
f1 (ppm)

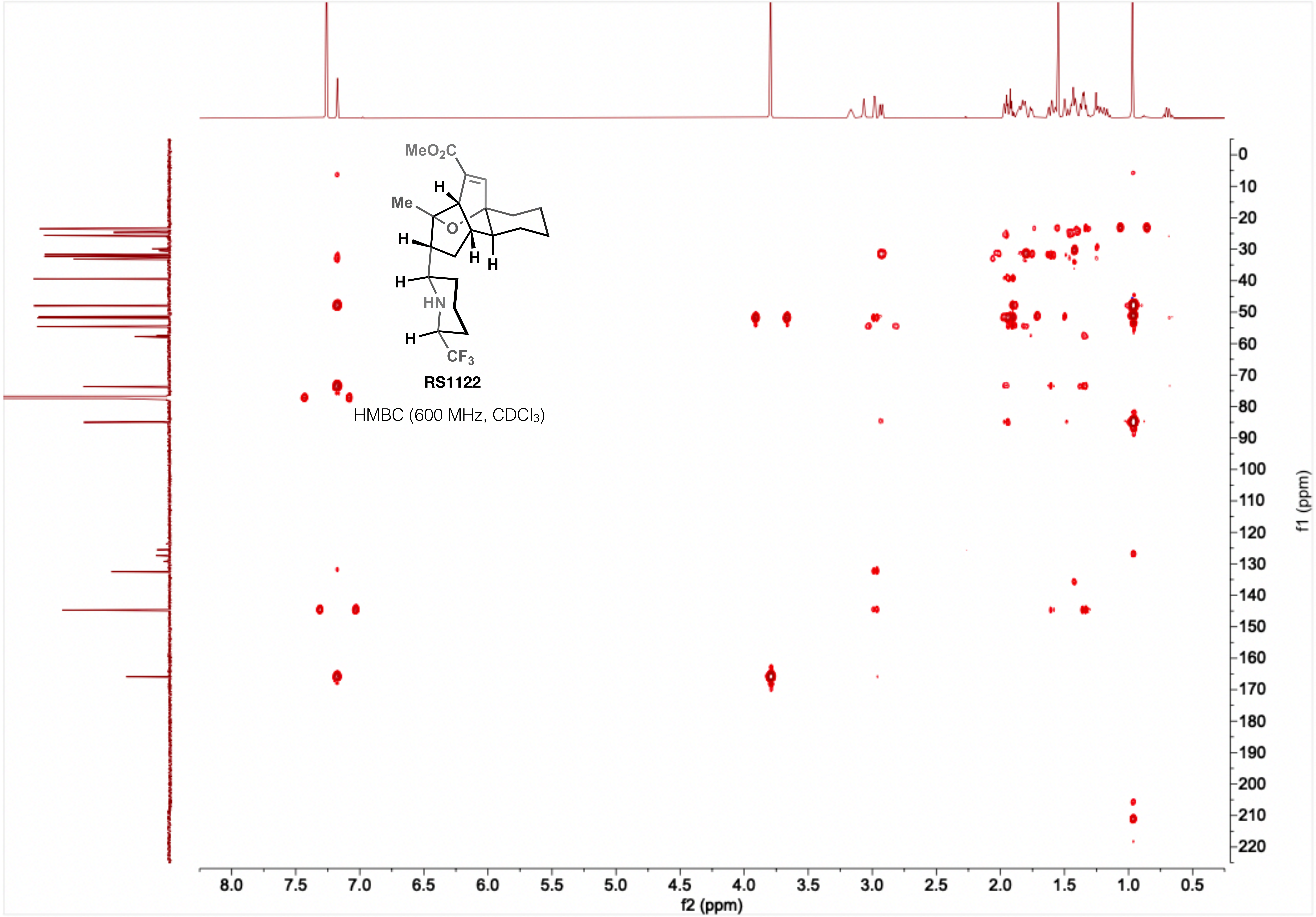
MeO2C
Me
H
O
H
H
H
H
HN
H
CF3
RS1122
HMBC (600 MHz, CDCl3)
f2 (ppm)
f1 (ppm)

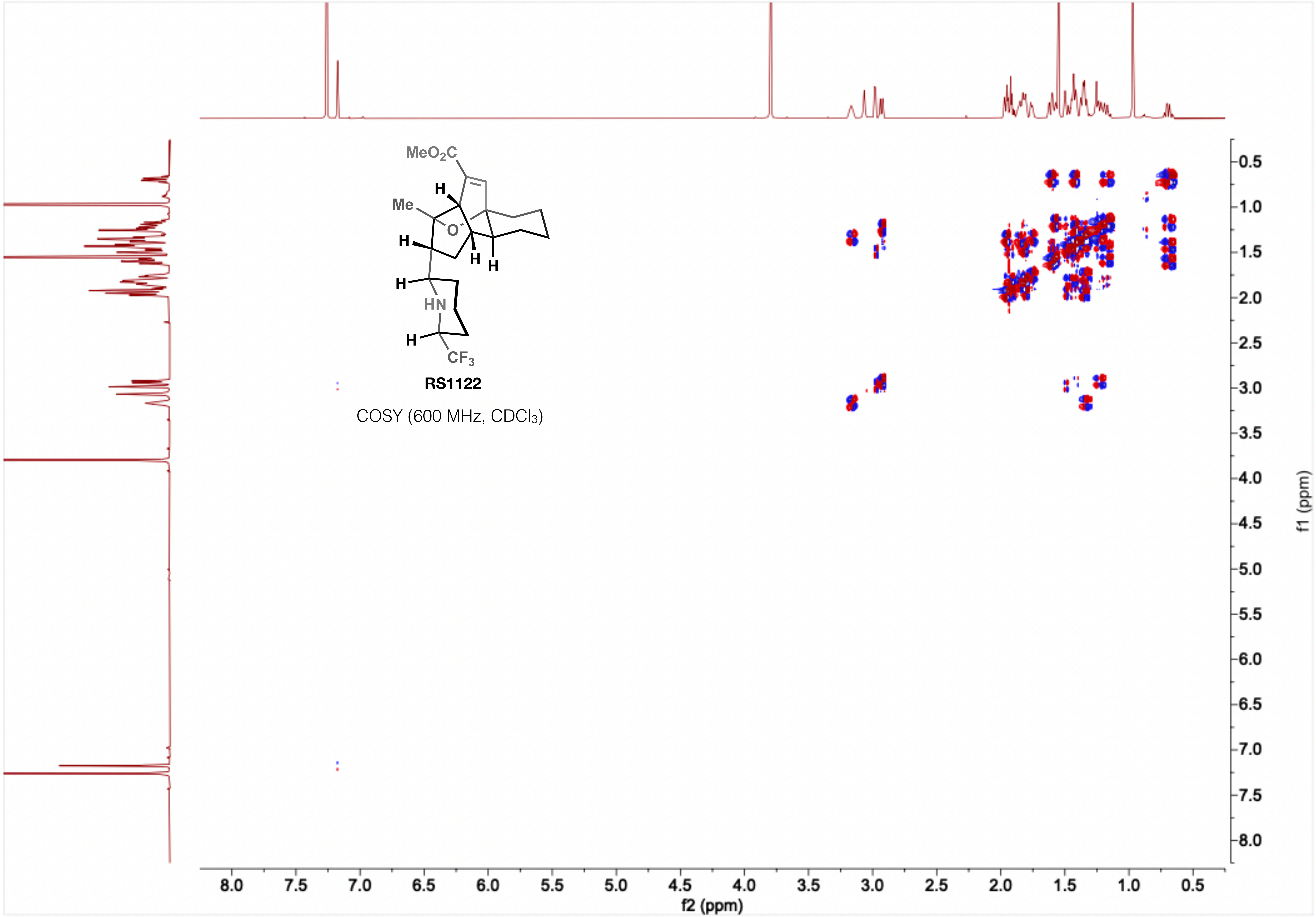
MeO2C
Me
H
O
HN
CF3
RS1122
COSY (600 MHz, CDCl3)
f2 (ppm)
f1 (ppm)

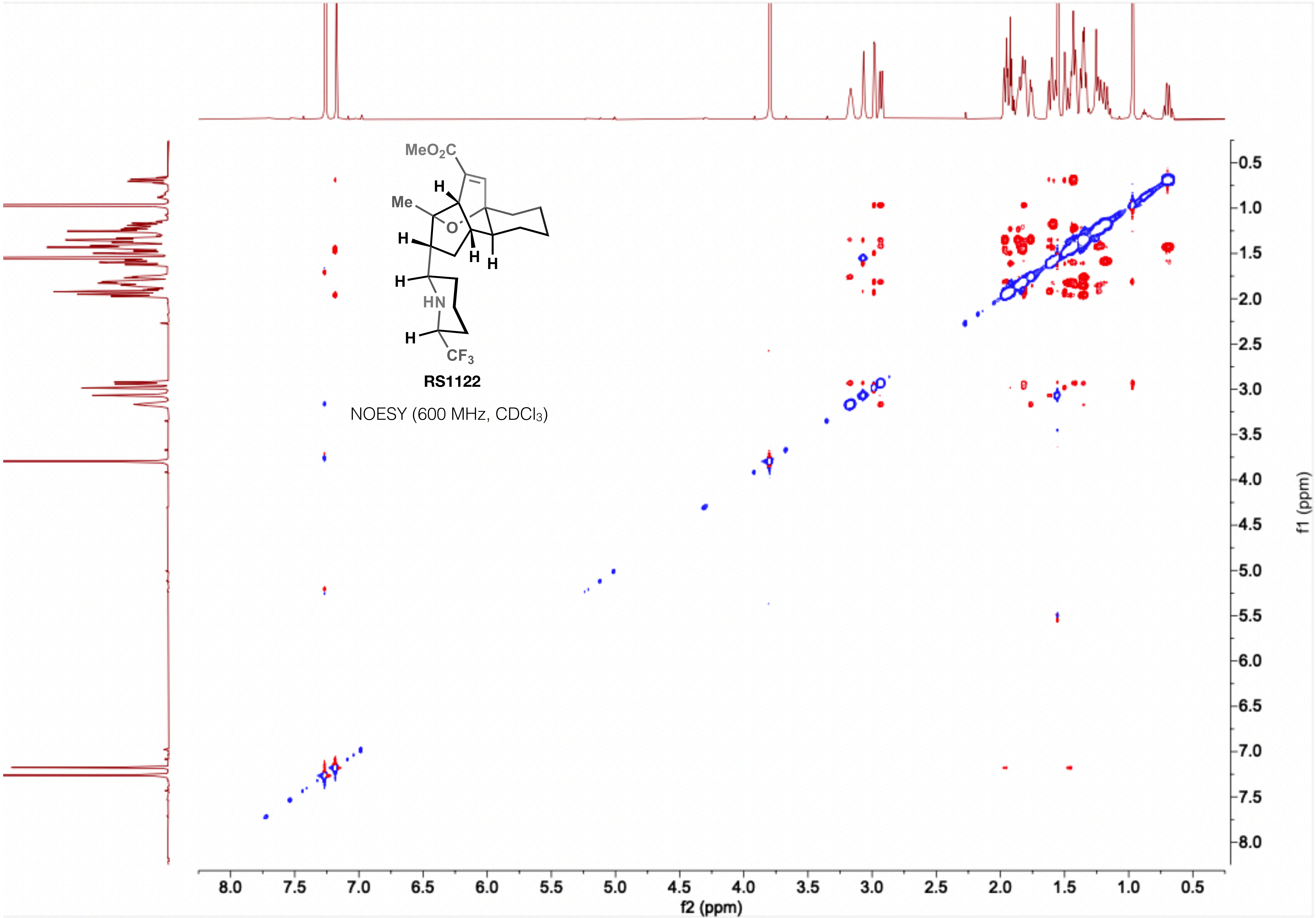

MeO2C
Me
H
O
H
H
H
H
HN
H
CF3
RS1122
NOESY (600 MHz, CDCl3)
f2 (ppm)
f1 (ppm)
8.0
7.5
7.0
6.5
6.0
5.5
5.0
4.5
4.0
3.5
3.0
2.5
2.0
1.5
1.0
0.5

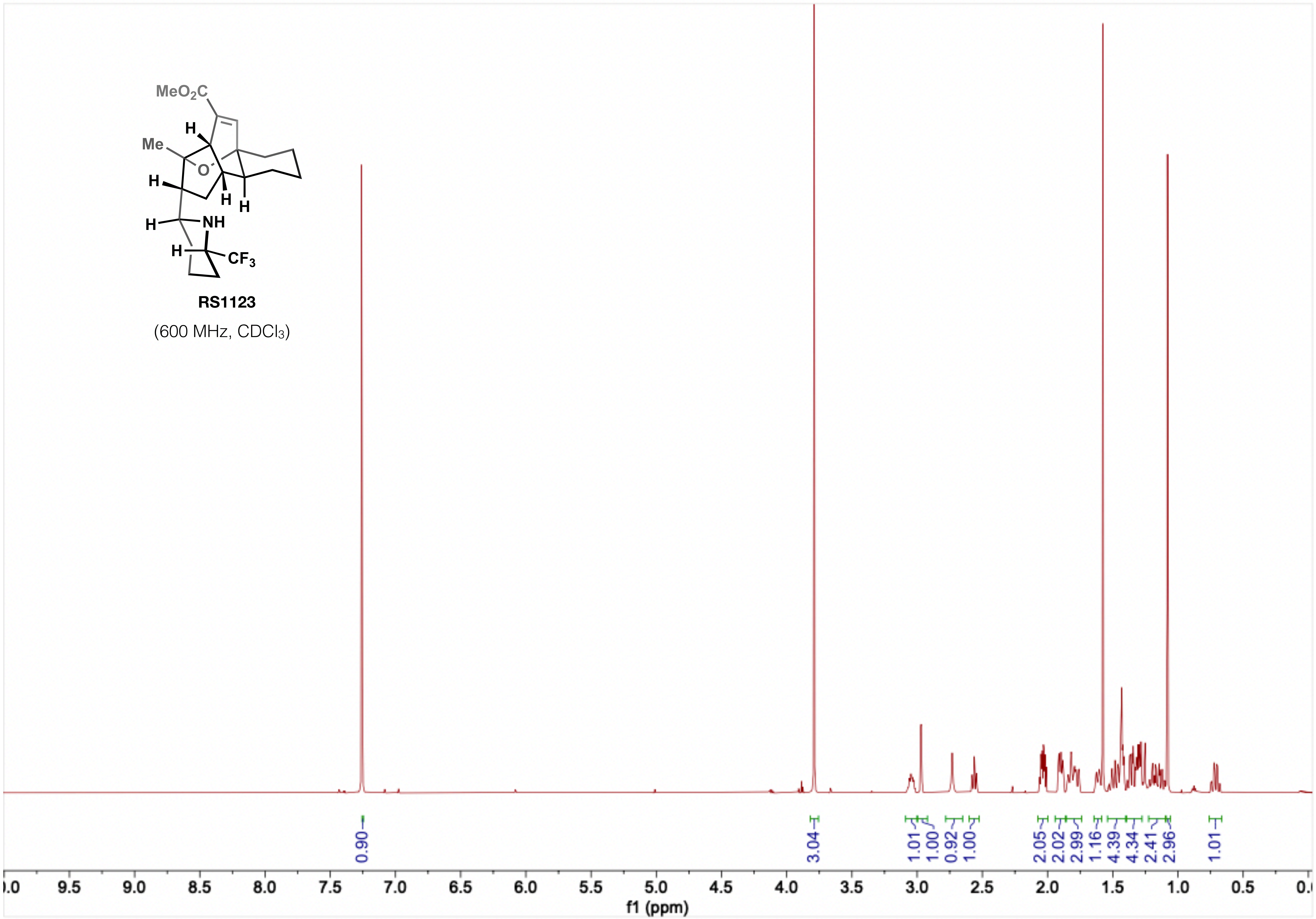
MeO$_2$C
Me
O
H
NH
CF$_3$
RS1123
(600 MHz, CDCl$_3$)
0.90
3.04
1.01
1.00
0.92
1.00
2.05
2.02
2.99
1.16
4.39
4.34
2.41
2.96
1.01
10.0 9.5 9.0 8.5 8.0 7.5 7.0 6.5 6.0 5.5 5.0 4.5 4.0 3.5 3.0 2.5 2.0 1.5 1.0 0.5 0.0
f1 (ppm)

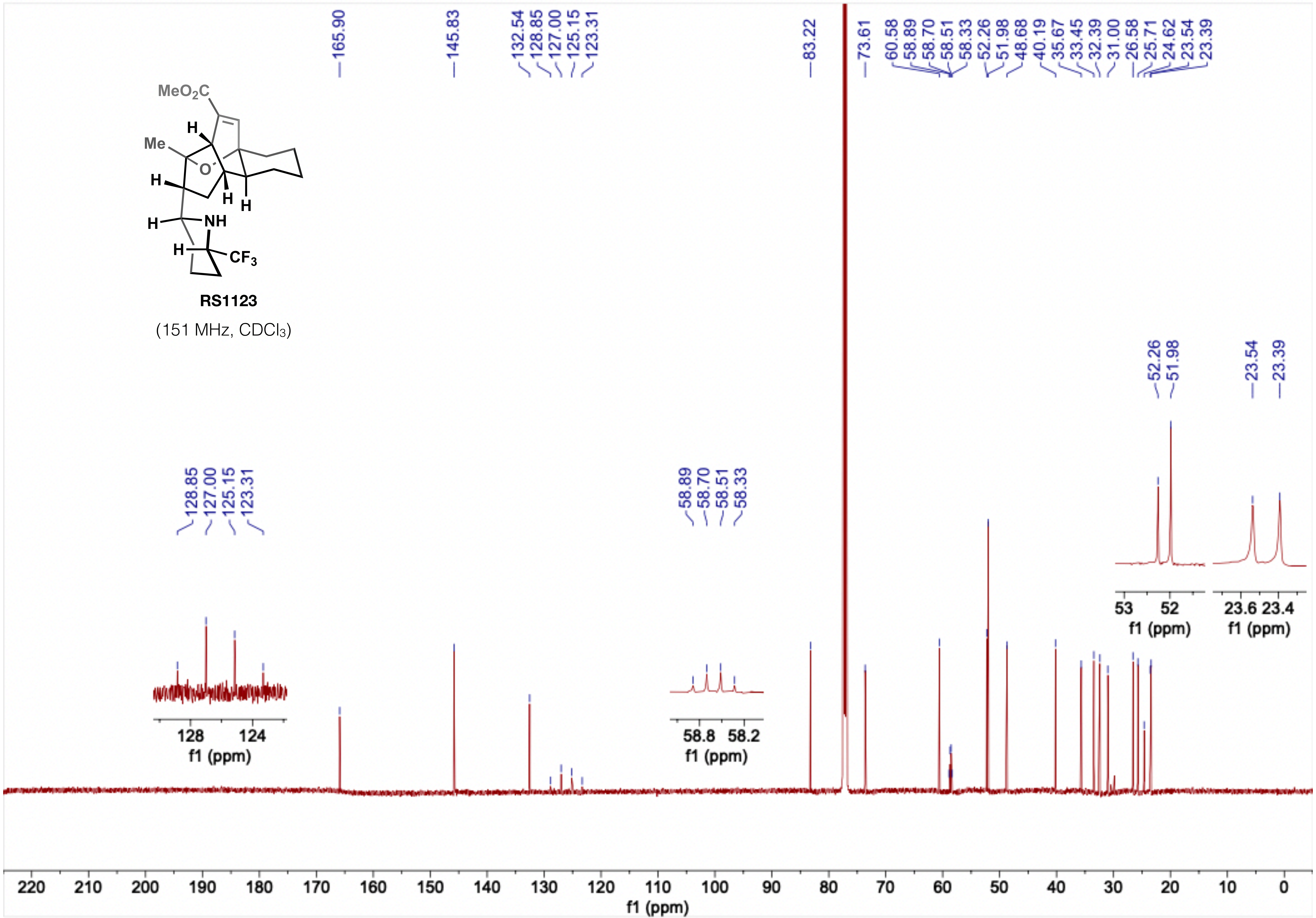
165.90
145.83
132.54
128.85
127.00
125.15
123.31
83.22
73.61
60.58
58.89
58.70
58.51
58.33
52.26
51.98
48.68
40.19
35.67
33.45
32.39
31.00
26.58
25.71
24.62
23.54
23.39
MeO2C
Me
H
O
H
H
H
H
NH
H
CF3
RS1123
(151 MHz, CDCl3)
128 124
58.8 58.2
53 52
23.6 23.4
f1 (ppm)
220 210 200 190 180 170 160 150 140 130 120 110 100 90 80 70 60 50 40 30 20 10 0
f1 (ppm)

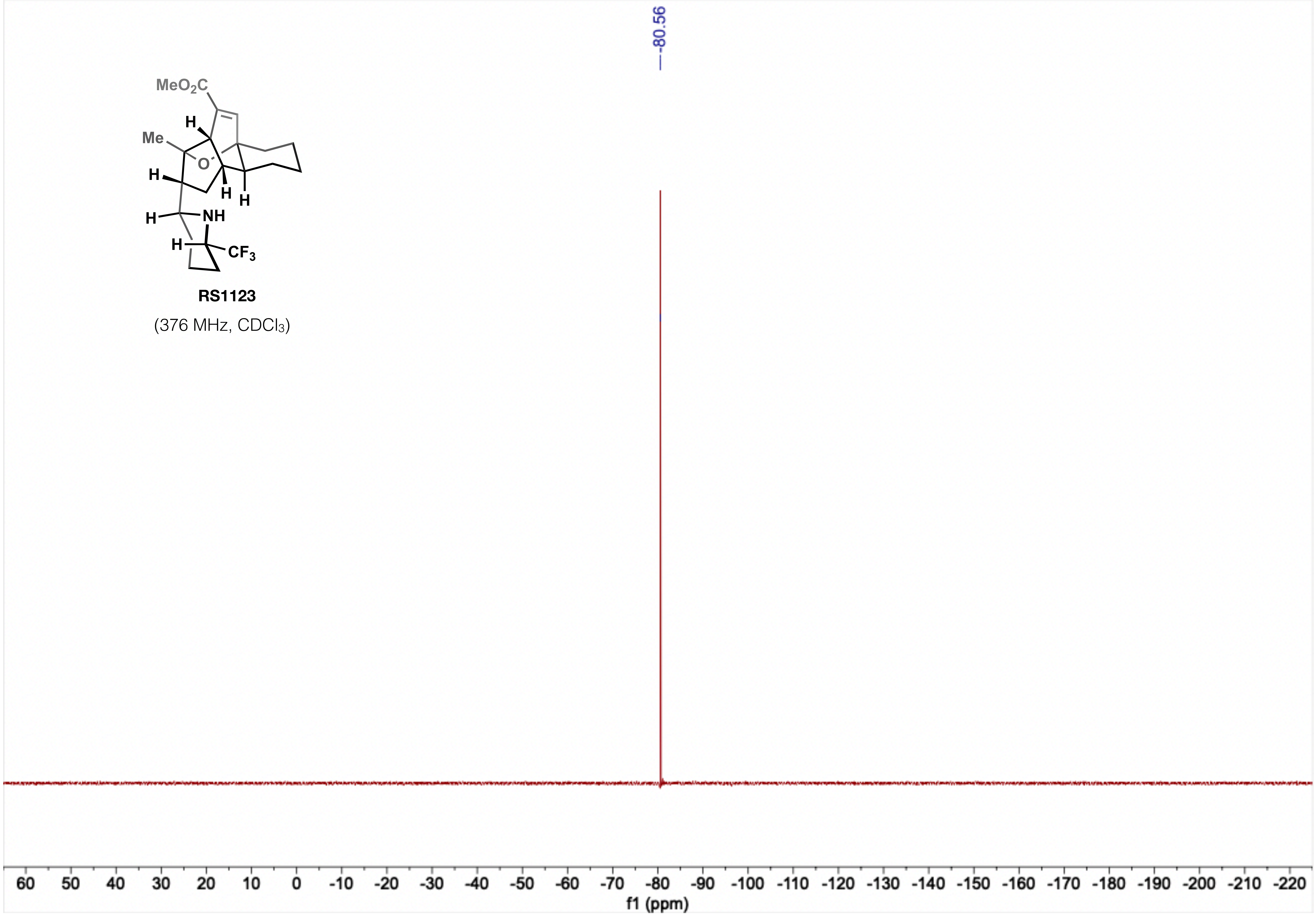

-80.56
MeO2C
Me
H
O
H
H
H
H
NH
H
CF3
RS1123
(376 MHz, CDCl3)
60 50 40 30 20 10 0 -10 -20 -30 -40 -50 -60 -70 -80 -90 -100 -110 -120 -130 -140 -150 -160 -170 -180 -190 -200 -210 -220
f1 (ppm)

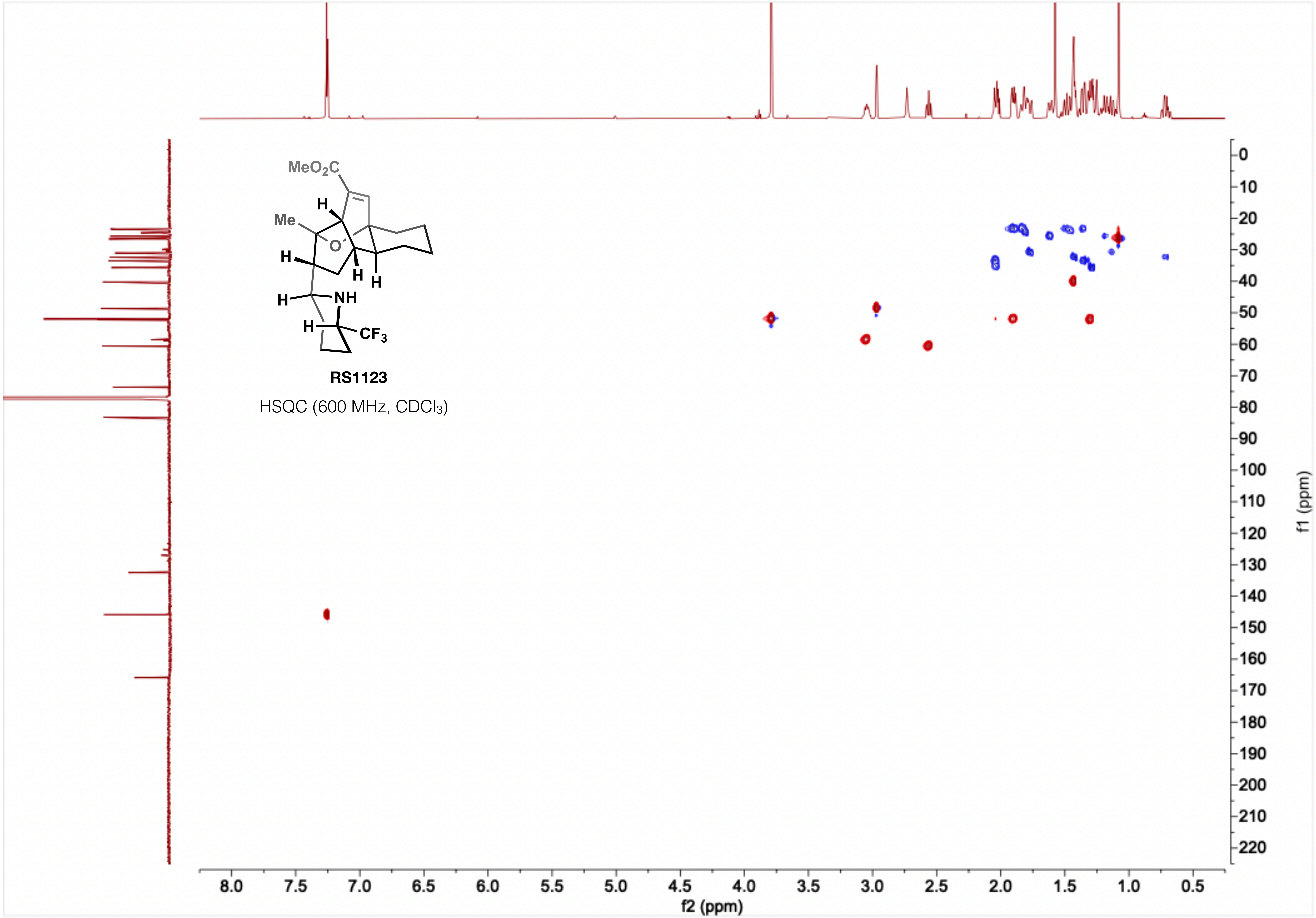

MeO2C
Me
O
H
NH
CF3
RS1123
HSQC (600 MHz, CDCl3)
f2 (ppm)
f1 (ppm)

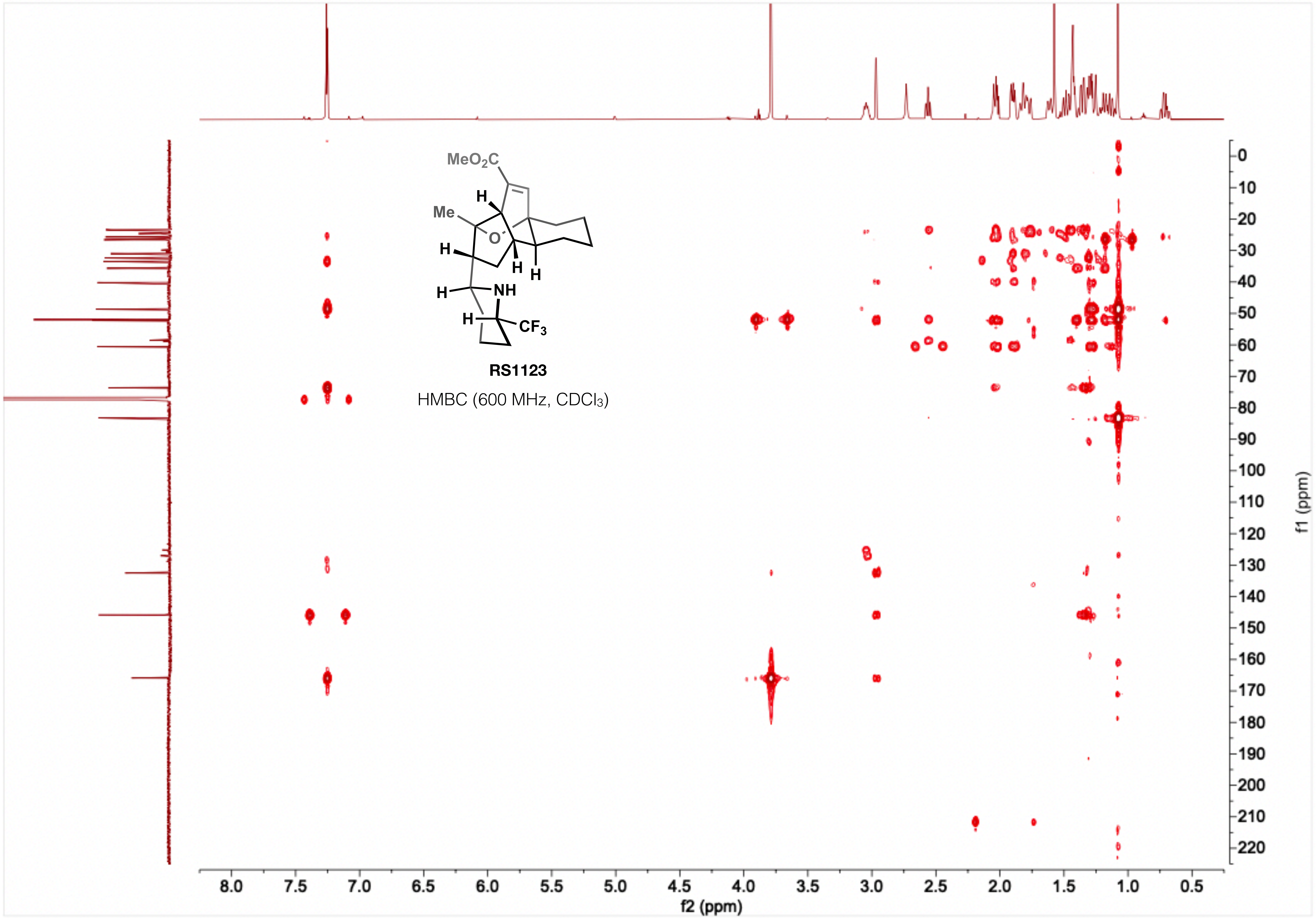
MeO2C
Me
O
H
H
H
H
H
NH
H
CF3
RS1123
HMBC (600 MHz, CDCl3)
f2 (ppm)
f1 (ppm)

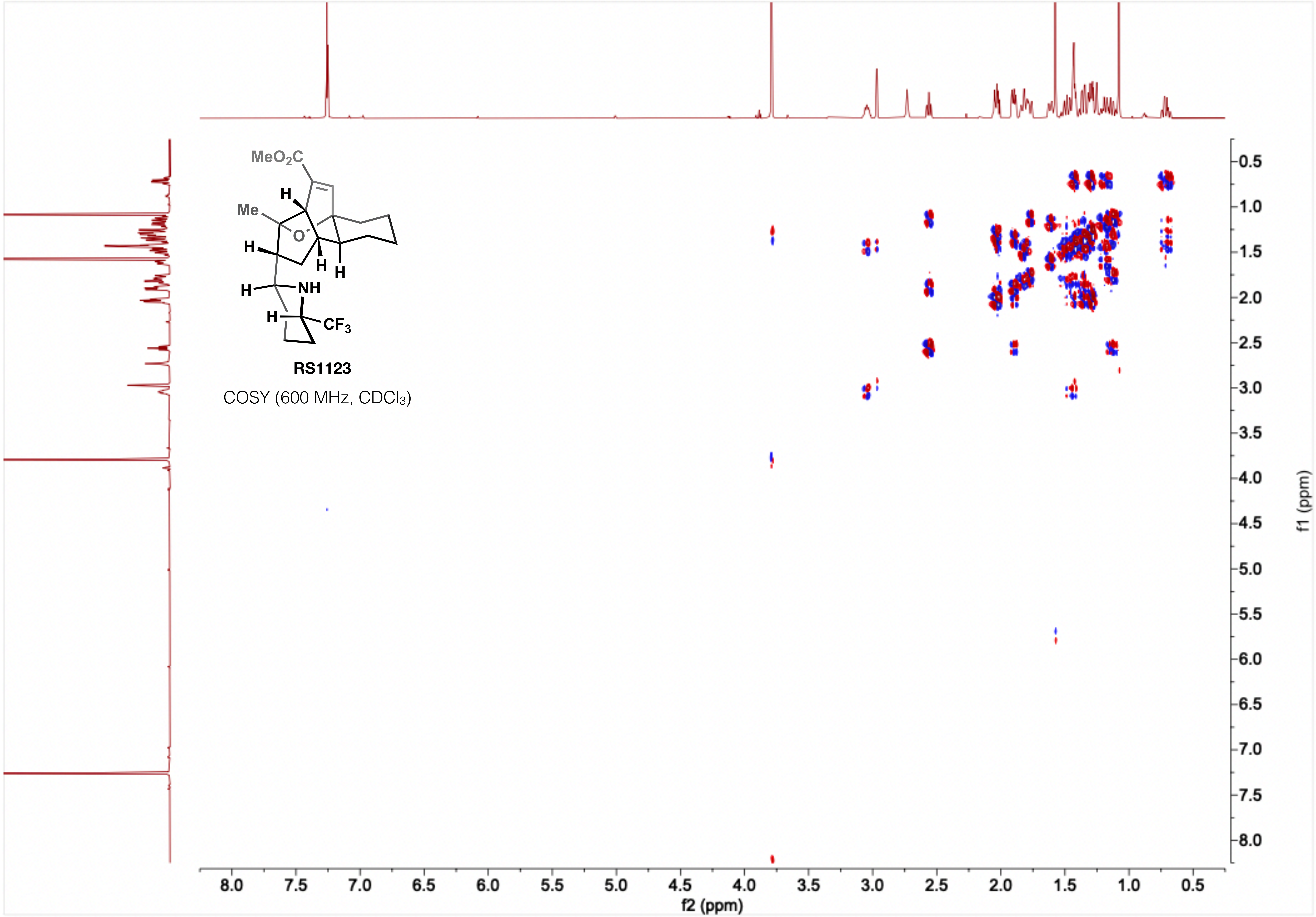
MeO2C
Me
O
H
NH
CF3
RS1123
COSY (600 MHz, CDCl3)
f2 (ppm)
f1 (ppm)
8.0
7.5
7.0
6.5
6.0
5.5
5.0
4.5
4.0
3.5
3.0
2.5
2.0
1.5
1.0
0.5

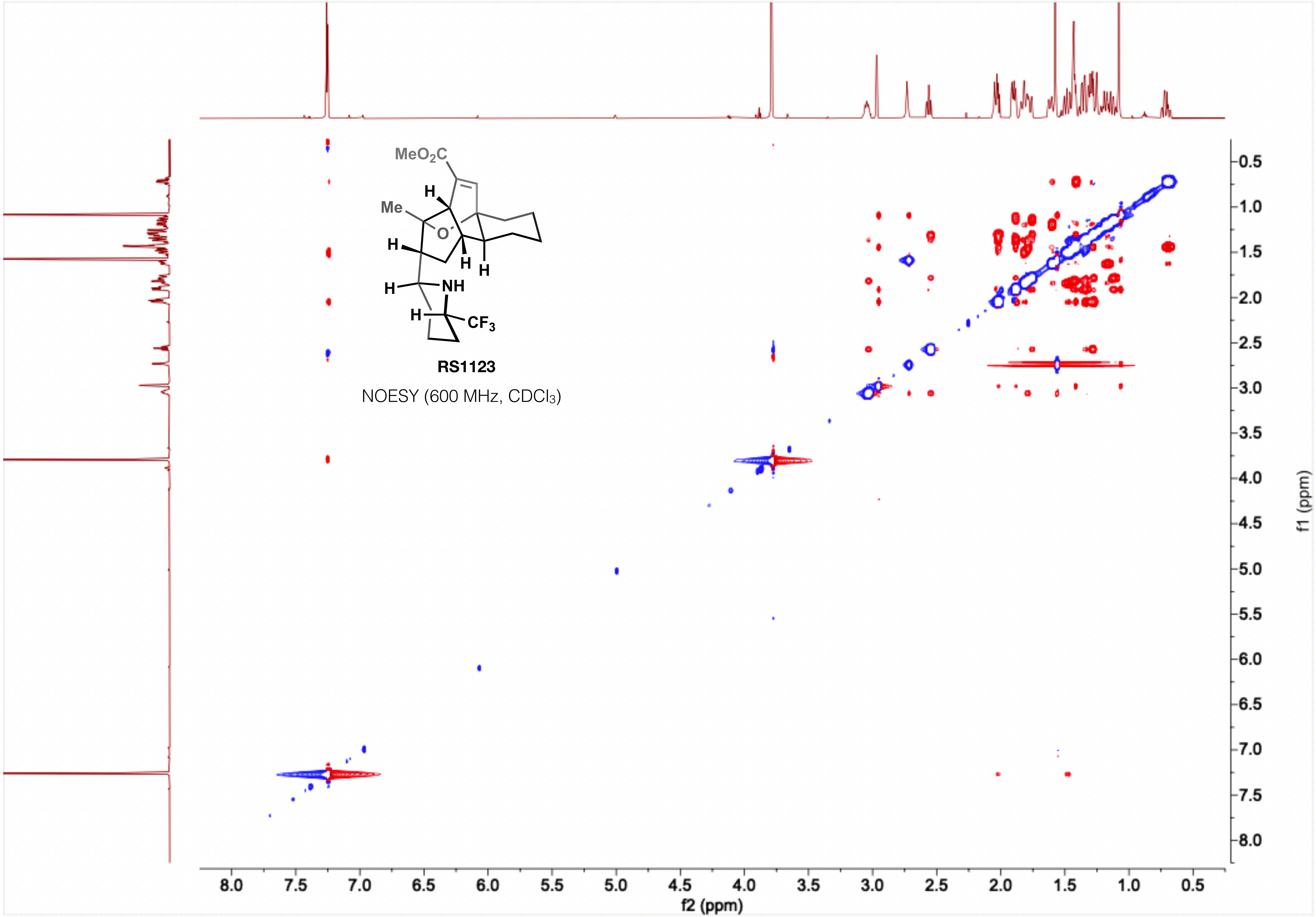
MeO2C
Me
O
H
NH
CF3
RS1123
NOESY (600 MHz, CDCl3)
f2 (ppm)
f1 (ppm)

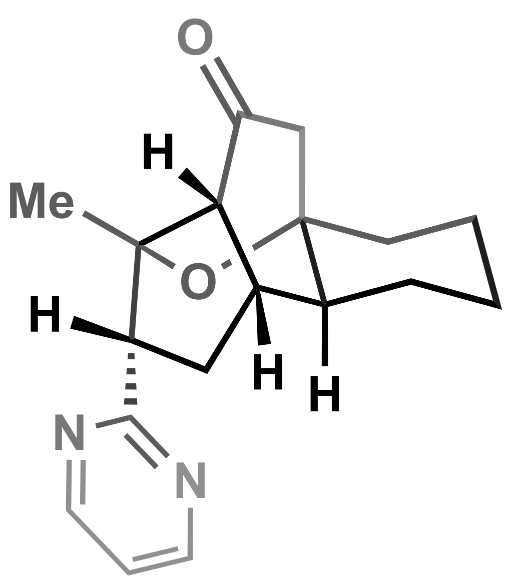

7h

(600 MHz, $CDCl_3$)

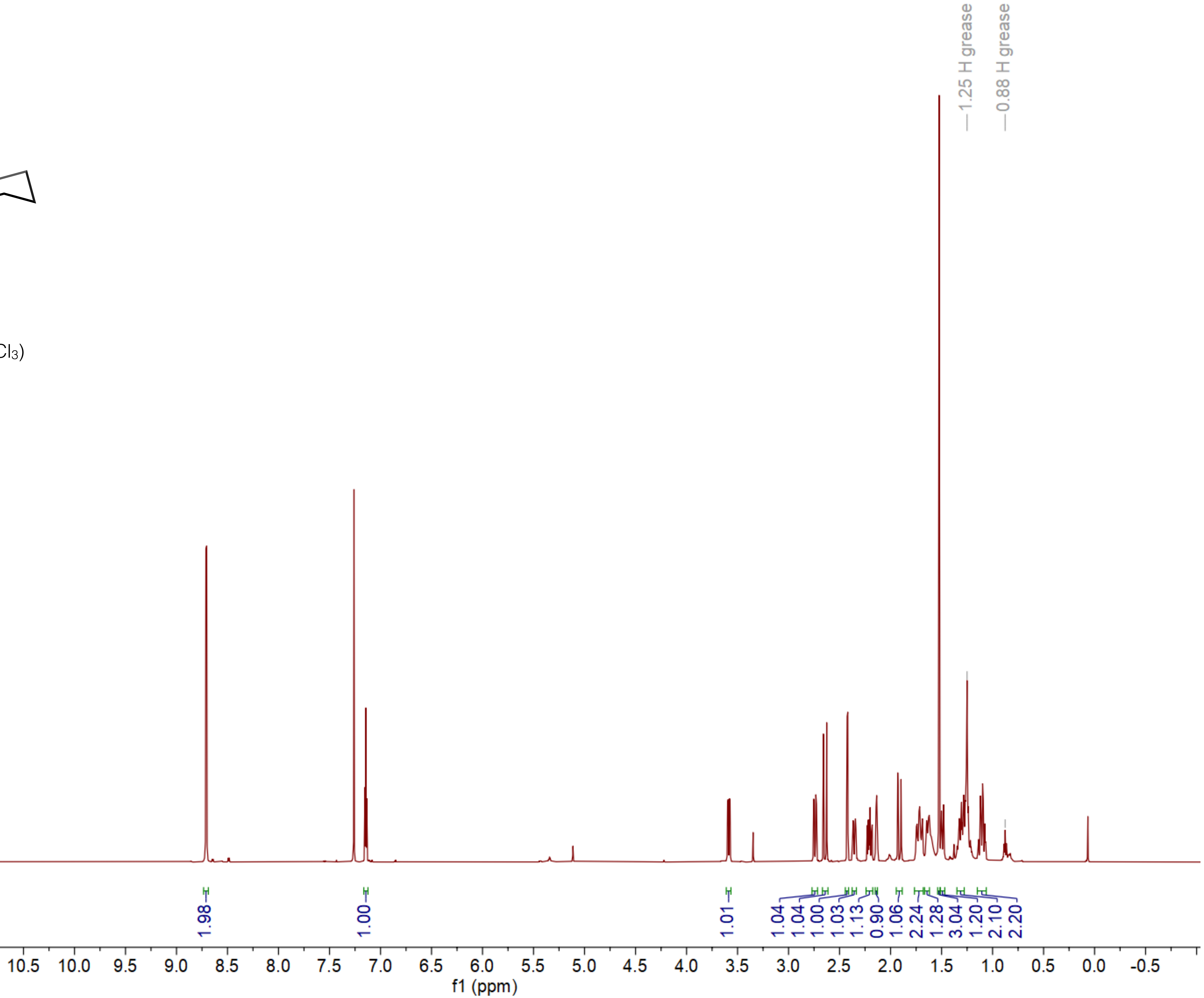

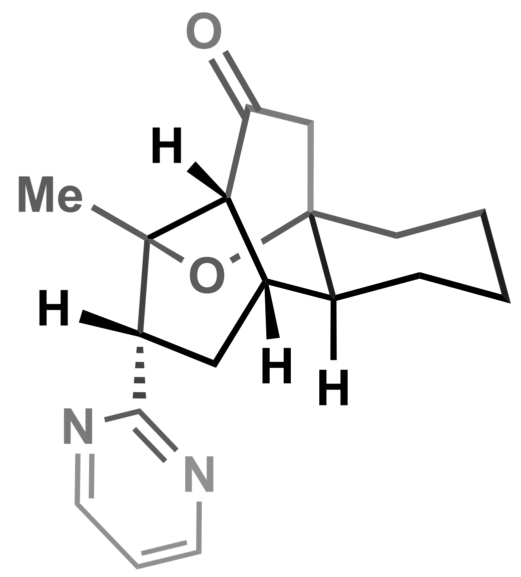

7h

(151 MHz, $CDCl_3$)

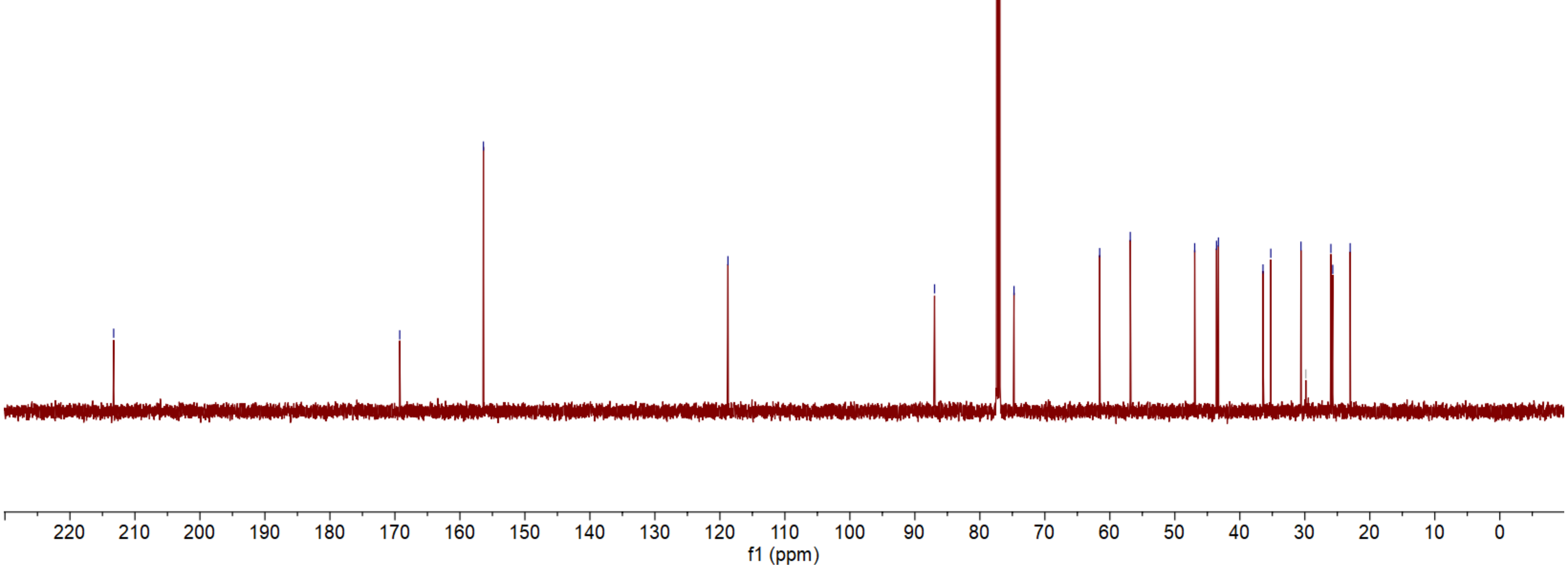

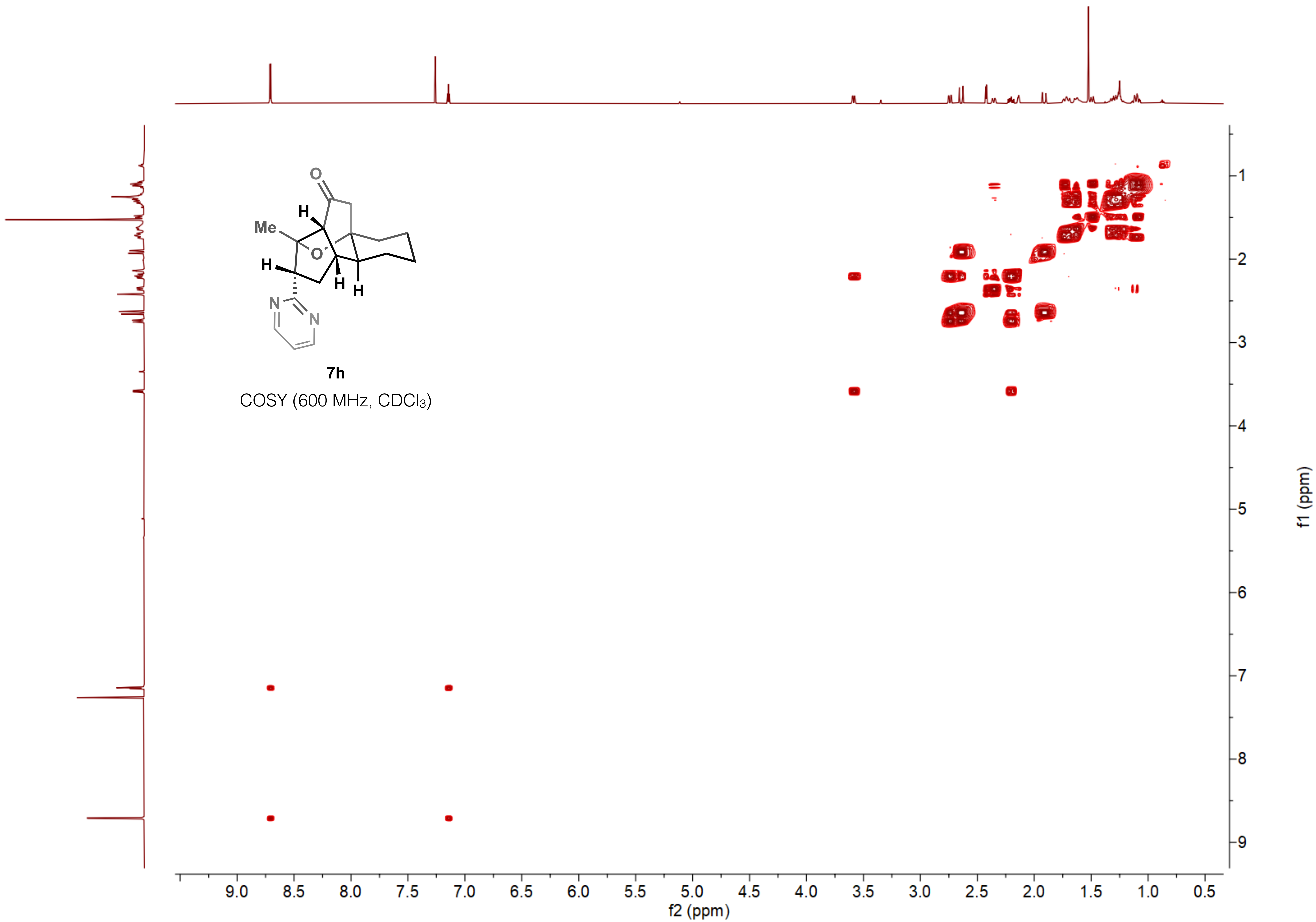

7h

COSY (600 MHz, $CDCl_3$)

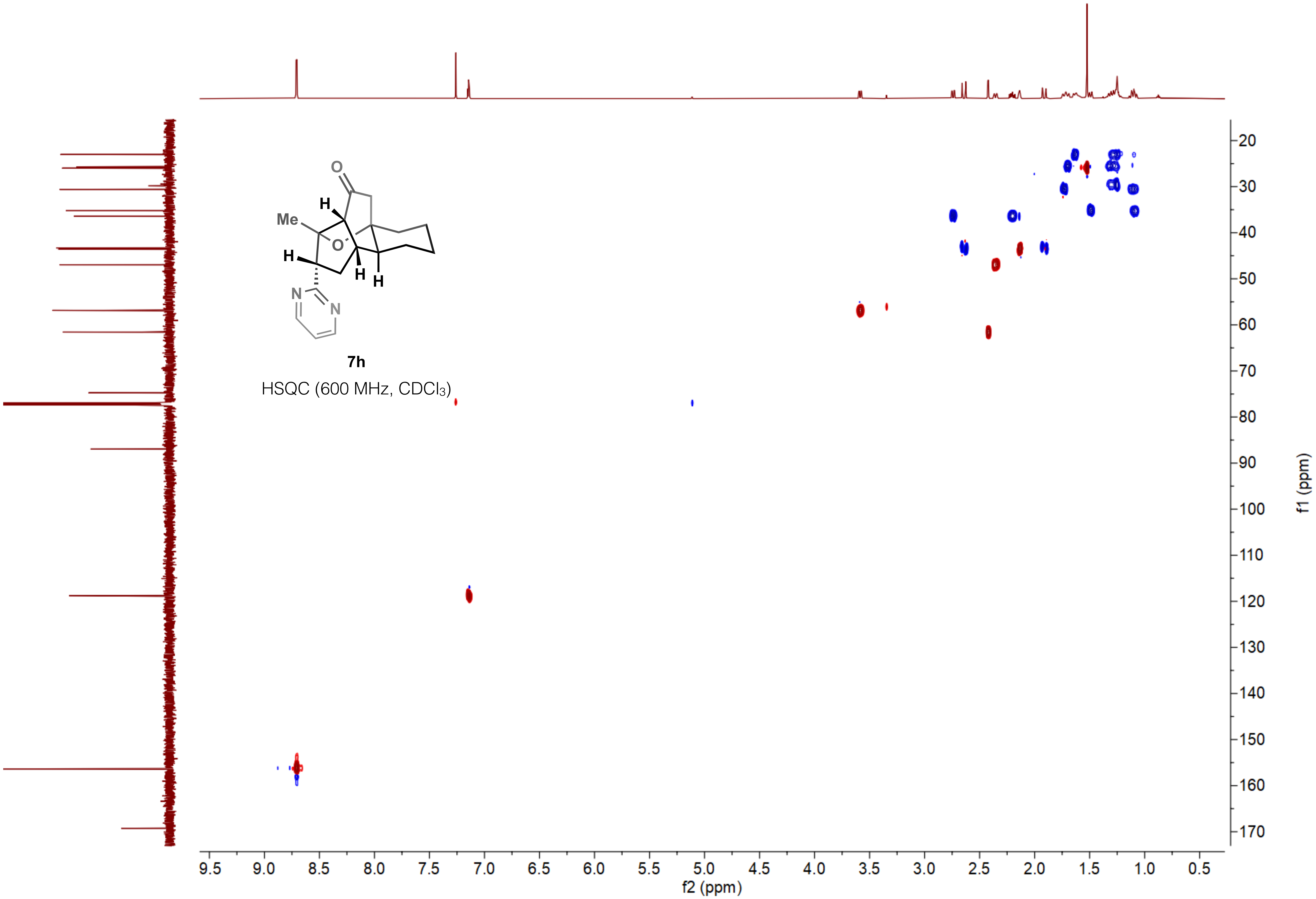

7h

HSQC (600 MHz, $CDCl_3$)

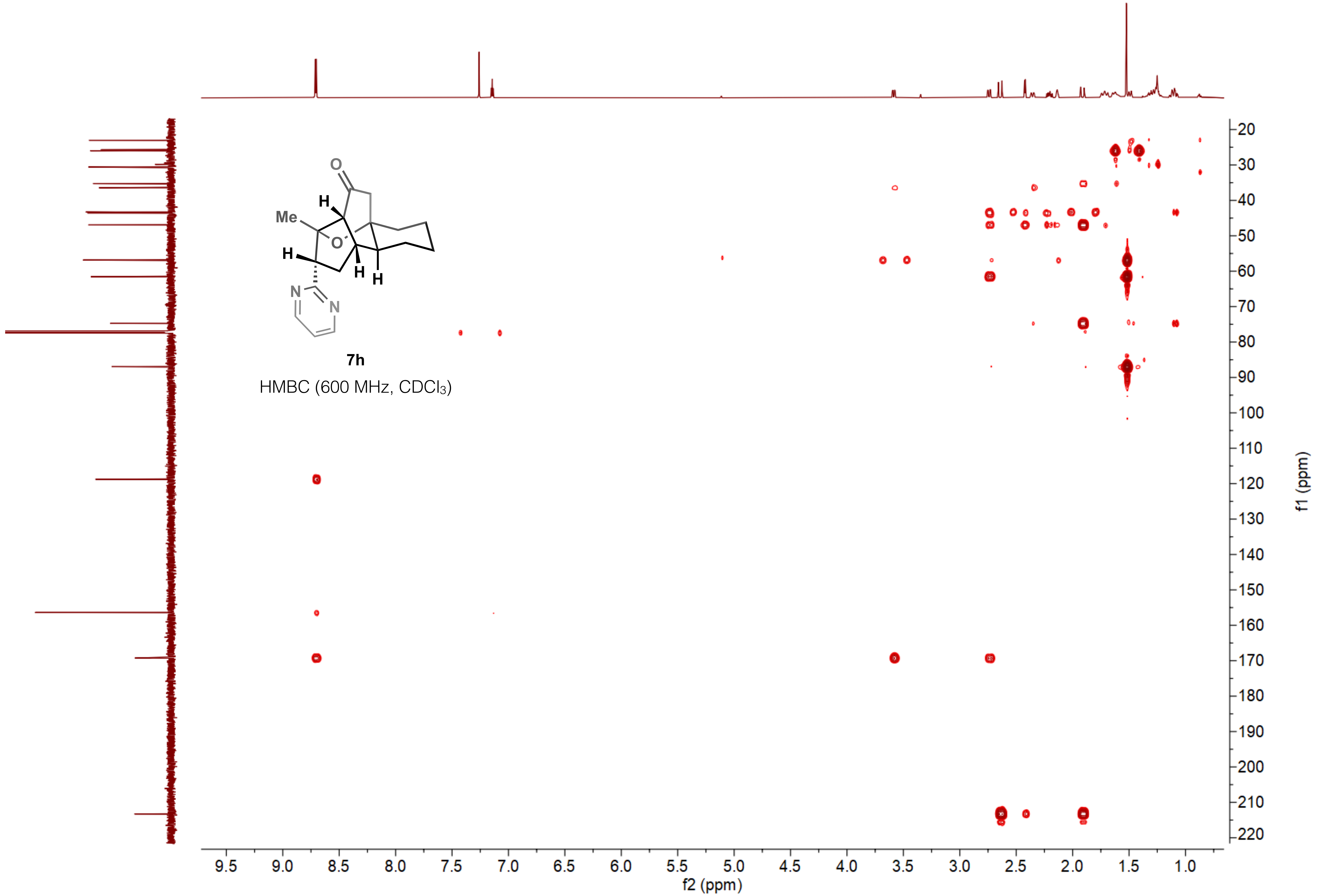
O
H
Me
O
H
H
H
N
N
7h
HMBC (600 MHz, CDCl3)
f2 (ppm)
f1 (ppm)

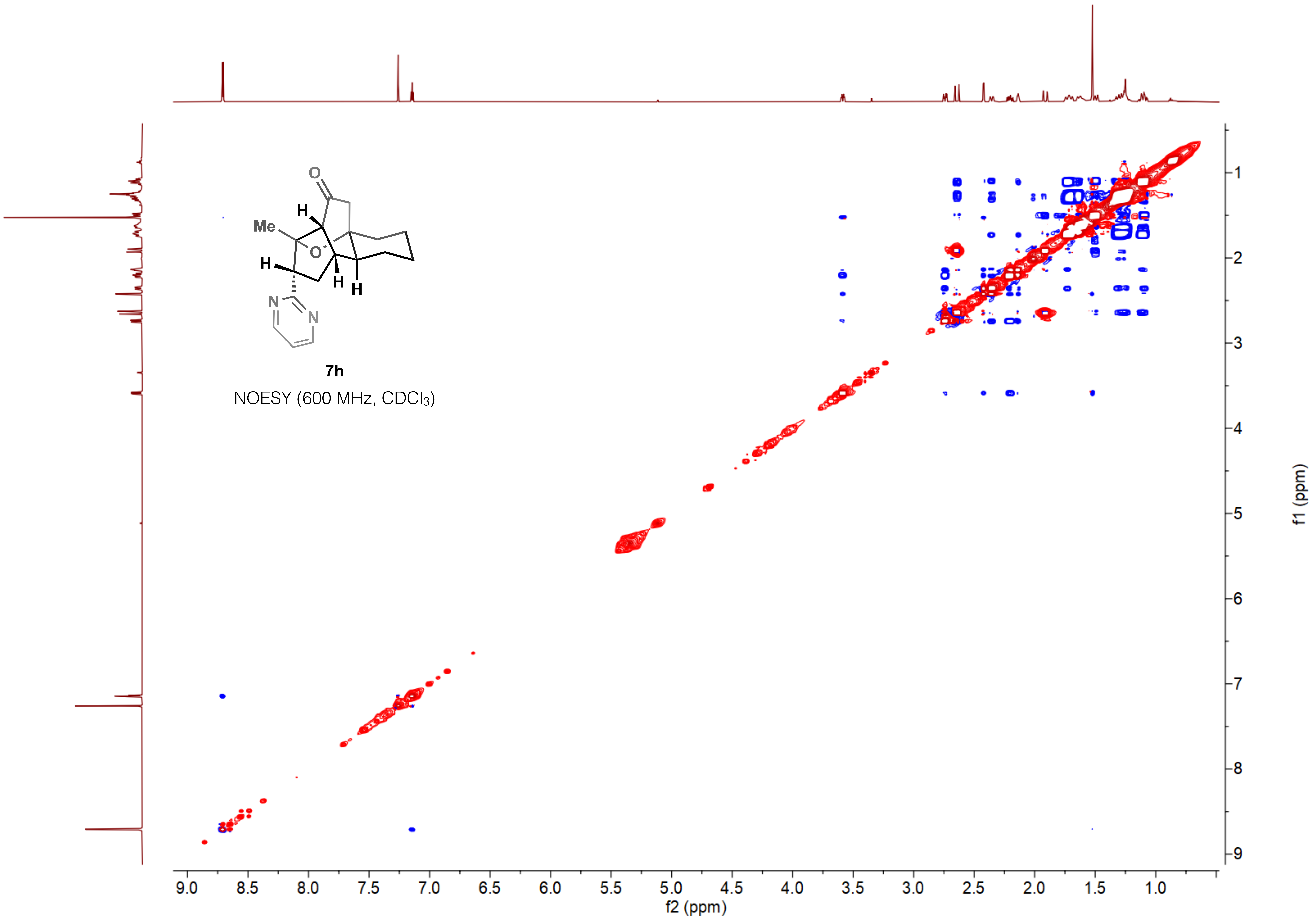
O
Me
H
O
H
H
H
N
N
7h
NOESY (600 MHz, CDCl3)
f2 (ppm)
f1 (ppm)
9.0
8.5
8.0
7.5
7.0
6.5
6.0
5.5
5.0
4.5
4.0
3.5
3.0
2.5
2.0
1.5
1.0
1
2
3
4
5
6
7
8
9

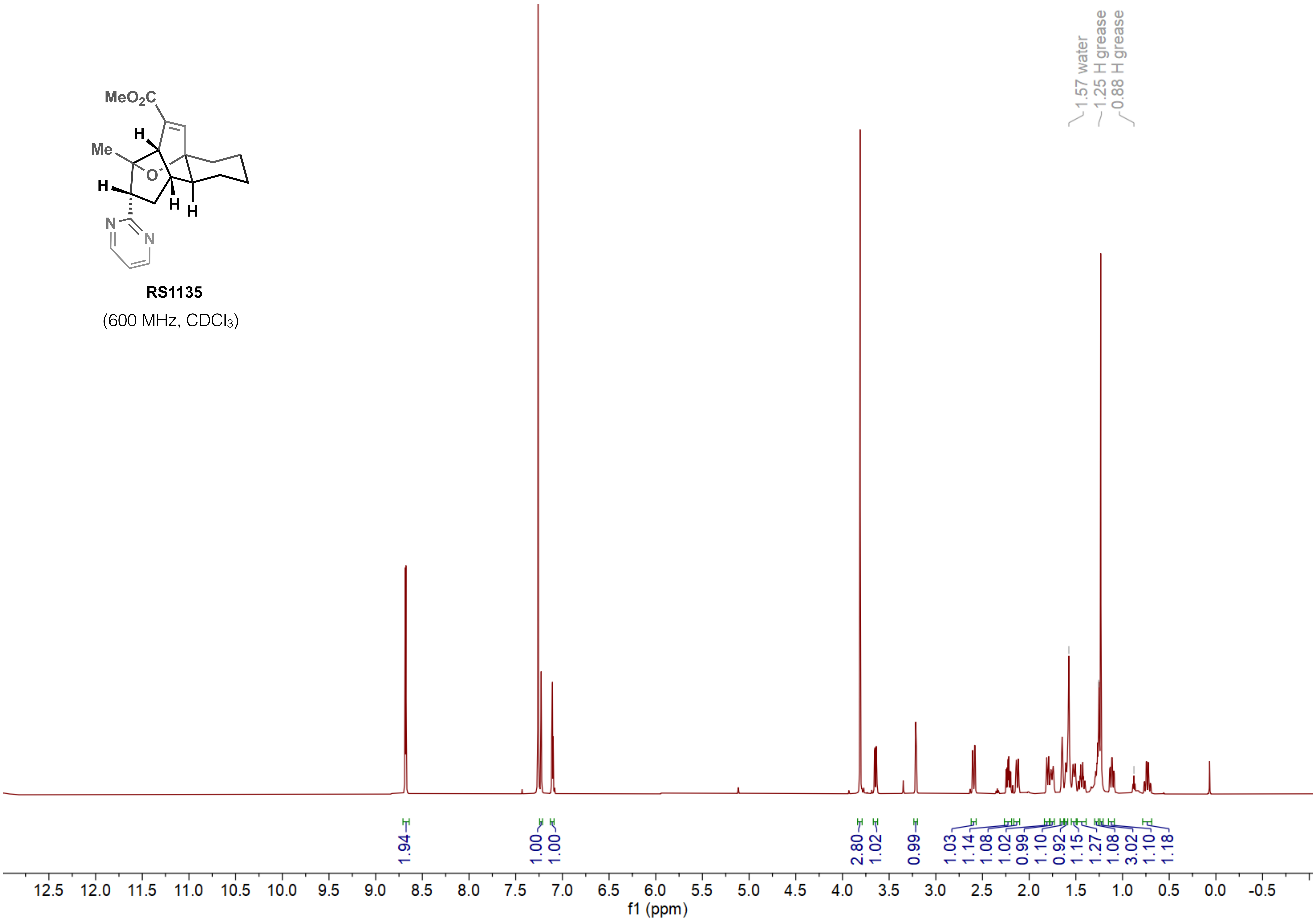

MeO2C
Me
H
O
H
H
H
N
N
RS1135
(600 MHz, CDCl3)
1.57 water
1.25 H grease
0.88 H grease
1.94
1.00
1.00
2.80
1.02
0.99
1.03
1.14
1.08
1.02
0.99
1.10
0.92
1.15
1.27
1.08
3.02
1.10
1.18
12.5
12.0
11.5
11.0
10.5
10.0
9.5
9.0
8.5
8.0
7.5
7.0
6.5
6.0
5.5
5.0
4.5
4.0
3.5
3.0
2.5
2.0
1.5
1.0
0.5
0.0
-0.5
f1 (ppm)

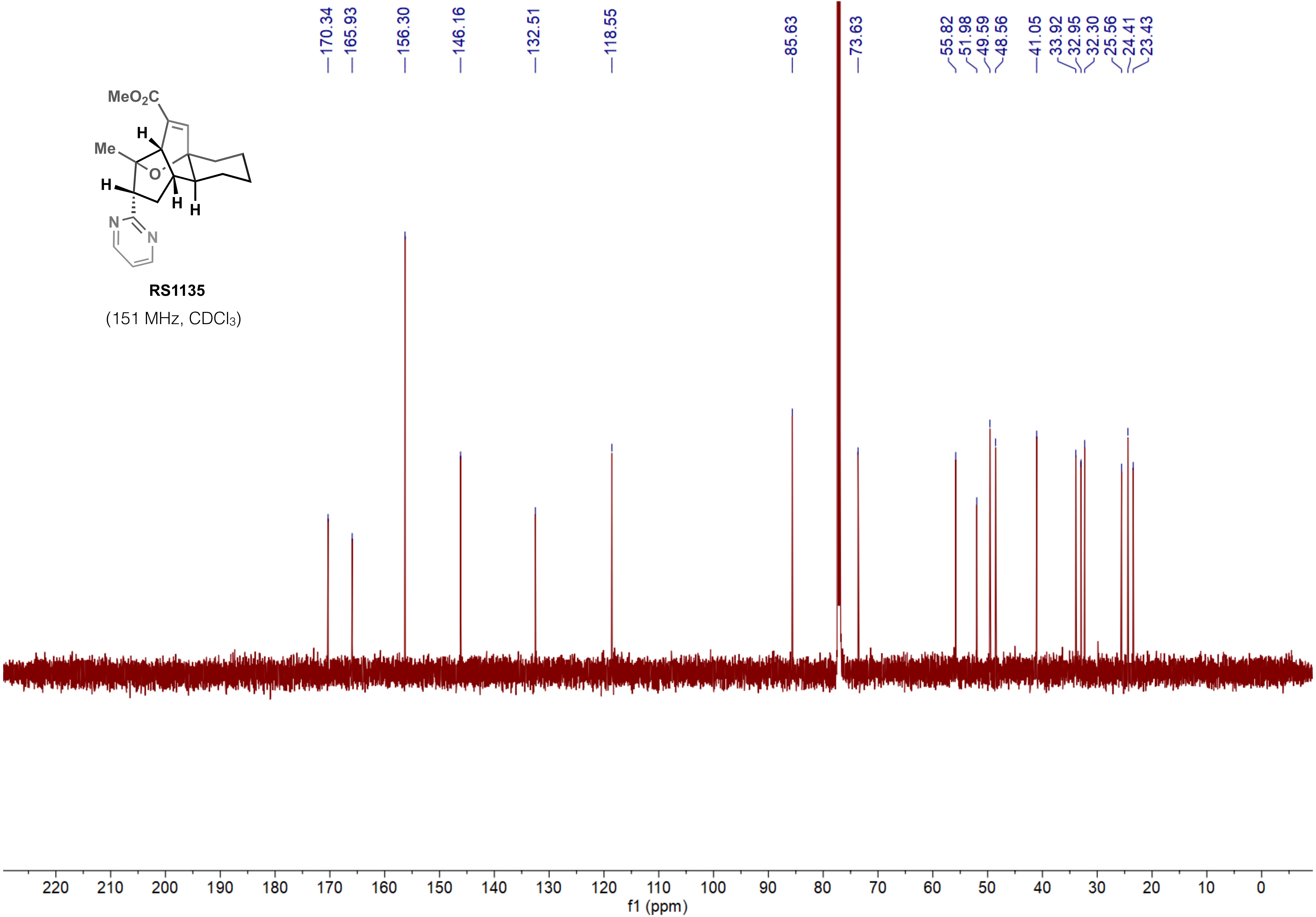
170.34
165.93
156.30
146.16
132.51
118.55
85.63
73.63
55.82
51.98
49.59
48.56
41.05
33.92
32.95
32.30
25.56
24.41
23.43
MeO$_2$C
H
Me
O
H
H
H
N
N
RS1135
(151 MHz, $CDCl_3$)
220
210
200
190
180
170
160
150
140
130
120
110
100
90
80
70
60
50
40
30
20
10
0
f1 (ppm)

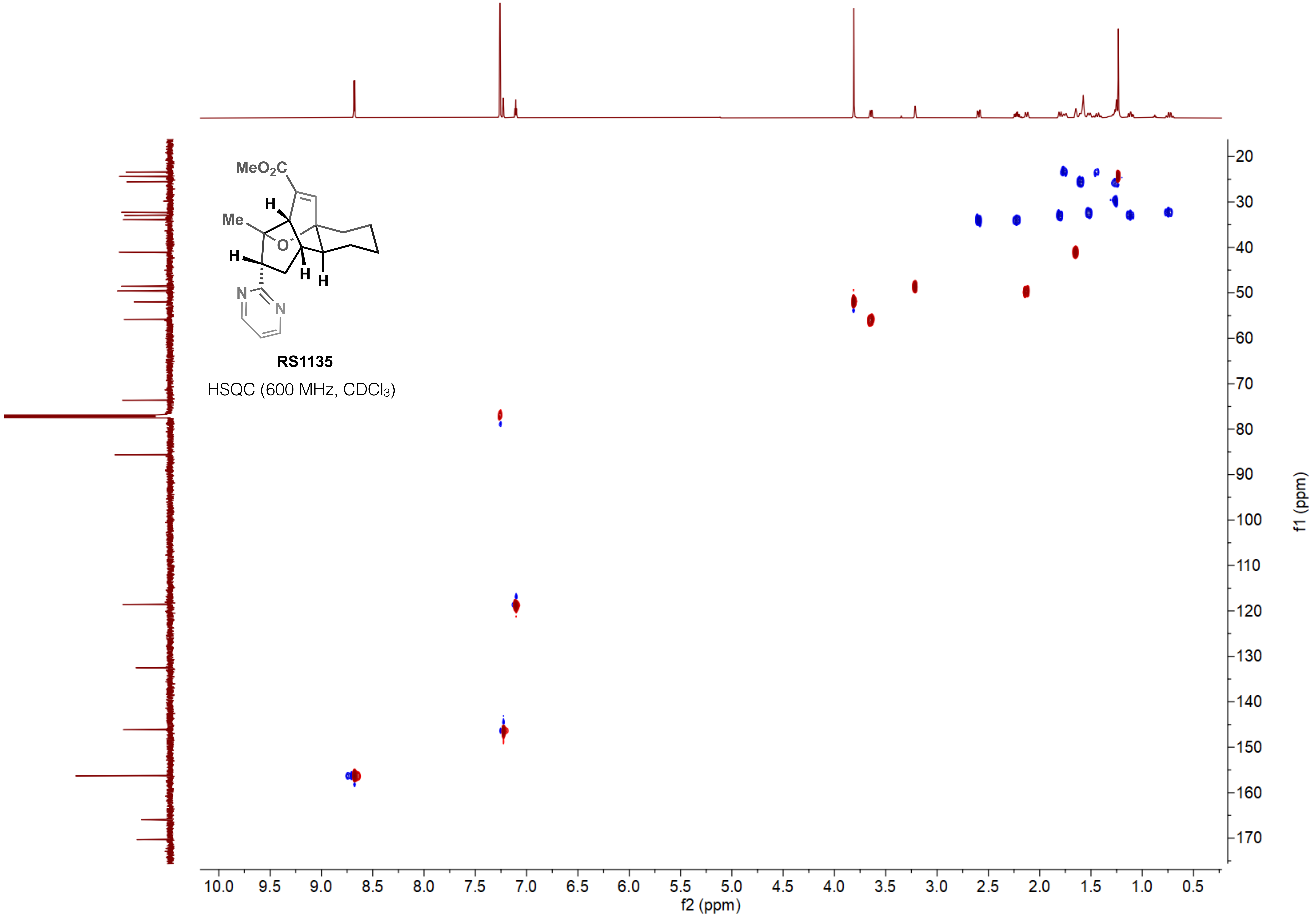

RS1135

HSQC (600 MHz, $CDCl_3$)

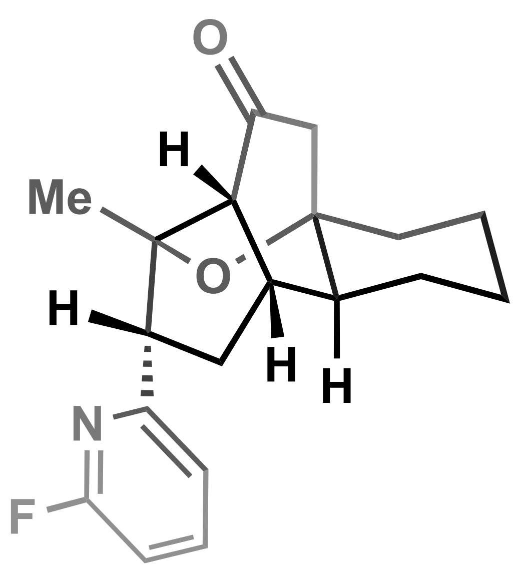

7i

(600 MHz, $CDCl_3$)

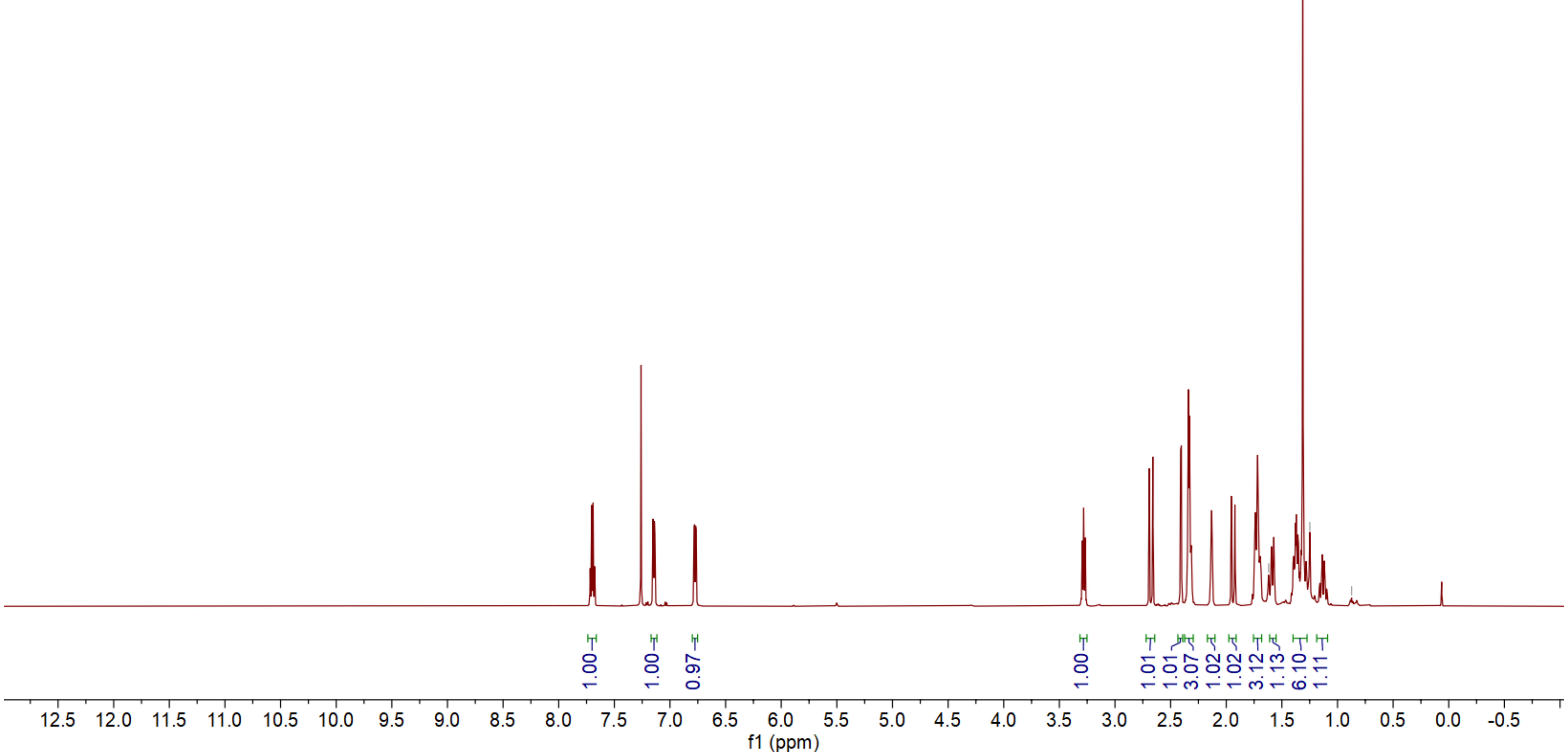

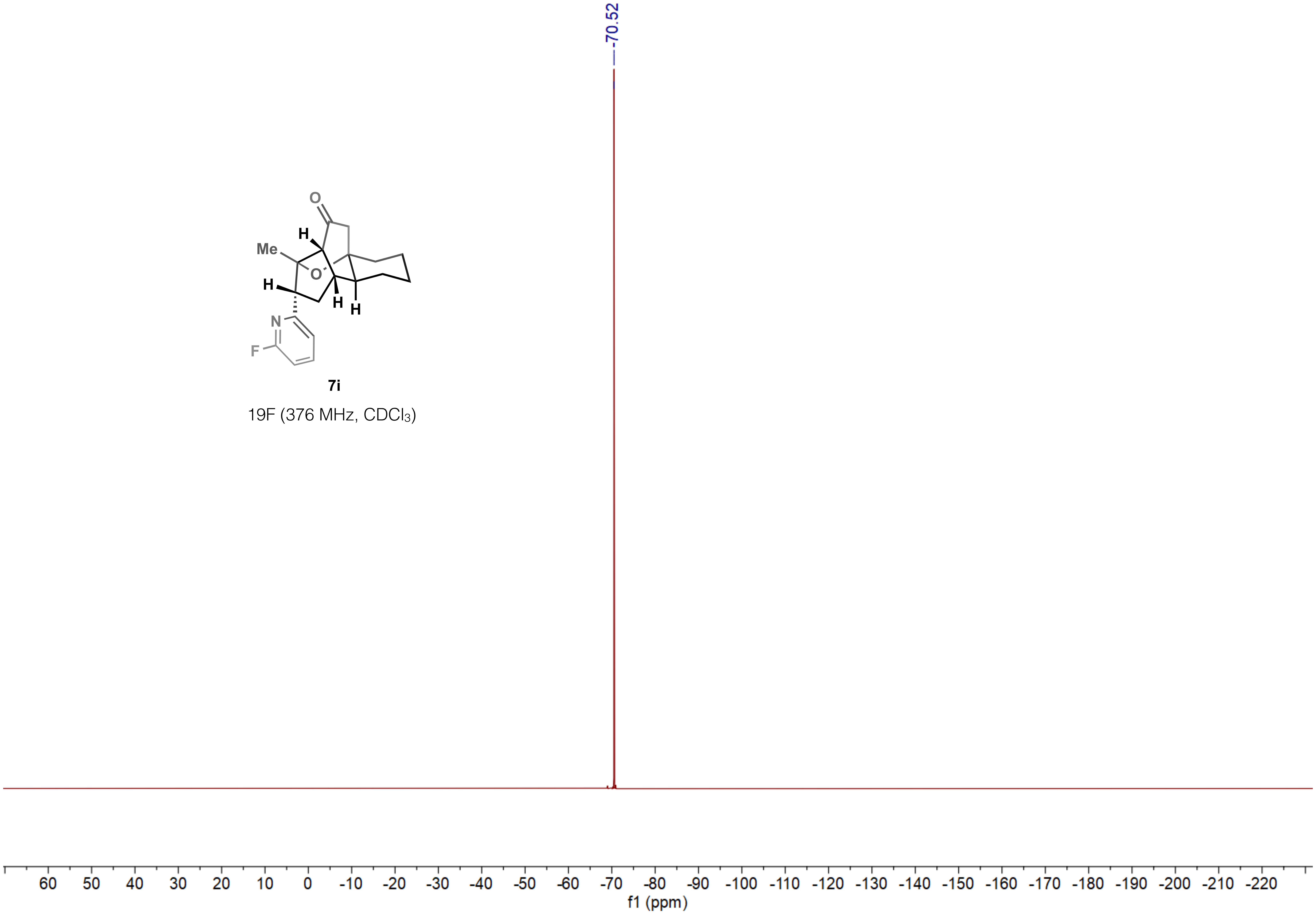

7i

19F (376 MHz, CDCl3)

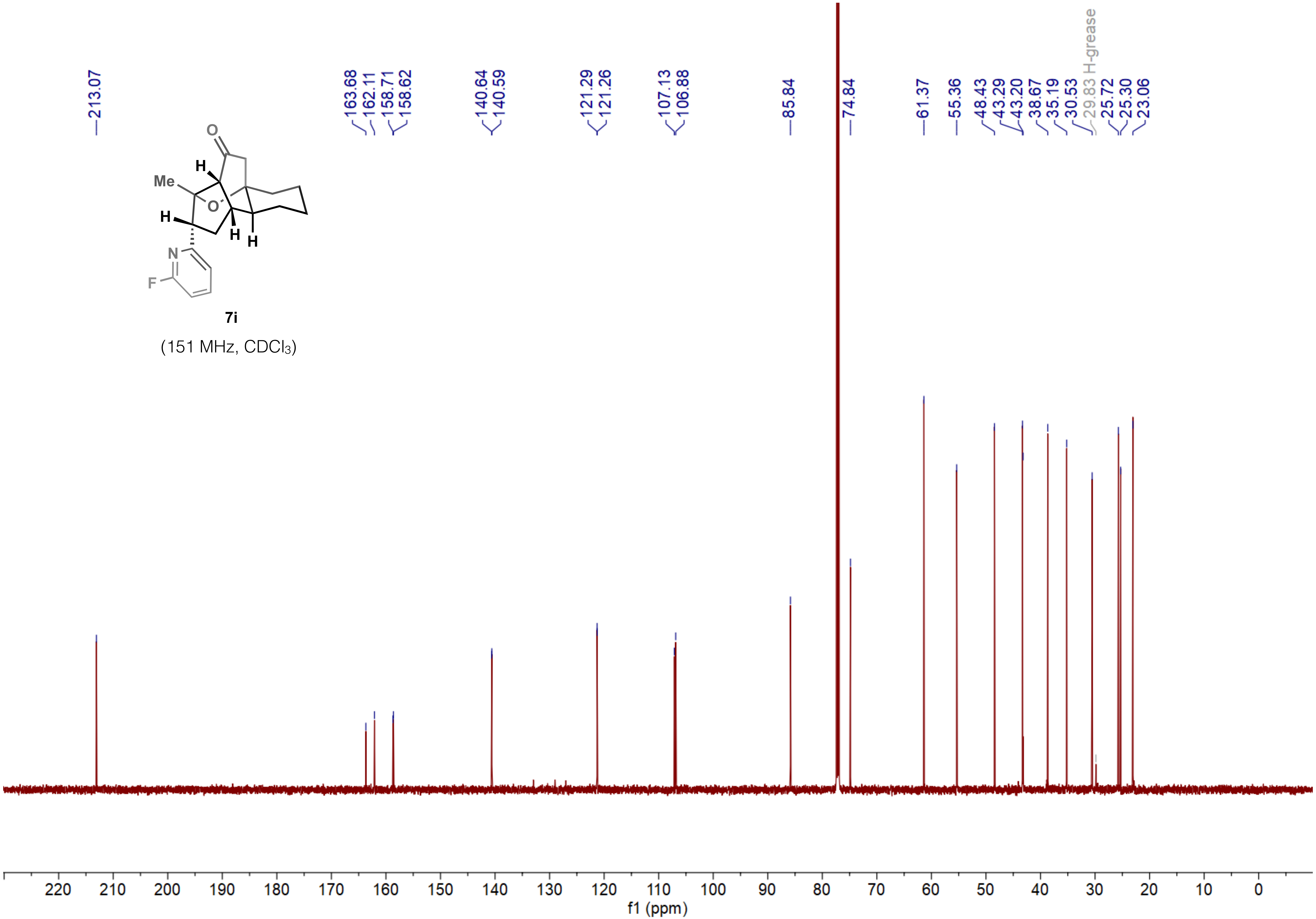
213.07
163.68
162.11
158.71
158.62
140.64
140.59
121.29
121.26
107.13
106.88
85.84
74.84
61.37
55.36
48.43
43.29
43.20
38.67
35.19
30.53
29.83 H-grease
25.72
25.30
23.06
O
H
Me
O
H
H
H
N
F
7i
(151 MHz, CDCl3)
220 210 200 190 180 170 160 150 140 130 120 110 100 90 80 70 60 50 40 30 20 10 0
f1 (ppm)

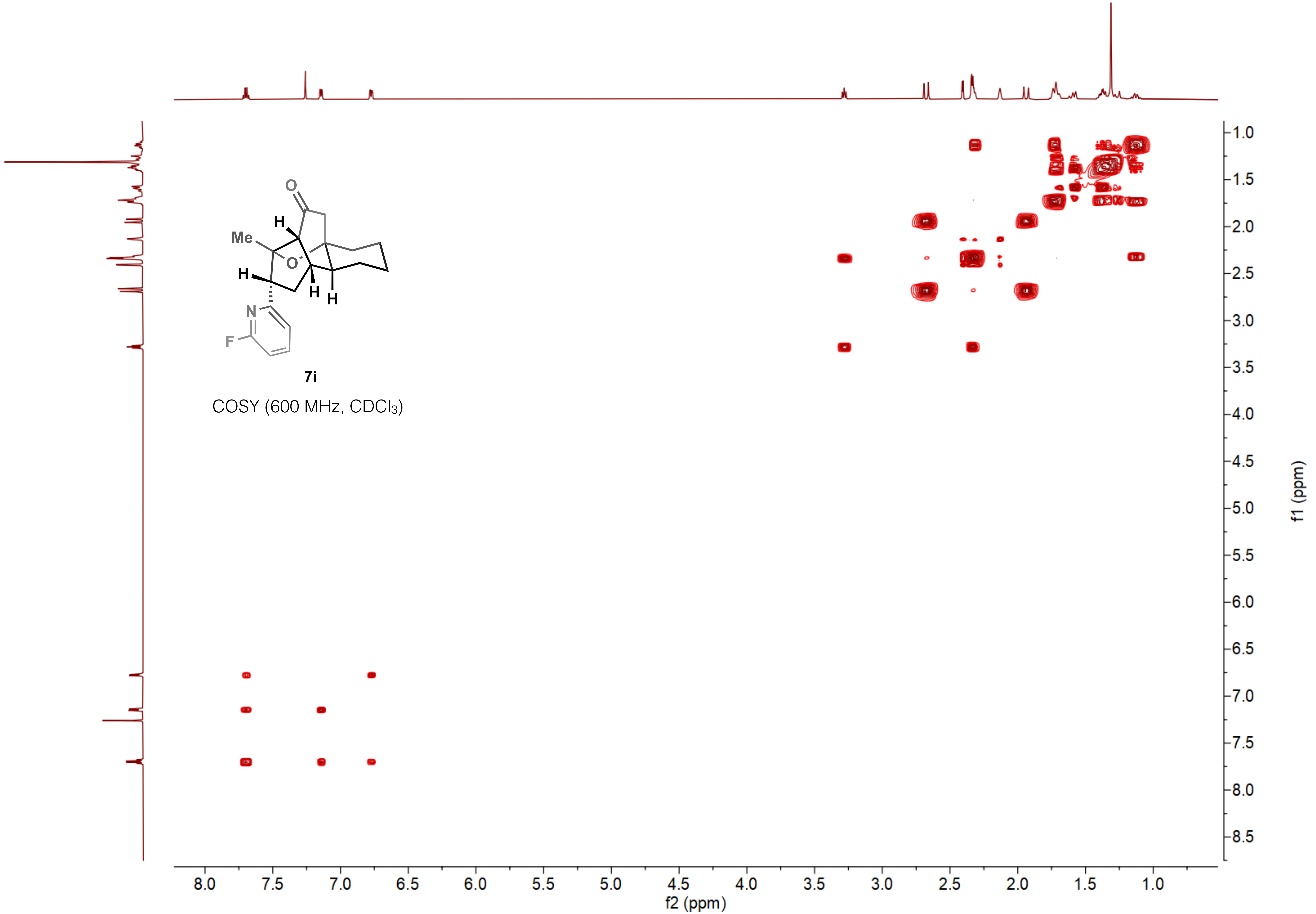
O
Me
H
O
H
H
H
N
F
7i
COSY (600 MHz, CDCl3)
f1 (ppm)
f2 (ppm)
8.0
7.5
7.0
6.5
6.0
5.5
5.0
4.5
4.0
3.5
3.0
2.5
2.0
1.5
1.0

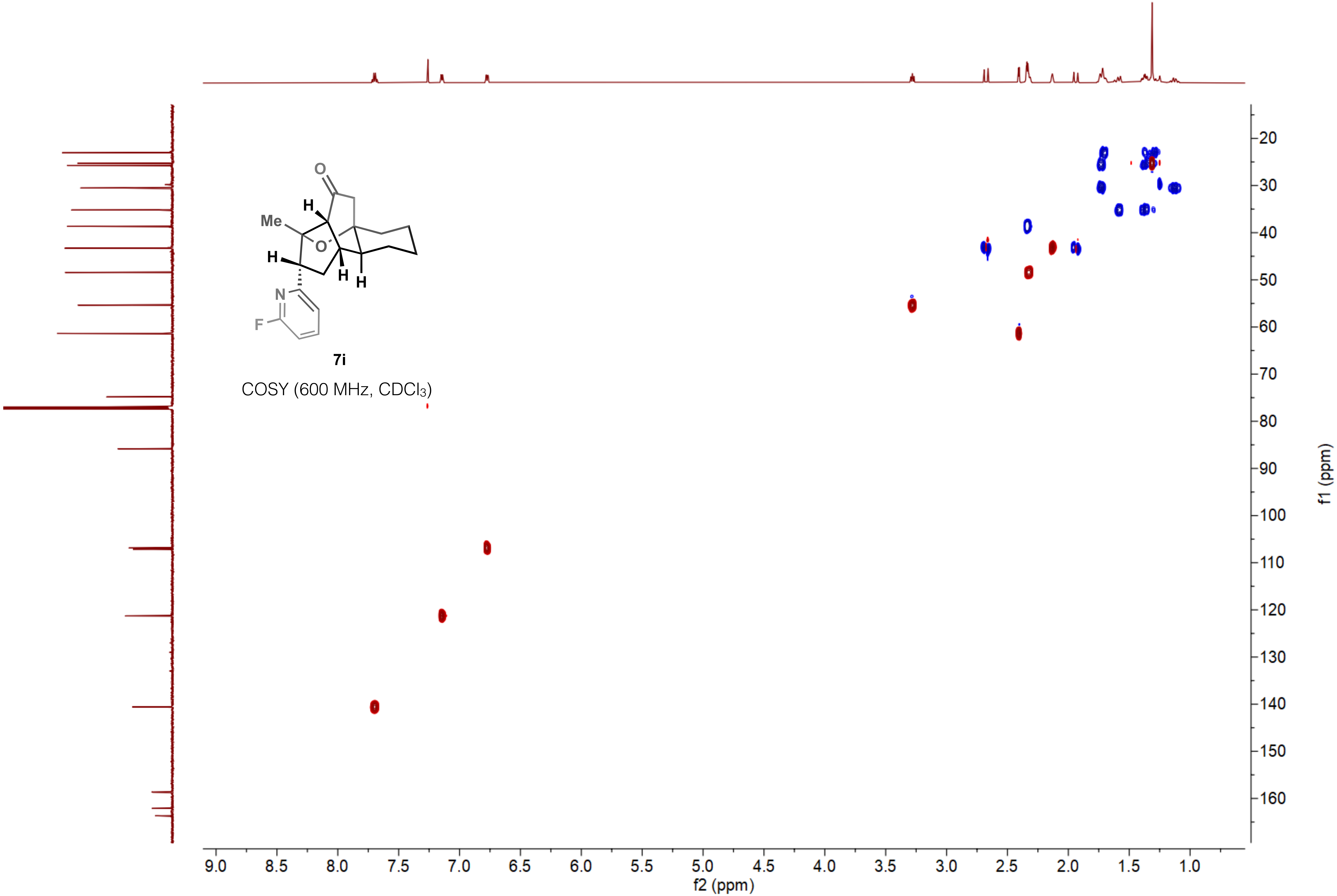

7i

COSY (600 MHz, $CDCl_3$)

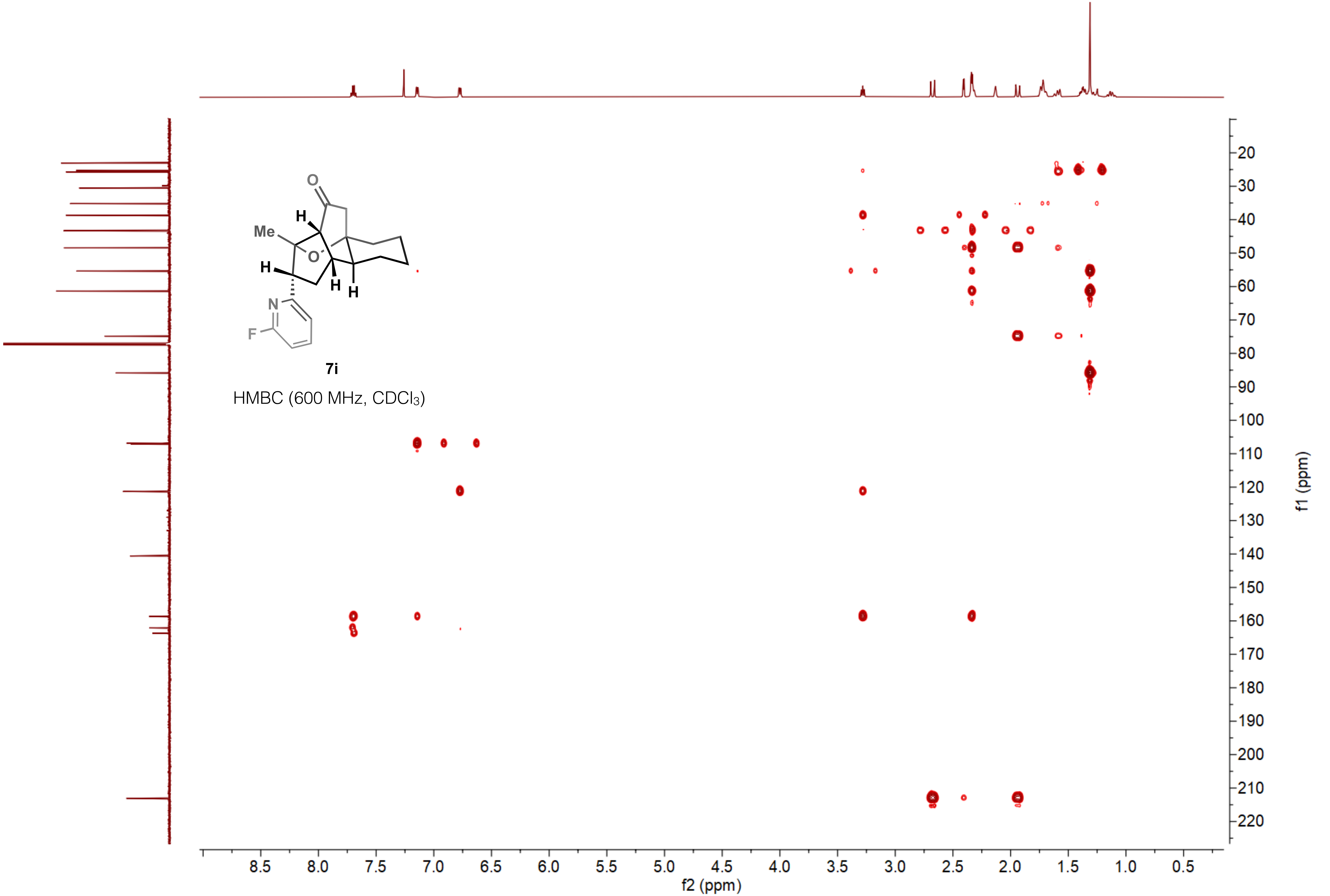

O
H
Me
O
H
H
H
N
F
7i
HMBC (600 MHz, CDCl3)
f2 (ppm)
f1 (ppm)

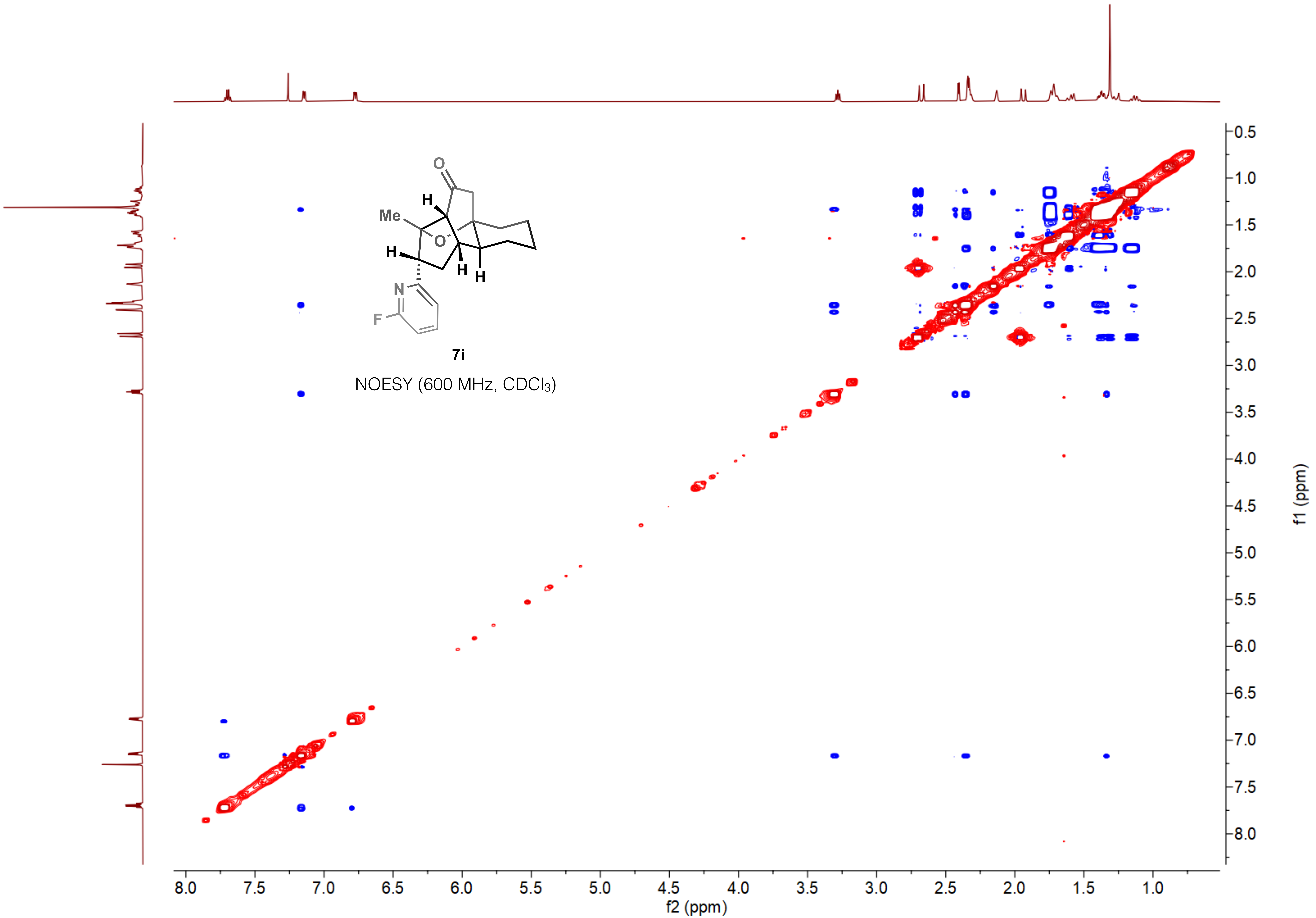

Me
O
H
N
F
7i
NOESY (600 MHz, CDCl3)
f2 (ppm)
f1 (ppm)

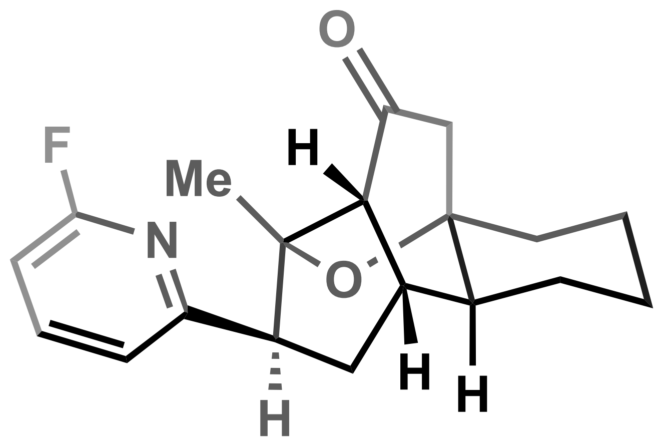

8i

(600 MHz, $CDCl_3$)

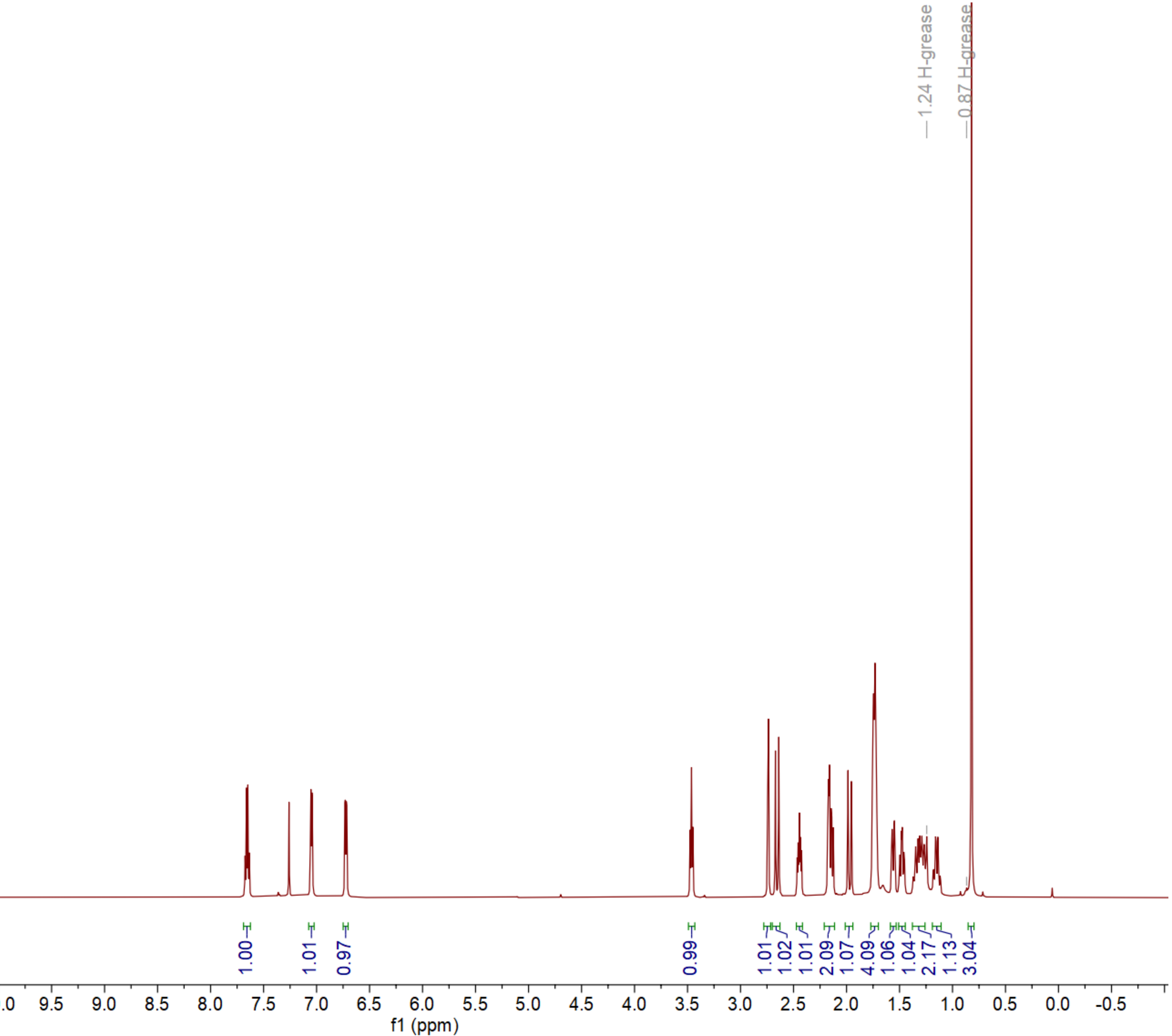

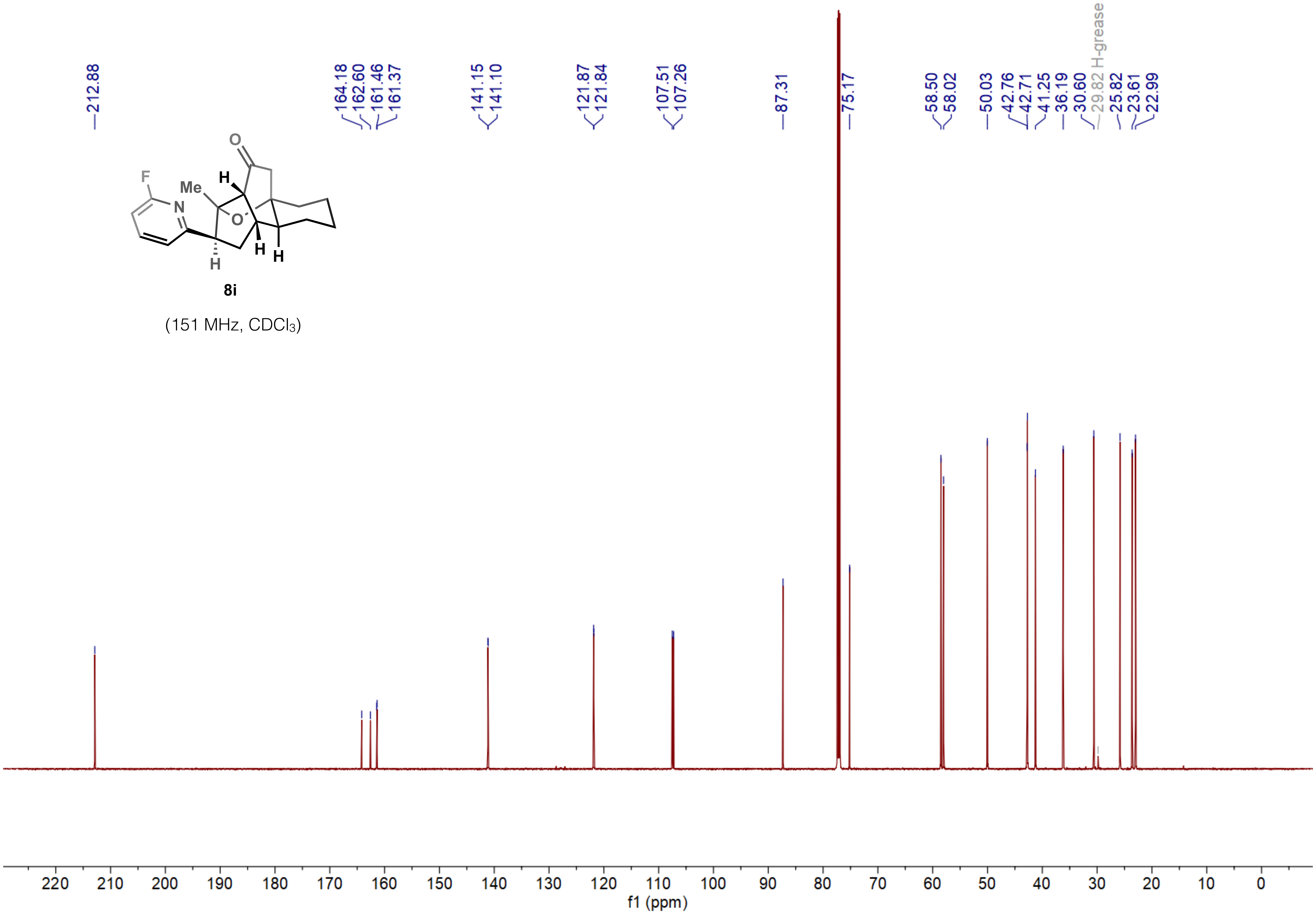

212.88
164.18
162.60
161.46
161.37
141.15
141.10
121.87
121.84
107.51
107.26
87.31
75.17
58.50
58.02
50.03
42.76
42.71
41.25
36.19
30.60
29.82 H-grease
25.82
23.61
22.99
F
N
Me
H
O
H
H
H
8i
(151 MHz, CDCl3)
220 210 200 190 180 170 160 150 140 130 120 110 100 90 80 70 60 50 40 30 20 10 0
f1 (ppm)

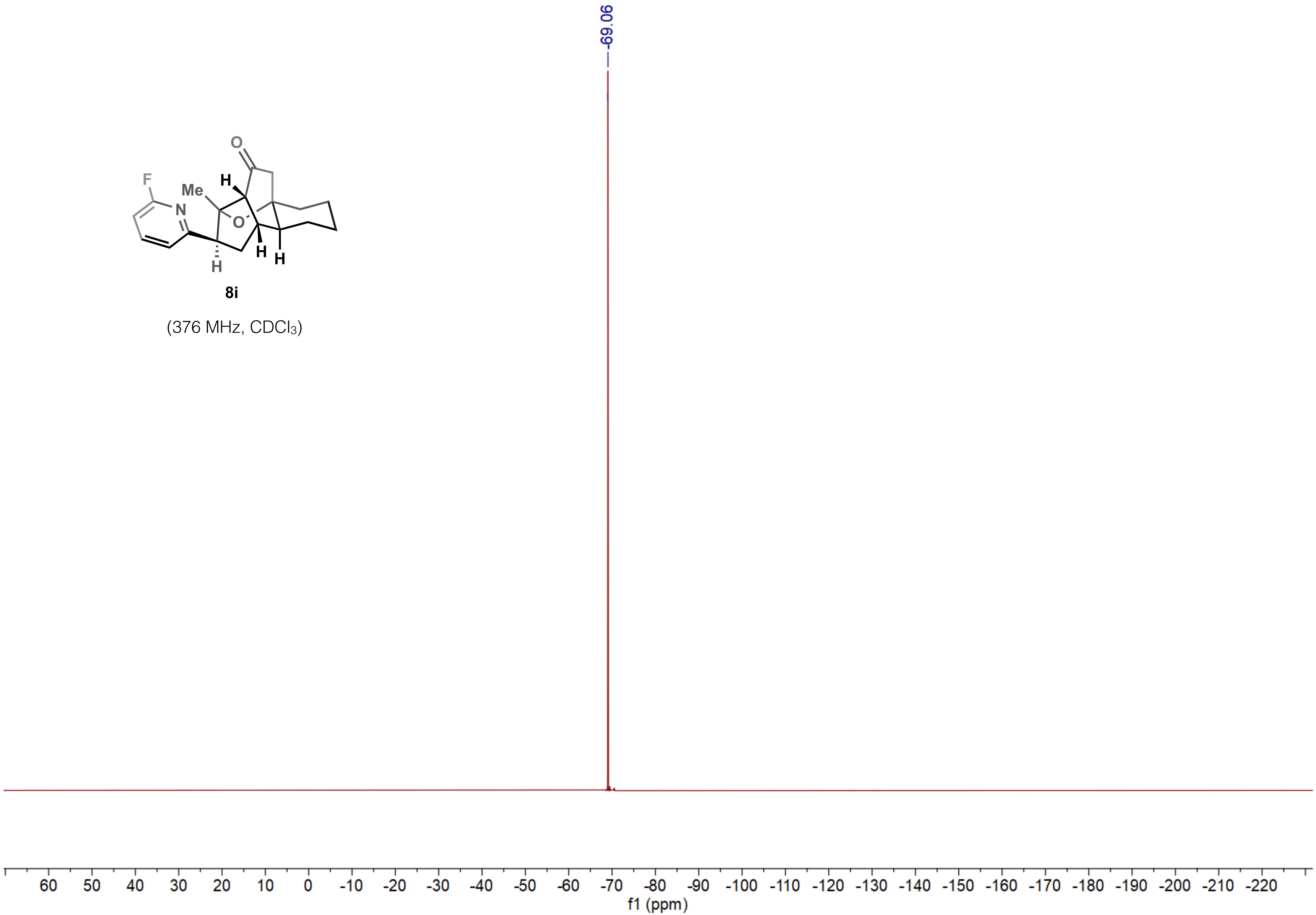

-69.06
F
N
Me
O
H
8i
(376 MHz, CDCl3)
60 50 40 30 20 10 0 -10 -20 -30 -40 -50 -60 -70 -80 -90 -100 -110 -120 -130 -140 -150 -160 -170 -180 -190 -200 -210 -220
f1 (ppm)

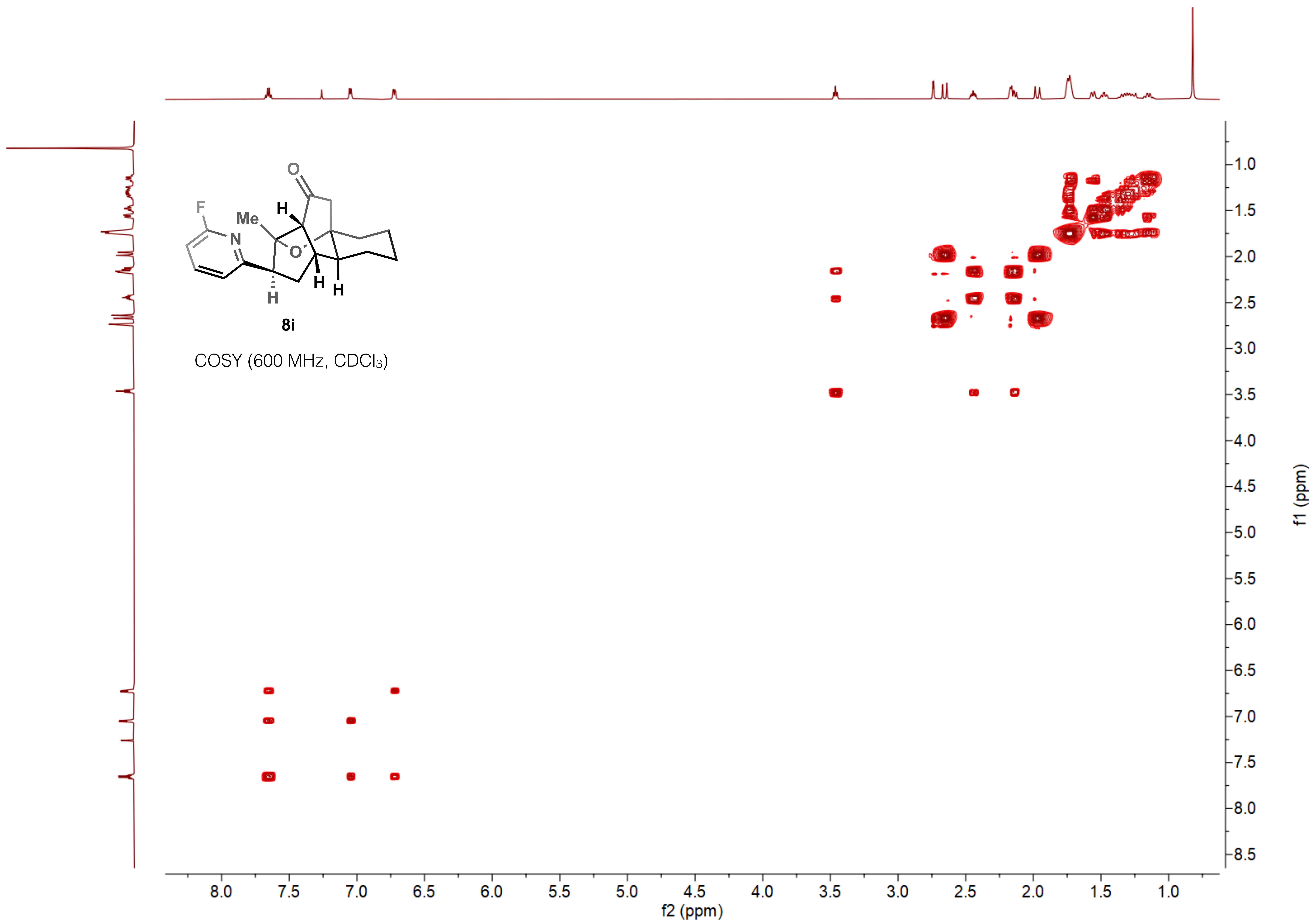
F
N
Me
H
O
O
H
H
H
8i
COSY (600 MHz, CDCl3)
f1 (ppm)
f2 (ppm)

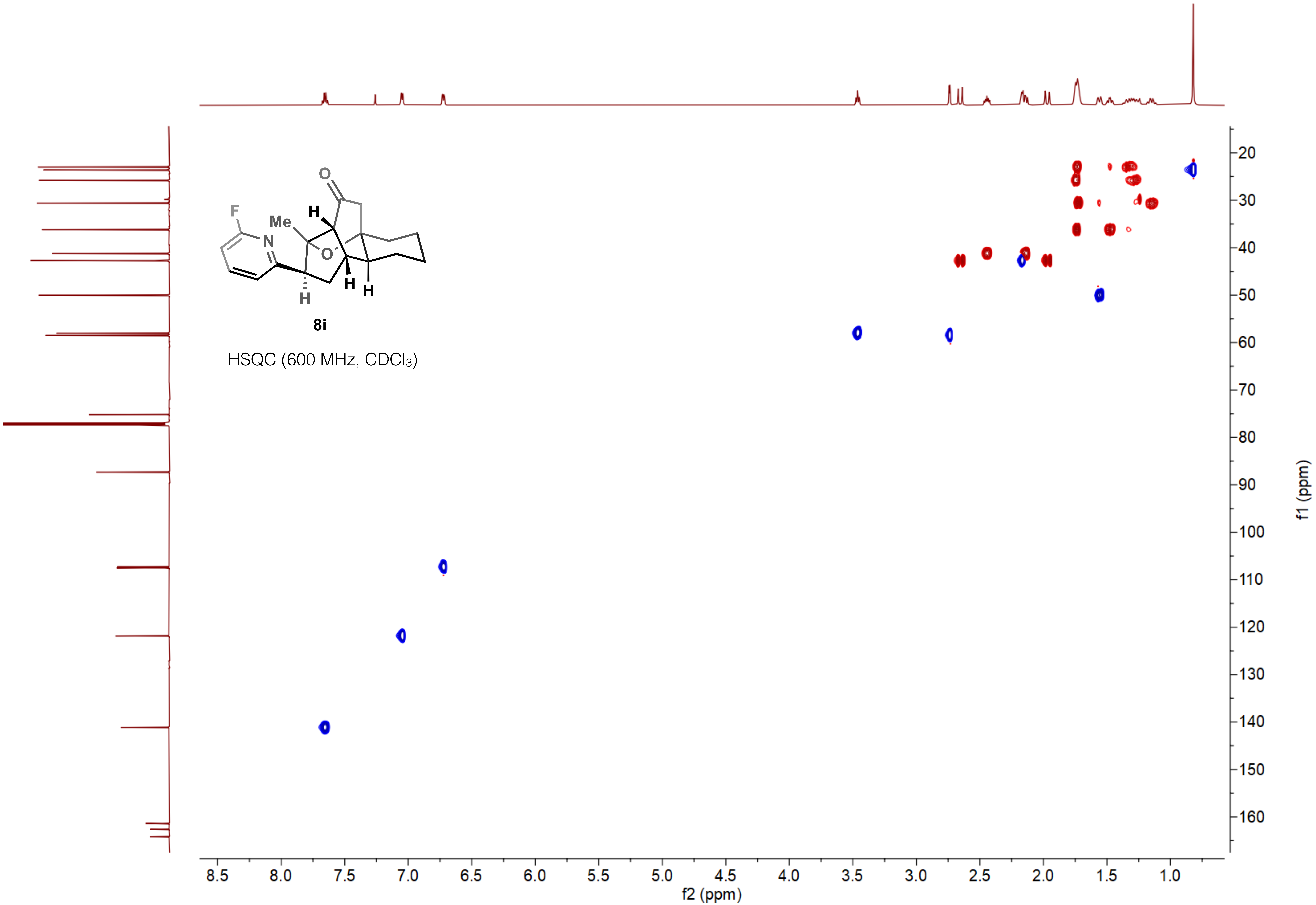

8i

HSQC (600 MHz, $CDCl_3$)

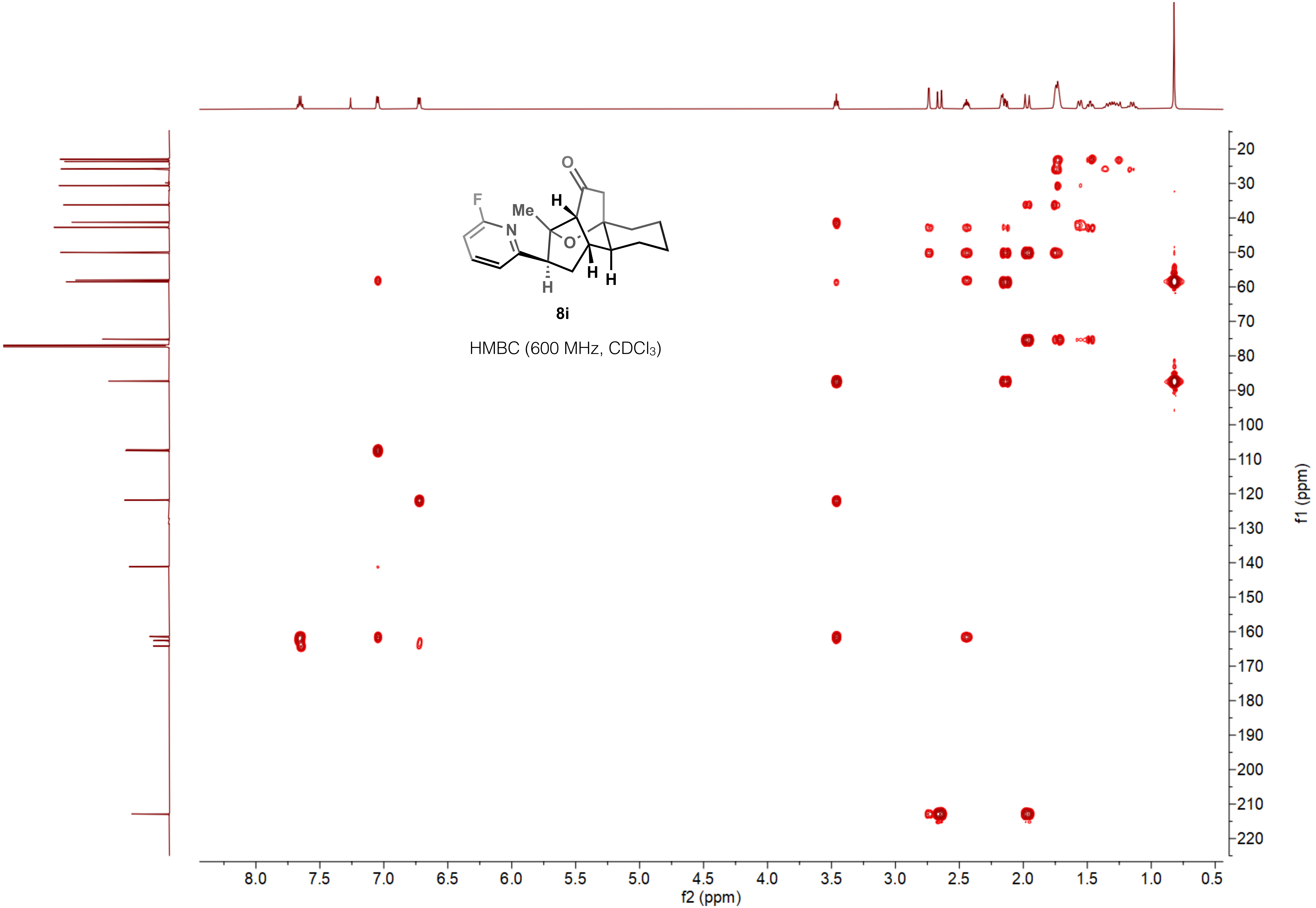

F
Me
N
O
H
H
H
H
8i
HMBC (600 MHz, CDCl3)
f2 (ppm)
f1 (ppm)

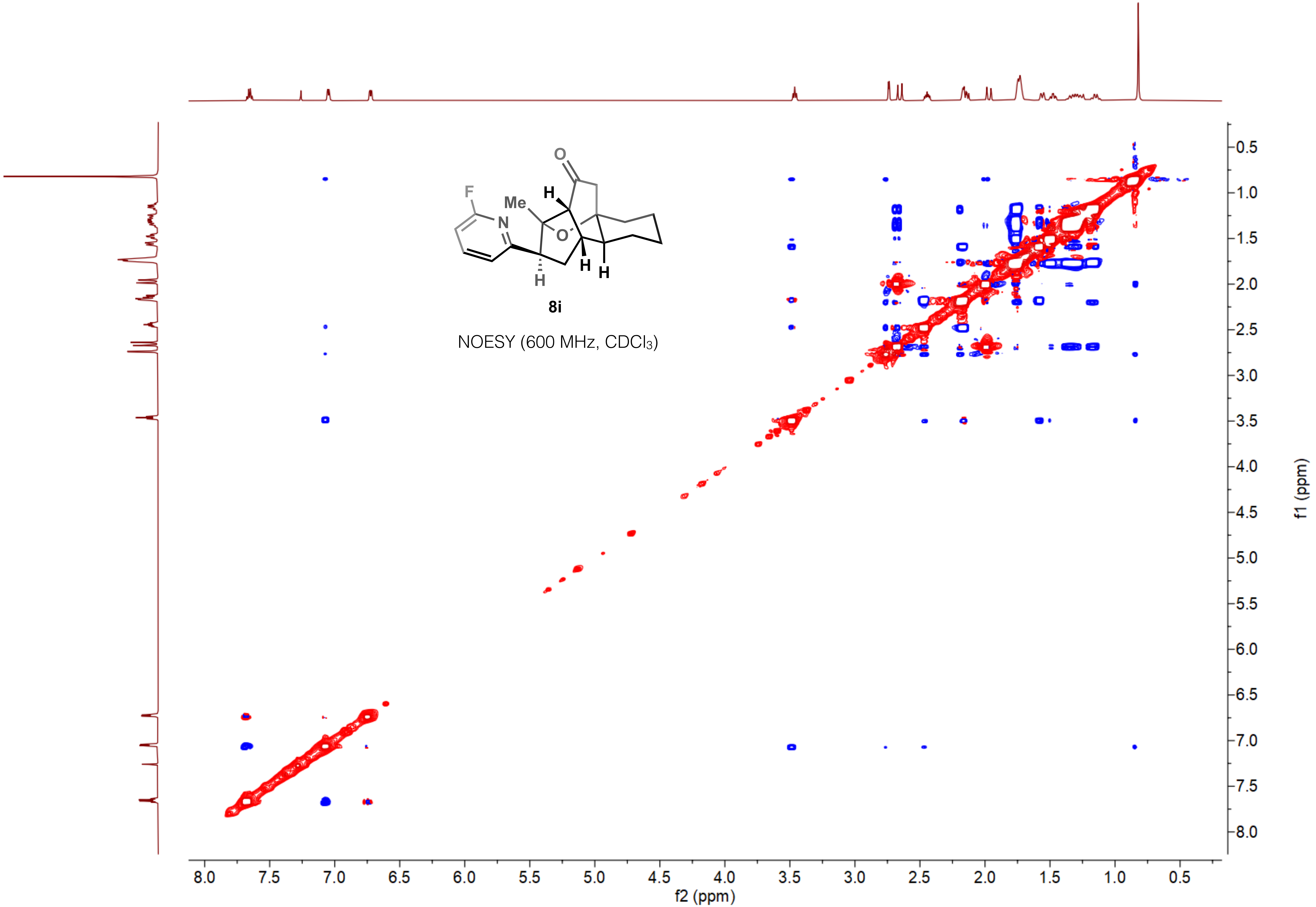

F
Me
N
O
H
H
H
H
8i
NOESY (600 MHz, CDCl3)
f2 (ppm)
f1 (ppm)

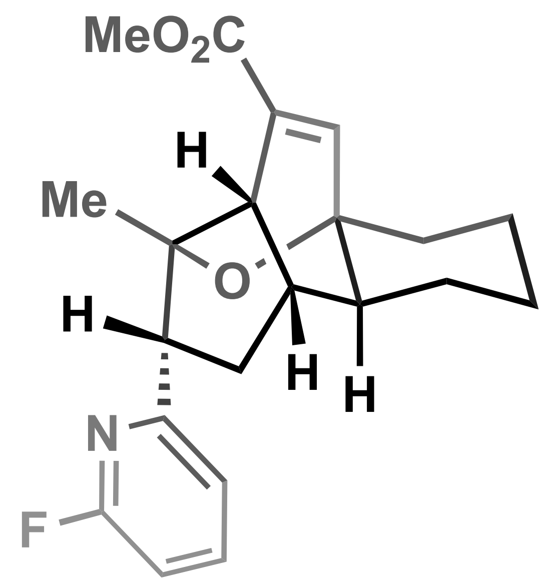

RS1134

(600 MHz, $CDCl_3$)

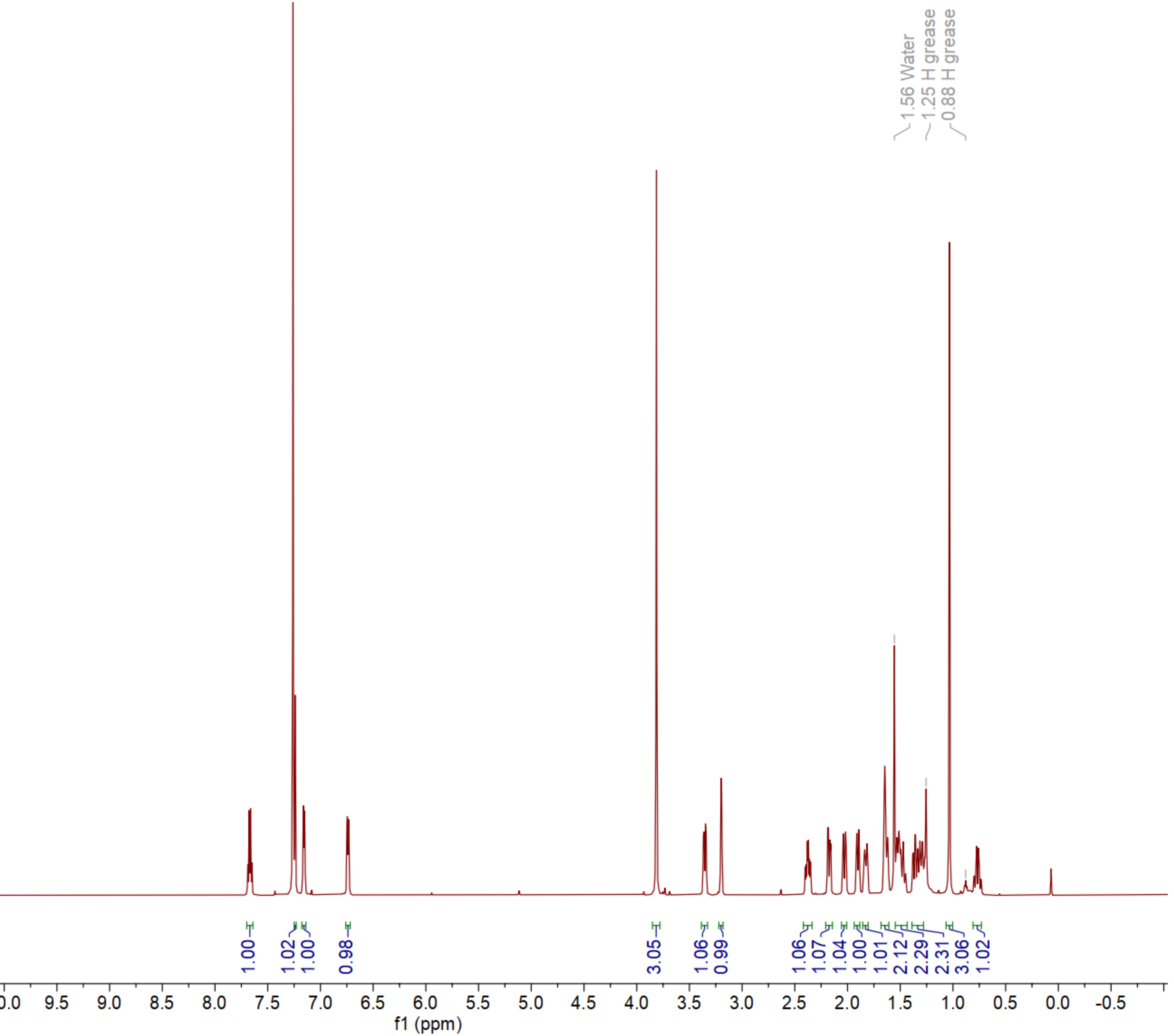

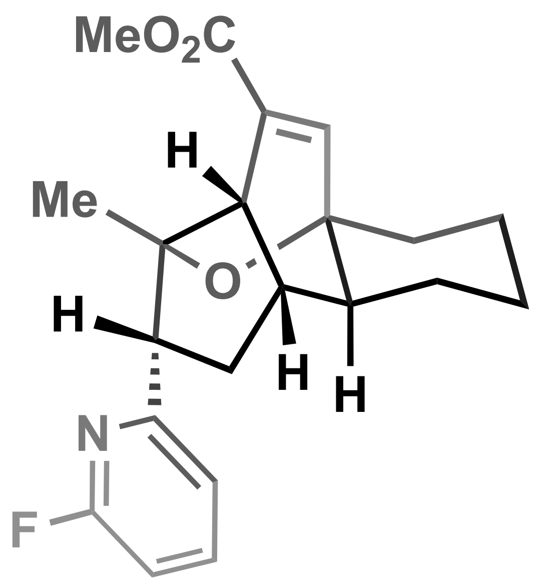

RS1134

(151 MHz, $CDCl_3$)

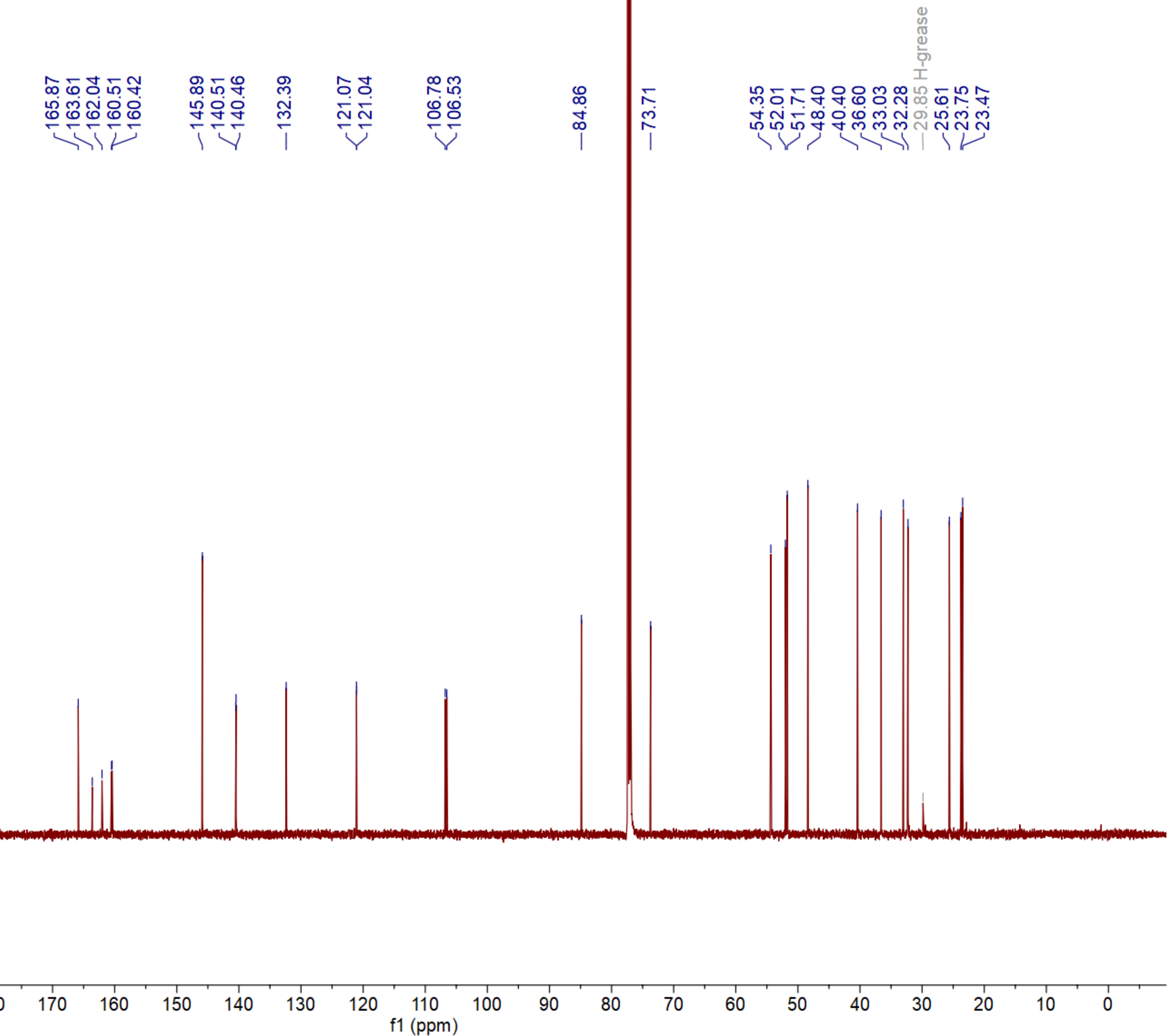

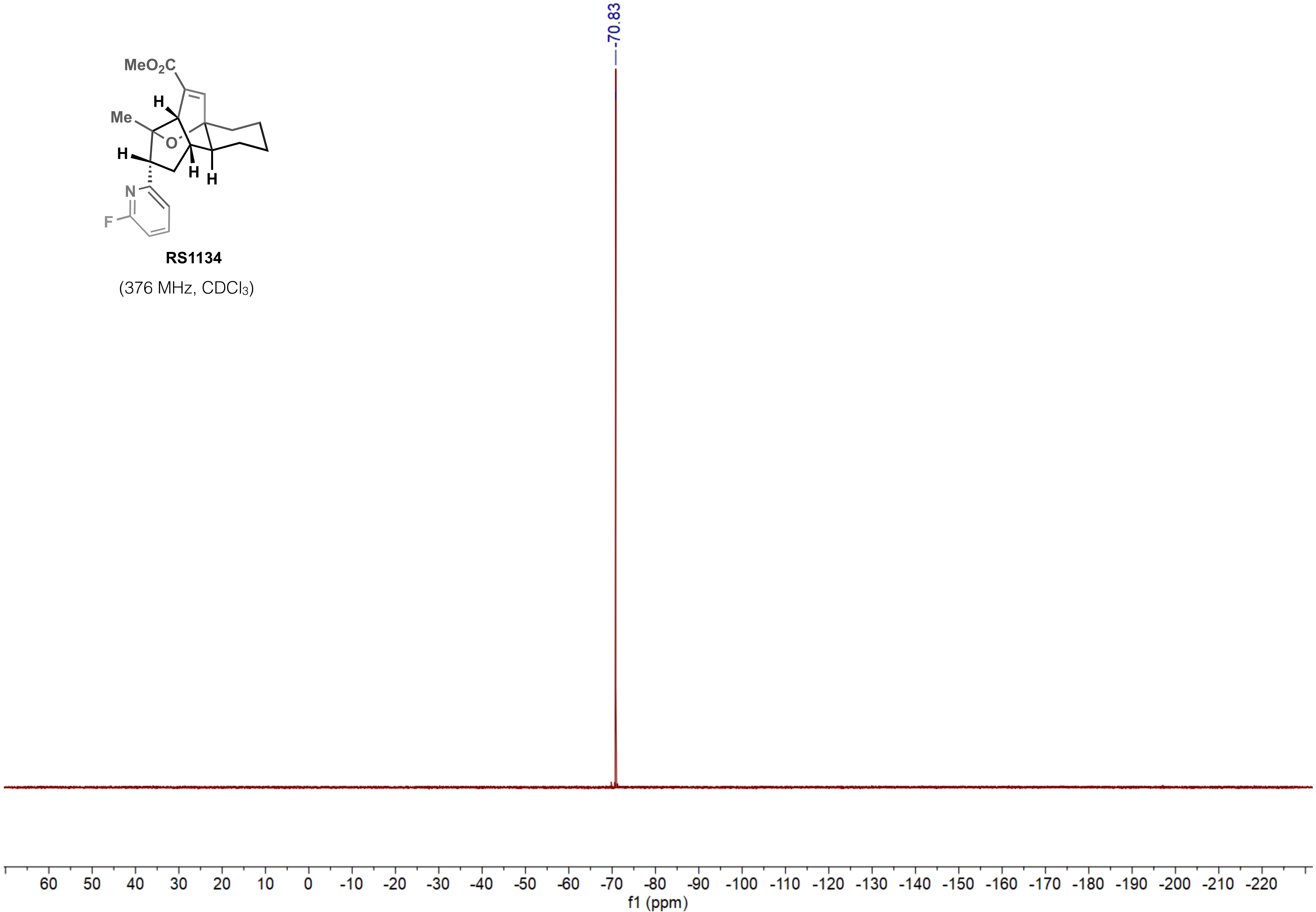
MeO$_2$C
H
Me
O
H
H
H
N
F
RS1134
(376 MHz, $CDCl_3$)
-70.83
60 50 40 30 20 10 0 -10 -20 -30 -40 -50 -60 -70 -80 -90 -100 -110 -120 -130 -140 -150 -160 -170 -180 -190 -200 -210 -220
f1 (ppm)

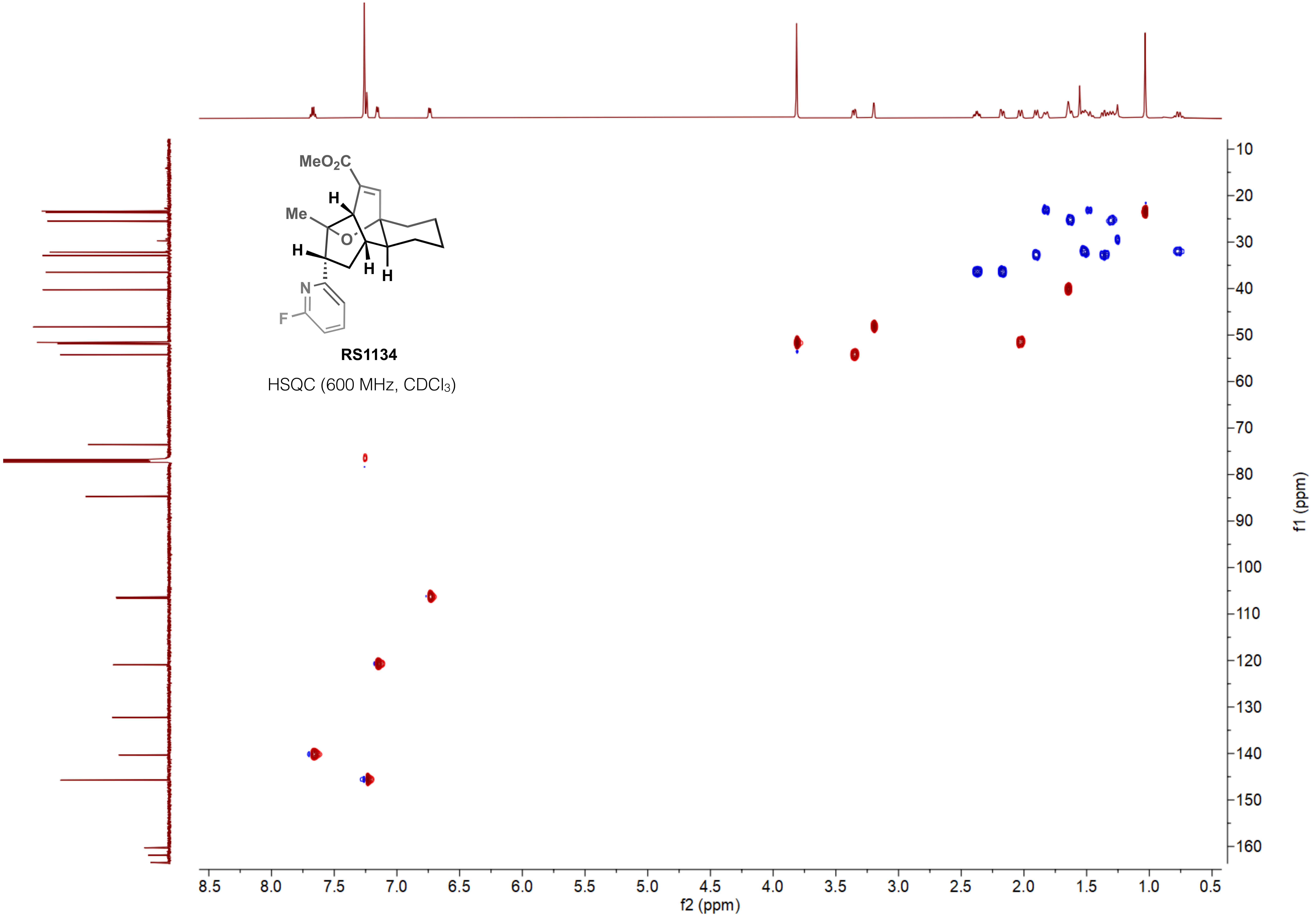

MeO2C
Me
H
O
H
H
H
N
F
RS1134
HSQC (600 MHz, CDCl3)
f2 (ppm)
f1 (ppm)

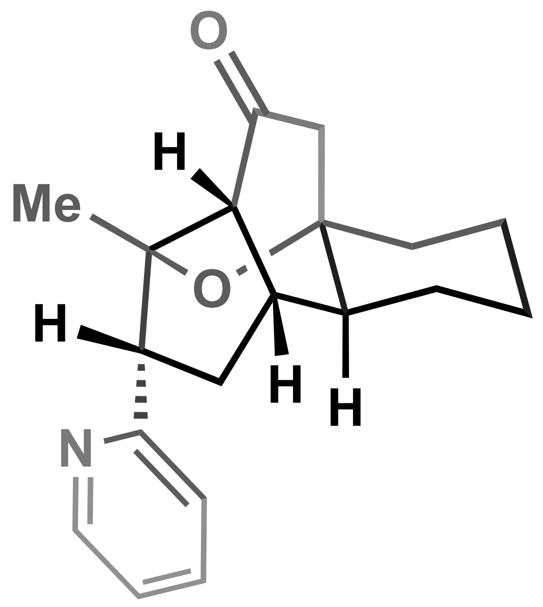

7j

(600 MHz, $CDCl_3$)

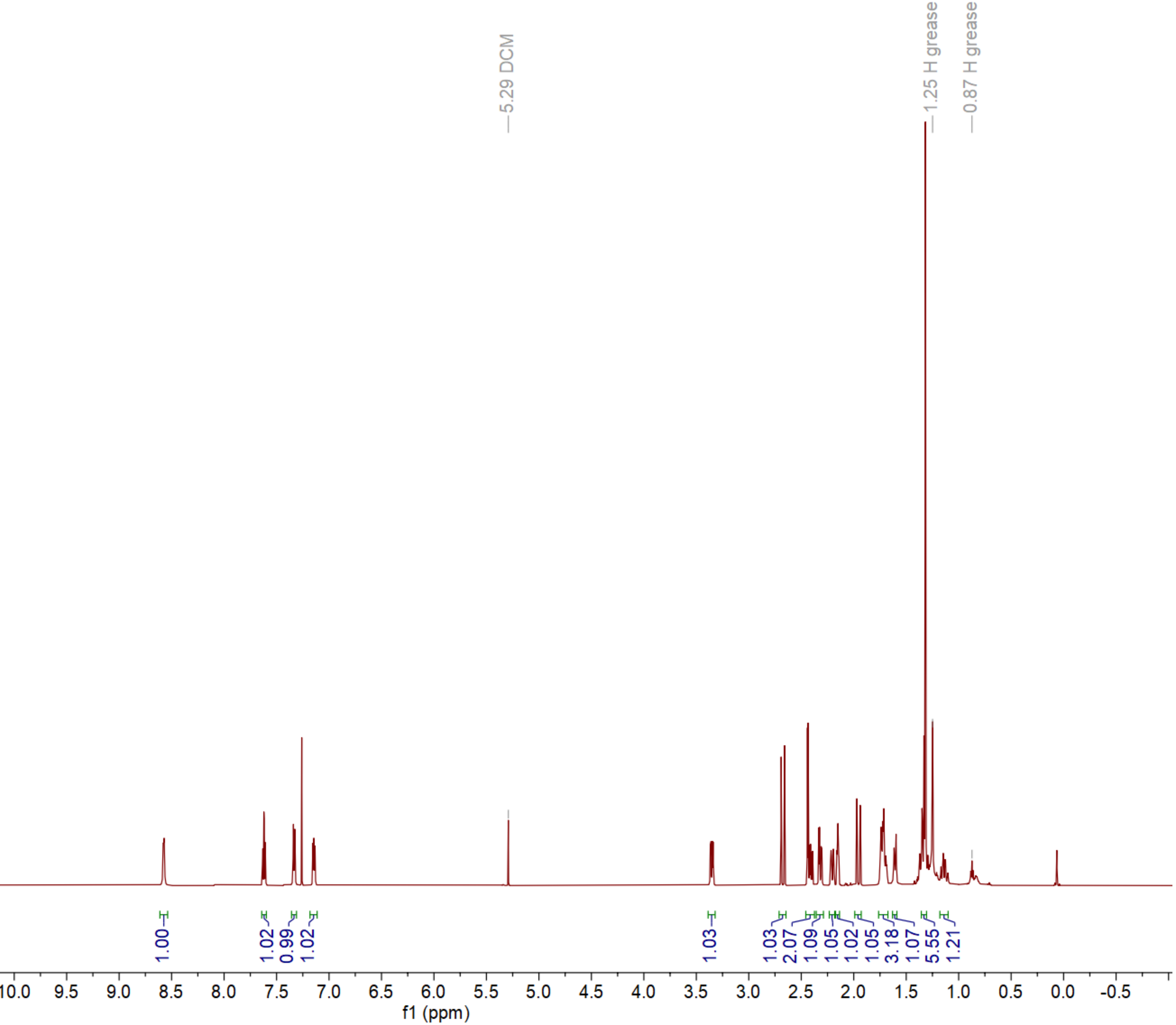

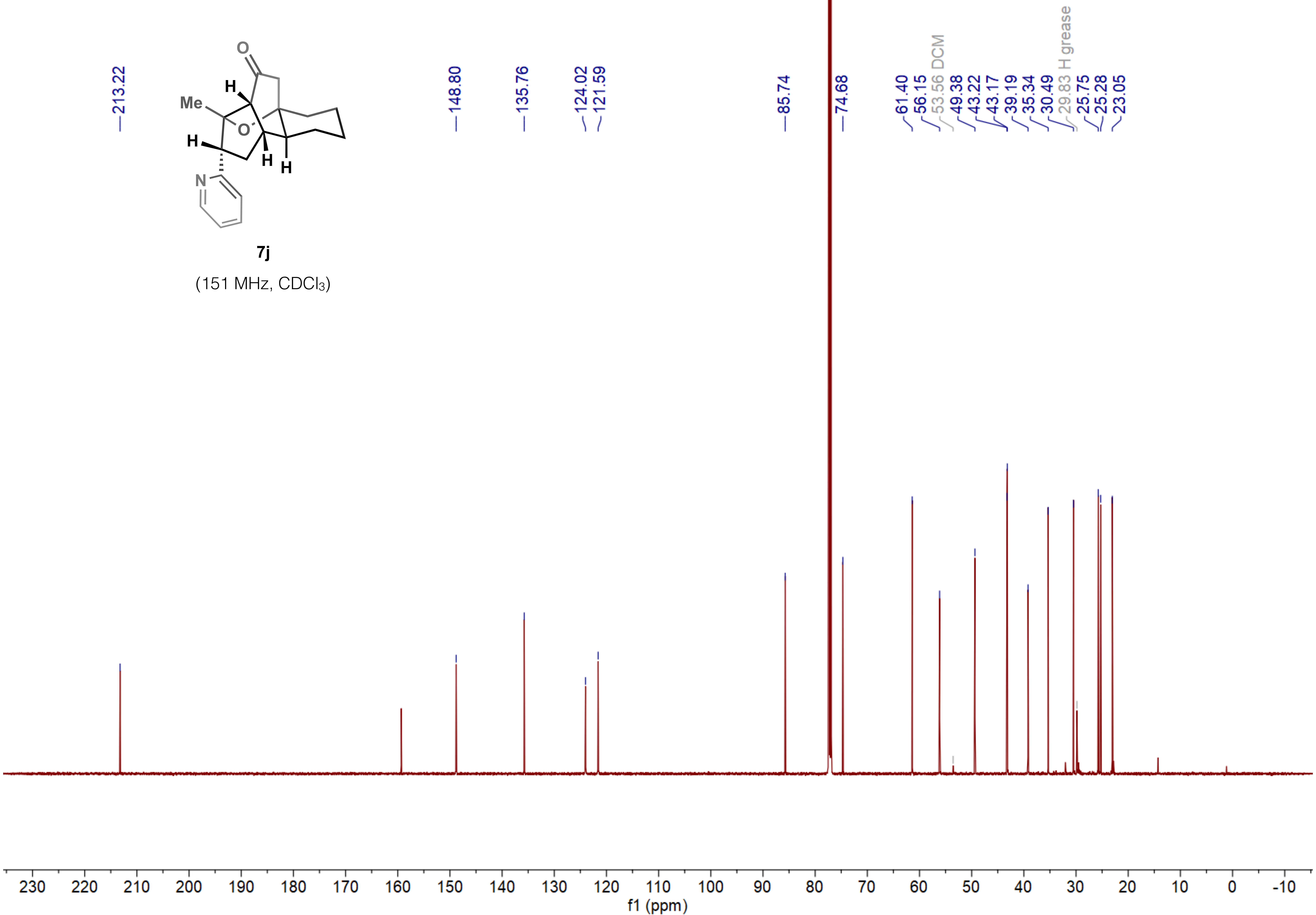

213.22
148.80
135.76
124.02
121.59
85.74
74.68
61.40
56.15
53.56 DCM
49.38
43.22
43.17
39.19
35.34
30.49
29.83 H grease
25.75
25.28
23.05
Me
O
H
N
7j
(151 MHz, CDCl3)
230 220 210 200 190 180 170 160 150 140 130 120 110 100 90 80 70 60 50 40 30 20 10 0 -10
f1 (ppm)

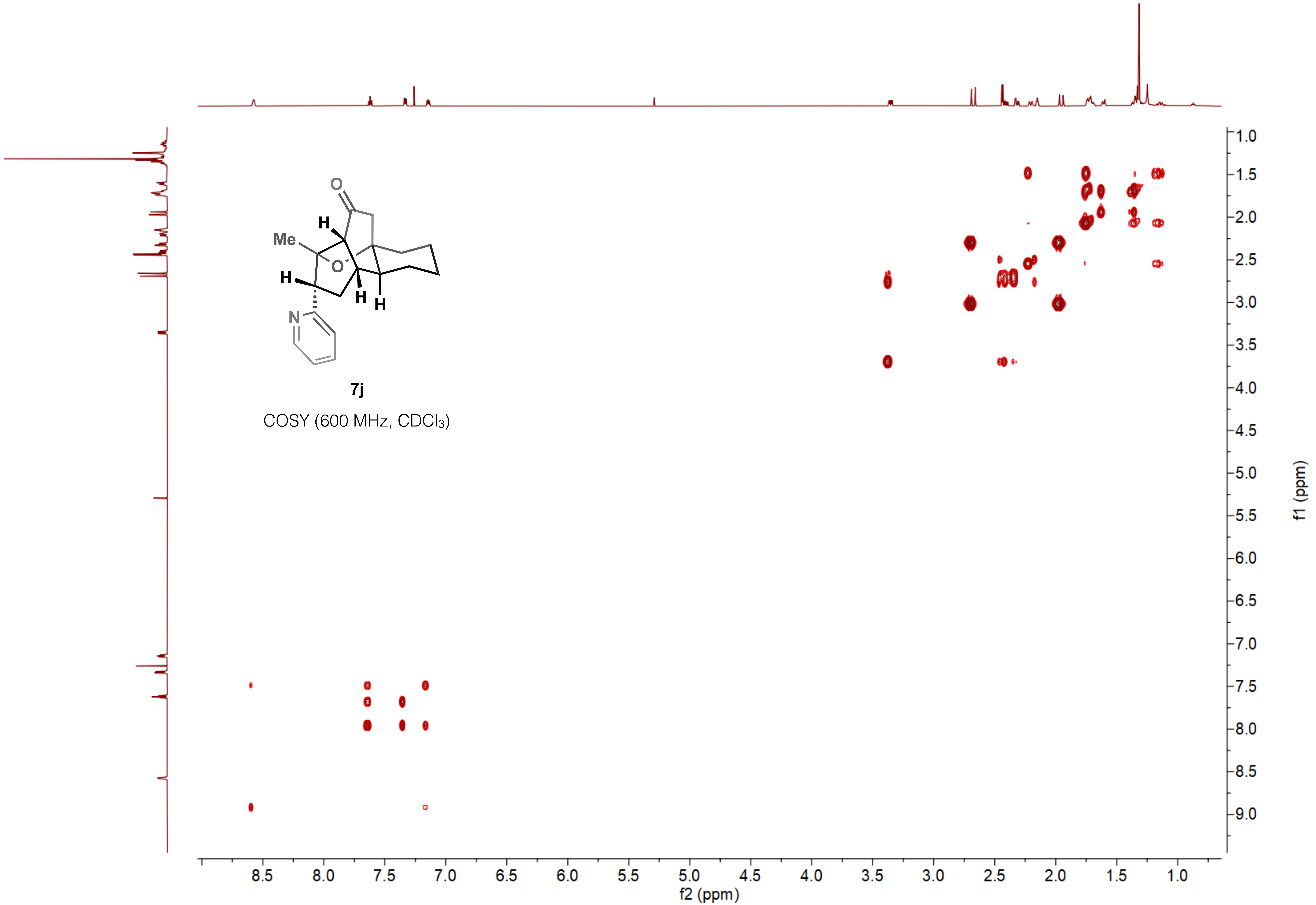

7j
COSY (600 MHz, CDCl3)
f2 (ppm)
f1 (ppm)

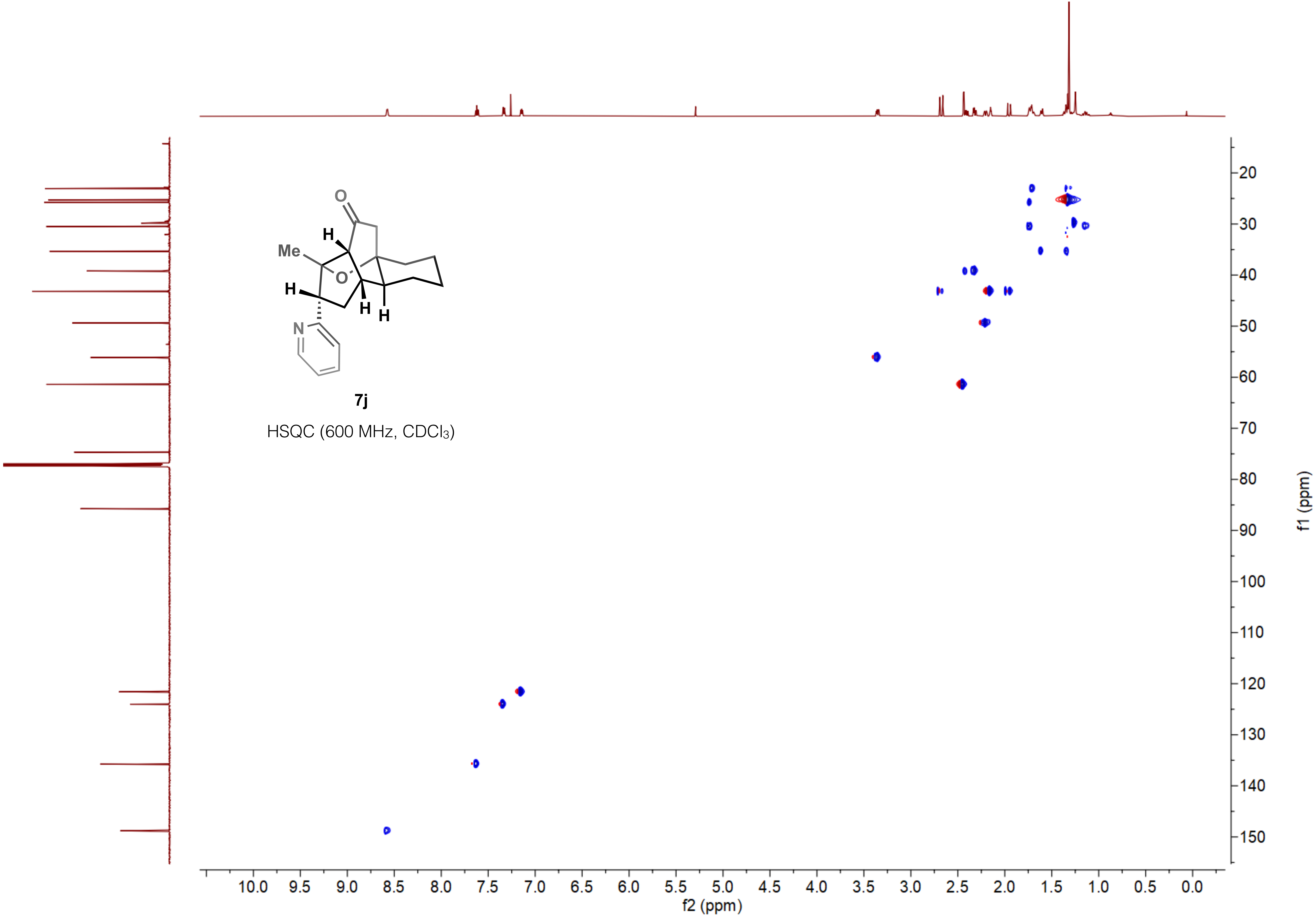

7j

HSQC (600 MHz, $CDCl_3$)

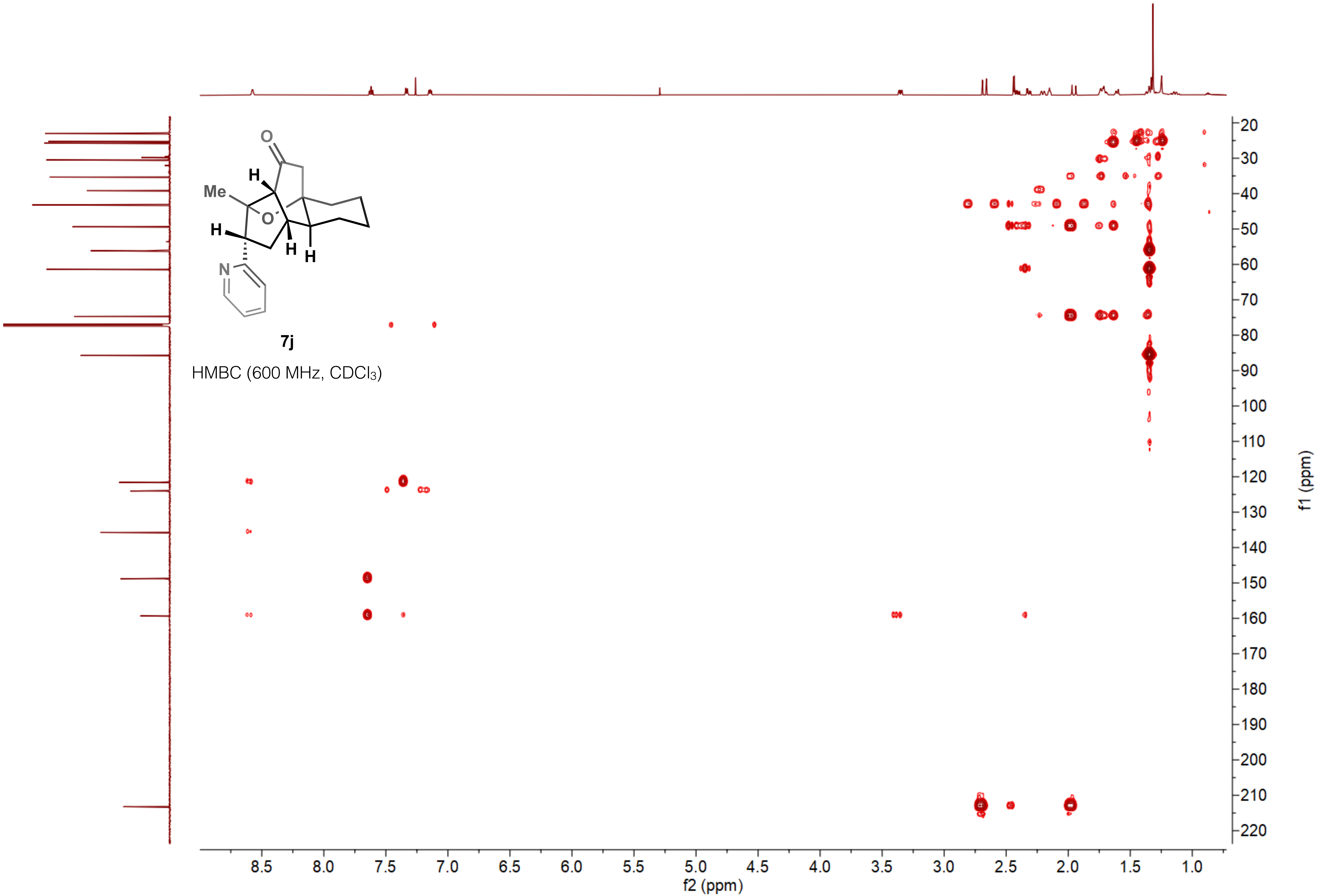

7j

HMBC (600 MHz, CDCl₃)

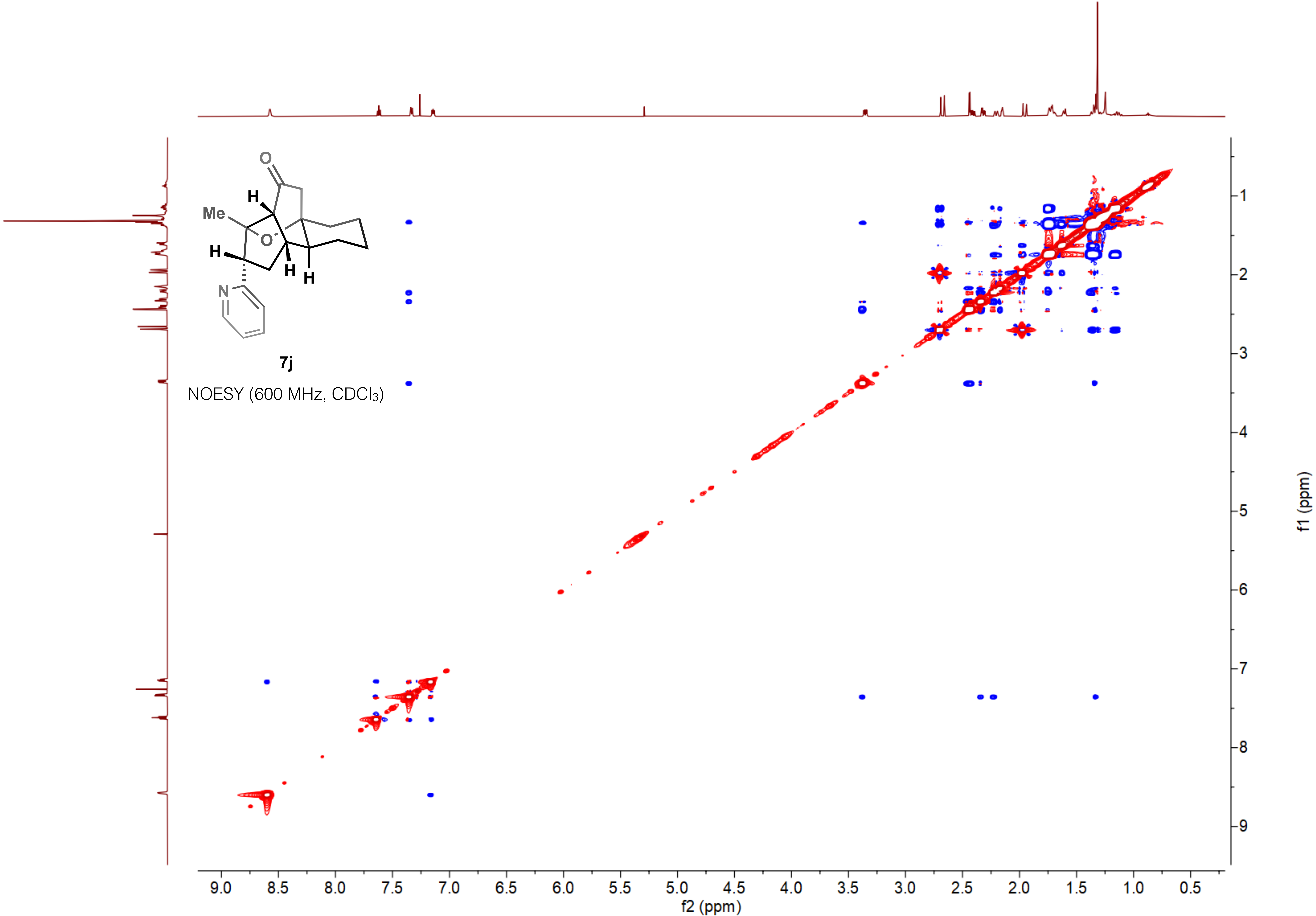
O
H
Me
O
H
H
H
N
7j
NOESY (600 MHz, CDCl3)
f2 (ppm)
f1 (ppm)

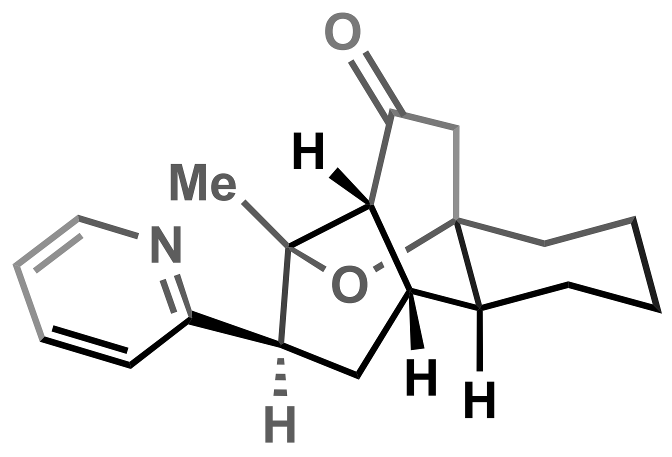

8j

(600 MHz, $CDCl_3$)

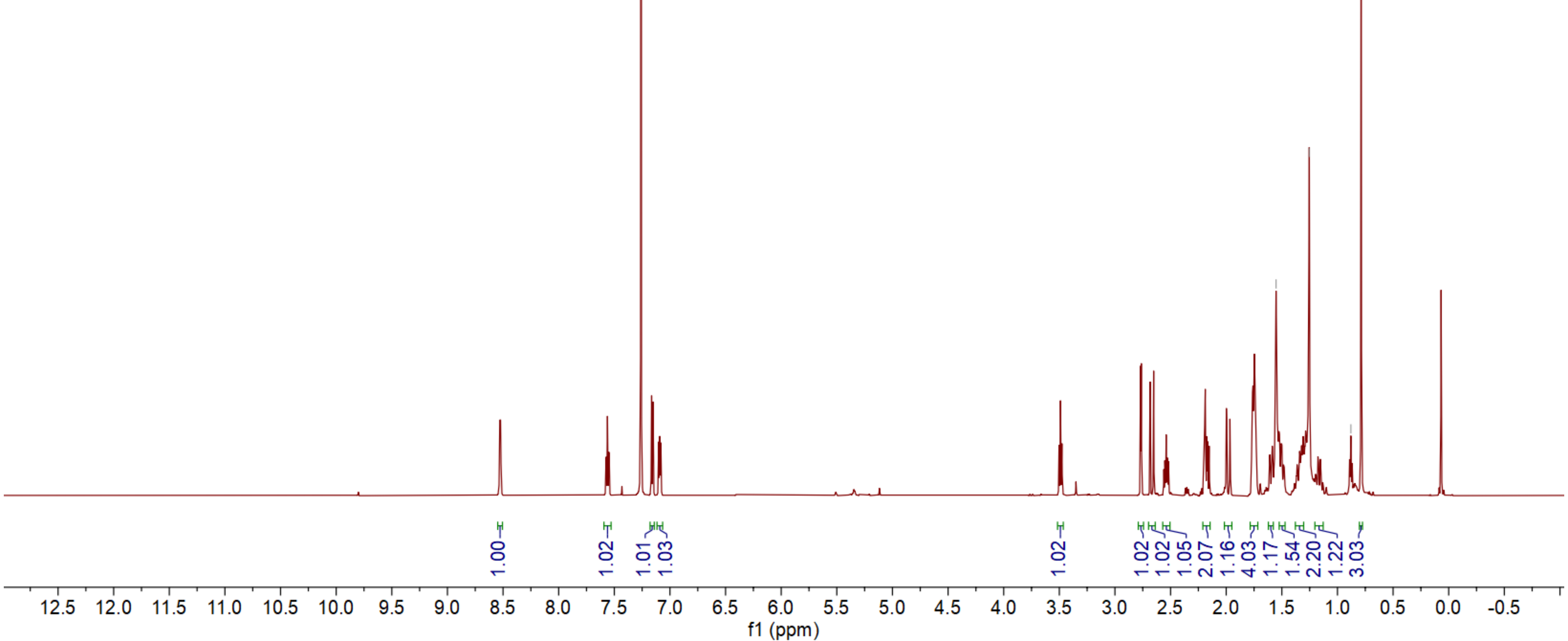

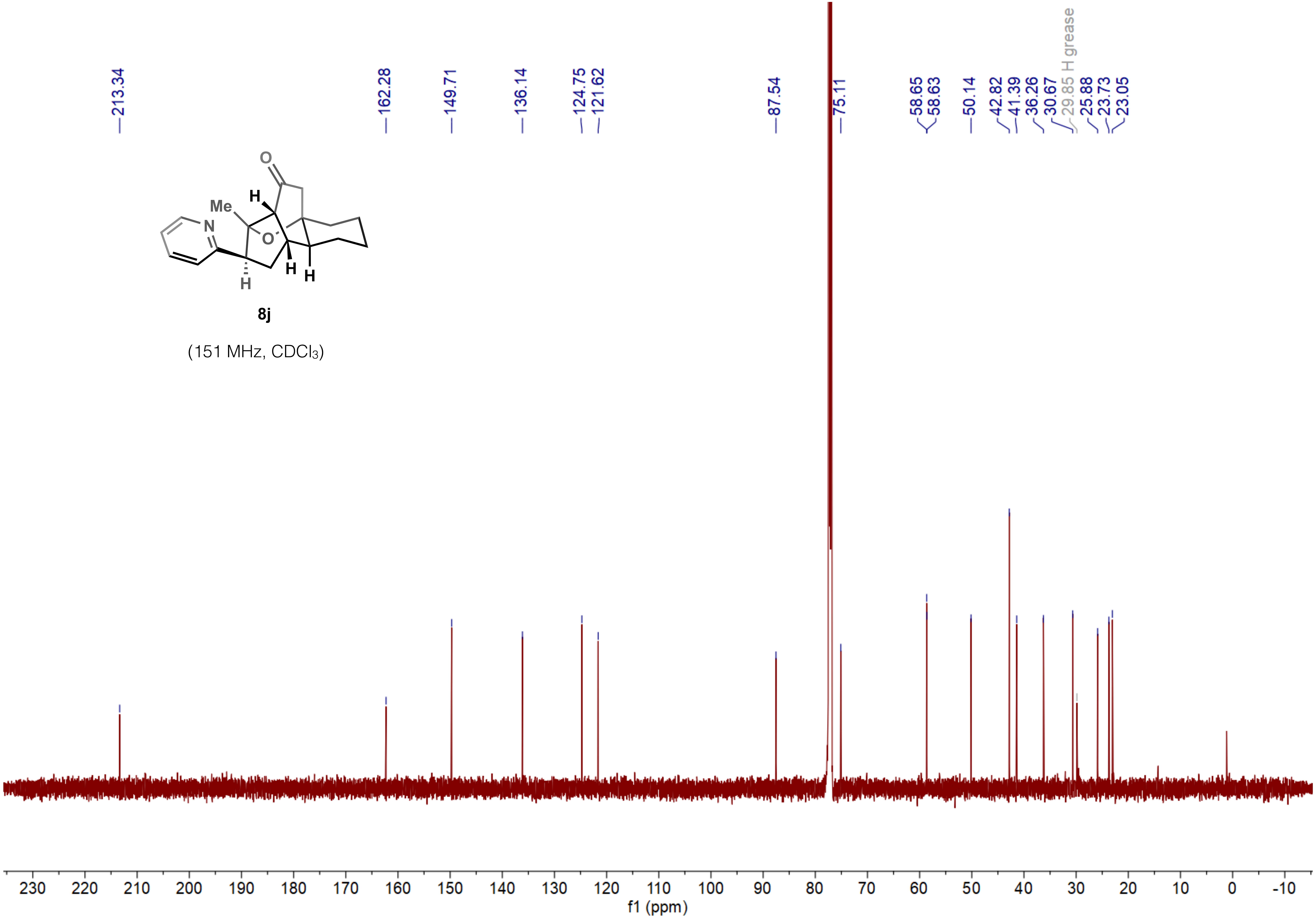
213.34
162.28
149.71
136.14
124.75
121.62
87.54
75.11
58.65
58.63
50.14
42.82
41.39
36.26
30.67
29.85 H grease
25.88
23.73
23.05
O
Me
H
N
O
H
H
H
8j
(151 MHz, $CDCl_3$)
230 220 210 200 190 180 170 160 150 140 130 120 110 100 90 80 70 60 50 40 30 20 10 0 -10
f1 (ppm)

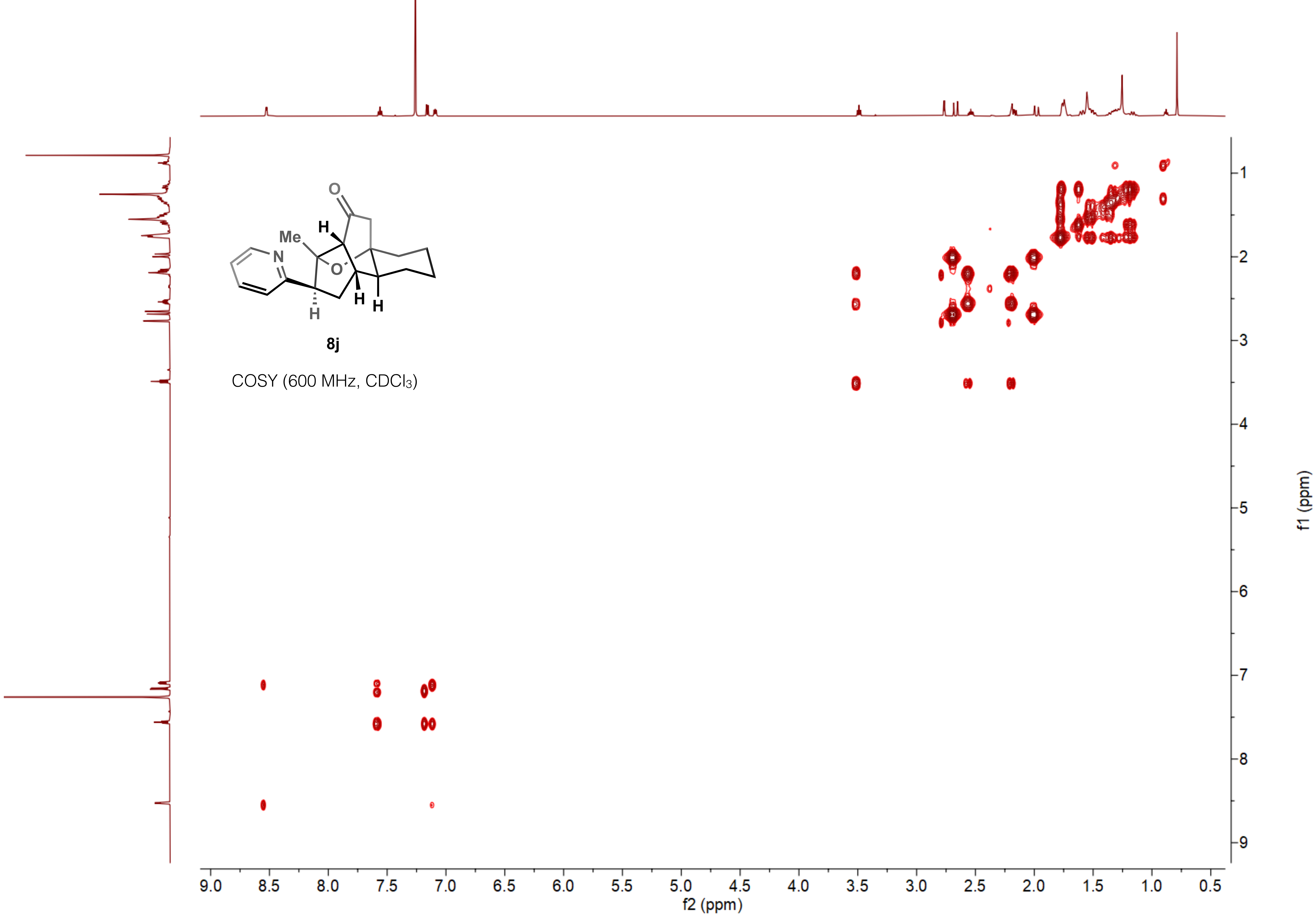

8j

COSY (600 MHz, $CDCl_3$)

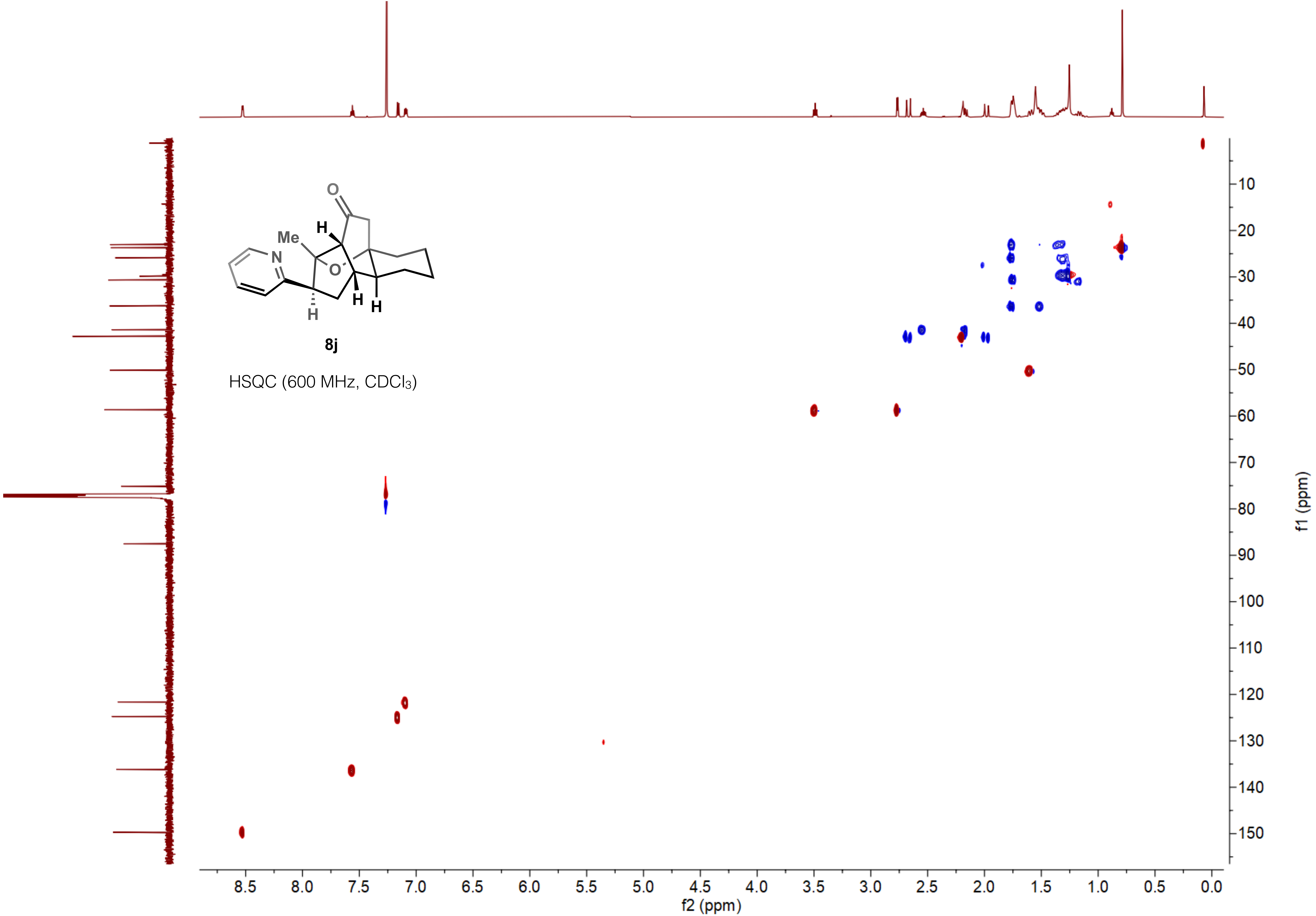

Me
N
O
H
H
H
H
O
8j
HSQC (600 MHz, CDCl3)
f2 (ppm)
f1 (ppm)

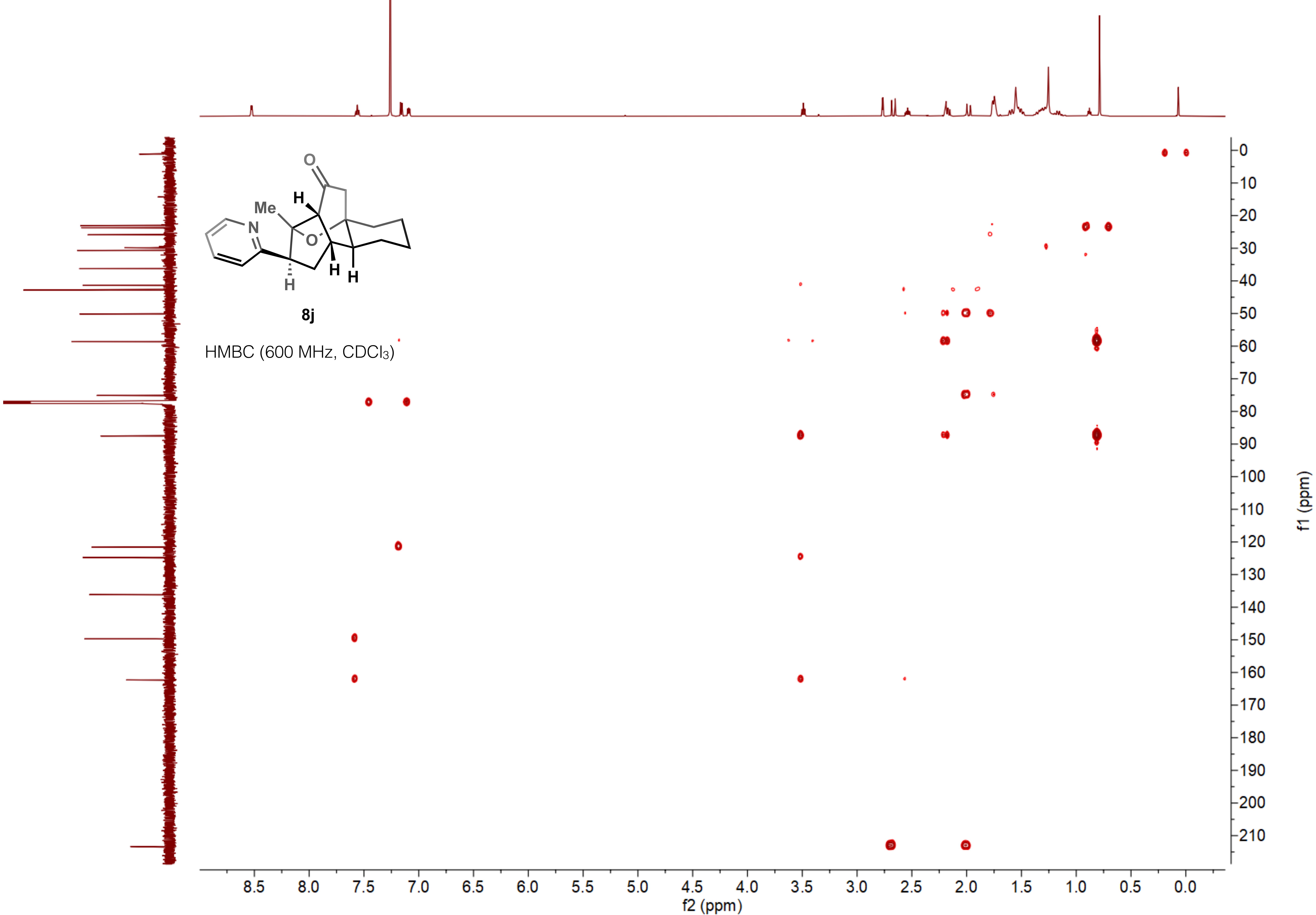

Me
N
O
H
8j
HMBC (600 MHz, CDCl3)
f2 (ppm)
f1 (ppm)

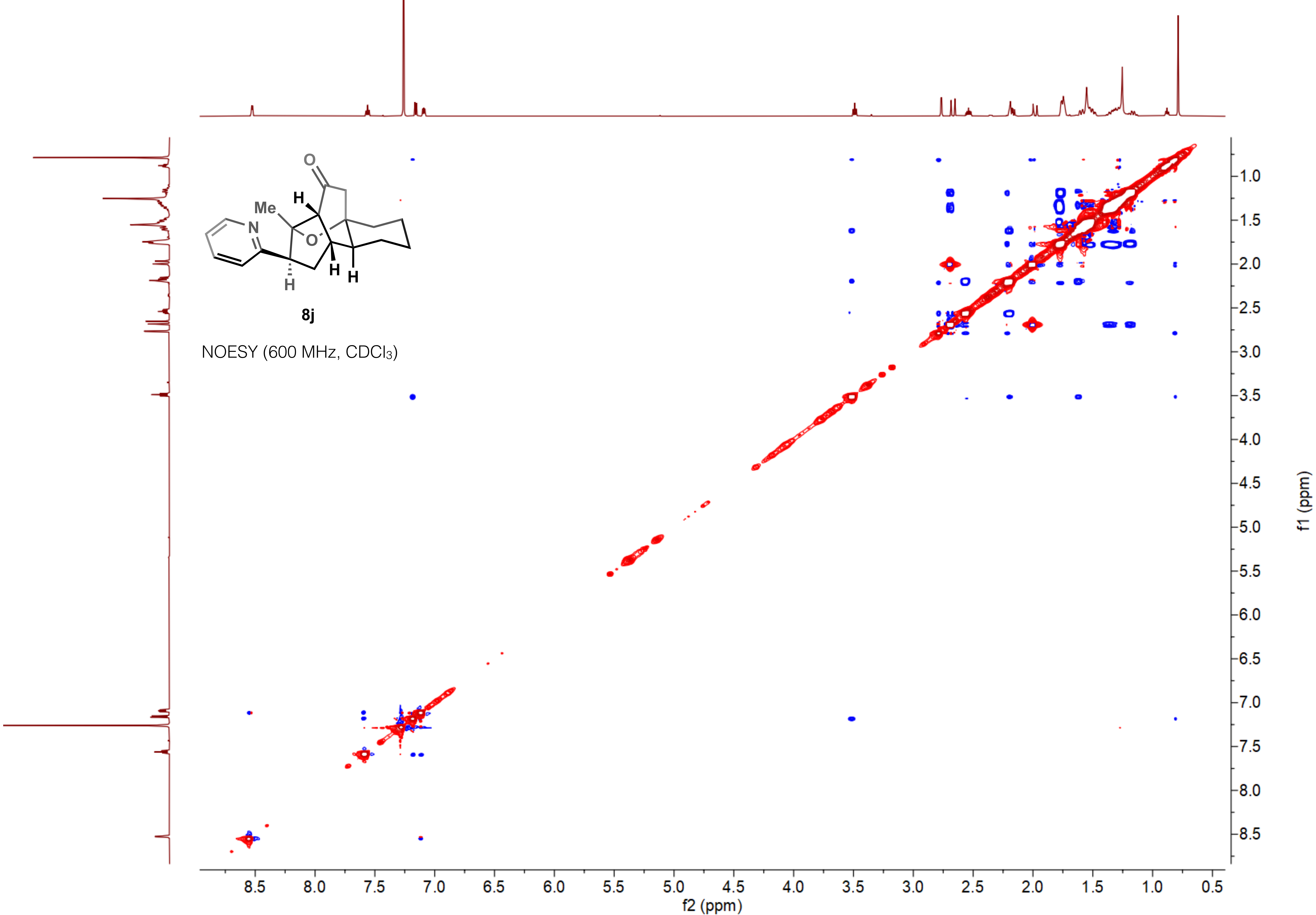

Me
N
O
H
H
H
H
O
8j
NOESY (600 MHz, CDCl3)
f2 (ppm)
f1 (ppm)

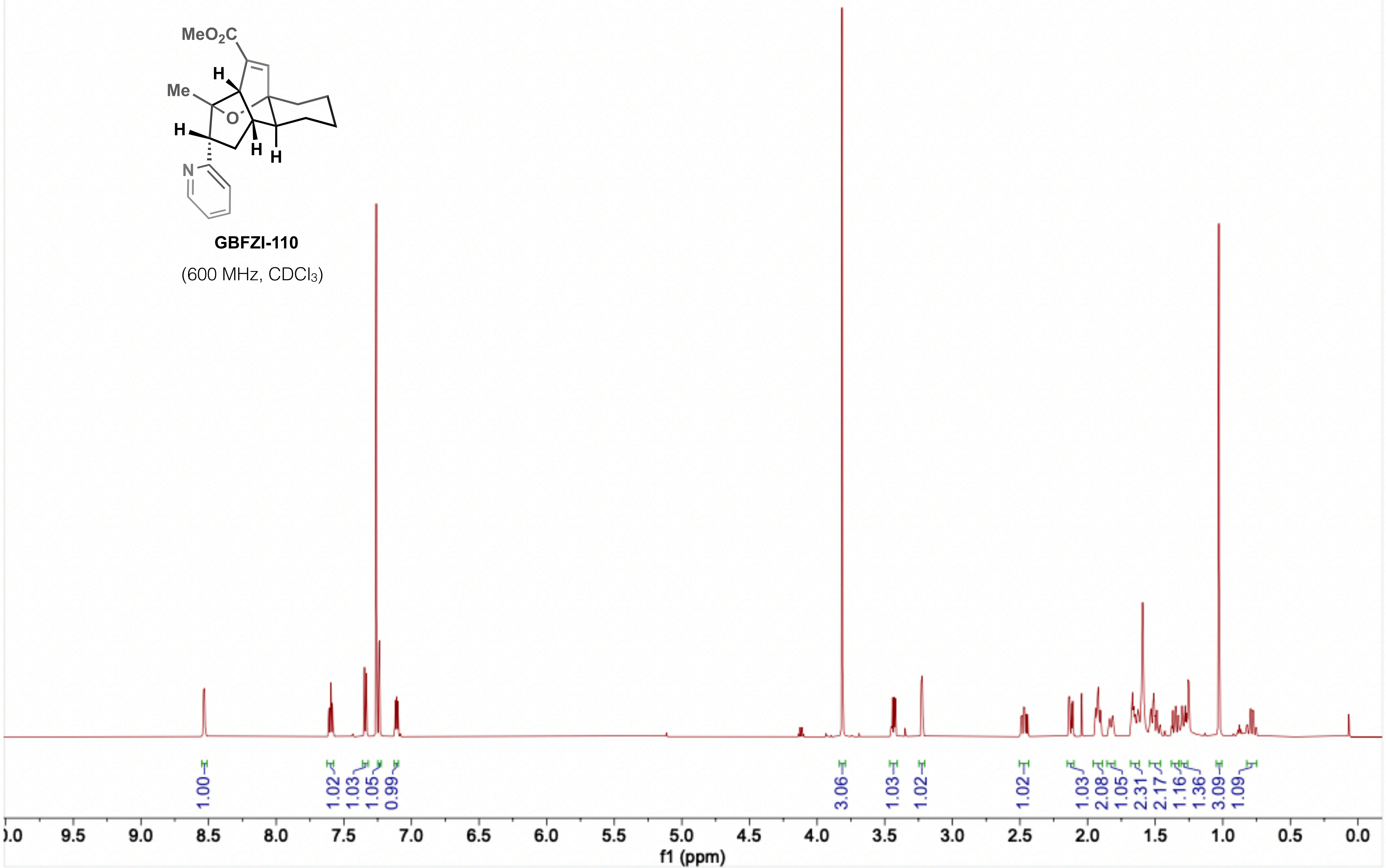
MeO2C
Me
H
O
H
H
H
N
GBFZI-110
(600 MHz, CDCl3)
1.00
1.02
1.03
1.05
0.99
3.06
1.03
1.02
1.02
1.03
2.08
1.05
2.31
2.17
1.16
1.36
3.09
1.09
10.0 9.5 9.0 8.5 8.0 7.5 7.0 6.5 6.0 5.5 5.0 4.5 4.0 3.5 3.0 2.5 2.0 1.5 1.0 0.5 0.0
f1 (ppm)

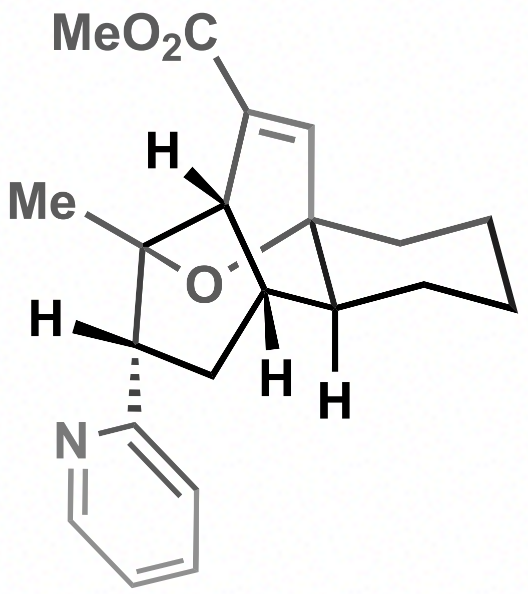

GBFZI-110

(151 MHz, $CDCl_3$)

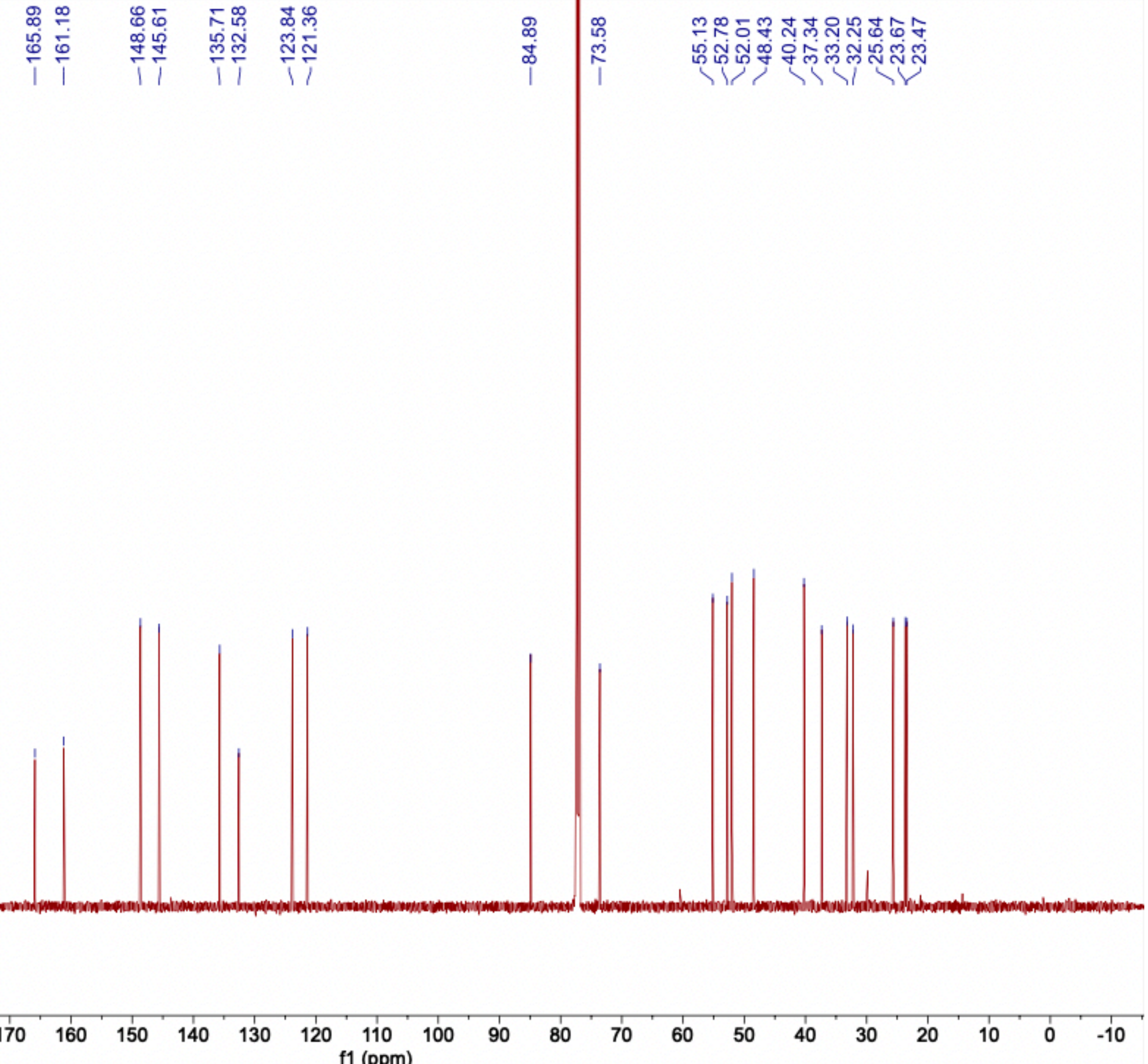

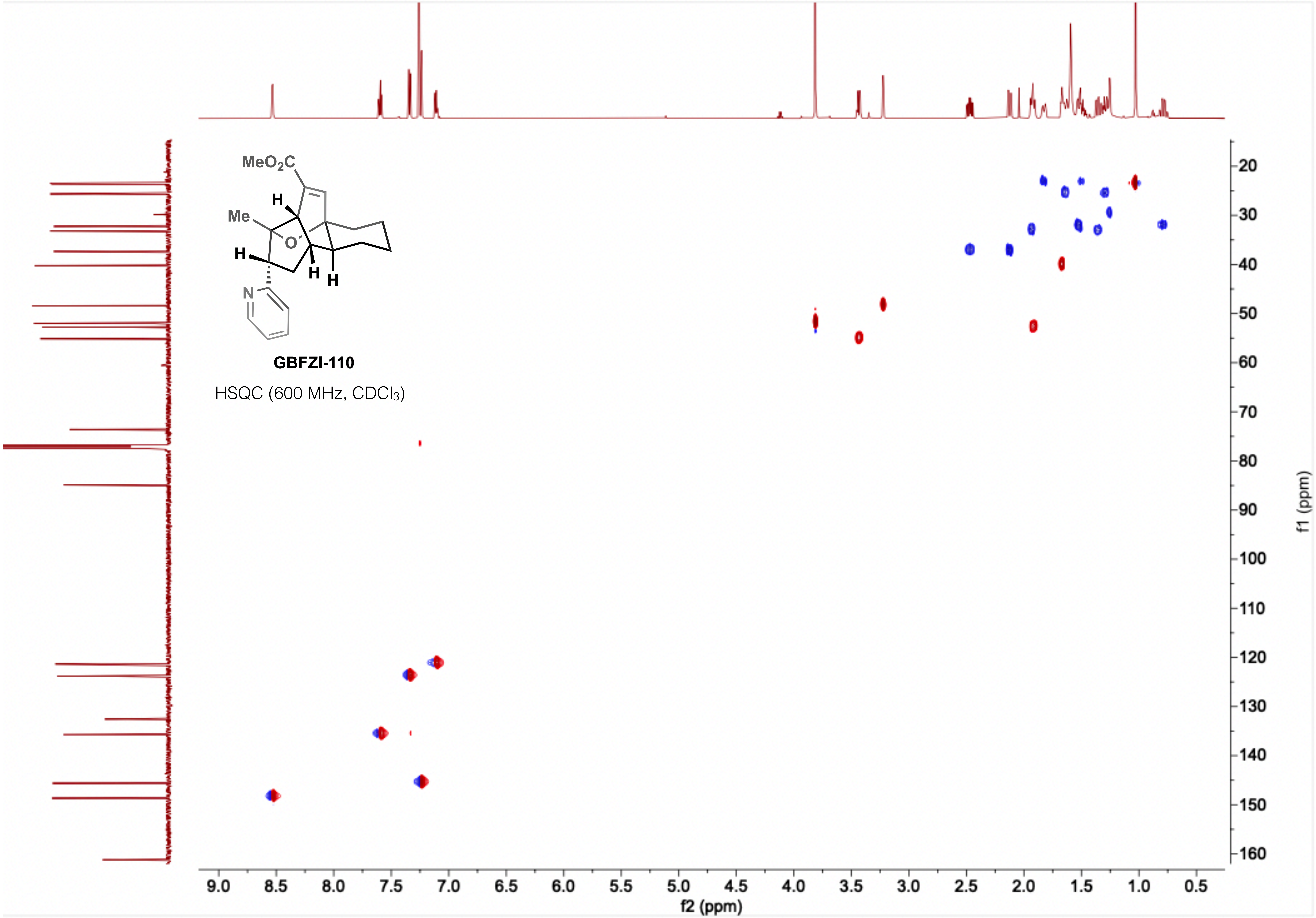
MeO2C
Me
H
O
H
H
H
N
GBFZI-110
HSQC (600 MHz, CDCl3)
f1 (ppm)
f2 (ppm)

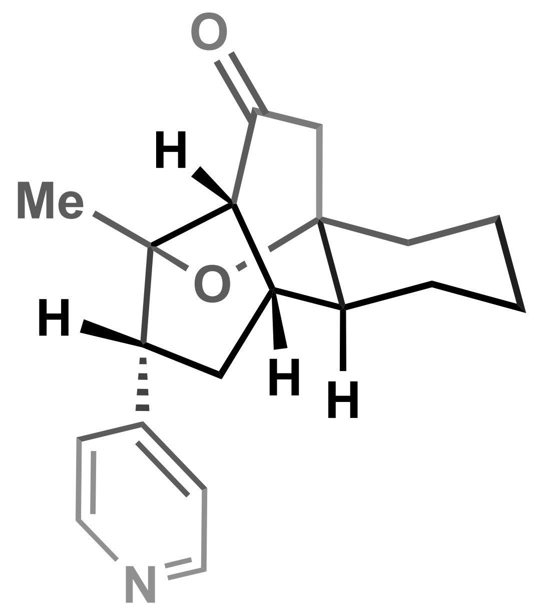

7k

(600 MHz, $CDCl_3$)

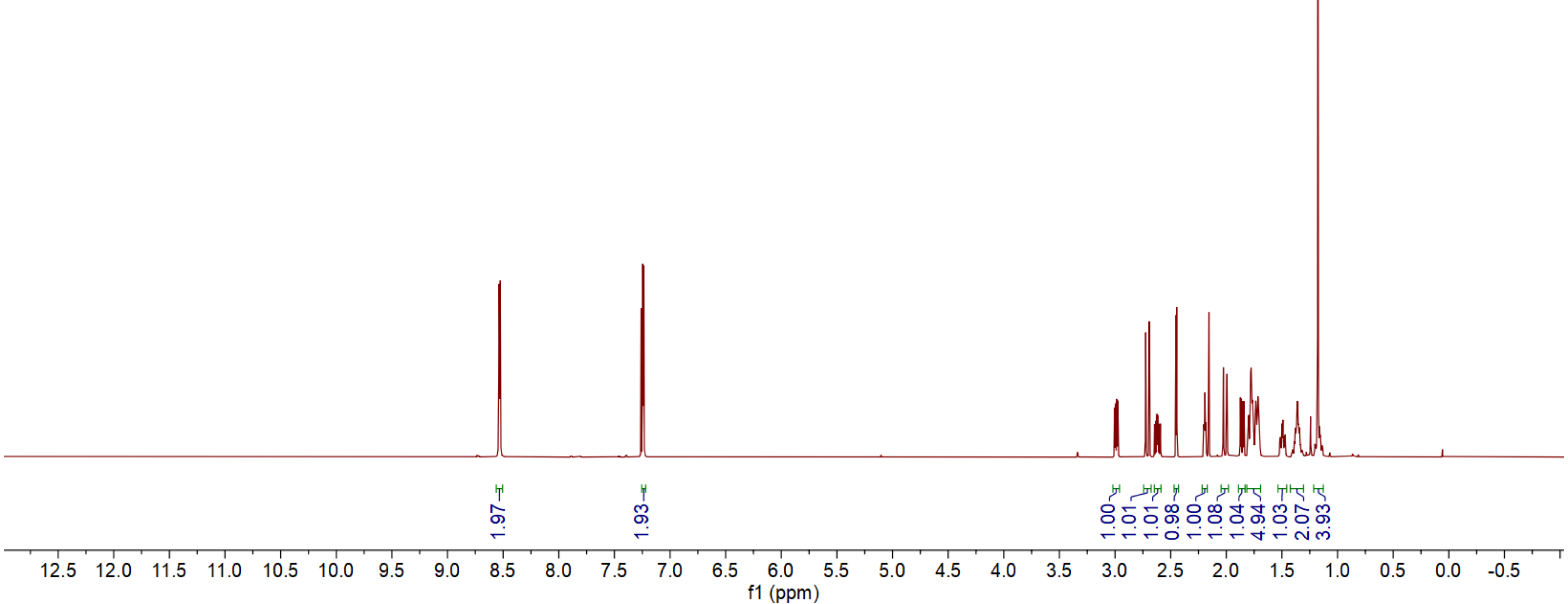

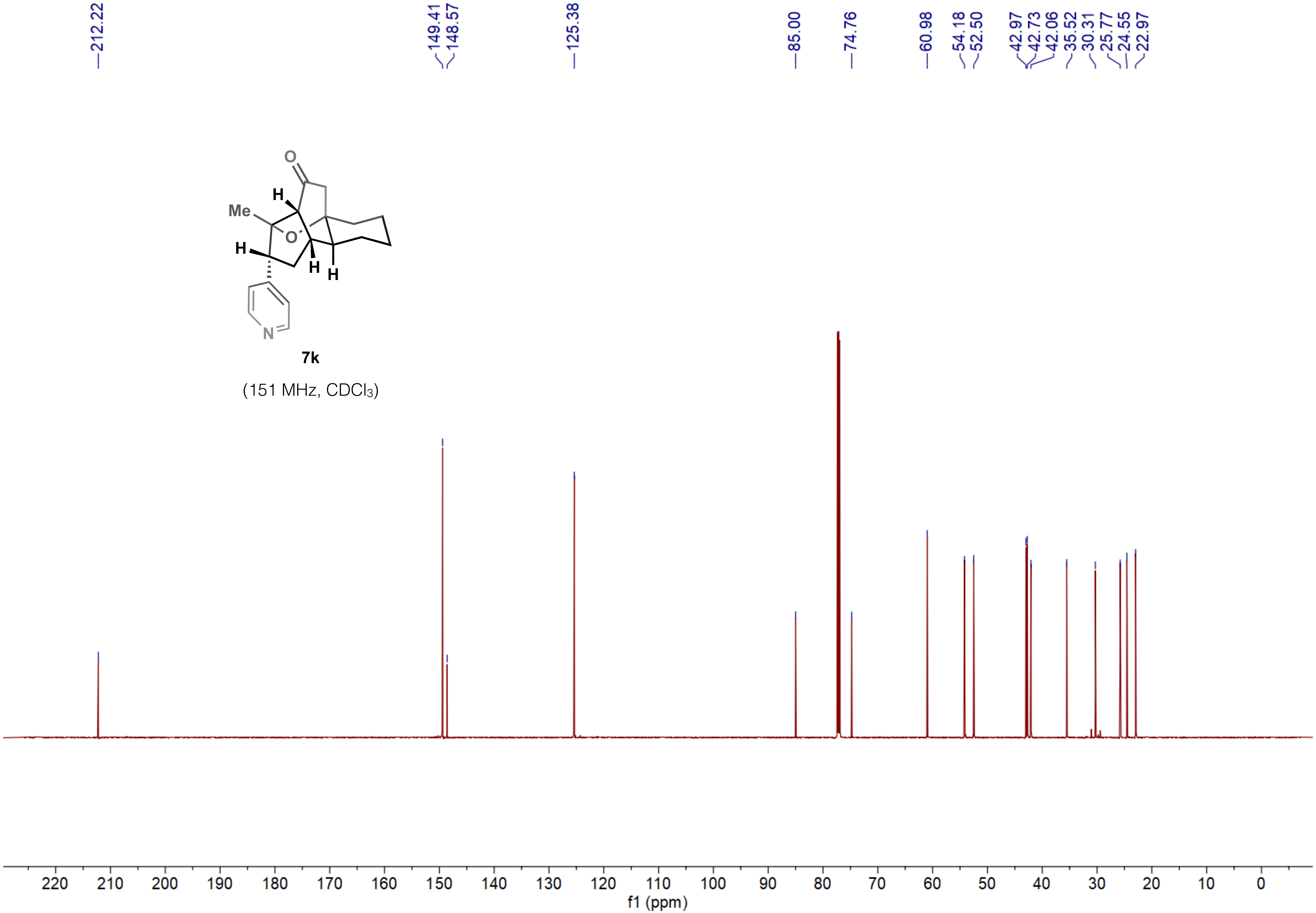
212.22
149.41
148.57
125.38
85.00
74.76
60.98
54.18
52.50
42.97
42.73
42.06
35.52
30.31
25.77
24.55
22.97
Me
O
H
N
7k
(151 MHz, CDCl3)
220 210 200 190 180 170 160 150 140 130 120 110 100 90 80 70 60 50 40 30 20 10 0
f1 (ppm)

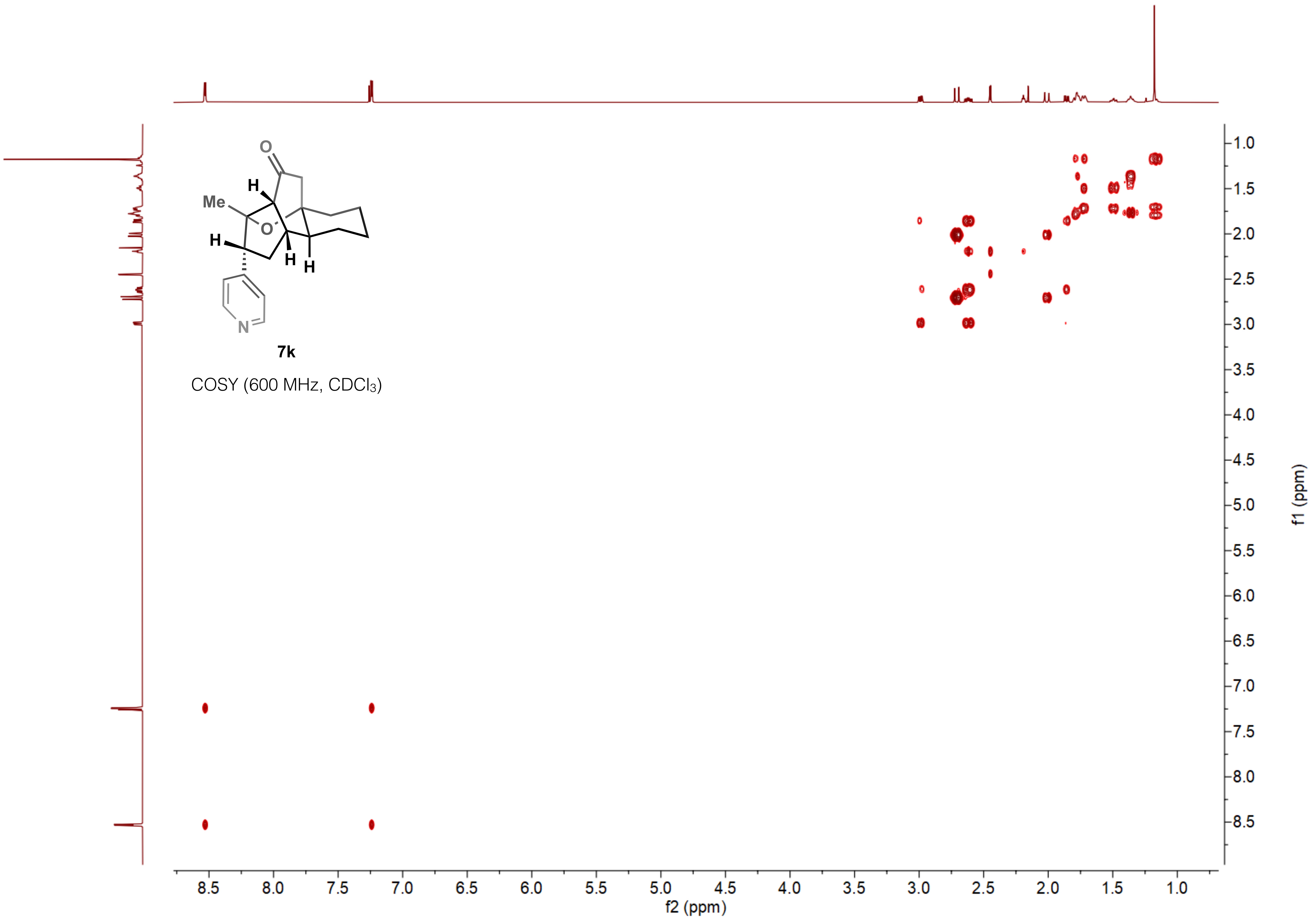
7k
COSY (600 MHz, CDCl3)
f2 (ppm)
f1 (ppm)

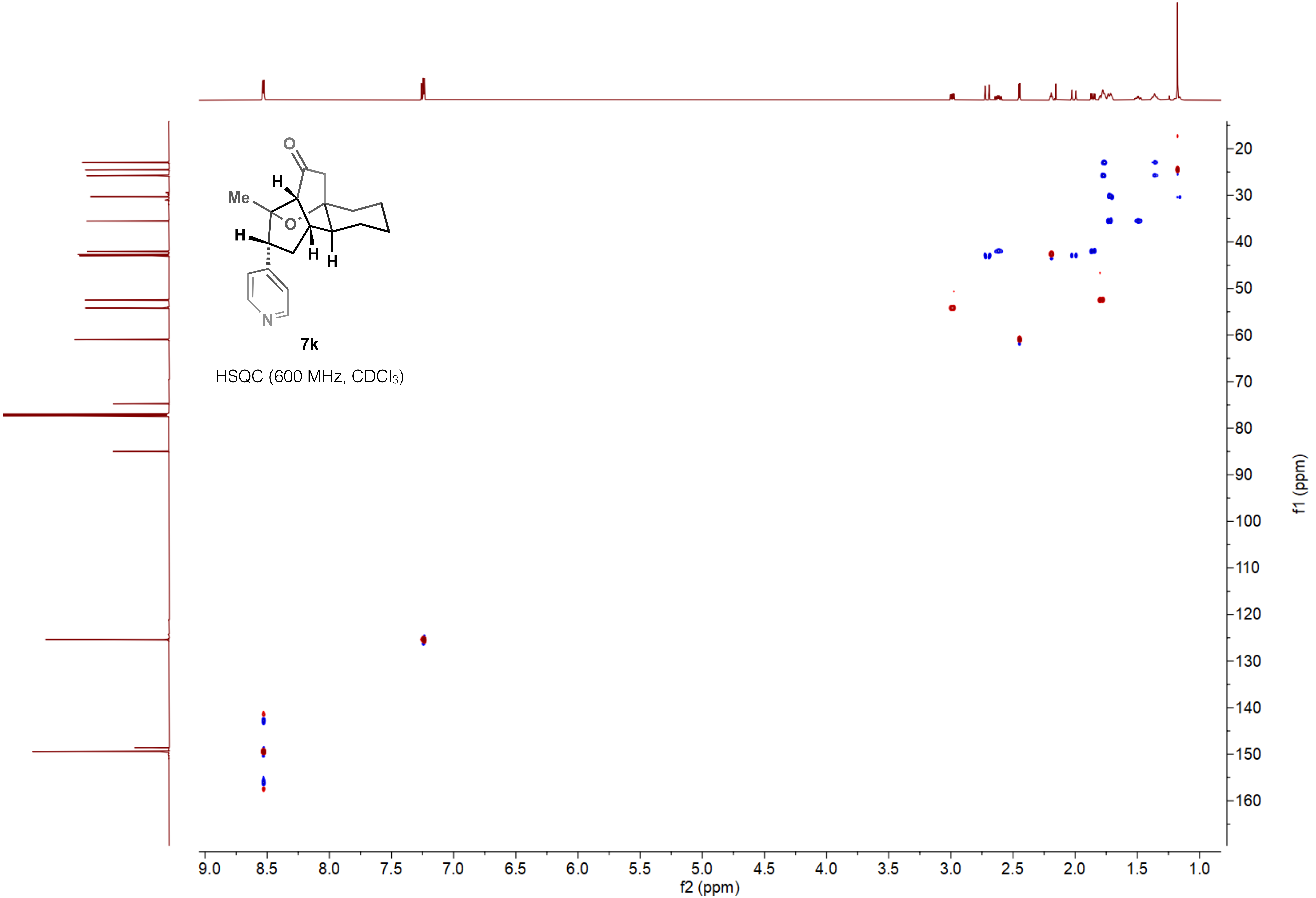

7k

HSQC (600 MHz, $CDCl_3$)

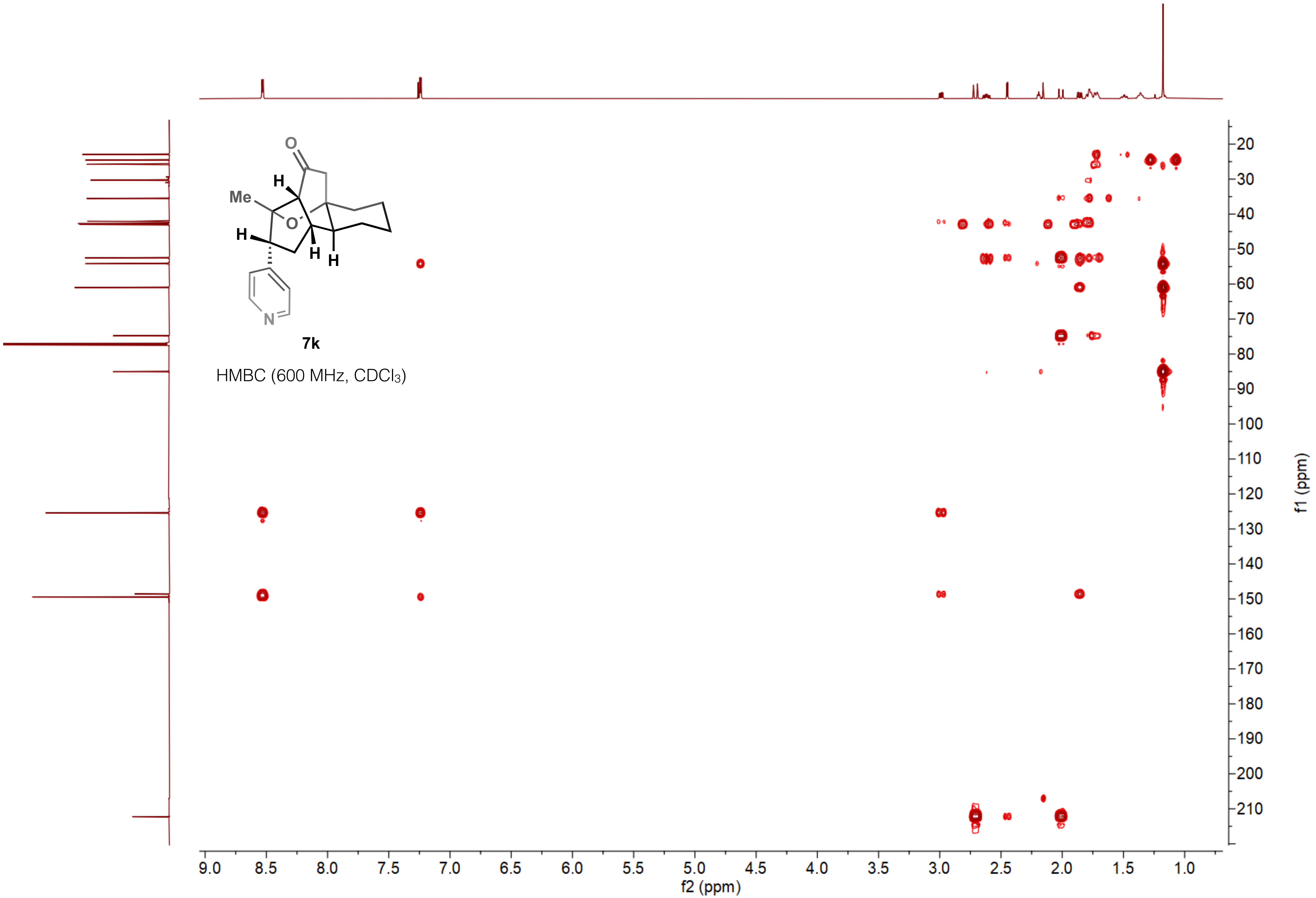
O
H
Me
O
H
H
H
N
7k
HMBC (600 MHz, CDCl3)
f2 (ppm)
f1 (ppm)

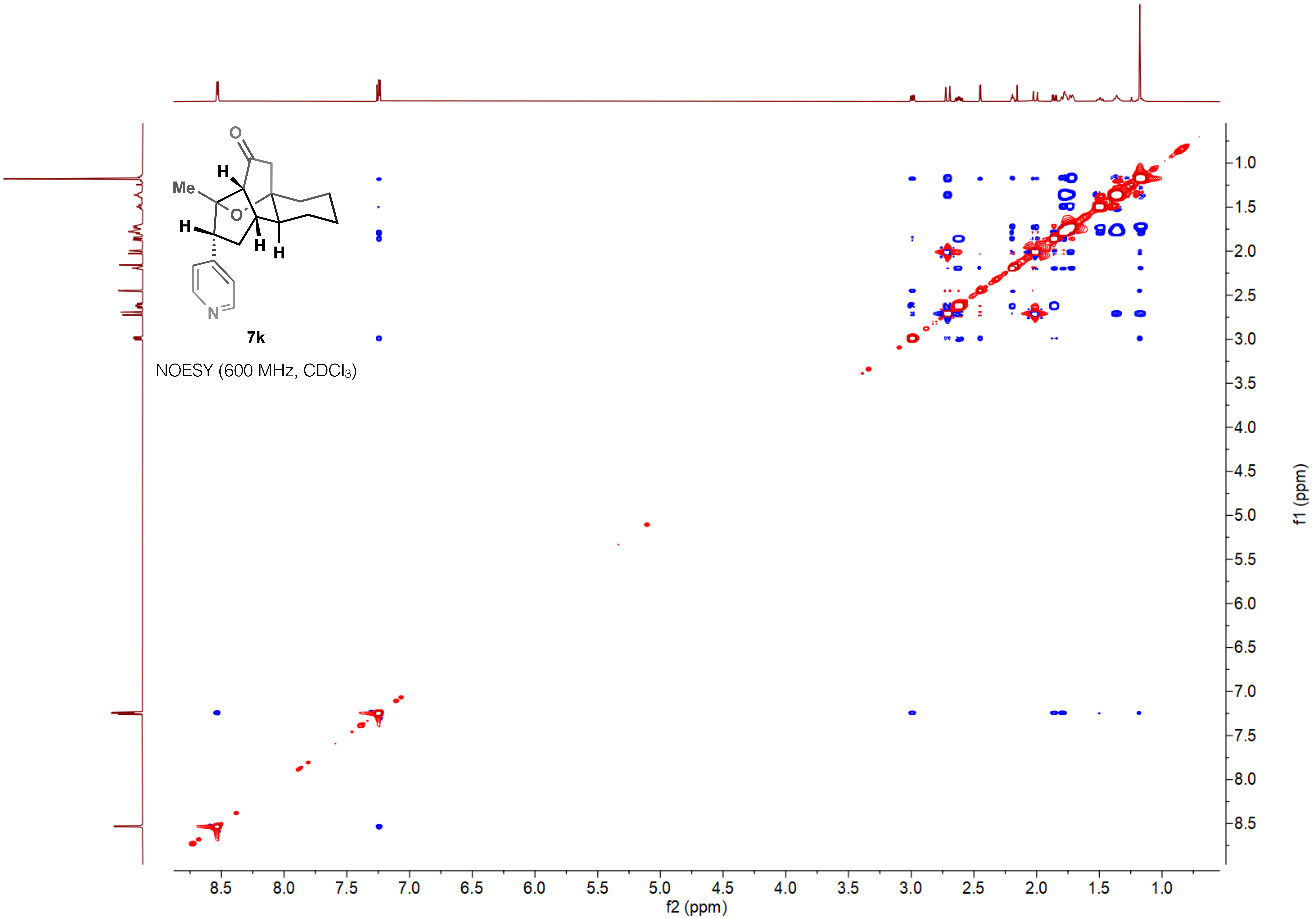

O
H
Me
O
H
H
H
N
7k
NOESY (600 MHz, CDCl3)
f2 (ppm)
f1 (ppm)

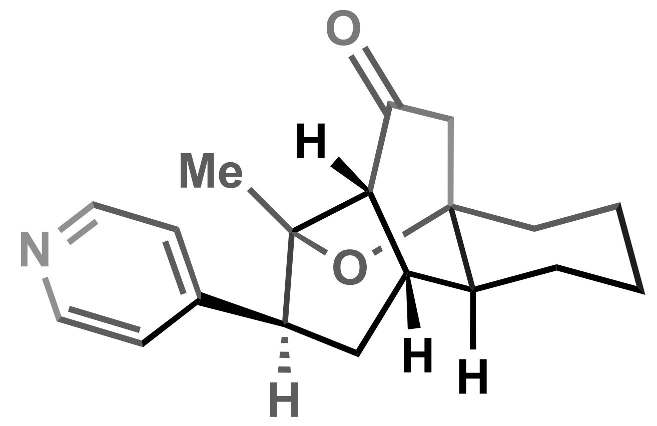

8k

(600 MHz, $CDCl_3$)

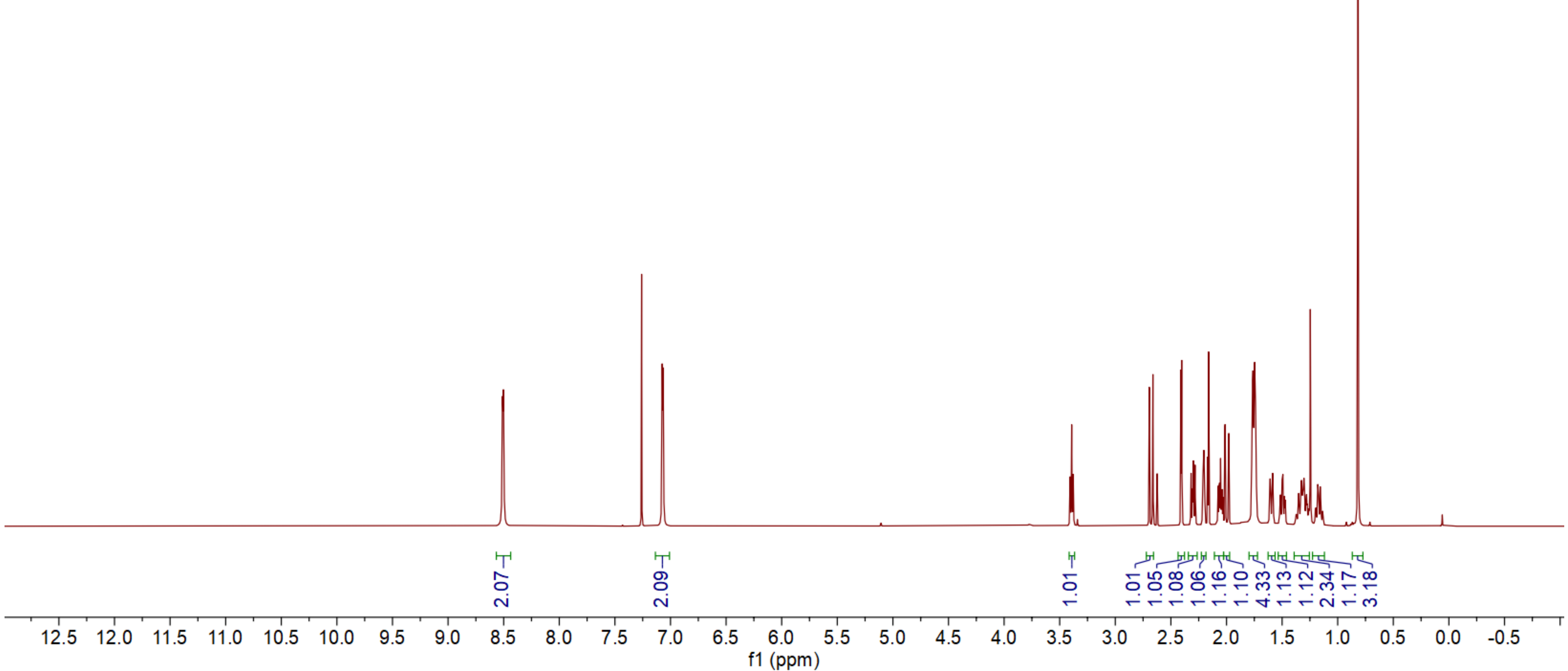

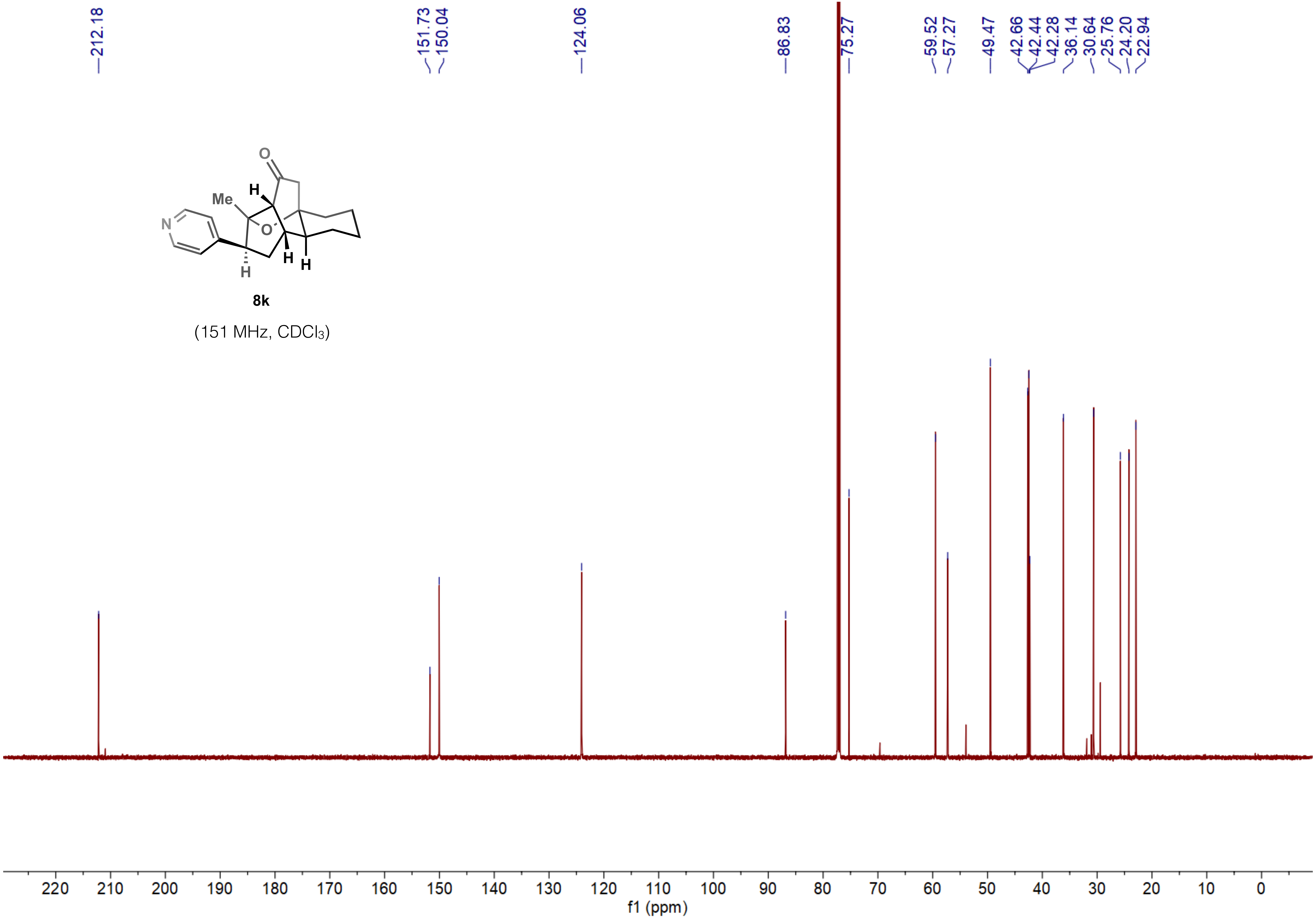

212.18
151.73
150.04
124.06
86.83
75.27
59.52
57.27
49.47
42.66
42.44
42.28
36.14
30.64
25.76
24.20
22.94
O
Me
H
N
O
H
H
H
8k
(151 MHz, CDCl3)
220 210 200 190 180 170 160 150 140 130 120 110 100 90 80 70 60 50 40 30 20 10 0
f1 (ppm)

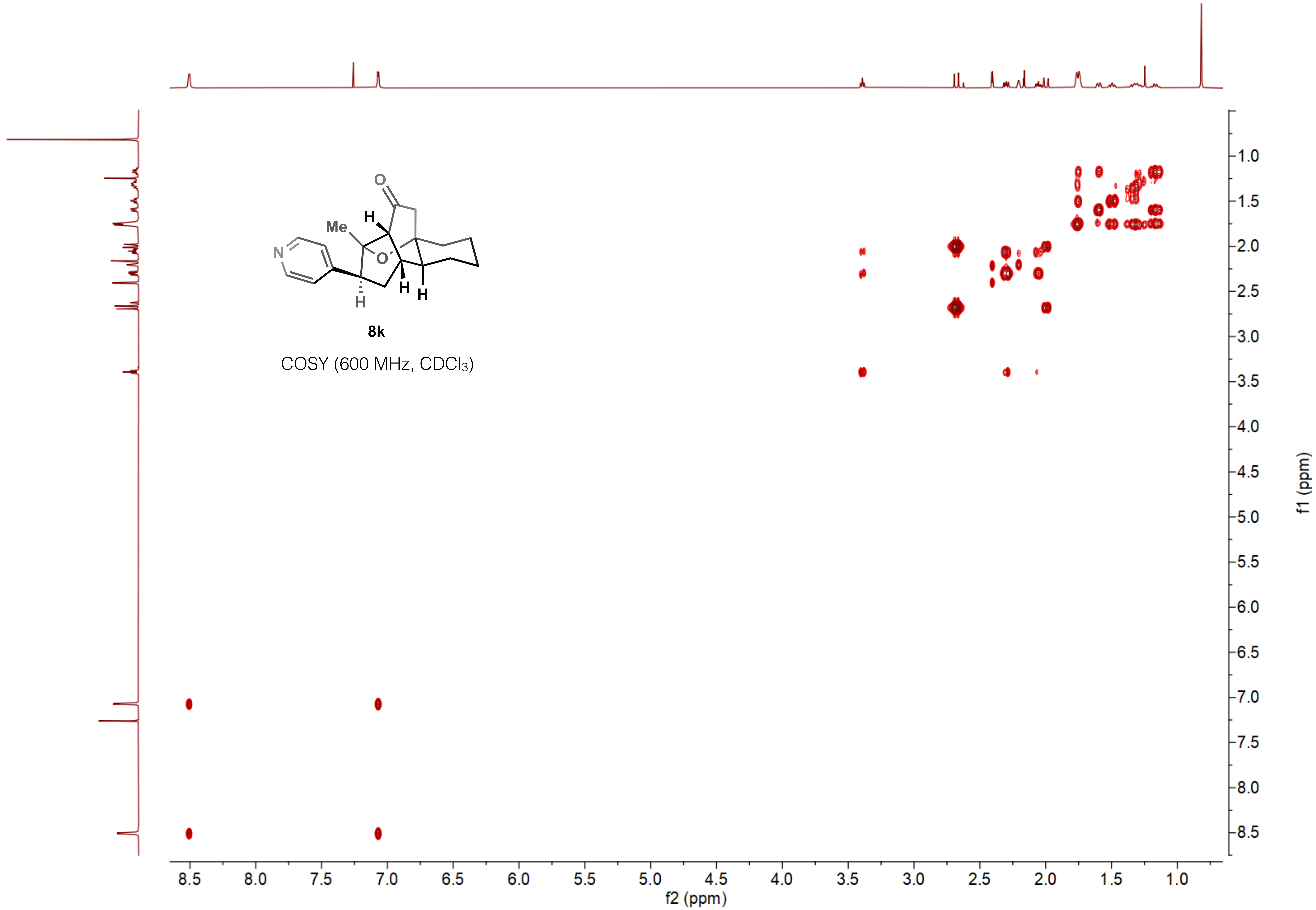

Me
H
O
N
O
H
H
H
8k
COSY (600 MHz, CDCl3)
f2 (ppm)
f1 (ppm)

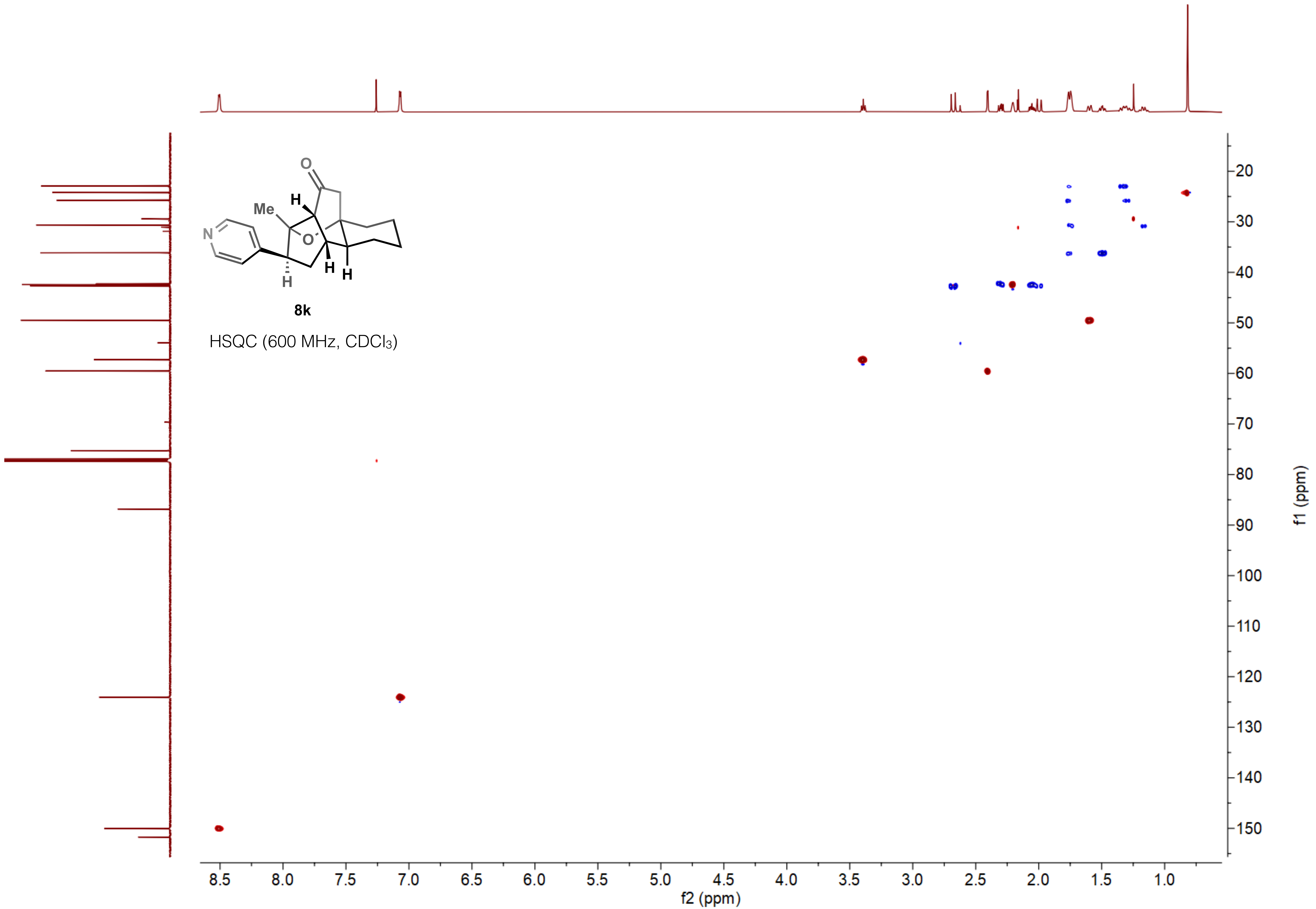
Me
H
N
O
H
H
H
O
8k
HSQC (600 MHz, CDCl3)
f2 (ppm)
f1 (ppm)

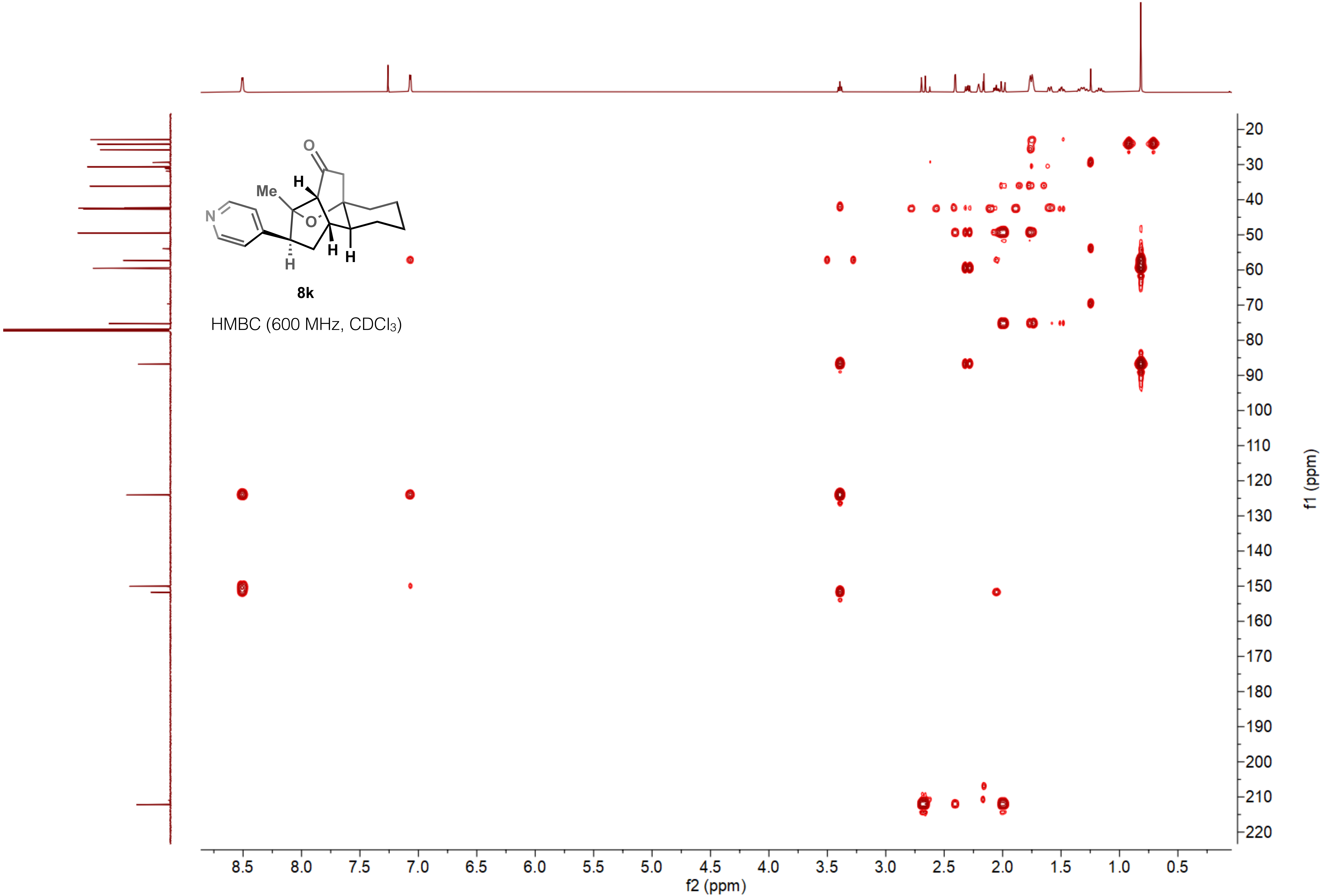

O
Me
H
N
O
H
H
H
8k
HMBC (600 MHz, CDCl3)
f2 (ppm)
f1 (ppm)

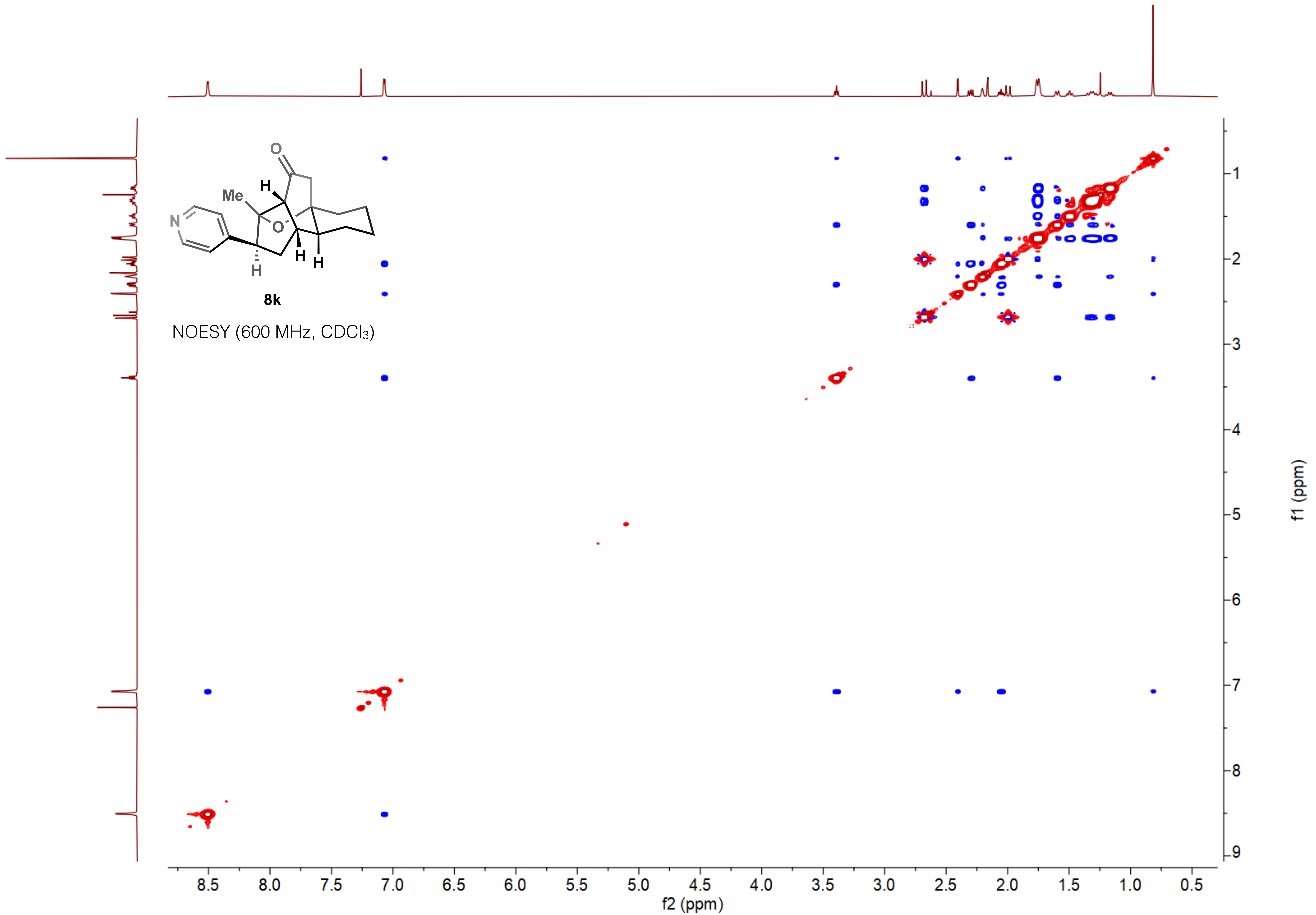

8k
NOESY (600 MHz, CDCl3)
f2 (ppm)
f1 (ppm)

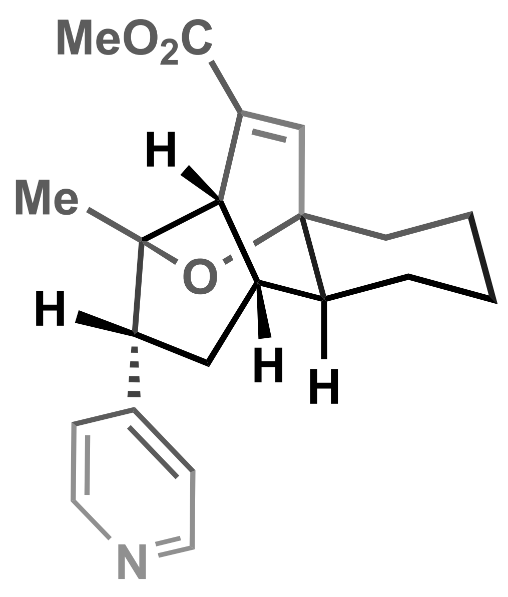

RS1130

(600 MHz, $CDCl_3$)

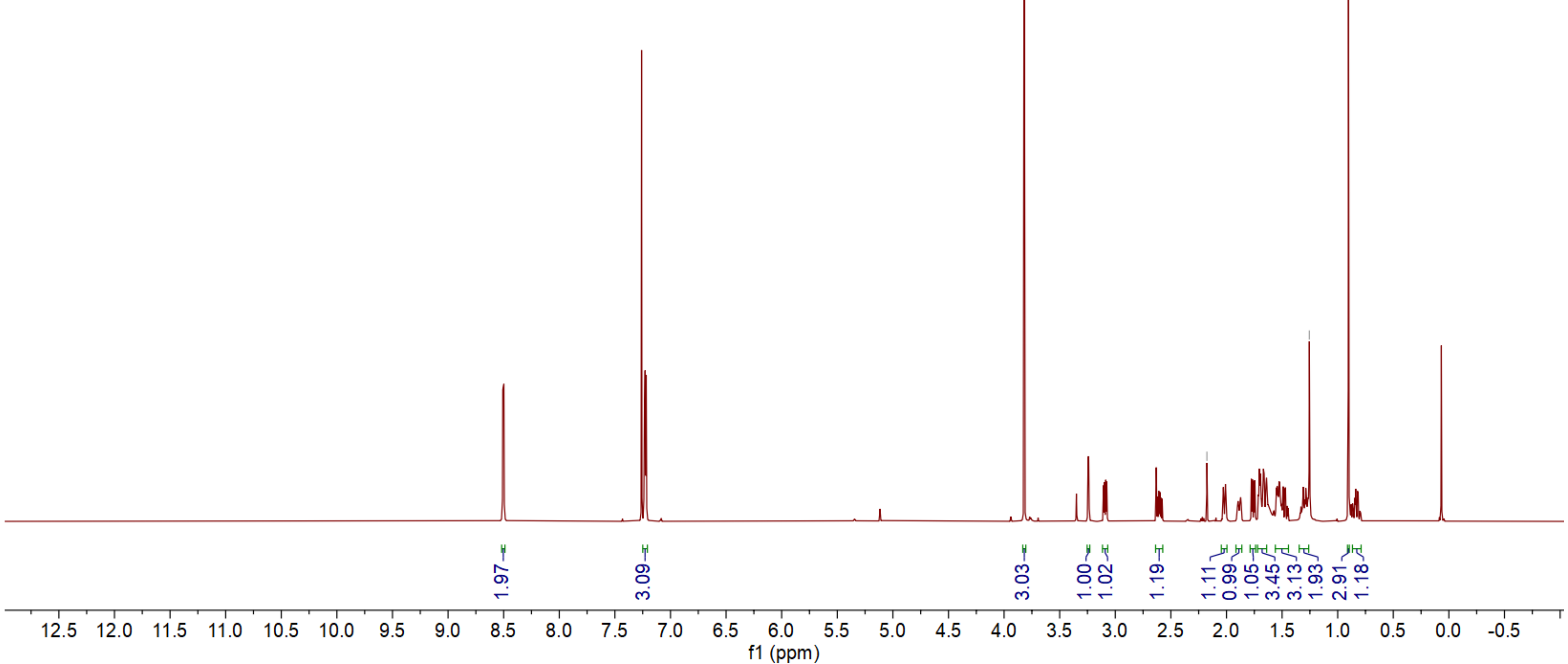

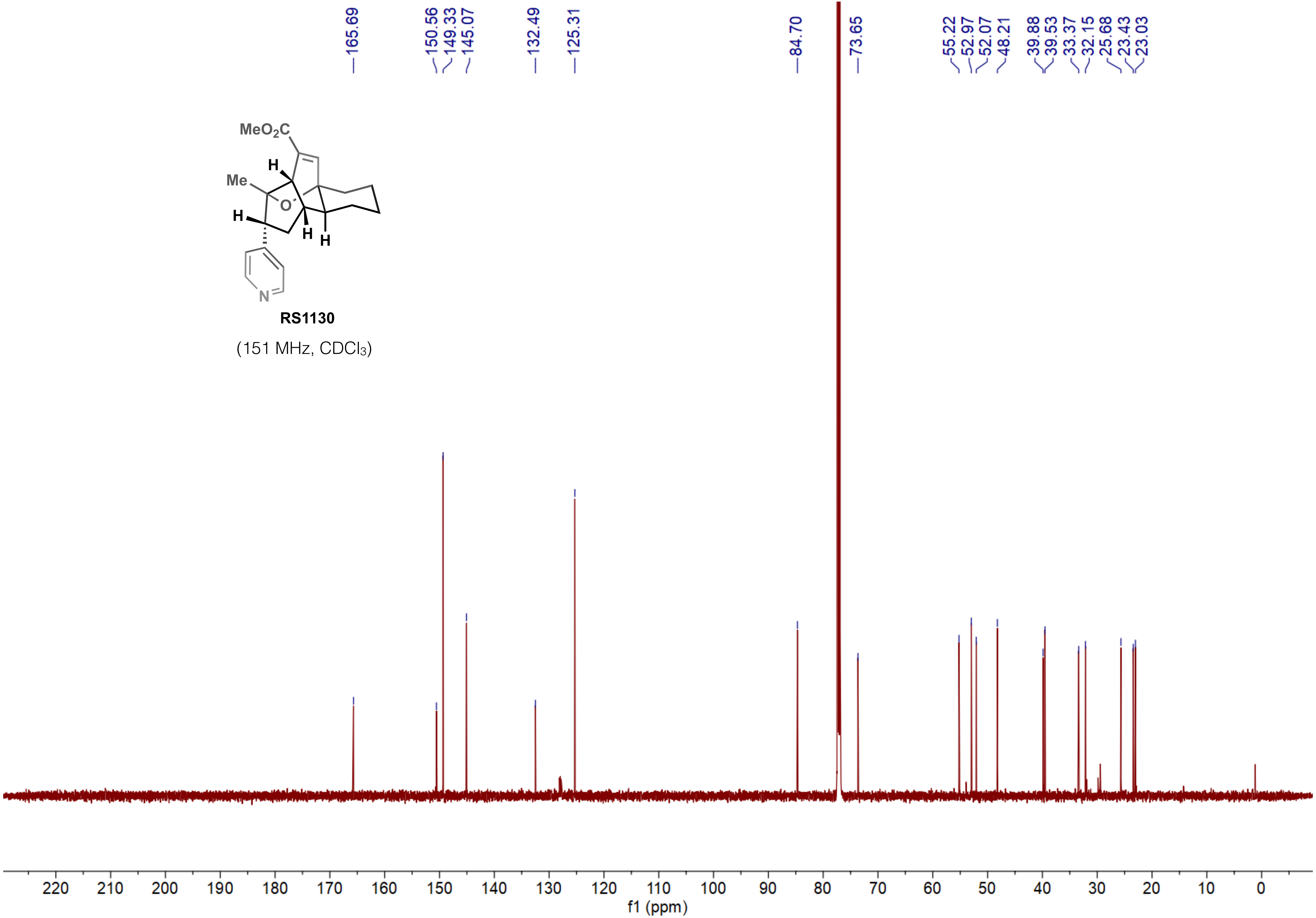
165.69
150.56
149.33
145.07
132.49
125.31
84.70
73.65
55.22
52.97
52.07
48.21
39.88
39.53
33.37
32.15
25.68
23.43
23.03
MeO2C
Me
H
O
N
RS1130
(151 MHz, CDCl3)
220
210
200
190
180
170
160
150
140
130
120
110
100
90
80
70
60
50
40
30
20
10
0
f1 (ppm)

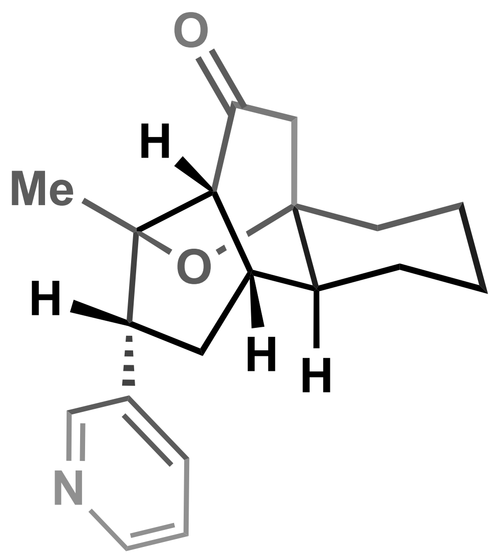

7l

(600 MHz, $CDCl_3$)

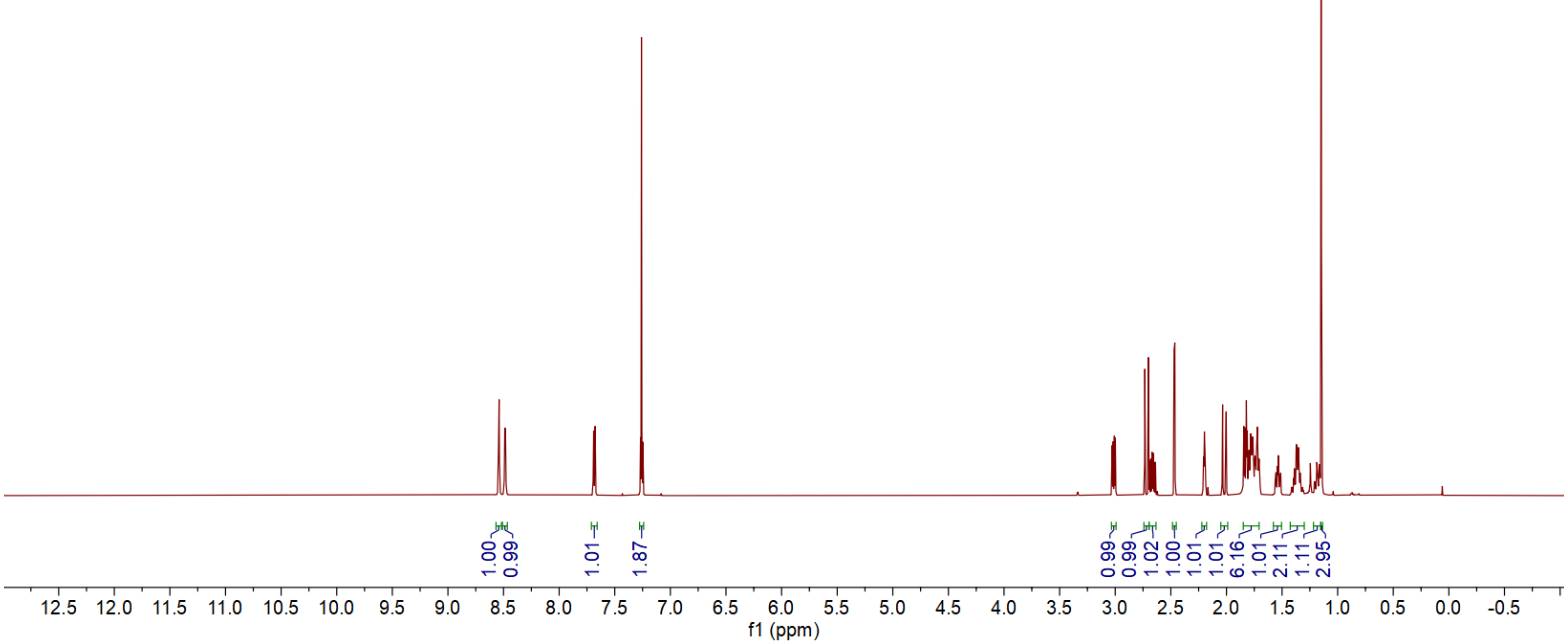

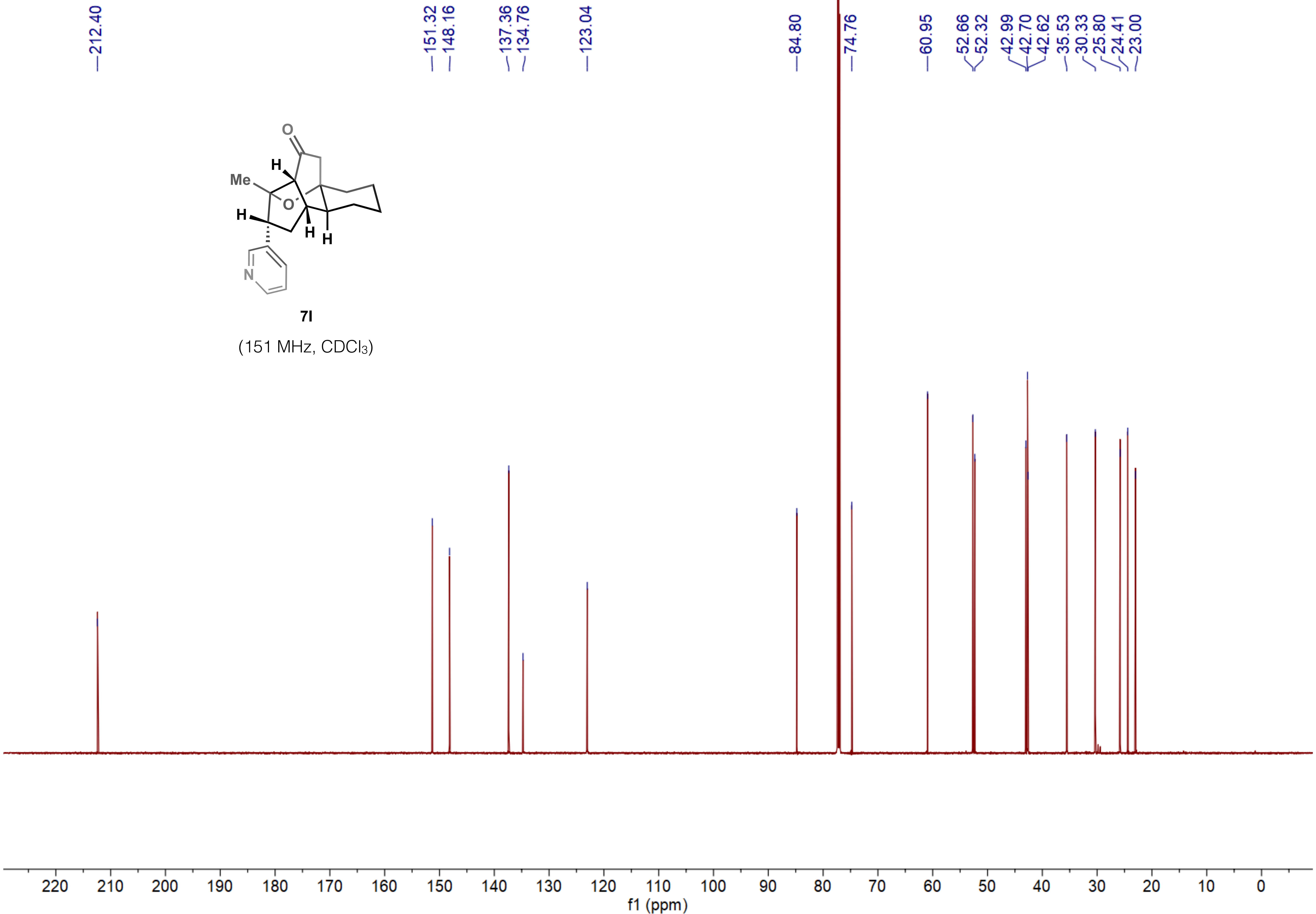
212.40
151.32
148.16
137.36
134.76
123.04
84.80
74.76
60.95
52.66
52.32
42.99
42.70
42.62
35.53
30.33
25.80
24.41
23.00
O
Me
H
O
H
H
H
N
7l
(151 MHz, CDCl3)
220 210 200 190 180 170 160 150 140 130 120 110 100 90 80 70 60 50 40 30 20 10 0
f1 (ppm)

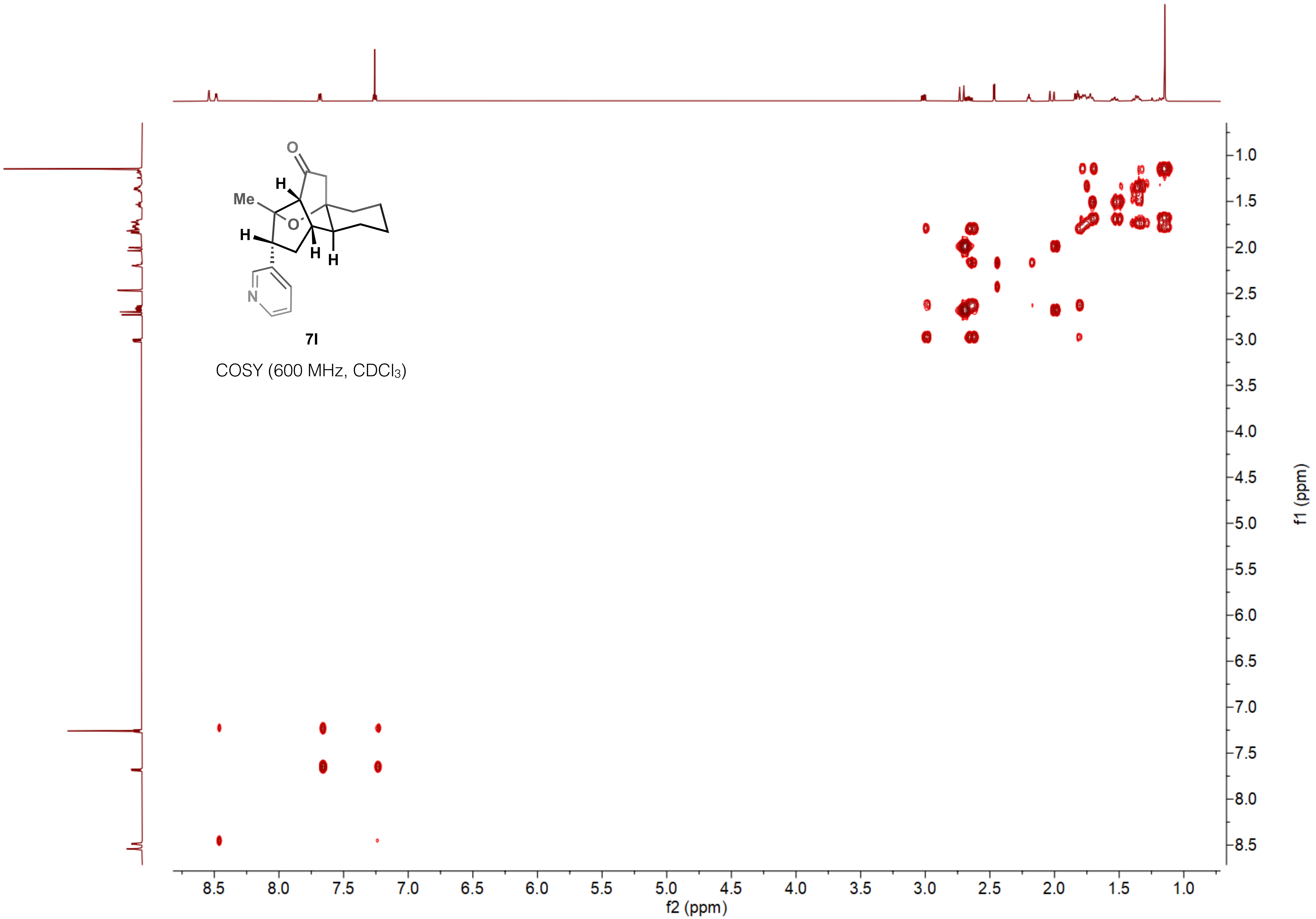

7l

COSY (600 MHz, $CDCl_3$)

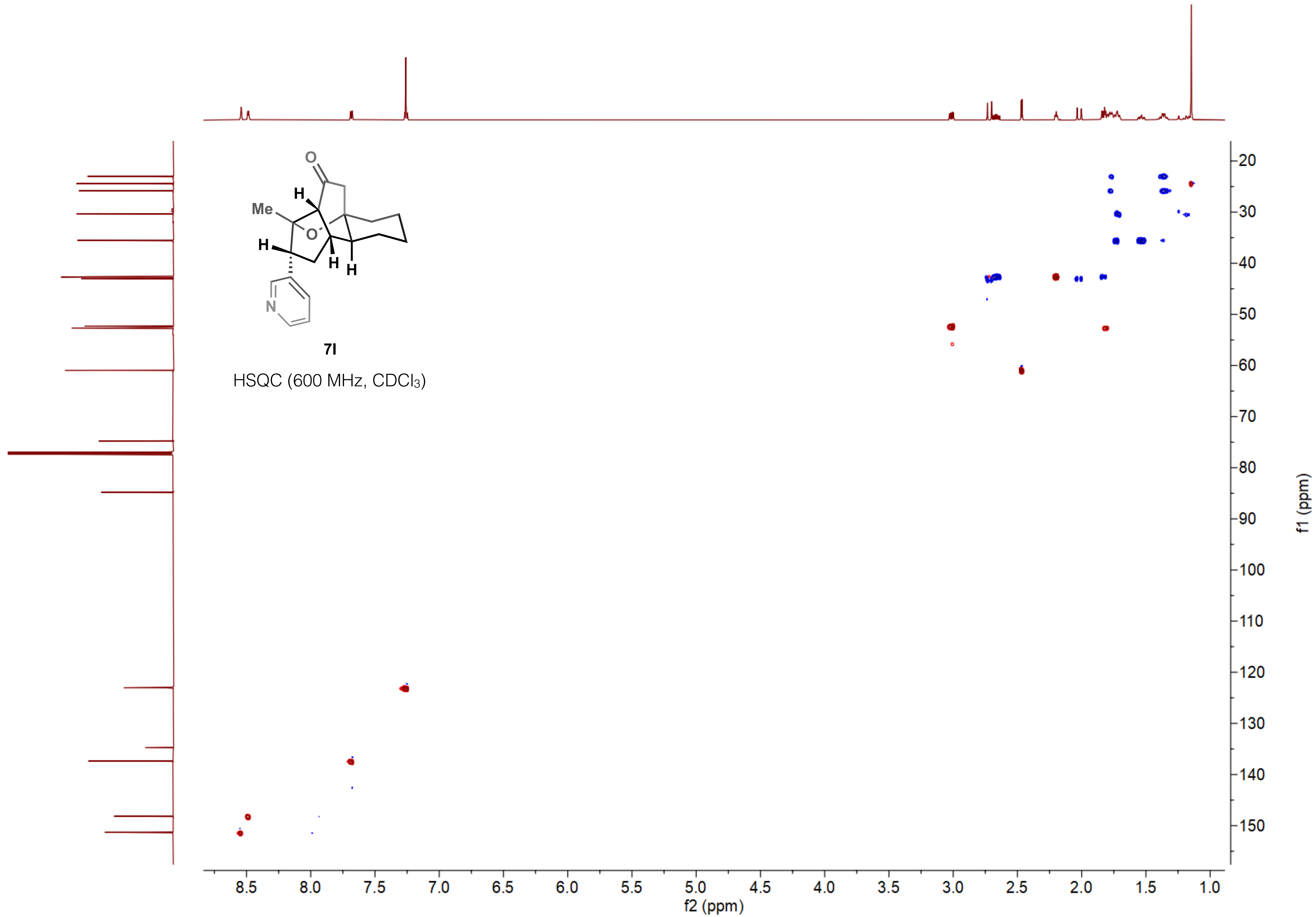
O
Me
H
O
H
H
H
N
7l
HSQC (600 MHz, CDCl3)
f2 (ppm)
f1 (ppm)
8.5
8.0
7.5
7.0
6.5
6.0
5.5
5.0
4.5
4.0
3.5
3.0
2.5
2.0
1.5
1.0
20
30
40
50
60
70
80
90
100
110
120
130
140
150

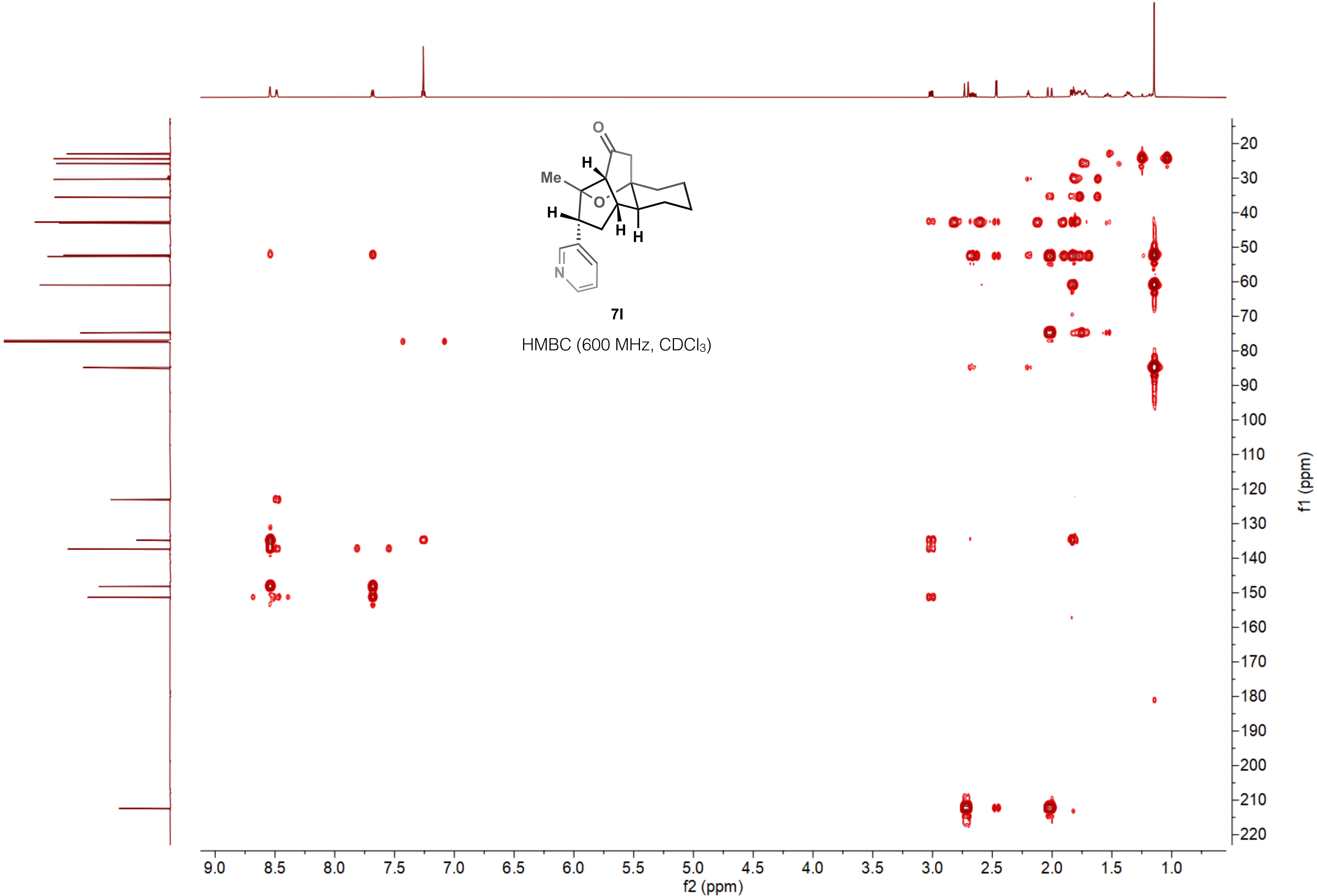
O
Me
H
O
H
H
H
N
7l
HMBC (600 MHz, CDCl3)
f2 (ppm)
f1 (ppm)

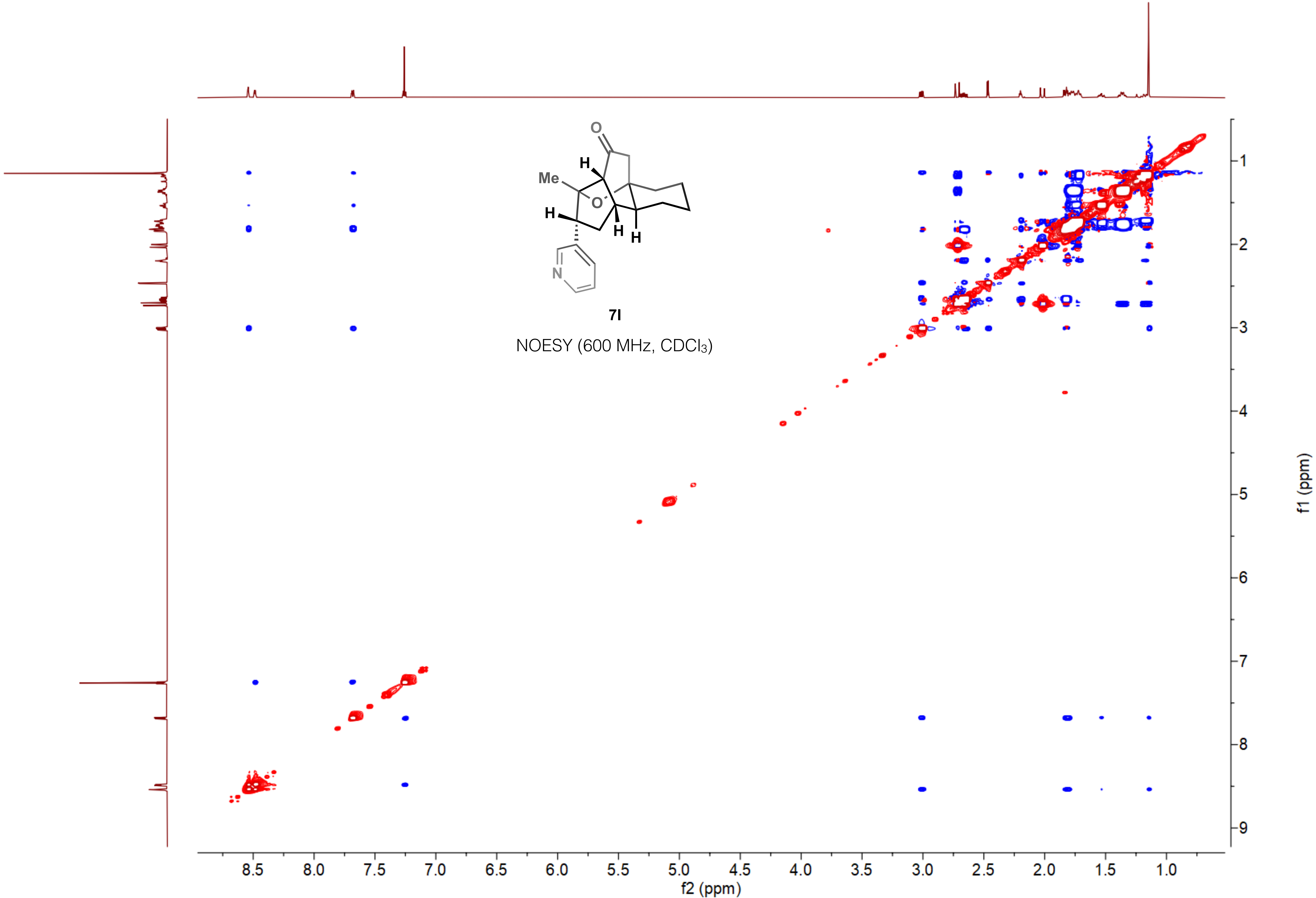
O
Me
O
H
H
H
H
N
7l
NOESY (600 MHz, CDCl3)
f2 (ppm)
f1 (ppm)
8.5 8.0 7.5 7.0 6.5 6.0 5.5 5.0 4.5 4.0 3.5 3.0 2.5 2.0 1.5 1.0
1 2 3 4 5 6 7 8 9

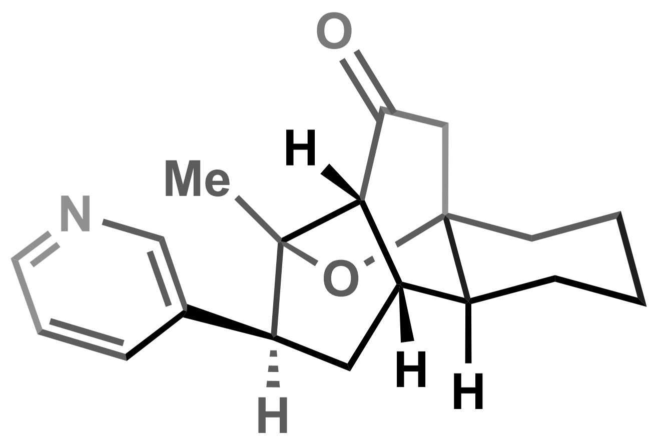

8l

(600 MHz, $CDCl_3$)

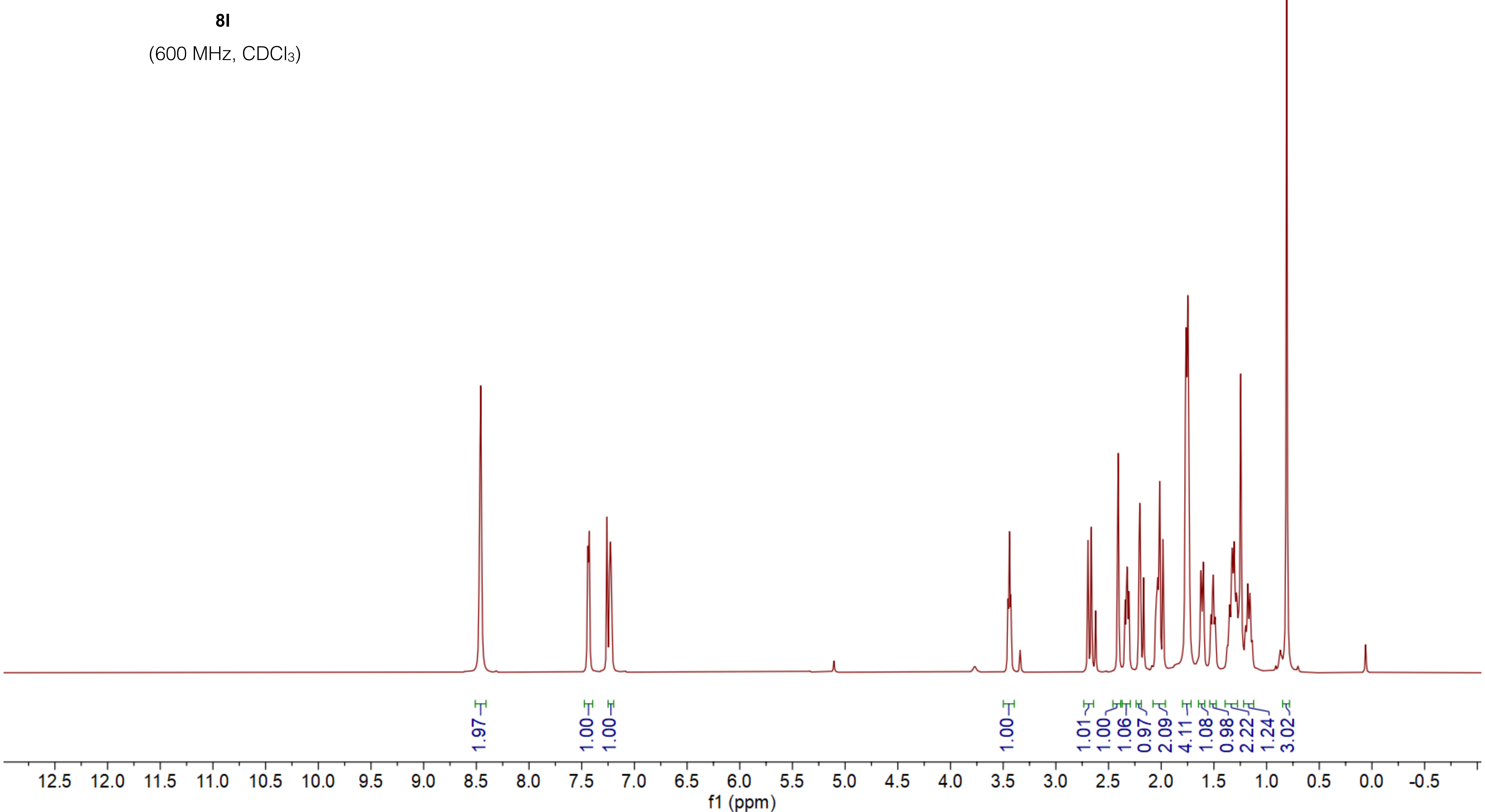

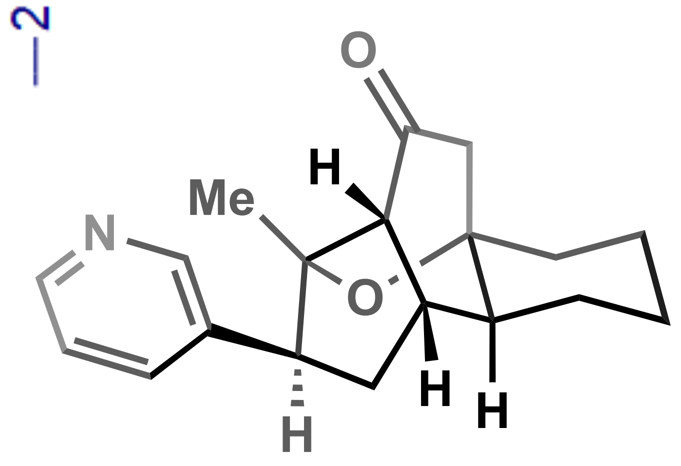

8l

(151 MHz, $CDCl_3$)

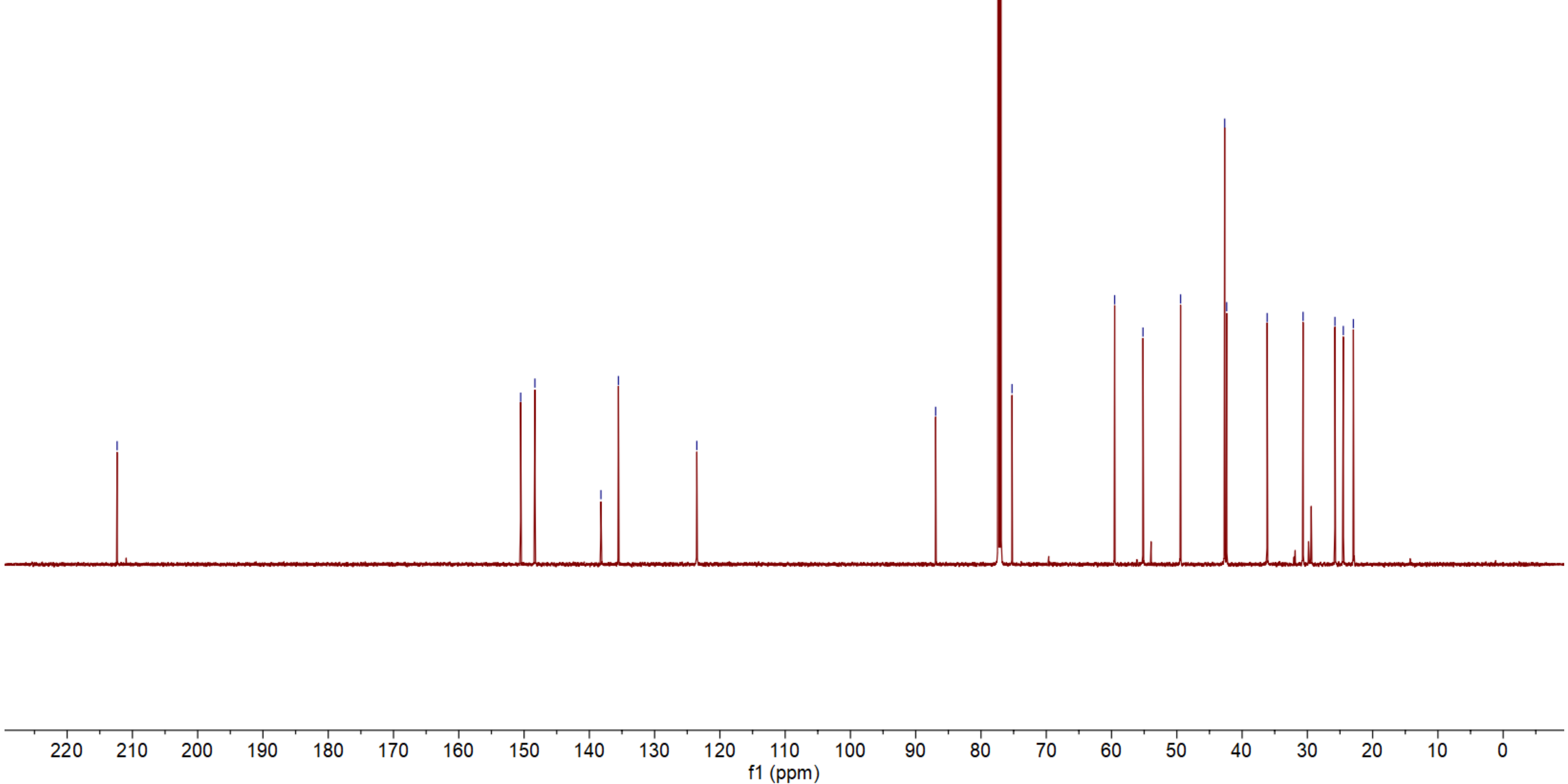

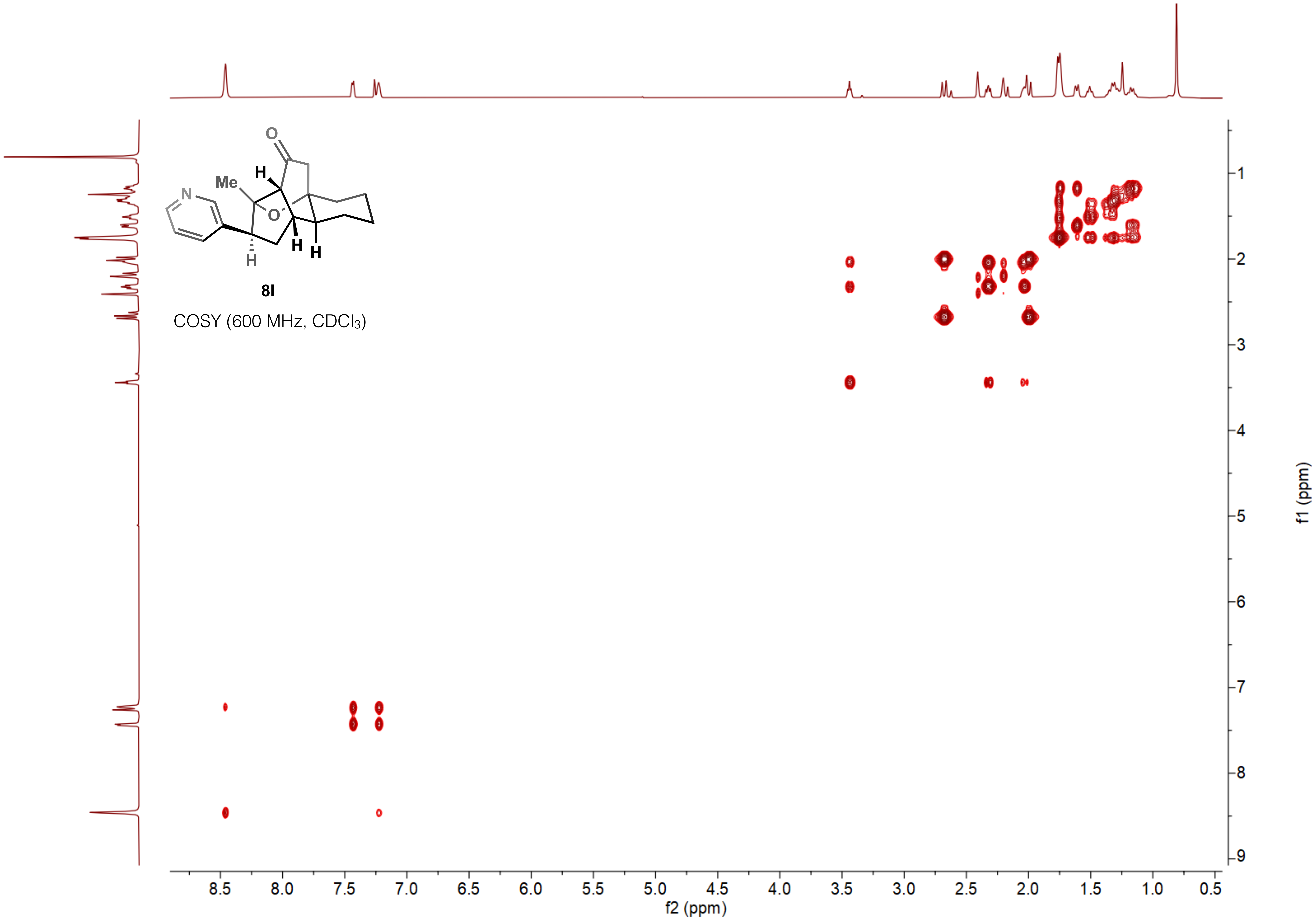

8l

COSY (600 MHz, $CDCl_3$)

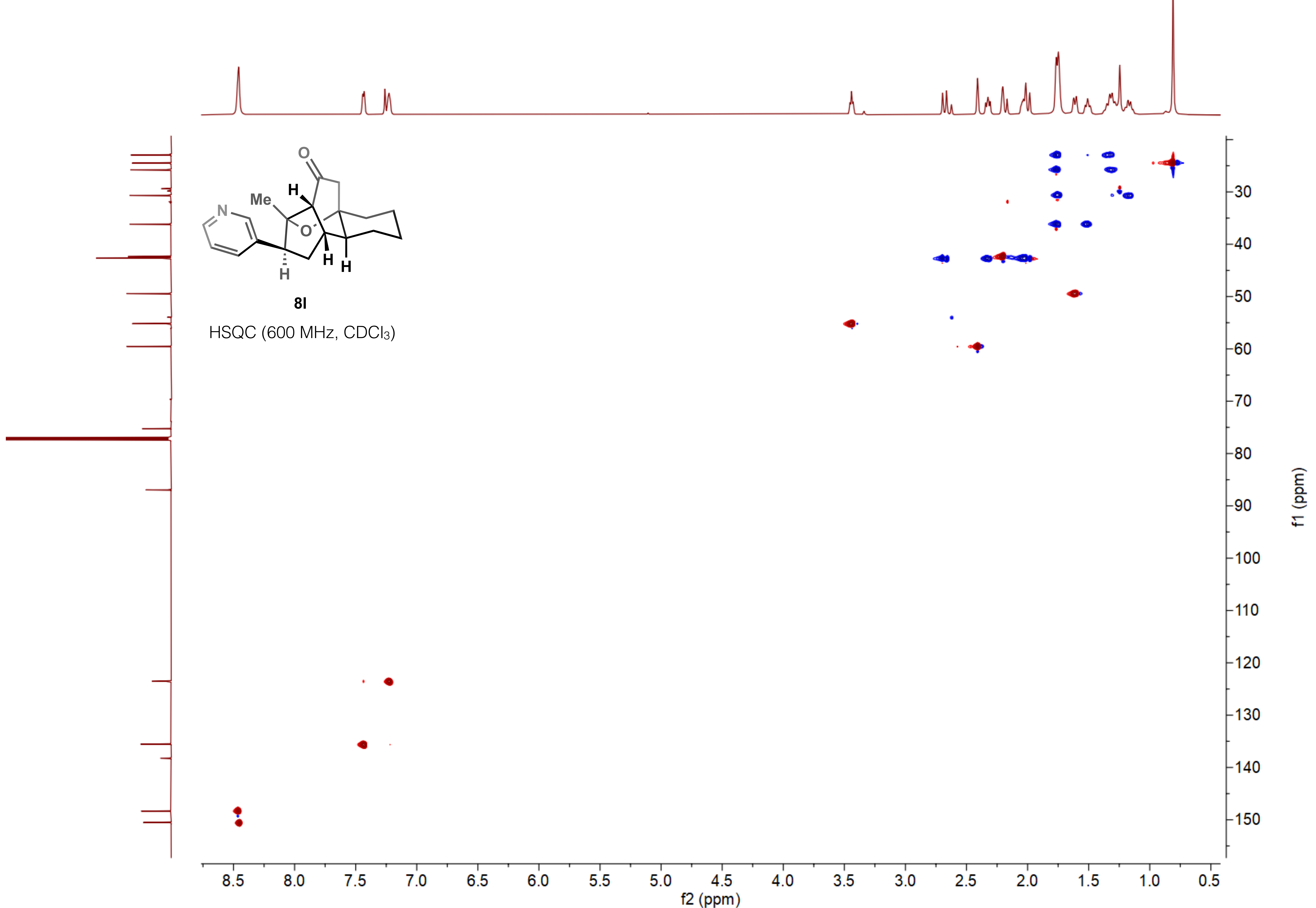

8l

HSQC (600 MHz, $CDCl_3$)

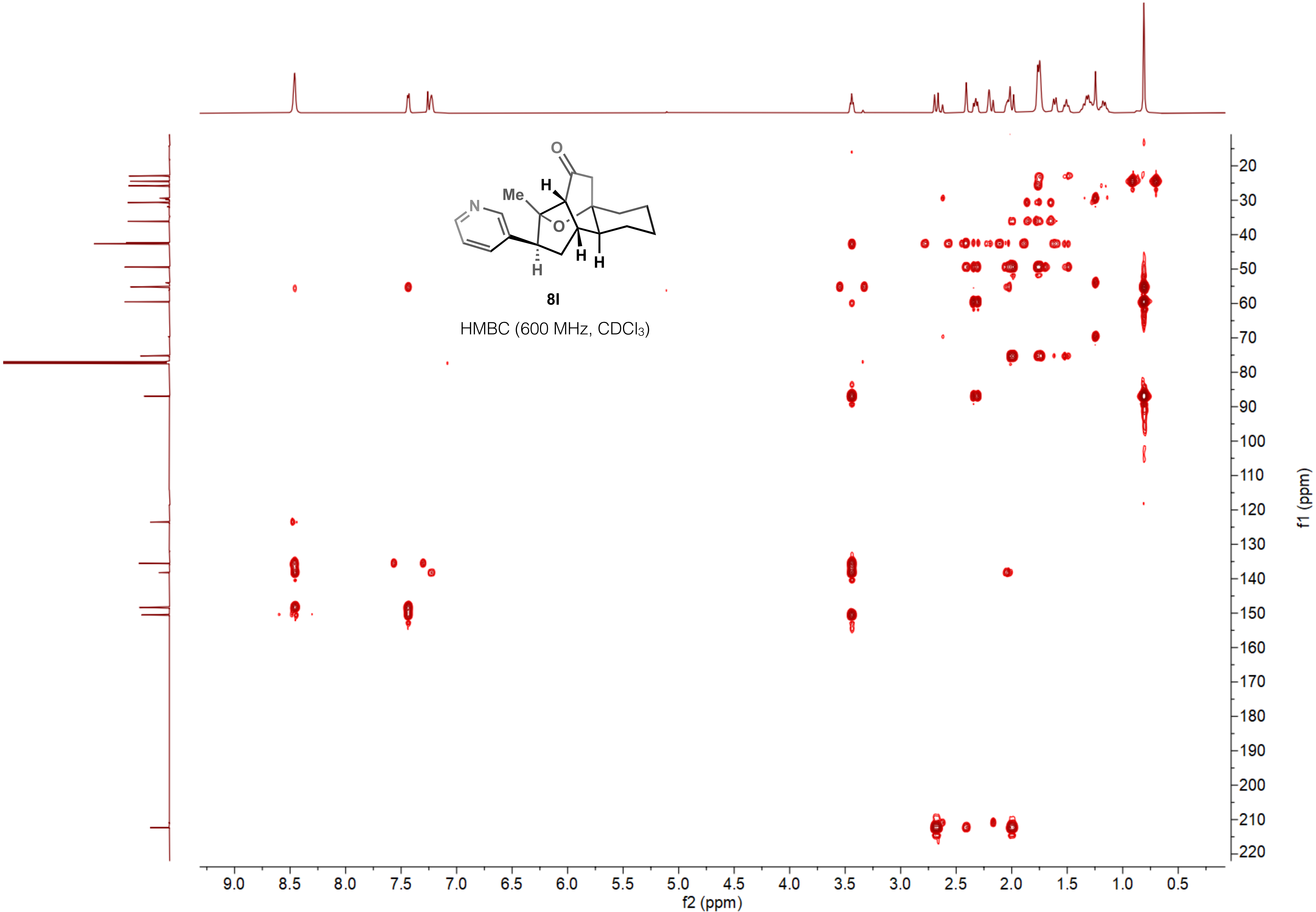

8l

HMBC (600 MHz, $CDCl_3$)

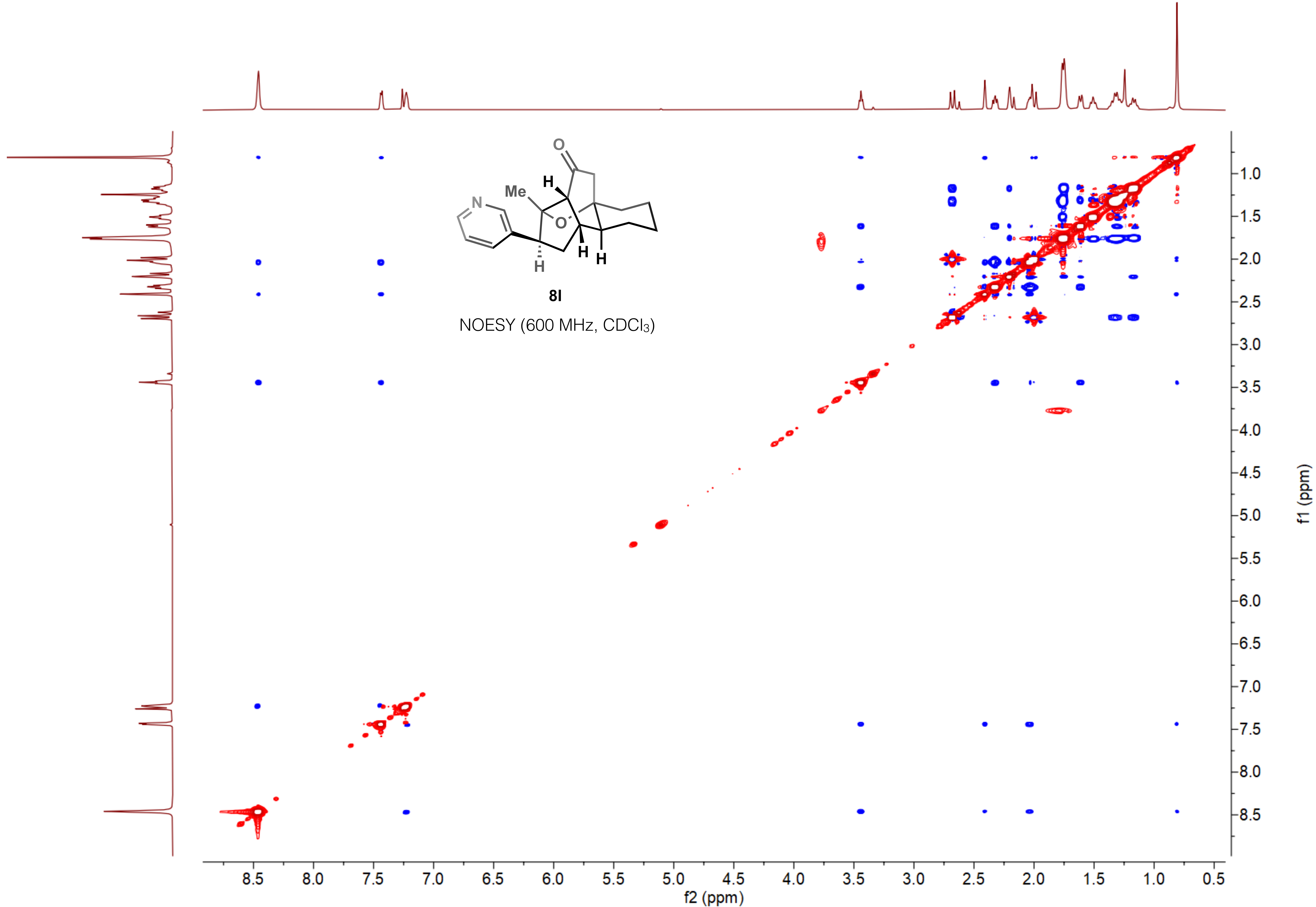
Me
H
N
O
O
H
H
H
8l
NOESY (600 MHz, CDCl3)
f2 (ppm)
f1 (ppm)
8.5 8.0 7.5 7.0 6.5 6.0 5.5 5.0 4.5 4.0 3.5 3.0 2.5 2.0 1.5 1.0 0.5
1.0 1.5 2.0 2.5 3.0 3.5 4.0 4.5 5.0 5.5 6.0 6.5 7.0 7.5 8.0 8.5

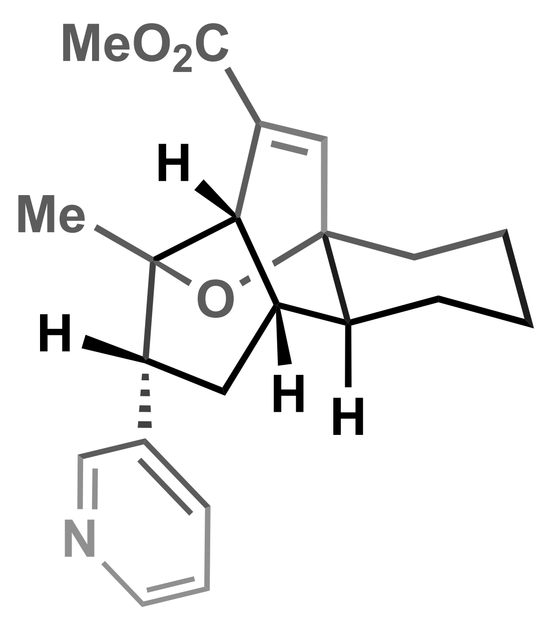

RS1121

(600 MHz, $CDCl_3$)

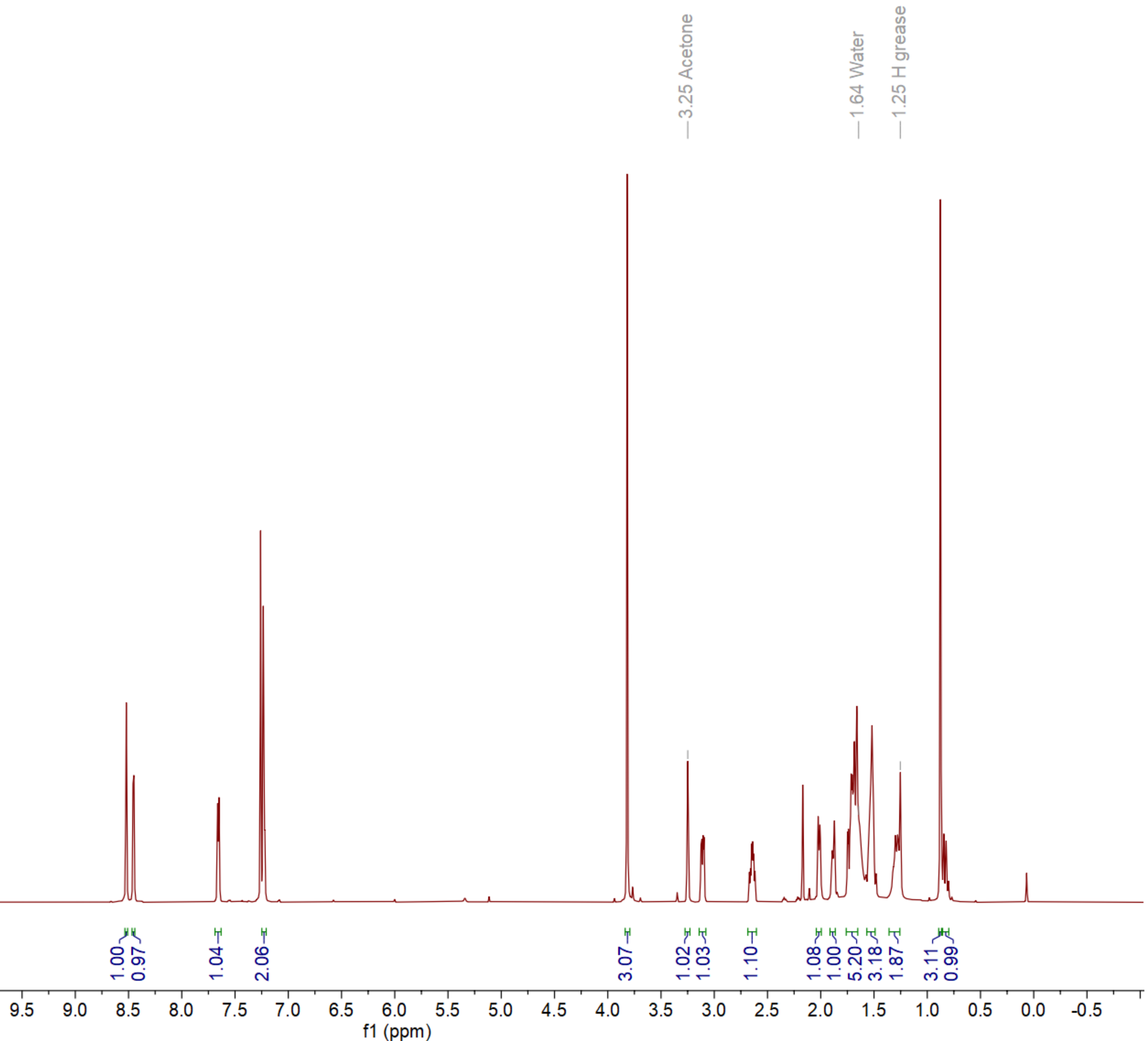

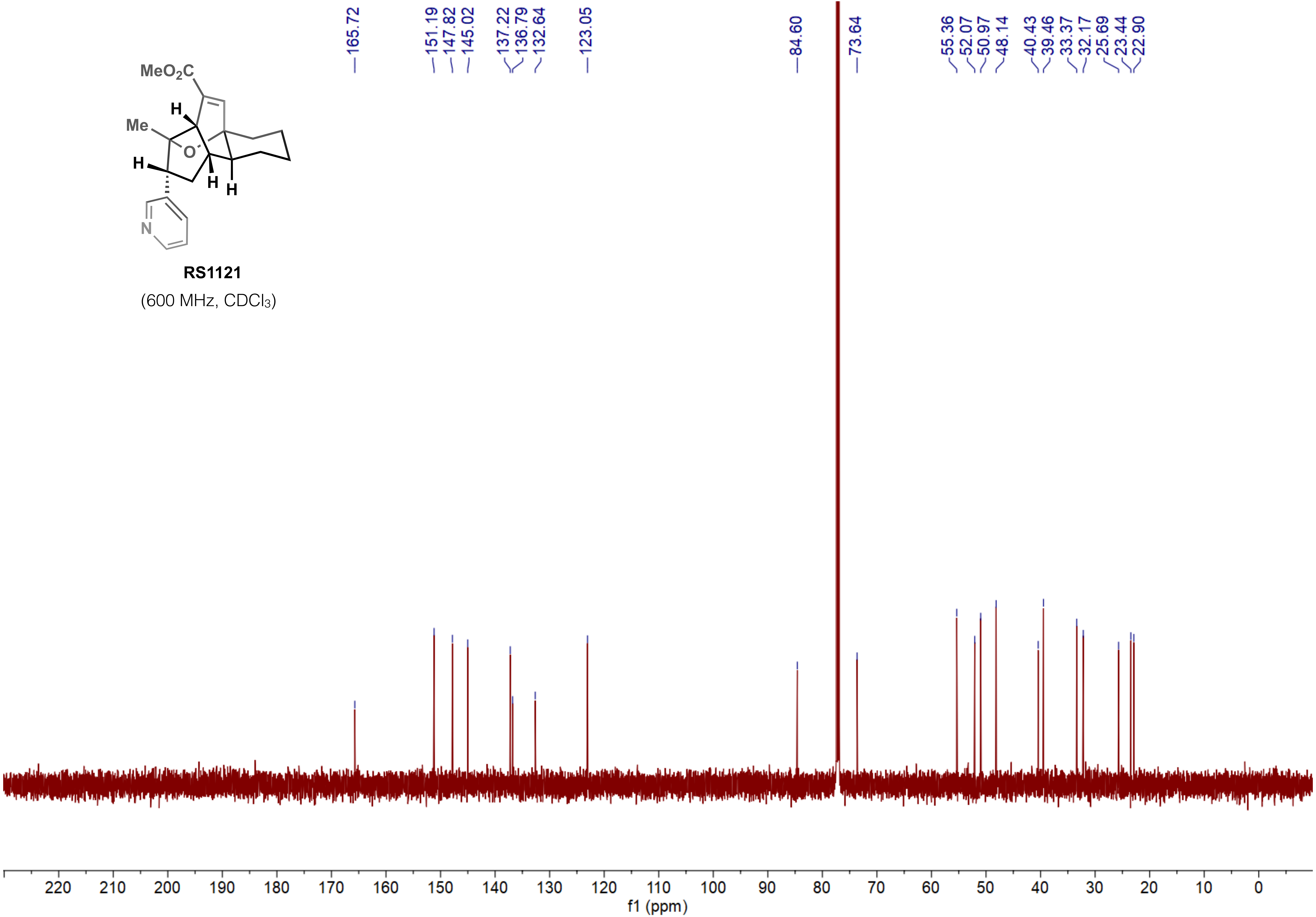
MeO2C
Me
O
H
H
H
H
N
RS1121
(600 MHz, CDCl3)
165.72
151.19
147.82
145.02
137.22
136.79
132.64
123.05
84.60
73.64
55.36
52.07
50.97
48.14
40.43
39.46
33.37
32.17
25.69
23.44
22.90
f1 (ppm)

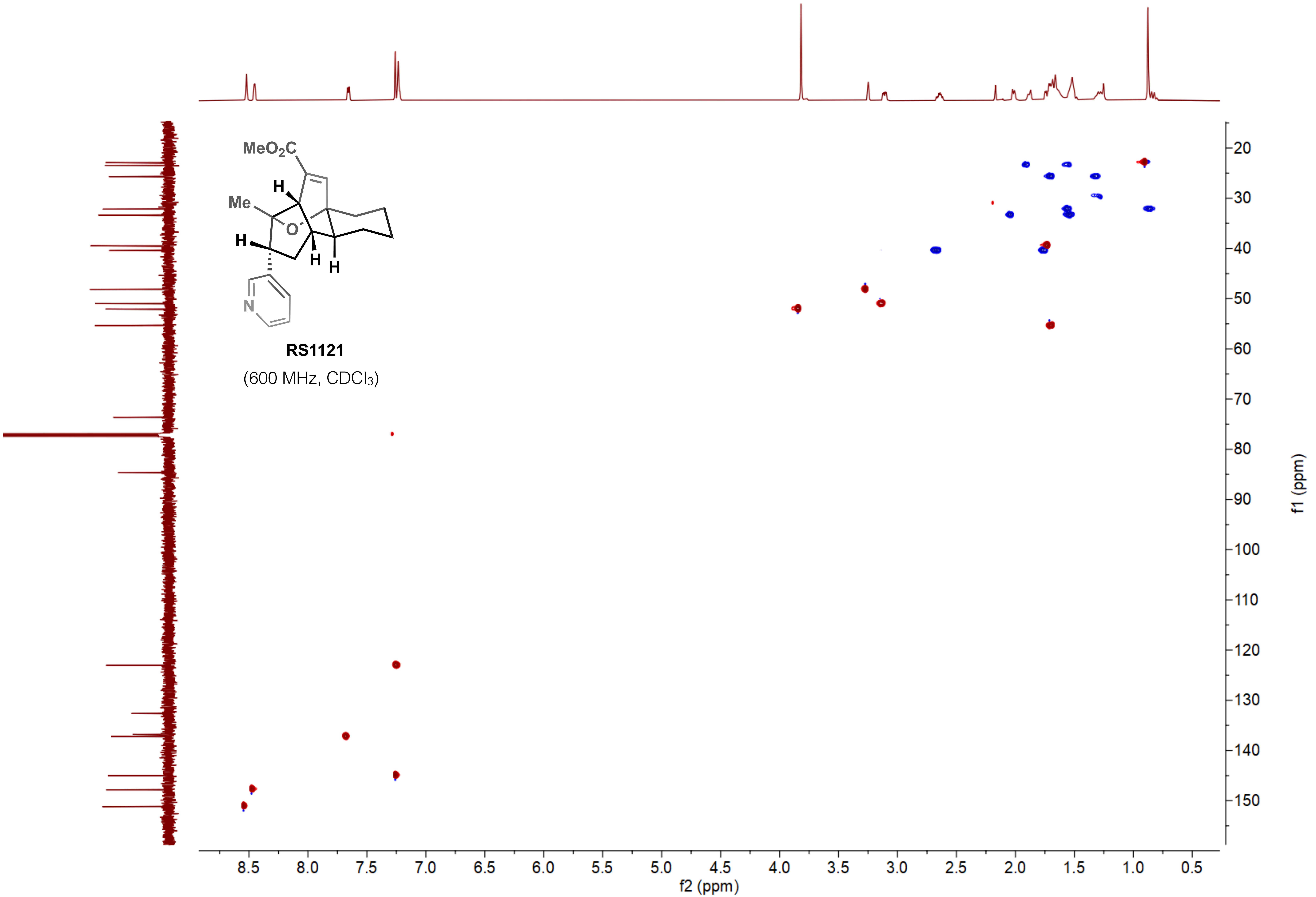

MeO2C
Me
H
O
H
H
H
N
RS1121
(600 MHz, CDCl3)
f2 (ppm)
f1 (ppm)

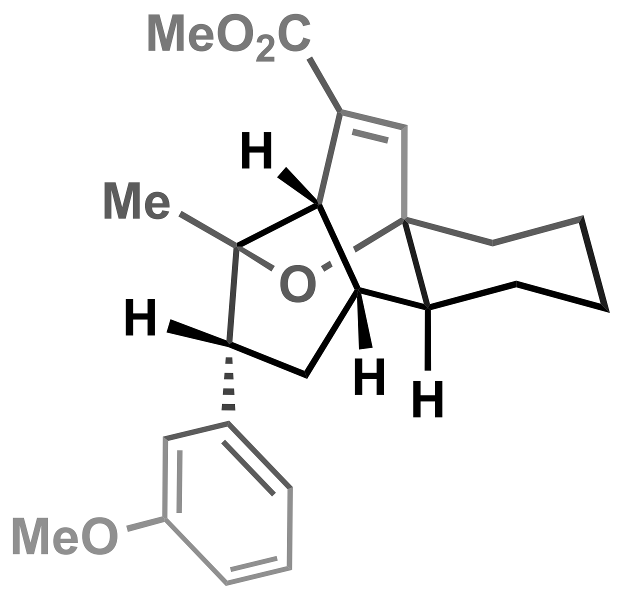

RS1167
(7m)
(600 MHz, $CDCl_3$)

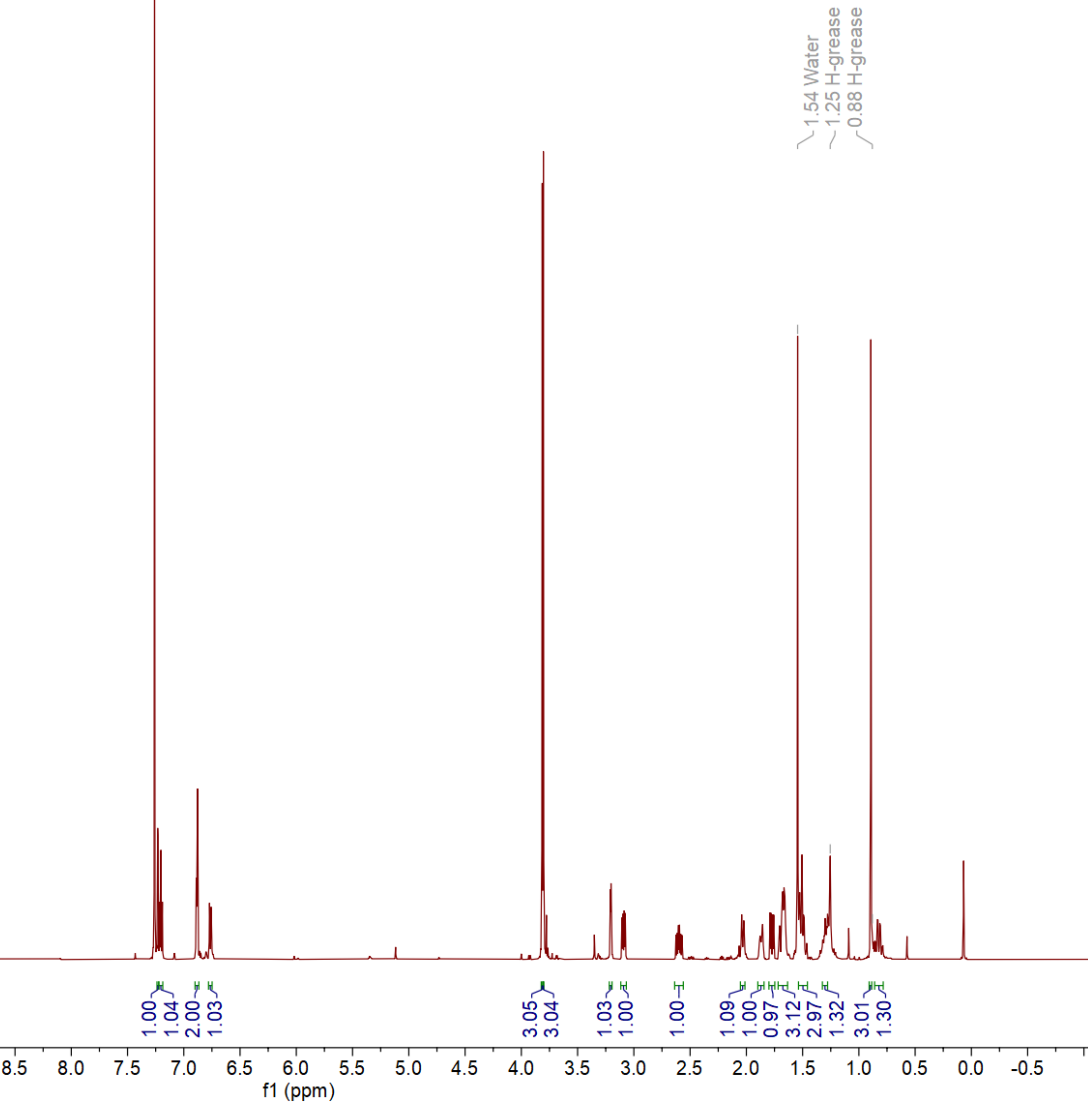

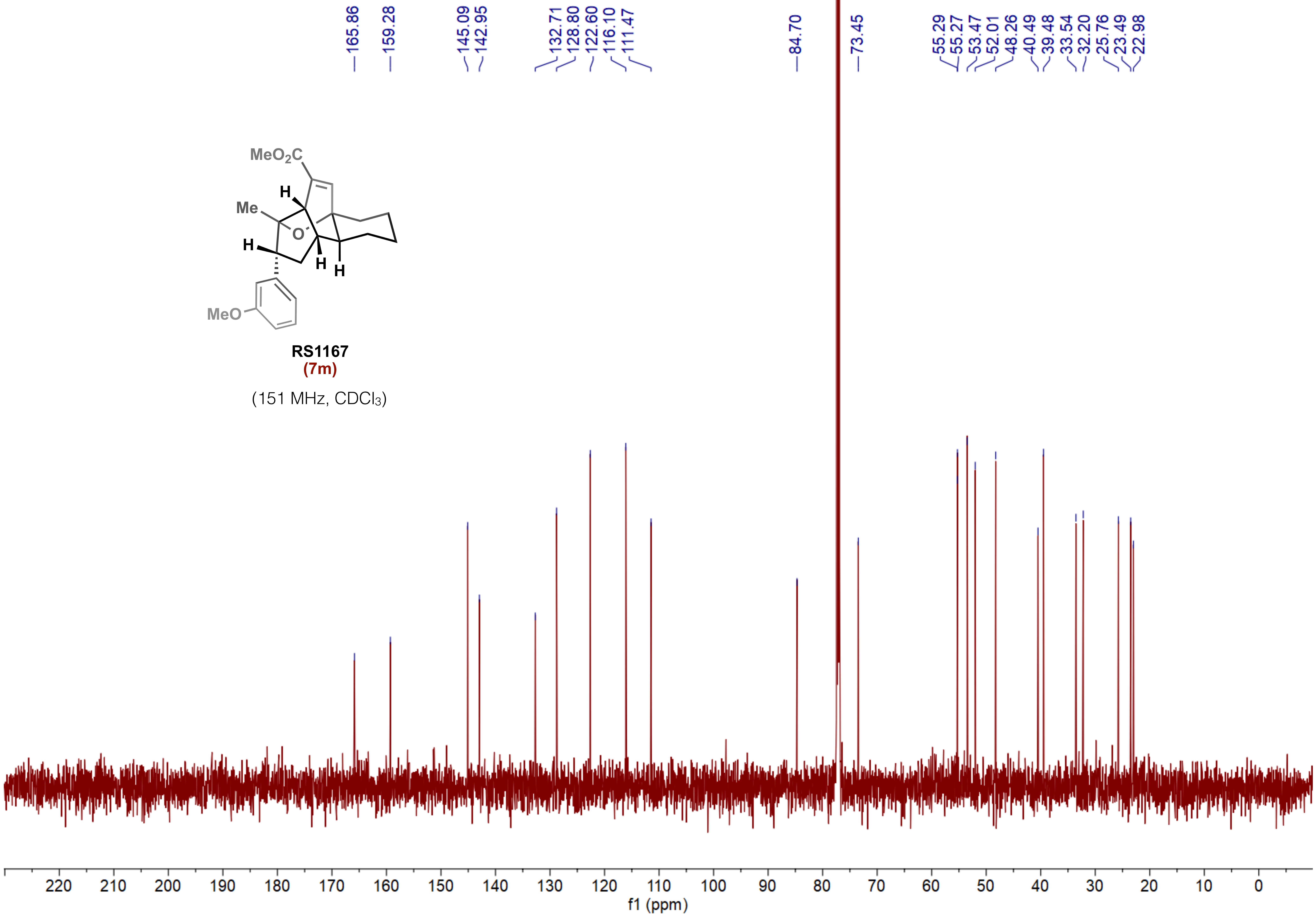

RS1167
(7m)
(151 MHz, $CDCl_3$)

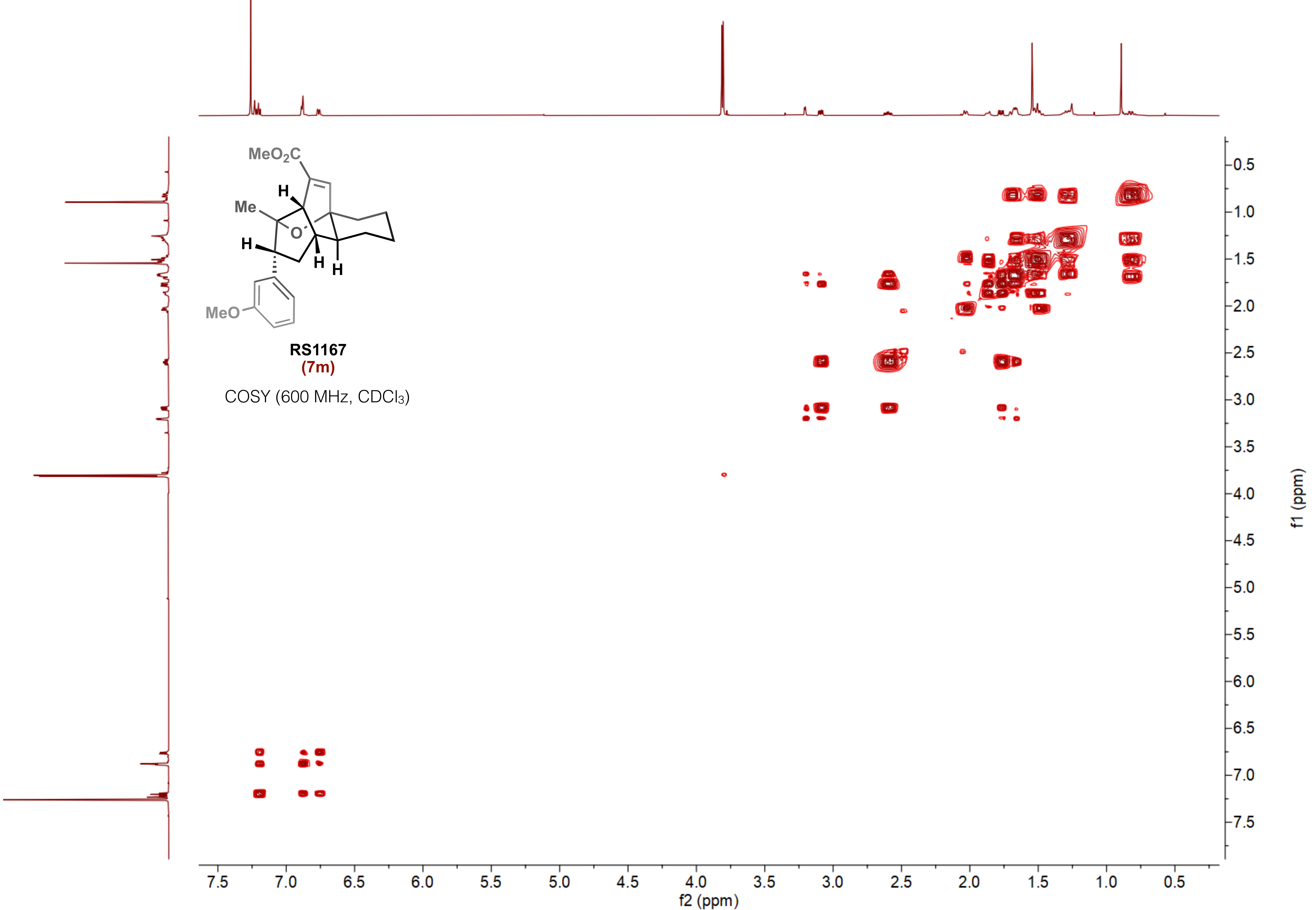

RS1167
(7m)
COSY (600 MHz, $CDCl_3$)

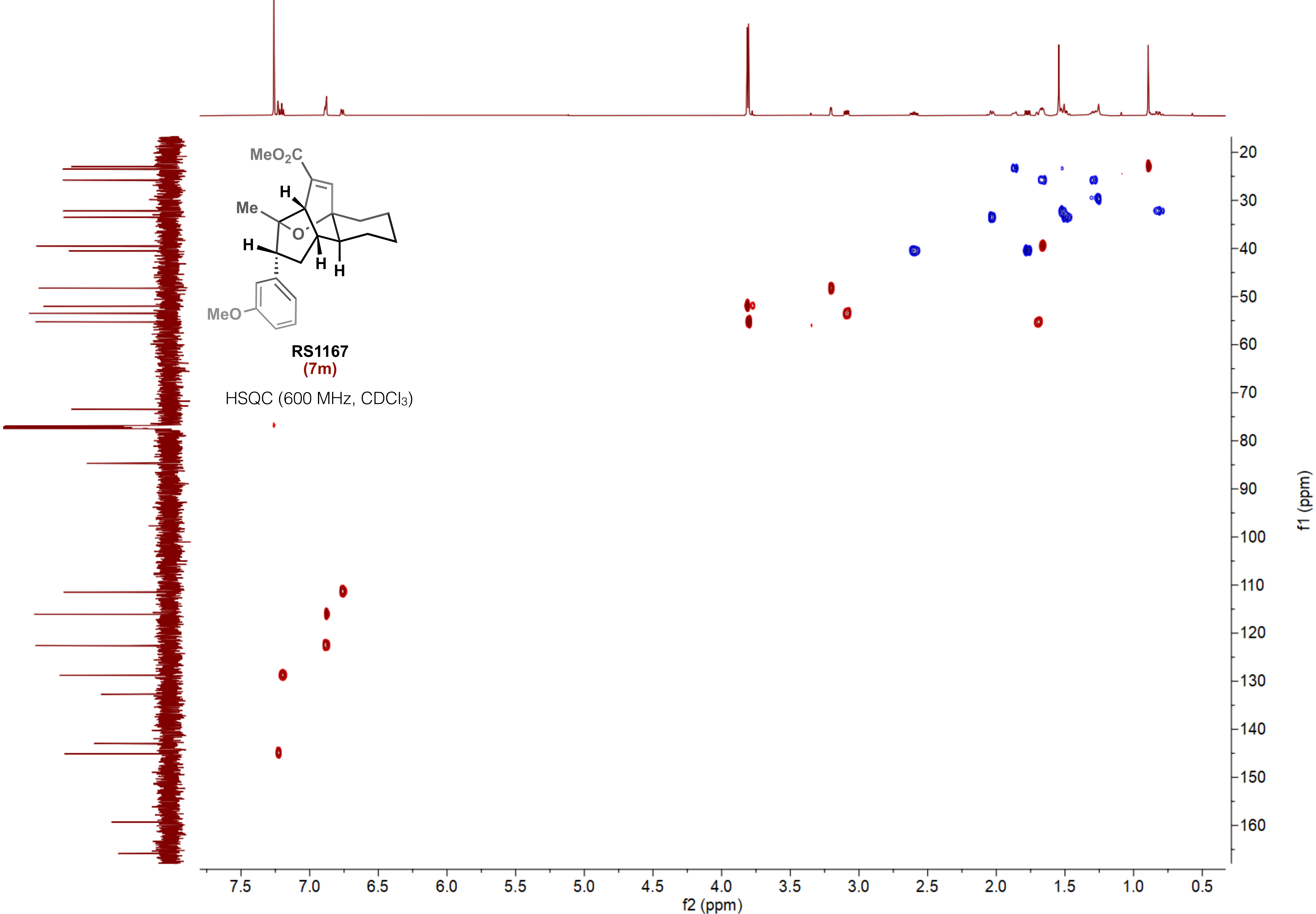
MeO2C
Me
H
O
H
H
H
MeO
RS1167
(7m)
HSQC (600 MHz, CDCl3)
f1 (ppm)
f2 (ppm)
20
30
40
50
60
70
80
90
100
110
120
130
140
150
160
7.5
7.0
6.5
6.0
5.5
5.0
4.5
4.0
3.5
3.0
2.5
2.0
1.5
1.0
0.5

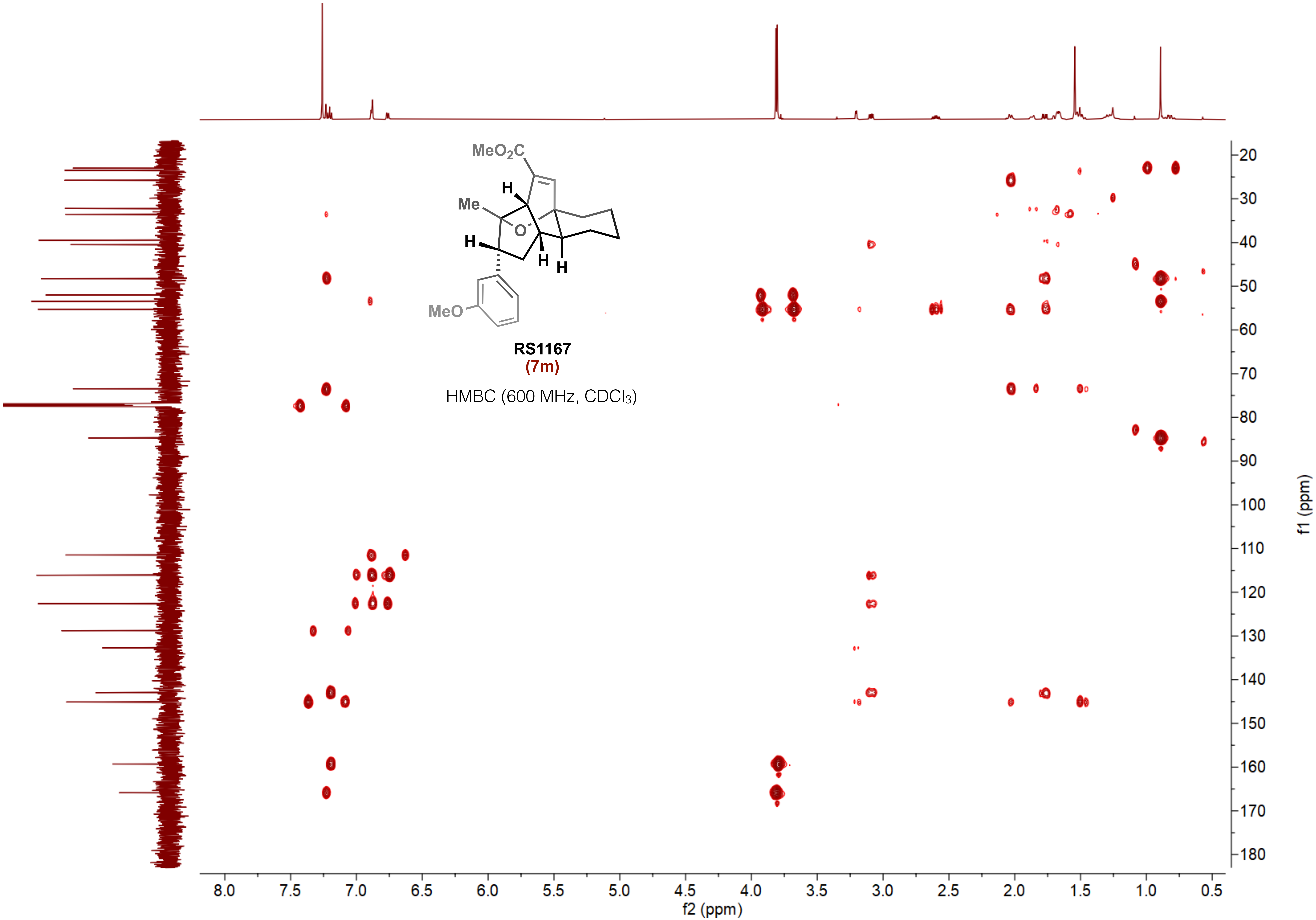
MeO2C
Me
H
H
O
H
H
MeO
RS1167
(7m)
HMBC (600 MHz, CDCl3)
f1 (ppm)
f2 (ppm)
20
30
40
50
60
70
80
90
100
110
120
130
140
150
160
170
180
8.0
7.5
7.0
6.5
6.0
5.5
5.0
4.5
4.0
3.5
3.0
2.5
2.0
1.5
1.0
0.5

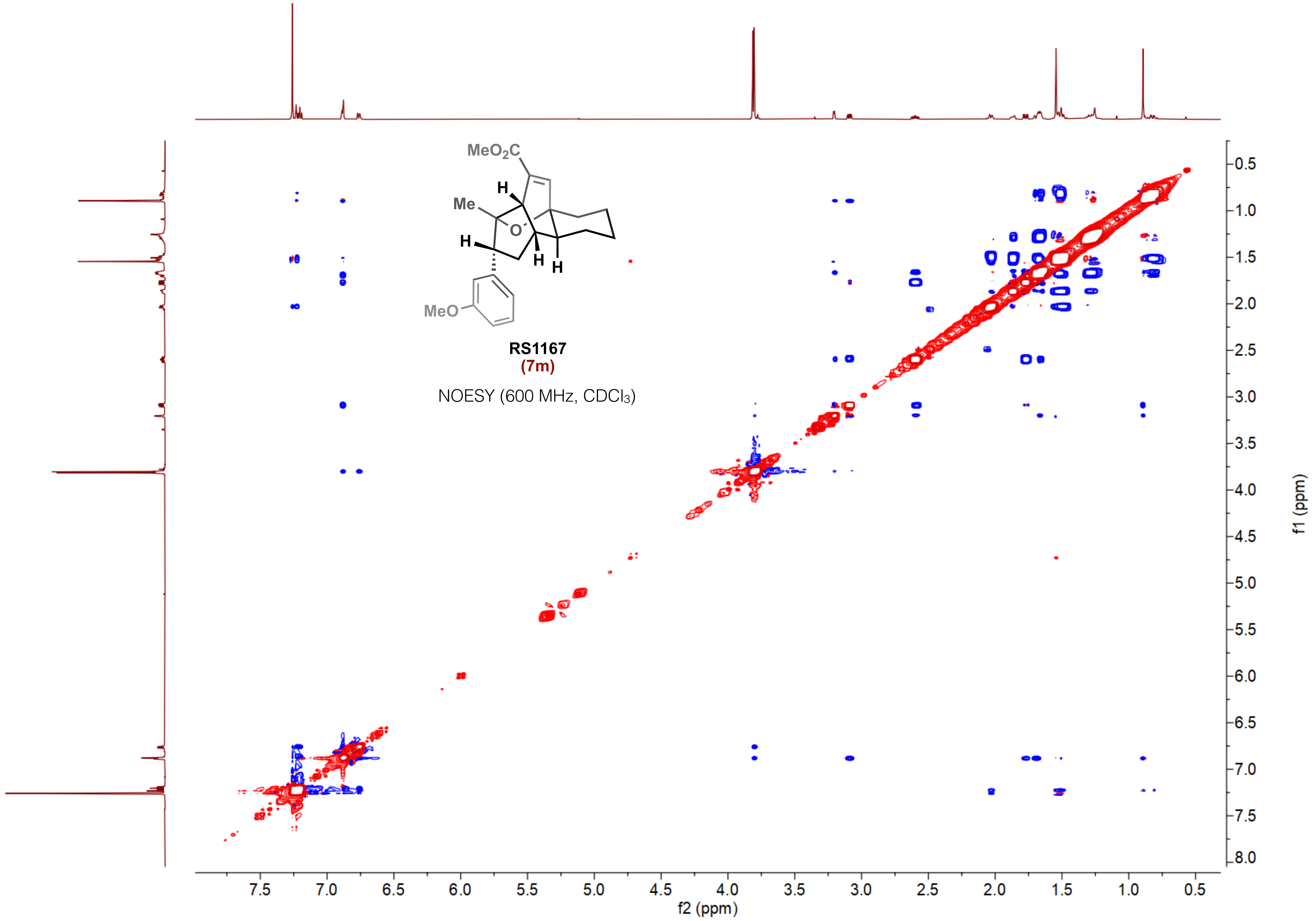
MeO2C
Me
H
H
O
H
H
MeO
RS1167
(7m)
NOESY (600 MHz, CDCl3)
f2 (ppm)
f1 (ppm)

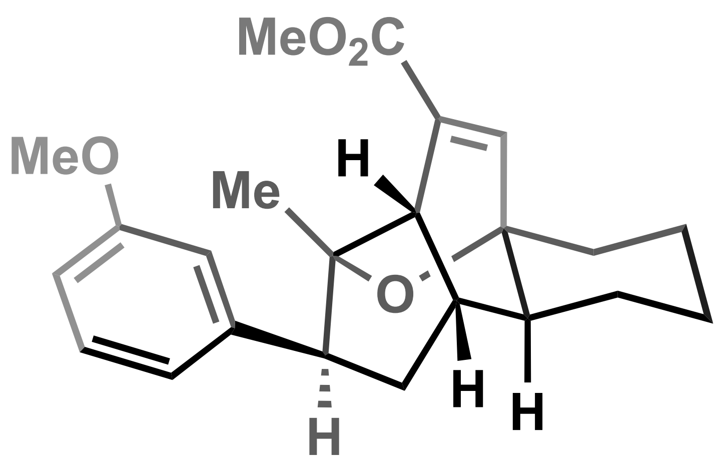

8m

(600 MHz, $CDCl_3$)

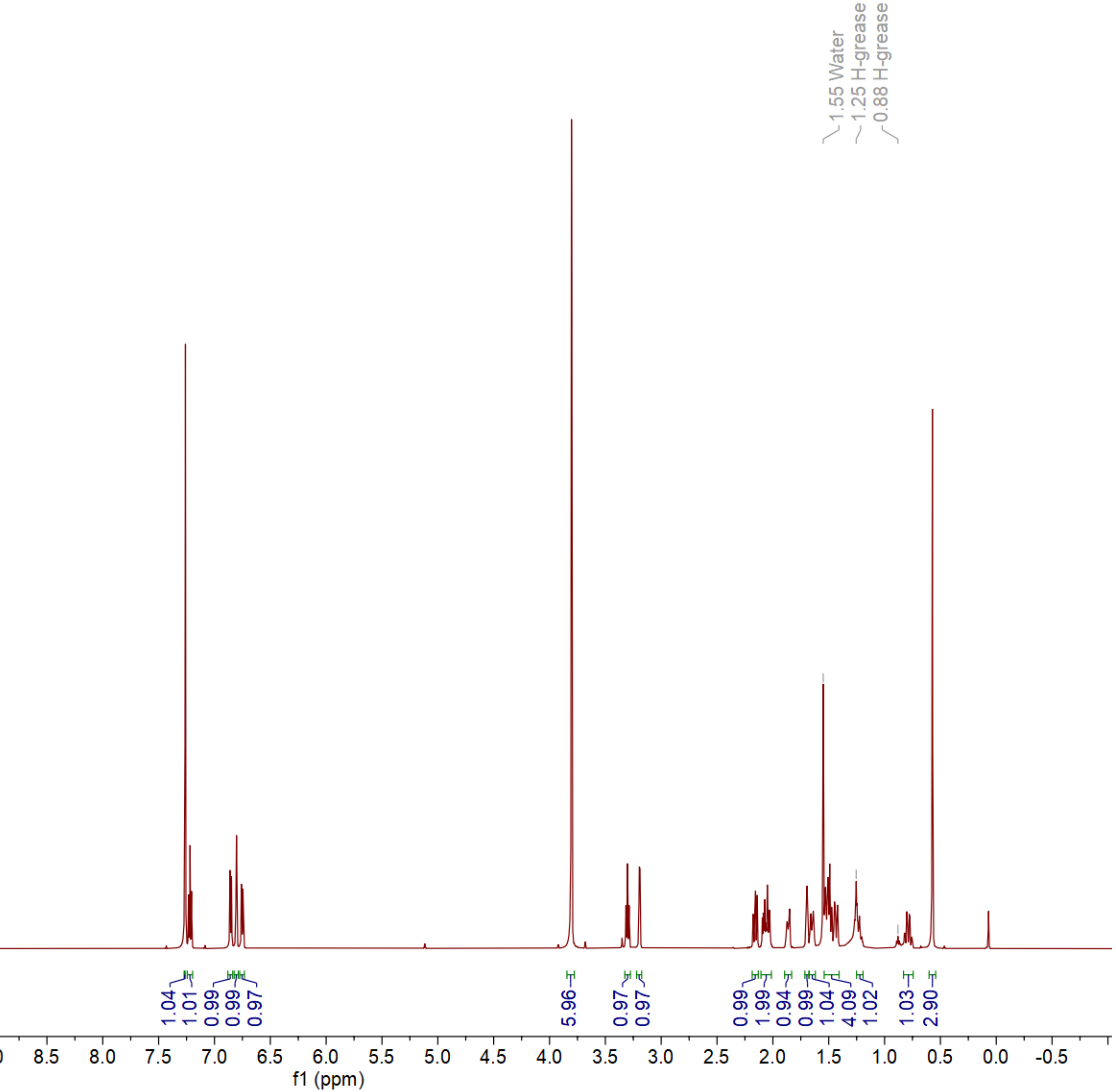

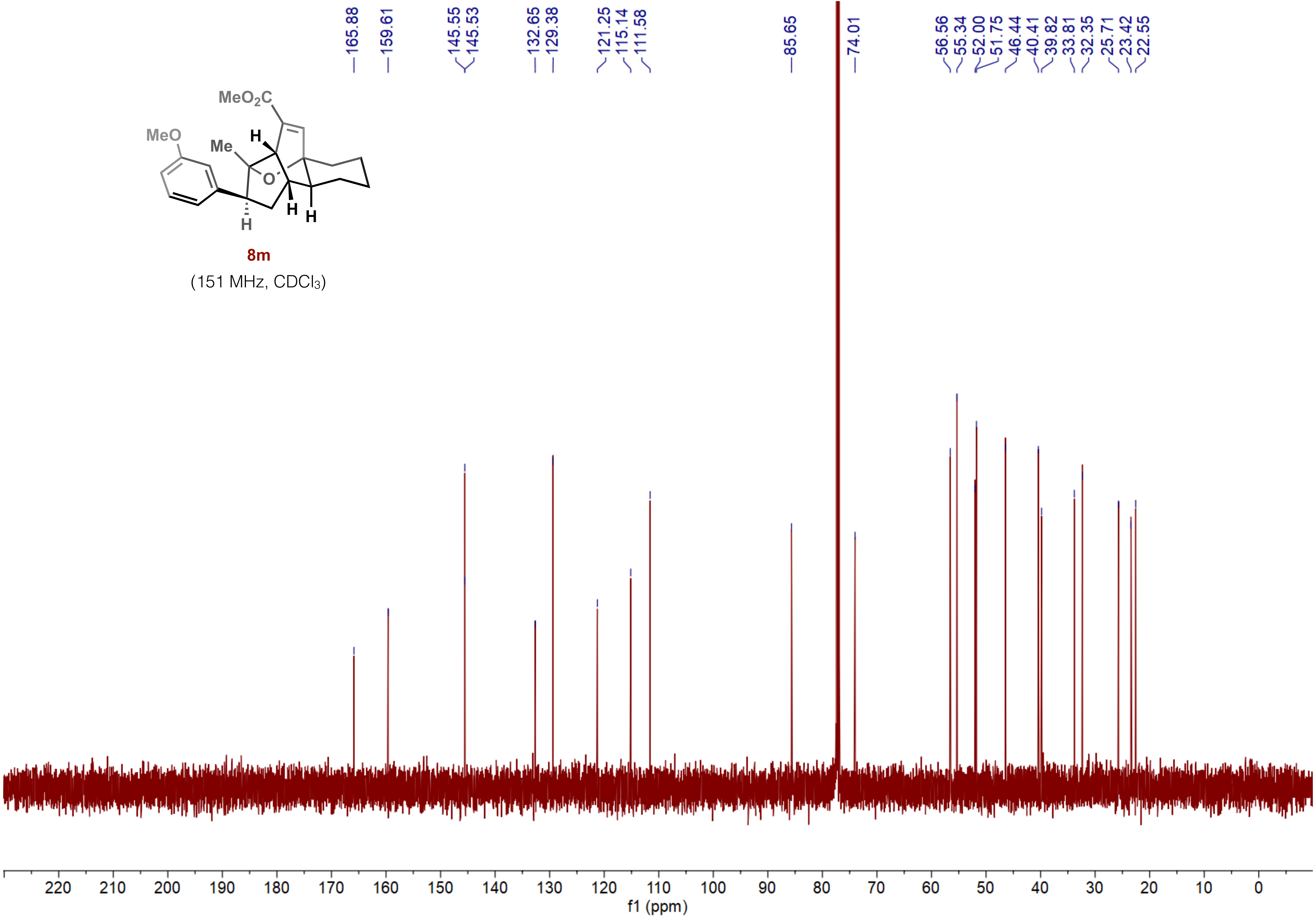

8m

(151 MHz, $CDCl_3$)

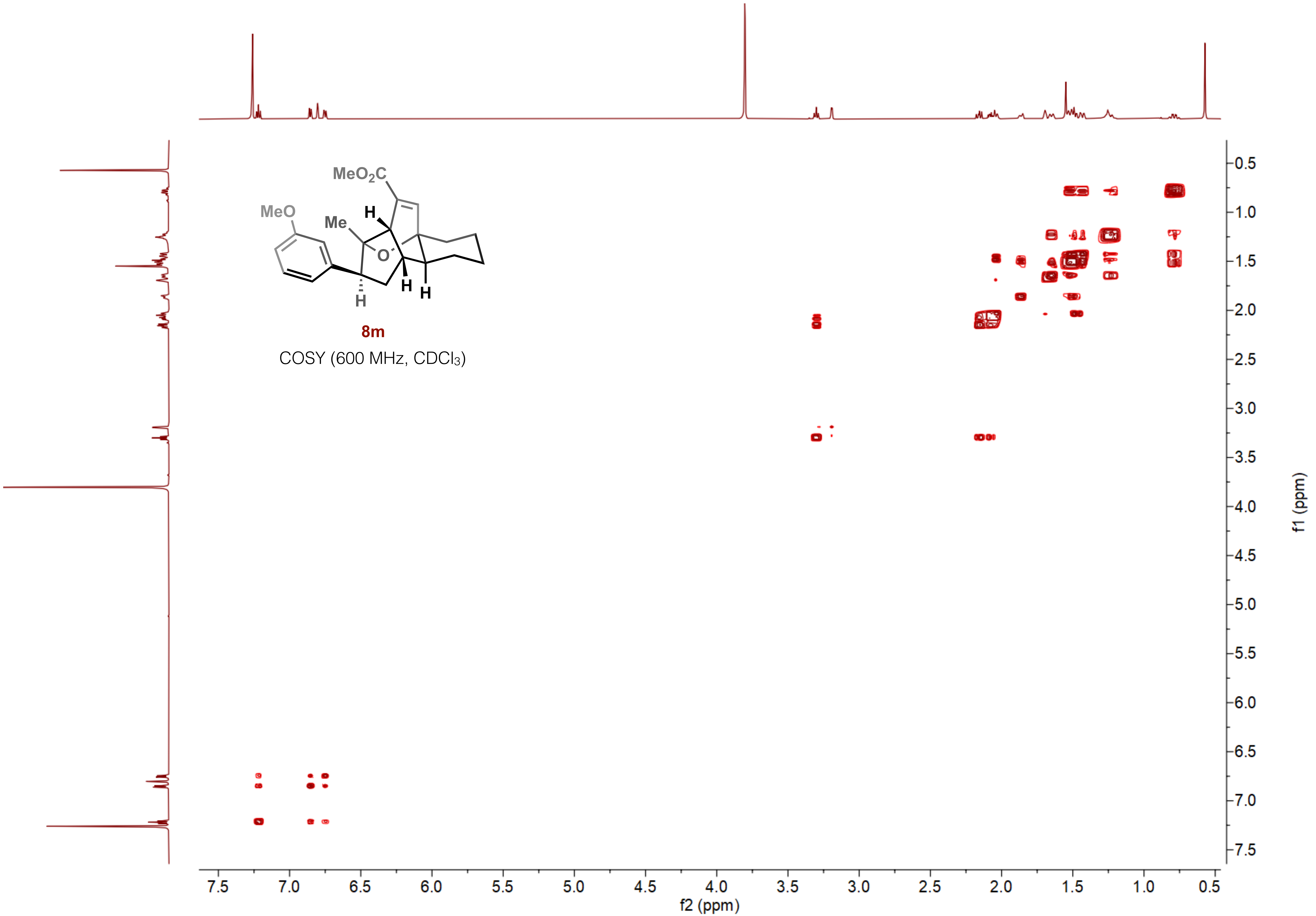

COSY (600 MHz, $CDCl_3$)

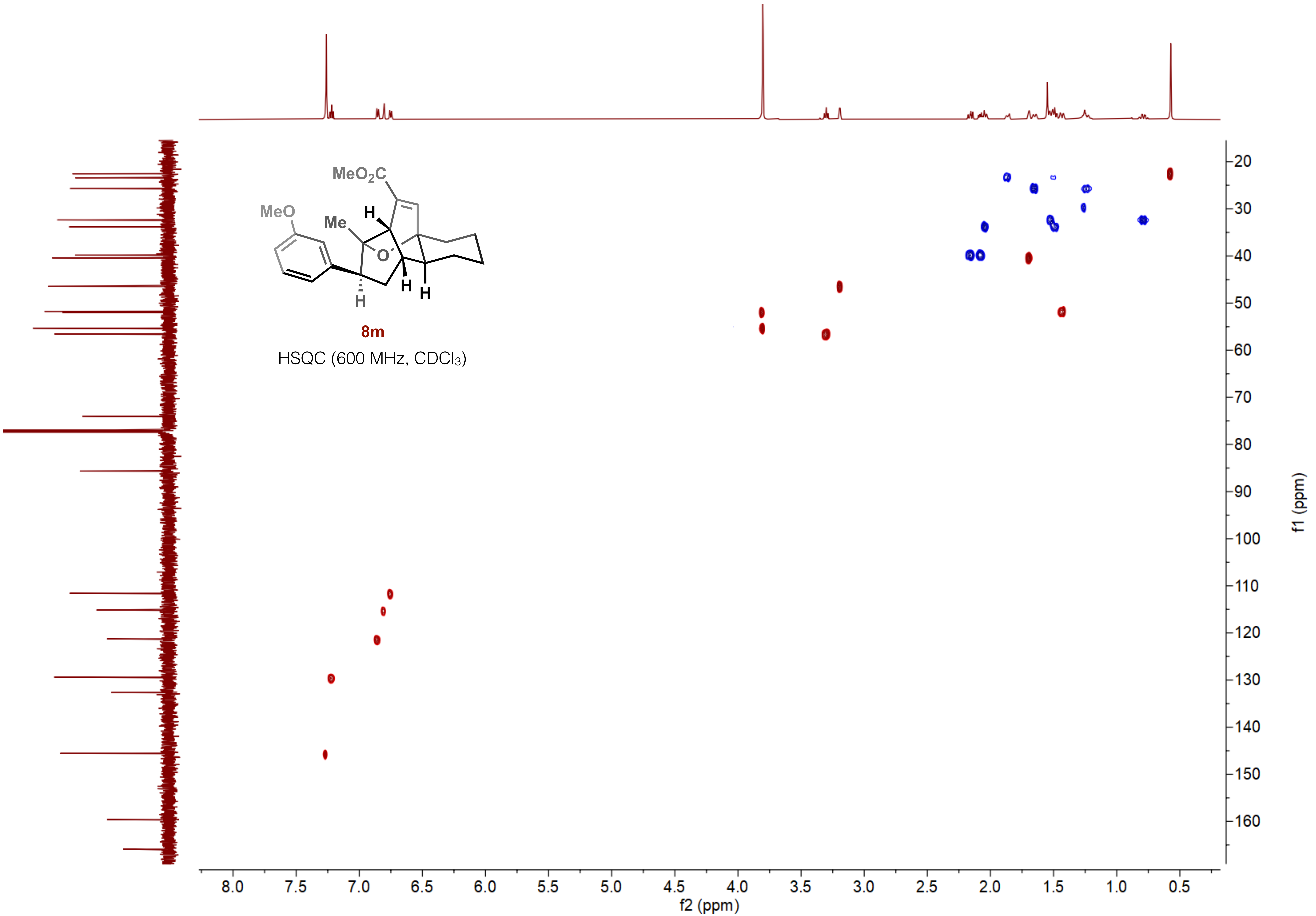
MeO₂C
MeO
Me
H
O
H
H
H
8m
HSQC (600 MHz, CDCl3)
f2 (ppm)
f1 (ppm)

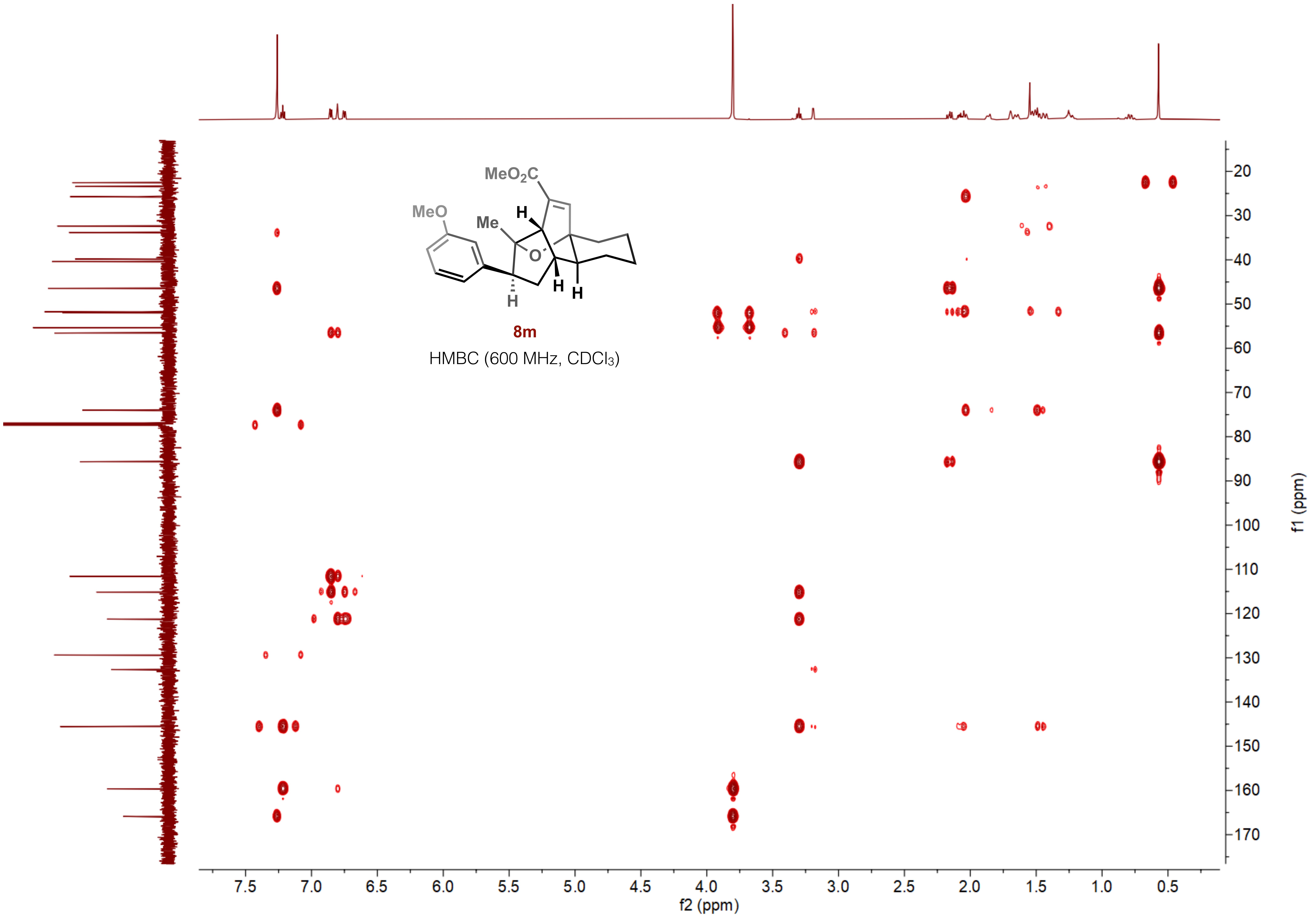
MeO$_2$C
MeO
Me
H
O
H
H
H
8m
HMBC (600 MHz, CDCl$_3$)
f2 (ppm)
f1 (ppm)

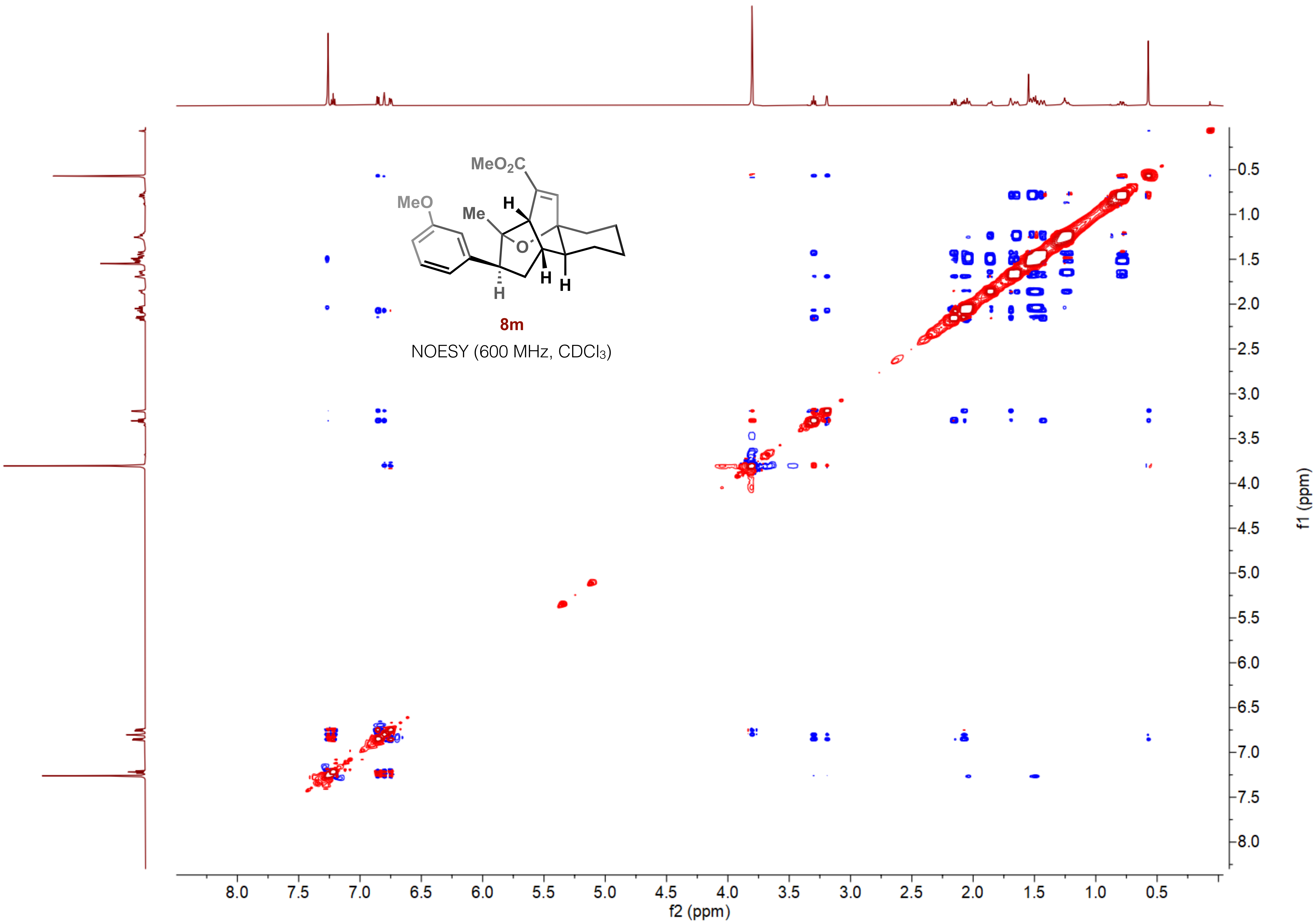
MeO$_2$C
MeO
Me
H
O
H
H
H
8m
NOESY (600 MHz, CDCl$_3$)
f2 (ppm)
f1 (ppm)

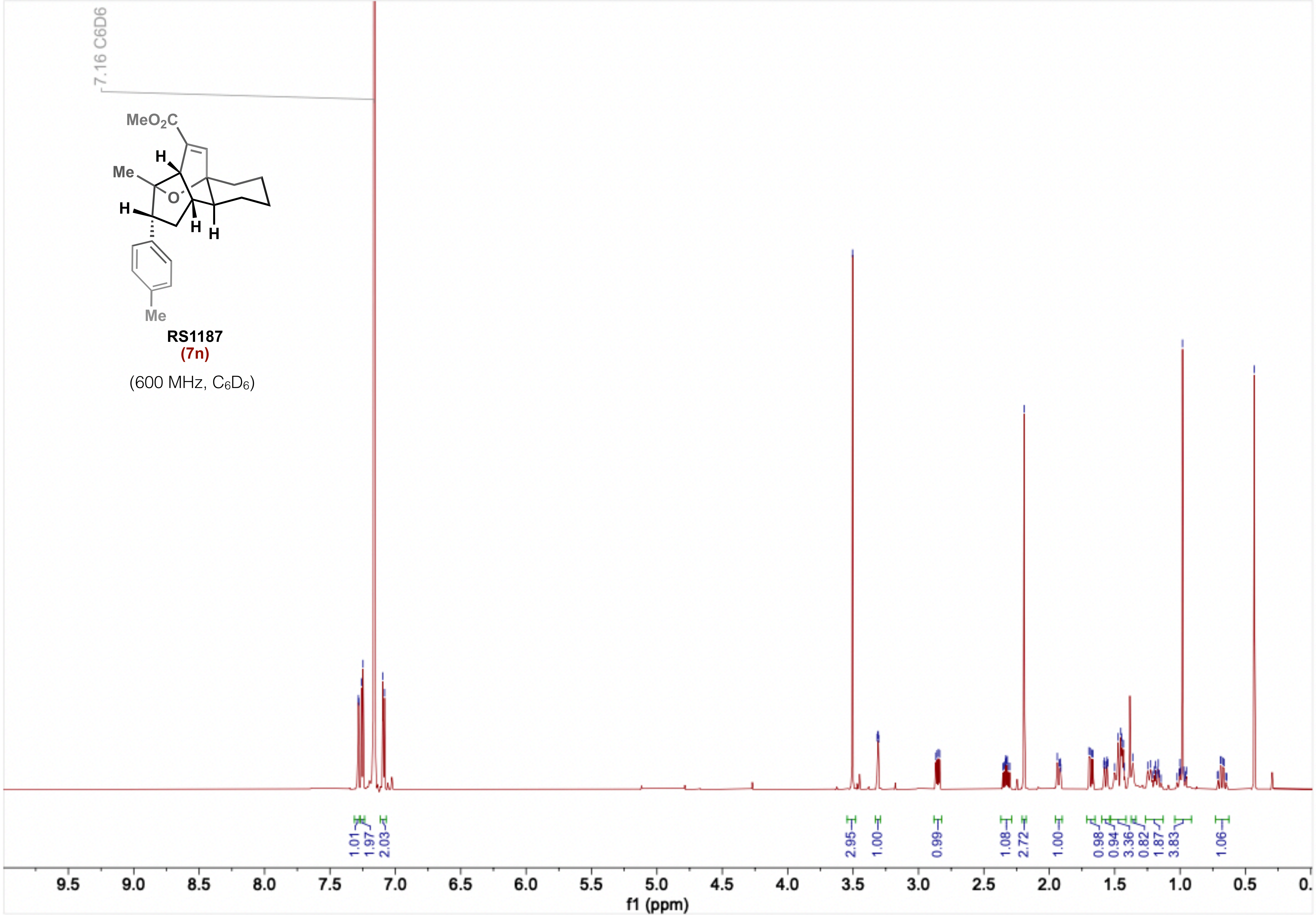
7.16 C6D6
MeO2C
Me
H
O
Me
RS1187
(7n)
(600 MHz, C6D6)
1.01
1.97
2.03
2.95
1.00
0.99
1.08
2.72
1.00
0.98
0.94
3.36
0.82
1.87
3.83
1.06
9.5 9.0 8.5 8.0 7.5 7.0 6.5 6.0 5.5 5.0 4.5 4.0 3.5 3.0 2.5 2.0 1.5 1.0 0.5 0.
f1 (ppm)

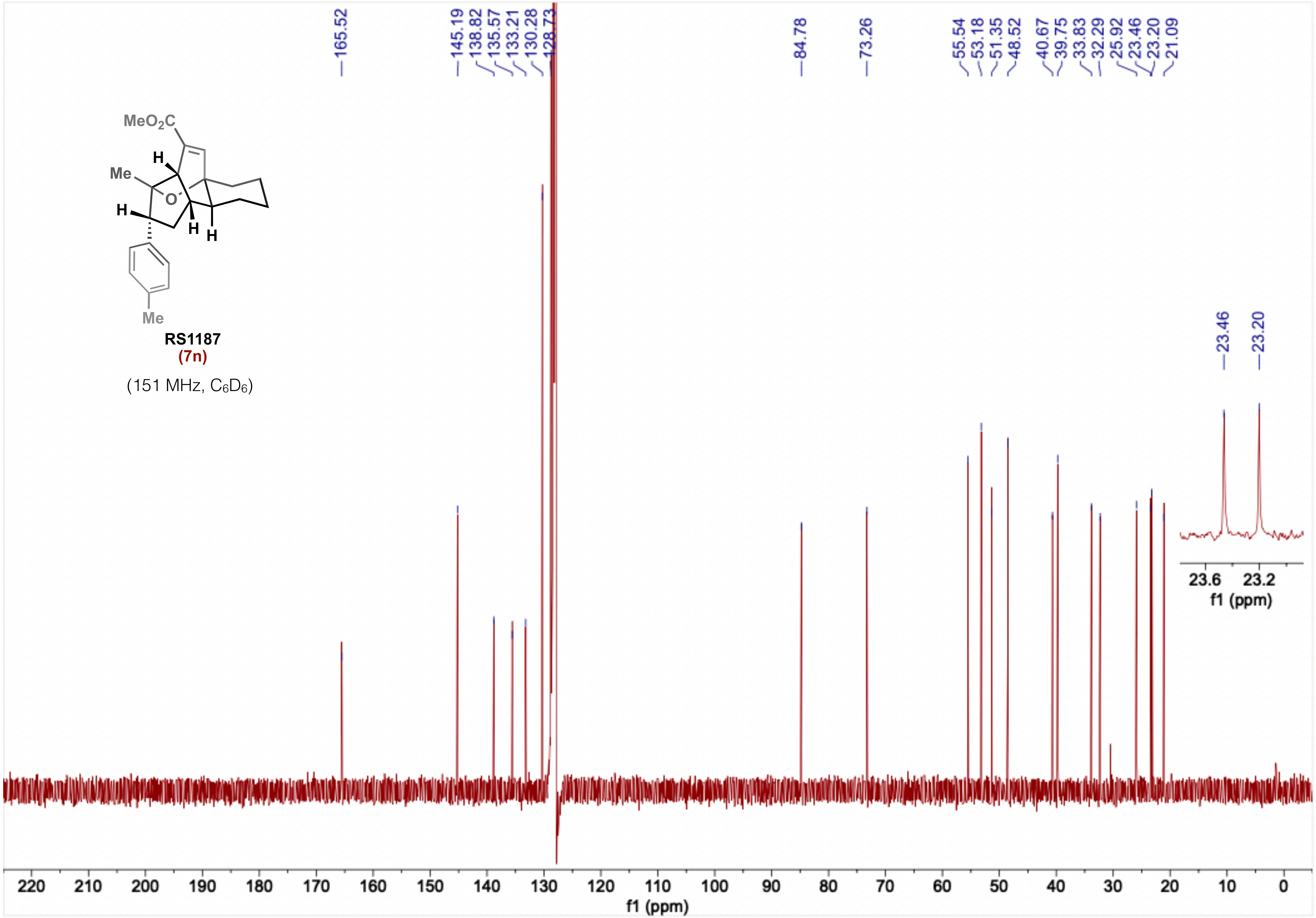

165.52
145.19
138.82
135.57
133.21
130.28
128.73
84.78
73.26
55.54
53.18
51.35
48.52
40.67
39.75
33.83
32.29
25.92
23.46
23.20
21.09
MeO2C
H
Me
O
H
H
H
Me
RS1187
(7n)
(151 MHz, C6D6)
23.46
23.20
23.6
23.2
f1 (ppm)
220 210 200 190 180 170 160 150 140 130 120 110 100 90 80 70 60 50 40 30 20 10 0
f1 (ppm)

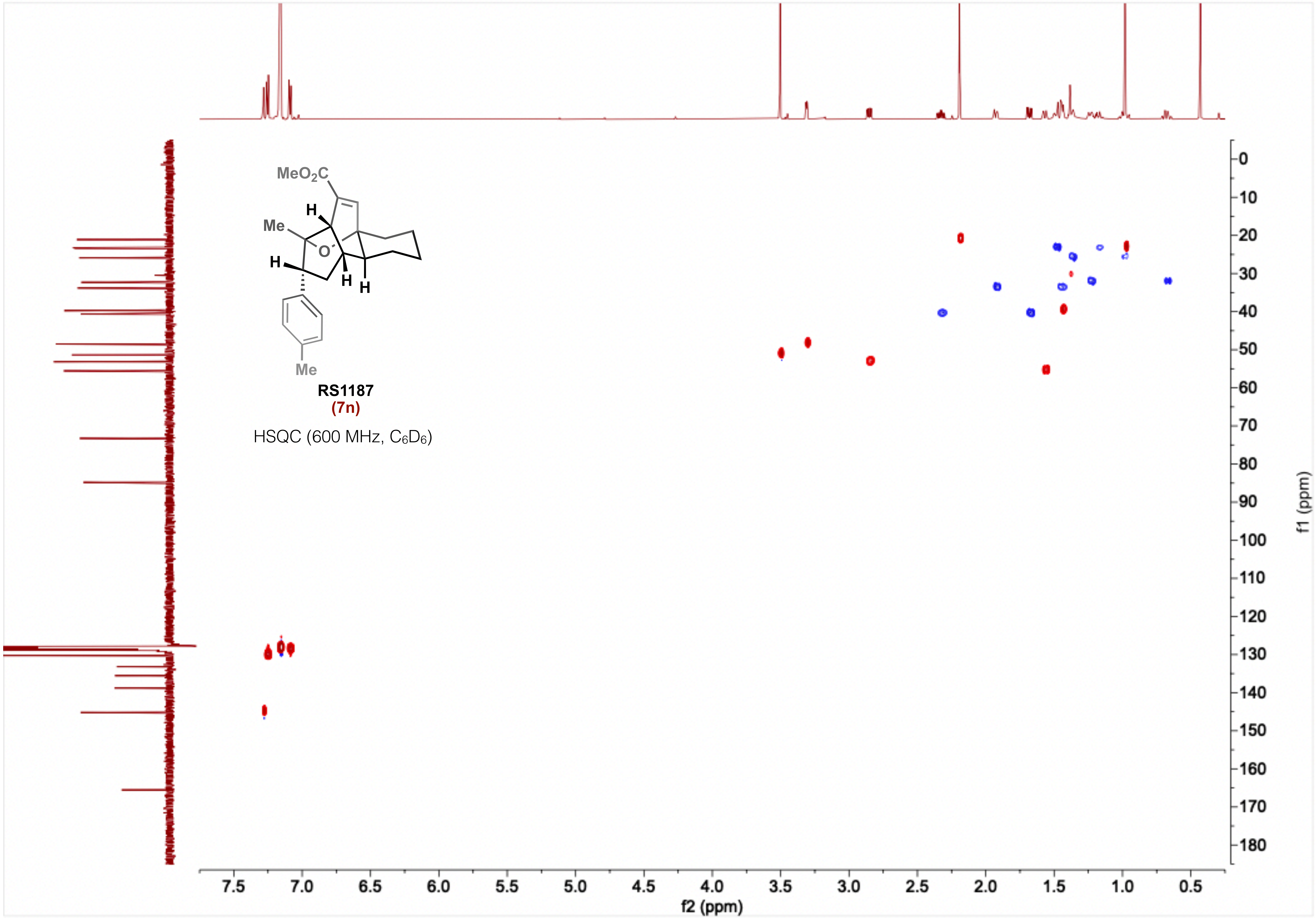

MeO2C
H
Me
O
H
H
H
Me
RS1187
(7n)
HSQC (600 MHz, C6D6)
f2 (ppm)
f1 (ppm)

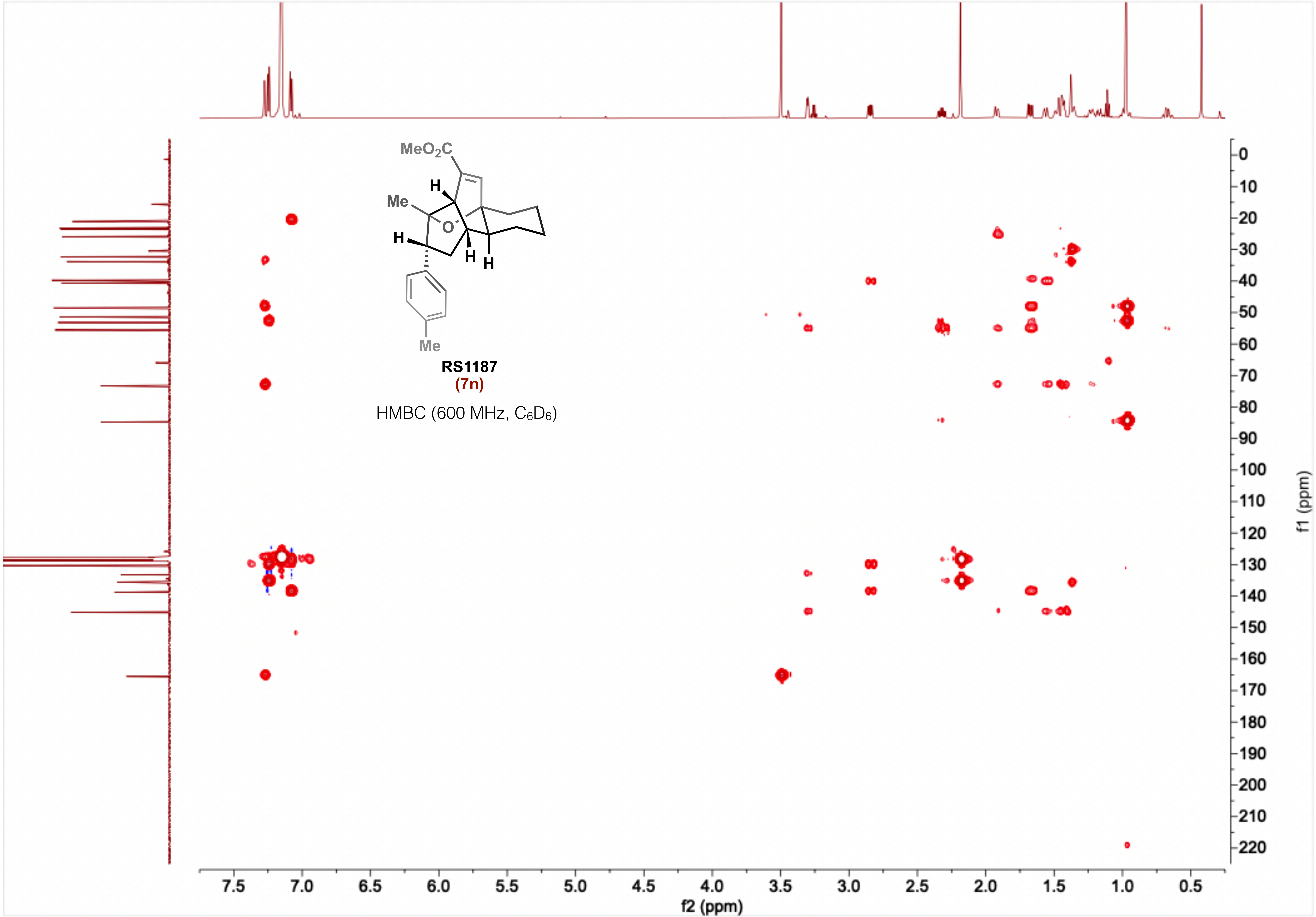
MeO2C
H
Me
O
H
H H
Me
RS1187
(7n)
HMBC (600 MHz, C6D6)
f1 (ppm)
f2 (ppm)
0
10
20
30
40
50
60
70
80
90
100
110
120
130
140
150
160
170
180
190
200
210
220
7.5 7.0 6.5 6.0 5.5 5.0 4.5 4.0 3.5 3.0 2.5 2.0 1.5 1.0 0.5

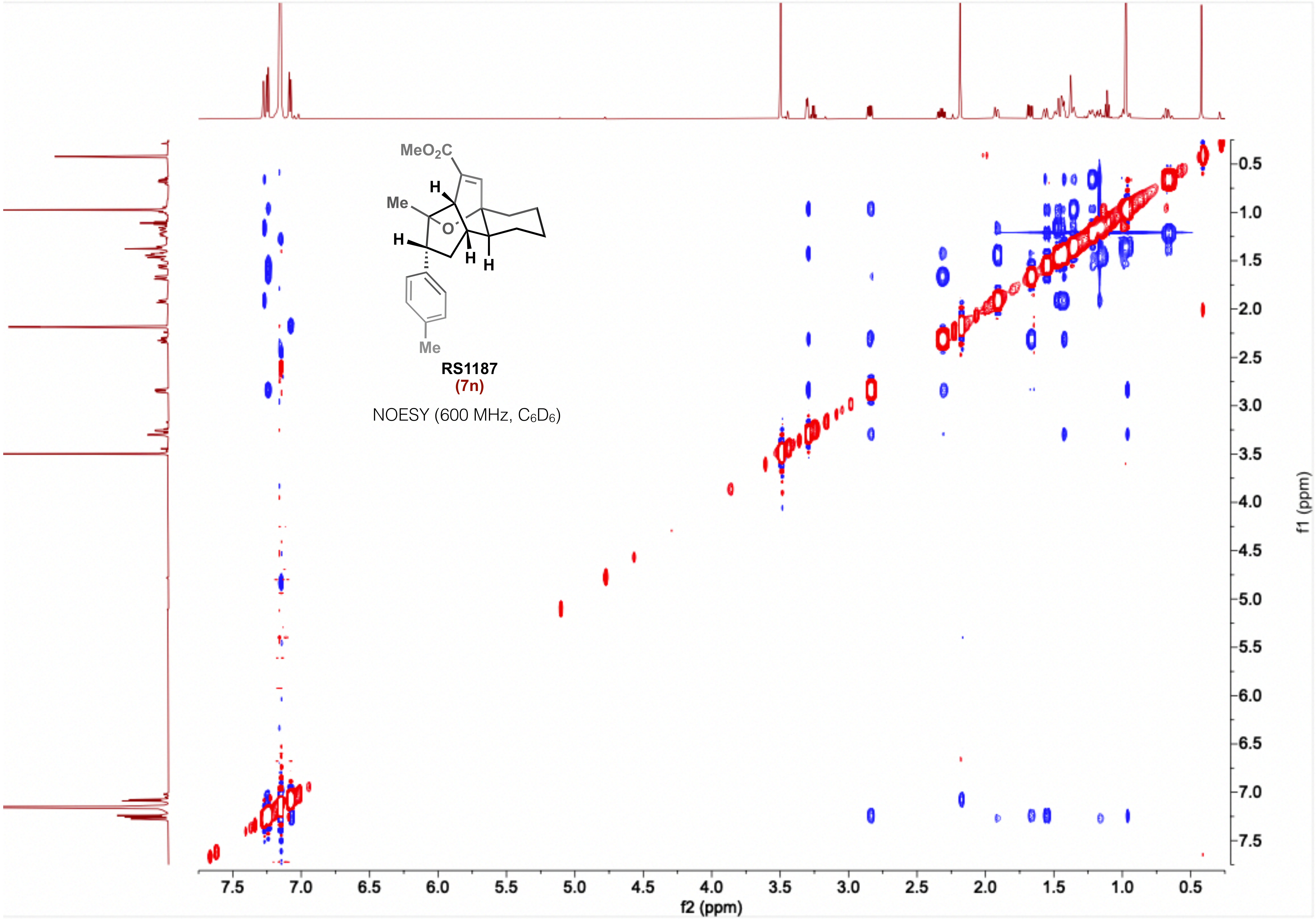
MeO2C
H
Me
O
H
H
H
Me
RS1187
(7n)
NOESY (600 MHz, C6D6)
f2 (ppm)
f1 (ppm)

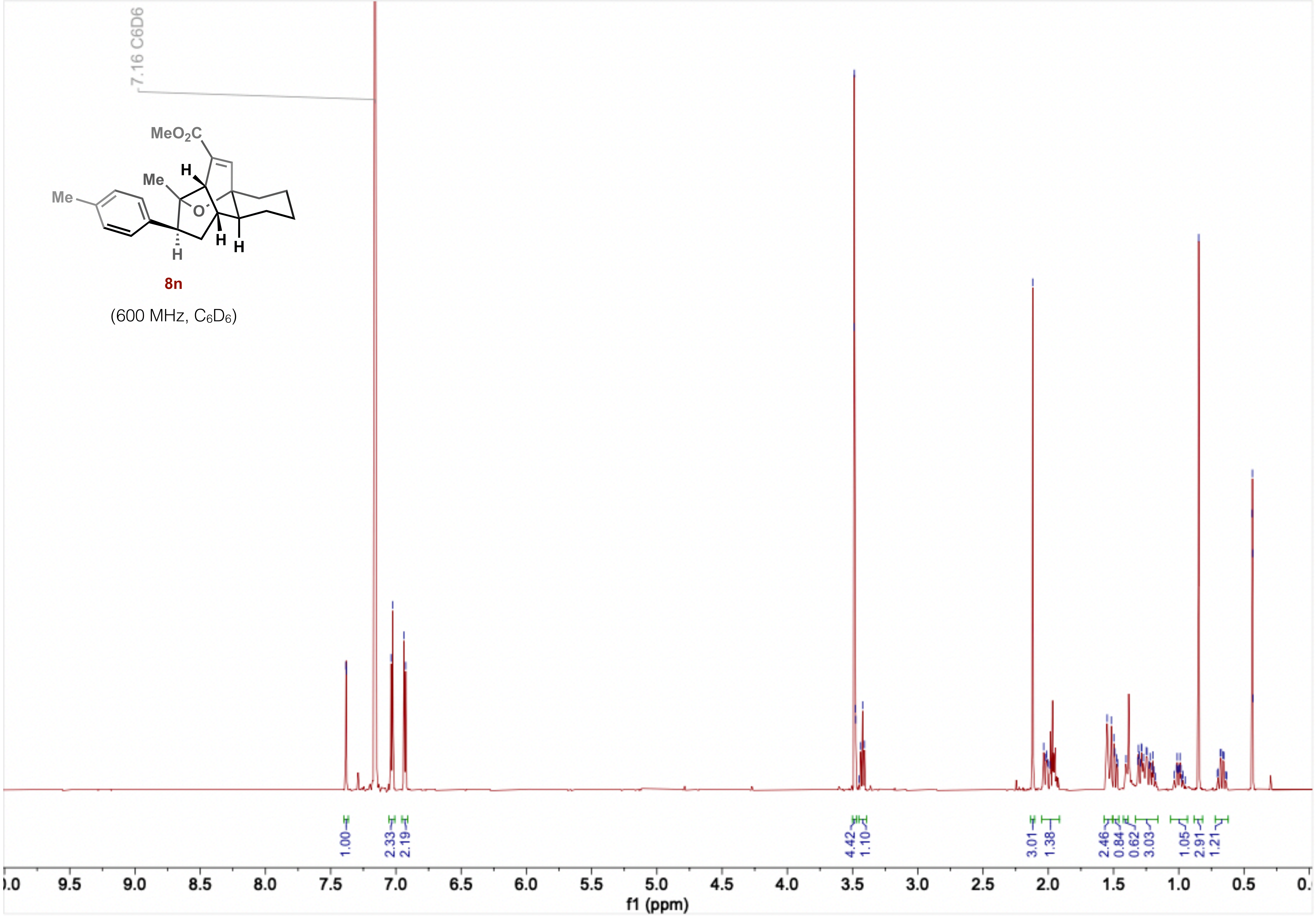

7.16 C6D6
MeO2C
Me
H
O
Me
H
H
H
8n
(600 MHz, C6D6)
1.00
2.33
2.19
4.42
1.10
3.01
1.38
2.46
0.84
0.62
3.03
1.05
2.91
1.21
f1 (ppm)

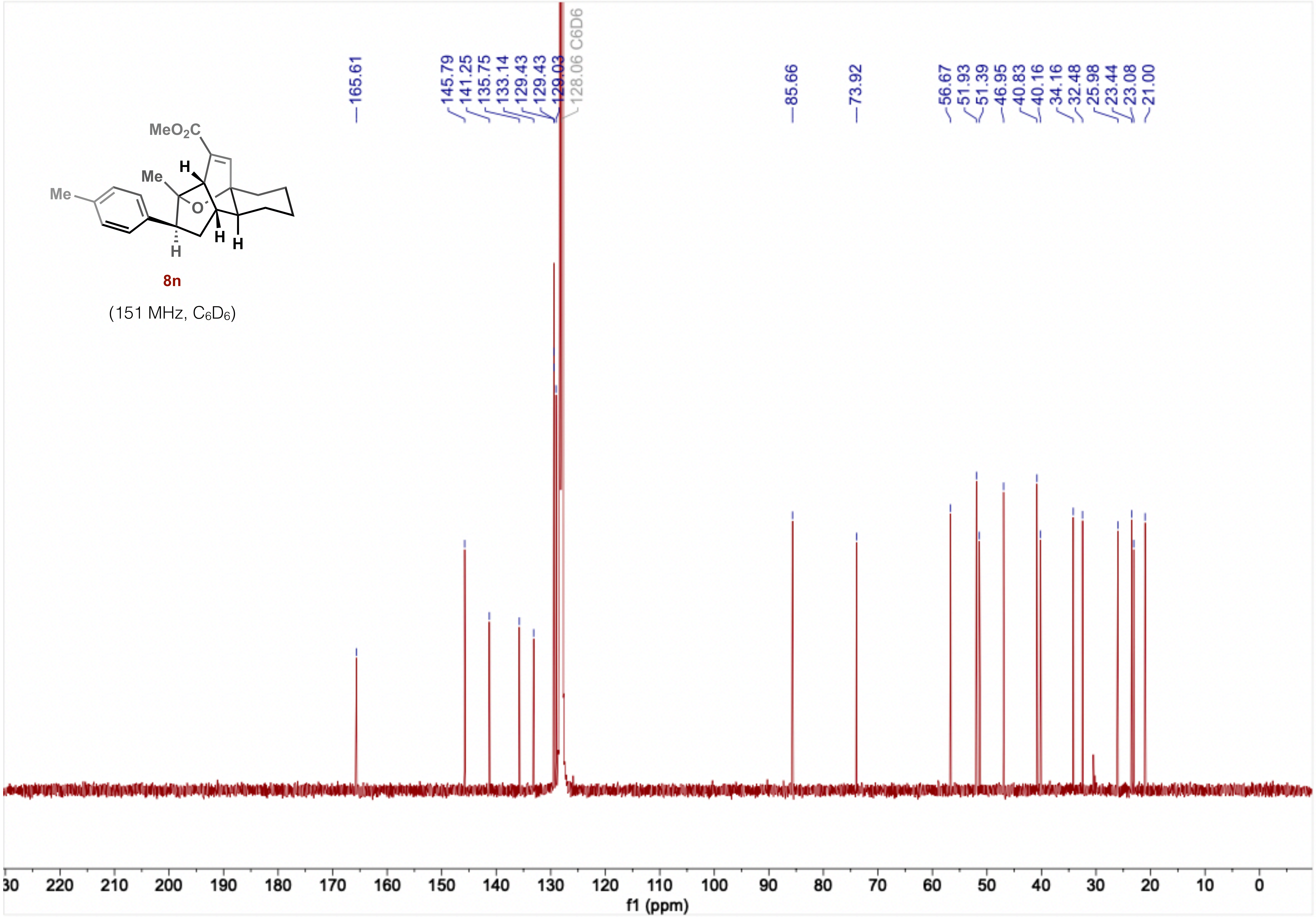

MeO2C
Me
H
O
Me
H
H
H
8n
(151 MHz, C6D6)
165.61
145.79
141.25
135.75
133.14
129.43
129.43
129.03
128.06 C6D6
85.66
73.92
56.67
51.93
51.39
46.95
40.83
40.16
34.16
32.48
25.98
23.44
23.08
21.00
30
220
210
200
190
180
170
160
150
140
130
120
110
100
90
80
70
60
50
40
30
20
10
0
f1 (ppm)

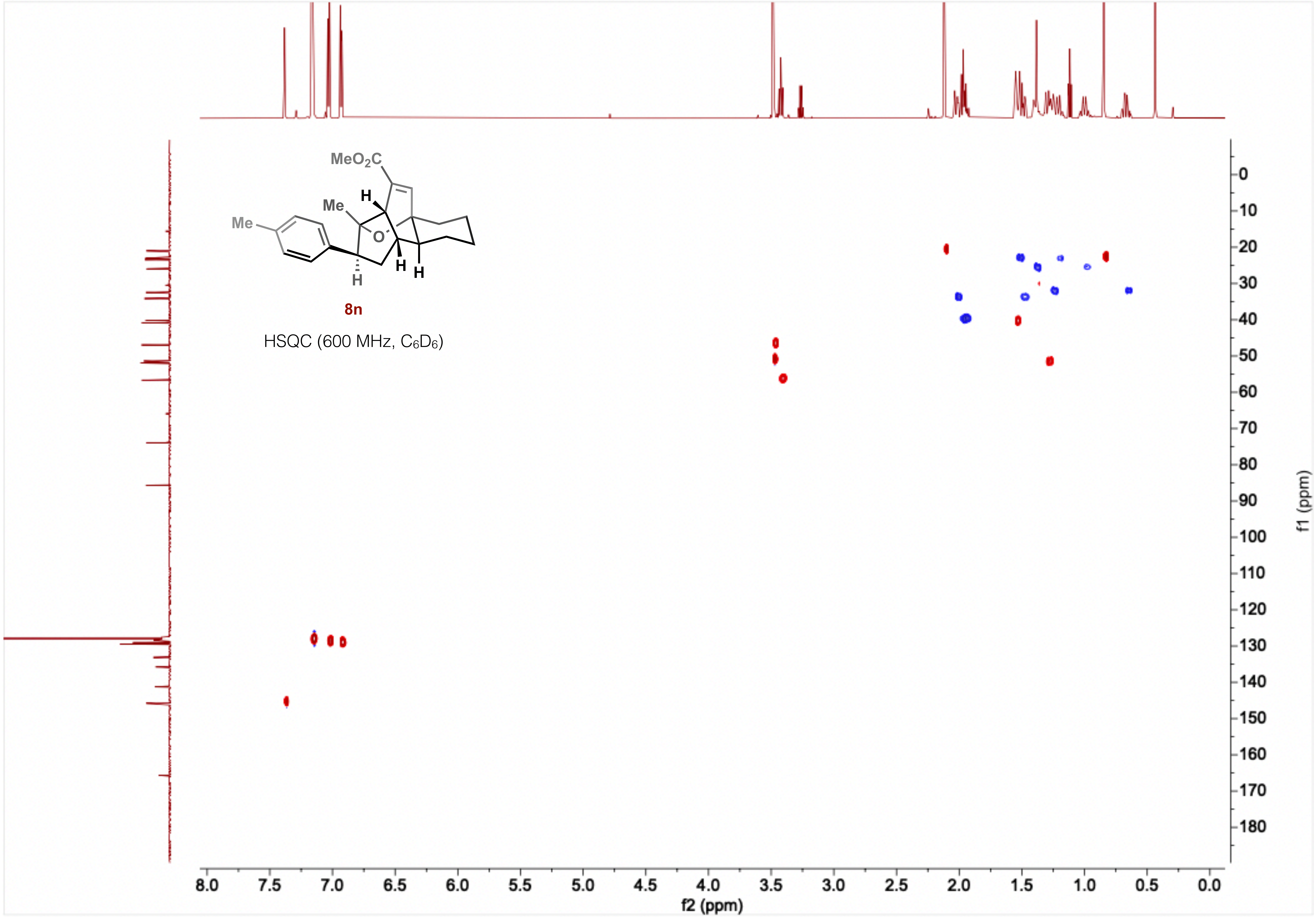
MeO2C
H
Me
Me
O
H
H
H
8n
HSQC (600 MHz, C6D6)
f1 (ppm)
f2 (ppm)

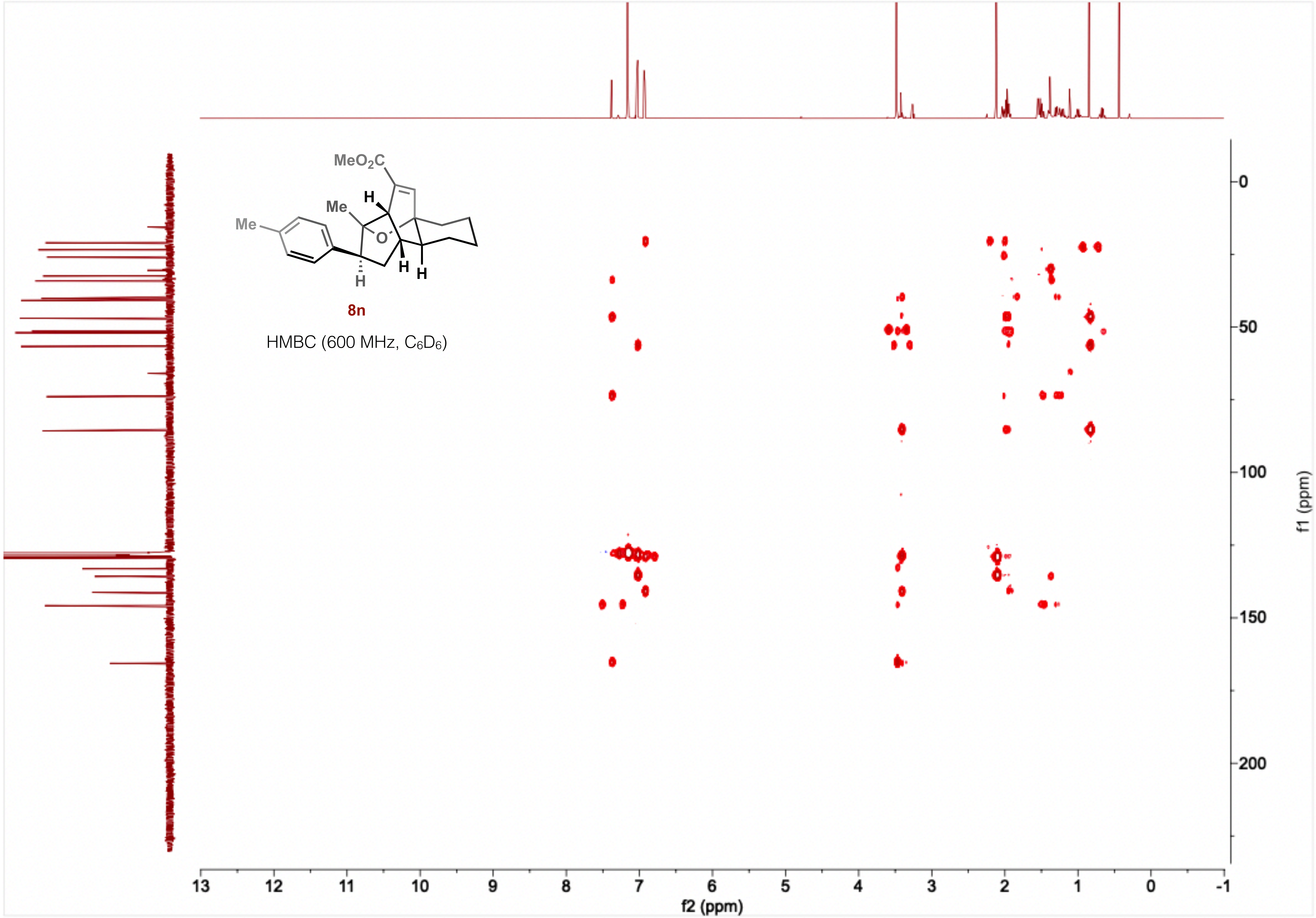

MeO2C
Me
Me
H
O
H
H
H
8n
HMBC (600 MHz, C6D6)
f1 (ppm)
f2 (ppm)

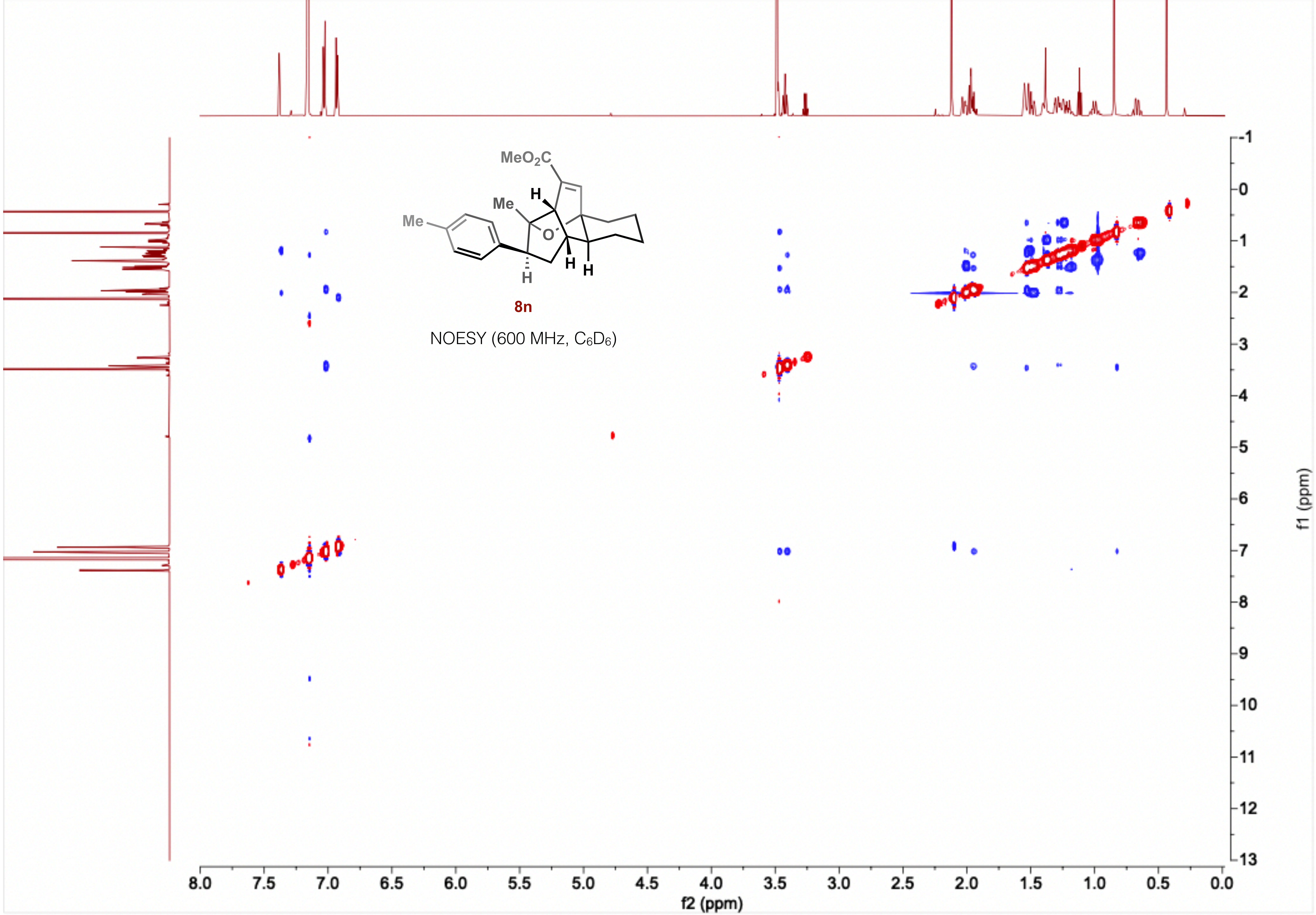
MeO2C
Me
Me
O
H
H
H
H
8n
NOESY (600 MHz, C6D6)
f2 (ppm)
f1 (ppm)

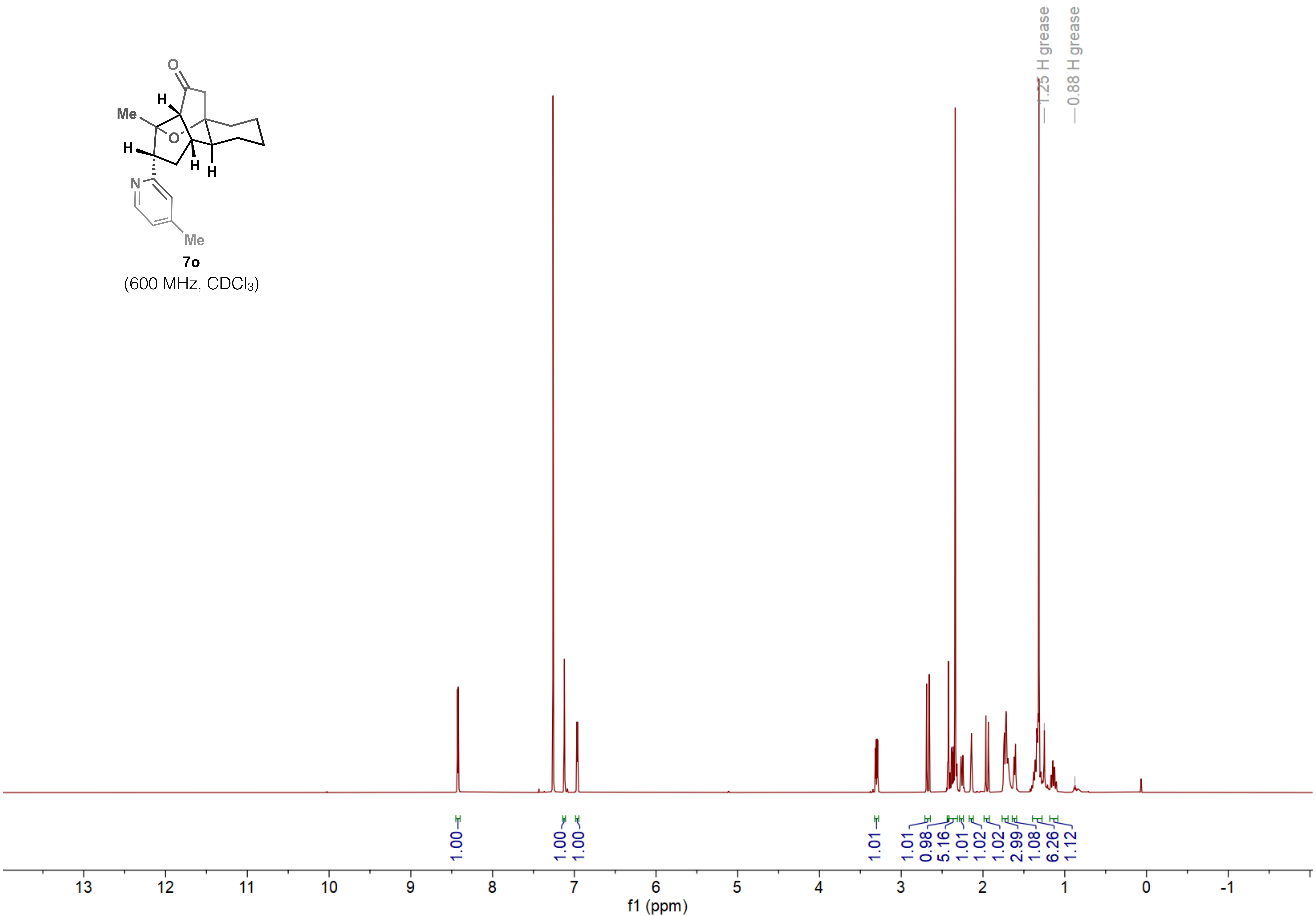

Me
H
O
H
N
H
H
Me
7o
(600 MHz, CDCl3)
1.25 H grease
0.88 H grease
1.00
1.00
1.00
1.01
1.01
0.98
5.16
1.01
1.02
1.02
2.99
1.08
6.26
1.12
13 12 11 10 9 8 7 6 5 4 3 2 1 0 -1
f1 (ppm)

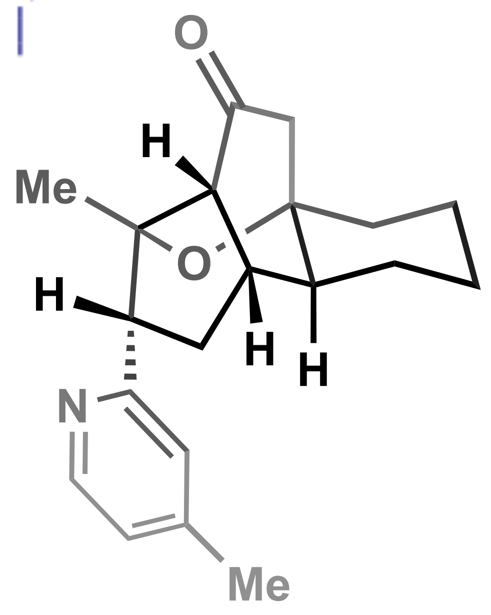

7o

(151 MHz, $CDCl_3$)

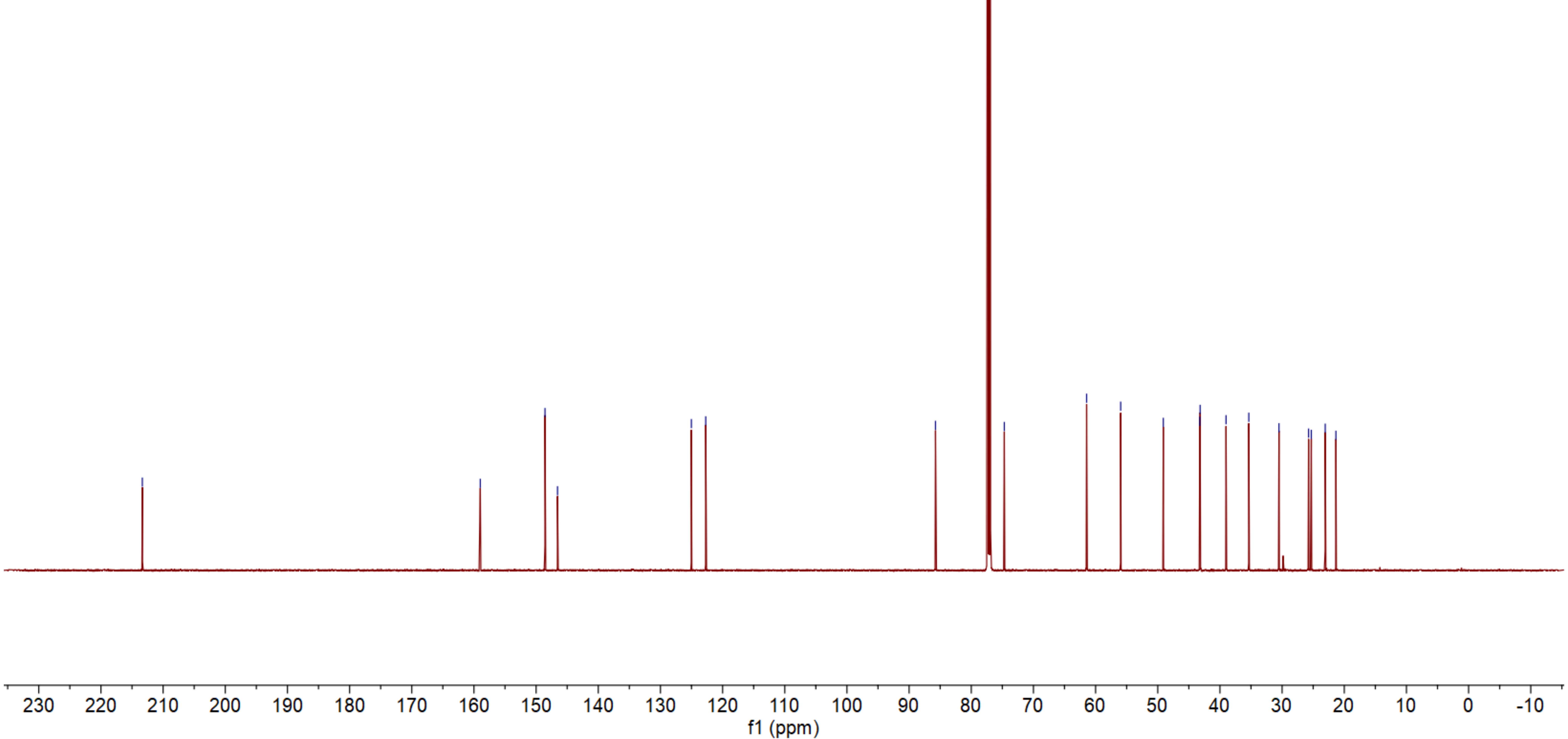

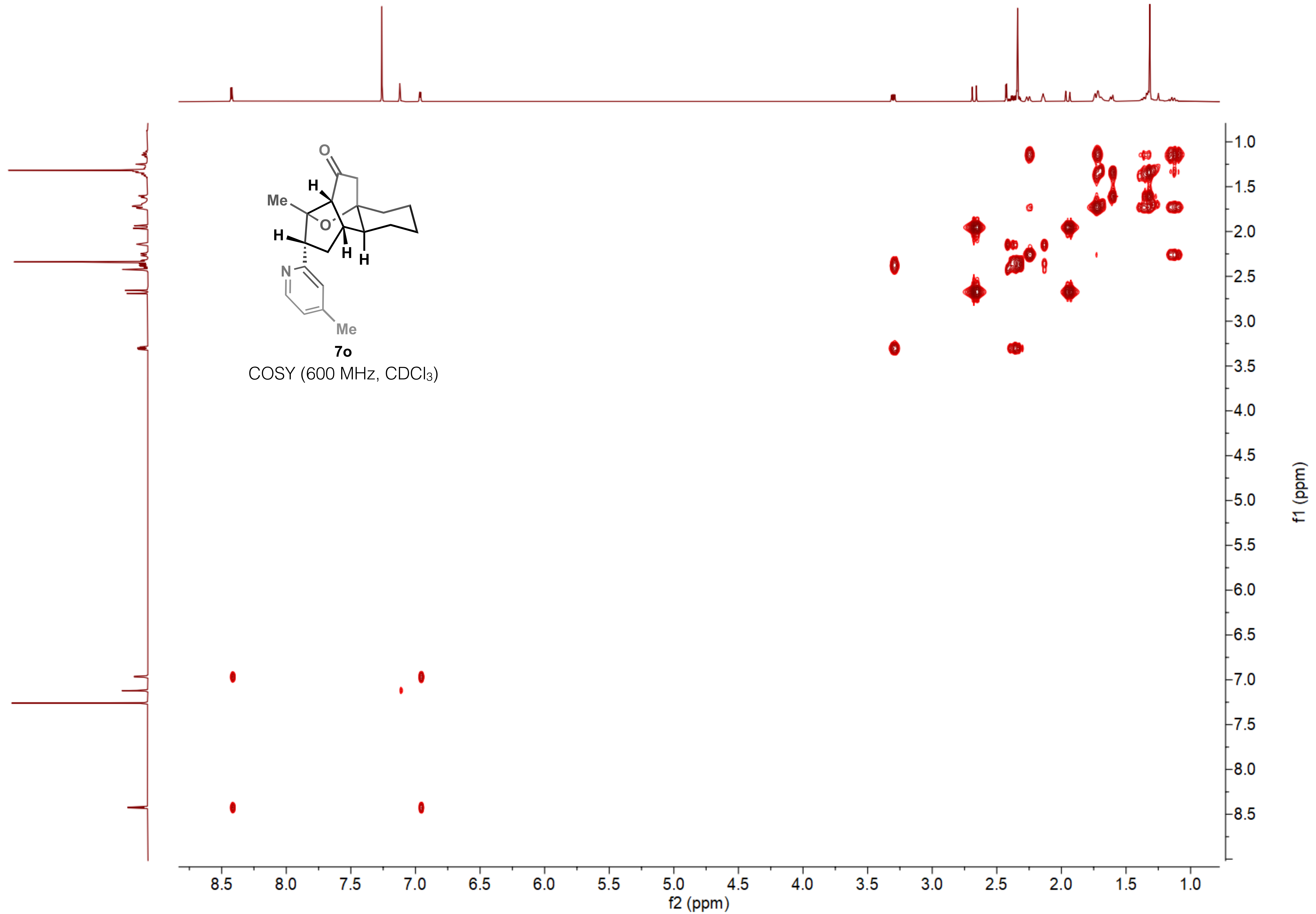

O
H
Me
O
H
H
H
N
Me
7o
COSY (600 MHz, CDCl3)
f2 (ppm)
f1 (ppm)

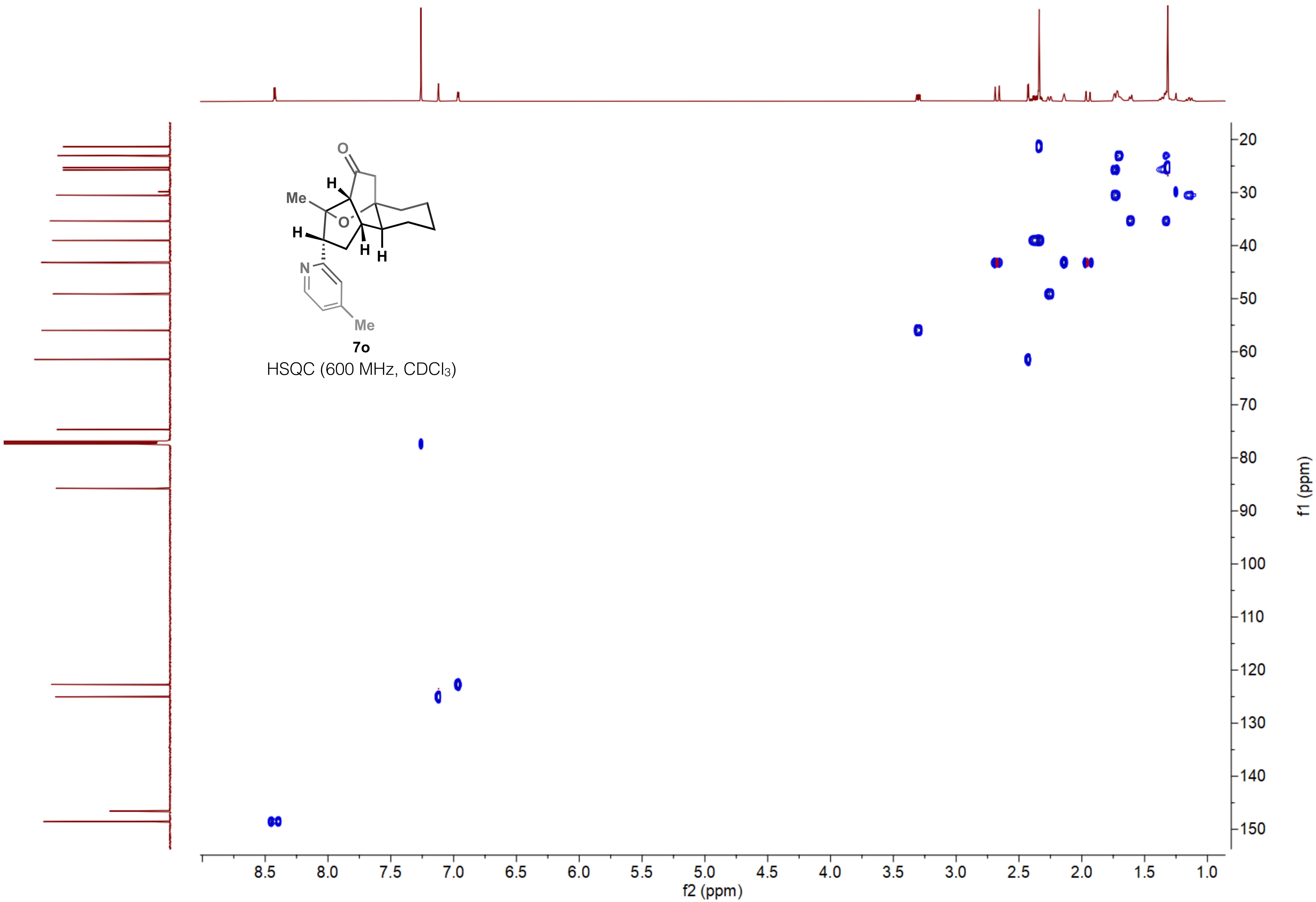

O
H
Me
O
H
H
H
N
Me
7o
HSQC (600 MHz, CDCl3)
f2 (ppm)
f1 (ppm)

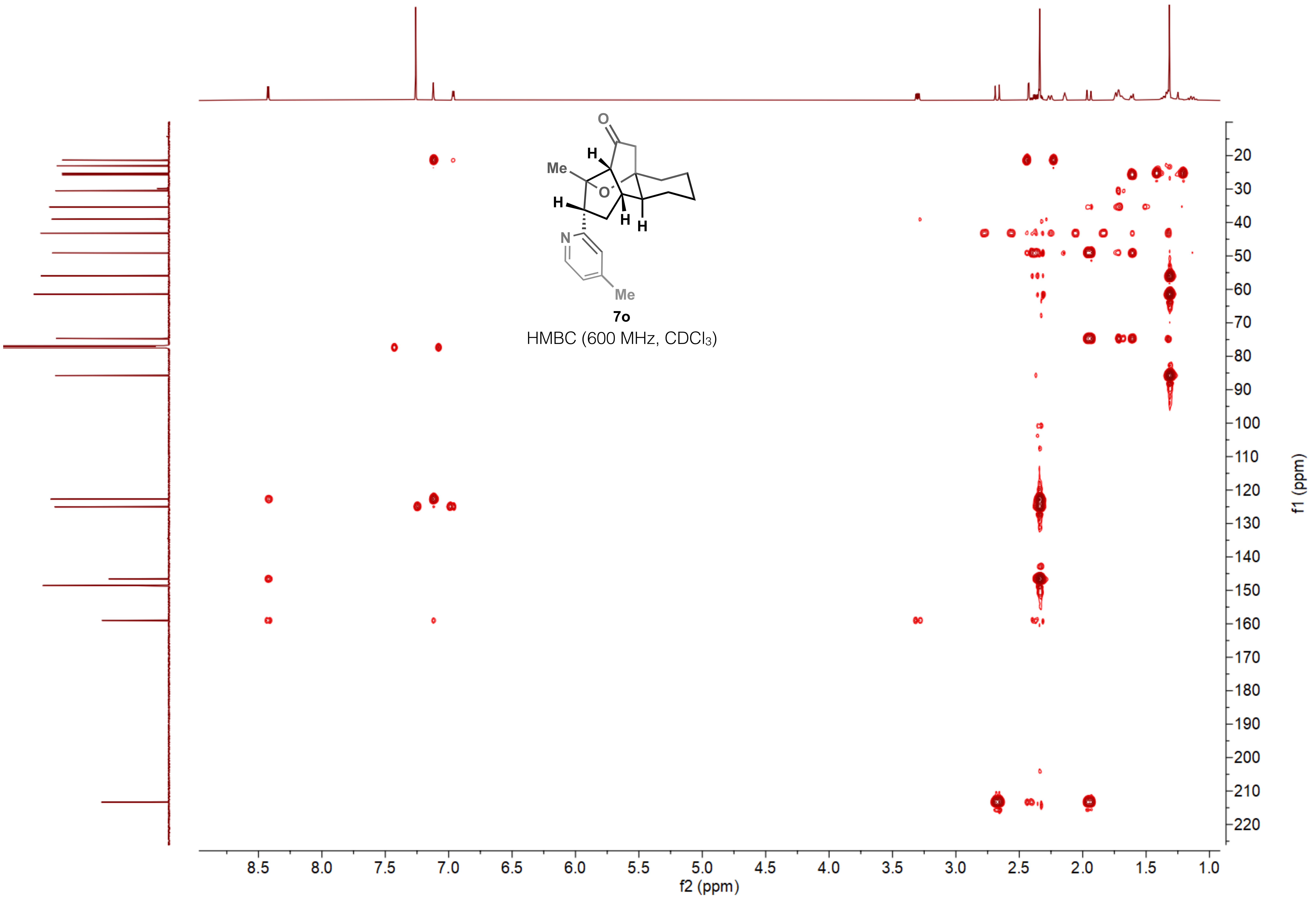

O
H
Me
O
H
H
H
N
Me
7o
HMBC (600 MHz, CDCl3)
f2 (ppm)
f1 (ppm)

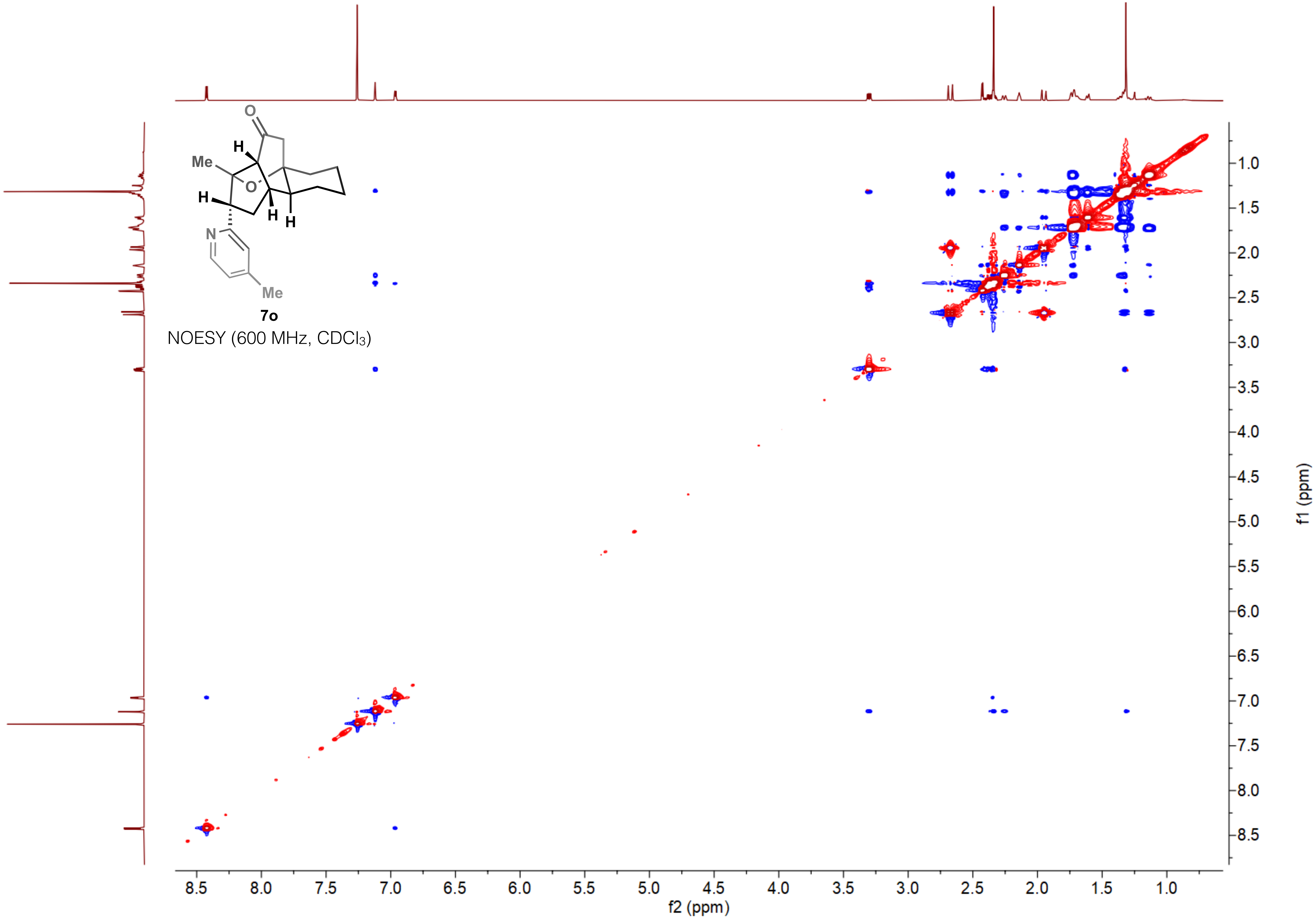
O
H
Me
O
H
N
H
H
Me
7o
NOESY (600 MHz, CDCl3)
f2 (ppm)
f1 (ppm)
8.5
8.0
7.5
7.0
6.5
6.0
5.5
5.0
4.5
4.0
3.5
3.0
2.5
2.0
1.5
1.0

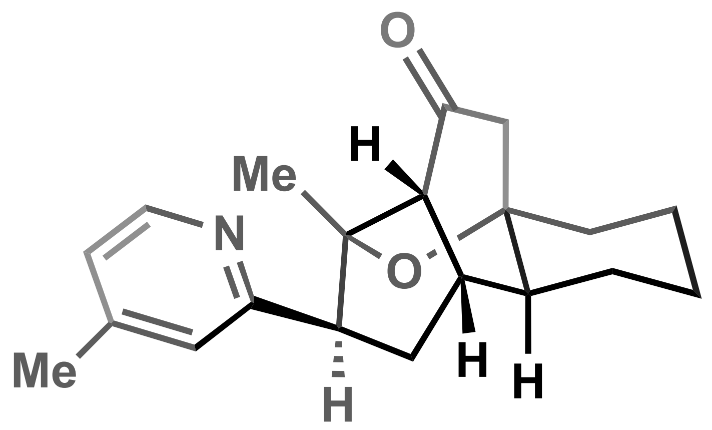

8o

(600 MHz, $CDCl_3$)

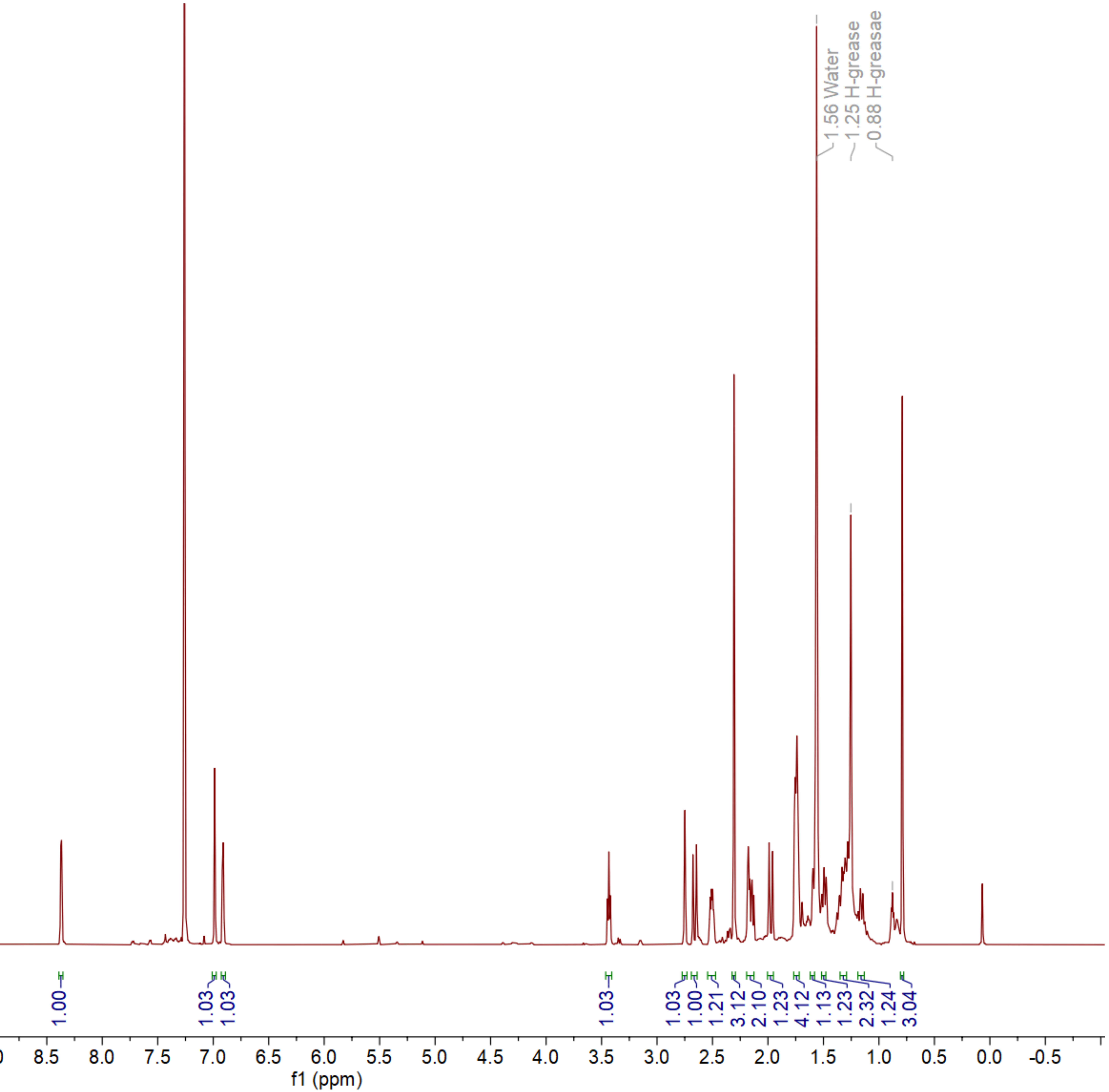

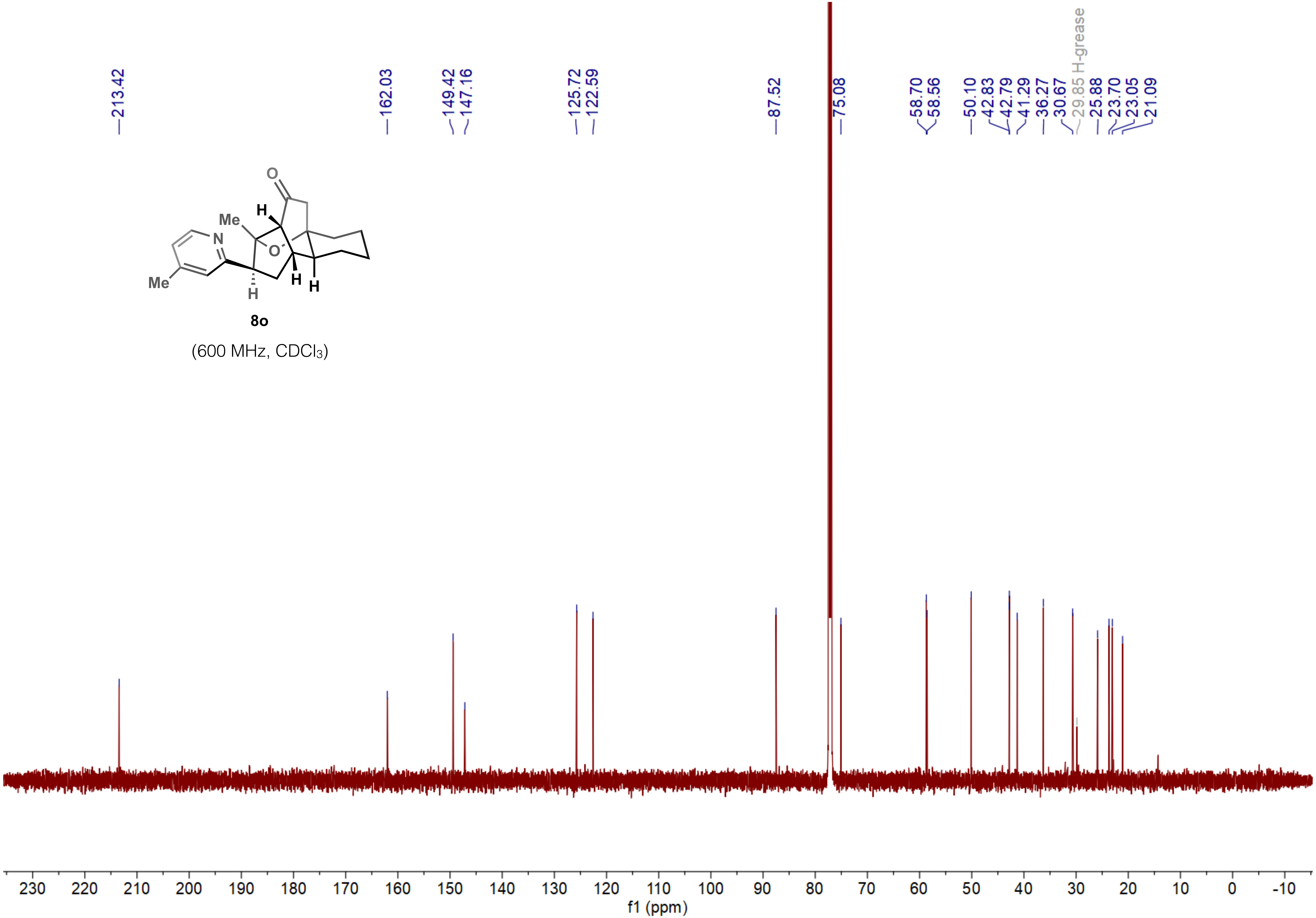

8o

(600 MHz, $CDCl_3$)

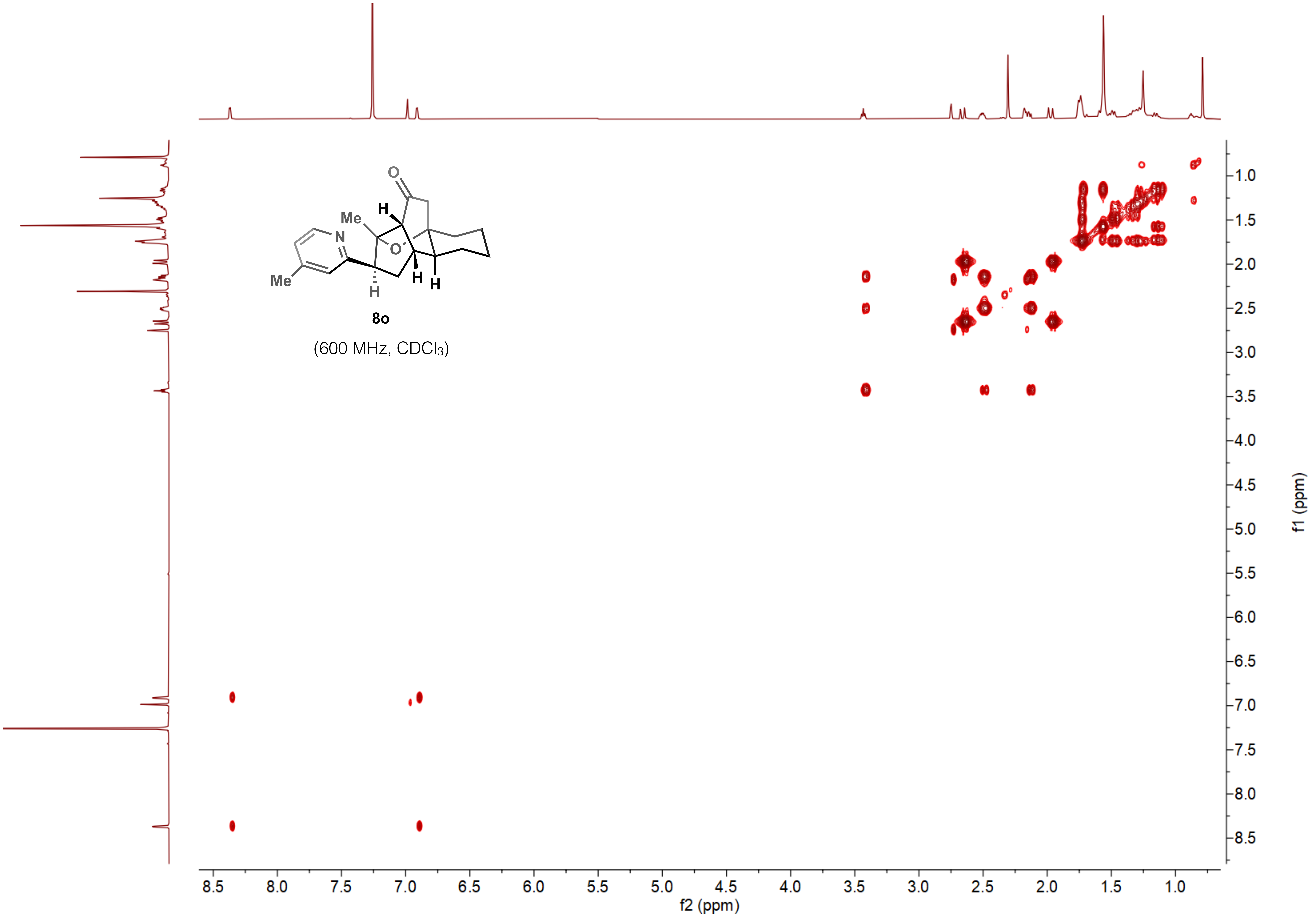

O
Me
N
H
O
Me
H
H
H
8o
(600 MHz, CDCl3)
8.5
8.0
7.5
7.0
6.5
6.0
5.5
5.0
4.5
4.0
3.5
3.0
2.5
2.0
1.5
1.0
f2 (ppm)
f1 (ppm)

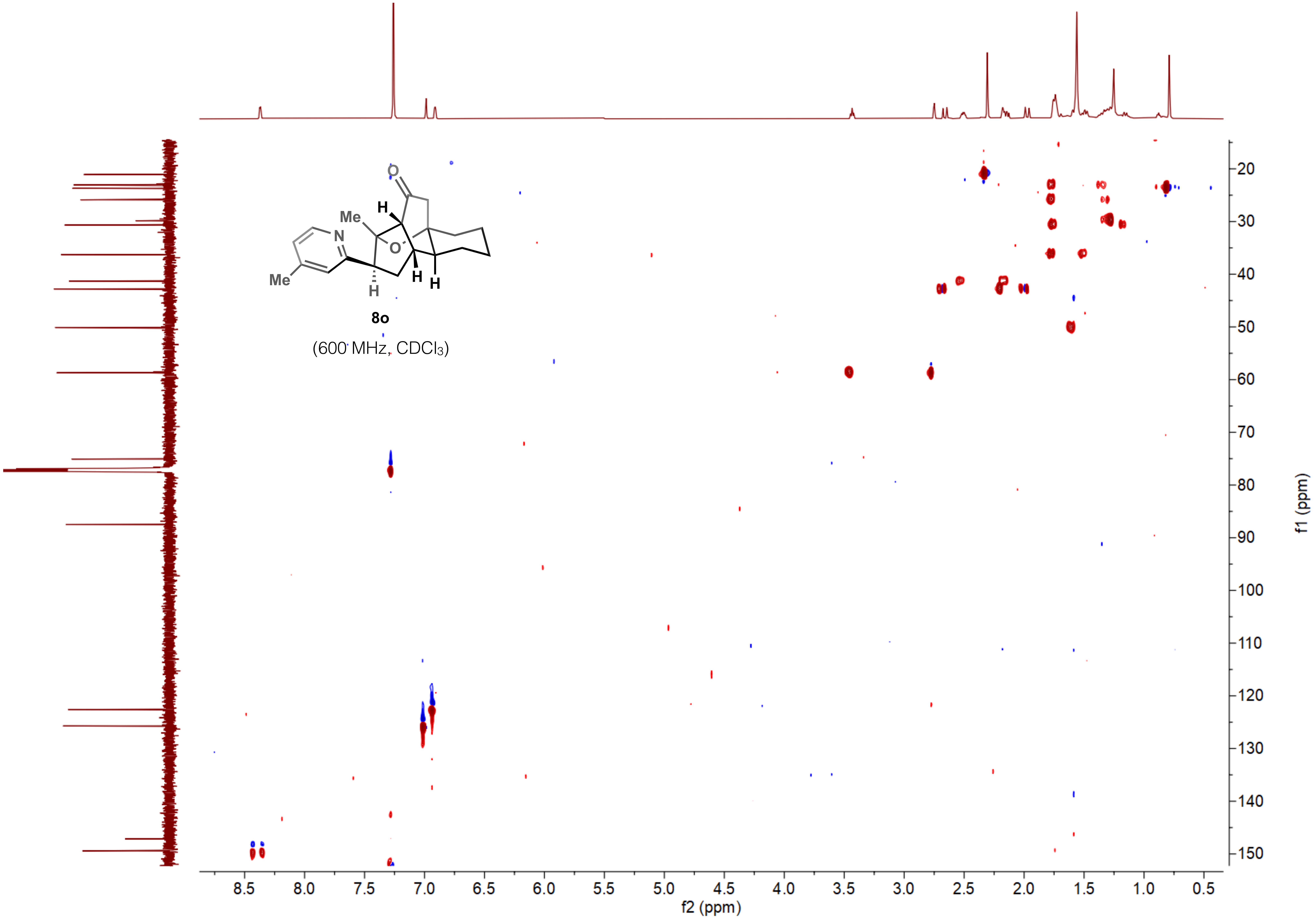
8o
(600 MHz, CDCl3)
f1 (ppm)
f2 (ppm)

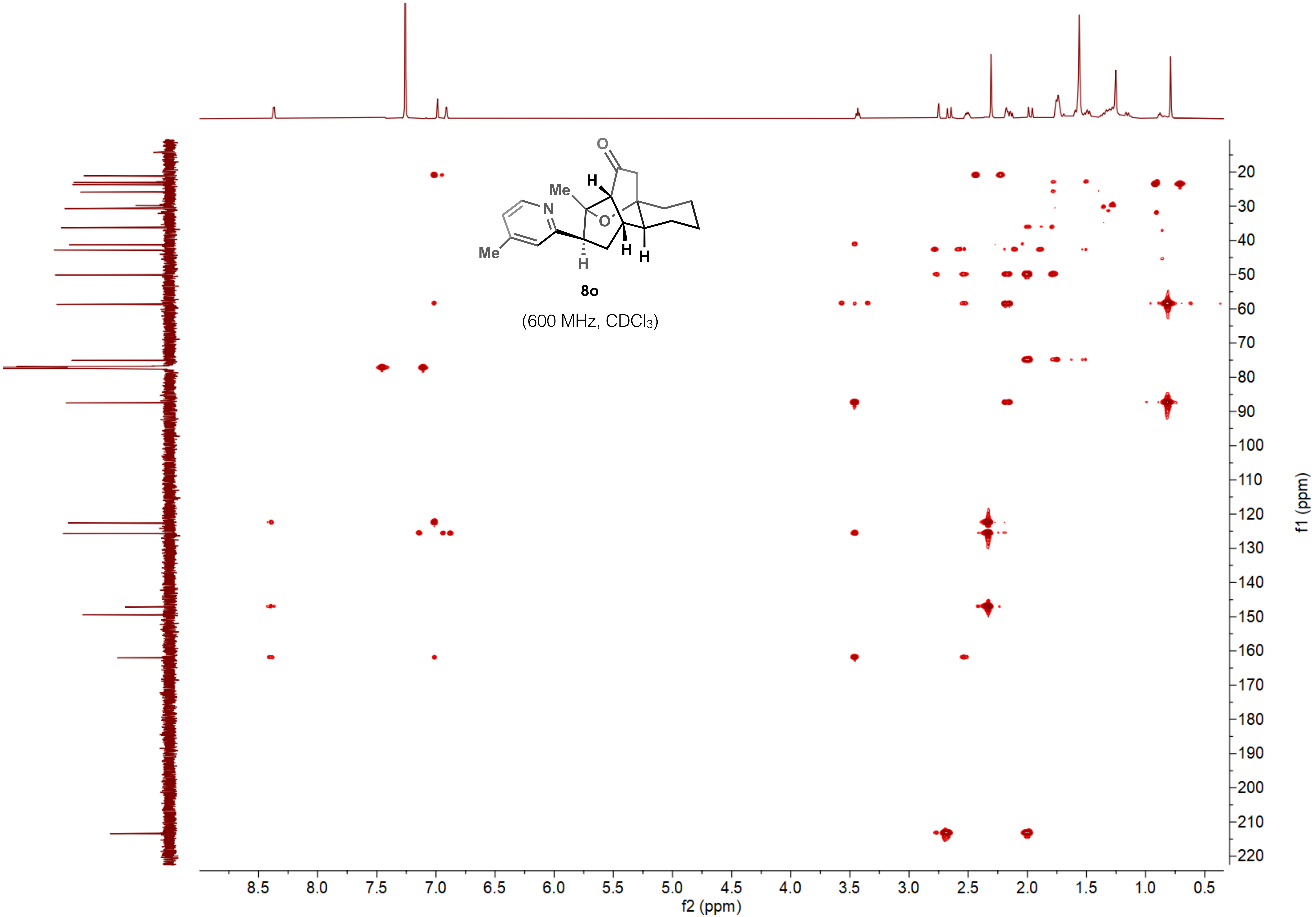

8o

(600 MHz, $CDCl_3$)

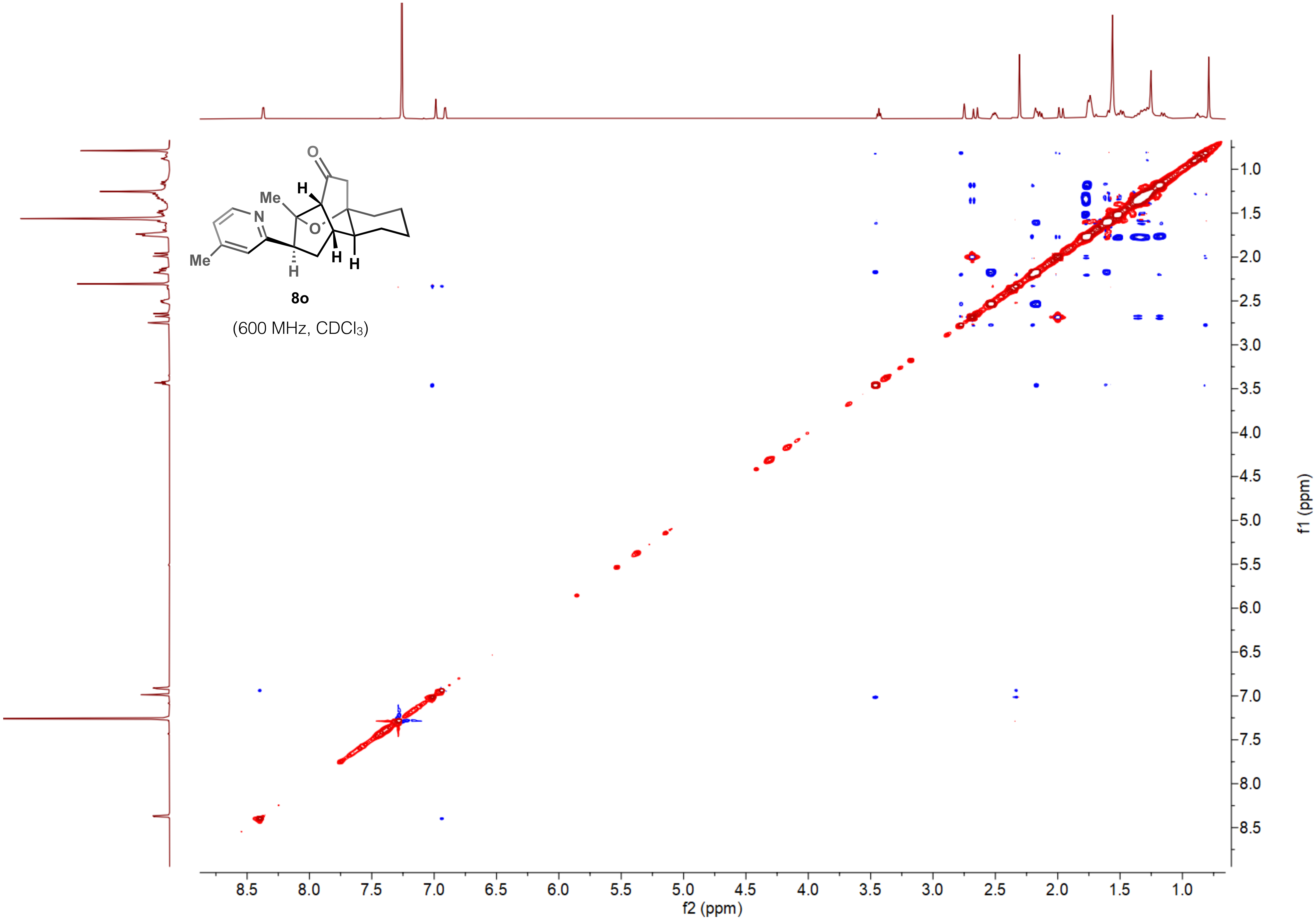

8o
(600 MHz, CDCl3)
f2 (ppm)
f1 (ppm)

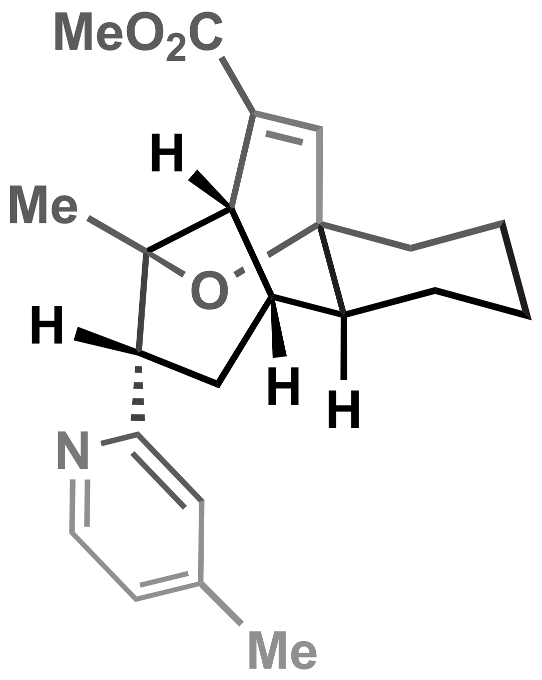

RS1129

(600 MHz, $CDCl_3$)

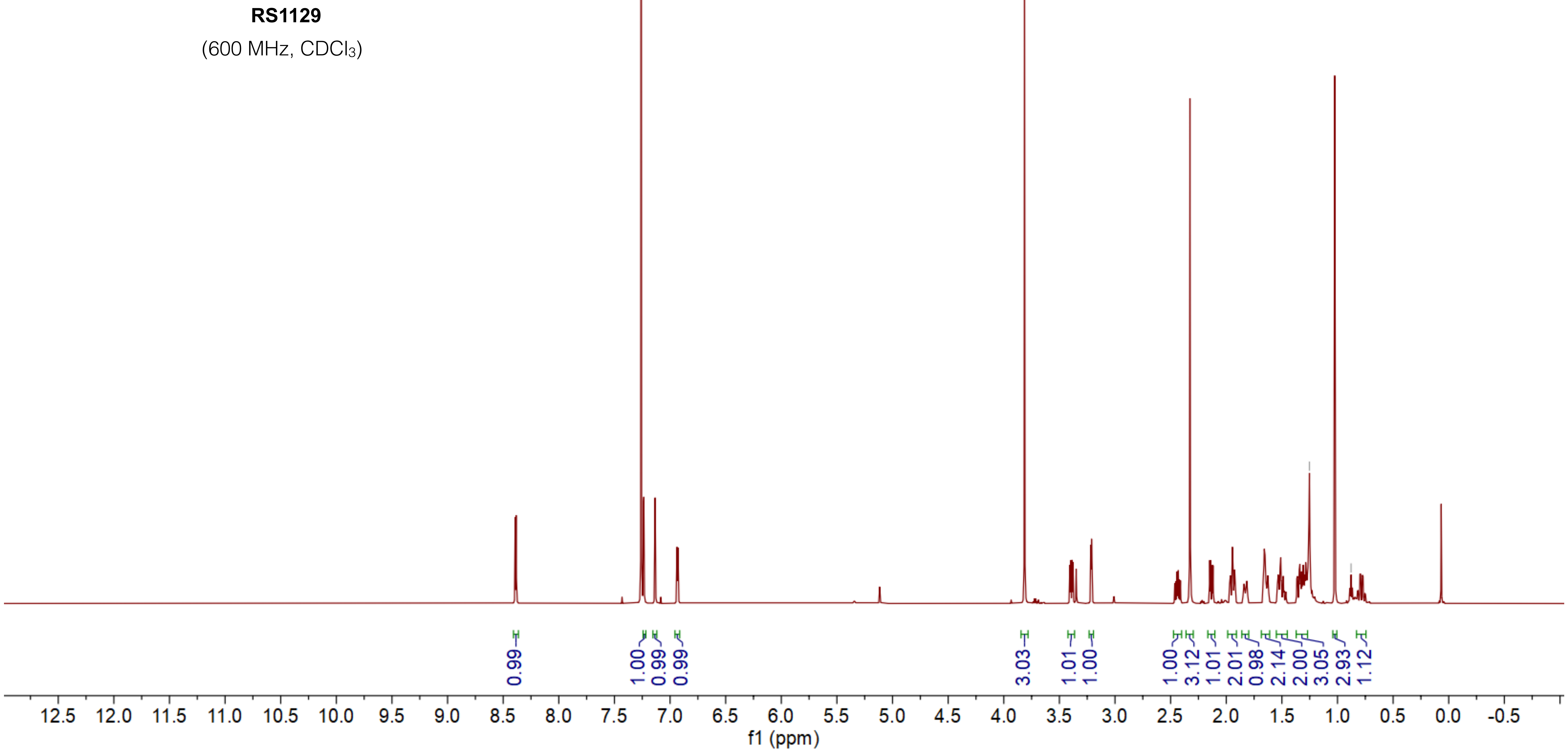

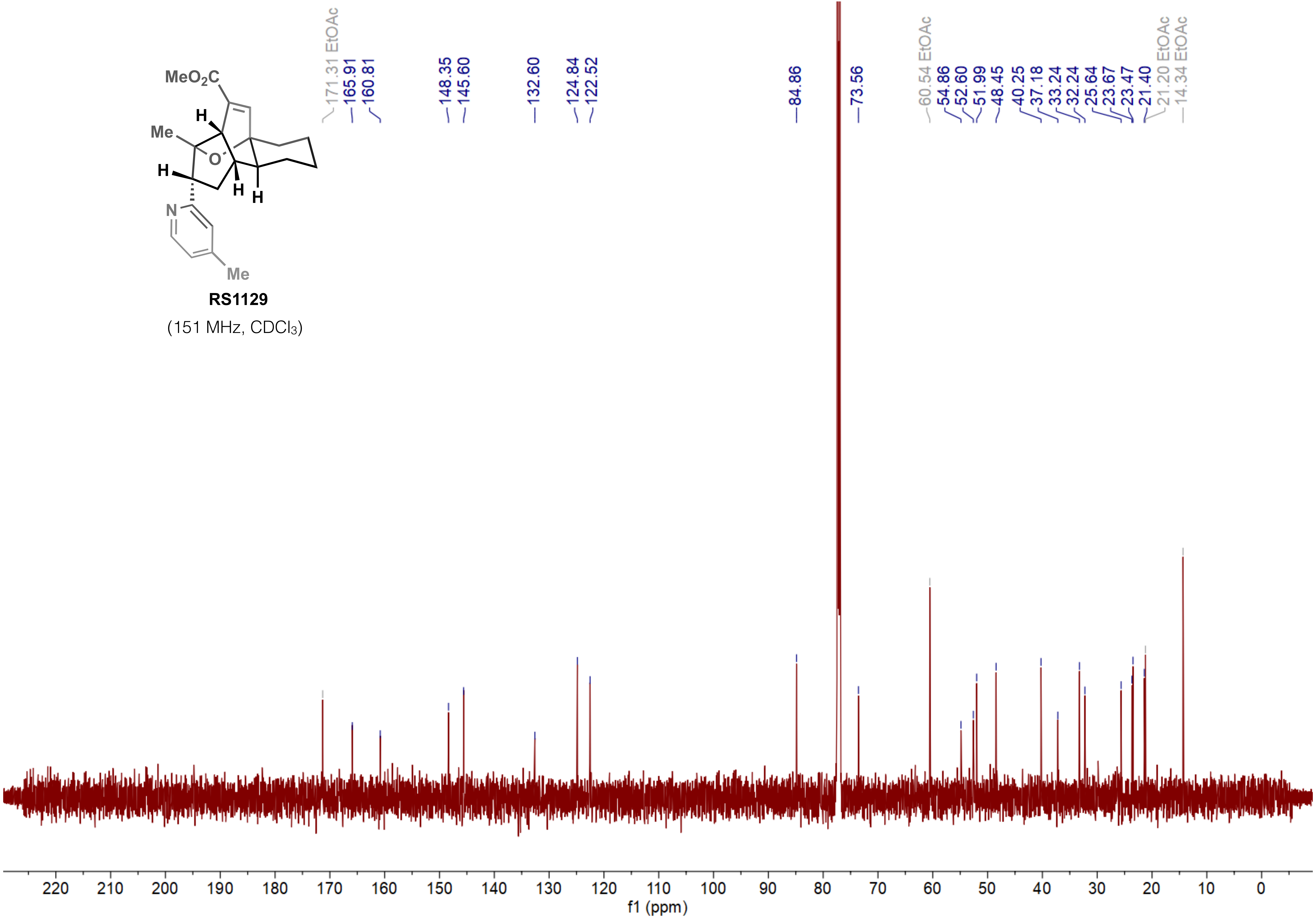
MeO2C
Me
H
O
H
H
H
N
Me
RS1129
(151 MHz, CDCl3)
171.31 EtOAc
165.91
160.81
148.35
145.60
132.60
124.84
122.52
84.86
73.56
60.54 EtOAc
54.86
52.60
51.99
48.45
40.25
37.18
33.24
32.24
25.64
23.67
23.47
21.40
21.20 EtOAc
14.34 EtOAc
220
210
200
190
180
170
160
150
140
130
120
110
100
90
80
70
60
50
40
30
20
10
0
f1 (ppm)

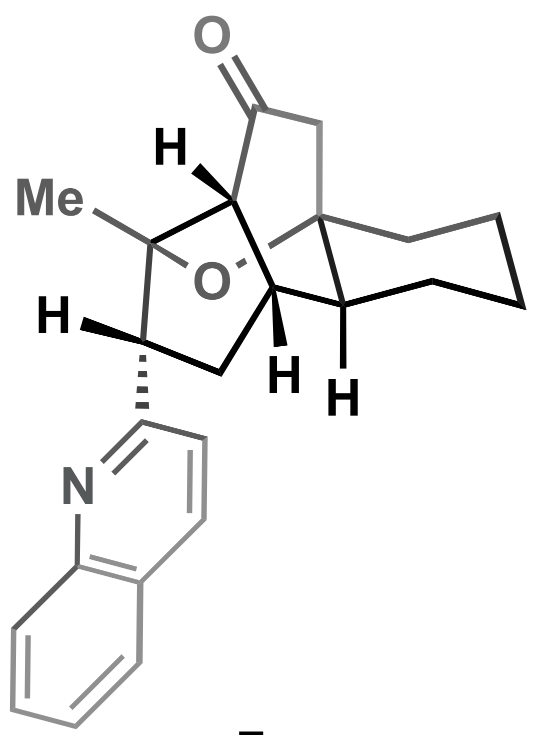

7p

(600 MHz, $CDCl_3$)

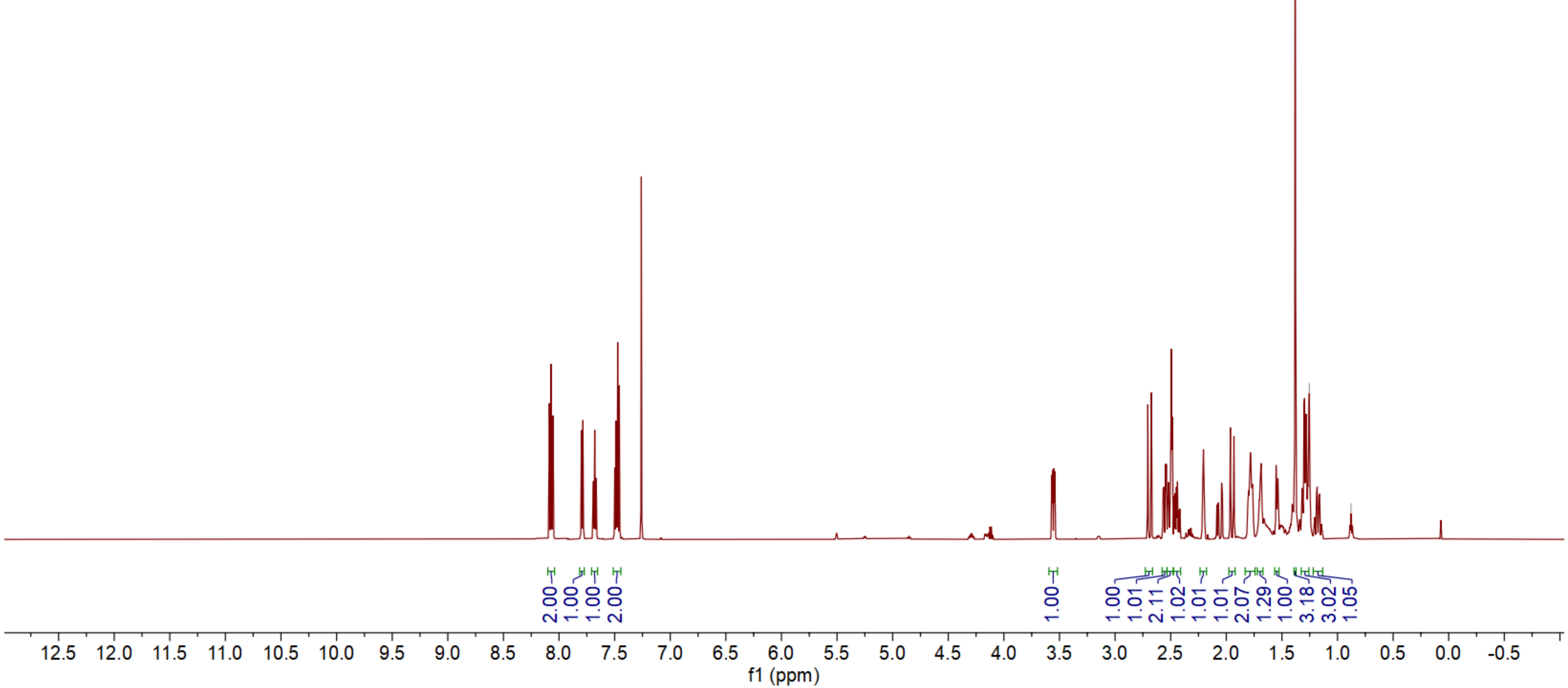

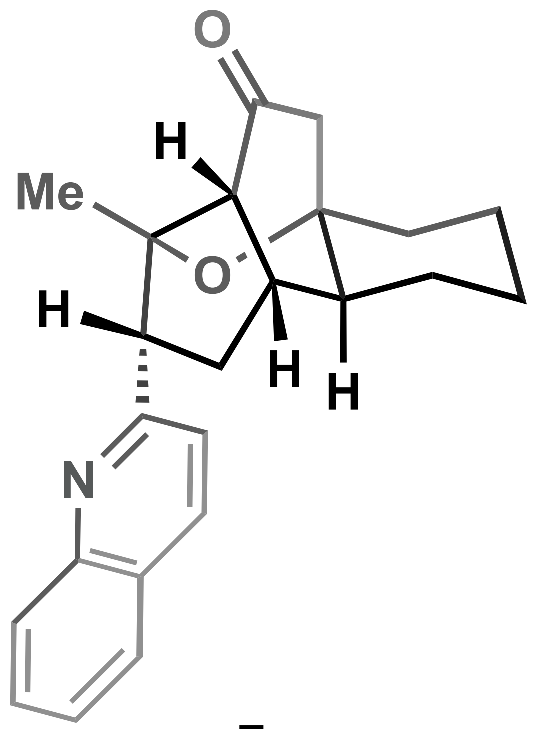

7p

(151 MHz, $CDCl_3$)

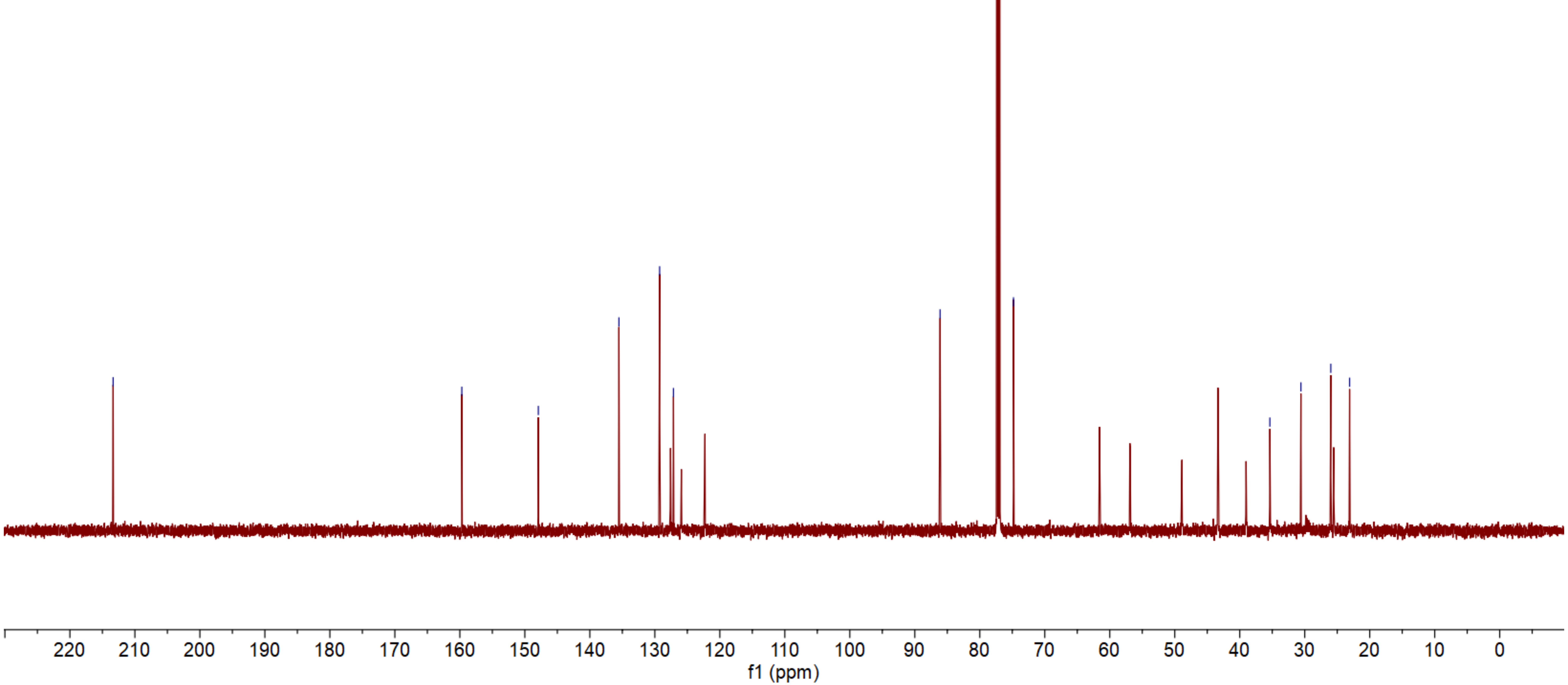

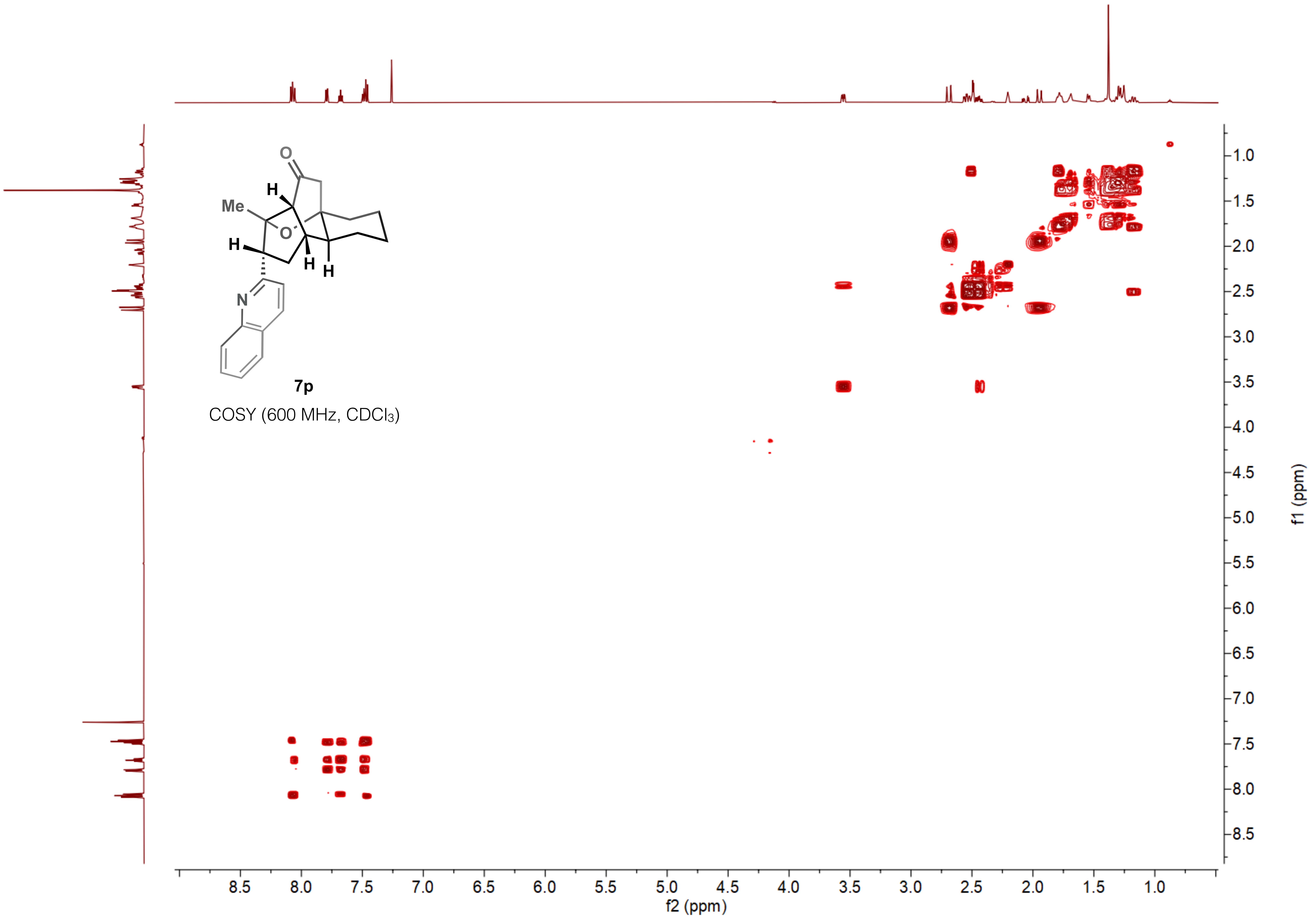

7p

COSY (600 MHz, $CDCl_3$)

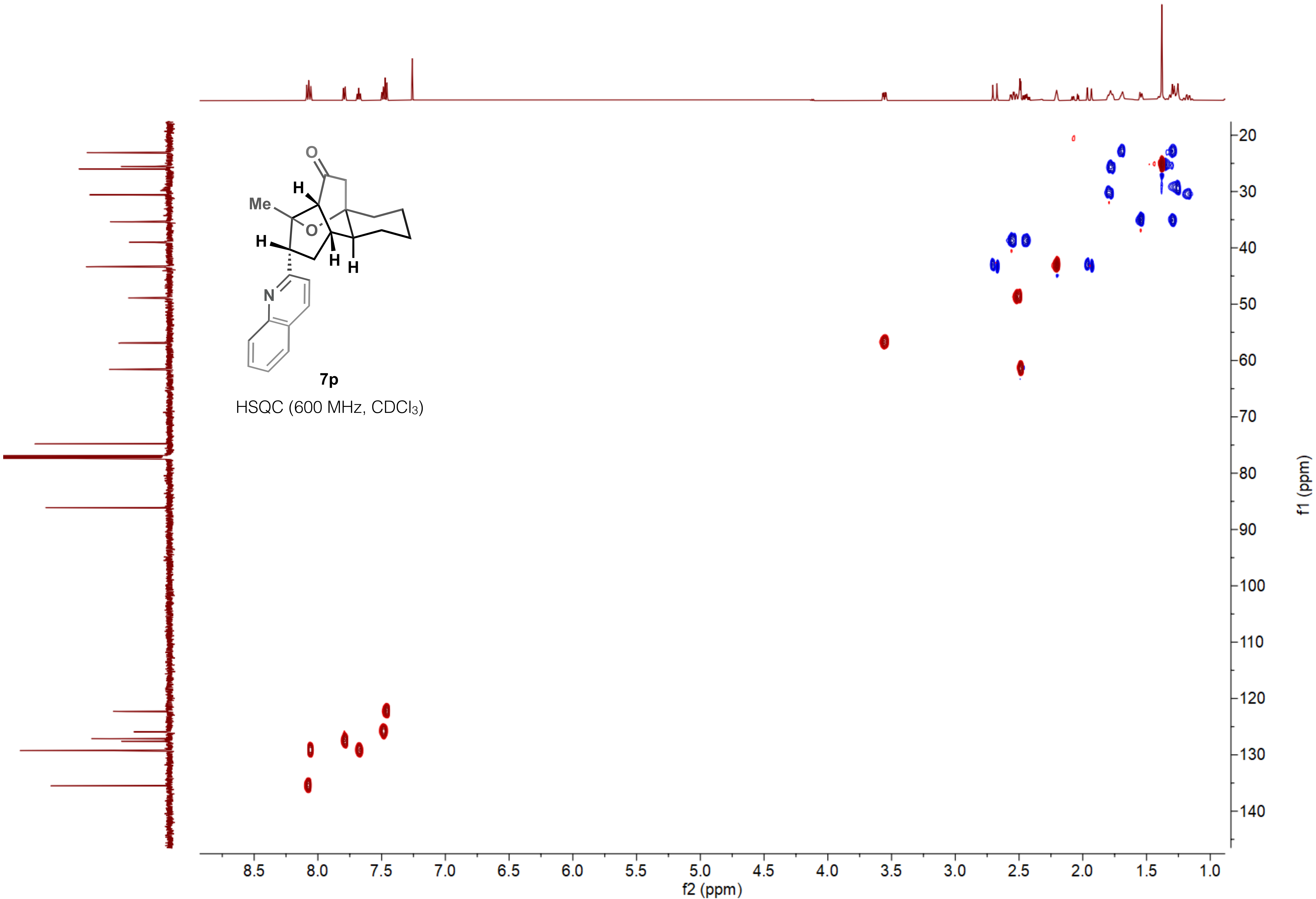

O
Me
H
H
H
H
N
7p
HSQC (600 MHz, CDCl3)
f1 (ppm)
f2 (ppm)

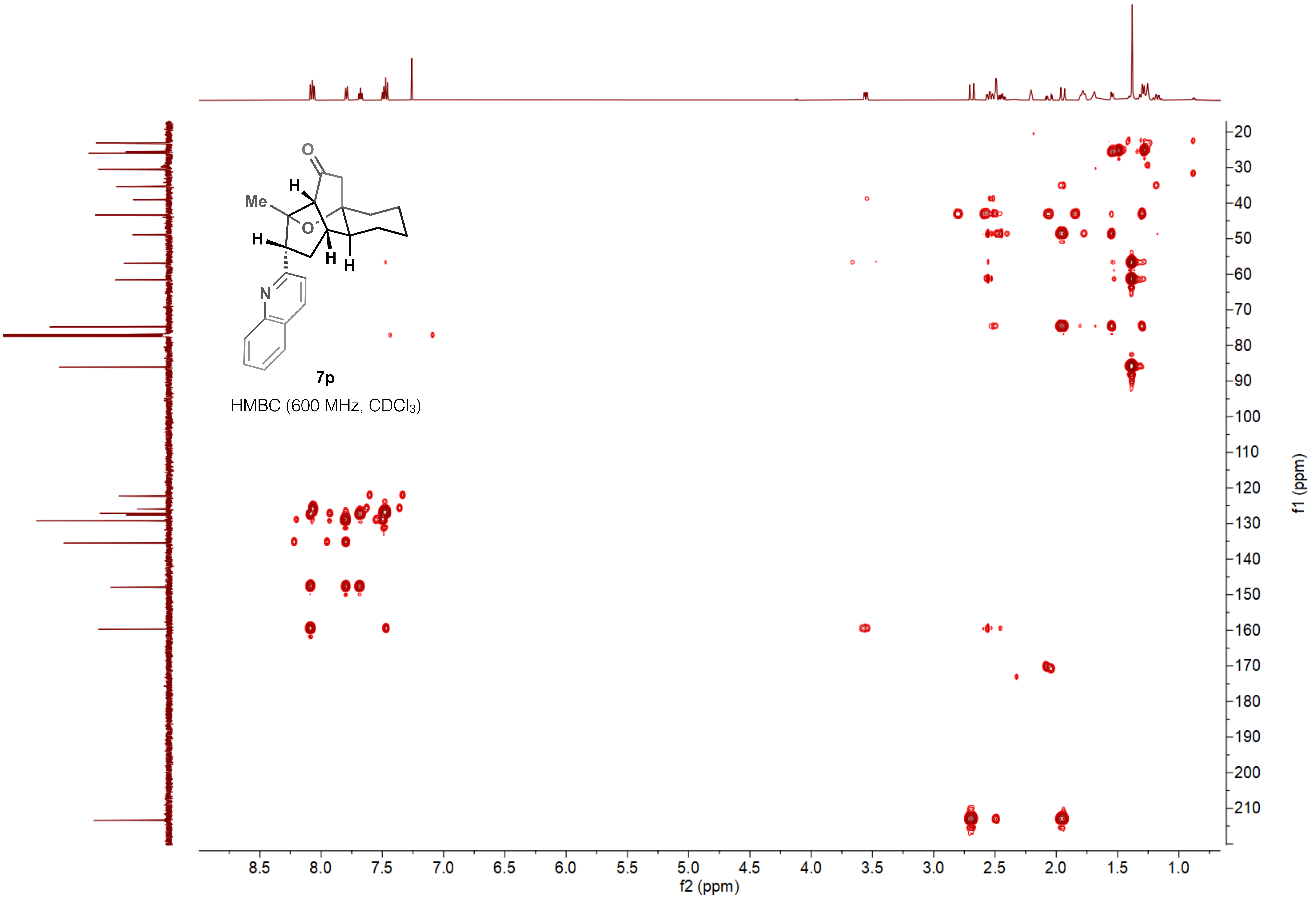
O
H
Me
O
H
H
H
N
7p
HMBC (600 MHz, CDCl3)
f1 (ppm)
f2 (ppm)

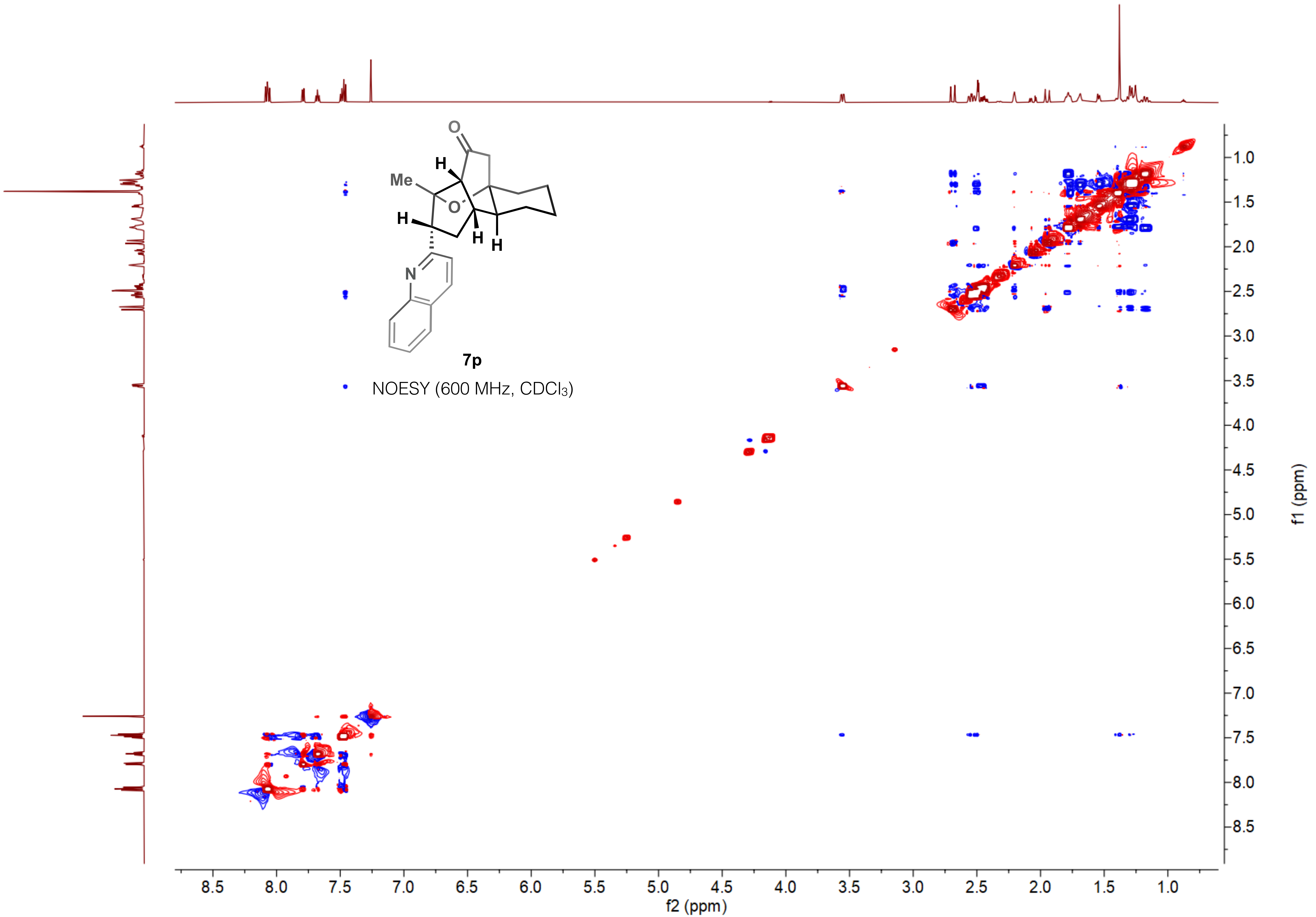

O
H
Me
O
H
N
H
H
7p
NOESY (600 MHz, CDCl3)
f2 (ppm)
f1 (ppm)

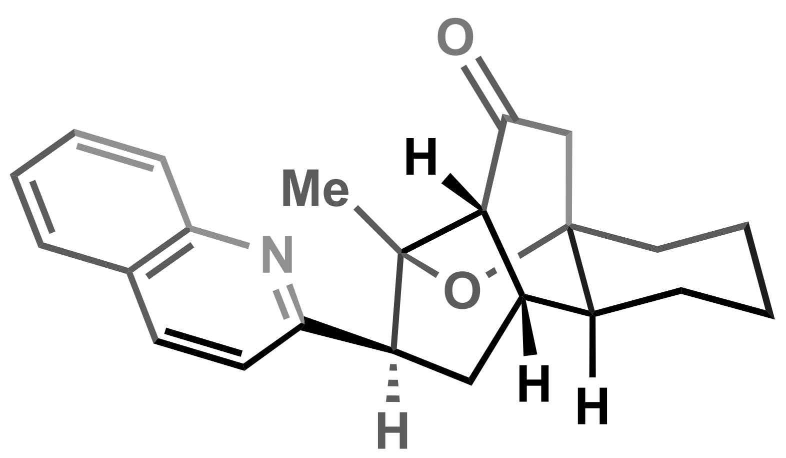

8p

(600 MHz, $CDCl_3$)

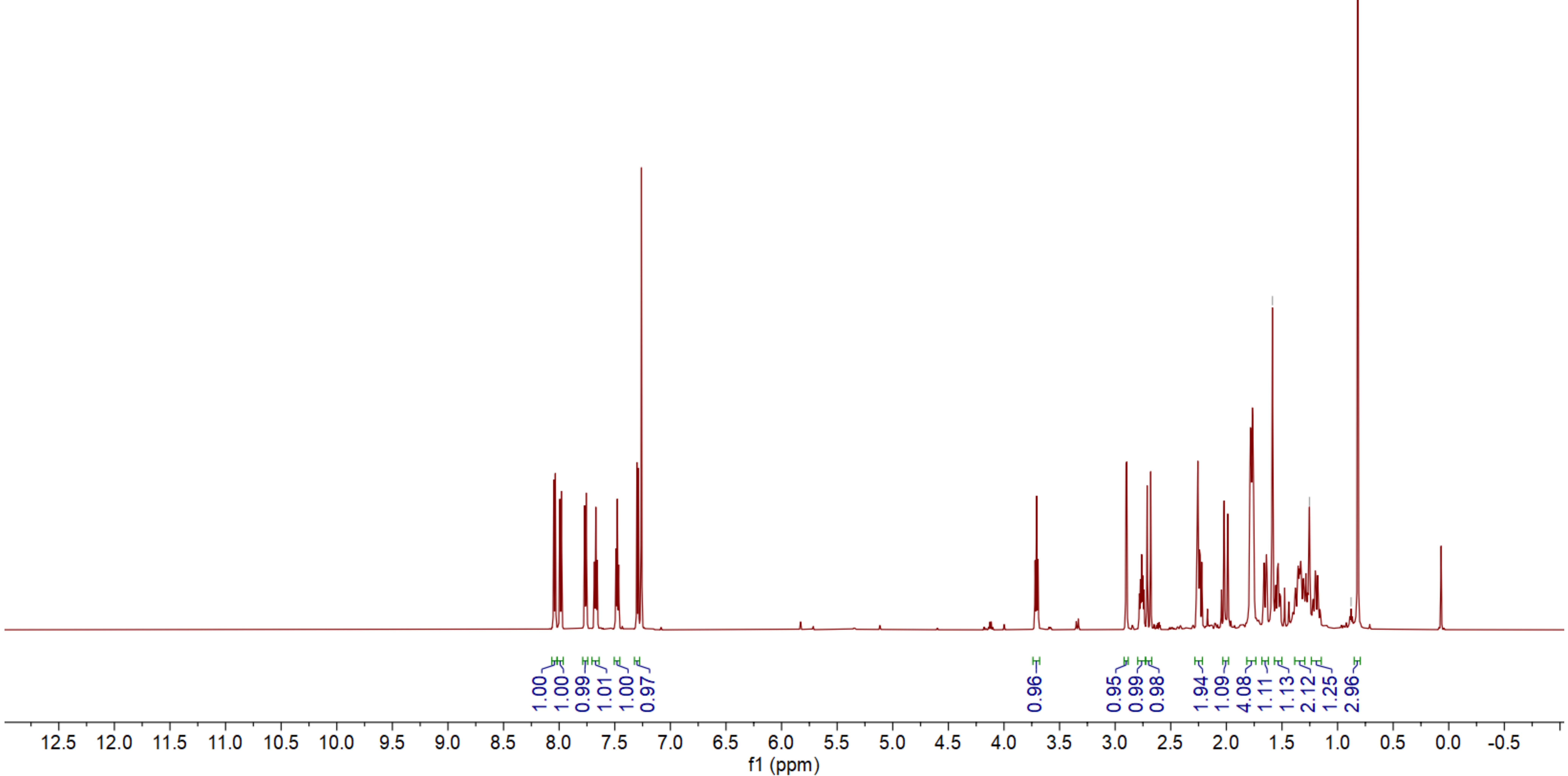

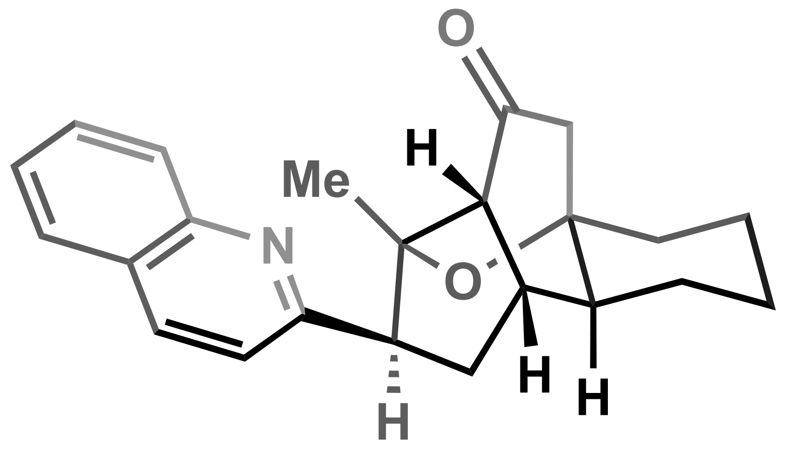

8p

(151 MHz, $CDCl_3$)

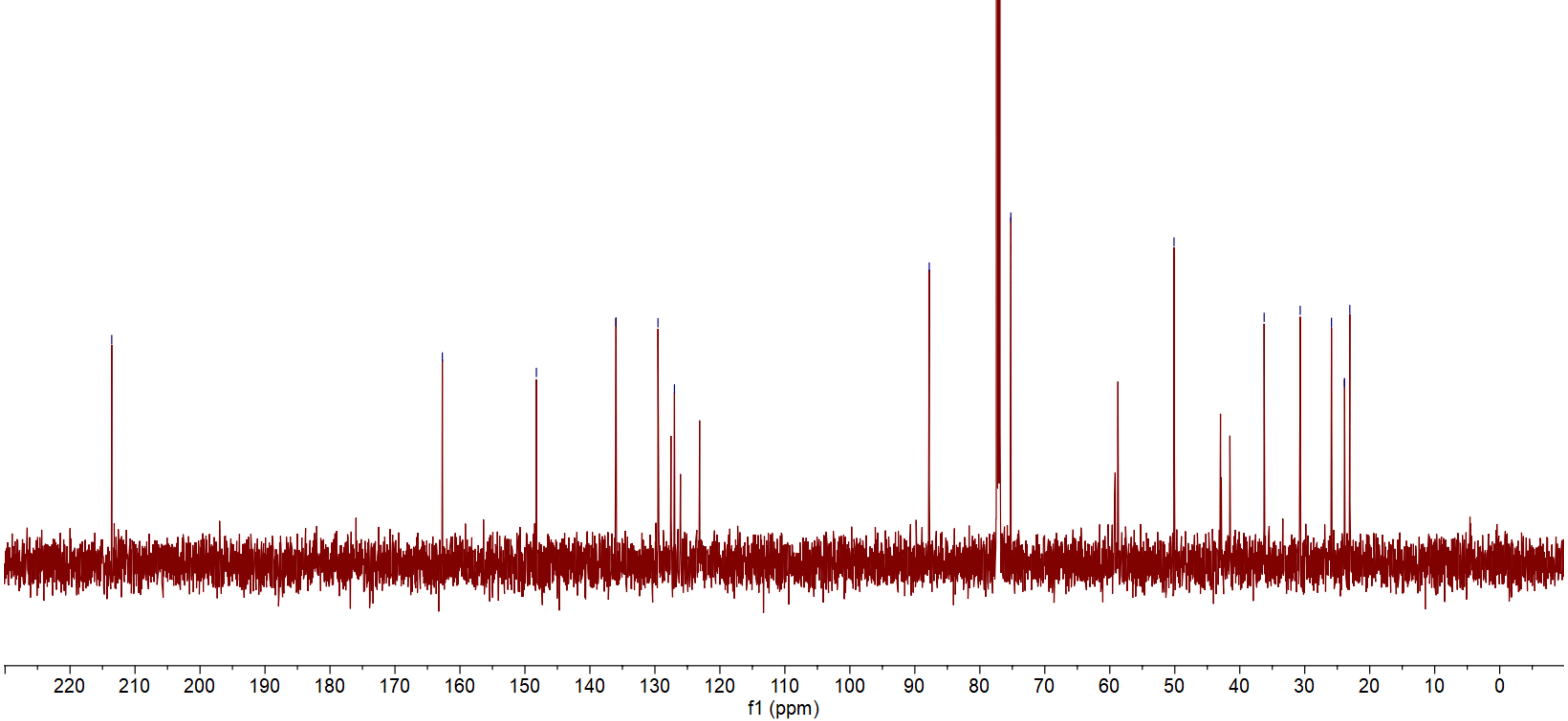

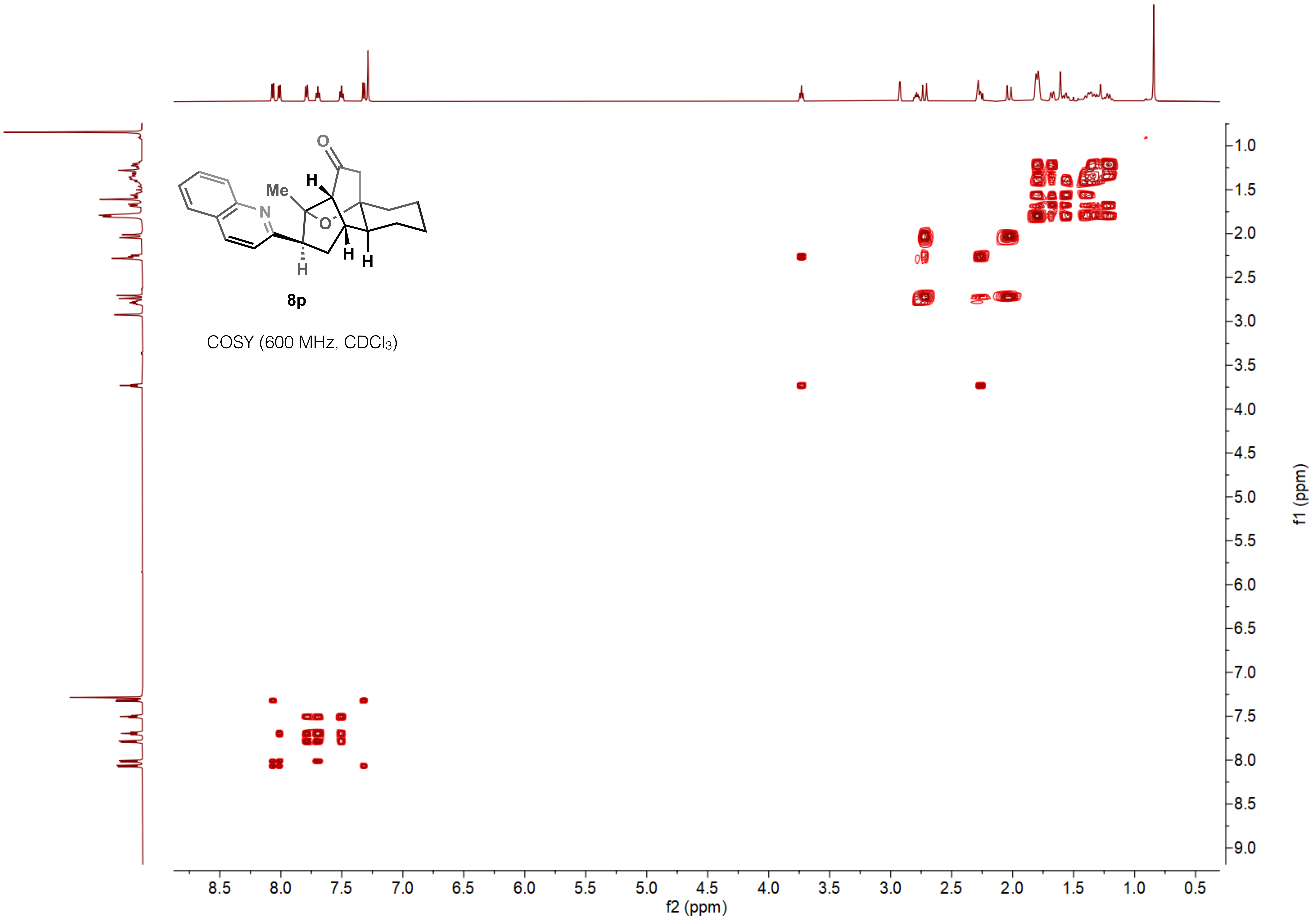
O
Me
N
O
H
H
H
H
8p
COSY (600 MHz, CDCl3)
f2 (ppm)
f1 (ppm)

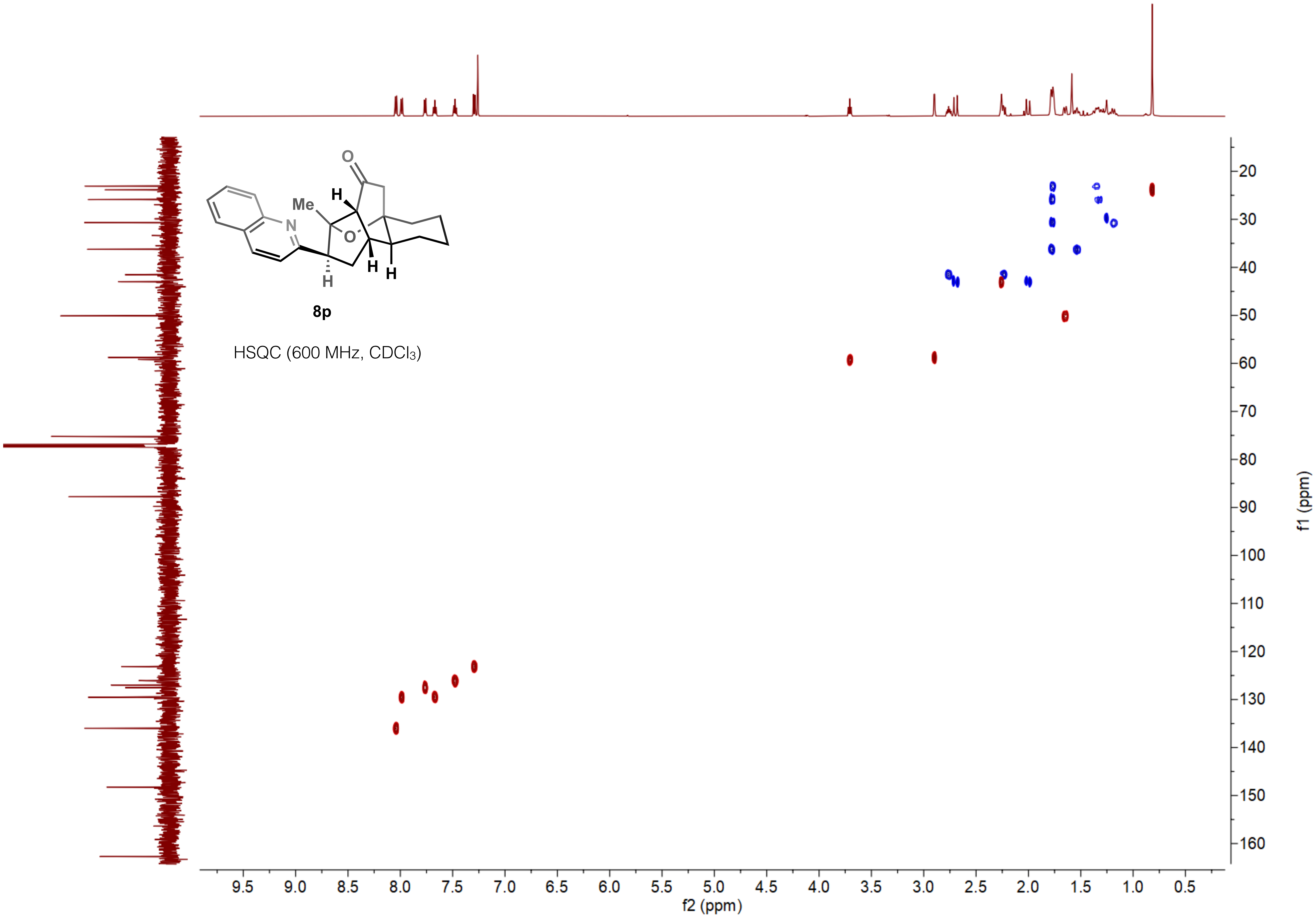

8p
HSQC (600 MHz, CDCl3)
f2 (ppm)
f1 (ppm)

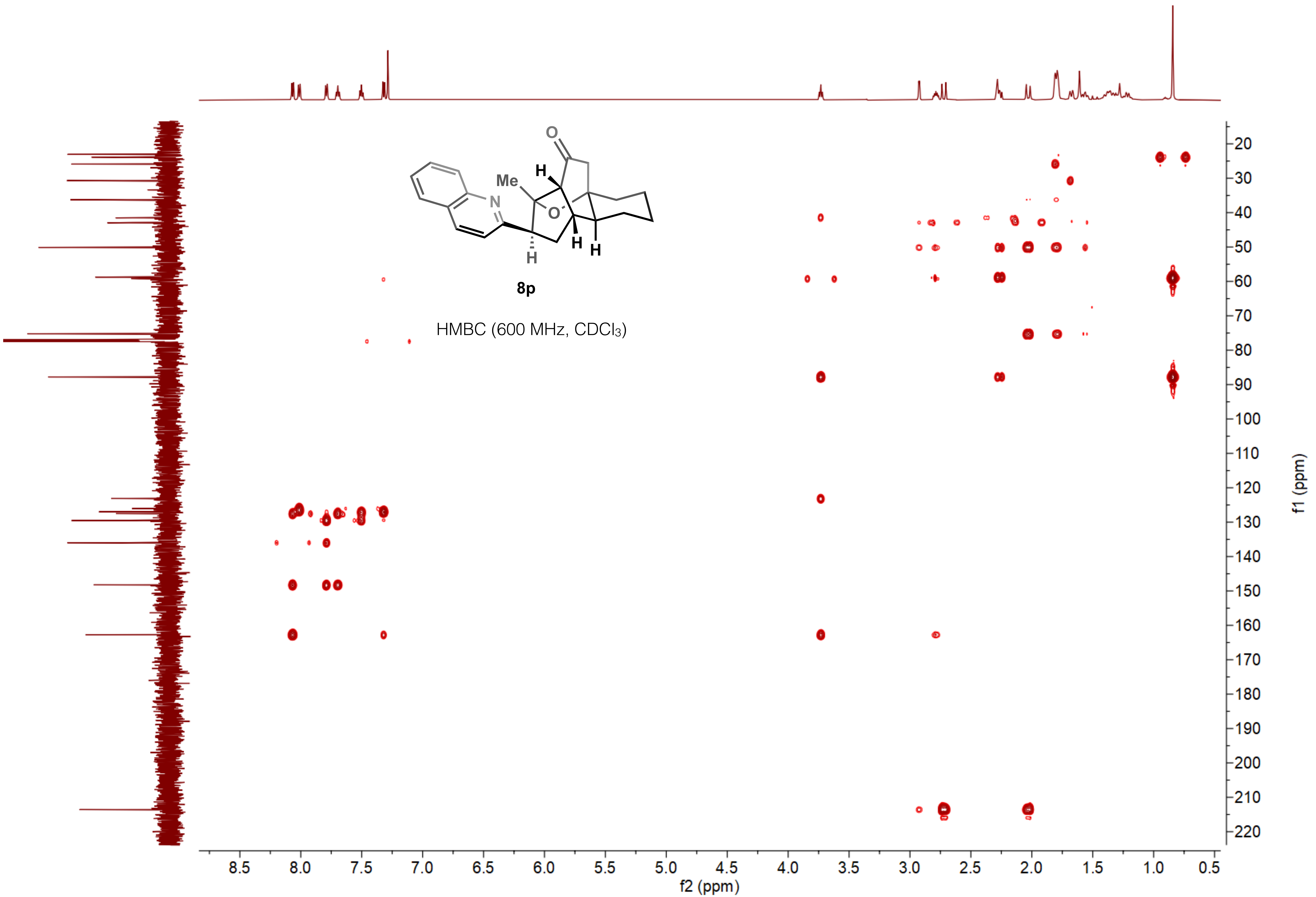

Me
H
N
O
O
H
H
H
8p
HMBC (600 MHz, CDCl3)
f2 (ppm)
f1 (ppm)

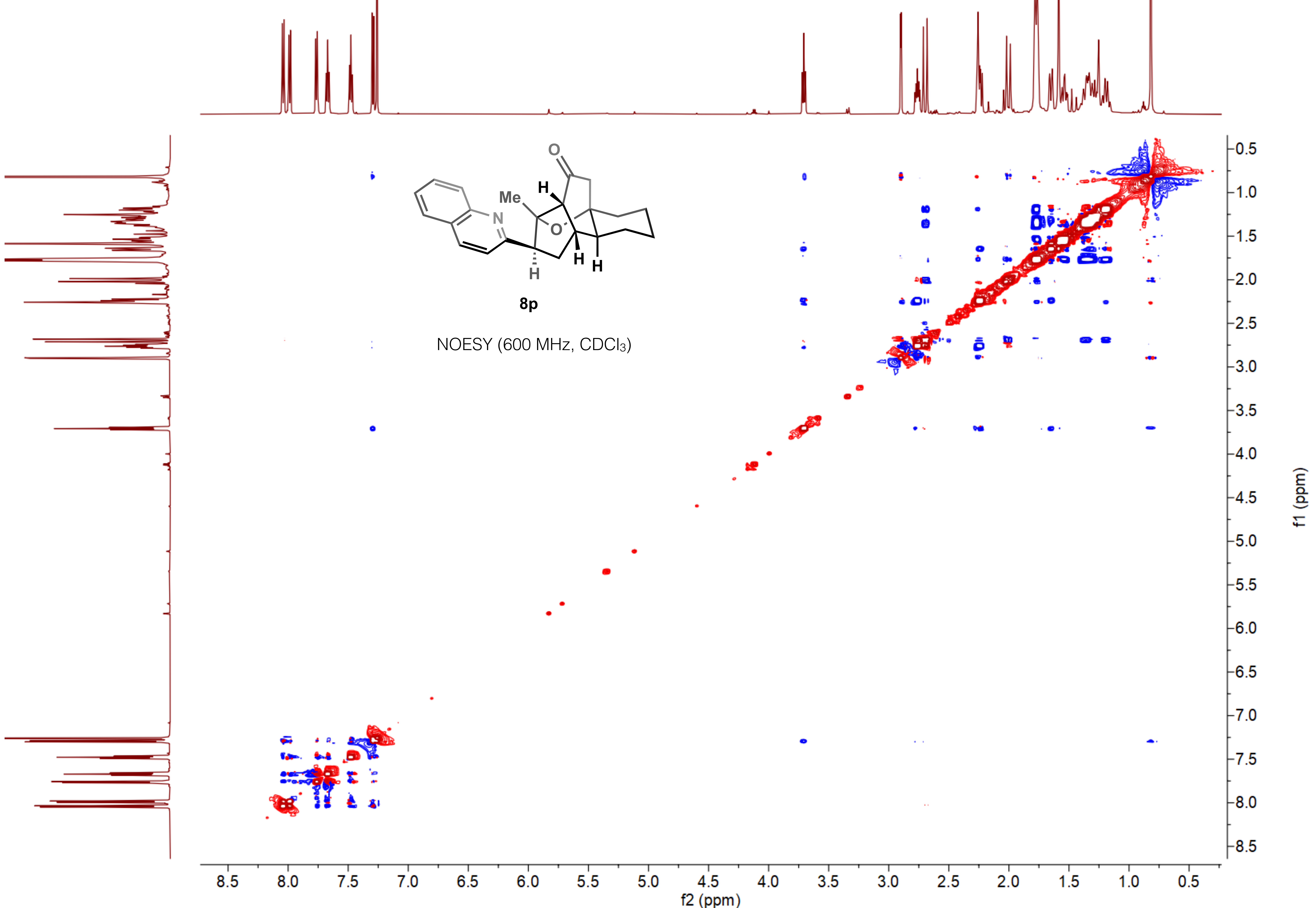

8p
NOESY (600 MHz, CDCl3)
f1 (ppm)
f2 (ppm)

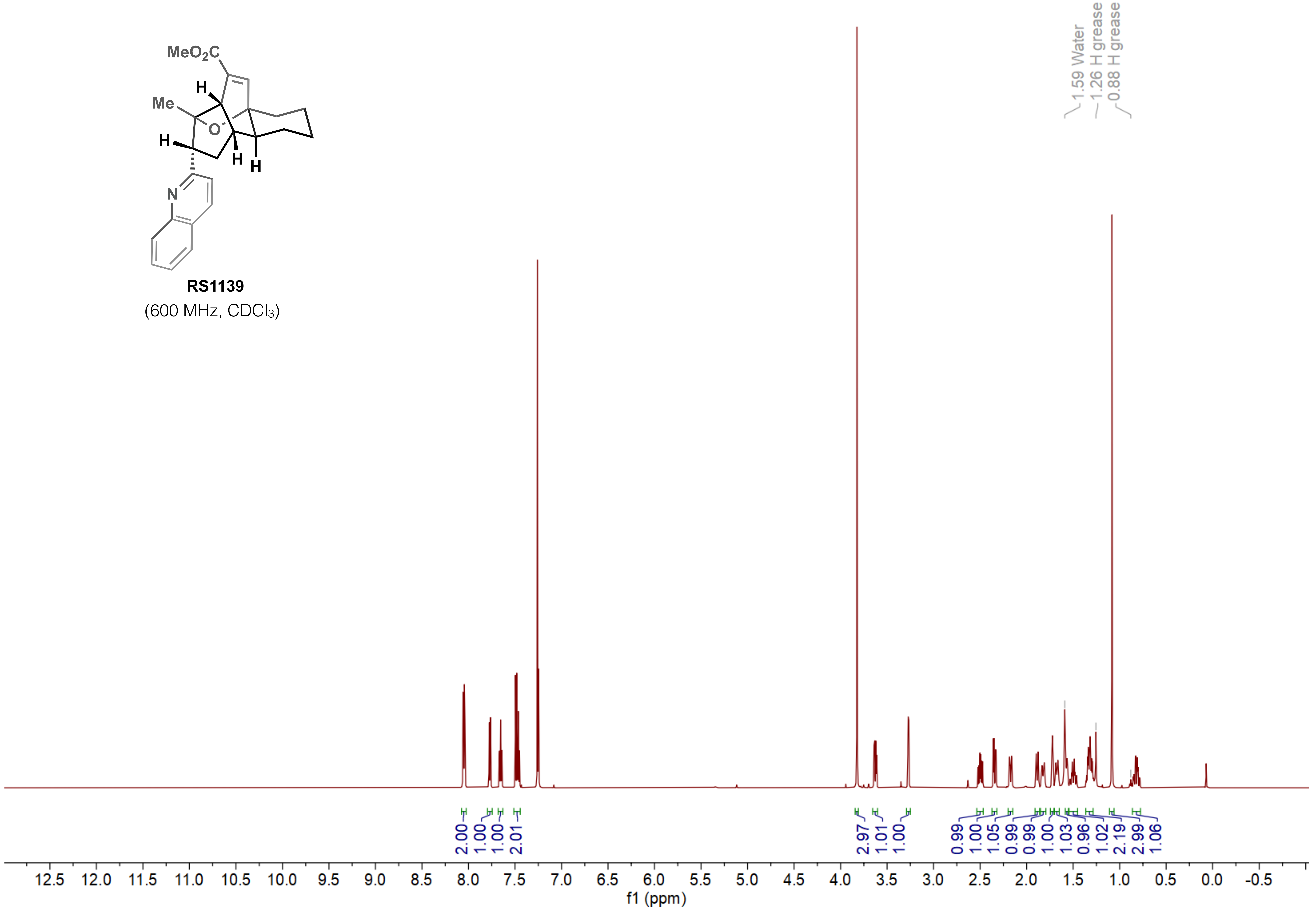

RS1139

(600 MHz, $CDCl_3$)

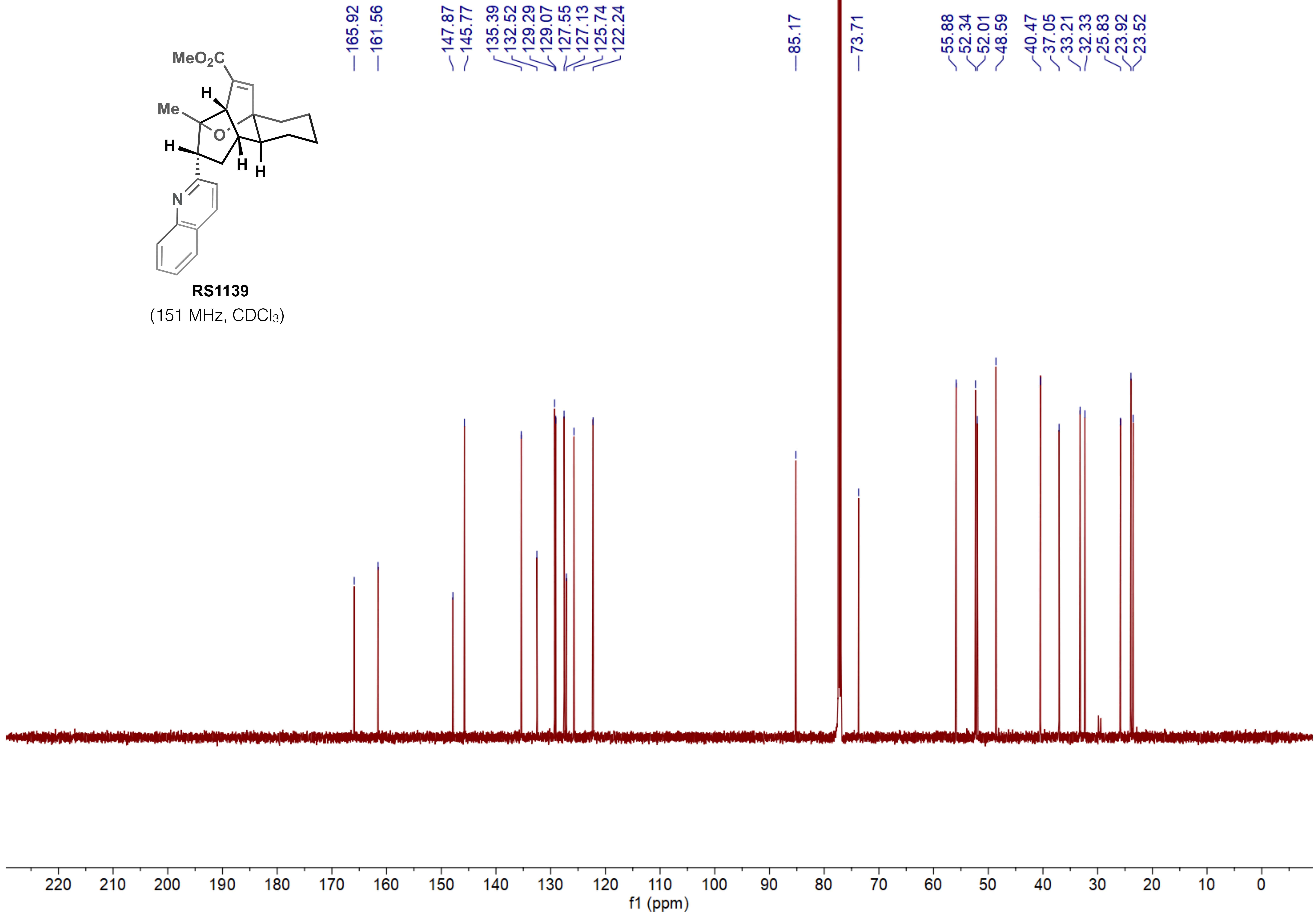

MeO2C
H
Me
O
H
H
H
N
RS1139
(151 MHz, CDCl3)
165.92
161.56
147.87
145.77
135.39
132.52
129.29
129.07
127.55
127.13
125.74
122.24
85.17
73.71
55.88
52.34
52.01
48.59
40.47
37.05
33.21
32.33
25.83
23.92
23.52
220
210
200
190
180
170
160
150
140
130
120
110
100
90
80
70
60
50
40
30
20
10
0
f1 (ppm)

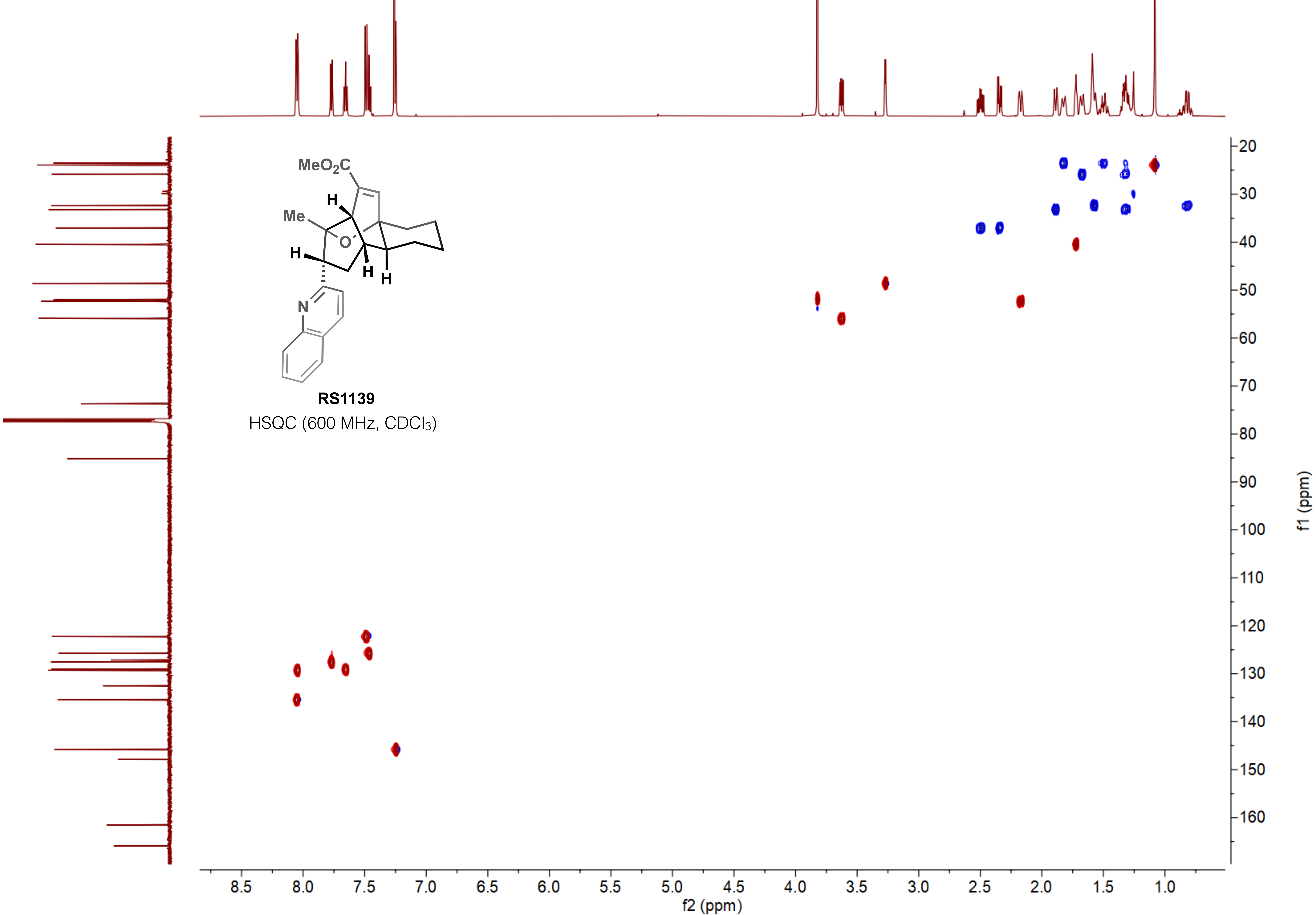
MeO2C
Me
H
O
H
H H
N
RS1139
HSQC (600 MHz, CDCl3)
f1 (ppm)
f2 (ppm)

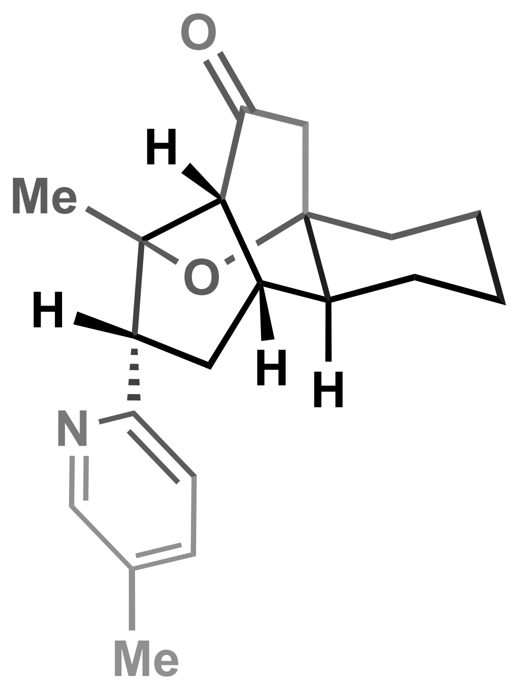

7q

(600 MHz, $CDCl_3$)

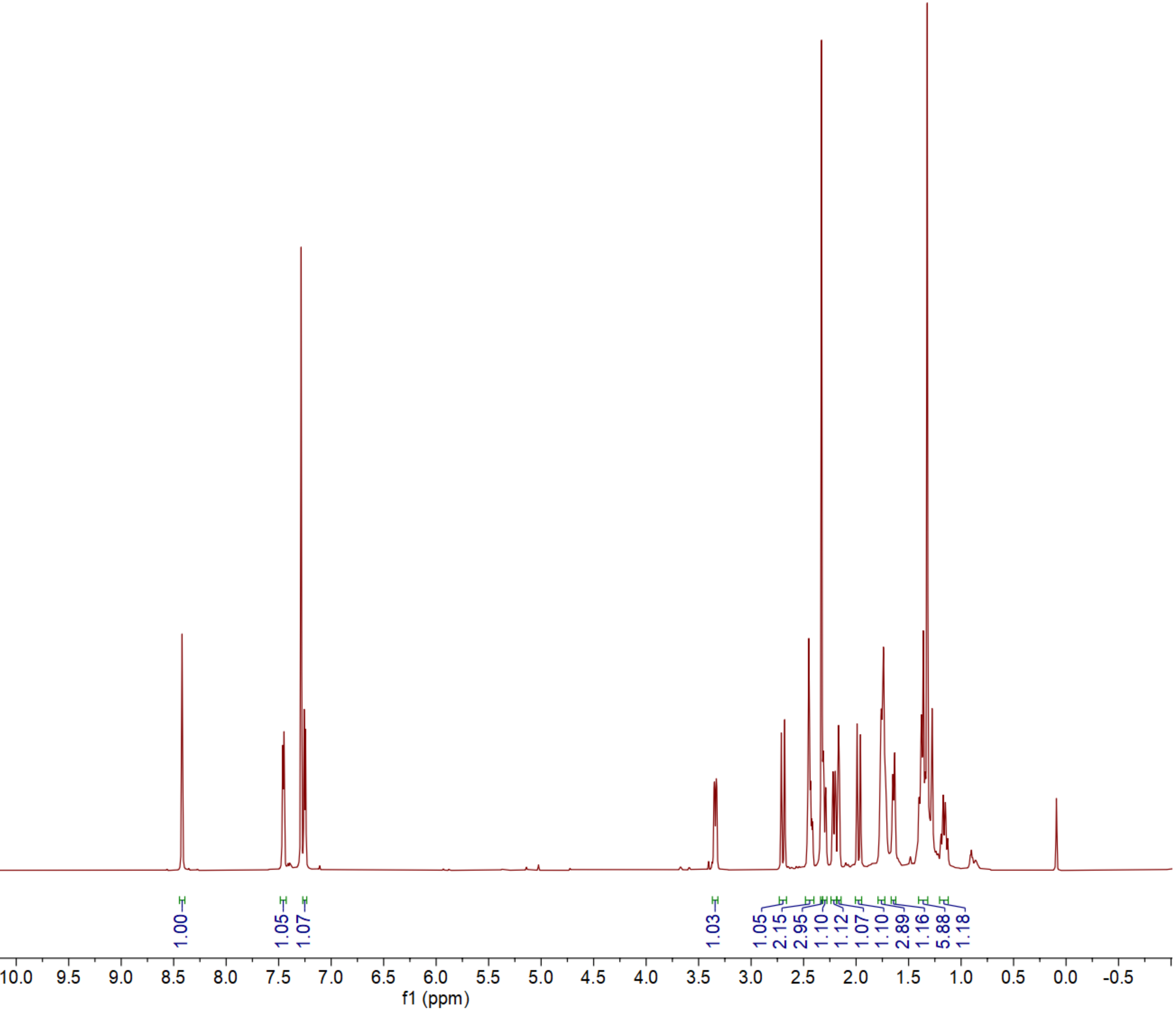

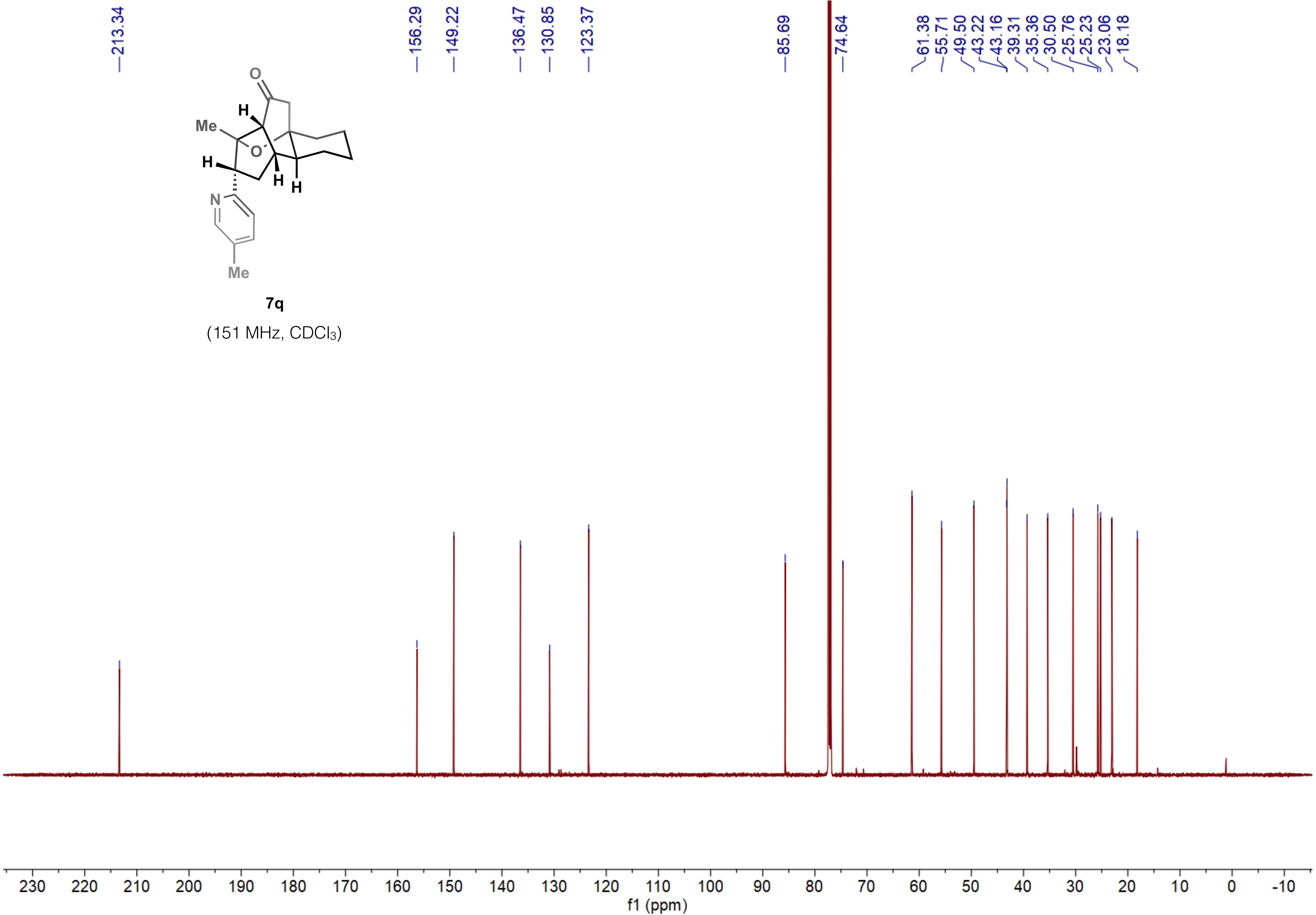

7q
(151 MHz, $CDCl_3$)
213.34
156.29
149.22
136.47
130.85
123.37
85.69
74.64
61.38
55.71
49.50
43.22
43.16
39.31
35.36
30.50
25.76
25.23
23.06
18.18
230
220
210
200
190
180
170
160
150
140
130
120
110
100
90
80
70
60
50
40
30
20
10
0
-10
f1 (ppm)

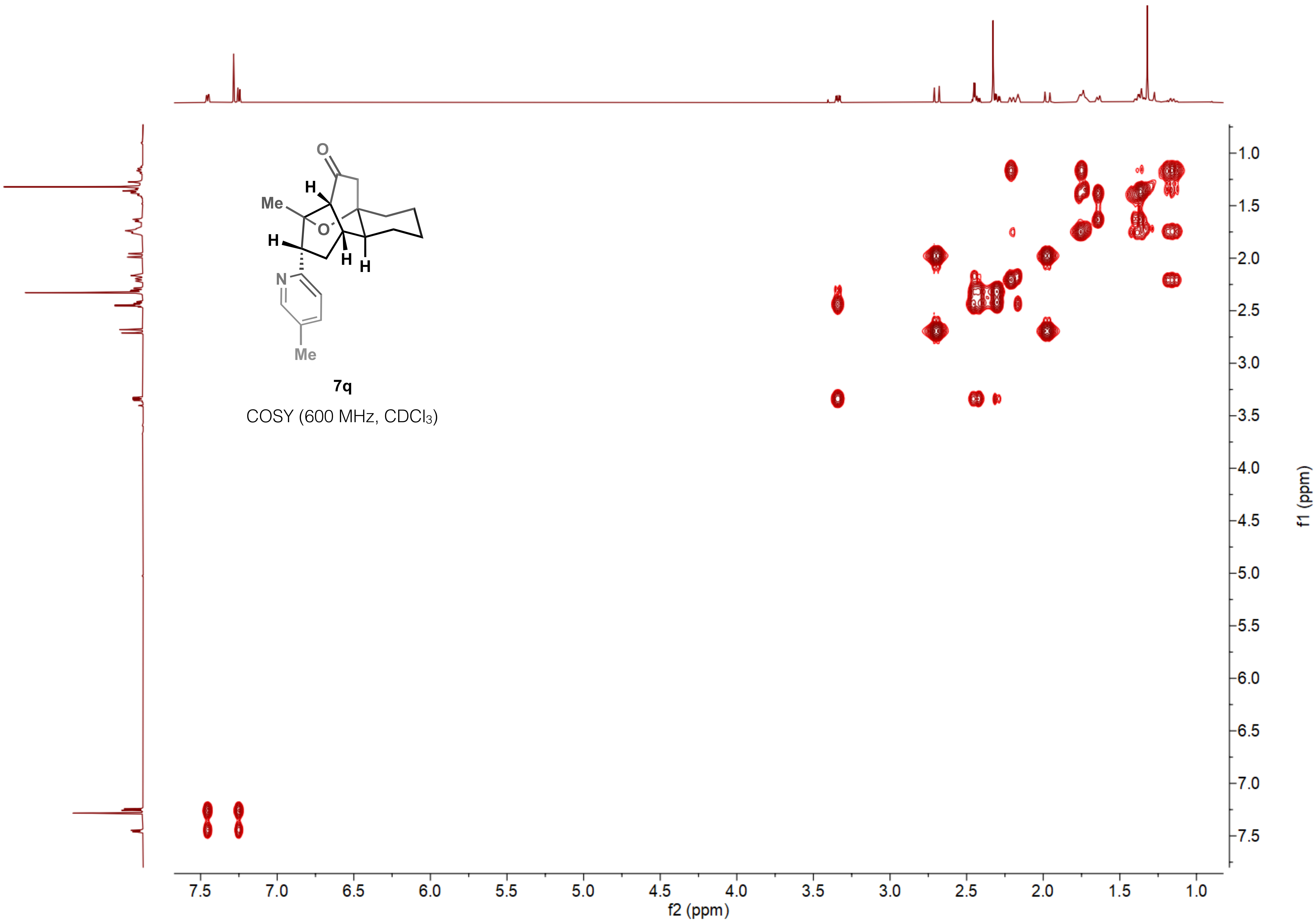

7q

COSY (600 MHz, $CDCl_3$)

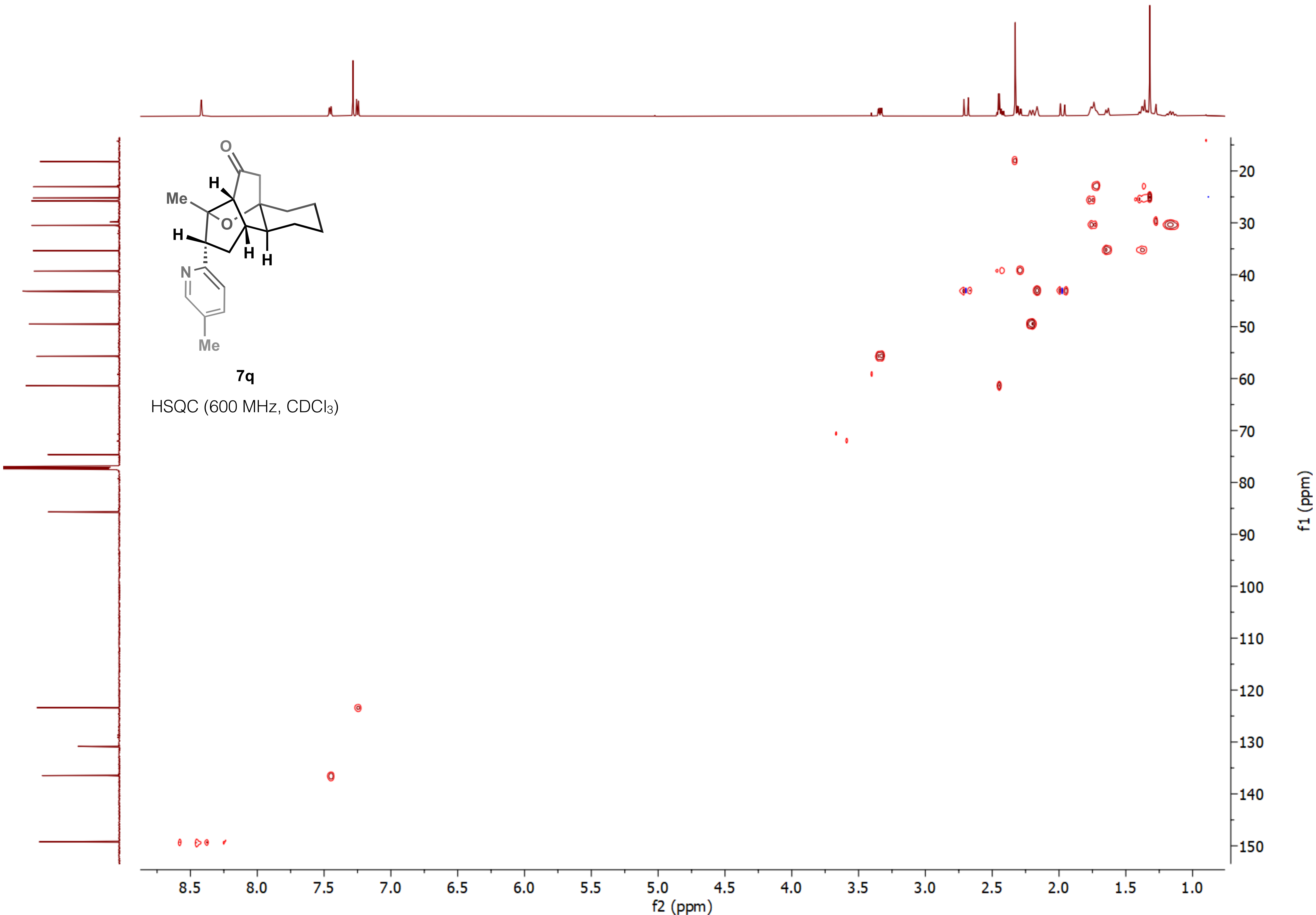

O
H
Me
O
H
H
H
N
Me
7q
HSQC (600 MHz, CDCl3)
f2 (ppm)
f1 (ppm)

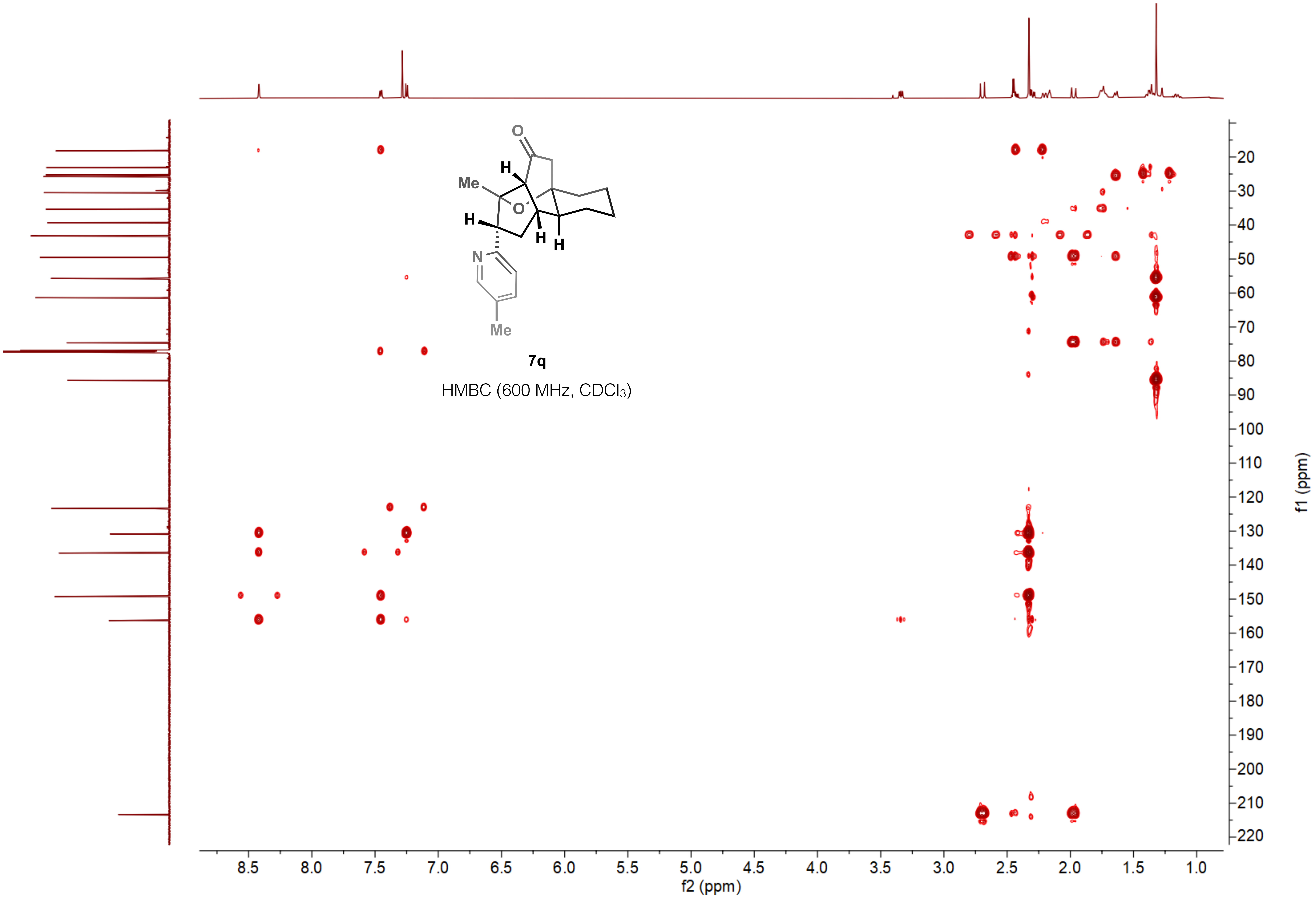

7q

HMBC (600 MHz, $CDCl_3$)

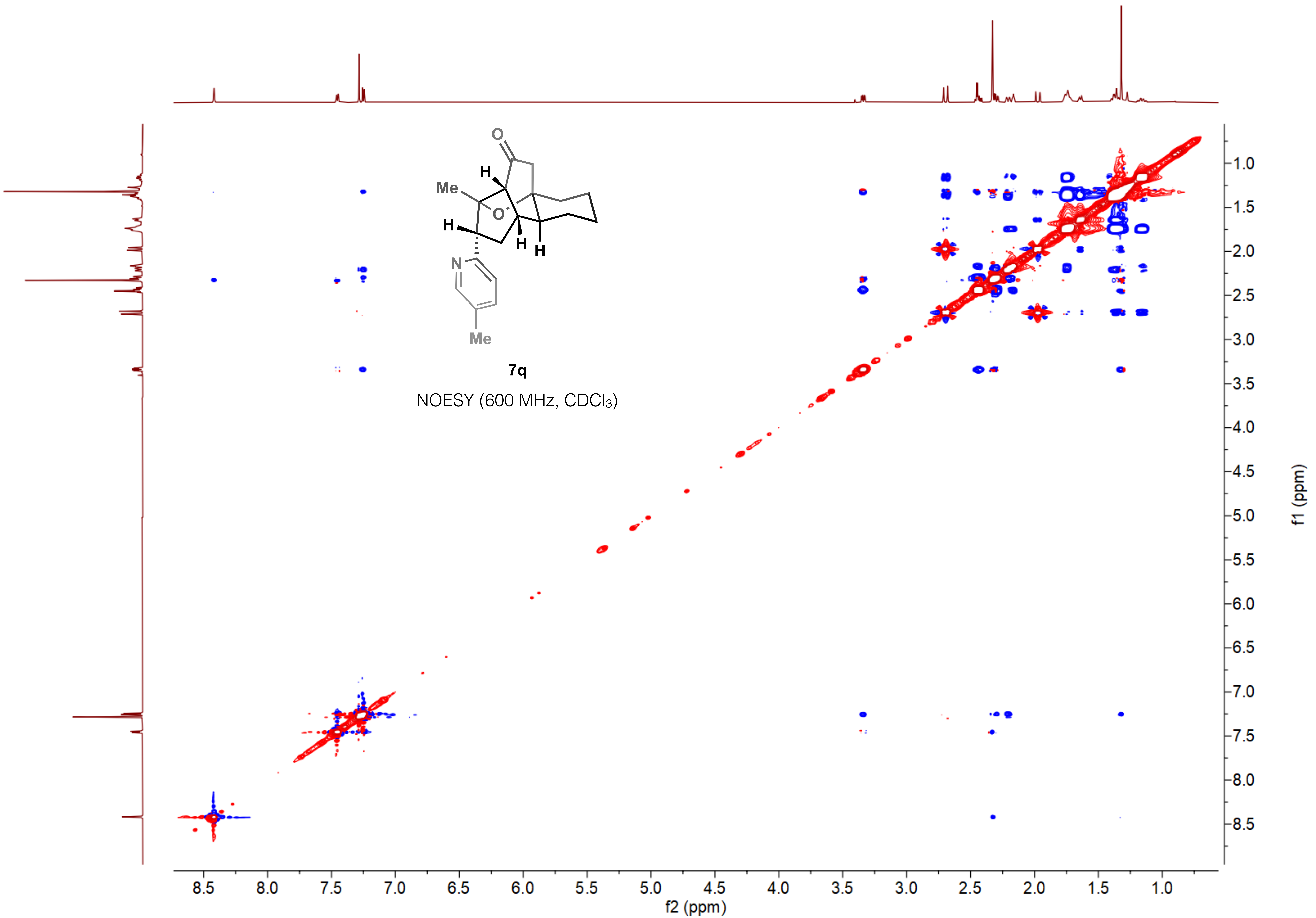

O
H
Me
O
H
H
H
N
Me
7q
NOESY (600 MHz, CDCl3)
f2 (ppm)
f1 (ppm)

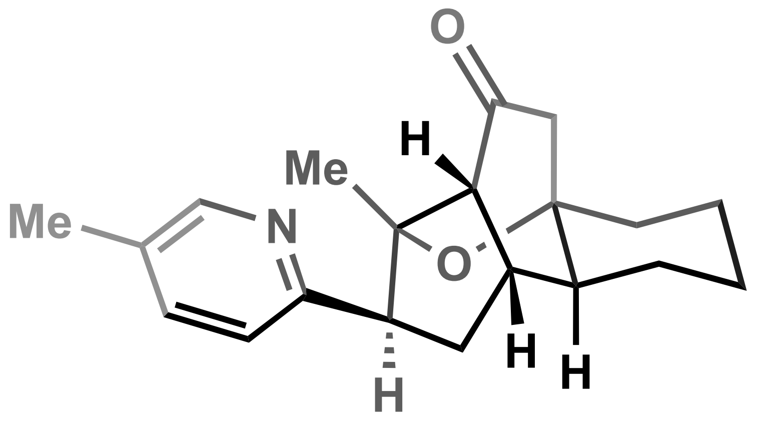

8q

(600 MHz, $CDCl_3$)

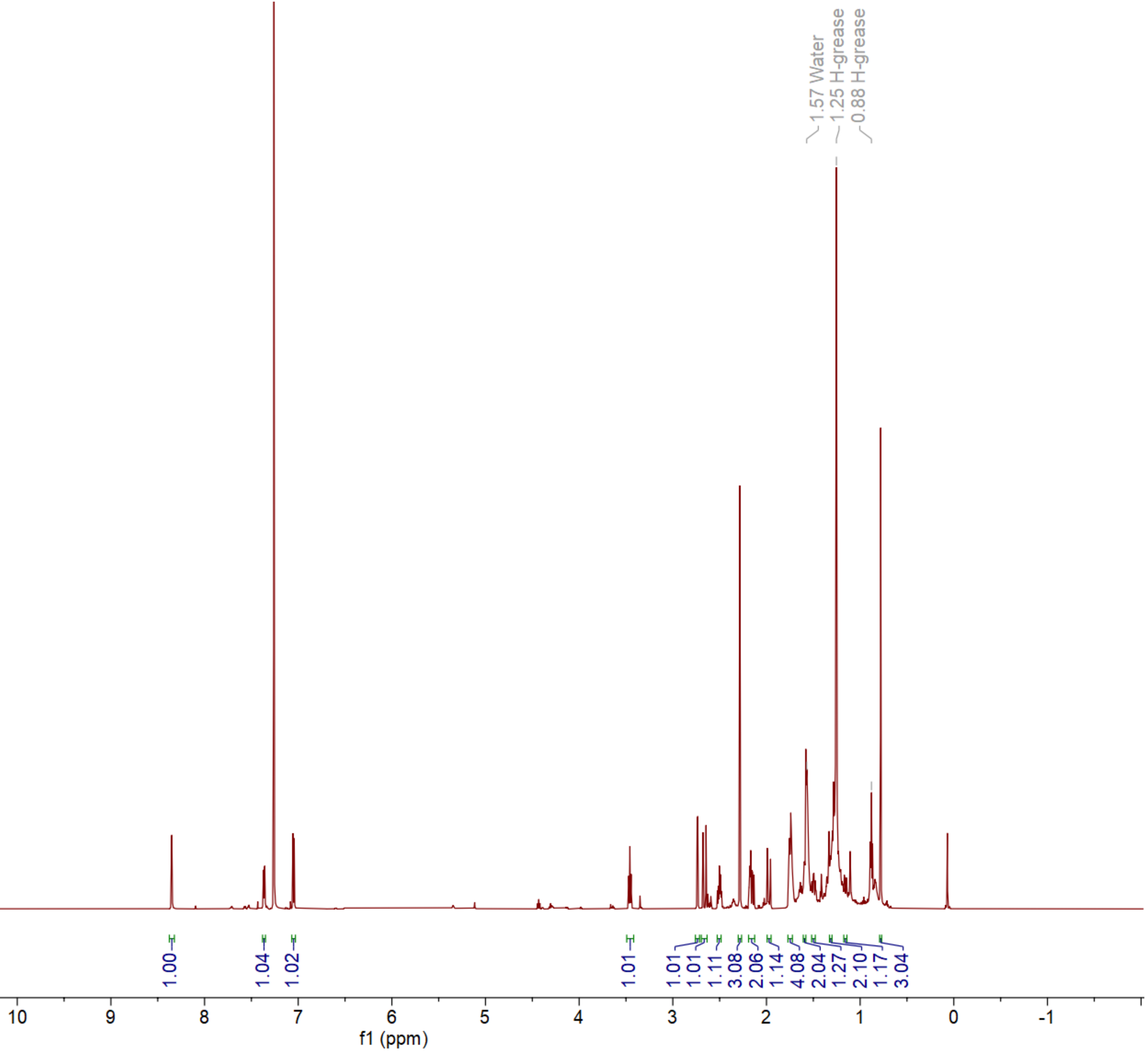

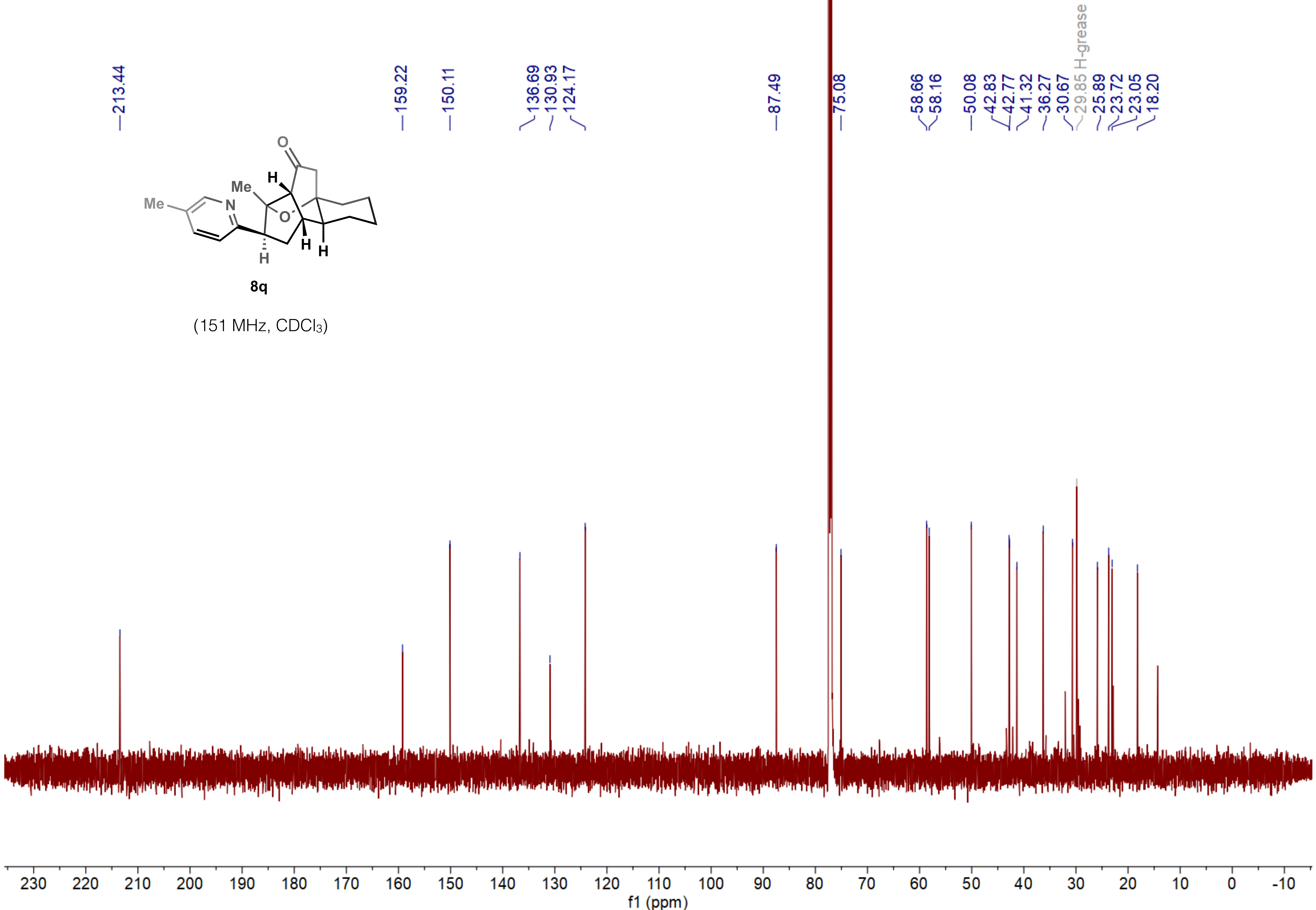

8q

(151 MHz, $CDCl_3$)

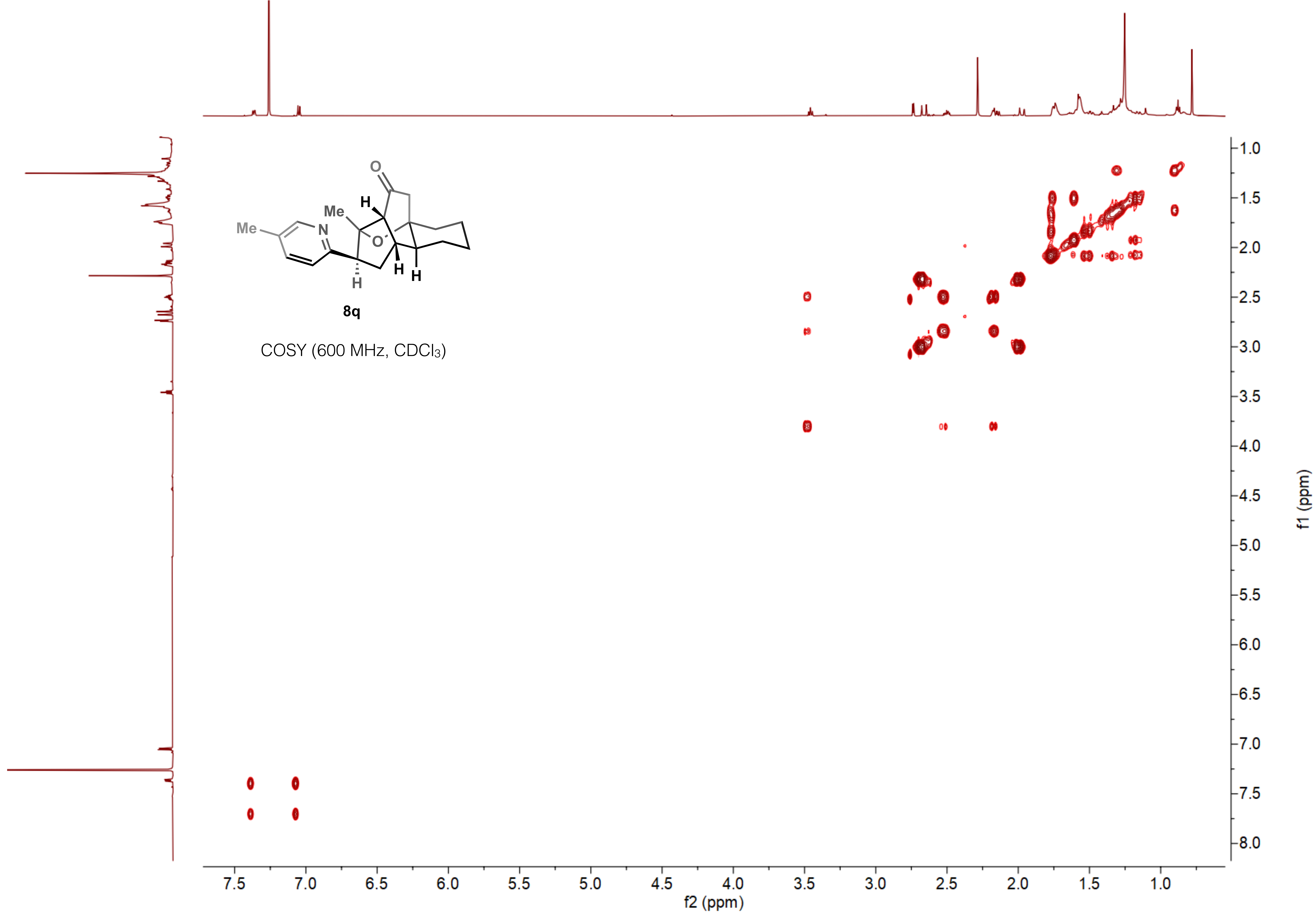

O
Me
H
Me
N
O
H
H
H
8q
COSY (600 MHz, CDCl3)
f1 (ppm)
f2 (ppm)

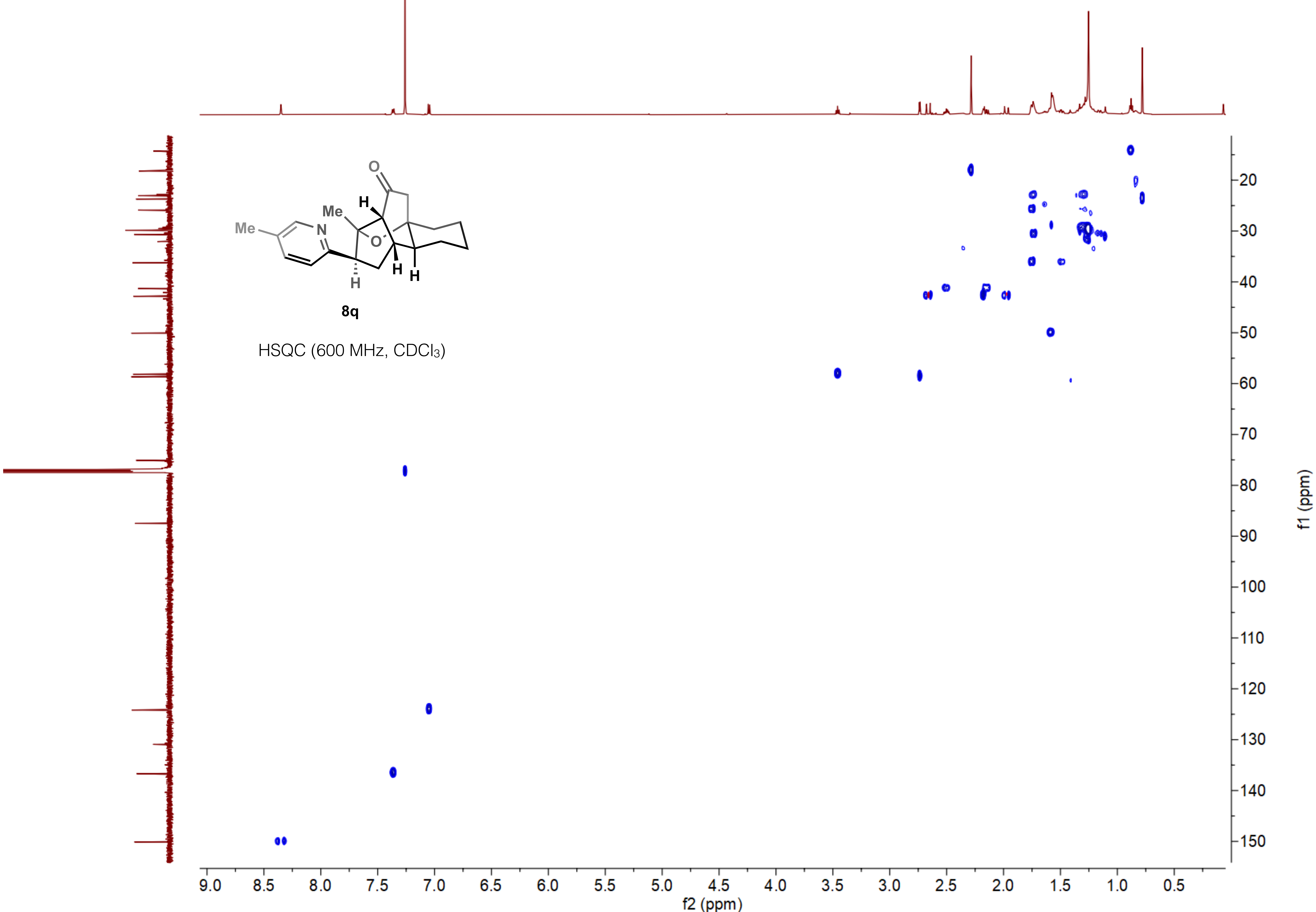

8q

HSQC (600 MHz, $CDCl_3$)

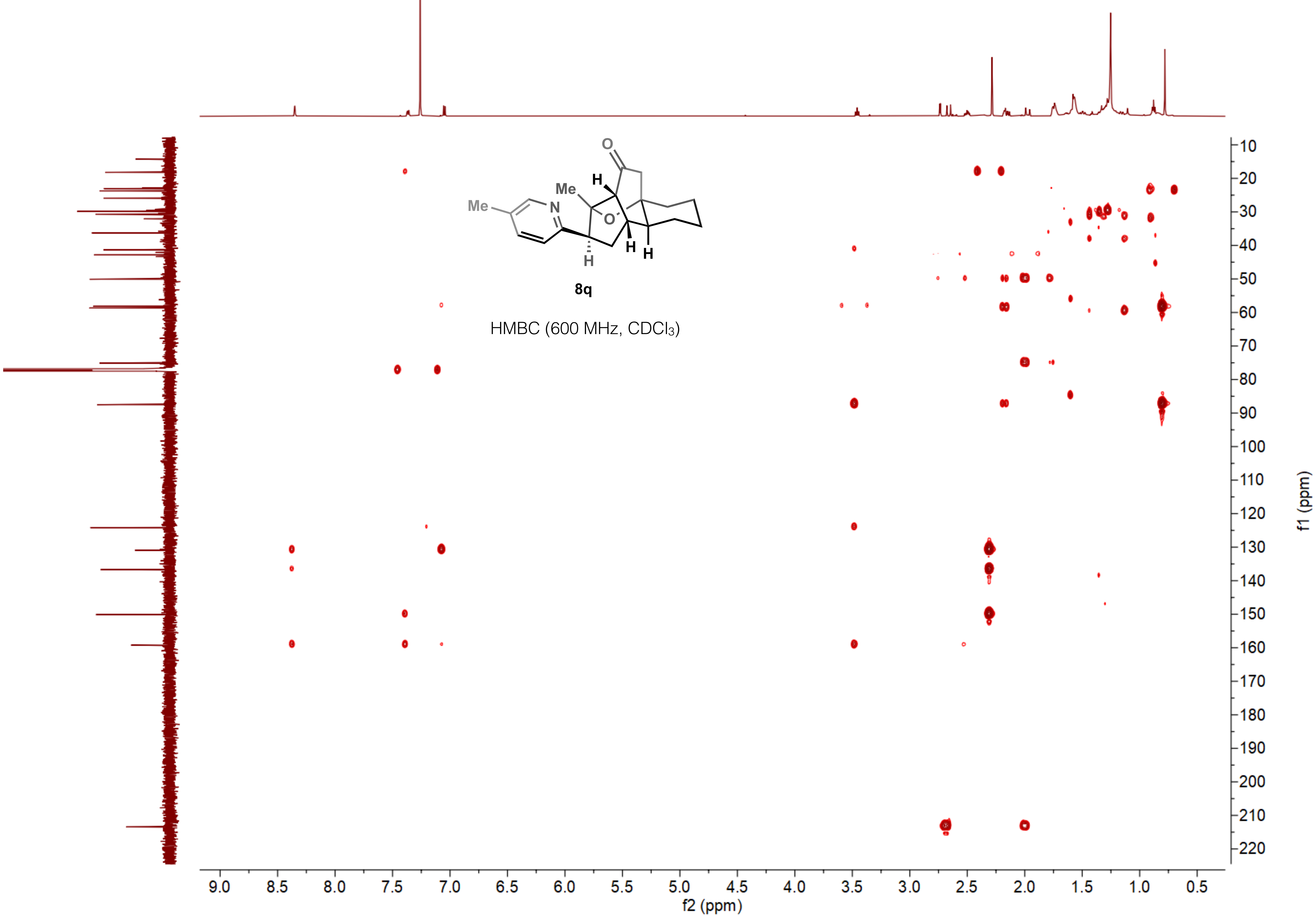
Me
N
Me
H
O
O
H
H
H
8q
HMBC (600 MHz, CDCl3)
f1 (ppm)
f2 (ppm)

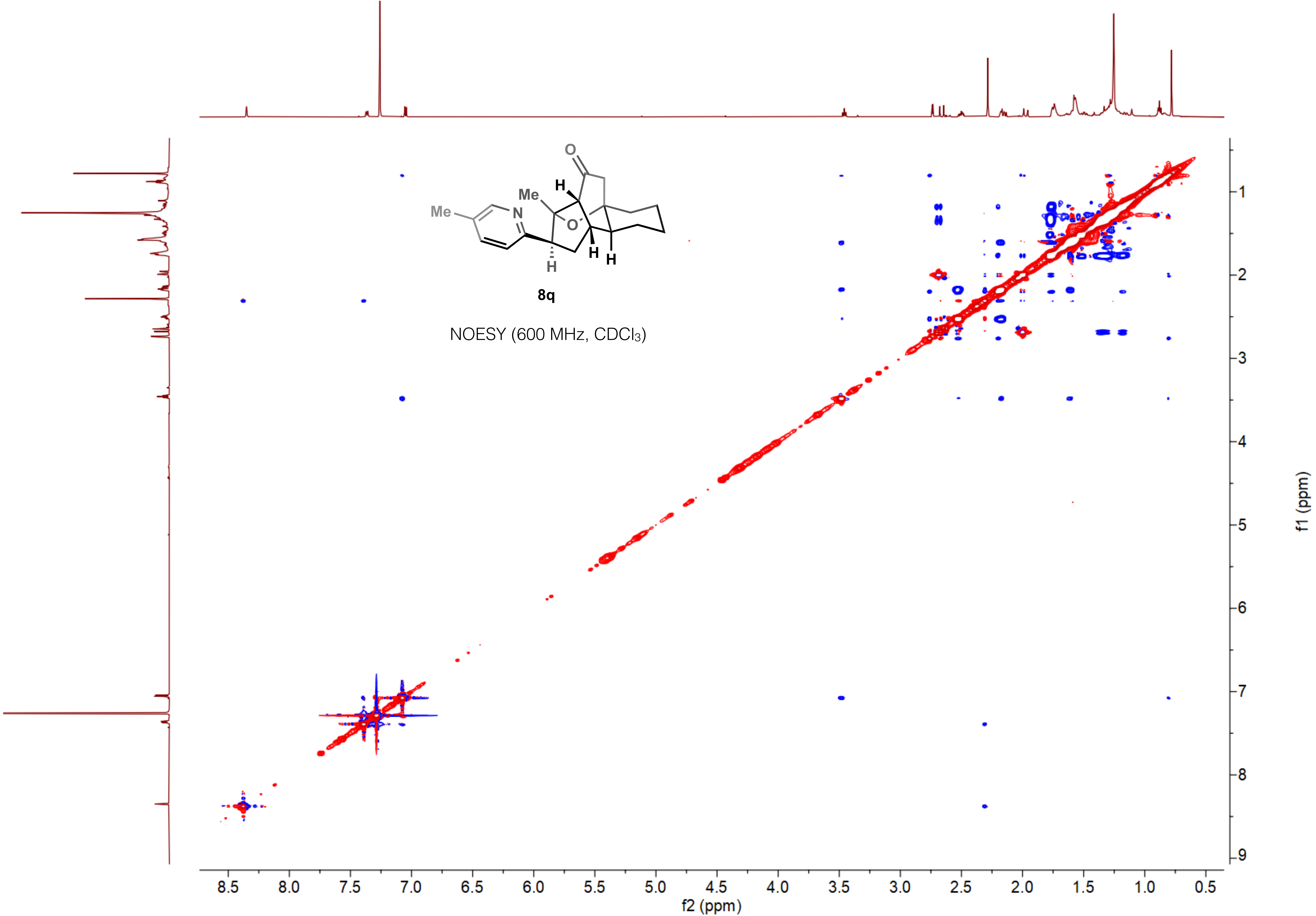

O
H
Me
Me
N
O
H
H
H
8q
NOESY (600 MHz, CDCl3)
f2 (ppm)
f1 (ppm)

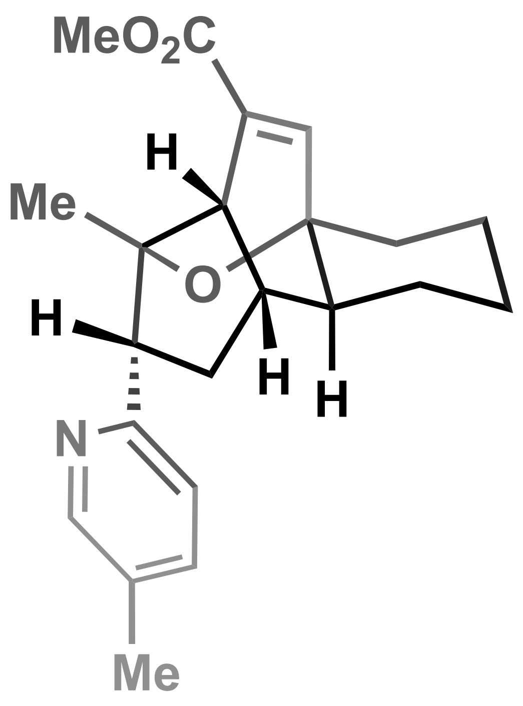

RS1128

(600 MHz, $CDCl_3$)

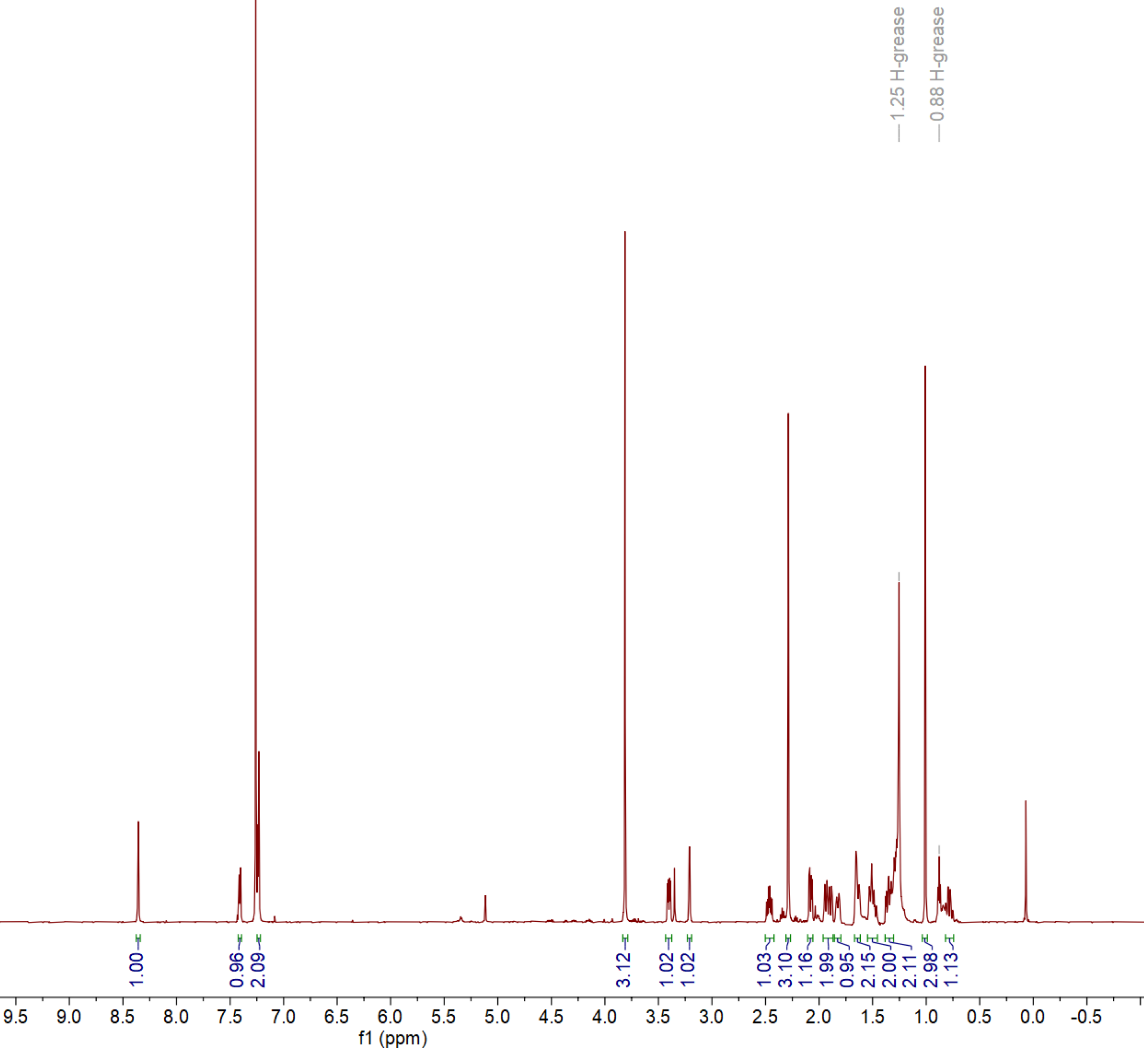

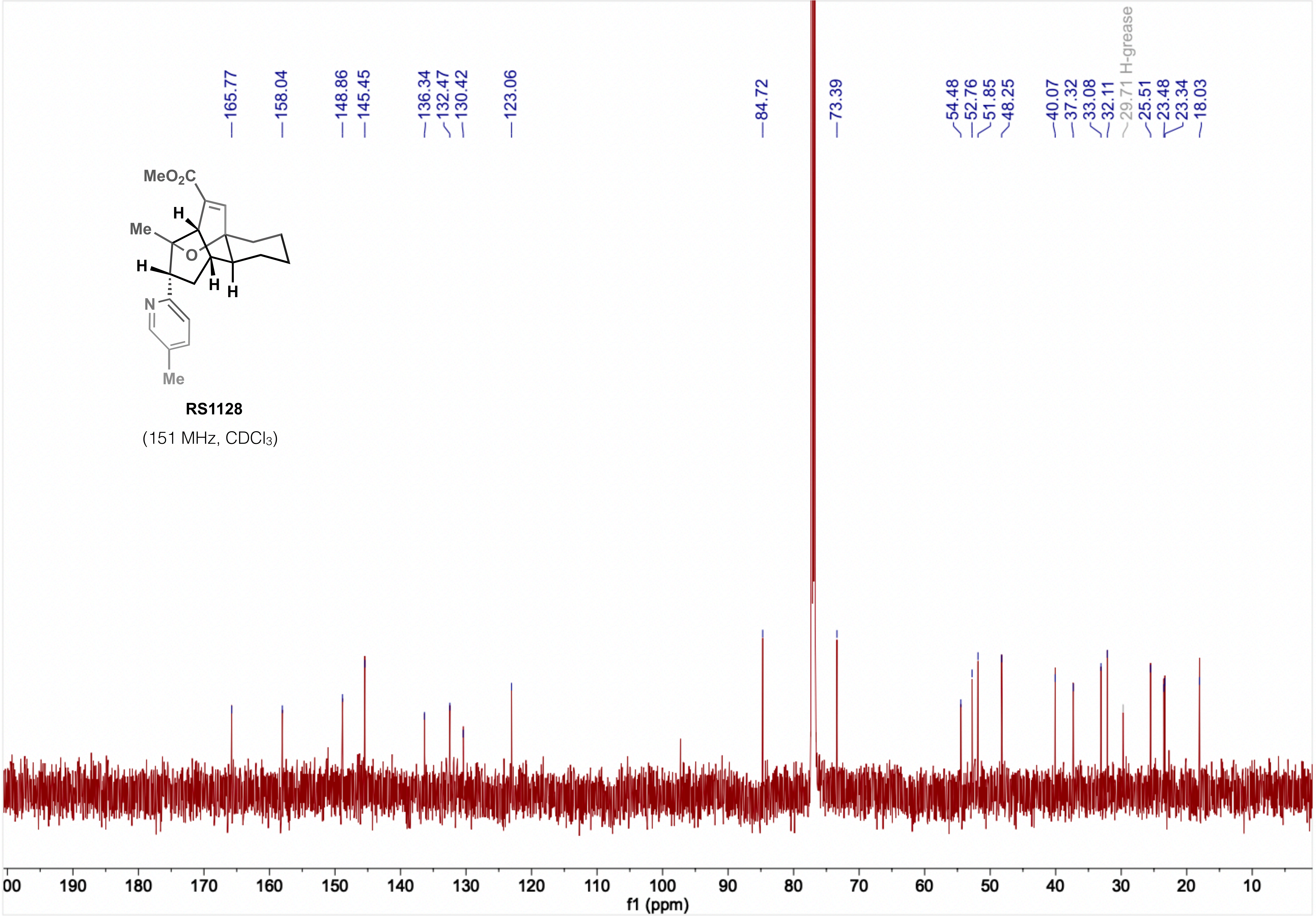
165.77
158.04
148.86
145.45
136.34
132.47
130.42
123.06
84.72
73.39
54.48
52.76
51.85
48.25
40.07
37.32
33.08
32.11
29.71 H-grease
25.51
23.48
23.34
18.03
MeO2C
Me
H
O
H
H
H
N
Me
RS1128
(151 MHz, CDCl3)
00
190
180
170
160
150
140
130
120
110
100
90
80
70
60
50
40
30
20
10
f1 (ppm)

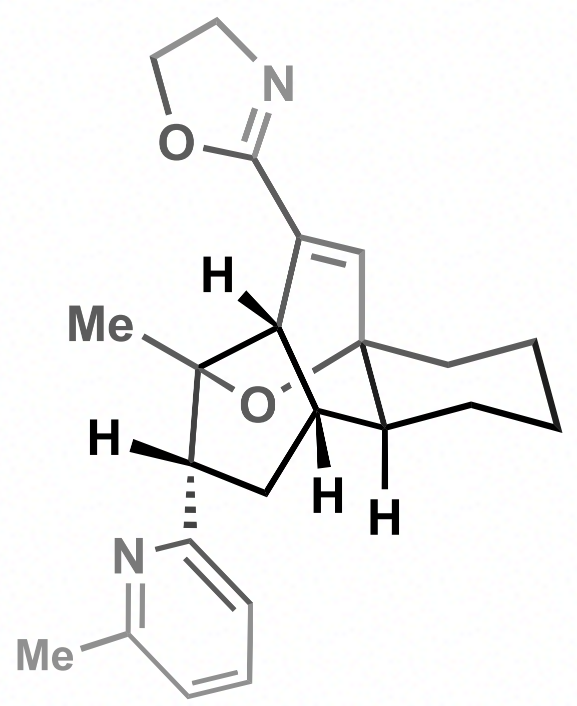

RS1125

(600 MHz, $CDCl_3$)

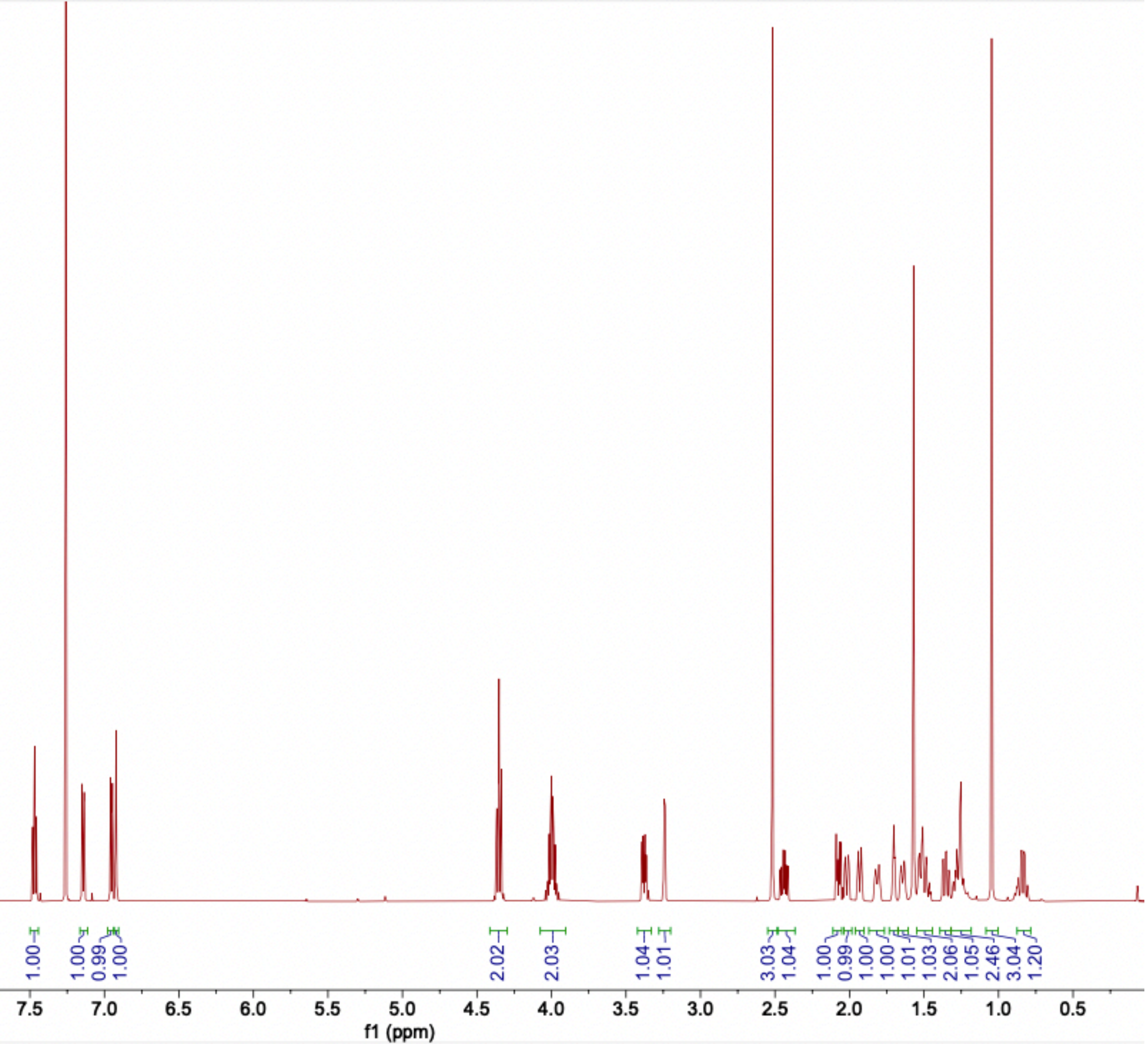

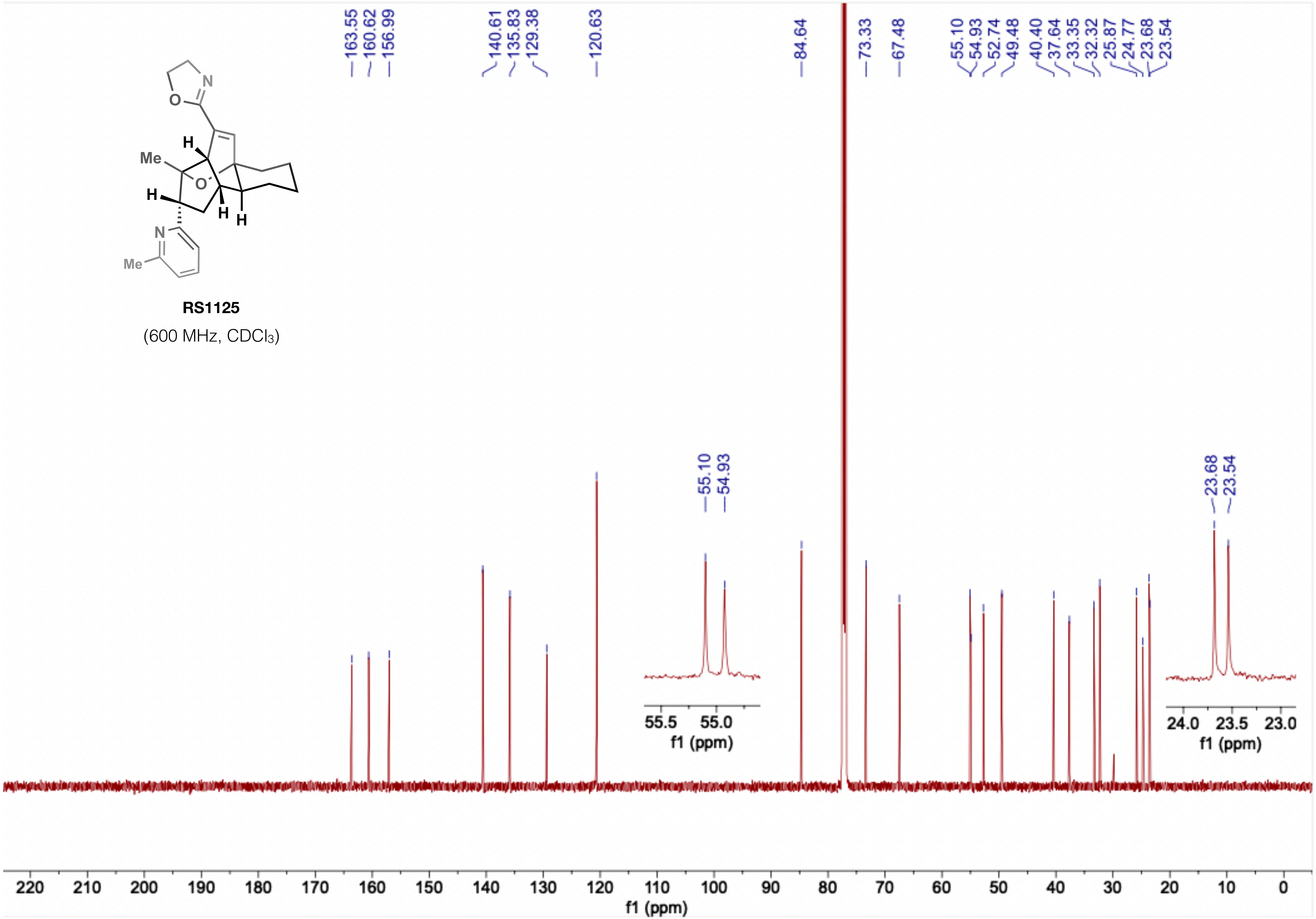

RS1125
(600 MHz, CDCl3)
163.55
160.62
156.99
140.61
135.83
129.38
120.63
84.64
73.33
67.48
55.10
54.93
52.74
49.48
40.40
37.64
33.35
32.32
25.87
24.77
23.68
23.54
f1 (ppm)

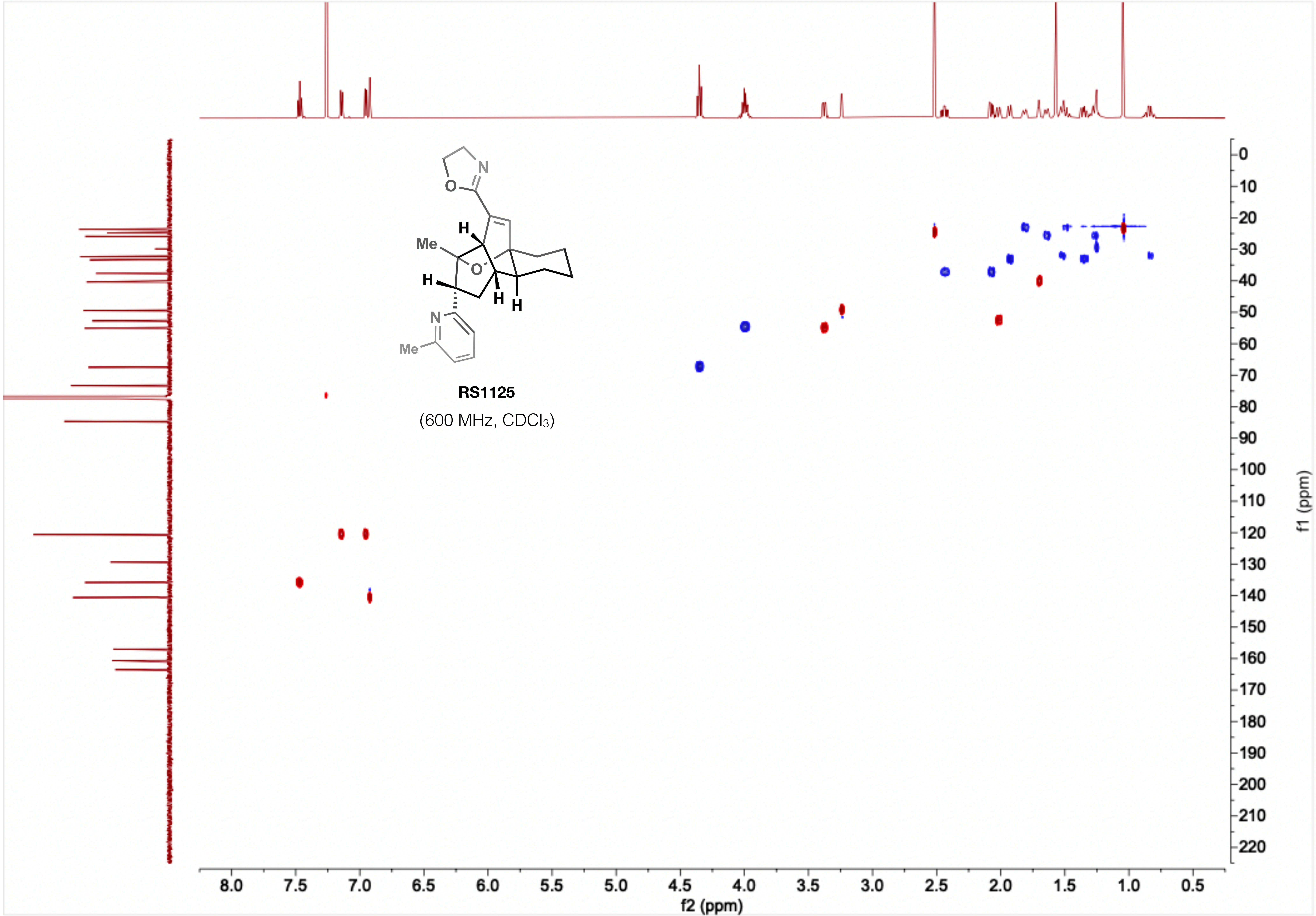

N
O
H
Me
O
H
H
H
N
Me
RS1125
(600 MHz, CDCl3)
f1 (ppm)
0
10
20
30
40
50
60
70
80
90
100
110
120
130
140
150
160
170
180
190
200
210
220
8.0
7.5
7.0
6.5
6.0
5.5
5.0
4.5
4.0
3.5
3.0
2.5
2.0
1.5
1.0
0.5
f2 (ppm)

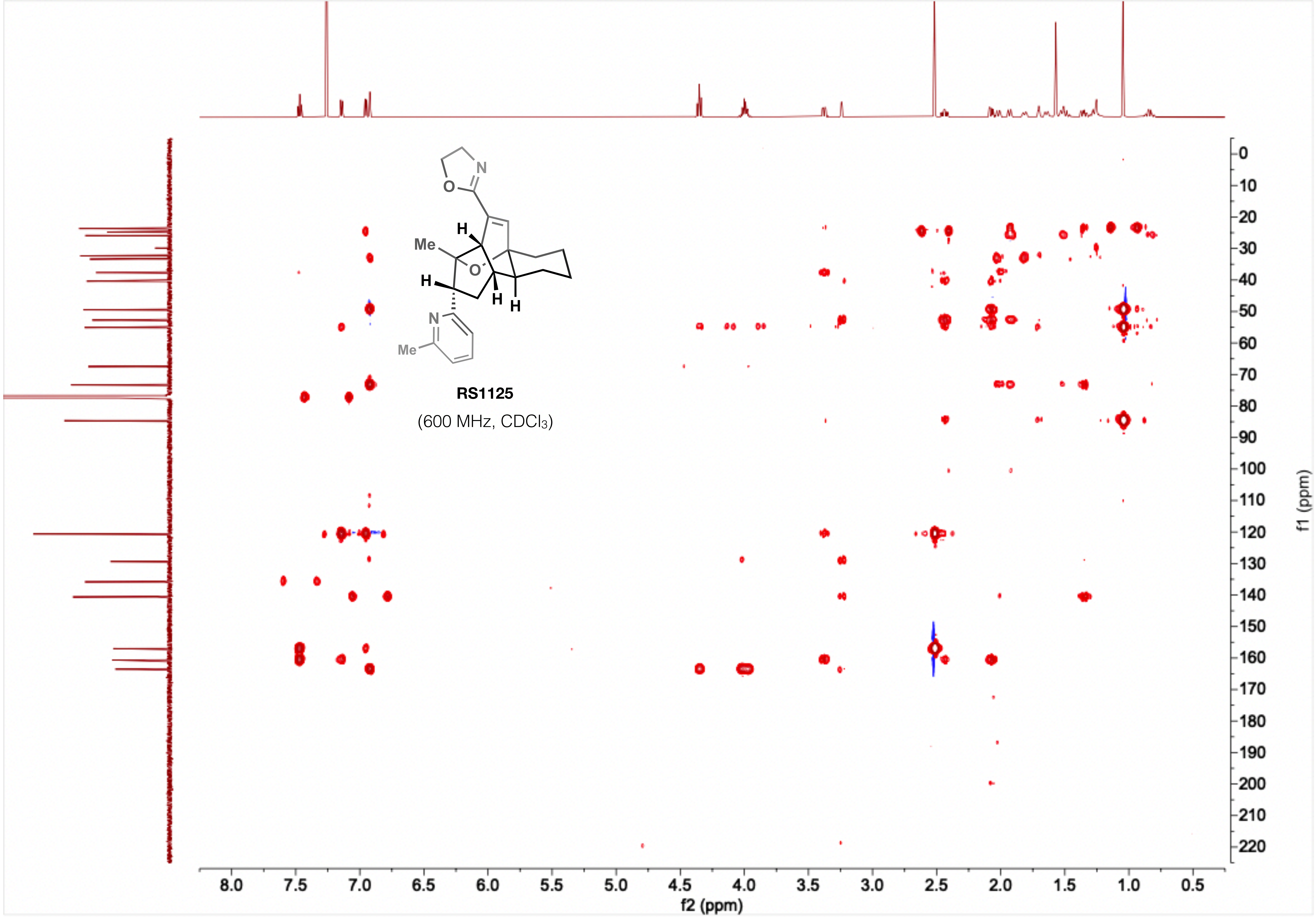

Me
O
H
H
H
H
N
O
N
Me
RS1125
(600 MHz, CDCl3)
f2 (ppm)
f1 (ppm)

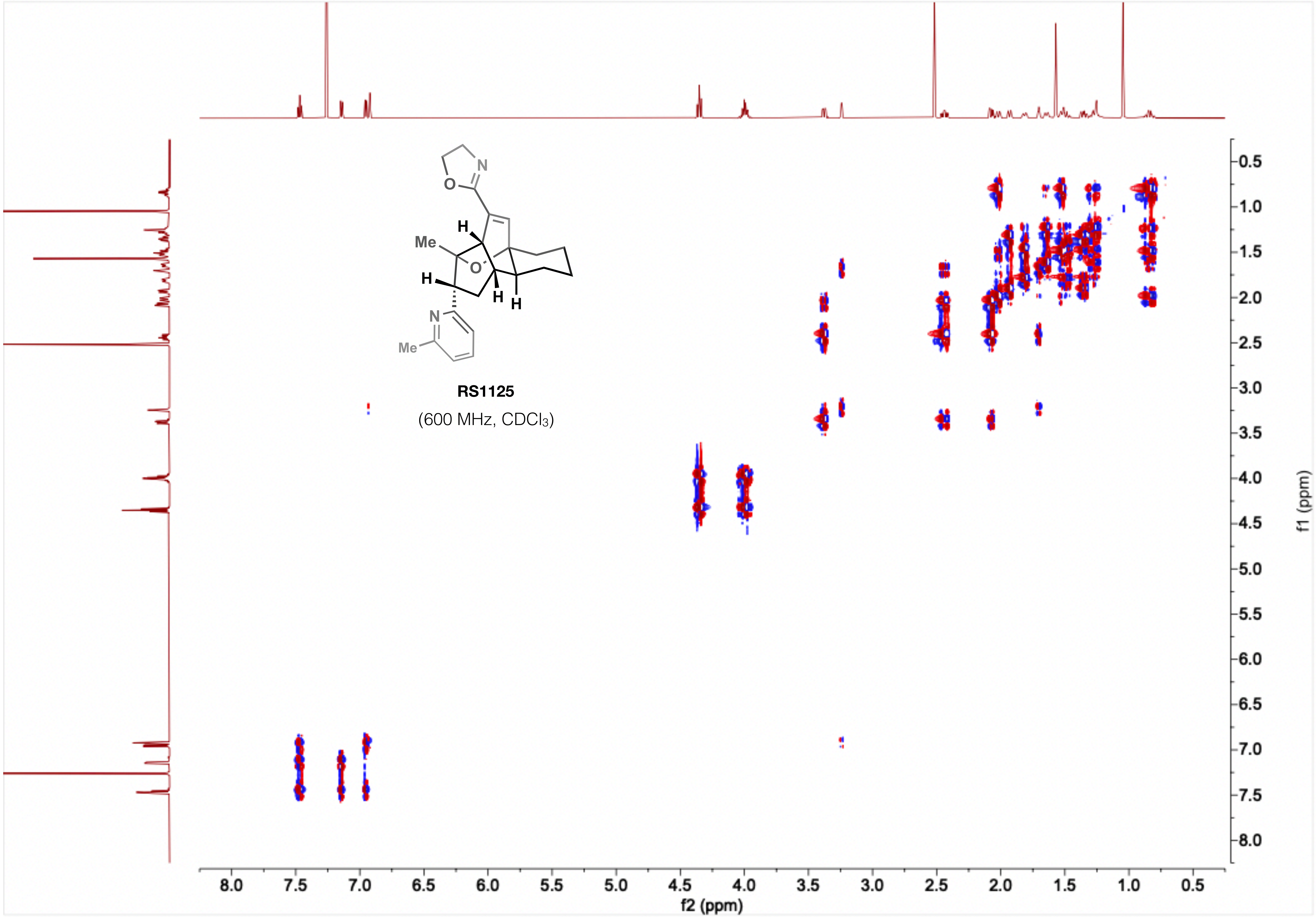

RS1125
(600 MHz, $CDCl_3$)
Me
O
N
H
Me
f2 (ppm)
f1 (ppm)

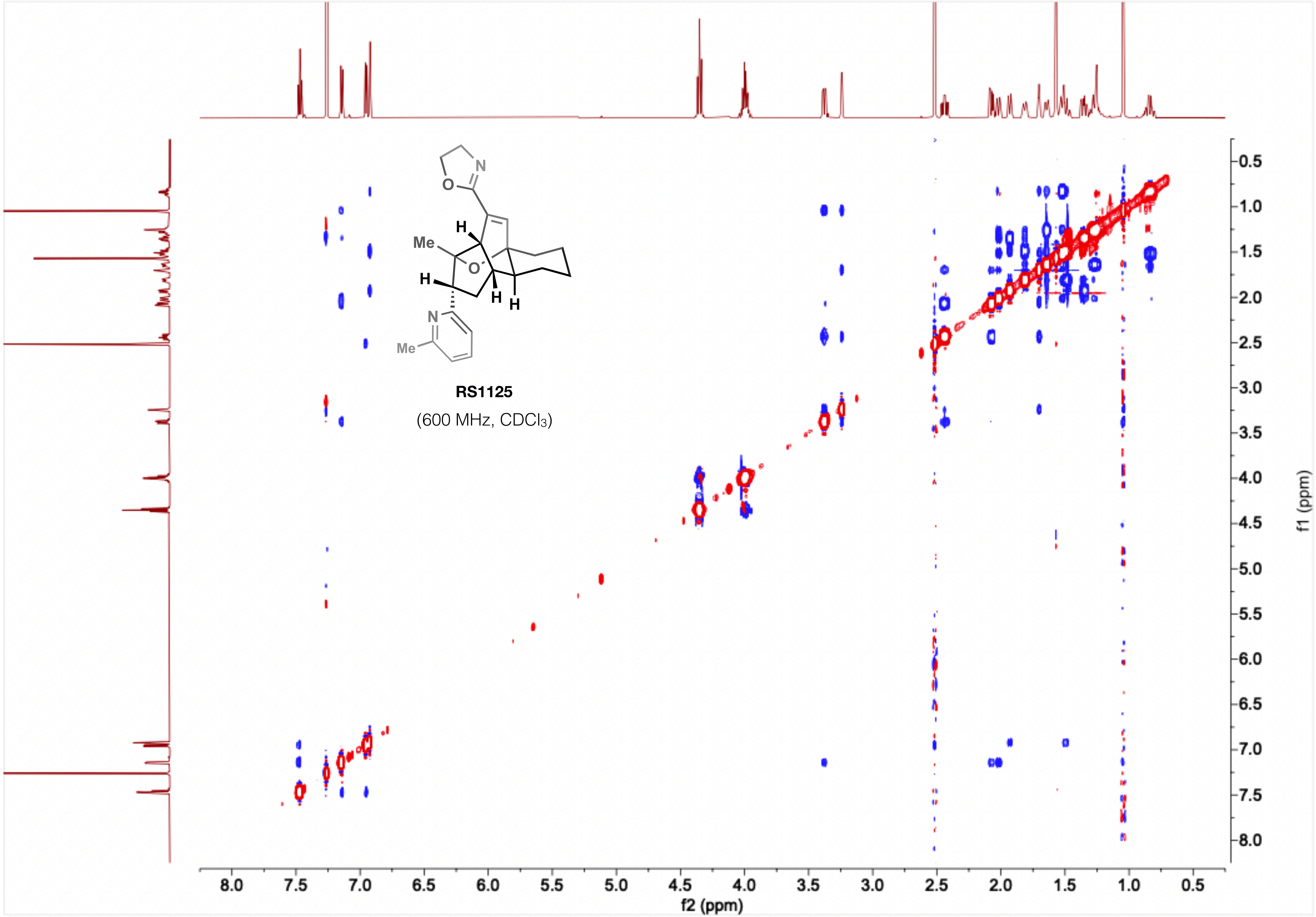

RS1125
(600 MHz, CDCl3)
f2 (ppm)
f1 (ppm)

X-ray Crystal Structure Reports

Experimental Summary

The single crystal X-ray diffraction studies were carried out on a Bruker D8 Venture diffractometer equipped with Mo K_{α} radiation ($\lambda = 0.71073$). Crystals of the subject compound were used as received (grown from Benzene). A 0.140 x 0.080 x 0.035 mm colorless crystal was mounted on a Cryoloop with Paratone oil.

Data were collected in a nitrogen gas stream at 100(2) K using ϕ and ϖ scans. Crystal-to-detector distance was 50 mm using exposure time 3.0 second (depending on the 2θ position) with a scan width of 0.70°. Data collection was 99.8 complete to 25.242° in θ. A total of 24105 reflections were collected. 2731 reflections were found to be symmetry independent, with a R_{int} of 0.0433. Indexing and unit cell refinement indicated a **Primitive Orthorhombic** lattice. The space group was found to be $\boldsymbol{P2_12_12_1}$. The data were integrated using the Bruker SAINT Software program and scaled using the SADABS software program. Solution by direct methods (SHELXT) produced a complete phasing model consistent with the proposed structure.

All nonhydrogen atoms were refined anisotropically by full-matrix least-squares (SHELXL-2014). All carbon bonded hydrogen atoms were placed using a riding model. Their positions were constrained relative to their parent atom using the appropriate HFIX command in SHELXL-2014.

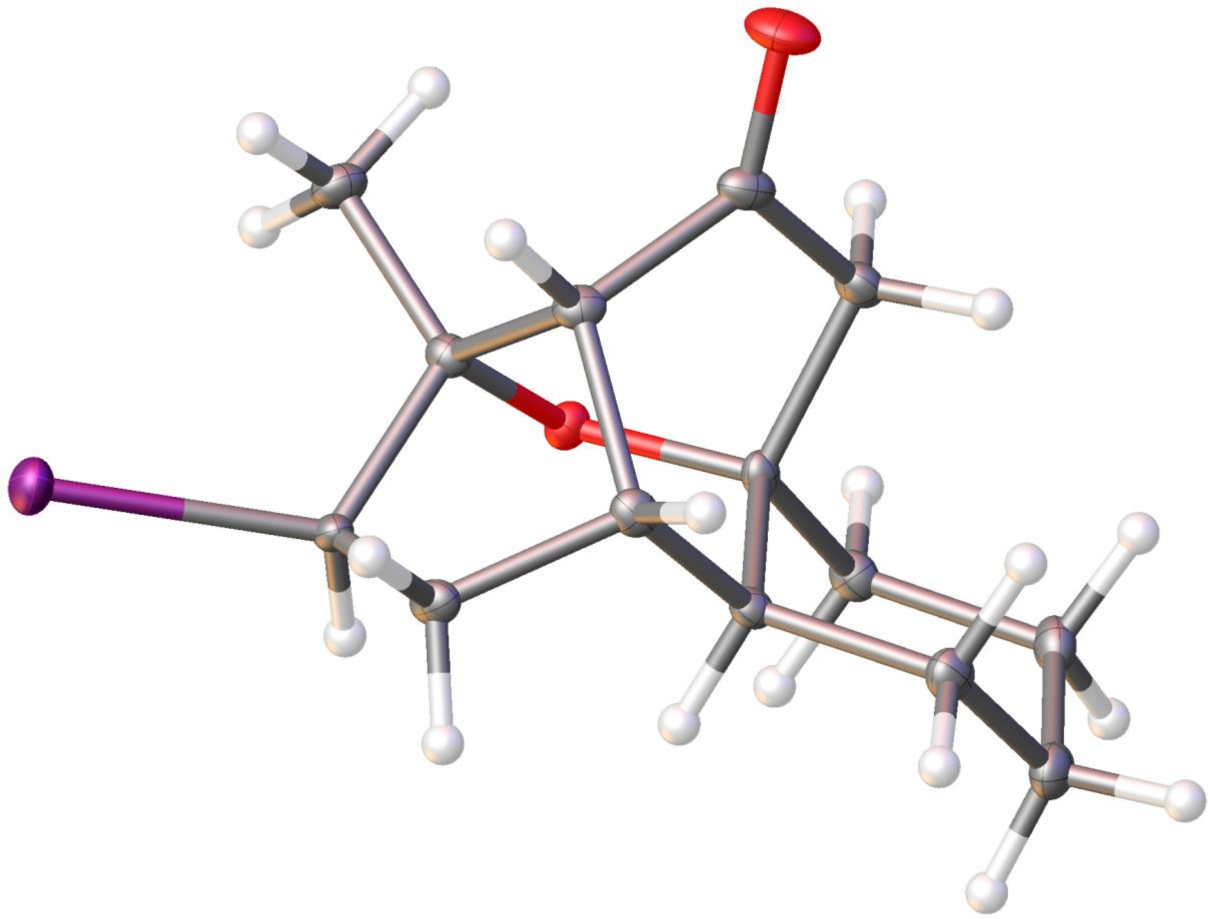

Figure S10. X-ray Structure of *(−)*-**4** (Shenvi284)

Crystal data and structure refinement for Shenvi284.

Report date	2022-04-25	
Identification code	shenvi284	
Empirical formula	C14 H19 I O2	
Molecular formula	C14 H19 I O2	
Formula weight	346.19	
Temperature	100.00 K	
Wavelength	0.71073 Å	
Crystal system	Orthorhombic	
Space group	$P2_12_12_1$	
Unit cell dimensions	a = 6.1927(2) Å	α= 90°.
	b = 12.8445(4) Å	β= 90°.
	c = 16.7729(5) Å	γ = 90°.
Volume	1334.15(7) $Å^3$	
Z	4	
Density (calculated)	1.724 Mg/m^3	
Absorption coefficient	2.389 mm^{-1}	
F(000)	688	
Crystal size	0.14 x 0.08 x 0.035 mm^3	
Crystal color, habit	colorless plank	
Theta range for data collection	2.901 to 26.371°.	
Index ranges	-7<=h<=7, -15<=k<=16, -20<=l<=20	
Reflections collected	24105	
Independent reflections	2731 [R(int) = 0.0433]	
Completeness to theta = 25.242°	99.8 %	
Absorption correction	Semi-empirical from equivalents	
Max. and min. transmission	0.6466 and 0.5664	
Refinement method	Full-matrix least-squares on F^2	
Data / restraints / parameters	2731 / 0 / 155	
Goodness-of-fit on F^2	1.055	
Final R indices [I>2sigma(I)]	R1 = 0.0150, wR2 = 0.0307	
R indices (all data)	R1 = 0.0165, wR2 = 0.0312	
Absolute structure parameter	-0.001(10)	
Largest diff. peak and hole	0.242 and -0.275 e.$Å^{-3}$	

Atomic coordinates (x 10^4) and equivalent isotropic displacement parameters ($Å^2$x 10^3) for Shenvi284. U(eq) is defined as one third of the trace of the orthogonalized U^{ij} tensor.

	x	y	z	U(eq)
I(1)	6439(1)	6540(1)	9532(1)	23(1)
O(1)	5954(3)	5375(1)	7164(1)	14(1)
O(2)	1524(4)	3285(1)	7542(1)	28(1)
C(1)	6547(5)	4447(2)	8372(2)	19(1)
C(2)	5156(4)	5263(2)	7976(2)	14(1)
C(3)	5137(4)	6387(2)	8338(2)	14(1)
C(4)	2783(4)	6764(2)	8297(2)	16(1)
C(5)	1775(5)	6042(2)	7671(2)	15(1)
C(6)	2725(5)	4979(2)	7944(2)	15(1)
C(7)	2347(5)	4109(2)	7364(2)	18(1)
C(8)	3180(5)	4364(2)	6541(2)	17(1)
C(9)	4250(4)	5442(2)	6571(2)	13(1)
C(10)	2640(4)	6283(2)	6825(2)	13(1)
C(11)	892(4)	6441(2)	6197(2)	18(1)
C(12)	1910(4)	6700(2)	5387(2)	21(1)
C(13)	3608(6)	5905(2)	5135(2)	19(1)
C(14)	5310(5)	5741(2)	5786(2)	16(1)

Bond lengths [Å] and angles [°] for Shenvi284.

I(1)-C(3)	2.168(3)	C(13)-H(13A)	0.9900
O(1)-C(2)	1.456(3)	C(13)-H(13B)	0.9900
O(1)-C(9)	1.453(3)	C(13)-C(14)	1.532(4)
O(2)-C(7)	1.212(3)	C(14)-H(14A)	0.9900
C(1)-H(1A)	0.9800	C(14)-H(14B)	0.9900
C(1)-H(1B)	0.9800		
C(1)-H(1C)	0.9800	C(9)-O(1)-C(2)	113.57(19)
C(1)-C(2)	1.511(4)	H(1A)-C(1)-H(1B)	109.5
C(2)-C(3)	1.565(4)	H(1A)-C(1)-H(1C)	109.5
C(2)-C(6)	1.550(4)	H(1B)-C(1)-H(1C)	109.5
C(3)-H(3)	1.0000	C(2)-C(1)-H(1A)	109.5
C(3)-C(4)	1.538(4)	C(2)-C(1)-H(1B)	109.5
C(4)-H(4A)	0.9900	C(2)-C(1)-H(1C)	109.5
C(4)-H(4B)	0.9900	O(1)-C(2)-C(1)	106.6(2)
C(4)-C(5)	1.534(4)	O(1)-C(2)-C(3)	105.9(2)
C(5)-H(5)	1.0000	O(1)-C(2)-C(6)	108.7(2)
C(5)-C(6)	1.555(4)	C(1)-C(2)-C(3)	118.3(2)
C(5)-C(10)	1.549(4)	C(1)-C(2)-C(6)	113.9(2)
C(6)-H(6)	1.0000	C(6)-C(2)-C(3)	102.9(2)
C(6)-C(7)	1.500(4)	I(1)-C(3)-H(3)	107.6
C(7)-C(8)	1.510(4)	C(2)-C(3)-I(1)	116.04(17)
C(8)-H(8A)	0.9900	C(2)-C(3)-H(3)	107.6
C(8)-H(8B)	0.9900	C(4)-C(3)-I(1)	111.37(18)
C(8)-C(9)	1.536(4)	C(4)-C(3)-C(2)	106.3(2)
C(9)-C(10)	1.530(4)	C(4)-C(3)-H(3)	107.6
C(9)-C(14)	1.520(4)	C(3)-C(4)-H(4A)	111.2
C(10)-H(10)	1.0000	C(3)-C(4)-H(4B)	111.2
C(10)-C(11)	1.524(4)	H(4A)-C(4)-H(4B)	109.1
C(11)-H(11A)	0.9900	C(5)-C(4)-C(3)	103.0(2)
C(11)-H(11B)	0.9900	C(5)-C(4)-H(4A)	111.2
C(11)-C(12)	1.534(4)	C(5)-C(4)-H(4B)	111.2
C(12)-H(12A)	0.9900	C(4)-C(5)-H(5)	112.1
C(12)-H(12B)	0.9900	C(4)-C(5)-C(6)	100.1(2)
C(12)-C(13)	1.525(4)	C(4)-C(5)-C(10)	111.5(2)

C(6)-C(5)-H(5)	112.1
C(10)-C(5)-H(5)	112.1
C(10)-C(5)-C(6)	108.3(2)
C(2)-C(6)-C(5)	99.8(2)
C(2)-C(6)-H(6)	110.8
C(5)-C(6)-H(6)	110.8
C(7)-C(6)-C(2)	110.4(2)
C(7)-C(6)-C(5)	113.8(3)
C(7)-C(6)-H(6)	110.8
O(2)-C(7)-C(6)	123.8(3)
O(2)-C(7)-C(8)	123.9(3)
C(6)-C(7)-C(8)	112.3(2)
C(7)-C(8)-H(8A)	110.1
C(7)-C(8)-H(8B)	110.1
C(7)-C(8)-C(9)	108.2(2)
H(8A)-C(8)-H(8B)	108.4
C(9)-C(8)-H(8A)	110.1
C(9)-C(8)-H(8B)	110.1
O(1)-C(9)-C(8)	106.4(2)
O(1)-C(9)-C(10)	109.0(2)
O(1)-C(9)-C(14)	107.1(2)
C(10)-C(9)-C(8)	111.3(2)
C(14)-C(9)-C(8)	112.6(2)
C(14)-C(9)-C(10)	110.2(2)
C(5)-C(10)-H(10)	106.9
C(9)-C(10)-C(5)	109.8(2)
C(9)-C(10)-H(10)	106.9
C(11)-C(10)-C(5)	114.5(2)
C(11)-C(10)-C(9)	111.4(2)
C(11)-C(10)-H(10)	106.9
C(10)-C(11)-H(11A)	109.6
C(10)-C(11)-H(11B)	109.6
C(10)-C(11)-C(12)	110.4(2)
H(11A)-C(11)-H(11B)	108.1
C(12)-C(11)-H(11A)	109.6
C(12)-C(11)-H(11B)	109.6
C(11)-C(12)-H(12A)	109.1
C(11)-C(12)-H(12B)	109.1
H(12A)-C(12)-H(12B)	107.8
C(13)-C(12)-C(11)	112.6(2)
C(13)-C(12)-H(12A)	109.1
C(13)-C(12)-H(12B)	109.1
C(12)-C(13)-H(13A)	109.3
C(12)-C(13)-H(13B)	109.3
C(12)-C(13)-C(14)	111.7(2)
H(13A)-C(13)-H(13B)	107.9
C(14)-C(13)-H(13A)	109.3
C(14)-C(13)-H(13B)	109.3
C(9)-C(14)-C(13)	110.8(2)
C(9)-C(14)-H(14A)	109.5
C(9)-C(14)-H(14B)	109.5
C(13)-C(14)-H(14A)	109.5
C(13)-C(14)-H(14B)	109.5
H(14A)-C(14)-H(14B)	108.1

Anisotropic displacement parameters ($Å^2$x 10^3) for Shenvi284. The anisotropic displacement factor exponent takes the form: $-2\pi^2[h^2 a^{*2}U^{11} + ... + 2 h k a^* b^* U^{12}]$

	U^{11}	U^{22}	U^{33}	U^{23}	U^{13}	U^{12}
I(1)	28(1)	22(1)	17(1)	-1(1)	-7(1)	1(1)
O(1)	12(1)	17(1)	14(1)	1(1)	0(1)	2(1)
O(2)	34(1)	14(1)	37(1)	2(1)	6(1)	-7(1)
C(1)	20(2)	16(1)	21(1)	4(1)	-2(1)	3(1)
C(2)	14(2)	13(1)	14(1)	-1(1)	1(1)	-1(1)
C(3)	19(1)	10(1)	12(1)	1(1)	-3(1)	1(1)
C(4)	21(2)	14(2)	14(1)	-1(1)	2(1)	2(1)
C(5)	11(2)	15(1)	18(1)	-2(1)	1(1)	1(1)
C(6)	13(2)	15(1)	17(1)	2(1)	4(1)	0(1)
C(7)	14(1)	13(2)	26(2)	2(1)	0(1)	0(1)
C(8)	18(2)	13(1)	20(1)	-3(1)	-1(1)	0(1)
C(9)	10(1)	14(1)	13(1)	-2(1)	-2(1)	1(1)
C(10)	12(1)	12(1)	16(1)	0(1)	-1(1)	-2(1)
C(11)	14(1)	19(1)	21(1)	-1(1)	-4(1)	1(1)
C(12)	19(1)	23(1)	20(1)	1(1)	-6(1)	-2(1)
C(13)	21(2)	23(1)	13(1)	-1(1)	-2(1)	-5(2)
C(14)	15(2)	17(1)	17(1)	-3(1)	2(1)	0(1)

Hydrogen coordinates (x 10^4) and isotropic displacement parameters ($Å^2$x 10^3) for Shenvi284.

	x	y	z	U(eq)
H(1A)	6429	3790	8078	28
H(1B)	6059	4342	8922	28
H(1C)	8055	4679	8374	28
H(3)	6012	6843	7978	17
H(4A)	2056	6688	8820	19
H(4B)	2704	7501	8127	19
H(5)	162	6049	7693	18
H(6)	2180	4789	8486	18
H(8A)	1974	4367	6154	20
H(8B)	4244	3834	6370	20
H(10)	3462	6951	6862	16
H(11A)	-74	7015	6362	22
H(11B)	14	5800	6149	22
H(12A)	761	6727	4977	25
H(12B)	2587	7397	5416	25
H(13A)	4325	6148	4642	23
H(13B)	2892	5234	5017	23
H(14A)	6319	5183	5620	20
H(14B)	6152	6389	5858	20

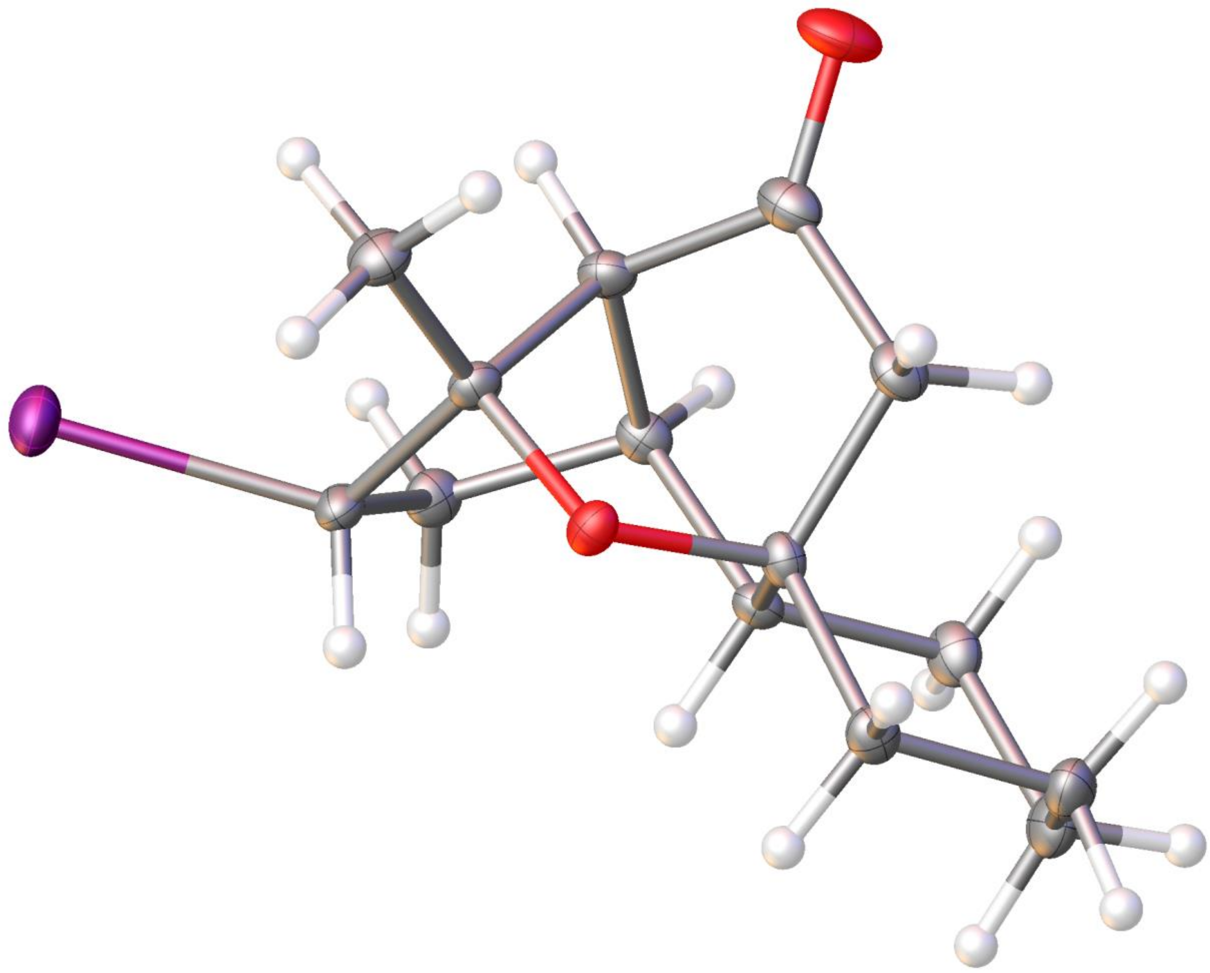

Figure S11. X-ray structure of *(+)*-**4** (Shenvi283)

Crystal data and structure refinement for Shenvi283.

Report date	2022-04-19	
Identification code	shenvi283	
Empirical formula	C14 H19 I O2	
Molecular formula	C14 H19 I O2	
Formula weight	346.19	
Temperature	100.00 K	
Wavelength	0.71073 Å	
Crystal system	Orthorhombic	
Space group	$P2_12_12_1$	
Unit cell dimensions	a = 6.1913(2) Å	α= 90°.
	b = 12.8492(3) Å	β= 90°.
	c = 16.7666(4) Å	γ = 90°.
Volume	1333.84(6) Å^3	
Z	4	
Density (calculated)	1.724 Mg/m^3	
Absorption coefficient	2.389 mm^{-1}	
F(000)	688	
Crystal size	0.18 x 0.12 x 0.08 mm^3	
Crystal color, habit	colorless irregular	
Theta range for data collection	2.429 to 26.367°.	
Index ranges	-7<=h<=7, -16<=k<=16, -20<=l<=20	
Reflections collected	23975	
Independent reflections	2732 [R(int) = 0.0346]	
Completeness to theta = 25.242°	99.9 %	
Absorption correction	Semi-empirical from equivalents	
Max. and min. transmission	0.6466 and 0.5896	
Refinement method	Full-matrix least-squares on F^2	
Data / restraints / parameters	2732 / 0 / 155	
Goodness-of-fit on F^2	1.068	
Final R indices [I>2sigma(I)]	R1 = 0.0123, wR2 = 0.0281	
R indices (all data)	R1 = 0.0128, wR2 = 0.0282	
Absolute structure parameter	-0.015(8)	
Largest diff. peak and hole	0.221 and -0.195 e.Å^{-3}	

Atomic coordinates (x 10^4) and equivalent isotropic displacement parameters ($Å^2$x 10^3) for Shenvi283. U(eq) is defined as one third of the trace of the orthogonalized U^{ij} tensor.

	x	y	z	U(eq)
I(1)	3558(1)	6541(1)	9532(1)	22(1)
O(1)	4047(2)	5376(1)	7162(1)	14(1)
O(2)	8480(3)	3286(1)	7543(1)	28(1)
C(1)	3449(4)	4449(2)	8372(1)	19(1)
C(2)	4845(4)	5262(2)	7975(1)	13(1)
C(3)	4861(4)	6388(2)	8337(1)	14(1)
C(4)	7215(4)	6766(2)	8297(1)	16(1)
C(5)	8226(4)	6042(2)	7672(1)	14(1)
C(6)	7273(4)	4984(2)	7947(2)	15(1)
C(7)	7647(4)	4108(2)	7364(2)	18(1)
C(8)	6820(4)	4365(2)	6544(1)	17(1)
C(9)	5747(4)	5440(2)	6571(1)	13(1)
C(10)	7359(4)	6285(2)	6825(1)	13(1)
C(11)	9113(3)	6438(2)	6195(1)	18(1)
C(12)	8091(4)	6699(2)	5384(1)	20(1)
C(13)	6394(5)	5906(2)	5133(1)	20(1)
C(14)	4689(4)	5740(2)	5786(1)	16(1)

Bond lengths [Å] and angles [°] for Shenvi283.

I(1)-C(3)	2.169(2)	C(13)-H(13A)	0.9900
O(1)-C(2)	1.456(3)	C(13)-H(13B)	0.9900
O(1)-C(9)	1.449(3)	C(13)-C(14)	1.536(3)
O(2)-C(7)	1.213(3)	C(14)-H(14A)	0.9900
C(1)-H(1A)	0.9800	C(14)-H(14B)	0.9900
C(1)-H(1B)	0.9800		
C(1)-H(1C)	0.9800	C(9)-O(1)-C(2)	113.55(16)
C(1)-C(2)	1.511(3)	H(1A)-C(1)-H(1B)	109.5
C(2)-C(3)	1.569(3)	H(1A)-C(1)-H(1C)	109.5
C(2)-C(6)	1.545(3)	H(1B)-C(1)-H(1C)	109.5
C(3)-H(3)	1.0000	C(2)-C(1)-H(1A)	109.5
C(3)-C(4)	1.538(3)	C(2)-C(1)-H(1B)	109.5
C(4)-H(4A)	0.9900	C(2)-C(1)-H(1C)	109.5
C(4)-H(4B)	0.9900	O(1)-C(2)-C(1)	106.73(18)
C(4)-C(5)	1.534(3)	O(1)-C(2)-C(3)	105.78(17)
C(5)-H(5)	1.0000	O(1)-C(2)-C(6)	108.93(19)
C(5)-C(6)	1.552(3)	C(1)-C(2)-C(3)	118.06(19)
C(5)-C(10)	1.551(3)	C(1)-C(2)-C(6)	114.2(2)
C(6)-H(6)	1.0000	C(6)-C(2)-C(3)	102.65(19)
C(6)-C(7)	1.508(4)	I(1)-C(3)-H(3)	107.6
C(7)-C(8)	1.504(3)	C(2)-C(3)-I(1)	116.02(14)
C(8)-H(8A)	0.9900	C(2)-C(3)-H(3)	107.6
C(8)-H(8B)	0.9900	C(4)-C(3)-I(1)	111.36(15)
C(8)-C(9)	1.533(3)	C(4)-C(3)-C(2)	106.29(18)
C(9)-C(10)	1.535(3)	C(4)-C(3)-H(3)	107.6
C(9)-C(14)	1.520(3)	C(3)-C(4)-H(4A)	111.2
C(10)-H(10)	1.0000	C(3)-C(4)-H(4B)	111.2
C(10)-C(11)	1.527(3)	H(4A)-C(4)-H(4B)	109.1
C(11)-H(11A)	0.9900	C(5)-C(4)-C(3)	102.98(18)
C(11)-H(11B)	0.9900	C(5)-C(4)-H(4A)	111.2
C(11)-C(12)	1.537(3)	C(5)-C(4)-H(4B)	111.2
C(12)-H(12A)	0.9900	C(4)-C(5)-H(5)	112.2
C(12)-H(12B)	0.9900	C(4)-C(5)-C(6)	99.99(19)
C(12)-C(13)	1.524(3)	C(4)-C(5)-C(10)	111.26(18)

C(6)-C(5)-H(5)	112.2
C(10)-C(5)-H(5)	112.2
C(10)-C(5)-C(6)	108.46(19)
C(2)-C(6)-C(5)	100.14(19)
C(2)-C(6)-H(6)	110.8
C(5)-C(6)-H(6)	110.8
C(7)-C(6)-C(2)	110.0(2)
C(7)-C(6)-C(5)	113.7(2)
C(7)-C(6)-H(6)	110.8
O(2)-C(7)-C(6)	123.7(2)
O(2)-C(7)-C(8)	124.2(2)
C(8)-C(7)-C(6)	112.1(2)
C(7)-C(8)-H(8A)	110.0
C(7)-C(8)-H(8B)	110.0
C(7)-C(8)-C(9)	108.55(18)
H(8A)-C(8)-H(8B)	108.4
C(9)-C(8)-H(8A)	110.0
C(9)-C(8)-H(8B)	110.0
O(1)-C(9)-C(8)	106.48(17)
O(1)-C(9)-C(10)	108.85(17)
O(1)-C(9)-C(14)	107.10(17)
C(8)-C(9)-C(10)	111.35(18)
C(14)-C(9)-C(8)	112.93(19)
C(14)-C(9)-C(10)	109.92(19)
C(5)-C(10)-H(10)	107.1
C(9)-C(10)-C(5)	109.65(18)
C(9)-C(10)-H(10)	107.1
C(11)-C(10)-C(5)	114.39(18)
C(11)-C(10)-C(9)	111.23(18)
C(11)-C(10)-H(10)	107.1
C(10)-C(11)-H(11A)	109.6
C(10)-C(11)-H(11B)	109.6
C(10)-C(11)-C(12)	110.29(18)
H(11A)-C(11)-H(11B)	108.1
C(12)-C(11)-H(11A)	109.6
C(12)-C(11)-H(11B)	109.6
C(11)-C(12)-H(12A)	109.1
C(11)-C(12)-H(12B)	109.1
H(12A)-C(12)-H(12B)	107.8
C(13)-C(12)-C(11)	112.50(19)
C(13)-C(12)-H(12A)	109.1
C(13)-C(12)-H(12B)	109.1
C(12)-C(13)-H(13A)	109.3
C(12)-C(13)-H(13B)	109.3
C(12)-C(13)-C(14)	111.68(19)
H(13A)-C(13)-H(13B)	107.9
C(14)-C(13)-H(13A)	109.3
C(14)-C(13)-H(13B)	109.3
C(9)-C(14)-C(13)	110.88(19)
C(9)-C(14)-H(14A)	109.5
C(9)-C(14)-H(14B)	109.5
C(13)-C(14)-H(14A)	109.5
C(13)-C(14)-H(14B)	109.5
H(14A)-C(14)-H(14B)	108.1

Anisotropic displacement parameters ($Å^2 x 10^3$) for Shenvi283. The anisotropic displacement factor exponent takes the form: $-2\pi^2[h^2 a^{*2}U^{11} + ... + 2 h k a^* b^* U^{12}]$

	U^{11}	U^{22}	U^{33}	U^{23}	U^{13}	U^{12}
I(1)	28(1)	22(1)	17(1)	-1(1)	7(1)	-1(1)
O(1)	11(1)	17(1)	12(1)	1(1)	0(1)	-2(1)
O(2)	33(1)	15(1)	37(1)	2(1)	-5(1)	9(1)
C(1)	20(1)	16(1)	19(1)	3(1)	1(1)	-3(1)
C(2)	13(1)	14(1)	12(1)	1(1)	-2(1)	0(1)
C(3)	18(1)	12(1)	12(1)	2(1)	2(1)	0(1)
C(4)	18(1)	16(1)	15(1)	-2(1)	-2(1)	-2(1)
C(5)	11(1)	14(1)	17(1)	-1(1)	-2(1)	-1(1)
C(6)	14(1)	15(1)	17(1)	2(1)	-2(1)	1(1)
C(7)	15(1)	14(1)	26(1)	1(1)	2(1)	1(1)
C(8)	19(1)	12(1)	20(1)	-3(1)	0(1)	-1(1)
C(9)	10(1)	14(1)	14(1)	-2(1)	2(1)	0(1)
C(10)	12(1)	11(1)	15(1)	0(1)	-1(1)	2(1)
C(11)	14(1)	18(1)	21(1)	-1(1)	4(1)	-3(1)
C(12)	18(1)	23(1)	18(1)	1(1)	6(1)	0(1)
C(13)	21(1)	24(1)	13(1)	-1(1)	1(1)	4(1)
C(14)	15(1)	18(1)	15(1)	-2(1)	-2(1)	1(1)

Hydrogen coordinates (x 10^4) and isotropic displacement parameters ($Å^2$x 10^3) for Shenvi283.

	x	y	z	U(eq)
H(1A)	1942	4682	8371	28
H(1B)	3932	4348	8924	28
H(1C)	3569	3791	8081	28
H(3)	3986	6843	7977	17
H(4A)	7294	7502	8126	20
H(4B)	7941	6691	8820	20
H(5)	9840	6049	7694	17
H(6)	7816	4794	8489	18
H(8A)	5759	3835	6372	21
H(8B)	8029	4370	6158	21
H(10)	6537	6953	6861	15
H(11A)	9984	5795	6148	21
H(11B)	10086	7011	6360	21
H(12A)	7414	7396	5414	24
H(12B)	9240	6727	4974	24
H(13A)	7109	5235	5014	23
H(13B)	5674	6150	4640	23
H(14A)	3847	6388	5859	19
H(14B)	3679	5184	5620	19

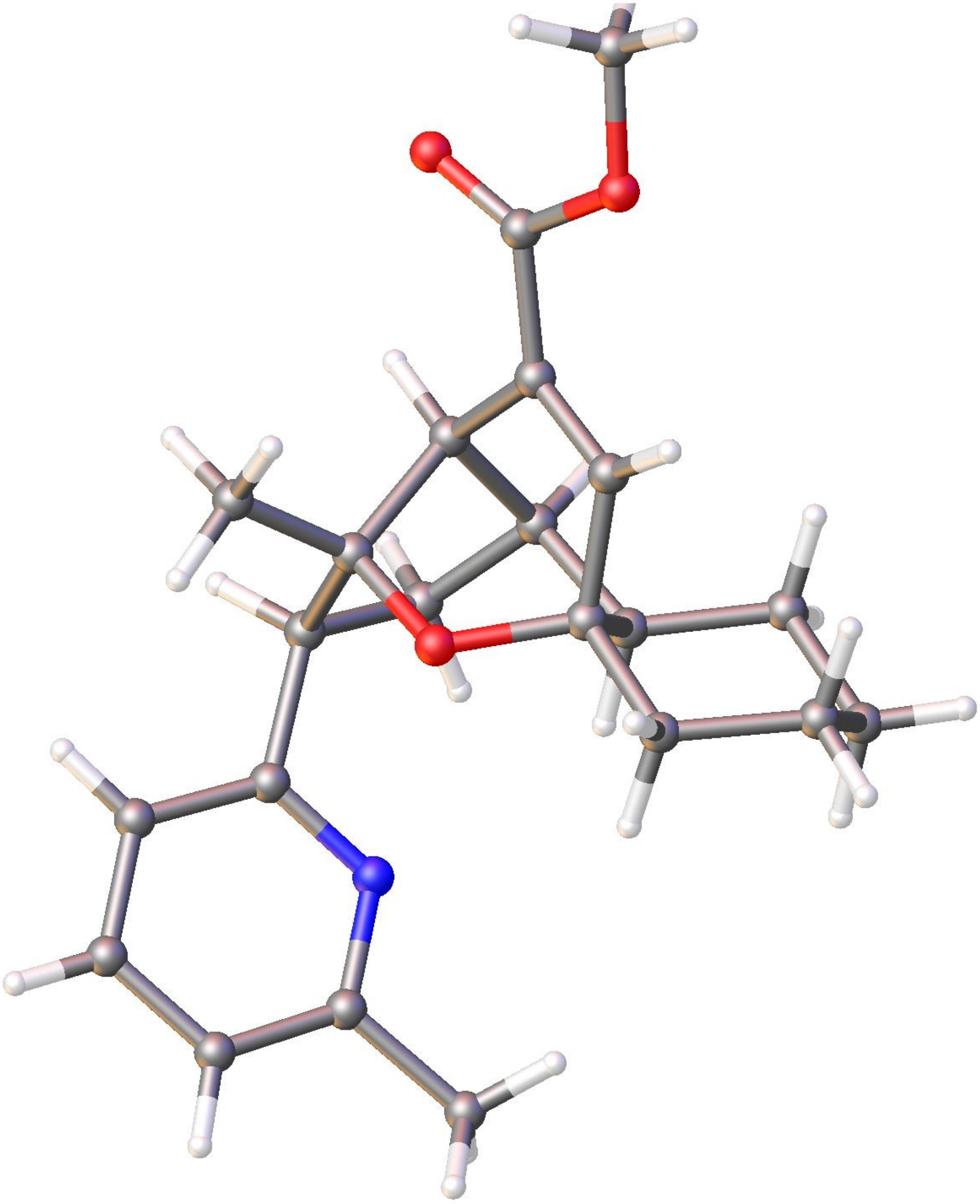

Figure S12. X-ray structure of *(+)*-**2** (Shenvi285)

Crystal data and structure refinement for Shenvi285.

Identification code	shenvi285_0m_a	
Empirical formula	C22 H27 N O3	
Formula weight	353.44	
Temperature	100.00 K	
Wavelength	1.54178 Å	
Crystal system	Monoclinic	
Space group	P 1 21 1	
Unit cell dimensions	a = 8.9728(3) Å	α= 90°.
	b = 9.8544(3) Å	β= 94.8200(10)°.
	c = 10.3733(3) Å	γ = 90°.
Volume	913.98(5) $Å^3$	
Z	2	
Density (calculated)	1.284 Mg/m^3	
Absorption coefficient	0.674 mm^{-1}	
F(000)	380	
Crystal size	0.25 x 0.24 x 0.2 mm^3	
Theta range for data collection	6.267 to 68.695°.	
Index ranges	-10<=h<=10, -11<=k<=11, -12<=l<=12	
Reflections collected	14279	
Independent reflections	3300 [R(int) = 0.0296]	
Completeness to theta = 67.679°	99.2 %	
Absorption correction	Semi-empirical from equivalents	
Max. and min. transmission	0.5213 and 0.4353	
Refinement method	Full-matrix least-squares on F^2	
Data / restraints / parameters	3300 / 1 / 238	
Goodness-of-fit on F^2	1.060	
Final R indices [I>2sigma(I)]	R1 = 0.0251, wR2 = 0.0642	
R indices (all data)	R1 = 0.0254, wR2 = 0.0645	
Absolute structure parameter	-0.03(4)	
Largest diff. peak and hole	0.228 and -0.154 e.$Å^{-3}$	

Atomic coordinates (x 10^4) and equivalent isotropic displacement parameters ($Å^2$x 10^3) for Shenvi285. U(eq) is defined as one third of the trace of the orthogonalized U^{ij} tensor.

	x	y	z	U(eq)
O(1)	6688(1)	5739(1)	3143(1)	13(1)
O(2)	5322(1)	3812(1)	7045(1)	20(1)
O(3)	3077(1)	4225(1)	5980(1)	17(1)
N(1)	9136(2)	5207(2)	1408(1)	16(1)
C(1)	8965(2)	5839(2)	-857(2)	20(1)
C(2)	9554(2)	6095(2)	524(2)	16(1)
C(3)	10508(2)	7183(2)	845(2)	18(1)
C(4)	11088(2)	7331(2)	2114(2)	19(1)
C(5)	10648(2)	6421(2)	3032(2)	18(1)
C(6)	9650(2)	5388(2)	2650(2)	15(1)
C(7)	9044(2)	4477(2)	3656(2)	16(1)
C(8)	8608(2)	3009(2)	3231(2)	17(1)
C(9)	6924(2)	2899(2)	3391(2)	15(1)
C(10)	6754(2)	3803(2)	4591(2)	14(1)
C(11)	7592(2)	5087(2)	4179(2)	14(1)
C(12)	7892(2)	6127(2)	5246(2)	17(1)
C(13)	5410(2)	4927(2)	2629(2)	13(1)
C(14)	4655(2)	5675(2)	1474(2)	14(1)
C(15)	3390(2)	4805(2)	821(2)	16(1)
C(16)	3949(2)	3407(2)	435(2)	17(1)
C(17)	4725(2)	2666(2)	1603(2)	17(1)
C(18)	6000(2)	3541(2)	2218(2)	14(1)
C(19)	4442(2)	4739(2)	3733(2)	14(1)
C(20)	5164(2)	4162(2)	4768(2)	14(1)
C(21)	4565(2)	4030(2)	6050(2)	15(1)
C(22)	2408(2)	4089(2)	7191(2)	21(1)

Bond lengths [Å] and angles [°] for Shenvi285.

O(1)-C(11)	1.4415(19)	C(12)-H(12C)	0.9800
O(1)-C(13)	1.4623(19)	C(13)-C(14)	1.517(2)
O(2)-C(21)	1.206(2)	C(13)-C(18)	1.538(2)
O(3)-C(21)	1.345(2)	C(13)-C(19)	1.505(2)
O(3)-C(22)	1.4430(19)	C(14)-H(14A)	0.9900
N(1)-C(2)	1.344(2)	C(14)-H(14B)	0.9900
N(1)-C(6)	1.343(2)	C(14)-C(15)	1.534(2)
C(1)-H(1A)	0.9800	C(15)-H(15A)	0.9900
C(1)-H(1B)	0.9800	C(15)-H(15B)	0.9900
C(1)-H(1C)	0.9800	C(15)-C(16)	1.531(2)
C(1)-C(2)	1.506(2)	C(16)-H(16A)	0.9900
C(2)-C(3)	1.395(3)	C(16)-H(16B)	0.9900
C(3)-H(3)	0.9500	C(16)-C(17)	1.531(2)
C(3)-C(4)	1.382(2)	C(17)-H(17A)	0.9900
C(4)-H(4)	0.9500	C(17)-H(17B)	0.9900
C(4)-C(5)	1.390(3)	C(17)-C(18)	1.529(2)
C(5)-H(5)	0.9500	C(18)-H(18)	1.0000
C(5)-C(6)	1.391(2)	C(19)-H(19)	0.9500
C(6)-C(7)	1.512(2)	C(19)-C(20)	1.333(2)
C(7)-H(7)	1.0000	C(20)-C(21)	1.481(2)
C(7)-C(8)	1.553(2)	C(22)-H(22A)	0.9800
C(7)-C(11)	1.573(2)	C(22)-H(22B)	0.9800
C(8)-H(8A)	0.9900	C(22)-H(22C)	0.9800
C(8)-H(8B)	0.9900		
C(8)-C(9)	1.537(2)	C(11)-O(1)-C(13)	113.78(12)
C(9)-H(9)	1.0000	C(21)-O(3)-C(22)	115.05(13)
C(9)-C(10)	1.549(2)	C(6)-N(1)-C(2)	118.36(15)
C(9)-C(18)	1.548(2)	H(1A)-C(1)-H(1B)	109.5
C(10)-H(10)	1.0000	H(1A)-C(1)-H(1C)	109.5
C(10)-C(11)	1.550(2)	H(1B)-C(1)-H(1C)	109.5
C(10)-C(20)	1.497(2)	C(2)-C(1)-H(1A)	109.5
C(11)-C(12)	1.515(2)	C(2)-C(1)-H(1B)	109.5
C(12)-H(12A)	0.9800	C(2)-C(1)-H(1C)	109.5
C(12)-H(12B)	0.9800	N(1)-C(2)-C(1)	116.37(15)

N(1)-C(2)-C(3)	122.59(15)	C(20)-C(10)-H(10)	111.4
C(3)-C(2)-C(1)	121.03(15)	C(20)-C(10)-C(11)	109.27(13)
C(2)-C(3)-H(3)	120.6	O(1)-C(11)-C(7)	110.11(12)
C(4)-C(3)-C(2)	118.89(16)	O(1)-C(11)-C(10)	108.30(12)
C(4)-C(3)-H(3)	120.6	O(1)-C(11)-C(12)	107.31(13)
C(3)-C(4)-H(4)	120.7	C(10)-C(11)-C(7)	102.59(13)
C(3)-C(4)-C(5)	118.62(17)	C(12)-C(11)-C(7)	114.10(13)
C(5)-C(4)-H(4)	120.7	C(12)-C(11)-C(10)	114.29(14)
C(4)-C(5)-H(5)	120.3	C(11)-C(12)-H(12A)	109.5
C(4)-C(5)-C(6)	119.33(16)	C(11)-C(12)-H(12B)	109.5
C(6)-C(5)-H(5)	120.3	C(11)-C(12)-H(12C)	109.5
N(1)-C(6)-C(5)	122.12(15)	H(12A)-C(12)-H(12B)	109.5
N(1)-C(6)-C(7)	117.97(14)	H(12A)-C(12)-H(12C)	109.5
C(5)-C(6)-C(7)	119.83(15)	H(12B)-C(12)-H(12C)	109.5
C(6)-C(7)-H(7)	107.7	O(1)-C(13)-C(14)	107.86(13)
C(6)-C(7)-C(8)	116.97(14)	O(1)-C(13)-C(18)	107.99(12)
C(6)-C(7)-C(11)	111.54(13)	O(1)-C(13)-C(19)	105.91(12)
C(8)-C(7)-H(7)	107.7	C(14)-C(13)-C(18)	110.75(13)
C(8)-C(7)-C(11)	104.92(13)	C(19)-C(13)-C(14)	114.40(13)
C(11)-C(7)-H(7)	107.7	C(19)-C(13)-C(18)	109.62(13)
C(7)-C(8)-H(8A)	110.7	C(13)-C(14)-H(14A)	109.7
C(7)-C(8)-H(8B)	110.7	C(13)-C(14)-H(14B)	109.7
H(8A)-C(8)-H(8B)	108.8	C(13)-C(14)-C(15)	109.96(13)
C(9)-C(8)-C(7)	105.17(13)	H(14A)-C(14)-H(14B)	108.2
C(9)-C(8)-H(8A)	110.7	C(15)-C(14)-H(14A)	109.7
C(9)-C(8)-H(8B)	110.7	C(15)-C(14)-H(14B)	109.7
C(8)-C(9)-H(9)	111.8	C(14)-C(15)-H(15A)	109.3
C(8)-C(9)-C(10)	102.12(13)	C(14)-C(15)-H(15B)	109.3
C(8)-C(9)-C(18)	110.65(13)	H(15A)-C(15)-H(15B)	107.9
C(10)-C(9)-H(9)	111.8	C(16)-C(15)-C(14)	111.80(14)
C(18)-C(9)-H(9)	111.8	C(16)-C(15)-H(15A)	109.3
C(18)-C(9)-C(10)	108.34(13)	C(16)-C(15)-H(15B)	109.3
C(9)-C(10)-H(10)	111.4	C(15)-C(16)-H(16A)	109.4
C(9)-C(10)-C(11)	99.49(13)	C(15)-C(16)-H(16B)	109.4
C(11)-C(10)-H(10)	111.4	H(16A)-C(16)-H(16B)	108.0
C(20)-C(10)-C(9)	113.18(13)	C(17)-C(16)-C(15)	111.09(14)

C(17)-C(16)-H(16A)	109.4
C(17)-C(16)-H(16B)	109.4
C(16)-C(17)-H(17A)	109.7
C(16)-C(17)-H(17B)	109.7
H(17A)-C(17)-H(17B)	108.2
C(18)-C(17)-C(16)	109.60(14)
C(18)-C(17)-H(17A)	109.7
C(18)-C(17)-H(17B)	109.7
C(9)-C(18)-H(18)	107.2
C(13)-C(18)-C(9)	108.62(13)
C(13)-C(18)-H(18)	107.2
C(17)-C(18)-C(9)	115.48(14)
C(17)-C(18)-C(13)	110.67(13)
C(17)-C(18)-H(18)	107.2
C(13)-C(19)-H(19)	123.4
C(20)-C(19)-C(13)	113.17(14)
C(20)-C(19)-H(19)	123.4
C(19)-C(20)-C(10)	113.91(14)
C(19)-C(20)-C(21)	124.92(15)
C(21)-C(20)-C(10)	120.71(14)
O(2)-C(21)-O(3)	123.79(15)
O(2)-C(21)-C(20)	124.31(15)
O(3)-C(21)-C(20)	111.87(13)
O(3)-C(22)-H(22A)	109.5
O(3)-C(22)-H(22B)	109.5
O(3)-C(22)-H(22C)	109.5
H(22A)-C(22)-H(22B)	109.5
H(22A)-C(22)-H(22C)	109.5
H(22B)-C(22)-H(22C)	109.5

Anisotropic displacement parameters (Å^{2}x 10^3) for Shenvi285. The anisotropic displacement factor exponent takes the form: $-2\pi^2[h^2 a^{*2}U^{11} + ... + 2 h k a^* b^* U^{12}]$

	U^{11}	U^{22}	U^{33}	U^{23}	U^{13}	U^{12}
O(1)	11(1)	13(1)	16(1)	2(1)	-1(1)	-1(1)
O(2)	20(1)	26(1)	15(1)	4(1)	2(1)	0(1)
O(3)	15(1)	21(1)	16(1)	0(1)	5(1)	1(1)
N(1)	12(1)	18(1)	18(1)	0(1)	3(1)	2(1)
C(1)	22(1)	21(1)	18(1)	2(1)	4(1)	0(1)
C(2)	12(1)	17(1)	18(1)	0(1)	3(1)	5(1)
C(3)	17(1)	16(1)	21(1)	2(1)	6(1)	2(1)
C(4)	13(1)	18(1)	24(1)	-4(1)	2(1)	-1(1)
C(5)	13(1)	22(1)	18(1)	-3(1)	2(1)	3(1)
C(6)	10(1)	18(1)	17(1)	0(1)	3(1)	4(1)
C(7)	12(1)	20(1)	16(1)	2(1)	1(1)	2(1)
C(8)	16(1)	16(1)	18(1)	2(1)	2(1)	5(1)
C(9)	16(1)	12(1)	17(1)	2(1)	3(1)	2(1)
C(10)	14(1)	14(1)	13(1)	1(1)	1(1)	1(1)
C(11)	12(1)	15(1)	15(1)	2(1)	0(1)	1(1)
C(12)	15(1)	19(1)	18(1)	-2(1)	0(1)	-1(1)
C(13)	11(1)	13(1)	14(1)	0(1)	1(1)	-2(1)
C(14)	14(1)	14(1)	14(1)	2(1)	1(1)	2(1)
C(15)	15(1)	20(1)	14(1)	1(1)	0(1)	1(1)
C(16)	18(1)	17(1)	16(1)	-3(1)	0(1)	-2(1)
C(17)	19(1)	14(1)	18(1)	-2(1)	1(1)	-1(1)
C(18)	15(1)	13(1)	13(1)	0(1)	3(1)	0(1)
C(19)	12(1)	12(1)	17(1)	-2(1)	2(1)	0(1)
C(20)	14(1)	12(1)	16(1)	-2(1)	2(1)	-2(1)
C(21)	16(1)	10(1)	18(1)	-1(1)	3(1)	-1(1)
C(22)	21(1)	24(1)	19(1)	1(1)	10(1)	-2(1)

Hydrogen coordinates (x 10^4) and isotropic displacement parameters (Å^{2}x 10 ^{3}) for Shenvi285.

	x	y	z	U(eq)
H(1A)	9646	5227	-1267	30
H(1B)	8893	6701	-1330	30
H(1C)	7972	5421	-874	30
H(3)	10756	7811	202	21
H(4)	11773	8040	2354	22
H(5)	11025	6503	3912	21
H(7)	9824	4412	4402	19
H(8A)	8811	2853	2319	20
H(8B)	9177	2336	3784	20
H(9)	6616	1941	3541	18
H(10)	7260	3389	5393	17
H(12A)	8404	6913	4908	26
H(12B)	8525	5722	5960	26
H(12C)	6942	6418	5558	26
H(14A)	5398	5877	846	17
H(14B)	4244	6546	1761	17
H(15A)	2598	4689	1422	20
H(15B)	2944	5279	41	20
H(16A)	3094	2858	65	20
H(16B)	4660	3515	-238	20
H(17A)	3994	2480	2245	20
H(17B)	5125	1787	1323	20
H(18)	6704	3714	1538	17
H(19)	3422	5005	3696	16
H(22A)	1335	4283	7057	31
H(22B)	2881	4729	7822	31
H(22C)	2555	3160	7516	31

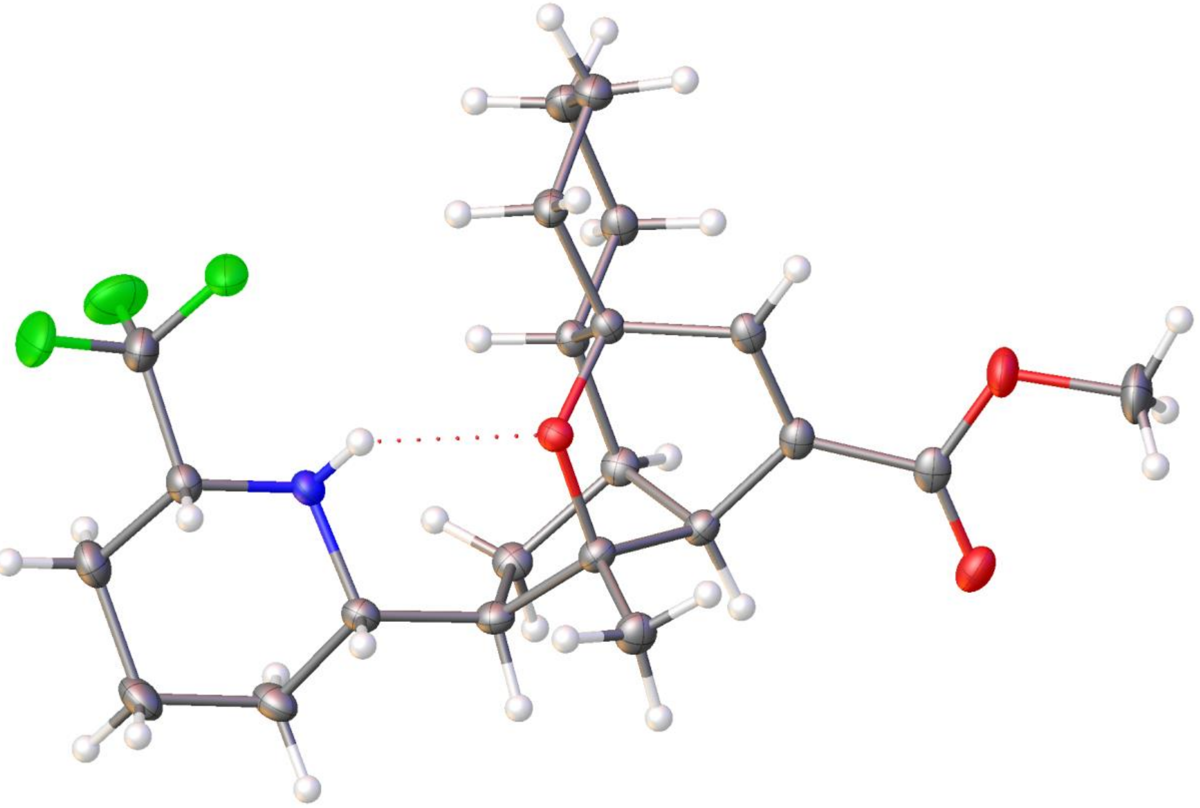

Figure S13. X-ray structure of **RS1122** (Shenvi293)

Crystal data and structure refinement for shenvi293.

Report date	2023-03-12	
Identification code	shenvi293	
Empirical formula	C22 H30 F3 N O3	
Molecular formula	C22 H30 F3 N O3	
Formula weight	413.47	
Temperature	100.15 K	
Wavelength	1.54178 Å	
Crystal system	Orthorhombic	
Space group	$P2_12_12_1$	
Unit cell dimensions	a = 7.4030(2) Å	α= 90°.
	b = 11.0678(2) Å	β= 90°.
	c = 25.2218(5) Å	γ = 90°.
Volume	2066.55(8) $Å^3$	
Z	4	
Density (calculated)	1.329 Mg/m^3	
Absorption coefficient	0.886 mm^{-1}	
F(000)	880	
Crystal size	0.32 x 0.2 x 0.175 mm^3	
Crystal color, habit	colorless block	
Theta range for data collection	3.505 to 70.083°.	
Index ranges	-7<=h<=8, -12<=k<=13, -19<=l<=30	
Reflections collected	11993	
Independent reflections	3871 [R(int) = 0.0243]	
Completeness to theta = 67.679°	100.0 %	
Absorption correction	Semi-empirical from equivalents	
Max. and min. transmission	0.7533 and 0.6412	
Refinement method	Full-matrix least-squares on F^2	
Data / restraints / parameters	3871 / 0 / 268	
Goodness-of-fit on F^2	1.050	
Final R indices [I>2sigma(I)]	R1 = 0.0257, wR2 = 0.0655	
R indices (all data)	R1 = 0.0263, wR2 = 0.0659	
Absolute structure parameter	0.01(4)	
Extinction coefficient	0.0011(2)	
Largest diff. peak and hole	0.200 and -0.161 e.$Å^{-3}$	

Atomic coordinates (x 10^4) and equivalent isotropic displacement parameters ($Å^2$x 10^3) for shenvi293. U(eq) is defined as one third of the trace of the orthogonalized U^{ij} tensor.

	x	y	z	U(eq)
F(1)	7249(2)	3219(1)	4731(1)	43(1)
F(2)	8835(2)	4216(1)	5294(1)	44(1)
F(3)	6534(2)	5049(1)	4926(1)	33(1)
O(1)	3415(2)	6605(1)	5979(1)	15(1)
O(2)	1367(2)	10400(1)	6744(1)	25(1)
O(3)	847(2)	9085(1)	7408(1)	23(1)
N(1)	5613(2)	4499(1)	5950(1)	17(1)
C(1)	770(2)	5974(2)	6438(1)	21(1)
C(2)	2764(2)	6250(1)	6503(1)	16(1)
C(3)	3235(2)	7283(1)	6895(1)	16(1)
C(4)	2696(2)	8485(2)	6678(1)	17(1)
C(5)	3341(2)	8705(1)	6195(1)	16(1)
C(6)	4509(2)	7702(1)	5980(1)	15(1)
C(7)	5191(2)	7926(1)	5421(1)	18(1)
C(8)	6500(3)	9006(2)	5415(1)	21(1)
C(9)	8046(2)	8859(2)	5813(1)	23(1)
C(10)	7352(2)	8582(1)	6371(1)	19(1)
C(11)	6093(2)	7487(1)	6364(1)	15(1)
C(12)	5323(2)	7123(1)	6912(1)	17(1)
C(13)	5518(2)	5759(1)	7015(1)	18(1)
C(14)	3863(2)	5178(1)	6735(1)	17(1)
C(15)	4288(2)	4136(1)	6354(1)	17(1)
C(16)	5027(3)	3040(2)	6655(1)	23(1)
C(17)	5487(3)	2006(2)	6276(1)	27(1)
C(18)	6751(3)	2439(2)	5839(1)	26(1)
C(19)	5922(2)	3539(1)	5566(1)	20(1)
C(20)	1537(2)	9321(1)	6988(1)	18(1)
C(21)	7147(3)	4007(2)	5136(1)	27(1)
C(22)	270(3)	11278(2)	7019(1)	31(1)

Bond lengths [Å] and angles [°] for shenvi293.

F(1)-C(21)	1.346(2)	C(9)-C(10)	1.529(2)
F(2)-C(21)	1.332(2)	C(10)-H(10A)	0.9900
F(3)-C(21)	1.347(2)	C(10)-H(10B)	0.9900
O(1)-C(2)	1.4612(18)	C(10)-C(11)	1.529(2)
O(1)-C(6)	1.4594(19)	C(11)-H(11)	1.0000
O(2)-C(20)	1.348(2)	C(11)-C(12)	1.548(2)
O(2)-C(22)	1.445(2)	C(12)-H(12)	1.0000
O(3)-C(20)	1.206(2)	C(12)-C(13)	1.539(2)
N(1)-H(1)	0.89(2)	C(13)-H(13A)	0.9900
N(1)-C(15)	1.471(2)	C(13)-H(13B)	0.9900
N(1)-C(19)	1.456(2)	C(13)-C(14)	1.554(2)
C(1)-H(1A)	0.9800	C(14)-H(14)	1.0000
C(1)-H(1B)	0.9800	C(14)-C(15)	1.533(2)
C(1)-H(1C)	0.9800	C(15)-H(15)	1.0000
C(1)-C(2)	1.516(2)	C(15)-C(16)	1.532(2)
C(2)-C(3)	1.552(2)	C(16)-H(16A)	0.9900
C(2)-C(14)	1.553(2)	C(16)-H(16B)	0.9900
C(3)-H(3)	1.0000	C(16)-C(17)	1.529(2)
C(3)-C(4)	1.494(2)	C(17)-H(17A)	0.9900
C(3)-C(12)	1.556(2)	C(17)-H(17B)	0.9900
C(4)-C(5)	1.331(2)	C(17)-C(18)	1.524(3)
C(4)-C(20)	1.484(2)	C(18)-H(18A)	0.9900
C(5)-H(5)	0.9500	C(18)-H(18B)	0.9900
C(5)-C(6)	1.508(2)	C(18)-C(19)	1.527(2)
C(6)-C(7)	1.518(2)	C(19)-H(19)	1.0000
C(6)-C(11)	1.539(2)	C(19)-C(21)	1.506(2)
C(7)-H(7A)	0.9900	C(22)-H(22A)	0.9800
C(7)-H(7B)	0.9900	C(22)-H(22B)	0.9800
C(7)-C(8)	1.538(2)	C(22)-H(22C)	0.9800
C(8)-H(8A)	0.9900		
C(8)-H(8B)	0.9900	C(6)-O(1)-C(2)	113.86(11)
C(8)-C(9)	1.530(2)	C(20)-O(2)-C(22)	115.35(14)
C(9)-H(9A)	0.9900	C(15)-N(1)-H(1)	105.1(13)
C(9)-H(9B)	0.9900	C(19)-N(1)-H(1)	108.3(13)

C(19)-N(1)-C(15)	111.53(13)	C(8)-C(7)-H(7B)	109.6
H(1A)-C(1)-H(1B)	109.5	C(7)-C(8)-H(8A)	109.1
H(1A)-C(1)-H(1C)	109.5	C(7)-C(8)-H(8B)	109.1
H(1B)-C(1)-H(1C)	109.5	H(8A)-C(8)-H(8B)	107.8
C(2)-C(1)-H(1A)	109.5	C(9)-C(8)-C(7)	112.47(13)
C(2)-C(1)-H(1B)	109.5	C(9)-C(8)-H(8A)	109.1
C(2)-C(1)-H(1C)	109.5	C(9)-C(8)-H(8B)	109.1
O(1)-C(2)-C(1)	106.09(13)	C(8)-C(9)-H(9A)	109.2
O(1)-C(2)-C(3)	107.73(12)	C(8)-C(9)-H(9B)	109.2
O(1)-C(2)-C(14)	111.97(12)	H(9A)-C(9)-H(9B)	107.9
C(1)-C(2)-C(3)	115.88(14)	C(10)-C(9)-C(8)	111.90(14)
C(1)-C(2)-C(14)	113.40(13)	C(10)-C(9)-H(9A)	109.2
C(3)-C(2)-C(14)	101.77(12)	C(10)-C(9)-H(9B)	109.2
C(2)-C(3)-H(3)	111.3	C(9)-C(10)-H(10A)	109.5
C(2)-C(3)-C(12)	99.04(13)	C(9)-C(10)-H(10B)	109.5
C(4)-C(3)-C(2)	111.23(13)	C(9)-C(10)-C(11)	110.67(13)
C(4)-C(3)-H(3)	111.3	H(10A)-C(10)-H(10B)	108.1
C(4)-C(3)-C(12)	112.09(14)	C(11)-C(10)-H(10A)	109.5
C(12)-C(3)-H(3)	111.3	C(11)-C(10)-H(10B)	109.5
C(5)-C(4)-C(3)	113.77(14)	C(6)-C(11)-H(11)	107.5
C(5)-C(4)-C(20)	125.14(15)	C(6)-C(11)-C(12)	108.73(13)
C(20)-C(4)-C(3)	121.09(14)	C(10)-C(11)-C(6)	110.42(12)
C(4)-C(5)-H(5)	123.2	C(10)-C(11)-H(11)	107.5
C(4)-C(5)-C(6)	113.55(14)	C(10)-C(11)-C(12)	114.87(13)
C(6)-C(5)-H(5)	123.2	C(12)-C(11)-H(11)	107.5
O(1)-C(6)-C(5)	107.18(12)	C(3)-C(12)-H(12)	111.4
O(1)-C(6)-C(7)	108.56(12)	C(11)-C(12)-C(3)	108.20(13)
O(1)-C(6)-C(11)	107.22(12)	C(11)-C(12)-H(12)	111.4
C(5)-C(6)-C(7)	113.79(13)	C(13)-C(12)-C(3)	102.05(14)
C(5)-C(6)-C(11)	108.96(13)	C(13)-C(12)-C(11)	111.84(13)
C(7)-C(6)-C(11)	110.86(13)	C(13)-C(12)-H(12)	111.4
C(6)-C(7)-H(7A)	109.6	C(12)-C(13)-H(13A)	110.8
C(6)-C(7)-H(7B)	109.6	C(12)-C(13)-H(13B)	110.8
C(6)-C(7)-C(8)	110.23(13)	C(12)-C(13)-C(14)	104.77(13)
H(7A)-C(7)-H(7B)	108.1	H(13A)-C(13)-H(13B)	108.9
C(8)-C(7)-H(7A)	109.6	C(14)-C(13)-H(13A)	110.8

C(14)-C(13)-H(13B)	110.8	C(21)-C(19)-H(19)	109.0
C(2)-C(14)-C(13)	105.58(12)	O(2)-C(20)-C(4)	111.44(14)
C(2)-C(14)-H(14)	106.1	O(3)-C(20)-O(2)	123.58(15)
C(13)-C(14)-H(14)	106.1	O(3)-C(20)-C(4)	124.96(15)
C(15)-C(14)-C(2)	116.46(13)	F(1)-C(21)-F(3)	105.99(14)
C(15)-C(14)-C(13)	115.76(14)	F(1)-C(21)-C(19)	110.93(15)
C(15)-C(14)-H(14)	106.1	F(2)-C(21)-F(1)	106.64(16)
N(1)-C(15)-C(14)	111.48(13)	F(2)-C(21)-F(3)	106.58(15)
N(1)-C(15)-H(15)	108.5	F(2)-C(21)-C(19)	114.20(16)
N(1)-C(15)-C(16)	108.77(14)	F(3)-C(21)-C(19)	112.00(15)
C(14)-C(15)-H(15)	108.5	O(2)-C(22)-H(22A)	109.5
C(16)-C(15)-C(14)	111.04(13)	O(2)-C(22)-H(22B)	109.5
C(16)-C(15)-H(15)	108.5	O(2)-C(22)-H(22C)	109.5
C(15)-C(16)-H(16A)	109.4	H(22A)-C(22)-H(22B)	109.5
C(15)-C(16)-H(16B)	109.4	H(22A)-C(22)-H(22C)	109.5
H(16A)-C(16)-H(16B)	108.0	H(22B)-C(22)-H(22C)	109.5
C(17)-C(16)-C(15)	111.28(13)		
C(17)-C(16)-H(16A)	109.4		
C(17)-C(16)-H(16B)	109.4		
C(16)-C(17)-H(17A)	109.5		
C(16)-C(17)-H(17B)	109.5		
H(17A)-C(17)-H(17B)	108.1		
C(18)-C(17)-C(16)	110.69(14)		
C(18)-C(17)-H(17A)	109.5		
C(18)-C(17)-H(17B)	109.5		
C(17)-C(18)-H(18A)	109.8		
C(17)-C(18)-H(18B)	109.8		
C(17)-C(18)-C(19)	109.32(15)		
H(18A)-C(18)-H(18B)	108.3		
C(19)-C(18)-H(18A)	109.8		
C(19)-C(18)-H(18B)	109.8		
N(1)-C(19)-C(18)	110.19(14)		
N(1)-C(19)-H(19)	109.0		
N(1)-C(19)-C(21)	108.78(14)		
C(18)-C(19)-H(19)	109.0		
C(21)-C(19)-C(18)	110.95(15)		

Anisotropic displacement parameters ($Å^2 x 10^3$) for shenvi293. The anisotropic displacement factor exponent takes the form: $-2\pi^2[h^2 a^{*2}U^{11} + ... + 2 h k a^* b^* U^{12}]$

	U^{11}	U^{22}	U^{33}	U^{23}	U^{13}	U^{12}
F(1)	66(1)	32(1)	32(1)	-7(1)	22(1)	10(1)
F(2)	25(1)	54(1)	53(1)	9(1)	12(1)	-3(1)
F(3)	47(1)	25(1)	27(1)	7(1)	15(1)	8(1)
O(1)	16(1)	15(1)	14(1)	0(1)	-1(1)	-1(1)
O(2)	27(1)	22(1)	26(1)	-4(1)	4(1)	9(1)
O(3)	20(1)	28(1)	22(1)	-6(1)	4(1)	1(1)
N(1)	20(1)	13(1)	18(1)	2(1)	2(1)	2(1)
C(1)	17(1)	23(1)	24(1)	-1(1)	0(1)	-1(1)
C(2)	16(1)	18(1)	15(1)	-1(1)	1(1)	-1(1)
C(3)	16(1)	18(1)	15(1)	-1(1)	1(1)	-1(1)
C(4)	14(1)	18(1)	19(1)	-4(1)	-2(1)	0(1)
C(5)	13(1)	15(1)	19(1)	-2(1)	-4(1)	0(1)
C(6)	14(1)	15(1)	17(1)	0(1)	-1(1)	0(1)
C(7)	20(1)	17(1)	15(1)	0(1)	0(1)	3(1)
C(8)	24(1)	18(1)	21(1)	3(1)	4(1)	2(1)
C(9)	20(1)	21(1)	28(1)	2(1)	4(1)	-2(1)
C(10)	15(1)	18(1)	23(1)	-2(1)	-1(1)	-1(1)
C(11)	14(1)	15(1)	16(1)	0(1)	-2(1)	1(1)
C(12)	18(1)	20(1)	13(1)	-1(1)	-3(1)	0(1)
C(13)	20(1)	20(1)	14(1)	1(1)	-2(1)	0(1)
C(14)	19(1)	18(1)	15(1)	2(1)	1(1)	-1(1)
C(15)	19(1)	15(1)	18(1)	1(1)	0(1)	-2(1)
C(16)	34(1)	16(1)	20(1)	4(1)	-1(1)	-2(1)
C(17)	39(1)	14(1)	27(1)	4(1)	-1(1)	1(1)
C(18)	31(1)	17(1)	31(1)	1(1)	2(1)	6(1)
C(19)	22(1)	16(1)	21(1)	-1(1)	1(1)	2(1)
C(20)	12(1)	21(1)	22(1)	-6(1)	-4(1)	0(1)
C(21)	31(1)	22(1)	28(1)	-1(1)	9(1)	6(1)
C(22)	32(1)	27(1)	34(1)	-8(1)	3(1)	14(1)

Hydrogen bonds for shenvi293 [Å and °].

D-H...A	d(D-H)	d(H...A)	d(D...A)	<(DHA)
N(1)-H(1)...O(1)	0.89(2)	2.12(2)	2.8436(18)	137.2(17)
